# Men’s and women’s endorsement of hegemonic masculinity and responses to COVID-19

**DOI:** 10.1177/13591053221081905

**Published:** 2022-03-11

**Authors:** Nathaniel EC Schermerhorn, Theresa K Vescio

**Affiliations:** 1 The Pennsylvania State University, USA

**Keywords:** COVID-19, gendered health, health attitudes, hegemonic masculinity, political attitudes

## Abstract

Using a gendered psychology of health approach, we examine the effects of the culturally idealized form of masculinity—hegemonic masculinity—for both men *and* women’s health attitudes and behaviors. Using data collected across four studies (*N =* 805) during the COVID-19 pandemic, we found that stronger endorsement of hegemonic masculinity related to health attitudes antithetical to mitigation strategies (e.g. more engagement in risky behaviors, less support for federal mandates) and evaluations of how political leaders have responded to COVID-19. These effects did not differ by gender suggesting that hegemonic masculinity has implications for both men and women’s health.

Since January 2020, COVID-19 has and continues to threaten the global population; therefore, it is important to understand factors that influence perceptions of and compliance with related health guidelines. The present work uses a gendered psychology of health (Lee and Owens, 2002) to examine how the culturally idealized form of masculinity—hegemonic masculinity—affects the health attitudes and behaviors of both men and women. More specifically, we examine how men *and* women’s endorsement of hegemonic masculinity influences their evaluations of political leaders’ responses to COVID-19, personal perceived impact of COVID-19, risk-taking associated with COVID-19, responses to COVID-19 mandates, and belief in COVID-19 conspiracy theories.

## Hegemonic masculinity (HM)

HM refers to the idealized form of masculinity within a given culture that is defined by the interests of the dominant group but maintained through the attitudes and behaviors of most people (Connell, 1995). In other words, HM elevates certain masculine traits above others and positions dominant men within a society (e.g. white, heterosexual, able-bodied men in the United States) above women and marginalized men (e.g. gay, non-white, feminine; Connell, 1995). In the U.S., HM prescribes that men should be: (1) high in power/status, (2) emotionally, physically, and mentally tough, and (3) reject all that is associated with femininity and gayness (Brannon, 1976; Courtenay, 2000; Levant et al., 2007; Pascoe, 2007; Pleck et al., 1994; Thompson and Pleck, 1986; Trujillo, 1991). These prescriptions are impossible for most men to achieve; therefore, men consistently attempt to prove their masculinity and must compensate (via their attitudes/behaviors) when their masculinity is questioned or threatened (Vandello and Bosson, 2013).

## HM and health

Men’s identities are constructed in relation to the prescriptions of HM and their health-related behaviors are central to this identity formation (de Visser and Smith, 2006; de Visser et al., 2009). In fact, masculinity affects men’s health in two ways. First, men may engage in unhealthy and risky behaviors to prove their masculinity to others. Research shows that men prove their masculinity by eschewing health guidelines and engaging in risky, health-related behaviors (Courtenay, 2000; Novak et al., 2019; Saltonstall, 1993) despite the negative effects that these practices have for men’s health and life expectancy (Courtenay, 1998, 2000). These risky, health-related behaviors include binge drinking and substance abuse (e.g. Liu and Iwamoto, 2007; Peralta, 2007; Perrotte et al., 2020), risky sexual practices (e.g. Mahalik et al., 2006; Parmenter et al., 2020), physical fighting (e.g. Mahalik et al., 2006), and unhealthy patterns of food consumption (e.g. energy drinks, Miller, 2008). Men are also less likely than women to seek help for both physical and mental health concerns (Addis and Mahalik, 2003; Vaidya et al., 2012). Second, when men’s masculinity is threatened (i.e. they receive feedback that they are not masculine), they experience negative physiological responses with implications for cardiac health (Kramer et al., 2017).

Beyond HM’s importance to individual men’s identities, HM is a cultural ideology that most people (men and women) accept and perceive as beneficial to the self even when it is personally detrimental. To our knowledge, only one paper has examined HM and health with both male and female participants (Campos et al., 2020). We suggest understanding women’s endorsement of HM and their health-related attitudes and behaviors is important for two reasons. First, a more complete understanding of the gendering of health requires an understanding of how all people (men *and* women) contribute to the association of healthy behaviors with femininity. Second, women who more strongly endorse HM may engage in behaviors (i.e. unhealthy behaviors) in order to confer a higher status within their ingroups by being rewarded for appealing to and reinforcing the widely accepted, albeit status quo maintaining, tenets of HM.

## HM and COVID-19

COVID-19 has become both politicized and gendered. In the U.S., research shows that Republicans (vs. Democrats) were more likely to downplay the severity of the virus and refuse to follow medical guidelines (e.g. Calvillo et al., 2020). In eight countries (including the U.S.), men (vs. women) downplayed the seriousness of COVID-19, and were less likely to agree and comply with state-sanctioned mitigation efforts (Galasso et al., 2020). The politicization and masculinization of responses to COVID-19 was reinforced, in part, by former President Donald Trump.

Trump continually incorporated the rhetoric of HM into discussions of the virus (e.g. Neville-Shepard, 2021). In addition, Trump refused to wear a mask and mocked those who did including, then President-elect, Joe Biden (LeBlanc, 2020). Consistent with Trump’s own attempt to eschew weakness, men who identify as masculine have been less likely to report wearing a mask especially if gender identity is important to their self-concept (Cassino and Besen-Cassino, 2020). In fact, the refusal to wear a mask is linked to beliefs that wearing a mask is “shameful” and shows weakness, particularly for men (Capraro and Barcelo, 2020; Glick, 2020). Extending these findings, we suggest that a more thorough understanding of COVID-19 related perceptions and behavior may be provided given a consideration of men *and* women’s endorsement of HM.

Importantly, research suggests that women’s, like men’s, endorsement of HM affects one’s response to COVID-19. For example, among both men and women, believing that men should be tough predicted more negative views of mask-wearing (Palmer and Peterson, 2020) and higher sexism predicted less concern with COVID-19 and less support of and engagement in mitigation efforts (Reny, 2020). Some female politicians, including Republican Governor Kristi Noem and Republican Congresswoman Marjorie-Taylor Greene, have staunchly advocated against mask and vaccine mandates while downplaying the severity of COVID-19. HM has also been reinforced in media accounts of COVID-19. Conservative commentator Tomi Lahren tweeted a video of Biden wearing a mask with the comment “might as well carry a purse with that mask, Joe” (Lahren, 2020), and colloquial forms of communication (e.g. memes) rely on gendered language surrounding COVID-19 reinforcing social norms based on HM (e.g. “Karen memes”; Bhasin et al., 2020). In addition, both men and women have participated in anti-lockdown protests across the United States, with some specifically organized by women’s organizations (e.g. Women for America First; Steakin, 2020).

## The present research

The present work aims to add to existing research on masculinity and health by examining the influence that HM has on both men and women’s health attitudes and behaviors. As noted above, men both engage in unhealthy behaviors to prove their masculinity and experience physiological stress when they fail to demonstrate their masculinity. Many interventions for men’s health focus on promoting health for individual men and may inadvertently reinforce HM in the process (e.g. Fleming et al., 2014). A more complete examination of the “feminization” of health and health-related behaviors must also examine how women’s endorsement of HM contributes to the gendering of health crises such as COVID-19. For HM to remain culturally valued, it requires the endorsement of both men and women and the present research seeks to examine this endorsement during the COVID-19 pandemic.

In four studies, we hypothesized that over and above political party, gender, race, and socioeconomic status, for both men and women, stronger endorsement of HM would be associated with (1) more positive evaluations of how Republican (vs. Democratic) politicians have responded to COVID-19, (2) a greater likelihood to engage in risky behaviors during COVID-19, (3) less reported personal impact of COVID-19, (4) less agreement with mandates seeking to mitigate the spread of COVID-19, and (5) greater belief in COVID-19 conspiracy theories. All studies were approved by the authors’ Institutional Review Board. The current article includes the complete raw datasets collected in the studies including the participants’ data set, syntax file, and log files for analysis.

## Studies 1a and 1b

Study 1a was conducted in April 2020, prior to the 2020 U.S Presidential election. At the time of data collection, the Centers for Disease Control and Prevention (CDC, 2021) reported 47,223 deaths resulting from COVID-19 in the U.S. Study 1b was conducted in December 2020 when the CDC (2021) reported 291,989 cumulative COVID-19 related deaths in the U.S. In addition, Study 1b data were collected after both the 2020 Presidential election and the announcement of Joe Biden as the President-elect. With two exceptions, the procedure was identical in both studies; therefore, we describe them together.^1^

### Method

#### Participants

Participants in both Study 1a (*N =* 178; 55.1% female; *M*_age_ _=_ 19.76) and Study 1b (*N =* 241; 49.8% female; *M*_age_ _=_ 19.05) were undergraduate students from the Pennsylvania State University online psychology subject pool who received partial course credit for their participation. The supplemental materials contain full demographic information.

### Procedure and measures

In both studies, participants completed measures of HM, likelihood to engage in various COVID-19 related risks, concern about contracting COVID-19, and perceptions that the COVID-19 pandemic would personally impact their finances, access to resources, and psychological well-being. After reporting their personal experience with COVID-19 (e.g. symptoms), participants answered political and demographic questions.

In Study 1b, participants completed all COVID-related and political questions first, then reported their endorsement of HM. In addition, participants in Study 1b completed a measure of precarious masculinity (PM). The changes to Study 1b allowed us to examine if (1) masculinity questions primed responses to the COVID related questions and (2) if men’s PM would be associated with responses to COVID. Only one significant finding emerged in analyses of PM; therefore, for parsimony, we fully present and discuss all analyses including PM in the supplemental materials. Supplemental materials also contain descriptive statistics for all measures.

#### HM

Participants completed the 26-item Male Role Norms Scale (1 = strongly disagree, 7 = strongly agree; Thompson and Pleck, 1986), which assesses beliefs that men should be (1) high in status and power (e.g. “Success in his work has to be man’s central goal in this life”), (2) physically, emotionally, and mentally tough (e.g. “I think a young man should try to become physically tough even if he’s not big”), and (3) nothing like women (e.g. “It’s a bit embarrassing for a man to have a job that is usually filled by a woman”). After reverse-scoring appropriate items, a HM variable was created by averaging across items (Study 1a: α = 0.92; Study 1b: α = 0.91).

#### COVID-19 risk taking

Participants indicated how likely they would be to engage in 18 risky behaviors associated with COVID-19 (1 = extremely unlikely, 7 = extremely likely). An exploratory factor analysis revealed a two-factor solution. More specifically, Study 1a findings revealed that 15 items loaded on the first factor, which accounted for 29.01% of the total variance; we averaged across these items to create a rule-based risk-taking variable (e.g. “Continuing to have friends over who don’t live with you;” Study 1a: α = 0.85; Study 1b: α = 0.84). Three items loaded on the second factor, which accounted for 14.53% of variance; we averaged across these items to create a help-based risk-taking variable (e.g. “Volunteering at an understaffed medical facility;” Study 1a: α = 0.81; Study 1b: α = 0.80). See supplemental materials for all items and factor loadings.

#### COVID-19 impact

Participants completed a 6-item perceived coronavirus threat questionnaire (1 = strongly disagree, 7 = strongly agree; Gideon Conway et al., 2020; e.g. “I am afraid of COVID-19”). After reverse-scoring appropriate items, we averaged across items to create a personal threat variable (Study 1a: α = 0.89; Study 1b: α = 0.87). Participants also completed the short version of the Coronavirus Impacts Questionnaire (Gideon Conway et al., 2020), which indicated perceptions of the personal impact of the pandemic on one’s psychological well-being (2 items, e.g. “I have become depressed because of COVID-19”; Study 1a: α = 0.84; Study 1b: α = 0.82), finances (2 items, e.g. “I have lost job-related income due to COVID-19”; Study 1a: α = 0.77; Study 1b: α = 0.75), and access to resources (2 items, e.g. “It has been difficult for me to get the things I need due to COVID-19”; Study 1a: α = 0.79; Study 1b: α = 0.67). Analyses of resource impact produced no significant findings in either study; therefore, this variable will not be discussed further.

#### Personal experiences with COVID-19

To permit analyses controlling for personal experiences with COVID-19, participants completed the 7-item short version of the Coronavirus Experiences Questionnaire (Gideon Conway et al., 2020; e.g. “I have been diagnosed with COVID-19”). All analyses reported were also conducted controlling for the personal experience items that were affirmatively reported by over 50% of participants and the same pattern of results emerged. In other words, personal experiences with COVID-19 did not alter the reported findings.

#### Political affiliation

Participants indicated their political party affiliation (1 = Democrat, 3 = Independent, 5 = Republican) and ideology (1 = Very Liberal, 7 = Very Conservative). Because one’s loyalty to their political party drives their political attitudes (Barber and Pope, 2019) including one’s attitudes and behaviors regarding COVID-19 (Makridis and Rothwell, 2020), particularly following the increased partisan divide following the election of Trump (Bartels, 2020), we used political party in all analyses reported below. However, the two questions were highly correlated (Study 1a: *r* = 0.81, *p* < 0.001; Study 1b: *r* = 0.84, *p* < 0.001). Therefore, we also present and discuss all analyses using political ideology (in place of political party) in the supplemental materials.

#### Socioeconomic status (SES)

Participants self-reported their SES as poor, working class, middle class, upper middle class, or upper class; higher scores indicated higher SES.

#### Donald trump approval

Participants indicated their answer (1 = strongly disapprove, 7 = strongly approve) to the following question: “Do you approve or disapprove of the way Donald Trump is handling his job as President?” (Pew Research Center, 2020).

#### Evaluation of political responses to COVID-19

Using a 7-point scale (1 = Strongly Disapprove, 7 = Strongly Approve), participants indicated their opinion as to how President Trump, Republican leaders (Mitch McConnell, Republican Congress), Democratic leaders (Nancy Pelosi, Joe Biden, Democratic Congress), state leaders, and non-partisan scientist, Dr. Anthony Fauci), have handled COVID-19.

## Results

To examine the unique variance of HM over and above political party affiliation and demographic variables (gender, race, SES), we conducted a series of hierarchical regressions. In Step 1, we entered political party affiliation. In Step 2, we entered gender (−1 = female, 1 = male), race (−1 = non-White, 1 = White), and SES. In Step 3, we entered HM (as well as PM in Study 1b). Finally, we entered all two-way interactions between masculinity variables and the demographic variables in Step 4 (i.e. HM × party, HM × gender, HM × race, HM × SES). As noted, although HM is culturally valued and must be endorsed by most people, its prescriptions are based on a White, heterosexual, conservative, middle-class male prototype (e.g. Connell, 1995). Therefore, the interactions tested if HM interacted with related demographics in predicting outcomes. We present and interpret all significant interactions that were also associated with a significant Δ*R*^2^ in Step 4 in the main text and provide all interaction coefficients in the supplemental materials. Simple effects tests including continuous variables were conducted for participants who scored high (+1 SD) and low (−1 SD) on the relevant variables (Aiken and West, 1991). We present standardized coefficients in all tables.

Consistent with the notion that the COVID-19 pandemic has been politicized in the U.S., across most analyses, we found political party to be the variable most strongly associated with outcomes. We therefore examined the Δ*R*^2^ associated with HM (Step 3) and focus on interpreting those results below.

### Evaluations of political leaders’ responses to COVID-19

Consistent with predictions, and as shown in Table 1, HM was associated with evaluations of Republican leaders’ responses to COVID-19 in both April 2020 (Study 1a, left panel) and December 2020 (Study 1b, right panel); stronger endorsement of HM was associated with more positive evaluations of both Trump and McConnell. Interestingly, endorsement of HM was only associated with more negative evaluations of Democratic leaders (Biden and Pelosi) as well as Dr. Fauci’s responses to COVID-19 in December 2020 (Study 1b, Table 1 right panel).^2^

**Table 1.** Results of hierarchical regressions for evaluations of political leaders’ responses to COVID-19, Studies 1a and 1b.

Independent variables	Study 1a (April 2020)					Study 1b (December 2020)				
	Trump response	McConnell response	Biden response	Pelosi response	Fauci response	Trump response	McConnell response	Biden response	Pelosi response	Fauci response
Step 1: *R*^2^	0.512***	0.249***	0.075**	0.274***	0.022^t^	0.606***	0.315***	0.494***	0.384***	0.114***
Political party	0.72***	0.50***	−0.27**	−0.52***	−0.15^t^	0.78***	0.56***	−0.70***	−0.62***	−0.34***
Step 2: ∆ *R*^2^	0.013	0.015	0.009	0.016	0.015	0.011	0.027^t^	0.005	0.019	0.018
Political party	0.70***	0.50***	−0.29**	−0.50***	−0.18*	0.75***	0.59***	−0.70***	−0.60***	−0.37***
Gender	0.00	−0.08	0.01	0.00	0.11	0.03	−0.16**	−0.03	0.12*	0.01
Race	0.11*	0.06	0.07	−0.13	0.08	0.08^t^	0.04	0.01	0.05	0.10
SES	0.04	0.08	0.06	−0.01	−0.02	−0.07	−0.01	0.07	−0.05	0.09
Step 3: ∆ *R*^2^	0.021**	0.026*	0.009	0.001	0.000	0.024***	0.037***	0.012*	0.020*	0.023*
Political party	0.61***	0.39***	−0.35**	−0.47***	−0.18	0.68***	0.50***	−0.65***	−0.53***	−0.30***
Gender	−0.07	−0.16^t^	−0.03	0.01	0.11	−0.03	−0.24***	0.02	−0.06	0.08
Race	0.11*	0.06	0.08	−0.13	0.08	0.10*	0.06	−0.01	0.04	0.08
SES	0.02	0.07	0.05	−0.01	−0.02	−0.09*	−0.03	0.08	−0.04	0.11
HM	0.19**	0.22*	0.13	−0.05	0.00	0.19***	0.24***	−0.14*	−0.17*	−0.19*
Step 4: ∆ *R*^2^	0.009	0.040	0.020	0.008	0.015	0.007	0.029^t^	0.004	0.005	0.020

SES: socioeconomic status; HM: hegemonic masculinity.

t *p <* 0.075. *p < 0.05. ** p < 0.01. *** p < 0.001.

### COVID-19 risk-taking and personal impact

Consistent with predictions, and as shown in Table 2, HM was associated with a greater likelihood of engaging in behaviors considered risky during the ongoing pandemic in both April 2020 (Study 1a, left panel) and December 2020 (Study 1b, right panel). More specifically, stronger endorsement of HM was associated with a greater reported likelihood of taking risks that were antithetical to mitigation strategies (e.g. not wearing a mask, continuing to gather with others), but not risks that would be considered helpful (e.g. volunteering at a medical facility).

**Table 2.** Results of hierarchical regressions for risk-taking during and perceived personal impact of COVID-19, Studies 1a and 1b.

Independent variables	Study 1a (April 2020)					Study 1b (December 2020)				
	Risk-taking		Personal impact			Risk-taking		Personal impact		
	Rule-based risks	Helping risks	Personal threat	Psych. impact	Financial impact	Rule-based risks	Helping risks	Personal threat	Psych. impact	Financial impact
Step 1: *R*^2^	0.089***	0.000	0.074***	0.021^t^	0.002	0.179***	0.028**	0.189***	0.068***	0.016^t^
Political party	0.30***	0.00	−0.27***	−0.14^t^	0.05	0.42***	−0.17**	−0.43***	−0.26***	−0.13^t^
Step 2: ∆ *R*^2^	0.114***	0.042^t^	0.142***	0.068**	0.133***	0.016	0.050**	0.023	0.017	0.032^t^
Political party	0.22**	0.02	−0.22**	−0.12	0.05	0.38***	−0.10	−0.38***	−0.22**	−0.09
Gender	0.33***	−0.18*	−0.38***	−0.25**	−0.03	0.12*	−0.08	−0.15*	−0.13*	−0.12^t^
Race	0.11	0.09	0.01	0.08	0.21**	0.05	−0.16*	−0.05	−0.03	−0.02
SES	−0.04	−0.05	0.11	0.04	−0.31***	−0.02	−0.14*	0.00	0.03	−0.13*
Step 3: ∆ *R*^2^	0.051**	0.005	0.002	0.044**	0.000	0.098***	0.000	0.031**	0.014^t^	0.005
Political party	0.09	−0.03	−0.19*	0.01	0.06	0.22***	−0.11	−0.30***	−0.16*	−0.05
Gender	0.22**	−0.21*	−0.36***	−0.15^t^	−0.02	0.00	−0.09	−0.08	−0.09	−0.09
Race	0.12	0.09	0.00	0.07	0.21**	0.09	−0.16*	−0.07	−0.04	−0.03
SES	−0.05	−0.05	0.11	0.06	−0.31***	−0.06	−0.14*	0.02	0.05	−0.12^t^
HM	0.30**	0.10	−0.06	−0.28**	−0.03	0.38***	0.03	−0.22**	−0.14^t^	−0.09
Step 4: ∆ *R*^2^	0.016	0.040	0.007	0.038	0.084**	0.016	0.017	0.036*	0.008	0.042*
HM × Gender					−0.18*					
HM × SES					0.27***			0.13*		0.19**
HM × Race								−0.17*		

SES: socioeconomic status; HM: hegemonic masculinity.

t *p <* 0.075. *p < 0.05. ** p < 0.01. *** p < 0.001.

Differences emerged when examining the effect of HM on the personal impact of COVID-19 between April 2020 (Study 1a, Table 2 left panel) and December 2020 (Study 1b, Table 2 right panel). While HM was not related to personal threat of COVID-19 early in the pandemic (Study 1a, April 2020), endorsement of HM was associated with less personal threat a year into the pandemic (Study 1b, December 2020). Significant interactions revealed that endorsement of HM was associated with less personal threat of COVID-19 for White (but not non-White) participants (White: *b = −*0.45, *t*(228) = *−*3.48, *p* < 0.001; non-White: *b =* 0.09, *t*(228) = 0.46, *p =* 0.648) and endorsement of HM was associated with less personal threat of COVID-19 for those low (but not high) in SES (low: *b* = *−*0.37, *t*(228) = *−*2.64, *p =* 0.009; high: *b =* 0.01, *t*(228) = 0.03, *p =* 0.973).

An opposite pattern emerged for HM’s association with psychological impact of COVID-19: stronger endorsement of HM was associated with less COVID-related negative affect and depression early in the pandemic (Study 1a, April 2020) but this effect was only marginally significant in December 2020 (Study 1b). HM, on its own, was not related to financial impact of COVID-19 in either study. However, in Study 1a (Table 2, left panel), men (but not women) reported marginally less financial impact from the pandemic when they were high, but not low, in HM (high: *b = −*0.64, *t*(168) = *−*1.86, *p =* 0.065; low: *b* = 0.66, *t*(168) = 1.64, *p =* 0.103). In addition, in both studies, as one’s SES increases, they reported less financial impact from the pandemic when they were low, but not high, in HM (Study 1a: low: *b = −*1.06, *t*(168) = *−*5.75, *p* < 0.001; high: *b* = 0.02, *t*(170) = 0.09, *p =* 0.928; Study 1b: low: *b = −*0.55, *t*(228) = *−*3.33, *p =* 0.001; high: *b =* 0.09, *t*(228) = 0.53, *p =* 0.599). Taken together, stronger endorsement of HM was consistently associated with a greater likelihood to engage in risky behavior contrary to virus mitigation strategies but had differential effects on the personal impact of COVID-19 throughout the first year of the pandemic.

## Studies 2a and 2b

Both Studies 2a and 2b were conducted in late October 2021, approximately 21 months since the first reports of COVID-19 in January 2020. At the time of data collection, the CDC (2021) reported 740,348 cumulative COVID-19 related deaths in the U.S. Importantly, unlike during the data collection for Studies 1a and 1b, the COVID-19 vaccine was widely available in the U.S. during data collection for Studies 2a and 2b. The CDC (2021) reported that 78.0% of eligible adults had received at least one vaccination in October 2021. In addition, while Studies 1a and 1b were conducted during the Trump administration, Studies 2a and 2b were conducted during the Biden administration. In the present studies, instead of examining evaluations of political leaders, we included measures of approval of federal mandates, belief in COVID-19 conspiracy theories, and national identity. The procedure was identical for both Studies 2a (student sample) and 2b (non-student sample).

### Method

#### Participants

Study 2a recruited 188 participants (50.0% female; *M*_age_ _=_ 18.84) from the Pennsylvania State University psychology subject pool who received partial course credit for their participation. Study 2b recruited 198 participants (50.0% female; *M*_age_ _=_ 36.74*)* from Prolific.co who were compensated $2.85 ($17.80/hour) for their participation. The supplemental materials contain full demographic information.

### Procedure and measures

In both studies, participants first completed a measure of HM, their likelihood to engage in various COVID-19 related risks, concern about contracting COVID-19, and perceptions that the COVID-19 pandemic would personally impact their finances and psychological well-being. Participants then reported their opinions toward various COVID-19 mandates and belief in COVID-19 conspiracy theories before indicating their national identity, political affiliation, and providing demographic information.

#### HM

As in Studies 1a and 1b, participants completed the Male Role Norms Scale (Study 2a: α = 0.91; Study 2b: α = 0.94).

#### COVID-19 risk taking

Participants completed the 15 items that measured rule-based risk taking in Studies 1a and 1b (Study 2a: α = 0.86; Study 2b: α = 0.84). We removed the help-based risk-taking items.

#### COVID-19 threat and impact

Participants completed the same scale as in Studies 1a and 1b measuring personal threat (Study 2a: α = 0.86; Study 2b: α = 0.92), psychological impact (Study 2a: α = 0.85; Study 2b: α = 0.86), and financial impact (Study 2a: α = 0.77; Study 2b: α = 0.91). We removed the items measuring personal impact on resources.

#### COVID-19 mandates

Using a 7-point scale (1 = strongly disagree, 7 = strongly agree), participants rated their opinion on 6 COVID-19 related mandates presented by President Biden (e.g. “People should continue to be required to wear masks indoors,” “People should have to provide proof of vaccination to travel”). We averaged items to create a mandate score (Study 2a: α = 0.94; Study 2b: α = 0.96); higher scores indicated greater agreement with the mandates. All items are presented in supplemental materials.

#### COVID-19 conspiracy beliefs

Using 5-point scales (1 = not at all credible, 5 = extremely credible), participants responded to 9 COVID-related conspiracy theories (e.g. “COVID-19 was developed to control population growth”; Van Prooijen et al., 2021). We averaged across items to create a conspiracy score (Study 2a: α = 0.88; Study 2b: α = 0.83); higher scores indicate greater belief in COVID-19 conspiracy theories.

#### National identity

Participants completed 2 items using a 7-point scale (1 = strongly disagree, 7 = strongly agree) to measure their national identity: “I identify as American” and “Being an American is an important reflection of who I am” (Study 2a: α = 0.78; Study 2b: α = 0.62; van Bavel et al., 2020).

#### Political affiliation

As in the previous studies, participants indicated both their political party affiliation and ideology, which were highly correlated (Study 2a: *r* = 0.79, *p* < 0.001; Study 2b: *r* = 0.90, *p* < 0.001). We continued to use political party in all analyses and report analyses with political ideology in the supplemental materials.

#### SES

We used the same measure as in Studies 1a and 1b.

## Results

Analyses were identical to those in Studies 1a and 1b. Similar to Studies 1a and 1b, political party was the variable most strongly related to each outcome (see Step 1) with one exception—financial impact of COVID-19. We continued to examine the unique Δ*R*^2^ associated with HM (see Step 3).

### COVID-19 risk-taking and personal impact

Consistent with predictions and with Studies 1a and 1b, in both the student sample (Study 2a) and the non-student sample (Study 2b), stronger endorsement of HM was associated with a greater likelihood of engaging in risks that go against COVID-19 mitigation strategies (see Table 3). However, there was no evidence that HM was associated with the personal impact of COVID-19 (i.e. personal threat, psychological impact, financial impact) in either sample (see Supplemental materials).

**Table 3.** Results of hierarchical regressions for risk-taking, COVID-19 mandates, and belief in COVID conspiracies, Studies 2a and 2b.

Independent variables	Study 2a			Study 2b		
	Rule-based risks	Mandates	Conspiracies	Rule-based risks	Mandates	Conspiracies
Step 1: *R*^2^	0.265***	0.300***	0.130***	0.284***	0.526***	0.216***
Political party	0.52***	−0.55***	0.36***	0.53***	−0.73***	0.47***
Step 2: ∆ *R*^2^	0.072***	0.068***	0.017	0.016	0.002	0.002
Political party	0.44***	−0.54***	0.36***	0.53***	−0.73***	0.47***
Gender	0.22**	−0.23***	0.05	0.12*	0.01	0.03
Race	0.17**	−0.10	−0.08	−0.02	0.02	−0.01
SES	0.09	0.13*	0.09	−0.02	0.04	−0.03
Step 3: ∆ *R*^2^	0.068***	0.027**	0.045**	0.028**	0.007	0.122***
Political party	0.33***	−0.47***	0.27***	0.45***	−0.68***	0.28***
Gender	0.07	−0.14*	−0.07	0.06	0.04	−0.09
Race	0.16**	−0.10	−0.08	0.01	0.00	0.06
SES	0.06	0.15*	0.07	−0.04	0.05	−0.07
HM	0.32***	−0.21**	0.26**	0.20**	−0.10	0.42***
Step 4: ∆ *R*^2^	0.008	0.016	0.081**	0.010	0.007	0.004
HM × Party			0.21**			

SES: socioeconomic status; HM: hegemonic masculinity.

* *p <* 0.05. ** p < 0.01. *** p < 0.001.

### COVID-19 mandates and conspiracies

Consistent with predictions, in both samples, stronger endorsement of HM was associated with a greater likelihood of reporting COVID-19 conspiracy theories as credible. However, this effect was larger in the non-student sample (see Table 3, right panel) and, in the student sample, was significant for students who identified as Republican but not Democrat (Republican: *b =* 0.35, *t*(178) = 4.00, *p* < 0.001; Democrat: *b =* 0.02, *t*(178) = 0.26, *p =* 0.797). In addition, for students, endorsement of HM was associated with less agreement with the various mandates presented by the Biden administration (Table 3). Similar to risk-taking, students who strongly endorsed HM were less supportive of mandates (such as vaccine requirements) that sought to reduce COVID-19 infections and deaths. Interestingly, however, this effect was not replicated in the non-student sample.

### National identity

National identity was correlated with HM among both students (*r =* 0.236, *p =* 0.001) and non-students (*r =* 0.502, *p* < 0.001). Given that national identity predicts compliance with COVID-19 mitigation efforts globally (van Bavel et al., 2020), we conducted a series of hierarchical regressions including national identity in Step 3, HM in Step 4, and appropriate injouteractions in Step 5. We replicated the pattern of results reported above even when controlling for national identity (see Supple-mental materials).

## Discussion

Using data collected in the U.S. during the COVID-19 pandemic, the present research adds to the gendered psychology of health by examining how HM influences both men *and* women’s health-related attitudes and behaviors. Consistent with predictions—and across four studies—men and women who strongly endorsed HM were more likely to report engaging in risky behavior that health officials considered fundamental to the rapid spread of COVID-19 (e.g. not wearing a mask). In both Studies 1a (April 2020) and 1b (December 2020), endorsement of HM was associated with greater approval of how Republican leaders (Trump and McConnell) responded to COVID-19. In Study 1b (December 2020), endorsement of HM was also associated with disapproval of how Democratic leaders (Biden and Pelosi) and Fauci responded to COVID-19. In addition, in Study 2a (October 2021), men and women who more strongly endorsed HM were less supportive of federal mandates that would help mitigate the spread of COVID-19 (e.g. vaccine requirements) and, in both Studies 2a and 2b, endorsement of HM was associated with greater belief in COVID-19 related conspiracy theories.

Consistent with the prescription that men should be emotionally and mentally tough, HM was associated with the perception of fewer personal psychological effects of the pandemic (Study 1a) and less personal threat of the pandemic (Study 1b) for both men and women; however, HM was not associated with personal impacts of COVID-19 in Studies 2a and 2b. While it is possible that concern has diminished over time, the means were relatively stable across studies (see Supplemental materials). Therefore, the association of HM with perceptions of personal impact may be stronger when HM is made salient (e.g. through Trump’s pandemic rhetoric; Neville-Shepard, 2021) than when it is not made salient.

Interestingly, HM was associated with evaluations of Republican leaders in both Study 1a and 1b but Democratic leaders in Study 1b only. Given that Study 1b was conducted after Biden was named President-elect and a month before he took office, it is possible that those who anticipated his COVID-19 policies to be more restrictive than those of the Trump administration were also those who more strongly endorse HM. In the present research, as in our past work (Vescio and Schermerhorn, 2021), the effects of HM emerge independently from the effects of political party and, overall, do not appear to be stronger for Republicans (vs. Democrats). Unexpectedly, when we included political ideology (rather than party), the effects of HM on evaluations of political leaders’ responses to COVID-19 were minimized (see Supplemental materials). In addition, recent research has shown a strong association between traditional masculinity and conservativism (McDermott et al., 2021). Although beyond the scope of the present data and analyses, it is possible that evaluations of political leaders’ responses to COVID-19 were confounded with participants’ general political beliefs. It has also been said that Trump and Biden’s responses to COVID-19 reflect differences in the type of masculinity they embodied (e.g. Viser, 2020). In other words, conservative (vs. liberal) politicians were more likely to engage in risky behaviors during COVID-19 and masculinity is often associated with conservative politicians (Katz, 2016). Therefore, to better understand the distinction between conservatism and HM and its effect on evaluations of political leaders’ public health responses, future research should examine health events that are gendered, but not politicized in the same way that COVID-19 has been.

We also tested the influence of HM on health-related attitudes and behaviors alongside other explanations for risky (or non-risky) health decisions during the pandemic. Recent research has shown that the belief in conspiracy theories related to COVID-19 decrease the likelihood of engaging in mitigation efforts (van Prooijen et al., 2021) while stronger national identity increases the likelihood of engaging in mitigation efforts (van Bavel et al., 2020). We found that stronger endorsement of HM was associated with belief in COVID-19 conspiracy theories and continued to be associated with outcomes when controlling for national identity.

The relationship between masculinity and health-related behavior, as well as the consequences of this relationship, has been widely studied for men. The present research contributes to this literature by demonstrating a relationship between the endorsement of HM with positive evaluations of political leaders who have downplayed the severity of COVID-19 and supported limited mitigation strategies and a greater likelihood of personally eschewing health guidelines. The present research also adds to the very limited literature on how HM influences women’s health-related attitudes and behavior. It is not surprising that women who more strongly endorse HM also support politicians whose rhetoric connects HM to COVID-19. However, women who more strongly endorsed HM also reported a greater likelihood of taking COVID-19 related risks. In other words, their endorsement of HM seems to also lead them to engage in unhealthy and risky behaviors. The present research joins one other study finding that women’s endorsement of HM leads to greater meat consumption (which is considered masculine; Campos et al., 2020) but cannot speak to the specific reasons why women’s endorsement of HM might influence their own health behaviors. One possibility is that, in certain situations, women who engage in masculine behaviors can increase their social status (e.g. Pascoe, 2007). For example, women who refuse to wear masks may increase their social standing within groups that have downplayed COVID-19 or seen it as a “hoax.” This, however, is one possibility and future research should more closely examine the influence of HM on individual women’s health.

Beyond the effect of HM on individuals’ health-related attitudes and behaviors, there are important implications for public health initiatives. As discussed, masculinity is associated with negative health outcomes for men, and women who endorse HM can contribute to this association. At the same time, public health campaigns seeking to promote healthy habits for men may have the unintended outcome of reinforcing, rather than dismantling, HM. For example, the phrase “real men wear masks” became popular early in the COVID-19 pandemic, a phrase touted by Democratic Congresswoman Nancy Pelosi at a press conference. However, in adopting this strategy, public health campaigns catered to the bolstering of men’s masculinity through their rhetorical strategies rather than attempting to sever the link between masculinity and unhealthy/risky behaviors. In addition, images associated with “real men wear masks” reinforced a White, heterosexual masculinity (e.g. former Vice President Dick Cheney wearing a mask as well as a cowboy hat) that continues to marginalize other forms of masculinity (see Hesse, 2020; Laughney, 2020). Thus, understanding how to promote healthy behaviors for men without reinforcing HM is an important area for future research in public health strategies but also for health practitioners’ (e.g. nurses, doctors) who may reinforce HM through their regular interactions with patients (Seymour-Smith et al., 2002).

Despite the consistencies across our four studies, it is important to address some of the limitations of generalizability based on our samples. While most of our participants were undergraduate students, we included one sample of adults. Similar to recent research that has revealed parallel patterns between undergraduate students and representative adult samples when examining HM’s relationship to political attitudes—including evaluations of Trump and Biden (Vescio and Schermerhorn, 2021), the present work also finds relatively consistent patterns when examining responses to COVID-19. Additionally, while beliefs about masculinity are relatively consistent between samples of undergraduate and adult men, masculinity is more fragile for younger (vs. older) men (Stanaland and Gaither, 2021). The current research does find that among our student (but not non-student) sample, HM was associated with disapproval of federal mandates. It is possible that this is an anomaly, but it also is possible that this is due to a more precarious masculine identity among younger men. Because current U.S. conceptualizations of HM are based on White prototypicality, it is not surprising that, among majority White participants, the endorsement of HM is associated with attitudes and behaviors that embody toughness (e.g. taking risks associated with COVID-19). Important to the conceptualization of HM, non-White men can and will endorse HM in order to benefit from the societal rewards for “good men” (Connell, 1995). Research with more diverse samples is needed to better understand how race and HM interact to understand health-related attitudes and behaviors, particularly in light of racial differences in health outcomes including COVID-19 (e.g. Alcendor, 2020).

The present work contributes to an understanding of how HM influences responses to and the consequences of COVID-19 in men and women. Masculinity has largely been studied as an influence of individual men’s health-related behaviors. However, the present research documents the role of masculinity as an ideology that can be endorsed by both men and women with health-related consequences beyond the effects of threats to masculinity that any individual man may experience.

## Supplemental Material

sj-docx-13-hpq-10.1177_13591053221081905 – Supplemental material for Men’s and women’s endorsement of hegemonic masculinity and responses to COVID-19 Click here for additional data file. Supplemental material, sj-docx-13-hpq-10.1177_13591053221081905 for Men’s and women’s endorsement of hegemonic masculinity and responses to COVID-19 by Nathaniel EC Schermerhorn and Theresa K Vescio in Journal of Health Psychology

sj-pdf-1-hpq-10.1177_13591053221081905 – for Men’s and women’s endorsement of hegemonic masculinity and responses to COVID-19 Click here for additional data file. sj-pdf-1-hpq-10.1177_13591053221081905 for Men’s and women’s endorsement of hegemonic masculinity and responses to COVID-19 by Nathaniel EC Schermerhorn and Theresa K Vescio in Journal of Health Psychology This article is distributed under the terms of the Creative
Commons Attribution 4.0 License ( https://creativecommons.org/licenses/by/4.0/) which
permits any use, reproduction and distribution of the work without
further permission provided the original work is attributed as specified
on the SAGE and Open Access pages ( https://us.sagepub.com/en-us/nam/open-access-at-sage).

sj-pdf-2-hpq-10.1177_13591053221081905 – for Men’s and women’s endorsement of hegemonic masculinity and responses to COVID-19 Click here for additional data file. sj-pdf-2-hpq-10.1177_13591053221081905 for Men’s and women’s endorsement of hegemonic masculinity and responses to COVID-19 by Nathaniel EC Schermerhorn and Theresa K Vescio in Journal of Health Psychology This article is distributed under the terms of the Creative
Commons Attribution 4.0 License ( https://creativecommons.org/licenses/by/4.0/) which
permits any use, reproduction and distribution of the work without
further permission provided the original work is attributed as specified
on the SAGE and Open Access pages ( https://us.sagepub.com/en-us/nam/open-access-at-sage).

sj-pdf-3-hpq-10.1177_13591053221081905 – for Men’s and women’s endorsement of hegemonic masculinity and responses to COVID-19 Click here for additional data file. sj-pdf-3-hpq-10.1177_13591053221081905 for Men’s and women’s endorsement of hegemonic masculinity and responses to COVID-19 by Nathaniel EC Schermerhorn and Theresa K Vescio in Journal of Health Psychology This article is distributed under the terms of the Creative
Commons Attribution 4.0 License ( https://creativecommons.org/licenses/by/4.0/) which
permits any use, reproduction and distribution of the work without
further permission provided the original work is attributed as specified
on the SAGE and Open Access pages ( https://us.sagepub.com/en-us/nam/open-access-at-sage).

sj-pdf-4-hpq-10.1177_13591053221081905 – for Men’s and women’s endorsement of hegemonic masculinity and responses to COVID-19 Click here for additional data file. sj-pdf-4-hpq-10.1177_13591053221081905 for Men’s and women’s endorsement of hegemonic masculinity and responses to COVID-19 by Nathaniel EC Schermerhorn and Theresa K Vescio in Journal of Health Psychology This article is distributed under the terms of the Creative
Commons Attribution 4.0 License ( https://creativecommons.org/licenses/by/4.0/) which
permits any use, reproduction and distribution of the work without
further permission provided the original work is attributed as specified
on the SAGE and Open Access pages ( https://us.sagepub.com/en-us/nam/open-access-at-sage).

sj-sav-11-hpq-10.1177_13591053221081905 – for Men’s and women’s endorsement of hegemonic masculinity and responses to COVID-19 Click here for additional data file. sj-sav-11-hpq-10.1177_13591053221081905 for Men’s and women’s endorsement of hegemonic masculinity and responses to COVID-19 by Nathaniel EC Schermerhorn and Theresa K Vescio in Journal of Health Psychology This article is distributed under the terms of the Creative
Commons Attribution 4.0 License ( https://creativecommons.org/licenses/by/4.0/) which
permits any use, reproduction and distribution of the work without
further permission provided the original work is attributed as specified
on the SAGE and Open Access pages ( https://us.sagepub.com/en-us/nam/open-access-at-sage).

sj-sav-5-hpq-10.1177_13591053221081905 – for Men’s and women’s endorsement of hegemonic masculinity and responses to COVID-19 Click here for additional data file. sj-sav-5-hpq-10.1177_13591053221081905 for Men’s and women’s endorsement of hegemonic masculinity and responses to COVID-19 by Nathaniel EC Schermerhorn and Theresa K Vescio in Journal of Health Psychology This article is distributed under the terms of the Creative
Commons Attribution 4.0 License ( https://creativecommons.org/licenses/by/4.0/) which
permits any use, reproduction and distribution of the work without
further permission provided the original work is attributed as specified
on the SAGE and Open Access pages ( https://us.sagepub.com/en-us/nam/open-access-at-sage).

sj-sav-7-hpq-10.1177_13591053221081905 – for Men’s and women’s endorsement of hegemonic masculinity and responses to COVID-19 Click here for additional data file. sj-sav-7-hpq-10.1177_13591053221081905 for Men’s and women’s endorsement of hegemonic masculinity and responses to COVID-19 by Nathaniel EC Schermerhorn and Theresa K Vescio in Journal of Health Psychology This article is distributed under the terms of the Creative
Commons Attribution 4.0 License ( https://creativecommons.org/licenses/by/4.0/) which
permits any use, reproduction and distribution of the work without
further permission provided the original work is attributed as specified
on the SAGE and Open Access pages ( https://us.sagepub.com/en-us/nam/open-access-at-sage).

sj-sav-9-hpq-10.1177_13591053221081905 – for Men’s and women’s endorsement of hegemonic masculinity and responses to COVID-19 Click here for additional data file. sj-sav-9-hpq-10.1177_13591053221081905 for Men’s and women’s endorsement of hegemonic masculinity and responses to COVID-19 by Nathaniel EC Schermerhorn and Theresa K Vescio in Journal of Health Psychology This article is distributed under the terms of the Creative
Commons Attribution 4.0 License ( https://creativecommons.org/licenses/by/4.0/) which
permits any use, reproduction and distribution of the work without
further permission provided the original work is attributed as specified
on the SAGE and Open Access pages ( https://us.sagepub.com/en-us/nam/open-access-at-sage).

sj-sps-10-hpq-10.1177_13591053221081905 – for Men’s and women’s endorsement of hegemonic masculinity and responses to COVID-19 Click here for additional data file. sj-sps-10-hpq-10.1177_13591053221081905 for Men’s and women’s endorsement of hegemonic masculinity and responses to COVID-19 by Nathaniel EC Schermerhorn and Theresa K Vescio in Journal of Health Psychology This article is distributed under the terms of the Creative
Commons Attribution 4.0 License ( https://creativecommons.org/licenses/by/4.0/) which
permits any use, reproduction and distribution of the work without
further permission provided the original work is attributed as specified
on the SAGE and Open Access pages ( https://us.sagepub.com/en-us/nam/open-access-at-sage).

sj-sps-12-hpq-10.1177_13591053221081905 – for Men’s and women’s endorsement of hegemonic masculinity and responses to COVID-19 Click here for additional data file. sj-sps-12-hpq-10.1177_13591053221081905 for Men’s and women’s endorsement of hegemonic masculinity and responses to COVID-19 by Nathaniel EC Schermerhorn and Theresa K Vescio in Journal of Health Psychology This article is distributed under the terms of the Creative
Commons Attribution 4.0 License ( https://creativecommons.org/licenses/by/4.0/) which
permits any use, reproduction and distribution of the work without
further permission provided the original work is attributed as specified
on the SAGE and Open Access pages ( https://us.sagepub.com/en-us/nam/open-access-at-sage).

sj-sps-6-hpq-10.1177_13591053221081905 – for Men’s and women’s endorsement of hegemonic masculinity and responses to COVID-19 Click here for additional data file. sj-sps-6-hpq-10.1177_13591053221081905 for Men’s and women’s endorsement of hegemonic masculinity and responses to COVID-19 by Nathaniel EC Schermerhorn and Theresa K Vescio in Journal of Health Psychology This article is distributed under the terms of the Creative
Commons Attribution 4.0 License ( https://creativecommons.org/licenses/by/4.0/) which
permits any use, reproduction and distribution of the work without
further permission provided the original work is attributed as specified
on the SAGE and Open Access pages ( https://us.sagepub.com/en-us/nam/open-access-at-sage).

sj-sps-8-hpq-10.1177_13591053221081905 – for Men’s and women’s endorsement of hegemonic masculinity and responses to COVID-19 Click here for additional data file. sj-sps-8-hpq-10.1177_13591053221081905 for Men’s and women’s endorsement of hegemonic masculinity and responses to COVID-19 by Nathaniel EC Schermerhorn and Theresa K Vescio in Journal of Health Psychology This article is distributed under the terms of the Creative
Commons Attribution 4.0 License ( https://creativecommons.org/licenses/by/4.0/) which
permits any use, reproduction and distribution of the work without
further permission provided the original work is attributed as specified
on the SAGE and Open Access pages ( https://us.sagepub.com/en-us/nam/open-access-at-sage).

## Notes

1. The studies were not preregistered, and we provide details of the a-priori power analyses in supplemental materials. Seven participants were removed from Study 1a for failing an attention check. No participants were removed from Study 1b nor from Studies 2a and 2b.

2. In contrast, when examining political leaders as a group (i.e. Republican Congress, Democratic Congress, State leaders), HM was only associated with evaluations of Republican Congress in Study 1b. Full results for these outcome variables are presented in supplemental materials.
