# Supplementary material for: Men’s and women’s endorsement of hegemonic masculinity and responses to COVID-19
Source: J Health Psychol. 2022 Mar 11;28(3):251–66. doi: 10.1177/13591053221081905 (PMC9982413; doi:10.1177/13591053221081905)
Supplement: sj-docx-13-hpq-10.1177_13591053221081905 – Supplemental material for Men’s and women’s endorsement of hegemonic masculinity and responses to COVID-19 [file sj-docx-13-hpq-10.1177_13591053221081905.docx]

Supplemental Materials for

**Men’s and Women’s Endorsement of Hegemonic Masculinity and Reponses to COVID-19**

Nathaniel E.C. Schermerhorn & Theresa K. Vescio

**Contents:**

[Details of A-Priori Power Analysis](#Power)

Table S1a: [Demographic information for all studies](#Demographic)

Table S1b: [Correlations between HM and demographic predictors](#Table1b)

Table S2: [*Factor Analysis for Risk-Taking During COVID-19 Items, Studies 1a and 1b*](#Power)

Table S3: [*Descriptive statistics for all measures in Studies 1a and 1b*](#Table1)

Table S4: [*Descriptive statistics for all measures in Studies 2a and 2b*](#Table3)

Table S5: [*Percentage of participants reporting personal experiences with COVID-19, Studies 1a & 1b*](#table4)

Main analyses including political party:

Table S6: [*Results of Hierarchical Regressions for Evaluations of Political Leaders’ Responses to COVID-19, Studies 1a and 1b (with all interactions)*](#S6)

Table S7: [*Results of Hierarchical Regressions for Risk-Taking During and Perceived Personal Impact of COVID-19, Studies 1a and 1b (with all interactions)*](#S7)

Table S8: [*Results of Hierarchical Regressions for Risk-Taking,COVID-19 Mandates, and Belief in COVID Conspiracies, Studies 2a and 2b (with all interactions)*](#S8)

Table S9: [*Results of hierarchical regressions for evaluations of Republican and Democratic leaders’ response to COVID-19, Studies 1a and 1b*](#SS9)

[Results including precarious masculinity, Study 1b](#DiscussionPM)

Table S10. [*Results of hierarchical regressions for evaluations of political leaders’ responses to COVID-19, Study 1b including precarious masculinity*](#TS10)

Table S11. [*Results of hierarchical regressions for risk-taking during and perceived personal impact of COVID-19, Study 1b including precarious masculinity*](#TS11)

Table S12: [*Results from Study 1b, men only*](#TS12)

Table S13: [*Results of hierarchical regressions for perceived personal impact of COVID-19, Studies 2a and 2b*](#TS13)

Table S14: [*Results from Study 2a including national identity*](#TS14)

Table S15: [*Results from Study 2b including national identity*](#TS15)

[A Note on Analyses Including National Identity](#NationalNote)

Supplemental analyses including political ideology:

[Discussion of results including political ideology](#Ideology)

Tables S16 – S25: [All analyses including political ideology](#TS16)

[References](#References)

**A-Priori Power Analysis: Calculations Using Effects from Vescio & Schermerhorn, 2021**

**Smallest Effect Size Detected:**

ΔR^2^ = .020

Cumulative R^2^ = .409

Effect size f^2^ = .03384

**A:** $\boldsymbol{Y}_{\boldsymbol{i}}\boldsymbol{=}\boldsymbol{\beta}_{\boldsymbol{0}}\boldsymbol{+}\boldsymbol{\beta}_{\boldsymbol{1}}\mathbf{PoliticalParty}_{\mathbf{i}}\boldsymbol{+}\boldsymbol{\beta}_{\boldsymbol{2}}\mathbf{Gender}_{\boldsymbol{i}}\boldsymbol{+}\boldsymbol{\beta}_{\boldsymbol{1}}\mathbf{Race}_{\boldsymbol{i}}\boldsymbol{+}\boldsymbol{\beta}_{\boldsymbol{1}}\mathbf{Education}_{\boldsymbol{i}}\boldsymbol{+}\boldsymbol{\beta}_{\boldsymbol{1}}\mathbf{HM}_{\boldsymbol{i}}\boldsymbol{+}\boldsymbol{\varepsilon}_{\boldsymbol{i}}$

**A:** $\boldsymbol{Y}_{\boldsymbol{i}}\boldsymbol{=}\boldsymbol{\beta}_{\boldsymbol{0}}\boldsymbol{+}\boldsymbol{\beta}_{\boldsymbol{1}}\mathbf{PoliticalParty}_{\mathbf{i}}\boldsymbol{+}\boldsymbol{\beta}_{\boldsymbol{2}}\mathbf{Gender}_{\boldsymbol{i}}\boldsymbol{+}\boldsymbol{\beta}_{\boldsymbol{1}}\mathbf{Race}_{\boldsymbol{i}}\boldsymbol{+}\boldsymbol{\beta}_{\boldsymbol{1}}\mathbf{Education}_{\boldsymbol{i}}\boldsymbol{+}\boldsymbol{\varepsilon}_{\boldsymbol{i}}$

Total Predictors: 5

Tested Predictors: 1

^*^A-priori power analysis in G^*^Power revealed a required sample size of 234 for 80% power at the alpha level of .05.

**Largest Effect Size Detected:**

ΔR^2^ = .072

Cumulative R^2^ = .392

Effect size f^2^ = .11842

**A:** $\boldsymbol{Y}_{\boldsymbol{i}}\boldsymbol{=}\boldsymbol{\beta}_{\boldsymbol{0}}\boldsymbol{+}\boldsymbol{\beta}_{\boldsymbol{1}}\mathbf{PoliticalParty}_{\mathbf{i}}\boldsymbol{+}\boldsymbol{\beta}_{\boldsymbol{2}}\mathbf{Gender}_{\boldsymbol{i}}\boldsymbol{+}\boldsymbol{\beta}_{\boldsymbol{1}}\mathbf{Race}_{\boldsymbol{i}}\boldsymbol{+}\boldsymbol{\beta}_{\boldsymbol{1}}\mathbf{Education}_{\boldsymbol{i}}\boldsymbol{+}\boldsymbol{\beta}_{\boldsymbol{1}}\mathbf{PMI}_{\boldsymbol{i}}\boldsymbol{+}\boldsymbol{\beta}_{\boldsymbol{1}}\mathbf{HM}_{\boldsymbol{i}}\boldsymbol{+}\boldsymbol{\varepsilon}_{\boldsymbol{i}}$

**A:** $\boldsymbol{Y}_{\boldsymbol{i}}\boldsymbol{=}\boldsymbol{\beta}_{\boldsymbol{0}}\boldsymbol{+}\boldsymbol{\beta}_{\boldsymbol{1}}\mathbf{PoliticalParty}_{\mathbf{i}}\boldsymbol{+}\boldsymbol{\beta}_{\boldsymbol{2}}\mathbf{Gender}_{\boldsymbol{i}}\boldsymbol{+}\boldsymbol{\beta}_{\boldsymbol{1}}\mathbf{Race}_{\boldsymbol{i}}\boldsymbol{+}\boldsymbol{\beta}_{\boldsymbol{1}}\mathbf{Education}_{\boldsymbol{i}}\boldsymbol{+}\boldsymbol{\varepsilon}_{\boldsymbol{i}}$

Total Predictors: 6

Tested Predictors: 2

^*^A-priori power analysis in G^*^Power revealed a required sample size of 85 for 80% power at the alpha level of .05.

Therefore, we sought to recruit a minimum of 85 participants per study; in Study 2b, we used Prolific’s recruitment tools to sample an equal number of men/women and Democrats/Republicans.

**Table S1a**

*Demographic information for participants in all studies*

|  | **Study 1a** | | **Study 1b** | | **Study 2a** | | **Study 2b** |
| --- | --- | --- | --- | --- | --- | --- | --- |
| **Age** | 19.76  (Range: 18-36) | 19.05  (Range: 18–41) | | 18.84  (Range: 18-47) | | 36.74  (Range: 18-68) | |
| **Gender** |  |  | |  | |  | |
| Male | 80 (44.9%) | 119  (49.4%) | | 94  (50.0%) | | 97  (49.0%) | |
| Female | 98 (55.1%) | 120  (49.8%) | | 94  (50.0%) | | 99  (50.0%) | |
| Not Listed | 0  (0.0%) | 2  (0.8%) | | 0  (0.0%) | | 0  (1.0%) | |
| **Race** |  |  | |  | |  | |
| White | 139 (78.1%) | 181  (75.1%) | | 132  (70.2%) | | 159  (80.4%) | |
| Black | 11  (6.2%) | 14  (5.8%) | | 10  (5.3%) | | 8  (4.0%) | |
| Asian | 12  (6.7%) | 24  (10.0%) | | 25  (13.3%) | | 14  (7.1%) | |
| Hispanic | 8  (4.5%) | 9  (3.7%) | | 10  (5.3%) | | 9  (4.5%) | |
| Native American | 0  (0.0%) | 0  (0.0%) | | 1  (0.5%) | | 0  (0.0%) | |
| Biracial | 4  (2.2%) | 7  (2.9%) | | 5  (2.7&) | | 3  (1.5%) | |
| Multiracial | 4  (2.2%) | 5  (2.1%) | | 2  (1.1%) | | 5  (2.5%) | |
| Not Listed | 0  (0.0%) | 1  (0.4%) | | 3  (1.6%) | | 0  (0.0%) | |
| **Socioeconomic Status** |  |  | |  | |  | |
| Poor | 1  (0.6%) | 4  (1.7%) | | 1  (0.5%) | | 14  (7.1%) | |
| Working Class | 24  (13.5%) | 17  (7.1%) | | 19  (10.1%) | | 62  (31.3%) | |
| Middle Class | 65  (36.5%) | 117  (48.5%) | | 88  (46.8%) | | 88  (44.4%) | |
| Upper Middle Class | 75  (42.1%) | 83  (34.4%) | | 73  (38.8%) | | 32  (16.2%) | |
| Upper Class | 13  (7.3%) | 20  (8.3%) | | 7  (3.7%) | | 2  (1.0%) | |
| **Political Party** |  |  | |  | |  | |
| Democrat | 42  (23.6%) | 73  (30.3%) | | 32  (17.0%) | | 62  (31.3%) | |
| Democrat Leaning | 30  (16.9%) | 43  (17.8%) | | 53  (28.2%) | | 33  (16.7%) | |
| Independent | 40  (22.5%) | 58  (24.1%) | | 49  (26.1%) | | 9  (4.5%) | |
| Republican Leaning | 40  (22.5%) | 36  (14.9%) | | 36  (19.1%) | | 58  (29.3%) | |
| Republican | 26  (14.6%) | 31  (12.9%) | | 18  (9.6%) | | 36  (18.2%) | |

**Table S1b**

*Correlations between hegemonic masculinity and each demographic variable, all studies*

|  | Political Party | Political Ideology | Gender | Race | Socioeconomic Status |
| --- | --- | --- | --- | --- | --- |
| Hegemonic Masculinity | .453^***^  [.396, .507] | .553^***^  [.502, .599] | .420^***^  [.360, .476] | .013  [-.057, .083] | .145^***^  [.076, .213] |

*Note.* Correlations calculated using a mini meta-analysis approach across the four studies (Goh et al., 2016).
^*^*p* < .05, ^**^*p* < .01, ^***^*p* < .001

**Table S2**

Factor analyses for COVID-19 risk-taking scale, Studies 1a and 1b

|  | **Study 1a** | | **Study 1b** | |
| --- | --- | --- | --- | --- |
|  | Factor loading | | Factor loading | |
| Risk-Taking Item | 1 | 2 | 1 | 2 |
| **Factor 1: Rule-Based Risks** |  |  |  |  |
| 5. Playing a pick-up sport with friends | **.81** | .02 | **.68** | .07 |
| 6. Continuing to have friends over who do not live with you. | **.75** | -.05 | **.60** | .13 |
| 12. Hugging friends you run into while grocery shopping. | **.75** | .19 | **.66** | .29 |
| 1. Defending to family and close friends the belief that COVID- 19 is a serious risk. (R) | **-.63** | **.34** | **-.61** | .15 |
| 16. Leaving your home when you feel ill. | **.62** | **.33** | **.53** | .26 |
| 11. Refusing to shake hands with acquaintances. (R) | **-.60** | .21 | **-.68** | .10 |
| 18. Grocery shopping during hours reserved for high-risk individuals. | **.58** | .23 | **.38** | .20 |
| 10. Not washing your hands upon reentry to your home. | **.57** | .10 | **.53** | .05 |
| 9. Not wearing a mask when you go out in public. | **.55** | -.06 | **.70** | .03 |
| 4. Cancelling a planned vacation because you were planning to fly there (R). | **-.52** | .08 | **-.60** | -.03 |
| 17. Going out in public for a break from caretaking for a friend/family member with coronavirus. | **.50** | .11 | **.36** | .29 |
| 3. Defending the need to self-quarantine on social media. (R) | **-.48** | **.45** | **-.52** | **.40** |
| 2. Disagreeing with your boss’s decision that you should work remotely. | **.47** | .09 | **.55** | .17 |
| 8. Asking the person behind you in line to step away to maintain a six-foot distance. (R) | **-.46** | **.35** | **-.62** | .23 |
| 7. Regularly going out to pick up take-out food. | **.41** | .00 | **.35** | .06 |
| **Factor 2: Helping Risks** |  |  |  |  |
| 15. Distributing medical supplies to the homes of people who have been diagnosed with coronavirus, so they do not have to go out in public. | -.13 | **.79** | -.23 | **.76** |
| 13. Volunteering to distribute food at the local food bank. | -.06 | **.79** | -.15 | **.80** |
| 14. Volunteering at an understaffed medical facility. | .09 | **.78** | -.02 | **.77** |

*Note*. The extraction method for our factor analyses was a principal axis factoring with a varimax rotation. Factor loadings above .30 are in both and reverse-scored items are denoted with (R).

**Table S3**

*Descriptive statistics for all measures in Studies 1a and 1b*

|  | Study 1a | | | Study 1b | | |
| --- | --- | --- | --- | --- | --- | --- |
| Measure | *M* | *SD* | Range | *M* | *SD* | Range |
| **Masculinity** |  |  |  |  |  |  |
| Hegemonic Masc. | 3.47 | 0.91 | 1.27 – 5.88 | 3.26 | 0.87 | 1.08 – 5.35 |
| Precarious Masc. | - | - | - | 2.46 | 0.64 | 1.00 – 2.46 |
| **Political** |  |  |  |  |  |  |
| Trump Approval | 3.53 | 2.13 | 1 – 7 | 3.15 | 2.11 | 1 - 7 |
| **Responses to COVID-19** |  |  |  |  |  |  |
| Trump | 3.43 | 2.15 | 1 – 7 | 3.04 | 2.06 | 1 - 7 |
| McConnell | 3.42 | 1.32 | 1 - 7 | 3.19 | 1.54 | 1 – 7 |
| Rep. Congress | 3.78 | 1.64 | 1 - 7 | 3.37 | 1.74 | 1 – 7 |
| Pelosi | 3.42 | 1.58 | 1 - 7 | 3.51 | 1.63 | 1 – 7 |
| Dem. Congress | 4.09 | 1.51 | 1 - 7 | 4.21 | 1.60 | 1 – 7 |
| Biden | 3.69 | 1.41 | 1 - 7 | 4.49 | 1.79 | 1 – 7 |
| State Leaders | 4.79 | 1.65 | 1 - 7 | 4.05 | 1.60 | 1 – 7 |
| Fauci | 5.26 | 1.55 | 1 - 7 | 5.12 | 1.65 | 1 – 7 |
| **Personal Impact of COVID-19** | | | | | | |
| Psychological | 4.56 | 1.49 | 1 - 7 | 4.66 | 1.79 | 1 – 7 |
| Financial | 4.06 | 1.62 | 1 - 7 | 3.90 | 1.54 | 1 – 7 |
| Personal Threat | 4.70 | 1.44 | 1 - 7 | 4.33 | 1.39 | 1 – 7 |
| **Risk-Taking During COVID-19** | | | | | | |
| Rule-Based | 2.64 | 1.01 | 1.00 – 6.07 | 3.30 | 1.00 | 1.00 – 6.73 |
| Helping | 3.53 | 1.57 | 1 - 7 | 3.80 | 1.51 | 1 - 7 |

**Table S4**

*Descriptive statistics for all measures in Studies 2a and 2b*

|  | Study 2a | | | Study 2b | | |
| --- | --- | --- | --- | --- | --- | --- |
| Measure | *M* | *SD* | Range | *M* | *SD* | Range |
| **Masculinity** |  |  |  |  |  |  |
| Hegemonic Masc. | 3.22 | .85 | 1.27 - 5.42 | 3.30 | 1.03 | 1.23 - 5.81 |
| **Political** |  |  |  |  |  |  |
| National Identity | 5.44 | 1.52 | 1.00 - 7.00 | 5.73 | 1.28 | 1.50 – 7.00 |
| **Personal Impact of COVID-19** | | | | | | |
| Psychological | 4.54 | 1.43 | 1.00 - 7.00 | 4.12 | 1.70 | 1.00 – 7.00 |
| Financial | 3.77 | 1.46 | 1.00 - 7.00 | 3.80 | 1.99 | 1.00 – 7.00 |
| Personal Threat | 3.63 | 1.36 | 1.00 - 7.00 | 4.28 | 1.63 | 1.00 – 7.00 |
| **Risk-Taking During COVID-19** | | | | | | |
| Rule-Based | 3.93 | 1.07 | 1.20 - 6.93 | 3.31 | 1.10 | 1.13 – 5.93 |
| **Responses to COVID-19** |  |  |  |  |  |  |
| COVID-19 Mandates | 4.68 | 1.75 | 1.00 - 7.00 | 4.50 | 2.22 | 1.00 – 7.00 |
| COVID-19 Conspiracies | 1.54 | .63 | 1.00 - 5.00 | 1.71 | 0.65 | 1.00 – 3.67 |

**Table S5**

*Percentage of participants reporting personal experiences with COVID-19, Studies 1a & 1b*

|  | % Yes | |
| --- | --- | --- |
|  | Study 1a | Study 1b |
| I have been diagnosed with COVID-19. | 0.0 | 13.3 |
| I have had COVID-like symptoms at some point in the last two months. | 14.0 | 29.0 |
| I have been sick with something other than COVID-19 in the last two months. | 30.9 | 34.0 |
| I have been in close proximity with someone who has been diagnosed with COVID-19.^*^ | 4.5 | 50.6 |
| I have been in close proximity with someone who has had COVID-like symptoms in the last two months.^*^ | 16.9 | 50.2 |
| I watch a lot of news about COVID-19.^*^ | 65.7 | 59.3 |
| I spend a huge percentage of my time trying to find updates online or on TV about COVID-19. | 20.8 | 21.2 |

*Note.* ^*^ indicates that Study 1b analyses were also conducted controlling for these items.

The inclusion of these variables did not alter the pattern of results reported.

**Table S6**

*Results of Hierarchical Regressions for Evaluations of Political Leaders’ Responses to COVID-19, Studies 1a and 1b*

|  | **Study 1a (April 2020)** | | | | | **Study 1b (December 2020)** | | | | |
| --- | --- | --- | --- | --- | --- | --- | --- | --- | --- | --- |
| **Independent Variables** | **Trump Response** | **McConnell Response** | **Biden Response** | **Pelosi Response** | **Fauci Response** | **Trump Response** | **McConnell Response** | **Biden Response** | **Pelosi Response** | **Fauci Response** |
|  | **ꞵ** | **ꞵ** | **ꞵ** | **ꞵ** | **ꞵ** | **ꞵ** | **ꞵ** | **ꞵ** | **ꞵ** | **ꞵ** |
| **Step 1: R^2^** | **.512^***^** | **.249^***^** | **.075^**^** | **.274^***^** | **.022*^t^*** | **.606^***^** | **.315^***^** | **.494^***^** | **.384^***^** | **.114^***^** |
| Political Party | .72^***^ | .50^***^ | -.27^**^ | -.52^***^ | -.15*^t^* | .75^***^ | .56^***^ | -.70^***^ | -.62^***^ | -.34^***^ |
| **Step 2: ∆ R^2^** | **.013** | **.015** | **.009** | **.016** | **.015** | **.011** | **.027*^t^*** | **.005** | **.019** | **.018** |
| Political Party | .70^***^ | .50^***^ | -.29^**^ | -.50^***^ | -.18^*^ | .75^***^ | .59^***^ | -.70^***^ | -60^***^ | -.37^***^ |
| Gender | .00 | -.08 | .01 | .00 | .11 | .03 | -.16^**^ | -.03 | .12^*^ | .01 |
| Race | .11^*^ | .06 | .07 | -.13 | .08 | .08*^t^* | .04 | .01 | .05 | .10 |
| SES | .04 | .08 | .06 | -.01 | -.02 | -.07 | -.01 | .07 | -.05 | .09 |
| **Step 3: ∆ R^2^** | **.012^**^** | **.026^*^** | **.009** | **.001** | **.000** | **.024^***^** | **.037^***^** | **.012^*^** | **.020^*^** | **.023^*^** |
| Political Party | .61^***^ | .39^***^ | -.35^**^ | -.47^***^ | -.18 | .68^***^ | .50^***^ | -.65^***^ | -.53^***^ | -.30^***^ |
| Gender | -.07 | -.16*^t^* | -.03 | .01 | .11 | -.03 | -.24^***^ | .02 | -.06 | .08 |
| Race | .11^*^ | .06 | .08 | -.13 | .08 | .10^*^ | .06 | -.01 | .04 | .08 |
| SES | .02 | .07 | .05 | -.01 | -.02 | -.09^*^ | -.03 | .08 | -.04 | .11 |
| HM | .19^**^ | .22^*^ | .13 | -.05 | .00 | .19^***^ | .24^***^ | -.14^*^ | -.17^*^ | -.19^*^ |
| **Step 4: ∆ R^2^** | **.009** | **.040** | **.020** | **.008** | **.015** | **.007** | **.029*^t^*** | **.004** | **.005** | **.020** |
| Political Party | .61^***^ | .42^***^ | -.34^**^ | -.48^***^ | -.19*^t^* | .66^***^ | .46^***^ | -.64^***^ | -.52^***^ | -.30^***^ |
| Gender | -.08 | -.18^*^ | -.05 | .02 | .12 | -.03 | -.23^**^ | .00 | -.08 | .06 |
| Race | .11^*^ | .07 | .08 | -.14 | .07 | .11^*^ | .08 | -.02 | .03 | .06 |
| SES | .02 | .05 | .05 | -.01 | -.02 | -.08^*^ | -.03 | .08 | -.04 | .11 |
| HM | .23^*^ | .31^*^ | .19 | -.08 | -.02 | .16^**^ | .16*^t^* | -.11 | -.15*^t^* | -.15 |
| HM^*^Party | .04 | -.13 | -.10 | .06 | .10 | .02 | .05 | .03 | .01 | .06 |
| HM^*^Gender | .06 | .06 | .00 | -.01 | -.03 | .06 | .00 | -.06 | -.06 | -.14^*^ |
| HM^*^Race | -.06 | -.19 | -.12 | .06*^t^* | .03 | .05 | .16^*^ | -.04 | -.02 | -.03 |
| HM^*^SES | .02 | .07 | .02 | -.06 | .05 | .02 | -.08 | -.01 | .03 | .02 |

*Note.* SES = socioeconomic status; HM = hegemonic masculinity

*^t^ p* < .075. *^*^p* < .05. *^**^* *p* < .01. *^***^* *p* < .001

**Table S7**

*Results of Hierarchical Regressions for Risk-Taking During and Perceived Personal Impact of COVID-19, Studies 1a and 1b*

|  | **Study 1a (April 2020)** | | | | | **Study 1b (December 2020)** | | | | |
| --- | --- | --- | --- | --- | --- | --- | --- | --- | --- | --- |
|  | **Risk-Taking** | | **Personal Impact** | | | **Risk-Taking** | | **Personal Impact** | | |
| **Independent Variables** | **Rule-Based**  **Risks** | **Helping**  **Risks** | **Personal**  **Threat** | **Psych.**  **Impact** | **Financial**  **Impact** | **Rule-Based**  **Risks** | **Helping**  **Risks** | **Personal**  **Threat** | **Psych. Impact** | **Financial**  **Impact** |
|  | **ꞵ** | **ꞵ** | **ꞵ** | **ꞵ** | **ꞵ** | **ꞵ** | **ꞵ** | **ꞵ** | **ꞵ** | **ꞵ** |
| **Step 1: R^2^** | **.089^***^** | **.000** | **.074^***^** | **.021*^t^*** | **.002** | **.179^***^** | **.028^**^** | **.189^***^** | **.068^***^** | **.016*^t^*** |
| Political Party | .30^***^ | .00 | -.27^***^ | -.14*^t^* | .05 | .42^***^ | -.17^**^ | -.43^***^ | -.26^***^ | -.13*^t^* |
| **Step 2: ∆ R^2^** | **.114^***^** | **.042*^t^*** | **.142^***^** | **.068^**^** | **.133^***^** | **.016** | **.050^**^** | **.023** | **.017** | **.032*^t^*** |
| Political Party | .22^**^ | .02 | -.22^**^ | -.12 | .05 | .38^***^ | -.10 | -.38^***^ | -.22^**^ | -.09 |
| Gender | .33^***^ | -.18^*^ | -.38^***^ | -.25^**^ | -.03 | .12^*^ | -.08 | -.15^*^ | -.13^*^ | -.12*^t^* |
| Race | .11 | .09 | .01 | .08 | .21^**^ | .05 | -.16^*^ | -.05 | -.03 | -.02 |
| SES | -.04 | -.05 | .11 | .04 | -.31^***^ | -.02 | -.14^*^ | .00 | .03 | -.13^*^ |
| **Step 3: ∆ R^2^** | **.051^**^** | **.005** | **.002** | **.044^**^** | **.000** | **.098^***^** | **.000** | **.031^**^** | **.014^.061^** | **.005** |
| Political Party | .09 | -.03 | -.19^*^ | .01 | .06 | .22^***^ | -.11 | -.30^***^ | -.16^*^ | -.05 |
| Gender | .22^**^ | -.21^*^ | -.36^***^ | -.15*^t^* | -.02 | .00 | -.09 | -.08 | -.09 | -.09 |
| Race | .12 | .09 | .00 | .07 | .21^**^ | .09 | -.16^*^ | -.07 | -.04 | -.03 |
| SES | -.05 | -.05 | .11 | .06 | -.31^***^ | -.06 | -.14^*^ | .02 | .05 | -.12*^t^* |
| HM | .30^**^ | .10 | -.06 | -.28^**^ | -.03 | .38^***^ | .03 | -.22^**^ | -.14*^t^* | -.09 |
| **Step 4: ∆ R^2^** | **.016** | **.040** | **.007** | **.038** | **.084^**^** | **.016** | **.017** | **.036^*^** | **.008** | **.042^*^** |
| Political Party | .09 | -.02 | -.19^*^ | .02 | .06 | .20^**^ | -.08 | -.27^***^ | -.16^*^ | -.05 |
| Gender | .22^**^ | -.21^*^ | -.35^***^ | -.06^*^ | .00 | .02 | -.11 | -.11 | -.09 | -.12 |
| Race | .13*^t^* | .10 | .01 | .06 | .21^**^ | .10 | -.16^*^ | -.08 | -.05 | -.02 |
| SES | -.04 | -.03 | .12 | .06 | -.27^**^ | -.05 | -.15^*^ | .01 | .05 | -.12*^t^* |
| HM | .24^*^ | .05 | -.05 | -.15 | -.06 | .31^***^ | .08 | -.11 | -.11 | -.03 |
| HM^*^Party | -.05 | .08 | -.04 | .10 | -.01 | -.02 | -.03 | .04 | .05 | -.04 |
| HM^*^Gender | .04 | .10 | -.04 | .03 | -.18^*^ | .03 | .01 | .01 | .04 | .09 |
| HM^*^Race | .10 | .07 | -.03 | -.21^*^ | -.01 | .12*^t^* | -.11 | -.17^*^ | -.09 | -.06 |
| HM^*^SES | .08 | .08 | .07 | .08 | .27^***^ | -.06 | .08 | .13^*^ | -.01 | .19^**^ |

*Note.* SES = socioeconomic status; HM = hegemonic masculinity

*^t^ p* < .075. *^*^p* < .05. *^**^* *p* < .01. *^***^* *p* < .001.

**Table S8**

*Results of Hierarchical Regressions for Risk-Taking,COVID-19 Mandates, and Belief in COVID Conspiracies, Studies 2a and 2b*

|  | **Study 2a** | | | **Study 2b** | | |
| --- | --- | --- | --- | --- | --- | --- |
| **Independent Variables** | **Rule-Based**  **Risks** | **Mandates** | **Conspiracies** | **Rule-Based**  **Risks** | **Mandates** | **Conspiracies** |
|  | **ꞵ** | **ꞵ** | **ꞵ** | **ꞵ** | **ꞵ** | **ꞵ** |
| **Step 1: R^2^** | **.265^***^** | **.300^***^** | **.130^***^** | **.284^***^** | **.526^***^** | **.216^***^** |
| Political Party | .52^***^ | -.55^***^ | .36^***^ | .53^***^ | -.73^***^ | .47^***^ |
| **Step 2: ∆ R^2^** | **.072^***^** | **.068^***^** | **.017** | **.016** | **.002** | **.002** |
| Political Party | .44^***^ | -.54^***^ | .36^***^ | .53^***^ | -.73^***^ | .47^***^ |
| Gender | .22^**^ | -.23^***^ | .05 | .12^*^ | .01 | .03 |
| Race | .17^**^ | -.10 | -.08 | -.02 | .02 | -.01 |
| SES | .09 | .13^*^ | .09 | -.02 | .04 | -.03 |
| **Step 3: ∆ R^2^** | **.068^***^** | **.027^**^** | **.045^**^** | **.028^**^** | **.007** | **.122^***^** |
| Political Party | .33^***^ | -.47^***^ | .27^***^ | .45^***^ | -.68^***^ | .285^***^ |
| Gender | .07 | -.14^*^ | -.07 | .06 | .04 | -.09 |
| Race | .16^**^ | -.10 | -.08 | .01 | .00 | .06 |
| SES | .06 | .15^*^ | .07 | -.04 | .05 | -.07 |
| HM | .32^***^ | -.21^**^ | .26^**^ | .20^**^ | -.10 | .12^***^ |
| **Step 4: ∆ R^2^** | **.008** | **.016** | **.081^**^** | **.010** | **.007** | **.004** |
| Political Party | .31^***^ | -.44^***^ | .25^**^ | .44^***^ | -.68^***^ | .28^***^ |
| Gender | .08 | -.14^*^ | -.05 | .06 | .05 | -.08 |
| Race | .16^*^ | -.10 | -.09 | -.01 | -.01 | .07 |
| SES | .05 | .15^*^ | .04 | -.04 | .06 | -.06 |
| HM | .32^***^ | -.19^*^ | .25^**^ | .17^*^ | -.07 | .44^***^ |
| HM^*^Party | .07 | -.08 | .21^**^ | .04 | .08 | .01 |
| HM^*^Gender | -.03 | .00 | .13*^t^* | .04 | -.01 | -.02 |
| HM^*^Race | .05 | -.08 | .08 | .09 | -.04 | -.03 |
| HM^*^SES | -.01 | -.02 | -.11 | -.04 | .05 | -.05 |

*Note.* SES = socioeconomic status; HM = hegemonic masculinity

*^t^ p* < .075. *^*^p* < .05. *^**^* *p* < .01. *^***^* *p* < .001.

**Table S****9**

*Results of Hierarchical Regressions for Evaluations of Republican and Democratic Leaders’ Response to COVID-19, Studies 1a and 1b*

|  | **Study 1a** | | | **Study 1b** | | |
| --- | --- | --- | --- | --- | --- | --- |
| **Independent Variables** | **Republican**  **Congress** | **Democratic Congress** | **State Leaders** | **Republican Congress** | **Democratic Congress** | **State Leaders** |
|  | **ꞵ** | **ꞵ** | **ꞵ** | **ꞵ** | **ꞵ** | **ꞵ** |
| **Step 1: R^2^** | **.443^***^** | **.181^***^** | **.024^*^** | **.491^***^** | **.373^***^** | **.113^***^** |
| Political Party | .67^***^ | -.43^***^ | -.16^*^ | .70^***^ | -.61^***^ | -.34^***^ |
| **Step 2: ∆ R^2^** | **.060^**^** | **.003** | **.005** | **.024^*^** | **.003** | **.001** |
| Political Party | .68^***^ | -.42^***^ | -.15*^t^* | .65^***^ | -.61^***^ | -.32^***^ |
| Gender | -.18^**^ | -.04 | .04 | .02 | -.05 | -.01 |
| Race | .16^**^ | .04 | -.06 | .16^**^ | .03 | -.04 |
| SES | .02 | -.01 |  | -.01 | .01 | .00 |
| **Step 3: ∆ R^2^** | **.011** | **.001** | **.001** | **.014^*^** | **.007** | **^.^000** |
| Political Party | .60^***^ | -.40^***^ | -.13 | .60^***^ | -.57^***^ | -.32^***^ |
| Gender | -.22^**^ | -.02 | .00 | -.03 | -.01 | -.01 |
| Race | .17^**^ | .04 | .04 | .17^**^ | .03 | -.04 |
| SES | .01 | -.01 | -.06 | -.02 | .01 | .00 |
| HM | .14 | -.04 | -.04 | .14^*^ | -.10 | -.01 |
| **Step 4: ∆ R^2^** | **.018** | **.039** | **.040** | **.014** | **.026*^t^*** | **.036*^t^*** |
| Political Party | .61^***^ | -.39^***^ | -.12 | .57^***^ | -.55^***^ | -.33^***^ |
| Gender | -.24^**^ | -.04 | -.01 | -.03 | -.04 | -.05 |
| Race | .18^**^ | .05 | .03 | .17^**^ | .01 | -.06 |
| SES | .01 | -.03 | -.06 | -.01 | .02 | .02 |
| HM | .22^*^ | .09 | .11 | .11 | -.06 | .03 |
| HM^*^Party | -.01 | .03 | .08 | .06 | .03 | .09 |
| HM^*^Gender | .00 | .07 | -.05 | .01 | -.17^**^ | -.17^*^ |
| HM^*^Race | -.12 | -.23^*^ | -.25^*^ | .08 | -.01 | .05 |
| HM^*^SES | .12*^t^* | -.01 | .03 | -.06 | .02 | .04 |

*Note.* SES = socioeconomic status; HM = hegemonic masculinity

*^t^ p* < .075. *^*^p* < .05. *^**^* *p* < .01. *^***^* *p* < .001

**Discussion of Precarious Masculinity in Study 1b**

As noted in the main text, Study 1b including a measure of precarious masculinity to examine if men’s stress at the failure to live up to the standards of hegemonic masculinity would predict their COVID-related responses and their increased likelihood to take COVID-related risks.

**Precarious Masculinity (PM).** In Study 1b only, participants completed the 40-item Male Gender Role Stress Scale (1 = not at all stressful, 5 = extremely stressful; Eisler & Skidmore, 1987). Participants imagined being unmanly and reported the stress they would feel in response to thoughts of being physical inadequacy (e.g., “appearing less athletic than a friend”), emotional (e.g., “comforting a male friend who is upset”), subordinate to women (e.g., “having a female boss”), intellectually inferior (e.g., “working with people who are more ambitious than you”), and failing to uphold expectations of men (e.g., “not making enough money”). Because the scale is written to measure men’s stress at failing to embody a masculine identity, we asked men for their personal stress at imagining the violations and asked women to imagine how stressed they believed the “typical man” would be when imagining his experience with the violations. We averaged across items to create a PM variable (α = .95), which was reliable for men (α = .93) and women (α = .96).

**Results**

We tested the hypothesis that men with higher precarious masculinity would respond with (1) more positive evaluations of how Republican (vs. Democratic) politicians have responded to COVID-19, (2) a greater likelihood to engage in risky behaviors during COVID-19, (3) less reported personal impact of COVID-19, (4) less agreement with mandates seeking to mitigate the spread of COVID-19, and (5) greater belief in COVID-19 conspiracy theories in two ways. First, we conducted identical hierarchical regressions to those reported in the main text including precarious masculinity in Step 3 (alongside hegemonic masculinity) and examined if the interaction between precarious masculinity and gender was significant (i.e., if precarious masculinity predicted outcomes differently for men vs. women). In addition, we conducted hierarchical regressions for men only (eliminating gender from Step 2).

There was weak and inconsistent evidence that precarious masculinity was associated with evaluations of political leaders’ responses to COVID-19. In fact, the only effect to emerge was that higher precarious masculinity was associated with evaluations of McConnell. This effect emerged when examining the full sample (see Table S7) and when examining men only (see Table S9).

When examining personal impacts of COVID-19 and engagement in risky behaviors, no effects of precarious masculinity emerged for men only (see Table S9). However, when examining both men and women, a significant interaction of hegemonic masculinity and precarious masculinity emerged (see Table S8) on personal threat of COVID-19. Endorsement of hegemonic masculinity was associated with less personal threat of COVID-19 for those low (but not high) in PM [low: *b* = -.56, *t*(221) = -3.13, *p* = .002; high: *b* = .11, *t*(221) = 0.62, *p* = .539].

**Discussion**

When including precarious masculinity, we found that, in contrast to the notion that individual men may be more defiant towards mitigation efforts to preserve their masculine identity, men’s stress at imagining masculine transgressions was not associated with their responses to COVID-19. However, it is important to note that the present work included a measure of chronic (not situational) PM. Therefore, it is possible that situational threats to men’s masculinity (e.g., being told they look feminine for wearing a mask) would elicit and/or amplify negative responses to health guidelines for COVID-19.

**Table S10**

*Results of Hierarchical Regressions for Evaluations of Political Leaders’ Responses to COVID-19, Study 1b Including Precarious Masculinity*

| **Independent Variables** | **Trump Response** | **McConnell Response** | **Biden Response** | **Pelosi Response** | **Fauci Response** | **Republican Congress** | **Democratic Congress** | **State Leaders** |
| --- | --- | --- | --- | --- | --- | --- | --- | --- |
|  | **ꞵ** | **ꞵ** | **ꞵ** | **ꞵ** | **ꞵ** | **ꞵ** | **ꞵ** | **ꞵ** |
| **Step 1: R^2^** | **.605^***^** | **.317^***^** | **.495^***^** | **.384^***^** | **.113^***^** | **.491^***^** | **.373^***^** | **.112^***^** |
| Political Party | .78^***^ | .56^***^ | -.70^***^ | -.62^***^ | -.34^***^ | .70^***^ | -.61^***^ | -.34^***^ |
| **Step 2: ∆ R^2^** | **.011** | **.026*^t^*** | **.005** | **.019** | **.017** | **.024^*^** | **.004** | **.001** |
| Political Party | .75^***^ | .59^***^ | -.70^***^ | -.60^***^ | -.37^***^ | .65^***^ | -.61^***^ | -.32^***^ |
| Gender | .03 | -.16^**^ | -.03 | ^-.12*^ | .01 | .02 | -.05 | -.01 |
| Race | .08*^t^* | .04 | .01 | .05 | .09 | .16^**^ | .04 | -.04 |
| SES | -.07 | -.01 | .07 | -.05 | .09 | -.01 | .01 | .00 |
| **Step 3: ∆ R^2^** | **.026^***^** | **.049^**^** | **.012*^t^*** | **.023^*^** | **.027^*^** | **.014^*^** | **.007** | **.000** |
| Political Party | .68^***^ | .51^***^ | -.65^***^ | -.53^***^ | -.29^***^ | .60^***^ | -.57^***^ | -.32^***^ |
| Gender | -.01 | -.17^*^ | .01 | -.09 | .11 | -.02 | -.01 | .00 |
| Race | .10^*^ | .08 | .00 | .03 | .08 | .17^**^ | .03 | .04 |
| SES | -.08^*^ | -.03 | .08 | -.04 | .11 | -.02 | .02 | .00 |
| PM | .05 | .13^*^ | -.01 | -.06 | .06 | .02 | .01 | .01 |
| HM | .17^**^ | .18^*^ | -.13^*^ | -.15^*^ | -.22^*^ | .13^*^ | -.11 | -.01 |
| **Step 4: ∆ R^2^** | **.016** | **.042** | **.019** | **.022** | **.054** | **.033** | **.031** | **.041** |
| Political Party | .65^***^ | .47^***^ | -.62^***^ | -.53^***^ | -.27^**^ | .55^***^ | -.55^***^ | -.33^***^ |
| Gender | .01 | -.15- | -.01 | -.10 | .08 | -.01 | -.03 | -.05 |
| Race | .11^*^ | .09 | -.03 | .02 | .09 | .18^***^ | .00 | -.05 |
| SES | -.09 | -.03 | .08 | -.03 | .13*^t^* | -.01 | .02 | .01 |
| PMI | .10 | .19^*^ | -.06 | -.02 | .02 | .09 | .02 | -.04 |
| HM | .14^*^ | .08 | -.09 | -.13 | -.18*^t^* | .09 | -.07 | .03 |
| HM^*^Party | .01 | .02 | .07 | .02 | .01 | .02 | .04 | .08 |
| HM^*^Gender | .04 | -.01 | -.10 | -.06 | -.05 | .03 | -.19^*^ | -.15 |
| HM^*^Race | .05 | .19^**^ | -.05 | -.01 | -.02 | .11*^t^* | -.01 | .06 |
| HM^*^SES | .03 | -.05 | -.01 | .02 | .00 | -.05 | .02 | .04 |
| PMI^*^Party | .12*^t^* | .08 | -.18^*^ | -.02 | -.08 | .17^*^ | -.01 | -.02 |
| PMI^*^Gender | .03 | .07 | .04 | .09 | -.20^*^ | -.01 | .07 | -.04 |
| PMI^*^Race | -.07 | -.10 | .09 | -.01 | -.03 | -.13^*^ | .04 | .04 |
| PMI^*^SES | -.02 | -.01 | -.02 | .08 | -.04 | -.02 | .02 | -.06 |
| HM^*^PMI | -.12*^t^* | .00 | .07 | .05 | .14 | -.05 | -.01 | .07 |

*Note.* SES = socioeconomic status; PM = precarious masculinity; HM = hegemonic masculinity

*^t^ p* < .075. *^*^p* < .05. *^**^* *p* < .01. *^***^* *p* < .001

**Table S11**

*Results of Hierarchical Regressions for Risk-Taking During and Perceived Personal Impact of COVID-19, Study 1b Including Precarious Masculinity*

|  | **Risk-Taking** | | **Personal Impact** | | |
| --- | --- | --- | --- | --- | --- |
| **Independent Variables** | **Rule-Based**  **Risks** | **Helping**  **Risks** | **Personal**  **Threat** | **Psych. Impact** | **Financial**  **Impact** |
|  | **ꞵ** | **ꞵ** | **ꞵ** | **ꞵ** | **ꞵ** |
| **Step 1: R^2^** | **.179^***^** | **.028^*^** | **.188^***^** | **.068^***^** | **.016*^t^*** |
| Political Party | .42^***^ | -.17^*^ | -.43^***^ | -.26^***^ | -.13*^t^* |
| **Step 2: ∆ R^2^** | **.017** | **.050^**^** | **.022** | **.018** | **.032*^t^*** |
| Political Party | .38^***^ | -.10 | -.38^***^ | -.22^**^ | -.09 |
| Gender | .12^*^ | -.08 | -.15^*^ | -.13^*^ | -.12*^t^* |
| Race | .05 | -.16^*^ | -.05 | -.03 | -.02 |
| SES | -.02 | -.14^*^ | .00 | .04 | -.13^*^ |
| **Step 3: ∆ R^2^** | **.098^***^** | **.004** | **.039^**^** | **.014** | **.008** |
| Political Party | .22^**^ | -.11 | -.28^***^ | -.16^*^ | -.06 |
| Gender | -.02 | -.12 | -.03 | -.07 | -.12 |
| Race | .08 | -.17^*^ | -.07 | -.04 | -.03 |
| SES | -.06 | -.15^*^ | .03 | .05 | -.13^.051^ |
| PM | -.04 | -.07 | .10 | .03 | -.06 |
| HM | .40^***^ | .05 | -.26^**^ | -.15*^t^* | -.06 |
| **Step 4: ∆ R^2^** | **.029** | **.029** | **.069^**^** | **.009** | **.059** |
| Political Party | .19^**^ | -.08 | -.24^**^ | -.15*^t^* | -.04 |
| Gender | .03 | -.15*^t^* | -.10 | -.09 | -.14 |
| Race | .09 | -.17^*^ | -.07 | -.04 | -.03 |
| SES | -.0 | -.16^*^ | .05 | .06 | -.14^*^ |
| PM | .03 | -.16 | .05 | .02 | -.09 |
| HM | .31^***^ | .11 | -.14 | -.11 | -.02 |
| HM^*^Party | .01 | -.02 | .01 | .04 | .09 |
| HM^*^Gender | -.04 | .03 | .10 | .06 | .02 |
| HM^*^Race | .13*^t^* | -.12 | -.16^*^ | -.09 | -.06 |
| HM^*^SES | -.04 | .07 | .11*^t^* | -.01 | .20^**^ |
| PMI^*^Party | .06 | -.08 | -.13 | -.03 | -.15 |
| PMI^*^Gender | .11 | -.05 | -.12 | -.02 | .12 |
| PMI^*^Race | -.08 | .11 | .05 | .01 | .07 |
| PMI^*^SES | -.03 | -.06 | .07 | .04 | -.05 |
| HM^*^PM | -.12 | .08 | .24^**^ | .05 | .07 |

*Note:* SES = socioeconomic status; PM = precarious masculinity; HM = hegemonic masculinity

*^t^ p* < .075. *^*^p* < .05. *^**^* *p* < .01. *^***^* *p* < .001

HM^*^Race: Endorsement of HM was associated with less personal threat of COVID-19

for White (but not non-White) participants [White: *b* = -.48, *t*(221) = -3.57, *p* < .001;

non-White: *b* = .03, *t*(221) = .16, *p* = .874].

**Table S12**

*Results of Hierarchical Regressions for Study 1b, Males Only*

| **Independent Variables** | **Trump** | **McConnell** | **Biden** | **Pelosi** | **Fauci** | **Risk-Taking** | **Personal Threat** | **Psych. Impact** | **Financial Impact** |
| --- | --- | --- | --- | --- | --- | --- | --- | --- | --- |
|  | **ꞵ** | **ꞵ** | **ꞵ** | **ꞵ** | **ꞵ** | **ꞵ** | **ꞵ** | **ꞵ** | **ꞵ** |
| **Step 1: R^2^** | **.557^***^** | **.404^***^** | **.456^***^** | **.217^***^** | **.148^***^** | **.268^***^** | **.287^***^** | **.092^**^** | **.003** |
| Political Party | .75^***^ | .64^***^ | -.68^***^ | -.47^***^ | -.39^***^ | .52^***^ | -.54^***^ | -.30^**^ | -.059 |
| **Step 2: ∆ R^2^** | **.020*^t^*** | **.028** | **.005** | **.026** | **.003** | **.012** | **.023** | **.006** | **.020** |
| Political Party | .71^***^ | .60^***^ | -.65^***^ | -.45^***^ | -.40^***^ | .48^***^ | -.49^***^ | -.28^**^ | -.04 |
| Race | .15^*^ | .17^*^ | -.07 | -.05 | .06 | .12 | -.15*^t^* | -.08 | -.06 |
| SES | -.01 | .04 | .02 | -.16*^t^* | .03 | .01 | -.06 | .00 | -.13 |
| **Step 3: ∆ R^2^** | **.054^***^** | **.066^**^** | **.028*^t^*** | **.025** | **.071** | **.080^**^** | **.018** | **.003** | **.002** |
| Political Party | .56^***^ | .49^***^ | -.55^***^ | -.36^***^ | -.28^*^ | .31^**^ | -.41^***^ | -.25^*^ | -.02 |
| Race | .20^**^ | .20^*^ | -.11 | -.08 | .00 | .18^*^ | -.18^*^ | -.09 | -.07 |
| SES | -.04 | .02 | .04 | -.14 | .05 | -.02 | -.04 | .01 | -.13 |
| PMI | .04 | .20^*^ | -.01 | -.04 | -.12 | .05 | .01 | .01 | .00 |
| HM | .25^**^ | .13 | -.19^*^ | -.16 | -.22^*^ | .31^**^ | -.16 | -.07 | -.05 |
| **Step 4: ∆ R^2^** | **.008** | **.018** | **.012** | **.026** | **.031** | **.025** | **.059** | **.045** | **.067** |
| Political Party | .53^***^ | .46^***^ | -.60^***^ | -.33^**^ | -.27^*^ | .30^**^ | -.38^***^ | -.24*^t^* | -.06 |
| Race | .17*^t^* | .13 | -.10 | -.02 | .01 | .14 | -.12 | -.14 | .03 |
| SES | -.05 | .03 | .08 | -.29^*^ | -.08 | -.12 | .02 | .20 | -.20 |
| PMI | .10 | .22 | .00 | -.06 | -.02 | .09 | -.07 | -.09 | .02 |
| HM | .21*^t^* | .03 | .18 | -.19 | -.33*^t^* | .17 | .05 | .06 | .07 |
| HM^*^Party | .06 | .03 | .11 | -.06 | .04 | -.03 | .06 | .03 | .14 |
| HM^*^Race | .05 | .19 | -.03 | -.05 | -.04 | .18 | -.17 | .05 | -.22 |
| HM^*^SES | .03 | -.02 | -.03 | .12 | .11 | .07 | .08 | -.15 | .21 |
| PMI^*^Party | .05 | -.07 | -.11 | -.14 | -.12 | -.01 | -.03 | .11 | -.09 |
| PMI^*^Race | -.02 | -.05 | .05 | .07 | -.05 | .01 | .01 | -.11 | .17 |
| PMI^*^SES | .02 | -.03 | .05 | -.20 | -.20 | -.14 | .07 | .15 | .02 |
| HM^*^PMI | -.09 | .11 | .04 | .06 | -.04 | -.03 | .18 | .17 | -.06 |

*Note.* SES = socioeconomic status; PMI = precarious masculine identity; HM = hegemonic masculinity

*^t^ p* < .075. *^*^p* < .05. *^**^* *p* < .01. *^***^* *p* < .001

**Table S13**

*Results of Hierarchical Regressions for Perceived Personal Impact of COVID-19, Studies 2a and 2b*

|  | **Study 2a** | | | **Study 2b** | | |
| --- | --- | --- | --- | --- | --- | --- |
| **Independent Variables** | **Personal**  **Threat** | **Psych. Impact** | **Financial Impact** | **Personal**  **Threat** | **Psych.**  **Impact** | **Financial**  **Impact** |
|  | **ꞵ** | **ꞵ** | **ꞵ** | **ꞵ** | **ꞵ** | **ꞵ** |
| **Step 1: R^2^** | **.243^***^** | **.033^*^** | **.005** | **.235^***^** | **.125^***^** | **.001** |
| Political Party | -.49^***^ | -.18^*^ | -.07 | -.49^***^ | -.35^***^ | -.03 |
| **Step 2: ∆ R^2^** | **.090^***^** | **.019** | **.123^***^** | **.044^*^** | **.093^***^** | **.115^***^** |
| Political Party | -.45^***^ | -.19^*^ | .02 | .48^***^ | -.36^***^ | -.03 |
| Gender | -.30^***^ | -.12 | -.10 | -.20^**^ | -.24^***^ | -.19^**^ |
| Race | -.12*^t^* | .06 | -.06 | .03 | .11 | .04 |
| SES | .00 | -.03 | -.33^***^ | -.03 | -.12*^t^* | -.26^***^ |
| **Step 3: ∆ R^2^** | **.013*^t^*** | **.013** | **.006** | **.002** | **.001** | **.009** |
| Political Party | -.40^***^ | -.14 | .05 | -.46^***^ | -.38^***^ | -.08 |
| Gender | -.23^**^ | -.06 | -.06 | -.18^**^ | -.25^***^ | -.22^**^ |
| Race | -.12*^t^* | .06 | -.06 | .02 | .11 | .06 |
| SES | .01 | -.01 | -.32^***^ | -.03 | -.13^*^ | -.27^***^ |
| HM | -.14*^t^* | -.14 | -.09 | -.06 | .04 | .12 |
| **Step 4: ∆ R^2^** | **.014** | **.052^*^** | **.005** | **.017** | **.031** | **.000** |
| Political Party | -.38^***^ | -.14 | .03 | -.46^***^ | -.38^***^ | -.08 |
| Gender | -.23^**^ | -.05 | -.06 | -.17^*^ | -.23^**^ | -.22^**^ |
| Race | -.14^*^ | .02 | -.07 | .02 | .11 | .06 |
| SES | .02 | -.01 | -.32^***^ | -.02 | -.12*^t^* | -.27^***^ |
| HM | -.10 | -.08 | -.08 | .01 | .13 | .12 |
| HM^*^Party | .04 | .22^**^ | .06 | .11 | .18^**^ | .00 |
| HM^*^Gender | .01 | .04 | -.05 | -.07 | -.05 | -.02 |
| HM^*^Race | -.13*^t^* | -.16 | -.01 | -.08 | -.09 | -.01 |
| HM^*^SES | .01 | .03 | .02 | .05 | .03 | .01 |

*Note.* SES = socioeconomic status; HM = hegemonic masculinity

*^t^ p* < .075. *^*^p* < .05. *^**^* *p* < .01. *^***^* *p* < .001

HM^*^Party: Undergraduates who identified as Democrats, but not Republicans, reported less psychological impact of COVID-19 the more strongly they endorsed HM [Democrats: *b* = -.52, *t*(178) = -2.73, *p* = .007;

Republicans: *b* = .24, *t*(178) = 1.11, *p* = .268].

**Table S14**

*Results of Hierarchical Regressions for Study 2a Including National Identity*

| **Independent Variables** | **Risk-Taking** | **Personal Threat** | **Psych. Impact** | **Financial Impact** | **Mandates** | **Conspiracy** |
| --- | --- | --- | --- | --- | --- | --- |
|  | **ꞵ** | **ꞵ** | **ꞵ** | **ꞵ** | **ꞵ** | **ꞵ** |
| **Step 1: R^2^** | **.265^***^** | **.243^***^** | **.033^*^** | **.005** | **.300^***^** | **.130^***^** |
| Political Party | .52^***^ | -.49^***^ | -.18^*^ | -.07 | -.55^***^ | .36^***^ |
| **Step 2: ∆ R^2^** | **.072^***^** | **.090^***^** | **.019** | **.123^***^** | **.068^***^** | **.017** |
| Political Party | .44^***^ | -.45^***^ | -.19^*^ | .02 | -.54^***^ | .36^***^ |
| Gender | .22^**^ | -.30^***^ | -.12 | -.10 | -.23^***^ | .05 |
| Race | .17^*^ | -.12*^t^* | .06 | -.06 | -.10 | -.08 |
| SES | .09 | .00 | -.03 | -.33^***^ | .13^*^ | .09 |
| **Step 3: ∆ R^2^** | **.020^*^** | **.015^*^** | **.001** | **.003** | **.014^*^** | **.003** |
| Political Party | .40^***^ | -.41^***^ | -.19^*^ | -.03 | -.50^***^ | .38^***^ |
| Gender | .22^***^ | -.30^***^ | -.12 | -.11 | -.23^***^ | .05 |
| Race | .12 | -.08 | .05 | -.04 | -.06 | -.06 |
| SES | .08 | .01 | -.03 | -.33^***^ | .14^*^ | .10 |
| National ID | .16^*^ | -.14^*^ | .03 | -.06 | -.14^*^ | -.06 |
| **Step 4: ∆ R^2^** | **.058^***^** | **.009** | **.014** | **.005** | **.022^*^** | **.050^**^** |
| Political Party | .31^***^ | -.38^***^ | -.15 | .06 | -.45^***^ | .29^***^ |
| Gender | .08 | -.24^**^ | -.05 | -.07 | -.15^*^ | -.08 |
| Race | .13*^t^* | -.08 | .04 | -.04 | -.06 | -.05 |
| SES | .05 | .02 | -.02 | -.32^***^ | .15^*^ | .07 |
| National ID | .12 | -.12 | .05 | -.05 | -.11 | -.11 |
| HM | .30^***^ | -.12 | -.15 | -.09 | -.19^*^ | .28^**^ |
| **Step 5: ∆ R^2^** | **.009** | **.017** | **.066^*^** | **.006** | **.018** | **.094^***^** |
| Political Party | .29^***^ | -.35^***^ | -.15*^t^* | .04 | -.42^***^ | .25^**^ |
| Gender | .09 | -.24^**^ | -.04 | -.07 | -.15^*^ | -.06 |
| Race | .13*^t^* | -.09 | .02 | -.05 | -.06 | -.05 |
| SES | .05 | .02 | -.02 | -.32^***^ | .15^*^ | .03 |
| National ID | .12*^t^* | -.14*^t^* | .05 | -.05 | -.12 | -.07 |
| HM | .30^***^ | -.07 | -.06 | -.08 | -.16^*^ | .31^***^ |
| HM^*^Party | .06 | .03 | .17^*^ | .07 | -.08 | .17^*^ |
| HM^*^Gender | -.02 | -.01 | .05 | -.05 | -.01 | .12*^t^* |
| HM^*^Race | .05 | -.16^*^ | .20 | -.01 | -.10 | .02 |
| HM^*^SES | -.02 | .02 | .02 | .02 | -.01 | -.11 |
| HM^*^National ID | .03 | .04 | .14 | -.02 | .02 | .16^*^ |

*Note.* SES = socioeconomic status; HM = hegemonic masculinity

*^t^ p* < .075. *^*^p* < .05. *^**^* *p* < .01. *^***^* *p* < .001

HM^*^National: National identification was associated with less belief in conspiracy theories for those low, but not high, in hegemonic masculinity

[low HM: *b* = -.09, *t*(176) = -2.19, *p* = .030; high HM: *b* = .03, *t*(176) = 0.70, *p* = .487].

**Table S15**

*Results of Hierarchical Regressions for Study 2b Including National Identity*

| **Independent Variables** | **Risk-Taking** | **Personal Threat** | **Psych. Impact** | **Financial Impact** | **Mandates** | **Conspiracy** |
| --- | --- | --- | --- | --- | --- | --- |
|  | **ꞵ** | **ꞵ** | **ꞵ** | **ꞵ** | **ꞵ** | **ꞵ** |
| **Step 1: R^2^** | **.284^***^** | **.235^***^** | **.125^***^** | **.001** | **.526^***^** | **.216^***^** |
| Political Party | .53^***^ | -.4^***^ | -.35^***^ | -.03 | -.73^***^ | .47^***^ |
| **Step 2: ∆ R^2^** | **.016** | **.044^*^** | **.093^***^** | **.115^***^** | **.002** | **.002** |
| Political Party | .53^***^ | -.48^***^ | -.36^***^ | -.03 | -.73^***^ | .47^***^ |
| Gender | .12^*^ | -.20^**^ | -.24^***^ | -.19^**^ | .01 | .03 |
| Race | -.02 | .03 | .11 | .04 | .02 | -.01 |
| SES | -.02 | -.03 | -.12*^t^* | -.26^***^ | .04 | -.03 |
| **Step 3: ∆ R^2^** | **.013*^t^*** | **.002** | **.000** | **.000** | **.009*^t^*** | **.040^**^** |
| Political Party | .48^***^ | -.51^***^ | -.37^***^ | -.02 | -.69^***^ | .37^***^ |
| Gender | .12*^t^* | -.20^**^ | -.24^****^ | -.19^**^ | .01 | .02 |
| Race | -.03 | .02 | .11 | .04 | .02 | -.02 |
| SES | -.03 | -.04 | -.12*^t^* | -.26^***^ | .05 | -.05 |
| National ID | .13*^t^* | .05 | .00 | -.01 | -.10*^t^* | .23^**^ |
| **Step 4: ∆ R^2^** | **.018^*^** | **.005** | **.002** | **.012** | **.003** | **.086^***^** |
| Political Party | .43^***^ | -.48^***^ | -.38^***^ | -.06 | -.67^***^ | .27^***^ |
| Gender | .07 | -.18^**^ | -.25^***^ | -.23^**^ | .03 | -.08 |
| Race | .00 | .01 | .11 | .07 | .01 | .05 |
| SES | -.04 | -.04 | -.13*^t^* | -.26^***^ | .06 | -.07 |
| National ID | .06 | .09 | -.02 | -.06 | -.08 | -.08 |
| HM | .18^*^ | -.09 | .05 | .14 | -.07 | .39^***^ |
| **Step 5: ∆ R^2^** | **.011** | **.020** | **.031** | **.003** | **.007** | **.012** |
| Political Party | .42^***^ | -.49^***^ | -.38^***^ | -.06 | -.66^***^ | .26^***^ |
| Gender | .06 | -.16^*^ | -.23^**^ | -.23^**^ | .04 | -.08 |
| Race | -.01 | .01 | .11 | .07 | .00 | .06 |
| SES | -.05 | -.03 | -.12*^t^* | -.27^***^ | .06 | -.06 |
| National ID | .04 | .11 | .00 | -.08 | -.06 | .11 |
| HM | .14 | -.04 | .12 | .14 | -.04 | .41^***^ |
| HM^*^Party | .06 | .13*^t^* | .18^*^ | .01 | .06 | -.02 |
| HM^*^Gender | .04 | -.08 | -.05 | -.01 | .00 | -.03 |
| HM^*^Race | .10 | -.08 | -.09 | .01 | -.05 | -.06 |
| HM^*^SES | -.03 | .06 | .03 | .01 | .04 | -.07 |
| HM^*^National ID | -.05 | -.01 | -.02 | -.06 | .04 | .11 |

*Note.* SES = socioeconomic status; HM = hegemonic masculinity

*^t^ p* < .075. *^*^p* < .05. *^**^* *p* < .01. *^***^* *p* < .001

**A Note on Analyses Including National Identity**

As noted in the main text, the findings were not influenced by the inclusion of national identity (as shown in Tables S14 and S15). Interestingly, however, in both studies national identity emerged as positively related to risk-taking and negatively related to agreement with mandates. In other words, the more one identified as being American and that being American was important to their identity, the more likely they were to take COVID-19 risks and the less likely they were to agree with Biden’s COVID-19 mandates. In addition, among students (but not non-students) stronger national identity was associated with greater belief in COVID-19 conspiracy theories.

**All Analyses Conducted Using Political Ideology (Instead of Political Party)**

As noted in the main text, our analyses included political party (Democrat – Republican) given that partisanship is a strong predictor of both political attitudes/behaviors and responses to COVID-19. However, we conducted all analyses replacing political party with political ideology in Step 1 of the hierarchical regressions. In the pages that follow, we include all tables reflecting these analyses. Major differences between the analyses presented below and those presented in the main text are highlighted prior to the tables. The focus of the present work was not to determine differences based on political party affiliation vs. political ideology; therefore, additional research is needed to better understand how these two measures are (1) differentially related to outcomes, and (2) the differential association of party vs. ideology to the endorsement of hegemonic masculinity.

***Study 1a:*** When including political ideology (instead of political party):

HM is only marginally significant in predicting evaluations of Trump’s COVID response.

***Study 1b:*** When including political ideology (instead of political party):

HM no longer predicts evaluations of Biden’s COVID response.
 HM no longer predicts evaluations of Pelosi’s COVID response.

HM no longer predicts evaluations of Republican Congress’s COVID response.

***Study 2a:*** When including political ideology (instead of political party):

There are no major differences in patterns of results.

***Study 2b:***  When including political ideology (instead of political party):

HM no longer predicts risk-taking.

**Table S16**

*Results of Hierarchical Regressions for Evaluations of Political Leaders’ Responses to COVID-19, Studies 1a and 1b*

|  | **Study 1a (April 2020)** | | | | | **Study 1b (December 2020)** | | | | |
| --- | --- | --- | --- | --- | --- | --- | --- | --- | --- | --- |
| **Independent Variables** | **Trump Response** | **McConnell Response** | **Biden Response** | **Pelosi Response** | **Fauci Response** | **Trump Response** | **McConnell Response** | **Biden Response** | **Pelosi Response** | **Fauci Response** |
|  | **ꞵ** | **ꞵ** | **ꞵ** | **ꞵ** | **ꞵ** | **ꞵ** | **ꞵ** | **ꞵ** | **ꞵ** | **ꞵ** |
| **Step 1: R^2^** | **.437^***^** | **.218^***^** | **.032^*^** | **.194^***^** | **.018** | **.607^***^** | **.287^***^** | **.491^***^** | **.406^***^** | **.095^***^** |
| P. Ideology | .66^***^ | .47^***^ | -.18^*^ | -.44^***^ | -.13 | .78^***^ | .54^***^ | -.70^***^ | -.64^***^ | -.31^***^ |
| **Step 2: ∆ R^2^** | **.021** | **.027** | **.009** | **.024** | **.019** | **.008** | **.028*^t^*** | **.007** | **.019** | **.020** |
| P. Ideology | .67^***^ | .50^***^ | -.20^*^ | -.42^***^ | -.19^*^ | .77^***^ | .58^***^ | -.71^***^ | -.63^***^ | -.36^***^ |
| Gender | -.08 | -.15*^t^* | .02 | .04 | .13 | .01 | -.17^**^ | -.01 | -.11*^t^* | .02 |
| Race | .12^*^ | .04 | .06 | -.15*^t^* | .08 | .05 | .02 | .04 | .08 | .10 |
| SES | .03 | .08 | .07 | .00 | -.01 | -.08^*^ | -.04 | .07 | -.04 | .10 |
| **Step 3: ∆ R^2^** | **.012*^t^*** | **.024^*^** | **.003** | **.004** | **.000** | **.009^*^** | **.028^**^** | **.003** | **.008** | **.021^*^** |
| P. Ideology | .58^***^ | .38^***^ | -.25^*^ | -.38^***^ | -.20 | .71^***^ | .48^***^ | -.68^***^ | -.58^***^ | -.27^***^ |
| Gender | -.13^*^ | -.21^*^ | .00 | .06 | .12 | -.03 | -.23^***^ | .01 | -.07 | .08 |
| Race | .12^*^ | .05 | .06 | -.16^*^ | .08 | .06 | .04 | .03 | .07 | .08 |
| SES | .02 | .07 | .07 | .00 | -.02 | -.09^*^ | -.05 | .08 | -.03 | .11 |
| HM | .16*^t^* | .22^*^ | .08 | -.08 | .02 | .12^*^ | .21^**^ | -.07 | -.12 | -.19^*^ |
| **Step 4: ∆ R^2^** | **.011** | **.052*^t^*** | **.027** | **.008** | **.011** | **.011** | **.028** | **.006** | **.008** | **.024** |
| P. Ideology | .59^**^ | .42^***^ | -.23^*^ | -.38^***^ | -.21*^t^* | .70^***^ | .44^***^ | -.68^***^ | -.58^***^ | -.27^**^ |
| Gender | -.14^*^ | -.24^**^ | -.02 | .06 | .13 | -.03 | -.22^**^ | .00 | -.08 | .07 |
| Race | .12^*^ | .06 | .08 | -.15*^t^* | .07 | .06 | .06 | .03 | .07 | .06 |
| SES | .02 | .07 | .07 | .01 | -.01 | -.09^*^ | -.05 | .08 | -.03 | .12 |
| HM | .22^*^ | .30^*^ | .13 | -.15 | -.02 | .12^*^ | .16*^t^* | -.07 | -.12 | -.14 |
| HM^*^Ideology | .01 | -.18 | -.16 | -.05 | .06 | .06 | .08 | -.03 | -.02 | .06 |
| HM^*^Gender | .07 | .10 | .05 | .05 | -.01 | .08*^t^* | .03 | -.07 | -.07 | -.15^*^ |
| HM^*^Race | -.09 | -.19*^t^* | -.09 | .11 | .06 | .00 | .11 | .02 | .05 | -.03 |
| HM^*^SES | .02 | .05 | .04 | -.04 | .06 | .01 | -.09 | -.01 | .05 | .02 |

*Note.* SES = socioeconomic status; HM = hegemonic masculinity

*^t^ p* < .075. *^*^p* < .05. *^**^* *p* < .01. *^***^* *p* < .001

**Table S17**

*Results of Hierarchical Regressions for Risk-Taking During and Perceived Personal Impact of COVID-19, Studies 1a and 1b*

|  | **Study 1a (April 2020)** | | | | | **Study 1b (December 2020)** | | | | |
| --- | --- | --- | --- | --- | --- | --- | --- | --- | --- | --- |
|  | **Risk-Taking** | | **Personal Impact** | | | **Risk-Taking** | | **Personal Impact** | | |
| **Independent Variables** | **Rule-Based**  **Risks** | **Helping**  **Risks** | **Personal**  **Threat** | **Psych.**  **Impact** | **Financial**  **Impact** | **Rule-Based**  **Risks** | **Helping**  **Risks** | **Personal**  **Threat** | **Psych. Impact** | **Financial**  **Impact** |
|  | **ꞵ** | **ꞵ** | **ꞵ** | **ꞵ** | **ꞵ** | **ꞵ** | **ꞵ** | **ꞵ** | **ꞵ** | **ꞵ** |
| **Step 1: R^2^** | **.131^***^** | **.002** | **.101^***^** | **.048^**^** | **.000** | **.225^***^** | **.025^*^** | **.248^***^** | **.073^***^** | **.020^*^** |
| P. Ideology | .36^***^ | -.04 | -.32^***^ | -.22^**^ | -.01 | .47^***^ | -.16^*^ | -.50^***^ | -.27^***^ | -.14^*^ |
| **Step 2: ∆ R^2^** | **.087^***^** | **.040*^t^*** | **.119^***^** | **.055^*^** | **.133^***^** | **.010** | **.049^**^** | **.014** | **.015** | **.030*^t^*** |
| P. Ideology | .26^***^ | .00 | -.23^**^ | -.18^*^ | .01 | .45^***^ | -.07 | -.46^***^ | -.24^***^ | -.10 |
| Gender | .30^***^ | -.18^*^ | -.35^***^ | -.22^**^ | -.02 | .10 | -.09 | -.12^*^ | -.12*^t^* | -.12 |
| Race | .11 | .09 | .00 | .09 | .21^**^ | .02 | -.16^*^ | -.01 | -.01 | -.01 |
| SES | -.05 | -.05 | .11 | .05 | -.31^***^ | -.03 | -.13^*^ | .01 | .04 | -.13^*^ |
| **Step 3: ∆ R^2^** | **.038^**^** | **.007** | **.000** | **.031^*^** | **.000** | **.070^***^** | **.000** | **.012^*^** | **.010** | **.004** |
| P. Ideology | .11 | -.07 | -.22^*^ | -.04 | .00 | .28^***^ | -.09 | -.39^***^ | -.17^*^ | -.06 |
| Gender | *.22^**^* | -.21^*^ | -.34^***^ | -.15*^t^* | -.03 | .00 | -.09 | -.08 | -.09 | -.09 |
| Race | .12 | .09 | .00 | .08 | .21^**^ | .06 | -.16^*^ | -.03 | -.03 | -.02 |
| SES | -.05 | -.05 | 12 | .06 | -.31^***^ | -.06 | -.14^*^ | .02 | .05 | -.12*^t^* |
| HM | .28^**^ | .12 | -.03 | -.25^*^ | .01 | .34^***^ | .02 | -.14^*^ | -.13 | -.08 |
| **Step 4: ∆ R^2^** | **.013** | **.044** | **.014** | **.041** | **.098^***^** | **.013** | **.017** | **.029^*^** | **.011** | **.040^*^** |
| P. Ideology | .10 | -.07 | -.22^*^ | -.01 | .00 | .24^**^ | -.05 | -.35^***^ | -.16*^t^* | -.04 |
| Gender | .22^**^ | -.21^*^ | -.34^***^ | -.16^*^ | .01 | .02 | -.11 | -.11*^t^* | -.10 | -.11 |
| Race | .13*^t^* | .10 | .02 | .05 | .24^**^ | .08 | -.17^*^ | -.05 | -.05 | -.02 |
| SES | -.04 | -.03 | .13*^t^* | .06 | -.26^***^ | -.06 | -.15^*^ | .02 | .05 | -.13^*^ |
| HM | .23^*^ | .08 | -.03 | -.13 | -.03 | .28^***^ | .07 | -.05 | -.08 | -.03 |
| HM^*^Ideology | -.01 | .11 | -.11 | .13 | -.13 | -.02 | -.04 | .05 | .09 | .02 |
| HM^*^Gender | .03 | .08 | .00 | .00 | -.12 | .04 | .01 | .00 | .03 | .09 |
| HM^*^Race | .08 | .08 | .01 | -.22^*^ | .02 | .11 | -.11 | -.15^*^ | -.10 | -.05 |
| HM^*^SES | .08 | .07 | .08 | .07 | .28^***^ | -.06 | .08 | .12^*^ | -.02 | .19^**^ |

*Note.* SES = socioeconomic status; HM = hegemonic masculinity

*^t^ p* < .075. *^*^p* < .05. *^**^* *p* < .01. *^***^* *p* < .001

**Table S18**

*Results of Hierarchical Regressions for Risk-Taking,COVID-19 Mandates, and Belief in COVID Conspiracies, Studies 2a and 2b*

|  | **Study 2a** | | | **Study 2b** | | |
| --- | --- | --- | --- | --- | --- | --- |
| **Independent Variables** | **Rule-Based**  **Risks** | **Mandates** | **Conspiracies** | **Rule-Based**  **Risks** | **Mandates** | **Conspiracies** |
|  | **ꞵ** | **ꞵ** | **ꞵ** | **ꞵ** | **ꞵ** | **ꞵ** |
| **Step 1: R^2^** | **.279^***^** | **.349^***^** | **.128^***^** | **.302^***^** | **.524^***^** | **.264^***^** |
| P. Ideology | .53^***^ | -.59^***^ | .36^***^ | .55^***^ | -.73^***^ | .51^***^ |
| **Step 2: ∆ R^2^** | **.071^***^** | **.044^**^** | **.019** | **.011** | **.005** | **.002** |
| P. Ideology | .45^***^ | -.55^***^ | .36^***^ | .55^***^ | -.73^***^ | .52^***^ |
| Gender | .18^**^ | -.18^**^ | .02 | .10 | .04 | .01 |
| Race | .18^**^ | -.12^.058^ | -.07 | -.01 | -.01 | .00 |
| SES | .12^*^ | .09 | .13*^t^* | -.03 | .06 | -.04 |
| **Step 3: ∆ R^2^** | **.056^***^** | **.017^*^** | **.039^**^** | **.008** | **.001** | **.075^***^** |
| P. Ideology | .34^***^ | -.49^***^ | .26^***^ | .48^***^ | -.75^***^ | .31^***^ |
| Gender | .06 | -.12 | -.09 | .07 | .03 | -.08 |
| Race | .18^**^ | -.11*^t^* | -.07 | .01 | .00 | .06 |
| SES | .09 | .11*^t^* | .10 | -.04 | .05 | -.07 |
| HM | .30^***^ | -.17^*^ | .25^**^ | .12 | .03 | .36^***^ |
| **Step 4: ∆ R^2^** | **.003** | **.004** | **.049^*^** | **.009** | **.012** | **.003** |
| P. Ideology | .33^***^ | -.47^***^ | .25^**^ | .47^***^ | -.74^***^ | .31^***^ |
| Gender | .05 | -.12 | -.08 | .06 | .04 | -.08 |
| Race | .19^**^ | -.12*^t^* | -.07 | -.01 | .00 | .06 |
| SES | .09 | .11 | .06 | -.05 | .07 | -.06 |
| HM | .29^***^ | -.15^*^ | .23^**^ | .06 | .08 | .39^***^ |
| HM^*^Ideology | -.03 | .00 | .10 | -.02 | .11^*^ | .04 |
| HM^*^Gender | -.02 | .00 | .14^*^ | .05 | -.01 | -.03 |
| HM^*^Race | .05 | -.07 | .10 | .11 | -.07 | -.03 |
| HM^*^SES | -.02 | .00 | -.11 | -.02 | .01 | -.03 |

*Note.* SES = socioeconomic status; HM = hegemonic masculinity

*^t^ p* < .075. *^*^p* < .05. *^**^* *p* < .01. *^***^* *p* < .001.

HM^*^Gender: HM predicts belief in conspiracy theories for men, but not women [men: *b* = .28, *t*(178) = 3.45, *p* < .001; women: *b* = .05, *t*(178) = 0.32, *p* = .534].

**Table S19**

*Results of Hierarchical Regressions for Evaluations of Republican and Democratic Leaders’ Response to COVID-19, Studies 1a and 1b*

|  | **Study 1a** | | | **Study 1b** | | |
| --- | --- | --- | --- | --- | --- | --- |
| **Independent Variables** | **Republican**  **Congress** | **Democratic Congress** | **State Leaders** | **Republican Congress** | **Democratic Congress** | **State Leaders** |
|  | **ꞵ** | **ꞵ** | **ꞵ** | **ꞵ** | **ꞵ** | **ꞵ** |
| **Step 1: R^2^** | **.388^***^** | **.177^***^** | **.022*^t^*** | **.525^***^** | **.367^***^** | **.090^***^** |
| P. Ideology | .62^***^ | -.42^***^ | -.15*^t^* | .72^***^ | -.61^***^ | -.30^***^ |
| **Step 2: ∆ R^2^** | **.088^***^** | **.003** | **.005** | **.012** | **.007** | **.001** |
| P. Ideology | .68^***^ | -.44^***^ | -.15 | .69^***^ | -.62^***^ | -.29^***^ |
| Gender | -.26^***^ | .02 | .00 | .00 | -.04 | -.02 |
| Race | .14^*^ | .06 | .05 | .11^*^ | .07 | -.03 |
| SES | -.01 | .01 | -.06 | -.03 | .02 | .00 |
| **Step 3: ∆ R^2^** | **.006** | **.000** | **.000** | **.002** | **.001** | **.000** |
| P. Ideology | .62^***^ | -.43^***^ | -.13 | .66^***^ | -.60^***^ | -.28^***^ |
| Gender | -.29^***^ | .03 | .01 | -.02 | -.03 | -.01 |
| Race | .15^*^ | .06 | .04 | .12^*^ | .07 | -.03 |
| SES | -.01 | .01 | -.05 | -.03 | .02 | .00 |
| HM | .11 | -.02 | -.03 | .06 | -.04 | -.01 |
| **Step 4: ∆ R^2^** | **.024** | **.037** | **.034** | **.012** | **.037^*^** | **.041^*^** |
| P. Ideology | .63^***^ | -.40^***^ | -.10 | .64^***^ | -.60^***^ | -.29^**^ |
| Gender | -.30^***^ | .00 | -.01 | -.01 | -.04 | -.05 |
| Race | .15^*^ | .07 | .04 | .12^*^ | .06 | -.06 |
| SES | -.02 | .00 | -.06 | -.02 | .02 | .02 |
| HM | .20^*^ | .09 | .10 | .04 | -.02 | .05 |
| HM^*^Ideology | .05 | -.08 | -.02 | .07 | -.02 | .10 |
| HM^*^Gender | -.04 | .13 | -.01 | .03 | -.19^***^ | -.19^**^ |
| HM^*^Race | -.17^*^ | -.19*^t^* | -.24^*^ | .03 | .04 | .05 |
| HM^*^SES | .11 | .00 | .04 | -.07 | .02 | .02 |

*Note.* SES = socioeconomic status; HM = hegemonic masculinity

*^t^ p* < .075. *^*^p* < .05. *^**^* *p* < .01. *^***^* *p* < .001

HM^*^Gender on DemCongress: HM predicted evaluations of Democratic Congress for men, but not women [men: *b* = -.42,

*t*(203) = -2.49, *p* = .013; women: *b* = .36, *t*(203) = 1.86, *p* = .064].

HM^*^Gender on State: HM predicted evaluations of State Leaders for women, but not men [women *b* = .47, *t*(213) = 2.00,

*p* = .047; men: *b* = -.30, *t*(213) = -1.50, *p* = .135].

**Table S20**

*Results of Hierarchical Regressions for Evaluations of Political Leaders’ Responses to COVID-19, Study 1b Including Precarious Masculinity*

| **Independent Variables** | **Trump Response** | **McConnell Response** | **Biden Response** | **Pelosi Response** | **Fauci Response** | **Republican Congress** | **Democratic Congress** | **State Leaders** |
| --- | --- | --- | --- | --- | --- | --- | --- | --- |
|  | **ꞵ** | **ꞵ** | **ꞵ** | **ꞵ** | **ꞵ** | **ꞵ** | **ꞵ** | **ꞵ** |
| **Step 1: R^2^** | **.609^***^** | **.286^***^** | **.491^***^** | **.407^***^** | **.096^***^** | **.526^***^** | **.367^***^** | **.091^***^** |
| P. Ideology | .78^***^ | .54^***^ | -.70^***^ | -.64^***^ | -.31^***^ | .73^***^ | -.61^***^ | -.30^***^ |
| **Step 2: ∆ R^2^** | **.008** | **.028*^t^*** | **.007** | **.019** | **.020** | **.012** | **.007** | **.001** |
| P. Ideology | .77^***^ | .58^***^ | -.71^***^ | -.63^***^ | -.36^***^ | .69^***^ | -.62^***^ | -.29^***^ |
| Gender | .00 | -.17^**^ | -.01 | -.10*^t^* | .03 | .00 | -.04 | -.01 |
| Race | .05 | .02 | .04 | .08 | .10 | .12^*^ | .07 | -.04 |
| SES | -.08*^t^* | -.04 | .08 | -.04 | .10 | -.03 | .02 | .00 |
| **Step 3: ∆ R^2^** | **.011^*^** | **.040^**^** | **.003** | **.012** | **.026^*^** | **.003** | **.001** | **.000** |
| P. Ideology | .71^***^ | .49^***^ | -.68^***^ | -.58^***^ | -.27^***^ | .66^***^ | -.601^***^ | -.28^***^ |
| Gender | -.01 | -.17^*^ | .00 | -.10 | .12 | .00 | -.02 | .00 |
| Race | .07 | .05 | .03 | .06 | .09 | .12^*^ | .07 | -.04 |
| SES | -.09^*^ | -.05 | .08 | -.04 | .12 | -.03 | .02 | .00 |
| PM | .05 | .13*^t^* | -.01 | -.07 | .07 | .03 | .01 | .01 |
| HM | .11*^t^* | .16^*^ | -.07 | -.09 | -.22^*^ | .05 | -.04 | -.01 |
| **Step 4: ∆ R^2^** | **.022** | **.049** | **.022** | **.021** | **.057** | **.032** | **.041** | **.048** |
| P. Ideology | .69^***^ | .43^***^ | -.66^***^ | -.59^***^ | -.26^**^ | .62^***^ | -.61^***^ | -.31^***^ |
| Gender | .01 | -.14*^t^* | -.02 | -.09 | .09 | .02 | -.02 | -.04 |
| Race | .07 | .07 | .02 | .06 | .09 | .13^*^ | .05 | -.05 |
| SES | -.09^*^ | -.04 | .07 | -.03 | .13^*^ | -.01 | .02 | .01 |
| PMI | .12^*^ | .20^*^ | -.08 | -.01 | .05 | .12 | .04 | -.01 |
| HM | .10 | .09 | -.05 | -.12 | -.17 | .02 | -.02 | .04 |
| HM^*^Ideology | .05 | .04 | .01 | -.02 | .01 | .03 | -.02 | .08 |
| HM^*^Gender | .05 | .02 | -.10 | -.08 | -.06 | .06 | -.22^**^ | -.17*^t^* |
| HM^*^Race | .00 | .15*^t^* | .00 | .07 | -.02 | .06 | .04 | .06 |
| HM^*^SES | .03 | -.06 | -.01 | .05 | .01 | -.05 | .02 | .03 |
| PMI^*^Ideology | .15^*^ | .15 | -.17^*^ | .03 | .00 | .17^*^ | .05 | .06 |
| PMI^*^Gender | .06 | .09 | -.01 | .09 | -.21^**^ | .02 | .06 | -.04 |
| PMI^*^Race | -.07 | -.09 | .07 | -.04 | -.06 | -.12*^t^* | .01 | .01 |
| PMI^*^SES | .02 | .01 | -.05 | .05 | -.05 | .01 | .00 | -.07 |
| HM^*^PMI | -.14^*^ | -.04 | .07 | .00 | .08 | .03 | -.06 | .00 |

*Note.* SES = socioeconomic status; PM = precarious masculinity; HM = hegemonic masculinity

*^t^ p* < .075. *^*^p* < .05. *^**^* *p* < .01. *^***^* *p* < .001

**Table S21**

*Results of Hierarchical Regressions for Risk-Taking During and Perceived Personal Impact of COVID-19, Study 1b Including Precarious Masculinity*

|  | **Risk-Taking** | | **Personal Impact** | | |
| --- | --- | --- | --- | --- | --- |
| **Independent Variables** | **Rule-Based**  **Risks** | **Helping**  **Risks** | **Personal**  **Threat** | **Psych. Impact** | **Financial**  **Impact** |
|  | **ꞵ** | **ꞵ** | **ꞵ** | **ꞵ** | **ꞵ** |
| **Step 1: R^2^** | **.225^***^** | **.025^*^** | **.249^***^** | **.073^***^** | **.020^*^** |
| P. Ideology | .48^***^ | -.16^*^ | -.50^***^ | -.27^***^ | -.14^*^ |
| **Step 2: ∆ R^2^** | **.010** | **.050^**^** | **.013** | **.015** | **.029*^t^*** |
| P. Ideology | .44^***^ | -.08 | -.46^***^ | -.24^***^ | -.10 |
| Gender | .10 | -.08 | -.12^*^ | -.12*^t^* | -.11 |
| Race | .02 | -.17^*^ | -.01 | -.01 | -.01 |
| SES | -.03 | -.14^*^ | .01 | .04 | -.13^*^ |
| **Step 3: ∆ R^2^** | **.070^***^** | **.004** | **.019^*^** | **.010** | **.007** |
| P. Ideology | .27^***^ | -.10 | -.38^***^ | -.17^*^ | -.07 |
| Gender | -.02 | -.12 | -.03 | -.07 | -.12 |
| Race | .06 | -.17^*^ | -.03 | -.03 | -.03 |
| SES | -.06 | -.14^*^ | .03 | .05 | -.13*^t^* |
| PM | -.03 | -.07 | .09 | .03 | -.07 |
| HM | .35^***^ | .05 | -.18^*^ | -.14 | -.05 |
| **Step 4: ∆ R^2^** | **.024** | **.028** | **.051*^t^*** | **.011** | **.048** |
| P. Ideology | .24^**^ | -.08 | -.33^***^ | -.15 | -.04 |
| Gender | .03 | -.14 | -.10 | -.10 | -.14 |
| Race | .07 | -.17^*^ | -.03 | -.05 | -.03 |
| SES | -.07 | -.16^*^ | .05 | .06 | -.14^*^ |
| PMI | .01 | -.12 | 07 | .03 | -.08 |
| HM | .28^**^ | .10 | -.09 | -.09 | -.02 |
| HM^*^Ideology | .01 | -.05 | .01 | .09 | .05 |
| HM^*^Gender | -.03 | .04 | .10 | .04 | .04 |
| HM^*^Race | .11 | -.11 | -.14*^t^* | -.10 | -.05 |
| HM^*^SES | -.04 | .07 | .11*^t^* | -.02 | .20^**^ |
| PMI^*^Ideology | -.01 | .04 | -.02 | -.01 | -.09 |
| PMI^*^Gender | .11 | -.06 | -.13*^t^* | -.02 | .09 |
| PMI^*^Race | -.05 | .06 | .02 | .00 | .04 |
| PMI^*^SES | -.02 | -.06 | .06 | .03 | -.05 |
| HM^*^PMI | -.07 | .01 | .16 | .01 | .04 |

*Note.* SES = socioeconomic status; PM = precarious masculinity; HM = hegemonic masculinity

*^t^ p* < .075. *^*^p* < .05. *^**^* *p* < .01. *^***^* *p* < .001

**Table S22**

*Results of Hierarchical Regressions for Study 1b, Males Only*

| **Independent Variables** | **Trump** | **McConnell** | **Biden** | **Pelosi** | **Fauci** | **Rule-Based Risks** | **Personal Threat** | **Psych. Impact** | **Financial Impact** |
| --- | --- | --- | --- | --- | --- | --- | --- | --- | --- |
|  | **ꞵ** | **ꞵ** | **ꞵ** | **ꞵ** | **ꞵ** | **ꞵ** | **ꞵ** | **ꞵ** | **ꞵ** |
| **Step 1: R^2^** | **.556^***^** | **.348^***^** | **.445^***^** | **.226^***^** | **.125^***^** | **.297^***^** | **.342^***^** | **.091^***^** | **.017** |
| P. Ideology | .75^***^ | .59^***^ | -.67^***^ | -.48^***^ | -.35^***^ | .55^***^ | -.59^***^ | -.30^***^ | -.13 |
| **Step 2: ∆ R^2^** | **.011** | **.014** | **.005** | **.015** | **.009** | **.005** | **.007** | **.004** | **.016** |
| P. Ideology | .72^***^ | .55^***^ | -.67^***^ | -.47^***^ | -.39^***^ | .52^***^ | -.55^***^ | -.28^**^ | -.11 |
| Race | .09 | .13 | -.02 | -.01 | .09 | .06 | -.09 | -.06 | -.03 |
| SES | -.06 | -.01 | .07 | -.12 | .06 | -.03 | -.02 | .03 | -.13 |
| **Step 3: ∆ R^2^** | **.044^**^** | **.076^**^** | **.021** | **.020** | **.076^*^** | **.065^**^** | **.007** | **.002** | **.000** |
| P. Ideology | .57^***^ | .42^***^ | -.56^***^ | -.37^***^ | -.25^*^ | .35^***^ | -.49^***^ | -.25^*^ | -.12 |
| Race | .15^*^ | .18^*^ | -.06 | -.05 | .02 | .14 | -.12 | -.07 | -.03 |
| SES | -.08 | -.02 | .08 | -.11 | .07 | -.04 | -.01 | .03 | -.13 |
| PMI | .05 | .20^*^ | -.02 | -.05 | -.12 | .06 | .00 | .01 | -.02 |
| HM | .23^**^ | .17 | -.17*^t^* | -.14 | -.24^*^ | .27^**^ | -.10 | -.06 | .01 |
| **Step 4: ∆ R^2^** | **.027** | **.025** | **.021** | **.028** | **.026** | **.023** | **.050** | **.060** | **.071** |
| P. Ideology | .57^***^ | .39^***^ | -.61^***^ | -.37^**^ | -.25*^t^* | .33^**^ | -.46^***^ | -.25*^t^* | -.15 |
| Race | .12 | .12 | -.07 | -.02 | -.02 | .10 | -.08 | -.12 | .05 |
| SES | -.06 | .06 | .11 | -.26^*^ | -.05 | -.12 | .04 | .23 | -.18 |
| PMI | .13 | .20 | -.04 | -.06 | .00 | .11 | -.08 | -.06 | .01 |
| HM | .24^*^ | .17 | -.20 | -.21 | -.35^*^ | .17 | .06 | .07 | .11 |
| HM^*^Ideology | .08 | .05 | .06 | -.06 | .02 | -.03 | .09 | .12 | .18 |
| HM^*^Race | -.02 | .10 | .03 | .00 | .01 | .16 | -.15 | .01 | -.23 |
| HM^*^SES | .01 | -.11 | -.02 | .13 | .11 | .06 | .07 | -.18 | .18 |
| PMI^*^Ideology | .17^*^ | .13 | -.18*^t^* | -.14 | -.02 | .10 | -.08 | .12 | -.10 |
| PMI^*^Race | -.03 | -.07 | .04 | .03 | -.12 | -.02 | .02 | -.08 | .16 |
| PMI^*^SES | .02 | .04 | .05 | -.17 | -.18 | -.12 | .06 | .13 | .04 |
| HM^*^PMI | .08 | .01 | .07 | .03 | -.09 | -.07 | .18 | .14 | -.08 |

*Note.* SES = socioeconomic status; PMI = precarious masculine identity; HM = hegemonic masculinity

*^t^ p* < .075. *^*^p* < .05. *^**^* *p* < .01. *^***^* *p* < .001

**Table S23**

*Results of Hierarchical Regressions for Perceived Personal Impact of COVID-19, Studies 2a and 2b*

|  | **Study 2a** | | | **Study 2b** | | |
| --- | --- | --- | --- | --- | --- | --- |
| **Independent Variables** | **Personal**  **Threat** | **Psych. Impact** | **Financial Impact** | **Personal**  **Threat** | **Psych.**  **Impact** | **Financial**  **Impact** |
|  | **ꞵ** | **ꞵ** | **ꞵ** | **ꞵ** | **ꞵ** | **ꞵ** |
| **Step 1: R^2^** | **.254^***^** | **.030^*^** | **.006** | **.246^***^** | **.131^***^** | **.001** |
| P. Ideology | -.50^***^ | -.17^*^ | -.08 | -.50^***^ | -.36^***^ | -.04 |
| **Step 2: ∆ R^2^** | **.077^***^** | **.016** | **.121^***^** | **.035^*^** | **.082^***^** | **.114^***^** |
| Political Party | -.44^***^ | -.17^*^ | -.02 | -.49^***^ | -.36^***^ | -.02 |
| Gender | -.26^***^ | -.11 | -.10 | -.18^**^ | -.22^***^ | -.19^**^ |
| Race | -.14^*^ | .04 | -.05 | .01 | .09 | .04 |
| SES | -.04 | -.04 | -.33^***^ | -.02 | -.12*^t^* | -.26^***^ |
| **Step 3: ∆ R^2^** | **.009** | **.013** | **.004** | **.001** | **.009** | **.011** |
| P. Ideology | -.39^***^ | -.11 | .01 | -.51^***^ | -.42^***^ | -.10 |
| Gender | -.21^**^ | -.05 | -.07 | -.19^**^ | -.26^***^ | -.23^**^ |
| Race | -.14^*^ | .05 | -.05 | .02 | .11 | .06 |
| SES | -.03 | -.03 | -.33^***^ | -.03 | -.13*^t^* | -.27^***^ |
| HM | -.12 | -.14 | -.08 | .04 | .12 | .14 |
| **Step 4: ∆ R^2^** | **.023** | **.046*^t^*** | **.006** | **.019** | **.023** | **.001** |
| P. Ideology | -.37^***^ | -.09 | .00 | -.50^***^ | -.42^***^ | -.10 |
| Gender | -.21^**^ | -.04 | -.07 | -.18^*^ | -.24^**^ | -.23^**^ |
| Race | -.18^**^ | .00 | -.06 | .03 | .12*^t^* | .07 |
| SES | -.03 | -.04 | -.32^***^ | -.01 | -.11 | -.27^***^ |
| HM | -.08 | -.10 | -.07 | .10 | .19^*^ | .14 |
| HM^*^Ideology | .13^*^ | .19^*^ | .07 | .12*^t^* | .14^*^ | -.03 |
| HM^*^Gender | .00 | .05 | -.05 | -.07 | -.04 | -.02 |
| HM^*^Race | -.14*^t^* | -.14 | -.01 | -.10 | -.11 | .00 |
| HM^*^SES | .02 | .03 | .02 | .02 | .00 | -.01 |

*Note.* SES = socioeconomic status; HM = hegemonic masculinity

*^t^ p* < .075. *^*^p* < .05. *^**^* *p* < .01. *^***^* *p* < .001

**Table S24**

*Results of Hierarchical Regressions for Study 2a Including National Identity*

| **Independent Variables** | **Rule Based Risks** | **Personal Threat** | **Psych. Impact** | **Financial Impact** | **Mandates** | **Conspiracy** |
| --- | --- | --- | --- | --- | --- | --- |
|  | **ꞵ** | **ꞵ** | **ꞵ** | **ꞵ** | **ꞵ** | **ꞵ** |
| **Step 1: R^2^** | **.279^***^** | **.254^***^** | **.030^*^** | **.006** | **.349^***^** | **.128^***^** |
| P. Ideology | .53^***^ | -.50^***^ | -.17^*^ | -.08 | -.59^***^ | .36^***^ |
| **Step 2: ∆ R^2^** | **.071^***^** | **.077^***^** | **.016** | **.121^***^** | **.044^**^** | **.019** |
| P. Ideology | .45^***^ | -.44^***^ | -.17^*^ | -.02 | -.55^***^ | .36^***^ |
| Gender | .18^**^ | -.26^***^ | -.11 | -.10 | -.18^**^ | .02 |
| Race | .18^**^ | -.14^*^ | .04 | -.05 | -.12*^t^* | -.07 |
| SES | .12^*^ | -.04 | -.04 | -.33^***^ | .09 | .13*^t^* |
| **Step 3: ∆ R^2^** | **.021^*^** | **.017^*^** | **.000** | **.002** | **.015^*^** | **.002** |
| P. Ideology | .42^***^ | -.40^***^ | -.17^*^ | -.01 | -.52^***^ | .37^***^ |
| Gender | .18^**^ | -.27^***^ | -.11 | -.10 | -.19^**^ | .01 |
| Race | .13*^t^* | -.09 | .04 | -.03 | -.07 | -.05 |
| SES | .11*^t^* | -.03 | -.05 | -.32^***^ | .10 | .13*^t^* |
| National ID | .17^*^ | -.15^*^ | .02 | -.05 | -.14^*^ | -.06 |
| **Step 4: ∆ R^2^** | **.046^***^** | **.006** | **.014** | **.003** | **.012^*^** | **.044^**^** |
| P. Ideology | .32^***^ | -.37^***^ | -.12 | .02 | -.47^***^ | .28^***^ |
| Gender | .07 | -.23^**^ | -.04 | -.07 | -.13^*^ | -.10 |
| Race | .13^*^ | -.09 | .03 | -.04 | -.07 | -.04 |
| SES | .08 | -.02 | -.03 | -.32^***^ | .11*^t^* | .10 |
| National ID | .12*^t^* | -.13*^t^* | .04 | -.04 | -.12^.^*^t^* | -.10 |
| HM | .28^***^ | -.10 | -.15 | -.07 | -.14^*^ | .27^**^ |
| **Step 5: ∆ R^2^** | **.007** | **.025** | **.061^*^** | **.006** | **.006** | **.077^**^** |
| Political Party | .31^***^ | -.35^***^ | -.10 | .00 | -.45^***^ | .25^**^ |
| Gender | .07 | -.22^**^ | -.03 | -.07 | -.13^*^ | -.08 |
| Race | .15^*^ | -.13*^t^* | .00 | -.05 | -.08 | -.03 |
| SES | .07 | -.02 | -.05 | -.32^***^ | .12*^t^* | .05 |
| National ID | .13*^t^* | -.14^*^ | .05 | -.04 | -.13*^t^* | -.06 |
| HM | .28^***^ | -.05 | -.07 | -.06 | -.13 | .29^**^ |
| HM^*^Party | -.04 | .13*^t^* | .15*^t^* | .07 | .00 | .04 |
| HM^*^Gender | -.01 | -.01 | .06 | -.05 | -.02 | .14^*^ |
| HM^*^Race | .04 | -.15^*^ | -.19^*^ | -.01 | -.07 | .03 |
| HM^*^SES | -.03 | .03 | .02 | .02 | .01 | -.11 |
| HM^*^National ID | .07 | -.01 | .14 | -.01 | -.02 | .21^**^ |

*Note.* SES = socioeconomic status; HM = hegemonic masculinity

*^t^ p* < .075. *^*^p* < .05. *^**^* *p* < .01. *^***^* *p* < .001

HM^*^Race: HM predicts psychological impact for White, but not non-White participants [White: *b* = -.43, *t*(176) = -2.32, *p* = .021; non-White: *b* = .19, *t*(176) = 0.79, *p* = .429].

HM^*^Gender: HM predicts conspiracy beliefs for men, but not women [men: *b*= .33, *t*(176) = 4.00, *p* < .001; women: *b* = .10, *t*(176) = 1.17, *p* = .242].

HM^*^Ideology: HM is a stronger predictor for those with more conservative (vs. more liberal) beliefs [conservative: *b* = .24, *t*(176) = 2.76, *p* = .006; liberal: *b* = .19, *t*(176) = 2.25, *p* = .026].

**Table S25**

*Results of Hierarchical Regressions for Study 2b Including National Identity*

| **Independent Variables** | **Risk-Taking** | **Personal Threat** | **Psych. Impact** | **Financial Impact** | **Mandates** | **Conspiracy** |
| --- | --- | --- | --- | --- | --- | --- |
|  | **ꞵ** | **ꞵ** | **ꞵ** | **ꞵ** | **ꞵ** | **ꞵ** |
| **Step 1: R^2^** | **.302^***^** | **.246^***^** | **.131^***^** | **.001** | **.527^***^** | **.264^***^** |
| P. Ideology | .55^***^ | -.50^***^ | -.36^***^ | -.04 | -.73^***^ | .52^***^ |
| **Step 2: ∆ R^2^** | **.011** | **.035^*^** | **.082^***^** | **.114^***^** | **.005** | **.002** |
| P. Ideology | .55^***^ | -.49^***^ | -.36^***^ | -.02 | -.73^***^ | .52^***^ |
| Gender | .10 | -.18^**^ | -.22^***^ | -.19^**^ | .04 | .01 |
| Race | -.01 | .01 | .09 | .04 | -.01 | .00 |
| SES | -.03 | -.02 | -.12*^t^* | -.26^***^ | .06 | -.04 |
| **Step 3: ∆ R^2^** | **.002** | **.013*^t^*** | **.002** | **.000** | **.000** | **.015^*^** |
| P. Ideology | .52^***^ | -.56^***^ | -.39^***^ | -.02 | -.73^***^ | .44^***^ |
| Gender | .10 | -.19^**^ | -.22^***^ | -.19^**^ | .04 | .01 |
| Race | -.01 | .00 | .09 | .04 | -.01 | -.01 |
| SES | -.04 | -.04 | -.12*^t^* | -.26^***^ | .06 | -.05 |
| National ID | .05 | .14*^t^* | .06 | -.01 | .00 | .15^*^ |
| **Step 4: ∆ R^2^** | **.007** | **.000** | **.007** | **.013** | **.001** | **.062^***^** |
| P. Ideology | .47^***^ | -.55^***^ | -.43^***^ | -.08 | -.74^***^ | .30^***^ |
| Gender | .07 | -.18^**^ | -.25^***^ | -.23^**^ | .03 | -.08 |
| Race | .01 | .00 | .11 | .07 | .00 | .05 |
| SES | -.04 | -.04 | -.13*^t^* | -.27^***^ | .05 | -.07 |
| National ID | .02 | .14*^t^* | .02 | -.05 | -.01 | .05 |
| HM | .11 | -.01 | .12 | .16 | .04 | .35^***^ |
| **Step 5: ∆ R^2^** | **.010** | **.025** | **.024** | **.003** | **.014** | **.009** |
| Political Party | .47^***^ | -.56^***^ | -.44^***^ | -.08 | -.74^***^ | .28^**^ |
| Gender | .06 | -.16^*^ | -.23^**^ | -.23^**^ | .04 | -.08 |
| Race | -.01 | .01 | .12 | .07 | .00 | .06 |
| SES | -.05 | -.02 | -.11 | -.27^***^ | .07 | -.06 |
| National ID | -.01 | .17^*^ | .05 | -.07 | .02 | .09 |
| HM | .06 | .05 | .18*^t^* | .16 | .08 | .37^***^ |
| HM^*^Party | .00 | .15^*^ | .15^*^ | -.02 | .09 | .02 |
| HM^*^Gender | .05 | -.09 | -.05 | -.01 | -.01 | -.04 |
| HM^*^Race | .12 | -.11 | -.11 | .01 | -.08 | -.05 |
| HM^*^SES | -.01 | .03 | .01 | .00 | .00 | -.04 |
| HM^*^National ID | -.05 | .00 | .00 | -.04 | .05 | .08 |

*Note.* SES = socioeconomic status; HM = hegemonic masculinity

*^t^ p* < .075. *^*^p* < .05. *^**^* *p* < .01. *^***^* *p* < .001

**References**

Eisler, R. M., & Skidmore, J. R. (1987). Masculine gender role stress: Scale development and component factors in the appraisal of stressful situations. *Behavior Modification*, *11*(2), 123-136. https://doi.org/10.1177/01454455870112001

Goh, J. X., Hall, J. A., & Rosenthal, R. (2016). Mini meta‐analysis of your own studies: Some arguments on why and a primer on how. *Social and Personality Psychology Compass*, *10*(10), 535-549. https://doi.org/10.1111/spc3.12267
