# Supplementary material for: Men’s and women’s endorsement of hegemonic masculinity and responses to COVID-19
Source: J Health Psychol. 2022 Mar 11;28(3):251–66. doi: 10.1177/13591053221081905 (PMC9982413; doi:10.1177/13591053221081905)
Supplement: sj-pdf-1-hpq-10.1177_13591053221081905 – for Men’s and women’s endorsement of hegemonic masculinity and responses to COVID-19 [file sj-pdf-1-hpq-10.1177_13591053221081905.pdf]

```
 * Encoding: UTF-8.

**COVID-19 Study 1a**

FREQUENCIES VARIABLES=ExclusionReason
  /ORDER=ANALYSIS.
```

Frequencies

Notes

Output Created		15-DEC-2021 13:06:13
Comments		
Input	Data	C:\Users\njs5478\Dropbox\HM and COVID\0. Revise and Resubmit\2. R and R Data\Study 1a\Study1a_Data.sav
	Active Dataset	DataSet1
	Filter	<none>
	Weight	<none>
	Split File	<none>
	N of Rows in Working Data File	185
Missing Value Handling	Definition of Missing	User-defined missing values are treated as missing.
	Cases Used	Statistics are based on all cases with valid data.
Syntax		FREQUENCIES VARIABLES=ExclusionReason /ORDER=ANALYSIS.
Resources	Processor Time	00:00:00.02
	Elapsed Time	00:00:00.02

```
[DataSet1] C:\Users\njs5478\Dropbox\HM and COVID\0. Revise and Resubmit\2. R and R Data\Study 1a\Study1a_Data.sav
```

Statistics

ExclusionReason

N	Valid	7
	Missing	178

ExclusionReason

		Frequency	Percent	Valid Percent	Cumulative Percent
Valid	Failed Attention Check	7	3.8	100.0	100.0
Missing	System	178	96.2		
Total		185	100.0		

```
 USE ALL.
COMPUTE filter_$=(Inclusion = 1).
VARIABLE LABELS filter_$ 'Inclusion = 1 (FILTER)'.
VALUE LABELS filter_$ 0 'Not Selected' 1 'Selected'.
FORMATS filter_$ (f1.0).
FILTER BY filter_$.
EXECUTE.

***Variable Creation***

**Male Role Norms**

RECODE Tough_8 Fem_6 (1=7) (2=6) (3=5) (4=4) (5=3) (6=2) (7=1) INTO Tough_8_Recode Fem_6_
Recode.
EXECUTE.

COMPUTE MRN=(Power_1 + Power_2 + Power_3 + Power_4 + Power_5 + Power_6 + Power_7 + Power_
8 +
    Power_9  + Power_10 + Power_11 + Tough_1 + Tough_2 + Tough_3 + Tough_4 + Tough_5 + To
ugh_6 +
    Tough_7 + Tough_8_Recode + Fem_1 + Fem_2 + Fem_3 + Fem_4 + Fem_5 + Fem_6_Recode + Fem
_7)/26.
EXECUTE.

COMPUTE Power=(Power_1 + Power_2 + Power_3 + Power_4 + Power_5 + Power_6 + Power_7 + Powe
r_8 +
    Power_9  + Power_10 + Power_11)/11.
EXECUTE.

COMPUTE Tough=(Tough_1 + Tough_2 + Tough_3 + Tough_4 + Tough_5 + Tough_6 +
```

```
    Tough_7 + Tough_8_Recode)/8.
EXECUTE.

COMPUTE Fem=(Fem_1 + Fem_2 + Fem_3 + Fem_4 + Fem_5 + Fem_6_Recode + Fem_7)/7.
EXECUTE.

RELIABILITY
  /VARIABLES=Power_1 Power_2 Power_3 Power_4 Power_5 Power_6 Power_7 Power_8 Power_9 Powe
r_10
    Power_11 Tough_1 Tough_2 Tough_3 Tough_4 Tough_5 Tough_6 Tough_7 Tough_8_Recode Fem_1
 Fem_2 Fem_3
    Fem_4 Fem_5 Fem_6_Recode Fem_7
  /SCALE('ALL VARIABLES') ALL
  /MODEL=ALPHA.
```

Reliability

Notes

Output Created		15-DEC-2021 13:06:13
Comments		
Input	Data	C:\Users\njs5478\Dropbox\HM and COVID\0. Revise and Resubmit\2. R and R Data\Study 1a\Study1a_Data.sav
	Active Dataset	DataSet1
	Filter	Inclusion = 1 (FILTER)
	Weight	<none>
	Split File	<none>
	N of Rows in Working Data File	178
	Matrix Input	
Missing Value Handling	Definition of Missing	User-defined missing values are treated as missing.
	Cases Used	Statistics are based on all cases with valid data for all variables in the procedure.

Notes

Syntax		RELIABILITY<br>/VARIABLES=Power_1 Power_2 Power_3 Power_4 Power_5 Power_6 Power_7 Power_8 Power_9 Power_10<br>Power_11 Tough_1 Tough_2 Tough_3 Tough_4 Tough_5 Tough_6 Tough_7 Tough_8_Recode Fem_1 Fem_2 Fem_3<br>Fem_4 Fem_5 Fem_6_Recode Fem_7<br>/SCALE('ALL VARIABLES') ALL<br>/MODEL=ALPHA.
Resources	Processor Time	00:00:00.00
	Elapsed Time	00:00:00.00

Scale: ALL VARIABLES

Case Processing Summary

		N	%
Cases	Valid	178	100.0
	Excluded[a]	0	.0
	Total	178	100.0

a. Listwise deletion based on all variables in the procedure.

Reliability Statistics

Cronbach's Alpha	N of Items
.915	26

```
 **Risk During COVID-19*

FACTOR
  /VARIABLES Risk1 Risk2 Risk3 Risk4 Risk5 Risk6 Risk7 Risk8 Risk9 Risk10 Risk11 Risk12 R
isk13
```

```
    Risk14 Risk15 Risk16 Risk17 Risk18
  /MISSING LISTWISE
  /ANALYSIS Risk1 Risk2 Risk3 Risk4 Risk5 Risk6 Risk7 Risk8 Risk9 Risk10 Risk11 Risk12 Ri
sk13
    Risk14 Risk15 Risk16 Risk17 Risk18
  /PRINT INITIAL EXTRACTION ROTATION
  /FORMAT SORT
  /PLOT EIGEN
  /CRITERIA MINEIGEN(1) ITERATE(25)
  /EXTRACTION PC
  /CRITERIA ITERATE(25)
  /ROTATION VARIMAX
  /METHOD=CORRELATION.
```

Factor Analysis

Notes

Output Created		15-DEC-2021 13:06:13
Comments		
Input	Data	C:\Users\njs5478\Dropbox\HM and COVID\0. Revise and Resubmit\2. R and R Data\Study 1a\Study1a_Data.sav
	Active Dataset	DataSet1
	Filter	Inclusion = 1 (FILTER)
	Weight	<none>
	Split File	<none>
	N of Rows in Working Data File	178
Missing Value Handling	Definition of Missing	MISSING=EXCLUDE: User-defined missing values are treated as missing.
	Cases Used	LISTWISE: Statistics are based on cases with no missing values for any variable used.

Notes

Syntax		FACTOR<br>/VARIABLES Risk1 Risk2 Risk3 Risk4 Risk5 Risk6 Risk7 Risk8 Risk9 Risk10 Risk11 Risk12 Risk13<br>Risk14 Risk15 Risk16 Risk17 Risk18<br>/MISSING LISTWISE<br>/ANALYSIS Risk1 Risk2 Risk3 Risk4 Risk5 Risk6 Risk7 Risk8 Risk9 Risk10 Risk11 Risk12 Risk13<br>Risk14 Risk15 Risk16 Risk17 Risk18<br>/PRINT INITIAL EXTRACTION ROTATION<br>/FORMAT SORT<br>/PLOT EIGEN<br>/CRITERIA MINEIGEN (1) ITERATE(25)<br>/EXTRACTION PC<br>/CRITERIA ITERATE(25)<br>/ROTATION VARIMAX<br><br>/METHOD=CORRELATION.
Resources	Processor Time	00:00:03.31
	Elapsed Time	00:00:01.30
	Maximum Memory Required	40024 (39.086K) bytes

Communalities

	Initial	Extraction
For each of the following statements, please indicate how likely or unlikely you would be to engage in each activity or behavior during the period of COVID-19 (Coronavirus) social isolation. - Defending to family and close friends the belief that CoVID-19 is a serious risk.	1.000	.670
For each of the following statements, please indicate how likely or unlikely you would be to engage in each activity or behavior during the period of COVID-19 (Coronavirus) social isolation. - Disagreeing with your boss's decision that you should work remotely.	1.000	.413
For each of the following statements, please indicate how likely or unlikely you would be to engage in each activity or behavior during the period of COVID-19 (Coronavirus) social isolation. - Defending the need to self-quarantine on social media.	1.000	.543

Communalities

	Initial	Extraction
For each of the following statements, please indicate how likely or unlikely you would be to engage in each activity or behavior during the period of COVID-19 (Coronavirus) social isolation. - Cancelling a planned vacation because you were planning to fly there.	1.000	.438
For each of the following statements, please indicate how likely or unlikely you would be to engage in each activity or behavior during the period of COVID-19 (Coronavirus) social isolation. - Playing a pick-up sport with friends.	1.000	.665
For each of the following statements, please indicate how likely or unlikely you would be to engage in each activity or behavior during the period of COVID-19 (Coronavirus) social isolation. - Continuing to have friends over who do not live with you.	1.000	.572

Communalities

	Initial	Extraction
For each of the following statements, please indicate how likely or unlikely you would be to engage in each activity or behavior during the period of COVID-19 (Coronavirus) social isolation. - Regularly going to pick up take-out food.	1.000	.611
For each of the following statements, please indicate how likely or unlikely you would be to engage in each activity or behavior during the period of COVID-19 (Coronavirus) social isolation. - Asking the person behind you in line to step away to maintain a six-foot distance.	1.000	.479
For each of the following statements, please indicate how likely or unlikely you would be to engage in each activity or behavior during the period of COVID-19 (Coronavirus) social isolation. - Not wearing a mask when you go out in public.	1.000	.519

Communalities

	Initial	Extraction
For each of the following statements, please indicate how likely or unlikely you would be to engage in each activity or behavior during the period of COVID-19 (Coronavirus) social isolation. - Not washing your hands upon reentry to your home.	1.000	.426
For each of the following statements, please indicate how likely or unlikely you would be to engage in each activity or behavior during the period of COVID-19 (Coronavirus) social isolation. - Refusing to shake hands with acquaintances.	1.000	.702
For each of the following statements, please indicate how likely or unlikely you would be to engage in each activity or behavior during the period of COVID-19 (Coronavirus) social isolation. - Hugging friends you run into while grocery shopping.	1.000	.644

Communalities

	Initial	Extraction
For each of the following statements, please indicate how likely or unlikely you would be to engage in each activity or behavior during the period of COVID-19 (Coronavirus) social isolation. - Volunteering to distribute food at the local food bank.	1.000	.744
For each of the following statements, please indicate how likely or unlikely you would be to engage in each activity or behavior during the period of COVID-19 (Coronavirus) social isolation. - Volunteering at an understaffed medical facility.	1.000	.642
For each of the following statements, please indicate how likely or unlikely you would be to engage in each activity or behavior during the period of COVID-19 (Coronavirus) social isolation. - Distributing medical supplies to the homes of people who have been diagnosed with coronavirus so they do not have to go out in public.	1.000	.755

Communalities

	Initial	Extraction
For each of the following statements, please indicate how likely or unlikely you would be to engage in each activity or behavior during the period of COVID-19 (Coronavirus) social isolation. - Leaving your home when you feel ill.	1.000	.574
For each of the following statements, please indicate how likely or unlikely you would be to engage in each activity or behavior during the period of COVID-19 (Coronavirus) social isolation. - Going out in public for a break from care-taking for a friend/family member with coronavirus.	1.000	.448
For each of the following statements, please indicate how likely or unlikely you would be to engage in each activity or behavior during the period of COVID-19 (Coronavirus) social isolation. - Grocery shopping during hours reserved for high-risk individuals.	1.000	.405

Extraction Method: Principal Component Analysis.

Total Variance Explained

Component	Initial Eigenvalues			Extraction Sums of Squared Loadings		
	Total	% of Variance	Cumulative %	Total	% of Variance	Cumulative %
1	5.222	29.013	29.013	5.222	29.013	29.013
2	2.615	14.530	43.543	2.615	14.530	43.543
3	1.311	7.286	50.829	1.311	7.286	50.829
4	1.101	6.115	56.944	1.101	6.115	56.944
5	.955	5.304	62.248			
6	.862	4.792	67.039			
7	.821	4.562	71.601			
8	.743	4.125	75.726			
9	.637	3.540	79.267			
10	.583	3.240	82.507			
11	.547	3.039	85.546			
12	.499	2.774	88.319			
13	.451	2.507	90.826			
14	.419	2.328	93.154			
15	.366	2.033	95.187			
16	.306	1.701	96.889			
17	.292	1.625	98.513			
18	.268	1.487	100.000			

Total Variance Explained

Component	Rotation Sums of Squared Loadings		
	Total	% of Variance	Cumulative %
1	3.597	19.985	19.985
2	2.353	13.070	33.056
3	2.330	12.945	46.001
4	1.970	10.943	56.944
5			
6			
7			
8			
9			
10			
11			
12			
13			
14			
15			
16			
17			
18			

Extraction Method: Principal Component Analysis.

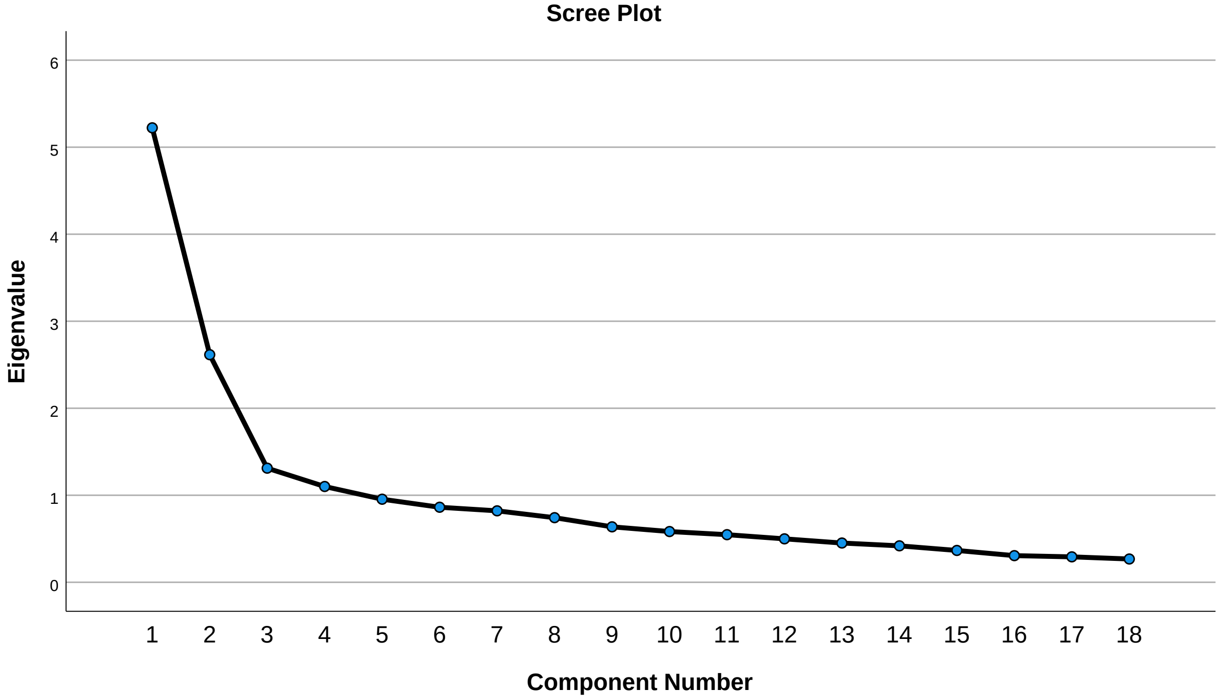

Component Matrix[a]

	Component			
	1	2	3	4
For each of the following statements, please indicate how likely or unlikely you would be to engage in each activity or behavior during the period of COVID-19 (Coronavirus) social isolation. - Playing a pick-up sport with friends.	.791	.190	-.020	-.047

Component Matrix[a]

	Component			
	1	2	3	4
For each of the following statements, please indicate how likely or unlikely you would be to engage in each activity or behavior during the period of COVID-19 (Coronavirus) social isolation. - Continuing to have friends over who do not live with you.	.748	.105	-.021	-.005
For each of the following statements, please indicate how likely or unlikely you would be to engage in each activity or behavior during the period of COVID-19 (Coronavirus) social isolation. - Hugging friends you run into while grocery shopping.	.731	.187	.248	-.112
For each of the following statements, please indicate how likely or unlikely you would be to engage in each activity or behavior during the period of COVID-19 (Coronavirus) social isolation. - Defending to family and close friends the belief that CoVID-19 is a serious risk.	-.631	.343	.326	.219

Component Matrix[a]

	Component			
	1	2	3	4
For each of the following statements, please indicate how likely or unlikely you would be to engage in each activity or behavior during the period of COVID-19 (Coronavirus) social isolation. - Leaving your home when you feel ill.	.620	.334	.198	.198
For each of the following statements, please indicate how likely or unlikely you would be to engage in each activity or behavior during the period of COVID-19 (Coronavirus) social isolation. - Refusing to shake hands with acquaintances.	-.599	.209	.125	.533
For each of the following statements, please indicate how likely or unlikely you would be to engage in each activity or behavior during the period of COVID-19 (Coronavirus) social isolation. - Grocery shopping during hours reserved for high-risk individuals.	.580	.233	.117	-.019

Component Matrix[a]

	Component			
	1	2	3	4
For each of the following statements, please indicate how likely or unlikely you would be to engage in each activity or behavior during the period of COVID-19 (Coronavirus) social isolation. - Not washing your hands upon reentry to your home.	.574	.103	.290	.043
For each of the following statements, please indicate how likely or unlikely you would be to engage in each activity or behavior during the period of COVID-19 (Coronavirus) social isolation. - Not wearing a mask when you go out in public.	.554	-.058	-.113	.443
For each of the following statements, please indicate how likely or unlikely you would be to engage in each activity or behavior during the period of COVID-19 (Coronavirus) social isolation. - Cancelling a planned vacation because you were planning to fly there.	-.524	.076	-.066	.392

Component Matrix[a]

	Component			
	1	2	3	4
For each of the following statements, please indicate how likely or unlikely you would be to engage in each activity or behavior during the period of COVID-19 (Coronavirus) social isolation. - Going out in public for a break from care-taking for a friend/family member with coronavirus.	.504	.110	.194	.381
For each of the following statements, please indicate how likely or unlikely you would be to engage in each activity or behavior during the period of COVID-19 (Coronavirus) social isolation. - Defending the need to self-quarantine on social media.	-.482	.445	.329	.066
For each of the following statements, please indicate how likely or unlikely you would be to engage in each activity or behavior during the period of COVID-19 (Coronavirus) social isolation. - Disagreeing with your boss’s decision that you should work remotely.	.467	.092	.432	-.009

Component Matrix[a]

	Component			
	1	2	3	4
For each of the following statements, please indicate how likely or unlikely you would be to engage in each activity or behavior during the period of COVID-19 (Coronavirus) social isolation. - Asking the person behind you in line to step away to maintain a six-foot distance.	-.457	.346	.356	-.154
For each of the following statements, please indicate how likely or unlikely you would be to engage in each activity or behavior during the period of COVID-19 (Coronavirus) social isolation. - Distributing medical supplies to the homes of people who have been diagnosed with coronavirus so they do not have to go out in public.	-.131	.794	-.313	-.099
For each of the following statements, please indicate how likely or unlikely you would be to engage in each activity or behavior during the period of COVID-19 (Coronavirus) social isolation. - Volunteering to distribute food at the local food bank.	-.057	.786	-.320	-.143

Component Matrix[a]

	Component			
	1	2	3	4
For each of the following statements, please indicate how likely or unlikely you would be to engage in each activity or behavior during the period of COVID-19 (Coronavirus) social isolation. - Volunteering at an understaffed medical facility.	.091	.775	-.171	-.069
For each of the following statements, please indicate how likely or unlikely you would be to engage in each activity or behavior during the period of COVID-19 (Coronavirus) social isolation. - Regularly going to pick up take-out food.	.414	.001	-.533	.393

Extraction Method: Principal Component Analysis.

a. 4 components extracted.

Rotated Component Matrix[a]

	Component			
	1	2	3	4
For each of the following statements, please indicate how likely or unlikely you would be to engage in each activity or behavior during the period of COVID-19 (Coronavirus) social isolation. - Hugging friends you run into while grocery shopping.	.717	.070	-.352	.038
For each of the following statements, please indicate how likely or unlikely you would be to engage in each activity or behavior during the period of COVID-19 (Coronavirus) social isolation. - Leaving your home when you feel ill.	.716	.170	-.030	.178
For each of the following statements, please indicate how likely or unlikely you would be to engage in each activity or behavior during the period of COVID-19 (Coronavirus) social isolation. - Not washing your hands upon reentry to your home.	.629	-.046	-.162	.050

Rotated Component Matrix[a]

	Component			
	1	2	3	4
For each of the following statements, please indicate how likely or unlikely you would be to engage in each activity or behavior during the period of COVID-19 (Coronavirus) social isolation. - Playing a pick-up sport with friends.	.627	.169	-.404	.282
For each of the following statements, please indicate how likely or unlikely you would be to engage in each activity or behavior during the period of COVID-19 (Coronavirus) social isolation. - Disagreeing with your boss's decision that you should work remotely.	.613	-.103	-.111	-.119
For each of the following statements, please indicate how likely or unlikely you would be to engage in each activity or behavior during the period of COVID-19 (Coronavirus) social isolation. - Going out in public for a break from care-taking for a friend/family member with coronavirus.	.596	-.057	.111	.280

Rotated Component Matrix[a]

	Component			
	1	2	3	4
For each of the following statements, please indicate how likely or unlikely you would be to engage in each activity or behavior during the period of COVID-19 (Coronavirus) social isolation. - Continuing to have friends over who do not live with you.	.578	.088	-.371	.305
For each of the following statements, please indicate how likely or unlikely you would be to engage in each activity or behavior during the period of COVID-19 (Coronavirus) social isolation. - Grocery shopping during hours reserved for high-risk individuals.	.565	.152	-.224	.109
For each of the following statements, please indicate how likely or unlikely you would be to engage in each activity or behavior during the period of COVID-19 (Coronavirus) social isolation. - Volunteering to distribute food at the local food bank.	-.005	.861	.038	-.038

Rotated Component Matrix[a]

	Component			
	1	2	3	4
For each of the following statements, please indicate how likely or unlikely you would be to engage in each activity or behavior during the period of COVID-19 (Coronavirus) social isolation. - Distributing medical supplies to the homes of people who have been diagnosed with coronavirus so they do not have to go out in public.	-.045	.859	.114	-.049
For each of the following statements, please indicate how likely or unlikely you would be to engage in each activity or behavior during the period of COVID-19 (Coronavirus) social isolation. - Volunteering at an understaffed medical facility.	.199	.774	.055	-.031
For each of the following statements, please indicate how likely or unlikely you would be to engage in each activity or behavior during the period of COVID-19 (Coronavirus) social isolation. - Refusing to shake hands with acquaintances.	-.202	.057	.808	-.065

Rotated Component Matrix[a]

	Component			
	1	2	3	4
For each of the following statements, please indicate how likely or unlikely you would be to engage in each activity or behavior during the period of COVID-19 (Coronavirus) social isolation. - Defending to family and close friends the belief that CoVID-19 is a serious risk.	-.138	.150	.672	-.421
For each of the following statements, please indicate how likely or unlikely you would be to engage in each activity or behavior during the period of COVID-19 (Coronavirus) social isolation. - Cancelling a planned vacation because you were planning to fly there.	-.322	.038	.576	.039
For each of the following statements, please indicate how likely or unlikely you would be to engage in each activity or behavior during the period of COVID-19 (Coronavirus) social isolation. - Defending the need to self-quarantine on social media.	-.025	.262	.504	-.469

Rotated Component Matrix[a]

	Component			
	1	2	3	4
For each of the following statements, please indicate how likely or unlikely you would be to engage in each activity or behavior during the period of COVID-19 (Coronavirus) social isolation. - Regularly going to pick up take-out food.	.096	.143	-.054	.760
For each of the following statements, please indicate how likely or unlikely you would be to engage in each activity or behavior during the period of COVID-19 (Coronavirus) social isolation. - Asking the person behind you in line to step away to maintain a six-foot distance.	-.068	.200	.303	-.585
For each of the following statements, please indicate how likely or unlikely you would be to engage in each activity or behavior during the period of COVID-19 (Coronavirus) social isolation. - Not wearing a mask when you go out in public.	.424	-.095	.008	.575

Extraction Method: Principal Component Analysis.
Rotation Method: Varimax with Kaiser Normalization.

a. Rotation converged in 7 iterations.

Component Transformation Matrix

Component	1	2	3	4
1	.745	-.021	-.520	.418
2	.311	.896	.258	-.187
3	.553	-.409	.266	-.675
4	.207	-.173	.770	.579

Extraction Method: Principal Component Analysis.
Rotation Method: Varimax with Kaiser Normalization.

```
*Force Two Factors*

FACTOR
  /VARIABLES Risk1 Risk2 Risk3 Risk4 Risk5 Risk6 Risk7 Risk8 Risk9 Risk10 Risk11 Risk12 R
isk13
    Risk14 Risk15 Risk16 Risk17 Risk18
  /MISSING LISTWISE
  /ANALYSIS Risk1 Risk2 Risk3 Risk4 Risk5 Risk6 Risk7 Risk8 Risk9 Risk10 Risk11 Risk12 Ri
sk13
    Risk14 Risk15 Risk16 Risk17 Risk18
  /PRINT INITIAL EXTRACTION ROTATION
  /FORMAT SORT
  /PLOT EIGEN
  /CRITERIA FACTORS(3) ITERATE(25)
  /EXTRACTION PC
  /CRITERIA ITERATE(25)
  /ROTATION VARIMAX
  /METHOD=CORRELATION.
```

Factor Analysis

Notes

Output Created		15-DEC-2021 13:06:14
Comments		
Input	Data	C: \Users\njs5478\Dropbox\HM and COVID\0. Revise and Resubmit\2. R and R Data\Study 1a\Study1a_Data.sav
	Active Dataset	DataSet1
	Filter	Inclusion = 1 (FILTER)
	Weight	<none>
	Split File	<none>
	N of Rows in Working Data File	178
Missing Value Handling	Definition of Missing	MISSING=EXCLUDE: User-defined missing values are treated as missing.
	Cases Used	LISTWISE: Statistics are based on cases with no missing values for any variable used.

Notes

Syntax		FACTOR<br>/VARIABLES Risk1 Risk2 Risk3 Risk4 Risk5 Risk6 Risk7 Risk8 Risk9 Risk10 Risk11 Risk12 Risk13<br>Risk14 Risk15 Risk16 Risk17 Risk18<br>/MISSING LISTWISE<br>/ANALYSIS Risk1 Risk2 Risk3 Risk4 Risk5 Risk6 Risk7 Risk8 Risk9 Risk10 Risk11 Risk12 Risk13<br>Risk14 Risk15 Risk16 Risk17 Risk18<br>/PRINT INITIAL EXTRACTION ROTATION<br>/FORMAT SORT<br>/PLOT EIGEN<br>/CRITERIA FACTORS(3) ITERATE(25)<br>/EXTRACTION PC<br>/CRITERIA ITERATE(25)<br>/ROTATION VARIMAX<br><br>/METHOD=CORRELATION.
Resources	Processor Time	00:00:00.41
	Elapsed Time	00:00:00.17
	Maximum Memory Required	40024 (39.086K) bytes

Communalities

	Initial	Extraction
For each of the following statements, please indicate how likely or unlikely you would be to engage in each activity or behavior during the period of COVID-19 (Coronavirus) social isolation. - Defending to family and close friends the belief that CoVID-19 is a serious risk.	1.000	.622
For each of the following statements, please indicate how likely or unlikely you would be to engage in each activity or behavior during the period of COVID-19 (Coronavirus) social isolation. - Disagreeing with your boss's decision that you should work remotely.	1.000	.413
For each of the following statements, please indicate how likely or unlikely you would be to engage in each activity or behavior during the period of COVID-19 (Coronavirus) social isolation. - Defending the need to self-quarantine on social media.	1.000	.539

Communalities

	Initial	Extraction
For each of the following statements, please indicate how likely or unlikely you would be to engage in each activity or behavior during the period of COVID-19 (Coronavirus) social isolation. - Cancelling a planned vacation because you were planning to fly there.	1.000	.285
For each of the following statements, please indicate how likely or unlikely you would be to engage in each activity or behavior during the period of COVID-19 (Coronavirus) social isolation. - Playing a pick-up sport with friends.	1.000	.662
For each of the following statements, please indicate how likely or unlikely you would be to engage in each activity or behavior during the period of COVID-19 (Coronavirus) social isolation. - Continuing to have friends over who do not live with you.	1.000	.572

Communalities

	Initial	Extraction
For each of the following statements, please indicate how likely or unlikely you would be to engage in each activity or behavior during the period of COVID-19 (Coronavirus) social isolation. - Regularly going to pick up take-out food.	1.000	.456
For each of the following statements, please indicate how likely or unlikely you would be to engage in each activity or behavior during the period of COVID-19 (Coronavirus) social isolation. - Asking the person behind you in line to step away to maintain a six-foot distance.	1.000	.455
For each of the following statements, please indicate how likely or unlikely you would be to engage in each activity or behavior during the period of COVID-19 (Coronavirus) social isolation. - Not wearing a mask when you go out in public.	1.000	.323

Communalities

	Initial	Extraction
For each of the following statements, please indicate how likely or unlikely you would be to engage in each activity or behavior during the period of COVID-19 (Coronavirus) social isolation. - Not washing your hands upon reentry to your home.	1.000	.424
For each of the following statements, please indicate how likely or unlikely you would be to engage in each activity or behavior during the period of COVID-19 (Coronavirus) social isolation. - Refusing to shake hands with acquaintances.	1.000	.418
For each of the following statements, please indicate how likely or unlikely you would be to engage in each activity or behavior during the period of COVID-19 (Coronavirus) social isolation. - Hugging friends you run into while grocery shopping.	1.000	.631

Communalities

	Initial	Extraction
For each of the following statements, please indicate how likely or unlikely you would be to engage in each activity or behavior during the period of COVID-19 (Coronavirus) social isolation. - Volunteering to distribute food at the local food bank.	1.000	.724
For each of the following statements, please indicate how likely or unlikely you would be to engage in each activity or behavior during the period of COVID-19 (Coronavirus) social isolation. - Volunteering at an understaffed medical facility.	1.000	.637
For each of the following statements, please indicate how likely or unlikely you would be to engage in each activity or behavior during the period of COVID-19 (Coronavirus) social isolation. - Distributing medical supplies to the homes of people who have been diagnosed with coronavirus so they do not have to go out in public.	1.000	.746

Communalities

	Initial	Extraction
For each of the following statements, please indicate how likely or unlikely you would be to engage in each activity or behavior during the period of COVID-19 (Coronavirus) social isolation. - Leaving your home when you feel ill.	1.000	.534
For each of the following statements, please indicate how likely or unlikely you would be to engage in each activity or behavior during the period of COVID-19 (Coronavirus) social isolation. - Going out in public for a break from care-taking for a friend/family member with coronavirus.	1.000	.303
For each of the following statements, please indicate how likely or unlikely you would be to engage in each activity or behavior during the period of COVID-19 (Coronavirus) social isolation. - Grocery shopping during hours reserved for high-risk individuals.	1.000	.404

Extraction Method: Principal Component Analysis.

Total Variance Explained

Component	Initial Eigenvalues			Extraction Sums of Squared Loadings		
	Total	% of Variance	Cumulative %	Total	% of Variance	Cumulative %
1	5.222	29.013	29.013	5.222	29.013	29.013
2	2.615	14.530	43.543	2.615	14.530	43.543
3	1.311	7.286	50.829	1.311	7.286	50.829
4	1.101	6.115	56.944			
5	.955	5.304	62.248			
6	.862	4.792	67.039			
7	.821	4.562	71.601			
8	.743	4.125	75.726			
9	.637	3.540	79.267			
10	.583	3.240	82.507			
11	.547	3.039	85.546			
12	.499	2.774	88.319			
13	.451	2.507	90.826			
14	.419	2.328	93.154			
15	.366	2.033	95.187			
16	.306	1.701	96.889			
17	.292	1.625	98.513			
18	.268	1.487	100.000			

Total Variance Explained

Component	Rotation Sums of Squared Loadings		
	Total	% of Variance	Cumulative %
1	3.950	21.947	21.947
2	2.848	15.821	37.768
3	2.351	13.060	50.829
4			
5			
6			
7			
8			
9			
10			
11			
12			
13			
14			
15			
16			
17			
18			

Extraction Method: Principal Component Analysis.

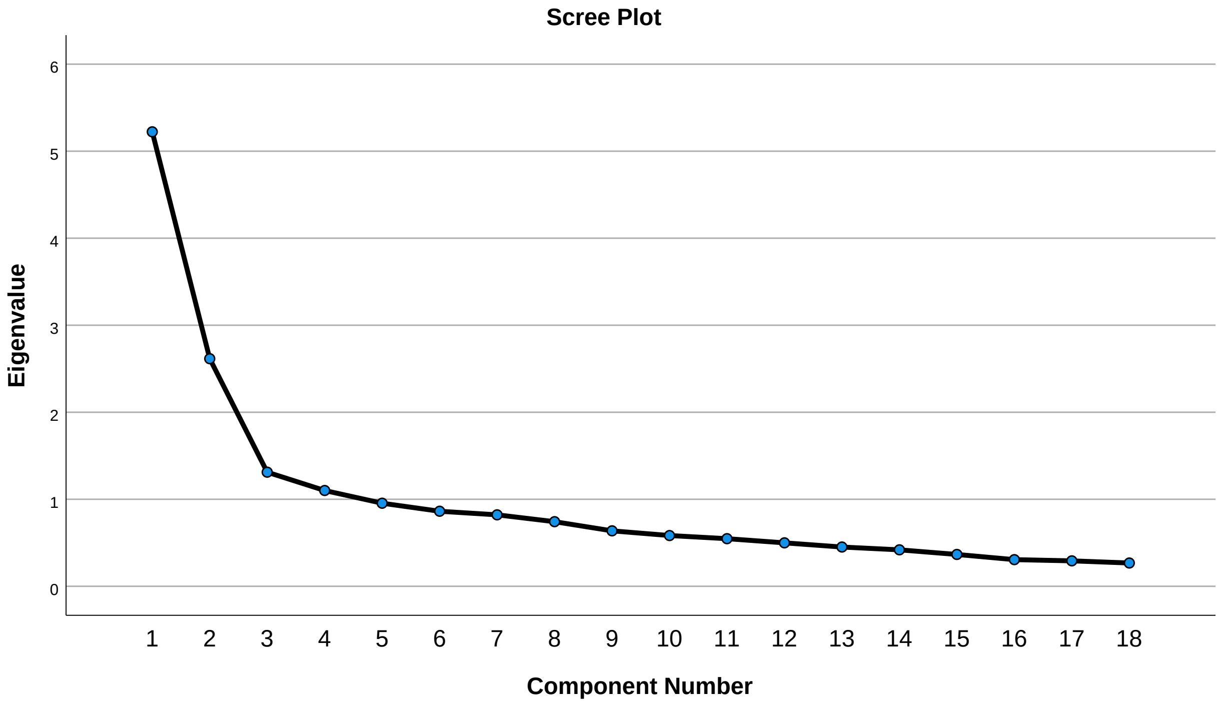

Component Matrix[a]

	Component		
	1	2	3
For each of the following statements, please indicate how likely or unlikely you would be to engage in each activity or behavior during the period of COVID-19 (Coronavirus) social isolation. - Playing a pick-up sport with friends.	.791	.190	-.020

Component Matrix[a]

	Component		
	1	2	3
For each of the following statements, please indicate how likely or unlikely you would be to engage in each activity or behavior during the period of COVID-19 (Coronavirus) social isolation. - Continuing to have friends over who do not live with you.	.748	.105	-.021
For each of the following statements, please indicate how likely or unlikely you would be to engage in each activity or behavior during the period of COVID-19 (Coronavirus) social isolation. - Hugging friends you run into while grocery shopping.	.731	.187	.248
For each of the following statements, please indicate how likely or unlikely you would be to engage in each activity or behavior during the period of COVID-19 (Coronavirus) social isolation. - Defending to family and close friends the belief that CoVID-19 is a serious risk.	-.631	.343	.326

Component Matrix[a]

	Component		
	1	2	3
For each of the following statements, please indicate how likely or unlikely you would be to engage in each activity or behavior during the period of COVID-19 (Coronavirus) social isolation. - Leaving your home when you feel ill.	.620	.334	.198
For each of the following statements, please indicate how likely or unlikely you would be to engage in each activity or behavior during the period of COVID-19 (Coronavirus) social isolation. - Refusing to shake hands with acquaintances.	-.599	.209	.125
For each of the following statements, please indicate how likely or unlikely you would be to engage in each activity or behavior during the period of COVID-19 (Coronavirus) social isolation. - Grocery shopping during hours reserved for high-risk individuals.	.580	.233	.117

Component Matrix[a]

	Component		
	1	2	3
For each of the following statements, please indicate how likely or unlikely you would be to engage in each activity or behavior during the period of COVID-19 (Coronavirus) social isolation. - Not washing your hands upon reentry to your home.	.574	.103	.290
For each of the following statements, please indicate how likely or unlikely you would be to engage in each activity or behavior during the period of COVID-19 (Coronavirus) social isolation. - Not wearing a mask when you go out in public.	.554	-.058	-.113
For each of the following statements, please indicate how likely or unlikely you would be to engage in each activity or behavior during the period of COVID-19 (Coronavirus) social isolation. - Cancelling a planned vacation because you were planning to fly there.	-.524	.076	-.066

Component Matrix[a]

	Component		
	1	2	3
For each of the following statements, please indicate how likely or unlikely you would be to engage in each activity or behavior during the period of COVID-19 (Coronavirus) social isolation. - Going out in public for a break from care-taking for a friend/family member with coronavirus.	.504	.110	.194
For each of the following statements, please indicate how likely or unlikely you would be to engage in each activity or behavior during the period of COVID-19 (Coronavirus) social isolation. - Defending the need to self-quarantine on social media.	-.482	.445	.329
For each of the following statements, please indicate how likely or unlikely you would be to engage in each activity or behavior during the period of COVID-19 (Coronavirus) social isolation. - Disagreeing with your boss's decision that you should work remotely.	.467	.092	.432

Component Matrix[a]

	Component		
	1	2	3
For each of the following statements, please indicate how likely or unlikely you would be to engage in each activity or behavior during the period of COVID-19 (Coronavirus) social isolation. - Asking the person behind you in line to step away to maintain a six-foot distance.	-.457	.346	.356
For each of the following statements, please indicate how likely or unlikely you would be to engage in each activity or behavior during the period of COVID-19 (Coronavirus) social isolation. - Distributing medical supplies to the homes of people who have been diagnosed with coronavirus so they do not have to go out in public.	-.131	.794	-.313
For each of the following statements, please indicate how likely or unlikely you would be to engage in each activity or behavior during the period of COVID-19 (Coronavirus) social isolation. - Volunteering to distribute food at the local food bank.	-.057	.786	-.320

Component Matrix[a]

	Component		
	1	2	3
For each of the following statements, please indicate how likely or unlikely you would be to engage in each activity or behavior during the period of COVID-19 (Coronavirus) social isolation. - Volunteering at an understaffed medical facility.	.091	.775	-.171
For each of the following statements, please indicate how likely or unlikely you would be to engage in each activity or behavior during the period of COVID-19 (Coronavirus) social isolation. - Regularly going to pick up take-out food.	.414	.001	-.533

Extraction Method: Principal Component Analysis.

a. 3 components extracted.

Rotated Component Matrix[a]

	Component		
	1	2	3
For each of the following statements, please indicate how likely or unlikely you would be to engage in each activity or behavior during the period of COVID-19 (Coronavirus) social isolation. - Hugging friends you run into while grocery shopping.	.771	-.187	.044
For each of the following statements, please indicate how likely or unlikely you would be to engage in each activity or behavior during the period of COVID-19 (Coronavirus) social isolation. - Leaving your home when you feel ill.	.695	-.105	.199
For each of the following statements, please indicate how likely or unlikely you would be to engage in each activity or behavior during the period of COVID-19 (Coronavirus) social isolation. - Playing a pick-up sport with friends.	.680	-.416	.167

Rotated Component Matrix[a]

	Component		
	1	2	3
For each of the following statements, please indicate how likely or unlikely you would be to engage in each activity or behavior during the period of COVID-19 (Coronavirus) social isolation. - Not washing your hands upon reentry to your home.	.643	-.094	-.047
For each of the following statements, please indicate how likely or unlikely you would be to engage in each activity or behavior during the period of COVID-19 (Coronavirus) social isolation. - Disagreeing with your boss's decision that you should work remotely.	.628	.069	-.120
For each of the following statements, please indicate how likely or unlikely you would be to engage in each activity or behavior during the period of COVID-19 (Coronavirus) social isolation. - Continuing to have friends over who do not live with you.	.621	-.421	.092

Rotated Component Matrix[a]

	Component		
	1	2	3
For each of the following statements, please indicate how likely or unlikely you would be to engage in each activity or behavior during the period of COVID-19 (Coronavirus) social isolation. - Grocery shopping during hours reserved for high-risk individuals.	.593	-.176	.147
For each of the following statements, please indicate how likely or unlikely you would be to engage in each activity or behavior during the period of COVID-19 (Coronavirus) social isolation. - Going out in public for a break from care-taking for a friend/family member with coronavirus.	.538	-.119	.003
For each of the following statements, please indicate how likely or unlikely you would be to engage in each activity or behavior during the period of COVID-19 (Coronavirus) social isolation. - Cancelling a planned vacation because you were planning to fly there.	-.436	.289	.105

Rotated Component Matrix[a]

	Component		
	1	2	3
For each of the following statements, please indicate how likely or unlikely you would be to engage in each activity or behavior during the period of COVID-19 (Coronavirus) social isolation. - Defending to family and close friends the belief that CoVID-19 is a serious risk.	-.243	.731	.169
For each of the following statements, please indicate how likely or unlikely you would be to engage in each activity or behavior during the period of COVID-19 (Coronavirus) social isolation. - Defending the need to self-quarantine on social media.	-.093	.682	.256
For each of the following statements, please indicate how likely or unlikely you would be to engage in each activity or behavior during the period of COVID-19 (Coronavirus) social isolation. - Asking the person behind you in line to step away to maintain a six-foot distance.	-.086	.651	.155

Rotated Component Matrix[a]

	Component		
	1	2	3
For each of the following statements, please indicate how likely or unlikely you would be to engage in each activity or behavior during the period of COVID-19 (Coronavirus) social isolation. - Regularly going to pick up take-out food.	.055	-.630	.236
For each of the following statements, please indicate how likely or unlikely you would be to engage in each activity or behavior during the period of COVID-19 (Coronavirus) social isolation. - Refusing to shake hands with acquaintances.	-.359	.519	.140
For each of the following statements, please indicate how likely or unlikely you would be to engage in each activity or behavior during the period of COVID-19 (Coronavirus) social isolation. - Not wearing a mask when you go out in public.	.371	-.430	-.009

Rotated Component Matrix[a]

	Component		
	1	2	3
For each of the following statements, please indicate how likely or unlikely you would be to engage in each activity or behavior during the period of COVID-19 (Coronavirus) social isolation. - Distributing medical supplies to the homes of people who have been diagnosed with coronavirus so they do not have to go out in public.	-.049	.133	.852
For each of the following statements, please indicate how likely or unlikely you would be to engage in each activity or behavior during the period of COVID-19 (Coronavirus) social isolation. - Volunteering to distribute food at the local food bank.	.005	.082	.847
For each of the following statements, please indicate how likely or unlikely you would be to engage in each activity or behavior during the period of COVID-19 (Coronavirus) social isolation. - Volunteering at an understaffed medical facility.	.199	.098	.767

Extraction Method: Principal Component Analysis.
Rotation Method: Varimax with Kaiser Normalization.

a. Rotation converged in 5 iterations.

Component Transformation Matrix

Component	1	2	3
1	.806	-.592	-.015
2	.278	.355	.892
3	.523	.723	-.451

Extraction Method: Principal Component Analysis.
Rotation Method: Varimax with Kaiser Normalization.

```
RECODE Risk1 Risk3 Risk4 Risk8 Risk11 (1=7) (2=6) (3=5) (4=4) (5=3) (6=2) (7=1) INTO Risk
1_R Risk3_R Risk4_R Risk8_R Risk11_R.
EXECUTE.

RELIABILITY
  /VARIABLES= Risk13 Risk14 Risk15
  /SCALE('ALL VARIABLES') ALL
  /MODEL=ALPHA.
```

Reliability

Notes

Output Created		15-DEC-2021 13:06:14
Comments		
Input	Data	C:\Users\njs5478\Dropbox\HM and COVID\0. Revise and Resubmit\2. R and R Data\Study 1a\Study1a_Data.sav
	Active Dataset	DataSet1
	Filter	Inclusion = 1 (FILTER)
	Weight	<none>
	Split File	<none>
	N of Rows in Working Data File	178
	Matrix Input	
Missing Value Handling	Definition of Missing	User-defined missing values are treated as missing.
	Cases Used	Statistics are based on all cases with valid data for all variables in the procedure.
Syntax		RELIABILITY /VARIABLES= Risk13 Risk14 Risk15 /SCALE('ALL VARIABLES') ALL /MODEL=ALPHA.
Resources	Processor Time	00:00:00.02
	Elapsed Time	00:00:00.02

Scale: ALL VARIABLES

Case Processing Summary

		N	%
Cases	Valid	178	100.0
	Excluded[a]	0	.0
	Total	178	100.0

a. Listwise deletion based on all variables in the procedure.

Reliability Statistics

Cronbach's Alpha	N of Items
.806	3

```
RELIABILITY
  /VARIABLES=Risk1_R Risk2 Risk3_R Risk4_R Risk5 Risk6 Risk7 Risk8_R Risk9 Risk10 Risk11_
R Risk12 Risk16 Risk17 Risk18
  /SCALE('ALL VARIABLES') ALL
  /MODEL=ALPHA.
```

Reliability

Notes

Output Created		15-DEC-2021 13:06:14
Comments		
Input	Data	C: \Users\njs5478\Dropbox\HM and COVID\0. Revise and Resubmit\2. R and R Data\Study 1a\Study1a_Data.sav
	Active Dataset	DataSet1
	Filter	Inclusion = 1 (FILTER)
	Weight	<none>
	Split File	<none>
	N of Rows in Working Data File	178
	Matrix Input	
Missing Value Handling	Definition of Missing	User-defined missing values are treated as missing.
	Cases Used	Statistics are based on all cases with valid data for all variables in the procedure.

Notes

Syntax		RELIABILITY<br>/VARIABLES=Risk1_R Risk2 Risk3_R Risk4_R Risk5 Risk6 Risk7 Risk8_R Risk9 Risk10 Risk11_R Risk12 Risk16 Risk17 Risk18<br>/SCALE('ALL VARIABLES') ALL<br>/MODEL=ALPHA.
Resources	Processor Time	00:00:00.02
	Elapsed Time	00:00:00.02

Scale: ALL VARIABLES

Case Processing Summary

		N	%
Cases	Valid	178	100.0
	Excluded[a]	0	.0
	Total	178	100.0

a. Listwise deletion based on all variables in the procedure.

Reliability Statistics

Cronbach's Alpha	N of Items
.853	15

```
COMPUTE Risk_Rules = (Risk1_R + Risk2 + Risk3_R + Risk4_R + Risk5 + Risk6 + Risk7 + Risk8
_R + Risk9 + Risk10 + Risk11_R + Risk12 + Risk16 + Risk17 + Risk18)/15.

COMPUTE Risk_Help=(Risk13 + Risk14 + RIsk15)/3.

**Coronavirus Concern**

RECODE Concern3 (1=7) (2=6) (3=5) (4=4) (5=3) (6=2) (7=1) INTO Concern3_R.
EXECUTE.

RELIABILITY
```

```
/VARIABLES=Concern1 Concern2 Concern3_R Concern4 Concern5 Concern6
/SCALE('ALL VARIABLES') ALL
/MODEL=ALPHA
/SUMMARY=TOTAL.
```

Reliability

Notes

Output Created		15-DEC-2021 13:06:14
Comments		
Input	Data	C:\Users\njs5478\Dropbox\HM and COVID\0. Revise and Resubmit\2. R and R Data\Study 1a\Study1a_Data.sav
	Active Dataset	DataSet1
	Filter	Inclusion = 1 (FILTER)
	Weight	<none>
	Split File	<none>
	N of Rows in Working Data File	178
	Matrix Input	
Missing Value Handling	Definition of Missing	User-defined missing values are treated as missing.
	Cases Used	Statistics are based on all cases with valid data for all variables in the procedure.
Syntax		RELIABILITY /VARIABLES=Concern1 Concern2 Concern3_R Concern4 Concern5 Concern6 /SCALE('ALL VARIABLES') ALL /MODEL=ALPHA /SUMMARY=TOTAL.
Resources	Processor Time	00:00:00.02
	Elapsed Time	00:00:00.02

Scale: ALL VARIABLES

Case Processing Summary

		N	%
Cases	Valid	178	100.0
	Excluded[a]	0	.0
	Total	178	100.0

a. Listwise deletion based on all variables in the procedure.

Reliability Statistics

Cronbach's Alpha	N of Items
.893	6

Item-Total Statistics

	Scale Mean if Item Deleted	Scale Variance if Item Deleted	Corrected Item-Total Correlation	Cronbach's Alpha if Item Deleted
Please indicate your agreement with each statement using the scale provided: - Thinking about the coronavirus (COVID-19) makes me feel threatened.	24.4157	52.685	.750	.869
Please indicate your agreement with each statement using the scale provided: - I am afraid of the coronavirus (COVID-19).	23.9382	48.363	.843	.852
Concern3_R	23.4045	52.039	.697	.877

Item-Total Statistics

	Scale Mean if Item Deleted	Scale Variance if Item Deleted	Corrected Item-Total Correlation	Cronbach's Alpha if Item Deleted
Please indicate your agreement with each statement using the scale provided: - I am worried that I or people I love will get sick from the coronavirus (COVID-19).	22.8090	57.884	.640	.886
Please indicate your agreement with each statement using the scale provided: - I am stressed around other people because I worry I'll catch the coronavirus (COVID-19).	24.0730	50.023	.747	.869
Please indicate your agreement with each statement using the scale provided: - I have tried hard to avoid other people because I don't want to get sick.	23.0169	55.892	.618	.889

```
 COMPUTE Concern_Tot=mean(concern1, Concern2, Concern3_R, Concern4, Concern5, Concern6).

**Financial

RECODE Finance3 (1=7) (2=6) (3=5) (4=4) (5=3) (6=2) (7=1) INTO Finance3_R.
EXECUTE.

RELIABILITY
  /VARIABLES=Finance1 Finance2 Finance3_R
  /SCALE('ALL VARIABLES') ALL
  /MODEL=ALPHA
  /SUMMARY=TOTAL.
```

Reliability

Notes

Output Created		15-DEC-2021 13:06:14
Comments		
Input	Data	C: \Users\njs5478\Dropbox\HM and COVID\0. Revise and Resubmit\2. R and R Data\Study 1a\Study1a_Data.sav
	Active Dataset	DataSet1
	Filter	Inclusion = 1 (FILTER)
	Weight	<none>
	Split File	<none>
	N of Rows in Working Data File	178
	Matrix Input	
Missing Value Handling	Definition of Missing	User-defined missing values are treated as missing.
	Cases Used	Statistics are based on all cases with valid data for all variables in the procedure.
Syntax		RELIABILITY /VARIABLES=Finance1 Finance2 Finance3_R /SCALE('ALL VARIABLES') ALL /MODEL=ALPHA /SUMMARY=TOTAL.
Resources	Processor Time	00:00:00.00
	Elapsed Time	00:00:00.00

Scale: ALL VARIABLES

Case Processing Summary

		N	%
Cases	Valid	178	100.0
	Excluded[a]	0	.0
	Total	178	100.0

a. Listwise deletion based on all variables in the procedure.

Reliability Statistics

Cronbach's Alpha	N of Items
.769	3

Item-Total Statistics

	Scale Mean if Item Deleted	Scale Variance if Item Deleted	Corrected Item-Total Correlation	Cronbach's Alpha if Item Deleted
Please indicate your agreement with each statement using the scale provided: - The Coronavirus (COVID-19) has impacted me negatively from a financial point of view.	8.0281	11.157	.691	.590
Please indicate your agreement with each statement using the scale provided: - I have lost job-related income due to the Coronavirus (COVID-19).	8.5000	12.138	.513	.793
Finance3_R	7.4494	12.045	.614	.678

```
 COMPUTE Finance_Tot=mean(Finance1, Finance2, Finance3_R).

*Resources

RECODE Resource3 (1=7) (2=6) (3=5) (4=4) (5=3) (6=2) (7=1) INTO Resource3_R.
EXECUTE.

RELIABILITY
  /VARIABLES=Resource1 Resource2 Resource3_R
  /SCALE('ALL VARIABLES') ALL
  /MODEL=ALPHA
  /SUMMARY=TOTAL.
```

Reliability

Notes

Output Created		15-DEC-2021 13:06:14
Comments		
Input	Data	C:\Users\njs5478\Dropbox\HM and COVID\0. Revise and Resubmit\2. R and R Data\Study 1a\Study1a_Data.sav
	Active Dataset	DataSet1
	Filter	Inclusion = 1 (FILTER)
	Weight	<none>
	Split File	<none>
	N of Rows in Working Data File	178
	Matrix Input	
Missing Value Handling	Definition of Missing	User-defined missing values are treated as missing.
	Cases Used	Statistics are based on all cases with valid data for all variables in the procedure.
Syntax		RELIABILITY /VARIABLES=Resource1 Resource2 Resource3_R /SCALE('ALL VARIABLES') ALL /MODEL=ALPHA /SUMMARY=TOTAL.
Resources	Processor Time	00:00:00.00
	Elapsed Time	00:00:00.00

Scale: ALL VARIABLES

Case Processing Summary

		N	%
Cases	Valid	178	100.0
	Excluded[a]	0	.0
	Total	178	100.0

a. Listwise deletion based on all variables in the procedure.

Reliability Statistics

Cronbach's Alpha	N of Items
.785	3

Item-Total Statistics

	Scale Mean if Item Deleted	Scale Variance if Item Deleted	Corrected Item-Total Correlation	Cronbach's Alpha if Item Deleted
Please indicate your agreement with each statement using the scale provided: - I have had a hard time getting needed resources (food, toilet paper) due to the Coronavirus (COVID-19).	7.9831	9.700	.628	.704
Please indicate your agreement with each statement using the scale provided: - It has been difficult for me to get the things I need due to the Coronavirus (COVID-19).	7.2472	9.509	.652	.678
Resource3_R	7.3202	9.970	.592	.742

```
 COMPUTE Resource_Tot=mean(Resource1, Resource2, Resource3_R).

*Psychology

RECODE Psychology3 (1=7) (2=6) (3=5) (4=4) (5=3) (6=2) (7=1) INTO Psychology3_R.
EXECUTE.

RELIABILITY
  /VARIABLES=Psychology1 Psychology2 Psychology3_R
  /SCALE('ALL VARIABLES') ALL
  /MODEL=ALPHA
  /SUMMARY=TOTAL.
```

Reliability

Notes

Output Created		15-DEC-2021 13:06:15
Comments		
Input	Data	C: \Users\njs5478\Dropbox\HM and COVID\0. Revise and Resubmit\2. R and R Data\Study 1a\Study1a_Data.sav
	Active Dataset	DataSet1
	Filter	Inclusion = 1 (FILTER)
	Weight	<none>
	Split File	<none>
	N of Rows in Working Data File	178
	Matrix Input	
Missing Value Handling	Definition of Missing	User-defined missing values are treated as missing.
	Cases Used	Statistics are based on all cases with valid data for all variables in the procedure.
Syntax		RELIABILITY /VARIABLES=Psychology1 Psychology2 Psychology3_R /SCALE('ALL VARIABLES') ALL /MODEL=ALPHA /SUMMARY=TOTAL.
Resources	Processor Time	00:00:00.00
	Elapsed Time	00:00:00.00

Scale: ALL VARIABLES

Case Processing Summary

		N	%
Cases	Valid	178	100.0
	Excluded[a]	0	.0
	Total	178	100.0

a. Listwise deletion based on all variables in the procedure.

Reliability Statistics

Cronbach's Alpha	N of Items
.839	3

Item-Total Statistics

	Scale Mean if Item Deleted	Scale Variance if Item Deleted	Corrected Item-Total Correlation	Cronbach's Alpha if Item Deleted
Please indicate your agreement with each statement using the scale provided: - I have become depressed because of the Coronavirus (COVID-19).	9.4157	9.171	.714	.765
Please indicate your agreement with each statement using the scale provided: - The Coronavirus (COVID-19) outbreak has impacted my psychological health negatively.	8.4101	9.577	.741	.740
Psychology3_R	8.2079	10.188	.655	.821

```
 COMPUTE Psychology_Tot=mean(Psychology1, Psychology2, Psychology3_R).

*Personal Covid*

RELIABILITY
  /VARIABLES=Covid1 Covid2 Covid3 Covid4 Covid5 Covid6 Covid7
  /SCALE('ALL VARIABLES') ALL
  /MODEL=ALPHA
  /SUMMARY=TOTAL.
```

Reliability

Notes

Output Created		15-DEC-2021 13:06:15
Comments		
Input	Data	C:\Users\njs5478\Dropbox\HM and COVID\0. Revise and Resubmit\2. R and R Data\Study 1a\Study1a_Data.sav
	Active Dataset	DataSet1
	Filter	Inclusion = 1 (FILTER)
	Weight	<none>
	Split File	<none>
	N of Rows in Working Data File	178
	Matrix Input	
Missing Value Handling	Definition of Missing	User-defined missing values are treated as missing.
	Cases Used	Statistics are based on all cases with valid data for all variables in the procedure.
Syntax		RELIABILITY /VARIABLES=Covid1 Covid2 Covid3 Covid4 Covid5 Covid6 Covid7 /SCALE('ALL VARIABLES') ALL /MODEL=ALPHA /SUMMARY=TOTAL.
Resources	Processor Time	00:00:00.00
	Elapsed Time	00:00:00.00

Scale: ALL VARIABLES

Case Processing Summary

		N	%
Cases	Valid	178	100.0
	Excluded[a]	0	.0
	Total	178	100.0

a. Listwise deletion based on all variables in the procedure.

Reliability Statistics

Cronbach's Alpha	N of Items
.454	7

Item-Total Statistics

	Scale Mean if Item Deleted	Scale Variance if Item Deleted	Corrected Item-Total Correlation	Cronbach's Alpha if Item Deleted
I have been diagnosed with coronavirus (COVID-19).	10.47	1.494	.000	.467
I have had coronavirus-like symptoms at some point in the last two months.	10.61	1.109	.359	.344
I have been sick with something other than the coronavirus in the last two months.	10.78	1.133	.148	.461
I have been in close proximity with someone who has been diagnosed with coronavirus (COVID-19).	10.52	1.347	.214	.426
I have been in close proximity with someone who has had coronavirus-like symptoms in the last two months.	10.64	1.034	.418	.305
I watch a lot of news about the Coronavirus (COVID-19).	11.13	1.096	.171	.449
I spent a huge percentage of my time trying to find updates online or on TV about Coronavirus (COVID-19).	10.68	1.168	.182	.433

```
FREQUENCIES VARIABLES=Covid1 Covid2 Covid3 Covid4 Covid5 Covid6 Covid7
 /ORDER=ANALYSIS.
```

Frequencies

Notes

Output Created		15-DEC-2021 13:06:15
Comments		
Input	Data	C:\Users\njs5478\Dropbox\HM and COVID\0. Revise and Resubmit\2. R and R Data\Study 1a\Study1a_Data.sav
	Active Dataset	DataSet1
	Filter	Inclusion = 1 (FILTER)
	Weight	<none>
	Split File	<none>
	N of Rows in Working Data File	178
Missing Value Handling	Definition of Missing	User-defined missing values are treated as missing.
	Cases Used	Statistics are based on all cases with valid data.
Syntax		FREQUENCIES VARIABLES=Covid1 Covid2 Covid3 Covid4 Covid5 Covid6 Covid7 /ORDER=ANALYSIS.
Resources	Processor Time	00:00:00.02
	Elapsed Time	00:00:00.02

Statistics

		I have been diagnosed with coronavirus (COVID-19).	I have had coronavirus-like symptoms at some point in the last two months.	I have been sick with something other than the coronavirus in the last two months.	I have been in close proximity with someone who has been diagnosed with coronavirus (COVID-19).	I have been in close proximity with someone who has had coronavirus-like symptoms in the last two months.
N	Valid	178	178	178	178	178
	Missing	0	0	0	0	0

Statistics

		I watch a lot of news about the Coronavirus (COVID-19).	I spent a huge percentage of my time trying to find updates online or on TV about Coronavirus (COVID-19).
N	Valid	178	178
	Missing	0	0

Frequency Table

I have been diagnosed with coronavirus (COVID-19).

		Frequency	Percent	Valid Percent	Cumulative Percent
Valid	No	178	100.0	100.0	100.0

I have had coronavirus-like symptoms at some point in the last two months.

		Frequency	Percent	Valid Percent	Cumulative Percent
Valid	Yes	25	14.0	14.0	14.0
	No	153	86.0	86.0	100.0
	Total	178	100.0	100.0	

I have been sick with something other than the coronavirus in the last two months.

		Frequency	Percent	Valid Percent	Cumulative Percent
Valid	Yes	55	30.9	30.9	30.9
	No	123	69.1	69.1	100.0
	Total	178	100.0	100.0	

I have been in close proximity with someone who has been diagnosed with coronavirus (COVID-19).

		Frequency	Percent	Valid Percent	Cumulative Percent
Valid	Yes	8	4.5	4.5	4.5
	No	170	95.5	95.5	100.0
	Total	178	100.0	100.0	

I have been in close proximity with someone who has had coronavirus-like symptoms in the last two months.

		Frequency	Percent	Valid Percent	Cumulative Percent
Valid	Yes	30	16.9	16.9	16.9
	No	148	83.1	83.1	100.0
	Total	178	100.0	100.0	

I watch a lot of news about the Coronavirus (COVID-19).

		Frequency	Percent	Valid Percent	Cumulative Percent
Valid	Yes	117	65.7	65.7	65.7
	No	61	34.3	34.3	100.0
	Total	178	100.0	100.0	

I spent a huge percentage of my time trying to find updates online or on TV about Coronavirus (COVID-19).

		Frequency	Percent	Valid Percent	Cumulative Percent
Valid	Yes	37	20.8	20.8	20.8
	No	141	79.2	79.2	100.0
	Total	178	100.0	100.0	

```
RELIABILITY
 /VARIABLES=Covid6 Covid7
 /SCALE('ALL VARIABLES') ALL
 /MODEL=ALPHA
 /SUMMARY=TOTAL.
```

Reliability

Notes

Output Created		15-DEC-2021 13:06:15
Comments		
Input	Data	C:\Users\njs5478\Dropbox\HM and COVID\0. Revise and Resubmit\2. R and R Data\Study 1a\Study1a_Data.sav
	Active Dataset	DataSet1
	Filter	Inclusion = 1 (FILTER)
	Weight	<none>
	Split File	<none>
	N of Rows in Working Data File	178
	Matrix Input	
Missing Value Handling	Definition of Missing	User-defined missing values are treated as missing.
	Cases Used	Statistics are based on all cases with valid data for all variables in the procedure.
Syntax		RELIABILITY /VARIABLES=Covid6 Covid7 /SCALE('ALL VARIABLES') ALL /MODEL=ALPHA /SUMMARY=TOTAL.
Resources	Processor Time	00:00:00.00
	Elapsed Time	00:00:00.00

Scale: ALL VARIABLES

Case Processing Summary

		N	%
Cases	Valid	178	100.0
	Excluded[a]	0	.0
	Total	178	100.0

a. Listwise deletion based on all variables in the procedure.

Reliability Statistics

Cronbach's Alpha	N of Items
.504	2

Item-Total Statistics

	Scale Mean if Item Deleted	Scale Variance if Item Deleted	Corrected Item-Total Correlation	Cronbach's Alpha if Item Deleted
I watch a lot of news about the Coronavirus (COVID-19).	1.79	.166	.341	.
I spent a huge percentage of my time trying to find updates online or on TV about Coronavirus (COVID-19).	1.34	.227	.341	.

```
 **Political Identity

CORRELATIONS
  /VARIABLES=PParty PIdeology
  /PRINT=TWOTAIL NOSIG FULL
  /MISSING=PAIRWISE.
```

Correlations

Notes

Output Created		15-DEC-2021 13:06:15
Comments		
Input	Data	C: \Users\njs5478\Dropbox\HM and COVID\0. Revise and Resubmit\2. R and R Data\Study 1a\Study1a_Data.sav
	Active Dataset	DataSet1
	Filter	Inclusion = 1 (FILTER)
	Weight	<none>
	Split File	<none>
	N of Rows in Working Data File	178
Missing Value Handling	Definition of Missing	User-defined missing values are treated as missing.
	Cases Used	Statistics for each pair of variables are based on all the cases with valid data for that pair.
Syntax		CORRELATIONS /VARIABLES=PParty PIdeology /PRINT=TWOTAIL NOSIG FULL /MISSING=PAIRWISE.
Resources	Processor Time	00:00:00.03
	Elapsed Time	00:00:00.02

Correlations

		Which of the following best describes your political party affiliation?	Which of the following best describes your political ideology?
Which of the following best describes your political party affiliation?	Pearson Correlation	1	.811**
	Sig. (2-tailed)		.000
	N	178	178
Which of the following best describes your political ideology?	Pearson Correlation	.811**	1
	Sig. (2-tailed)	.000	
	N	178	178

**. Correlation is significant at the 0.01 level (2-tailed).

```
 **New variable removing those who had no opinion (x=8 on the scale)

IF (Trump<8) TrumpX=Trump.
IF(Biden<8) BidenX=Biden.
IF (Pelosi<8) PelosiX=Pelosi.
IF (McConnell<8) McConnellX=McConnell.
IF (RepCongress<8) RepCongressX=RepCongress.
IF (DemCongress<8) DemCongressX=DemCongress.
IF(Fauci<8) FauciX=Fauci.
IF(State<8)StateX=State.

**Descriptives and Frequencies**

FREQUENCIES VARIABLES=PParty PIdeology SES Education Gender Race Age MRN Finance_Tot Reso
urce_Tot Psychology_Tot
  /STATISTICS=STDDEV MINIMUM MAXIMUM MEAN
  /ORDER=ANALYSIS.
```

Frequencies

Notes

Output Created		15-DEC-2021 13:06:15
Comments		
Input	Data	C:\Users\njs5478\Dropbox\HM and COVID\0. Revise and Resubmit\2. R and R Data\Study 1a\Study1a_Data.sav
	Active Dataset	DataSet1
	Filter	Inclusion = 1 (FILTER)
	Weight	<none>
	Split File	<none>
	N of Rows in Working Data File	178
Missing Value Handling	Definition of Missing	User-defined missing values are treated as missing.
	Cases Used	Statistics are based on all cases with valid data.
Syntax		FREQUENCIES VARIABLES=PParty PIdeology SES Education Gender Race Age MRN Finance_Tot Resource_Tot Psychology_Tot /STATISTICS=STDDEV MINIMUM MAXIMUM MEAN...
Resources	Processor Time	00:00:00.02
	Elapsed Time	00:00:00.02

Statistics

		Which of the following best describes your political party affiliation?	Which of the following best describes your political ideology?	Self Reported Socioeconomic Status	Please indicate the highest level of education that you have received:	Gender - Selected Choice
N	Valid	178	178	178	178	178
	Missing	0	0	0	0	0
Mean		2.88	3.70	3.42	2.99	2.10
Std. Deviation		1.385	1.652	.835	.606	.998
Minimum		1	1	1	2	1
Maximum		5	7	5	6	3

Statistics

		Racial Identity - Selected Choice	Age	MRN	Finance_Tot	Resource_Tot	Psychology_Tot
N	Valid	178	177	178	178	178	178
	Missing	0	1	0	0	0	0
Mean		1.71	19.76	3.4304	3.9963	3.7584	4.3390
Std. Deviation		1.695	2.671	.90563	1.62490	1.48174	1.49386
Minimum		1	18	1.27	1.00	1.00	1.00
Maximum		8	36	5.88	7.00	7.00	7.00

Frequency Table

Which of the following best describes your political party affiliation?

		Frequency	Percent	Valid Percent	Cumulative Percent
Valid	Democrat	42	23.6	23.6	23.6
	Democrat Leaning	30	16.9	16.9	40.4
	Independent	40	22.5	22.5	62.9
	Republican Leaning	40	22.5	22.5	85.4
	Republican	26	14.6	14.6	100.0
	Total	178	100.0	100.0	

Which of the following best describes your political ideology?

		Frequency	Percent	Valid Percent	Cumulative Percent
Valid	Very Liberal	16	9.0	9.0	9.0
	Liberal	37	20.8	20.8	29.8
	Somewhat Liberal	27	15.2	15.2	44.9
	Neither Liberal Nor Conservative	37	20.8	20.8	65.7
	Somewhat Conservative	33	18.5	18.5	84.3
	Conservative	21	11.8	11.8	96.1
	Very Conservative	7	3.9	3.9	100.0
	Total	178	100.0	100.0	

Self Reported Socioeconomic Status

		Frequency	Percent	Valid Percent	Cumulative Percent
Valid	Poor	1	.6	.6	.6
	Working Class	24	13.5	13.5	14.0
	Middle Class	65	36.5	36.5	50.6
	Upper Middle Class	75	42.1	42.1	92.7
	Upper Class	13	7.3	7.3	100.0
	Total	178	100.0	100.0	

Please indicate the highest level of education that you have received:

		Frequency	Percent	Valid Percent	Cumulative Percent
Valid	Completed high school	22	12.4	12.4	12.4
	Some college	145	81.5	81.5	93.8
	Associate's Degree	2	1.1	1.1	94.9
	Bachelor's Degree	8	4.5	4.5	99.4
	Master's Degree	1	.6	.6	100.0
	Total	178	100.0	100.0	

Gender - Selected Choice

		Frequency	Percent	Valid Percent	Cumulative Percent
Valid	Biologically Born Male	80	44.9	44.9	44.9
	Biologically Born Female	98	55.1	55.1	100.0
	Total	178	100.0	100.0	

Racial Identity - Selected Choice

		Frequency	Percent	Valid Percent	Cumulative Percent
Valid	White/Caucasian	139	78.1	78.1	78.1
	Black/African American	11	6.2	6.2	84.3
	Asian	12	6.7	6.7	91.0
	Hispanic/Latino(a)	8	4.5	4.5	95.5
	Biracial	4	2.2	2.2	97.8
	Multiracial	4	2.2	2.2	100.0
	Total	178	100.0	100.0	

Age

		Frequency	Percent	Valid Percent	Cumulative Percent
Valid	18	40	22.5	22.6	22.6
	19	83	46.6	46.9	69.5
	20	23	12.9	13.0	82.5
	21	13	7.3	7.3	89.8
	22	6	3.4	3.4	93.2
	23	2	1.1	1.1	94.4
	24	3	1.7	1.7	96.0
	28	1	.6	.6	96.6
	29	2	1.1	1.1	97.7
	30	1	.6	.6	98.3
	33	2	1.1	1.1	99.4
	36	1	.6	.6	100.0
	Total	177	99.4	100.0	
Missing	System	1	.6		
Total		178	100.0		

MRN

		Frequency	Percent	Valid Percent	Cumulative Percent
Valid	1.27	1	.6	.6	.6
	1.38	1	.6	.6	1.1
	1.42	1	.6	.6	1.7
	1.58	1	.6	.6	2.2
	1.69	1	.6	.6	2.8
	1.77	1	.6	.6	3.4
	1.81	2	1.1	1.1	4.5
	1.92	1	.6	.6	5.1
	1.96	2	1.1	1.1	6.2
	2.00	2	1.1	1.1	7.3
	2.04	1	.6	.6	7.9
	2.12	1	.6	.6	8.4
	2.15	1	.6	.6	9.0
	2.23	1	.6	.6	9.6
	2.27	2	1.1	1.1	10.7
	2.31	1	.6	.6	11.2
	2.35	1	.6	.6	11.8
	2.38	4	2.2	2.2	14.0
	2.42	1	.6	.6	14.6
	2.46	4	2.2	2.2	16.9
	2.50	1	.6	.6	17.4
	2.54	1	.6	.6	18.0
	2.58	3	1.7	1.7	19.7
	2.62	1	.6	.6	20.2
	2.65	1	.6	.6	20.8
	2.69	2	1.1	1.1	21.9
	2.73	2	1.1	1.1	23.0
	2.77	2	1.1	1.1	24.2
	2.85	2	1.1	1.1	25.3
	2.88	2	1.1	1.1	26.4
	2.92	3	1.7	1.7	28.1
	2.96	3	1.7	1.7	29.8
	3.00	3	1.7	1.7	31.5
	3.04	2	1.1	1.1	32.6
	3.08	2	1.1	1.1	33.7

MRN

	Frequency	Percent	Valid Percent	Cumulative Percent
3.12	4	2.2	2.2	36.0
3.15	1	.6	.6	36.5
3.19	4	2.2	2.2	38.8
3.23	4	2.2	2.2	41.0
3.27	2	1.1	1.1	42.1
3.31	3	1.7	1.7	43.8
3.35	2	1.1	1.1	44.9
3.38	6	3.4	3.4	48.3
3.42	2	1.1	1.1	49.4
3.46	4	2.2	2.2	51.7
3.50	3	1.7	1.7	53.4
3.54	2	1.1	1.1	54.5
3.58	1	.6	.6	55.1
3.62	2	1.1	1.1	56.2
3.65	5	2.8	2.8	59.0
3.69	4	2.2	2.2	61.2
3.73	3	1.7	1.7	62.9
3.77	3	1.7	1.7	64.6
3.81	4	2.2	2.2	66.9
3.85	3	1.7	1.7	68.5
3.88	4	2.2	2.2	70.8
3.92	2	1.1	1.1	71.9
3.96	1	.6	.6	72.5
4.00	8	4.5	4.5	77.0
4.04	3	1.7	1.7	78.7
4.08	6	3.4	3.4	82.0
4.12	2	1.1	1.1	83.1
4.15	1	.6	.6	83.7
4.19	1	.6	.6	84.3
4.27	1	.6	.6	84.8
4.31	1	.6	.6	85.4
4.35	1	.6	.6	86.0
4.38	1	.6	.6	86.5
4.42	2	1.1	1.1	87.6
4.46	2	1.1	1.1	88.8

MRN

	Frequency	Percent	Valid Percent	Cumulative Percent
4.50	3	1.7	1.7	90.4
4.54	1	.6	.6	91.0
4.62	1	.6	.6	91.6
4.65	2	1.1	1.1	92.7
4.69	1	.6	.6	93.3
4.73	1	.6	.6	93.8
4.77	1	.6	.6	94.4
4.88	2	1.1	1.1	95.5
5.00	1	.6	.6	96.1
5.19	1	.6	.6	96.6
5.23	1	.6	.6	97.2
5.50	1	.6	.6	97.8
5.69	1	.6	.6	98.3
5.73	1	.6	.6	98.9
5.85	1	.6	.6	99.4
5.88	1	.6	.6	100.0
Total	178	100.0	100.0	

Finance_Tot

		Frequency	Percent	Valid Percent	Cumulative Percent
Valid	1.00	6	3.4	3.4	3.4
	1.33	6	3.4	3.4	6.7
	1.67	3	1.7	1.7	8.4
	2.00	16	9.0	9.0	17.4
	2.33	11	6.2	6.2	23.6
	2.67	9	5.1	5.1	28.7
	3.00	8	4.5	4.5	33.1
	3.33	8	4.5	4.5	37.6
	3.67	11	6.2	6.2	43.8
	4.00	18	10.1	10.1	53.9
	4.33	10	5.6	5.6	59.6
	4.67	12	6.7	6.7	66.3
	5.00	11	6.2	6.2	72.5
	5.33	16	9.0	9.0	81.5
	5.67	10	5.6	5.6	87.1
	6.00	6	3.4	3.4	90.4
	6.33	5	2.8	2.8	93.3
	6.67	1	.6	.6	93.8
	7.00	11	6.2	6.2	100.0
	Total	178	100.0	100.0	

Resource_Tot

		Frequency	Percent	Valid Percent	Cumulative Percent
Valid	1.00	11	6.2	6.2	6.2
	1.33	4	2.2	2.2	8.4
	1.67	5	2.8	2.8	11.2
	2.00	13	7.3	7.3	18.5
	2.33	8	4.5	4.5	23.0
	2.67	10	5.6	5.6	28.7
	3.00	10	5.6	5.6	34.3
	3.33	11	6.2	6.2	40.4
	3.67	16	9.0	9.0	49.4
	4.00	12	6.7	6.7	56.2
	4.33	18	10.1	10.1	66.3
	4.67	13	7.3	7.3	73.6
	5.00	15	8.4	8.4	82.0
	5.33	11	6.2	6.2	88.2
	5.67	9	5.1	5.1	93.3
	6.00	4	2.2	2.2	95.5
	6.33	5	2.8	2.8	98.3
	6.67	1	.6	.6	98.9
	7.00	2	1.1	1.1	100.0
	Total	178	100.0	100.0	

Psychology_Tot

		Frequency	Percent	Valid Percent	Cumulative Percent
Valid	1.00	4	2.2	2.2	2.2
	1.33	3	1.7	1.7	3.9
	1.67	3	1.7	1.7	5.6
	2.00	7	3.9	3.9	9.6
	2.33	5	2.8	2.8	12.4
	2.67	10	5.6	5.6	18.0
	3.00	11	6.2	6.2	24.2
	3.33	9	5.1	5.1	29.2
	3.67	8	4.5	4.5	33.7
	4.00	18	10.1	10.1	43.8
	4.33	11	6.2	6.2	50.0
	4.67	18	10.1	10.1	60.1
	5.00	16	9.0	9.0	69.1
	5.33	10	5.6	5.6	74.7
	5.67	14	7.9	7.9	82.6
	6.00	15	8.4	8.4	91.0
	6.33	5	2.8	2.8	93.8
	6.67	1	.6	.6	94.4
	7.00	10	5.6	5.6	100.0
	Total	178	100.0	100.0	

```
 **Frequencies for Personal Experience with COVID-19

FREQUENCIES VARIABLES=Covid1 Covid2 Covid3 Covid4 Covid5 Covid6 Covid7
  /ORDER=ANALYSIS.
```

Frequencies

Notes

Output Created		15-DEC-2021 13:06:15
Comments		
Input	Data	C:\Users\njs5478\Dropbox\HM and COVID\0. Revise and Resubmit\2. R and R Data\Study 1a\Study1a_Data.sav
	Active Dataset	DataSet1
	Filter	Inclusion = 1 (FILTER)
	Weight	<none>
	Split File	<none>
	N of Rows in Working Data File	178
Missing Value Handling	Definition of Missing	User-defined missing values are treated as missing.
	Cases Used	Statistics are based on all cases with valid data.
Syntax		FREQUENCIES VARIABLES=Covid1 Covid2 Covid3 Covid4 Covid5 Covid6 Covid7 /ORDER=ANALYSIS.
Resources	Processor Time	00:00:00.02
	Elapsed Time	00:00:00.02

Statistics

		I have been diagnosed with coronavirus (COVID-19).	I have had coronavirus-like symptoms at some point in the last two months.	I have been sick with something other than the coronavirus in the last two months.	I have been in close proximity with someone who has been diagnosed with coronavirus (COVID-19).	I have been in close proximity with someone who has had coronavirus-like symptoms in the last two months.
N	Valid	178	178	178	178	178
	Missing	0	0	0	0	0

Statistics

		I watch a lot of news about the Coronavirus (COVID-19).	I spent a huge percentage of my time trying to find updates online or on TV about Coronavirus (COVID-19).
N	Valid	178	178
	Missing	0	0

Frequency Table

I have been diagnosed with coronavirus (COVID-19).

		Frequency	Percent	Valid Percent	Cumulative Percent
Valid	No	178	100.0	100.0	100.0

I have had coronavirus-like symptoms at some point in the last two months.

		Frequency	Percent	Valid Percent	Cumulative Percent
Valid	Yes	25	14.0	14.0	14.0
	No	153	86.0	86.0	100.0
	Total	178	100.0	100.0	

I have been sick with something other than the coronavirus in the last two months.

		Frequency	Percent	Valid Percent	Cumulative Percent
Valid	Yes	55	30.9	30.9	30.9
	No	123	69.1	69.1	100.0
	Total	178	100.0	100.0	

I have been in close proximity with someone who has been diagnosed with coronavirus (COVID-19).

		Frequency	Percent	Valid Percent	Cumulative Percent
Valid	Yes	8	4.5	4.5	4.5
	No	170	95.5	95.5	100.0
	Total	178	100.0	100.0	

I have been in close proximity with someone who has had coronavirus-like symptoms in the last two months.

		Frequency	Percent	Valid Percent	Cumulative Percent
Valid	Yes	30	16.9	16.9	16.9
	No	148	83.1	83.1	100.0
	Total	178	100.0	100.0	

I watch a lot of news about the Coronavirus (COVID-19).

		Frequency	Percent	Valid Percent	Cumulative Percent
Valid	Yes	117	65.7	65.7	65.7
	No	61	34.3	34.3	100.0
	Total	178	100.0	100.0	

I spent a huge percentage of my time trying to find updates online or on TV about Coronavirus (COVID-19).

		Frequency	Percent	Valid Percent	Cumulative Percent
Valid	Yes	37	20.8	20.8	20.8
	No	141	79.2	79.2	100.0
	Total	178	100.0	100.0	

```
**Contrast Codes and Mean Centering**

IF (Gender=1) GenderCC=1.
IF (Gender=2) GenderCC=1.
IF(Gender=3) GenderCC=-1.
IF (Gender=4) GenderCC=-1.
```

```
IF (Race=1) RaceCC=1.
IF (Race>=2) RaceCC=-1.

**Mean Centering

DESCRIPTIVES VARIABLES=PParty PIdeology SES MRN
  /STATISTICS=MEAN STDDEV MIN MAX.
```

Descriptives

Notes

Output Created		15-DEC-2021 13:06:15
Comments		
Input	Data	C:\Users\njs5478\Dropbox\HM and COVID\0. Revise and Resubmit\2. R and R Data\Study 1a\Study1a_Data.sav
	Active Dataset	DataSet1
	Filter	Inclusion = 1 (FILTER)
	Weight	<none>
	Split File	<none>
	N of Rows in Working Data File	178
Missing Value Handling	Definition of Missing	User defined missing values are treated as missing.
	Cases Used	All non-missing data are used.
Syntax		DESCRIPTIVES VARIABLES=PParty PIdeology SES MRN /STATISTICS=MEAN STDDEV MIN MAX.
Resources	Processor Time	00:00:00.02
	Elapsed Time	00:00:00.02

Descriptive Statistics

	N	Minimum	Maximum	Mean	Std. Deviation
Which of the following best describes your political party affiliation?	178	1	5	2.88	1.385
Which of the following best describes your political ideology?	178	1	7	3.70	1.652
Self Reported Socioeconomic Status	178	1	5	3.42	.835
MRN	178	1.27	5.88	3.4304	.90563
Valid N (listwise)	178				

```
 COMPUTE Party0=PParty-2.88.
COMPUTE Ideology0=PIdeology-3.70.
COMPUTE SES0=SES-3.42.
COMPUTE MRN0=MRN-3.4304.

**Interactions**

COMPUTE MRN0xRace=MRN0 * RaceCC.
COMPUTE MRN0xSES0=MRN0 * SES0.
COMPUTE MRN0xGender=MRN0 * GenderCC.
COMPUTE MRN0xParty0=MRN0 * Party0.
COMPUTE MRN0xIdeology0=MRN0*Ideology0.

**Regression Analyses**

REGRESSION
  /MISSING LISTWISE
  /STATISTICS COEFF OUTS R ANOVA CHANGE ZPP
  /CRITERIA=PIN(.05) POUT(.10)
  /NOORIGIN
  /DEPENDENT Concern_Tot
  /METHOD=ENTER Party0
  /METHOD=ENTER GenderCC RaceCC SES0
  /METHOD=ENTER MRN0
  /METHOD=ENTER MRN0xRace MRN0xSES0 MRN0xGender MRN0xParty0.
```

Regression

Notes

Output Created		15-DEC-2021 13:06:15
Comments		
Input	Data	C:\Users\njs5478\Dropbox\HM and COVID\0. Revise and Resubmit\2. R and R Data\Study 1a\Study1a_Data.sav
	Active Dataset	DataSet1
	Filter	Inclusion = 1 (FILTER)
	Weight	<none>
	Split File	<none>
	N of Rows in Working Data File	178
Missing Value Handling	Definition of Missing	User-defined missing values are treated as missing.
	Cases Used	Statistics are based on cases with no missing values for any variable used.
Syntax		REGRESSION /MISSING LISTWISE /STATISTICS COEFF OUTS R ANOVA CHANGE ZPP /CRITERIA=PIN(.05) POUT(.10) /NOORIGIN /DEPENDENT Concern_Tot /METHOD=ENTER Party0 /METHOD=ENTER GenderCC RaceCC SES0 /METHOD=ENTER MRN0 /METHOD=ENTER MRN0xRace MRN0xSES0 MRN0xGender MRN0xParty0.
Resources	Processor Time	00:00:00.05
	Elapsed Time	00:00:00.02
	Memory Required	43600 bytes
	Additional Memory Required for Residual Plots	0 bytes

Variables Entered/Removed[a]

Model	Variables Entered	Variables Removed	Method
1	Party0[b]	.	Enter
2	SES0, RaceCC, GenderCC[b]	.	Enter
3	MRN0[b]	.	Enter
4	MRN0xParty0, MRN0xSES0, MRN0xGender, ...[b]	.	Enter

a. Dependent Variable: Concern_Tot

b. All requested variables entered.

Model Summary

Model	R	R Square	Adjusted R Square	Std. Error of the Estimate	Change Statistics				
					R Square Change	F Change	df1	df2	Sig. F Change
1	.271[a]	.074	.068	1.38816	.074	13.984	1	176	.000
2	.465[b]	.216	.198	1.28812	.142	10.466	3	173	.000
3	.467[c]	.218	.195	1.29005	.002	.485	1	172	.487
4	.475[d]	.225	.184	1.29941	.007	.383	4	168	.821

a. Predictors: (Constant), Party0

b. Predictors: (Constant), Party0, SES0, RaceCC, GenderCC

c. Predictors: (Constant), Party0, SES0, RaceCC, GenderCC, MRN0

d. Predictors: (Constant), Party0, SES0, RaceCC, GenderCC, MRN0, MRN0xParty0, MRN0xSES0, MRN0xGender, MRN0xRace

ANOVA[a]

Model		Sum of Squares	df	Mean Square	F	Sig.
1	Regression	26.948	1	26.948	13.984	.000[b]
	Residual	339.148	176	1.927		
	Total	366.096	177			
2	Regression	79.043	4	19.761	11.909	.000[c]
	Residual	287.053	173	1.659		
	Total	366.096	177			
3	Regression	79.850	5	15.970	9.596	.000[d]
	Residual	286.246	172	1.664		
	Total	366.096	177			
4	Regression	82.435	9	9.159	5.425	.000[e]
	Residual	283.660	168	1.688		
	Total	366.096	177			

a. Dependent Variable: Concern_Tot

b. Predictors: (Constant), Party0

c. Predictors: (Constant), Party0, SES0, RaceCC, GenderCC

d. Predictors: (Constant), Party0, SES0, RaceCC, GenderCC, MRN0

e. Predictors: (Constant), Party0, SES0, RaceCC, GenderCC, MRN0, MRN0xParty0, MRN0xSES0, MRN0xGender, MRN0xRace

Coefficients[a]

Model		Unstandardized Coefficients B	Unstandardized Coefficients Std. Error	Standardized Coefficients Beta	t	Sig.
1	(Constant)	4.721	.104		45.373	.000
	Party0	-.282	.075	-.271	-3.740	.000
2	(Constant)	4.660	.118		39.644	.000
	Party0	-.225	.073	-.217	-3.101	.002
	GenderCC	-.545	.099	-.378	-5.492	.000
	RaceCC	.010	.119	.006	.084	.933
	SES0	.183	.117	.106	1.558	.121
3	(Constant)	4.665	.118		39.552	.000
	Party0	-.196	.084	-.188	-2.323	.021
	GenderCC	-.512	.110	-.355	-4.648	.000
	RaceCC	.007	.119	.004	.059	.953
	SES0	.189	.118	.110	1.603	.111
	MRN0	-.098	.141	-.062	-.696	.487
4	(Constant)	4.706	.135		34.937	.000
	Party0	-.195	.085	-.188	-2.298	.023
	GenderCC	-.511	.112	-.354	-4.545	.000
	RaceCC	.010	.121	.005	.079	.937
	SES0	.203	.120	.118	1.690	.093
	MRN0	-.081	.168	-.051	-.478	.633
	MRN0xRace	-.043	.142	-.027	-.298	.766
	MRN0xSES0	.140	.140	.072	.996	.321
	MRN0xGender	-.073	.133	-.041	-.545	.586
	MRN0xParty0	-.039	.083	-.035	-.470	.639

Coefficients[a]

Model		Correlations		
		Zero-order	Partial	Part
1	(Constant)			
	Party0	-.271	-.271	-.271
2	(Constant)			
	Party0	-.271	-.229	-.209
	GenderCC	-.405	-.385	-.370
	RaceCC	-.022	.006	.006
	SES0	.039	.118	.105
3	(Constant)			
	Party0	-.271	-.174	-.157
	GenderCC	-.405	-.334	-.313
	RaceCC	-.022	.004	.004
	SES0	.039	.121	.108
	MRN0	-.308	-.053	-.047
4	(Constant)			
	Party0	-.271	-.175	-.156
	GenderCC	-.405	-.331	-.309
	RaceCC	-.022	.006	.005
	SES0	.039	.129	.115
	MRN0	-.308	-.037	-.032
	MRN0xRace	-.182	-.023	-.020
	MRN0xSES0	.038	.077	.068
	MRN0xGender	-.036	-.042	-.037
	MRN0xParty0	-.044	-.036	-.032

a. Dependent Variable: Concern_Tot

Excluded Variables[a]

Model		Beta In	t	Sig.	Partial Correlation	Collinearity Statistics Tolerance
1	GenderCC	-.368[b]	-5.373	.000	-.376	.968
	RaceCC	.027[b]	.368	.714	.028	.968
	SES0	.071[b]	.973	.332	.073	.987
	MRN0	-.229[b]	-2.724	.007	-.202	.720
	MRN0xRace	-.105[b]	-1.369	.173	-.103	.893
	MRN0xSES0	.033[b]	.454	.650	.034	1.000
	MRN0xGender	-.061[b]	-.835	.405	-.063	.992
	MRN0xParty0	-.035[b]	-.475	.635	-.036	.999
2	MRN0	-.062[c]	-.696	.487	-.053	.578
	MRN0xRace	-.037[c]	-.510	.611	-.039	.859
	MRN0xSES0	.052[c]	.769	.443	.059	.976
	MRN0xGender	-.034[c]	-.506	.613	-.039	.985
	MRN0xParty0	-.045[c]	-.667	.505	-.051	.996
3	MRN0xRace	-.014[d]	-.166	.868	-.013	.618
	MRN0xSES0	.054[d]	.797	.427	.061	.974
	MRN0xGender	-.040[d]	-.582	.561	-.044	.974
	MRN0xParty0	-.042[d]	-.621	.536	-.047	.992

a. Dependent Variable: Concern_Tot

b. Predictors in the Model: (Constant), Party0

c. Predictors in the Model: (Constant), Party0, SES0, RaceCC, GenderCC

d. Predictors in the Model: (Constant), Party0, SES0, RaceCC, GenderCC, MRN0

```
REGRESSION
 /MISSING LISTWISE
 /STATISTICS COEFF OUTS R ANOVA CHANGE ZPP
 /CRITERIA=PIN(.05) POUT(.10)
 /NOORIGIN
 /DEPENDENT Concern_Tot
 /METHOD=ENTER Ideology0
 /METHOD=ENTER GenderCC RaceCC SES0
 /METHOD=ENTER MRN0
 /METHOD=ENTER MRN0xRace MRN0xSES0 MRN0xGender MRN0xIdeology0.
```

Regression

Notes

Output Created		15-DEC-2021 13:06:15
Comments		
Input	Data	C:\Users\njs5478\Dropbox\HM and COVID\0. Revise and Resubmit\2. R and R Data\Study 1a\Study1a_Data.sav
	Active Dataset	DataSet1
	Filter	Inclusion = 1 (FILTER)
	Weight	<none>
	Split File	<none>
	N of Rows in Working Data File	178
Missing Value Handling	Definition of Missing	User-defined missing values are treated as missing.
	Cases Used	Statistics are based on cases with no missing values for any variable used.
Syntax		REGRESSION /MISSING LISTWISE /STATISTICS COEFF OUTS R ANOVA CHANGE ZPP /CRITERIA=PIN(.05) POUT(.10) /NOORIGIN /DEPENDENT Concern_Tot /METHOD=ENTER Ideology0 /METHOD=ENTER GenderCC RaceCC SES0 /METHOD=ENTER MRN0 /METHOD=ENTER MRN0xRace MRN0xSES0 MRN0xGender MRN0xIdeology0.
Resources	Processor Time	00:00:00.02
	Elapsed Time	00:00:00.02

Notes

	Memory Required	43600 bytes
	Additional Memory Required for Residual Plots	0 bytes

Variables Entered/Removed[a]

Model	Variables Entered	Variables Removed	Method
1	Ideology0[b]	.	Enter
2	SES0, RaceCC, GenderCC[b]	.	Enter
3	MRN0[b]	.	Enter
4	MRN0xIdeology0, MRN0xSES0, MRN0xGender, MRN0xRace[b]	.	Enter

a. Dependent Variable: Concern_Tot

b. All requested variables entered.

Model Summary

Model	R	R Square	Adjusted R Square	Std. Error of the Estimate	Change Statistics		
					R Square Change	F Change	df1
1	.318[a]	.101	.096	1.36721	.101	19.851	1
2	.469[b]	.220	.202	1.28457	.119	8.791	3
3	.470[c]	.221	.198	1.28802	.000	.073	1
4	.484[d]	.235	.194	1.29138	.014	.777	4

Model Summary

Model	Change Statistics	
	df2	Sig. F Change
1	176	.000
2	173	.000
3	172	.787
4	168	.542

a. Predictors: (Constant), Ideology0

b. Predictors: (Constant), Ideology0, SES0, RaceCC, GenderCC

c. Predictors: (Constant), Ideology0, SES0, RaceCC, GenderCC, MRN0

d. Predictors: (Constant), Ideology0, SES0, RaceCC, GenderCC, MRN0, MRN0xIdeology0, MRN0xSES0, MRN0xGender, MRN0xRace

ANOVA[a]

Model		Sum of Squares	df	Mean Square	F	Sig.
1	Regression	37.107	1	37.107	19.851	.000[b]
	Residual	328.989	176	1.869		
	Total	366.096	177			
2	Regression	80.627	4	20.157	12.215	.000[c]
	Residual	285.469	173	1.650		
	Total	366.096	177			
3	Regression	80.748	5	16.150	9.735	.000[d]
	Residual	285.347	172	1.659		
	Total	366.096	177			
4	Regression	85.930	9	9.548	5.725	.000[e]
	Residual	280.165	168	1.668		
	Total	366.096	177			

a. Dependent Variable: Concern_Tot

b. Predictors: (Constant), Ideology0

c. Predictors: (Constant), Ideology0, SES0, RaceCC, GenderCC

d. Predictors: (Constant), Ideology0, SES0, RaceCC, GenderCC, MRN0

e. Predictors: (Constant), Ideology0, SES0, RaceCC, GenderCC, MRN0, MRN0xIdeology0, MRN0xSES0, MRN0xGender, MRN0xRace

Coefficients[a]

Model		Unstandardized Coefficients B	Std. Error	Standardized Coefficients Beta	t	Sig.
1	(Constant)	4.723	.102		46.084	.000
	Ideology0	-.277	.062	-.318	-4.455	.000
2	(Constant)	4.667	.117		39.881	.000
	Ideology0	-.204	.062	-.234	-3.261	.001
	GenderCC	-.503	.102	-.349	-4.946	.000
	RaceCC	.007	.118	.004	.063	.950
	SES0	.197	.117	.114	1.674	.096
3	(Constant)	4.669	.118		39.717	.000
	Ideology0	-.191	.078	-.219	-2.440	.016
	GenderCC	-.492	.110	-.341	-4.493	.000
	RaceCC	.006	.119	.003	.050	.960
	SES0	.198	.118	.115	1.680	.095
	MRN0	-.041	.152	-.026	-.270	.787
4	(Constant)	4.728	.133		35.553	.000
	Ideology0	-.192	.079	-.221	-2.435	.016
	GenderCC	-.493	.111	-.342	-4.440	.000
	RaceCC	.030	.121	.017	.247	.806
	SES0	.217	.120	.126	1.813	.072
	MRN0	-.050	.173	-.031	-.288	.774
	MRN0xRace	.008	.143	.005	.055	.956
	MRN0xSES0	.153	.140	.078	1.094	.276
	MRN0xGender	-.006	.141	-.003	-.042	.967
	MRN0xIdeology0	-.091	.068	-.106	-1.347	.180

Coefficients[a]

Model		Correlations Zero-order	Partial	Part
1	(Constant)			
	Ideology0	-.318	-.318	-.318
2	(Constant)			
	Ideology0	-.318	-.241	-.219
	GenderCC	-.405	-.352	-.332
	RaceCC	-.022	.005	.004
	SES0	.039	.126	.112
3	(Constant)			
	Ideology0	-.318	-.183	-.164
	GenderCC	-.405	-.324	-.302
	RaceCC	-.022	.004	.003
	SES0	.039	.127	.113
	MRN0	-.308	-.021	-.018
4	(Constant)			
	Ideology0	-.318	-.185	-.164
	GenderCC	-.405	-.324	-.300
	RaceCC	-.022	.019	.017
	SES0	.039	.139	.122
	MRN0	-.308	-.022	-.019
	MRN0xRace	-.182	.004	.004
	MRN0xSES0	.038	.084	.074
	MRN0xGender	-.036	-.003	-.003
	MRN0xIdeology0	-.088	-.103	-.091

a. Dependent Variable: Concern_Tot

Excluded Variables[a]

Model		Beta In	t	Sig.	Partial Correlation	Collinearity Statistics Tolerance
1	GenderCC	-.341[b]	-4.842	.000	-.344	.914
	RaceCC	.029[b]	.405	.686	.031	.974
	SES0	.090[b]	1.248	.214	.094	.977
	MRN0	-.176[b]	-1.882	.062	-.141	.578
	MRN0xRace	-.051[b]	-.644	.520	-.049	.804
	MRN0xSES0	.036[b]	.506	.614	.038	1.000
	MRN0xGender	-.066[b]	-.920	.359	-.069	.991
	MRN0xIdeology0	-.086[b]	-1.202	.231	-.090	1.000
2	MRN0	-.026[c]	-.270	.787	-.021	.496
	MRN0xRace	-.007[c]	-.096	.923	-.007	.784
	MRN0xSES0	.056[c]	.826	.410	.063	.975
	MRN0xGender	-.038[c]	-.564	.573	-.043	.983
	MRN0xIdeology0	-.092[c]	-1.364	.174	-.103	.993
3	MRN0xRace	.003[d]	.034	.973	.003	.612
	MRN0xSES0	.057[d]	.832	.406	.064	.974
	MRN0xGender	-.040[d]	-.590	.556	-.045	.974
	MRN0xIdeology0	-.092[d]	-1.358	.176	-.103	.993

a. Dependent Variable: Concern_Tot

b. Predictors in the Model: (Constant), Ideology0

c. Predictors in the Model: (Constant), Ideology0, SES0, RaceCC, GenderCC

d. Predictors in the Model: (Constant), Ideology0, SES0, RaceCC, GenderCC, MRN0

```
REGRESSION
 /MISSING LISTWISE
 /STATISTICS COEFF OUTS R ANOVA CHANGE ZPP
 /CRITERIA=PIN(.05) POUT(.10)
 /NOORIGIN
 /DEPENDENT Finance_Tot
 /METHOD=ENTER Party0
 /METHOD=ENTER GenderCC RaceCC SES0
 /METHOD=ENTER MRN0
 /METHOD=ENTER MRN0xRace MRN0xSES0 MRN0xGender MRN0xParty0.
```

Regression

Notes

Output Created		15-DEC-2021 13:06:15
Comments		
Input	Data	C:\Users\njs5478\Dropbox\HM and COVID\0. Revise and Resubmit\2. R and R Data\Study 1a\Study1a_Data.sav
	Active Dataset	DataSet1
	Filter	Inclusion = 1 (FILTER)
	Weight	<none>
	Split File	<none>
	N of Rows in Working Data File	178
Missing Value Handling	Definition of Missing	User-defined missing values are treated as missing.
	Cases Used	Statistics are based on cases with no missing values for any variable used.
Syntax		REGRESSION /MISSING LISTWISE /STATISTICS COEFF OUTS R ANOVA CHANGE ZPP /CRITERIA=PIN(.05) POUT(.10) /NOORIGIN /DEPENDENT Finance_Tot /METHOD=ENTER Party0 /METHOD=ENTER GenderCC RaceCC SES0 /METHOD=ENTER MRN0 /METHOD=ENTER MRN0xRace MRN0xSES0 MRN0xGender MRN0xParty0.
Resources	Processor Time	00:00:00.05
	Elapsed Time	00:00:00.02

Notes

	Memory Required	43600 bytes
	Additional Memory Required for Residual Plots	0 bytes

Variables Entered/Removed[a]

Model	Variables Entered	Variables Removed	Method
1	Party0[b]	.	Enter
2	SES0, RaceCC, GenderCC[b]	.	Enter
3	MRN0[b]	.	Enter
4	MRN0xParty0 , MRN0xSES0, MRN0xGende r, ...[b]	.	Enter

a. Dependent Variable: Finance_Tot

b. All requested variables entered.

Model Summary

Model	R	R Square	Adjusted R Square	Std. Error of the Estimate	Change Statistics		
					R Square Change	F Change	df1
1	.045[a]	.002	-.004	1.62786	.002	.357	1
2	.368[b]	.135	.115	1.52847	.133	8.877	3
3	.368[c]	.136	.110	1.53258	.000	.074	1
4	.469[d]	.220	.178	1.47299	.084	4.550	4

Model Summary

Model	Change Statistics	
	df2	Sig. F Change
1	176	.551
2	173	.000
3	172	.786
4	168	.002

a. Predictors: (Constant), Party0

b. Predictors: (Constant), Party0, SES0, RaceCC, GenderCC

c. Predictors: (Constant), Party0, SES0, RaceCC, GenderCC, MRN0

d. Predictors: (Constant), Party0, SES0, RaceCC, GenderCC, MRN0, MRN0xParty0, MRN0xSES0, MRN0xGender, MRN0xRace

ANOVA[a]

Model		Sum of Squares	df	Mean Square	F	Sig.
1	Regression	.946	1	.946	.357	.551[b]
	Residual	466.385	176	2.650		
	Total	467.331	177			
2	Regression	63.163	4	15.791	6.759	.000[c]
	Residual	404.168	173	2.336		
	Total	467.331	177			
3	Regression	63.337	5	12.667	5.393	.000[d]
	Residual	403.994	172	2.349		
	Total	467.331	177			
4	Regression	102.822	9	11.425	5.266	.000[e]
	Residual	364.509	168	2.170		
	Total	467.331	177			

a. Dependent Variable: Finance_Tot

b. Predictors: (Constant), Party0

c. Predictors: (Constant), Party0, SES0, RaceCC, GenderCC

d. Predictors: (Constant), Party0, SES0, RaceCC, GenderCC, MRN0

e. Predictors: (Constant), Party0, SES0, RaceCC, GenderCC, MRN0, MRN0xParty0, MRN0xSES0, MRN0xGender, MRN0xRace

Coefficients[a]

Model		Unstandardized Coefficients B	Std. Error	Standardized Coefficients Beta	t	Sig.
1	(Constant)	3.996	.122		32.754	.000
	Party0	.053	.088	.045	.598	.551
2	(Constant)	3.766	.139		27.003	.000
	Party0	.057	.086	.049	.663	.508
	GenderCC	-.048	.118	-.030	-.411	.681
	RaceCC	.402	.141	.205	2.852	.005
	SES0	-.600	.139	-.308	-4.307	.000
3	(Constant)	3.769	.140		26.895	.000
	Party0	.071	.100	.060	.708	.480
	GenderCC	-.033	.131	-.020	-.253	.801
	RaceCC	.401	.141	.205	2.833	.005
	SES0	-.597	.140	-.307	-4.264	.000
	MRN0	-.046	.167	-.025	-.272	.786
4	(Constant)	3.852	.153		25.230	.000
	Party0	.070	.096	.060	.733	.465
	GenderCC	.001	.127	.000	.004	.996
	RaceCC	.414	.137	.211	3.023	.003
	SES0	-.521	.136	-.268	-3.828	.000
	MRN0	-.107	.191	-.059	-.558	.577
	MRN0xRace	-.010	.162	-.006	-.065	.948
	MRN0xSES0	.596	.159	.270	3.746	.000
	MRN0xGender	-.358	.151	-.177	-2.375	.019
	MRN0xParty0	-.009	.094	-.007	-.097	.923

Coefficients[a]

Model		Correlations Zero-order	Partial	Part
1	(Constant)			
	Party0	.045	.045	.045
2	(Constant)			
	Party0	.045	.050	.047
	GenderCC	-.058	-.031	-.029
	RaceCC	.197	.212	.202
	SES0	-.294	-.311	-.305
3	(Constant)			
	Party0	.045	.054	.050
	GenderCC	-.058	-.019	-.018
	RaceCC	.197	.211	.201
	SES0	-.294	-.309	-.302
	MRN0	-.038	-.021	-.019
4	(Constant)			
	Party0	.045	.056	.050
	GenderCC	-.058	.000	.000
	RaceCC	.197	.227	.206
	SES0	-.294	-.283	-.261
	MRN0	-.038	-.043	-.038
	MRN0xRace	.024	-.005	-.004
	MRN0xSES0	.259	.278	.255
	MRN0xGender	-.139	-.180	-.162
	MRN0xParty0	-.017	-.007	-.007

a. Dependent Variable: Finance_Tot

Excluded Variables[a]

Model		Beta In	t	Sig.	Partial Correlation	Collinearity Statistics Tolerance
1	GenderCC	-.069[b]	-.895	.372	-.068	.968
	RaceCC	.195[b]	2.594	.010	.192	.968
	SES0	-.304[b]	-4.189	.000	-.302	.987
	MRN0	-.086[b]	-.965	.336	-.073	.720
	MRN0xRace	.011[b]	.135	.893	.010	.893
	MRN0xSES0	.260[b]	3.561	.000	.260	1.000
	MRN0xGender	-.136[b]	-1.810	.072	-.136	.992
	MRN0xParty0	-.019[b]	-.250	.803	-.019	.999
2	MRN0	-.025[c]	-.272	.786	-.021	.578
	MRN0xRace	.036[c]	.474	.636	.036	.859
	MRN0xSES0	.234[c]	3.371	.001	.249	.976
	MRN0xGender	-.127[c]	-1.789	.075	-.135	.985
	MRN0xParty0	-.033[c]	-.461	.646	-.035	.996
3	MRN0xRace	.066[d]	.727	.468	.056	.618
	MRN0xSES0	.236[d]	3.377	.001	.250	.974
	MRN0xGender	-.130[d]	-1.824	.070	-.138	.974
	MRN0xParty0	-.032[d]	-.442	.659	-.034	.992

a. Dependent Variable: Finance_Tot

b. Predictors in the Model: (Constant), Party0

c. Predictors in the Model: (Constant), Party0, SES0, RaceCC, GenderCC

d. Predictors in the Model: (Constant), Party0, SES0, RaceCC, GenderCC, MRN0

```
REGRESSION
 /MISSING LISTWISE
 /STATISTICS COEFF OUTS R ANOVA CHANGE ZPP
 /CRITERIA=PIN(.05) POUT(.10)
 /NOORIGIN
 /DEPENDENT Finance_Tot
 /METHOD=ENTER Ideology0
 /METHOD=ENTER GenderCC RaceCC SES0
 /METHOD=ENTER MRN0
 /METHOD=ENTER MRN0xRace MRN0xSES0 MRN0xGender MRN0xIdeology0.
```

Regression

Notes

Output Created		15-DEC-2021 13:06:15
Comments		
Input	Data	C: \Users\njs5478\Dropbox\HM and COVID\0. Revise and Resubmit\2. R and R Data\Study 1a\Study1a_Data.sav
	Active Dataset	DataSet1
	Filter	Inclusion = 1 (FILTER)
	Weight	<none>
	Split File	<none>
	N of Rows in Working Data File	178
Missing Value Handling	Definition of Missing	User-defined missing values are treated as missing.
	Cases Used	Statistics are based on cases with no missing values for any variable used.
Syntax		REGRESSION /MISSING LISTWISE /STATISTICS COEFF OUTS R ANOVA CHANGE ZPP /CRITERIA=PIN(.05) POUT(.10) /NOORIGIN /DEPENDENT Finance_Tot /METHOD=ENTER Ideology0 /METHOD=ENTER GenderCC RaceCC SES0 /METHOD=ENTER MRN0 /METHOD=ENTER MRN0xRace MRN0xSES0 MRN0xGender MRN0xIdeology0.
Resources	Processor Time	00:00:00.02
	Elapsed Time	00:00:00.02

Notes

	Memory Required	43600 bytes
	Additional Memory Required for Residual Plots	0 bytes

Variables Entered/Removed[a]

Model	Variables Entered	Variables Removed	Method
1	Ideology0[b]	.	Enter
2	SES0, RaceCC, GenderCC[b]	.	Enter
3	MRN0[b]	.	Enter
4	MRN0xIdeology0, MRN0xSES0, MRN0xGender, MRN0xRace[b]	.	Enter

a. Dependent Variable: Finance_Tot

b. All requested variables entered.

Model Summary

Model	R	R Square	Adjusted R Square	Std. Error of the Estimate	Change Statistics		
					R Square Change	F Change	df1
1	.013[a]	.000	-.006	1.62937	.000	.030	1
2	.365[b]	.133	.113	1.53038	.133	8.835	3
3	.365[c]	.133	.108	1.53481	.000	.004	1
4	.480[d]	.231	.189	1.46290	.098	5.331	4

Model Summary

Model	Change Statistics	
	df2	Sig. F Change
1	176	.863
2	173	.000
3	172	.952
4	168	.000

a. Predictors: (Constant), Ideology0

b. Predictors: (Constant), Ideology0, SES0, RaceCC, GenderCC

c. Predictors: (Constant), Ideology0, SES0, RaceCC, GenderCC, MRN0

d. Predictors: (Constant), Ideology0, SES0, RaceCC, GenderCC, MRN0, MRN0xIdeology0, MRN0xSES0, MRN0xGender, MRN0xRace

ANOVA[a]

Model		Sum of Squares	df	Mean Square	F	Sig.
1	Regression	.080	1	.080	.030	.863[b]
	Residual	467.251	176	2.655		
	Total	467.331	177			
2	Regression	62.153	4	15.538	6.634	.000[c]
	Residual	405.178	173	2.342		
	Total	467.331	177			
3	Regression	62.162	5	12.432	5.278	.000[d]
	Residual	405.169	172	2.356		
	Total	467.331	177			
4	Regression	107.798	9	11.978	5.597	.000[e]
	Residual	359.533	168	2.140		
	Total	467.331	177			

a. Dependent Variable: Finance_Tot

b. Predictors: (Constant), Ideology0

c. Predictors: (Constant), Ideology0, SES0, RaceCC, GenderCC

d. Predictors: (Constant), Ideology0, SES0, RaceCC, GenderCC, MRN0

e. Predictors: (Constant), Ideology0, SES0, RaceCC, GenderCC, MRN0, MRN0xIdeology0, MRN0xSES0, MRN0xGender, MRN0xRace

Coefficients[a]

Model		Unstandardized Coefficients B	Std. Error	Standardized Coefficients Beta	t	Sig.
1	(Constant)	3.996	.122		32.723	.000
	Ideology0	-.013	.074	-.013	-.173	.863
2	(Constant)	3.759	.139		26.962	.000
	Ideology0	.006	.074	.006	.083	.934
	GenderCC	-.038	.121	-.023	-.311	.756
	RaceCC	.417	.141	.213	2.961	.003
	SES0	-.593	.140	-.305	-4.241	.000
3	(Constant)	3.758	.140		26.830	.000
	Ideology0	.003	.093	.003	.030	.976
	GenderCC	-.041	.131	-.025	-.311	.756
	RaceCC	.417	.141	.213	2.952	.004
	SES0	-.593	.140	-.305	-4.227	.000
	MRN0	.011	.181	.006	.061	.952
4	(Constant)	3.888	.151		25.804	.000
	Ideology0	-.004	.089	-.004	-.047	.962
	GenderCC	-.018	.126	-.011	-.142	.887
	RaceCC	.464	.137	.237	3.388	.001
	SES0	-.513	.135	-.264	-3.788	.000
	MRN0	-.057	.196	-.032	-.292	.770
	MRN0xRace	.037	.162	.021	.228	.820
	MRN0xSES0	.619	.159	.281	3.902	.000
	MRN0xGender	-.239	.159	-.118	-1.497	.136
	MRN0xIdeology0	-.130	.077	-.134	-1.696	.092

Coefficients[a]

Model		Correlations Zero-order	Partial	Part
1	(Constant)			
	Ideology0	-.013	-.013	-.013
2	(Constant)			
	Ideology0	-.013	.006	.006
	GenderCC	-.058	-.024	-.022
	RaceCC	.197	.220	.210
	SES0	-.294	-.307	-.300
3	(Constant)			
	Ideology0	-.013	.002	.002
	GenderCC	-.058	-.024	-.022
	RaceCC	.197	.220	.210
	SES0	-.294	-.307	-.300
	MRN0	-.038	.005	.004
4	(Constant)			
	Ideology0	-.013	-.004	-.003
	GenderCC	-.058	-.011	-.010
	RaceCC	.197	.253	.229
	SES0	-.294	-.281	-.256
	MRN0	-.038	-.023	-.020
	MRN0xRace	.024	.018	.015
	MRN0xSES0	.259	.288	.264
	MRN0xGender	-.139	-.115	-.101
	MRN0xIdeology0	-.111	-.130	-.115

a. Dependent Variable: Finance_Tot

Excluded Variables[a]

Model		Beta In	t	Sig.	Partial Correlation	Collinearity Statistics Tolerance
1	GenderCC	-.060[b]	-.755	.451	-.057	.914
	RaceCC	.204[b]	2.726	.007	.202	.974
	SES0	-.299[b]	-4.099	.000	-.296	.977
	MRN0	-.051[b]	-.511	.610	-.039	.578
	MRN0xRace	.038[b]	.445	.657	.034	.804
	MRN0xSES0	.259[b]	3.544	.001	.259	1.000
	MRN0xGender	-.141[b]	-1.880	.062	-.141	.991
	MRN0xIdeology0	-.110[b]	-1.470	.143	-.110	1.000
2	MRN0	.006[c]	.061	.952	.005	.496
	MRN0xRace	.055[c]	.684	.495	.052	.784
	MRN0xSES0	.235[c]	3.369	.001	.249	.975
	MRN0xGender	-.131[c]	-1.842	.067	-.139	.983
	MRN0xIdeology0	-.131[c]	-1.860	.065	-.140	.993
3	MRN0xRace	.067[d]	.739	.461	.056	.612
	MRN0xSES0	.235[d]	3.359	.001	.249	.974
	MRN0xGender	-.131[d]	-1.839	.068	-.139	.974
	MRN0xIdeology0	-.131[d]	-1.855	.065	-.140	.993

a. Dependent Variable: Finance_Tot

b. Predictors in the Model: (Constant), Ideology0

c. Predictors in the Model: (Constant), Ideology0, SES0, RaceCC, GenderCC

d. Predictors in the Model: (Constant), Ideology0, SES0, RaceCC, GenderCC, MRN0

```
REGRESSION
 /MISSING LISTWISE
 /STATISTICS COEFF OUTS R ANOVA CHANGE ZPP
 /CRITERIA=PIN(.05) POUT(.10)
 /NOORIGIN
 /DEPENDENT Resource_Tot
 /METHOD=ENTER Party0
 /METHOD=ENTER GenderCC RaceCC SES0
 /METHOD=ENTER MRN0
 /METHOD=ENTER MRN0xRace MRN0xSES0 MRN0xGender MRN0xParty0.
```

Regression

Notes

Output Created		15-DEC-2021 13:06:15
Comments		
Input	Data	C:\Users\njs5478\Dropbox\HM and COVID\0. Revise and Resubmit\2. R and R Data\Study 1a\Study1a_Data.sav
	Active Dataset	DataSet1
	Filter	Inclusion = 1 (FILTER)
	Weight	<none>
	Split File	<none>
	N of Rows in Working Data File	178
Missing Value Handling	Definition of Missing	User-defined missing values are treated as missing.
	Cases Used	Statistics are based on cases with no missing values for any variable used.
Syntax		REGRESSION /MISSING LISTWISE /STATISTICS COEFF OUTS R ANOVA CHANGE ZPP /CRITERIA=PIN(.05) POUT(.10) /NOORIGIN /DEPENDENT Resource_Tot /METHOD=ENTER Party0 /METHOD=ENTER GenderCC RaceCC SES0 /METHOD=ENTER MRN0 /METHOD=ENTER MRN0xRace MRN0xSES0 MRN0xGender MRN0xParty0.
Resources	Processor Time	00:00:00.05
	Elapsed Time	00:00:00.02

Notes

	Memory Required	43600 bytes
	Additional Memory Required for Residual Plots	0 bytes

Variables Entered/Removed[a]

Model	Variables Entered	Variables Removed	Method
1	Party0[b]	.	Enter
2	SES0, RaceCC, GenderCC[b]	.	Enter
3	MRN0[b]	.	Enter
4	MRN0xParty0 , MRN0xSES0, MRN0xGende r, ...[b]	.	Enter

a. Dependent Variable: Resource_Tot

b. All requested variables entered.

Model Summary

Model	R	R Square	Adjusted R Square	Std. Error of the Estimate	Change Statistics		
					R Square Change	F Change	df1
1	.013[a]	.000	-.006	1.48582	.000	.029	1
2	.085[b]	.007	-.016	1.49332	.007	.412	3
3	.138[c]	.019	-.009	1.48871	.012	2.073	1
4	.202[d]	.041	-.011	1.48954	.022	.953	4

Model Summary

Model	Change Statistics	
	df2	Sig. F Change
1	176	.865
2	173	.745
3	172	.152
4	168	.435

a. Predictors: (Constant), Party0

b. Predictors: (Constant), Party0, SES0, RaceCC, GenderCC

c. Predictors: (Constant), Party0, SES0, RaceCC, GenderCC, MRN0

d. Predictors: (Constant), Party0, SES0, RaceCC, GenderCC, MRN0, MRN0xParty0, MRN0xSES0, MRN0xGender, MRN0xRace

ANOVA[a]

Model		Sum of Squares	df	Mean Square	F	Sig.
1	Regression	.064	1	.064	.029	.865[b]
	Residual	388.549	176	2.208		
	Total	388.612	177			
2	Regression	2.820	4	.705	.316	.867[c]
	Residual	385.793	173	2.230		
	Total	388.612	177			
3	Regression	7.415	5	1.483	.669	.647[d]
	Residual	381.198	172	2.216		
	Total	388.612	177			
4	Regression	15.868	9	1.763	.795	.622[e]
	Residual	372.744	168	2.219		
	Total	388.612	177			

a. Dependent Variable: Resource_Tot

b. Predictors: (Constant), Party0

c. Predictors: (Constant), Party0, SES0, RaceCC, GenderCC

d. Predictors: (Constant), Party0, SES0, RaceCC, GenderCC, MRN0

e. Predictors: (Constant), Party0, SES0, RaceCC, GenderCC, MRN0, MRN0xParty0, MRN0xSES0, MRN0xGender, MRN0xRace

Coefficients[a]

Model		Unstandardized Coefficients B	Unstandardized Coefficients Std. Error	Standardized Coefficients Beta	t	Sig.
1	(Constant)	3.758	.111		33.748	.000
	Party0	-.014	.081	-.013	-.170	.865
2	(Constant)	3.793	.136		27.832	.000
	Party0	.008	.084	.007	.089	.929
	GenderCC	-.045	.115	-.030	-.388	.699
	RaceCC	-.069	.138	-.039	-.500	.618
	SES0	-.118	.136	-.066	-.864	.389
3	(Constant)	3.781	.136		27.775	.000
	Party0	-.063	.097	-.059	-.649	.517
	GenderCC	-.123	.127	-.083	-.972	.333
	RaceCC	-.062	.137	-.035	-.450	.653
	SES0	-.132	.136	-.074	-.972	.333
	MRN0	.234	.163	.143	1.440	.152
4	(Constant)	3.780	.154		24.482	.000
	Party0	-.059	.097	-.055	-.602	.548
	GenderCC	-.155	.129	-.104	-1.201	.232
	RaceCC	-.042	.138	-.024	-.307	.760
	SES0	-.122	.138	-.069	-.886	.377
	MRN0	.288	.193	.176	1.494	.137
	MRN0xRace	-.032	.163	-.019	-.193	.847
	MRN0xSES0	.139	.161	.069	.863	.389
	MRN0xGender	.172	.152	.094	1.131	.260
	MRN0xParty0	-.150	.095	-.130	-1.578	.116

Coefficients[a]

Model		Correlations Zero-order	Partial	Part
1	(Constant)			
	Party0	-.013	-.013	-.013
2	(Constant)			
	Party0	-.013	.007	.007
	GenderCC	-.036	-.029	-.029
	RaceCC	-.041	-.038	-.038
	SES0	-.071	-.066	-.065
3	(Constant)			
	Party0	-.013	-.049	-.049
	GenderCC	-.036	-.074	-.073
	RaceCC	-.041	-.034	-.034
	SES0	-.071	-.074	-.073
	MRN0	.061	.109	.109
4	(Constant)			
	Party0	-.013	-.046	-.046
	GenderCC	-.036	-.092	-.091
	RaceCC	-.041	-.024	-.023
	SES0	-.071	-.068	-.067
	MRN0	.061	.115	.113
	MRN0xRace	.033	-.015	-.015
	MRN0xSES0	.079	.066	.065
	MRN0xGender	.049	.087	.085
	MRN0xParty0	-.081	-.121	-.119

a. Dependent Variable: Resource_Tot

Excluded Variables[a]

Model		Beta In	t	Sig.	Partial Correlation	Collinearity Statistics Tolerance
1	GenderCC	-.035[b]	-.450	.653	-.034	.968
	RaceCC	-.040[b]	-.516	.606	-.039	.968
	SES0	-.070[b]	-.928	.354	-.070	.987
	MRN0	.093[b]	1.053	.294	.079	.720
	MRN0xRace	.041[b]	.517	.606	.039	.893
	MRN0xSES0	.079[b]	1.044	.298	.079	1.000
	MRN0xGender	.048[b]	.632	.528	.048	.992
	MRN0xParty0	-.081[b]	-1.071	.286	-.081	.999
2	MRN0	.143[c]	1.440	.152	.109	.578
	MRN0xRace	.044[c]	.540	.590	.041	.859
	MRN0xSES0	.069[c]	.897	.371	.068	.976
	MRN0xGender	.048[c]	.634	.527	.048	.985
	MRN0xParty0	-.082[c]	-1.085	.279	-.082	.996
3	MRN0xRace	-.025[d]	-.258	.797	-.020	.618
	MRN0xSES0	.065[d]	.843	.401	.064	.974
	MRN0xGender	.061[d]	.792	.430	.060	.974
	MRN0xParty0	-.090[d]	-1.189	.236	-.091	.992

a. Dependent Variable: Resource_Tot

b. Predictors in the Model: (Constant), Party0

c. Predictors in the Model: (Constant), Party0, SES0, RaceCC, GenderCC

d. Predictors in the Model: (Constant), Party0, SES0, RaceCC, GenderCC, MRN0

```
REGRESSION
 /MISSING LISTWISE
 /STATISTICS COEFF OUTS R ANOVA CHANGE ZPP
 /CRITERIA=PIN(.05) POUT(.10)
 /NOORIGIN
 /DEPENDENT Resource_Tot
 /METHOD=ENTER Ideology0
 /METHOD=ENTER GenderCC RaceCC SES0
 /METHOD=ENTER MRN0
 /METHOD=ENTER MRN0xRace MRN0xSES0 MRN0xGender MRN0xIdeology0.
```

Regression

Notes

Output Created		15-DEC-2021 13:06:15
Comments		
Input	Data	C: \Users\njs5478\Dropbox\HM and COVID\0. Revise and Resubmit\2. R and R Data\Study 1a\Study1a_Data.sav
	Active Dataset	DataSet1
	Filter	Inclusion = 1 (FILTER)
	Weight	<none>
	Split File	<none>
	N of Rows in Working Data File	178
Missing Value Handling	Definition of Missing	User-defined missing values are treated as missing.
	Cases Used	Statistics are based on cases with no missing values for any variable used.
Syntax		REGRESSION /MISSING LISTWISE /STATISTICS COEFF OUTS R ANOVA CHANGE ZPP /CRITERIA=PIN(.05) POUT(.10) /NOORIGIN /DEPENDENT Resource_Tot /METHOD=ENTER Ideology0 /METHOD=ENTER GenderCC RaceCC SES0 /METHOD=ENTER MRN0 /METHOD=ENTER MRN0xRace MRN0xSES0 MRN0xGender MRN0xIdeology0.
Resources	Processor Time	00:00:00.02
	Elapsed Time	00:00:00.02

Notes

	Memory Required	43600 bytes
	Additional Memory Required for Residual Plots	0 bytes

Variables Entered/Removed[a]

Model	Variables Entered	Variables Removed	Method
1	Ideology0[b]	.	Enter
2	SES0, RaceCC, GenderCC[b]	.	Enter
3	MRN0[b]	.	Enter
4	MRN0xIdeology0, MRN0xSES0, MRN0xGender, MRN0xRace[b]	.	Enter

a. Dependent Variable: Resource_Tot

b. All requested variables entered.

Model Summary

Model	R	R Square	Adjusted R Square	Std. Error of the Estimate	Change Statistics		
					R Square Change	F Change	df1
1	.072[a]	.005	.000	1.48210	.005	.913	1
2	.099[b]	.010	-.013	1.49143	.005	.269	3
3	.188[c]	.035	.007	1.47640	.025	4.540	1
4	.241[d]	.058	.008	1.47591	.023	1.028	4

Model Summary

Model	Change Statistics	
	df2	Sig. F Change
1	176	.341
2	173	.848
3	172	.035
4	168	.394

a. Predictors: (Constant), Ideology0

b. Predictors: (Constant), Ideology0, SES0, RaceCC, GenderCC

c. Predictors: (Constant), Ideology0, SES0, RaceCC, GenderCC, MRN0

d. Predictors: (Constant), Ideology0, SES0, RaceCC, GenderCC, MRN0, MRN0xIdeology0, MRN0xSES0, MRN0xGender, MRN0xRace

ANOVA[a]

Model		Sum of Squares	df	Mean Square	F	Sig.
1	Regression	2.006	1	2.006	.913	.341[b]
	Residual	386.606	176	2.197		
	Total	388.612	177			
2	Regression	3.800	4	.950	.427	.789[c]
	Residual	384.813	173	2.224		
	Total	388.612	177			
3	Regression	13.695	5	2.739	1.257	.285[d]
	Residual	374.918	172	2.180		
	Total	388.612	177			
4	Regression	22.656	9	2.517	1.156	.327[e]
	Residual	365.957	168	2.178		
	Total	388.612	177			

a. Dependent Variable: Resource_Tot

b. Predictors: (Constant), Ideology0

c. Predictors: (Constant), Ideology0, SES0, RaceCC, GenderCC

d. Predictors: (Constant), Ideology0, SES0, RaceCC, GenderCC, MRN0

e. Predictors: (Constant), Ideology0, SES0, RaceCC, GenderCC, MRN0, MRN0xIdeology0, MRN0xSES0, MRN0xGender, MRN0xRace

Coefficients[a]

Model		Unstandardized Coefficients B	Unstandardized Coefficients Std. Error	Standardized Coefficients Beta	t	Sig.
1	(Constant)	3.759	.111		33.834	.000
	Ideology0	-.064	.067	-.072	-.956	.341
2	(Constant)	3.786	.136		27.862	.000
	Ideology0	-.049	.073	-.054	-.670	.504
	GenderCC	-.020	.118	-.013	-.170	.865
	RaceCC	-.051	.137	-.029	-.375	.708
	SES0	-.106	.136	-.060	-.776	.439
3	(Constant)	3.768	.135		27.963	.000
	Ideology0	-.163	.090	-.182	-1.819	.071
	GenderCC	-.118	.126	-.079	-.938	.349
	RaceCC	-.037	.136	-.021	-.275	.784
	SES0	-.119	.135	-.067	-.881	.380
	MRN0	.371	.174	.227	2.131	.035
4	(Constant)	3.765	.152		24.767	.000
	Ideology0	-.161	.090	-.179	-1.781	.077
	GenderCC	-.142	.127	-.096	-1.120	.264
	RaceCC	.003	.138	.002	.020	.984
	SES0	-.102	.137	-.057	-.746	.457
	MRN0	.391	.198	.239	1.974	.050
	MRN0xRace	.004	.163	.002	.023	.982
	MRN0xSES0	.149	.160	.074	.929	.354
	MRN0xGender	.215	.161	.117	1.339	.182
	MRN0xIdeology0	-.132	.077	-.150	-1.709	.089

Coefficients[a]

Model		Correlations Zero-order	Partial	Part
1	(Constant)			
	Ideology0	-.072	-.072	-.072
2	(Constant)			
	Ideology0	-.072	-.051	-.051
	GenderCC	-.036	-.013	-.013
	RaceCC	-.041	-.029	-.028
	SES0	-.071	-.059	-.059
3	(Constant)			
	Ideology0	-.072	-.137	-.136
	GenderCC	-.036	-.071	-.070
	RaceCC	-.041	-.021	-.021
	SES0	-.071	-.067	-.066
	MRN0	.061	.160	.160
4	(Constant)			
	Ideology0	-.072	-.136	-.133
	GenderCC	-.036	-.086	-.084
	RaceCC	-.041	.002	.002
	SES0	-.071	-.057	-.056
	MRN0	.061	.151	.148
	MRN0xRace	.033	.002	.002
	MRN0xSES0	.079	.072	.070
	MRN0xGender	.049	.103	.100
	MRN0xIdeology0	-.080	-.131	-.128

a. Dependent Variable: Resource_Tot

Excluded Variables[a]

Model		Beta In	t	Sig.	Partial Correlation	Collinearity Statistics Tolerance
1	GenderCC	-.016[b]	-.203	.839	-.015	.914
	RaceCC	-.030[b]	-.392	.696	-.030	.974
	SES0	-.061[b]	-.807	.421	-.061	.977
	MRN0	.185[b]	1.889	.061	.141	.578
	MRN0xRace	.080[b]	.958	.339	.072	.804
	MRN0xSES0	.079[b]	1.045	.298	.079	1.000
	MRN0xGender	.042[b]	.561	.576	.042	.991
	MRN0xIdeology0	-.079[b]	-1.051	.295	-.079	1.000
2	MRN0	.227[c]	2.131	.035	.160	.496
	MRN0xRace	.078[c]	.907	.366	.069	.784
	MRN0xSES0	.070[c]	.913	.362	.069	.975
	MRN0xGender	.043[c]	.558	.578	.042	.983
	MRN0xIdeology0	-.078[c]	-1.024	.307	-.078	.993
3	MRN0xRace	-.009[d]	-.091	.928	-.007	.612
	MRN0xSES0	.065[d]	.858	.392	.065	.974
	MRN0xGender	.058[d]	.765	.445	.058	.974
	MRN0xIdeology0	-.079[d]	-1.051	.295	-.080	.993

a. Dependent Variable: Resource_Tot

b. Predictors in the Model: (Constant), Ideology0

c. Predictors in the Model: (Constant), Ideology0, SES0, RaceCC, GenderCC

d. Predictors in the Model: (Constant), Ideology0, SES0, RaceCC, GenderCC, MRN0

```
REGRESSION
 /MISSING LISTWISE
 /STATISTICS COEFF OUTS R ANOVA CHANGE ZPP
 /CRITERIA=PIN(.05) POUT(.10)
 /NOORIGIN
 /DEPENDENT Psychology_Tot
 /METHOD=ENTER Party0
 /METHOD=ENTER GenderCC RaceCC SES0
 /METHOD=ENTER MRN0
 /METHOD=ENTER MRN0xRace MRN0xSES0 MRN0xGender MRN0xParty0.
```

Regression

Notes

Output Created		15-DEC-2021 13:06:15
Comments		
Input	Data	C: \Users\njs5478\Dropbox\HM and COVID\0. Revise and Resubmit\2. R and R Data\Study 1a\Study1a_Data.sav
	Active Dataset	DataSet1
	Filter	Inclusion = 1 (FILTER)
	Weight	<none>
	Split File	<none>
	N of Rows in Working Data File	178
Missing Value Handling	Definition of Missing	User-defined missing values are treated as missing.
	Cases Used	Statistics are based on cases with no missing values for any variable used.
Syntax		REGRESSION<br>/MISSING LISTWISE<br>/STATISTICS COEFF OUTS R ANOVA CHANGE ZPP<br>/CRITERIA=PIN(.05) POUT(.10)<br>/NOORIGIN<br>/DEPENDENT Psychology_Tot<br>/METHOD=ENTER Party0<br>/METHOD=ENTER GenderCC RaceCC SES0<br>/METHOD=ENTER MRN0<br>/METHOD=ENTER MRN0xRace MRN0xSES0 MRN0xGender MRN0xParty0.
Resources	Processor Time	00:00:00.03
	Elapsed Time	00:00:00.03

Notes

	Memory Required	43600 bytes
	Additional Memory Required for Residual Plots	0 bytes

Variables Entered/Removed[a]

Model	Variables Entered	Variables Removed	Method
1	Party0[b]	.	Enter
2	SES0, RaceCC, GenderCC[b]	.	Enter
3	MRN0[b]	.	Enter
4	MRN0xParty0 , MRN0xSES0, MRN0xGende r, [b] ...	.	Enter

a. Dependent Variable: Psychology_Tot

b. All requested variables entered.

Model Summary

					Change Statistics		
Model	R	R Square	Adjusted R Square	Std. Error of the Estimate	R Square Change	F Change	df1
1	.144[a]	.021	.015	1.48258	.021	3.702	1
2	.297[b]	.088	.067	1.44264	.068	4.293	3
3	.364[c]	.133	.107	1.41130	.044	8.769	1
4	.414[d]	.171	.127	1.39609	.038	1.942	4

Model Summary

	Change Statistics	
Model	df2	Sig. F Change
1	176	.056
2	173	.006
3	172	.003
4	168	.106

a. Predictors: (Constant), Party0

b. Predictors: (Constant), Party0, SES0, RaceCC, GenderCC

c. Predictors: (Constant), Party0, SES0, RaceCC, GenderCC, MRN0

d. Predictors: (Constant), Party0, SES0, RaceCC, GenderCC, MRN0, MRN0xParty0, MRN0xSES0, MRN0xGender, MRN0xRace

ANOVA[a]

Model		Sum of Squares	df	Mean Square	F	Sig.
1	Regression	8.137	1	8.137	3.702	.056[b]
	Residual	386.857	176	2.198		
	Total	394.994	177			
2	Regression	34.944	4	8.736	4.198	.003[c]
	Residual	360.051	173	2.081		
	Total	394.994	177			
3	Regression	52.409	5	10.482	5.263	.000[d]
	Residual	342.585	172	1.992		
	Total	394.994	177			
4	Regression	67.551	9	7.506	3.851	.000[e]
	Residual	327.444	168	1.949		
	Total	394.994	177			

a. Dependent Variable: Psychology_Tot

b. Predictors: (Constant), Party0

c. Predictors: (Constant), Party0, SES0, RaceCC, GenderCC

d. Predictors: (Constant), Party0, SES0, RaceCC, GenderCC, MRN0

e. Predictors: (Constant), Party0, SES0, RaceCC, GenderCC, MRN0, MRN0xParty0, MRN0xSES0, MRN0xGender, MRN0xRace

Coefficients[a]

Model		Unstandardized Coefficients B	Std. Error	Standardized Coefficients Beta	t	Sig.
1	(Constant)	4.338	.111		39.041	.000
	Party0	-.155	.080	-.144	-1.924	.056
2	(Constant)	4.222	.132		32.066	.000
	Party0	-.127	.081	-.118	-1.565	.119
	GenderCC	-.373	.111	-.249	-3.357	.001
	RaceCC	.141	.133	.078	1.058	.292
	SES0	.079	.131	.044	.602	.548
3	(Constant)	4.245	.129		32.899	.000
	Party0	.010	.092	.010	.112	.911
	GenderCC	-.219	.120	-.146	-1.819	.071
	RaceCC	.127	.130	.071	.976	.331
	SES0	.107	.129	.060	.833	.406
	MRN0	-.456	.154	-.277	-2.961	.003
4	(Constant)	4.152	.145		28.690	.000
	Party0	.017	.091	.016	.186	.853
	GenderCC	-.238	.121	-.159	-1.972	.050
	RaceCC	.107	.130	.059	.822	.412
	SES0	.113	.129	.063	.873	.384
	MRN0	-.247	.181	-.150	-1.367	.174
	MRN0xRace	-.348	.153	-.211	-2.276	.024
	MRN0xSES0	.162	.151	.080	1.074	.284
	MRN0xGender	.050	.143	.027	.348	.728
	MRN0xParty0	.119	.089	.101	1.328	.186

Coefficients[a]

Model		Correlations Zero-order	Partial	Part
1	(Constant)			
	Party0	-.144	-.144	-.144
2	(Constant)			
	Party0	-.144	-.118	-.114
	GenderCC	-.266	-.247	-.244
	RaceCC	.063	.080	.077
	SES0	.007	.046	.044
3	(Constant)			
	Party0	-.144	.009	.008
	GenderCC	-.266	-.137	-.129
	RaceCC	.063	.074	.069
	SES0	.007	.063	.059
	MRN0	-.326	-.220	-.210
4	(Constant)			
	Party0	-.144	.014	.013
	GenderCC	-.266	-.150	-.139
	RaceCC	.063	.063	.058
	SES0	.007	.067	.061
	MRN0	-.326	-.105	-.096
	MRN0xRace	-.301	-.173	-.160
	MRN0xSES0	.042	.083	.075
	MRN0xGender	.088	.027	.024
	MRN0xParty0	.085	.102	.093

a. Dependent Variable: Psychology_Tot

Excluded Variables[a]

Model		Beta In	t	Sig.	Partial Correlation	Collinearity Statistics Tolerance
1	GenderCC	-.249[b]	-3.373	.001	-.247	.968
	RaceCC	.091[b]	1.207	.229	.091	.968
	SES0	.024[b]	.316	.752	.024	.987
	MRN0	-.348[b]	-4.130	.000	-.298	.720
	MRN0xRace	-.285[b]	-3.738	.000	-.272	.893
	MRN0xSES0	.039[b]	.524	.601	.040	1.000
	MRN0xGender	.075[b]	1.008	.315	.076	.992
	MRN0xParty0	.090[b]	1.209	.228	.091	.999
2	MRN0	-.277[c]	-2.961	.003	-.220	.578
	MRN0xRace	-.241[c]	-3.157	.002	-.234	.859
	MRN0xSES0	.053[c]	.725	.470	.055	.976
	MRN0xGender	.097[c]	1.328	.186	.101	.985
	MRN0xParty0	.081[c]	1.112	.268	.084	.996
3	MRN0xRace	-.168[d]	-1.875	.063	-.142	.618
	MRN0xSES0	.062[d]	.861	.391	.066	.974
	MRN0xGender	.075[d]	1.049	.296	.080	.974
	MRN0xParty0	.095[d]	1.342	.181	.102	.992

a. Dependent Variable: Psychology_Tot

b. Predictors in the Model: (Constant), Party0

c. Predictors in the Model: (Constant), Party0, SES0, RaceCC, GenderCC

d. Predictors in the Model: (Constant), Party0, SES0, RaceCC, GenderCC, MRN0

```
REGRESSION
 /MISSING LISTWISE
 /STATISTICS COEFF OUTS R ANOVA CHANGE ZPP
 /CRITERIA=PIN(.05) POUT(.10)
 /NOORIGIN
 /DEPENDENT Psychology_Tot
 /METHOD=ENTER Ideology0
 /METHOD=ENTER GenderCC RaceCC SES0
 /METHOD=ENTER MRN0
 /METHOD=ENTER MRN0xRace MRN0xSES0 MRN0xGender MRN0xIdeology0.
```

Regression

Notes

Output Created		15-DEC-2021 13:06:15
Comments		
Input	Data	C:\Users\njs5478\Dropbox\HM and COVID\0. Revise and Resubmit\2. R and R Data\Study 1a\Study1a_Data.sav
	Active Dataset	DataSet1
	Filter	Inclusion = 1 (FILTER)
	Weight	<none>
	Split File	<none>
	N of Rows in Working Data File	178
Missing Value Handling	Definition of Missing	User-defined missing values are treated as missing.
	Cases Used	Statistics are based on cases with no missing values for any variable used.
Syntax		REGRESSION /MISSING LISTWISE /STATISTICS COEFF OUTS R ANOVA CHANGE ZPP /CRITERIA=PIN(.05) POUT(.10) /NOORIGIN /DEPENDENT Psychology_Tot /METHOD=ENTER Ideology0 /METHOD=ENTER GenderCC RaceCC SES0 /METHOD=ENTER MRN0 /METHOD=ENTER MRN0xRace MRN0xSES0 MRN0xGender MRN0xIdeology0.
Resources	Processor Time	00:00:00.02
	Elapsed Time	00:00:00.02

Notes

	Memory Required	43600 bytes
	Additional Memory Required for Residual Plots	0 bytes

Variables Entered/Removed[a]

Model	Variables Entered	Variables Removed	Method
1	Ideology0[b]	.	Enter
2	SES0, RaceCC, GenderCC[b]	.	Enter
3	MRN0[b]	.	Enter
4	MRN0xIdeology0, MRN0xSES0, MRN0xGender, MRN0xRace[b]	.	Enter

a. Dependent Variable: Psychology_Tot

b. All requested variables entered.

Model Summary

Model	R	R Square	Adjusted R Square	Std. Error of the Estimate	Change Statistics R Square Change	F Change	df1
1	.218[a]	.048	.042	1.46197	.048	8.806	1
2	.320[b]	.103	.082	1.43146	.055	3.527	3
3	.365[c]	.133	.108	1.41079	.031	6.105	1
4	.418[d]	.175	.130	1.39309	.041	2.100	4

Model Summary

Model	Change Statistics df2	Sig. F Change
1	176	.003
2	173	.016
3	172	.014
4	168	.083

a. Predictors: (Constant), Ideology0

b. Predictors: (Constant), Ideology0, SES0, RaceCC, GenderCC

c. Predictors: (Constant), Ideology0, SES0, RaceCC, GenderCC, MRN0

d. Predictors: (Constant), Ideology0, SES0, RaceCC, GenderCC, MRN0, MRN0xIdeology0, MRN0xSES0, MRN0xGender, MRN0xRace

ANOVA[a]

Model		Sum of Squares	df	Mean Square	F	Sig.
1	Regression	18.822	1	18.822	8.806	.003[b]
	Residual	376.172	176	2.137		
	Total	394.994	177			
2	Regression	40.505	4	10.126	4.942	.001[c]
	Residual	354.490	173	2.049		
	Total	394.994	177			
3	Regression	52.656	5	10.531	5.291	.000[d]
	Residual	342.338	172	1.990		
	Total	394.994	177			
4	Regression	68.957	9	7.662	3.948	.000[e]
	Residual	326.037	168	1.941		
	Total	394.994	177			

a. Dependent Variable: Psychology_Tot

b. Predictors: (Constant), Ideology0

c. Predictors: (Constant), Ideology0, SES0, RaceCC, GenderCC

d. Predictors: (Constant), Ideology0, SES0, RaceCC, GenderCC, MRN0

e. Predictors: (Constant), Ideology0, SES0, RaceCC, GenderCC, MRN0, MRN0xIdeology0, MRN0xSES0, MRN0xGender, MRN0xRace

Coefficients[a]

Model		Unstandardized Coefficients B	Std. Error	Standardized Coefficients Beta	t	Sig.
1	(Constant)	4.339	.110		39.601	.000
	Ideology0	-.197	.067	-.218	-2.968	.003
2	(Constant)	4.220	.130		32.360	.000
	Ideology0	-.159	.070	-.176	-2.281	.024
	GenderCC	-.329	.113	-.220	-2.902	.004
	RaceCC	.153	.132	.085	1.162	.247
	SES0	.097	.131	.054	.738	.462
3	(Constant)	4.239	.129		32.924	.000
	Ideology0	-.032	.086	-.035	-.370	.712
	GenderCC	-.220	.120	-.147	-1.837	.068
	RaceCC	.137	.130	.076	1.057	.292
	SES0	.111	.129	.062	.861	.390
	MRN0	-.411	.166	-.249	-2.471	.014
4	(Constant)	4.146	.143		28.899	.000
	Ideology0	-.010	.085	-.011	-.120	.905
	GenderCC	-.243	.120	-.162	-2.023	.045
	RaceCC	.096	.130	.053	.733	.464
	SES0	.110	.129	.061	.849	.397
	MRN0	-.209	.187	-.126	-1.116	.266
	MRN0xRace	-.357	.154	-.216	-2.315	.022
	MRN0xSES0	.148	.151	.073	.982	.328
	MRN0xGender	.000	.152	.000	.001	.999
	MRN0xIdeology0	.116	.073	.130	1.586	.115

Coefficients[a]

Model		Correlations		
		Zero-order	Partial	Part
1	(Constant)			
	Ideology0	-.218	-.218	-.218
2	(Constant)			
	Ideology0	-.218	-.171	-.164
	GenderCC	-.266	-.215	-.209
	RaceCC	.063	.088	.084
	SES0	.007	.056	.053
3	(Constant)			
	Ideology0	-.218	-.028	-.026
	GenderCC	-.266	-.139	-.130
	RaceCC	.063	.080	.075
	SES0	.007	.066	.061
	MRN0	-.326	-.185	-.175
4	(Constant)			
	Ideology0	-.218	-.009	-.008
	GenderCC	-.266	-.154	-.142
	RaceCC	.063	.056	.051
	SES0	.007	.065	.060
	MRN0	-.326	-.086	-.078
	MRN0xRace	-.301	-.176	-.162
	MRN0xSES0	.042	.076	.069
	MRN0xGender	.088	.000	.000
	MRN0xIdeology0	.122	.121	.111

a. Dependent Variable: Psychology_Tot

Excluded Variables[a]

Model		Beta In	t	Sig.	Partial Correlation	Collinearity Statistics Tolerance
1	GenderCC	-.221[b]	-2.936	.004	-.217	.914
	RaceCC	.100[b]	1.348	.179	.101	.974
	SES0	.041[b]	.556	.579	.042	.977
	MRN0	-.319[b]	-3.394	.001	-.249	.578
	MRN0xRace	-.254[b]	-3.181	.002	-.234	.804
	MRN0xSES0	.041[b]	.551	.582	.042	1.000
	MRN0xGender	.068[b]	.922	.358	.070	.991
	MRN0xIdeology0	.123[b]	1.683	.094	.126	1.000
2	MRN0	-.249[c]	-2.471	.014	-.185	.496
	MRN0xRace	-.222[c]	-2.788	.006	-.208	.784
	MRN0xSES0	.056[c]	.772	.441	.059	.975
	MRN0xGender	.090[c]	1.246	.215	.095	.983
	MRN0xIdeology0	.114[c]	1.581	.116	.120	.993
3	MRN0xRace	-.166[d]	-1.845	.067	-.140	.612
	MRN0xSES0	.062[d]	.859	.392	.066	.974
	MRN0xGender	.075[d]	1.036	.301	.079	.974
	MRN0xIdeology0	.115[d]	1.623	.106	.123	.993

a. Dependent Variable: Psychology_Tot

b. Predictors in the Model: (Constant), Ideology0

c. Predictors in the Model: (Constant), Ideology0, SES0, RaceCC, GenderCC

d. Predictors in the Model: (Constant), Ideology0, SES0, RaceCC, GenderCC, MRN0

```
REGRESSION
 /MISSING LISTWISE
 /STATISTICS COEFF OUTS R ANOVA CHANGE ZPP
 /CRITERIA=PIN(.05) POUT(.10)
 /NOORIGIN
 /DEPENDENT TrumpApproval
 /METHOD=ENTER Party0
 /METHOD=ENTER GenderCC RaceCC SES0
 /METHOD=ENTER MRN0
 /METHOD=ENTER MRN0xRace MRN0xSES0 MRN0xGender MRN0xParty0.
```

Regression

Notes

Output Created		15-DEC-2021 13:06:15
Comments		
Input	Data	C: \Users\njs5478\Dropbox\HM and COVID\0. Revise and Resubmit\2. R and R Data\Study 1a\Study1a_Data.sav
	Active Dataset	DataSet1
	Filter	Inclusion = 1 (FILTER)
	Weight	<none>
	Split File	<none>
	N of Rows in Working Data File	178
Missing Value Handling	Definition of Missing	User-defined missing values are treated as missing.
	Cases Used	Statistics are based on cases with no missing values for any variable used.
Syntax		REGRESSION<br>/MISSING LISTWISE<br>/STATISTICS COEFF OUTS R ANOVA CHANGE ZPP<br>/CRITERIA=PIN(.05) POUT(.10)<br>/NOORIGIN<br>/DEPENDENT TrumpApproval<br>/METHOD=ENTER Party0<br>/METHOD=ENTER GenderCC RaceCC SES0<br>/METHOD=ENTER MRN0<br>/METHOD=ENTER MRN0xRace MRN0xSES0 MRN0xGender MRN0xParty0.
Resources	Processor Time	00:00:00.02
	Elapsed Time	00:00:00.02

Notes

	Memory Required	43600 bytes
	Additional Memory Required for Residual Plots	0 bytes

Variables Entered/Removed[a]

Model	Variables Entered	Variables Removed	Method
1	Party0[b]	.	Enter
2	SES0, RaceCC, GenderCC[b]	.	Enter
3	MRN0[b]	.	Enter
4	MRN0xParty0 , MRN0xSES0, MRN0xGende r, ...[b]	.	Enter

a. Dependent Variable: Do you approve or disapprove of the way Donald Trump is handling his job as President?

b. All requested variables entered.

Model Summary

Model	R	R Square	Adjusted R Square	Std. Error of the Estimate	Change Statistics		
					R Square Change	F Change	df1
1	.769[a]	.591	.589	1.358	.591	254.531	1
2	.779[b]	.607	.598	1.343	.016	2.345	3
3	.789[c]	.622	.611	1.321	.015	6.805	1
4	.794[d]	.630	.610	1.322	.008	.921	4

Model Summary

Model	Change Statistics df2	Sig. F Change
1	176	.000
2	173	.075
3	172	.010
4	168	.453

a. Predictors: (Constant), Party0

b. Predictors: (Constant), Party0, SES0, RaceCC, GenderCC

c. Predictors: (Constant), Party0, SES0, RaceCC, GenderCC, MRN0

d. Predictors: (Constant), Party0, SES0, RaceCC, GenderCC, MRN0, MRN0xParty0, MRN0xSES0, MRN0xGender, MRN0xRace

ANOVA[a]

Model		Sum of Squares	df	Mean Square	F	Sig.
1	Regression	469.548	1	469.548	254.531	.000[b]
	Residual	324.677	176	1.845		
	Total	794.225	177			
2	Regression	482.237	4	120.559	66.851	.000[c]
	Residual	311.987	173	1.803		
	Total	794.225	177			
3	Regression	494.112	5	98.822	56.637	.000[d]
	Residual	300.113	172	1.745		
	Total	794.225	177			
4	Regression	500.553	9	55.617	31.817	.000[e]
	Residual	293.672	168	1.748		
	Total	794.225	177			

a. Dependent Variable: Do you approve or disapprove of the way Donald Trump is handling his job as President?

b. Predictors: (Constant), Party0

c. Predictors: (Constant), Party0, SES0, RaceCC, GenderCC

d. Predictors: (Constant), Party0, SES0, RaceCC, GenderCC, MRN0

e. Predictors: (Constant), Party0, SES0, RaceCC, GenderCC, MRN0, MRN0xParty0, MRN0xSES0, MRN0xGender, MRN0xRace

Coefficients[a]

Model		Unstandardized Coefficients B	Unstandardized Coefficients Std. Error	Standardized Coefficients Beta	t	Sig.
1	(Constant)	3.465	.102		34.035	.000
	Party0	1.176	.074	.769	15.954	.000
2	(Constant)	3.304	.123		26.964	.000
	Party0	1.128	.076	.737	14.906	.000
	GenderCC	.080	.103	.037	.770	.443
	RaceCC	.299	.124	.117	2.416	.017
	SES0	.088	.122	.035	.717	.474
3	(Constant)	3.285	.121		27.199	.000
	Party0	1.015	.086	.663	11.768	.000
	GenderCC	-.047	.113	-.022	-.419	.676
	RaceCC	.311	.122	.122	2.547	.012
	SES0	.064	.121	.025	.533	.594
	MRN0	.376	.144	.161	2.609	.010
4	(Constant)	3.217	.137		23.473	.000
	Party0	1.019	.086	.666	11.801	.000
	GenderCC	-.077	.114	-.036	-.669	.504
	RaceCC	.306	.123	.120	2.492	.014
	SES0	.047	.122	.019	.386	.700
	MRN0	.493	.171	.211	2.880	.004
	MRN0xRace	-.139	.145	-.059	-.956	.340
	MRN0xSES0	-.070	.143	-.024	-.489	.625
	MRN0xGender	.201	.135	.076	1.483	.140
	MRN0xParty0	-.003	.085	-.002	-.035	.972

Coefficients[a]

Model		Correlations Zero-order	Partial	Part
1	(Constant)			
	Party0	.769	.769	.769
2	(Constant)			
	Party0	.769	.750	.710
	GenderCC	.172	.058	.037
	RaceCC	.251	.181	.115
	SES0	.129	.054	.034
3	(Constant)			
	Party0	.769	.668	.552
	GenderCC	.172	-.032	-.020
	RaceCC	.251	.191	.119
	SES0	.129	.041	.025
	MRN0	.512	.195	.122
4	(Constant)			
	Party0	.769	.673	.554
	GenderCC	.172	-.052	-.031
	RaceCC	.251	.189	.117
	SES0	.129	.030	.018
	MRN0	.512	.217	.135
	MRN0xRace	.267	-.074	-.045
	MRN0xSES0	-.041	-.038	-.023
	MRN0xGender	-.015	.114	.070
	MRN0xParty0	.051	-.003	-.002

a. Dependent Variable: Do you approve or disapprove of the way Donald Trump is handling his job as President?

Excluded Variables[a]

Model		Beta In	t	Sig.	Partial Correlation	Collinearity Statistics Tolerance
1	GenderCC	.035[b]	.720	.472	.054	.968
	RaceCC	.117[b]	2.416	.017	.180	.968
	SES0	.042[b]	.872	.385	.066	.987
	MRN0	.146[b]	2.608	.010	.193	.720
	MRN0xRace	.017[b]	.330	.742	.025	.893
	MRN0xSES0	-.027[b]	-.566	.572	-.043	1.000
	MRN0xGender	.054[b]	1.113	.267	.084	.992
	MRN0xParty0	.023[b]	.482	.631	.036	.999
2	MRN0	.161[c]	2.609	.010	.195	.578
	MRN0xRace	.023[c]	.443	.658	.034	.859
	MRN0xSES0	-.016[c]	-.330	.742	-.025	.976
	MRN0xGender	.057[c]	1.187	.237	.090	.985
	MRN0xParty0	.022[c]	.460	.646	.035	.996
3	MRN0xRace	-.065[d]	-1.096	.274	-.084	.618
	MRN0xSES0	-.021[d]	-.440	.660	-.034	.974
	MRN0xGender	.071[d]	1.493	.137	.113	.974
	MRN0xParty0	.014[d]	.292	.771	.022	.992

a. Dependent Variable: Do you approve or disapprove of the way Donald Trump is handling his job as President?

b. Predictors in the Model: (Constant), Party0

c. Predictors in the Model: (Constant), Party0, SES0, RaceCC, GenderCC

d. Predictors in the Model: (Constant), Party0, SES0, RaceCC, GenderCC, MRN0

```
REGRESSION
 /MISSING LISTWISE
 /STATISTICS COEFF OUTS R ANOVA CHANGE ZPP
 /CRITERIA=PIN(.05) POUT(.10)
 /NOORIGIN
 /DEPENDENT TrumpApproval
 /METHOD=ENTER Ideology0
 /METHOD=ENTER GenderCC RaceCC SES0
 /METHOD=ENTER MRN0
```

```
/METHOD=ENTER MRN0xRace MRN0xSES0 MRN0xGender MRN0xIdeology0.
```

Regression

Notes

Output Created		15-DEC-2021 13:06:15
Comments		
Input	Data	C: \Users\njs5478\Dropbox\HM and COVID\0. Revise and Resubmit\2. R and R Data\Study 1a\Study1a_Data.sav
	Active Dataset	DataSet1
	Filter	Inclusion = 1 (FILTER)
	Weight	<none>
	Split File	<none>
	N of Rows in Working Data File	178
Missing Value Handling	Definition of Missing	User-defined missing values are treated as missing.
	Cases Used	Statistics are based on cases with no missing values for any variable used.
Syntax		REGRESSION /MISSING LISTWISE /STATISTICS COEFF OUTS R ANOVA CHANGE ZPP /CRITERIA=PIN(.05) POUT(.10) /NOORIGIN /DEPENDENT TrumpApproval /METHOD=ENTER Ideology0 /METHOD=ENTER GenderCC RaceCC SES0 /METHOD=ENTER MRN0 /METHOD=ENTER MRN0xRace MRN0xSES0 MRN0xGender MRN0xIdeology0.
Resources	Processor Time	00:00:00.03
	Elapsed Time	00:00:00.02

Notes

	Memory Required	43600 bytes
	Additional Memory Required for Residual Plots	0 bytes

Variables Entered/Removed[a]

Model	Variables Entered	Variables Removed	Method
1	Ideology0[b]	.	Enter
2	SES0, RaceCC, GenderCC[b]	.	Enter
3	MRN0[b]	.	Enter
4	MRN0xIdeology0, MRN0xSES0, MRN0xGender, MRN0xRace[b]	.	Enter

a. Dependent Variable: Do you approve or disapprove of the way Donald Trump is handling his job as President?

b. All requested variables entered.

Model Summary

					Change Statistics		
Model	R	R Square	Adjusted R Square	Std. Error of the Estimate	R Square Change	F Change	df1
1	.730[a]	.533	.531	1.451	.533	201.132	1
2	.744[b]	.553	.543	1.432	.020	2.573	3
3	.748[c]	.559	.546	1.427	.006	2.289	1
4	.758[d]	.574	.552	1.418	.015	1.518	4

Model Summary

Model	Change Statistics df2	Sig. F Change
1	176	.000
2	173	.056
3	172	.132
4	168	.199

a. Predictors: (Constant), Ideology0

b. Predictors: (Constant), Ideology0, SES0, RaceCC, GenderCC

c. Predictors: (Constant), Ideology0, SES0, RaceCC, GenderCC, MRN0

d. Predictors: (Constant), Ideology0, SES0, RaceCC, GenderCC, MRN0, MRN0xIdeology0, MRN0xSES0, MRN0xGender, MRN0xRace

ANOVA[a]

Model		Sum of Squares	df	Mean Square	F	Sig.
1	Regression	423.576	1	423.576	201.132	.000[b]
	Residual	370.649	176	2.106		
	Total	794.225	177			
2	Regression	439.405	4	109.851	53.560	.000[c]
	Residual	354.820	173	2.051		
	Total	794.225	177			
3	Regression	444.065	5	88.813	43.625	.000[d]
	Residual	350.160	172	2.036		
	Total	794.225	177			
4	Regression	456.280	9	50.698	25.203	.000[e]
	Residual	337.945	168	2.012		
	Total	794.225	177			

a. Dependent Variable: Do you approve or disapprove of the way Donald Trump is handling his job as President?

b. Predictors: (Constant), Ideology0

c. Predictors: (Constant), Ideology0, SES0, RaceCC, GenderCC

d. Predictors: (Constant), Ideology0, SES0, RaceCC, GenderCC, MRN0

e. Predictors: (Constant), Ideology0, SES0, RaceCC, GenderCC, MRN0, MRN0xIdeology0, MRN0xSES0, MRN0xGender, MRN0xRace

Coefficients[a]

Model		Unstandardized Coefficients B	Unstandardized Coefficients Std. Error	Standardized Coefficients Beta	t	Sig.
1	(Constant)	3.459	.109		31.797	.000
	Ideology0	.936	.066	.730	14.182	.000
2	(Constant)	3.257	.130		24.967	.000
	Ideology0	.920	.070	.718	13.209	.000
	GenderCC	-.083	.113	-.039	-.728	.467
	RaceCC	.343	.132	.134	2.604	.010
	SES0	.041	.131	.016	.317	.752
3	(Constant)	3.245	.130		24.921	.000
	Ideology0	.841	.087	.656	9.701	.000
	GenderCC	-.150	.121	-.071	-1.233	.219
	RaceCC	.353	.131	.138	2.684	.008
	SES0	.032	.131	.013	.248	.804
	MRN0	.254	.168	.109	1.513	.132
4	(Constant)	3.209	.146		21.971	.000
	Ideology0	.859	.087	.670	9.908	.000
	GenderCC	-.193	.122	-.091	-1.583	.115
	RaceCC	.352	.133	.138	2.652	.009
	SES0	.009	.131	.003	.067	.947
	MRN0	.427	.190	.182	2.243	.026
	MRN0xRace	-.237	.157	-.101	-1.511	.133
	MRN0xSES0	-.056	.154	-.020	-.365	.715
	MRN0xGender	.258	.155	.098	1.669	.097
	MRN0xIdeology0	-.061	.074	-.048	-.821	.413

Coefficients[a]

Model		Correlations Zero-order	Partial	Part
1	(Constant)			
	Ideology0	.730	.730	.730
2	(Constant)			
	Ideology0	.730	.709	.671
	GenderCC	.172	-.055	-.037
	RaceCC	.251	.194	.132
	SES0	.129	.024	.016
3	(Constant)			
	Ideology0	.730	.595	.491
	GenderCC	.172	-.094	-.062
	RaceCC	.251	.201	.136
	SES0	.129	.019	.013
	MRN0	.512	.115	.077
4	(Constant)			
	Ideology0	.730	.607	.499
	GenderCC	.172	-.121	-.080
	RaceCC	.251	.200	.133
	SES0	.129	.005	.003
	MRN0	.512	.170	.113
	MRN0xRace	.267	-.116	-.076
	MRN0xSES0	-.041	-.028	-.018
	MRN0xGender	-.015	.128	.084
	MRN0xIdeology0	-.002	-.063	-.041

a. Dependent Variable: Do you approve or disapprove of the way Donald Trump is handling his job as President?

Excluded Variables[a]

Model		Beta In	t	Sig.	Partial Correlation	Collinearity Statistics Tolerance
1	GenderCC	-.046[b]	-.862	.390	-.065	.914
	RaceCC	.137[b]	2.679	.008	.198	.974
	SES0	.018[b]	.344	.731	.026	.977
	MRN0	.065[b]	.958	.339	.072	.578
	MRN0xRace	-.071[b]	-1.234	.219	-.093	.804
	MRN0xSES0	-.037[b]	-.722	.471	-.055	1.000
	MRN0xGender	.052[b]	1.014	.312	.076	.991
	MRN0xIdeology0	-.007[b]	-.143	.887	-.011	1.000
2	MRN0	.109[c]	1.513	.132	.115	.496
	MRN0xRace	-.050[c]	-.872	.384	-.066	.784
	MRN0xSES0	-.027[c]	-.521	.603	-.040	.975
	MRN0xGender	.062[c]	1.215	.226	.092	.983
	MRN0xIdeology0	-.019[c]	-.375	.708	-.029	.993
3	MRN0xRace	-.116[d]	-1.804	.073	-.137	.612
	MRN0xSES0	-.029[d]	-.569	.570	-.043	.974
	MRN0xGender	.070[d]	1.368	.173	.104	.974
	MRN0xIdeology0	-.020[d]	-.387	.699	-.030	.993

a. Dependent Variable: Do you approve or disapprove of the way Donald Trump is handling his job as President?

b. Predictors in the Model: (Constant), Ideology0

c. Predictors in the Model: (Constant), Ideology0, SES0, RaceCC, GenderCC

d. Predictors in the Model: (Constant), Ideology0, SES0, RaceCC, GenderCC, MRN0

```
REGRESSION
 /MISSING LISTWISE
 /STATISTICS COEFF OUTS R ANOVA CHANGE ZPP
 /CRITERIA=PIN(.05) POUT(.10)
 /NOORIGIN
 /DEPENDENT TrumpX
 /METHOD=ENTER Party0
 /METHOD=ENTER GenderCC RaceCC SES0
 /METHOD=ENTER MRN0
```

```
/METHOD=ENTER MRN0xRace MRN0xSES0 MRN0xGender MRN0xParty0.
```

Regression

Notes

Output Created		15-DEC-2021 13:06:15
Comments		
Input	Data	C:\Users\njs5478\Dropbox\HM and COVID\0. Revise and Resubmit\2. R and R Data\Study 1a\Study1a_Data.sav
	Active Dataset	DataSet1
	Filter	Inclusion = 1 (FILTER)
	Weight	<none>
	Split File	<none>
	N of Rows in Working Data File	178
Missing Value Handling	Definition of Missing	User-defined missing values are treated as missing.
	Cases Used	Statistics are based on cases with no missing values for any variable used.
Syntax		REGRESSION /MISSING LISTWISE /STATISTICS COEFF OUTS R ANOVA CHANGE ZPP /CRITERIA=PIN(.05) POUT(.10) /NOORIGIN /DEPENDENT TrumpX /METHOD=ENTER Party0 /METHOD=ENTER GenderCC RaceCC SES0 /METHOD=ENTER MRN0 /METHOD=ENTER MRN0xRace MRN0xSES0 MRN0xGender MRN0xParty0.
Resources	Processor Time	00:00:00.02
	Elapsed Time	00:00:00.02

Notes

	Memory Required	43600 bytes
	Additional Memory Required for Residual Plots	0 bytes

Variables Entered/Removed[a]

Model	Variables Entered	Variables Removed	Method
1	Party0[b]	.	Enter
2	SES0, RaceCC, GenderCC[b]	.	Enter
3	MRN0[b]	.	Enter
4	MRN0xParty0 , MRN0xSES0, MRN0xGende r, [b] ...	.	Enter

a. Dependent Variable: TrumpX

b. All requested variables entered.

Model Summary

Model	R	R Square	Adjusted R Square	Std. Error of the Estimate	Change Statistics		
					R Square Change	F Change	df1
1	.715[a]	.512	.509	1.50368	.512	181.258	1
2	.724[b]	.525	.513	1.49660	.013	1.546	3
3	.738[c]	.545	.532	1.46830	.021	7.617	1
4	.744[d]	.554	.530	1.47162	.009	.810	4

Model Summary

Model	Change Statistics	
	df2	Sig. F Change
1	173	.000
2	170	.204
3	169	.006
4	165	.521

a. Predictors: (Constant), Party0

b. Predictors: (Constant), Party0, SES0, RaceCC, GenderCC

c. Predictors: (Constant), Party0, SES0, RaceCC, GenderCC, MRN0

d. Predictors: (Constant), Party0, SES0, RaceCC, GenderCC, MRN0, MRN0xParty0, MRN0xSES0, MRN0xGender, MRN0xRace

ANOVA[a]

Model		Sum of Squares	df	Mean Square	F	Sig.
1	Regression	409.833	1	409.833	181.258	.000[b]
	Residual	391.161	173	2.261		
	Total	800.994	174			
2	Regression	420.224	4	105.056	46.904	.000[c]
	Residual	380.770	170	2.240		
	Total	800.994	174			
3	Regression	436.645	5	87.329	40.507	.000[d]
	Residual	364.349	169	2.156		
	Total	800.994	174			
4	Regression	443.658	9	49.295	22.762	.000[e]
	Residual	357.336	165	2.166		
	Total	800.994	174			

a. Dependent Variable: TrumpX

b. Predictors: (Constant), Party0

c. Predictors: (Constant), Party0, SES0, RaceCC, GenderCC

d. Predictors: (Constant), Party0, SES0, RaceCC, GenderCC, MRN0

e. Predictors: (Constant), Party0, SES0, RaceCC, GenderCC, MRN0, MRN0xParty0, MRN0xSES0, MRN0xGender, MRN0xRace

Coefficients[a]

Model		Unstandardized Coefficients B	Unstandardized Coefficients Std. Error	Standardized Coefficients Beta	t	Sig.
1	(Constant)	3.441	.114		30.269	.000
	Party0	1.109	.082	.715	13.463	.000
2	(Constant)	3.282	.138		23.776	.000
	Party0	1.077	.085	.695	12.681	.000
	GenderCC	-.011	.116	-.005	-.096	.924
	RaceCC	.281	.139	.108	2.013	.046
	SES0	.090	.137	.035	.660	.510
3	(Constant)	3.259	.136		24.025	.000
	Party0	.941	.097	.607	9.728	.000
	GenderCC	-.160	.126	-.074	-1.266	.207
	RaceCC	.291	.137	.112	2.130	.035
	SES0	.062	.135	.024	.463	.644
	MRN0	.445	.161	.189	2.760	.006
4	(Constant)	3.149	.154		20.452	.000
	Party0	.946	.097	.610	9.751	.000
	GenderCC	-.179	.128	-.083	-1.399	.164
	RaceCC	.289	.138	.112	2.097	.037
	SES0	.063	.137	.024	.458	.648
	MRN0	.545	.191	.231	2.853	.005
	MRN0xRace	-.140	.162	-.060	-.867	.387
	MRN0xSES0	.056	.160	.019	.350	.727
	MRN0xGender	.165	.151	.062	1.092	.276
	MRN0xParty0	.059	.095	.035	.624	.533

Coefficients[a]

Model		Correlations		
		Zero-order	Partial	Part
1	(Constant)			
	Party0	.715	.715	.715
2	(Constant)			
	Party0	.715	.697	.671
	GenderCC	.125	-.007	-.005
	RaceCC	.224	.153	.106
	SES0	.121	.051	.035
3	(Constant)			
	Party0	.715	.599	.505
	GenderCC	.125	-.097	-.066
	RaceCC	.224	.162	.111
	SES0	.121	.036	.024
	MRN0	.488	.208	.143
4	(Constant)			
	Party0	.715	.605	.507
	GenderCC	.125	-.108	-.073
	RaceCC	.224	.161	.109
	SES0	.121	.036	.024
	MRN0	.488	.217	.148
	MRN0xRace	.273	-.067	-.045
	MRN0xSES0	.005	.027	.018
	MRN0xGender	-.007	.085	.057
	MRN0xParty0	.096	.049	.032

a. Dependent Variable: TrumpX

Excluded Variables[a]

Model		Beta In	t	Sig.	Partial Correlation	Collinearity Statistics Tolerance
1	GenderCC	-.007[b]	-.123	.902	-.009	.966
	RaceCC	.110[b]	2.060	.041	.155	.973
	SES0	.039[b]	.735	.463	.056	.987
	MRN0	.149[b]	2.395	.018	.180	.715
	MRN0xRace	.035[b]	.627	.531	.048	.886
	MRN0xSES0	.018[b]	.337	.737	.026	1.000
	MRN0xGender	.057[b]	1.069	.286	.081	.992
	MRN0xParty0	.064[b]	1.205	.230	.091	.998
2	MRN0	.189[c]	2.760	.006	.208	.576
	MRN0xRace	.048[c]	.836	.404	.064	.854
	MRN0xSES0	.031[c]	.571	.569	.044	.977
	MRN0xGender	.063[c]	1.178	.240	.090	.986
	MRN0xParty0	.061[c]	1.149	.252	.088	.995
3	MRN0xRace	-.046[d]	-.699	.485	-.054	.619
	MRN0xSES0	.025[d]	.480	.632	.037	.975
	MRN0xGender	.078[d]	1.489	.138	.114	.976
	MRN0xParty0	.053[d]	1.008	.315	.078	.991

a. Dependent Variable: TrumpX

b. Predictors in the Model: (Constant), Party0

c. Predictors in the Model: (Constant), Party0, SES0, RaceCC, GenderCC

d. Predictors in the Model: (Constant), Party0, SES0, RaceCC, GenderCC, MRN0

```
REGRESSION
 /MISSING LISTWISE
 /STATISTICS COEFF OUTS R ANOVA CHANGE ZPP
 /CRITERIA=PIN(.05) POUT(.10)
 /NOORIGIN
 /DEPENDENT TrumpX
 /METHOD=ENTER Ideology0
 /METHOD=ENTER GenderCC RaceCC SES0
 /METHOD=ENTER MRN0
 /METHOD=ENTER MRN0xRace MRN0xSES0 MRN0xGender MRN0xIdeology0.
```

Regression

Notes

Output Created		15-DEC-2021 13:06:15
Comments		
Input	Data	C:\Users\njs5478\Dropbox\HM and COVID\0. Revise and Resubmit\2. R and R Data\Study 1a\Study1a_Data.sav
	Active Dataset	DataSet1
	Filter	Inclusion = 1 (FILTER)
	Weight	<none>
	Split File	<none>
	N of Rows in Working Data File	178
Missing Value Handling	Definition of Missing	User-defined missing values are treated as missing.
	Cases Used	Statistics are based on cases with no missing values for any variable used.
Syntax		REGRESSION<br>/MISSING LISTWISE<br>/STATISTICS COEFF OUTS R ANOVA CHANGE ZPP<br>/CRITERIA=PIN(.05) POUT(.10)<br>/NOORIGIN<br>/DEPENDENT TrumpX<br>/METHOD=ENTER Ideology0<br>/METHOD=ENTER GenderCC RaceCC SES0<br>/METHOD=ENTER MRN0<br>/METHOD=ENTER MRN0xRace MRN0xSES0 MRN0xGender MRN0xIdeology0.
Resources	Processor Time	00:00:00.02
	Elapsed Time	00:00:00.02

Notes

	Memory Required	43600 bytes
	Additional Memory Required for Residual Plots	0 bytes

Variables Entered/Removed[a]

Model	Variables Entered	Variables Removed	Method
1	Ideology0[b]	.	Enter
2	SES0, RaceCC, GenderCC[b]	.	Enter
3	MRN0[b]	.	Enter
4	MRN0xIdeology0, MRN0xSES0, MRN0xGender, MRN0xRace[b]	.	Enter

a. Dependent Variable: TrumpX

b. All requested variables entered.

Model Summary

Model	R	R Square	Adjusted R Square	Std. Error of the Estimate	Change Statistics		
					R Square Change	F Change	df1
1	.661[a]	.437	.434	1.61423	.437	134.398	1
2	.677[b]	.458	.445	1.59770	.021	2.199	3
3	.686[c]	.470	.454	1.58480	.012	3.779	1
4	.693[d]	.481	.453	1.58745	.011	.859	4

Model Summary

Model	Change Statistics	
	df2	Sig. F Change
1	173	.000
2	170	.090
3	169	.054
4	165	.490

a. Predictors: (Constant), Ideology0

b. Predictors: (Constant), Ideology0, SES0, RaceCC, GenderCC

c. Predictors: (Constant), Ideology0, SES0, RaceCC, GenderCC, MRN0

d. Predictors: (Constant), Ideology0, SES0, RaceCC, GenderCC, MRN0, MRN0xIdeology0, MRN0xSES0, MRN0xGender, MRN0xRace

ANOVA[a]

Model		Sum of Squares	df	Mean Square	F	Sig.
1	Regression	350.204	1	350.204	134.398	.000[b]
	Residual	450.790	173	2.606		
	Total	800.994	174			
2	Regression	367.044	4	91.761	35.947	.000[c]
	Residual	433.950	170	2.553		
	Total	800.994	174			
3	Regression	376.536	5	75.307	29.984	.000[d]
	Residual	424.459	169	2.512		
	Total	800.994	174			
4	Regression	385.193	9	42.799	16.984	.000[e]
	Residual	415.801	165	2.520		
	Total	800.994	174			

a. Dependent Variable: TrumpX

b. Predictors: (Constant), Ideology0

c. Predictors: (Constant), Ideology0, SES0, RaceCC, GenderCC

d. Predictors: (Constant), Ideology0, SES0, RaceCC, GenderCC, MRN0

e. Predictors: (Constant), Ideology0, SES0, RaceCC, GenderCC, MRN0, MRN0xIdeology0, MRN0xSES0, MRN0xGender, MRN0xRace

Coefficients[a]

Model		Unstandardized Coefficients B	Unstandardized Coefficients Std. Error	Standardized Coefficients Beta	t	Sig.
1	(Constant)	3.451	.122		28.283	.000
	Ideology0	.858	.074	.661	11.593	.000
2	(Constant)	3.268	.147		22.181	.000
	Ideology0	.862	.079	.665	10.967	.000
	GenderCC	-.178	.128	-.083	-1.387	.167
	RaceCC	.299	.149	.115	2.008	.046
	SES0	.070	.147	.027	.478	.633
3	(Constant)	3.247	.147		22.154	.000
	Ideology0	.745	.098	.575	7.569	.000
	GenderCC	-.270	.136	-.126	-1.988	.048
	RaceCC	.314	.148	.121	2.127	.035
	SES0	.054	.146	.021	.373	.710
	MRN0	.368	.189	.156	1.944	.054
4	(Constant)	3.161	.166		19.074	.000
	Ideology0	.762	.099	.587	7.678	.000
	GenderCC	-.303	.138	-.141	-2.200	.029
	RaceCC	.307	.150	.118	2.042	.043
	SES0	.045	.148	.018	.306	.760
	MRN0	.515	.215	.219	2.395	.018
	MRN0xRace	-.219	.176	-.093	-1.245	.215
	MRN0xSES0	.042	.173	.015	.245	.807
	MRN0xGender	.191	.175	.072	1.093	.276
	MRN0xIdeology0	.011	.084	.009	.129	.898

Coefficients[a]

Model		Correlations Zero-order	Partial	Part
1	(Constant)			
	Ideology0	.661	.661	.661
2	(Constant)			
	Ideology0	.661	.644	.619
	GenderCC	.125	-.106	-.078
	RaceCC	.224	.152	.113
	SES0	.121	.037	.027
3	(Constant)			
	Ideology0	.661	.503	.424
	GenderCC	.125	-.151	-.111
	RaceCC	.224	.161	.119
	SES0	.121	.029	.021
	MRN0	.488	.148	.109
4	(Constant)			
	Ideology0	.661	.513	.431
	GenderCC	.125	-.169	-.123
	RaceCC	.224	.157	.115
	SES0	.121	.024	.017
	MRN0	.488	.183	.134
	MRN0xRace	.273	-.096	-.070
	MRN0xSES0	.005	.019	.014
	MRN0xGender	-.007	.085	.061
	MRN0xIdeology0	.059	.010	.007

a. Dependent Variable: TrumpX

Excluded Variables[a]

Model		Beta In	t	Sig.	Partial Correlation	Collinearity Statistics Tolerance
1	GenderCC	-.089[b]	-1.488	.139	-.113	.904
	RaceCC	.122[b]	2.132	.034	.160	.975
	SES0	.025[b]	.434	.665	.033	.979
	MRN0	.090[b]	1.177	.241	.089	.561
	MRN0xRace	-.034[b]	-.523	.602	-.040	.794
	MRN0xSES0	.004[b]	.065	.948	.005	1.000
	MRN0xGender	.056[b]	.969	.334	.074	.991
	MRN0xIdeology0	.044[b]	.773	.441	.059	.999
2	MRN0	.156[c]	1.944	.054	.148	.487
	MRN0xRace	-.010[c]	-.163	.870	-.013	.778
	MRN0xSES0	.016[c]	.273	.785	.021	.976
	MRN0xGender	.068[c]	1.193	.234	.091	.983
	MRN0xIdeology0	.032[c]	.565	.573	.043	.992
3	MRN0xRace	-.086[d]	-1.197	.233	-.092	.613
	MRN0xSES0	.013[d]	.235	.815	.018	.975
	MRN0xGender	.078[d]	1.377	.170	.106	.976
	MRN0xIdeology0	.034[d]	.597	.552	.046	.992

a. Dependent Variable: TrumpX

b. Predictors in the Model: (Constant), Ideology0

c. Predictors in the Model: (Constant), Ideology0, SES0, RaceCC, GenderCC

d. Predictors in the Model: (Constant), Ideology0, SES0, RaceCC, GenderCC, MRN0

```
REGRESSION
 /MISSING LISTWISE
 /STATISTICS COEFF OUTS R ANOVA CHANGE ZPP
 /CRITERIA=PIN(.05) POUT(.10)
 /NOORIGIN
 /DEPENDENT BidenX
 /METHOD=ENTER Party0
 /METHOD=ENTER GenderCC RaceCC SES0
 /METHOD=ENTER MRN0
 /METHOD=ENTER MRN0xRace MRN0xSES0 MRN0xGender MRN0xParty0.
```

Regression

Notes

Output Created		15-DEC-2021 13:06:15
Comments		
Input	Data	C:\Users\njs5478\Dropbox\HM and COVID\0. Revise and Resubmit\2. R and R Data\Study 1a\Study1a_Data.sav
	Active Dataset	DataSet1
	Filter	Inclusion = 1 (FILTER)
	Weight	<none>
	Split File	<none>
	N of Rows in Working Data File	178
Missing Value Handling	Definition of Missing	User-defined missing values are treated as missing.
	Cases Used	Statistics are based on cases with no missing values for any variable used.
Syntax		REGRESSION /MISSING LISTWISE /STATISTICS COEFF OUTS R ANOVA CHANGE ZPP /CRITERIA=PIN(.05) POUT(.10) /NOORIGIN /DEPENDENT BidenX /METHOD=ENTER Party0 /METHOD=ENTER GenderCC RaceCC SES0 /METHOD=ENTER MRN0 /METHOD=ENTER MRN0xRace MRN0xSES0 MRN0xGender MRN0xParty0.
Resources	Processor Time	00:00:00.02
	Elapsed Time	00:00:00.02

Notes

	Memory Required	43600 bytes
	Additional Memory Required for Residual Plots	0 bytes

Variables Entered/Removed[a]

Model	Variables Entered	Variables Removed	Method
1	Party0[b]	.	Enter
2	SES0, RaceCC, GenderCC[b]	.	Enter
3	MRN0[b]	.	Enter
4	MRN0xSES0, MRN0xParty0 , MRN0xGender, ...[b]	.	Enter

a. Dependent Variable: BidenX

b. All requested variables entered.

Model Summary

Model	R	R Square	Adjusted R Square	Std. Error of the Estimate	Change Statistics		
					R Square Change	F Change	df1
1	.273[a]	.075	.068	1.36586	.075	11.232	1
2	.289[b]	.084	.057	1.37408	.009	.447	3
3	.305[c]	.093	.059	1.37236	.009	1.341	1
4	.335[d]	.112	.052	1.37796	.020	.726	4

Model Summary

Model	Change Statistics	
	df2	Sig. F Change
1	139	.001
2	136	.720
3	135	.249
4	131	.575

a. Predictors: (Constant), Party0

b. Predictors: (Constant), Party0, SES0, RaceCC, GenderCC

c. Predictors: (Constant), Party0, SES0, RaceCC, GenderCC, MRN0

d. Predictors: (Constant), Party0, SES0, RaceCC, GenderCC, MRN0, MRN0xSES0, MRN0xParty0, MRN0xGender, MRN0xRace

ANOVA[a]

Model		Sum of Squares	df	Mean Square	F	Sig.
1	Regression	20.953	1	20.953	11.232	.001[b]
	Residual	259.316	139	1.866		
	Total	280.270	140			
2	Regression	23.487	4	5.872	3.110	.017[c]
	Residual	256.782	136	1.888		
	Total	280.270	140			
3	Regression	26.014	5	5.203	2.762	.021[d]
	Residual	254.256	135	1.883		
	Total	280.270	140			
4	Regression	31.530	9	3.503	1.845	.066[e]
	Residual	248.739	131	1.899		
	Total	280.270	140			

a. Dependent Variable: BidenX

b. Predictors: (Constant), Party0

c. Predictors: (Constant), Party0, SES0, RaceCC, GenderCC

d. Predictors: (Constant), Party0, SES0, RaceCC, GenderCC, MRN0

e. Predictors: (Constant), Party0, SES0, RaceCC, GenderCC, MRN0, MRN0xSES0, MRN0xParty0, MRN0xGender, MRN0xRace

Coefficients[a]

Model		Unstandardized Coefficients B	Unstandardized Coefficients Std. Error	Standardized Coefficients Beta	t	Sig.
1	(Constant)	3.684	.115		32.025	.000
	Party0	-.272	.081	-.273	-3.351	.001
2	(Constant)	3.615	.144		25.071	.000
	Party0	-.289	.084	-.290	-3.428	.001
	GenderCC	.013	.119	.009	.111	.912
	RaceCC	.118	.146	.068	.810	.419
	SES0	.112	.146	.063	.764	.446
3	(Constant)	3.595	.145		24.771	.000
	Party0	-.352	.100	-.354	-3.508	.001
	GenderCC	-.041	.127	-.029	-.321	.749
	RaceCC	.130	.146	.075	.892	.374
	SES0	.094	.147	.053	.638	.525
	MRN0	.198	.171	.125	1.158	.249
4	(Constant)	3.663	.164		22.309	.000
	Party0	-.338	.102	-.339	-3.320	.001
	GenderCC	-.068	.130	-.048	-.521	.604
	RaceCC	.136	.148	.078	.922	.358
	SES0	.092	.148	.052	.626	.533
	MRN0	.303	.197	.191	1.538	.126
	MRN0xRace	-.183	.164	-.115	-1.114	.267
	MRN0xSES0	.035	.169	.018	.210	.834
	MRN0xGender	-.005	.156	-.003	-.035	.972
	MRN0xParty0	-.103	.097	-.096	-1.065	.289

Coefficients[a]

Model		Correlations Zero-order	Partial	Part
1	(Constant)			
	Party0	-.273	-.273	-.273
2	(Constant)			
	Party0	-.273	-.282	-.281
	GenderCC	-.035	.010	.009
	RaceCC	.024	.069	.067
	SES0	.051	.065	.063
3	(Constant)			
	Party0	-.273	-.289	-.288
	GenderCC	-.035	-.028	-.026
	RaceCC	.024	.077	.073
	SES0	.051	.055	.052
	MRN0	-.075	.099	.095
4	(Constant)			
	Party0	-.273	-.279	-.273
	GenderCC	-.035	-.045	-.043
	RaceCC	.024	.080	.076
	SES0	.051	.055	.052
	MRN0	-.075	.133	.127
	MRN0xRace	-.135	-.097	-.092
	MRN0xSES0	-.010	.018	.017
	MRN0xGender	-.015	-.003	-.003
	MRN0xParty0	-.132	-.093	-.088

a. Dependent Variable: BidenX

Excluded Variables[a]

Model		Beta In	t	Sig.	Partial Correlation	Collinearity Statistics Tolerance
1	GenderCC	.010[b]	.121	.904	.010	.973
	RaceCC	.071[b]	.852	.396	.072	.974
	SES0	.068[b]	.831	.407	.071	.996
	MRN0	.112[b]	1.139	.257	.096	.689
	MRN0xRace	-.044[b]	-.501	.617	-.043	.876
	MRN0xSES0	-.011[b]	-.133	.894	-.011	1.000
	MRN0xGender	-.041[b]	-.499	.618	-.042	.991
	MRN0xParty0	-.107[b]	-1.314	.191	-.111	.991
2	MRN0	.125[c]	1.158	.249	.099	.580
	MRN0xRace	-.048[c]	-.541	.589	-.047	.866
	MRN0xSES0	-.012[c]	-.140	.889	-.012	.999
	MRN0xGender	-.037[c]	-.449	.654	-.039	.972
	MRN0xParty0	-.108[c]	-1.308	.193	-.112	.983
3	MRN0xRace	-.128[d]	-1.272	.205	-.109	.660
	MRN0xSES0	-.014[d]	-.165	.869	-.014	.998
	MRN0xGender	-.033[d]	-.390	.697	-.034	.970
	MRN0xParty0	-.107[d]	-1.296	.197	-.111	.983

a. Dependent Variable: BidenX

b. Predictors in the Model: (Constant), Party0

c. Predictors in the Model: (Constant), Party0, SES0, RaceCC, GenderCC

d. Predictors in the Model: (Constant), Party0, SES0, RaceCC, GenderCC, MRN0

```
REGRESSION
 /MISSING LISTWISE
 /STATISTICS COEFF OUTS R ANOVA CHANGE ZPP
 /CRITERIA=PIN(.05) POUT(.10)
 /NOORIGIN
 /DEPENDENT BidenX
 /METHOD=ENTER Ideology0
 /METHOD=ENTER GenderCC RaceCC SES0
 /METHOD=ENTER MRN0
 /METHOD=ENTER MRN0xRace MRN0xSES0 MRN0xGender MRN0xIdeology0.
```

Regression

Notes

Output Created		15-DEC-2021 13:06:16
Comments		
Input	Data	C:\Users\njs5478\Dropbox\HM and COVID\0. Revise and Resubmit\2. R and R Data\Study 1a\Study1a_Data.sav
	Active Dataset	DataSet1
	Filter	Inclusion = 1 (FILTER)
	Weight	<none>
	Split File	<none>
	N of Rows in Working Data File	178
Missing Value Handling	Definition of Missing	User-defined missing values are treated as missing.
	Cases Used	Statistics are based on cases with no missing values for any variable used.
Syntax		REGRESSION /MISSING LISTWISE /STATISTICS COEFF OUTS R ANOVA CHANGE ZPP /CRITERIA=PIN(.05) POUT(.10) /NOORIGIN /DEPENDENT BidenX /METHOD=ENTER Ideology0 /METHOD=ENTER GenderCC RaceCC SES0 /METHOD=ENTER MRN0 /METHOD=ENTER MRN0xRace MRN0xSES0 MRN0xGender MRN0xIdeology0.
Resources	Processor Time	00:00:00.02
	Elapsed Time	00:00:00.02

Notes

	Memory Required	43600 bytes
	Additional Memory Required for Residual Plots	0 bytes

Variables Entered/Removed[a]

Model	Variables Entered	Variables Removed	Method
1	Ideology0[b]	.	Enter
2	SES0, RaceCC, GenderCC[b]	.	Enter
3	MRN0[b]	.	Enter
4	MRN0xSES0, MRN0xGender, MRN0xIdeology0, MRN0xRace[b]	.	Enter

a. Dependent Variable: BidenX

b. All requested variables entered.

Model Summary

Model	R	R Square	Adjusted R Square	Std. Error of the Estimate	Change Statistics		
					R Square Change	F Change	df1
1	.180[a]	.032	.025	1.39681	.032	4.649	1
2	.203[b]	.041	.013	1.40571	.009	.415	3
3	.211[c]	.044	.009	1.40848	.003	.466	1
4	.268[d]	.072	.008	1.40919	.027	.966	4

Model Summary

Model	Change Statistics	
	df2	Sig. F Change
1	139	.033
2	136	.742
3	135	.496
4	131	.429

a. Predictors: (Constant), Ideology0

b. Predictors: (Constant), Ideology0, SES0, RaceCC, GenderCC

c. Predictors: (Constant), Ideology0, SES0, RaceCC, GenderCC, MRN0

d. Predictors: (Constant), Ideology0, SES0, RaceCC, GenderCC, MRN0, MRN0xSES0, MRN0xGender, MRN0xIdeology0, MRN0xRace

ANOVA[a]

Model		Sum of Squares	df	Mean Square	F	Sig.
1	Regression	9.071	1	9.071	4.649	.033[b]
	Residual	271.199	139	1.951		
	Total	280.270	140			
2	Regression	11.532	4	2.883	1.459	.218[c]
	Residual	268.738	136	1.976		
	Total	280.270	140			
3	Regression	12.456	5	2.491	1.256	.287[d]
	Residual	267.814	135	1.984		
	Total	280.270	140			
4	Regression	20.128	9	2.236	1.126	.349[e]
	Residual	260.142	131	1.986		
	Total	280.270	140			

a. Dependent Variable: BidenX

b. Predictors: (Constant), Ideology0

c. Predictors: (Constant), Ideology0, SES0, RaceCC, GenderCC

d. Predictors: (Constant), Ideology0, SES0, RaceCC, GenderCC, MRN0

e. Predictors: (Constant), Ideology0, SES0, RaceCC, GenderCC, MRN0, MRN0xSES0, MRN0xGender, MRN0xIdeology0, MRN0xRace

Coefficients[a]

Model		Unstandardized Coefficients B	Std. Error	Standardized Coefficients Beta	t	Sig.
1	(Constant)	3.684	.118		31.315	.000
	Ideology0	-.148	.069	-.180	-2.156	.033
2	(Constant)	3.628	.148		24.562	.000
	Ideology0	-.168	.074	-.204	-2.276	.024
	GenderCC	.026	.125	.018	.207	.836
	RaceCC	.096	.150	.055	.644	.521
	SES0	.128	.150	.073	.853	.395
3	(Constant)	3.613	.150		24.131	.000
	Ideology0	-.208	.094	-.253	-2.202	.029
	GenderCC	.001	.130	.001	.006	.995
	RaceCC	.107	.151	.062	.712	.478
	SES0	.122	.151	.069	.808	.420
	MRN0	.128	.188	.081	.683	.496
4	(Constant)	3.692	.168		21.980	.000
	Ideology0	-.188	.095	-.229	-1.979	.050
	GenderCC	-.031	.132	-.022	-.231	.818
	RaceCC	.130	.153	.075	.851	.396
	SES0	.123	.151	.070	.818	.415
	MRN0	.203	.209	.128	.970	.334
	MRN0xRace	-.137	.170	-.086	-.804	.423
	MRN0xSES0	.069	.174	.035	.396	.693
	MRN0xGender	.081	.168	.047	.480	.632
	MRN0xIdeology0	-.126	.079	-.157	-1.585	.115

Coefficients[a]

Model		Correlations Zero-order	Partial	Part
1	(Constant)			
	Ideology0	-.180	-.180	-.180
2	(Constant)			
	Ideology0	-.180	-.192	-.191
	GenderCC	-.035	.018	.017
	RaceCC	.024	.055	.054
	SES0	.051	.073	.072
3	(Constant)			
	Ideology0	-.180	-.186	-.185
	GenderCC	-.035	.000	.000
	RaceCC	.024	.061	.060
	SES0	.051	.069	.068
	MRN0	-.075	.059	.057
4	(Constant)			
	Ideology0	-.180	-.170	-.167
	GenderCC	-.035	-.020	-.019
	RaceCC	.024	.074	.072
	SES0	.051	.071	.069
	MRN0	-.075	.084	.082
	MRN0xRace	-.135	-.070	-.068
	MRN0xSES0	-.010	.035	.033
	MRN0xGender	-.015	.042	.040
	MRN0xIdeology0	-.150	-.137	-.133

a. Dependent Variable: BidenX

Excluded Variables[a]

Model		Beta In	t	Sig.	Partial Correlation	Collinearity Statistics Tolerance
1	GenderCC	.018[b]	.205	.838	.017	.919
	RaceCC	.056[b]	.664	.508	.056	.971
	SES0	.076[b]	.907	.366	.077	.982
	MRN0	.077[b]	.693	.489	.059	.563
	MRN0xRace	-.068[b]	-.732	.465	-.062	.804
	MRN0xSES0	-.004[b]	-.048	.962	-.004	.999
	MRN0xGender	-.025[b]	-.296	.768	-.025	.997
	MRN0xIdeology0	-.138[b]	-1.656	.100	-.140	.995
2	MRN0	.081[c]	.683	.496	.059	.506
	MRN0xRace	-.068[c]	-.722	.472	-.062	.802
	MRN0xSES0	-.004[c]	-.052	.959	-.004	.998
	MRN0xGender	-.023[c]	-.272	.786	-.023	.977
	MRN0xIdeology0	-.142[c]	-1.689	.094	-.144	.989
3	MRN0xRace	-.117[d]	-1.126	.262	-.097	.654
	MRN0xSES0	-.004[d]	-.049	.961	-.004	.998
	MRN0xGender	-.019[d]	-.224	.823	-.019	.972
	MRN0xIdeology0	-.141[d]	-1.672	.097	-.143	.989

a. Dependent Variable: BidenX

b. Predictors in the Model: (Constant), Ideology0

c. Predictors in the Model: (Constant), Ideology0, SES0, RaceCC, GenderCC

d. Predictors in the Model: (Constant), Ideology0, SES0, RaceCC, GenderCC, MRN0

```
REGRESSION
 /MISSING LISTWISE
 /STATISTICS COEFF OUTS R ANOVA CHANGE ZPP
 /CRITERIA=PIN(.05) POUT(.10)
 /NOORIGIN
 /DEPENDENT PelosiX
 /METHOD=ENTER Party0
 /METHOD=ENTER GenderCC RaceCC SES0
 /METHOD=ENTER MRN0
 /METHOD=ENTER MRN0xRace MRN0xSES0 MRN0xGender MRN0xParty0.
```

Regression

Notes

Output Created		15-DEC-2021 13:06:16
Comments		
Input	Data	C: \Users\njs5478\Dropbox\HM and COVID\0. Revise and Resubmit\2. R and R Data\Study 1a\Study1a_Data.sav
	Active Dataset	DataSet1
	Filter	Inclusion = 1 (FILTER)
	Weight	<none>
	Split File	<none>
	N of Rows in Working Data File	178
Missing Value Handling	Definition of Missing	User-defined missing values are treated as missing.
	Cases Used	Statistics are based on cases with no missing values for any variable used.
Syntax		REGRESSION /MISSING LISTWISE /STATISTICS COEFF OUTS R ANOVA CHANGE ZPP /CRITERIA=PIN(.05) POUT(.10) /NOORIGIN /DEPENDENT PelosiX /METHOD=ENTER Party0 /METHOD=ENTER GenderCC RaceCC SES0 /METHOD=ENTER MRN0 /METHOD=ENTER MRN0xRace MRN0xSES0 MRN0xGender MRN0xParty0.
Resources	Processor Time	00:00:00.03
	Elapsed Time	00:00:00.03

Notes

	Memory Required	43600 bytes
	Additional Memory Required for Residual Plots	0 bytes

Variables Entered/Removed[a]

Model	Variables Entered	Variables Removed	Method
1	Party0[b]	.	Enter
2	SES0, RaceCC, GenderCC[b]	.	Enter
3	MRN0[b]	.	Enter
4	MRN0xSES0, MRN0xGender, MRN0xParty0, MRN0xRace[b]	.	Enter

a. Dependent Variable: PelosiX

b. All requested variables entered.

Model Summary

Model	R	R Square	Adjusted R Square	Std. Error of the Estimate	Change Statistics		
					R Square Change	F Change	df1
1	.523[a]	.274	.268	1.34617	.274	51.613	1
2	.539[b]	.290	.269	1.34569	.016	1.032	3
3	.540[c]	.291	.265	1.34963	.001	.221	1
4	.547[d]	.299	.250	1.36290	.008	.355	4

Model Summary

Model	Change Statistics	
	df2	Sig. F Change
1	137	.000
2	134	.381
3	133	.639
4	129	.840

a. Predictors: (Constant), Party0

b. Predictors: (Constant), Party0, SES0, RaceCC, GenderCC

c. Predictors: (Constant), Party0, SES0, RaceCC, GenderCC, MRN0

d. Predictors: (Constant), Party0, SES0, RaceCC, GenderCC, MRN0, MRN0xSES0, MRN0xGender, MRN0xParty0, MRN0xRace

ANOVA[a]

Model		Sum of Squares	df	Mean Square	F	Sig.
1	Regression	93.531	1	93.531	51.613	.000[b]
	Residual	248.267	137	1.812		
	Total	341.799	138			
2	Regression	99.139	4	24.785	13.686	.000[c]
	Residual	242.660	134	1.811		
	Total	341.799	138			
3	Regression	99.541	5	19.908	10.930	.000[d]
	Residual	242.258	133	1.821		
	Total	341.799	138			
4	Regression	102.180	9	11.353	6.112	.000[e]
	Residual	239.619	129	1.858		
	Total	341.799	138			

a. Dependent Variable: PelosiX

b. Predictors: (Constant), Party0

c. Predictors: (Constant), Party0, SES0, RaceCC, GenderCC

d. Predictors: (Constant), Party0, SES0, RaceCC, GenderCC, MRN0

e. Predictors: (Constant), Party0, SES0, RaceCC, GenderCC, MRN0, MRN0xSES0, MRN0xGender, MRN0xParty0, MRN0xRace

Coefficients[a]

Model		Unstandardized Coefficients B	Unstandardized Coefficients Std. Error	Standardized Coefficients Beta	t	Sig.
1	(Constant)	3.403	.114		29.801	.000
	Party0	-.590	.082	-.523	-7.184	.000
2	(Constant)	3.555	.144		24.712	.000
	Party0	-.557	.086	-.495	-6.489	.000
	GenderCC	-.002	.117	-.001	-.015	.988
	RaceCC	-.253	.146	-.130	-1.737	.085
	SES0	-.025	.143	-.013	-.176	.861
3	(Constant)	3.562	.145		24.547	.000
	Party0	-.533	.101	-.473	-5.275	.000
	GenderCC	.022	.128	.014	.170	.865
	RaceCC	-.257	.146	-.131	-1.753	.082
	SES0	-.017	.144	-.009	-.117	.907
	MRN0	-.080	.171	-.045	-.470	.639
4	(Constant)	3.541	.164		21.538	.000
	Party0	-.544	.103	-.482	-5.267	.000
	GenderCC	.035	.130	.022	.271	.787
	RaceCC	-.267	.149	-.136	-1.787	.076
	SES0	-.017	.146	-.009	-.116	.908
	MRN0	-.134	.214	-.075	-.625	.533
	MRN0xRace	.114	.184	.064	.616	.539
	MRN0xSES0	-.132	.170	-.061	-.778	.438
	MRN0xGender	-.027	.162	-.014	-.169	.866
	MRN0xParty0	.072	.111	.056	.649	.518

Coefficients[a]

Model		Correlations		
		Zero-order	Partial	Part
1	(Constant)			
	Party0	-.523	-.523	-.523
2	(Constant)			
	Party0	-.523	-.489	-.472
	GenderCC	-.099	-.001	-.001
	RaceCC	-.232	-.148	-.126
	SES0	-.084	-.015	-.013
3	(Constant)			
	Party0	-.523	-.416	-.385
	GenderCC	-.099	.015	.012
	RaceCC	-.232	-.150	-.128
	SES0	-.084	-.010	-.009
	MRN0	-.311	-.041	-.034
4	(Constant)			
	Party0	-.523	-.421	-.388
	GenderCC	-.099	.024	.020
	RaceCC	-.232	-.155	-.132
	SES0	-.084	-.010	-.009
	MRN0	-.311	-.055	-.046
	MRN0xRace	-.169	.054	.045
	MRN0xSES0	-.059	-.068	-.057
	MRN0xGender	.047	-.015	-.012
	MRN0xParty0	.013	.057	.048

a. Dependent Variable: PelosiX

Excluded Variables[a]

Model		Beta In	t	Sig.	Partial Correlation	Collinearity Statistics Tolerance
1	GenderCC	.004[b]	.047	.963	.004	.962
	RaceCC	-.130[b]	-1.763	.080	-.150	.958
	SES0	-.020[b]	-.270	.788	-.023	.985
	MRN0	-.032[b]	-.361	.719	-.031	.694
	MRN0xRace	.038[b]	.476	.635	.041	.852
	MRN0xSES0	-.033[b]	-.446	.656	-.038	.997
	MRN0xGender	.009[b]	.123	.902	.011	.995
	MRN0xParty0	.044[b]	.605	.546	.052	.996
2	MRN0	-.045[c]	-.470	.639	-.041	.572
	MRN0xRace	.028[c]	.354	.724	.031	.830
	MRN0xSES0	-.042[c]	-.578	.564	-.050	.992
	MRN0xGender	.002[c]	.020	.984	.002	.981
	MRN0xParty0	.053[c]	.714	.476	.062	.984
3	MRN0xRace	.071[d]	.734	.464	.064	.577
	MRN0xSES0	-.040[d]	-.538	.591	-.047	.984
	MRN0xGender	.003[d]	.036	.971	.003	.980
	MRN0xParty0	.050[d]	.683	.496	.059	.980

a. Dependent Variable: PelosiX

b. Predictors in the Model: (Constant), Party0

c. Predictors in the Model: (Constant), Party0, SES0, RaceCC, GenderCC

d. Predictors in the Model: (Constant), Party0, SES0, RaceCC, GenderCC, MRN0

```
REGRESSION
 /MISSING LISTWISE
 /STATISTICS COEFF OUTS R ANOVA CHANGE ZPP
 /CRITERIA=PIN(.05) POUT(.10)
 /NOORIGIN
 /DEPENDENT PelosiX
 /METHOD=ENTER Ideology0
 /METHOD=ENTER GenderCC RaceCC SES0
 /METHOD=ENTER MRN0
 /METHOD=ENTER MRN0xRace MRN0xSES0 MRN0xGender MRN0xIdeology0.
```

Regression

Notes

Output Created		15-DEC-2021 13:06:16
Comments		
Input	Data	C: \Users\njs5478\Dropbox\HM and COVID\0. Revise and Resubmit\2. R and R Data\Study 1a\Study1a_Data.sav
	Active Dataset	DataSet1
	Filter	Inclusion = 1 (FILTER)
	Weight	<none>
	Split File	<none>
	N of Rows in Working Data File	178
Missing Value Handling	Definition of Missing	User-defined missing values are treated as missing.
	Cases Used	Statistics are based on cases with no missing values for any variable used.
Syntax		REGRESSION /MISSING LISTWISE /STATISTICS COEFF OUTS R ANOVA CHANGE ZPP /CRITERIA=PIN(.05) POUT(.10) /NOORIGIN /DEPENDENT PelosiX /METHOD=ENTER Ideology0 /METHOD=ENTER GenderCC RaceCC SES0 /METHOD=ENTER MRN0 /METHOD=ENTER MRN0xRace MRN0xSES0 MRN0xGender MRN0xIdeology0.
Resources	Processor Time	00:00:00.02
	Elapsed Time	00:00:00.02

Notes

	Memory Required	43600 bytes
	Additional Memory Required for Residual Plots	0 bytes

Variables Entered/Removed[a]

Model	Variables Entered	Variables Removed	Method
1	Ideology0[b]	.	Enter
2	SES0, RaceCC, GenderCC[b]	.	Enter
3	MRN0[b]	.	Enter
4	MRN0xGender, MRN0xSES0, MRN0xIdeology0, MRN0xRace[b]	.	Enter

a. Dependent Variable: PelosiX

b. All requested variables entered.

Model Summary

Model	R	R Square	Adjusted R Square	Std. Error of the Estimate	Change Statistics		
					R Square Change	F Change	df1
1	.441[a]	.194	.188	1.41795	.194	33.000	1
2	.467[b]	.218	.195	1.41209	.024	1.380	3
3	.471[c]	.222	.193	1.41410	.004	.620	1
4	.480[d]	.230	.176	1.42823	.008	.345	4

Model Summary

Model	Change Statistics	
	df2	Sig. F Change
1	137	.000
2	134	.252
3	133	.432
4	129	.847

a. Predictors: (Constant), Ideology0

b. Predictors: (Constant), Ideology0, SES0, RaceCC, GenderCC

c. Predictors: (Constant), Ideology0, SES0, RaceCC, GenderCC, MRN0

d. Predictors: (Constant), Ideology0, SES0, RaceCC, GenderCC, MRN0, MRN0xGender, MRN0xSES0, MRN0xIdeology0, MRN0xRace

ANOVA[a]

Model		Sum of Squares	df	Mean Square	F	Sig.
1	Regression	66.349	1	66.349	33.000	.000[b]
	Residual	275.449	137	2.011		
	Total	341.799	138			
2	Regression	74.601	4	18.650	9.353	.000[c]
	Residual	267.197	134	1.994		
	Total	341.799	138			
3	Regression	75.841	5	15.168	7.585	.000[d]
	Residual	265.957	133	2.000		
	Total	341.799	138			
4	Regression	78.658	9	8.740	4.284	.000[e]
	Residual	263.141	129	2.040		
	Total	341.799	138			

a. Dependent Variable: PelosiX

b. Predictors: (Constant), Ideology0

c. Predictors: (Constant), Ideology0, SES0, RaceCC, GenderCC

d. Predictors: (Constant), Ideology0, SES0, RaceCC, GenderCC, MRN0

e. Predictors: (Constant), Ideology0, SES0, RaceCC, GenderCC, MRN0, MRN0xGender, MRN0xSES0, MRN0xIdeology0, MRN0xRace

Coefficients[a]

Model		Unstandardized Coefficients B	Unstandardized Coefficients Std. Error	Standardized Coefficients Beta	t	Sig.
1	(Constant)	3.416	.120		28.406	.000
	Ideology0	-.416	.072	-.441	-5.745	.000
2	(Constant)	3.594	.151		23.875	.000
	Ideology0	-.399	.078	-.423	-5.093	.000
	GenderCC	.056	.127	.036	.442	.659
	RaceCC	-.297	.153	-.152	-1.946	.054
	SES0	-.007	.150	-.003	-.044	.965
3	(Constant)	3.607	.152		23.786	.000
	Ideology0	-.355	.097	-.376	-3.674	.000
	GenderCC	.089	.134	.057	.664	.508
	RaceCC	-.304	.153	-.155	-1.985	.049
	SES0	.004	.151	.002	.027	.978
	MRN0	-.148	.188	-.084	-.787	.432
4	(Constant)	3.605	.170		21.151	.000
	Ideology0	-.360	.099	-.382	-3.656	.000
	GenderCC	.099	.137	.063	.722	.472
	RaceCC	-.286	.158	-.146	-1.806	.073
	SES0	.020	.154	.010	.130	.897
	MRN0	-.273	.229	-.154	-1.194	.235
	MRN0xRace	.196	.195	.110	1.009	.315
	MRN0xSES0	-.091	.180	-.042	-.505	.614
	MRN0xGender	.090	.183	.046	.494	.622
	MRN0xIdeology0	-.043	.096	-.045	-.446	.656

Coefficients[a]

Model		Correlations Zero-order	Partial	Part
1	(Constant)			
	Ideology0	-.441	-.441	-.441
2	(Constant)			
	Ideology0	-.441	-.403	-.389
	GenderCC	-.099	.038	.034
	RaceCC	-.232	-.166	-.149
	SES0	-.084	-.004	-.003
3	(Constant)			
	Ideology0	-.441	-.304	-.281
	GenderCC	-.099	.057	.051
	RaceCC	-.232	-.170	-.152
	SES0	-.084	.002	.002
	MRN0	-.311	-.068	-.060
4	(Constant)			
	Ideology0	-.441	-.306	-.282
	GenderCC	-.099	.063	.056
	RaceCC	-.232	-.157	-.140
	SES0	-.084	.011	.010
	MRN0	-.311	-.105	-.092
	MRN0xRace	-.169	.088	.078
	MRN0xSES0	-.059	-.044	-.039
	MRN0xGender	.047	.043	.038
	MRN0xIdeology0	-.029	-.039	-.034

a. Dependent Variable: PelosiX

Excluded Variables[a]

Model		Beta In	t	Sig.	Partial Correlation	Collinearity Statistics Tolerance
1	GenderCC	.046[b]	.572	.568	.049	.898
	RaceCC	-.154[b]	-1.999	.048	-.169	.964
	SES0	-.008[b]	-.103	.918	-.009	.970
	MRN0	-.047[b]	-.462	.645	-.040	.584
	MRN0xRace	.049[b]	.567	.572	.049	.777
	MRN0xSES0	-.021[b]	-.278	.782	-.024	.992
	MRN0xGender	.039[b]	.503	.616	.043	1.000
	MRN0xIdeology0	-.014[b]	-.184	.855	-.016	.999
2	MRN0	-.084[c]	-.787	.432	-.068	.519
	MRN0xRace	.032[c]	.363	.717	.031	.768
	MRN0xSES0	-.034[c]	-.438	.662	-.038	.986
	MRN0xGender	.025[c]	.324	.747	.028	.986
	MRN0xIdeology0	.001[c]	.008	.994	.001	.984
3	MRN0xRace	.090[d]	.886	.377	.077	.570
	MRN0xSES0	-.030[d]	-.392	.696	-.034	.983
	MRN0xGender	.026[d]	.331	.741	.029	.986
	MRN0xIdeology0	-.004[d]	-.054	.957	-.005	.978

a. Dependent Variable: PelosiX

b. Predictors in the Model: (Constant), Ideology0

c. Predictors in the Model: (Constant), Ideology0, SES0, RaceCC, GenderCC

d. Predictors in the Model: (Constant), Ideology0, SES0, RaceCC, GenderCC, MRN0

```
REGRESSION
 /MISSING LISTWISE
 /STATISTICS COEFF OUTS R ANOVA CHANGE ZPP
 /CRITERIA=PIN(.05) POUT(.10)
 /NOORIGIN
 /DEPENDENT McConnellX
 /METHOD=ENTER Party0
 /METHOD=ENTER GenderCC RaceCC SES0
 /METHOD=ENTER MRN0
 /METHOD=ENTER MRN0xRace MRN0xSES0 MRN0xGender MRN0xParty0.
```

Regression

Notes

Output Created		15-DEC-2021 13:06:16
Comments		
Input	Data	C: \Users\njs5478\Dropbox\HM and COVID\0. Revise and Resubmit\2. R and R Data\Study 1a\Study1a_Data.sav
	Active Dataset	DataSet1
	Filter	Inclusion = 1 (FILTER)
	Weight	<none>
	Split File	<none>
	N of Rows in Working Data File	178
Missing Value Handling	Definition of Missing	User-defined missing values are treated as missing.
	Cases Used	Statistics are based on cases with no missing values for any variable used.
Syntax		REGRESSION /MISSING LISTWISE /STATISTICS COEFF OUTS R ANOVA CHANGE ZPP /CRITERIA=PIN(.05) POUT(.10) /NOORIGIN /DEPENDENT McConnellX /METHOD=ENTER Party0 /METHOD=ENTER GenderCC RaceCC SES0 /METHOD=ENTER MRN0 /METHOD=ENTER MRN0xRace MRN0xSES0 MRN0xGender MRN0xParty0.
Resources	Processor Time	00:00:00.02
	Elapsed Time	00:00:00.02

Notes

	Memory Required	43600 bytes
	Additional Memory Required for Residual Plots	0 bytes

Variables Entered/Removed[a]

Model	Variables Entered	Variables Removed	Method
1	Party0[b]	.	Enter
2	SES0, RaceCC, GenderCC[b]	.	Enter
3	MRN0[b]	.	Enter
4	MRN0xSES0, MRN0xGender, MRN0xParty0, MRN0xRace[b]	.	Enter

a. Dependent Variable: McConnellX

b. All requested variables entered.

Model Summary

Model	R	R Square	Adjusted R Square	Std. Error of the Estimate	Change Statistics		
					R Square Change	F Change	df1
1	.499[a]	.249	.244	1.14498	.249	42.535	1
2	.514[b]	.264	.241	1.14721	.015	.835	3
3	.538[c]	.290	.261	1.13167	.026	4.455	1
4	.575[d]	.330	.280	1.11721	.040	1.808	4

Model Summary

Model	Change Statistics	
	df2	Sig. F Change
1	128	.000
2	125	.477
3	124	.037
4	120	.132

a. Predictors: (Constant), Party0

b. Predictors: (Constant), Party0, SES0, RaceCC, GenderCC

c. Predictors: (Constant), Party0, SES0, RaceCC, GenderCC, MRN0

d. Predictors: (Constant), Party0, SES0, RaceCC, GenderCC, MRN0, MRN0xSES0, MRN0xGender, MRN0xParty0, MRN0xRace

ANOVA[a]

Model		Sum of Squares	df	Mean Square	F	Sig.
1	Regression	55.763	1	55.763	42.535	.000[b]
	Residual	167.806	128	1.311		
	Total	223.569	129			
2	Regression	59.058	4	14.765	11.219	.000[c]
	Residual	164.511	125	1.316		
	Total	223.569	129			
3	Regression	64.764	5	12.953	10.114	.000[d]
	Residual	158.805	124	1.281		
	Total	223.569	129			
4	Regression	73.791	9	8.199	6.569	.000[e]
	Residual	149.778	120	1.248		
	Total	223.569	129			

a. Dependent Variable: McConnellX

b. Predictors: (Constant), Party0

c. Predictors: (Constant), Party0, SES0, RaceCC, GenderCC

d. Predictors: (Constant), Party0, SES0, RaceCC, GenderCC, MRN0

e. Predictors: (Constant), Party0, SES0, RaceCC, GenderCC, MRN0, MRN0xSES0, MRN0xGender, MRN0xParty0, MRN0xRace

Coefficients[a]

Model		Unstandardized Coefficients B	Std. Error	Standardized Coefficients Beta	t	Sig.
1	(Constant)	3.446	.101		34.277	.000
	Party0	.473	.072	.499	6.522	.000
2	(Constant)	3.397	.126		27.019	.000
	Party0	.471	.075	.498	6.253	.000
	GenderCC	-.107	.104	-.081	-1.026	.307
	RaceCC	.092	.126	.057	.727	.468
	SES0	.130	.131	.077	.990	.324
3	(Constant)	3.382	.124		27.227	.000
	Party0	.368	.089	.389	4.136	.000
	GenderCC	-.210	.114	-.160	-1.848	.067
	RaceCC	.096	.125	.059	.770	.443
	SES0	.115	.130	.068	.882	.379
	MRN0	.324	.153	.218	2.111	.037
4	(Constant)	3.431	.139		24.699	.000
	Party0	.398	.089	.421	4.478	.000
	GenderCC	-.239	.113	-.182	-2.115	.036
	RaceCC	.107	.125	.066	.857	.393
	SES0	.089	.129	.053	.689	.492
	MRN0	.459	.184	.309	2.500	.014
	MRN0xRace	-.277	.151	-.186	-1.841	.068
	MRN0xSES0	.134	.149	.073	.904	.368
	MRN0xGender	.093	.143	.055	.653	.515
	MRN0xParty0	-.133	.094	-.125	-1.420	.158

Coefficients[a]

Model		Correlations Zero-order	Partial	Part
1	(Constant)			
	Party0	.499	.499	.499
2	(Constant)			
	Party0	.499	.488	.480
	GenderCC	.025	-.091	-.079
	RaceCC	.148	.065	.056
	SES0	.120	.088	.076
3	(Constant)			
	Party0	.499	.348	.313
	GenderCC	.025	-.164	-.140
	RaceCC	.148	.069	.058
	SES0	.120	.079	.067
	MRN0	.380	.186	.160
4	(Constant)			
	Party0	.499	.378	.335
	GenderCC	.025	-.190	-.158
	RaceCC	.148	.078	.064
	SES0	.120	.063	.051
	MRN0	.380	.222	.187
	MRN0xRace	.127	-.166	-.138
	MRN0xSES0	.020	.082	.068
	MRN0xGender	-.040	.059	.049
	MRN0xParty0	-.124	-.129	-.106

a. Dependent Variable: McConnellX

Excluded Variables[a]

Model		Beta In	t	Sig.	Partial Correlation	Collinearity Statistics Tolerance
1	GenderCC	-.074[b]	-.943	.347	-.083	.963
	RaceCC	.067[b]	.867	.387	.077	.973
	SES0	.068[b]	.886	.377	.078	.989
	MRN0	.139[b]	1.498	.137	.132	.672
	MRN0xRace	-.080[b]	-.957	.340	-.085	.849
	MRN0xSES0	.031[b]	.410	.683	.036	1.000
	MRN0xGender	.004[b]	.049	.961	.004	.992
	MRN0xParty0	-.126[b]	-1.662	.099	-.146	1.000
2	MRN0	.218[c]	2.111	.037	.186	.538
	MRN0xRace	-.061[c]	-.714	.477	-.064	.822
	MRN0xSES0	.039[c]	.500	.618	.045	.991
	MRN0xGender	.007[c]	.096	.923	.009	.986
	MRN0xParty0	-.140[c]	-1.827	.070	-.162	.983
3	MRN0xRace	-.208[d]	-2.158	.033	-.191	.601
	MRN0xSES0	.028[d]	.365	.716	.033	.987
	MRN0xGender	.005[d]	.062	.951	.006	.986
	MRN0xParty0	-.123[d]	-1.603	.111	-.143	.969

a. Dependent Variable: McConnellX

b. Predictors in the Model: (Constant), Party0

c. Predictors in the Model: (Constant), Party0, SES0, RaceCC, GenderCC

d. Predictors in the Model: (Constant), Party0, SES0, RaceCC, GenderCC, MRN0

```
REGRESSION
 /MISSING LISTWISE
 /STATISTICS COEFF OUTS R ANOVA CHANGE ZPP
 /CRITERIA=PIN(.05) POUT(.10)
 /NOORIGIN
 /DEPENDENT McConnellX
 /METHOD=ENTER Ideology0
 /METHOD=ENTER GenderCC RaceCC SES0
 /METHOD=ENTER MRN0
 /METHOD=ENTER MRN0xRace MRN0xSES0 MRN0xGender MRN0xIdeology0.
```

Regression

Notes

Output Created		15-DEC-2021 13:06:16
Comments		
Input	Data	C: \Users\njs5478\Dropbox\HM and COVID\0. Revise and Resubmit\2. R and R Data\Study 1a\Study1a_Data.sav
	Active Dataset	DataSet1
	Filter	Inclusion = 1 (FILTER)
	Weight	<none>
	Split File	<none>
	N of Rows in Working Data File	178
Missing Value Handling	Definition of Missing	User-defined missing values are treated as missing.
	Cases Used	Statistics are based on cases with no missing values for any variable used.
Syntax		REGRESSION /MISSING LISTWISE /STATISTICS COEFF OUTS R ANOVA CHANGE ZPP /CRITERIA=PIN(.05) POUT(.10) /NOORIGIN /DEPENDENT McConnellX /METHOD=ENTER Ideology0 /METHOD=ENTER GenderCC RaceCC SES0 /METHOD=ENTER MRN0 /METHOD=ENTER MRN0xRace MRN0xSES0 MRN0xGender MRN0xIdeology0.
Resources	Processor Time	00:00:00.02
	Elapsed Time	00:00:00.02

Notes

	Memory Required	43600 bytes
	Additional Memory Required for Residual Plots	0 bytes

Variables Entered/Removed[a]

Model	Variables Entered	Variables Removed	Method
1	Ideology0[b]	.	Enter
2	SES0, RaceCC, GenderCC[b]	.	Enter
3	MRN0[b]	.	Enter
4	MRN0xGender, MRN0xSES0, MRN0xIdeology0, MRN0xRace[b]	.	Enter

a. Dependent Variable: McConnellX

b. All requested variables entered.

Model Summary

Model	R	R Square	Adjusted R Square	Std. Error of the Estimate	Change Statistics		
					R Square Change	F Change	df1
1	.467[a]	.218	.212	1.16897	.218	35.608	1
2	.495[b]	.245	.220	1.16233	.027	1.489	3
3	.518[c]	.268	.239	1.14869	.024	3.986	1
4	.566[d]	.320	.269	1.12569	.052	2.280	4

Model Summary

Model	Change Statistics	
	df2	Sig. F Change
1	128	.000
2	125	.221
3	124	.048
4	120	.065

a. Predictors: (Constant), Ideology0

b. Predictors: (Constant), Ideology0, SES0, RaceCC, GenderCC

c. Predictors: (Constant), Ideology0, SES0, RaceCC, GenderCC, MRN0

d. Predictors: (Constant), Ideology0, SES0, RaceCC, GenderCC, MRN0, MRN0xGender, MRN0xSES0, MRN0xIdeology0, MRN0xRace

ANOVA[a]

Model		Sum of Squares	df	Mean Square	F	Sig.
1	Regression	48.659	1	48.659	35.608	.000[b]
	Residual	174.911	128	1.366		
	Total	223.569	129			
2	Regression	54.693	4	13.673	10.121	.000[c]
	Residual	168.877	125	1.351		
	Total	223.569	129			
3	Regression	59.953	5	11.991	9.087	.000[d]
	Residual	163.617	124	1.319		
	Total	223.569	129			
4	Regression	71.508	9	7.945	6.270	.000[e]
	Residual	152.061	120	1.267		
	Total	223.569	129			

a. Dependent Variable: McConnellX

b. Predictors: (Constant), Ideology0

c. Predictors: (Constant), Ideology0, SES0, RaceCC, GenderCC

d. Predictors: (Constant), Ideology0, SES0, RaceCC, GenderCC, MRN0

e. Predictors: (Constant), Ideology0, SES0, RaceCC, GenderCC, MRN0, MRN0xGender, MRN0xSES0, MRN0xIdeology0, MRN0xRace

Coefficients[a]

Model		Unstandardized Coefficients B	Unstandardized Coefficients Std. Error	Standardized Coefficients Beta	t	Sig.
1	(Constant)	3.427	.103		33.417	.000
	Ideology0	.366	.061	.467	5.967	.000
2	(Constant)	3.399	.128		26.647	.000
	Ideology0	.392	.066	.500	5.904	.000
	GenderCC	-.201	.110	-.153	-1.826	.070
	RaceCC	.063	.129	.039	.484	.629
	SES0	.134	.133	.080	1.008	.315
3	(Constant)	3.380	.126		26.743	.000
	Ideology0	.295	.082	.376	3.600	.000
	GenderCC	-.277	.115	-.211	-2.407	.018
	RaceCC	.078	.128	.048	.610	.543
	SES0	.119	.132	.071	.904	.368
	MRN0	.324	.162	.218	1.997	.048
4	(Constant)	3.441	.139		24.825	.000
	Ideology0	.327	.081	.417	4.027	.000
	GenderCC	-.309	.114	-.235	-2.714	.008
	RaceCC	.099	.129	.061	.771	.442
	SES0	.111	.130	.066	.852	.396
	MRN0	.448	.187	.301	2.392	.018
	MRN0xRace	-.284	.154	-.190	-1.840	.068
	MRN0xSES0	.093	.150	.051	.622	.535
	MRN0xGender	.161	.159	.095	1.012	.313
	MRN0xIdeology0	-.140	.078	-.178	-1.788	.076

Coefficients[a]

Model		Correlations		
		Zero-order	Partial	Part
1	(Constant)			
	Ideology0	.467	.467	.467
2	(Constant)			
	Ideology0	.467	.467	.459
	GenderCC	.025	-.161	-.142
	RaceCC	.148	.043	.038
	SES0	.120	.090	.078
3	(Constant)			
	Ideology0	.467	.308	.277
	GenderCC	.025	-.211	-.185
	RaceCC	.148	.055	.047
	SES0	.120	.081	.069
	MRN0	.380	.176	.153
4	(Constant)			
	Ideology0	.467	.345	.303
	GenderCC	.025	-.240	-.204
	RaceCC	.148	.070	.058
	SES0	.120	.077	.064
	MRN0	.380	.213	.180
	MRN0xRace	.127	-.166	-.138
	MRN0xSES0	.020	.057	.047
	MRN0xGender	-.040	.092	.076
	MRN0xIdeology0	-.150	-.161	-.135

a. Dependent Variable: McConnellX

Excluded Variables[a]

Model		Beta In	t	Sig.	Partial Correlation	Collinearity Statistics Tolerance
1	GenderCC	-.146[b]	-1.778	.078	-.156	.889
	RaceCC	.060[b]	.746	.457	.066	.962
	SES0	.062[b]	.783	.435	.069	.984
	MRN0	.129[b]	1.245	.215	.110	.568
	MRN0xRace	-.119[b]	-1.349	.180	-.119	.778
	MRN0xSES0	-.005[b]	-.066	.948	-.006	.997
	MRN0xGender	-.018[b]	-.227	.821	-.020	.998
	MRN0xIdeology0	-.148[b]	-1.908	.059	-.167	1.000
2	MRN0	.218[c]	1.997	.048	.176	.496
	MRN0xRace	-.100[c]	-1.122	.264	-.100	.763
	MRN0xSES0	-.001[c]	-.012	.991	-.001	.985
	MRN0xGender	-.013[c]	-.162	.871	-.015	.991
	MRN0xIdeology0	-.168[c]	-2.171	.032	-.191	.984
3	MRN0xRace	-.235[d]	-2.402	.018	-.212	.592
	MRN0xSES0	-.002[d]	-.027	.979	-.002	.985
	MRN0xGender	-.012[d]	-.150	.881	-.014	.991
	MRN0xIdeology0	-.152[d]	-1.975	.051	-.175	.972

a. Dependent Variable: McConnellX

b. Predictors in the Model: (Constant), Ideology0

c. Predictors in the Model: (Constant), Ideology0, SES0, RaceCC, GenderCC

d. Predictors in the Model: (Constant), Ideology0, SES0, RaceCC, GenderCC, MRN0

```
REGRESSION
 /MISSING LISTWISE
 /STATISTICS COEFF OUTS R ANOVA CHANGE ZPP
 /CRITERIA=PIN(.05) POUT(.10)
 /NOORIGIN
 /DEPENDENT FauciX
 /METHOD=ENTER Party0
 /METHOD=ENTER GenderCC RaceCC SES0
 /METHOD=ENTER MRN0
 /METHOD=ENTER MRN0xRace MRN0xSES0 MRN0xGender MRN0xParty0.
```

Regression

Notes

Output Created		15-DEC-2021 13:06:16
Comments		
Input	Data	C:\Users\njs5478\Dropbox\HM and COVID\0. Revise and Resubmit\2. R and R Data\Study 1a\Study1a_Data.sav
	Active Dataset	DataSet1
	Filter	Inclusion = 1 (FILTER)
	Weight	<none>
	Split File	<none>
	N of Rows in Working Data File	178
Missing Value Handling	Definition of Missing	User-defined missing values are treated as missing.
	Cases Used	Statistics are based on cases with no missing values for any variable used.
Syntax		REGRESSION /MISSING LISTWISE /STATISTICS COEFF OUTS R ANOVA CHANGE ZPP /CRITERIA=PIN(.05) POUT(.10) /NOORIGIN /DEPENDENT FauciX /METHOD=ENTER Party0 /METHOD=ENTER GenderCC RaceCC SES0 /METHOD=ENTER MRN0 /METHOD=ENTER MRN0xRace MRN0xSES0 MRN0xGender MRN0xParty0.
Resources	Processor Time	00:00:00.05
	Elapsed Time	00:00:00.03

Notes

	Memory Required	43600 bytes
	Additional Memory Required for Residual Plots	0 bytes

Variables Entered/Removed[a]

Model	Variables Entered	Variables Removed	Method
1	Party0[b]	.	Enter
2	SES0, RaceCC, GenderCC[b]	.	Enter
3	MRN0[b]	.	Enter
4	MRN0xGender, MRN0xSES0, MRN0xParty0, MRN0xRace[b]	.	Enter

a. Dependent Variable: FauciX

b. All requested variables entered.

Model Summary

Model	R	R Square	Adjusted R Square	Std. Error of the Estimate	Change Statistics		
					R Square Change	F Change	df1
1	.147[a]	.022	.015	1.53999	.022	3.241	1
2	.192[b]	.037	.010	1.54376	.015	.761	3
3	.192[c]	.037	.003	1.54914	.000	.001	1
4	.228[d]	.052	-.010	1.55898	.015	.550	4

Model Summary

Model	Change Statistics	
	df2	Sig. F Change
1	147	.074
2	144	.518
3	143	.970
4	139	.699

a. Predictors: (Constant), Party0

b. Predictors: (Constant), Party0, SES0, RaceCC, GenderCC

c. Predictors: (Constant), Party0, SES0, RaceCC, GenderCC, MRN0

d. Predictors: (Constant), Party0, SES0, RaceCC, GenderCC, MRN0, MRN0xGender, MRN0xSES0, MRN0xParty0, MRN0xRace

ANOVA[a]

Model		Sum of Squares	df	Mean Square	F	Sig.
1	Regression	7.687	1	7.687	3.241	.074[b]
	Residual	348.622	147	2.372		
	Total	356.309	148			
2	Regression	13.129	4	3.282	1.377	.245[c]
	Residual	343.180	144	2.383		
	Total	356.309	148			
3	Regression	13.132	5	2.626	1.094	.366[d]
	Residual	343.176	143	2.400		
	Total	356.309	148			
4	Regression	18.480	9	2.053	.845	.576[e]
	Residual	337.828	139	2.430		
	Total	356.309	148			

a. Dependent Variable: FauciX

b. Predictors: (Constant), Party0

c. Predictors: (Constant), Party0, SES0, RaceCC, GenderCC

d. Predictors: (Constant), Party0, SES0, RaceCC, GenderCC, MRN0

e. Predictors: (Constant), Party0, SES0, RaceCC, GenderCC, MRN0, MRN0xGender, MRN0xSES0, MRN0xParty0, MRN0xRace

Coefficients[a]

Model		Unstandardized Coefficients B	Std. Error	Standardized Coefficients Beta	t	Sig.
1	(Constant)	5.247	.126		41.563	.000
	Party0	-.168	.094	-.147	-1.800	.074
2	(Constant)	5.156	.160		32.321	.000
	Party0	-.206	.097	-.179	-2.111	.037
	GenderCC	.164	.130	.106	1.262	.209
	RaceCC	.147	.161	.076	.916	.361
	SES0	-.031	.156	-.016	-.196	.845
3	(Constant)	5.156	.161		32.026	.000
	Party0	-.203	.115	-.177	-1.764	.080
	GenderCC	.167	.143	.108	1.163	.247
	RaceCC	.147	.162	.076	.908	.365
	SES0	-.030	.158	-.016	-.191	.849
	MRN0	-.007	.191	-.004	-.038	.970
4	(Constant)	5.085	.179		28.380	.000
	Party0	-.218	.117	-.190	-1.861	.065
	GenderCC	.185	.145	.120	1.273	.205
	RaceCC	.134	.164	.069	.813	.418
	SES0	-.034	.161	-.018	-.213	.831
	MRN0	-.041	.251	-.023	-.164	.870
	MRN0xRace	.052	.216	.029	.238	.812
	MRN0xSES0	.096	.183	.046	.525	.600
	MRN0xGender	-.049	.178	-.025	-.273	.785
	MRN0xParty0	.133	.122	.104	1.090	.278

Coefficients[a]

Model		Correlations Zero-order	Partial	Part
1	(Constant)			
	Party0	-.147	-.147	-.147
2	(Constant)			
	Party0	-.147	-.173	-.173
	GenderCC	.068	.105	.103
	RaceCC	.040	.076	.075
	SES0	-.014	-.016	-.016
3	(Constant)			
	Party0	-.147	-.146	-.145
	GenderCC	.068	.097	.095
	RaceCC	.040	.076	.075
	SES0	-.014	-.016	-.016
	MRN0	-.054	-.003	-.003
4	(Constant)			
	Party0	-.147	-.156	-.154
	GenderCC	.068	.107	.105
	RaceCC	.040	.069	.067
	SES0	-.014	-.018	-.018
	MRN0	-.054	-.014	-.014
	MRN0xRace	-.011	.020	.020
	MRN0xSES0	.059	.044	.043
	MRN0xGender	.043	-.023	-.023
	MRN0xParty0	.098	.092	.090

a. Dependent Variable: FauciX

Excluded Variables[a]

Model		Beta In	t	Sig.	Partial Correlation	Collinearity Statistics Tolerance
1	GenderCC	.099[b]	1.193	.235	.098	.966
	RaceCC	.069[b]	.832	.406	.069	.965
	SES0	-.001[b]	-.010	.992	-.001	.993
	MRN0	.039[b]	.395	.694	.033	.700
	MRN0xRace	.061[b]	.679	.498	.056	.824
	MRN0xSES0	.069[b]	.845	.400	.070	.996
	MRN0xGender	.032[b]	.386	.700	.032	.994
	MRN0xParty0	.106[b]	1.297	.197	.107	.997
2	MRN0	-.004[c]	-.038	.970	-.003	.567
	MRN0xRace	.045[c]	.491	.624	.041	.788
	MRN0xSES0	.070[c]	.854	.394	.071	.988
	MRN0xGender	.026[c]	.317	.752	.026	.987
	MRN0xParty0	.112[c]	1.354	.178	.112	.973
3	MRN0xRace	.077[d]	.651	.516	.055	.489
	MRN0xSES0	.072[d]	.860	.391	.072	.976
	MRN0xGender	.026[d]	.315	.753	.026	.987
	MRN0xParty0	.112[d]	1.349	.179	.113	.969

a. Dependent Variable: FauciX

b. Predictors in the Model: (Constant), Party0

c. Predictors in the Model: (Constant), Party0, SES0, RaceCC, GenderCC

d. Predictors in the Model: (Constant), Party0, SES0, RaceCC, GenderCC, MRN0

```
REGRESSION
 /MISSING LISTWISE
 /STATISTICS COEFF OUTS R ANOVA CHANGE ZPP
 /CRITERIA=PIN(.05) POUT(.10)
 /NOORIGIN
 /DEPENDENT FauciX
 /METHOD=ENTER Ideology0
 /METHOD=ENTER GenderCC RaceCC SES0
 /METHOD=ENTER MRN0
 /METHOD=ENTER MRN0xRace MRN0xSES0 MRN0xGender MRN0xIdeology0.
```

Regression

Notes

Output Created		15-DEC-2021 13:06:16
Comments		
Input	Data	C: \Users\njs5478\Dropbox\HM and COVID\0. Revise and Resubmit\2. R and R Data\Study 1a\Study1a_Data.sav
	Active Dataset	DataSet1
	Filter	Inclusion = 1 (FILTER)
	Weight	<none>
	Split File	<none>
	N of Rows in Working Data File	178
Missing Value Handling	Definition of Missing	User-defined missing values are treated as missing.
	Cases Used	Statistics are based on cases with no missing values for any variable used.
Syntax		REGRESSION<br>/MISSING LISTWISE<br>/STATISTICS COEFF OUTS R ANOVA CHANGE ZPP<br>/CRITERIA=PIN(.05) POUT(.10)<br>/NOORIGIN<br>/DEPENDENT FauciX<br>/METHOD=ENTER Ideology0<br>/METHOD=ENTER GenderCC RaceCC SES0<br>/METHOD=ENTER MRN0<br>/METHOD=ENTER MRN0xRace MRN0xSES0 MRN0xGender MRN0xIdeology0.
Resources	Processor Time	00:00:00.02
	Elapsed Time	00:00:00.02

Notes

	Memory Required	43600 bytes
	Additional Memory Required for Residual Plots	0 bytes

Variables Entered/Removed[a]

Model	Variables Entered	Variables Removed	Method
1	Ideology0[b]	.	Enter
2	SES0, RaceCC, GenderCC[b]	.	Enter
3	MRN0[b]	.	Enter
4	MRN0xGender, MRN0xSES0, MRN0xIdeology0, MRN0xRace[b]	.	Enter

a. Dependent Variable: FauciX

b. All requested variables entered.

Model Summary

Model	R	R Square	Adjusted R Square	Std. Error of the Estimate	Change Statistics		
					R Square Change	F Change	df1
1	.134[a]	.018	.011	1.54281	.018	2.693	1
2	.192[b]	.037	.010	1.54384	.019	.934	3
3	.192[c]	.037	.003	1.54906	.000	.032	1
4	.218[d]	.048	-.014	1.56241	.011	.392	4

Model Summary

Model	Change Statistics	
	df2	Sig. F Change
1	147	.103
2	144	.426
3	143	.858
4	139	.814

a. Predictors: (Constant), Ideology0

b. Predictors: (Constant), Ideology0, SES0, RaceCC, GenderCC

c. Predictors: (Constant), Ideology0, SES0, RaceCC, GenderCC, MRN0

d. Predictors: (Constant), Ideology0, SES0, RaceCC, GenderCC, MRN0, MRN0xGender, MRN0xSES0, MRN0xIdeology0, MRN0xRace

ANOVA[a]

Model		Sum of Squares	df	Mean Square	F	Sig.
1	Regression	6.411	1	6.411	2.693	.103[b]
	Residual	349.898	147	2.380		
	Total	356.309	148			
2	Regression	13.092	4	3.273	1.373	.246[c]
	Residual	343.216	144	2.383		
	Total	356.309	148			
3	Regression	13.169	5	2.634	1.098	.364[d]
	Residual	343.140	143	2.400		
	Total	356.309	148			
4	Regression	16.994	9	1.888	.773	.641[e]
	Residual	339.315	139	2.441		
	Total	356.309	148			

a. Dependent Variable: FauciX

b. Predictors: (Constant), Ideology0

c. Predictors: (Constant), Ideology0, SES0, RaceCC, GenderCC

d. Predictors: (Constant), Ideology0, SES0, RaceCC, GenderCC, MRN0

e. Predictors: (Constant), Ideology0, SES0, RaceCC, GenderCC, MRN0, MRN0xGender, MRN0xSES0, MRN0xIdeology0, MRN0xRace

Coefficients[a]

Model		Unstandardized Coefficients B	Unstandardized Coefficients Std. Error	Standardized Coefficients Beta	t	Sig.
1	(Constant)	5.253	.126		41.560	.000
	Ideology0	-.128	.078	-.134	-1.641	.103
2	(Constant)	5.164	.159		32.475	.000
	Ideology0	-.176	.084	-.185	-2.107	.037
	GenderCC	.199	.134	.129	1.484	.140
	RaceCC	.145	.161	.075	.904	.367
	SES0	-.027	.157	-.014	-.173	.863
3	(Constant)	5.161	.161		32.140	.000
	Ideology0	-.188	.106	-.197	-1.768	.079
	GenderCC	.191	.143	.123	1.336	.184
	RaceCC	.148	.162	.077	.914	.362
	SES0	-.029	.158	-.016	-.186	.852
	MRN0	.037	.205	.021	.179	.858
4	(Constant)	5.109	.178		28.707	.000
	Ideology0	-.201	.108	-.211	-1.858	.065
	GenderCC	.202	.145	.130	1.391	.167
	RaceCC	.141	.167	.073	.848	.398
	SES0	-.026	.162	-.014	-.159	.874
	MRN0	-.038	.257	-.021	-.146	.884
	MRN0xRace	.110	.218	.063	.503	.615
	MRN0xSES0	.117	.185	.056	.631	.529
	MRN0xGender	-.024	.194	-.012	-.124	.902
	MRN0xIdeology0	.053	.101	.055	.522	.603

Coefficients[a]

Model		Correlations Zero-order	Partial	Part
1	(Constant)			
	Ideology0	-.134	-.134	-.134
2	(Constant)			
	Ideology0	-.134	-.173	-.172
	GenderCC	.068	.123	.121
	RaceCC	.040	.075	.074
	SES0	-.014	-.014	-.014
3	(Constant)			
	Ideology0	-.134	-.146	-.145
	GenderCC	.068	.111	.110
	RaceCC	.040	.076	.075
	SES0	-.014	-.016	-.015
	MRN0	-.054	.015	.015
4	(Constant)			
	Ideology0	-.134	-.156	-.154
	GenderCC	.068	.117	.115
	RaceCC	.040	.072	.070
	SES0	-.014	-.014	-.013
	MRN0	-.054	-.012	-.012
	MRN0xRace	-.011	.043	.042
	MRN0xSES0	.059	.053	.052
	MRN0xGender	.043	-.010	-.010
	MRN0xIdeology0	.076	.044	.043

a. Dependent Variable: FauciX

Excluded Variables[a]

Model		Beta In	t	Sig.	Partial Correlation	Collinearity Statistics Tolerance
1	GenderCC	.120[b]	1.406	.162	.116	.908
	RaceCC	.064[b]	.775	.440	.064	.971
	SES0	.002[b]	.019	.985	.002	.987
	MRN0	.061[b]	.562	.575	.046	.568
	MRN0xRace	.083[b]	.864	.389	.071	.719
	MRN0xSES0	.074[b]	.899	.370	.074	.990
	MRN0xGender	.038[b]	.457	.648	.038	.998
	MRN0xIdeology0	.077[b]	.945	.346	.078	1.000
2	MRN0	.021[c]	.179	.858	.015	.492
	MRN0xRace	.075[c]	.763	.447	.064	.704
	MRN0xSES0	.077[c]	.937	.350	.078	.981
	MRN0xGender	.031[c]	.380	.705	.032	.992
	MRN0xIdeology0	.075[c]	.906	.367	.076	.966
3	MRN0xRace	.094[d]	.798	.426	.067	.482
	MRN0xSES0	.077[d]	.923	.358	.077	.975
	MRN0xGender	.032[d]	.383	.703	.032	.991
	MRN0xIdeology0	.077[d]	.920	.359	.077	.959

a. Dependent Variable: FauciX

b. Predictors in the Model: (Constant), Ideology0

c. Predictors in the Model: (Constant), Ideology0, SES0, RaceCC, GenderCC

d. Predictors in the Model: (Constant), Ideology0, SES0, RaceCC, GenderCC, MRN0

```
REGRESSION
 /MISSING LISTWISE
 /STATISTICS COEFF OUTS R ANOVA CHANGE ZPP
 /CRITERIA=PIN(.05) POUT(.10)
 /NOORIGIN
 /DEPENDENT RepCongressX
 /METHOD=ENTER Party0
 /METHOD=ENTER GenderCC RaceCC SES0
 /METHOD=ENTER MRN0
 /METHOD=ENTER MRN0xRace MRN0xSES0 MRN0xGender MRN0xParty0.
```

Regression

Notes

Output Created		15-DEC-2021 13:06:16
Comments		
Input	Data	C:\Users\njs5478\Dropbox\HM and COVID\0. Revise and Resubmit\2. R and R Data\Study 1a\Study1a_Data.sav
	Active Dataset	DataSet1
	Filter	Inclusion = 1 (FILTER)
	Weight	<none>
	Split File	<none>
	N of Rows in Working Data File	178
Missing Value Handling	Definition of Missing	User-defined missing values are treated as missing.
	Cases Used	Statistics are based on cases with no missing values for any variable used.
Syntax		REGRESSION /MISSING LISTWISE /STATISTICS COEFF OUTS R ANOVA CHANGE ZPP /CRITERIA=PIN(.05) POUT(.10) /NOORIGIN /DEPENDENT RepCongressX /METHOD=ENTER Party0 /METHOD=ENTER GenderCC RaceCC SES0 /METHOD=ENTER MRN0 /METHOD=ENTER MRN0xRace MRN0xSES0 MRN0xGender MRN0xParty0.
Resources	Processor Time	00:00:00.03
	Elapsed Time	00:00:00.02

Notes

	Memory Required	43600 bytes
	Additional Memory Required for Residual Plots	0 bytes

Variables Entered/Removed[a]

Model	Variables Entered	Variables Removed	Method
1	Party0[b]	.	Enter
2	SES0, RaceCC, GenderCC[b]	.	Enter
3	MRN0[b]	.	Enter
4	MRN0xParty0 , MRN0xSES0, MRN0xGende r, ...[b]	.	Enter

a. Dependent Variable: RepCongressX

b. All requested variables entered.

Model Summary

Model	R	R Square	Adjusted R Square	Std. Error of the Estimate	Change Statistics		
					R Square Change	F Change	df1
1	.665[a]	.443	.439	1.22811	.443	110.416	1
2	.709[b]	.503	.488	1.17261	.060	5.490	3
3	.717[c]	.514	.496	1.16330	.011	3.187	1
4	.730[d]	.533	.501	1.15836	.018	1.289	4

Model Summary

Model	Change Statistics	
	df2	Sig. F Change
1	139	.000
2	136	.001
3	135	.076
4	131	.278

a. Predictors: (Constant), Party0

b. Predictors: (Constant), Party0, SES0, RaceCC, GenderCC

c. Predictors: (Constant), Party0, SES0, RaceCC, GenderCC, MRN0

d. Predictors: (Constant), Party0, SES0, RaceCC, GenderCC, MRN0, MRN0xParty0, MRN0xSES0, MRN0xGender, MRN0xRace

ANOVA[a]

Model		Sum of Squares	df	Mean Square	F	Sig.
1	Regression	166.536	1	166.536	110.416	.000[b]
	Residual	209.648	139	1.508		
	Total	376.184	140			
2	Regression	189.181	4	47.295	34.396	.000[c]
	Residual	187.003	136	1.375		
	Total	376.184	140			
3	Regression	193.494	5	38.699	28.597	.000[d]
	Residual	182.690	135	1.353		
	Total	376.184	140			
4	Regression	200.410	9	22.268	16.596	.000[e]
	Residual	175.775	131	1.342		
	Total	376.184	140			

a. Dependent Variable: RepCongressX

b. Predictors: (Constant), Party0

c. Predictors: (Constant), Party0, SES0, RaceCC, GenderCC

d. Predictors: (Constant), Party0, SES0, RaceCC, GenderCC, MRN0

e. Predictors: (Constant), Party0, SES0, RaceCC, GenderCC, MRN0, MRN0xParty0, MRN0xSES0, MRN0xGender, MRN0xRace

Coefficients[a]

Model		Unstandardized Coefficients B	Std. Error	Standardized Coefficients Beta	t	Sig.
1	(Constant)	3.781	.103		36.554	.000
	Party0	.796	.076	.665	10.508	.000
2	(Constant)	3.576	.124		28.784	.000
	Party0	.811	.074	.678	10.942	.000
	GenderCC	-.288	.101	-.176	-2.843	.005
	RaceCC	.337	.125	.164	2.693	.008
	SES0	.044	.125	.021	.353	.725
3	(Constant)	3.560	.124		28.812	.000
	Party0	.722	.089	.604	8.136	.000
	GenderCC	-.365	.109	-.224	-3.340	.001
	RaceCC	.341	.124	.166	2.748	.007
	SES0	.025	.124	.012	.205	.838
	MRN0	.263	.147	.144	1.785	.076
4	(Constant)	3.535	.140		25.297	.000
	Party0	.725	.089	.606	8.151	.000
	GenderCC	-.392	.110	-.240	-3.550	.001
	RaceCC	.361	.125	.176	2.897	.004
	SES0	.020	.124	.010	.158	.875
	MRN0	.393	.167	.216	2.354	.020
	MRN0xRace	-.223	.138	-.122	-1.621	.107
	MRN0xSES0	.273	.147	.117	1.859	.065
	MRN0xGender	.001	.134	.000	.007	.995
	MRN0xParty0	-.008	.082	-.007	-.102	.919

Coefficients[a]

Model		Correlations Zero-order	Partial	Part
1	(Constant)			
	Party0	.665	.665	.665
2	(Constant)			
	Party0	.665	.684	.662
	GenderCC	-.057	-.237	-.172
	RaceCC	.249	.225	.163
	SES0	.042	.030	.021
3	(Constant)			
	Party0	.665	.574	.488
	GenderCC	-.057	-.276	-.200
	RaceCC	.249	.230	.165
	SES0	.042	.018	.012
	MRN0	.403	.152	.107
4	(Constant)			
	Party0	.665	.580	.487
	GenderCC	-.057	-.296	-.212
	RaceCC	.249	.245	.173
	SES0	.042	.014	.009
	MRN0	.403	.201	.141
	MRN0xRace	.208	-.140	-.097
	MRN0xSES0	.089	.160	.111
	MRN0xGender	-.066	.001	.000
	MRN0xParty0	.056	-.009	-.006

a. Dependent Variable: RepCongressX

Excluded Variables[a]

Model		Beta In	t	Sig.	Partial Correlation	Collinearity Statistics Tolerance
1	GenderCC	-.184[b]	-2.934	.004	-.242	.967
	RaceCC	.176[b]	2.833	.005	.234	.987
	SES0	.012[b]	.187	.852	.016	.998
	MRN0	.029[b]	.370	.712	.031	.668
	MRN0xRace	-.043[b]	-.632	.529	-.054	.864
	MRN0xSES0	.068[b]	1.079	.283	.091	.999
	MRN0xGender	-.013[b]	-.200	.842	-.017	.994
	MRN0xParty0	.011[b]	.174	.862	.015	.995
2	MRN0	.144[c]	1.785	.076	.152	.553
	MRN0xRace	-.017[c]	-.252	.801	-.022	.850
	MRN0xSES0	.094[c]	1.559	.121	.133	.987
	MRN0xGender	.017[c]	.275	.784	.024	.973
	MRN0xParty0	-.006[c]	-.102	.919	-.009	.988
3	MRN0xRace	-.091[d]	-1.244	.216	-.107	.662
	MRN0xSES0	.094[d]	1.574	.118	.135	.987
	MRN0xGender	.020[d]	.329	.742	.028	.972
	MRN0xParty0	-.006[d]	-.103	.918	-.009	.988

a. Dependent Variable: RepCongressX

b. Predictors in the Model: (Constant), Party0

c. Predictors in the Model: (Constant), Party0, SES0, RaceCC, GenderCC

d. Predictors in the Model: (Constant), Party0, SES0, RaceCC, GenderCC, MRN0

```
REGRESSION
 /MISSING LISTWISE
 /STATISTICS COEFF OUTS R ANOVA CHANGE ZPP
 /CRITERIA=PIN(.05) POUT(.10)
 /NOORIGIN
 /DEPENDENT RepCongressX
 /METHOD=ENTER Ideology0
 /METHOD=ENTER GenderCC RaceCC SES0
 /METHOD=ENTER MRN0
 /METHOD=ENTER MRN0xRace MRN0xSES0 MRN0xGender MRN0xIdeology0.
```

Regression

Notes

Output Created		15-DEC-2021 13:06:16
Comments		
Input	Data	C: \Users\njs5478\Dropbox\HM and COVID\0. Revise and Resubmit\2. R and R Data\Study 1a\Study1a_Data.sav
	Active Dataset	DataSet1
	Filter	Inclusion = 1 (FILTER)
	Weight	<none>
	Split File	<none>
	N of Rows in Working Data File	178
Missing Value Handling	Definition of Missing	User-defined missing values are treated as missing.
	Cases Used	Statistics are based on cases with no missing values for any variable used.
Syntax		REGRESSION /MISSING LISTWISE /STATISTICS COEFF OUTS R ANOVA CHANGE ZPP /CRITERIA=PIN(.05) POUT(.10) /NOORIGIN /DEPENDENT RepCongressX /METHOD=ENTER Ideology0 /METHOD=ENTER GenderCC RaceCC SES0 /METHOD=ENTER MRN0 /METHOD=ENTER MRN0xRace MRN0xSES0 MRN0xGender MRN0xIdeology0.
Resources	Processor Time	00:00:00.03
	Elapsed Time	00:00:00.03

Notes

	Memory Required	43600 bytes
	Additional Memory Required for Residual Plots	0 bytes

Variables Entered/Removed[a]

Model	Variables Entered	Variables Removed	Method
1	Ideology0[b]	.	Enter
2	SES0, RaceCC, GenderCC[b]	.	Enter
3	MRN0[b]	.	Enter
4	MRN0xIdeology0, MRN0xSES0, MRN0xGender, MRN0xRace[b]	.	Enter

a. Dependent Variable: RepCongressX

b. All requested variables entered.

Model Summary

Model	R	R Square	Adjusted R Square	Std. Error of the Estimate	Change Statistics		
					R Square Change	F Change	df1
1	.623[a]	.388	.384	1.28653	.388	88.280	1
2	.690[b]	.476	.461	1.20376	.088	7.591	3
3	.694[c]	.482	.463	1.20128	.006	1.562	1
4	.712[d]	.506	.472	1.19059	.024	1.609	4

Model Summary

Model	Change Statistics	
	df2	Sig. F Change
1	139	.000
2	136	.000
3	135	.214
4	131	.176

a. Predictors: (Constant), Ideology0

b. Predictors: (Constant), Ideology0, SES0, RaceCC, GenderCC

c. Predictors: (Constant), Ideology0, SES0, RaceCC, GenderCC, MRN0

d. Predictors: (Constant), Ideology0, SES0, RaceCC, GenderCC, MRN0, MRN0xIdeology0, MRN0xSES0, MRN0xGender, MRN0xRace

ANOVA[a]

Model		Sum of Squares	df	Mean Square	F	Sig.
1	Regression	146.117	1	146.117	88.280	.000[b]
	Residual	230.067	139	1.655		
	Total	376.184	140			
2	Regression	179.117	4	44.779	30.903	.000[c]
	Residual	197.068	136	1.449		
	Total	376.184	140			
3	Regression	181.370	5	36.274	25.137	.000[d]
	Residual	194.814	135	1.443		
	Total	376.184	140			
4	Regression	190.493	9	21.166	14.932	.000[e]
	Residual	185.692	131	1.417		
	Total	376.184	140			

a. Dependent Variable: RepCongressX

b. Predictors: (Constant), Ideology0

c. Predictors: (Constant), Ideology0, SES0, RaceCC, GenderCC

d. Predictors: (Constant), Ideology0, SES0, RaceCC, GenderCC, MRN0

e. Predictors: (Constant), Ideology0, SES0, RaceCC, GenderCC, MRN0, MRN0xIdeology0, MRN0xSES0, MRN0xGender, MRN0xRace

Coefficients[a]

Model		Unstandardized Coefficients B	Unstandardized Coefficients Std. Error	Standardized Coefficients Beta	t	Sig.
1	(Constant)	3.766	.108		34.756	.000
	Ideology0	.603	.064	.623	9.396	.000
2	(Constant)	3.584	.128		28.085	.000
	Ideology0	.661	.064	.683	10.328	.000
	GenderCC	-.424	.107	-.259	-3.951	.000
	RaceCC	.294	.129	.144	2.281	.024
	SES0	-.019	.128	-.009	-.149	.882
3	(Constant)	3.570	.128		27.924	.000
	Ideology0	.598	.082	.618	7.327	.000
	GenderCC	-.468	.113	-.286	-4.152	.000
	RaceCC	.303	.129	.148	2.353	.020
	SES0	-.027	.128	-.013	-.210	.834
	MRN0	.201	.161	.110	1.250	.214
4	(Constant)	3.542	.143		24.778	.000
	Ideology0	.606	.082	.627	7.432	.000
	GenderCC	-.490	.113	-.300	-4.345	.000
	RaceCC	.306	.130	.149	2.357	.020
	SES0	-.039	.128	-.019	-.308	.759
	MRN0	.368	.177	.202	2.085	.039
	MRN0xRace	-.310	.144	-.170	-2.156	.033
	MRN0xSES0	.256	.151	.110	1.694	.093
	MRN0xGender	-.087	.145	-.043	-.600	.550
	MRN0xIdeology0	.046	.067	.049	.687	.494

Coefficients[a]

Model		Correlations		
		Zero-order	Partial	Part
1	(Constant)			
	Ideology0	.623	.623	.623
2	(Constant)			
	Ideology0	.623	.663	.641
	GenderCC	-.057	-.321	-.245
	RaceCC	.249	.192	.142
	SES0	.042	-.013	-.009
3	(Constant)			
	Ideology0	.623	.533	.454
	GenderCC	-.057	-.336	-.257
	RaceCC	.249	.199	.146
	SES0	.042	-.018	-.013
	MRN0	.403	.107	.077
4	(Constant)			
	Ideology0	.623	.545	.456
	GenderCC	-.057	-.355	-.267
	RaceCC	.249	.202	.145
	SES0	.042	-.027	-.019
	MRN0	.403	.179	.128
	MRN0xRace	.208	-.185	-.132
	MRN0xSES0	.089	.146	.104
	MRN0xGender	-.066	-.052	-.037
	MRN0xIdeology0	.070	.060	.042

a. Dependent Variable: RepCongressX

Excluded Variables[a]

Model		Beta In	t	Sig.	Partial Correlation	Collinearity Statistics Tolerance
1	GenderCC	-.273[b]	-4.144	.000	-.333	.907
	RaceCC	.165[b]	2.508	.013	.209	.981
	SES0	-.024[b]	-.355	.723	-.030	.989
	MRN0	-.024[b]	-.271	.787	-.023	.554
	MRN0xRace	-.093[b]	-1.254	.212	-.106	.795
	MRN0xSES0	.051[b]	.772	.441	.066	.996
	MRN0xGender	-.039[b]	-.587	.558	-.050	.998
	MRN0xIdeology0	.038[b]	.576	.566	.049	.997
2	MRN0	.110[c]	1.250	.214	.107	.494
	MRN0xRace	-.073[c]	-1.051	.295	-.090	.790
	MRN0xSES0	.076[c]	1.218	.225	.104	.984
	MRN0xGender	-.003[c]	-.055	.956	-.005	.977
	MRN0xIdeology0	.022[c]	.348	.729	.030	.991
3	MRN0xRace	-.132[d]	-1.741	.084	-.149	.654
	MRN0xSES0	.078[d]	1.256	.211	.108	.984
	MRN0xGender	.000[d]	.004	.997	.000	.974
	MRN0xIdeology0	.023[d]	.372	.710	.032	.990

a. Dependent Variable: RepCongressX

b. Predictors in the Model: (Constant), Ideology0

c. Predictors in the Model: (Constant), Ideology0, SES0, RaceCC, GenderCC

d. Predictors in the Model: (Constant), Ideology0, SES0, RaceCC, GenderCC, MRN0

```
REGRESSION
 /MISSING LISTWISE
 /STATISTICS COEFF OUTS R ANOVA CHANGE ZPP
 /CRITERIA=PIN(.05) POUT(.10)
 /NOORIGIN
 /DEPENDENT DemCongressX
 /METHOD=ENTER Party0
 /METHOD=ENTER GenderCC RaceCC SES0
 /METHOD=ENTER MRN0
 /METHOD=ENTER MRN0xRace MRN0xSES0 MRN0xGender MRN0xParty0.
```

Regression

Notes

Output Created		15-DEC-2021 13:06:16
Comments		
Input	Data	C: \Users\njs5478\Dropbox\HM and COVID\0. Revise and Resubmit\2. R and R Data\Study 1a\Study1a_Data.sav
	Active Dataset	DataSet1
	Filter	Inclusion = 1 (FILTER)
	Weight	<none>
	Split File	<none>
	N of Rows in Working Data File	178
Missing Value Handling	Definition of Missing	User-defined missing values are treated as missing.
	Cases Used	Statistics are based on cases with no missing values for any variable used.
Syntax		REGRESSION<br>/MISSING LISTWISE<br>/STATISTICS COEFF OUTS R ANOVA CHANGE ZPP<br>/CRITERIA=PIN(.05) POUT(.10)<br>/NOORIGIN<br>/DEPENDENT DemCongressX<br>/METHOD=ENTER Party0<br>/METHOD=ENTER GenderCC RaceCC SES0<br>/METHOD=ENTER MRN0<br>/METHOD=ENTER MRN0xRace MRN0xSES0 MRN0xGender MRN0xParty0.
Resources	Processor Time	00:00:00.03
	Elapsed Time	00:00:00.03

Notes

	Memory Required	43600 bytes
	Additional Memory Required for Residual Plots	0 bytes

Variables Entered/Removed[a]

Model	Variables Entered	Variables Removed	Method
1	Party0[b]	.	Enter
2	SES0, RaceCC, GenderCC[b]	.	Enter
3	MRN0[b]	.	Enter
4	MRN0xParty0 , MRN0xSES0, MRN0xGender, ...[b]	.	Enter

a. Dependent Variable: DemCongressX

b. All requested variables entered.

Model Summary

Model	R	R Square	Adjusted R Square	Std. Error of the Estimate	Change Statistics		
					R Square Change	F Change	df1
1	.426[a]	.181	.176	1.37057	.181	30.808	1
2	.430[b]	.185	.161	1.38277	.003	.186	3
3	.431[c]	.186	.156	1.38701	.001	.168	1
4	.474[d]	.225	.171	1.37401	.039	1.642	4

Model Summary

Model	Change Statistics	
	df2	Sig. F Change
1	139	.000
2	136	.905
3	135	.682
4	131	.168

a. Predictors: (Constant), Party0

b. Predictors: (Constant), Party0, SES0, RaceCC, GenderCC

c. Predictors: (Constant), Party0, SES0, RaceCC, GenderCC, MRN0

d. Predictors: (Constant), Party0, SES0, RaceCC, GenderCC, MRN0, MRN0xParty0, MRN0xSES0, MRN0xGender, MRN0xRace

ANOVA[a]

Model		Sum of Squares	df	Mean Square	F	Sig.
1	Regression	57.872	1	57.872	30.808	.000[b]
	Residual	261.107	139	1.878		
	Total	318.979	140			
2	Regression	58.941	4	14.735	7.707	.000[c]
	Residual	260.038	136	1.912		
	Total	318.979	140			
3	Regression	59.265	5	11.853	6.161	.000[d]
	Residual	259.714	135	1.924		
	Total	318.979	140			
4	Regression	71.664	9	7.963	4.218	.000[e]
	Residual	247.315	131	1.888		
	Total	318.979	140			

a. Dependent Variable: DemCongressX

b. Predictors: (Constant), Party0

c. Predictors: (Constant), Party0, SES0, RaceCC, GenderCC

d. Predictors: (Constant), Party0, SES0, RaceCC, GenderCC, MRN0

e. Predictors: (Constant), Party0, SES0, RaceCC, GenderCC, MRN0, MRN0xParty0, MRN0xSES0, MRN0xGender, MRN0xRace

Coefficients[a]

Model		Unstandardized Coefficients B	Unstandardized Coefficients Std. Error	Standardized Coefficients Beta	t	Sig.
1	(Constant)	4.095	.115		35.473	.000
	Party0	-.471	.085	-.426	-5.550	.000
2	(Constant)	4.046	.146		27.629	.000
	Party0	-.470	.088	-.424	-5.349	.000
	GenderCC	-.054	.119	-.036	-.450	.654
	RaceCC	.082	.148	.043	.553	.581
	SES0	-.017	.147	-.009	-.118	.906
3	(Constant)	4.051	.147		27.496	.000
	Party0	-.445	.107	-.402	-4.171	.000
	GenderCC	-.032	.131	-.021	-.242	.809
	RaceCC	.081	.148	.043	.545	.587
	SES0	-.012	.148	-.006	-.078	.938
	MRN0	-.071	.172	-.043	-.410	.682
4	(Constant)	3.979	.165		24.051	.000
	Party0	-.430	.106	-.389	-4.047	.000
	GenderCC	-.061	.131	-.041	-.466	.642
	RaceCC	.092	.148	.049	.623	.534
	SES0	-.048	.147	-.026	-.327	.744
	MRN0	.153	.197	.093	.776	.439
	MRN0xRace	-.373	.163	-.228	-2.292	.024
	MRN0xSES0	-.010	.174	-.005	-.056	.956
	MRN0xGender	.132	.157	.073	.843	.401
	MRN0xParty0	.034	.098	.029	.349	.727

Coefficients[a]

Model		Correlations Zero-order	Partial	Part
1	(Constant)			
	Party0	-.426	-.426	-.426
2	(Constant)			
	Party0	-.426	-.417	-.414
	GenderCC	-.114	-.039	-.035
	RaceCC	-.007	.047	.043
	SES0	-.030	-.010	-.009
3	(Constant)			
	Party0	-.426	-.338	-.324
	GenderCC	-.114	-.021	-.019
	RaceCC	-.007	.047	.042
	SES0	-.030	-.007	-.006
	MRN0	-.284	-.035	-.032
4	(Constant)			
	Party0	-.426	-.333	-.311
	GenderCC	-.114	-.041	-.036
	RaceCC	-.007	.054	.048
	SES0	-.030	-.029	-.025
	MRN0	-.284	.068	.060
	MRN0xRace	-.321	-.196	-.176
	MRN0xSES0	-.052	-.005	-.004
	MRN0xGender	.093	.073	.065
	MRN0xParty0	.010	.030	.027

a. Dependent Variable: DemCongressX

Excluded Variables[a]

Model		Beta In	t	Sig.	Partial Correlation	Collinearity Statistics Tolerance
1	GenderCC	-.039[b]	-.499	.618	-.042	.968
	RaceCC	.045[b]	.577	.565	.049	.985
	SES0	-.011[b]	-.137	.891	-.012	.998
	MRN0	-.056[b]	-.599	.550	-.051	.665
	MRN0xRace	-.188[b]	-2.302	.023	-.192	.859
	MRN0xSES0	-.035[b]	-.453	.651	-.039	.998
	MRN0xGender	.065[b]	.842	.401	.071	.995
	MRN0xParty0	.041[b]	.531	.597	.045	.995
2	MRN0	-.043[c]	-.410	.682	-.035	.544
	MRN0xRace	-.185[c]	-2.229	.027	-.188	.843
	MRN0xSES0	-.030[c]	-.378	.706	-.033	.987
	MRN0xGender	.074[c]	.947	.345	.081	.974
	MRN0xParty0	.038[c]	.487	.627	.042	.987
3	MRN0xRace	-.222[d]	-2.321	.022	-.197	.639
	MRN0xSES0	-.029[d]	-.364	.716	-.031	.986
	MRN0xGender	.075[d]	.952	.343	.082	.973
	MRN0xParty0	.039[d]	.498	.619	.043	.987

a. Dependent Variable: DemCongressX

b. Predictors in the Model: (Constant), Party0

c. Predictors in the Model: (Constant), Party0, SES0, RaceCC, GenderCC

d. Predictors in the Model: (Constant), Party0, SES0, RaceCC, GenderCC, MRN0

```
REGRESSION
 /MISSING LISTWISE
 /STATISTICS COEFF OUTS R ANOVA CHANGE ZPP
 /CRITERIA=PIN(.05) POUT(.10)
 /NOORIGIN
 /DEPENDENT DemCongressX
 /METHOD=ENTER Ideology0
 /METHOD=ENTER GenderCC RaceCC SES0
 /METHOD=ENTER MRN0
 /METHOD=ENTER MRN0xRace MRN0xSES0 MRN0xGender MRN0xIdeology0.
```

Regression

Notes

Output Created		15-DEC-2021 13:06:16
Comments		
Input	Data	C:\Users\njs5478\Dropbox\HM and COVID\0. Revise and Resubmit\2. R and R Data\Study 1a\Study1a_Data.sav
	Active Dataset	DataSet1
	Filter	Inclusion = 1 (FILTER)
	Weight	<none>
	Split File	<none>
	N of Rows in Working Data File	178
Missing Value Handling	Definition of Missing	User-defined missing values are treated as missing.
	Cases Used	Statistics are based on cases with no missing values for any variable used.
Syntax		REGRESSION /MISSING LISTWISE /STATISTICS COEFF OUTS R ANOVA CHANGE ZPP /CRITERIA=PIN(.05) POUT(.10) /NOORIGIN /DEPENDENT DemCongressX /METHOD=ENTER Ideology0 /METHOD=ENTER GenderCC RaceCC SES0 /METHOD=ENTER MRN0 /METHOD=ENTER MRN0xRace MRN0xSES0 MRN0xGender MRN0xIdeology0.
Resources	Processor Time	00:00:00.03
	Elapsed Time	00:00:00.05

Notes

	Memory Required	43600 bytes
	Additional Memory Required for Residual Plots	0 bytes

Variables Entered/Removed[a]

Model	Variables Entered	Variables Removed	Method
1	Ideology0[b]	.	Enter
2	SES0, RaceCC, GenderCC[b]	.	Enter
3	MRN0[b]	.	Enter
4	MRN0xIdeology0, MRN0xSES0, MRN0xGender, MRN0xRace[b]	.	Enter

a. Dependent Variable: DemCongressX

b. All requested variables entered.

Model Summary

Model	R	R Square	Adjusted R Square	Std. Error of the Estimate	Change Statistics		
					R Square Change	F Change	df1
1	.421[a]	.177	.171	1.37436	.177	29.873	1
2	.425[b]	.180	.156	1.38653	.003	.190	3
3	.425[c]	.180	.150	1.39156	.000	.018	1
4	.466[d]	.217	.164	1.38036	.037	1.550	4

Model Summary

Model	Change Statistics	
	df2	Sig. F Change
1	139	.000
2	136	.903
3	135	.893
4	131	.192

a. Predictors: (Constant), Ideology0

b. Predictors: (Constant), Ideology0, SES0, RaceCC, GenderCC

c. Predictors: (Constant), Ideology0, SES0, RaceCC, GenderCC, MRN0

d. Predictors: (Constant), Ideology0, SES0, RaceCC, GenderCC, MRN0, MRN0xIdeology0, MRN0xSES0, MRN0xGender, MRN0xRace

ANOVA[a]

Model		Sum of Squares	df	Mean Square	F	Sig.
1	Regression	56.426	1	56.426	29.873	.000[b]
	Residual	262.553	139	1.889		
	Total	318.979	140			
2	Regression	57.523	4	14.381	7.480	.000[c]
	Residual	261.455	136	1.922		
	Total	318.979	140			
3	Regression	57.559	5	11.512	5.945	.000[d]
	Residual	261.420	135	1.936		
	Total	318.979	140			
4	Regression	69.371	9	7.708	4.045	.000[e]
	Residual	249.608	131	1.905		
	Total	318.979	140			

a. Dependent Variable: DemCongressX

b. Predictors: (Constant), Ideology0

c. Predictors: (Constant), Ideology0, SES0, RaceCC, GenderCC

d. Predictors: (Constant), Ideology0, SES0, RaceCC, GenderCC, MRN0

e. Predictors: (Constant), Ideology0, SES0, RaceCC, GenderCC, MRN0, MRN0xIdeology0, MRN0xSES0, MRN0xGender, MRN0xRace

Coefficients[a]

Model		Unstandardized Coefficients B	Unstandardized Coefficients Std. Error	Standardized Coefficients Beta	t	Sig.
1	(Constant)	4.099	.116		35.408	.000
	Ideology0	-.374	.068	-.421	-5.466	.000
2	(Constant)	4.037	.147		27.465	.000
	Ideology0	-.388	.074	-.436	-5.265	.000
	GenderCC	.031	.124	.021	.255	.799
	RaceCC	.104	.149	.055	.701	.484
	SES0	.022	.147	.012	.147	.883
3	(Constant)	4.039	.148		27.258	.000
	Ideology0	-.380	.094	-.427	-4.050	.000
	GenderCC	.037	.131	.025	.284	.777
	RaceCC	.103	.149	.055	.691	.491
	SES0	.023	.148	.012	.155	.877
	MRN0	-.025	.181	-.015	-.135	.893
4	(Constant)	4.005	.166		24.194	.000
	Ideology0	-.352	.094	-.395	-3.745	.000
	GenderCC	-.006	.131	-.004	-.048	.962
	RaceCC	.130	.150	.069	.866	.388
	SES0	-.008	.148	-.004	-.057	.955
	MRN0	.148	.202	.090	.733	.465
	MRN0xRace	-.308	.166	-.188	-1.860	.065
	MRN0xSES0	-.003	.175	-.001	-.016	.987
	MRN0xGender	.234	.165	.129	1.415	.159
	MRN0xIdeology0	-.065	.078	-.075	-.833	.406

Coefficients[a]

Model		Correlations Zero-order	Partial	Part
1	(Constant)			
	Ideology0	-.421	-.421	-.421
2	(Constant)			
	Ideology0	-.421	-.411	-.409
	GenderCC	-.114	.022	.020
	RaceCC	-.007	.060	.054
	SES0	-.030	.013	.011
3	(Constant)			
	Ideology0	-.421	-.329	-.316
	GenderCC	-.114	.024	.022
	RaceCC	-.007	.059	.054
	SES0	-.030	.013	.012
	MRN0	-.284	-.012	-.011
4	(Constant)			
	Ideology0	-.421	-.311	-.289
	GenderCC	-.114	-.004	-.004
	RaceCC	-.007	.075	.067
	SES0	-.030	-.005	-.004
	MRN0	-.284	.064	.057
	MRN0xRace	-.321	-.160	-.144
	MRN0xSES0	-.052	-.001	-.001
	MRN0xGender	.093	.123	.109
	MRN0xIdeology0	-.058	-.073	-.064

a. Dependent Variable: DemCongressX

Excluded Variables[a]

Model		Beta In	t	Sig.	Partial Correlation	Collinearity Statistics Tolerance
1	GenderCC	.017[b]	.213	.832	.018	.906
	RaceCC	.054[b]	.696	.488	.059	.979
	SES0	.016[b]	.203	.840	.017	.988
	MRN0	-.008[b]	-.081	.935	-.007	.559
	MRN0xRace	-.164[b]	-1.913	.058	-.161	.793
	MRN0xSES0	-.023[b]	-.299	.765	-.025	.995
	MRN0xGender	.081[b]	1.051	.295	.089	.999
	MRN0xIdeology0	-.035[b]	-.452	.652	-.038	.997
2	MRN0	-.015[c]	-.135	.893	-.012	.496
	MRN0xRace	-.163[c]	-1.885	.062	-.160	.788
	MRN0xSES0	-.019[c]	-.243	.808	-.021	.984
	MRN0xGender	.085[c]	1.088	.279	.093	.977
	MRN0xIdeology0	-.038[c]	-.486	.627	-.042	.989
3	MRN0xRace	-.197[d]	-2.033	.044	-.173	.633
	MRN0xSES0	-.019[d]	-.241	.810	-.021	.984
	MRN0xGender	.085[d]	1.085	.280	.093	.977
	MRN0xIdeology0	-.038[d]	-.484	.629	-.042	.989

a. Dependent Variable: DemCongressX

b. Predictors in the Model: (Constant), Ideology0

c. Predictors in the Model: (Constant), Ideology0, SES0, RaceCC, GenderCC

d. Predictors in the Model: (Constant), Ideology0, SES0, RaceCC, GenderCC, MRN0

```
REGRESSION
 /MISSING LISTWISE
 /STATISTICS COEFF OUTS R ANOVA CHANGE ZPP
 /CRITERIA=PIN(.05) POUT(.10)
 /NOORIGIN
 /DEPENDENT StateX
 /METHOD=ENTER Party0
 /METHOD=ENTER GenderCC RaceCC SES0
 /METHOD=ENTER MRN0
 /METHOD=ENTER MRN0xRace MRN0xSES0 MRN0xGender MRN0xParty0.
```

Regression

Notes

Output Created		15-DEC-2021 13:06:16
Comments		
Input	Data	C:<br>\Users\njs5478\Dropbox\HM and COVID\0. Revise and Resubmit\2. R and R Data\Study 1a\Study1a_Data.sav
	Active Dataset	DataSet1
	Filter	Inclusion = 1 (FILTER)
	Weight	<none>
	Split File	<none>
	N of Rows in Working Data File	178
Missing Value Handling	Definition of Missing	User-defined missing values are treated as missing.
	Cases Used	Statistics are based on cases with no missing values for any variable used.
Syntax		REGRESSION<br>/MISSING LISTWISE<br>/STATISTICS COEFF OUTS R ANOVA CHANGE ZPP<br>/CRITERIA=PIN(.05) POUT(.10)<br>/NOORIGIN<br>/DEPENDENT StateX<br>/METHOD=ENTER Party0<br>/METHOD=ENTER GenderCC RaceCC SES0<br>/METHOD=ENTER MRN0<br>/METHOD=ENTER MRN0xRace MRN0xSES0 MRN0xGender MRN0xParty0.
Resources	Processor Time	00:00:00.03
	Elapsed Time	00:00:00.03

Notes

	Memory Required	43600 bytes
	Additional Memory Required for Residual Plots	0 bytes

Variables Entered/Removed[a]

Model	Variables Entered	Variables Removed	Method
1	Party0[b]	.	Enter
2	SES0, RaceCC, GenderCC[b]	.	Enter
3	MRN0[b]	.	Enter
4	MRN0xParty0 , MRN0xSES0, MRN0xGende r, [b] ...	.	Enter

a. Dependent Variable: StateX

b. All requested variables entered.

Model Summary

Model	R	R Square	Adjusted R Square	Std. Error of the Estimate	Change Statistics		
					R Square Change	F Change	df1
1	.155[a]	.024	.018	1.63943	.024	4.030	1
2	.171[b]	.029	.005	1.65023	.005	.286	3
3	.173[c]	.030	.000	1.65463	.001	.146	1
4	.264[d]	.070	.016	1.64093	.040	1.671	4

Model Summary

Model	Change Statistics	
	df2	Sig. F Change
1	164	.046
2	161	.835
3	160	.703
4	156	.159

a. Predictors: (Constant), Party0

b. Predictors: (Constant), Party0, SES0, RaceCC, GenderCC

c. Predictors: (Constant), Party0, SES0, RaceCC, GenderCC, MRN0

d. Predictors: (Constant), Party0, SES0, RaceCC, GenderCC, MRN0, MRN0xParty0, MRN0xSES0, MRN0xGender, MRN0xRace

ANOVA[a]

Model		Sum of Squares	df	Mean Square	F	Sig.
1	Regression	10.833	1	10.833	4.030	.046[b]
	Residual	440.788	164	2.688		
	Total	451.620	165			
2	Regression	13.173	4	3.293	1.209	.309[c]
	Residual	438.447	161	2.723		
	Total	451.620	165			
3	Regression	13.572	5	2.714	.991	.425[d]
	Residual	438.049	160	2.738		
	Total	451.620	165			
4	Regression	31.569	9	3.508	1.303	.240[e]
	Residual	420.052	156	2.693		
	Total	451.620	165			

a. Dependent Variable: StateX

b. Predictors: (Constant), Party0

c. Predictors: (Constant), Party0, SES0, RaceCC, GenderCC

d. Predictors: (Constant), Party0, SES0, RaceCC, GenderCC, MRN0

e. Predictors: (Constant), Party0, SES0, RaceCC, GenderCC, MRN0, MRN0xParty0, MRN0xSES0, MRN0xGender, MRN0xRace

Coefficients[a]

Model		Unstandardized Coefficients B	Unstandardized Coefficients Std. Error	Standardized Coefficients Beta	t	Sig.
1	(Constant)	4.796	.127		37.677	.000
	Party0	-.184	.092	-.155	-2.008	.046
2	(Constant)	4.738	.161		29.416	.000
	Party0	-.179	.095	-.151	-1.883	.061
	GenderCC	-.025	.131	-.015	-.191	.849
	RaceCC	.089	.162	.043	.546	.586
	SES0	-.114	.157	-.057	-.728	.468
3	(Constant)	4.743	.162		29.298	.000
	Party0	-.157	.111	-.132	-1.408	.161
	GenderCC	-.002	.145	-.001	-.012	.990
	RaceCC	.088	.163	.042	.539	.591
	SES0	-.109	.158	-.055	-.690	.491
	MRN0	-.071	.187	-.039	-.382	.703
4	(Constant)	4.739	.181		26.191	.000
	Party0	-.147	.111	-.124	-1.327	.187
	GenderCC	-.015	.146	-.009	-.104	.917
	RaceCC	.059	.163	.029	.362	.718
	SES0	-.119	.158	-.060	-.755	.452
	MRN0	.199	.217	.109	.914	.362
	MRN0xRace	-.462	.184	-.254	-2.503	.013
	MRN0xSES0	.070	.180	.032	.387	.699
	MRN0xGender	-.096	.173	-.047	-.553	.581
	MRN0xParty0	.097	.108	.076	.902	.368

Coefficients[a]

Model		Correlations Zero-order	Partial	Part
1	(Constant)			
	Party0	-.155	-.155	-.155
2	(Constant)			
	Party0	-.155	-.147	-.146
	GenderCC	-.050	-.015	-.015
	RaceCC	.019	.043	.042
	SES0	-.074	-.057	-.057
3	(Constant)			
	Party0	-.155	-.111	-.110
	GenderCC	-.050	-.001	-.001
	RaceCC	.019	.043	.042
	SES0	-.074	-.054	-.054
	MRN0	-.119	-.030	-.030
4	(Constant)			
	Party0	-.155	-.106	-.102
	GenderCC	-.050	-.008	-.008
	RaceCC	.019	.029	.028
	SES0	-.074	-.060	-.058
	MRN0	-.119	.073	.071
	MRN0xRace	-.220	-.197	-.193
	MRN0xSES0	-.004	.031	.030
	MRN0xGender	-.005	-.044	-.043
	MRN0xParty0	.029	.072	.070

a. Dependent Variable: StateX

Excluded Variables[a]

Model		Beta In	t	Sig.	Partial Correlation	Collinearity Statistics Tolerance
1	GenderCC	-.023[b]	-.289	.773	-.023	.968
	RaceCC	.040[b]	.512	.609	.040	.982
	SES0	-.056[b]	-.720	.472	-.056	.985
	MRN0	-.049[b]	-.531	.596	-.042	.705
	MRN0xRace	-.189[b]	-2.307	.022	-.178	.865
	MRN0xSES0	-.008[b]	-.102	.919	-.008	.999
	MRN0xGender	-.019[b]	-.249	.804	-.020	.991
	MRN0xParty0	.039[b]	.499	.619	.039	.996
2	MRN0	-.039[c]	-.382	.703	-.030	.571
	MRN0xRace	-.188[c]	-2.253	.026	-.175	.841
	MRN0xSES0	-.012[c]	-.155	.877	-.012	.985
	MRN0xGender	-.014[c]	-.183	.855	-.014	.976
	MRN0xParty0	.035[c]	.452	.652	.036	.992
3	MRN0xRace	-.233[d]	-2.391	.018	-.186	.620
	MRN0xSES0	-.011[d]	-.142	.887	-.011	.984
	MRN0xGender	-.016[d]	-.207	.836	-.016	.972
	MRN0xParty0	.037[d]	.466	.642	.037	.990

a. Dependent Variable: StateX

b. Predictors in the Model: (Constant), Party0

c. Predictors in the Model: (Constant), Party0, SES0, RaceCC, GenderCC

d. Predictors in the Model: (Constant), Party0, SES0, RaceCC, GenderCC, MRN0

```
REGRESSION
 /MISSING LISTWISE
 /STATISTICS COEFF OUTS R ANOVA CHANGE ZPP
 /CRITERIA=PIN(.05) POUT(.10)
 /NOORIGIN
 /DEPENDENT StateX
 /METHOD=ENTER Ideology0
 /METHOD=ENTER GenderCC RaceCC SES0
 /METHOD=ENTER MRN0
 /METHOD=ENTER MRN0xRace MRN0xSES0 MRN0xGender MRN0xIdeology0.
```

Regression

Notes

Output Created		15-DEC-2021 13:06:16
Comments		
Input	Data	C: \Users\njs5478\Dropbox\HM and COVID\0. Revise and Resubmit\2. R and R Data\Study 1a\Study1a_Data.sav
	Active Dataset	DataSet1
	Filter	Inclusion = 1 (FILTER)
	Weight	<none>
	Split File	<none>
	N of Rows in Working Data File	178
Missing Value Handling	Definition of Missing	User-defined missing values are treated as missing.
	Cases Used	Statistics are based on cases with no missing values for any variable used.
Syntax		REGRESSION /MISSING LISTWISE /STATISTICS COEFF OUTS R ANOVA CHANGE ZPP /CRITERIA=PIN(.05) POUT(.10) /NOORIGIN /DEPENDENT StateX /METHOD=ENTER Ideology0 /METHOD=ENTER GenderCC RaceCC SES0 /METHOD=ENTER MRN0 /METHOD=ENTER MRN0xRace MRN0xSES0 MRN0xGender MRN0xIdeology0.
Resources	Processor Time	00:00:00.02
	Elapsed Time	00:00:00.02

Notes

	Memory Required	43600 bytes
	Additional Memory Required for Residual Plots	0 bytes

Variables Entered/Removed[a]

Model	Variables Entered	Variables Removed	Method
1	Ideology0[b]	.	Enter
2	RaceCC, SES0, GenderCC[b]	.	Enter
3	MRN0[b]	.	Enter
4	MRN0xIdeology0, MRN0xSES0, MRN0xGender, MRN0xRace[b]	.	Enter

a. Dependent Variable: StateX

b. All requested variables entered.

Model Summary

Model	R	R Square	Adjusted R Square	Std. Error of the Estimate	Change Statistics		
					R Square Change	F Change	df1
1	.149[a]	.022	.016	1.64088	.022	3.733	1
2	.164[b]	.027	.003	1.65210	.005	.260	3
3	.166[c]	.027	-.003	1.65688	.000	.073	1
4	.247[d]	.061	.007	1.64863	.034	1.401	4

Model Summary

Model	Change Statistics	
	df2	Sig. F Change
1	164	.055
2	161	.854
3	160	.787
4	156	.236

a. Predictors: (Constant), Ideology0

b. Predictors: (Constant), Ideology0, RaceCC, SES0, GenderCC

c. Predictors: (Constant), Ideology0, RaceCC, SES0, GenderCC, MRN0

d. Predictors: (Constant), Ideology0, RaceCC, SES0, GenderCC, MRN0, MRN0xIdeology0, MRN0xSES0, MRN0xGender, MRN0xRace

ANOVA[a]

Model		Sum of Squares	df	Mean Square	F	Sig.
1	Regression	10.052	1	10.052	3.733	.055[b]
	Residual	441.568	164	2.692		
	Total	451.620	165			
2	Regression	12.179	4	3.045	1.116	.351[c]
	Residual	439.441	161	2.729		
	Total	451.620	165			
3	Regression	12.379	5	2.476	.902	.481[d]
	Residual	439.241	160	2.745		
	Total	451.620	165			
4	Regression	27.614	9	3.068	1.129	.346[e]
	Residual	424.006	156	2.718		
	Total	451.620	165			

a. Dependent Variable: StateX

b. Predictors: (Constant), Ideology0

c. Predictors: (Constant), Ideology0, RaceCC, SES0, GenderCC

d. Predictors: (Constant), Ideology0, RaceCC, SES0, GenderCC, MRN0

e. Predictors: (Constant), Ideology0, RaceCC, SES0, GenderCC, MRN0, MRN0xIdeology0, MRN0xSES0, MRN0xGender, MRN0xRace

Coefficients[a]

Model		Unstandardized Coefficients B	Unstandardized Coefficients Std. Error	Standardized Coefficients Beta	t	Sig.
1	(Constant)	4.798	.127		37.649	.000
	Ideology0	-.149	.077	-.149	-1.932	.055
2	(Constant)	4.740	.161		29.400	.000
	Ideology0	-.147	.083	-.148	-1.782	.077
	GenderCC	.001	.135	.001	.007	.995
	RaceCC	.092	.163	.045	.567	.572
	SES0	-.111	.157	-.056	-.706	.481
3	(Constant)	4.744	.162		29.254	.000
	Ideology0	-.130	.105	-.130	-1.242	.216
	GenderCC	.015	.145	.009	.102	.919
	RaceCC	.090	.164	.044	.552	.582
	SES0	-.108	.158	-.054	-.683	.496
	MRN0	-.055	.202	-.030	-.270	.787
4	(Constant)	4.781	.181		26.369	.000
	Ideology0	-.100	.105	-.100	-.952	.342
	GenderCC	-.014	.146	-.009	-.098	.922
	RaceCC	.076	.165	.037	.458	.647
	SES0	-.119	.159	-.060	-.751	.454
	MRN0	.185	.227	.102	.815	.416
	MRN0xRace	-.427	.188	-.235	-2.274	.024
	MRN0xSES0	.087	.181	.040	.482	.630
	MRN0xGender	-.016	.184	-.008	-.088	.930
	MRN0xIdeology0	-.021	.088	-.022	-.244	.808

Coefficients[a]

Model		Correlations Zero-order	Partial	Part
1	(Constant)			
	Ideology0	-.149	-.149	-.149
2	(Constant)			
	Ideology0	-.149	-.139	-.139
	GenderCC	-.050	.001	.001
	RaceCC	.019	.045	.044
	SES0	-.074	-.056	-.055
3	(Constant)			
	Ideology0	-.149	-.098	-.097
	GenderCC	-.050	.008	.008
	RaceCC	.019	.044	.043
	SES0	-.074	-.054	-.053
	MRN0	-.119	-.021	-.021
4	(Constant)			
	Ideology0	-.149	-.076	-.074
	GenderCC	-.050	-.008	-.008
	RaceCC	.019	.037	.036
	SES0	-.074	-.060	-.058
	MRN0	-.119	.065	.063
	MRN0xRace	-.220	-.179	-.176
	MRN0xSES0	-.004	.039	.037
	MRN0xGender	-.005	-.007	-.007
	MRN0xIdeology0	-.053	-.020	-.019

a. Dependent Variable: StateX

Excluded Variables[a]

Model		Beta In	t	Sig.	Partial Correlation	Collinearity Statistics Tolerance
1	GenderCC	-.007[b]	-.086	.932	-.007	.916
	RaceCC	.041[b]	.530	.597	.041	.978
	SES0	-.053[b]	-.679	.498	-.053	.977
	MRN0	-.036[b]	-.351	.726	-.027	.567
	MRN0xRace	-.193[b]	-2.233	.027	-.172	.781
	MRN0xSES0	-.004[b]	-.051	.959	-.004	1.000
	MRN0xGender	-.017[b]	-.217	.828	-.017	.993
	MRN0xIdeology0	-.047[b]	-.610	.542	-.048	.999
2	MRN0	-.030[c]	-.270	.787	-.021	.490
	MRN0xRace	-.192[c]	-2.199	.029	-.171	.771
	MRN0xSES0	-.008[c]	-.101	.919	-.008	.984
	MRN0xGender	-.013[c]	-.169	.866	-.013	.977
	MRN0xIdeology0	-.050[c]	-.643	.521	-.051	.993
3	MRN0xRace	-.228[d]	-2.325	.021	-.181	.613
	MRN0xSES0	-.008[d]	-.097	.923	-.008	.984
	MRN0xGender	-.015[d]	-.185	.854	-.015	.973
	MRN0xIdeology0	-.050[d]	-.639	.524	-.051	.993

a. Dependent Variable: StateX

b. Predictors in the Model: (Constant), Ideology0

c. Predictors in the Model: (Constant), Ideology0, RaceCC, SES0, GenderCC

d. Predictors in the Model: (Constant), Ideology0, RaceCC, SES0, GenderCC, MRN0

```
REGRESSION
 /MISSING LISTWISE
 /STATISTICS COEFF OUTS R ANOVA CHANGE ZPP
 /CRITERIA=PIN(.05) POUT(.10)
 /NOORIGIN
 /DEPENDENT Risk_Rules
 /METHOD=ENTER Party0
 /METHOD=ENTER GenderCC RaceCC SES0
 /METHOD=ENTER MRN0
 /METHOD=ENTER MRN0xRace MRN0xSES0 MRN0xGender MRN0xParty0.
```

Regression

Notes

Output Created		15-DEC-2021 13:06:16
Comments		
Input	Data	C: \Users\njs5478\Dropbox\HM and COVID\0. Revise and Resubmit\2. R and R Data\Study 1a\Study1a_Data.sav
	Active Dataset	DataSet1
	Filter	Inclusion = 1 (FILTER)
	Weight	<none>
	Split File	<none>
	N of Rows in Working Data File	178
Missing Value Handling	Definition of Missing	User-defined missing values are treated as missing.
	Cases Used	Statistics are based on cases with no missing values for any variable used.
Syntax		REGRESSION /MISSING LISTWISE /STATISTICS COEFF OUTS R ANOVA CHANGE ZPP /CRITERIA=PIN(.05) POUT(.10) /NOORIGIN /DEPENDENT Risk_Rules /METHOD=ENTER Party0 /METHOD=ENTER GenderCC RaceCC SES0 /METHOD=ENTER MRN0 /METHOD=ENTER MRN0xRace MRN0xSES0 MRN0xGender MRN0xParty0.
Resources	Processor Time	00:00:00.03
	Elapsed Time	00:00:00.02

Notes

	Memory Required	43600 bytes
	Additional Memory Required for Residual Plots	0 bytes

Variables Entered/Removed[a]

Model	Variables Entered	Variables Removed	Method
1	Party0[b]	.	Enter
2	SES0, RaceCC, GenderCC[b]	.	Enter
3	MRN0[b]	.	Enter
4	MRN0xParty0 , MRN0xSES0, MRN0xGende r, ...[b]	.	Enter

a. Dependent Variable: Risk_Rules

b. All requested variables entered.

Model Summary

Model	R	R Square	Adjusted R Square	Std. Error of the Estimate	Change Statistics		
					R Square Change	F Change	df1
1	.299[a]	.089	.084	.95923	.089	17.228	1
2	.451[b]	.203	.185	.90503	.114	8.237	3
3	.504[c]	.254	.233	.87798	.051	11.823	1
4	.520[d]	.270	.231	.87887	.016	.913	4

Model Summary

Model	Change Statistics	
	df2	Sig. F Change
1	176	.000
2	173	.000
3	172	.001
4	168	.458

a. Predictors: (Constant), Party0

b. Predictors: (Constant), Party0, SES0, RaceCC, GenderCC

c. Predictors: (Constant), Party0, SES0, RaceCC, GenderCC, MRN0

d. Predictors: (Constant), Party0, SES0, RaceCC, GenderCC, MRN0, MRN0xParty0, MRN0xSES0, MRN0xGender, MRN0xRace

ANOVA[a]

Model		Sum of Squares	df	Mean Square	F	Sig.
1	Regression	15.852	1	15.852	17.228	.000[b]
	Residual	161.941	176	.920		
	Total	177.793	177			
2	Regression	36.093	4	9.023	11.017	.000[c]
	Residual	141.700	173	.819		
	Total	177.793	177			
3	Regression	45.207	5	9.041	11.729	.000[d]
	Residual	132.586	172	.771		
	Total	177.793	177			
4	Regression	48.028	9	5.336	6.909	.000[e]
	Residual	129.765	168	.772		
	Total	177.793	177			

a. Dependent Variable: Risk_Rules

b. Predictors: (Constant), Party0

c. Predictors: (Constant), Party0, SES0, RaceCC, GenderCC

d. Predictors: (Constant), Party0, SES0, RaceCC, GenderCC, MRN0

e. Predictors: (Constant), Party0, SES0, RaceCC, GenderCC, MRN0, MRN0xParty0, MRN0xSES0, MRN0xGender, MRN0xRace

Coefficients[a]

Model		Unstandardized Coefficients		Standardized Coefficients	t	Sig.
		B	Std. Error	Beta		
1	(Constant)	2.619	.072		36.429	.000
	Party0	.216	.052	.299	4.151	.000
2	(Constant)	2.579	.083		31.222	.000
	Party0	.162	.051	.224	3.173	.002
	GenderCC	.334	.070	.332	4.787	.000
	RaceCC	.132	.084	.109	1.579	.116
	SES0	-.041	.082	-.035	-.503	.616
3	(Constant)	2.562	.080		31.909	.000
	Party0	.062	.057	.086	1.089	.278
	GenderCC	.223	.075	.222	2.970	.003
	RaceCC	.142	.081	.117	1.748	.082
	SES0	-.062	.080	-.052	-.772	.441
	MRN0	.330	.096	.298	3.438	.001
4	(Constant)	2.539	.091		27.871	.000
	Party0	.063	.057	.087	1.099	.273
	GenderCC	.222	.076	.221	2.917	.004
	RaceCC	.159	.082	.131	1.942	.054
	SES0	-.044	.081	-.036	-.536	.593
	MRN0	.267	.114	.242	2.349	.020
	MRN0xRace	.107	.096	.096	1.107	.270
	MRN0xSES0	.106	.095	.078	1.111	.268
	MRN0xGender	.052	.090	.042	.583	.561
	MRN0xParty0	-.038	.056	-.048	-.672	.503

Coefficients[a]

Model		Correlations Zero-order	Partial	Part
1	(Constant)			
	Party0	.299	.299	.299
2	(Constant)			
	Party0	.299	.234	.215
	GenderCC	.367	.342	.325
	RaceCC	.143	.119	.107
	SES0	.034	-.038	-.034
3	(Constant)			
	Party0	.299	.083	.072
	GenderCC	.367	.221	.196
	RaceCC	.143	.132	.115
	SES0	.034	-.059	-.051
	MRN0	.444	.254	.226
4	(Constant)			
	Party0	.299	.085	.072
	GenderCC	.367	.220	.192
	RaceCC	.143	.148	.128
	SES0	.034	-.041	-.035
	MRN0	.444	.178	.155
	MRN0xRace	.320	.085	.073
	MRN0xSES0	.097	.085	.073
	MRN0xGender	.007	.045	.038
	MRN0xParty0	.008	-.052	-.044

a. Dependent Variable: Risk_Rules

Excluded Variables[a]

Model		Beta In	t	Sig.	Partial Correlation	Collinearity Statistics Tolerance
1	GenderCC	.324[b]	4.684	.000	.334	.968
	RaceCC	.092[b]	1.267	.207	.095	.968
	SES0	.000[b]	.006	.995	.000	.987
	MRN0	.397[b]	4.995	.000	.353	.720
	MRN0xRace	.249[b]	3.364	.001	.246	.893
	MRN0xSES0	.103[b]	1.433	.154	.108	1.000
	MRN0xGender	.034[b]	.469	.640	.035	.992
	MRN0xParty0	-.003[b]	-.035	.972	-.003	.999
2	MRN0	.298[c]	3.438	.001	.254	.578
	MRN0xRace	.208[c]	2.895	.004	.216	.859
	MRN0xSES0	.105[c]	1.527	.129	.116	.976
	MRN0xGender	.016[c]	.231	.818	.018	.985
	MRN0xParty0	.005[c]	.068	.946	.005	.996
3	MRN0xRace	.109[d]	1.302	.195	.099	.618
	MRN0xSES0	.096[d]	1.436	.153	.109	.974
	MRN0xGender	.040[d]	.602	.548	.046	.974
	MRN0xParty0	-.011[d]	-.162	.871	-.012	.992

a. Dependent Variable: Risk_Rules

b. Predictors in the Model: (Constant), Party0

c. Predictors in the Model: (Constant), Party0, SES0, RaceCC, GenderCC

d. Predictors in the Model: (Constant), Party0, SES0, RaceCC, GenderCC, MRN0

```
REGRESSION
 /MISSING LISTWISE
 /STATISTICS COEFF OUTS R ANOVA CHANGE ZPP
 /CRITERIA=PIN(.05) POUT(.10)
 /NOORIGIN
 /DEPENDENT Risk_Rules
 /METHOD=ENTER Ideology0
 /METHOD=ENTER GenderCC RaceCC SES0
 /METHOD=ENTER MRN0
 /METHOD=ENTER MRN0xRace MRN0xSES0 MRN0xGender MRN0xIdeology0.
```

Regression

Notes

Output Created		15-DEC-2021 13:06:16
Comments		
Input	Data	C: \Users\njs5478\Dropbox\HM and COVID\0. Revise and Resubmit\2. R and R Data\Study 1a\Study1a_Data.sav
	Active Dataset	DataSet1
	Filter	Inclusion = 1 (FILTER)
	Weight	<none>
	Split File	<none>
	N of Rows in Working Data File	178
Missing Value Handling	Definition of Missing	User-defined missing values are treated as missing.
	Cases Used	Statistics are based on cases with no missing values for any variable used.
Syntax		REGRESSION /MISSING LISTWISE /STATISTICS COEFF OUTS R ANOVA CHANGE ZPP /CRITERIA=PIN(.05) POUT(.10) /NOORIGIN /DEPENDENT Risk_Rules /METHOD=ENTER Ideology0 /METHOD=ENTER GenderCC RaceCC SES0 /METHOD=ENTER MRN0 /METHOD=ENTER MRN0xRace MRN0xSES0 MRN0xGender MRN0xIdeology0.
Resources	Processor Time	00:00:00.02
	Elapsed Time	00:00:00.02

Notes

	Memory Required	43600 bytes
	Additional Memory Required for Residual Plots	0 bytes

Variables Entered/Removed[a]

Model	Variables Entered	Variables Removed	Method
1	Ideology0[b]	.	Enter
2	SES0, RaceCC, GenderCC[b]	.	Enter
3	MRN0[b]	.	Enter
4	MRN0xIdeology0, MRN0xSES0, MRN0xGender, MRN0xRace[b]	.	Enter

a. Dependent Variable: Risk_Rules

b. All requested variables entered.

Model Summary

Model	R	R Square	Adjusted R Square	Std. Error of the Estimate	Change Statistics		
					R Square Change	F Change	df1
1	.361[a]	.131	.126	.93718	.131	26.427	1
2	.467[b]	.218	.200	.89662	.087	6.428	3
3	.506[c]	.256	.234	.87715	.038	8.767	1
4	.518[d]	.269	.230	.87974	.013	.747	4

Model Summary

Model	Change Statistics	
	df2	Sig. F Change
1	176	.000
2	173	.000
3	172	.004
4	168	.562

a. Predictors: (Constant), Ideology0

b. Predictors: (Constant), Ideology0, SES0, RaceCC, GenderCC

c. Predictors: (Constant), Ideology0, SES0, RaceCC, GenderCC, MRN0

d. Predictors: (Constant), Ideology0, SES0, RaceCC, GenderCC, MRN0, MRN0xIdeology0, MRN0xSES0, MRN0xGender, MRN0xRace

ANOVA[a]

Model		Sum of Squares	df	Mean Square	F	Sig.
1	Regression	23.211	1	23.211	26.427	.000[b]
	Residual	154.582	176	.878		
	Total	177.793	177			
2	Regression	38.714	4	9.679	12.039	.000[c]
	Residual	139.079	173	.804		
	Total	177.793	177			
3	Regression	45.459	5	9.092	11.817	.000[d]
	Residual	132.334	172	.769		
	Total	177.793	177			
4	Regression	47.770	9	5.308	6.858	.000[e]
	Residual	130.023	168	.774		
	Total	177.793	177			

a. Dependent Variable: Risk_Rules

b. Predictors: (Constant), Ideology0

c. Predictors: (Constant), Ideology0, SES0, RaceCC, GenderCC

d. Predictors: (Constant), Ideology0, SES0, RaceCC, GenderCC, MRN0

e. Predictors: (Constant), Ideology0, SES0, RaceCC, GenderCC, MRN0, MRN0xIdeology0, MRN0xSES0, MRN0xGender, MRN0xRace

Coefficients[a]

Model		Unstandardized Coefficients B	Unstandardized Coefficients Std. Error	Standardized Coefficients Beta	t	Sig.
1	(Constant)	2.618	.070		37.268	.000
	Ideology0	.219	.043	.361	5.141	.000
2	(Constant)	2.575	.082		31.531	.000
	Ideology0	.160	.044	.264	3.676	.000
	GenderCC	.297	.071	.296	4.185	.000
	RaceCC	.129	.083	.107	1.567	.119
	SES0	-.054	.082	-.045	-.664	.508
3	(Constant)	2.561	.080		31.990	.000
	Ideology0	.066	.053	.108	1.231	.220
	GenderCC	.216	.075	.215	2.899	.004
	RaceCC	.141	.081	.117	1.744	.083
	SES0	-.065	.080	-.054	-.813	.417
	MRN0	.306	.103	.276	2.961	.004
4	(Constant)	2.527	.091		27.896	.000
	Ideology0	.061	.054	.101	1.137	.257
	GenderCC	.219	.076	.218	2.887	.004
	RaceCC	.157	.082	.130	1.901	.059
	SES0	-.046	.081	-.038	-.567	.572
	MRN0	.254	.118	.229	2.151	.033
	MRN0xRace	.090	.097	.082	.930	.354
	MRN0xSES0	.104	.095	.077	1.092	.276
	MRN0xGender	.039	.096	.031	.403	.688
	MRN0xIdeology0	-.007	.046	-.011	-.142	.887

Coefficients[a]

Model		Correlations		
		Zero-order	Partial	Part
1	(Constant)			
	Ideology0	.361	.361	.361
2	(Constant)			
	Ideology0	.361	.269	.247
	GenderCC	.367	.303	.281
	RaceCC	.143	.118	.105
	SES0	.034	-.050	-.045
3	(Constant)			
	Ideology0	.361	.093	.081
	GenderCC	.367	.216	.191
	RaceCC	.143	.132	.115
	SES0	.034	-.062	-.054
	MRN0	.444	.220	.195
4	(Constant)			
	Ideology0	.361	.087	.075
	GenderCC	.367	.217	.190
	RaceCC	.143	.145	.125
	SES0	.034	-.044	-.037
	MRN0	.444	.164	.142
	MRN0xRace	.320	.072	.061
	MRN0xSES0	.097	.084	.072
	MRN0xGender	.007	.031	.027
	MRN0xIdeology0	.039	-.011	-.009

a. Dependent Variable: Risk_Rules

Excluded Variables[a]

Model		Beta In	t	Sig.	Partial Correlation	Collinearity Statistics Tolerance
1	GenderCC	.285[b]	4.048	.000	.293	.914
	RaceCC	.087[b]	1.229	.221	.093	.974
	SES0	-.021[b]	-.300	.765	-.023	.977
	MRN0	.362[b]	4.089	.000	.295	.578
	MRN0xRace	.199[b]	2.577	.011	.191	.804
	MRN0xSES0	.099[b]	1.416	.158	.106	1.000
	MRN0xGender	.041[b]	.575	.566	.043	.991
	MRN0xIdeology0	.036[b]	.513	.609	.039	1.000
2	MRN0	.276[c]	2.961	.004	.220	.496
	MRN0xRace	.182[c]	2.430	.016	.182	.784
	MRN0xSES0	.100[c]	1.479	.141	.112	.975
	MRN0xGender	.022[c]	.324	.747	.025	.983
	MRN0xIdeology0	.032[c]	.473	.637	.036	.993
3	MRN0xRace	.101[d]	1.205	.230	.092	.612
	MRN0xSES0	.094[d]	1.421	.157	.108	.974
	MRN0xGender	.041[d]	.609	.543	.047	.974
	MRN0xIdeology0	.031[d]	.461	.645	.035	.993

a. Dependent Variable: Risk_Rules

b. Predictors in the Model: (Constant), Ideology0

c. Predictors in the Model: (Constant), Ideology0, SES0, RaceCC, GenderCC

d. Predictors in the Model: (Constant), Ideology0, SES0, RaceCC, GenderCC, MRN0

```
REGRESSION
 /MISSING LISTWISE
 /STATISTICS COEFF OUTS R ANOVA CHANGE ZPP
 /CRITERIA=PIN(.05) POUT(.10)
 /NOORIGIN
 /DEPENDENT Risk_Help
 /METHOD=ENTER Party0
 /METHOD=ENTER GenderCC RaceCC SES0
 /METHOD=ENTER MRN0
 /METHOD=ENTER MRN0xRace MRN0xSES0 MRN0xGender MRN0xParty0.
```

Regression

Notes

Output Created		15-DEC-2021 13:06:16
Comments		
Input	Data	C: \Users\njs5478\Dropbox\HM and COVID\0. Revise and Resubmit\2. R and R Data\Study 1a\Study1a_Data.sav
	Active Dataset	DataSet1
	Filter	Inclusion = 1 (FILTER)
	Weight	<none>
	Split File	<none>
	N of Rows in Working Data File	178
Missing Value Handling	Definition of Missing	User-defined missing values are treated as missing.
	Cases Used	Statistics are based on cases with no missing values for any variable used.
Syntax		REGRESSION /MISSING LISTWISE /STATISTICS COEFF OUTS R ANOVA CHANGE ZPP /CRITERIA=PIN(.05) POUT(.10) /NOORIGIN /DEPENDENT Risk_Help /METHOD=ENTER Party0 /METHOD=ENTER GenderCC RaceCC SES0 /METHOD=ENTER MRN0 /METHOD=ENTER MRN0xRace MRN0xSES0 MRN0xGender MRN0xParty0.
Resources	Processor Time	00:00:00.02
	Elapsed Time	00:00:00.02

Notes

	Memory Required	43600 bytes
	Additional Memory Required for Residual Plots	0 bytes

Variables Entered/Removed[a]

Model	Variables Entered	Variables Removed	Method
1	Party0[b]	.	Enter
2	SES0, RaceCC, GenderCC[b]	.	Enter
3	MRN0[b]	.	Enter
4	MRN0xParty0 , MRN0xSES0, MRN0xGende r, ...[b]	.	Enter

a. Dependent Variable: Risk_Help

b. All requested variables entered.

Model Summary

Model	R	R Square	Adjusted R Square	Std. Error of the Estimate	Change Statistics		
					R Square Change	F Change	df1
1	.004[a]	.000	-.006	1.58108	.000	.003	1
2	.205[b]	.042	.020	1.56092	.042	2.526	3
3	.217[c]	.047	.020	1.56117	.005	.945	1
4	.296[d]	.088	.039	1.54578	.040	1.860	4

Model Summary

Model	Change Statistics	
	df2	Sig. F Change
1	176	.957
2	173	.059
3	172	.332
4	168	.120

a. Predictors: (Constant), Party0

b. Predictors: (Constant), Party0, SES0, RaceCC, GenderCC

c. Predictors: (Constant), Party0, SES0, RaceCC, GenderCC, MRN0

d. Predictors: (Constant), Party0, SES0, RaceCC, GenderCC, MRN0, MRN0xParty0, MRN0xSES0, MRN0xGender, MRN0xRace

ANOVA[a]

Model		Sum of Squares	df	Mean Square	F	Sig.
1	Regression	.007	1	.007	.003	.957[b]
	Residual	439.968	176	2.500		
	Total	439.976	177			
2	Regression	18.468	4	4.617	1.895	.113[c]
	Residual	421.508	173	2.436		
	Total	439.976	177			
3	Regression	20.770	5	4.154	1.704	.136[d]
	Residual	419.205	172	2.437		
	Total	439.976	177			
4	Regression	38.548	9	4.283	1.793	.073[e]
	Residual	401.427	168	2.389		
	Total	439.976	177			

a. Dependent Variable: Risk_Help

b. Predictors: (Constant), Party0

c. Predictors: (Constant), Party0, SES0, RaceCC, GenderCC

d. Predictors: (Constant), Party0, SES0, RaceCC, GenderCC, MRN0

e. Predictors: (Constant), Party0, SES0, RaceCC, GenderCC, MRN0, MRN0xParty0, MRN0xSES0, MRN0xGender, MRN0xRace

Coefficients[a]

Model		Unstandardized Coefficients B	Unstandardized Coefficients Std. Error	Standardized Coefficients Beta	t	Sig.
1	(Constant)	3.459	.119		29.186	.000
	Party0	-.005	.086	-.004	-.054	.957
2	(Constant)	3.339	.142		23.444	.000
	Party0	.020	.088	.017	.227	.821
	GenderCC	-.279	.120	-.177	-2.323	.021
	RaceCC	.163	.144	.086	1.128	.261
	SES0	-.087	.142	-.046	-.609	.543
3	(Constant)	3.331	.143		23.335	.000
	Party0	-.030	.102	-.026	-.295	.769
	GenderCC	-.335	.133	-.212	-2.515	.013
	RaceCC	.167	.144	.088	1.162	.247
	SES0	-.097	.143	-.051	-.680	.498
	MRN0	.166	.170	.095	.972	.332
4	(Constant)	3.144	.160		19.626	.000
	Party0	-.026	.101	-.023	-.259	.796
	GenderCC	-.337	.134	-.213	-2.523	.013
	RaceCC	.189	.144	.099	1.315	.190
	SES0	-.064	.143	-.034	-.446	.656
	MRN0	.087	.200	.050	.434	.665
	MRN0xRace	.129	.170	.074	.762	.447
	MRN0xSES0	.168	.167	.078	1.005	.316
	MRN0xGender	.200	.158	.102	1.266	.207
	MRN0xParty0	.102	.099	.082	1.028	.305

Coefficients[a]

Model		Correlations Zero-order	Partial	Part
1	(Constant)			
	Party0	-.004	-.004	-.004
2	(Constant)			
	Party0	-.004	.017	.017
	GenderCC	-.180	-.174	-.173
	RaceCC	.088	.085	.084
	SES0	-.059	-.046	-.045
3	(Constant)			
	Party0	-.004	-.022	-.022
	GenderCC	-.180	-.188	-.187
	RaceCC	.088	.088	.086
	SES0	-.059	-.052	-.051
	MRN0	-.019	.074	.072
4	(Constant)			
	Party0	-.004	-.020	-.019
	GenderCC	-.180	-.191	-.186
	RaceCC	.088	.101	.097
	SES0	-.059	-.034	-.033
	MRN0	-.019	.033	.032
	MRN0xRace	.069	.059	.056
	MRN0xSES0	.122	.077	.074
	MRN0xGender	.120	.097	.093
	MRN0xParty0	.151	.079	.076

a. Dependent Variable: Risk_Help

Excluded Variables[a]

Model		Beta In	t	Sig.	Partial Correlation	Collinearity Statistics Tolerance
1	GenderCC	-.185[b]	-2.450	.015	-.182	.968
	RaceCC	.092[b]	1.203	.231	.091	.968
	SES0	-.059[b]	-.781	.436	-.059	.987
	MRN0	-.024[b]	-.270	.787	-.020	.720
	MRN0xRace	.079[b]	.984	.326	.074	.893
	MRN0xSES0	.122[b]	1.623	.106	.122	1.000
	MRN0xGender	.120[b]	1.597	.112	.120	.992
	MRN0xParty0	.151[b]	2.023	.045	.151	.999
2	MRN0	.095[c]	.972	.332	.074	.578
	MRN0xRace	.123[c]	1.541	.125	.117	.859
	MRN0xSES0	.125[c]	1.665	.098	.126	.976
	MRN0xGender	.137[c]	1.841	.067	.139	.985
	MRN0xParty0	.142[c]	1.923	.056	.145	.996
3	MRN0xRace	.114[d]	1.204	.230	.092	.618
	MRN0xSES0	.122[d]	1.626	.106	.123	.974
	MRN0xGender	.146[d]	1.957	.052	.148	.974
	MRN0xParty0	.138[d]	1.859	.065	.141	.992

a. Dependent Variable: Risk_Help

b. Predictors in the Model: (Constant), Party0

c. Predictors in the Model: (Constant), Party0, SES0, RaceCC, GenderCC

d. Predictors in the Model: (Constant), Party0, SES0, RaceCC, GenderCC, MRN0

```
REGRESSION
 /MISSING LISTWISE
 /STATISTICS COEFF OUTS R ANOVA CHANGE ZPP
 /CRITERIA=PIN(.05) POUT(.10)
 /NOORIGIN
 /DEPENDENT Risk_Help
 /METHOD=ENTER Ideology0
 /METHOD=ENTER GenderCC RaceCC SES0
 /METHOD=ENTER MRN0
 /METHOD=ENTER MRN0xRace MRN0xSES0 MRN0xGender MRN0xIdeology0.
```

Regression

Notes

Output Created		15-DEC-2021 13:06:16
Comments		
Input	Data	C:<br>\Users\njs5478\Dropbox\HM and COVID\0. Revise and Resubmit\2. R and R Data\Study 1a\Study1a_Data.sav
	Active Dataset	DataSet1
	Filter	Inclusion = 1 (FILTER)
	Weight	<none>
	Split File	<none>
	N of Rows in Working Data File	178
Missing Value Handling	Definition of Missing	User-defined missing values are treated as missing.
	Cases Used	Statistics are based on cases with no missing values for any variable used.
Syntax		REGRESSION<br>/MISSING LISTWISE<br>/STATISTICS COEFF OUTS R ANOVA CHANGE ZPP<br>/CRITERIA=PIN(.05) POUT(.10)<br>/NOORIGIN<br>/DEPENDENT Risk_Help<br>/METHOD=ENTER Ideology0<br>/METHOD=ENTER GenderCC RaceCC SES0<br>/METHOD=ENTER MRN0<br>/METHOD=ENTER MRN0xRace MRN0xSES0 MRN0xGender MRN0xIdeology0.
Resources	Processor Time	00:00:00.02
	Elapsed Time	00:00:00.02

Notes

	Memory Required	43600 bytes
	Additional Memory Required for Residual Plots	0 bytes

Variables Entered/Removed[a]

Model	Variables Entered	Variables Removed	Method
1	Ideology0[b]	.	Enter
2	SES0, RaceCC, GenderCC[b]	.	Enter
3	MRN0[b]	.	Enter
4	MRN0xIdeology0, MRN0xSES0, MRN0xGender, MRN0xRace[b]	.	Enter

a. Dependent Variable: Risk_Help

b. All requested variables entered.

Model Summary

Model	R	R Square	Adjusted R Square	Std. Error of the Estimate	Change Statistics		
					R Square Change	F Change	df1
1	.041[a]	.002	-.004	1.57980	.002	.289	1
2	.204[b]	.042	.020	1.56114	.040	2.411	3
3	.222[c]	.049	.021	1.55959	.007	1.344	1
4	.305[d]	.093	.044	1.54140	.044	2.021	4

Model Summary

Model	Change Statistics	
	df2	Sig. F Change
1	176	.591
2	173	.069
3	172	.248
4	168	.094

a. Predictors: (Constant), Ideology0

b. Predictors: (Constant), Ideology0, SES0, RaceCC, GenderCC

c. Predictors: (Constant), Ideology0, SES0, RaceCC, GenderCC, MRN0

d. Predictors: (Constant), Ideology0, SES0, RaceCC, GenderCC, MRN0, MRN0xIdeology0, MRN0xSES0, MRN0xGender, MRN0xRace

ANOVA[a]

Model		Sum of Squares	df	Mean Square	F	Sig.
1	Regression	.722	1	.722	.289	.591[b]
	Residual	439.253	176	2.496		
	Total	439.976	177			
2	Regression	18.348	4	4.587	1.882	.116[c]
	Residual	421.628	173	2.437		
	Total	439.976	177			
3	Regression	21.617	5	4.323	1.777	.120[d]
	Residual	418.359	172	2.432		
	Total	439.976	177			
4	Regression	40.824	9	4.536	1.909	.054[e]
	Residual	399.152	168	2.376		
	Total	439.976	177			

a. Dependent Variable: Risk_Help

b. Predictors: (Constant), Ideology0

c. Predictors: (Constant), Ideology0, SES0, RaceCC, GenderCC

d. Predictors: (Constant), Ideology0, SES0, RaceCC, GenderCC, MRN0

e. Predictors: (Constant), Ideology0, SES0, RaceCC, GenderCC, MRN0, MRN0xIdeology0, MRN0xSES0, MRN0xGender, MRN0xRace

Coefficients[a]

Model		Unstandardized Coefficients B	Unstandardized Coefficients Std. Error	Standardized Coefficients Beta	t	Sig.
1	(Constant)	3.459	.118		29.211	.000
	Ideology0	-.039	.072	-.041	-.538	.591
2	(Constant)	3.337	.142		23.464	.000
	Ideology0	.003	.076	.004	.045	.965
	GenderCC	-.276	.124	-.175	-2.234	.027
	RaceCC	.167	.144	.088	1.165	.246
	SES0	-.085	.143	-.045	-.593	.554
3	(Constant)	3.327	.142		23.372	.000
	Ideology0	-.063	.095	-.066	-.660	.510
	GenderCC	-.332	.133	-.210	-2.505	.013
	RaceCC	.175	.144	.092	1.221	.224
	SES0	-.092	.143	-.049	-.646	.519
	MRN0	.213	.184	.122	1.159	.248
4	(Constant)	3.137	.159		19.763	.000
	Ideology0	-.066	.094	-.070	-.705	.482
	GenderCC	-.336	.133	-.213	-2.535	.012
	RaceCC	.184	.144	.097	1.272	.205
	SES0	-.062	.143	-.033	-.436	.664
	MRN0	.147	.207	.084	.711	.478
	MRN0xRace	.130	.170	.075	.766	.445
	MRN0xSES0	.155	.167	.072	.926	.356
	MRN0xGender	.155	.168	.079	.923	.357
	MRN0xIdeology0	.101	.081	.108	1.253	.212

Coefficients[a]

Model		Correlations Zero-order	Partial	Part
1	(Constant)			
	Ideology0	-.041	-.041	-.041
2	(Constant)			
	Ideology0	-.041	.003	.003
	GenderCC	-.180	-.167	-.166
	RaceCC	.088	.088	.087
	SES0	-.059	-.045	-.044
3	(Constant)			
	Ideology0	-.041	-.050	-.049
	GenderCC	-.180	-.188	-.186
	RaceCC	.088	.093	.091
	SES0	-.059	-.049	-.048
	MRN0	-.019	.088	.086
4	(Constant)			
	Ideology0	-.041	-.054	-.052
	GenderCC	-.180	-.192	-.186
	RaceCC	.088	.098	.094
	SES0	-.059	-.034	-.032
	MRN0	-.019	.055	.052
	MRN0xRace	.069	.059	.056
	MRN0xSES0	.122	.071	.068
	MRN0xGender	.120	.071	.068
	MRN0xIdeology0	.180	.096	.092

a. Dependent Variable: Risk_Help

Excluded Variables[a]

Model		Beta In	t	Sig.	Partial Correlation	Collinearity Statistics Tolerance
1	GenderCC	-.184[b]	-2.363	.019	-.176	.914
	RaceCC	.097[b]	1.278	.203	.096	.974
	SES0	-.054[b]	-.708	.480	-.053	.977
	MRN0	.012[b]	.119	.905	.009	.578
	MRN0xRace	.108[b]	1.287	.200	.097	.804
	MRN0xSES0	.122[b]	1.622	.107	.122	1.000
	MRN0xGender	.117[b]	1.553	.122	.117	.991
	MRN0xIdeology0	.180[b]	2.426	.016	.180	1.000
2	MRN0	.122[c]	1.159	.248	.088	.496
	MRN0xRace	.140[c]	1.672	.096	.126	.784
	MRN0xSES0	.125[c]	1.665	.098	.126	.975
	MRN0xGender	.136[c]	1.827	.069	.138	.983
	MRN0xIdeology0	.171[c]	2.320	.022	.174	.993
3	MRN0xRace	.121[d]	1.273	.205	.097	.612
	MRN0xSES0	.122[d]	1.632	.105	.124	.974
	MRN0xGender	.146[d]	1.949	.053	.147	.974
	MRN0xIdeology0	.171[d]	2.314	.022	.174	.993

a. Dependent Variable: Risk_Help

b. Predictors in the Model: (Constant), Ideology0

c. Predictors in the Model: (Constant), Ideology0, SES0, RaceCC, GenderCC

d. Predictors in the Model: (Constant), Ideology0, SES0, RaceCC, GenderCC, MRN0

```
***Simple Slopes***

DESCRIPTIVES VARIABLES=MRN SES
  /STATISTICS=MEAN STDDEV MIN MAX.
```

Descriptives

Notes

Output Created		15-DEC-2021 13:06:16
Comments		
Input	Data	C:\Users\njs5478\Dropbox\HM and COVID\0. Revise and Resubmit\2. R and R Data\Study 1a\Study1a_Data.sav
	Active Dataset	DataSet1
	Filter	Inclusion = 1 (FILTER)
	Weight	<none>
	Split File	<none>
	N of Rows in Working Data File	178
Missing Value Handling	Definition of Missing	User defined missing values are treated as missing.
	Cases Used	All non-missing data are used.
Syntax		DESCRIPTIVES VARIABLES=MRN SES /STATISTICS=MEAN STDDEV MIN MAX.
Resources	Processor Time	00:00:00.00
	Elapsed Time	00:00:00.00

Descriptive Statistics

	N	Minimum	Maximum	Mean	Std. Deviation
MRN	178	1.27	5.88	3.4304	.90563
Self Reported Socioeconomic Status	178	1	5	3.42	.835
Valid N (listwise)	178				

```
 COMPUTE MRN.High = MRN0 - .90563.
COMPUTE MRN.Low = MRN0 +.90563.

COMPUTE SES.High = SES0 - .835.
COMPUTE SES.Low = SES0 + .835.

IF (GenderCC=1) Male = 0.
```

```
EXECUTE.
IF (GenderCC=-1) Male=1.
EXECUTE.
IF (GenderCC=-1) Female=0.
EXECUTE.
IF (GenderCC=1) Female=1.
EXECUTE.

**MRN*Gender on Financial

  UNIANOVA Finance_Tot WITH MRN0 Male Party0 RaceCC SES0
  /PRINT=ETASQ PARAMETER
  /DESIGN=MRN0 Male Party0 RaceCC SES0 MRN0*Male MRN0*Party0 MRN0*RaceCC
    MRN0*SES0.
```

Univariate Analysis of Variance

Notes

Output Created		15-DEC-2021 13:06:16
Comments		
Input	Data	C:\Users\njs5478\Dropbox\HM and COVID\0. Revise and Resubmit\2. R and R Data\Study 1a\Study1a_Data.sav
	Active Dataset	DataSet1
	Filter	Inclusion = 1 (FILTER)
	Weight	<none>
	Split File	<none>
	N of Rows in Working Data File	178
Missing Value Handling	Definition of Missing	User-defined missing values are treated as missing.
	Cases Used	Statistics are based on all cases with valid data for all variables in the model.
Syntax		UNIANOVA Finance_Tot WITH MRN0 Male Party0 RaceCC SES0 /PRINT=ETASQ PARAMETER /DESIGN=MRN0 Male Party0 RaceCC SES0 MRN0*Male MRN0*Party0 MRN0*RaceCC MRN0*SES0.

Notes

Resources	Processor Time	00:00:00.02
	Elapsed Time	00:00:00.02

Tests of Between-Subjects Effects

Dependent Variable: Finance_Tot

Source	Type III Sum of Squares	df	Mean Square	F	Sig.	Partial Eta Squared
Corrected Model	102.822[a]	9	11.425	5.266	.000	.220
Intercept	750.613	1	750.613	345.953	.000	.673
MRN0	7.350	1	7.350	3.388	.067	.020
Male	4.304E-5	1	4.304E-5	.000	.996	.000
Party0	1.165	1	1.165	.537	.465	.003
RaceCC	19.833	1	19.833	9.141	.003	.052
SES0	31.794	1	31.794	14.653	.000	.080
MRN0 * Male	12.233	1	12.233	5.638	.019	.032
MRN0 * Party0	.020	1	.020	.009	.923	.000
MRN0 * RaceCC	.009	1	.009	.004	.948	.000
MRN0 * SES0	30.452	1	30.452	14.035	.000	.077
Error	364.509	168	2.170			
Total	3310.000	178				
Corrected Total	467.331	177				

a. R Squared = .220 (Adjusted R Squared = .178)

Parameter Estimates

Dependent Variable: Finance_Tot

Parameter	B	Std. Error	t	Sig.	95% Confidence Interval	
					Lower Bound	Upper Bound
Intercept	3.853	.207	18.600	.000	3.444	4.262
MRN0	-.465	.252	-1.841	.067	-.963	.034
Male	-.001	.255	-.004	.996	-.504	.502
Party0	.070	.096	.733	.465	-.119	.260
RaceCC	.414	.137	3.023	.003	.144	.684
SES0	-.521	.136	-3.828	.000	-.790	-.252
MRN0 * Male	.716	.302	2.375	.019	.121	1.311
MRN0 * Party0	-.009	.094	-.097	.923	-.195	.177
MRN0 * RaceCC	-.010	.162	-.065	.948	-.329	.308
MRN0 * SES0	.596	.159	3.746	.000	.282	.911

Parameter Estimates

Dependent Variable: Finance_Tot

Parameter	Partial Eta Squared
Intercept	.673
MRN0	.020
Male	.000
Party0	.003
RaceCC	.052
SES0	.080
MRN0 * Male	.032
MRN0 * Party0	.000
MRN0 * RaceCC	.000
MRN0 * SES0	.077

```
UNIANOVA Finance_Tot WITH MRN0 Female Party0 RaceCC SES0
 /PRINT=ETASQ PARAMETER
 /DESIGN=MRN0 Female Party0 RaceCC SES0 MRN0*Female MRN0*Party0 MRN0*RaceCC
   MRN0*SES0.
```

Univariate Analysis of Variance

Notes

Output Created		15-DEC-2021 13:06:16
Comments		
Input	Data	C: \Users\njs5478\Dropbox\HM and COVID\0. Revise and Resubmit\2. R and R Data\Study 1a\Study1a_Data.sav
	Active Dataset	DataSet1
	Filter	Inclusion = 1 (FILTER)
	Weight	<none>
	Split File	<none>
	N of Rows in Working Data File	178
Missing Value Handling	Definition of Missing	User-defined missing values are treated as missing.
	Cases Used	Statistics are based on all cases with valid data for all variables in the model.
Syntax		UNIANOVA Finance_Tot WITH MRN0 Female Party0 RaceCC SES0 /PRINT=ETASQ PARAMETER /DESIGN=MRN0 Female Party0 RaceCC SES0 MRN0*Female MRN0*Party0 MRN0*RaceCC MRN0*SES0.
Resources	Processor Time	00:00:00.00
	Elapsed Time	00:00:00.00

Tests of Between-Subjects Effects

Dependent Variable: Finance_Tot

Source	Type III Sum of Squares	df	Mean Square	F	Sig.	Partial Eta Squared
Corrected Model	102.822[a]	9	11.425	5.266	.000	.220
Intercept	889.578	1	889.578	410.001	.000	.709
MRN0	2.511	1	2.511	1.158	.284	.007
Female	4.304E-5	1	4.304E-5	.000	.996	.000
Party0	1.165	1	1.165	.537	.465	.003
RaceCC	19.833	1	19.833	9.141	.003	.052
SES0	31.794	1	31.794	14.653	.000	.080
MRN0 * Female	12.233	1	12.233	5.638	.019	.032
MRN0 * Party0	.020	1	.020	.009	.923	.000
MRN0 * RaceCC	.009	1	.009	.004	.948	.000
MRN0 * SES0	30.452	1	30.452	14.035	.000	.077
Error	364.509	168	2.170			
Total	3310.000	178				
Corrected Total	467.331	177				

a. R Squared = .220 (Adjusted R Squared = .178)

Parameter Estimates

Dependent Variable: Finance_Tot

Parameter	B	Std. Error	t	Sig.	95% Confidence Interval Lower Bound	95% Confidence Interval Upper Bound
Intercept	3.851	.190	20.248	.000	3.476	4.227
MRN0	.251	.234	1.076	.284	-.210	.713
Female	.001	.255	.004	.996	-.502	.504
Party0	.070	.096	.733	.465	-.119	.260
RaceCC	.414	.137	3.023	.003	.144	.684
SES0	-.521	.136	-3.828	.000	-.790	-.252
MRN0 * Female	-.716	.302	-2.375	.019	-1.311	-.121
MRN0 * Party0	-.009	.094	-.097	.923	-.195	.177
MRN0 * RaceCC	-.010	.162	-.065	.948	-.329	.308
MRN0 * SES0	.596	.159	3.746	.000	.282	.911

Parameter Estimates

Dependent Variable: Finance_Tot

Parameter	Partial Eta Squared
Intercept	.709
MRN0	.007
Female	.000
Party0	.003
RaceCC	.052
SES0	.080
MRN0 * Female	.032
MRN0 * Party0	.000
MRN0 * RaceCC	.000
MRN0 * SES0	.077

```
UNIANOVA Finance_Tot WITH GenderCC MRN.Low Party0 RaceCC SES0
 /PRINT=ETASQ PARAMETER
 /DESIGN=GenderCC MRN.Low Party0 RaceCC SES0 Party0*MRN.Low
   MRN.Low*GenderCC MRN.Low*RaceCC MRN.Low*SES0.
```

Univariate Analysis of Variance

Notes

Output Created		15-DEC-2021 13:06:16
Comments		
Input	Data	C:\Users\njs5478\Dropbox\HM and COVID\0. Revise and Resubmit\2. R and R Data\Study 1a\Study1a_Data.sav
	Active Dataset	DataSet1
	Filter	Inclusion = 1 (FILTER)
	Weight	<none>
	Split File	<none>
	N of Rows in Working Data File	178
Missing Value Handling	Definition of Missing	User-defined missing values are treated as missing.
	Cases Used	Statistics are based on all cases with valid data for all variables in the model.
Syntax		UNIANOVA Finance_Tot WITH GenderCC MRN.Low Party0 RaceCC SES0 /PRINT=ETASQ PARAMETER /DESIGN=GenderCC MRN.Low Party0 RaceCC SES0 Party0*MRN.Low MRN.Low*GenderCC MRN.Low*RaceCC MRN.Low*SES0.
Resources	Processor Time	00:00:00.00
	Elapsed Time	00:00:00.00

Tests of Between-Subjects Effects

Dependent Variable: Finance_Tot

Source	Type III Sum of Squares	df	Mean Square	F	Sig.
Corrected Model	102.822[a]	9	11.425	5.266	.000
Intercept	607.798	1	607.798	280.131	.000
GenderCC	5.816	1	5.816	2.681	.103
MRN.Low	.676	1	.676	.312	.577
Party0	.809	1	.809	.373	.542
RaceCC	10.545	1	10.545	4.860	.029
SES0	71.797	1	71.797	33.091	.000
MRN.Low * Party0	.020	1	.020	.009	.923
GenderCC * MRN.Low	12.233	1	12.233	5.638	.019
MRN.Low * RaceCC	.009	1	.009	.004	.948
MRN.Low * SES0	30.452	1	30.452	14.035	.000
Error	364.509	168	2.170		
Total	3310.000	178			
Corrected Total	467.331	177			

Tests of Between-Subjects Effects

Dependent Variable: Finance_Tot

Source	Partial Eta Squared
Corrected Model	.220
Intercept	.625
GenderCC	.016
MRN.Low	.002
Party0	.002
RaceCC	.028
SES0	.165
MRN.Low * Party0	.000
GenderCC * MRN.Low	.032
MRN.Low * RaceCC	.000
MRN.Low * SES0	.077
Error	
Total	
Corrected Total	

a. R Squared = .220 (Adjusted R Squared = .178)

Parameter Estimates

Dependent Variable: Finance_Tot

Parameter	B	Std. Error	t	Sig.	95% Confidence Interval	
					Lower Bound	Upper Bound
Intercept	3.949	.236	16.737	.000	3.483	4.414
GenderCC	.325	.198	1.637	.103	-.067	.716
MRN.Low	-.107	.191	-.558	.577	-.483	.270
Party0	.079	.129	.611	.542	-.176	.333
RaceCC	.423	.192	2.205	.029	.044	.803
SES0	-1.061	.184	-5.752	.000	-1.425	-.697
MRN.Low * Party0	-.009	.094	-.097	.923	-.195	.177
GenderCC * MRN.Low	-.358	.151	-2.375	.019	-.656	-.060
MRN.Low * RaceCC	-.010	.162	-.065	.948	-.329	.308
MRN.Low * SES0	.596	.159	3.746	.000	.282	.911

Parameter Estimates

Dependent Variable: Finance_Tot

Parameter	Partial Eta Squared
Intercept	.625
GenderCC	.016
MRN.Low	.002
Party0	.002
RaceCC	.028
SES0	.165
MRN.Low * Party0	.000
GenderCC * MRN.Low	.032
MRN.Low * RaceCC	.000
MRN.Low * SES0	.077

```
UNIANOVA Finance_Tot WITH GenderCC MRN.High Party0 RaceCC SES0
 /PRINT=ETASQ PARAMETER
 /DESIGN=Party0 MRN.High GenderCC RaceCC SES0 Party0*MRN.High
   MRN.High*GenderCC MRN.High*RaceCC MRN.High*SES0.
```

Univariate Analysis of Variance

Notes

Output Created		15-DEC-2021 13:06:16
Comments		
Input	Data	C: \Users\njs5478\Dropbox\HM and COVID\0. Revise and Resubmit\2. R and R Data\Study 1a\Study1a_Data.sav
	Active Dataset	DataSet1
	Filter	Inclusion = 1 (FILTER)
	Weight	<none>
	Split File	<none>
	N of Rows in Working Data File	178
Missing Value Handling	Definition of Missing	User-defined missing values are treated as missing.
	Cases Used	Statistics are based on all cases with valid data for all variables in the model.
Syntax		UNIANOVA Finance_Tot WITH GenderCC MRN.High Party0 RaceCC SES0 /PRINT=ETASQ PARAMETER /DESIGN=Party0 MRN.High GenderCC RaceCC SES0 Party0*MRN.High MRN.High*GenderCC MRN.High*RaceCC MRN.High*SES0.
Resources	Processor Time	00:00:00.02
	Elapsed Time	00:00:00.02

Tests of Between-Subjects Effects

Dependent Variable: Finance_Tot

Source	Type III Sum of Squares	df	Mean Square	F	Sig.
Corrected Model	102.822[a]	9	11.425	5.266	.000
Intercept	603.251	1	603.251	278.035	.000
Party0	.511	1	.511	.236	.628
MRN.High	.676	1	.676	.312	.577
GenderCC	7.477	1	7.477	3.446	.065
RaceCC	8.181	1	8.181	3.771	.054
SES0	.018	1	.018	.008	.928
MRN.High * Party0	.020	1	.020	.009	.923
GenderCC * MRN.High	12.233	1	12.233	5.638	.019
MRN.High * RaceCC	.009	1	.009	.004	.948
MRN.High * SES0	30.452	1	30.452	14.035	.000
Error	364.509	168	2.170		
Total	3310.000	178			
Corrected Total	467.331	177			

Tests of Between-Subjects Effects

Dependent Variable: Finance_Tot

Source	Partial Eta Squared
Corrected Model	.220
Intercept	.623
Party0	.001
MRN.High	.002
GenderCC	.020
RaceCC	.022
SES0	.000
MRN.High * Party0	.000
GenderCC * MRN.High	.032
MRN.High * RaceCC	.000
MRN.High * SES0	.077
Error	
Total	
Corrected Total	

a. R Squared = .220 (Adjusted R Squared = .178)

Parameter Estimates

Dependent Variable: Finance_Tot

Parameter	B	Std. Error	t	Sig.	95% Confidence Interval Lower Bound	95% Confidence Interval Upper Bound
Intercept	3.756	.225	16.674	.000	3.311	4.200
Party0	.062	.128	.486	.628	-.191	.315
MRN.High	-.107	.191	-.558	.577	-.483	.270
GenderCC	-.324	.174	-1.856	.065	-.668	.021
RaceCC	.405	.208	1.942	.054	-.007	.816
SES0	.019	.211	.091	.928	-.398	.436
MRN.High * Party0	-.009	.094	-.097	.923	-.195	.177
GenderCC * MRN.High	-.358	.151	-2.375	.019	-.656	-.060
MRN.High * RaceCC	-.010	.162	-.065	.948	-.329	.308
MRN.High * SES0	.596	.159	3.746	.000	.282	.911

Parameter Estimates

Dependent Variable: Finance_Tot

Parameter	Partial Eta Squared
Intercept	.623
Party0	.001
MRN.High	.002
GenderCC	.020
RaceCC	.022
SES0	.000
MRN.High * Party0	.000
GenderCC * MRN.High	.032
MRN.High * RaceCC	.000
MRN.High * SES0	.077

```
**MRN*SES on Financial

 UNIANOVA Finance_Tot WITH MRN0 GenderCC RaceCC SES.Low Party0
 /PRINT=ETASQ PARAMETER
 /DESIGN=MRN0 GenderCC RaceCC SES.Low MRN0*GenderCC MRN0*RaceCC
   MRN0*SES.Low Party0 MRN0*Party0.
```

Univariate Analysis of Variance

Notes

Output Created		15-DEC-2021 13:06:16
Comments		
Input	Data	C: \Users\njs5478\Dropbox\HM and COVID\0. Revise and Resubmit\2. R and R Data\Study 1a\Study1a_Data.sav
	Active Dataset	DataSet1
	Filter	Inclusion = 1 (FILTER)
	Weight	<none>
	Split File	<none>
	N of Rows in Working Data File	178
Missing Value Handling	Definition of Missing	User-defined missing values are treated as missing.
	Cases Used	Statistics are based on all cases with valid data for all variables in the model.
Syntax		UNIANOVA Finance_Tot WITH MRN0 GenderCC RaceCC SES.Low Party0 /PRINT=ETASQ PARAMETER /DESIGN=MRN0 GenderCC RaceCC SES.Low MRN0*GenderCC MRN0*RaceCC MRN0*SES.Low Party0 MRN0*Party0.
Resources	Processor Time	00:00:00.02
	Elapsed Time	00:00:00.02

Tests of Between-Subjects Effects

Dependent Variable: Finance_Tot

Source	Type III Sum of Squares	df	Mean Square	F	Sig.	Partial Eta Squared
Corrected Model	102.822[a]	9	11.425	5.266	.000	.220
Intercept	1102.876	1	1102.876	508.310	.000	.752
MRN0	15.673	1	15.673	7.223	.008	.041
GenderCC	4.304E-5	1	4.304E-5	.000	.996	.000
RaceCC	19.833	1	19.833	9.141	.003	.052
SES.Low	31.794	1	31.794	14.653	.000	.080
MRN0 * GenderCC	12.233	1	12.233	5.638	.019	.032
MRN0 * RaceCC	.009	1	.009	.004	.948	.000
MRN0 * SES.Low	30.452	1	30.452	14.035	.000	.077
Party0	1.165	1	1.165	.537	.465	.003
MRN0 * Party0	.020	1	.020	.009	.923	.000
Error	364.509	168	2.170			
Total	3310.000	178				
Corrected Total	467.331	177				

a. R Squared = .220 (Adjusted R Squared = .178)

Parameter Estimates

Dependent Variable: Finance_Tot

Parameter	B	Std. Error	t	Sig.	95% Confidence Interval	
					Lower Bound	Upper Bound
Intercept	4.287	.190	22.546	.000	3.912	4.662
MRN0	-.605	.225	-2.688	.008	-1.049	-.161
GenderCC	.001	.127	.004	.996	-.251	.252
RaceCC	.414	.137	3.023	.003	.144	.684
SES.Low	-.521	.136	-3.828	.000	-.790	-.252
MRN0 * GenderCC	-.358	.151	-2.375	.019	-.656	-.060
MRN0 * RaceCC	-.010	.162	-.065	.948	-.329	.308
MRN0 * SES.Low	.596	.159	3.746	.000	.282	.911
Party0	.070	.096	.733	.465	-.119	.260
MRN0 * Party0	-.009	.094	-.097	.923	-.195	.177

Parameter Estimates

Dependent Variable: Finance_Tot

Parameter	Partial Eta Squared
Intercept	.752
MRN0	.041
GenderCC	.000
RaceCC	.052
SES.Low	.080
MRN0 * GenderCC	.032
MRN0 * RaceCC	.000
MRN0 * SES.Low	.077
Party0	.003
MRN0 * Party0	.000

```
UNIANOVA Finance_Tot WITH MRN0 GenderCC RaceCC SES.High Party0
 /PRINT=ETASQ PARAMETER
 /DESIGN=MRN0 GenderCC RaceCC SES.High MRN0*GenderCC MRN0*RaceCC
   MRN0*SES.High Party0 MRN0*Party0.
```

Univariate Analysis of Variance

Notes

Output Created		15-DEC-2021 13:06:16
Comments		
Input	Data	C:\Users\njs5478\Dropbox\HM and COVID\0. Revise and Resubmit\2. R and R Data\Study 1a\Study1a_Data.sav
	Active Dataset	DataSet1
	Filter	Inclusion = 1 (FILTER)
	Weight	<none>
	Split File	<none>
	N of Rows in Working Data File	178
Missing Value Handling	Definition of Missing	User-defined missing values are treated as missing.
	Cases Used	Statistics are based on all cases with valid data for all variables in the model.
Syntax		UNIANOVA Finance_Tot WITH MRN0 GenderCC RaceCC SES.High Party0 /PRINT=ETASQ PARAMETER /DESIGN=MRN0 GenderCC RaceCC SES.High MRN0*GenderCC MRN0*RaceCC MRN0*SES.High Party0 MRN0*Party0.
Resources	Processor Time	00:00:00.00
	Elapsed Time	00:00:00.02

Tests of Between-Subjects Effects

Dependent Variable: Finance_Tot

Source	Type III Sum of Squares	df	Mean Square	F	Sig.	Partial Eta Squared
Corrected Model	102.822[a]	9	11.425	5.266	.000	.220
Intercept	697.895	1	697.895	321.656	.000	.657
MRN0	5.773	1	5.773	2.661	.105	.016
GenderCC	4.304E-5	1	4.304E-5	.000	.996	.000
RaceCC	19.833	1	19.833	9.141	.003	.052
SES.High	31.794	1	31.794	14.653	.000	.080
MRN0 * GenderCC	12.233	1	12.233	5.638	.019	.032
MRN0 * RaceCC	.009	1	.009	.004	.948	.000
MRN0 * SES.High	30.452	1	30.452	14.035	.000	.077
Party0	1.165	1	1.165	.537	.465	.003
MRN0 * Party0	.020	1	.020	.009	.923	.000
Error	364.509	168	2.170			
Total	3310.000	178				
Corrected Total	467.331	177				

a. R Squared = .220 (Adjusted R Squared = .178)

Parameter Estimates

Dependent Variable: Finance_Tot

Parameter	B	Std. Error	t	Sig.	95% Confidence Interval	
					Lower Bound	Upper Bound
Intercept	3.417	.191	17.935	.000	3.041	3.793
MRN0	.392	.240	1.631	.105	-.082	.865
GenderCC	.001	.127	.004	.996	-.251	.252
RaceCC	.414	.137	3.023	.003	.144	.684
SES.High	-.521	.136	-3.828	.000	-.790	-.252
MRN0 * GenderCC	-.358	.151	-2.375	.019	-.656	-.060
MRN0 * RaceCC	-.010	.162	-.065	.948	-.329	.308
MRN0 * SES.High	.596	.159	3.746	.000	.282	.911
Party0	.070	.096	.733	.465	-.119	.260
MRN0 * Party0	-.009	.094	-.097	.923	-.195	.177

Parameter Estimates

Dependent Variable: Finance_Tot

Parameter	Partial Eta Squared
Intercept	.657
MRN0	.016
GenderCC	.000
RaceCC	.052
SES.High	.080
MRN0 * GenderCC	.032
MRN0 * RaceCC	.000
MRN0 * SES.High	.077
Party0	.003
MRN0 * Party0	.000

```
UNIANOVA Finance_Tot WITH GenderCC MRN.Low RaceCC SES0 Party0
 /PRINT=ETASQ PARAMETER
 /DESIGN=GenderCC MRN.Low RaceCC SES0
   MRN.Low*GenderCC MRN.Low*RaceCC MRN.Low*SES0 Party0 MRN.Low*Party0.
```

Univariate Analysis of Variance

Notes

Output Created		15-DEC-2021 13:06:16
Comments		
Input	Data	C: \Users\njs5478\Dropbox\HM and COVID\0. Revise and Resubmit\2. R and R Data\Study 1a\Study1a_Data.sav
	Active Dataset	DataSet1
	Filter	Inclusion = 1 (FILTER)
	Weight	<none>
	Split File	<none>
	N of Rows in Working Data File	178
Missing Value Handling	Definition of Missing	User-defined missing values are treated as missing.
	Cases Used	Statistics are based on all cases with valid data for all variables in the model.
Syntax		UNIANOVA Finance_Tot WITH GenderCC MRN.Low RaceCC SES0 Party0 /PRINT=ETASQ PARAMETER /DESIGN=GenderCC MRN.Low RaceCC SES0 MRN.Low*GenderCC MRN.Low*RaceCC MRN.Low*SES0 Party0 MRN.Low*Party0.
Resources	Processor Time	00:00:00.00
	Elapsed Time	00:00:00.00

Tests of Between-Subjects Effects

Dependent Variable: Finance_Tot

Source	Type III Sum of Squares	df	Mean Square	F	Sig.
Corrected Model	102.822[a]	9	11.425	5.266	.000
Intercept	607.798	1	607.798	280.131	.000
GenderCC	5.816	1	5.816	2.681	.103
MRN.Low	.676	1	.676	.312	.577
RaceCC	10.545	1	10.545	4.860	.029
SES0	71.797	1	71.797	33.091	.000
GenderCC * MRN.Low	12.233	1	12.233	5.638	.019
MRN.Low * RaceCC	.009	1	.009	.004	.948
MRN.Low * SES0	30.452	1	30.452	14.035	.000
Party0	.809	1	.809	.373	.542
MRN.Low * Party0	.020	1	.020	.009	.923
Error	364.509	168	2.170		
Total	3310.000	178			
Corrected Total	467.331	177			

Tests of Between-Subjects Effects

Dependent Variable: Finance_Tot

Source	Partial Eta Squared
Corrected Model	.220
Intercept	.625
GenderCC	.016
MRN.Low	.002
RaceCC	.028
SES0	.165
GenderCC * MRN.Low	.032
MRN.Low * RaceCC	.000
MRN.Low * SES0	.077
Party0	.002
MRN.Low * Party0	.000
Error	
Total	
Corrected Total	

a. R Squared = .220 (Adjusted R Squared = .178)

Parameter Estimates

Dependent Variable: Finance_Tot

Parameter	B	Std. Error	t	Sig.	95% Confidence Interval Lower Bound	95% Confidence Interval Upper Bound
Intercept	3.949	.236	16.737	.000	3.483	4.414
GenderCC	.325	.198	1.637	.103	-.067	.716
MRN.Low	-.107	.191	-.558	.577	-.483	.270
RaceCC	.423	.192	2.205	.029	.044	.803
SES0	-1.061	.184	-5.752	.000	-1.425	-.697
GenderCC * MRN.Low	-.358	.151	-2.375	.019	-.656	-.060
MRN.Low * RaceCC	-.010	.162	-.065	.948	-.329	.308
MRN.Low * SES0	.596	.159	3.746	.000	.282	.911
Party0	.079	.129	.611	.542	-.176	.333
MRN.Low * Party0	-.009	.094	-.097	.923	-.195	.177

Parameter Estimates

Dependent Variable: Finance_Tot

Parameter	Partial Eta Squared
Intercept	.625
GenderCC	.016
MRN.Low	.002
RaceCC	.028
SES0	.165
GenderCC * MRN.Low	.032
MRN.Low * RaceCC	.000
MRN.Low * SES0	.077
Party0	.002
MRN.Low * Party0	.000

```
UNIANOVA Finance_Tot WITH GenderCC MRN.High RaceCC SES0 Party0
 /PRINT=ETASQ PARAMETER
 /DESIGN=MRN.High GenderCC RaceCC SES0
   MRN.High*GenderCC MRN.High*RaceCC MRN.High*SES0 Party0 MRN.High*Party0.
```

Univariate Analysis of Variance

Notes

Output Created		15-DEC-2021 13:06:16
Comments		
Input	Data	C: \Users\njs5478\Dropbox\HM and COVID\0. Revise and Resubmit\2. R and R Data\Study 1a\Study1a_Data.sav
	Active Dataset	DataSet1
	Filter	Inclusion = 1 (FILTER)
	Weight	<none>
	Split File	<none>
	N of Rows in Working Data File	178
Missing Value Handling	Definition of Missing	User-defined missing values are treated as missing.
	Cases Used	Statistics are based on all cases with valid data for all variables in the model.
Syntax		UNIANOVA Finance_Tot WITH GenderCC MRN.High RaceCC SES0 Party0 /PRINT=ETASQ PARAMETER /DESIGN=MRN.High GenderCC RaceCC SES0 MRN.High*GenderCC MRN.High*RaceCC MRN.High*SES0 Party0 MRN.High*Party0.
Resources	Processor Time	00:00:00.02
	Elapsed Time	00:00:00.02

Tests of Between-Subjects Effects

Dependent Variable: Finance_Tot

Source	Type III Sum of Squares	df	Mean Square	F	Sig.
Corrected Model	102.822[a]	9	11.425	5.266	.000
Intercept	603.251	1	603.251	278.035	.000
MRN.High	.676	1	.676	.312	.577
GenderCC	7.477	1	7.477	3.446	.065
RaceCC	8.181	1	8.181	3.771	.054
SES0	.018	1	.018	.008	.928
GenderCC * MRN.High	12.233	1	12.233	5.638	.019
MRN.High * RaceCC	.009	1	.009	.004	.948
MRN.High * SES0	30.452	1	30.452	14.035	.000
Party0	.511	1	.511	.236	.628
MRN.High * Party0	.020	1	.020	.009	.923
Error	364.509	168	2.170		
Total	3310.000	178			
Corrected Total	467.331	177			

Tests of Between-Subjects Effects

Dependent Variable: Finance_Tot

Source	Partial Eta Squared
Corrected Model	.220
Intercept	.623
MRN.High	.002
GenderCC	.020
RaceCC	.022
SES0	.000
GenderCC * MRN.High	.032
MRN.High * RaceCC	.000
MRN.High * SES0	.077
Party0	.001
MRN.High * Party0	.000
Error	
Total	
Corrected Total	

a. R Squared = .220 (Adjusted R Squared = .178)

Parameter Estimates

Dependent Variable: Finance_Tot

Parameter	B	Std. Error	t	Sig.	95% Confidence Interval	
					Lower Bound	Upper Bound
Intercept	3.756	.225	16.674	.000	3.311	4.200
MRN.High	-.107	.191	-.558	.577	-.483	.270
GenderCC	-.324	.174	-1.856	.065	-.668	.021
RaceCC	.405	.208	1.942	.054	-.007	.816
SES0	.019	.211	.091	.928	-.398	.436
GenderCC * MRN.High	-.358	.151	-2.375	.019	-.656	-.060
MRN.High * RaceCC	-.010	.162	-.065	.948	-.329	.308
MRN.High * SES0	.596	.159	3.746	.000	.282	.911
Party0	.062	.128	.486	.628	-.191	.315
MRN.High * Party0	-.009	.094	-.097	.923	-.195	.177

Parameter Estimates

Dependent Variable: Finance_Tot

Parameter	Partial Eta Squared
Intercept	.623
MRN.High	.002
GenderCC	.020
RaceCC	.022
SES0	.000
GenderCC * MRN.High	.032
MRN.High * RaceCC	.000
MRN.High * SES0	.077
Party0	.001
MRN.High * Party0	.000
