# Supplementary material for: Men’s and women’s endorsement of hegemonic masculinity and responses to COVID-19
Source: J Health Psychol. 2022 Mar 11;28(3):251–66. doi: 10.1177/13591053221081905 (PMC9982413; doi:10.1177/13591053221081905)
Supplement: sj-pdf-2-hpq-10.1177_13591053221081905 – for Men’s and women’s endorsement of hegemonic masculinity and responses to COVID-19 [file sj-pdf-2-hpq-10.1177_13591053221081905.pdf]

```
 * Encoding: UTF-8.

**COVD-19 Study 1b**

***Variable Creation***

**Male Role Norms**

RECODE Tough_8 Fem_6 (1=7) (2=6) (3=5) (4=4) (5=3) (6=2) (7=1) INTO Tough_8_Recode Fem_6_
Recode.
EXECUTE.

COMPUTE MRN=(Power_1 + Power_2 + Power_3 + Power_4 + Power_5 + Power_6 + Power_7 + Power_
8 +
    Power_9  + Power_10 + Power_11 + Tough_1 + Tough_2 + Tough_3 + Tough_4 + Tough_5 + To
ugh_6 +
    Tough_7 + Tough_8_Recode + Fem_1 + Fem_2 + Fem_3 + Fem_4 + Fem_5 + Fem_6_Recode + Fem
_7)/26.
EXECUTE.

COMPUTE Power=(Power_1 + Power_2 + Power_3 + Power_4 + Power_5 + Power_6 + Power_7 + Powe
r_8 +
    Power_9  + Power_10 + Power_11)/11.
EXECUTE.

COMPUTE Tough=(Tough_1 + Tough_2 + Tough_3 + Tough_4 + Tough_5 + Tough_6 +
    Tough_7 + Tough_8_Recode)/8.
EXECUTE.

COMPUTE Fem=(Fem_1 + Fem_2 + Fem_3 + Fem_4 + Fem_5 + Fem_6_Recode + Fem_7)/7.
EXECUTE.

RELIABILITY
  /VARIABLES=Power_1 Power_2 Power_3 Power_4 Power_5 Power_6 Power_7 Power_8 Power_9 Powe
r_10
    Power_11 Tough_1 Tough_2 Tough_3 Tough_4 Tough_5 Tough_6 Tough_7 Tough_8_Recode Fem_1
 Fem_2 Fem_3
    Fem_4 Fem_5 Fem_6_Recode Fem_7
  /SCALE('ALL VARIABLES') ALL
  /MODEL=ALPHA.
```

Reliability

Notes

Output Created		15-DEC-2021 13:07:54
Comments		
Input	Data	C:\Users\njs5478\Dropbox\HM and COVID\0. Revise and Resubmit\2. R and R Data\Study 1b\Study1b_Data.sav
	Active Dataset	DataSet1
	Filter	<none>
	Weight	<none>
	Split File	<none>
	N of Rows in Working Data File	241
	Matrix Input	
Missing Value Handling	Definition of Missing	User-defined missing values are treated as missing.
	Cases Used	Statistics are based on all cases with valid data for all variables in the procedure.
Syntax		RELIABILITY /VARIABLES=Power_1 Power_2 Power_3 Power_4 Power_5 Power_6 Power_7 Power_8 Power_9 Power_10 Power_11 Tough_1 Tough_2 Tough_3 Tough_4 Tough_5 Tough_6 Tough_7 Tough_8_Recode Fem_1 Fem_2 Fem_3 Fem_4 Fem_5 Fem_6_Recode Fem_7 /SCALE('ALL VARIABLES') ALL /MODEL=ALPHA.
Resources	Processor Time	00:00:00.00
	Elapsed Time	00:00:00.00

```
[DataSet1] C:\Users\njs5478\Dropbox\HM and COVID\0. Revise and Resubmit\2. R and R Data\Study 1b\Study1b_Data.sav
```

Scale: ALL VARIABLES

Case Processing Summary

		N	%
Cases	Valid	240	99.6
	Excluded[a]	1	.4
	Total	241	100.0

a. Listwise deletion based on all variables in the procedure.

Reliability Statistics

Cronbach's Alpha	N of Items
.909	26

```
 *Male Gender Role Stress*

COMPUTE MGRS=(MGRS1 + MGRS2 + MGRS3 + MGRS4 + MGRS5 + MGRS6 + MGRS7 + MGRS8 + MGRS9 + MGR
S10 + MGRS11 + MGRS12 + MGRS13 +
    MGRS14 + MGRS15 + MGRS16 + MGRS17 + MGRS18 + MGRS19 + MGRS20 + MGRS21 + MGRS22 + MGRS
23 + MGRS24 + MGRS25 + MGRS26 + MGRS27 +
    MGRS28 + MGRS29 + MGRS30 + MGRS31 + MGRS32 + MGRS33 + MGRS34 + MGRS35 + MGRS36 + MGRS
37 + MGRS38 + MGRS39 + MGRS40)/40.

RELIABILITY
  /VARIABLES=MGRS1 MGRS2 MGRS3 MGRS4 MGRS5 MGRS6 MGRS7 MGRS8 MGRS9 MGRS10 MGRS11 MGRS12 M
GRS13
    MGRS14 MGRS15 MGRS16 MGRS17 MGRS18 MGRS19 MGRS20 MGRS21 MGRS22 MGRS23 MGRS24 MGRS25 M
GRS26 MGRS27
    MGRS28 MGRS29 MGRS30 MGRS31 MGRS32 MGRS33 MGRS34 MGRS35 MGRS36 MGRS37 MGRS38 MGRS39 M
GRS40
  /SCALE('ALL VARIABLES') ALL
  /MODEL=ALPHA.
```

Reliability

Notes

Output Created		15-DEC-2021 13:07:54
Comments		
Input	Data	C:\Users\njs5478\Dropbox\HM and COVID\0. Revise and Resubmit\2. R and R Data\Study 1b\Study1b_Data.sav
	Active Dataset	DataSet1
	Filter	<none>
	Weight	<none>
	Split File	<none>
	N of Rows in Working Data File	241
	Matrix Input	
Missing Value Handling	Definition of Missing	User-defined missing values are treated as missing.
	Cases Used	Statistics are based on all cases with valid data for all variables in the procedure.
Syntax		RELIABILITY /VARIABLES=MGRS1 MGRS2 MGRS3 MGRS4 MGRS5 MGRS6 MGRS7 MGRS8 MGRS9 MGRS10 MGRS11 MGRS12 MGRS13 MGRS14 MGRS15 MGRS16 MGRS17 MGRS18 MGRS19 MGRS20 MGRS21 MGRS22 MGRS23 MGRS24 MGRS25 MGRS26 MGRS27 MGRS28 MGRS29 MGRS30 MGRS31 MGRS32 MGRS33 MGRS34 MGRS35 MGRS36 MGRS37 MGRS38 MGRS39 MGRS40 /SCALE('ALL VARIABLES') ALL /MODEL=ALPHA.

Notes

Resources	Processor Time	00:00:00.00
	Elapsed Time	00:00:00.00

Scale: ALL VARIABLES

Case Processing Summary

		N	%
Cases	Valid	240	99.6
	Excluded[a]	1	.4
	Total	241	100.0

a. Listwise deletion based on all variables in the procedure.

Reliability Statistics

Cronbach's Alpha	N of Items
.953	40

```
 **Risk During COVID-19*

FACTOR
  /VARIABLES Risk1 Risk2 Risk3 Risk4 Risk5 Risk6 Risk7 Risk8 Risk9 Risk10 Risk11 Risk12 R
isk13
    Risk14 Risk15 Risk16 Risk17 Risk18
  /MISSING LISTWISE
  /ANALYSIS Risk1 Risk2 Risk3 Risk4 Risk5 Risk6 Risk7 Risk8 Risk9 Risk10 Risk11 Risk12 Ri
sk13
    Risk14 Risk15 Risk16 Risk17 Risk18
  /PRINT INITIAL EXTRACTION ROTATION
  /FORMAT SORT
  /PLOT EIGEN
  /CRITERIA MINEIGEN(1) ITERATE(25)
  /EXTRACTION PC
  /CRITERIA ITERATE(25)
  /ROTATION VARIMAX
  /METHOD=CORRELATION.
```

Factor Analysis

Notes

Output Created		15-DEC-2021 13:07:54
Comments		
Input	Data	C:\Users\njs5478\Dropbox\HM and COVID\0. Revise and Resubmit\2. R and R Data\Study 1b\Study1b_Data.sav
	Active Dataset	DataSet1
	Filter	<none>
	Weight	<none>
	Split File	<none>
	N of Rows in Working Data File	241
Missing Value Handling	Definition of Missing	MISSING=EXCLUDE: User-defined missing values are treated as missing.
	Cases Used	LISTWISE: Statistics are based on cases with no missing values for any variable used.

Notes

Syntax		FACTOR<br>/VARIABLES Risk1 Risk2 Risk3 Risk4 Risk5 Risk6 Risk7 Risk8 Risk9 Risk10 Risk11 Risk12 Risk13<br>Risk14 Risk15 Risk16 Risk17 Risk18<br>/MISSING LISTWISE<br>/ANALYSIS Risk1 Risk2 Risk3 Risk4 Risk5 Risk6 Risk7 Risk8 Risk9 Risk10 Risk11 Risk12 Risk13<br>Risk14 Risk15 Risk16 Risk17 Risk18<br>/PRINT INITIAL EXTRACTION ROTATION<br>/FORMAT SORT<br>/PLOT EIGEN<br>/CRITERIA MINEIGEN (1) ITERATE(25)<br>/EXTRACTION PC<br>/CRITERIA ITERATE(25)<br>/ROTATION VARIMAX<br><br>/METHOD=CORRELATION.
Resources	Processor Time	00:00:03.41
	Elapsed Time	00:00:01.29
	Maximum Memory Required	40024 (39.086K) bytes

Communalities

	Initial	Extraction
For each of the following statements, please indicate how likely or unlikely you would be to engage in each activity or behavior during the period of COVID-19 (Coronavirus) social isolation. - Defending to family and close friends the belief that CoVID-19 is a serious risk.	1.000	.468
For each of the following statements, please indicate how likely or unlikely you would be to engage in each activity or behavior during the period of COVID-19 (Coronavirus) social isolation. - Disagreeing with your boss’s decision that you should work remotely.	1.000	.431
For each of the following statements, please indicate how likely or unlikely you would be to engage in each activity or behavior during the period of COVID-19 (Coronavirus) social isolation. - Defending the need to self-quarantine on social media.	1.000	.634

Communalities

	Initial	Extraction
For each of the following statements, please indicate how likely or unlikely you would be to engage in each activity or behavior during the period of COVID-19 (Coronavirus) social isolation. - Cancelling a planned vacation because you were planning to fly there.	1.000	.446
For each of the following statements, please indicate how likely or unlikely you would be to engage in each activity or behavior during the period of COVID-19 (Coronavirus) social isolation. - Playing a pick-up sport with friends.	1.000	.539
For each of the following statements, please indicate how likely or unlikely you would be to engage in each activity or behavior during the period of COVID-19 (Coronavirus) social isolation. - Continuing to have friends over who do not live with you.	1.000	.603

Communalities

	Initial	Extraction
For each of the following statements, please indicate how likely or unlikely you would be to engage in each activity or behavior during the period of COVID-19 (Coronavirus) social isolation. - Regularly going to pick up take-out food.	1.000	.779
For each of the following statements, please indicate how likely or unlikely you would be to engage in each activity or behavior during the period of COVID-19 (Coronavirus) social isolation. - Asking the person behind you in line to step away to maintain a six-foot distance.	1.000	.566
For each of the following statements, please indicate how likely or unlikely you would be to engage in each activity or behavior during the period of COVID-19 (Coronavirus) social isolation. - Not wearing a mask when you go out in public.	1.000	.595

Communalities

	Initial	Extraction
For each of the following statements, please indicate how likely or unlikely you would be to engage in each activity or behavior during the period of COVID-19 (Coronavirus) social isolation. - Not washing your hands upon reentry to your home.	1.000	.379
For each of the following statements, please indicate how likely or unlikely you would be to engage in each activity or behavior during the period of COVID-19 (Coronavirus) social isolation. - Refusing to shake hands with acquaintances.	1.000	.580
For each of the following statements, please indicate how likely or unlikely you would be to engage in each activity or behavior during the period of COVID-19 (Coronavirus) social isolation. - Hugging friends you run into while grocery shopping.	1.000	.547

Communalities

	Initial	Extraction
For each of the following statements, please indicate how likely or unlikely you would be to engage in each activity or behavior during the period of COVID-19 (Coronavirus) social isolation. - Volunteering to distribute food at the local food bank.	1.000	.755
For each of the following statements, please indicate how likely or unlikely you would be to engage in each activity or behavior during the period of COVID-19 (Coronavirus) social isolation. - Volunteering at an understaffed medical facility.	1.000	.696
For each of the following statements, please indicate how likely or unlikely you would be to engage in each activity or behavior during the period of COVID-19 (Coronavirus) social isolation. - Distributing medical supplies to the homes of people who have been diagnosed with coronavirus so they do not have to go out in public.	1.000	.677

Communalities

	Initial	Extraction
For each of the following statements, please indicate how likely or unlikely you would be to engage in each activity or behavior during the period of COVID-19 (Coronavirus) social isolation. - Leaving your home when you feel ill.	1.000	.560
For each of the following statements, please indicate how likely or unlikely you would be to engage in each activity or behavior during the period of COVID-19 (Coronavirus) social isolation. - Going out in public for a break from care-taking for a friend/family member with coronavirus.	1.000	.321
For each of the following statements, please indicate how likely or unlikely you would be to engage in each activity or behavior during the period of COVID-19 (Coronavirus) social isolation. - Grocery shopping during hours reserved for high-risk individuals.	1.000	.524

Extraction Method: Principal Component Analysis.

Total Variance Explained

Component	Initial Eigenvalues			Extraction Sums of Squared Loadings		
	Total	% of Variance	Cumulative %	Total	% of Variance	Cumulative %
1	4.932	27.403	27.403	4.932	27.403	27.403
2	2.386	13.256	40.658	2.386	13.256	40.658
3	1.680	9.331	49.989	1.680	9.331	49.989
4	1.102	6.124	56.113	1.102	6.124	56.113
5	.872	4.846	60.959			
6	.854	4.744	65.703			
7	.787	4.374	70.077			
8	.722	4.009	74.086			
9	.657	3.649	77.734			
10	.563	3.126	80.861			
11	.550	3.054	83.915			
12	.508	2.822	86.737			
13	.499	2.770	89.507			
14	.425	2.363	91.870			
15	.412	2.289	94.159			
16	.379	2.107	96.266			
17	.344	1.913	98.179			
18	.328	1.821	100.000			

Total Variance Explained

Component	Rotation Sums of Squared Loadings		
	Total	% of Variance	Cumulative %
1	3.398	18.877	18.877
2	2.899	16.104	34.981
3	2.272	12.625	47.606
4	1.531	8.507	56.113
5			
6			
7			
8			
9			
10			
11			
12			
13			
14			
15			
16			
17			
18			

Extraction Method: Principal Component Analysis.

Scree Plot

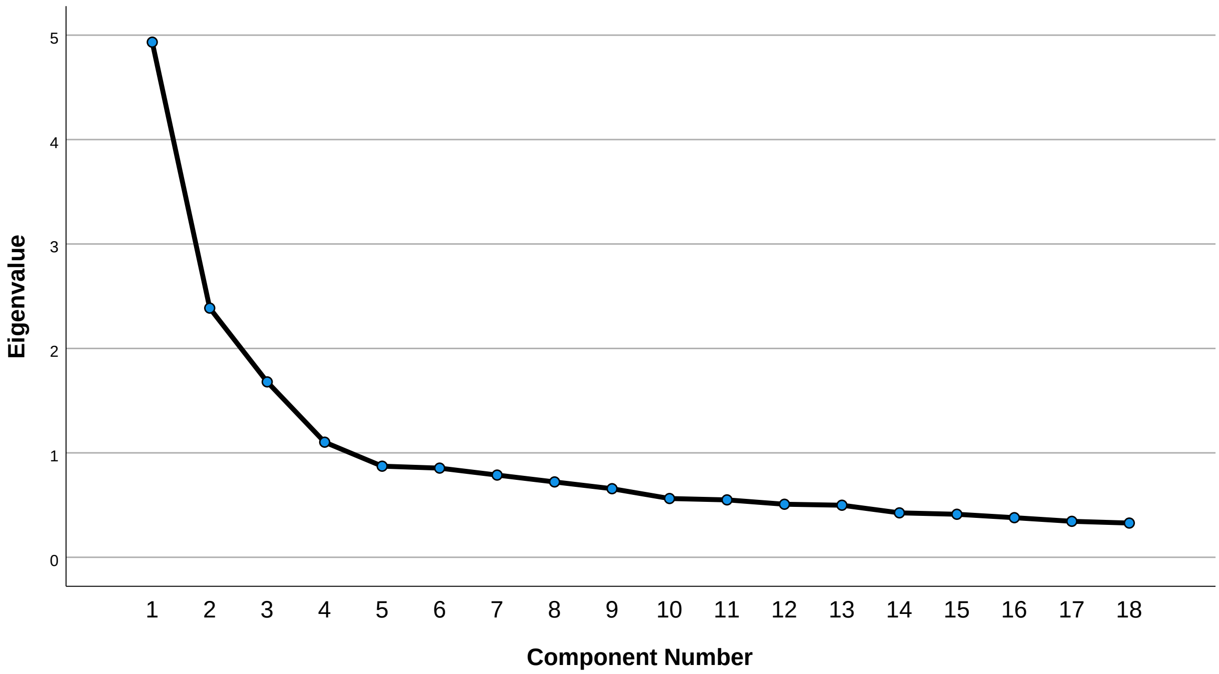

Component Matrix[a]

	Component			
	1	2	3	4
For each of the following statements, please indicate how likely or unlikely you would be to engage in each activity or behavior during the period of COVID-19 (Coronavirus) social isolation. - Not wearing a mask when you go out in public.	.696	.032	.324	-.063

Component Matrix[a]

	Component			
	1	2	3	4
For each of the following statements, please indicate how likely or unlikely you would be to engage in each activity or behavior during the period of COVID-19 (Coronavirus) social isolation. - Refusing to shake hands with acquaintances.	-.683	.101	.310	.083
For each of the following statements, please indicate how likely or unlikely you would be to engage in each activity or behavior during the period of COVID-19 (Coronavirus) social isolation. - Playing a pick-up sport with friends.	.679	.073	-.270	.013
For each of the following statements, please indicate how likely or unlikely you would be to engage in each activity or behavior during the period of COVID-19 (Coronavirus) social isolation. - Hugging friends you run into while grocery shopping.	.664	.291	-.060	.136

Component Matrix[a]

	Component			
	1	2	3	4
For each of the following statements, please indicate how likely or unlikely you would be to engage in each activity or behavior during the period of COVID-19 (Coronavirus) social isolation. - Asking the person behind you in line to step away to maintain a six-foot distance.	-.615	.233	.357	.075
For each of the following statements, please indicate how likely or unlikely you would be to engage in each activity or behavior during the period of COVID-19 (Coronavirus) social isolation. - Defending to family and close friends the belief that CoVID-19 is a serious risk.	-.614	.147	.124	.230
For each of the following statements, please indicate how likely or unlikely you would be to engage in each activity or behavior during the period of COVID-19 (Coronavirus) social isolation. - Continuing to have friends over who do not live with you.	.604	.127	-.356	.309

Component Matrix[a]

	Component			
	1	2	3	4
For each of the following statements, please indicate how likely or unlikely you would be to engage in each activity or behavior during the period of COVID-19 (Coronavirus) social isolation. - Cancelling a planned vacation because you were planning to fly there.	-.595	-.026	.078	.292
For each of the following statements, please indicate how likely or unlikely you would be to engage in each activity or behavior during the period of COVID-19 (Coronavirus) social isolation. - Disagreeing with your boss's decision that you should work remotely.	.545	.170	.290	.146
For each of the following statements, please indicate how likely or unlikely you would be to engage in each activity or behavior during the period of COVID-19 (Coronavirus) social isolation. - Not washing your hands upon reentry to your home.	.532	.045	.304	-.039

Component Matrix[a]

	Component			
	1	2	3	4
For each of the following statements, please indicate how likely or unlikely you would be to engage in each activity or behavior during the period of COVID-19 (Coronavirus) social isolation. - Leaving your home when you feel ill.	.531	.262	.458	-.006
For each of the following statements, please indicate how likely or unlikely you would be to engage in each activity or behavior during the period of COVID-19 (Coronavirus) social isolation. - Defending the need to self-quarantine on social media.	-.522	.398	.317	.321
For each of the following statements, please indicate how likely or unlikely you would be to engage in each activity or behavior during the period of COVID-19 (Coronavirus) social isolation. - Going out in public for a break from care-taking for a friend/family member with coronavirus.	.357	.289	.327	.054

Component Matrix[a]

	Component			
	1	2	3	4
For each of the following statements, please indicate how likely or unlikely you would be to engage in each activity or behavior during the period of COVID-19 (Coronavirus) social isolation. - Volunteering to distribute food at the local food bank.	-.147	.802	-.277	-.114
For each of the following statements, please indicate how likely or unlikely you would be to engage in each activity or behavior during the period of COVID-19 (Coronavirus) social isolation. - Volunteering at an understaffed medical facility.	-.018	.771	-.222	-.228
For each of the following statements, please indicate how likely or unlikely you would be to engage in each activity or behavior during the period of COVID-19 (Coronavirus) social isolation. - Distributing medical supplies to the homes of people who have been diagnosed with coronavirus so they do not have to go out in public.	-.226	.755	-.225	-.072

Component Matrix[a]

	Component			
	1	2	3	4
For each of the following statements, please indicate how likely or unlikely you would be to engage in each activity or behavior during the period of COVID-19 (Coronavirus) social isolation. - Grocery shopping during hours reserved for high-risk individuals.	.383	.198	.570	-.117
For each of the following statements, please indicate how likely or unlikely you would be to engage in each activity or behavior during the period of COVID-19 (Coronavirus) social isolation. - Regularly going to pick up take-out food.	.351	.062	-.177	.788

Extraction Method: Principal Component Analysis.

a. 4 components extracted.

Rotated Component Matrix[a]

	Component			
	1	2	3	4
For each of the following statements, please indicate how likely or unlikely you would be to engage in each activity or behavior during the period of COVID-19 (Coronavirus) social isolation. - Defending the need to self-quarantine on social media.	.753	.046	.238	.090
For each of the following statements, please indicate how likely or unlikely you would be to engage in each activity or behavior during the period of COVID-19 (Coronavirus) social isolation. - Refusing to shake hands with acquaintances.	.717	-.146	.032	-.210
For each of the following statements, please indicate how likely or unlikely you would be to engage in each activity or behavior during the period of COVID-19 (Coronavirus) social isolation. - Asking the person behind you in line to step away to maintain a six-foot distance.	.715	-.028	.133	-.193

Rotated Component Matrix[a]

	Component			
	1	2	3	4
For each of the following statements, please indicate how likely or unlikely you would be to engage in each activity or behavior during the period of COVID-19 (Coronavirus) social isolation. - Defending to family and close friends the belief that CoVID-19 is a serious risk.	.636	-.230	.100	.003
For each of the following statements, please indicate how likely or unlikely you would be to engage in each activity or behavior during the period of COVID-19 (Coronavirus) social isolation. - Playing a pick-up sport with friends.	-.620	.228	.094	.306
For each of the following statements, please indicate how likely or unlikely you would be to engage in each activity or behavior during the period of COVID-19 (Coronavirus) social isolation. - Cancelling a planned vacation because you were planning to fly there.	.586	-.309	-.057	.055

Rotated Component Matrix[a]

	Component			
	1	2	3	4
For each of the following statements, please indicate how likely or unlikely you would be to engage in each activity or behavior during the period of COVID-19 (Coronavirus) social isolation. - Leaving your home when you feel ill.	-.106	.737	.041	.062
For each of the following statements, please indicate how likely or unlikely you would be to engage in each activity or behavior during the period of COVID-19 (Coronavirus) social isolation. - Grocery shopping during hours reserved for high-risk individuals.	.005	.713	-.019	-.123
For each of the following statements, please indicate how likely or unlikely you would be to engage in each activity or behavior during the period of COVID-19 (Coronavirus) social isolation. - Not wearing a mask when you go out in public.	-.365	.663	-.128	.069

Rotated Component Matrix[a]

	Component			
	1	2	3	4
For each of the following statements, please indicate how likely or unlikely you would be to engage in each activity or behavior during the period of COVID-19 (Coronavirus) social isolation. - Disagreeing with your boss’s decision that you should work remotely.	-.162	.590	-.020	.239
For each of the following statements, please indicate how likely or unlikely you would be to engage in each activity or behavior during the period of COVID-19 (Coronavirus) social isolation. - Not washing your hands upon reentry to your home.	-.243	.555	-.099	.048
For each of the following statements, please indicate how likely or unlikely you would be to engage in each activity or behavior during the period of COVID-19 (Coronavirus) social isolation. - Going out in public for a break from care-taking for a friend/family member with coronavirus.	-.016	.545	.113	.103

Rotated Component Matrix[a]

	Component			
	1	2	3	4
For each of the following statements, please indicate how likely or unlikely you would be to engage in each activity or behavior during the period of COVID-19 (Coronavirus) social isolation. - Hugging friends you run into while grocery shopping.	-.410	.441	.199	.381
For each of the following statements, please indicate how likely or unlikely you would be to engage in each activity or behavior during the period of COVID-19 (Coronavirus) social isolation. - Volunteering to distribute food at the local food bank.	.085	-.031	.863	.035
For each of the following statements, please indicate how likely or unlikely you would be to engage in each activity or behavior during the period of COVID-19 (Coronavirus) social isolation. - Volunteering at an understaffed medical facility.	-.032	.078	.829	-.048

Rotated Component Matrix[a]

	Component			
	1	2	3	4
For each of the following statements, please indicate how likely or unlikely you would be to engage in each activity or behavior during the period of COVID-19 (Coronavirus) social isolation. - Distributing medical supplies to the homes of people who have been diagnosed with coronavirus so they do not have to go out in public.	.177	-.055	.801	.028
For each of the following statements, please indicate how likely or unlikely you would be to engage in each activity or behavior during the period of COVID-19 (Coronavirus) social isolation. - Regularly going to pick up take-out food.	-.035	.084	-.078	.875
For each of the following statements, please indicate how likely or unlikely you would be to engage in each activity or behavior during the period of COVID-19 (Coronavirus) social isolation. - Continuing to have friends over who do not live with you.	-.484	.132	.118	.581

Extraction Method: Principal Component Analysis.
Rotation Method: Varimax with Kaiser Normalization.

a. Rotation converged in 6 iterations.

Component Transformation Matrix

Component	1	2	3	4
1	-.739	.594	-.089	.307
2	.203	.322	.915	.130
3	.515	.737	-.334	-.283
4	.385	-.017	-.207	.899

Extraction Method: Principal Component Analysis.
Rotation Method: Varimax with Kaiser Normalization.

```
*Force Two Factors*

FACTOR
  /VARIABLES Risk1 Risk2 Risk3 Risk4 Risk5 Risk6 Risk7 Risk8 Risk9 Risk10 Risk11 Risk12 R
isk13
    Risk14 Risk15 Risk16 Risk17 Risk18
  /MISSING LISTWISE
  /ANALYSIS Risk1 Risk2 Risk3 Risk4 Risk5 Risk6 Risk7 Risk8 Risk9 Risk10 Risk11 Risk12 Ri
sk13
    Risk14 Risk15 Risk16 Risk17 Risk18
  /PRINT INITIAL EXTRACTION ROTATION
  /FORMAT SORT
  /PLOT EIGEN
  /CRITERIA FACTORS(2) ITERATE(25)
  /EXTRACTION PC
  /CRITERIA ITERATE(25)
  /ROTATION VARIMAX
  /METHOD=CORRELATION.
```

Factor Analysis

Notes

Output Created		15-DEC-2021 13:07:56
Comments		
Input	Data	C:\Users\njs5478\Dropbox\HM and COVID\0. Revise and Resubmit\2. R and R Data\Study 1b\Study1b_Data.sav
	Active Dataset	DataSet1
	Filter	<none>
	Weight	<none>
	Split File	<none>
	N of Rows in Working Data File	241
Missing Value Handling	Definition of Missing	MISSING=EXCLUDE: User-defined missing values are treated as missing.
	Cases Used	LISTWISE: Statistics are based on cases with no missing values for any variable used.

Notes

Syntax		FACTOR<br>/VARIABLES Risk1 Risk2 Risk3 Risk4 Risk5 Risk6 Risk7 Risk8 Risk9 Risk10 Risk11 Risk12 Risk13<br>Risk14 Risk15 Risk16 Risk17 Risk18<br>/MISSING LISTWISE<br>/ANALYSIS Risk1 Risk2 Risk3 Risk4 Risk5 Risk6 Risk7 Risk8 Risk9 Risk10 Risk11 Risk12 Risk13<br>Risk14 Risk15 Risk16 Risk17 Risk18<br>/PRINT INITIAL EXTRACTION ROTATION<br>/FORMAT SORT<br>/PLOT EIGEN<br>/CRITERIA FACTORS(2) ITERATE(25)<br>/EXTRACTION PC<br>/CRITERIA ITERATE(25)<br>/ROTATION VARIMAX<br><br>/METHOD=CORRELATION.
Resources	Processor Time	00:00:00.36
	Elapsed Time	00:00:00.17
	Maximum Memory Required	40024 (39.086K) bytes

Communalities

	Initial	Extraction
For each of the following statements, please indicate how likely or unlikely you would be to engage in each activity or behavior during the period of COVID-19 (Coronavirus) social isolation. - Defending to family and close friends the belief that CoVID-19 is a serious risk.	1.000	.399
For each of the following statements, please indicate how likely or unlikely you would be to engage in each activity or behavior during the period of COVID-19 (Coronavirus) social isolation. - Disagreeing with your boss's decision that you should work remotely.	1.000	.326
For each of the following statements, please indicate how likely or unlikely you would be to engage in each activity or behavior during the period of COVID-19 (Coronavirus) social isolation. - Defending the need to self-quarantine on social media.	1.000	.431

Communalities

	Initial	Extraction
For each of the following statements, please indicate how likely or unlikely you would be to engage in each activity or behavior during the period of COVID-19 (Coronavirus) social isolation. - Cancelling a planned vacation because you were planning to fly there.	1.000	.354
For each of the following statements, please indicate how likely or unlikely you would be to engage in each activity or behavior during the period of COVID-19 (Coronavirus) social isolation. - Playing a pick-up sport with friends.	1.000	.466
For each of the following statements, please indicate how likely or unlikely you would be to engage in each activity or behavior during the period of COVID-19 (Coronavirus) social isolation. - Continuing to have friends over who do not live with you.	1.000	.381

Communalities

	Initial	Extraction
For each of the following statements, please indicate how likely or unlikely you would be to engage in each activity or behavior during the period of COVID-19 (Coronavirus) social isolation. - Regularly going to pick up take-out food.	1.000	.127
For each of the following statements, please indicate how likely or unlikely you would be to engage in each activity or behavior during the period of COVID-19 (Coronavirus) social isolation. - Asking the person behind you in line to step away to maintain a six-foot distance.	1.000	.433
For each of the following statements, please indicate how likely or unlikely you would be to engage in each activity or behavior during the period of COVID-19 (Coronavirus) social isolation. - Not wearing a mask when you go out in public.	1.000	.486

Communalities

	Initial	Extraction
For each of the following statements, please indicate how likely or unlikely you would be to engage in each activity or behavior during the period of COVID-19 (Coronavirus) social isolation. - Not washing your hands upon reentry to your home.	1.000	.286
For each of the following statements, please indicate how likely or unlikely you would be to engage in each activity or behavior during the period of COVID-19 (Coronavirus) social isolation. - Refusing to shake hands with acquaintances.	1.000	.477
For each of the following statements, please indicate how likely or unlikely you would be to engage in each activity or behavior during the period of COVID-19 (Coronavirus) social isolation. - Hugging friends you run into while grocery shopping.	1.000	.525

Communalities

	Initial	Extraction
For each of the following statements, please indicate how likely or unlikely you would be to engage in each activity or behavior during the period of COVID-19 (Coronavirus) social isolation. - Volunteering to distribute food at the local food bank.	1.000	.665
For each of the following statements, please indicate how likely or unlikely you would be to engage in each activity or behavior during the period of COVID-19 (Coronavirus) social isolation. - Volunteering at an understaffed medical facility.	1.000	.595
For each of the following statements, please indicate how likely or unlikely you would be to engage in each activity or behavior during the period of COVID-19 (Coronavirus) social isolation. - Distributing medical supplies to the homes of people who have been diagnosed with coronavirus so they do not have to go out in public.	1.000	.621

Communalities

	Initial	Extraction
For each of the following statements, please indicate how likely or unlikely you would be to engage in each activity or behavior during the period of COVID-19 (Coronavirus) social isolation. - Leaving your home when you feel ill.	1.000	.351
For each of the following statements, please indicate how likely or unlikely you would be to engage in each activity or behavior during the period of COVID-19 (Coronavirus) social isolation. - Going out in public for a break from care-taking for a friend/family member with coronavirus.	1.000	.211
For each of the following statements, please indicate how likely or unlikely you would be to engage in each activity or behavior during the period of COVID-19 (Coronavirus) social isolation. - Grocery shopping during hours reserved for high-risk individuals.	1.000	.186

Extraction Method: Principal Component Analysis.

Total Variance Explained

Component	Initial Eigenvalues			Extraction Sums of Squared Loadings		
	Total	% of Variance	Cumulative %	Total	% of Variance	Cumulative %
1	4.932	27.403	27.403	4.932	27.403	27.403
2	2.386	13.256	40.658	2.386	13.256	40.658
3	1.680	9.331	49.989			
4	1.102	6.124	56.113			
5	.872	4.846	60.959			
6	.854	4.744	65.703			
7	.787	4.374	70.077			
8	.722	4.009	74.086			
9	.657	3.649	77.734			
10	.563	3.126	80.861			
11	.550	3.054	83.915			
12	.508	2.822	86.737			
13	.499	2.770	89.507			
14	.425	2.363	91.870			
15	.412	2.289	94.159			
16	.379	2.107	96.266			
17	.344	1.913	98.179			
18	.328	1.821	100.000			

Total Variance Explained

Component	Rotation Sums of Squared Loadings		
	Total	% of Variance	Cumulative %
1	4.797	26.648	26.648
2	2.522	14.010	40.658
3			
4			
5			
6			
7			
8			
9			
10			
11			
12			
13			
14			
15			
16			
17			
18			

Extraction Method: Principal Component Analysis.

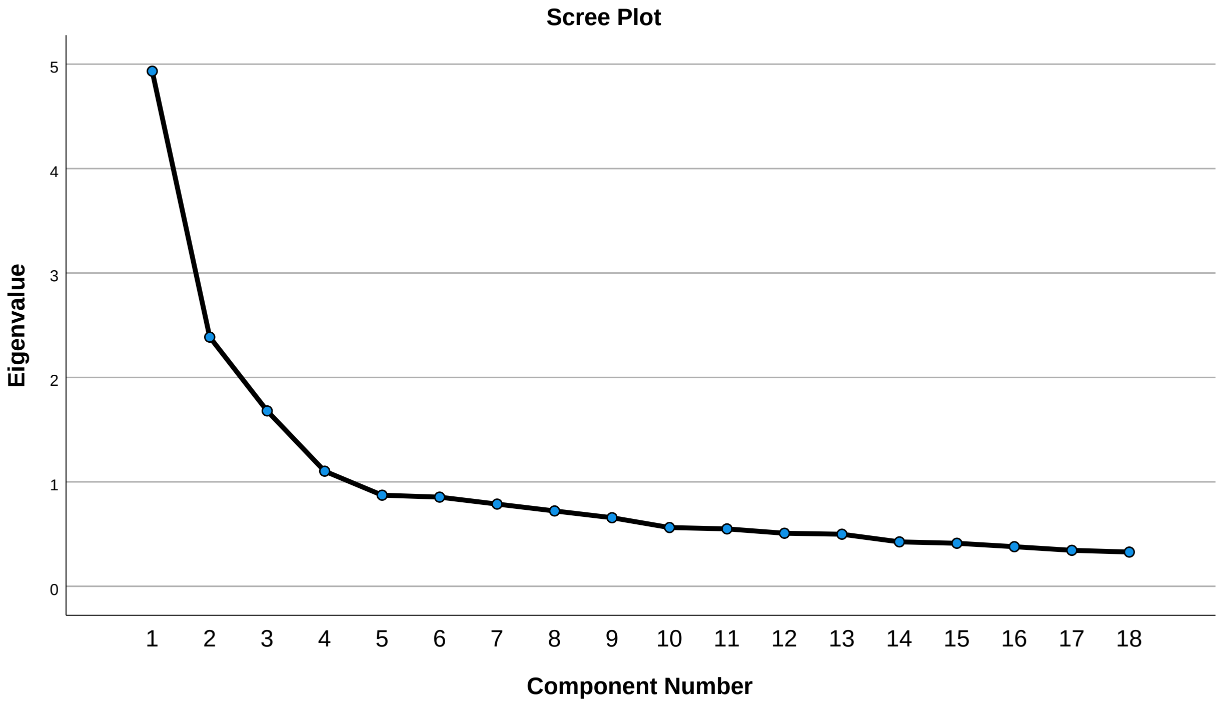

Component Matrix[a]

	Component	
	1	2
For each of the following statements, please indicate how likely or unlikely you would be to engage in each activity or behavior during the period of COVID-19 (Coronavirus) social isolation. - Not wearing a mask when you go out in public.	.696	.032

Component Matrix[a]

	Component	
	1	2
For each of the following statements, please indicate how likely or unlikely you would be to engage in each activity or behavior during the period of COVID-19 (Coronavirus) social isolation. - Refusing to shake hands with acquaintances.	-.683	.101
For each of the following statements, please indicate how likely or unlikely you would be to engage in each activity or behavior during the period of COVID-19 (Coronavirus) social isolation. - Playing a pick-up sport with friends.	.679	.073
For each of the following statements, please indicate how likely or unlikely you would be to engage in each activity or behavior during the period of COVID-19 (Coronavirus) social isolation. - Hugging friends you run into while grocery shopping.	.664	.291

Component Matrix[a]

	Component 1	Component 2
For each of the following statements, please indicate how likely or unlikely you would be to engage in each activity or behavior during the period of COVID-19 (Coronavirus) social isolation. - Asking the person behind you in line to step away to maintain a six-foot distance.	-.615	.233
For each of the following statements, please indicate how likely or unlikely you would be to engage in each activity or behavior during the period of COVID-19 (Coronavirus) social isolation. - Defending to family and close friends the belief that CoVID-19 is a serious risk.	-.614	.147
For each of the following statements, please indicate how likely or unlikely you would be to engage in each activity or behavior during the period of COVID-19 (Coronavirus) social isolation. - Continuing to have friends over who do not live with you.	.604	.127

Component Matrix[a]

	Component	
	1	2
For each of the following statements, please indicate how likely or unlikely you would be to engage in each activity or behavior during the period of COVID-19 (Coronavirus) social isolation. - Cancelling a planned vacation because you were planning to fly there.	-.595	-.026
For each of the following statements, please indicate how likely or unlikely you would be to engage in each activity or behavior during the period of COVID-19 (Coronavirus) social isolation. - Disagreeing with your boss's decision that you should work remotely.	.545	.170
For each of the following statements, please indicate how likely or unlikely you would be to engage in each activity or behavior during the period of COVID-19 (Coronavirus) social isolation. - Not washing your hands upon reentry to your home.	.532	.045

Component Matrix[a]

	Component	
	1	2
For each of the following statements, please indicate how likely or unlikely you would be to engage in each activity or behavior during the period of COVID-19 (Coronavirus) social isolation. - Leaving your home when you feel ill.	.531	.262
For each of the following statements, please indicate how likely or unlikely you would be to engage in each activity or behavior during the period of COVID-19 (Coronavirus) social isolation. - Defending the need to self-quarantine on social media.	-.522	.398
For each of the following statements, please indicate how likely or unlikely you would be to engage in each activity or behavior during the period of COVID-19 (Coronavirus) social isolation. - Grocery shopping during hours reserved for high-risk individuals.	.383	.198

Component Matrix[a]

	Component 1	Component 2
For each of the following statements, please indicate how likely or unlikely you would be to engage in each activity or behavior during the period of COVID-19 (Coronavirus) social isolation. - Going out in public for a break from care-taking for a friend/family member with coronavirus.	.357	.289
For each of the following statements, please indicate how likely or unlikely you would be to engage in each activity or behavior during the period of COVID-19 (Coronavirus) social isolation. - Regularly going to pick up take-out food.	.351	.062
For each of the following statements, please indicate how likely or unlikely you would be to engage in each activity or behavior during the period of COVID-19 (Coronavirus) social isolation. - Volunteering to distribute food at the local food bank.	-.147	.802

Component Matrix[a]

	Component	
	1	2
For each of the following statements, please indicate how likely or unlikely you would be to engage in each activity or behavior during the period of COVID-19 (Coronavirus) social isolation. - Volunteering at an understaffed medical facility.	-.018	.771
For each of the following statements, please indicate how likely or unlikely you would be to engage in each activity or behavior during the period of COVID-19 (Coronavirus) social isolation. - Distributing medical supplies to the homes of people who have been diagnosed with coronavirus so they do not have to go out in public.	-.226	.755

Extraction Method: Principal Component Analysis.

a. 2 components extracted.

Rotated Component Matrix[a]

	Component	
	1	2
For each of the following statements, please indicate how likely or unlikely you would be to engage in each activity or behavior during the period of COVID-19 (Coronavirus) social isolation. - Hugging friends you run into while grocery shopping.	.713	.129
For each of the following statements, please indicate how likely or unlikely you would be to engage in each activity or behavior during the period of COVID-19 (Coronavirus) social isolation. - Not wearing a mask when you go out in public.	.685	-.130
For each of the following statements, please indicate how likely or unlikely you would be to engage in each activity or behavior during the period of COVID-19 (Coronavirus) social isolation. - Playing a pick-up sport with friends.	.677	-.085

Rotated Component Matrix[a]

	Component	
	1	2
For each of the following statements, please indicate how likely or unlikely you would be to engage in each activity or behavior during the period of COVID-19 (Coronavirus) social isolation. - Refusing to shake hands with acquaintances.	-.642	.256
For each of the following statements, please indicate how likely or unlikely you would be to engage in each activity or behavior during the period of COVID-19 (Coronavirus) social isolation. - Continuing to have friends over who do not live with you.	.617	-.016
For each of the following statements, please indicate how likely or unlikely you would be to engage in each activity or behavior during the period of COVID-19 (Coronavirus) social isolation. - Cancelling a planned vacation because you were planning to fly there.	-.584	.112

Rotated Component Matrix[a]

	Component	
	1	2
For each of the following statements, please indicate how likely or unlikely you would be to engage in each activity or behavior during the period of COVID-19 (Coronavirus) social isolation. - Leaving your home when you feel ill.	.577	.132
For each of the following statements, please indicate how likely or unlikely you would be to engage in each activity or behavior during the period of COVID-19 (Coronavirus) social isolation. - Disagreeing with your boss's decision that you should work remotely.	.570	.039
For each of the following statements, please indicate how likely or unlikely you would be to engage in each activity or behavior during the period of COVID-19 (Coronavirus) social isolation. - Defending to family and close friends the belief that CoVID-19 is a serious risk.	-.564	.285

Rotated Component Matrix[a]

	Component	
	1	2
For each of the following statements, please indicate how likely or unlikely you would be to engage in each activity or behavior during the period of COVID-19 (Coronavirus) social isolation. - Asking the person behind you in line to step away to maintain a six-foot distance.	-.545	.369
For each of the following statements, please indicate how likely or unlikely you would be to engage in each activity or behavior during the period of COVID-19 (Coronavirus) social isolation. - Not washing your hands upon reentry to your home.	.528	-.080
For each of the following statements, please indicate how likely or unlikely you would be to engage in each activity or behavior during the period of COVID-19 (Coronavirus) social isolation. - Grocery shopping during hours reserved for high-risk individuals.	.418	.104

Rotated Component Matrix[a]

	Component	
	1	2
For each of the following statements, please indicate how likely or unlikely you would be to engage in each activity or behavior during the period of COVID-19 (Coronavirus) social isolation. - Going out in public for a break from care-taking for a friend/family member with coronavirus.	.414	.199
For each of the following statements, please indicate how likely or unlikely you would be to engage in each activity or behavior during the period of COVID-19 (Coronavirus) social isolation. - Regularly going to pick up take-out food.	.356	-.021

Rotated Component Matrix[a]

	Component	
	1	2
For each of the following statements, please indicate how likely or unlikely you would be to engage in each activity or behavior during the period of COVID-19 (Coronavirus) social isolation. - Volunteering to distribute food at the local food bank.	.042	.814
For each of the following statements, please indicate how likely or unlikely you would be to engage in each activity or behavior during the period of COVID-19 (Coronavirus) social isolation. - Distributing medical supplies to the homes of people who have been diagnosed with coronavirus so they do not have to go out in public.	-.045	.787
For each of the following statements, please indicate how likely or unlikely you would be to engage in each activity or behavior during the period of COVID-19 (Coronavirus) social isolation. - Volunteering at an understaffed medical facility.	.160	.755
For each of the following statements, please indicate how likely or unlikely you would be to engage in each activity or behavior during the period of COVID-19 (Coronavirus) social isolation. - Defending the need to self-quarantine on social media.	-.416	.507

Extraction Method: Principal Component Analysis.
Rotation Method: Varimax with Kaiser Normalization.

a. Rotation converged in 3 iterations.

Component Transformation Matrix

Component	1	2
1	.973	-.231
2	.231	.973

Extraction Method: Principal Component Analysis.
Rotation Method: Varimax with Kaiser Normalization.

```
RECODE Risk1 Risk3 Risk4 Risk8 Risk11 (1=7) (2=6) (3=5) (4=4) (5=3) (6=2) (7=1) INTO Risk
1_R Risk3_R Risk4_R Risk8_R Risk11_R.
EXECUTE.

RELIABILITY
  /VARIABLES= Risk13 Risk14 Risk15
  /SCALE('ALL VARIABLES') ALL
  /MODEL=ALPHA.
```

Reliability

Notes

Output Created		15-DEC-2021 13:07:56
Comments		
Input	Data	C:\Users\njs5478\Dropbox\HM and COVID\0. Revise and Resubmit\2. R and R Data\Study 1b\Study1b_Data.sav
	Active Dataset	DataSet1
	Filter	<none>
	Weight	<none>
	Split File	<none>
	N of Rows in Working Data File	241
	Matrix Input	
Missing Value Handling	Definition of Missing	User-defined missing values are treated as missing.
	Cases Used	Statistics are based on all cases with valid data for all variables in the procedure.
Syntax		RELIABILITY /VARIABLES= Risk13 Risk14 Risk15 /SCALE('ALL VARIABLES') ALL /MODEL=ALPHA.
Resources	Processor Time	00:00:00.00
	Elapsed Time	00:00:00.00

Scale: ALL VARIABLES

Case Processing Summary

		N	%
Cases	Valid	241	100.0
	Excluded[a]	0	.0
	Total	241	100.0

a. Listwise deletion based on all variables in the procedure.

Reliability Statistics

Cronbach's Alpha	N of Items
.798	3

```
RELIABILITY
  /VARIABLES=Risk1_R Risk2 Risk3_R Risk4_R Risk5 Risk6 Risk7 Risk8_R Risk9 Risk10 Risk11_
R Risk12 Risk16 Risk17 Risk18
  /SCALE('ALL VARIABLES') ALL
  /MODEL=ALPHA.
```

Reliability

Notes

Output Created		15-DEC-2021 13:07:56
Comments		
Input	Data	C:\Users\njs5478\Dropbox\HM and COVID\0. Revise and Resubmit\2. R and R Data\Study 1b\Study1b_Data.sav
	Active Dataset	DataSet1
	Filter	<none>
	Weight	<none>
	Split File	<none>
	N of Rows in Working Data File	241
	Matrix Input	
Missing Value Handling	Definition of Missing	User-defined missing values are treated as missing.
	Cases Used	Statistics are based on all cases with valid data for all variables in the procedure.

Notes

Syntax		RELIABILITY<br>/VARIABLES=Risk1_R Risk2 Risk3_R Risk4_R Risk5 Risk6 Risk7 Risk8_R Risk9 Risk10 Risk11_R Risk12 Risk16 Risk17 Risk18<br>/SCALE('ALL VARIABLES') ALL<br>/MODEL=ALPHA.
Resources	Processor Time	00:00:00.02
	Elapsed Time	00:00:00.01

Scale: ALL VARIABLES

Case Processing Summary

		N	%
Cases	Valid	241	100.0
	Excluded[a]	0	.0
	Total	241	100.0

a. Listwise deletion based on all variables in the procedure.

Reliability Statistics

Cronbach's Alpha	N of Items
.844	15

```
COMPUTE Risk_Rules = (Risk1_R + Risk2 + Risk3_R + Risk4_R + Risk5 + Risk6 + Risk7 + Risk8
_R + Risk9 + Risk10 + Risk11_R + Risk12 + Risk16 + Risk17 + Risk18)/15.

COMPUTE Risk_Help=(Risk13 + Risk14 + RIsk15)/3.

**Coronavirus Concern**

RECODE Concern3 (1=7) (2=6) (3=5) (4=4) (5=3) (6=2) (7=1) INTO Concern3_R.
EXECUTE.

RELIABILITY
```

```
/VARIABLES=Concern1 Concern2 Concern3_R Concern4 Concern5 Concern6
/SCALE('ALL VARIABLES') ALL
/MODEL=ALPHA
/SUMMARY=TOTAL.
```

Reliability

Notes

Output Created		15-DEC-2021 13:07:56
Comments		
Input	Data	C:\Users\njs5478\Dropbox\HM and COVID\0. Revise and Resubmit\2. R and R Data\Study 1b\Study1b_Data.sav
	Active Dataset	DataSet1
	Filter	<none>
	Weight	<none>
	Split File	<none>
	N of Rows in Working Data File	241
	Matrix Input	
Missing Value Handling	Definition of Missing	User-defined missing values are treated as missing.
	Cases Used	Statistics are based on all cases with valid data for all variables in the procedure.
Syntax		RELIABILITY /VARIABLES=Concern1 Concern2 Concern3_R Concern4 Concern5 Concern6 /SCALE('ALL VARIABLES') ALL /MODEL=ALPHA /SUMMARY=TOTAL.
Resources	Processor Time	00:00:00.00
	Elapsed Time	00:00:00.00

Scale: ALL VARIABLES

Case Processing Summary

		N	%
Cases	Valid	241	100.0
	Excluded[a]	0	.0
	Total	241	100.0

a. Listwise deletion based on all variables in the procedure.

Reliability Statistics

Cronbach's Alpha	N of Items
.873	6

Item-Total Statistics

	Scale Mean if Item Deleted	Scale Variance if Item Deleted	Corrected Item-Total Correlation	Cronbach's Alpha if Item Deleted
Please indicate your agreement with each statement using the scale provided: - Thinking about the coronavirus (COVID-19) makes me feel threatened.	22.3900	49.331	.704	.847
Please indicate your agreement with each statement using the scale provided: - I am afraid of the coronavirus (COVID-19).	22.0124	45.404	.790	.830
Concern3_R	21.2863	51.389	.531	.878

Item-Total Statistics

	Scale Mean if Item Deleted	Scale Variance if Item Deleted	Corrected Item-Total Correlation	Cronbach's Alpha if Item Deleted
Please indicate your agreement with each statement using the scale provided: - I am worried that I or people I love will get sick from the coronavirus (COVID-19).	20.4979	53.268	.621	.861
Please indicate your agreement with each statement using the scale provided: - I am stressed around other people because I worry I'll catch the coronavirus (COVID-19).	22.0747	47.544	.753	.838
Please indicate your agreement with each statement using the scale provided: - I have tried hard to avoid other people because I don't want to get sick.	21.7178	50.612	.672	.852

```
 COMPUTE Concern_Tot=mean(concern1, Concern2, Concern3_R, Concern4, Concern5, Concern6).

**Financial

RECODE Finance3 (1=7) (2=6) (3=5) (4=4) (5=3) (6=2) (7=1) INTO Finance3_R.
EXECUTE.

RELIABILITY
  /VARIABLES=Finance1 Finance2 Finance3_R
  /SCALE('ALL VARIABLES') ALL
  /MODEL=ALPHA
  /SUMMARY=TOTAL.
```

Reliability

Notes

Output Created		15-DEC-2021 13:07:56
Comments		
Input	Data	C:\Users\njs5478\Dropbox\HM and COVID\0. Revise and Resubmit\2. R and R Data\Study 1b\Study1b_Data.sav
	Active Dataset	DataSet1
	Filter	<none>
	Weight	<none>
	Split File	<none>
	N of Rows in Working Data File	241
	Matrix Input	
Missing Value Handling	Definition of Missing	User-defined missing values are treated as missing.
	Cases Used	Statistics are based on all cases with valid data for all variables in the procedure.
Syntax		RELIABILITY /VARIABLES=Finance1 Finance2 Finance3_R /SCALE('ALL VARIABLES') ALL /MODEL=ALPHA /SUMMARY=TOTAL.
Resources	Processor Time	00:00:00.02
	Elapsed Time	00:00:00.01

Scale: ALL VARIABLES

Case Processing Summary

		N	%
Cases	Valid	241	100.0
	Excluded[a]	0	.0
	Total	241	100.0

a. Listwise deletion based on all variables in the procedure.

Reliability Statistics

Cronbach's Alpha	N of Items
.749	3

Item-Total Statistics

	Scale Mean if Item Deleted	Scale Variance if Item Deleted	Corrected Item-Total Correlation	Cronbach's Alpha if Item Deleted
Please indicate your agreement with each statement using the scale provided: - The Coronavirus (COVID-19) has impacted me negatively from a financial point of view.	7.7967	10.329	.656	.574
Please indicate your agreement with each statement using the scale provided: - I have lost job-related income due to the Coronavirus (COVID-19).	8.4232	10.837	.517	.734
Finance3_R	7.1909	10.780	.561	.682

```
 COMPUTE Finance_Tot=mean(Finance1, Finance2, Finance3_R).

*Resources

RECODE Resource3 (1=7) (2=6) (3=5) (4=4) (5=3) (6=2) (7=1) INTO Resource3_R.
EXECUTE.

RELIABILITY
  /VARIABLES=Resource1 Resource2 Resource3_R
  /SCALE('ALL VARIABLES') ALL
  /MODEL=ALPHA
  /SUMMARY=TOTAL.
```

Reliability

Notes

Output Created		15-DEC-2021 13:07:56
Comments		
Input	Data	C:\Users\njs5478\Dropbox\HM and COVID\0. Revise and Resubmit\2. R and R Data\Study 1b\Study1b_Data.sav
	Active Dataset	DataSet1
	Filter	<none>
	Weight	<none>
	Split File	<none>
	N of Rows in Working Data File	241
	Matrix Input	
Missing Value Handling	Definition of Missing	User-defined missing values are treated as missing.
	Cases Used	Statistics are based on all cases with valid data for all variables in the procedure.
Syntax		RELIABILITY<br>/VARIABLES=Resource1 Resource2 Resource3_R<br>/SCALE('ALL VARIABLES') ALL<br>/MODEL=ALPHA<br>/SUMMARY=TOTAL.
Resources	Processor Time	00:00:00.02
	Elapsed Time	00:00:00.02

Scale: ALL VARIABLES

Case Processing Summary

		N	%
Cases	Valid	241	100.0
	Excluded[a]	0	.0
	Total	241	100.0

a. Listwise deletion based on all variables in the procedure.

Reliability Statistics

Cronbach's Alpha	N of Items
.669	3

Item-Total Statistics

	Scale Mean if Item Deleted	Scale Variance if Item Deleted	Corrected Item-Total Correlation	Cronbach's Alpha if Item Deleted
Please indicate your agreement with each statement using the scale provided: - I have had a hard time getting needed resources (food, toilet paper) due to the Coronavirus (COVID-19).	7.7718	9.385	.502	.550
Please indicate your agreement with each statement using the scale provided: - It has been difficult for me to get the things I need due to the Coronavirus (COVID-19).	7.0788	8.373	.523	.515
Resource3_R	6.8174	9.100	.423	.651

```
 COMPUTE Resource_Tot=mean(Resource1, Resource2, Resource3_R).

*Psychology

RECODE Psychology3 (1=7) (2=6) (3=5) (4=4) (5=3) (6=2) (7=1) INTO Psychology3_R.
EXECUTE.

RELIABILITY
  /VARIABLES=Psychology1 Psychology2 Psychology3_R
  /SCALE('ALL VARIABLES') ALL
  /MODEL=ALPHA
  /SUMMARY=TOTAL.
```

Reliability

Notes

Output Created		15-DEC-2021 13:07:56
Comments		
Input	Data	C:\Users\njs5478\Dropbox\HM and COVID\0. Revise and Resubmit\2. R and R Data\Study 1b\Study1b_Data.sav
	Active Dataset	DataSet1
	Filter	<none>
	Weight	<none>
	Split File	<none>
	N of Rows in Working Data File	241
	Matrix Input	
Missing Value Handling	Definition of Missing	User-defined missing values are treated as missing.
	Cases Used	Statistics are based on all cases with valid data for all variables in the procedure.
Syntax		RELIABILITY /VARIABLES=Psychology1 Psychology2 Psychology3_R /SCALE('ALL VARIABLES') ALL /MODEL=ALPHA /SUMMARY=TOTAL.
Resources	Processor Time	00:00:00.00
	Elapsed Time	00:00:00.00

Scale: ALL VARIABLES

Case Processing Summary

		N	%
Cases	Valid	241	100.0
	Excluded[a]	0	.0
	Total	241	100.0

a. Listwise deletion based on all variables in the procedure.

Reliability Statistics

Cronbach's Alpha	N of Items
.815	3

Item-Total Statistics

	Scale Mean if Item Deleted	Scale Variance if Item Deleted	Corrected Item-Total Correlation	Cronbach's Alpha if Item Deleted
Please indicate your agreement with each statement using the scale provided: - I have become depressed because of the Coronavirus (COVID-19).	10.0000	8.800	.695	.719
Please indicate your agreement with each statement using the scale provided: - The Coronavirus (COVID-19) outbreak has impacted my psychological health negatively.	9.1618	8.753	.770	.635
Psychology3_R	8.8050	11.466	.554	.852

```
 COMPUTE Psychology_Tot=mean(Psychology1, Psychology2, Psychology3_R).

*Personal Covid*

RELIABILITY
  /VARIABLES=Covid1 Covid2 Covid3 Covid4 Covid5 Covid6 Covid7
  /SCALE('ALL VARIABLES') ALL
  /MODEL=ALPHA
  /SUMMARY=TOTAL.
```

Reliability

Notes

Output Created		15-DEC-2021 13:07:56
Comments		
Input	Data	C:\Users\njs5478\Dropbox\HM and COVID\0. Revise and Resubmit\2. R and R Data\Study 1b\Study1b_Data.sav
	Active Dataset	DataSet1
	Filter	<none>
	Weight	<none>
	Split File	<none>
	N of Rows in Working Data File	241
	Matrix Input	
Missing Value Handling	Definition of Missing	User-defined missing values are treated as missing.
	Cases Used	Statistics are based on all cases with valid data for all variables in the procedure.
Syntax		RELIABILITY /VARIABLES=Covid1 Covid2 Covid3 Covid4 Covid5 Covid6 Covid7 /SCALE('ALL VARIABLES') ALL /MODEL=ALPHA /SUMMARY=TOTAL.
Resources	Processor Time	00:00:00.00
	Elapsed Time	00:00:00.00

Scale: ALL VARIABLES

Case Processing Summary

		N	%
Cases	Valid	241	100.0
	Excluded[a]	0	.0
	Total	241	100.0

a. Listwise deletion based on all variables in the procedure.

Reliability Statistics

Cronbach's Alpha	N of Items
.559	7

Item-Total Statistics

	Scale Mean if Item Deleted	Scale Variance if Item Deleted	Corrected Item-Total Correlation	Cronbach's Alpha if Item Deleted
I have been diagnosed with coronavirus (COVID-19).	9.56	2.340	.335	.511
I have had coronavirus-like symptoms at some point in the last two months.	9.71	1.989	.474	.444
I have been sick with something other than the coronavirus in the last two months.	9.76	2.156	.302	.513
I have been in close proximity with someone who has been diagnosed with coronavirus (COVID-19).	9.93	1.941	.438	.453
I have been in close proximity with someone who has had coronavirus-like symptoms in the last two months.	9.93	2.019	.374	.482
I watch a lot of news about the Coronavirus (COVID-19).	10.02	2.566	-.003	.631
I spent a huge percentage of my time trying to find updates online or on TV about Coronavirus (COVID-19).	9.63	2.466	.132	.571

```
FREQUENCIES VARIABLES=Covid1 Covid2 Covid3 Covid4 Covid5 Covid6 Covid7
  /ORDER=ANALYSIS.
```

Frequencies

Notes

Output Created		15-DEC-2021 13:07:56
Comments		
Input	Data	C:\Users\njs5478\Dropbox\HM and COVID\0. Revise and Resubmit\2. R and R Data\Study 1b\Study1b_Data.sav
	Active Dataset	DataSet1
	Filter	<none>
	Weight	<none>
	Split File	<none>
	N of Rows in Working Data File	241
Missing Value Handling	Definition of Missing	User-defined missing values are treated as missing.
	Cases Used	Statistics are based on all cases with valid data.
Syntax		FREQUENCIES VARIABLES=Covid1 Covid2 Covid3 Covid4 Covid5 Covid6 Covid7 /ORDER=ANALYSIS.
Resources	Processor Time	00:00:00.00
	Elapsed Time	00:00:00.00

Statistics

		I have been diagnosed with coronavirus (COVID-19).	I have had coronavirus-like symptoms at some point in the last two months.	I have been sick with something other than the coronavirus in the last two months.	I have been in close proximity with someone who has been diagnosed with coronavirus (COVID-19).	I have been in close proximity with someone who has had coronavirus-like symptoms in the last two months.
N	Valid	241	241	241	241	241
	Missing	0	0	0	0	0

Statistics

		I watch a lot of news about the Coronavirus (COVID-19).	I spent a huge percentage of my time trying to find updates online or on TV about Coronavirus (COVID-19).
N	Valid	241	241
	Missing	0	0

Frequency Table

I have been diagnosed with coronavirus (COVID-19).

		Frequency	Percent	Valid Percent	Cumulative Percent
Valid	Yes	32	13.3	13.3	13.3
	No	209	86.7	86.7	100.0
	Total	241	100.0	100.0	

I have had coronavirus-like symptoms at some point in the last two months.

		Frequency	Percent	Valid Percent	Cumulative Percent
Valid	Yes	70	29.0	29.0	29.0
	No	171	71.0	71.0	100.0
	Total	241	100.0	100.0	

I have been sick with something other than the coronavirus in the last two months.

		Frequency	Percent	Valid Percent	Cumulative Percent
Valid	Yes	82	34.0	34.0	34.0
	No	159	66.0	66.0	100.0
	Total	241	100.0	100.0	

I have been in close proximity with someone who has been diagnosed with coronavirus (COVID-19).

		Frequency	Percent	Valid Percent	Cumulative Percent
Valid	Yes	122	50.6	50.6	50.6
	No	119	49.4	49.4	100.0
	Total	241	100.0	100.0	

I have been in close proximity with someone who has had coronavirus-like symptoms in the last two months.

		Frequency	Percent	Valid Percent	Cumulative Percent
Valid	Yes	121	50.2	50.2	50.2
	No	120	49.8	49.8	100.0
	Total	241	100.0	100.0	

I watch a lot of news about the Coronavirus (COVID-19).

		Frequency	Percent	Valid Percent	Cumulative Percent
Valid	Yes	143	59.3	59.3	59.3
	No	98	40.7	40.7	100.0
	Total	241	100.0	100.0	

I spent a huge percentage of my time trying to find updates online or on TV about Coronavirus (COVID-19).

		Frequency	Percent	Valid Percent	Cumulative Percent
Valid	Yes	51	21.2	21.2	21.2
	No	190	78.8	78.8	100.0
	Total	241	100.0	100.0	

```
RELIABILITY
 /VARIABLES=Covid6 Covid7
 /SCALE('ALL VARIABLES') ALL
 /MODEL=ALPHA
 /SUMMARY=TOTAL.
```

Reliability

Notes

Output Created		15-DEC-2021 13:07:56
Comments		
Input	Data	C: \Users\njs5478\Dropbox\HM and COVID\0. Revise and Resubmit\2. R and R Data\Study 1b\Study1b_Data.sav
	Active Dataset	DataSet1
	Filter	<none>
	Weight	<none>
	Split File	<none>
	N of Rows in Working Data File	241
	Matrix Input	
Missing Value Handling	Definition of Missing	User-defined missing values are treated as missing.
	Cases Used	Statistics are based on all cases with valid data for all variables in the procedure.
Syntax		RELIABILITY /VARIABLES=Covid6 Covid7 /SCALE('ALL VARIABLES') ALL /MODEL=ALPHA /SUMMARY=TOTAL.
Resources	Processor Time	00:00:00.00
	Elapsed Time	00:00:00.00

Scale: ALL VARIABLES

Case Processing Summary

		N	%
Cases	Valid	241	100.0
	Excluded[a]	0	.0
	Total	241	100.0

a. Listwise deletion based on all variables in the procedure.

Reliability Statistics

Cronbach's Alpha	N of Items
.385	2

Item-Total Statistics

	Scale Mean if Item Deleted	Scale Variance if Item Deleted	Corrected Item-Total Correlation	Cronbach's Alpha if Item Deleted
I watch a lot of news about the Coronavirus (COVID-19).	1.79	.168	.243	.
I spent a huge percentage of my time trying to find updates online or on TV about Coronavirus (COVID-19).	1.41	.242	.243	.

```
**Political Identity

CORRELATIONS
  /VARIABLES=PParty PIdeology
  /PRINT=TWOTAIL NOSIG
  /MISSING=PAIRWISE.
```

Correlations

Notes

Output Created		15-DEC-2021 13:07:56
Comments		
Input	Data	C:\Users\njs5478\Dropbox\HM and COVID\0. Revise and Resubmit\2. R and R Data\Study 1b\Study1b_Data.sav
	Active Dataset	DataSet1
	Filter	<none>
	Weight	<none>
	Split File	<none>
	N of Rows in Working Data File	241
Missing Value Handling	Definition of Missing	User-defined missing values are treated as missing.
	Cases Used	Statistics for each pair of variables are based on all the cases with valid data for that pair.
Syntax		CORRELATIONS /VARIABLES=PParty PIdeology /PRINT=TWOTAIL NOSIG /MISSING=PAIRWISE.
Resources	Processor Time	00:00:00.02
	Elapsed Time	00:00:00.02

Correlations

		Which of the following best describes your political party affiliation?	Which of the following best describes your political ideology?
Which of the following best describes your political party affiliation?	Pearson Correlation	1	.840**
	Sig. (2-tailed)		.000
	N	241	241
Which of the following best describes your political ideology?	Pearson Correlation	.840**	1
	Sig. (2-tailed)	.000	
	N	241	241

**. Correlation is significant at the 0.01 level (2-tailed).

```
 **Descriptives and Frequencies**

FREQUENCIES VARIABLES=PParty PIdeology SES Education Gender Race Age MRN Finance_Tot Reso
urce_Tot Psychology_Tot
  /STATISTICS=STDDEV MINIMUM MAXIMUM MEAN
  /ORDER=ANALYSIS.
```

Frequencies

Notes

Output Created		15-DEC-2021 13:07:56
Comments		
Input	Data	C:<br>\Users\njs5478\Dropbox\HM and COVID\0. Revise and Resubmit\2. R and R Data\Study 1b\Study1b_Data.sav
	Active Dataset	DataSet1
	Filter	<none>
	Weight	<none>
	Split File	<none>
	N of Rows in Working Data File	241
Missing Value Handling	Definition of Missing	User-defined missing values are treated as missing.
	Cases Used	Statistics are based on all cases with valid data.
Syntax		FREQUENCIES VARIABLES=PParty PIdeology SES Education Gender Race Age MRN Finance_Tot Resource_Tot Psychology_Tot<br>/STATISTICS=STDDEV MINIMUM MAXIMUM MEAN...
Resources	Processor Time	00:00:00.02
	Elapsed Time	00:00:00.02

Statistics

		Which of the following best describes your political party affiliation?	Which of the following best describes your political ideology?	Self Reported Socioeconomic Status	Please indicate the highest level of education that you have received:	Gender - Selected Choice
N	Valid	241	241	241	241	241
	Missing	0	0	0	0	0
Mean		2.62	3.58	3.41	2.74	2.04
Std. Deviation		1.385	1.585	.807	.585	1.062
Minimum		1	1	1	1	1
Maximum		5	7	5	5	6

Statistics

		Racial Identity - Selected Choice	Age	MRN	Finance_Tot	Resource_Tot	Psychology_Tot
N	Valid	241	241	240	241	241	241
	Missing	0	0	1	0	0	0
Mean		1.80	19.05	3.2638	3.9018	3.6113	4.6611
Std. Deviation		1.760	1.949	.86575	1.53781	1.38567	1.48790
Minimum		1	18	1.08	1.00	1.00	1.00
Maximum		9	41	5.35	7.00	7.00	7.00

Frequency Table

Which of the following best describes your political party affiliation?

		Frequency	Percent	Valid Percent	Cumulative Percent
Valid	Democrat	73	30.3	30.3	30.3
	Democrat Leaning	43	17.8	17.8	48.1
	Independent	58	24.1	24.1	72.2
	Republican Leaning	36	14.9	14.9	87.1
	Republican	31	12.9	12.9	100.0
	Total	241	100.0	100.0	

Which of the following best describes your political ideology?

		Frequency	Percent	Valid Percent	Cumulative Percent
Valid	Very Liberal	23	9.5	9.5	9.5
	Liberal	48	19.9	19.9	29.5
	Somewhat Liberal	47	19.5	19.5	49.0
	Neither Liberal Nor Conservative	52	21.6	21.6	70.5
	Somewhat Conservative	38	15.8	15.8	86.3
	Conservative	27	11.2	11.2	97.5
	Very Conservative	6	2.5	2.5	100.0
	Total	241	100.0	100.0	

Self Reported Socioeconomic Status

		Frequency	Percent	Valid Percent	Cumulative Percent
Valid	Poor	4	1.7	1.7	1.7
	Working Class	17	7.1	7.1	8.7
	Middle Class	117	48.5	48.5	57.3
	Upper Middle Class	83	34.4	34.4	91.7
	Upper Class	20	8.3	8.3	100.0
	Total	241	100.0	100.0	

Please indicate the highest level of education that you have received:

		Frequency	Percent	Valid Percent	Cumulative Percent
Valid	Some high school	6	2.5	2.5	2.5
	Completed high school	59	24.5	24.5	27.0
	Some college	170	70.5	70.5	97.5
	Associate's Degree	3	1.2	1.2	98.8
	Bachelor's Degree	3	1.2	1.2	100.0
	Total	241	100.0	100.0	

Gender - Selected Choice

		Frequency	Percent	Valid Percent	Cumulative Percent
Valid	Biologically Born Male	119	49.4	49.4	49.4
	Biologically Born Female	120	49.8	49.8	99.2
	Gender Identity Not Listed	2	.8	.8	100.0
	Total	241	100.0	100.0	

Racial Identity - Selected Choice

		Frequency	Percent	Valid Percent	Cumulative Percent
Valid	White/Caucasian	181	75.1	75.1	75.1
	Black/African American	14	5.8	5.8	80.9
	Asian	24	10.0	10.0	90.9
	Hispanic/Latino(a)	9	3.7	3.7	94.6
	Biracial	7	2.9	2.9	97.5
	Multiracial	5	2.1	2.1	99.6
	Racial Identity Not Listed	1	.4	.4	100.0
	Total	241	100.0	100.0	

Age

		Frequency	Percent	Valid Percent	Cumulative Percent
Valid	18	105	43.6	43.6	43.6
	19	80	33.2	33.2	76.8
	20	36	14.9	14.9	91.7
	21	12	5.0	5.0	96.7
	22	3	1.2	1.2	97.9
	23	2	.8	.8	98.8
	25	1	.4	.4	99.2
	31	1	.4	.4	99.6
	41	1	.4	.4	100.0
	Total	241	100.0	100.0	

MRN

		Frequency	Percent	Valid Percent	Cumulative Percent
Valid	1.08	1	.4	.4	.4
	1.31	1	.4	.4	.8
	1.46	1	.4	.4	1.3
	1.50	2	.8	.8	2.1
	1.54	1	.4	.4	2.5
	1.58	1	.4	.4	2.9
	1.62	1	.4	.4	3.3
	1.65	1	.4	.4	3.8
	1.69	1	.4	.4	4.2
	1.77	1	.4	.4	4.6
	1.81	2	.8	.8	5.4
	1.85	4	1.7	1.7	7.1
	1.88	1	.4	.4	7.5
	1.92	2	.8	.8	8.3
	1.96	2	.8	.8	9.2
	2.04	1	.4	.4	9.6
	2.08	1	.4	.4	10.0
	2.12	6	2.5	2.5	12.5
	2.15	3	1.2	1.3	13.8
	2.23	3	1.2	1.3	15.0
	2.27	2	.8	.8	15.8
	2.31	3	1.2	1.3	17.1
	2.35	4	1.7	1.7	18.8
	2.38	1	.4	.4	19.2
	2.46	1	.4	.4	19.6
	2.50	2	.8	.8	20.4
	2.54	2	.8	.8	21.3
	2.58	1	.4	.4	21.7
	2.62	5	2.1	2.1	23.8
	2.65	3	1.2	1.3	25.0
	2.69	1	.4	.4	25.4
	2.73	2	.8	.8	26.3
	2.77	4	1.7	1.7	27.9
	2.81	3	1.2	1.3	29.2
	2.85	2	.8	.8	30.0

MRN

	Frequency	Percent	Valid Percent	Cumulative Percent
2.88	2	.8	.8	30.8
2.92	7	2.9	2.9	33.8
2.96	3	1.2	1.3	35.0
3.00	2	.8	.8	35.8
3.04	6	2.5	2.5	38.3
3.08	9	3.7	3.8	42.1
3.12	4	1.7	1.7	43.8
3.15	4	1.7	1.7	45.4
3.19	1	.4	.4	45.8
3.23	4	1.7	1.7	47.5
3.27	4	1.7	1.7	49.2
3.31	4	1.7	1.7	50.8
3.35	5	2.1	2.1	52.9
3.38	3	1.2	1.3	54.2
3.42	3	1.2	1.3	55.4
3.46	4	1.7	1.7	57.1
3.50	7	2.9	2.9	60.0
3.54	3	1.2	1.3	61.3
3.58	6	2.5	2.5	63.7
3.62	6	2.5	2.5	66.3
3.65	2	.8	.8	67.1
3.69	3	1.2	1.3	68.3
3.73	6	2.5	2.5	70.8
3.77	4	1.7	1.7	72.5
3.81	2	.8	.8	73.3
3.85	6	2.5	2.5	75.8
3.88	1	.4	.4	76.3
3.92	1	.4	.4	76.7
3.96	1	.4	.4	77.1
4.00	7	2.9	2.9	80.0
4.04	5	2.1	2.1	82.1
4.08	3	1.2	1.3	83.3
4.12	3	1.2	1.3	84.6
4.15	5	2.1	2.1	86.7
4.19	2	.8	.8	87.5

MRN

		Frequency	Percent	Valid Percent	Cumulative Percent
	4.23	5	2.1	2.1	89.6
	4.31	1	.4	.4	90.0
	4.35	1	.4	.4	90.4
	4.38	1	.4	.4	90.8
	4.42	1	.4	.4	91.3
	4.46	3	1.2	1.3	92.5
	4.54	2	.8	.8	93.3
	4.65	2	.8	.8	94.2
	4.69	2	.8	.8	95.0
	4.77	1	.4	.4	95.4
	4.81	1	.4	.4	95.8
	4.85	2	.8	.8	96.7
	4.88	5	2.1	2.1	98.8
	5.12	1	.4	.4	99.2
	5.31	1	.4	.4	99.6
	5.35	1	.4	.4	100.0
	Total	240	99.6	100.0	
Missing	System	1	.4		
Total		241	100.0		

Finance_Tot

		Frequency	Percent	Valid Percent	Cumulative Percent
Valid	1.00	10	4.1	4.1	4.1
	1.33	9	3.7	3.7	7.9
	1.67	7	2.9	2.9	10.8
	2.00	14	5.8	5.8	16.6
	2.33	11	4.6	4.6	21.2
	2.67	6	2.5	2.5	23.7
	3.00	22	9.1	9.1	32.8
	3.33	16	6.6	6.6	39.4
	3.67	22	9.1	9.1	48.5
	4.00	20	8.3	8.3	56.8
	4.33	18	7.5	7.5	64.3
	4.67	9	3.7	3.7	68.0
	5.00	18	7.5	7.5	75.5
	5.33	19	7.9	7.9	83.4
	5.67	14	5.8	5.8	89.2
	6.00	7	2.9	2.9	92.1
	6.33	11	4.6	4.6	96.7
	6.67	4	1.7	1.7	98.3
	7.00	4	1.7	1.7	100.0
	Total	241	100.0	100.0	

Resource_Tot

		Frequency	Percent	Valid Percent	Cumulative Percent
Valid	1.00	12	5.0	5.0	5.0
	1.33	7	2.9	2.9	7.9
	1.67	8	3.3	3.3	11.2
	2.00	18	7.5	7.5	18.7
	2.33	8	3.3	3.3	22.0
	2.67	16	6.6	6.6	28.6
	3.00	16	6.6	6.6	35.3
	3.33	26	10.8	10.8	46.1
	3.67	25	10.4	10.4	56.4
	4.00	22	9.1	9.1	65.6
	4.33	25	10.4	10.4	75.9
	4.67	9	3.7	3.7	79.7
	5.00	12	5.0	5.0	84.6
	5.33	14	5.8	5.8	90.5
	5.67	8	3.3	3.3	93.8
	6.00	8	3.3	3.3	97.1
	6.33	4	1.7	1.7	98.8
	6.67	2	.8	.8	99.6
	7.00	1	.4	.4	100.0
	Total	241	100.0	100.0	

Psychology_Tot

		Frequency	Percent	Valid Percent	Cumulative Percent
Valid	1.00	3	1.2	1.2	1.2
	1.33	6	2.5	2.5	3.7
	1.67	5	2.1	2.1	5.8
	2.00	3	1.2	1.2	7.1
	2.33	5	2.1	2.1	9.1
	2.67	9	3.7	3.7	12.9
	3.00	11	4.6	4.6	17.4
	3.33	13	5.4	5.4	22.8
	3.67	10	4.1	4.1	27.0
	4.00	17	7.1	7.1	34.0
	4.33	21	8.7	8.7	42.7
	4.67	18	7.5	7.5	50.2
	5.00	14	5.8	5.8	56.0
	5.33	23	9.5	9.5	65.6
	5.67	26	10.8	10.8	76.3
	6.00	18	7.5	7.5	83.8
	6.33	12	5.0	5.0	88.8
	6.67	20	8.3	8.3	97.1
	7.00	7	2.9	2.9	100.0
	Total	241	100.0	100.0	

```
**Frequencies and Correlations for Personal Experiences with COVID-19

FREQUENCIES VARIABLES=Covid1 Covid2 Covid3 Covid4 Covid5 Covid6 Covid7
  /ORDER=ANALYSIS.
```

Frequencies

Notes

Output Created		15-DEC-2021 13:07:56
Comments		
Input	Data	C: \Users\njs5478\Dropbox\HM and COVID\0. Revise and Resubmit\2. R and R Data\Study 1b\Study1b_Data.sav
	Active Dataset	DataSet1
	Filter	<none>
	Weight	<none>
	Split File	<none>
	N of Rows in Working Data File	241
Missing Value Handling	Definition of Missing	User-defined missing values are treated as missing.
	Cases Used	Statistics are based on all cases with valid data.
Syntax		FREQUENCIES VARIABLES=Covid1 Covid2 Covid3 Covid4 Covid5 Covid6 Covid7 /ORDER=ANALYSIS.
Resources	Processor Time	00:00:00.00
	Elapsed Time	00:00:00.00

Statistics

		I have been diagnosed with coronavirus (COVID-19).	I have had coronavirus-like symptoms at some point in the last two months.	I have been sick with something other than the coronavirus in the last two months.	I have been in close proximity with someone who has been diagnosed with coronavirus (COVID-19).	I have been in close proximity with someone who has had coronavirus-like symptoms in the last two months.
N	Valid	241	241	241	241	241
	Missing	0	0	0	0	0

Statistics

		I watch a lot of news about the Coronavirus (COVID-19).	I spent a huge percentage of my time trying to find updates online or on TV about Coronavirus (COVID-19).
N	Valid	241	241
	Missing	0	0

Frequency Table

I have been diagnosed with coronavirus (COVID-19).

		Frequency	Percent	Valid Percent	Cumulative Percent
Valid	Yes	32	13.3	13.3	13.3
	No	209	86.7	86.7	100.0
	Total	241	100.0	100.0	

I have had coronavirus-like symptoms at some point in the last two months.

		Frequency	Percent	Valid Percent	Cumulative Percent
Valid	Yes	70	29.0	29.0	29.0
	No	171	71.0	71.0	100.0
	Total	241	100.0	100.0	

I have been sick with something other than the coronavirus in the last two months.

		Frequency	Percent	Valid Percent	Cumulative Percent
Valid	Yes	82	34.0	34.0	34.0
	No	159	66.0	66.0	100.0
	Total	241	100.0	100.0	

I have been in close proximity with someone who has been diagnosed with coronavirus (COVID-19).

		Frequency	Percent	Valid Percent	Cumulative Percent
Valid	Yes	122	50.6	50.6	50.6
	No	119	49.4	49.4	100.0
	Total	241	100.0	100.0	

I have been in close proximity with someone who has had coronavirus-like symptoms in the last two months.

		Frequency	Percent	Valid Percent	Cumulative Percent
Valid	Yes	121	50.2	50.2	50.2
	No	120	49.8	49.8	100.0
	Total	241	100.0	100.0	

I watch a lot of news about the Coronavirus (COVID-19).

		Frequency	Percent	Valid Percent	Cumulative Percent
Valid	Yes	143	59.3	59.3	59.3
	No	98	40.7	40.7	100.0
	Total	241	100.0	100.0	

I spent a huge percentage of my time trying to find updates online or on TV about Coronavirus (COVID-19).

		Frequency	Percent	Valid Percent	Cumulative Percent
Valid	Yes	51	21.2	21.2	21.2
	No	190	78.8	78.8	100.0
	Total	241	100.0	100.0	

```
CORRELATIONS
  /VARIABLES=Covid1 Covid2 Covid3 Covid4 Covid5 Covid6 Covid7 PParty TrumpApproval Trump
Biden
    Pelosi McConnell Fauci RepCongress DemCongress State MRN MGRS Concern_Tot Finance_Tot
    Psychology_Tot Risk_Rules Risk_Help
  /PRINT=TWOTAIL NOSIG
```

```
/MISSING=PAIRWISE.
```

Correlations

Notes

Output Created		15-DEC-2021 13:07:56
Comments		
Input	Data	C: \Users\njs5478\Dropbox\HM and COVID\0. Revise and Resubmit\2. R and R Data\Study 1b\Study1b_Data.sav
	Active Dataset	DataSet1
	Filter	<none>
	Weight	<none>
	Split File	<none>
	N of Rows in Working Data File	241
Missing Value Handling	Definition of Missing	User-defined missing values are treated as missing.
	Cases Used	Statistics for each pair of variables are based on all the cases with valid data for that pair.
Syntax		CORRELATIONS /VARIABLES=Covid1 Covid2 Covid3 Covid4 Covid5 Covid6 Covid7 PParty TrumpApproval Trump Biden Pelosi McConnell Fauci RepCongress DemCongress State MRN MGRS Concern_Tot Finance_Tot Psychology_Tot Risk_Rules Risk_Help /PRINT=TWOTAIL NOSIG /MISSING=PAIRWISE.
Resources	Processor Time	00:00:00.08
	Elapsed Time	00:00:00.04

Correlations

		I have been diagnosed with coronavirus (COVID-19).	I have had coronavirus-like symptoms at some point in the last two months.	I have been sick with something other than the coronavirus in the last two months.
I have been diagnosed with coronavirus (COVID-19).	Pearson Correlation	1	.396**	.080
	Sig. (2-tailed)		.000	.214
	N	241	241	241
I have had coronavirus-like symptoms at some point in the last two months.	Pearson Correlation	.396**	1	.428**
	Sig. (2-tailed)	.000		.000
	N	241	241	241
I have been sick with something other than the coronavirus in the last two months.	Pearson Correlation	.080	.428**	1
	Sig. (2-tailed)	.214	.000	
	N	241	241	241
I have been in close proximity with someone who has been diagnosed with coronavirus (COVID-19).	Pearson Correlation	.338**	.321**	.184**
	Sig. (2-tailed)	.000	.000	.004
	N	241	241	241
I have been in close proximity with someone who has had coronavirus-like symptoms in the last two months.	Pearson Correlation	.170**	.400**	.207**
	Sig. (2-tailed)	.008	.000	.001
	N	241	241	241
I watch a lot of news about the Coronavirus (COVID-19).	Pearson Correlation	.000	-.066	.042
	Sig. (2-tailed)	.996	.309	.518
	N	241	241	241

Correlations

		I have been in close proximity with someone who has been diagnosed with coronavirus (COVID-19).	I have been in close proximity with someone who has had coronavirus-like symptoms in the last two months.	I watch a lot of news about the Coronavirus (COVID-19).
I have been diagnosed with coronavirus (COVID-19).	Pearson Correlation	.338**	.170**	.000
	Sig. (2-tailed)	.000	.008	.996
	N	241	241	241
I have had coronavirus-like symptoms at some point in the last two months.	Pearson Correlation	.321**	.400**	-.066
	Sig. (2-tailed)	.000	.000	.309
	N	241	241	241
I have been sick with something other than the coronavirus in the last two months.	Pearson Correlation	.184**	.207**	.042
	Sig. (2-tailed)	.004	.001	.518
	N	241	241	241
I have been in close proximity with someone who has been diagnosed with coronavirus (COVID-19).	Pearson Correlation	1	.510**	-.057
	Sig. (2-tailed)		.000	.376
	N	241	241	241
I have been in close proximity with someone who has had coronavirus-like symptoms in the last two months.	Pearson Correlation	.510**	1	-.132*
	Sig. (2-tailed)	.000		.041
	N	241	241	241
I watch a lot of news about the Coronavirus (COVID-19).	Pearson Correlation	-.057	-.132*	1
	Sig. (2-tailed)	.376	.041	
	N	241	241	241

Correlations

		I spent a huge percentage of my time trying to find updates online or on TV about Coronavirus (COVID-19).	Which of the following best describes your political party affiliation?	Do you approve or disapprove of the way Donald Trump is handling his job as President?
I have been diagnosed with coronavirus (COVID-19).	Pearson Correlation	.097	.017	-.013
	Sig. (2-tailed)	.135	.793	.843
	N	241	241	240
I have had coronavirus-like symptoms at some point in the last two months.	Pearson Correlation	.004	.010	-.024
	Sig. (2-tailed)	.949	.873	.712
	N	241	241	240
I have been sick with something other than the coronavirus in the last two months.	Pearson Correlation	.014	-.038	-.074
	Sig. (2-tailed)	.830	.560	.254
	N	241	241	240
I have been in close proximity with someone who has been diagnosed with coronavirus (COVID-19).	Pearson Correlation	.085	.036	-.003
	Sig. (2-tailed)	.189	.582	.966
	N	241	241	240
I have been in close proximity with someone who has had coronavirus-like symptoms in the last two months.	Pearson Correlation	.008	-.088	-.102
	Sig. (2-tailed)	.902	.172	.113
	N	241	241	240
I watch a lot of news about the Coronavirus (COVID-19).	Pearson Correlation	.243**	.183**	.211**
	Sig. (2-tailed)	.000	.004	.001
	N	241	241	240

Correlations

		Using the scale provided, please indicate your personal opinion regarding how each of the following is handling the response to COVID-19 (Coronavirus) in the United States: - Donald Trump	Using the scale provided, please indicate your personal opinion regarding how each of the following is handling the response to COVID-19 (Coronavirus) in the United States: - Joe Biden	Using the scale provided, please indicate your personal opinion regarding how each of the following is handling the response to COVID-19 (Coronavirus) in the United States: - Nancy Pelosi
I have been diagnosed with coronavirus (COVID-19).	Pearson Correlation	-.043	.007	.070
	Sig. (2-tailed)	.508	.914	.283
	N	240	240	240
I have had coronavirus-like symptoms at some point in the last two months.	Pearson Correlation	-.018	.033	.039
	Sig. (2-tailed)	.779	.609	.552
	N	240	240	240
I have been sick with something other than the coronavirus in the last two months.	Pearson Correlation	-.089	.028	.039
	Sig. (2-tailed)	.171	.669	.550
	N	240	240	240
I have been in close proximity with someone who has been diagnosed with coronavirus (COVID-19).	Pearson Correlation	-.051	.012	.022
	Sig. (2-tailed)	.431	.852	.731
	N	240	240	240
I have been in close proximity with someone who has had coronavirus-like symptoms in the last two months.	Pearson Correlation	-.119	.090	.127*
	Sig. (2-tailed)	.066	.163	.049
	N	240	240	240
I watch a lot of news about the Coronavirus (COVID-19).	Pearson Correlation	.193**	-.194**	.000
	Sig. (2-tailed)	.003	.003	1.000
	N	240	240	240

Correlations

		Using the scale provided, please indicate your personal opinion regarding how each of the following is handling the response to COVID-19 (Coronavirus) in the United States: - Mitch McConnell	Using the scale provided, please indicate your personal opinion regarding how each of the following is handling the response to COVID-19 (Coronavirus) in the United States: - Dr. Anthony Fauci	Using the scale provided, please indicate your personal opinion regarding how each of the following is handling the response to COVID-19 (Coronavirus) in the United States: - Republican Congress Members
I have been diagnosed with coronavirus (COVID-19).	Pearson Correlation	-.071	-.005	-.050
	Sig. (2-tailed)	.272	.944	.441
	N	240	240	240
I have had coronavirus-like symptoms at some point in the last two months.	Pearson Correlation	-.026	.001	-.033
	Sig. (2-tailed)	.691	.989	.614
	N	240	240	240
I have been sick with something other than the coronavirus in the last two months.	Pearson Correlation	-.030	.089	-.033
	Sig. (2-tailed)	.643	.168	.607
	N	240	240	240
I have been in close proximity with someone who has been diagnosed with coronavirus (COVID-19).	Pearson Correlation	-.032	.009	-.139*
	Sig. (2-tailed)	.620	.895	.032
	N	240	240	240
I have been in close proximity with someone who has had coronavirus-like symptoms in the last two months.	Pearson Correlation	-.029	.081	-.165*
	Sig. (2-tailed)	.658	.210	.010
	N	240	240	240
I watch a lot of news about the Coronavirus (COVID-19).	Pearson Correlation	.144*	-.023	.269**
	Sig. (2-tailed)	.026	.724	.000
	N	240	240	240

Correlations

		Using the scale provided, please indicate your personal opinion regarding how each of the following is handling the response to COVID-19 (Coronavirus) in the United States: - Democratic Congress Members	Using the scale provided, please indicate your personal opinion regarding how each of the following is handling the response to COVID-19 (Coronavirus) in the United States: - State Leaders/Governors	MRN	MGRS
I have been diagnosed with coronavirus (COVID-19).	Pearson Correlation	.006	-.034	.012	-.074
	Sig. (2-tailed)	.926	.605	.850	.255
	N	240	240	240	240
I have had coronavirus-like symptoms at some point in the last two months.	Pearson Correlation	-.022	.040	.076	-.049
	Sig. (2-tailed)	.733	.539	.241	.450
	N	240	240	240	240
I have been sick with something other than the coronavirus in the last two months.	Pearson Correlation	-.067	.089	.041	-.081
	Sig. (2-tailed)	.300	.170	.531	.212
	N	240	240	240	240
I have been in close proximity with someone who has been diagnosed with coronavirus (COVID-19).	Pearson Correlation	-.074	.067	.023	.029
	Sig. (2-tailed)	.254	.298	.728	.655
	N	240	240	240	240
I have been in close proximity with someone who has had coronavirus-like symptoms in the last two months.	Pearson Correlation	.011	.108	-.022	.064
	Sig. (2-tailed)	.861	.096	.732	.324
	N	240	240	240	240
I watch a lot of news about the Coronavirus (COVID-19).	Pearson Correlation	.009	-.107	.118	-.137*
	Sig. (2-tailed)	.889	.098	.068	.034
	N	240	240	240	240

Correlations

		Concern_Tot	Finance_Tot	Psychology_Tot
I have been diagnosed with coronavirus (COVID-19).	Pearson Correlation	.047	-.036	-.106
	Sig. (2-tailed)	.470	.582	.101
	N	241	241	241
I have had coronavirus-like symptoms at some point in the last two months.	Pearson Correlation	-.019	-.021	-.123
	Sig. (2-tailed)	.769	.745	.056
	N	241	241	241
I have been sick with something other than the coronavirus in the last two months.	Pearson Correlation	.041	.021	-.071
	Sig. (2-tailed)	.530	.750	.269
	N	241	241	241
I have been in close proximity with someone who has been diagnosed with coronavirus (COVID-19).	Pearson Correlation	.074	.011	-.039
	Sig. (2-tailed)	.251	.866	.545
	N	241	241	241
I have been in close proximity with someone who has had coronavirus-like symptoms in the last two months.	Pearson Correlation	.093	.100	-.041
	Sig. (2-tailed)	.149	.122	.526
	N	241	241	241
I watch a lot of news about the Coronavirus (COVID-19).	Pearson Correlation	-.243**	-.075	-.130*
	Sig. (2-tailed)	.000	.243	.044
	N	241	241	241

Correlations

		Risk_Rules	Risk_Help
I have been diagnosed with coronavirus (COVID-19).	Pearson Correlation	.002	.028
	Sig. (2-tailed)	.977	.665
	N	241	241
I have had coronavirus-like symptoms at some point in the last two months.	Pearson Correlation	.015	.038
	Sig. (2-tailed)	.822	.552
	N	241	241
I have been sick with something other than the coronavirus in the last two months.	Pearson Correlation	-.043	-.022
	Sig. (2-tailed)	.509	.734
	N	241	241
I have been in close proximity with someone who has been diagnosed with coronavirus (COVID-19).	Pearson Correlation	-.038	-.029
	Sig. (2-tailed)	.558	.651
	N	241	241
I have been in close proximity with someone who has had coronavirus-like symptoms in the last two months.	Pearson Correlation	-.156*	.080
	Sig. (2-tailed)	.015	.214
	N	241	241
I watch a lot of news about the Coronavirus (COVID-19).	Pearson Correlation	.215**	-.103
	Sig. (2-tailed)	.001	.112
	N	241	241

Correlations

		I have been diagnosed with coronavirus (COVID-19).	I have had coronavirus-like symptoms at some point in the last two months.	I have been sick with something other than the coronavirus in the last two months.
I spent a huge percentage of my time trying to find updates online or on TV about Coronavirus (COVID-19).	Pearson Correlation	.097	.004	.014
	Sig. (2-tailed)	.135	.949	.830
	N	241	241	241
Which of the following best describes your political party affiliation?	Pearson Correlation	.017	.010	-.038
	Sig. (2-tailed)	.793	.873	.560
	N	241	241	241
Do you approve or disapprove of the way Donald Trump is handling his job as President?	Pearson Correlation	-.013	-.024	-.074
	Sig. (2-tailed)	.843	.712	.254
	N	240	240	240
Using the scale provided, please indicate your personal opinion regarding how each of the following is handling the response to COVID-19 (Coronavirus) in the United States: - Donald Trump	Pearson Correlation	-.043	-.018	-.089
	Sig. (2-tailed)	.508	.779	.171
	N	240	240	240

Correlations

		I have been in close proximity with someone who has been diagnosed with coronavirus (COVID-19).	I have been in close proximity with someone who has had coronavirus-like symptoms in the last two months.	I watch a lot of news about the Coronavirus (COVID-19).
I spent a huge percentage of my time trying to find updates online or on TV about Coronavirus (COVID-19).	Pearson Correlation	.085	.008	.243**
	Sig. (2-tailed)	.189	.902	.000
	N	241	241	241
Which of the following best describes your political party affiliation?	Pearson Correlation	.036	-.088	.183**
	Sig. (2-tailed)	.582	.172	.004
	N	241	241	241
Do you approve or disapprove of the way Donald Trump is handling his job as President?	Pearson Correlation	-.003	-.102	.211**
	Sig. (2-tailed)	.966	.113	.001
	N	240	240	240
Using the scale provided, please indicate your personal opinion regarding how each of the following is handling the response to COVID-19 (Coronavirus) in the United States: - Donald Trump	Pearson Correlation	-.051	-.119	.193**
	Sig. (2-tailed)	.431	.066	.003
	N	240	240	240

Correlations

		I spent a huge percentage of my time trying to find updates online or on TV about Coronavirus (COVID-19).	Which of the following best describes your political party affiliation?	Do you approve or disapprove of the way Donald Trump is handling his job as President?
I spent a huge percentage of my time trying to find updates online or on TV about Coronavirus (COVID-19).	Pearson Correlation	1	.138*	.154*
	Sig. (2-tailed)		.033	.017
	N	241	241	240
Which of the following best describes your political party affiliation?	Pearson Correlation	.138*	1	.776**
	Sig. (2-tailed)	.033		.000
	N	241	241	240
Do you approve or disapprove of the way Donald Trump is handling his job as President?	Pearson Correlation	.154*	.776**	1
	Sig. (2-tailed)	.017	.000	
	N	240	240	240
Using the scale provided, please indicate your personal opinion regarding how each of the following is handling the response to COVID-19 (Coronavirus) in the United States: - Donald Trump	Pearson Correlation	.104	.760**	.906**
	Sig. (2-tailed)	.109	.000	.000
	N	240	240	240

Correlations

		Using the scale provided, please indicate your personal opinion regarding how each of the following is handling the response to COVID-19 (Coronavirus) in the United States: - Donald Trump	Using the scale provided, please indicate your personal opinion regarding how each of the following is handling the response to COVID-19 (Coronavirus) in the United States: - Joe Biden	Using the scale provided, please indicate your personal opinion regarding how each of the following is handling the response to COVID-19 (Coronavirus) in the United States: - Nancy Pelosi
I spent a huge percentage of my time trying to find updates online or on TV about Coronavirus (COVID-19).	Pearson Correlation	.104	-.134*	-.013
	Sig. (2-tailed)	.109	.038	.841
	N	240	240	240
Which of the following best describes your political party affiliation?	Pearson Correlation	.760**	-.626**	-.465**
	Sig. (2-tailed)	.000	.000	.000
	N	240	240	240
Do you approve or disapprove of the way Donald Trump is handling his job as President?	Pearson Correlation	.906**	-.637**	-.488**
	Sig. (2-tailed)	.000	.000	.000
	N	240	240	240
Using the scale provided, please indicate your personal opinion regarding how each of the following is handling the response to COVID-19 (Coronavirus) in the United States: - Donald Trump	Pearson Correlation	1	-.603**	-.457**
	Sig. (2-tailed)		.000	.000
	N	240	240	240

Correlations

		Using the scale provided, please indicate your personal opinion regarding how each of the following is handling the response to COVID-19 (Coronavirus) in the United States: - Mitch McConnell	Using the scale provided, please indicate your personal opinion regarding how each of the following is handling the response to COVID-19 (Coronavirus) in the United States: - Dr. Anthony Fauci	Using the scale provided, please indicate your personal opinion regarding how each of the following is handling the response to COVID-19 (Coronavirus) in the United States: - Republican Congress Members
I spent a huge percentage of my time trying to find updates online or on TV about Coronavirus (COVID-19).	Pearson Correlation	.082	-.070	.148*
	Sig. (2-tailed)	.204	.281	.022
	N	240	240	240
Which of the following best describes your political party affiliation?	Pearson Correlation	.313**	-.303**	.508**
	Sig. (2-tailed)	.000	.000	.000
	N	240	240	240
Do you approve or disapprove of the way Donald Trump is handling his job as President?	Pearson Correlation	.391**	-.273**	.597**
	Sig. (2-tailed)	.000	.000	.000
	N	240	240	240
Using the scale provided, please indicate your personal opinion regarding how each of the following is handling the response to COVID-19 (Coronavirus) in the United States: - Donald Trump	Pearson Correlation	.393**	-.302**	.640**
	Sig. (2-tailed)	.000	.000	.000
	N	240	240	240

Correlations

		Using the scale provided, please indicate your personal opinion regarding how each of the following is handling the response to COVID-19 (Coronavirus) in the United States: - Democratic Congress Members	Using the scale provided, please indicate your personal opinion regarding how each of the following is handling the response to COVID-19 (Coronavirus) in the United States: - State Leaders/Governors	MRN	MGRS
I spent a huge percentage of my time trying to find updates online or on TV about Coronavirus (COVID-19).	Pearson Correlation	-.089	-.136*	.077	-.068
	Sig. (2-tailed)	.170	.036	.235	.291
	N	240	240	240	240
Which of the following best describes your political party affiliation?	Pearson Correlation	-.464**	-.323**	.465**	-.070
	Sig. (2-tailed)	.000	.000	.000	.282
	N	240	240	240	240
Do you approve or disapprove of the way Donald Trump is handling his job as President?	Pearson Correlation	-.413**	-.305**	.480**	.008
	Sig. (2-tailed)	.000	.000	.000	.906
	N	240	240	239	239
Using the scale provided, please indicate your personal opinion regarding how each of the following is handling the response to COVID-19 (Coronavirus) in the United States: - Donald Trump	Pearson Correlation	-.354**	-.263**	.474**	.012
	Sig. (2-tailed)	.000	.000	.000	.857
	N	240	240	239	239

Correlations

		Concern_Tot	Finance_Tot	Psychology_Tot
I spent a huge percentage of my time trying to find updates online or on TV about Coronavirus (COVID-19).	Pearson Correlation	-.230**	-.033	-.118
	Sig. (2-tailed)	.000	.609	.067
	N	241	241	241
Which of the following best describes your political party affiliation?	Pearson Correlation	-.429**	-.120	-.256**
	Sig. (2-tailed)	.000	.062	.000
	N	241	241	241
Do you approve or disapprove of the way Donald Trump is handling his job as President?	Pearson Correlation	-.468**	-.178**	-.349**
	Sig. (2-tailed)	.000	.006	.000
	N	240	240	240
Using the scale provided, please indicate your personal opinion regarding how each of the following is handling the response to COVID-19 (Coronavirus) in the United States: - Donald Trump	Pearson Correlation	-.458**	-.147*	-.322**
	Sig. (2-tailed)	.000	.023	.000
	N	240	240	240

Correlations

		Risk_Rules	Risk_Help
I spent a huge percentage of my time trying to find updates online or on TV about Coronavirus (COVID-19).	Pearson Correlation	.149*	.006
	Sig. (2-tailed)	.021	.926
	N	241	241
Which of the following best describes your political party affiliation?	Pearson Correlation	.421**	-.166**
	Sig. (2-tailed)	.000	.010
	N	241	241
Do you approve or disapprove of the way Donald Trump is handling his job as President?	Pearson Correlation	.436**	-.111
	Sig. (2-tailed)	.000	.087
	N	240	240
Using the scale provided, please indicate your personal opinion regarding how each of the following is handling the response to COVID-19 (Coronavirus) in the United States: - Donald Trump	Pearson Correlation	.442**	-.097
	Sig. (2-tailed)	.000	.133
	N	240	240

Correlations

		I have been diagnosed with coronavirus (COVID-19).	I have had coronavirus-like symptoms at some point in the last two months.	I have been sick with something other than the coronavirus in the last two months.
Using the scale provided, please indicate your personal opinion regarding how each of the following is handling the response to COVID-19 (Coronavirus) in the United States: - Joe Biden	Pearson Correlation	.007	.033	.028
	Sig. (2-tailed)	.914	.609	.669
	N	240	240	240
Using the scale provided, please indicate your personal opinion regarding how each of the following is handling the response to COVID-19 (Coronavirus) in the United States: - Nancy Pelosi	Pearson Correlation	.070	.039	.039
	Sig. (2-tailed)	.283	.552	.550
	N	240	240	240
Using the scale provided, please indicate your personal opinion regarding how each of the following is handling the response to COVID-19 (Coronavirus) in the United States: - Mitch McConnell	Pearson Correlation	-.071	-.026	-.030
	Sig. (2-tailed)	.272	.691	.643
	N	240	240	240

Correlations

		I have been in close proximity with someone who has been diagnosed with coronavirus (COVID-19).	I have been in close proximity with someone who has had coronavirus-like symptoms in the last two months.	I watch a lot of news about the Coronavirus (COVID-19).
Using the scale provided, please indicate your personal opinion regarding how each of the following is handling the response to COVID-19 (Coronavirus) in the United States: - Joe Biden	Pearson Correlation	.012	.090	-.194**
	Sig. (2-tailed)	.852	.163	.003
	N	240	240	240
Using the scale provided, please indicate your personal opinion regarding how each of the following is handling the response to COVID-19 (Coronavirus) in the United States: - Nancy Pelosi	Pearson Correlation	.022	.127*	.000
	Sig. (2-tailed)	.731	.049	1.000
	N	240	240	240
Using the scale provided, please indicate your personal opinion regarding how each of the following is handling the response to COVID-19 (Coronavirus) in the United States: - Mitch McConnell	Pearson Correlation	-.032	-.029	.144*
	Sig. (2-tailed)	.620	.658	.026
	N	240	240	240

Correlations

		I spent a huge percentage of my time trying to find updates online or on TV about Coronavirus (COVID-19).	Which of the following best describes your political party affiliation?	Do you approve or disapprove of the way Donald Trump is handling his job as President?
Using the scale provided, please indicate your personal opinion regarding how each of the following is handling the response to COVID-19 (Coronavirus) in the United States: - Joe Biden	Pearson Correlation	-.134*	-.626**	-.637**
	Sig. (2-tailed)	.038	.000	.000
	N	240	240	240
Using the scale provided, please indicate your personal opinion regarding how each of the following is handling the response to COVID-19 (Coronavirus) in the United States: - Nancy Pelosi	Pearson Correlation	-.013	-.465**	-.488**
	Sig. (2-tailed)	.841	.000	.000
	N	240	240	240
Using the scale provided, please indicate your personal opinion regarding how each of the following is handling the response to COVID-19 (Coronavirus) in the United States: - Mitch McConnell	Pearson Correlation	.082	.313**	.391**
	Sig. (2-tailed)	.204	.000	.000
	N	240	240	240

Correlations

		Using the scale provided, please indicate your personal opinion regarding how each of the following is handling the response to COVID-19 (Coronavirus) in the United States: - Donald Trump	Using the scale provided, please indicate your personal opinion regarding how each of the following is handling the response to COVID-19 (Coronavirus) in the United States: - Joe Biden	Using the scale provided, please indicate your personal opinion regarding how each of the following is handling the response to COVID-19 (Coronavirus) in the United States: - Nancy Pelosi
Using the scale provided, please indicate your personal opinion regarding how each of the following is handling the response to COVID-19 (Coronavirus) in the United States: - Joe Biden	Pearson Correlation	-.603**	1	.597**
	Sig. (2-tailed)	.000		.000
	N	240	240	240
Using the scale provided, please indicate your personal opinion regarding how each of the following is handling the response to COVID-19 (Coronavirus) in the United States: - Nancy Pelosi	Pearson Correlation	-.457**	.597**	1
	Sig. (2-tailed)	.000	.000	
	N	240	240	240
Using the scale provided, please indicate your personal opinion regarding how each of the following is handling the response to COVID-19 (Coronavirus) in the United States: - Mitch McConnell	Pearson Correlation	.393**	-.137*	.178**
	Sig. (2-tailed)	.000	.033	.006
	N	240	240	240

Correlations

		Using the scale provided, please indicate your personal opinion regarding how each of the following is handling the response to COVID-19 (Coronavirus) in the United States: - Mitch McConnell	Using the scale provided, please indicate your personal opinion regarding how each of the following is handling the response to COVID-19 (Coronavirus) in the United States: - Dr. Anthony Fauci	Using the scale provided, please indicate your personal opinion regarding how each of the following is handling the response to COVID-19 (Coronavirus) in the United States: - Republican Congress Members
Using the scale provided, please indicate your personal opinion regarding how each of the following is handling the response to COVID-19 (Coronavirus) in the United States: - Joe Biden	Pearson Correlation	-.137*	.380**	-.357**
	Sig. (2-tailed)	.033	.000	.000
	N	240	240	240
Using the scale provided, please indicate your personal opinion regarding how each of the following is handling the response to COVID-19 (Coronavirus) in the United States: - Nancy Pelosi	Pearson Correlation	.178**	.502**	-.069
	Sig. (2-tailed)	.006	.000	.287
	N	240	240	240
Using the scale provided, please indicate your personal opinion regarding how each of the following is handling the response to COVID-19 (Coronavirus) in the United States: - Mitch McConnell	Pearson Correlation	1	-.032	.517**
	Sig. (2-tailed)		.618	.000
	N	240	240	240

Correlations

		Using the scale provided, please indicate your personal opinion regarding how each of the following is handling the response to COVID-19 (Coronavirus) in the United States: - Democratic Congress Members	Using the scale provided, please indicate your personal opinion regarding how each of the following is handling the response to COVID-19 (Coronavirus) in the United States: - State Leaders/Governors	MRN	MGRS
Using the scale provided, please indicate your personal opinion regarding how each of the following is handling the response to COVID-19 (Coronavirus) in the United States: - Joe Biden	Pearson Correlation	.571**	.447**	-.386**	-.018
	Sig. (2-tailed)	.000	.000	.000	.783
	N	240	240	239	239
Using the scale provided, please indicate your personal opinion regarding how each of the following is handling the response to COVID-19 (Coronavirus) in the United States: - Nancy Pelosi	Pearson Correlation	.687**	.478**	-.351**	-.013
	Sig. (2-tailed)	.000	.000	.000	.843
	N	240	240	239	239
Using the scale provided, please indicate your personal opinion regarding how each of the following is handling the response to COVID-19 (Coronavirus) in the United States: - Mitch McConnell	Pearson Correlation	.136*	-.002	.257**	.121
	Sig. (2-tailed)	.035	.970	.000	.061
	N	240	240	239	239

Correlations

		Concern_Tot	Finance_Tot	Psychology_Tot
Using the scale provided, please indicate your personal opinion regarding how each of the following is handling the response to COVID-19 (Coronavirus) in the United States: - Joe Biden	Pearson Correlation	.444**	.137*	.228**
	Sig. (2-tailed)	.000	.035	.000
	N	240	240	240
Using the scale provided, please indicate your personal opinion regarding how each of the following is handling the response to COVID-19 (Coronavirus) in the United States: - Nancy Pelosi	Pearson Correlation	.341**	.210**	.158*
	Sig. (2-tailed)	.000	.001	.014
	N	240	240	240
Using the scale provided, please indicate your personal opinion regarding how each of the following is handling the response to COVID-19 (Coronavirus) in the United States: - Mitch McConnell	Pearson Correlation	-.275**	.010	-.035
	Sig. (2-tailed)	.000	.877	.593
	N	240	240	240

Correlations

		Risk_Rules	Risk_Help
Using the scale provided, please indicate your personal opinion regarding how each of the following is handling the response to COVID-19 (Coronavirus) in the United States: - Joe Biden	Pearson Correlation	-.489**	.127
	Sig. (2-tailed)	.000	.050
	N	240	240
Using the scale provided, please indicate your personal opinion regarding how each of the following is handling the response to COVID-19 (Coronavirus) in the United States: - Nancy Pelosi	Pearson Correlation	-.331**	.041
	Sig. (2-tailed)	.000	.530
	N	240	240
Using the scale provided, please indicate your personal opinion regarding how each of the following is handling the response to COVID-19 (Coronavirus) in the United States: - Mitch McConnell	Pearson Correlation	.259**	-.055
	Sig. (2-tailed)	.000	.396
	N	240	240

Correlations

		I have been diagnosed with coronavirus (COVID-19).	I have had coronavirus-like symptoms at some point in the last two months.	I have been sick with something other than the coronavirus in the last two months.
Using the scale provided, please indicate your personal opinion regarding how each of the following is handling the response to COVID-19 (Coronavirus) in the United States: - Dr. Anthony Fauci	Pearson Correlation	-.005	.001	.089
	Sig. (2-tailed)	.944	.989	.168
	N	240	240	240
Using the scale provided, please indicate your personal opinion regarding how each of the following is handling the response to COVID-19 (Coronavirus) in the United States: - Republican Congress Members	Pearson Correlation	-.050	-.033	-.033
	Sig. (2-tailed)	.441	.614	.607
	N	240	240	240
Using the scale provided, please indicate your personal opinion regarding how each of the following is handling the response to COVID-19 (Coronavirus) in the United States: - Democratic Congress Members	Pearson Correlation	.006	-.022	-.067
	Sig. (2-tailed)	.926	.733	.300
	N	240	240	240

Correlations

		I have been in close proximity with someone who has been diagnosed with coronavirus (COVID-19).	I have been in close proximity with someone who has had coronavirus-like symptoms in the last two months.	I watch a lot of news about the Coronavirus (COVID-19).
Using the scale provided, please indicate your personal opinion regarding how each of the following is handling the response to COVID-19 (Coronavirus) in the United States: - Dr. Anthony Fauci	Pearson Correlation	.009	.081	-.023
	Sig. (2-tailed)	.895	.210	.724
	N	240	240	240
Using the scale provided, please indicate your personal opinion regarding how each of the following is handling the response to COVID-19 (Coronavirus) in the United States: - Republican Congress Members	Pearson Correlation	-.139*	-.165*	.269**
	Sig. (2-tailed)	.032	.010	.000
	N	240	240	240
Using the scale provided, please indicate your personal opinion regarding how each of the following is handling the response to COVID-19 (Coronavirus) in the United States: - Democratic Congress Members	Pearson Correlation	-.074	.011	.009
	Sig. (2-tailed)	.254	.861	.889
	N	240	240	240

Correlations

		I spent a huge percentage of my time trying to find updates online or on TV about Coronavirus (COVID-19).	Which of the following best describes your political party affiliation?	Do you approve or disapprove of the way Donald Trump is handling his job as President?
Using the scale provided, please indicate your personal opinion regarding how each of the following is handling the response to COVID-19 (Coronavirus) in the United States: - Dr. Anthony Fauci	Pearson Correlation	-.070	-.303**	-.273**
	Sig. (2-tailed)	.281	.000	.000
	N	240	240	240
Using the scale provided, please indicate your personal opinion regarding how each of the following is handling the response to COVID-19 (Coronavirus) in the United States: - Republican Congress Members	Pearson Correlation	.148*	.508**	.597**
	Sig. (2-tailed)	.022	.000	.000
	N	240	240	240
Using the scale provided, please indicate your personal opinion regarding how each of the following is handling the response to COVID-19 (Coronavirus) in the United States: - Democratic Congress Members	Pearson Correlation	-.089	-.464**	-.413**
	Sig. (2-tailed)	.170	.000	.000
	N	240	240	240

Correlations

		Using the scale provided, please indicate your personal opinion regarding how each of the following is handling the response to COVID-19 (Coronavirus) in the United States: - Donald Trump	Using the scale provided, please indicate your personal opinion regarding how each of the following is handling the response to COVID-19 (Coronavirus) in the United States: - Joe Biden	Using the scale provided, please indicate your personal opinion regarding how each of the following is handling the response to COVID-19 (Coronavirus) in the United States: - Nancy Pelosi
Using the scale provided, please indicate your personal opinion regarding how each of the following is handling the response to COVID-19 (Coronavirus) in the United States: - Dr. Anthony Fauci	Pearson Correlation	-.302**	.380**	.502**
	Sig. (2-tailed)	.000	.000	.000
	N	240	240	240
Using the scale provided, please indicate your personal opinion regarding how each of the following is handling the response to COVID-19 (Coronavirus) in the United States: - Republican Congress Members	Pearson Correlation	.640**	-.357**	-.069
	Sig. (2-tailed)	.000	.000	.287
	N	240	240	240
Using the scale provided, please indicate your personal opinion regarding how each of the following is handling the response to COVID-19 (Coronavirus) in the United States: - Democratic Congress Members	Pearson Correlation	-.354**	.571**	.687**
	Sig. (2-tailed)	.000	.000	.000
	N	240	240	240

Correlations

		Using the scale provided, please indicate your personal opinion regarding how each of the following is handling the response to COVID-19 (Coronavirus) in the United States: - Mitch McConnell	Using the scale provided, please indicate your personal opinion regarding how each of the following is handling the response to COVID-19 (Coronavirus) in the United States: - Dr. Anthony Fauci	Using the scale provided, please indicate your personal opinion regarding how each of the following is handling the response to COVID-19 (Coronavirus) in the United States: - Republican Congress Members
Using the scale provided, please indicate your personal opinion regarding how each of the following is handling the response to COVID-19 (Coronavirus) in the United States: - Dr. Anthony Fauci	Pearson Correlation	-.032	1	-.135*
	Sig. (2-tailed)	.618		.037
	N	240	240	240
Using the scale provided, please indicate your personal opinion regarding how each of the following is handling the response to COVID-19 (Coronavirus) in the United States: - Republican Congress Members	Pearson Correlation	.517**	-.135*	1
	Sig. (2-tailed)	.000	.037	
	N	240	240	240
Using the scale provided, please indicate your personal opinion regarding how each of the following is handling the response to COVID-19 (Coronavirus) in the United States: - Democratic Congress Members	Pearson Correlation	.136*	.419**	.041
	Sig. (2-tailed)	.035	.000	.528
	N	240	240	240

Correlations

		Using the scale provided, please indicate your personal opinion regarding how each of the following is handling the response to COVID-19 (Coronavirus) in the United States: - Democratic Congress Members	Using the scale provided, please indicate your personal opinion regarding how each of the following is handling the response to COVID-19 (Coronavirus) in the United States: - State Leaders/Governors	MRN	MGRS
Using the scale provided, please indicate your personal opinion regarding how each of the following is handling the response to COVID-19 (Coronavirus) in the United States: - Dr. Anthony Fauci	Pearson Correlation	.419**	.402**	-.284**	-.033
	Sig. (2-tailed)	.000	.000	.000	.612
	N	240	240	239	239
Using the scale provided, please indicate your personal opinion regarding how each of the following is handling the response to COVID-19 (Coronavirus) in the United States: - Republican Congress Members	Pearson Correlation	.041	-.094	.280**	-.057
	Sig. (2-tailed)	.528	.145	.000	.376
	N	240	240	239	239
Using the scale provided, please indicate your personal opinion regarding how each of the following is handling the response to COVID-19 (Coronavirus) in the United States: - Democratic Congress Members	Pearson Correlation	1	.501**	-.315**	-.026
	Sig. (2-tailed)		.000	.000	.686
	N	240	240	239	239

Correlations

		Concern_Tot	Finance_Tot	Psychology_Tot
Using the scale provided, please indicate your personal opinion regarding how each of the following is handling the response to COVID-19 (Coronavirus) in the United States: - Dr. Anthony Fauci	Pearson Correlation	.273**	.137*	.109
	Sig. (2-tailed)	.000	.034	.091
	N	240	240	240
Using the scale provided, please indicate your personal opinion regarding how each of the following is handling the response to COVID-19 (Coronavirus) in the United States: - Republican Congress Members	Pearson Correlation	-.401**	-.100	-.177**
	Sig. (2-tailed)	.000	.124	.006
	N	240	240	240
Using the scale provided, please indicate your personal opinion regarding how each of the following is handling the response to COVID-19 (Coronavirus) in the United States: - Democratic Congress Members	Pearson Correlation	.259**	.131*	.162*
	Sig. (2-tailed)	.000	.042	.012
	N	240	240	240

Correlations

		Risk_Rules	Risk_Help
Using the scale provided, please indicate your personal opinion regarding how each of the following is handling the response to COVID-19 (Coronavirus) in the United States: - Dr. Anthony Fauci	Pearson Correlation	-.374**	.129*
	Sig. (2-tailed)	.000	.046
	N	240	240
Using the scale provided, please indicate your personal opinion regarding how each of the following is handling the response to COVID-19 (Coronavirus) in the United States: - Republican Congress Members	Pearson Correlation	.351**	-.123
	Sig. (2-tailed)	.000	.057
	N	240	240
Using the scale provided, please indicate your personal opinion regarding how each of the following is handling the response to COVID-19 (Coronavirus) in the United States: - Democratic Congress Members	Pearson Correlation	-.260**	.099
	Sig. (2-tailed)	.000	.127
	N	240	240

Correlations

		I have been diagnosed with coronavirus (COVID-19).	I have had coronavirus-like symptoms at some point in the last two months.	I have been sick with something other than the coronavirus in the last two months.
Using the scale provided, please indicate your personal opinion regarding how each of the following is handling the response to COVID-19 (Coronavirus) in the United States: - State Leaders/Governors	Pearson Correlation	-.034	.040	.089
	Sig. (2-tailed)	.605	.539	.170
	N	240	240	240
MRN	Pearson Correlation	.012	.076	.041
	Sig. (2-tailed)	.850	.241	.531
	N	240	240	240
MGRS	Pearson Correlation	-.074	-.049	-.081
	Sig. (2-tailed)	.255	.450	.212
	N	240	240	240
Concern_Tot	Pearson Correlation	.047	-.019	.041
	Sig. (2-tailed)	.470	.769	.530
	N	241	241	241
Finance_Tot	Pearson Correlation	-.036	-.021	.021
	Sig. (2-tailed)	.582	.745	.750
	N	241	241	241
Psychology_Tot	Pearson Correlation	-.106	-.123	-.071
	Sig. (2-tailed)	.101	.056	.269
	N	241	241	241

Correlations

		I have been in close proximity with someone who has been diagnosed with coronavirus (COVID-19).	I have been in close proximity with someone who has had coronavirus-like symptoms in the last two months.	I watch a lot of news about the Coronavirus (COVID-19).
Using the scale provided, please indicate your personal opinion regarding how each of the following is handling the response to COVID-19 (Coronavirus) in the United States: - State Leaders/Governors	Pearson Correlation	.067	.108	-.107
	Sig. (2-tailed)	.298	.096	.098
	N	240	240	240
MRN	Pearson Correlation	.023	-.022	.118
	Sig. (2-tailed)	.728	.732	.068
	N	240	240	240
MGRS	Pearson Correlation	.029	.064	-.137*
	Sig. (2-tailed)	.655	.324	.034
	N	240	240	240
Concern_Tot	Pearson Correlation	.074	.093	-.243**
	Sig. (2-tailed)	.251	.149	.000
	N	241	241	241
Finance_Tot	Pearson Correlation	.011	.100	-.075
	Sig. (2-tailed)	.866	.122	.243
	N	241	241	241
Psychology_Tot	Pearson Correlation	-.039	-.041	-.130*
	Sig. (2-tailed)	.545	.526	.044
	N	241	241	241

Correlations

		I spent a huge percentage of my time trying to find updates online or on TV about Coronavirus (COVID-19).	Which of the following best describes your political party affiliation?	Do you approve or disapprove of the way Donald Trump is handling his job as President?
Using the scale provided, please indicate your personal opinion regarding how each of the following is handling the response to COVID-19 (Coronavirus) in the United States: - State Leaders/Governors	Pearson Correlation	-.136*	-.323**	-.305**
	Sig. (2-tailed)	.036	.000	.000
	N	240	240	240
MRN	Pearson Correlation	.077	.465**	.480**
	Sig. (2-tailed)	.235	.000	.000
	N	240	240	239
MGRS	Pearson Correlation	-.068	-.070	.008
	Sig. (2-tailed)	.291	.282	.906
	N	240	240	239
Concern_Tot	Pearson Correlation	-.230**	-.429**	-.468**
	Sig. (2-tailed)	.000	.000	.000
	N	241	241	240
Finance_Tot	Pearson Correlation	-.033	-.120	-.178**
	Sig. (2-tailed)	.609	.062	.006
	N	241	241	240
Psychology_Tot	Pearson Correlation	-.118	-.256**	-.349**
	Sig. (2-tailed)	.067	.000	.000
	N	241	241	240

Correlations

		Using the scale provided, please indicate your personal opinion regarding how each of the following is handling the response to COVID-19 (Coronavirus) in the United States: - Donald Trump	Using the scale provided, please indicate your personal opinion regarding how each of the following is handling the response to COVID-19 (Coronavirus) in the United States: - Joe Biden	Using the scale provided, please indicate your personal opinion regarding how each of the following is handling the response to COVID-19 (Coronavirus) in the United States: - Nancy Pelosi
Using the scale provided, please indicate your personal opinion regarding how each of the following is handling the response to COVID-19 (Coronavirus) in the United States: - State Leaders/Governors	Pearson Correlation	-.263**	.447**	.478**
	Sig. (2-tailed)	.000	.000	.000
	N	240	240	240
MRN	Pearson Correlation	.474**	-.386**	-.351**
	Sig. (2-tailed)	.000	.000	.000
	N	239	239	239
MGRS	Pearson Correlation	.012	-.018	-.013
	Sig. (2-tailed)	.857	.783	.843
	N	239	239	239
Concern_Tot	Pearson Correlation	-.458**	.444**	.341**
	Sig. (2-tailed)	.000	.000	.000
	N	240	240	240
Finance_Tot	Pearson Correlation	-.147*	.137*	.210**
	Sig. (2-tailed)	.023	.035	.001
	N	240	240	240
Psychology_Tot	Pearson Correlation	-.322**	.228**	.158*
	Sig. (2-tailed)	.000	.000	.014
	N	240	240	240

Correlations

		Using the scale provided, please indicate your personal opinion regarding how each of the following is handling the response to COVID-19 (Coronavirus) in the United States: - Mitch McConnell	Using the scale provided, please indicate your personal opinion regarding how each of the following is handling the response to COVID-19 (Coronavirus) in the United States: - Dr. Anthony Fauci	Using the scale provided, please indicate your personal opinion regarding how each of the following is handling the response to COVID-19 (Coronavirus) in the United States: - Republican Congress Members
Using the scale provided, please indicate your personal opinion regarding how each of the following is handling the response to COVID-19 (Coronavirus) in the United States: - State Leaders/Governors	Pearson Correlation	-.002	.402**	-.094
	Sig. (2-tailed)	.970	.000	.145
	N	240	240	240
MRN	Pearson Correlation	.257**	-.284**	.280**
	Sig. (2-tailed)	.000	.000	.000
	N	239	239	239
MGRS	Pearson Correlation	.121	-.033	-.057
	Sig. (2-tailed)	.061	.612	.376
	N	239	239	239
Concern_Tot	Pearson Correlation	-.275**	.273**	-.401**
	Sig. (2-tailed)	.000	.000	.000
	N	240	240	240
Finance_Tot	Pearson Correlation	.010	.137*	-.100
	Sig. (2-tailed)	.877	.034	.124
	N	240	240	240
Psychology_Tot	Pearson Correlation	-.035	.109	-.177**
	Sig. (2-tailed)	.593	.091	.006
	N	240	240	240

Correlations

		Using the scale provided, please indicate your personal opinion regarding how each of the following is handling the response to COVID-19 (Coronavirus) in the United States: - Democratic Congress Members	Using the scale provided, please indicate your personal opinion regarding how each of the following is handling the response to COVID-19 (Coronavirus) in the United States: - State Leaders/Governors	MRN	MGRS
Using the scale provided, please indicate your personal opinion regarding how each of the following is handling the response to COVID-19 (Coronavirus) in the United States: - State Leaders/Governors	Pearson Correlation	.501**	1	-.176**	.028
	Sig. (2-tailed)	.000		.007	.666
	N	240	240	239	239
MRN	Pearson Correlation	-.315**	-.176**	1	.112
	Sig. (2-tailed)	.000	.007		.084
	N	239	239	240	239
MGRS	Pearson Correlation	-.026	.028	.112	1
	Sig. (2-tailed)	.686	.666	.084	
	N	239	239	239	240
Concern_Tot	Pearson Correlation	.259**	.276**	-.391**	.109
	Sig. (2-tailed)	.000	.000	.000	.092
	N	240	240	240	240
Finance_Tot	Pearson Correlation	.131*	.099	-.168**	-.010
	Sig. (2-tailed)	.042	.127	.009	.878
	N	240	240	240	240
Psychology_Tot	Pearson Correlation	.162*	.031	-.255**	.056
	Sig. (2-tailed)	.012	.633	.000	.389
	N	240	240	240	240

Correlations

		Concern_Tot	Finance_Tot	Psychology_Tot
Using the scale provided, please indicate your personal opinion regarding how each of the following is handling the response to COVID-19 (Coronavirus) in the United States: - State Leaders/Governors	Pearson Correlation	.276**	.099	.031
	Sig. (2-tailed)	.000	.127	.633
	N	240	240	240
MRN	Pearson Correlation	-.391**	-.168**	-.255**
	Sig. (2-tailed)	.000	.009	.000
	N	240	240	240
MGRS	Pearson Correlation	.109	-.010	.056
	Sig. (2-tailed)	.092	.878	.389
	N	240	240	240
Concern_Tot	Pearson Correlation	1	.216**	.353**
	Sig. (2-tailed)		.001	.000
	N	241	241	241
Finance_Tot	Pearson Correlation	.216**	1	.246**
	Sig. (2-tailed)	.001		.000
	N	241	241	241
Psychology_Tot	Pearson Correlation	.353**	.246**	1
	Sig. (2-tailed)	.000	.000	
	N	241	241	241

Correlations

		Risk_Rules	Risk_Help
Using the scale provided, please indicate your personal opinion regarding how each of the following is handling the response to COVID-19 (Coronavirus) in the United States: - State Leaders/Governors	Pearson Correlation	-.309**	.144*
	Sig. (2-tailed)	.000	.026
	N	240	240
MRN	Pearson Correlation	.483**	-.091
	Sig. (2-tailed)	.000	.158
	N	240	240
MGRS	Pearson Correlation	-.006	.015
	Sig. (2-tailed)	.927	.816
	N	240	240
Concern_Tot	Pearson Correlation	-.700**	.076
	Sig. (2-tailed)	.000	.239
	N	241	241
Finance_Tot	Pearson Correlation	-.158*	.137*
	Sig. (2-tailed)	.014	.034
	N	241	241
Psychology_Tot	Pearson Correlation	-.167**	.004
	Sig. (2-tailed)	.010	.953
	N	241	241

Correlations

		I have been diagnosed with coronavirus (COVID-19).	I have had coronavirus-like symptoms at some point in the last two months.	I have been sick with something other than the coronavirus in the last two months.
Risk_Rules	Pearson Correlation	.002	.015	-.043
	Sig. (2-tailed)	.977	.822	.509
	N	241	241	241
Risk_Help	Pearson Correlation	.028	.038	-.022
	Sig. (2-tailed)	.665	.552	.734
	N	241	241	241

Correlations

		I have been in close proximity with someone who has been diagnosed with coronavirus (COVID-19).	I have been in close proximity with someone who has had coronavirus-like symptoms in the last two months.	I watch a lot of news about the Coronavirus (COVID-19).
Risk_Rules	Pearson Correlation	-.038	-.156*	.215**
	Sig. (2-tailed)	.558	.015	.001
	N	241	241	241
Risk_Help	Pearson Correlation	-.029	.080	-.103
	Sig. (2-tailed)	.651	.214	.112
	N	241	241	241

Correlations

		I spent a huge percentage of my time trying to find updates online or on TV about Coronavirus (COVID-19).	Which of the following best describes your political party affiliation?	Do you approve or disapprove of the way Donald Trump is handling his job as President?
Risk_Rules	Pearson Correlation	.149*	.421**	.436**
	Sig. (2-tailed)	.021	.000	.000
	N	241	241	240
Risk_Help	Pearson Correlation	.006	-.166**	-.111
	Sig. (2-tailed)	.926	.010	.087
	N	241	241	240

Correlations

		Using the scale provided, please indicate your personal opinion regarding how each of the following is handling the response to COVID-19 (Coronavirus) in the United States: - Donald Trump	Using the scale provided, please indicate your personal opinion regarding how each of the following is handling the response to COVID-19 (Coronavirus) in the United States: - Joe Biden	Using the scale provided, please indicate your personal opinion regarding how each of the following is handling the response to COVID-19 (Coronavirus) in the United States: - Nancy Pelosi
Risk_Rules	Pearson Correlation	.442**	-.489**	-.331**
	Sig. (2-tailed)	.000	.000	.000
	N	240	240	240
Risk_Help	Pearson Correlation	-.097	.127	.041
	Sig. (2-tailed)	.133	.050	.530
	N	240	240	240

Correlations

		Using the scale provided, please indicate your personal opinion regarding how each of the following is handling the response to COVID-19 (Coronavirus) in the United States: - Mitch McConnell	Using the scale provided, please indicate your personal opinion regarding how each of the following is handling the response to COVID-19 (Coronavirus) in the United States: - Dr. Anthony Fauci	Using the scale provided, please indicate your personal opinion regarding how each of the following is handling the response to COVID-19 (Coronavirus) in the United States: - Republican Congress Members
Risk_Rules	Pearson Correlation	.259**	-.374**	.351**
	Sig. (2-tailed)	.000	.000	.000
	N	240	240	240
Risk_Help	Pearson Correlation	-.055	.129*	-.123
	Sig. (2-tailed)	.396	.046	.057
	N	240	240	240

Correlations

		Using the scale provided, please indicate your personal opinion regarding how each of the following is handling the response to COVID-19 (Coronavirus) in the United States: - Democratic Congress Members	Using the scale provided, please indicate your personal opinion regarding how each of the following is handling the response to COVID-19 (Coronavirus) in the United States: - State Leaders/Governors	MRN	MGRS
Risk_Rules	Pearson Correlation	-.260**	-.309**	.483**	-.006
	Sig. (2-tailed)	.000	.000	.000	.927
	N	240	240	240	240
Risk_Help	Pearson Correlation	.099	.144*	-.091	.015
	Sig. (2-tailed)	.127	.026	.158	.816
	N	240	240	240	240

Correlations

		Concern_Tot	Finance_Tot	Psychology_Tot
Risk_Rules	Pearson Correlation	-.700**	-.158*	-.167**
	Sig. (2-tailed)	.000	.014	.010
	N	241	241	241
Risk_Help	Pearson Correlation	.076	.137*	.004
	Sig. (2-tailed)	.239	.034	.953
	N	241	241	241

Correlations

		Risk_Rules	Risk_Help
Risk_Rules	Pearson Correlation	1	-.084
	Sig. (2-tailed)		.193
	N	241	241
Risk_Help	Pearson Correlation	-.084	1
	Sig. (2-tailed)	.193	
	N	241	241

**. Correlation is significant at the 0.01 level (2-tailed).

*. Correlation is significant at the 0.05 level (2-tailed).

```
 **Contrast Codes and Mean Centering**

IF (Gender=1) GenderCC=1.
IF (Gender=2) GenderCC=1.
IF(Gender=3) GenderCC=-1.
IF (Gender=4) GenderCC=-1.

IF (Race=1) RaceCC=1.
IF (Race>=2) RaceCC=-1.

DESCRIPTIVES VARIABLES=MGRS MRN SES PParty PIdeology
  /STATISTICS=MEAN STDDEV MIN MAX.
```

Descriptives

Notes

Output Created		15-DEC-2021 13:07:56
Comments		
Input	Data	C: \Users\njs5478\Dropbox\HM and COVID\0. Revise and Resubmit\2. R and R Data\Study 1b\Study1b_Data.sav
	Active Dataset	DataSet1
	Filter	<none>
	Weight	<none>
	Split File	<none>
	N of Rows in Working Data File	241
Missing Value Handling	Definition of Missing	User defined missing values are treated as missing.
	Cases Used	All non-missing data are used.
Syntax		DESCRIPTIVES VARIABLES=MGRS MRN SES PParty PIdeology /STATISTICS=MEAN STDDEV MIN MAX.
Resources	Processor Time	00:00:00.00
	Elapsed Time	00:00:00.00

Descriptive Statistics

	N	Minimum	Maximum	Mean	Std. Deviation
MGRS	240	1.00	4.90	2.4581	.64339
MRN	240	1.08	5.35	3.2638	.86575
Self Reported Socioeconomic Status	241	1	5	3.41	.807
Which of the following best describes your political party affiliation?	241	1	5	2.62	1.385
Which of the following best describes your political ideology?	241	1	7	3.58	1.585
Valid N (listwise)	239				

```
 COMPUTE Party0=PParty-2.62.
COMPUTE Ideology0=PIdeology-3.58.
COMPUTE SES0=SES-3.41.
COMPUTE MRN0=MRN-3.2638.
COMPUTE MGRS0=MGRS-2.4581.

**Interactions**

COMPUTE MRN0xRace=MRN0*RaceCC.
COMPUTE MRN0xSES0=MRN0*SES0.
COMPUTE MRN0xGender=MRN0*GenderCC.
COMPUTE MRN0xParty0=MRN0*Party0.
COMPUTE MRN0xIdeology0=MRN0*Ideology0.
COMPUTE MRN0xMGRS0=MRN0*MGRS0.
COMPUTE MGRS0xGender=MGRS0*GenderCC.
COMPUTE MGRS0xRace=MGRS0*RaceCC.
COMPUTE MGRS0xSES0=MGRS0*SES0.
COMPUTE MGRS0xParty0=MGRS0*Party0.
COMPUTE MGRS0xIdeology0=MGRS0*Ideology0.

IF (Trump<8) TrumpX=Trump.
IF(Biden<8) BidenX=Biden.
IF (Pelosi<8) PelosiX=Pelosi.
IF (McConnell<8) McConnellX=McConnell.
IF (RepCongress<8) RepCongressX=RepCongress.
IF (DemCongress<8) DemCongressX=DemCongress.
IF(Fauci<8) FauciX=Fauci.
IF(State<8)StateX=State.

**Regression Analyses**

**Without Precarious Masculinity (with PParty)

REGRESSION
  /MISSING LISTWISE
  /STATISTICS COEFF OUTS R ANOVA CHANGE ZPP
  /CRITERIA=PIN(.05) POUT(.10)
  /NOORIGIN
  /DEPENDENT Concern_Tot
  /METHOD=ENTER Party0
  /METHOD=ENTER GenderCC RaceCC SES0
  /METHOD=ENTER MRN0
  /METHOD=ENTER MRN0xRace MRN0xSES0 MRN0xGender MRN0xParty0.
```

Regression

Notes

Output Created		15-DEC-2021 13:07:57
Comments		
Input	Data	C:\Users\njs5478\Dropbox\HM and COVID\0. Revise and Resubmit\2. R and R Data\Study 1b\Study1b_Data.sav
	Active Dataset	DataSet1
	Filter	<none>
	Weight	<none>
	Split File	<none>
	N of Rows in Working Data File	241
Missing Value Handling	Definition of Missing	User-defined missing values are treated as missing.
	Cases Used	Statistics are based on cases with no missing values for any variable used.
Syntax		REGRESSION /MISSING LISTWISE /STATISTICS COEFF OUTS R ANOVA CHANGE ZPP /CRITERIA=PIN(.05) POUT(.10) /NOORIGIN /DEPENDENT Concern_Tot /METHOD=ENTER Party0 /METHOD=ENTER GenderCC RaceCC SES0 /METHOD=ENTER MRN0 /METHOD=ENTER MRN0xRace MRN0xSES0 MRN0xGender MRN0xParty0.
Resources	Processor Time	00:00:00.03
	Elapsed Time	00:00:00.03
	Memory Required	45472 bytes
	Additional Memory Required for Residual Plots	0 bytes

Variables Entered/Removed[a]

Model	Variables Entered	Variables Removed	Method
1	Party0[b]	.	Enter
2	SES0, GenderCC, RaceCC[b]	.	Enter
3	MRN0[b]	.	Enter
4	MRN0xSES0, MRN0xGender, MRN0xParty0, MRN0xRace[b]	.	Enter

a. Dependent Variable: Concern_Tot

b. All requested variables entered.

Model Summary

Model	R	R Square	Adjusted R Square	Std. Error of the Estimate	Change Statistics		
					R Square Change	F Change	df1
1	.434[a]	.189	.185	1.25500	.189	54.872	1
2	.460[b]	.211	.198	1.24535	.023	2.224	3
3	.492[c]	.242	.226	1.22329	.031	9.482	1
4	.527[d]	.278	.250	1.20432	.036	2.841	4

Model Summary

Model	Change Statistics	
	df2	Sig. F Change
1	236	.000
2	233	.086
3	232	.002
4	228	.025

a. Predictors: (Constant), Party0

b. Predictors: (Constant), Party0, SES0, GenderCC, RaceCC

c. Predictors: (Constant), Party0, SES0, GenderCC, RaceCC, MRN0

d. Predictors: (Constant), Party0, SES0, GenderCC, RaceCC, MRN0, MRN0xSES0, MRN0xGender, MRN0xParty0, MRN0xRace

ANOVA[a]

Model		Sum of Squares	df	Mean Square	F	Sig.
1	Regression	86.425	1	86.425	54.872	.000[b]
	Residual	371.708	236	1.575		
	Total	458.133	237			
2	Regression	96.772	4	24.193	15.599	.000[c]
	Residual	361.361	233	1.551		
	Total	458.133	237			
3	Regression	110.962	5	22.192	14.830	.000[d]
	Residual	347.171	232	1.496		
	Total	458.133	237			
4	Regression	127.444	9	14.160	9.763	.000[e]
	Residual	330.689	228	1.450		
	Total	458.133	237			

a. Dependent Variable: Concern_Tot

b. Predictors: (Constant), Party0

c. Predictors: (Constant), Party0, SES0, GenderCC, RaceCC

d. Predictors: (Constant), Party0, SES0, GenderCC, RaceCC, MRN0

e. Predictors: (Constant), Party0, SES0, GenderCC, RaceCC, MRN0, MRN0xSES0, MRN0xGender, MRN0xParty0, MRN0xRace

Coefficients[a]

Model		Unstandardized Coefficients B	Unstandardized Coefficients Std. Error	Standardized Coefficients Beta	t	Sig.
1	(Constant)	4.335	.081		53.293	.000
	Party0	-.434	.059	-.434	-7.408	.000
2	(Constant)	4.375	.094		46.522	.000
	Party0	-.384	.063	-.384	-6.140	.000
	GenderCC	-.205	.083	-.148	-2.463	.015
	RaceCC	-.083	.097	-.052	-.860	.391
	SES0	-.001	.100	-.001	-.012	.991
3	(Constant)	4.389	.092		47.454	.000
	Party0	-.296	.068	-.296	-4.375	.000
	GenderCC	-.110	.087	-.080	-1.263	.208
	RaceCC	-.111	.096	-.070	-1.162	.246
	SES0	.032	.099	.019	.325	.745
	MRN0	-.345	.112	-.215	-3.079	.002
4	(Constant)	4.358	.105		41.537	.000
	Party0	-.268	.068	-.268	-3.925	.000
	GenderCC	-.155	.088	-.112	-1.760	.080
	RaceCC	-.131	.096	-.082	-1.362	.175
	SES0	.022	.098	.013	.220	.826
	MRN0	-.182	.124	-.113	-1.464	.144
	MRN0xRace	-.270	.108	-.168	-2.505	.013
	MRN0xSES0	.232	.103	.130	2.240	.026
	MRN0xGender	.022	.105	.012	.208	.836
	MRN0xParty0	.041	.070	.036	.587	.558

Coefficients[a]

Model		Correlations Zero-order	Partial	Part
1	(Constant)			
	Party0	-.434	-.434	-.434
2	(Constant)			
	Party0	-.434	-.373	-.357
	GenderCC	-.242	-.159	-.143
	RaceCC	-.166	-.056	-.050
	SES0	-.026	-.001	-.001
3	(Constant)			
	Party0	-.434	-.276	-.250
	GenderCC	-.242	-.083	-.072
	RaceCC	-.166	-.076	-.066
	SES0	-.026	.021	.019
	MRN0	-.387	-.198	-.176
4	(Constant)			
	Party0	-.434	-.252	-.221
	GenderCC	-.242	-.116	-.099
	RaceCC	-.166	-.090	-.077
	SES0	-.026	.015	.012
	MRN0	-.387	-.097	-.082
	MRN0xRace	-.325	-.164	-.141
	MRN0xSES0	.197	.147	.126
	MRN0xGender	-.077	.014	.012
	MRN0xParty0	-.052	.039	.033

a. Dependent Variable: Concern_Tot

Excluded Variables[a]

Model		Beta In	t	Sig.	Partial Correlation	Collinearity Statistics Tolerance
1	GenderCC	-.146[b]	-2.442	.015	-.157	.942
	RaceCC	-.047[b]	-.763	.446	-.050	.919
	SES0	-.008[b]	-.137	.891	-.009	.998
	MRN0	-.236[b]	-3.658	.000	-.232	.784
	MRN0xRace	-.203[b]	-3.326	.001	-.212	.888
	MRN0xSES0	.151[b]	2.582	.010	.166	.988
	MRN0xGender	-.026[b]	-.446	.656	-.029	.986
	MRN0xParty0	.014[b]	.236	.813	.015	.977
2	MRN0	-.215[c]	-3.079	.002	-.198	.671
	MRN0xRace	-.205[c]	-3.353	.001	-.215	.867
	MRN0xSES0	.158[c]	2.729	.007	.176	.984
	MRN0xGender	-.038[c]	-.645	.520	-.042	.968
	MRN0xParty0	.030[c]	.496	.620	.033	.949
3	MRN0xRace	-.157[d]	-2.410	.017	-.157	.753
	MRN0xSES0	.130[d]	2.240	.026	.146	.952
	MRN0xGender	-.009[d]	-.157	.875	-.010	.942
	MRN0xParty0	.004[d]	.061	.951	.004	.929

a. Dependent Variable: Concern_Tot

b. Predictors in the Model: (Constant), Party0

c. Predictors in the Model: (Constant), Party0, SES0, GenderCC, RaceCC

d. Predictors in the Model: (Constant), Party0, SES0, GenderCC, RaceCC, MRN0

```
REGRESSION
 /MISSING LISTWISE
 /STATISTICS COEFF OUTS R ANOVA CHANGE ZPP
 /CRITERIA=PIN(.05) POUT(.10)
 /NOORIGIN
 /DEPENDENT Finance_Tot
 /METHOD=ENTER Party0
 /METHOD=ENTER GenderCC RaceCC SES0
 /METHOD=ENTER MRN0
 /METHOD=ENTER MRN0xRace MRN0xSES0 MRN0xGender MRN0xParty0.
```

Regression

Notes

Output Created		15-DEC-2021 13:07:57
Comments		
Input	Data	C:\Users\njs5478\Dropbox\HM and COVID\0. Revise and Resubmit\2. R and R Data\Study 1b\Study1b_Data.sav
	Active Dataset	DataSet1
	Filter	<none>
	Weight	<none>
	Split File	<none>
	N of Rows in Working Data File	241
Missing Value Handling	Definition of Missing	User-defined missing values are treated as missing.
	Cases Used	Statistics are based on cases with no missing values for any variable used.
Syntax		REGRESSION<br>/MISSING LISTWISE<br>/STATISTICS COEFF OUTS R ANOVA CHANGE ZPP<br>/CRITERIA=PIN(.05) POUT(.10)<br>/NOORIGIN<br>/DEPENDENT Finance_Tot<br>/METHOD=ENTER Party0<br>/METHOD=ENTER GenderCC RaceCC SES0<br>/METHOD=ENTER MRN0<br>/METHOD=ENTER MRN0xRace MRN0xSES0 MRN0xGender MRN0xParty0.
Resources	Processor Time	00:00:00.02
	Elapsed Time	00:00:00.02

Notes

	Memory Required	45472 bytes
	Additional Memory Required for Residual Plots	0 bytes

Variables Entered/Removed[a]

Model	Variables Entered	Variables Removed	Method
1	Party0[b]	.	Enter
2	SES0, GenderCC, RaceCC[b]	.	Enter
3	MRN0[b]	.	Enter
4	MRN0xSES0, MRN0xGender, MRN0xParty0, MRN0xRace[b]	.	Enter

a. Dependent Variable: Finance_Tot

b. All requested variables entered.

Model Summary

Model	R	R Square	Adjusted R Square	Std. Error of the Estimate	Change Statistics		
					R Square Change	F Change	df1
1	.127[a]	.016	.012	1.52522	.016	3.859	1
2	.219[b]	.048	.031	1.51010	.032	2.583	3
3	.230[c]	.053	.032	1.50939	.005	1.218	1
4	.307[d]	.094	.059	1.48870	.042	2.623	4

Model Summary

Model	Change Statistics	
	df2	Sig. F Change
1	236	.051
2	233	.054
3	232	.271
4	228	.036

a. Predictors: (Constant), Party0

b. Predictors: (Constant), Party0, SES0, GenderCC, RaceCC

c. Predictors: (Constant), Party0, SES0, GenderCC, RaceCC, MRN0

d. Predictors: (Constant), Party0, SES0, GenderCC, RaceCC, MRN0, MRN0xSES0, MRN0xGender, MRN0xParty0, MRN0xRace

ANOVA[a]

Model		Sum of Squares	df	Mean Square	F	Sig.
1	Regression	8.976	1	8.976	3.859	.051[b]
	Residual	549.004	236	2.326		
	Total	557.980	237			
2	Regression	26.646	4	6.662	2.921	.022[c]
	Residual	531.334	233	2.280		
	Total	557.980	237			
3	Regression	29.422	5	5.884	2.583	.027[d]
	Residual	528.558	232	2.278		
	Total	557.980	237			
4	Regression	52.679	9	5.853	2.641	.006[e]
	Residual	505.301	228	2.216		
	Total	557.980	237			

a. Dependent Variable: Finance_Tot

b. Predictors: (Constant), Party0

c. Predictors: (Constant), Party0, SES0, GenderCC, RaceCC

d. Predictors: (Constant), Party0, SES0, GenderCC, RaceCC, MRN0

e. Predictors: (Constant), Party0, SES0, GenderCC, RaceCC, MRN0, MRN0xSES0, MRN0xGender, MRN0xParty0, MRN0xRace

Coefficients[a]

Model		Unstandardized Coefficients B	Unstandardized Coefficients Std. Error	Standardized Coefficients Beta	t	Sig.
1	(Constant)	3.900	.099		39.444	.000
	Party0	-.140	.071	-.127	-1.964	.051
2	(Constant)	3.914	.114		34.323	.000
	Party0	-.096	.076	-.087	-1.265	.207
	GenderCC	-.183	.101	-.120	-1.815	.071
	RaceCC	-.037	.118	-.021	-.318	.751
	SES0	-.244	.122	-.129	-2.008	.046
3	(Constant)	3.920	.114		34.353	.000
	Party0	-.057	.084	-.052	-.684	.495
	GenderCC	-.141	.108	-.092	-1.311	.191
	RaceCC	-.050	.118	-.028	-.421	.674
	SES0	-.229	.122	-.121	-1.876	.062
	MRN0	-.152	.138	-.086	-1.104	.271
4	(Constant)	3.795	.130		29.258	.000
	Party0	-.055	.084	-.050	-.653	.514
	GenderCC	-.181	.109	-.118	-1.654	.099
	RaceCC	-.034	.119	-.019	-.286	.775
	SES0	-.234	.122	-.124	-1.929	.055
	MRN0	-.052	.153	-.029	-.336	.737
	MRN0xRace	-.107	.133	-.060	-.804	.422
	MRN0xSES0	.370	.128	.188	2.889	.004
	MRN0xGender	.165	.129	.085	1.275	.204
	MRN0xParty0	.056	.087	.044	.643	.521

Coefficients[a]

Model		Correlations Zero-order	Partial	Part
1	(Constant)			
	Party0	-.127	-.127	-.127
2	(Constant)			
	Party0	-.127	-.083	-.081
	GenderCC	-.148	-.118	-.116
	RaceCC	-.055	-.021	-.020
	SES0	-.139	-.130	-.128
3	(Constant)			
	Party0	-.127	-.045	-.044
	GenderCC	-.148	-.086	-.084
	RaceCC	-.055	-.028	-.027
	SES0	-.139	-.122	-.120
	MRN0	-.165	-.072	-.071
4	(Constant)			
	Party0	-.127	-.043	-.041
	GenderCC	-.148	-.109	-.104
	RaceCC	-.055	-.019	-.018
	SES0	-.139	-.127	-.122
	MRN0	-.165	-.022	-.021
	MRN0xRace	-.099	-.053	-.051
	MRN0xSES0	.194	.188	.182
	MRN0xGender	.041	.084	.080
	MRN0xParty0	.043	.043	.041

a. Dependent Variable: Finance_Tot

Excluded Variables[a]

Model		Beta In	t	Sig.	Partial Correlation	Collinearity Statistics Tolerance
1	GenderCC	-.125[b]	-1.883	.061	-.122	.942
	RaceCC	-.020[b]	-.298	.766	-.019	.919
	SES0	-.134[b]	-2.090	.038	-.135	.998
	MRN0	-.135[b]	-1.858	.064	-.120	.784
	MRN0xRace	-.063[b]	-.923	.357	-.060	.888
	MRN0xSES0	.182[b]	2.838	.005	.182	.988
	MRN0xGender	.057[b]	.871	.385	.057	.986
	MRN0xParty0	.063[b]	.970	.333	.063	.977
2	MRN0	-.086[c]	-1.104	.271	-.072	.671
	MRN0xRace	-.061[c]	-.892	.374	-.058	.867
	MRN0xSES0	.188[c]	2.969	.003	.191	.984
	MRN0xGender	.056[c]	.857	.392	.056	.968
	MRN0xParty0	.059[c]	.902	.368	.059	.949
3	MRN0xRace	-.039[d]	-.527	.599	-.035	.753
	MRN0xSES0	.181[d]	2.809	.005	.182	.952
	MRN0xGender	.069[d]	1.052	.294	.069	.942
	MRN0xParty0	.050[d]	.750	.454	.049	.929

a. Dependent Variable: Finance_Tot

b. Predictors in the Model: (Constant), Party0

c. Predictors in the Model: (Constant), Party0, SES0, GenderCC, RaceCC

d. Predictors in the Model: (Constant), Party0, SES0, GenderCC, RaceCC, MRN0

```
REGRESSION
 /MISSING LISTWISE
 /STATISTICS COEFF OUTS R ANOVA CHANGE ZPP
 /CRITERIA=PIN(.05) POUT(.10)
 /NOORIGIN
 /DEPENDENT Resource_Tot
 /METHOD=ENTER Party0
 /METHOD=ENTER GenderCC RaceCC SES0
 /METHOD=ENTER MRN0
 /METHOD=ENTER MRN0xRace MRN0xSES0 MRN0xGender MRN0xParty0.
```

Regression

Notes

Output Created		15-DEC-2021 13:07:57
Comments		
Input	Data	C:<br>\Users\njs5478\Dropbox\HM and COVID\0. Revise and Resubmit\2. R and R Data\Study 1b\Study1b_Data.sav
	Active Dataset	DataSet1
	Filter	<none>
	Weight	<none>
	Split File	<none>
	N of Rows in Working Data File	241
Missing Value Handling	Definition of Missing	User-defined missing values are treated as missing.
	Cases Used	Statistics are based on cases with no missing values for any variable used.
Syntax		REGRESSION<br>/MISSING LISTWISE<br>/STATISTICS COEFF OUTS R ANOVA CHANGE ZPP<br>/CRITERIA=PIN(.05) POUT(.10)<br>/NOORIGIN<br>/DEPENDENT Resource_Tot<br>/METHOD=ENTER Party0<br>/METHOD=ENTER GenderCC RaceCC SES0<br>/METHOD=ENTER MRN0<br>/METHOD=ENTER MRN0xRace MRN0xSES0 MRN0xGender MRN0xParty0.
Resources	Processor Time	00:00:00.05
	Elapsed Time	00:00:00.02

Notes

	Memory Required	45472 bytes
	Additional Memory Required for Residual Plots	0 bytes

Variables Entered/Removed[a]

Model	Variables Entered	Variables Removed	Method
1	Party0[b]	.	Enter
2	SES0, GenderCC, RaceCC[b]	.	Enter
3	MRN0[b]	.	Enter
4	MRN0xSES0, MRN0xGender, MRN0xParty0, MRN0xRace[b]	.	Enter

a. Dependent Variable: Resource_Tot

b. All requested variables entered.

Model Summary

Model	R	R Square	Adjusted R Square	Std. Error of the Estimate	Change Statistics		
					R Square Change	F Change	df1
1	.077[a]	.006	.002	1.38793	.006	1.401	1
2	.139[b]	.019	.002	1.38739	.013	1.061	3
3	.149[c]	.022	.001	1.38832	.003	.687	1
4	.257[d]	.066	.029	1.36856	.044	2.687	4

Model Summary

Model	Change Statistics	
	df2	Sig. F Change
1	236	.238
2	233	.366
3	232	.408
4	228	.032

a. Predictors: (Constant), Party0

b. Predictors: (Constant), Party0, SES0, GenderCC, RaceCC

c. Predictors: (Constant), Party0, SES0, GenderCC, RaceCC, MRN0

d. Predictors: (Constant), Party0, SES0, GenderCC, RaceCC, MRN0, MRN0xSES0, MRN0xGender, MRN0xParty0, MRN0xRace

ANOVA[a]

Model		Sum of Squares	df	Mean Square	F	Sig.
1	Regression	2.699	1	2.699	1.401	.238[b]
	Residual	454.618	236	1.926		
	Total	457.317	237			
2	Regression	8.827	4	2.207	1.146	.335[c]
	Residual	448.490	233	1.925		
	Total	457.317	237			
3	Regression	10.151	5	2.030	1.053	.387[d]
	Residual	447.166	232	1.927		
	Total	457.317	237			
4	Regression	30.280	9	3.364	1.796	.070[e]
	Residual	427.037	228	1.873		
	Total	457.317	237			

a. Dependent Variable: Resource_Tot

b. Predictors: (Constant), Party0

c. Predictors: (Constant), Party0, SES0, GenderCC, RaceCC

d. Predictors: (Constant), Party0, SES0, GenderCC, RaceCC, MRN0

e. Predictors: (Constant), Party0, SES0, GenderCC, RaceCC, MRN0, MRN0xSES0, MRN0xGender, MRN0xParty0, MRN0xRace

Coefficients[a]

Model		Unstandardized Coefficients B	Unstandardized Coefficients Std. Error	Standardized Coefficients Beta	t	Sig.
1	(Constant)	3.617	.090		40.205	.000
	Party0	-.077	.065	-.077	-1.184	.238
2	(Constant)	3.664	.105		34.971	.000
	Party0	-.036	.070	-.036	-.517	.606
	GenderCC	-.124	.093	-.089	-1.332	.184
	RaceCC	-.098	.108	-.061	-.906	.366
	SES0	-.083	.112	-.048	-.741	.459
3	(Constant)	3.668	.105		34.947	.000
	Party0	-.009	.077	-.009	-.120	.905
	GenderCC	-.095	.099	-.068	-.954	.341
	RaceCC	-.107	.109	-.067	-.980	.328
	SES0	-.073	.112	-.042	-.646	.519
	MRN0	-.105	.127	-.066	-.829	.408
4	(Constant)	3.551	.119		29.784	.000
	Party0	.007	.078	.007	.092	.927
	GenderCC	-.127	.100	-.091	-1.262	.208
	RaceCC	-.112	.109	-.070	-1.028	.305
	SES0	-.083	.112	-.048	-.739	.461
	MRN0	.030	.141	.019	.214	.831
	MRN0xRace	-.285	.122	-.178	-2.331	.021
	MRN0xSES0	.195	.118	.109	1.659	.099
	MRN0xGender	.209	.119	.118	1.758	.080
	MRN0xParty0	.069	.080	.060	.863	.389

Coefficients[a]

Model		Correlations Zero-order	Partial	Part
1	(Constant)			
	Party0	-.077	-.077	-.077
2	(Constant)			
	Party0	-.077	-.034	-.034
	GenderCC	-.102	-.087	-.086
	RaceCC	-.076	-.059	-.059
	SES0	-.056	-.049	-.048
3	(Constant)			
	Party0	-.077	-.008	-.008
	GenderCC	-.102	-.063	-.062
	RaceCC	-.076	-.064	-.064
	SES0	-.056	-.042	-.042
	MRN0	-.107	-.054	-.054
4	(Constant)			
	Party0	-.077	.006	.006
	GenderCC	-.102	-.083	-.081
	RaceCC	-.076	-.068	-.066
	SES0	-.056	-.049	-.047
	MRN0	-.107	.014	.014
	MRN0xRace	-.152	-.153	-.149
	MRN0xSES0	.111	.109	.106
	MRN0xGender	.087	.116	.113
	MRN0xParty0	.031	.057	.055

a. Dependent Variable: Resource_Tot

Excluded Variables[a]

Model		Beta In	t	Sig.	Partial Correlation	Collinearity Statistics Tolerance
1	GenderCC	-.089[b]	-1.334	.183	-.087	.942
	RaceCC	-.059[b]	-.876	.382	-.057	.919
	SES0	-.053[b]	-.821	.413	-.053	.998
	MRN0	-.091[b]	-1.239	.216	-.081	.784
	MRN0xRace	-.142[b]	-2.082	.038	-.135	.888
	MRN0xSES0	.104[b]	1.594	.112	.103	.988
	MRN0xGender	.097[b]	1.490	.138	.097	.986
	MRN0xParty0	.043[b]	.659	.510	.043	.977
2	MRN0	-.066[c]	-.829	.408	-.054	.671
	MRN0xRace	-.149[c]	-2.159	.032	-.140	.867
	MRN0xSES0	.107[c]	1.646	.101	.107	.984
	MRN0xGender	.090[c]	1.370	.172	.090	.968
	MRN0xParty0	.050[c]	.750	.454	.049	.949
3	MRN0xRace	-.148[d]	-1.987	.048	-.130	.753
	MRN0xSES0	.101[d]	1.519	.130	.099	.952
	MRN0xGender	.102[d]	1.526	.128	.100	.942
	MRN0xParty0	.043[d]	.636	.525	.042	.929

a. Dependent Variable: Resource_Tot

b. Predictors in the Model: (Constant), Party0

c. Predictors in the Model: (Constant), Party0, SES0, GenderCC, RaceCC

d. Predictors in the Model: (Constant), Party0, SES0, GenderCC, RaceCC, MRN0

```
REGRESSION
 /MISSING LISTWISE
 /STATISTICS COEFF OUTS R ANOVA CHANGE ZPP
 /CRITERIA=PIN(.05) POUT(.10)
 /NOORIGIN
 /DEPENDENT Psychology_Tot
 /METHOD=ENTER Party0
 /METHOD=ENTER GenderCC RaceCC SES0
 /METHOD=ENTER MRN0
 /METHOD=ENTER MRN0xRace MRN0xSES0 MRN0xGender MRN0xParty0.
```

Regression

Notes

Output Created		15-DEC-2021 13:07:57
Comments		
Input	Data	C:<br>\Users\njs5478\Dropbox\HM and COVID\0. Revise and Resubmit\2. R and R Data\Study 1b\Study1b_Data.sav
	Active Dataset	DataSet1
	Filter	<none>
	Weight	<none>
	Split File	<none>
	N of Rows in Working Data File	241
Missing Value Handling	Definition of Missing	User-defined missing values are treated as missing.
	Cases Used	Statistics are based on cases with no missing values for any variable used.
Syntax		REGRESSION<br>/MISSING LISTWISE<br>/STATISTICS COEFF OUTS R ANOVA CHANGE ZPP<br>/CRITERIA=PIN(.05) POUT(.10)<br>/NOORIGIN<br>/DEPENDENT Psychology_Tot<br>/METHOD=ENTER Party0<br>/METHOD=ENTER GenderCC RaceCC SES0<br>/METHOD=ENTER MRN0<br>/METHOD=ENTER MRN0xRace MRN0xSES0 MRN0xGender MRN0xParty0.
Resources	Processor Time	00:00:00.03
	Elapsed Time	00:00:00.02

Notes

	Memory Required	45472 bytes
	Additional Memory Required for Residual Plots	0 bytes

Variables Entered/Removed[a]

Model	Variables Entered	Variables Removed	Method
1	Party0[b]	.	Enter
2	SES0, GenderCC, RaceCC[b]	.	Enter
3	MRN0[b]	.	Enter
4	MRN0xSES0, MRN0xGender, MRN0xParty0, MRN0xRace[b]	.	Enter

a. Dependent Variable: Psychology_Tot

b. All requested variables entered.

Model Summary

					Change Statistics		
Model	R	R Square	Adjusted R Square	Std. Error of the Estimate	R Square Change	F Change	df1
1	.260[a]	.068	.064	1.44099	.068	17.102	1
2	.291[b]	.085	.069	1.43678	.017	1.462	3
3	.314[c]	.099	.079	1.42904	.014	3.532	1
4	.326[d]	.106	.071	1.43546	.008	.482	4

Model Summary

	Change Statistics	
Model	df2	Sig. F Change
1	236	.000
2	233	.226
3	232	.061
4	228	.749

a. Predictors: (Constant), Party0

b. Predictors: (Constant), Party0, SES0, GenderCC, RaceCC

c. Predictors: (Constant), Party0, SES0, GenderCC, RaceCC, MRN0

d. Predictors: (Constant), Party0, SES0, GenderCC, RaceCC, MRN0, MRN0xSES0, MRN0xGender, MRN0xParty0, MRN0xRace

ANOVA[a]

Model		Sum of Squares	df	Mean Square	F	Sig.
1	Regression	35.511	1	35.511	17.102	.000[b]
	Residual	490.043	236	2.076		
	Total	525.554	237			
2	Regression	44.564	4	11.141	5.397	.000[c]
	Residual	480.989	233	2.064		
	Total	525.554	237			
3	Regression	51.777	5	10.355	5.071	.000[d]
	Residual	473.777	232	2.042		
	Total	525.554	237			
4	Regression	55.751	9	6.195	3.006	.002[e]
	Residual	469.803	228	2.061		
	Total	525.554	237			

a. Dependent Variable: Psychology_Tot

b. Predictors: (Constant), Party0

c. Predictors: (Constant), Party0, SES0, GenderCC, RaceCC

d. Predictors: (Constant), Party0, SES0, GenderCC, RaceCC, MRN0

e. Predictors: (Constant), Party0, SES0, GenderCC, RaceCC, MRN0, MRN0xSES0, MRN0xGender, MRN0xParty0, MRN0xRace

Coefficients[a]

Model		Unstandardized Coefficients B	Std. Error	Standardized Coefficients Beta	t	Sig.
1	(Constant)	4.662	.093		49.911	.000
	Party0	-.278	.067	-.260	-4.135	.000
2	(Constant)	4.685	.109		43.179	.000
	Party0	-.238	.072	-.222	-3.296	.001
	GenderCC	-.194	.096	-.130	-2.016	.045
	RaceCC	-.048	.112	-.028	-.428	.669
	SES0	.062	.116	.034	.539	.590
3	(Constant)	4.695	.108		43.453	.000
	Party0	-.175	.079	-.164	-2.216	.028
	GenderCC	-.126	.102	-.085	-1.236	.218
	RaceCC	-.068	.112	-.040	-.606	.545
	SES0	.086	.116	.047	.744	.457
	MRN0	-.246	.131	-.143	-1.879	.061
4	(Constant)	4.648	.125		37.165	.000
	Party0	-.171	.081	-.159	-2.095	.037
	GenderCC	-.138	.105	-.093	-1.309	.192
	RaceCC	-.081	.115	-.047	-.708	.479
	SES0	.087	.117	.047	.744	.458
	MRN0	-.187	.148	-.109	-1.264	.207
	MRN0xRace	-.148	.128	-.086	-1.154	.250
	MRN0xSES0	-.014	.123	-.007	-.112	.911
	MRN0xGender	.077	.125	.040	.614	.540
	MRN0xParty0	.058	.083	.047	.694	.488

Coefficients[a]

Model		Correlations		
		Zero-order	Partial	Part
1	(Constant)			
	Party0	-.260	-.260	-.260
2	(Constant)			
	Party0	-.260	-.211	-.207
	GenderCC	-.183	-.131	-.126
	RaceCC	-.094	-.028	-.027
	SES0	.017	.035	.034
3	(Constant)			
	Party0	-.260	-.144	-.138
	GenderCC	-.183	-.081	-.077
	RaceCC	-.094	-.040	-.038
	SES0	.017	.049	.046
	MRN0	-.251	-.122	-.117
4	(Constant)			
	Party0	-.260	-.137	-.131
	GenderCC	-.183	-.086	-.082
	RaceCC	-.094	-.047	-.044
	SES0	.017	.049	.047
	MRN0	-.251	-.083	-.079
	MRN0xRace	-.183	-.076	-.072
	MRN0xSES0	.035	-.007	-.007
	MRN0xGender	-.002	.041	.038
	MRN0xParty0	-.006	.046	.043

a. Dependent Variable: Psychology_Tot

Excluded Variables[a]

Model		Beta In	t	Sig.	Partial Correlation	Collinearity Statistics Tolerance
1	GenderCC	-.128[b]	-1.987	.048	-.129	.942
	RaceCC	-.022[b]	-.339	.735	-.022	.919
	SES0	.028[b]	.441	.660	.029	.998
	MRN0	-.166[b]	-2.360	.019	-.152	.784
	MRN0xRace	-.108[b]	-1.620	.107	-.105	.888
	MRN0xSES0	.006[b]	.090	.928	.006	.988
	MRN0xGender	.029[b]	.454	.650	.030	.986
	MRN0xParty0	.034[b]	.527	.599	.034	.977
2	MRN0	-.143[c]	-1.879	.061	-.122	.671
	MRN0xRace	-.105[c]	-1.566	.119	-.102	.867
	MRN0xSES0	.012[c]	.194	.847	.013	.984
	MRN0xGender	.020[c]	.319	.750	.021	.968
	MRN0xParty0	.051[c]	.792	.429	.052	.949
3	MRN0xRace	-.068[d]	-.951	.342	-.062	.753
	MRN0xSES0	-.009[d]	-.147	.884	-.010	.952
	MRN0xGender	.041[d]	.635	.526	.042	.942
	MRN0xParty0	.034[d]	.530	.596	.035	.929

a. Dependent Variable: Psychology_Tot

b. Predictors in the Model: (Constant), Party0

c. Predictors in the Model: (Constant), Party0, SES0, GenderCC, RaceCC

d. Predictors in the Model: (Constant), Party0, SES0, GenderCC, RaceCC, MRN0

```
REGRESSION
 /MISSING LISTWISE
 /STATISTICS COEFF OUTS R ANOVA CHANGE ZPP
 /CRITERIA=PIN(.05) POUT(.10)
 /NOORIGIN
 /DEPENDENT TrumpApproval
 /METHOD=ENTER Party0
 /METHOD=ENTER GenderCC RaceCC SES0
 /METHOD=ENTER MRN0
 /METHOD=ENTER MRN0xRace MRN0xSES0 MRN0xGender MRN0xParty0.
```

Regression

Notes

Output Created		15-DEC-2021 13:07:57
Comments		
Input	Data	C: \Users\njs5478\Dropbox\HM and COVID\0. Revise and Resubmit\2. R and R Data\Study 1b\Study1b_Data.sav
	Active Dataset	DataSet1
	Filter	<none>
	Weight	<none>
	Split File	<none>
	N of Rows in Working Data File	241
Missing Value Handling	Definition of Missing	User-defined missing values are treated as missing.
	Cases Used	Statistics are based on cases with no missing values for any variable used.
Syntax		REGRESSION /MISSING LISTWISE /STATISTICS COEFF OUTS R ANOVA CHANGE ZPP /CRITERIA=PIN(.05) POUT(.10) /NOORIGIN /DEPENDENT TrumpApproval /METHOD=ENTER Party0 /METHOD=ENTER GenderCC RaceCC SES0 /METHOD=ENTER MRN0 /METHOD=ENTER MRN0xRace MRN0xSES0 MRN0xGender MRN0xParty0.
Resources	Processor Time	00:00:00.02
	Elapsed Time	00:00:00.02

Notes

	Memory Required	45472 bytes
	Additional Memory Required for Residual Plots	0 bytes

Variables Entered/Removed[a]

Model	Variables Entered	Variables Removed	Method
1	Party0[b]	.	Enter
2	SES0, GenderCC, RaceCC[b]	.	Enter
3	MRN0[b]	.	Enter
4	MRN0xSES0, MRN0xGender, MRN0xParty0, MRN0xRace[b]	.	Enter

a. Dependent Variable: Do you approve or disapprove of the way Donald Trump is handling his job as President?

b. All requested variables entered.

Model Summary

					Change Statistics		
Model	R	R Square	Adjusted R Square	Std. Error of the Estimate	R Square Change	F Change	df1
1	.781[a]	.609	.608	1.311	.609	366.392	1
2	.793[b]	.629	.622	1.286	.019	4.051	3
3	.809[c]	.654	.646	1.244	.025	16.681	1
4	.812[d]	.660	.646	1.245	.006	.990	4

Model Summary

Model	Change Statistics df2	Sig. F Change
1	235	.000
2	232	.008
3	231	.000
4	227	.414

a. Predictors: (Constant), Party0

b. Predictors: (Constant), Party0, SES0, GenderCC, RaceCC

c. Predictors: (Constant), Party0, SES0, GenderCC, RaceCC, MRN0

d. Predictors: (Constant), Party0, SES0, GenderCC, RaceCC, MRN0, MRN0xSES0, MRN0xGender, MRN0xParty0, MRN0xRace

ANOVA[a]

Model		Sum of Squares	df	Mean Square	F	Sig.
1	Regression	629.312	1	629.312	366.392	.000[b]
	Residual	403.634	235	1.718		
	Total	1032.945	236			
2	Regression	649.402	4	162.350	98.203	.000[c]
	Residual	383.544	232	1.653		
	Total	1032.945	236			
3	Regression	675.233	5	135.047	87.209	.000[d]
	Residual	357.712	231	1.549		
	Total	1032.945	236			
4	Regression	681.366	9	75.707	48.881	.000[e]
	Residual	351.579	227	1.549		
	Total	1032.945	236			

a. Dependent Variable: Do you approve or disapprove of the way Donald Trump is handling his job as President?

b. Predictors: (Constant), Party0

c. Predictors: (Constant), Party0, SES0, GenderCC, RaceCC

d. Predictors: (Constant), Party0, SES0, GenderCC, RaceCC, MRN0

e. Predictors: (Constant), Party0, SES0, GenderCC, RaceCC, MRN0, MRN0xSES0, MRN0xGender, MRN0xParty0, MRN0xRace

Coefficients[a]

Model		Unstandardized Coefficients B	Std. Error	Standardized Coefficients Beta	t	Sig.
1	(Constant)	3.140	.085		36.889	.000
	Party0	1.172	.061	.781	19.141	.000
2	(Constant)	3.002	.097		30.889	.000
	Party0	1.119	.065	.745	17.335	.000
	GenderCC	.051	.086	.024	.591	.555
	RaceCC	.277	.100	.115	2.758	.006
	SES0	-.224	.104	-.087	-2.168	.031
3	(Constant)	2.984	.094		31.702	.000
	Party0	1.001	.069	.667	14.537	.000
	GenderCC	-.079	.089	-.038	-.882	.379
	RaceCC	.315	.097	.131	3.231	.001
	SES0	-.270	.101	-.105	-2.682	.008
	MRN0	.466	.114	.193	4.084	.000
4	(Constant)	2.928	.109		26.946	.000
	Party0	.971	.071	.647	13.766	.000
	GenderCC	-.067	.092	-.032	-.736	.462
	RaceCC	.330	.099	.138	3.319	.001
	SES0	-.255	.102	-.099	-2.505	.013
	MRN0	.393	.128	.163	3.056	.003
	MRN0xRace	.160	.111	.066	1.437	.152
	MRN0xSES0	-.044	.107	-.016	-.413	.680
	MRN0xGender	.056	.108	.021	.517	.606
	MRN0xParty0	.047	.072	.028	.656	.512

Coefficients[a]

Model		Correlations Zero-order	Partial	Part
1	(Constant)			
	Party0	.781	.781	.781
2	(Constant)			
	Party0	.781	.751	.693
	GenderCC	.204	.039	.024
	RaceCC	.325	.178	.110
	SES0	-.051	-.141	-.087
3	(Constant)			
	Party0	.781	.691	.563
	GenderCC	.204	-.058	-.034
	RaceCC	.325	.208	.125
	SES0	-.051	-.174	-.104
	MRN0	.481	.260	.158
4	(Constant)			
	Party0	.781	.675	.533
	GenderCC	.204	-.049	-.029
	RaceCC	.325	.215	.129
	SES0	-.051	-.164	-.097
	MRN0	.481	.199	.118
	MRN0xRace	.360	.095	.056
	MRN0xSES0	-.133	-.027	-.016
	MRN0xGender	.133	.034	.020
	MRN0xParty0	.160	.044	.025

a. Dependent Variable: Do you approve or disapprove of the way Donald Trump is handling his job as President?

Excluded Variables[a]

Model		Beta In	t	Sig.	Partial Correlation	Collinearity Statistics Tolerance
1	GenderCC	.017[b]	.400	.690	.026	.942
	RaceCC	.112[b]	2.667	.008	.172	.919
	SES0	-.083[b]	-2.049	.042	-.133	.998
	MRN0	.151[b]	3.344	.001	.214	.784
	MRN0xRace	.111[b]	2.599	.010	.168	.888
	MRN0xSES0	-.047[b]	-1.153	.250	-.075	.988
	MRN0xGender	.041[b]	.998	.319	.065	.986
	MRN0xParty0	.043[b]	1.033	.303	.067	.977
2	MRN0	.193[c]	4.084	.000	.260	.669
	MRN0xRace	.128[c]	3.031	.003	.196	.867
	MRN0xSES0	-.046[c]	-1.138	.256	-.075	.984
	MRN0xGender	.060[c]	1.469	.143	.096	.968
	MRN0xParty0	.021[c]	.520	.604	.034	.948
3	MRN0xRace	.077[d]	1.724	.086	.113	.754
	MRN0xSES0	-.018[d]	-.447	.655	-.029	.952
	MRN0xGender	.033[d]	.838	.403	.055	.940
	MRN0xParty0	.046[d]	1.135	.258	.075	.929

a. Dependent Variable: Do you approve or disapprove of the way Donald Trump is handling his job as President?

b. Predictors in the Model: (Constant), Party0

c. Predictors in the Model: (Constant), Party0, SES0, GenderCC, RaceCC

d. Predictors in the Model: (Constant), Party0, SES0, GenderCC, RaceCC, MRN0

```
REGRESSION
 /MISSING LISTWISE
 /STATISTICS COEFF OUTS R ANOVA CHANGE ZPP
 /CRITERIA=PIN(.05) POUT(.10)
 /NOORIGIN
 /DEPENDENT TrumpX
 /METHOD=ENTER Party0
 /METHOD=ENTER GenderCC RaceCC SES0
 /METHOD=ENTER MRN0
```

```
/METHOD=ENTER MRN0xRace MRN0xSES0 MRN0xGender MRN0xParty0.
```

Regression

Notes

Output Created		15-DEC-2021 13:07:57
Comments		
Input	Data	C: \Users\njs5478\Dropbox\HM and COVID\0. Revise and Resubmit\2. R and R Data\Study 1b\Study1b_Data.sav
	Active Dataset	DataSet1
	Filter	<none>
	Weight	<none>
	Split File	<none>
	N of Rows in Working Data File	241
Missing Value Handling	Definition of Missing	User-defined missing values are treated as missing.
	Cases Used	Statistics are based on cases with no missing values for any variable used.
Syntax		REGRESSION<br>/MISSING LISTWISE<br>/STATISTICS COEFF OUTS R ANOVA CHANGE ZPP<br>/CRITERIA=PIN(.05) POUT(.10)<br>/NOORIGIN<br>/DEPENDENT TrumpX<br>/METHOD=ENTER Party0<br>/METHOD=ENTER GenderCC RaceCC SES0<br>/METHOD=ENTER MRN0<br>/METHOD=ENTER MRN0xRace MRN0xSES0 MRN0xGender MRN0xParty0.
Resources	Processor Time	00:00:00.02
	Elapsed Time	00:00:00.06

Notes

	Memory Required	45472 bytes
	Additional Memory Required for Residual Plots	0 bytes

Variables Entered/Removed[a]

Model	Variables Entered	Variables Removed	Method
1	Party0[b]	.	Enter
2	SES0, GenderCC, RaceCC[b]	.	Enter
3	MRN0[b]	.	Enter
4	MRN0xSES0, MRN0xParty0 , MRN0xGende r, ...[b]	.	Enter

a. Dependent Variable: TrumpX

b. All requested variables entered.

Model Summary

Model	R	R Square	Adjusted R Square	Std. Error of the Estimate	Change Statistics		
					R Square Change	F Change	df1
1	.778[a]	.606	.604	1.29104	.606	357.750	1
2	.785[b]	.617	.610	1.28124	.011	2.193	3
3	.800[c]	.640	.632	1.24399	.024	14.979	1
4	.805[d]	.648	.633	1.24189	.007	1.193	4

Model Summary

Model	Change Statistics	
	df2	Sig. F Change
1	233	.000
2	230	.090
3	229	.000
4	225	.315

a. Predictors: (Constant), Party0

b. Predictors: (Constant), Party0, SES0, GenderCC, RaceCC

c. Predictors: (Constant), Party0, SES0, GenderCC, RaceCC, MRN0

d. Predictors: (Constant), Party0, SES0, GenderCC, RaceCC, MRN0, MRN0xSES0, MRN0xParty0, MRN0xGender, MRN0xRace

ANOVA[a]

Model		Sum of Squares	df	Mean Square	F	Sig.
1	Regression	596.294	1	596.294	357.750	.000[b]
	Residual	388.362	233	1.667		
	Total	984.655	234			
2	Regression	607.095	4	151.774	92.457	.000[c]
	Residual	377.560	230	1.642		
	Total	984.655	234			
3	Regression	630.275	5	126.055	81.457	.000[d]
	Residual	354.380	229	1.548		
	Total	984.655	234			
4	Regression	637.637	9	70.849	45.937	.000[e]
	Residual	347.018	225	1.542		
	Total	984.655	234			

a. Dependent Variable: TrumpX

b. Predictors: (Constant), Party0

c. Predictors: (Constant), Party0, SES0, GenderCC, RaceCC

d. Predictors: (Constant), Party0, SES0, GenderCC, RaceCC, MRN0

e. Predictors: (Constant), Party0, SES0, GenderCC, RaceCC, MRN0, MRN0xSES0, MRN0xParty0, MRN0xGender, MRN0xRace

Coefficients[a]

Model		Unstandardized Coefficients B	Std. Error	Standardized Coefficients Beta	t	Sig.
1	(Constant)	3.056	.084		36.288	.000
	Party0	1.143	.060	.778	18.914	.000
2	(Constant)	2.960	.097		30.507	.000
	Party0	1.104	.065	.751	17.101	.000
	GenderCC	.056	.086	.027	.648	.518
	RaceCC	.192	.100	.082	1.920	.056
	SES0	-.173	.103	-.068	-1.670	.096
3	(Constant)	2.945	.094		31.234	.000
	Party0	.993	.069	.676	14.407	.000
	GenderCC	-.069	.090	-.034	-.767	.444
	RaceCC	.229	.098	.098	2.347	.020
	SES0	-.217	.101	-.086	-2.150	.033
	MRN0	.443	.114	.188	3.870	.000
4	(Constant)	2.843	.109		26.109	.000
	Party0	.966	.070	.657	13.700	.000
	GenderCC	-.056	.092	-.027	-.606	.545
	RaceCC	.255	.099	.108	2.564	.011
	SES0	-.208	.102	-.083	-2.051	.041
	MRN0	.380	.129	.161	2.957	.003
	MRN0xRace	.117	.111	.049	1.049	.295
	MRN0xSES0	.040	.107	.015	.375	.708
	MRN0xGender	.163	.109	.063	1.495	.136
	MRN0xParty0	.037	.072	.022	.510	.610

Coefficients[a]

Model		Correlations Zero-order	Partial	Part
1	(Constant)			
	Party0	.778	.778	.778
2	(Constant)			
	Party0	.778	.748	.698
	GenderCC	.214	.043	.026
	RaceCC	.292	.126	.078
	SES0	-.032	-.109	-.068
3	(Constant)			
	Party0	.778	.690	.571
	GenderCC	.214	-.051	-.030
	RaceCC	.292	.153	.093
	SES0	-.032	-.141	-.085
	MRN0	.480	.248	.153
4	(Constant)			
	Party0	.778	.674	.542
	GenderCC	.214	-.040	-.024
	RaceCC	.292	.168	.101
	SES0	-.032	-.135	-.081
	MRN0	.480	.193	.117
	MRN0xRace	.350	.070	.042
	MRN0xSES0	-.103	.025	.015
	MRN0xGender	.177	.099	.059
	MRN0xParty0	.153	.034	.020

a. Dependent Variable: TrumpX

Excluded Variables[a]

Model		Beta In	t	Sig.	Partial Correlation	Collinearity Statistics Tolerance
1	GenderCC	.022[b]	.516	.606	.034	.938
	RaceCC	.079[b]	1.854	.065	.121	.920
	SES0	-.065[b]	-1.590	.113	-.104	.998
	MRN0	.153[b]	3.357	.001	.215	.786
	MRN0xRace	.102[b]	2.366	.019	.154	.889
	MRN0xSES0	-.018[b]	-.445	.657	-.029	.988
	MRN0xGender	.080[b]	1.948	.053	.127	.984
	MRN0xParty0	.037[b]	.884	.377	.058	.977
2	MRN0	.188[c]	3.870	.000	.248	.668
	MRN0xRace	.114[c]	2.639	.009	.172	.868
	MRN0xSES0	-.018[c]	-.436	.663	-.029	.985
	MRN0xGender	.095[c]	2.310	.022	.151	.966
	MRN0xParty0	.020[c]	.487	.627	.032	.949
3	MRN0xRace	.063[d]	1.385	.167	.091	.756
	MRN0xSES0	.010[d]	.247	.805	.016	.953
	MRN0xGender	.070[d]	1.707	.089	.112	.936
	MRN0xParty0	.044[d]	1.073	.284	.071	.929

a. Dependent Variable: TrumpX

b. Predictors in the Model: (Constant), Party0

c. Predictors in the Model: (Constant), Party0, SES0, GenderCC, RaceCC

d. Predictors in the Model: (Constant), Party0, SES0, GenderCC, RaceCC, MRN0

```
REGRESSION
 /MISSING LISTWISE
 /STATISTICS COEFF OUTS R ANOVA CHANGE ZPP
 /CRITERIA=PIN(.05) POUT(.10)
 /NOORIGIN
 /DEPENDENT BidenX
 /METHOD=ENTER Party0
 /METHOD=ENTER GenderCC RaceCC SES0
 /METHOD=ENTER MRN0
 /METHOD=ENTER MRN0xRace MRN0xSES0 MRN0xGender MRN0xParty0.
```

Regression

Notes

Output Created		15-DEC-2021 13:07:57
Comments		
Input	Data	C:<br>\Users\njs5478\Dropbox\HM and COVID\0. Revise and Resubmit\2. R and R Data\Study 1b\Study1b_Data.sav
	Active Dataset	DataSet1
	Filter	<none>
	Weight	<none>
	Split File	<none>
	N of Rows in Working Data File	241
Missing Value Handling	Definition of Missing	User-defined missing values are treated as missing.
	Cases Used	Statistics are based on cases with no missing values for any variable used.
Syntax		REGRESSION<br>/MISSING LISTWISE<br>/STATISTICS COEFF OUTS R ANOVA CHANGE ZPP<br>/CRITERIA=PIN(.05) POUT(.10)<br>/NOORIGIN<br>/DEPENDENT BidenX<br>/METHOD=ENTER Party0<br>/METHOD=ENTER GenderCC RaceCC SES0<br>/METHOD=ENTER MRN0<br>/METHOD=ENTER MRN0xRace MRN0xSES0 MRN0xGender MRN0xParty0.
Resources	Processor Time	00:00:00.03
	Elapsed Time	00:00:00.03

Notes

	Memory Required	45472 bytes
	Additional Memory Required for Residual Plots	0 bytes

Variables Entered/Removed[a]

Model	Variables Entered	Variables Removed	Method
1	Party0[b]	.	Enter
2	SES0, GenderCC, RaceCC[b]	.	Enter
3	MRN0[b]	.	Enter
4	MRN0xSES0, MRN0xParty0 , MRN0xGender, ...[b]	.	Enter

a. Dependent Variable: BidenX

b. All requested variables entered.

Model Summary

Model	R	R Square	Adjusted R Square	Std. Error of the Estimate	Change Statistics		
					R Square Change	F Change	df1
1	.703[a]	.494	.491	1.27810	.494	222.250	1
2	.706[b]	.498	.490	1.28039	.005	.728	3
3	.715[c]	.511	.500	1.26749	.012	5.605	1
4	.717[d]	.515	.495	1.27364	.004	.460	4

Model Summary

Model	Change Statistics	
	df2	Sig. F Change
1	228	.000
2	225	.536
3	224	.019
4	220	.765

a. Predictors: (Constant), Party0

b. Predictors: (Constant), Party0, SES0, GenderCC, RaceCC

c. Predictors: (Constant), Party0, SES0, GenderCC, RaceCC, MRN0

d. Predictors: (Constant), Party0, SES0, GenderCC, RaceCC, MRN0, MRN0xSES0, MRN0xParty0, MRN0xGender, MRN0xRace

ANOVA[a]

Model		Sum of Squares	df	Mean Square	F	Sig.
1	Regression	363.051	1	363.051	222.250	.000[b]
	Residual	372.444	228	1.634		
	Total	735.496	229			
2	Regression	366.631	4	91.658	55.909	.000[c]
	Residual	368.865	225	1.639		
	Total	735.496	229			
3	Regression	375.635	5	75.127	46.764	.000[d]
	Residual	359.860	224	1.607		
	Total	735.496	229			
4	Regression	378.622	9	42.069	25.934	.000[e]
	Residual	356.874	220	1.622		
	Total	735.496	229			

a. Dependent Variable: BidenX

b. Predictors: (Constant), Party0

c. Predictors: (Constant), Party0, SES0, GenderCC, RaceCC

d. Predictors: (Constant), Party0, SES0, GenderCC, RaceCC, MRN0

e. Predictors: (Constant), Party0, SES0, GenderCC, RaceCC, MRN0, MRN0xSES0, MRN0xParty0, MRN0xGender, MRN0xRace

Coefficients[a]

Model		Unstandardized Coefficients B	Unstandardized Coefficients Std. Error	Standardized Coefficients Beta	t	Sig.
1	(Constant)	4.475	.084		53.081	.000
	Party0	-.898	.060	-.703	-14.908	.000
2	(Constant)	4.472	.098		45.611	.000
	Party0	-.895	.065	-.700	-13.744	.000
	GenderCC	-.048	.087	-.027	-.546	.586
	RaceCC	.010	.101	.005	.098	.922
	SES0	.144	.104	.065	1.375	.170
3	(Constant)	4.482	.097		46.134	.000
	Party0	-.826	.071	-.646	-11.662	.000
	GenderCC	.030	.093	.017	.322	.748
	RaceCC	-.012	.100	-.006	-.123	.902
	SES0	.171	.104	.077	1.642	.102
	MRN0	-.277	.117	-.135	-2.367	.019
4	(Constant)	4.523	.113		40.132	.000
	Party0	-.817	.073	-.639	-11.183	.000
	GenderCC	.008	.096	.004	.080	.937
	RaceCC	-.038	.103	-.019	-.373	.710
	SES0	.176	.105	.080	1.675	.095
	MRN0	-.215	.133	-.105	-1.622	.106
	MRN0xRace	-.076	.115	-.037	-.662	.509
	MRN0xSES0	-.024	.110	-.011	-.220	.826
	MRN0xGender	-.125	.113	-.055	-1.107	.270
	MRN0xParty0	.041	.074	.028	.554	.580

Coefficients[a]

Model		Correlations Zero-order	Partial	Part
1	(Constant)			
	Party0	-.703	-.703	-.703
2	(Constant)			
	Party0	-.703	-.676	-.649
	GenderCC	-.207	-.036	-.026
	RaceCC	-.191	.007	.005
	SES0	.033	.091	.065
3	(Constant)			
	Party0	-.703	-.615	-.545
	GenderCC	-.207	.022	.015
	RaceCC	-.191	-.008	-.006
	SES0	.033	.109	.077
	MRN0	-.421	-.156	-.111
4	(Constant)			
	Party0	-.703	-.602	-.525
	GenderCC	-.207	.005	.004
	RaceCC	-.191	-.025	-.018
	SES0	.033	.112	.079
	MRN0	-.421	-.109	-.076
	MRN0xRace	-.304	-.045	-.031
	MRN0xSES0	.091	-.015	-.010
	MRN0xGender	-.160	-.074	-.052
	MRN0xParty0	-.092	.037	.026

a. Dependent Variable: BidenX

Excluded Variables[a]

Model		Beta In	t	Sig.	Partial Correlation	Collinearity Statistics Tolerance
1	GenderCC	-.025[b]	-.517	.605	-.034	.932
	RaceCC	.008[b]	.167	.867	.011	.920
	SES0	.065[b]	1.374	.171	.091	.998
	MRN0	-.119[b]	-2.248	.026	-.148	.783
	MRN0xRace	-.075[b]	-1.512	.132	-.100	.886
	MRN0xSES0	.013[b]	.269	.788	.018	.988
	MRN0xGender	-.068[b]	-1.438	.152	-.095	.982
	MRN0xParty0	.016[b]	.340	.734	.023	.976
2	MRN0	-.135[c]	-2.367	.019	-.156	.671
	MRN0xRace	-.075[c]	-1.475	.142	-.098	.863
	MRN0xSES0	.015[c]	.320	.750	.021	.985
	MRN0xGender	-.072[c]	-1.503	.134	-.100	.964
	MRN0xParty0	.027[c]	.550	.583	.037	.948
3	MRN0xRace	-.037[d]	-.679	.498	-.045	.751
	MRN0xSES0	-.005[d]	-.103	.918	-.007	.953
	MRN0xGender	-.053[d]	-1.096	.274	-.073	.931
	MRN0xParty0	.010[d]	.204	.839	.014	.927

a. Dependent Variable: BidenX

b. Predictors in the Model: (Constant), Party0

c. Predictors in the Model: (Constant), Party0, SES0, GenderCC, RaceCC

d. Predictors in the Model: (Constant), Party0, SES0, GenderCC, RaceCC, MRN0

```
REGRESSION
 /MISSING LISTWISE
 /STATISTICS COEFF OUTS R ANOVA CHANGE ZPP
 /CRITERIA=PIN(.05) POUT(.10)
 /NOORIGIN
 /DEPENDENT PelosiX
 /METHOD=ENTER Party0
 /METHOD=ENTER GenderCC RaceCC SES0
 /METHOD=ENTER MRN0
 /METHOD=ENTER MRN0xRace MRN0xSES0 MRN0xGender MRN0xParty0.
```

Regression

Notes

Output Created		15-DEC-2021 13:07:57
Comments		
Input	Data	C:\Users\njs5478\Dropbox\HM and COVID\0. Revise and Resubmit\2. R and R Data\Study 1b\Study1b_Data.sav
	Active Dataset	DataSet1
	Filter	<none>
	Weight	<none>
	Split File	<none>
	N of Rows in Working Data File	241
Missing Value Handling	Definition of Missing	User-defined missing values are treated as missing.
	Cases Used	Statistics are based on cases with no missing values for any variable used.
Syntax		REGRESSION /MISSING LISTWISE /STATISTICS COEFF OUTS R ANOVA CHANGE ZPP /CRITERIA=PIN(.05) POUT(.10) /NOORIGIN /DEPENDENT PelosiX /METHOD=ENTER Party0 /METHOD=ENTER GenderCC RaceCC SES0 /METHOD=ENTER MRN0 /METHOD=ENTER MRN0xRace MRN0xSES0 MRN0xGender MRN0xParty0.
Resources	Processor Time	00:00:00.02
	Elapsed Time	00:00:00.17

Notes

	Memory Required	45472 bytes
	Additional Memory Required for Residual Plots	0 bytes

Variables Entered/Removed[a]

Model	Variables Entered	Variables Removed	Method
1	Party0[b]	.	Enter
2	SES0, GenderCC, RaceCC[b]	.	Enter
3	MRN0[b]	.	Enter
4	MRN0xSES0, MRN0xParty0 , MRN0xGende r, ...[b]	.	Enter

a. Dependent Variable: PelosiX

b. All requested variables entered.

Model Summary

Model	R	R Square	Adjusted R Square	Std. Error of the Estimate	Change Statistics		
					R Square Change	F Change	df1
1	.620[a]	.384	.381	1.28365	.384	123.503	1
2	.635[b]	.403	.391	1.27310	.019	2.098	3
3	.651[c]	.423	.409	1.25477	.020	6.738	1
4	.654[d]	.428	.401	1.26277	.005	.388	4

Model Summary

Model	Change Statistics	
	df2	Sig. F Change
1	198	.000
2	195	.102
3	194	.010
4	190	.817

a. Predictors: (Constant), Party0

b. Predictors: (Constant), Party0, SES0, GenderCC, RaceCC

c. Predictors: (Constant), Party0, SES0, GenderCC, RaceCC, MRN0

d. Predictors: (Constant), Party0, SES0, GenderCC, RaceCC, MRN0, MRN0xSES0, MRN0xParty0, MRN0xGender, MRN0xRace

ANOVA[a]

Model		Sum of Squares	df	Mean Square	F	Sig.
1	Regression	203.501	1	203.501	123.503	.000[b]
	Residual	326.254	198	1.648		
	Total	529.755	199			
2	Regression	213.702	4	53.426	32.963	.000[c]
	Residual	316.053	195	1.621		
	Total	529.755	199			
3	Regression	224.310	5	44.862	28.494	.000[d]
	Residual	305.445	194	1.574		
	Total	529.755	199			
4	Regression	226.784	9	25.198	15.802	.000[e]
	Residual	302.971	190	1.595		
	Total	529.755	199			

a. Dependent Variable: PelosiX

b. Predictors: (Constant), Party0

c. Predictors: (Constant), Party0, SES0, GenderCC, RaceCC

d. Predictors: (Constant), Party0, SES0, GenderCC, RaceCC, MRN0

e. Predictors: (Constant), Party0, SES0, GenderCC, RaceCC, MRN0, MRN0xSES0, MRN0xParty0, MRN0xGender, MRN0xRace

Coefficients[a]

Model		Unstandardized Coefficients B	Std. Error	Standardized Coefficients Beta	t	Sig.
1	(Constant)	3.575	.091		39.355	.000
	Party0	-.726	.065	-.620	-11.113	.000
2	(Constant)	3.527	.106		33.284	.000
	Party0	-.703	.069	-.600	-10.172	.000
	GenderCC	-.197	.093	-.121	-2.117	.036
	RaceCC	.099	.109	.052	.909	.364
	SES0	-.104	.112	-.051	-.927	.355
3	(Constant)	3.544	.105		33.867	.000
	Party0	-.619	.075	-.528	-8.207	.000
	GenderCC	-.103	.098	-.063	-1.049	.295
	RaceCC	.070	.108	.037	.644	.520
	SES0	-.080	.110	-.040	-.725	.469
	MRN0	-.332	.128	-.174	-2.596	.010
4	(Constant)	3.583	.121		29.696	.000
	Party0	-.609	.078	-.519	-7.830	.000
	GenderCC	-.122	.102	-.075	-1.197	.233
	RaceCC	.057	.110	.030	.515	.607
	SES0	-.071	.112	-.035	-.634	.527
	MRN0	-.285	.147	-.149	-1.934	.055
	MRN0xRace	-.032	.130	-.017	-.244	.807
	MRN0xSES0	.062	.125	.028	.497	.620
	MRN0xGender	-.122	.122	-.058	-.996	.320
	MRN0xParty0	.014	.080	.010	.178	.859

Coefficients[a]

Model		Correlations		
		Zero-order	Partial	Part
1	(Constant)			
	Party0	-.620	-.620	-.620
2	(Constant)			
	Party0	-.620	-.589	-.563
	GenderCC	-.268	-.150	-.117
	RaceCC	-.110	.065	.050
	SES0	-.093	-.066	-.051
3	(Constant)			
	Party0	-.620	-.508	-.447
	GenderCC	-.268	-.075	-.057
	RaceCC	-.110	.046	.035
	SES0	-.093	-.052	-.040
	MRN0	-.455	-.183	-.142
4	(Constant)			
	Party0	-.620	-.494	-.430
	GenderCC	-.268	-.087	-.066
	RaceCC	-.110	.037	.028
	SES0	-.093	-.046	-.035
	MRN0	-.455	-.139	-.106
	MRN0xRace	-.319	-.018	-.013
	MRN0xSES0	.107	.036	.027
	MRN0xGender	-.187	-.072	-.055
	MRN0xParty0	-.094	.013	.010

a. Dependent Variable: PelosiX

Excluded Variables[a]

Model		Beta In	t	Sig.	Partial Correlation	Collinearity Statistics Tolerance
1	GenderCC	-.123[b]	-2.153	.033	-.152	.940
	RaceCC	.052[b]	.901	.369	.064	.934
	SES0	-.054[b]	-.974	.331	-.069	.996
	MRN0	-.208[b]	-3.361	.001	-.233	.775
	MRN0xRace	-.109[b]	-1.836	.068	-.130	.870
	MRN0xSES0	.044[b]	.786	.433	.056	.989
	MRN0xGender	-.089[b]	-1.572	.118	-.111	.973
	MRN0xParty0	.014[b]	.243	.809	.017	.970
2	MRN0	-.174[c]	-2.596	.010	-.183	.660
	MRN0xRace	-.089[c]	-1.487	.139	-.106	.849
	MRN0xSES0	.052[c]	.928	.354	.067	.979
	MRN0xGender	-.084[c]	-1.493	.137	-.107	.962
	MRN0xParty0	.017[c]	.300	.765	.021	.933
3	MRN0xRace	-.026[d]	-.390	.697	-.028	.681
	MRN0xSES0	.037[d]	.676	.500	.049	.969
	MRN0xGender	-.062[d]	-1.102	.272	-.079	.937
	MRN0xParty0	.000[d]	.001	.999	.000	.920

a. Dependent Variable: PelosiX

b. Predictors in the Model: (Constant), Party0

c. Predictors in the Model: (Constant), Party0, SES0, GenderCC, RaceCC

d. Predictors in the Model: (Constant), Party0, SES0, GenderCC, RaceCC, MRN0

```
REGRESSION
 /MISSING LISTWISE
 /STATISTICS COEFF OUTS R ANOVA CHANGE ZPP
 /CRITERIA=PIN(.05) POUT(.10)
 /NOORIGIN
 /DEPENDENT McConnellX
 /METHOD=ENTER Party0
 /METHOD=ENTER GenderCC RaceCC SES0
 /METHOD=ENTER MRN0
 /METHOD=ENTER MRN0xRace MRN0xSES0 MRN0xGender MRN0xParty0.
```

Regression

Notes

Output Created		15-DEC-2021 13:07:57
Comments		
Input	Data	C: \Users\njs5478\Dropbox\HM and COVID\0. Revise and Resubmit\2. R and R Data\Study 1b\Study1b_Data.sav
	Active Dataset	DataSet1
	Filter	<none>
	Weight	<none>
	Split File	<none>
	N of Rows in Working Data File	241
Missing Value Handling	Definition of Missing	User-defined missing values are treated as missing.
	Cases Used	Statistics are based on cases with no missing values for any variable used.
Syntax		REGRESSION /MISSING LISTWISE /STATISTICS COEFF OUTS R ANOVA CHANGE ZPP /CRITERIA=PIN(.05) POUT(.10) /NOORIGIN /DEPENDENT McConnellX /METHOD=ENTER Party0 /METHOD=ENTER GenderCC RaceCC SES0 /METHOD=ENTER MRN0 /METHOD=ENTER MRN0xRace MRN0xSES0 MRN0xGender MRN0xParty0.
Resources	Processor Time	00:00:00.05
	Elapsed Time	00:00:00.03

Notes

	Memory Required	45472 bytes
	Additional Memory Required for Residual Plots	0 bytes

Variables Entered/Removed[a]

Model	Variables Entered	Variables Removed	Method
1	Party0[b]	.	Enter
2	SES0, RaceCC, GenderCC[b]	.	Enter
3	MRN0[b]	.	Enter
4	MRN0xSES0, MRN0xParty0 , MRN0xGende r, ...[b]	.	Enter

a. Dependent Variable: McConnellX

b. All requested variables entered.

Model Summary

Model	R	R Square	Adjusted R Square	Std. Error of the Estimate	Change Statistics		
					R Square Change	F Change	df1
1	.561[a]	.315	.312	1.27416	.315	88.362	1
2	.585[b]	.342	.328	1.25867	.027	2.585	3
3	.616[c]	.379	.363	1.22584	.037	11.260	1
4	.639[d]	.408	.379	1.21019	.029	2.223	4

Model Summary

Model	Change Statistics	
	df2	Sig. F Change
1	192	.000
2	189	.055
3	188	.001
4	184	.068

a. Predictors: (Constant), Party0

b. Predictors: (Constant), Party0, SES0, RaceCC, GenderCC

c. Predictors: (Constant), Party0, SES0, RaceCC, GenderCC, MRN0

d. Predictors: (Constant), Party0, SES0, RaceCC, GenderCC, MRN0, MRN0xSES0, MRN0xParty0, MRN0xGender, MRN0xRace

ANOVA[a]

Model		Sum of Squares	df	Mean Square	F	Sig.
1	Regression	143.453	1	143.453	88.362	.000[b]
	Residual	311.707	192	1.623		
	Total	455.160	193			
2	Regression	155.737	4	38.934	24.576	.000[c]
	Residual	299.423	189	1.584		
	Total	455.160	193			
3	Regression	172.657	5	34.531	22.980	.000[d]
	Residual	282.503	188	1.503		
	Total	455.160	193			
4	Regression	185.683	9	20.631	14.087	.000[e]
	Residual	269.477	184	1.465		
	Total	455.160	193			

a. Dependent Variable: McConnellX

b. Predictors: (Constant), Party0

c. Predictors: (Constant), Party0, SES0, RaceCC, GenderCC

d. Predictors: (Constant), Party0, SES0, RaceCC, GenderCC, MRN0

e. Predictors: (Constant), Party0, SES0, RaceCC, GenderCC, MRN0, MRN0xSES0, MRN0xParty0, MRN0xGender, MRN0xRace

Coefficients[a]

Model		Unstandardized Coefficients B	Std. Error	Standardized Coefficients Beta	t	Sig.
1	(Constant)	3.218	.091		35.173	.000
	Party0	.632	.067	.561	9.400	.000
2	(Constant)	3.187	.107		29.885	.000
	Party0	.667	.071	.592	9.440	.000
	GenderCC	-.251	.093	-.164	-2.689	.008
	RaceCC	.077	.109	.043	.711	.478
	SES0	-.022	.114	-.011	-.191	.849
3	(Constant)	3.188	.104		30.690	.000
	Party0	.560	.076	.497	7.390	.000
	GenderCC	-.366	.097	-.239	-3.763	.000
	RaceCC	.110	.106	.062	1.035	.302
	SES0	-.062	.111	-.032	-.559	.577
	MRN0	.411	.123	.235	3.356	.001
4	(Constant)	3.140	.117		26.773	.000
	Party0	.517	.077	.459	6.759	.000
	GenderCC	-.347	.099	-.226	-3.507	.001
	RaceCC	.144	.107	.080	1.339	.182
	SES0	-.066	.111	-.034	-.597	.551
	MRN0	.270	.138	.155	1.959	.052
	MRN0xRace	.281	.123	.161	2.291	.023
	MRN0xSES0	-.153	.115	-.077	-1.332	.184
	MRN0xGender	.001	.115	.000	.005	.996
	MRN0xParty0	.058	.076	.046	.766	.445

Coefficients[a]

Model		Correlations		
		Zero-order	Partial	Part
1	(Constant)			
	Party0	.561	.561	.561
2	(Constant)			
	Party0	.561	.566	.557
	GenderCC	-.014	-.192	-.159
	RaceCC	.176	.052	.042
	SES0	.011	-.014	-.011
3	(Constant)			
	Party0	.561	.474	.425
	GenderCC	-.014	-.265	-.216
	RaceCC	.176	.075	.059
	SES0	.011	-.041	-.032
	MRN0	.367	.238	.193
4	(Constant)			
	Party0	.561	.446	.383
	GenderCC	-.014	-.250	-.199
	RaceCC	.176	.098	.076
	SES0	.011	-.044	-.034
	MRN0	.367	.143	.111
	MRN0xRace	.369	.167	.130
	MRN0xSES0	-.163	-.098	-.076
	MRN0xGender	.156	.000	.000
	MRN0xParty0	.094	.056	.043

a. Dependent Variable: McConnellX

Excluded Variables[a]

Model		Beta In	t	Sig.	Partial Correlation	Collinearity Statistics Tolerance
1	GenderCC	-.164[b]	-2.696	.008	-.191	.938
	RaceCC	.040[b]	.655	.513	.047	.939
	SES0	-.017[b]	-.286	.775	-.021	.998
	MRN0	.134[b]	2.000	.047	.143	.782
	MRN0xRace	.200[b]	3.215	.002	.227	.881
	MRN0xSES0	-.109[b]	-1.834	.068	-.132	.991
	MRN0xGender	.071[b]	1.171	.243	.084	.976
	MRN0xParty0	.033[b]	.552	.582	.040	.988
2	MRN0	.235[c]	3.356	.001	.238	.672
	MRN0xRace	.231[c]	3.730	.000	.263	.847
	MRN0xSES0	-.104[c]	-1.760	.080	-.127	.982
	MRN0xGender	.060[c]	.990	.324	.072	.954
	MRN0xParty0	.044[c]	.728	.468	.053	.956
3	MRN0xRace	.174[d]	2.556	.011	.184	.694
	MRN0xSES0	-.081[d]	-1.383	.168	-.101	.966
	MRN0xGender	.034[d]	.571	.569	.042	.937
	MRN0xParty0	.074[d]	1.254	.212	.091	.936

a. Dependent Variable: McConnellX

b. Predictors in the Model: (Constant), Party0

c. Predictors in the Model: (Constant), Party0, SES0, RaceCC, GenderCC

d. Predictors in the Model: (Constant), Party0, SES0, RaceCC, GenderCC, MRN0

```
REGRESSION
 /MISSING LISTWISE
 /STATISTICS COEFF OUTS R ANOVA CHANGE ZPP
 /CRITERIA=PIN(.05) POUT(.10)
 /NOORIGIN
 /DEPENDENT FauciX
 /METHOD=ENTER Party0
 /METHOD=ENTER GenderCC RaceCC SES0
 /METHOD=ENTER MRN0
 /METHOD=ENTER MRN0xRace MRN0xSES0 MRN0xGender MRN0xParty0.
```

Regression

Notes

Output Created		15-DEC-2021 13:07:57
Comments		
Input	Data	C:<br>\Users\njs5478\Dropbox\HM and COVID\0. Revise and Resubmit\2. R and R Data\Study 1b\Study1b_Data.sav
	Active Dataset	DataSet1
	Filter	<none>
	Weight	<none>
	Split File	<none>
	N of Rows in Working Data File	241
Missing Value Handling	Definition of Missing	User-defined missing values are treated as missing.
	Cases Used	Statistics are based on cases with no missing values for any variable used.
Syntax		REGRESSION<br>/MISSING LISTWISE<br>/STATISTICS COEFF OUTS R ANOVA CHANGE ZPP<br>/CRITERIA=PIN(.05) POUT(.10)<br>/NOORIGIN<br>/DEPENDENT FauciX<br>/METHOD=ENTER Party0<br>/METHOD=ENTER GenderCC RaceCC SES0<br>/METHOD=ENTER MRN0<br>/METHOD=ENTER MRN0xRace MRN0xSES0 MRN0xGender MRN0xParty0.
Resources	Processor Time	00:00:00.03
	Elapsed Time	00:00:00.05

Notes

	Memory Required	45472 bytes
	Additional Memory Required for Residual Plots	0 bytes

Variables Entered/Removed[a]

Model	Variables Entered	Variables Removed	Method
1	Party0[b]	.	Enter
2	SES0, GenderCC, RaceCC[b]	.	Enter
3	MRN0[b]	.	Enter
4	MRN0xSES0, MRN0xGender, MRN0xParty0, MRN0xRace[b]	.	Enter

a. Dependent Variable: FauciX

b. All requested variables entered.

Model Summary

Model	R	R Square	Adjusted R Square	Std. Error of the Estimate	Change Statistics		
					R Square Change	F Change	df1
1	.338[a]	.114	.110	1.55188	.114	27.016	1
2	.363[b]	.132	.115	1.54752	.018	1.395	3
3	.394[c]	.155	.134	1.53019	.023	5.715	1
4	.418[d]	.175	.138	1.52712	.020	1.207	4

Model Summary

Model	Change Statistics	
	df2	Sig. F Change
1	210	.000
2	207	.245
3	206	.018
4	202	.309

a. Predictors: (Constant), Party0

b. Predictors: (Constant), Party0, SES0, GenderCC, RaceCC

c. Predictors: (Constant), Party0, SES0, GenderCC, RaceCC, MRN0

d. Predictors: (Constant), Party0, SES0, GenderCC, RaceCC, MRN0, MRN0xSES0, MRN0xGender, MRN0xParty0, MRN0xRace

ANOVA[a]

Model		Sum of Squares	df	Mean Square	F	Sig.
1	Regression	65.063	1	65.063	27.016	.000[b]
	Residual	505.749	210	2.408		
	Total	570.811	211			
2	Regression	75.083	4	18.771	7.838	.000[c]
	Residual	495.729	207	2.395		
	Total	570.811	211			
3	Regression	88.464	5	17.693	7.556	.000[d]
	Residual	482.347	206	2.341		
	Total	570.811	211			
4	Regression	99.728	9	11.081	4.751	.000[e]
	Residual	471.084	202	2.332		
	Total	570.811	211			

a. Dependent Variable: FauciX

b. Predictors: (Constant), Party0

c. Predictors: (Constant), Party0, SES0, GenderCC, RaceCC

d. Predictors: (Constant), Party0, SES0, GenderCC, RaceCC, MRN0

e. Predictors: (Constant), Party0, SES0, GenderCC, RaceCC, MRN0, MRN0xSES0, MRN0xGender, MRN0xParty0, MRN0xRace

Coefficients[a]

Model		Unstandardized Coefficients B	Unstandardized Coefficients Std. Error	Standardized Coefficients Beta	t	Sig.
1	(Constant)	5.131	.107		48.137	.000
	Party0	-.400	.077	-.338	-5.198	.000
2	(Constant)	5.046	.121		41.773	.000
	Party0	-.443	.083	-.374	-5.333	.000
	GenderCC	.020	.111	.012	.181	.857
	RaceCC	.176	.125	.095	1.401	.163
	SES0	.187	.133	.091	1.401	.163
3	(Constant)	5.064	.120		42.313	.000
	Party0	-.358	.089	-.303	-4.005	.000
	GenderCC	.134	.119	.082	1.125	.262
	RaceCC	.149	.124	.081	1.198	.232
	SES0	.218	.132	.106	1.644	.102
	MRN0	-.362	.151	-.190	-2.391	.018
4	(Constant)	5.159	.140		36.742	.000
	Party0	-.351	.092	-.296	-3.812	.000
	GenderCC	.104	.121	.064	.863	.389
	RaceCC	.108	.126	.058	.853	.395
	SES0	.229	.133	.112	1.726	.086
	MRN0	-.282	.165	-.148	-1.706	.090
	MRN0xRace	-.056	.143	-.029	-.391	.696
	MRN0xSES0	.035	.142	.016	.246	.806
	MRN0xGender	-.295	.144	-.137	-2.045	.042
	MRN0xParty0	.075	.093	.056	.806	.421

Coefficients[a]

Model		Correlations Zero-order	Partial	Part
1	(Constant)			
	Party0	-.338	-.338	-.338
2	(Constant)			
	Party0	-.338	-.348	-.345
	GenderCC	-.075	.013	.012
	RaceCC	-.007	.097	.091
	SES0	.074	.097	.091
3	(Constant)			
	Party0	-.338	-.269	-.257
	GenderCC	-.075	.078	.072
	RaceCC	-.007	.083	.077
	SES0	.074	.114	.105
	MRN0	-.272	-.164	-.153
4	(Constant)			
	Party0	-.338	-.259	-.244
	GenderCC	-.075	.061	.055
	RaceCC	-.007	.060	.055
	SES0	.074	.121	.110
	MRN0	-.272	-.119	-.109
	MRN0xRace	-.195	-.028	-.025
	MRN0xSES0	.058	.017	.016
	MRN0xGender	-.190	-.142	-.131
	MRN0xParty0	-.023	.057	.051

a. Dependent Variable: FauciX

Excluded Variables[a]

Model		Beta In	t	Sig.	Partial Correlation	Collinearity Statistics Tolerance
1	GenderCC	.016[b]	.240	.811	.017	.928
	RaceCC	.099[b]	1.465	.144	.101	.917
	SES0	.096[b]	1.483	.140	.102	.996
	MRN0	-.148[b]	-2.044	.042	-.140	.790
	MRN0xRace	-.094[b]	-1.368	.173	-.094	.892
	MRN0xSES0	.035[b]	.544	.587	.038	.995
	MRN0xGender	-.156[b]	-2.411	.017	-.164	.988
	MRN0xParty0	.045[b]	.681	.497	.047	.961
2	MRN0	-.190[c]	-2.391	.018	-.164	.650
	MRN0xRace	-.086[c]	-1.252	.212	-.087	.879
	MRN0xSES0	.039[c]	.591	.555	.041	.992
	MRN0xGender	-.148[c]	-2.270	.024	-.156	.972
	MRN0xParty0	.048[c]	.712	.477	.050	.943
3	MRN0xRace	-.037[d]	-.505	.614	-.035	.784
	MRN0xSES0	.023[d]	.359	.720	.025	.982
	MRN0xGender	-.131[d]	-2.025	.044	-.140	.959
	MRN0xParty0	.026[d]	.382	.703	.027	.924

a. Dependent Variable: FauciX

b. Predictors in the Model: (Constant), Party0

c. Predictors in the Model: (Constant), Party0, SES0, GenderCC, RaceCC

d. Predictors in the Model: (Constant), Party0, SES0, GenderCC, RaceCC, MRN0

```
REGRESSION
 /MISSING LISTWISE
 /STATISTICS COEFF OUTS R ANOVA CHANGE ZPP
 /CRITERIA=PIN(.05) POUT(.10)
 /NOORIGIN
 /DEPENDENT RepCongressX
 /METHOD=ENTER Party0
 /METHOD=ENTER GenderCC RaceCC SES0
 /METHOD=ENTER MRN0
 /METHOD=ENTER MRN0xRace MRN0xSES0 MRN0xGender MRN0xParty0.
```

Regression

Notes

Output Created		15-DEC-2021 13:07:57
Comments		
Input	Data	C: \Users\njs5478\Dropbox\HM and COVID\0. Revise and Resubmit\2. R and R Data\Study 1b\Study1b_Data.sav
	Active Dataset	DataSet1
	Filter	<none>
	Weight	<none>
	Split File	<none>
	N of Rows in Working Data File	241
Missing Value Handling	Definition of Missing	User-defined missing values are treated as missing.
	Cases Used	Statistics are based on cases with no missing values for any variable used.
Syntax		REGRESSION<br>/MISSING LISTWISE<br>/STATISTICS COEFF OUTS R ANOVA CHANGE ZPP<br>/CRITERIA=PIN(.05) POUT(.10)<br>/NOORIGIN<br>/DEPENDENT RepCongressX<br>/METHOD=ENTER Party0<br>/METHOD=ENTER GenderCC RaceCC SES0<br>/METHOD=ENTER MRN0<br>/METHOD=ENTER MRN0xRace MRN0xSES0 MRN0xGender MRN0xParty0.
Resources	Processor Time	00:00:00.05
	Elapsed Time	00:00:00.02

Notes

	Memory Required	45472 bytes
	Additional Memory Required for Residual Plots	0 bytes

Variables Entered/Removed[a]

Model	Variables Entered	Variables Removed	Method
1	Party0[b]	.	Enter
2	SES0, GenderCC, RaceCC[b]	.	Enter
3	MRN0[b]	.	Enter
4	MRN0xSES0, MRN0xParty0 , MRN0xGende r, ...[b]	.	Enter

a. Dependent Variable: RepCongressX

b. All requested variables entered.

Model Summary

Model	R	R Square	Adjusted R Square	Std. Error of the Estimate	Change Statistics		
					R Square Change	F Change	df1
1	.701[a]	.491	.489	1.24249	.491	205.770	1
2	.718[b]	.516	.506	1.22113	.024	3.505	3
3	.728[c]	.529	.518	1.20655	.014	6.104	1
4	.737[d]	.544	.524	1.19946	.014	1.619	4

Model Summary

Model	Change Statistics	
	df2	Sig. F Change
1	213	.000
2	210	.016
3	209	.014
4	205	.171

a. Predictors: (Constant), Party0

b. Predictors: (Constant), Party0, SES0, GenderCC, RaceCC

c. Predictors: (Constant), Party0, SES0, GenderCC, RaceCC, MRN0

d. Predictors: (Constant), Party0, SES0, GenderCC, RaceCC, MRN0, MRN0xSES0, MRN0xParty0, MRN0xGender, MRN0xRace

ANOVA[a]

Model		Sum of Squares	df	Mean Square	F	Sig.
1	Regression	317.661	1	317.661	205.770	.000[b]
	Residual	328.823	213	1.544		
	Total	646.484	214			
2	Regression	333.342	4	83.335	55.887	.000[c]
	Residual	313.142	210	1.491		
	Total	646.484	214			
3	Regression	342.227	5	68.445	47.016	.000[d]
	Residual	304.257	209	1.456		
	Total	646.484	214			
4	Regression	351.547	9	39.061	27.150	.000[e]
	Residual	294.937	205	1.439		
	Total	646.484	214			

a. Dependent Variable: RepCongressX

b. Predictors: (Constant), Party0

c. Predictors: (Constant), Party0, SES0, GenderCC, RaceCC

d. Predictors: (Constant), Party0, SES0, GenderCC, RaceCC, MRN0

e. Predictors: (Constant), Party0, SES0, GenderCC, RaceCC, MRN0, MRN0xSES0, MRN0xParty0, MRN0xGender, MRN0xRace

Coefficients[a]

Model		Unstandardized Coefficients B	Std. Error	Standardized Coefficients Beta	t	Sig.
1	(Constant)	3.386	.085		39.960	.000
	Party0	.883	.062	.701	14.345	.000
2	(Constant)	3.228	.096		33.455	.000
	Party0	.824	.065	.654	12.667	.000
	GenderCC	.028	.086	.016	.321	.748
	RaceCC	.321	.099	.161	3.234	.001
	SES0	-.022	.100	-.011	-.221	.826
3	(Constant)	3.220	.095		33.758	.000
	Party0	.755	.070	.599	10.762	.000
	GenderCC	-.055	.092	-.032	-.600	.549
	RaceCC	.335	.098	.169	3.412	.001
	SES0	-.046	.099	-.022	-.466	.642
	MRN0	.284	.115	.143	2.471	.014
4	(Constant)	3.167	.112		28.381	.000
	Party0	.716	.072	.569	9.977	.000
	GenderCC	-.047	.094	-.027	-.504	.615
	RaceCC	.342	.099	.172	3.440	.001
	SES0	-.020	.100	-.010	-.200	.842
	MRN0	.213	.128	.107	1.664	.098
	MRN0xRace	.167	.110	.084	1.518	.131
	MRN0xSES0	-.130	.105	-.060	-1.234	.219
	MRN0xGender	.014	.111	.006	.129	.897
	MRN0xParty0	.082	.074	.057	1.114	.267

Coefficients[a]

Model		Correlations Zero-order	Partial	Part
1	(Constant)			
	Party0	.701	.701	.701
2	(Constant)			
	Party0	.701	.658	.608
	GenderCC	.193	.022	.015
	RaceCC	.336	.218	.155
	SES0	.035	-.015	-.011
3	(Constant)			
	Party0	.701	.597	.511
	GenderCC	.193	-.041	-.028
	RaceCC	.336	.230	.162
	SES0	.035	-.032	-.022
	MRN0	.416	.168	.117
4	(Constant)			
	Party0	.701	.572	.471
	GenderCC	.193	-.035	-.024
	RaceCC	.336	.234	.162
	SES0	.035	-.014	-.009
	MRN0	.416	.115	.079
	MRN0xRace	.319	.105	.072
	MRN0xSES0	-.171	-.086	-.058
	MRN0xGender	.141	.009	.006
	MRN0xParty0	.183	.078	.053

a. Dependent Variable: RepCongressX

Excluded Variables[a]

Model		Beta In	t	Sig.	Partial Correlation	Collinearity Statistics Tolerance
1	GenderCC	.011[b]	.210	.834	.014	.932
	RaceCC	.160[b]	3.234	.001	.217	.929
	SES0	-.005[b]	-.104	.918	-.007	.997
	MRN0	.116[b]	2.131	.034	.145	.786
	MRN0xRace	.109[b]	2.143	.033	.146	.901
	MRN0xSES0	-.085[b]	-1.744	.083	-.119	.985
	MRN0xGender	.028[b]	.565	.573	.039	.974
	MRN0xParty0	.066[b]	1.333	.184	.091	.971
2	MRN0	.143[c]	2.471	.014	.168	.672
	MRN0xRace	.131[c]	2.603	.010	.177	.883
	MRN0xSES0	-.082[c]	-1.698	.091	-.117	.981
	MRN0xGender	.051[c]	1.039	.300	.072	.953
	MRN0xParty0	.056[c]	1.126	.262	.078	.938
3	MRN0xRace	.101[d]	1.903	.058	.131	.787
	MRN0xSES0	-.061[d]	-1.260	.209	-.087	.944
	MRN0xGender	.031[d]	.628	.531	.044	.924
	MRN0xParty0	.077[d]	1.566	.119	.108	.913

a. Dependent Variable: RepCongressX

b. Predictors in the Model: (Constant), Party0

c. Predictors in the Model: (Constant), Party0, SES0, GenderCC, RaceCC

d. Predictors in the Model: (Constant), Party0, SES0, GenderCC, RaceCC, MRN0

```
REGRESSION
 /MISSING LISTWISE
 /STATISTICS COEFF OUTS R ANOVA CHANGE ZPP
 /CRITERIA=PIN(.05) POUT(.10)
 /NOORIGIN
 /DEPENDENT DemCongressX
 /METHOD=ENTER Party0
 /METHOD=ENTER GenderCC RaceCC SES0
 /METHOD=ENTER MRN0
 /METHOD=ENTER MRN0xRace MRN0xSES0 MRN0xGender MRN0xParty0.
```

Regression

Notes

Output Created		15-DEC-2021 13:07:57
Comments		
Input	Data	C: \Users\njs5478\Dropbox\HM and COVID\0. Revise and Resubmit\2. R and R Data\Study 1b\Study1b_Data.sav
	Active Dataset	DataSet1
	Filter	<none>
	Weight	<none>
	Split File	<none>
	N of Rows in Working Data File	241
Missing Value Handling	Definition of Missing	User-defined missing values are treated as missing.
	Cases Used	Statistics are based on cases with no missing values for any variable used.
Syntax		REGRESSION /MISSING LISTWISE /STATISTICS COEFF OUTS R ANOVA CHANGE ZPP /CRITERIA=PIN(.05) POUT(.10) /NOORIGIN /DEPENDENT DemCongressX /METHOD=ENTER Party0 /METHOD=ENTER GenderCC RaceCC SES0 /METHOD=ENTER MRN0 /METHOD=ENTER MRN0xRace MRN0xSES0 MRN0xGender MRN0xParty0.
Resources	Processor Time	00:00:00.02
	Elapsed Time	00:00:00.01

Notes

	Memory Required	45472 bytes
	Additional Memory Required for Residual Plots	0 bytes

Variables Entered/Removed[a]

Model	Variables Entered	Variables Removed	Method
1	Party0[b]	.	Enter
2	SES0, GenderCC, RaceCC[b]	.	Enter
3	MRN0[b]	.	Enter
4	MRN0xSES0, MRN0xGender, MRN0xParty0, MRN0xRace[b]	.	Enter

a. Dependent Variable: DemCongressX

b. All requested variables entered.

Model Summary

Model	R	R Square	Adjusted R Square	Std. Error of the Estimate	Change Statistics		
					R Square Change	F Change	df1
1	.610[a]	.373	.370	1.26172	.373	125.258	1
2	.613[b]	.376	.364	1.26747	.003	.364	3
3	.619[c]	.383	.368	1.26311	.007	2.438	1
4	.640[d]	.410	.383	1.24783	.026	2.275	4

Model Summary

Model	Change Statistics	
	df2	Sig. F Change
1	211	.000
2	208	.779
3	207	.120
4	203	.062

a. Predictors: (Constant), Party0

b. Predictors: (Constant), Party0, SES0, GenderCC, RaceCC

c. Predictors: (Constant), Party0, SES0, GenderCC, RaceCC, MRN0

d. Predictors: (Constant), Party0, SES0, GenderCC, RaceCC, MRN0, MRN0xSES0, MRN0xGender, MRN0xParty0, MRN0xRace

ANOVA[a]

Model		Sum of Squares	df	Mean Square	F	Sig.
1	Regression	199.404	1	199.404	125.258	.000[b]
	Residual	335.901	211	1.592		
	Total	535.305	212			
2	Regression	201.158	4	50.289	31.304	.000[c]
	Residual	334.147	208	1.606		
	Total	535.305	212			
3	Regression	205.048	5	41.010	25.704	.000[d]
	Residual	330.257	207	1.595		
	Total	535.305	212			
4	Regression	219.220	9	24.358	15.643	.000[e]
	Residual	316.085	203	1.557		
	Total	535.305	212			

a. Dependent Variable: DemCongressX

b. Predictors: (Constant), Party0

c. Predictors: (Constant), Party0, SES0, GenderCC, RaceCC

d. Predictors: (Constant), Party0, SES0, GenderCC, RaceCC, MRN0

e. Predictors: (Constant), Party0, SES0, GenderCC, RaceCC, MRN0, MRN0xSES0, MRN0xGender, MRN0xParty0, MRN0xRace

Coefficients[a]

Model		Unstandardized Coefficients B	Unstandardized Coefficients Std. Error	Standardized Coefficients Beta	t	Sig.
1	(Constant)	4.241	.086		49.052	.000
	Party0	-.702	.063	-.610	-11.192	.000
2	(Constant)	4.212	.100		42.007	.000
	Party0	-.699	.068	-.607	-10.271	.000
	GenderCC	-.075	.090	-.047	-.830	.407
	RaceCC	.061	.103	.034	.588	.557
	SES0	.012	.104	.006	.115	.909
3	(Constant)	4.217	.100		42.181	.000
	Party0	-.654	.074	-.568	-8.890	.000
	GenderCC	-.018	.097	-.011	-.184	.854
	RaceCC	.053	.103	.029	.511	.610
	SES0	.027	.104	.014	.261	.795
	MRN0	-.190	.122	-.104	-1.562	.120
4	(Constant)	4.348	.116		37.404	.000
	Party0	-.633	.075	-.550	-8.464	.000
	GenderCC	-.056	.098	-.035	-.566	.572
	RaceCC	.012	.104	.007	.115	.909
	SES0	.035	.104	.019	.338	.735
	MRN0	-.112	.133	-.061	-.841	.401
	MRN0xRace	-.022	.115	-.012	-.191	.848
	MRN0xSES0	.029	.109	.015	.260	.795
	MRN0xGender	-.339	.116	-.166	-2.913	.004
	MRN0xParty0	.035	.078	.026	.445	.657

Coefficients[a]

Model		Correlations Zero-order	Partial	Part
1	(Constant)			
	Party0	-.610	-.610	-.610
2	(Constant)			
	Party0	-.610	-.580	-.563
	GenderCC	-.208	-.057	-.045
	RaceCC	-.132	.041	.032
	SES0	-.027	.008	.006
3	(Constant)			
	Party0	-.610	-.526	-.485
	GenderCC	-.208	-.013	-.010
	RaceCC	-.132	.035	.028
	SES0	-.027	.018	.014
	MRN0	-.365	-.108	-.085
4	(Constant)			
	Party0	-.610	-.511	-.456
	GenderCC	-.208	-.040	-.031
	RaceCC	-.132	.008	.006
	SES0	-.027	.024	.018
	MRN0	-.365	-.059	-.045
	MRN0xRace	-.240	-.013	-.010
	MRN0xSES0	.122	.018	.014
	MRN0xGender	-.262	-.200	-.157
	MRN0xParty0	-.102	.031	.024

a. Dependent Variable: DemCongressX

Excluded Variables[a]

Model		Beta In	t	Sig.	Partial Correlation	Collinearity Statistics Tolerance
1	GenderCC	-.048[b]	-.856	.393	-.059	.929
	RaceCC	.036[b]	.628	.530	.043	.926
	SES0	.007[b]	.121	.904	.008	.997
	MRN0	-.109[b]	-1.788	.075	-.122	.791
	MRN0xRace	-.058[b]	-1.013	.312	-.070	.906
	MRN0xSES0	.048[b]	.880	.380	.061	.985
	MRN0xGender	-.174[b]	-3.229	.001	-.217	.978
	MRN0xParty0	.009[b]	.165	.869	.011	.967
2	MRN0	-.104[c]	-1.562	.120	-.108	.671
	MRN0xRace	-.051[c]	-.878	.381	-.061	.887
	MRN0xSES0	.052[c]	.938	.349	.065	.982
	MRN0xGender	-.175[c]	-3.198	.002	-.217	.960
	MRN0xParty0	.013[c]	.225	.822	.016	.938
3	MRN0xRace	-.025[d]	-.408	.684	-.028	.798
	MRN0xSES0	.037[d]	.655	.513	.046	.946
	MRN0xGender	-.165[d]	-2.990	.003	-.204	.937
	MRN0xParty0	.000[d]	-.001	.999	.000	.918

a. Dependent Variable: DemCongressX

b. Predictors in the Model: (Constant), Party0

c. Predictors in the Model: (Constant), Party0, SES0, GenderCC, RaceCC

d. Predictors in the Model: (Constant), Party0, SES0, GenderCC, RaceCC, MRN0

```
REGRESSION
 /MISSING LISTWISE
 /STATISTICS COEFF OUTS R ANOVA CHANGE ZPP
 /CRITERIA=PIN(.05) POUT(.10)
 /NOORIGIN
 /DEPENDENT StateX
 /METHOD=ENTER Party0
 /METHOD=ENTER GenderCC RaceCC SES0
 /METHOD=ENTER MRN0
 /METHOD=ENTER MRN0xRace MRN0xSES0 MRN0xGender MRN0xParty0.
```

Regression

Notes

Output Created		15-DEC-2021 13:07:57
Comments		
Input	Data	C: \Users\njs5478\Dropbox\HM and COVID\0. Revise and Resubmit\2. R and R Data\Study 1b\Study1b_Data.sav
	Active Dataset	DataSet1
	Filter	<none>
	Weight	<none>
	Split File	<none>
	N of Rows in Working Data File	241
Missing Value Handling	Definition of Missing	User-defined missing values are treated as missing.
	Cases Used	Statistics are based on cases with no missing values for any variable used.
Syntax		REGRESSION<br>/MISSING LISTWISE<br>/STATISTICS COEFF OUTS R ANOVA CHANGE ZPP<br>/CRITERIA=PIN(.05) POUT(.10)<br>/NOORIGIN<br>/DEPENDENT StateX<br>/METHOD=ENTER Party0<br>/METHOD=ENTER GenderCC RaceCC SES0<br>/METHOD=ENTER MRN0<br>/METHOD=ENTER MRN0xRace MRN0xSES0 MRN0xGender MRN0xParty0.
Resources	Processor Time	00:00:00.02
	Elapsed Time	00:00:00.01

Notes

	Memory Required	45472 bytes
	Additional Memory Required for Residual Plots	0 bytes

Variables Entered/Removed[a]

Model	Variables Entered	Variables Removed	Method
1	Party0[b]	.	Enter
2	SES0, GenderCC, RaceCC[b]	.	Enter
3	MRN0[b]	.	Enter
4	MRN0xSES0, MRN0xParty0 , MRN0xGende r, ...[b]	.	Enter

a. Dependent Variable: StateX

b. All requested variables entered.

Model Summary

Model	R	R Square	Adjusted R Square	Std. Error of the Estimate	Change Statistics		
					R Square Change	F Change	df1
1	.336[a]	.113	.109	1.51157	.113	28.085	1
2	.338[b]	.114	.098	1.52086	.001	.103	3
3	.338[c]	.114	.094	1.52431	.000	.014	1
4	.387[d]	.150	.114	1.50699	.036	2.254	4

Model Summary

Model	Change Statistics	
	df2	Sig. F Change
1	221	.000
2	218	.958
3	217	.905
4	213	.064

a. Predictors: (Constant), Party0

b. Predictors: (Constant), Party0, SES0, GenderCC, RaceCC

c. Predictors: (Constant), Party0, SES0, GenderCC, RaceCC, MRN0

d. Predictors: (Constant), Party0, SES0, GenderCC, RaceCC, MRN0, MRN0xSES0, MRN0xParty0, MRN0xGender, MRN0xRace

ANOVA[a]

Model		Sum of Squares	df	Mean Square	F	Sig.
1	Regression	64.171	1	64.171	28.085	.000[b]
	Residual	504.950	221	2.285		
	Total	569.121	222			
2	Regression	64.884	4	16.221	7.013	.000[c]
	Residual	504.237	218	2.313		
	Total	569.121	222			
3	Regression	64.917	5	12.983	5.588	.000[d]
	Residual	504.204	217	2.324		
	Total	569.121	222			
4	Regression	85.394	9	9.488	4.178	.000[e]
	Residual	483.727	213	2.271		
	Total	569.121	222			

a. Dependent Variable: StateX

b. Predictors: (Constant), Party0

c. Predictors: (Constant), Party0, SES0, GenderCC, RaceCC

d. Predictors: (Constant), Party0, SES0, GenderCC, RaceCC, MRN0

e. Predictors: (Constant), Party0, SES0, GenderCC, RaceCC, MRN0, MRN0xSES0, MRN0xParty0, MRN0xGender, MRN0xRace

Coefficients[a]

Model		Unstandardized Coefficients B	Std. Error	Standardized Coefficients Beta	t	Sig.
1	(Constant)	4.071	.101		40.214	.000
	Party0	-.387	.073	-.336	-5.300	.000
2	(Constant)	4.104	.119		34.421	.000
	Party0	-.373	.078	-.324	-4.746	.000
	GenderCC	-.020	.105	-.013	-.194	.846
	RaceCC	-.065	.123	-.035	-.528	.598
	SES0	.001	.124	.001	.011	.991
3	(Constant)	4.105	.120		34.309	.000
	Party0	-.368	.087	-.320	-4.240	.000
	GenderCC	-.016	.112	-.010	-.140	.889
	RaceCC	-.066	.124	-.036	-.536	.592
	SES0	.003	.125	.002	.024	.981
	MRN0	-.017	.143	-.009	-.119	.905
4	(Constant)	4.173	.136		30.666	.000
	Party0	-.379	.088	-.329	-4.289	.000
	GenderCC	-.075	.114	-.047	-.652	.515
	RaceCC	-.105	.124	-.057	-.843	.400
	SES0	.029	.125	.015	.236	.814
	MRN0	.057	.160	.031	.357	.721
	MRN0xRace	.099	.139	.054	.714	.476
	MRN0xSES0	.070	.131	.035	.539	.590
	MRN0xGender	-.346	.135	-.172	-2.565	.011
	MRN0xParty0	.122	.090	.093	1.358	.176

Coefficients[a]

Model		Correlations		
		Zero-order	Partial	Part
1	(Constant)			
	Party0	-.336	-.336	-.336
2	(Constant)			
	Party0	-.336	-.306	-.303
	GenderCC	-.091	-.013	-.012
	RaceCC	-.121	-.036	-.034
	SES0	-.022	.001	.001
3	(Constant)			
	Party0	-.336	-.277	-.271
	GenderCC	-.091	-.009	-.009
	RaceCC	-.121	-.036	-.034
	SES0	-.022	.002	.002
	MRN0	-.164	-.008	-.008
4	(Constant)			
	Party0	-.336	-.282	-.271
	GenderCC	-.091	-.045	-.041
	RaceCC	-.121	-.058	-.053
	SES0	-.022	.016	.015
	MRN0	-.164	.024	.023
	MRN0xRace	-.075	.049	.045
	MRN0xSES0	.091	.037	.034
	MRN0xGender	-.196	-.173	-.162
	MRN0xParty0	.012	.093	.086

a. Dependent Variable: StateX

Excluded Variables[a]

Model		Beta In	t	Sig.	Partial Correlation	Collinearity Statistics Tolerance
1	GenderCC	-.011[b]	-.172	.863	-.012	.943
	RaceCC	-.034[b]	-.523	.602	-.035	.930
	SES0	-.001[b]	-.010	.992	-.001	.996
	MRN0	-.009[b]	-.124	.902	-.008	.782
	MRN0xRace	.051[b]	.747	.456	.050	.874
	MRN0xSES0	.054[b]	.843	.400	.057	.987
	MRN0xGender	-.146[b]	-2.290	.023	-.153	.974
	MRN0xParty0	.073[b]	1.138	.257	.076	.969
2	MRN0	-.009[c]	-.119	.905	-.008	.671
	MRN0xRace	.048[c]	.702	.483	.048	.858
	MRN0xSES0	.053[c]	.829	.408	.056	.984
	MRN0xGender	-.153[c]	-2.371	.019	-.159	.957
	MRN0xParty0	.081[c]	1.226	.221	.083	.932
3	MRN0xRace	.060[d]	.802	.424	.054	.740
	MRN0xSES0	.054[d]	.819	.413	.056	.951
	MRN0xGender	-.157[d]	-2.389	.018	-.160	.921
	MRN0xParty0	.081[d]	1.218	.224	.083	.915

a. Dependent Variable: StateX

b. Predictors in the Model: (Constant), Party0

c. Predictors in the Model: (Constant), Party0, SES0, GenderCC, RaceCC

d. Predictors in the Model: (Constant), Party0, SES0, GenderCC, RaceCC, MRN0

```
REGRESSION
 /MISSING LISTWISE
 /STATISTICS COEFF OUTS R ANOVA CHANGE ZPP
 /CRITERIA=PIN(.05) POUT(.10)
 /NOORIGIN
 /DEPENDENT Risk_Rules
 /METHOD=ENTER Party0
 /METHOD=ENTER GenderCC RaceCC SES0
 /METHOD=ENTER MRN0
 /METHOD=ENTER MRN0xRace MRN0xSES0 MRN0xGender MRN0xParty0.
```

Regression

Notes

Output Created		15-DEC-2021 13:07:57
Comments		
Input	Data	C: \Users\njs5478\Dropbox\HM and COVID\0. Revise and Resubmit\2. R and R Data\Study 1b\Study1b_Data.sav
	Active Dataset	DataSet1
	Filter	<none>
	Weight	<none>
	Split File	<none>
	N of Rows in Working Data File	241
Missing Value Handling	Definition of Missing	User-defined missing values are treated as missing.
	Cases Used	Statistics are based on cases with no missing values for any variable used.
Syntax		REGRESSION /MISSING LISTWISE /STATISTICS COEFF OUTS R ANOVA CHANGE ZPP /CRITERIA=PIN(.05) POUT(.10) /NOORIGIN /DEPENDENT Risk_Rules /METHOD=ENTER Party0 /METHOD=ENTER GenderCC RaceCC SES0 /METHOD=ENTER MRN0 /METHOD=ENTER MRN0xRace MRN0xSES0 MRN0xGender MRN0xParty0.
Resources	Processor Time	00:00:00.03
	Elapsed Time	00:00:00.03

Notes

	Memory Required	45472 bytes
	Additional Memory Required for Residual Plots	0 bytes

Variables Entered/Removed[a]

Model	Variables Entered	Variables Removed	Method
1	Party0[b]	.	Enter
2	SES0, GenderCC, RaceCC[b]	.	Enter
3	MRN0[b]	.	Enter
4	MRN0xSES0, MRN0xGender, MRN0xParty0, MRN0xRace[b]	.	Enter

a. Dependent Variable: Risk_Rules

b. All requested variables entered.

Model Summary

Model	R	R Square	Adjusted R Square	Std. Error of the Estimate	Change Statistics		
					R Square Change	F Change	df1
1	.423[a]	.179	.175	.90793	.179	51.417	1
2	.442[b]	.195	.181	.90466	.016	1.570	3
3	.541[c]	.293	.278	.84963	.098	32.157	1
4	.556[d]	.309	.282	.84742	.016	1.304	4

Model Summary

Model	Change Statistics	
	df2	Sig. F Change
1	236	.000
2	233	.197
3	232	.000
4	228	.269

a. Predictors: (Constant), Party0

b. Predictors: (Constant), Party0, SES0, GenderCC, RaceCC

c. Predictors: (Constant), Party0, SES0, GenderCC, RaceCC, MRN0

d. Predictors: (Constant), Party0, SES0, GenderCC, RaceCC, MRN0, MRN0xSES0, MRN0xGender, MRN0xParty0, MRN0xRace

ANOVA[a]

Model		Sum of Squares	df	Mean Square	F	Sig.
1	Regression	42.386	1	42.386	51.417	.000[b]
	Residual	194.545	236	.824		
	Total	236.931	237			
2	Regression	46.241	4	11.560	14.125	.000[c]
	Residual	190.690	233	.818		
	Total	236.931	237			
3	Regression	69.455	5	13.891	19.243	.000[d]
	Residual	167.476	232	.722		
	Total	236.931	237			
4	Regression	73.201	9	8.133	11.326	.000[e]
	Residual	163.730	228	.718		
	Total	236.931	237			

a. Dependent Variable: Risk_Rules

b. Predictors: (Constant), Party0

c. Predictors: (Constant), Party0, SES0, GenderCC, RaceCC

d. Predictors: (Constant), Party0, SES0, GenderCC, RaceCC, MRN0

e. Predictors: (Constant), Party0, SES0, GenderCC, RaceCC, MRN0, MRN0xSES0, MRN0xGender, MRN0xParty0, MRN0xRace

Coefficients[a]

Model		Unstandardized Coefficients B	Unstandardized Coefficients Std. Error	Standardized Coefficients Beta	t	Sig.
1	(Constant)	3.303	.059		56.129	.000
	Party0	.304	.042	.423	7.171	.000
2	(Constant)	3.273	.068		47.905	.000
	Party0	.273	.045	.379	6.006	.000
	GenderCC	.121	.061	.121	1.993	.047
	RaceCC	.063	.071	.054	.887	.376
	SES0	-.028	.073	-.023	-.388	.699
3	(Constant)	3.256	.064		50.684	.000
	Party0	.161	.047	.223	3.416	.001
	GenderCC	-.001	.061	-.001	-.009	.993
	RaceCC	.098	.067	.085	1.476	.141
	SES0	-.071	.069	-.057	-1.031	.304
	MRN0	.441	.078	.382	5.671	.000
4	(Constant)	3.243	.074		43.932	.000
	Party0	.143	.048	.199	2.973	.003
	GenderCC	.021	.062	.021	.338	.736
	RaceCC	.114	.068	.099	1.687	.093
	SES0	-.065	.069	-.053	-.946	.345
	MRN0	.358	.087	.310	4.100	.000
	MRN0xRace	.142	.076	.123	1.868	.063
	MRN0xSES0	-.078	.073	-.061	-1.069	.286
	MRN0xGender	.035	.074	.027	.474	.636
	MRN0xParty0	-.012	.049	-.015	-.250	.803

Coefficients[a]

Model		Correlations		
		Zero-order	Partial	Part
1	(Constant)			
	Party0	.423	.423	.423
2	(Constant)			
	Party0	.423	.366	.353
	GenderCC	.213	.129	.117
	RaceCC	.166	.058	.052
	SES0	.001	-.025	-.023
3	(Constant)			
	Party0	.423	.219	.189
	GenderCC	.213	-.001	.000
	RaceCC	.166	.096	.081
	SES0	.001	-.068	-.057
	MRN0	.483	.349	.313
4	(Constant)			
	Party0	.423	.193	.164
	GenderCC	.213	.022	.019
	RaceCC	.166	.111	.093
	SES0	.001	-.063	-.052
	MRN0	.483	.262	.226
	MRN0xRace	.340	.123	.103
	MRN0xSES0	-.159	-.071	-.059
	MRN0xGender	.128	.031	.026
	MRN0xParty0	.050	-.017	-.014

a. Dependent Variable: Risk_Rules

Excluded Variables[a]

Model		Beta In	t	Sig.	Partial Correlation	Collinearity Statistics Tolerance
1	GenderCC	.118[b]	1.952	.052	.126	.942
	RaceCC	.049[b]	.801	.424	.052	.919
	SES0	-.017[b]	-.279	.780	-.018	.998
	MRN0	.365[b]	5.860	.000	.357	.784
	MRN0xRace	.223[b]	3.658	.000	.232	.888
	MRN0xSES0	-.113[b]	-1.915	.057	-.124	.988
	MRN0xGender	.080[b]	1.343	.180	.087	.986
	MRN0xParty0	-.014[b]	-.239	.811	-.016	.977
2	MRN0	.382[c]	5.671	.000	.349	.671
	MRN0xRace	.228[c]	3.709	.000	.237	.867
	MRN0xSES0	-.119[c]	-2.017	.045	-.131	.984
	MRN0xGender	.093[c]	1.557	.121	.102	.968
	MRN0xParty0	-.031[c]	-.515	.607	-.034	.949
3	MRN0xRace	.122[d]	1.928	.055	.126	.753
	MRN0xSES0	-.064[d]	-1.128	.261	-.074	.952
	MRN0xGender	.042[d]	.738	.462	.048	.942
	MRN0xParty0	.016[d]	.272	.786	.018	.929

a. Dependent Variable: Risk_Rules

b. Predictors in the Model: (Constant), Party0

c. Predictors in the Model: (Constant), Party0, SES0, GenderCC, RaceCC

d. Predictors in the Model: (Constant), Party0, SES0, GenderCC, RaceCC, MRN0

```
REGRESSION
 /MISSING LISTWISE
 /STATISTICS COEFF OUTS R ANOVA CHANGE ZPP
 /CRITERIA=PIN(.05) POUT(.10)
 /NOORIGIN
 /DEPENDENT Risk_Help
 /METHOD=ENTER Party0
 /METHOD=ENTER GenderCC RaceCC SES0
 /METHOD=ENTER MRN0
 /METHOD=ENTER MRN0xRace MRN0xSES0 MRN0xGender MRN0xParty0.
```

Regression

Notes

Output Created		15-DEC-2021 13:07:57
Comments		
Input	Data	C: \Users\njs5478\Dropbox\HM and COVID\0. Revise and Resubmit\2. R and R Data\Study 1b\Study1b_Data.sav
	Active Dataset	DataSet1
	Filter	<none>
	Weight	<none>
	Split File	<none>
	N of Rows in Working Data File	241
Missing Value Handling	Definition of Missing	User-defined missing values are treated as missing.
	Cases Used	Statistics are based on cases with no missing values for any variable used.
Syntax		REGRESSION<br>/MISSING LISTWISE<br>/STATISTICS COEFF OUTS R ANOVA CHANGE ZPP<br>/CRITERIA=PIN(.05) POUT(.10)<br>/NOORIGIN<br>/DEPENDENT Risk_Help<br>/METHOD=ENTER Party0<br>/METHOD=ENTER GenderCC RaceCC SES0<br>/METHOD=ENTER MRN0<br>/METHOD=ENTER MRN0xRace MRN0xSES0 MRN0xGender MRN0xParty0.
Resources	Processor Time	00:00:00.02
	Elapsed Time	00:00:00.02

Notes

	Memory Required	45472 bytes
	Additional Memory Required for Residual Plots	0 bytes

Variables Entered/Removed[a]

Model	Variables Entered	Variables Removed	Method
1	Party0[b]	.	Enter
2	SES0, GenderCC, RaceCC[b]	.	Enter
3	MRN0[b]	.	Enter
4	MRN0xSES0, MRN0xGender, MRN0xParty0, MRN0xRace[b]	.	Enter

a. Dependent Variable: Risk_Help

b. All requested variables entered.

Model Summary

Model	R	R Square	Adjusted R Square	Std. Error of the Estimate	Change Statistics		
					R Square Change	F Change	df1
1	.169[a]	.028	.024	1.48842	.028	6.920	1
2	.279[b]	.078	.062	1.45925	.050	4.177	3
3	.280[c]	.079	.059	1.46201	.000	.119	1
4	.308[d]	.095	.059	1.46148	.017	1.043	4

Model Summary

Model	Change Statistics	
	df2	Sig. F Change
1	236	.009
2	233	.007
3	232	.730
4	228	.386

a. Predictors: (Constant), Party0

b. Predictors: (Constant), Party0, SES0, GenderCC, RaceCC

c. Predictors: (Constant), Party0, SES0, GenderCC, RaceCC, MRN0

d. Predictors: (Constant), Party0, SES0, GenderCC, RaceCC, MRN0, MRN0xSES0, MRN0xGender, MRN0xParty0, MRN0xRace

ANOVA[a]

Model		Sum of Squares	df	Mean Square	F	Sig.
1	Regression	15.331	1	15.331	6.920	.009[b]
	Residual	522.832	236	2.215		
	Total	538.163	237			
2	Regression	42.011	4	10.503	4.932	.001[c]
	Residual	496.151	233	2.129		
	Total	538.163	237			
3	Regression	42.267	5	8.453	3.955	.002[d]
	Residual	495.896	232	2.137		
	Total	538.163	237			
4	Regression	51.174	9	5.686	2.662	.006[e]
	Residual	486.989	228	2.136		
	Total	538.163	237			

a. Dependent Variable: Risk_Help

b. Predictors: (Constant), Party0

c. Predictors: (Constant), Party0, SES0, GenderCC, RaceCC

d. Predictors: (Constant), Party0, SES0, GenderCC, RaceCC, MRN0

e. Predictors: (Constant), Party0, SES0, GenderCC, RaceCC, MRN0, MRN0xSES0, MRN0xGender, MRN0xParty0, MRN0xRace

Coefficients[a]

Model		Unstandardized Coefficients B	Std. Error	Standardized Coefficients Beta	t	Sig.
1	(Constant)	3.801	.096		39.400	.000
	Party0	-.183	.070	-.169	-2.631	.009
2	(Constant)	3.936	.110		35.719	.000
	Party0	-.106	.073	-.097	-1.442	.151
	GenderCC	-.126	.098	-.084	-1.290	.198
	RaceCC	-.279	.114	-.161	-2.452	.015
	SES0	-.251	.117	-.135	-2.136	.034
3	(Constant)	3.935	.111		35.596	.000
	Party0	-.117	.081	-.108	-1.451	.148
	GenderCC	-.139	.104	-.092	-1.326	.186
	RaceCC	-.275	.114	-.159	-2.404	.017
	SES0	-.255	.118	-.137	-2.157	.032
	MRN0	.046	.134	.027	.346	.730
4	(Constant)	3.949	.127		31.013	.000
	Party0	-.087	.083	-.080	-1.052	.294
	GenderCC	-.158	.107	-.105	-1.474	.142
	RaceCC	-.283	.117	-.163	-2.424	.016
	SES0	-.274	.119	-.147	-2.294	.023
	MRN0	.143	.151	.082	.950	.343
	MRN0xRace	-.191	.131	-.110	-1.461	.146
	MRN0xSES0	.149	.126	.077	1.184	.238
	MRN0xGender	.013	.127	.007	.102	.919
	MRN0xParty0	-.037	.085	-.030	-.435	.664

Coefficients[a]

Model		Correlations Zero-order	Partial	Part
1	(Constant)			
	Party0	-.169	-.169	-.169
2	(Constant)			
	Party0	-.169	-.094	-.091
	GenderCC	-.119	-.084	-.081
	RaceCC	-.196	-.159	-.154
	SES0	-.149	-.139	-.134
3	(Constant)			
	Party0	-.169	-.095	-.091
	GenderCC	-.119	-.087	-.084
	RaceCC	-.196	-.156	-.151
	SES0	-.149	-.140	-.136
	MRN0	-.086	.023	.022
4	(Constant)			
	Party0	-.169	-.069	-.066
	GenderCC	-.119	-.097	-.093
	RaceCC	-.196	-.159	-.153
	SES0	-.149	-.150	-.145
	MRN0	-.086	.063	.060
	MRN0xRace	-.121	-.096	-.092
	MRN0xSES0	.087	.078	.075
	MRN0xGender	-.013	.007	.006
	MRN0xParty0	-.078	-.029	-.027

a. Dependent Variable: Risk_Help

Excluded Variables[a]

Model		Beta In	t	Sig.	Partial Correlation	Collinearity Statistics Tolerance
1	GenderCC	-.084[b]	-1.269	.206	-.082	.942
	RaceCC	-.161[b]	-2.437	.016	-.157	.919
	SES0	-.142[b]	-2.229	.027	-.144	.998
	MRN0	-.010[b]	-.141	.888	-.009	.784
	MRN0xRace	-.072[b]	-1.065	.288	-.069	.888
	MRN0xSES0	.069[b]	1.069	.286	.070	.988
	MRN0xGender	.006[b]	.098	.922	.006	.986
	MRN0xParty0	-.053[b]	-.821	.412	-.053	.977
2	MRN0	.027[c]	.346	.730	.023	.671
	MRN0xRace	-.093[c]	-1.386	.167	-.091	.867
	MRN0xSES0	.069[c]	1.092	.276	.072	.984
	MRN0xGender	-.011[c]	-.178	.859	-.012	.968
	MRN0xParty0	-.053[c]	-.825	.410	-.054	.949
3	MRN0xRace	-.117[d]	-1.622	.106	-.106	.753
	MRN0xSES0	.076[d]	1.172	.242	.077	.952
	MRN0xGender	-.015[d]	-.237	.813	-.016	.942
	MRN0xParty0	-.051[d]	-.781	.435	-.051	.929

a. Dependent Variable: Risk_Help

b. Predictors in the Model: (Constant), Party0

c. Predictors in the Model: (Constant), Party0, SES0, GenderCC, RaceCC

d. Predictors in the Model: (Constant), Party0, SES0, GenderCC, RaceCC, MRN0

```
 *Ideology instead of party

**Regression Analyses**

REGRESSION
  /MISSING LISTWISE
  /STATISTICS COEFF OUTS R ANOVA CHANGE ZPP
  /CRITERIA=PIN(.05) POUT(.10)
  /NOORIGIN
  /DEPENDENT Concern_Tot
```

```
/METHOD=ENTER Ideology0
/METHOD=ENTER GenderCC RaceCC SES0
/METHOD=ENTER MRN0
/METHOD=ENTER MRN0xRace MRN0xSES0 MRN0xGender MRN0xIdeology0.
```

Regression

Notes

Output Created		15-DEC-2021 13:07:57
Comments		
Input	Data	C: \Users\njs5478\Dropbox\HM and COVID\0. Revise and Resubmit\2. R and R Data\Study 1b\Study1b_Data.sav
	Active Dataset	DataSet1
	Filter	<none>
	Weight	<none>
	Split File	<none>
	N of Rows in Working Data File	241
Missing Value Handling	Definition of Missing	User-defined missing values are treated as missing.
	Cases Used	Statistics are based on cases with no missing values for any variable used.
Syntax		REGRESSION /MISSING LISTWISE /STATISTICS COEFF OUTS R ANOVA CHANGE ZPP /CRITERIA=PIN(.05) POUT(.10) /NOORIGIN /DEPENDENT Concern_Tot /METHOD=ENTER Ideology0 /METHOD=ENTER GenderCC RaceCC SES0 /METHOD=ENTER MRN0 /METHOD=ENTER MRN0xRace MRN0xSES0 MRN0xGender MRN0xIdeology0.

Notes

Resources	Processor Time	00:00:00.05
	Elapsed Time	00:00:00.03
	Memory Required	45472 bytes
	Additional Memory Required for Residual Plots	0 bytes

Variables Entered/Removed[a]

Model	Variables Entered	Variables Removed	Method
1	Ideology0[b]	.	Enter
2	SES0, GenderCC, RaceCC[b]	.	Enter
3	MRN0[b]	.	Enter
4	MRN0xSES0, MRN0xGender, MRN0xIdeology0, MRN0xRace[b]	.	Enter

a. Dependent Variable: Concern_Tot

b. All requested variables entered.

Model Summary

Model	R	R Square	Adjusted R Square	Std. Error of the Estimate	Change Statistics		
					R Square Change	F Change	df1
1	.498[a]	.248	.245	1.20800	.248	77.948	1
2	.512[b]	.262	.249	1.20467	.014	1.436	3
3	.524[c]	.274	.259	1.19705	.012	3.973	1
4	.551[d]	.304	.276	1.18274	.029	2.412	4

Model Summary

Model	Change Statistics df2	Change Statistics Sig. F Change
1	236	.000
2	233	.233
3	232	.047
4	228	.050

a. Predictors: (Constant), Ideology0

b. Predictors: (Constant), Ideology0, SES0, GenderCC, RaceCC

c. Predictors: (Constant), Ideology0, SES0, GenderCC, RaceCC, MRN0

d. Predictors: (Constant), Ideology0, SES0, GenderCC, RaceCC, MRN0, MRN0xSES0, MRN0xGender, MRN0xIdeology0, MRN0xRace

ANOVA[a]

Model		Sum of Squares	df	Mean Square	F	Sig.
1	Regression	113.747	1	113.747	77.948	.000[b]
	Residual	344.386	236	1.459		
	Total	458.133	237			
2	Regression	119.999	4	30.000	20.672	.000[c]
	Residual	338.134	233	1.451		
	Total	458.133	237			
3	Regression	125.693	5	25.139	17.543	.000[d]
	Residual	332.440	232	1.433		
	Total	458.133	237			
4	Regression	139.189	9	15.465	11.056	.000[e]
	Residual	318.945	228	1.399		
	Total	458.133	237			

a. Dependent Variable: Concern_Tot

b. Predictors: (Constant), Ideology0

c. Predictors: (Constant), Ideology0, SES0, GenderCC, RaceCC

d. Predictors: (Constant), Ideology0, SES0, GenderCC, RaceCC, MRN0

e. Predictors: (Constant), Ideology0, SES0, GenderCC, RaceCC, MRN0, MRN0xSES0, MRN0xGender, MRN0xIdeology0, MRN0xRace

Coefficients[a]

Model		Unstandardized Coefficients B	Std. Error	Standardized Coefficients Beta	t	Sig.
1	(Constant)	4.336	.078		55.379	.000
	Ideology0	-.436	.049	-.498	-8.829	.000
2	(Constant)	4.344	.091		47.574	.000
	Ideology0	-.406	.054	-.464	-7.503	.000
	GenderCC	-.168	.081	-.121	-2.074	.039
	RaceCC	-.018	.095	-.011	-.188	.851
	SES0	.013	.097	.007	.132	.895
3	(Constant)	4.358	.091		47.888	.000
	Ideology0	-.343	.062	-.392	-5.502	.000
	GenderCC	-.111	.085	-.080	-1.299	.195
	RaceCC	-.047	.096	-.029	-.491	.624
	SES0	.033	.097	.019	.339	.735
	MRN0	-.230	.115	-.143	-1.993	.047
4	(Constant)	4.333	.102		42.312	.000
	Ideology0	-.308	.063	-.352	-4.849	.000
	GenderCC	-.158	.087	-.114	-1.823	.070
	RaceCC	-.080	.098	-.050	-.817	.415
	SES0	.028	.097	.016	.286	.775
	MRN0	-.085	.127	-.053	-.673	.502
	MRN0xRace	-.236	.110	-.147	-2.137	.034
	MRN0xSES0	.217	.102	.122	2.123	.035
	MRN0xGender	-.005	.103	-.003	-.050	.960
	MRN0xIdeology0	.046	.055	.052	.836	.404

Coefficients[a]

Model		Correlations Zero-order	Partial	Part
1	(Constant)			
	Ideology0	-.498	-.498	-.498
2	(Constant)			
	Ideology0	-.498	-.441	-.422
	GenderCC	-.242	-.135	-.117
	RaceCC	-.166	-.012	-.011
	SES0	-.026	.009	.007
3	(Constant)			
	Ideology0	-.498	-.340	-.308
	GenderCC	-.242	-.085	-.073
	RaceCC	-.166	-.032	-.027
	SES0	-.026	.022	.019
	MRN0	-.387	-.130	-.111
4	(Constant)			
	Ideology0	-.498	-.306	-.268
	GenderCC	-.242	-.120	-.101
	RaceCC	-.166	-.054	-.045
	SES0	-.026	.019	.016
	MRN0	-.387	-.045	-.037
	MRN0xRace	-.325	-.140	-.118
	MRN0xSES0	.197	.139	.117
	MRN0xGender	-.077	-.003	-.003
	MRN0xIdeology0	.010	.055	.046

a. Dependent Variable: Concern_Tot

Excluded Variables[a]

Model		Beta In	t	Sig.	Partial Correlation	Collinearity Statistics Tolerance
1	GenderCC	-.120[b]	-2.072	.039	-.134	.932
	RaceCC	-.004[b]	-.069	.945	-.004	.893
	SES0	.003[b]	.050	.960	.003	.997
	MRN0	-.167[b]	-2.517	.013	-.162	.709
	MRN0xRace	-.151[b]	-2.482	.014	-.160	.842
	MRN0xSES0	.135[b]	2.386	.018	.154	.983
	MRN0xGender	-.031[b]	-.545	.586	-.036	.991
	MRN0xIdeology0	.026[b]	.461	.645	.030	.999
2	MRN0	-.143[c]	-1.993	.047	-.130	.606
	MRN0xRace	-.153[c]	-2.482	.014	-.161	.812
	MRN0xSES0	.143[c]	2.534	.012	.164	.979
	MRN0xGender	-.037[c]	-.641	.522	-.042	.973
	MRN0xIdeology0	.038[c]	.661	.509	.043	.961
3	MRN0xRace	-.127[d]	-1.961	.051	-.128	.734
	MRN0xSES0	.127[d]	2.238	.026	.146	.951
	MRN0xGender	-.018[d]	-.315	.753	-.021	.946
	MRN0xIdeology0	.020[d]	.340	.734	.022	.934

a. Dependent Variable: Concern_Tot

b. Predictors in the Model: (Constant), Ideology0

c. Predictors in the Model: (Constant), Ideology0, SES0, GenderCC, RaceCC

d. Predictors in the Model: (Constant), Ideology0, SES0, GenderCC, RaceCC, MRN0

```
REGRESSION
 /MISSING LISTWISE
 /STATISTICS COEFF OUTS R ANOVA CHANGE ZPP
 /CRITERIA=PIN(.05) POUT(.10)
 /NOORIGIN
 /DEPENDENT Finance_Tot
 /METHOD=ENTER Ideology0
 /METHOD=ENTER GenderCC RaceCC SES0
 /METHOD=ENTER MRN0
 /METHOD=ENTER MRN0xRace MRN0xSES0 MRN0xGender MRN0xIdeology0.
```

Regression

Notes

Output Created		15-DEC-2021 13:07:57
Comments		
Input	Data	C:\Users\njs5478\Dropbox\HM and COVID\0. Revise and Resubmit\2. R and R Data\Study 1b\Study1b_Data.sav
	Active Dataset	DataSet1
	Filter	<none>
	Weight	<none>
	Split File	<none>
	N of Rows in Working Data File	241
Missing Value Handling	Definition of Missing	User-defined missing values are treated as missing.
	Cases Used	Statistics are based on cases with no missing values for any variable used.
Syntax		REGRESSION /MISSING LISTWISE /STATISTICS COEFF OUTS R ANOVA CHANGE ZPP /CRITERIA=PIN(.05) POUT(.10) /NOORIGIN /DEPENDENT Finance_Tot /METHOD=ENTER Ideology0 /METHOD=ENTER GenderCC RaceCC SES0 /METHOD=ENTER MRN0 /METHOD=ENTER MRN0xRace MRN0xSES0 MRN0xGender MRN0xIdeology0.
Resources	Processor Time	00:00:00.05
	Elapsed Time	00:00:00.03

Notes

	Memory Required	45472 bytes
	Additional Memory Required for Residual Plots	0 bytes

Variables Entered/Removed[a]

Model	Variables Entered	Variables Removed	Method
1	Ideology0[b]	.	Enter
2	SES0, GenderCC, RaceCC[b]	.	Enter
3	MRN0[b]	.	Enter
4	MRN0xSES0, MRN0xGender, MRN0xIdeology0, MRN0xRace[b]	.	Enter

a. Dependent Variable: Finance_Tot

b. All requested variables entered.

Model Summary

Model	R	R Square	Adjusted R Square	Std. Error of the Estimate	Change Statistics		
					R Square Change	F Change	df1
1	.141[a]	.020	.016	1.52230	.020	4.777	1
2	.222[b]	.049	.033	1.50881	.030	2.414	3
3	.230[c]	.053	.033	1.50913	.004	.899	1
4	.304[d]	.093	.057	1.49009	.040	2.492	4

Model Summary

Model	Change Statistics	
	df2	Sig. F Change
1	236	.030
2	233	.067
3	232	.344
4	228	.044

a. Predictors: (Constant), Ideology0

b. Predictors: (Constant), Ideology0, SES0, GenderCC, RaceCC

c. Predictors: (Constant), Ideology0, SES0, GenderCC, RaceCC, MRN0

d. Predictors: (Constant), Ideology0, SES0, GenderCC, RaceCC, MRN0, MRN0xSES0, MRN0xGender, MRN0xIdeology0, MRN0xRace

ANOVA[a]

Model		Sum of Squares	df	Mean Square	F	Sig.
1	Regression	11.071	1	11.071	4.777	.030[b]
	Residual	546.909	236	2.317		
	Total	557.980	237			
2	Regression	27.556	4	6.889	3.026	.018[c]
	Residual	530.423	233	2.276		
	Total	557.980	237			
3	Regression	29.604	5	5.921	2.600	.026[d]
	Residual	528.376	232	2.277		
	Total	557.980	237			
4	Regression	51.734	9	5.748	2.589	.007[e]
	Residual	506.246	228	2.220		
	Total	557.980	237			

a. Dependent Variable: Finance_Tot

b. Predictors: (Constant), Ideology0

c. Predictors: (Constant), Ideology0, SES0, GenderCC, RaceCC

d. Predictors: (Constant), Ideology0, SES0, GenderCC, RaceCC, MRN0

e. Predictors: (Constant), Ideology0, SES0, GenderCC, RaceCC, MRN0, MRN0xSES0, MRN0xGender, MRN0xIdeology0, MRN0xRace

Coefficients[a]

Model		Unstandardized Coefficients B	Std. Error	Standardized Coefficients Beta	t	Sig.
1	(Constant)	3.900	.099		39.523	.000
	Ideology0	-.136	.062	-.141	-2.186	.030
2	(Constant)	3.908	.114		34.172	.000
	Ideology0	-.096	.068	-.099	-1.415	.158
	GenderCC	-.176	.102	-.115	-1.735	.084
	RaceCC	-.024	.119	-.014	-.203	.839
	SES0	-.241	.121	-.127	-1.983	.049
3	(Constant)	3.916	.115		34.136	.000
	Ideology0	-.058	.079	-.060	-.740	.460
	GenderCC	-.142	.108	-.093	-1.318	.189
	RaceCC	-.042	.121	-.024	-.346	.730
	SES0	-.229	.122	-.121	-1.874	.062
	MRN0	-.138	.145	-.078	-.948	.344
4	(Constant)	3.810	.129		29.531	.000
	Ideology0	-.042	.080	-.044	-.528	.598
	GenderCC	-.174	.109	-.114	-1.595	.112
	RaceCC	-.027	.123	-.015	-.221	.825
	SES0	-.239	.122	-.126	-1.969	.050
	MRN0	-.060	.160	-.034	-.378	.706
	MRN0xRace	-.094	.139	-.053	-.674	.501
	MRN0xSES0	.369	.129	.187	2.860	.005
	MRN0xGender	.168	.130	.086	1.294	.197
	MRN0xIdeology0	.015	.069	.016	.221	.825

Coefficients[a]

Model		Correlations Zero-order	Partial	Part
1	(Constant)			
	Ideology0	-.141	-.141	-.141
2	(Constant)			
	Ideology0	-.141	-.092	-.090
	GenderCC	-.148	-.113	-.111
	RaceCC	-.055	-.013	-.013
	SES0	-.139	-.129	-.127
3	(Constant)			
	Ideology0	-.141	-.049	-.047
	GenderCC	-.148	-.086	-.084
	RaceCC	-.055	-.023	-.022
	SES0	-.139	-.122	-.120
	MRN0	-.165	-.062	-.061
4	(Constant)			
	Ideology0	-.141	-.035	-.033
	GenderCC	-.148	-.105	-.101
	RaceCC	-.055	-.015	-.014
	SES0	-.139	-.129	-.124
	MRN0	-.165	-.025	-.024
	MRN0xRace	-.099	-.045	-.042
	MRN0xSES0	.194	.186	.180
	MRN0xGender	.041	.085	.082
	MRN0xIdeology0	.045	.015	.014

a. Dependent Variable: Finance_Tot

Excluded Variables[a]

Model		Beta In	t	Sig.	Partial Correlation	Collinearity Statistics Tolerance
1	GenderCC	-.119[b]	-1.795	.074	-.116	.932
	RaceCC	-.010[b]	-.140	.889	-.009	.893
	SES0	-.131[b]	-2.050	.042	-.133	.997
	MRN0	-.125[b]	-1.639	.102	-.106	.709
	MRN0xRace	-.051[b]	-.720	.472	-.047	.842
	MRN0xSES0	.178[b]	2.782	.006	.179	.983
	MRN0xGender	.055[b]	.842	.400	.055	.991
	MRN0xIdeology0	.050[b]	.773	.440	.050	.999
2	MRN0	-.078[c]	-.948	.344	-.062	.606
	MRN0xRace	-.053[c]	-.749	.454	-.049	.812
	MRN0xSES0	.186[c]	2.931	.004	.189	.979
	MRN0xGender	.055[c]	.849	.397	.056	.973
	MRN0xIdeology0	.046[c]	.699	.485	.046	.961
3	MRN0xRace	-.036[d]	-.479	.632	-.032	.734
	MRN0xSES0	.181[d]	2.805	.005	.181	.951
	MRN0xGender	.067[d]	1.023	.307	.067	.946
	MRN0xIdeology0	.036[d]	.549	.583	.036	.934

a. Dependent Variable: Finance_Tot

b. Predictors in the Model: (Constant), Ideology0

c. Predictors in the Model: (Constant), Ideology0, SES0, GenderCC, RaceCC

d. Predictors in the Model: (Constant), Ideology0, SES0, GenderCC, RaceCC, MRN0

```
REGRESSION
 /MISSING LISTWISE
 /STATISTICS COEFF OUTS R ANOVA CHANGE ZPP
 /CRITERIA=PIN(.05) POUT(.10)
 /NOORIGIN
 /DEPENDENT Resource_Tot
 /METHOD=ENTER Ideology0
 /METHOD=ENTER GenderCC RaceCC SES0
 /METHOD=ENTER MRN0
 /METHOD=ENTER MRN0xRace MRN0xSES0 MRN0xGender MRN0xIdeology0.
```

Regression

Notes

Output Created		15-DEC-2021 13:07:57
Comments		
Input	Data	C: \Users\njs5478\Dropbox\HM and COVID\0. Revise and Resubmit\2. R and R Data\Study 1b\Study1b_Data.sav
	Active Dataset	DataSet1
	Filter	<none>
	Weight	<none>
	Split File	<none>
	N of Rows in Working Data File	241
Missing Value Handling	Definition of Missing	User-defined missing values are treated as missing.
	Cases Used	Statistics are based on cases with no missing values for any variable used.
Syntax		REGRESSION<br>/MISSING LISTWISE<br>/STATISTICS COEFF OUTS R ANOVA CHANGE ZPP<br>/CRITERIA=PIN(.05) POUT(.10)<br>/NOORIGIN<br>/DEPENDENT Resource_Tot<br>/METHOD=ENTER Ideology0<br>/METHOD=ENTER GenderCC RaceCC SES0<br>/METHOD=ENTER MRN0<br>/METHOD=ENTER MRN0xRace MRN0xSES0 MRN0xGender MRN0xIdeology0.
Resources	Processor Time	00:00:00.03
	Elapsed Time	00:00:00.03

Notes

	Memory Required	45472 bytes
	Additional Memory Required for Residual Plots	0 bytes

Variables Entered/Removed[a]

Model	Variables Entered	Variables Removed	Method
1	Ideology0[b]	.	Enter
2	SES0, GenderCC, RaceCC[b]	.	Enter
3	MRN0[b]	.	Enter
4	MRN0xSES0, MRN0xGender, MRN0xIdeology0, MRN0xRace[b]	.	Enter

a. Dependent Variable: Resource_Tot

b. All requested variables entered.

Model Summary

Model	R	R Square	Adjusted R Square	Std. Error of the Estimate	Change Statistics		
					R Square Change	F Change	df1
1	.111[a]	.012	.008	1.38337	.012	2.968	1
2	.150[b]	.023	.006	1.38512	.010	.802	3
3	.154[c]	.024	.003	1.38726	.001	.280	1
4	.262[d]	.069	.032	1.36686	.045	2.744	4

Model Summary

Model	Change Statistics	
	df2	Sig. F Change
1	236	.086
2	233	.494
3	232	.597
4	228	.029

a. Predictors: (Constant), Ideology0

b. Predictors: (Constant), Ideology0, SES0, GenderCC, RaceCC

c. Predictors: (Constant), Ideology0, SES0, GenderCC, RaceCC, MRN0

d. Predictors: (Constant), Ideology0, SES0, GenderCC, RaceCC, MRN0, MRN0xSES0, MRN0xGender, MRN0xIdeology0, MRN0xRace

ANOVA[a]

Model		Sum of Squares	df	Mean Square	F	Sig.
1	Regression	5.679	1	5.679	2.968	.086[b]
	Residual	451.638	236	1.914		
	Total	457.317	237			
2	Regression	10.296	4	2.574	1.342	.255[c]
	Residual	447.021	233	1.919		
	Total	457.317	237			
3	Regression	10.834	5	2.167	1.126	.347[d]
	Residual	446.483	232	1.924		
	Total	457.317	237			
4	Regression	31.341	9	3.482	1.864	.058[e]
	Residual	425.976	228	1.868		
	Total	457.317	237			

a. Dependent Variable: Resource_Tot

b. Predictors: (Constant), Ideology0

c. Predictors: (Constant), Ideology0, SES0, GenderCC, RaceCC

d. Predictors: (Constant), Ideology0, SES0, GenderCC, RaceCC, MRN0

e. Predictors: (Constant), Ideology0, SES0, GenderCC, RaceCC, MRN0, MRN0xSES0, MRN0xGender, MRN0xIdeology0, MRN0xRace

Coefficients[a]

Model		Unstandardized Coefficients B	Unstandardized Coefficients Std. Error	Standardized Coefficients Beta	t	Sig.
1	(Constant)	3.617	.090		40.339	.000
	Ideology0	-.097	.057	-.111	-1.723	.086
2	(Constant)	3.654	.105		34.803	.000
	Ideology0	-.063	.062	-.072	-1.017	.310
	GenderCC	-.110	.093	-.080	-1.183	.238
	RaceCC	-.077	.110	-.048	-.705	.481
	SES0	-.080	.112	-.046	-.716	.475
3	(Constant)	3.658	.105		34.686	.000
	Ideology0	-.044	.072	-.050	-.608	.544
	GenderCC	-.093	.099	-.067	-.936	.350
	RaceCC	-.086	.111	-.054	-.777	.438
	SES0	-.074	.112	-.043	-.656	.512
	MRN0	-.071	.134	-.044	-.529	.597
4	(Constant)	3.542	.118		29.929	.000
	Ideology0	-.006	.073	-.006	-.076	.940
	GenderCC	-.131	.100	-.094	-1.303	.194
	RaceCC	-.115	.113	-.072	-1.019	.310
	SES0	-.080	.112	-.046	-.715	.476
	MRN0	.061	.147	.038	.418	.677
	MRN0xRace	-.302	.127	-.188	-2.369	.019
	MRN0xSES0	.181	.118	.102	1.533	.127
	MRN0xGender	.200	.119	.113	1.677	.095
	MRN0xIdeology0	.073	.063	.083	1.158	.248

Coefficients[a]

Model		Correlations Zero-order	Partial	Part
1	(Constant)			
	Ideology0	-.111	-.111	-.111
2	(Constant)			
	Ideology0	-.111	-.066	-.066
	GenderCC	-.102	-.077	-.077
	RaceCC	-.076	-.046	-.046
	SES0	-.056	-.047	-.046
3	(Constant)			
	Ideology0	-.111	-.040	-.039
	GenderCC	-.102	-.061	-.061
	RaceCC	-.076	-.051	-.050
	SES0	-.056	-.043	-.043
	MRN0	-.107	-.035	-.034
4	(Constant)			
	Ideology0	-.111	-.005	-.005
	GenderCC	-.102	-.086	-.083
	RaceCC	-.076	-.067	-.065
	SES0	-.056	-.047	-.046
	MRN0	-.107	.028	.027
	MRN0xRace	-.152	-.155	-.151
	MRN0xSES0	.111	.101	.098
	MRN0xGender	.087	.110	.107
	MRN0xIdeology0	.052	.076	.074

a. Dependent Variable: Resource_Tot

Excluded Variables[a]

Model		Beta In	t	Sig.	Partial Correlation	Collinearity Statistics Tolerance
1	GenderCC	-.079[b]	-1.175	.241	-.076	.932
	RaceCC	-.045[b]	-.653	.515	-.043	.893
	SES0	-.050[b]	-.774	.440	-.050	.997
	MRN0	-.066[b]	-.858	.392	-.056	.709
	MRN0xRace	-.128[b]	-1.826	.069	-.118	.842
	MRN0xSES0	.098[b]	1.509	.133	.098	.983
	MRN0xGender	.098[b]	1.512	.132	.098	.991
	MRN0xIdeology0	.056[b]	.864	.388	.056	.999
2	MRN0	-.044[c]	-.529	.597	-.035	.606
	MRN0xRace	-.140[c]	-1.960	.051	-.128	.812
	MRN0xSES0	.103[c]	1.576	.116	.103	.979
	MRN0xGender	.093[c]	1.424	.156	.093	.973
	MRN0xIdeology0	.064[c]	.968	.334	.063	.961
3	MRN0xRace	-.142[d]	-1.884	.061	-.123	.734
	MRN0xSES0	.100[d]	1.505	.134	.099	.951
	MRN0xGender	.102[d]	1.534	.126	.100	.946
	MRN0xIdeology0	.060[d]	.891	.374	.059	.934

a. Dependent Variable: Resource_Tot

b. Predictors in the Model: (Constant), Ideology0

c. Predictors in the Model: (Constant), Ideology0, SES0, GenderCC, RaceCC

d. Predictors in the Model: (Constant), Ideology0, SES0, GenderCC, RaceCC, MRN0

```
REGRESSION
 /MISSING LISTWISE
 /STATISTICS COEFF OUTS R ANOVA CHANGE ZPP
 /CRITERIA=PIN(.05) POUT(.10)
 /NOORIGIN
 /DEPENDENT Psychology_Tot
 /METHOD=ENTER Ideology0
 /METHOD=ENTER GenderCC RaceCC SES0
 /METHOD=ENTER MRN0
 /METHOD=ENTER MRN0xRace MRN0xSES0 MRN0xGender MRN0xIdeology0.
```

Regression

Notes

Output Created		15-DEC-2021 13:07:57
Comments		
Input	Data	C: \Users\njs5478\Dropbox\HM and COVID\0. Revise and Resubmit\2. R and R Data\Study 1b\Study1b_Data.sav
	Active Dataset	DataSet1
	Filter	<none>
	Weight	<none>
	Split File	<none>
	N of Rows in Working Data File	241
Missing Value Handling	Definition of Missing	User-defined missing values are treated as missing.
	Cases Used	Statistics are based on cases with no missing values for any variable used.
Syntax		REGRESSION /MISSING LISTWISE /STATISTICS COEFF OUTS R ANOVA CHANGE ZPP /CRITERIA=PIN(.05) POUT(.10) /NOORIGIN /DEPENDENT Psychology_Tot /METHOD=ENTER Ideology0 /METHOD=ENTER GenderCC RaceCC SES0 /METHOD=ENTER MRN0 /METHOD=ENTER MRN0xRace MRN0xSES0 MRN0xGender MRN0xIdeology0.
Resources	Processor Time	00:00:00.02
	Elapsed Time	00:00:00.03

Notes

	Memory Required	45472 bytes
	Additional Memory Required for Residual Plots	0 bytes

Variables Entered/Removed[a]

Model	Variables Entered	Variables Removed	Method
1	Ideology0[b]	.	Enter
2	SES0, GenderCC, RaceCC[b]	.	Enter
3	MRN0[b]	.	Enter
4	MRN0xSES0, MRN0xGender, MRN0xIdeology0, MRN0xRace[b]	.	Enter

a. Dependent Variable: Psychology_Tot

b. All requested variables entered.

Model Summary

Model	R	R Square	Adjusted R Square	Std. Error of the Estimate	Change Statistics		
					R Square Change	F Change	df1
1	.271[a]	.073	.070	1.43644	.073	18.708	1
2	.298[b]	.089	.073	1.43383	.015	1.287	3
3	.313[c]	.098	.079	1.42940	.010	2.445	1
4	.330[d]	.109	.073	1.43341	.011	.676	4

Model Summary

Model	Change Statistics	
	df2	Sig. F Change
1	236	.000
2	233	.280
3	232	.119
4	228	.609

a. Predictors: (Constant), Ideology0

b. Predictors: (Constant), Ideology0, SES0, GenderCC, RaceCC

c. Predictors: (Constant), Ideology0, SES0, GenderCC, RaceCC, MRN0

d. Predictors: (Constant), Ideology0, SES0, GenderCC, RaceCC, MRN0, MRN0xSES0, MRN0xGender, MRN0xIdeology0, MRN0xRace

ANOVA[a]

Model		Sum of Squares	df	Mean Square	F	Sig.
1	Regression	38.601	1	38.601	18.708	.000[b]
	Residual	486.953	236	2.063		
	Total	525.554	237			
2	Regression	46.539	4	11.635	5.659	.000[c]
	Residual	479.015	233	2.056		
	Total	525.554	237			
3	Regression	51.535	5	10.307	5.045	.000[d]
	Residual	474.019	232	2.043		
	Total	525.554	237			
4	Regression	57.090	9	6.343	3.087	.002[e]
	Residual	468.464	228	2.055		
	Total	525.554	237			

a. Dependent Variable: Psychology_Tot

b. Predictors: (Constant), Ideology0

c. Predictors: (Constant), Ideology0, SES0, GenderCC, RaceCC

d. Predictors: (Constant), Ideology0, SES0, GenderCC, RaceCC, MRN0

e. Predictors: (Constant), Ideology0, SES0, GenderCC, RaceCC, MRN0, MRN0xSES0, MRN0xGender, MRN0xIdeology0, MRN0xRace

Coefficients[a]

Model		Unstandardized Coefficients B	Std. Error	Standardized Coefficients Beta	t	Sig.
1	(Constant)	4.663	.093		50.077	.000
	Ideology0	-.254	.059	-.271	-4.325	.000
2	(Constant)	4.674	.109		43.009	.000
	Ideology0	-.222	.064	-.237	-3.445	.001
	GenderCC	-.182	.096	-.123	-1.891	.060
	RaceCC	-.024	.113	-.014	-.216	.829
	SES0	.069	.115	.037	.598	.550
3	(Constant)	4.687	.109		43.134	.000
	Ideology0	-.163	.074	-.174	-2.188	.030
	GenderCC	-.129	.102	-.087	-1.264	.208
	RaceCC	-.052	.114	-.030	-.453	.651
	SES0	.088	.116	.048	.759	.449
	MRN0	-.215	.138	-.125	-1.564	.119
4	(Constant)	4.635	.124		37.349	.000
	Ideology0	-.146	.077	-.155	-1.895	.059
	GenderCC	-.152	.105	-.102	-1.444	.150
	RaceCC	-.088	.119	-.051	-.742	.459
	SES0	.097	.117	.053	.829	.408
	MRN0	-.138	.154	-.080	-.899	.370
	MRN0xRace	-.169	.134	-.098	-1.265	.207
	MRN0xSES0	-.032	.124	-.017	-.257	.797
	MRN0xGender	.048	.125	.025	.383	.702
	MRN0xIdeology0	.084	.066	.089	1.260	.209

Coefficients[a]

Model		Correlations Zero-order	Partial	Part
1	(Constant)			
	Ideology0	-.271	-.271	-.271
2	(Constant)			
	Ideology0	-.271	-.220	-.215
	GenderCC	-.183	-.123	-.118
	RaceCC	-.094	-.014	-.013
	SES0	.017	.039	.037
3	(Constant)			
	Ideology0	-.271	-.142	-.136
	GenderCC	-.183	-.083	-.079
	RaceCC	-.094	-.030	-.028
	SES0	.017	.050	.047
	MRN0	-.251	-.102	-.097
4	(Constant)			
	Ideology0	-.271	-.125	-.118
	GenderCC	-.183	-.095	-.090
	RaceCC	-.094	-.049	-.046
	SES0	.017	.055	.052
	MRN0	-.251	-.059	-.056
	MRN0xRace	-.183	-.083	-.079
	MRN0xSES0	.035	-.017	-.016
	MRN0xGender	-.002	.025	.024
	MRN0xIdeology0	.051	.083	.079

a. Dependent Variable: Psychology_Tot

Excluded Variables[a]

Model		Beta In	t	Sig.	Partial Correlation	Collinearity Statistics Tolerance
1	GenderCC	-.121[b]	-1.867	.063	-.121	.932
	RaceCC	-.007[b]	-.099	.921	-.006	.893
	SES0	.033[b]	.522	.602	.034	.997
	MRN0	-.148[b]	-1.998	.047	-.129	.709
	MRN0xRace	-.089[b]	-1.303	.194	-.085	.842
	MRN0xSES0	-.001[b]	-.011	.991	-.001	.983
	MRN0xGender	.023[b]	.369	.712	.024	.991
	MRN0xIdeology0	.060[b]	.951	.342	.062	.999
2	MRN0	-.125[c]	-1.564	.119	-.102	.606
	MRN0xRace	-.089[c]	-1.279	.202	-.084	.812
	MRN0xSES0	.007[c]	.107	.915	.007	.979
	MRN0xGender	.017[c]	.270	.787	.018	.973
	MRN0xIdeology0	.077[c]	1.211	.227	.079	.961
3	MRN0xRace	-.061[d]	-.838	.403	-.055	.734
	MRN0xSES0	-.010[d]	-.156	.876	-.010	.951
	MRN0xGender	.035[d]	.540	.589	.036	.946
	MRN0xIdeology0	.062[d]	.968	.334	.064	.934

a. Dependent Variable: Psychology_Tot

b. Predictors in the Model: (Constant), Ideology0

c. Predictors in the Model: (Constant), Ideology0, SES0, GenderCC, RaceCC

d. Predictors in the Model: (Constant), Ideology0, SES0, GenderCC, RaceCC, MRN0

```
REGRESSION
 /MISSING LISTWISE
 /STATISTICS COEFF OUTS R ANOVA CHANGE ZPP
 /CRITERIA=PIN(.05) POUT(.10)
 /NOORIGIN
 /DEPENDENT TrumpApproval
 /METHOD=ENTER Ideology0
 /METHOD=ENTER GenderCC RaceCC SES0
 /METHOD=ENTER MRN0
 /METHOD=ENTER MRN0xRace MRN0xSES0 MRN0xGender MRN0xIdeology0.
```

Regression

Notes

Output Created		15-DEC-2021 13:07:57
Comments		
Input	Data	C: \Users\njs5478\Dropbox\HM and COVID\0. Revise and Resubmit\2. R and R Data\Study 1b\Study1b_Data.sav
	Active Dataset	DataSet1
	Filter	<none>
	Weight	<none>
	Split File	<none>
	N of Rows in Working Data File	241
Missing Value Handling	Definition of Missing	User-defined missing values are treated as missing.
	Cases Used	Statistics are based on cases with no missing values for any variable used.
Syntax		REGRESSION /MISSING LISTWISE /STATISTICS COEFF OUTS R ANOVA CHANGE ZPP /CRITERIA=PIN(.05) POUT(.10) /NOORIGIN /DEPENDENT TrumpApproval /METHOD=ENTER Ideology0 /METHOD=ENTER GenderCC RaceCC SES0 /METHOD=ENTER MRN0 /METHOD=ENTER MRN0xRace MRN0xSES0 MRN0xGender MRN0xIdeology0.
Resources	Processor Time	00:00:00.02
	Elapsed Time	00:00:00.03

Notes

	Memory Required	45472 bytes
	Additional Memory Required for Residual Plots	0 bytes

Variables Entered/Removed[a]

Model	Variables Entered	Variables Removed	Method
1	Ideology0[b]	.	Enter
2	SES0, GenderCC, RaceCC[b]	.	Enter
3	MRN0[b]	.	Enter
4	MRN0xSES0, MRN0xGender, MRN0xIdeology0, MRN0xRace[b]	.	Enter

a. Dependent Variable: Do you approve or disapprove of the way Donald Trump is handling his job as President?

b. All requested variables entered.

Model Summary

Model	R	R Square	Adjusted R Square	Std. Error of the Estimate	Change Statistics		
					R Square Change	F Change	df1
1	.803[a]	.645	.644	1.249	.645	427.513	1
2	.812[b]	.660	.654	1.231	.014	3.281	3
3	.817[c]	.667	.660	1.220	.007	5.139	1
4	.821[d]	.673	.660	1.219	.006	1.083	4

Model Summary

Model	Change Statistics df2	Sig. F Change
1	235	.000
2	232	.022
3	231	.024
4	227	.366

a. Predictors: (Constant), Ideology0

b. Predictors: (Constant), Ideology0, SES0, GenderCC, RaceCC

c. Predictors: (Constant), Ideology0, SES0, GenderCC, RaceCC, MRN0

d. Predictors: (Constant), Ideology0, SES0, GenderCC, RaceCC, MRN0, MRN0xSES0, MRN0xGender, MRN0xIdeology0, MRN0xRace

ANOVA[a]

Model		Sum of Squares	df	Mean Square	F	Sig.
1	Regression	666.549	1	666.549	427.513	.000[b]
	Residual	366.396	235	1.559		
	Total	1032.945	236			
2	Regression	681.460	4	170.365	112.451	.000[c]
	Residual	351.485	232	1.515		
	Total	1032.945	236			
3	Regression	689.109	5	137.822	92.593	.000[d]
	Residual	343.836	231	1.488		
	Total	1032.945	236			
4	Regression	695.549	9	77.283	51.996	.000[e]
	Residual	337.397	227	1.486		
	Total	1032.945	236			

a. Dependent Variable: Do you approve or disapprove of the way Donald Trump is handling his job as President?

b. Predictors: (Constant), Ideology0

c. Predictors: (Constant), Ideology0, SES0, GenderCC, RaceCC

d. Predictors: (Constant), Ideology0, SES0, GenderCC, RaceCC, MRN0

e. Predictors: (Constant), Ideology0, SES0, GenderCC, RaceCC, MRN0, MRN0xSES0, MRN0xGender, MRN0xIdeology0, MRN0xRace

Coefficients[a]

Model		Unstandardized Coefficients B	Unstandardized Coefficients Std. Error	Standardized Coefficients Beta	t	Sig.
1	(Constant)	3.142	.081		38.735	.000
	Ideology0	1.057	.051	.803	20.676	.000
2	(Constant)	3.053	.093		32.689	.000
	Ideology0	1.035	.055	.786	18.683	.000
	GenderCC	-.003	.083	-.002	-.039	.969
	RaceCC	.174	.097	.073	1.791	.075
	SES0	-.258	.099	-.100	-2.602	.010
3	(Constant)	3.037	.093		32.715	.000
	Ideology0	.962	.064	.731	15.139	.000
	GenderCC	-.070	.087	-.034	-.800	.424
	RaceCC	.208	.098	.087	2.133	.034
	SES0	-.282	.099	-.109	-2.849	.005
	MRN0	.267	.118	.111	2.267	.024
4	(Constant)	2.946	.106		27.847	.000
	Ideology0	.950	.065	.722	14.515	.000
	GenderCC	-.072	.090	-.035	-.805	.422
	RaceCC	.204	.101	.085	2.022	.044
	SES0	-.265	.100	-.103	-2.663	.008
	MRN0	.257	.131	.107	1.965	.051
	MRN0xRace	.022	.114	.009	.193	.847
	MRN0xSES0	-.045	.106	-.017	-.431	.667
	MRN0xGender	.109	.107	.041	1.022	.308
	MRN0xIdeology0	.076	.056	.058	1.352	.178

Coefficients[a]

Model		Correlations Zero-order	Partial	Part
1	(Constant)			
	Ideology0	.803	.803	.803
2	(Constant)			
	Ideology0	.803	.775	.716
	GenderCC	.204	-.003	-.001
	RaceCC	.325	.117	.069
	SES0	-.051	-.168	-.100
3	(Constant)			
	Ideology0	.803	.706	.575
	GenderCC	.204	-.053	-.030
	RaceCC	.325	.139	.081
	SES0	-.051	-.184	-.108
	MRN0	.481	.148	.086
4	(Constant)			
	Ideology0	.803	.694	.551
	GenderCC	.204	-.053	-.031
	RaceCC	.325	.133	.077
	SES0	-.051	-.174	-.101
	MRN0	.481	.129	.075
	MRN0xRace	.360	.013	.007
	MRN0xSES0	-.133	-.029	-.016
	MRN0xGender	.133	.068	.039
	MRN0xIdeology0	.096	.089	.051

a. Dependent Variable: Do you approve or disapprove of the way Donald Trump is handling his job as President?

Excluded Variables[a]

Model		Beta In	t	Sig.	Partial Correlation	Collinearity Statistics Tolerance
1	GenderCC	-.009[b]	-.224	.823	-.015	.930
	RaceCC	.071[b]	1.730	.085	.112	.894
	SES0	-.099[b]	-2.566	.011	-.165	.997
	MRN0	.068[b]	1.480	.140	.096	.710
	MRN0xRace	.049[b]	1.149	.252	.075	.843
	MRN0xSES0	-.030[b]	-.769	.442	-.050	.983
	MRN0xGender	.056[b]	1.426	.155	.093	.991
	MRN0xIdeology0	.070[b]	1.797	.074	.117	.999
2	MRN0	.111[c]	2.267	.024	.148	.606
	MRN0xRace	.063[c]	1.475	.142	.097	.812
	MRN0xSES0	-.029[c]	-.747	.456	-.049	.979
	MRN0xGender	.068[c]	1.769	.078	.116	.973
	MRN0xIdeology0	.051[c]	1.299	.195	.085	.961
3	MRN0xRace	.037[d]	.824	.411	.054	.735
	MRN0xSES0	-.015[d]	-.381	.704	-.025	.952
	MRN0xGender	.055[d]	1.411	.159	.093	.944
	MRN0xIdeology0	.067[d]	1.714	.088	.112	.934

a. Dependent Variable: Do you approve or disapprove of the way Donald Trump is handling his job as President?

b. Predictors in the Model: (Constant), Ideology0

c. Predictors in the Model: (Constant), Ideology0, SES0, GenderCC, RaceCC

d. Predictors in the Model: (Constant), Ideology0, SES0, GenderCC, RaceCC, MRN0

```
REGRESSION
 /MISSING LISTWISE
 /STATISTICS COEFF OUTS R ANOVA CHANGE ZPP
 /CRITERIA=PIN(.05) POUT(.10)
 /NOORIGIN
 /DEPENDENT TrumpX
 /METHOD=ENTER Ideology0
 /METHOD=ENTER GenderCC RaceCC SES0
 /METHOD=ENTER MRN0
```

```
/METHOD=ENTER MRN0xRace MRN0xSES0 MRN0xGender MRN0xIdeology0.
```

Regression

Notes

Output Created		15-DEC-2021 13:07:57
Comments		
Input	Data	C:\Users\njs5478\Dropbox\HM and COVID\0. Revise and Resubmit\2. R and R Data\Study 1b\Study1b_Data.sav
	Active Dataset	DataSet1
	Filter	<none>
	Weight	<none>
	Split File	<none>
	N of Rows in Working Data File	241
Missing Value Handling	Definition of Missing	User-defined missing values are treated as missing.
	Cases Used	Statistics are based on cases with no missing values for any variable used.
Syntax		REGRESSION /MISSING LISTWISE /STATISTICS COEFF OUTS R ANOVA CHANGE ZPP /CRITERIA=PIN(.05) POUT(.10) /NOORIGIN /DEPENDENT TrumpX /METHOD=ENTER Ideology0 /METHOD=ENTER GenderCC RaceCC SES0 /METHOD=ENTER MRN0 /METHOD=ENTER MRN0xRace MRN0xSES0 MRN0xGender MRN0xIdeology0.
Resources	Processor Time	00:00:00.03
	Elapsed Time	00:00:00.03

Notes

	Memory Required	45472 bytes
	Additional Memory Required for Residual Plots	0 bytes

Variables Entered/Removed[a]

Model	Variables Entered	Variables Removed	Method
1	Ideology0[b]	.	Enter
2	SES0, GenderCC, RaceCC[b]	.	Enter
3	MRN0[b]	.	Enter
4	MRN0xSES0, MRN0xGender, MRN0xIdeology0, MRN0xRace[b]	.	Enter

a. Dependent Variable: TrumpX

b. All requested variables entered.

Model Summary

Model	R	R Square	Adjusted R Square	Std. Error of the Estimate	Change Statistics		
					R Square Change	F Change	df1
1	.779[a]	.607	.606	1.28817	.607	360.386	1
2	.785[b]	.616	.609	1.28251	.008	1.687	3
3	.790[c]	.625	.617	1.27018	.009	5.488	1
4	.798[d]	.636	.621	1.26209	.011	1.736	4

Model Summary

Model	Change Statistics	
	df2	Sig. F Change
1	233	.000
2	230	.171
3	229	.020
4	225	.143

a. Predictors: (Constant), Ideology0

b. Predictors: (Constant), Ideology0, SES0, GenderCC, RaceCC

c. Predictors: (Constant), Ideology0, SES0, GenderCC, RaceCC, MRN0

d. Predictors: (Constant), Ideology0, SES0, GenderCC, RaceCC, MRN0, MRN0xSES0, MRN0xGender, MRN0xIdeology0, MRN0xRace

ANOVA[a]

Model		Sum of Squares	df	Mean Square	F	Sig.
1	Regression	598.019	1	598.019	360.386	.000[b]
	Residual	386.637	233	1.659		
	Total	984.655	234			
2	Regression	606.343	4	151.586	92.159	.000[c]
	Residual	378.312	230	1.645		
	Total	984.655	234			
3	Regression	615.198	5	123.040	76.263	.000[d]
	Residual	369.458	229	1.613		
	Total	984.655	234			
4	Regression	626.258	9	69.584	43.685	.000[e]
	Residual	358.397	225	1.593		
	Total	984.655	234			

a. Dependent Variable: TrumpX

b. Predictors: (Constant), Ideology0

c. Predictors: (Constant), Ideology0, SES0, GenderCC, RaceCC

d. Predictors: (Constant), Ideology0, SES0, GenderCC, RaceCC, MRN0

e. Predictors: (Constant), Ideology0, SES0, GenderCC, RaceCC, MRN0, MRN0xSES0, MRN0xGender, MRN0xIdeology0, MRN0xRace

Coefficients[a]

Model		Unstandardized Coefficients B	Unstandardized Coefficients Std. Error	Standardized Coefficients Beta	t	Sig.
1	(Constant)	3.061	.084		36.423	.000
	Ideology0	1.007	.053	.779	18.984	.000
2	(Constant)	3.005	.098		30.800	.000
	Ideology0	.992	.058	.768	17.070	.000
	GenderCC	.010	.087	.005	.116	.907
	RaceCC	.110	.102	.047	1.086	.279
	SES0	-.206	.103	-.082	-1.991	.048
3	(Constant)	2.988	.097		30.845	.000
	Ideology0	.914	.066	.708	13.774	.000
	GenderCC	-.063	.092	-.031	-.681	.496
	RaceCC	.147	.102	.063	1.443	.150
	SES0	-.232	.103	-.092	-2.248	.026
	MRN0	.287	.123	.122	2.343	.020
4	(Constant)	2.849	.110		25.901	.000
	Ideology0	.905	.068	.701	13.317	.000
	GenderCC	-.065	.094	-.032	-.697	.487
	RaceCC	.149	.104	.064	1.429	.154
	SES0	-.219	.103	-.087	-2.121	.035
	MRN0	.286	.136	.121	2.104	.036
	MRN0xRace	-.010	.118	-.004	-.082	.934
	MRN0xSES0	.034	.109	.013	.315	.753
	MRN0xGender	.205	.111	.079	1.845	.066
	MRN0xIdeology0	.080	.059	.062	1.360	.175

Coefficients[a]

Model		Correlations Zero-order	Partial	Part
1	(Constant)			
	Ideology0	.779	.779	.779
2	(Constant)			
	Ideology0	.779	.748	.698
	GenderCC	.214	.008	.005
	RaceCC	.292	.071	.044
	SES0	-.032	-.130	-.081
3	(Constant)			
	Ideology0	.779	.673	.558
	GenderCC	.214	-.045	-.028
	RaceCC	.292	.095	.058
	SES0	-.032	-.147	-.091
	MRN0	.480	.153	.095
4	(Constant)			
	Ideology0	.779	.664	.536
	GenderCC	.214	-.046	-.028
	RaceCC	.292	.095	.057
	SES0	-.032	-.140	-.085
	MRN0	.480	.139	.085
	MRN0xRace	.350	-.005	-.003
	MRN0xSES0	-.103	.021	.013
	MRN0xGender	.177	.122	.074
	MRN0xIdeology0	.096	.090	.055

a. Dependent Variable: TrumpX

Excluded Variables[a]

Model		Beta In	t	Sig.	Partial Correlation	Collinearity Statistics Tolerance
1	GenderCC	.000[b]	-.009	.993	-.001	.924
	RaceCC	.045[b]	1.043	.298	.068	.896
	SES0	-.081[b]	-1.973	.050	-.128	.996
	MRN0	.087[b]	1.794	.074	.117	.712
	MRN0xRace	.050[b]	1.122	.263	.073	.844
	MRN0xSES0	-.005[b]	-.112	.911	-.007	.984
	MRN0xGender	.094[b]	2.302	.022	.149	.988
	MRN0xIdeology0	.073[b]	1.792	.074	.117	.999
2	MRN0	.122[c]	2.343	.020	.153	.607
	MRN0xRace	.059[c]	1.310	.191	.086	.814
	MRN0xSES0	-.004[c]	-.101	.919	-.007	.980
	MRN0xGender	.104[c]	2.540	.012	.166	.970
	MRN0xIdeology0	.060[c]	1.437	.152	.095	.961
3	MRN0xRace	.030[d]	.628	.531	.042	.737
	MRN0xSES0	.012[d]	.292	.771	.019	.953
	MRN0xGender	.090[d]	2.172	.031	.142	.939
	MRN0xIdeology0	.078[d]	1.877	.062	.123	.934

a. Dependent Variable: TrumpX

b. Predictors in the Model: (Constant), Ideology0

c. Predictors in the Model: (Constant), Ideology0, SES0, GenderCC, RaceCC

d. Predictors in the Model: (Constant), Ideology0, SES0, GenderCC, RaceCC, MRN0

```
REGRESSION
 /MISSING LISTWISE
 /STATISTICS COEFF OUTS R ANOVA CHANGE ZPP
 /CRITERIA=PIN(.05) POUT(.10)
 /NOORIGIN
 /DEPENDENT BidenX
 /METHOD=ENTER Ideology0
 /METHOD=ENTER GenderCC RaceCC SES0
 /METHOD=ENTER MRN0
 /METHOD=ENTER MRN0xRace MRN0xSES0 MRN0xGender MRN0xIdeology0.
```

Regression

Notes

Output Created		15-DEC-2021 13:07:57
Comments		
Input	Data	C: \Users\njs5478\Dropbox\HM and COVID\0. Revise and Resubmit\2. R and R Data\Study 1b\Study1b_Data.sav
	Active Dataset	DataSet1
	Filter	<none>
	Weight	<none>
	Split File	<none>
	N of Rows in Working Data File	241
Missing Value Handling	Definition of Missing	User-defined missing values are treated as missing.
	Cases Used	Statistics are based on cases with no missing values for any variable used.
Syntax		REGRESSION<br>/MISSING LISTWISE<br>/STATISTICS COEFF OUTS R ANOVA CHANGE ZPP<br>/CRITERIA=PIN(.05) POUT(.10)<br>/NOORIGIN<br>/DEPENDENT BidenX<br>/METHOD=ENTER Ideology0<br>/METHOD=ENTER GenderCC RaceCC SES0<br>/METHOD=ENTER MRN0<br>/METHOD=ENTER MRN0xRace MRN0xSES0 MRN0xGender MRN0xIdeology0.
Resources	Processor Time	00:00:00.03
	Elapsed Time	00:00:00.03

Notes

	Memory Required	45472 bytes
	Additional Memory Required for Residual Plots	0 bytes

Variables Entered/Removed[a]

Model	Variables Entered	Variables Removed	Method
1	Ideology0[b]	.	Enter
2	SES0, GenderCC, RaceCC[b]	.	Enter
3	MRN0[b]	.	Enter
4	MRN0xSES0, MRN0xGender, MRN0xIdeology0, MRN0xRace[b]	.	Enter

a. Dependent Variable: BidenX

b. All requested variables entered.

Model Summary

Model	R	R Square	Adjusted R Square	Std. Error of the Estimate	Change Statistics		
					R Square Change	F Change	df1
1	.701[a]	.491	.489	1.28165	.491	219.754	1
2	.706[b]	.498	.489	1.28092	.007	1.087	3
3	.708[c]	.501	.490	1.28002	.003	1.316	1
4	.712[d]	.507	.487	1.28343	.006	.703	4

Model Summary

Model	Change Statistics	
	df2	Sig. F Change
1	228	.000
2	225	.356
3	224	.252
4	220	.591

a. Predictors: (Constant), Ideology0

b. Predictors: (Constant), Ideology0, SES0, GenderCC, RaceCC

c. Predictors: (Constant), Ideology0, SES0, GenderCC, RaceCC, MRN0

d. Predictors: (Constant), Ideology0, SES0, GenderCC, RaceCC, MRN0, MRN0xSES0, MRN0xGender, MRN0xIdeology0, MRN0xRace

ANOVA[a]

Model		Sum of Squares	df	Mean Square	F	Sig.
1	Regression	360.976	1	360.976	219.754	.000[b]
	Residual	374.520	228	1.643		
	Total	735.496	229			
2	Regression	366.324	4	91.581	55.816	.000[c]
	Residual	369.172	225	1.641		
	Total	735.496	229			
3	Regression	368.480	5	73.696	44.979	.000[d]
	Residual	367.015	224	1.638		
	Total	735.496	229			
4	Regression	373.112	9	41.457	25.168	.000[e]
	Residual	362.384	220	1.647		
	Total	735.496	229			

a. Dependent Variable: BidenX

b. Predictors: (Constant), Ideology0

c. Predictors: (Constant), Ideology0, SES0, GenderCC, RaceCC

d. Predictors: (Constant), Ideology0, SES0, GenderCC, RaceCC, MRN0

e. Predictors: (Constant), Ideology0, SES0, GenderCC, RaceCC, MRN0, MRN0xSES0, MRN0xGender, MRN0xIdeology0, MRN0xRace

Coefficients[a]

Model		Unstandardized Coefficients B	Unstandardized Coefficients Std. Error	Standardized Coefficients Beta	t	Sig.
1	(Constant)	4.483	.085		53.034	.000
	Ideology0	-.783	.053	-.701	-14.824	.000
2	(Constant)	4.445	.098		45.154	.000
	Ideology0	-.798	.058	-.714	-13.732	.000
	GenderCC	-.024	.088	-.014	-.276	.783
	RaceCC	.081	.102	.040	.795	.427
	SES0	.164	.105	.074	1.566	.119
3	(Constant)	4.453	.099		45.156	.000
	Ideology0	-.760	.067	-.680	-11.357	.000
	GenderCC	.012	.093	.007	.128	.898
	RaceCC	.064	.104	.031	.614	.540
	SES0	.176	.105	.080	1.680	.094
	MRN0	-.142	.124	-.069	-1.147	.252
4	(Constant)	4.541	.113		40.278	.000
	Ideology0	-.759	.069	-.679	-10.962	.000
	GenderCC	.008	.097	.004	.080	.936
	RaceCC	.061	.108	.030	.568	.570
	SES0	.172	.106	.078	1.621	.106
	MRN0	-.138	.139	-.067	-.993	.322
	MRN0xRace	.036	.121	.018	.298	.766
	MRN0xSES0	-.024	.112	-.011	-.216	.829
	MRN0xGender	-.163	.114	-.072	-1.429	.155
	MRN0xIdeology0	-.034	.060	-.031	-.578	.564

Coefficients[a]

Model		Correlations		
		Zero-order	Partial	Part
1	(Constant)			
	Ideology0	-.701	-.701	-.701
2	(Constant)			
	Ideology0	-.701	-.675	-.649
	GenderCC	-.207	-.018	-.013
	RaceCC	-.191	.053	.038
	SES0	.033	.104	.074
3	(Constant)			
	Ideology0	-.701	-.604	-.536
	GenderCC	-.207	.009	.006
	RaceCC	-.191	.041	.029
	SES0	.033	.112	.079
	MRN0	-.421	-.076	-.054
4	(Constant)			
	Ideology0	-.701	-.594	-.519
	GenderCC	-.207	.005	.004
	RaceCC	-.191	.038	.027
	SES0	.033	.109	.077
	MRN0	-.421	-.067	-.047
	MRN0xRace	-.304	.020	.014
	MRN0xSES0	.091	-.015	-.010
	MRN0xGender	-.160	-.096	-.068
	MRN0xIdeology0	-.056	-.039	-.027

a. Dependent Variable: BidenX

Excluded Variables[a]

Model		Beta In	t	Sig.	Partial Correlation	Collinearity Statistics Tolerance
1	GenderCC	-.014[b]	-.279	.780	-.019	.923
	RaceCC	.043[b]	.862	.389	.057	.893
	SES0	.075[b]	1.596	.112	.105	.996
	MRN0	-.063[b]	-1.119	.264	-.074	.712
	MRN0xRace	-.031[b]	-.609	.543	-.040	.843
	MRN0xSES0	.001[b]	.019	.985	.001	.984
	MRN0xGender	-.084[b]	-1.768	.078	-.117	.988
	MRN0xIdeology0	-.035[b]	-.736	.462	-.049	.999
2	MRN0	-.069[c]	-1.147	.252	-.076	.609
	MRN0xRace	-.023[c]	-.434	.665	-.029	.810
	MRN0xSES0	.003[c]	.059	.953	.004	.980
	MRN0xGender	-.083[c]	-1.741	.083	-.116	.970
	MRN0xIdeology0	-.031[c]	-.646	.519	-.043	.960
3	MRN0xRace	-.005[d]	-.085	.933	-.006	.733
	MRN0xSES0	-.007[d]	-.134	.893	-.009	.953
	MRN0xGender	-.076[d]	-1.553	.122	-.103	.936
	MRN0xIdeology0	-.042[d]	-.850	.396	-.057	.934

a. Dependent Variable: BidenX

b. Predictors in the Model: (Constant), Ideology0

c. Predictors in the Model: (Constant), Ideology0, SES0, GenderCC, RaceCC

d. Predictors in the Model: (Constant), Ideology0, SES0, GenderCC, RaceCC, MRN0

```
REGRESSION
 /MISSING LISTWISE
 /STATISTICS COEFF OUTS R ANOVA CHANGE ZPP
 /CRITERIA=PIN(.05) POUT(.10)
 /NOORIGIN
 /DEPENDENT PelosiX
 /METHOD=ENTER Ideology0
 /METHOD=ENTER GenderCC RaceCC SES0
 /METHOD=ENTER MRN0
 /METHOD=ENTER MRN0xRace MRN0xSES0 MRN0xGender MRN0xIdeology0.
```

Regression

Notes

Output Created		15-DEC-2021 13:07:57
Comments		
Input	Data	C: \Users\njs5478\Dropbox\HM and COVID\0. Revise and Resubmit\2. R and R Data\Study 1b\Study1b_Data.sav
	Active Dataset	DataSet1
	Filter	<none>
	Weight	<none>
	Split File	<none>
	N of Rows in Working Data File	241
Missing Value Handling	Definition of Missing	User-defined missing values are treated as missing.
	Cases Used	Statistics are based on cases with no missing values for any variable used.
Syntax		REGRESSION<br>/MISSING LISTWISE<br>/STATISTICS COEFF OUTS R ANOVA CHANGE ZPP<br>/CRITERIA=PIN(.05) POUT(.10)<br>/NOORIGIN<br>/DEPENDENT PelosiX<br>/METHOD=ENTER Ideology0<br>/METHOD=ENTER GenderCC RaceCC SES0<br>/METHOD=ENTER MRN0<br>/METHOD=ENTER MRN0xRace MRN0xSES0 MRN0xGender MRN0xIdeology0.
Resources	Processor Time	00:00:00.03
	Elapsed Time	00:00:00.03

Notes

	Memory Required	45472 bytes
	Additional Memory Required for Residual Plots	0 bytes

Variables Entered/Removed[a]

Model	Variables Entered	Variables Removed	Method
1	Ideology0[b]	.	Enter
2	SES0, GenderCC, RaceCC[b]	.	Enter
3	MRN0[b]	.	Enter
4	MRN0xSES0, MRN0xIdeology0, MRN0xGender, MRN0xRace[b]	.	Enter

a. Dependent Variable: PelosiX

b. All requested variables entered.

Model Summary

Model	R	R Square	Adjusted R Square	Std. Error of the Estimate	Change Statistics		
					R Square Change	F Change	df1
1	.637[a]	.406	.403	1.26027	.406	135.540	1
2	.652[b]	.425	.414	1.24941	.019	2.152	3
3	.659[c]	.434	.419	1.24359	.008	2.830	1
4	.665[d]	.442	.416	1.24729	.008	.712	4

Model Summary

Model	Change Statistics	
	df2	Sig. F Change
1	198	.000
2	195	.095
3	194	.094
4	190	.585

a. Predictors: (Constant), Ideology0

b. Predictors: (Constant), Ideology0, SES0, GenderCC, RaceCC

c. Predictors: (Constant), Ideology0, SES0, GenderCC, RaceCC, MRN0

d. Predictors: (Constant), Ideology0, SES0, GenderCC, RaceCC, MRN0, MRN0xSES0, MRN0xIdeology0, MRN0xGender, MRN0xRace

ANOVA[a]

Model		Sum of Squares	df	Mean Square	F	Sig.
1	Regression	215.276	1	215.276	135.540	.000[b]
	Residual	314.479	198	1.588		
	Total	529.755	199			
2	Regression	225.355	4	56.339	36.091	.000[c]
	Residual	304.400	195	1.561		
	Total	529.755	199			
3	Regression	229.732	5	45.946	29.710	.000[d]
	Residual	300.023	194	1.547		
	Total	529.755	199			
4	Regression	234.164	9	26.018	16.724	.000[e]
	Residual	295.591	190	1.556		
	Total	529.755	199			

a. Dependent Variable: PelosiX

b. Predictors: (Constant), Ideology0

c. Predictors: (Constant), Ideology0, SES0, GenderCC, RaceCC

d. Predictors: (Constant), Ideology0, SES0, GenderCC, RaceCC, MRN0

e. Predictors: (Constant), Ideology0, SES0, GenderCC, RaceCC, MRN0, MRN0xSES0, MRN0xIdeology0, MRN0xGender, MRN0xRace

Coefficients[a]

Model		Unstandardized Coefficients B	Unstandardized Coefficients Std. Error	Standardized Coefficients Beta	t	Sig.
1	(Constant)	3.578	.089		40.113	.000
	Ideology0	-.656	.056	-.637	-11.642	.000
2	(Constant)	3.498	.104		33.555	.000
	Ideology0	-.650	.061	-.631	-10.719	.000
	GenderCC	-.173	.092	-.106	-1.888	.061
	RaceCC	.160	.108	.084	1.477	.141
	SES0	-.080	.110	-.040	-.729	.467
3	(Constant)	3.513	.104		33.729	.000
	Ideology0	-.592	.070	-.575	-8.490	.000
	GenderCC	-.115	.097	-.070	-1.176	.241
	RaceCC	.132	.109	.069	1.209	.228
	SES0	-.067	.109	-.033	-.612	.541
	MRN0	-.222	.132	-.117	-1.682	.094
4	(Constant)	3.570	.118		30.241	.000
	Ideology0	-.596	.072	-.578	-8.257	.000
	GenderCC	-.123	.100	-.075	-1.225	.222
	RaceCC	.134	.112	.070	1.198	.232
	SES0	-.064	.110	-.032	-.580	.563
	MRN0	-.234	.149	-.123	-1.571	.118
	MRN0xRace	.101	.134	.053	.754	.452
	MRN0xSES0	.102	.124	.046	.821	.413
	MRN0xGender	-.146	.121	-.069	-1.204	.230
	MRN0xIdeology0	-.021	.062	-.020	-.343	.732

Coefficients[a]

Model		Correlations Zero-order	Partial	Part
1	(Constant)			
	Ideology0	-.637	-.637	-.637
2	(Constant)			
	Ideology0	-.637	-.609	-.582
	GenderCC	-.268	-.134	-.102
	RaceCC	-.110	.105	.080
	SES0	-.093	-.052	-.040
3	(Constant)			
	Ideology0	-.637	-.520	-.459
	GenderCC	-.268	-.084	-.064
	RaceCC	-.110	.087	.065
	SES0	-.093	-.044	-.033
	MRN0	-.455	-.120	-.091
4	(Constant)			
	Ideology0	-.637	-.514	-.447
	GenderCC	-.268	-.088	-.066
	RaceCC	-.110	.087	.065
	SES0	-.093	-.042	-.031
	MRN0	-.455	-.113	-.085
	MRN0xRace	-.319	.055	.041
	MRN0xSES0	.107	.059	.045
	MRN0xGender	-.187	-.087	-.065
	MRN0xIdeology0	-.043	-.025	-.019

a. Dependent Variable: PelosiX

Excluded Variables[a]

Model		Beta In	t	Sig.	Partial Correlation	Collinearity Statistics Tolerance
1	GenderCC	-.109[b]	-1.934	.055	-.137	.932
	RaceCC	.086[b]	1.505	.134	.107	.912
	SES0	-.042[b]	-.764	.446	-.054	.994
	MRN0	-.160[b]	-2.499	.013	-.175	.714
	MRN0xRace	-.039[b]	-.627	.531	-.045	.796
	MRN0xSES0	.054[b]	.983	.327	.070	.993
	MRN0xGender	-.094[b]	-1.698	.091	-.120	.977
	MRN0xIdeology0	.004[b]	.065	.948	.005	.995
2	MRN0	-.117[c]	-1.682	.094	-.120	.607
	MRN0xRace	-.014[c]	-.226	.821	-.016	.772
	MRN0xSES0	.059[c]	1.079	.282	.077	.983
	MRN0xGender	-.087[c]	-1.573	.117	-.112	.966
	MRN0xIdeology0	-.002[c]	-.031	.975	-.002	.948
3	MRN0xRace	.031[d]	.463	.644	.033	.655
	MRN0xSES0	.050[d]	.908	.365	.065	.972
	MRN0xGender	-.074[d]	-1.323	.187	-.095	.941
	MRN0xIdeology0	-.015[d]	-.265	.791	-.019	.930

a. Dependent Variable: PelosiX

b. Predictors in the Model: (Constant), Ideology0

c. Predictors in the Model: (Constant), Ideology0, SES0, GenderCC, RaceCC

d. Predictors in the Model: (Constant), Ideology0, SES0, GenderCC, RaceCC, MRN0

```
REGRESSION
 /MISSING LISTWISE
 /STATISTICS COEFF OUTS R ANOVA CHANGE ZPP
 /CRITERIA=PIN(.05) POUT(.10)
 /NOORIGIN
 /DEPENDENT McConnellX
 /METHOD=ENTER Ideology0
 /METHOD=ENTER GenderCC RaceCC SES0
 /METHOD=ENTER MRN0
 /METHOD=ENTER MRN0xRace MRN0xSES0 MRN0xGender MRN0xIdeology0.
```

Regression

Notes

Output Created		15-DEC-2021 13:07:57
Comments		
Input	Data	C:\Users\njs5478\Dropbox\HM and COVID\0. Revise and Resubmit\2. R and R Data\Study 1b\Study1b_Data.sav
	Active Dataset	DataSet1
	Filter	<none>
	Weight	<none>
	Split File	<none>
	N of Rows in Working Data File	241
Missing Value Handling	Definition of Missing	User-defined missing values are treated as missing.
	Cases Used	Statistics are based on cases with no missing values for any variable used.
Syntax		REGRESSION<br>/MISSING LISTWISE<br>/STATISTICS COEFF OUTS R ANOVA CHANGE ZPP<br>/CRITERIA=PIN(.05) POUT(.10)<br>/NOORIGIN<br>/DEPENDENT McConnellX<br>/METHOD=ENTER Ideology0<br>/METHOD=ENTER GenderCC RaceCC SES0<br>/METHOD=ENTER MRN0<br>/METHOD=ENTER MRN0xRace MRN0xSES0 MRN0xGender MRN0xIdeology0.
Resources	Processor Time	00:00:00.02
	Elapsed Time	00:00:00.01

Notes

	Memory Required	45472 bytes
	Additional Memory Required for Residual Plots	0 bytes

Variables Entered/Removed[a]

Model	Variables Entered	Variables Removed	Method
1	Ideology0[b]	.	Enter
2	SES0, GenderCC, RaceCC[b]	.	Enter
3	MRN0[b]	.	Enter
4	MRN0xSES0, MRN0xGender, MRN0xIdeology0, MRN0xRace[b]	.	Enter

a. Dependent Variable: McConnellX

b. All requested variables entered.

Model Summary

Model	R	R Square	Adjusted R Square	Std. Error of the Estimate	Change Statistics		
					R Square Change	F Change	df1
1	.535[a]	.287	.283	1.30045	.287	77.140	1
2	.561[b]	.315	.300	1.28465	.028	2.583	3
3	.585[c]	.342	.325	1.26170	.028	7.941	1
4	.608[d]	.370	.339	1.24824	.028	2.018	4

Model Summary

Model	Change Statistics	
	df2	Sig. F Change
1	192	.000
2	189	.055
3	188	.005
4	184	.094

a. Predictors: (Constant), Ideology0

b. Predictors: (Constant), Ideology0, SES0, GenderCC, RaceCC

c. Predictors: (Constant), Ideology0, SES0, GenderCC, RaceCC, MRN0

d. Predictors: (Constant), Ideology0, SES0, GenderCC, RaceCC, MRN0, MRN0xSES0, MRN0xGender, MRN0xIdeology0, MRN0xRace

ANOVA[a]

Model		Sum of Squares	df	Mean Square	F	Sig.
1	Regression	130.456	1	130.456	77.140	.000[b]
	Residual	324.703	192	1.691		
	Total	455.160	193			
2	Regression	143.246	4	35.811	21.699	.000[c]
	Residual	311.914	189	1.650		
	Total	455.160	193			
3	Regression	155.887	5	31.177	19.585	.000[d]
	Residual	299.273	188	1.592		
	Total	455.160	193			
4	Regression	168.467	9	18.719	12.014	.000[e]
	Residual	286.693	184	1.558		
	Total	455.160	193			

a. Dependent Variable: McConnellX

b. Predictors: (Constant), Ideology0

c. Predictors: (Constant), Ideology0, SES0, GenderCC, RaceCC

d. Predictors: (Constant), Ideology0, SES0, GenderCC, RaceCC, MRN0

e. Predictors: (Constant), Ideology0, SES0, GenderCC, RaceCC, MRN0, MRN0xSES0, MRN0xGender, MRN0xIdeology0, MRN0xRace

Coefficients[a]

Model		Unstandardized Coefficients B	Unstandardized Coefficients Std. Error	Standardized Coefficients Beta	t	Sig.
1	(Constant)	3.219	.093		34.465	.000
	Ideology0	.525	.060	.535	8.783	.000
2	(Constant)	3.213	.109		29.352	.000
	Ideology0	.567	.064	.578	8.831	.000
	GenderCC	-.257	.096	-.168	-2.685	.008
	RaceCC	.032	.113	.018	.280	.780
	SES0	-.071	.116	-.037	-.611	.542
3	(Constant)	3.203	.108		29.785	.000
	Ideology0	.465	.073	.475	6.404	.000
	GenderCC	-.352	.100	-.230	-3.525	.001
	RaceCC	.078	.112	.044	.695	.488
	SES0	-.097	.115	-.051	-.851	.396
	MRN0	.372	.132	.213	2.818	.005
4	(Constant)	3.126	.120		26.090	.000
	Ideology0	.428	.075	.436	5.732	.000
	GenderCC	-.336	.102	-.219	-3.299	.001
	RaceCC	.099	.115	.055	.854	.394
	SES0	-.096	.114	-.050	-.839	.403
	MRN0	.287	.147	.164	1.950	.053
	MRN0xRace	.192	.134	.110	1.441	.151
	MRN0xSES0	-.183	.120	-.092	-1.525	.129
	MRN0xGender	.053	.118	.028	.449	.654
	MRN0xIdeology0	.075	.063	.077	1.188	.236

Coefficients[a]

Model		Correlations Zero-order	Partial	Part
1	(Constant)			
	Ideology0	.535	.535	.535
2	(Constant)			
	Ideology0	.535	.540	.532
	GenderCC	-.014	-.192	-.162
	RaceCC	.176	.020	.017
	SES0	.011	-.044	-.037
3	(Constant)			
	Ideology0	.535	.423	.379
	GenderCC	-.014	-.249	-.208
	RaceCC	.176	.051	.041
	SES0	.011	-.062	-.050
	MRN0	.367	.201	.167
4	(Constant)			
	Ideology0	.535	.389	.335
	GenderCC	-.014	-.236	-.193
	RaceCC	.176	.063	.050
	SES0	.011	-.062	-.049
	MRN0	.367	.142	.114
	MRN0xRace	.369	.106	.084
	MRN0xSES0	-.163	-.112	-.089
	MRN0xGender	.156	.033	.026
	MRN0xIdeology0	.051	.087	.070

a. Dependent Variable: McConnellX

Excluded Variables[a]

Model		Beta In	t	Sig.	Partial Correlation	Collinearity Statistics Tolerance
1	GenderCC	-.169[b]	-2.711	.007	-.193	.929
	RaceCC	.019[b]	.291	.772	.021	.911
	SES0	-.041[b]	-.670	.503	-.048	.991
	MRN0	.112[b]	1.563	.120	.112	.713
	MRN0xRace	.171[b]	2.566	.011	.183	.815
	MRN0xSES0	-.113[b]	-1.851	.066	-.133	.991
	MRN0xGender	.097[b]	1.583	.115	.114	.987
	MRN0xIdeology0	.062[b]	1.020	.309	.074	1.000
2	MRN0	.213[c]	2.818	.005	.201	.615
	MRN0xRace	.198[c]	2.940	.004	.210	.771
	MRN0xSES0	-.107[c]	-1.775	.078	-.128	.982
	MRN0xGender	.085[c]	1.394	.165	.101	.967
	MRN0xIdeology0	.071[c]	1.151	.251	.084	.956
3	MRN0xRace	.148[d]	2.059	.041	.149	.667
	MRN0xSES0	-.088[d]	-1.464	.145	-.106	.967
	MRN0xGender	.062[d]	1.021	.309	.074	.947
	MRN0xIdeology0	.103[d]	1.679	.095	.122	.929

a. Dependent Variable: McConnellX

b. Predictors in the Model: (Constant), Ideology0

c. Predictors in the Model: (Constant), Ideology0, SES0, GenderCC, RaceCC

d. Predictors in the Model: (Constant), Ideology0, SES0, GenderCC, RaceCC, MRN0

```
REGRESSION
 /MISSING LISTWISE
 /STATISTICS COEFF OUTS R ANOVA CHANGE ZPP
 /CRITERIA=PIN(.05) POUT(.10)
 /NOORIGIN
 /DEPENDENT FauciX
 /METHOD=ENTER Ideology0
 /METHOD=ENTER GenderCC RaceCC SES0
 /METHOD=ENTER MRN0
 /METHOD=ENTER MRN0xRace MRN0xSES0 MRN0xGender MRN0xIdeology0.
```

Regression

Notes

Output Created		15-DEC-2021 13:07:57
Comments		
Input	Data	C:\Users\njs5478\Dropbox\HM and COVID\0. Revise and Resubmit\2. R and R Data\Study 1b\Study1b_Data.sav
	Active Dataset	DataSet1
	Filter	<none>
	Weight	<none>
	Split File	<none>
	N of Rows in Working Data File	241
Missing Value Handling	Definition of Missing	User-defined missing values are treated as missing.
	Cases Used	Statistics are based on cases with no missing values for any variable used.
Syntax		REGRESSION /MISSING LISTWISE /STATISTICS COEFF OUTS R ANOVA CHANGE ZPP /CRITERIA=PIN(.05) POUT(.10) /NOORIGIN /DEPENDENT FauciX /METHOD=ENTER Ideology0 /METHOD=ENTER GenderCC RaceCC SES0 /METHOD=ENTER MRN0 /METHOD=ENTER MRN0xRace MRN0xSES0 MRN0xGender MRN0xIdeology0.
Resources	Processor Time	00:00:00.03
	Elapsed Time	00:00:00.03

Notes

	Memory Required	45472 bytes
	Additional Memory Required for Residual Plots	0 bytes

Variables Entered/Removed[a]

Model	Variables Entered	Variables Removed	Method
1	Ideology0[b]	.	Enter
2	SES0, GenderCC, RaceCC[b]	.	Enter
3	MRN0[b]	.	Enter
4	MRN0xSES0, MRN0xGender, MRN0xIdeology0, MRN0xRace[b]	.	Enter

a. Dependent Variable: FauciX

b. All requested variables entered.

Model Summary

Model	R	R Square	Adjusted R Square	Std. Error of the Estimate	Change Statistics		
					R Square Change	F Change	df1
1	.309[a]	.095	.091	1.56802	.095	22.160	1
2	.340[b]	.116	.099	1.56163	.020	1.574	3
3	.370[c]	.137	.116	1.54634	.021	5.114	1
4	.401[d]	.161	.123	1.54003	.024	1.423	4

Model Summary

Model	Change Statistics	
	df2	Sig. F Change
1	210	.000
2	207	.197
3	206	.025
4	202	.228

a. Predictors: (Constant), Ideology0

b. Predictors: (Constant), Ideology0, SES0, GenderCC, RaceCC

c. Predictors: (Constant), Ideology0, SES0, GenderCC, RaceCC, MRN0

d. Predictors: (Constant), Ideology0, SES0, GenderCC, RaceCC, MRN0, MRN0xSES0, MRN0xGender, MRN0xIdeology0, MRN0xRace

ANOVA[a]

Model		Sum of Squares	df	Mean Square	F	Sig.
1	Regression	54.484	1	54.484	22.160	.000[b]
	Residual	516.327	210	2.459		
	Total	570.811	211			
2	Regression	66.001	4	16.500	6.766	.000[c]
	Residual	504.810	207	2.439		
	Total	570.811	211			
3	Regression	78.230	5	15.646	6.543	.000[d]
	Residual	492.581	206	2.391		
	Total	570.811	211			
4	Regression	91.727	9	10.192	4.297	.000[e]
	Residual	479.084	202	2.372		
	Total	570.811	211			

a. Dependent Variable: FauciX

b. Predictors: (Constant), Ideology0

c. Predictors: (Constant), Ideology0, SES0, GenderCC, RaceCC

d. Predictors: (Constant), Ideology0, SES0, GenderCC, RaceCC, MRN0

e. Predictors: (Constant), Ideology0, SES0, GenderCC, RaceCC, MRN0, MRN0xSES0, MRN0xGender, MRN0xIdeology0, MRN0xRace

Coefficients[a]

Model		Unstandardized Coefficients B	Unstandardized Coefficients Std. Error	Standardized Coefficients Beta	t	Sig.
1	(Constant)	5.121	.108		47.554	.000
	Ideology0	-.324	.069	-.309	-4.707	.000
2	(Constant)	5.028	.123		41.015	.000
	Ideology0	-.376	.076	-.358	-4.920	.000
	GenderCC	.036	.113	.022	.320	.749
	RaceCC	.190	.128	.102	1.480	.140
	SES0	.202	.135	.099	1.500	.135
3	(Constant)	5.054	.122		41.452	.000
	Ideology0	-.288	.085	-.274	-3.381	.001
	GenderCC	.138	.121	.084	1.144	.254
	RaceCC	.152	.128	.082	1.184	.238
	SES0	.228	.134	.111	1.704	.090
	MRN0	-.357	.158	-.188	-2.261	.025
4	(Constant)	5.163	.141		36.669	.000
	Ideology0	-.282	.087	-.269	-3.225	.001
	GenderCC	.107	.122	.065	.879	.380
	RaceCC	.104	.131	.056	.796	.427
	SES0	.237	.134	.116	1.771	.078
	MRN0	-.264	.172	-.138	-1.532	.127
	MRN0xRace	-.050	.150	-.026	-.336	.737
	MRN0xSES0	.034	.145	.016	.237	.813
	MRN0xGender	-.331	.146	-.154	-2.267	.024
	MRN0xIdeology0	.063	.076	.060	.834	.405

Coefficients[a]

Model		Correlations Zero-order	Partial	Part
1	(Constant)			
	Ideology0	-.309	-.309	-.309
2	(Constant)			
	Ideology0	-.309	-.324	-.322
	GenderCC	-.075	.022	.021
	RaceCC	-.007	.102	.097
	SES0	.074	.104	.098
3	(Constant)			
	Ideology0	-.309	-.229	-.219
	GenderCC	-.075	.079	.074
	RaceCC	-.007	.082	.077
	SES0	.074	.118	.110
	MRN0	-.272	-.156	-.146
4	(Constant)			
	Ideology0	-.309	-.221	-.208
	GenderCC	-.075	.062	.057
	RaceCC	-.007	.056	.051
	SES0	.074	.124	.114
	MRN0	-.272	-.107	-.099
	MRN0xRace	-.195	-.024	-.022
	MRN0xSES0	.058	.017	.015
	MRN0xGender	-.190	-.158	-.146
	MRN0xIdeology0	.025	.059	.054

a. Dependent Variable: FauciX

Excluded Variables[a]

Model		Beta In	t	Sig.	Partial Correlation	Collinearity Statistics Tolerance
1	GenderCC	.023[b]	.327	.744	.023	.904
	RaceCC	.105[b]	1.519	.130	.105	.894
	SES0	.103[b]	1.571	.118	.108	.992
	MRN0	-.152[b]	-1.992	.048	-.137	.728
	MRN0xRace	-.088[b]	-1.244	.215	-.086	.851
	MRN0xSES0	.042[b]	.639	.523	.044	.997
	MRN0xGender	-.169[b]	-2.605	.010	-.177	.995
	MRN0xIdeology0	.050[b]	.754	.452	.052	.994
2	MRN0	-.188[c]	-2.261	.025	-.156	.609
	MRN0xRace	-.074[c]	-1.040	.300	-.072	.832
	MRN0xSES0	.045[c]	.689	.492	.048	.994
	MRN0xGender	-.161[c]	-2.472	.014	-.170	.980
	MRN0xIdeology0	.045[c]	.682	.496	.047	.972
3	MRN0xRace	-.031[d]	-.416	.678	-.029	.763
	MRN0xSES0	.030[d]	.456	.649	.032	.983
	MRN0xGender	-.145[d]	-2.216	.028	-.153	.964
	MRN0xIdeology0	.020[d]	.301	.764	.021	.943

a. Dependent Variable: FauciX

b. Predictors in the Model: (Constant), Ideology0

c. Predictors in the Model: (Constant), Ideology0, SES0, GenderCC, RaceCC

d. Predictors in the Model: (Constant), Ideology0, SES0, GenderCC, RaceCC, MRN0

```
REGRESSION
 /MISSING LISTWISE
 /STATISTICS COEFF OUTS R ANOVA CHANGE ZPP
 /CRITERIA=PIN(.05) POUT(.10)
 /NOORIGIN
 /DEPENDENT RepCongressX
 /METHOD=ENTER Ideology0
 /METHOD=ENTER GenderCC RaceCC SES0
 /METHOD=ENTER MRN0
 /METHOD=ENTER MRN0xRace MRN0xSES0 MRN0xGender MRN0xIdeology0.
```

Regression

Notes

Output Created		15-DEC-2021 13:07:57
Comments		
Input	Data	C:\Users\njs5478\Dropbox\HM and COVID\0. Revise and Resubmit\2. R and R Data\Study 1b\Study1b_Data.sav
	Active Dataset	DataSet1
	Filter	<none>
	Weight	<none>
	Split File	<none>
	N of Rows in Working Data File	241
Missing Value Handling	Definition of Missing	User-defined missing values are treated as missing.
	Cases Used	Statistics are based on cases with no missing values for any variable used.
Syntax		REGRESSION /MISSING LISTWISE /STATISTICS COEFF OUTS R ANOVA CHANGE ZPP /CRITERIA=PIN(.05) POUT(.10) /NOORIGIN /DEPENDENT RepCongressX /METHOD=ENTER Ideology0 /METHOD=ENTER GenderCC RaceCC SES0 /METHOD=ENTER MRN0 /METHOD=ENTER MRN0xRace MRN0xSES0 MRN0xGender MRN0xIdeology0.
Resources	Processor Time	00:00:00.03
	Elapsed Time	00:00:00.03

Notes

	Memory Required	45472 bytes
	Additional Memory Required for Residual Plots	0 bytes

Variables Entered/Removed[a]

Model	Variables Entered	Variables Removed	Method
1	Ideology0[b]	.	Enter
2	SES0, GenderCC, RaceCC[b]	.	Enter
3	MRN0[b]	.	Enter
4	MRN0xSES0, MRN0xIdeology0, MRN0xGender, MRN0xRace[b]	.	Enter

a. Dependent Variable: RepCongressX

b. All requested variables entered.

Model Summary

Model	R	R Square	Adjusted R Square	Std. Error of the Estimate	Change Statistics		
					R Square Change	F Change	df1
1	.724[a]	.525	.523	1.20092	.525	235.259	1
2	.733[b]	.537	.528	1.19378	.012	1.852	3
3	.734[c]	.539	.528	1.19390	.002	.957	1
4	.742[d]	.551	.531	1.18999	.012	1.344	4

Model Summary

Model	Change Statistics	
	df2	Sig. F Change
1	213	.000
2	210	.139
3	209	.329
4	205	.255

a. Predictors: (Constant), Ideology0

b. Predictors: (Constant), Ideology0, SES0, GenderCC, RaceCC

c. Predictors: (Constant), Ideology0, SES0, GenderCC, RaceCC, MRN0

d. Predictors: (Constant), Ideology0, SES0, GenderCC, RaceCC, MRN0, MRN0xSES0, MRN0xIdeology0, MRN0xGender, MRN0xRace

ANOVA[a]

Model		Sum of Squares	df	Mean Square	F	Sig.
1	Regression	339.293	1	339.293	235.259	.000[b]
	Residual	307.191	213	1.442		
	Total	646.484	214			
2	Regression	347.213	4	86.803	60.910	.000[c]
	Residual	299.271	210	1.425		
	Total	646.484	214			
3	Regression	348.577	5	69.715	48.910	.000[d]
	Residual	297.907	209	1.425		
	Total	646.484	214			
4	Regression	356.189	9	39.577	27.948	.000[e]
	Residual	290.295	205	1.416		
	Total	646.484	214			

a. Dependent Variable: RepCongressX

b. Predictors: (Constant), Ideology0

c. Predictors: (Constant), Ideology0, SES0, GenderCC, RaceCC

d. Predictors: (Constant), Ideology0, SES0, GenderCC, RaceCC, MRN0

e. Predictors: (Constant), Ideology0, SES0, GenderCC, RaceCC, MRN0, MRN0xSES0, MRN0xIdeology0, MRN0xGender, MRN0xRace

Coefficients[a]

Model		Unstandardized Coefficients B	Unstandardized Coefficients Std. Error	Standardized Coefficients Beta	t	Sig.
1	(Constant)	3.383	.082		41.305	.000
	Ideology0	.788	.051	.724	15.338	.000
2	(Constant)	3.271	.095		34.511	.000
	Ideology0	.749	.056	.689	13.327	.000
	GenderCC	.003	.085	.002	.031	.975
	RaceCC	.227	.099	.114	2.299	.022
	SES0	-.055	.098	-.027	-.564	.574
3	(Constant)	3.265	.095		34.362	.000
	Ideology0	.718	.065	.660	11.079	.000
	GenderCC	-.028	.091	-.016	-.315	.753
	RaceCC	.239	.100	.120	2.404	.017
	SES0	-.064	.098	-.031	-.646	.519
	MRN0	.118	.120	.059	.978	.329
4	(Constant)	3.190	.110		29.032	.000
	Ideology0	.695	.067	.639	10.430	.000
	GenderCC	-.024	.093	-.014	-.254	.800
	RaceCC	.237	.103	.119	2.315	.022
	SES0	-.042	.099	-.020	-.421	.674
	MRN0	.087	.133	.044	.657	.512
	MRN0xRace	.058	.114	.029	.510	.610
	MRN0xSES0	-.139	.105	-.065	-1.326	.186
	MRN0xGender	.075	.110	.034	.681	.496
	MRN0xIdeology0	.073	.058	.067	1.273	.204

Coefficients[a]

Model		Correlations Zero-order	Partial	Part
1	(Constant)			
	Ideology0	.724	.724	.724
2	(Constant)			
	Ideology0	.724	.677	.626
	GenderCC	.193	.002	.001
	RaceCC	.336	.157	.108
	SES0	.035	-.039	-.026
3	(Constant)			
	Ideology0	.724	.608	.520
	GenderCC	.193	-.022	-.015
	RaceCC	.336	.164	.113
	SES0	.035	-.045	-.030
	MRN0	.416	.068	.046
4	(Constant)			
	Ideology0	.724	.589	.488
	GenderCC	.193	-.018	-.012
	RaceCC	.336	.160	.108
	SES0	.035	-.029	-.020
	MRN0	.416	.046	.031
	MRN0xRace	.319	.036	.024
	MRN0xSES0	-.171	-.092	-.062
	MRN0xGender	.141	.048	.032
	MRN0xIdeology0	.111	.089	.060

a. Dependent Variable: RepCongressX

Excluded Variables[a]

Model		Beta In	t	Sig.	Partial Correlation	Collinearity Statistics Tolerance
1	GenderCC	-.005[b]	-.096	.924	-.007	.926
	RaceCC	.114[b]	2.298	.023	.156	.895
	SES0	-.024[b]	-.507	.612	-.035	.993
	MRN0	.031[b]	.558	.577	.038	.705
	MRN0xRace	.047[b]	.923	.357	.063	.852
	MRN0xSES0	-.072[b]	-1.507	.133	-.103	.981
	MRN0xGender	.047[b]	.990	.323	.068	.983
	MRN0xIdeology0	.077[b]	1.641	.102	.112	.998
2	MRN0	.059[c]	.978	.329	.068	.602
	MRN0xRace	.069[c]	1.341	.181	.092	.823
	MRN0xSES0	-.070[c]	-1.477	.141	-.102	.977
	MRN0xGender	.064[c]	1.336	.183	.092	.962
	MRN0xIdeology0	.064[c]	1.343	.181	.093	.959
3	MRN0xRace	.060[d]	1.118	.265	.077	.764
	MRN0xSES0	-.064[d]	-1.319	.189	-.091	.945
	MRN0xGender	.057[d]	1.180	.239	.082	.932
	MRN0xIdeology0	.076[d]	1.564	.119	.108	.923

a. Dependent Variable: RepCongressX

b. Predictors in the Model: (Constant), Ideology0

c. Predictors in the Model: (Constant), Ideology0, SES0, GenderCC, RaceCC

d. Predictors in the Model: (Constant), Ideology0, SES0, GenderCC, RaceCC, MRN0

```
REGRESSION
 /MISSING LISTWISE
 /STATISTICS COEFF OUTS R ANOVA CHANGE ZPP
 /CRITERIA=PIN(.05) POUT(.10)
 /NOORIGIN
 /DEPENDENT DemCongressX
 /METHOD=ENTER Ideology0
 /METHOD=ENTER GenderCC RaceCC SES0
 /METHOD=ENTER MRN0
 /METHOD=ENTER MRN0xRace MRN0xSES0 MRN0xGender MRN0xIdeology0.
```

Regression

Notes

Output Created		15-DEC-2021 13:07:57
Comments		
Input	Data	C: \Users\njs5478\Dropbox\HM and COVID\0. Revise and Resubmit\2. R and R Data\Study 1b\Study1b_Data.sav
	Active Dataset	DataSet1
	Filter	<none>
	Weight	<none>
	Split File	<none>
	N of Rows in Working Data File	241
Missing Value Handling	Definition of Missing	User-defined missing values are treated as missing.
	Cases Used	Statistics are based on cases with no missing values for any variable used.
Syntax		REGRESSION<br>/MISSING LISTWISE<br>/STATISTICS COEFF OUTS R ANOVA CHANGE ZPP<br>/CRITERIA=PIN(.05) POUT(.10)<br>/NOORIGIN<br>/DEPENDENT DemCongressX<br>/METHOD=ENTER Ideology0<br>/METHOD=ENTER GenderCC RaceCC SES0<br>/METHOD=ENTER MRN0<br>/METHOD=ENTER MRN0xRace MRN0xSES0 MRN0xGender MRN0xIdeology0.
Resources	Processor Time	00:00:00.02
	Elapsed Time	00:00:00.02

Notes

	Memory Required	45472 bytes
	Additional Memory Required for Residual Plots	0 bytes

Variables Entered/Removed[a]

Model	Variables Entered	Variables Removed	Method
1	Ideology0[b]	.	Enter
2	SES0, GenderCC, RaceCC[b]	.	Enter
3	MRN0[b]	.	Enter
4	MRN0xSES0, MRN0xGender, MRN0xIdeology0, MRN0xRace[b]	.	Enter

a. Dependent Variable: DemCongressX

b. All requested variables entered.

Model Summary

Model	R	R Square	Adjusted R Square	Std. Error of the Estimate	Change Statistics R Square Change	F Change	df1
1	.606[a]	.367	.364	1.26740	.367	122.251	1
2	.611[b]	.374	.361	1.26977	.007	.738	3
3	.612[c]	.374	.359	1.27188	.001	.310	1
4	.641[d]	.411	.385	1.24602	.037	3.171	4

Model Summary

Model	Change Statistics df2	Sig. F Change
1	211	.000
2	208	.530
3	207	.578
4	203	.015

a. Predictors: (Constant), Ideology0

b. Predictors: (Constant), Ideology0, SES0, GenderCC, RaceCC

c. Predictors: (Constant), Ideology0, SES0, GenderCC, RaceCC, MRN0

d. Predictors: (Constant), Ideology0, SES0, GenderCC, RaceCC, MRN0, MRN0xSES0, MRN0xGender, MRN0xIdeology0, MRN0xRace

ANOVA[a]

Model		Sum of Squares	df	Mean Square	F	Sig.
1	Regression	196.373	1	196.373	122.251	.000[b]
	Residual	338.932	211	1.606		
	Total	535.305	212			
2	Regression	199.945	4	49.986	31.003	.000[c]
	Residual	335.360	208	1.612		
	Total	535.305	212			
3	Regression	200.446	5	40.089	24.782	.000[d]
	Residual	334.859	207	1.618		
	Total	535.305	212			
4	Regression	220.137	9	24.460	15.754	.000[e]
	Residual	315.168	203	1.553		
	Total	535.305	212			

a. Dependent Variable: DemCongressX

b. Predictors: (Constant), Ideology0

c. Predictors: (Constant), Ideology0, SES0, GenderCC, RaceCC

d. Predictors: (Constant), Ideology0, SES0, GenderCC, RaceCC, MRN0

e. Predictors: (Constant), Ideology0, SES0, GenderCC, RaceCC, MRN0, MRN0xSES0, MRN0xGender, MRN0xIdeology0, MRN0xRace

Coefficients[a]

Model		Unstandardized Coefficients B	Unstandardized Coefficients Std. Error	Standardized Coefficients Beta	t	Sig.
1	(Constant)	4.243	.087		48.855	.000
	Ideology0	-.604	.055	-.606	-11.057	.000
2	(Constant)	4.180	.101		41.416	.000
	Ideology0	-.619	.061	-.620	-10.216	.000
	GenderCC	-.060	.091	-.038	-.666	.506
	RaceCC	.131	.106	.072	1.238	.217
	SES0	.035	.104	.018	.332	.740
3	(Constant)	4.184	.101		41.287	.000
	Ideology0	-.600	.069	-.602	-8.666	.000
	GenderCC	-.041	.098	-.026	-.419	.676
	RaceCC	.124	.106	.068	1.164	.246
	SES0	.040	.105	.021	.376	.707
	MRN0	-.072	.129	-.039	-.557	.578
4	(Constant)	4.357	.115		37.832	.000
	Ideology0	-.596	.070	-.597	-8.524	.000
	GenderCC	-.064	.098	-.040	-.654	.514
	RaceCC	.101	.107	.056	.938	.349
	SES0	.042	.104	.022	.403	.688
	MRN0	-.029	.139	-.016	-.209	.835
	MRN0xRace	.081	.120	.044	.673	.502
	MRN0xSES0	.030	.110	.016	.276	.783
	MRN0xGender	-.390	.117	-.191	-3.345	.001
	MRN0xIdeology0	-.025	.061	-.024	-.402	.688

Coefficients[a]

Model		Correlations Zero-order	Partial	Part
1	(Constant)			
	Ideology0	-.606	-.606	-.606
2	(Constant)			
	Ideology0	-.606	-.578	-.561
	GenderCC	-.208	-.046	-.037
	RaceCC	-.132	.086	.068
	SES0	-.027	.023	.018
3	(Constant)			
	Ideology0	-.606	-.516	-.476
	GenderCC	-.208	-.029	-.023
	RaceCC	-.132	.081	.064
	SES0	-.027	.026	.021
	MRN0	-.365	-.039	-.031
4	(Constant)			
	Ideology0	-.606	-.513	-.459
	GenderCC	-.208	-.046	-.035
	RaceCC	-.132	.066	.051
	SES0	-.027	.028	.022
	MRN0	-.365	-.015	-.011
	MRN0xRace	-.240	.047	.036
	MRN0xSES0	.122	.019	.015
	MRN0xGender	-.262	-.229	-.180
	MRN0xIdeology0	-.074	-.028	-.022

a. Dependent Variable: DemCongressX

Excluded Variables[a]

Model		Beta In	t	Sig.	Partial Correlation	Collinearity Statistics Tolerance
1	GenderCC	-.042[b]	-.742	.459	-.051	.922
	RaceCC	.075[b]	1.295	.197	.089	.892
	SES0	.020[b]	.355	.723	.024	.994
	MRN0	-.058[b]	-.892	.373	-.061	.714
	MRN0xRace	-.016[b]	-.270	.787	-.019	.861
	MRN0xSES0	.039[b]	.713	.476	.049	.981
	MRN0xGender	-.197[b]	-3.676	.000	-.246	.988
	MRN0xIdeology0	-.035[b]	-.634	.527	-.044	.996
2	MRN0	-.039[c]	-.557	.578	-.039	.606
	MRN0xRace	.000[c]	-.005	.996	.000	.832
	MRN0xSES0	.043[c]	.775	.439	.054	.978
	MRN0xGender	-.193[c]	-3.560	.000	-.240	.971
	MRN0xIdeology0	-.038[c]	-.676	.500	-.047	.963
3	MRN0xRace	.009[d]	.144	.886	.010	.776
	MRN0xSES0	.039[d]	.685	.494	.048	.946
	MRN0xGender	-.193[d]	-3.505	.001	-.237	.946
	MRN0xIdeology0	-.045[d]	-.788	.432	-.055	.932

a. Dependent Variable: DemCongressX

b. Predictors in the Model: (Constant), Ideology0

c. Predictors in the Model: (Constant), Ideology0, SES0, GenderCC, RaceCC

d. Predictors in the Model: (Constant), Ideology0, SES0, GenderCC, RaceCC, MRN0

```
REGRESSION
 /MISSING LISTWISE
 /STATISTICS COEFF OUTS R ANOVA CHANGE ZPP
 /CRITERIA=PIN(.05) POUT(.10)
 /NOORIGIN
 /DEPENDENT StateX
 /METHOD=ENTER Ideology0
 /METHOD=ENTER GenderCC RaceCC SES0
 /METHOD=ENTER MRN0
 /METHOD=ENTER MRN0xRace MRN0xSES0 MRN0xGender MRN0xIdeology0.
```

Regression

Notes

Output Created		15-DEC-2021 13:07:58
Comments		
Input	Data	C:<br>\Users\njs5478\Dropbox\HM and COVID\0. Revise and Resubmit\2. R and R Data\Study 1b\Study1b_Data.sav
	Active Dataset	DataSet1
	Filter	<none>
	Weight	<none>
	Split File	<none>
	N of Rows in Working Data File	241
Missing Value Handling	Definition of Missing	User-defined missing values are treated as missing.
	Cases Used	Statistics are based on cases with no missing values for any variable used.
Syntax		REGRESSION<br>/MISSING LISTWISE<br>/STATISTICS COEFF OUTS R ANOVA CHANGE ZPP<br>/CRITERIA=PIN(.05) POUT(.10)<br>/NOORIGIN<br>/DEPENDENT StateX<br>/METHOD=ENTER Ideology0<br>/METHOD=ENTER GenderCC RaceCC SES0<br>/METHOD=ENTER MRN0<br>/METHOD=ENTER MRN0xRace MRN0xSES0 MRN0xGender MRN0xIdeology0.
Resources	Processor Time	00:00:00.02
	Elapsed Time	00:00:00.02

Notes

	Memory Required	45472 bytes
	Additional Memory Required for Residual Plots	0 bytes

Variables Entered/Removed[a]

Model	Variables Entered	Variables Removed	Method
1	Ideology0[b]	.	Enter
2	SES0, GenderCC, RaceCC[b]	.	Enter
3	MRN0[b]	.	Enter
4	MRN0xSES0, MRN0xGender, MRN0xIdeology0, MRN0xRace[b]	.	Enter

a. Dependent Variable: StateX

b. All requested variables entered.

Model Summary

Model	R	R Square	Adjusted R Square	Std. Error of the Estimate	Change Statistics		
					R Square Change	F Change	df1
1	.301[a]	.090	.086	1.53052	.090	21.955	1
2	.303[b]	.092	.075	1.54002	.001	.094	3
3	.303[c]	.092	.071	1.54355	.000	.004	1
4	.365[d]	.133	.096	1.52215	.041	2.536	4

Model Summary

Model	Change Statistics	
	df2	Sig. F Change
1	221	.000
2	218	.963
3	217	.952
4	213	.041

a. Predictors: (Constant), Ideology0

b. Predictors: (Constant), Ideology0, SES0, GenderCC, RaceCC

c. Predictors: (Constant), Ideology0, SES0, GenderCC, RaceCC, MRN0

d. Predictors: (Constant), Ideology0, SES0, GenderCC, RaceCC, MRN0, MRN0xSES0, MRN0xGender, MRN0xIdeology0, MRN0xRace

ANOVA[a]

Model		Sum of Squares	df	Mean Square	F	Sig.
1	Regression	51.430	1	51.430	21.955	.000[b]
	Residual	517.691	221	2.342		
	Total	569.121	222			
2	Regression	52.102	4	13.026	5.492	.000[c]
	Residual	517.019	218	2.372		
	Total	569.121	222			
3	Regression	52.111	5	10.422	4.374	.001[d]
	Residual	517.010	217	2.383		
	Total	569.121	222			
4	Regression	75.615	9	8.402	3.626	.000[e]
	Residual	493.507	213	2.317		
	Total	569.121	222			

a. Dependent Variable: StateX

b. Predictors: (Constant), Ideology0

c. Predictors: (Constant), Ideology0, SES0, GenderCC, RaceCC

d. Predictors: (Constant), Ideology0, SES0, GenderCC, RaceCC, MRN0

e. Predictors: (Constant), Ideology0, SES0, GenderCC, RaceCC, MRN0, MRN0xSES0, MRN0xGender, MRN0xIdeology0, MRN0xRace

Coefficients[a]

Model		Unstandardized Coefficients B	Unstandardized Coefficients Std. Error	Standardized Coefficients Beta	t	Sig.
1	(Constant)	4.069	.103		39.699	.000
	Ideology0	-.305	.065	-.301	-4.686	.000
2	(Constant)	4.101	.121		33.843	.000
	Ideology0	-.291	.072	-.287	-4.071	.000
	GenderCC	-.025	.107	-.016	-.235	.815
	RaceCC	-.062	.126	-.033	-.489	.625
	SES0	.007	.126	.004	.058	.954
3	(Constant)	4.101	.122		33.672	.000
	Ideology0	-.289	.083	-.284	-3.487	.001
	GenderCC	-.023	.114	-.014	-.201	.841
	RaceCC	-.063	.128	-.034	-.492	.623
	SES0	.008	.127	.004	.063	.950
	MRN0	-.009	.152	-.005	-.061	.952
4	(Constant)	4.183	.136		30.666	.000
	Ideology0	-.297	.085	-.293	-3.515	.001
	GenderCC	-.086	.116	-.054	-.739	.461
	RaceCC	-.111	.130	-.060	-.853	.395
	SES0	.037	.126	.019	.291	.771
	MRN0	.086	.167	.047	.514	.607
	MRN0xRace	.098	.146	.053	.669	.505
	MRN0xSES0	.047	.133	.023	.352	.725
	MRN0xGender	-.381	.137	-.189	-2.783	.006
	MRN0xIdeology0	.102	.072	.102	1.424	.156

Coefficients[a]

Model		Correlations Zero-order	Partial	Part
1	(Constant)			
	Ideology0	-.301	-.301	-.301
2	(Constant)			
	Ideology0	-.301	-.266	-.263
	GenderCC	-.091	-.016	-.015
	RaceCC	-.121	-.033	-.032
	SES0	-.022	.004	.004
3	(Constant)			
	Ideology0	-.301	-.230	-.226
	GenderCC	-.091	-.014	-.013
	RaceCC	-.121	-.033	-.032
	SES0	-.022	.004	.004
	MRN0	-.164	-.004	-.004
4	(Constant)			
	Ideology0	-.301	-.234	-.224
	GenderCC	-.091	-.051	-.047
	RaceCC	-.121	-.058	-.054
	SES0	-.022	.020	.019
	MRN0	-.164	.035	.033
	MRN0xRace	-.075	.046	.043
	MRN0xSES0	.091	.024	.022
	MRN0xGender	-.196	-.187	-.178
	MRN0xIdeology0	.056	.097	.091

a. Dependent Variable: StateX

Excluded Variables[a]

Model		Beta In	t	Sig.	Partial Correlation	Collinearity Statistics Tolerance
1	GenderCC	-.014[b]	-.205	.838	-.014	.933
	RaceCC	-.032[b]	-.477	.634	-.032	.907
	SES0	.003[b]	.042	.967	.003	.993
	MRN0	-.004[b]	-.052	.958	-.004	.713
	MRN0xRace	.064[b]	.897	.371	.060	.821
	MRN0xSES0	.051[b]	.789	.431	.053	.981
	MRN0xGender	-.155[b]	-2.421	.016	-.161	.979
	MRN0xIdeology0	.074[b]	1.148	.252	.077	.997
2	MRN0	-.005[c]	-.061	.952	-.004	.613
	MRN0xRace	.061[c]	.838	.403	.057	.798
	MRN0xSES0	.051[c]	.786	.433	.053	.978
	MRN0xGender	-.163[c]	-2.503	.013	-.168	.961
	MRN0xIdeology0	.084[c]	1.272	.205	.086	.949
3	MRN0xRace	.069[d]	.902	.368	.061	.718
	MRN0xSES0	.052[d]	.785	.433	.053	.950
	MRN0xGender	-.168[d]	-2.534	.012	-.170	.925
	MRN0xIdeology0	.086[d]	1.276	.203	.086	.924

a. Dependent Variable: StateX

b. Predictors in the Model: (Constant), Ideology0

c. Predictors in the Model: (Constant), Ideology0, SES0, GenderCC, RaceCC

d. Predictors in the Model: (Constant), Ideology0, SES0, GenderCC, RaceCC, MRN0

```
REGRESSION
 /MISSING LISTWISE
 /STATISTICS COEFF OUTS R ANOVA CHANGE ZPP
 /CRITERIA=PIN(.05) POUT(.10)
 /NOORIGIN
 /DEPENDENT Risk_Rules
 /METHOD=ENTER Ideology0
 /METHOD=ENTER GenderCC RaceCC SES0
 /METHOD=ENTER MRN0
 /METHOD=ENTER MRN0xRace MRN0xSES0 MRN0xGender MRN0xIdeology0.
```

Regression

Notes

Output Created		15-DEC-2021 13:07:58
Comments		
Input	Data	C: \Users\njs5478\Dropbox\HM and COVID\0. Revise and Resubmit\2. R and R Data\Study 1b\Study1b_Data.sav
	Active Dataset	DataSet1
	Filter	<none>
	Weight	<none>
	Split File	<none>
	N of Rows in Working Data File	241
Missing Value Handling	Definition of Missing	User-defined missing values are treated as missing.
	Cases Used	Statistics are based on cases with no missing values for any variable used.
Syntax		REGRESSION /MISSING LISTWISE /STATISTICS COEFF OUTS R ANOVA CHANGE ZPP /CRITERIA=PIN(.05) POUT(.10) /NOORIGIN /DEPENDENT Risk_Rules /METHOD=ENTER Ideology0 /METHOD=ENTER GenderCC RaceCC SES0 /METHOD=ENTER MRN0 /METHOD=ENTER MRN0xRace MRN0xSES0 MRN0xGender MRN0xIdeology0.
Resources	Processor Time	00:00:00.03
	Elapsed Time	00:00:00.03

Notes

	Memory Required	45472 bytes
	Additional Memory Required for Residual Plots	0 bytes

Variables Entered/Removed[a]

Model	Variables Entered	Variables Removed	Method
1	Ideology0[b]	.	Enter
2	SES0, GenderCC, RaceCC[b]	.	Enter
3	MRN0[b]	.	Enter
4	MRN0xSES0, MRN0xGender, MRN0xIdeology0, MRN0xRace[b]	.	Enter

a. Dependent Variable: Risk_Rules

b. All requested variables entered.

Model Summary

Model	R	R Square	Adjusted R Square	Std. Error of the Estimate	Change Statistics		
					R Square Change	F Change	df1
1	.474[a]	.225	.222	.88216	.225	68.457	1
2	.484[b]	.235	.221	.88224	.010	.987	3
3	.551[c]	.304	.289	.84301	.070	23.186	1
4	.563[d]	.317	.290	.84243	.013	1.080	4

Model Summary

Model	Change Statistics	
	df2	Sig. F Change
1	236	.000
2	233	.400
3	232	.000
4	228	.367

a. Predictors: (Constant), Ideology0

b. Predictors: (Constant), Ideology0, SES0, GenderCC, RaceCC

c. Predictors: (Constant), Ideology0, SES0, GenderCC, RaceCC, MRN0

d. Predictors: (Constant), Ideology0, SES0, GenderCC, RaceCC, MRN0, MRN0xSES0, MRN0xGender, MRN0xIdeology0, MRN0xRace

ANOVA[a]

Model		Sum of Squares	df	Mean Square	F	Sig.
1	Regression	53.274	1	53.274	68.457	.000[b]
	Residual	183.657	236	.778		
	Total	236.931	237			
2	Regression	55.578	4	13.894	17.851	.000[c]
	Residual	181.353	233	.778		
	Total	236.931	237			
3	Regression	72.055	5	14.411	20.278	.000[d]
	Residual	164.875	232	.711		
	Total	236.931	237			
4	Regression	75.121	9	8.347	11.761	.000[e]
	Residual	161.810	228	.710		
	Total	236.931	237			

a. Dependent Variable: Risk_Rules

b. Predictors: (Constant), Ideology0

c. Predictors: (Constant), Ideology0, SES0, GenderCC, RaceCC

d. Predictors: (Constant), Ideology0, SES0, GenderCC, RaceCC, MRN0

e. Predictors: (Constant), Ideology0, SES0, GenderCC, RaceCC, MRN0, MRN0xSES0, MRN0xGender, MRN0xIdeology0, MRN0xRace

Coefficients[a]

Model		Unstandardized Coefficients B	Unstandardized Coefficients Std. Error	Standardized Coefficients Beta	t	Sig.
1	(Constant)	3.303	.057		57.758	.000
	Ideology0	.298	.036	.474	8.274	.000
2	(Constant)	3.293	.067		49.239	.000
	Ideology0	.280	.040	.445	7.066	.000
	GenderCC	.098	.059	.098	1.644	.101
	RaceCC	.021	.070	.018	.299	.765
	SES0	-.038	.071	-.030	-.529	.597
3	(Constant)	3.269	.064		51.004	.000
	Ideology0	.173	.044	.275	3.938	.000
	GenderCC	.001	.060	.001	.010	.992
	RaceCC	.071	.067	.061	1.045	.297
	SES0	-.072	.068	-.058	-1.050	.295
	MRN0	.391	.081	.339	4.815	.000
4	(Constant)	3.252	.073		44.585	.000
	Ideology0	.153	.045	.244	3.395	.001
	GenderCC	.022	.062	.022	.353	.725
	RaceCC	.091	.070	.079	1.302	.194
	SES0	-.068	.069	-.055	-.985	.326
	MRN0	.320	.090	.277	3.535	.000
	MRN0xRace	.123	.079	.106	1.561	.120
	MRN0xSES0	-.073	.073	-.057	-1.004	.317
	MRN0xGender	.047	.073	.037	.637	.525
	MRN0xIdeology0	-.011	.039	-.018	-.285	.776

Coefficients[a]

Model		Correlations Zero-order	Partial	Part
1	(Constant)			
	Ideology0	.474	.474	.474
2	(Constant)			
	Ideology0	.474	.420	.405
	GenderCC	.213	.107	.094
	RaceCC	.166	.020	.017
	SES0	.001	-.035	-.030
3	(Constant)			
	Ideology0	.474	.250	.216
	GenderCC	.213	.001	.001
	RaceCC	.166	.068	.057
	SES0	.001	-.069	-.058
	MRN0	.483	.301	.264
4	(Constant)			
	Ideology0	.474	.219	.186
	GenderCC	.213	.023	.019
	RaceCC	.166	.086	.071
	SES0	.001	-.065	-.054
	MRN0	.483	.228	.193
	MRN0xRace	.340	.103	.085
	MRN0xSES0	-.159	-.066	-.055
	MRN0xGender	.128	.042	.035
	MRN0xIdeology0	.000	-.019	-.016

a. Dependent Variable: Risk_Rules

Excluded Variables[a]

Model		Beta In	t	Sig.	Partial Correlation	Collinearity Statistics Tolerance
1	GenderCC	.096[b]	1.617	.107	.105	.932
	RaceCC	.012[b]	.198	.843	.013	.893
	SES0	-.027[b]	-.461	.645	-.030	.997
	MRN0	.321[b]	4.940	.000	.307	.709
	MRN0xRace	.180[b]	2.920	.004	.187	.842
	MRN0xSES0	-.099[b]	-1.715	.088	-.111	.983
	MRN0xGender	.085[b]	1.478	.141	.096	.991
	MRN0xIdeology0	-.015[b]	-.267	.789	-.017	.999
2	MRN0	.339[c]	4.815	.000	.301	.606
	MRN0xRace	.185[c]	2.952	.003	.190	.812
	MRN0xSES0	-.105[c]	-1.821	.070	-.119	.979
	MRN0xGender	.092[c]	1.597	.112	.104	.973
	MRN0xIdeology0	-.029[c]	-.495	.621	-.032	.961
3	MRN0xRace	.104[d]	1.636	.103	.107	.734
	MRN0xSES0	-.062[d]	-1.106	.270	-.073	.951
	MRN0xGender	.049[d]	.871	.385	.057	.946
	MRN0xIdeology0	.016[d]	.281	.779	.019	.934

a. Dependent Variable: Risk_Rules

b. Predictors in the Model: (Constant), Ideology0

c. Predictors in the Model: (Constant), Ideology0, SES0, GenderCC, RaceCC

d. Predictors in the Model: (Constant), Ideology0, SES0, GenderCC, RaceCC, MRN0

```
REGRESSION
 /MISSING LISTWISE
 /STATISTICS COEFF OUTS R ANOVA CHANGE ZPP
 /CRITERIA=PIN(.05) POUT(.10)
 /NOORIGIN
 /DEPENDENT Risk_Help
 /METHOD=ENTER Ideology0
 /METHOD=ENTER GenderCC RaceCC SES0
 /METHOD=ENTER MRN0
 /METHOD=ENTER MRN0xRace MRN0xSES0 MRN0xGender MRN0xIdeology0.
```

Regression

Notes

Output Created		15-DEC-2021 13:07:58
Comments		
Input	Data	C:\Users\njs5478\Dropbox\HM and COVID\0. Revise and Resubmit\2. R and R Data\Study 1b\Study1b_Data.sav
	Active Dataset	DataSet1
	Filter	<none>
	Weight	<none>
	Split File	<none>
	N of Rows in Working Data File	241
Missing Value Handling	Definition of Missing	User-defined missing values are treated as missing.
	Cases Used	Statistics are based on cases with no missing values for any variable used.
Syntax		REGRESSION<br>/MISSING LISTWISE<br>/STATISTICS COEFF OUTS R ANOVA CHANGE ZPP<br>/CRITERIA=PIN(.05) POUT(.10)<br>/NOORIGIN<br>/DEPENDENT Risk_Help<br>/METHOD=ENTER Ideology0<br>/METHOD=ENTER GenderCC RaceCC SES0<br>/METHOD=ENTER MRN0<br>/METHOD=ENTER MRN0xRace MRN0xSES0 MRN0xGender MRN0xIdeology0.
Resources	Processor Time	00:00:00.02
	Elapsed Time	00:00:00.05

Notes

	Memory Required	45472 bytes
	Additional Memory Required for Residual Plots	0 bytes

Variables Entered/Removed[a]

Model	Variables Entered	Variables Removed	Method
1	Ideology0[b]	.	Enter
2	SES0, GenderCC, RaceCC[b]	.	Enter
3	MRN0[b]	.	Enter
4	MRN0xSES0, MRN0xGender, MRN0xIdeology0, MRN0xRace[b]	.	Enter

a. Dependent Variable: Risk_Help

b. All requested variables entered.

Model Summary

Model	R	R Square	Adjusted R Square	Std. Error of the Estimate	Change Statistics		
					R Square Change	F Change	df1
1	.159[a]	.025	.021	1.49099	.025	6.084	1
2	.273[b]	.074	.059	1.46212	.049	4.137	3
3	.273[c]	.075	.055	1.46500	.000	.086	1
4	.303[d]	.092	.056	1.46394	.017	1.084	4

Model Summary

Model	Change Statistics	
	df2	Sig. F Change
1	236	.014
2	233	.007
3	232	.770
4	228	.365

a. Predictors: (Constant), Ideology0

b. Predictors: (Constant), Ideology0, SES0, GenderCC, RaceCC

c. Predictors: (Constant), Ideology0, SES0, GenderCC, RaceCC, MRN0

d. Predictors: (Constant), Ideology0, SES0, GenderCC, RaceCC, MRN0, MRN0xSES0, MRN0xGender, MRN0xIdeology0, MRN0xRace

ANOVA[a]

Model		Sum of Squares	df	Mean Square	F	Sig.
1	Regression	13.526	1	13.526	6.084	.014[b]
	Residual	524.637	236	2.223		
	Total	538.163	237			
2	Regression	40.058	4	10.015	4.685	.001[c]
	Residual	498.105	233	2.138		
	Total	538.163	237			
3	Regression	40.242	5	8.048	3.750	.003[d]
	Residual	497.921	232	2.146		
	Total	538.163	237			
4	Regression	49.534	9	5.504	2.568	.008[e]
	Residual	488.628	228	2.143		
	Total	538.163	237			

a. Dependent Variable: Risk_Help

b. Predictors: (Constant), Ideology0

c. Predictors: (Constant), Ideology0, SES0, GenderCC, RaceCC

d. Predictors: (Constant), Ideology0, SES0, GenderCC, RaceCC, MRN0

e. Predictors: (Constant), Ideology0, SES0, GenderCC, RaceCC, MRN0, MRN0xSES0, MRN0xGender, MRN0xIdeology0, MRN0xRace

Coefficients[a]

Model		Unstandardized Coefficients B	Std. Error	Standardized Coefficients Beta	t	Sig.
1	(Constant)	3.802	.097		39.338	.000
	Ideology0	-.150	.061	-.159	-2.467	.014
2	(Constant)	3.939	.111		35.547	.000
	Ideology0	-.071	.066	-.074	-1.076	.283
	GenderCC	-.132	.098	-.088	-1.340	.182
	RaceCC	-.285	.116	-.164	-2.460	.015
	SES0	-.250	.118	-.134	-2.121	.035
3	(Constant)	3.937	.111		35.349	.000
	Ideology0	-.082	.076	-.086	-1.074	.284
	GenderCC	-.142	.105	-.094	-1.358	.176
	RaceCC	-.279	.117	-.161	-2.382	.018
	SES0	-.253	.119	-.136	-2.136	.034
	MRN0	.041	.141	.024	.292	.770
4	(Constant)	3.958	.127		31.228	.000
	Ideology0	-.049	.079	-.052	-.627	.532
	GenderCC	-.160	.107	-.107	-1.493	.137
	RaceCC	-.290	.121	-.168	-2.394	.017
	SES0	-.273	.119	-.146	-2.283	.023
	MRN0	.123	.157	.071	.782	.435
	MRN0xRace	-.187	.137	-.108	-1.372	.171
	MRN0xSES0	.155	.127	.080	1.223	.223
	MRN0xGender	.010	.128	.005	.076	.939
	MRN0xIdeology0	-.035	.068	-.036	-.511	.610

Coefficients[a]

Model		Correlations Zero-order	Partial	Part
1	(Constant)			
	Ideology0	-.159	-.159	-.159
2	(Constant)			
	Ideology0	-.159	-.070	-.068
	GenderCC	-.119	-.087	-.084
	RaceCC	-.196	-.159	-.155
	SES0	-.149	-.138	-.134
3	(Constant)			
	Ideology0	-.159	-.070	-.068
	GenderCC	-.119	-.089	-.086
	RaceCC	-.196	-.154	-.150
	SES0	-.149	-.139	-.135
	MRN0	-.086	.019	.018
4	(Constant)			
	Ideology0	-.159	-.041	-.040
	GenderCC	-.119	-.098	-.094
	RaceCC	-.196	-.157	-.151
	SES0	-.149	-.149	-.144
	MRN0	-.086	.052	.049
	MRN0xRace	-.121	-.091	-.087
	MRN0xSES0	.087	.081	.077
	MRN0xGender	-.013	.005	.005
	MRN0xIdeology0	-.073	-.034	-.032

a. Dependent Variable: Risk_Help

Excluded Variables[a]

Model		Beta In	t	Sig.	Partial Correlation	Collinearity Statistics Tolerance
1	GenderCC	-.084[b]	-1.261	.209	-.082	.932
	RaceCC	-.162[b]	-2.404	.017	-.155	.893
	SES0	-.140[b]	-2.192	.029	-.142	.997
	MRN0	-.001[b]	-.018	.986	-.001	.709
	MRN0xRace	-.069[b]	-.980	.328	-.064	.842
	MRN0xSES0	.067[b]	1.041	.299	.068	.983
	MRN0xGender	.001[b]	.019	.985	.001	.991
	MRN0xIdeology0	-.068[b]	-1.057	.292	-.069	.999
2	MRN0	.024[c]	.292	.770	.019	.606
	MRN0xRace	-.103[c]	-1.476	.141	-.096	.812
	MRN0xSES0	.071[c]	1.110	.268	.073	.979
	MRN0xGender	-.016[c]	-.255	.799	-.017	.973
	MRN0xIdeology0	-.061[c]	-.948	.344	-.062	.961
3	MRN0xRace	-.121[d]	-1.647	.101	-.108	.734
	MRN0xSES0	.076[d]	1.174	.242	.077	.951
	MRN0xGender	-.020[d]	-.308	.759	-.020	.946
	MRN0xIdeology0	-.059[d]	-.910	.364	-.060	.934

a. Dependent Variable: Risk_Help

b. Predictors in the Model: (Constant), Ideology0

c. Predictors in the Model: (Constant), Ideology0, SES0, GenderCC, RaceCC

d. Predictors in the Model: (Constant), Ideology0, SES0, GenderCC, RaceCC, MRN0

```
**Regression analyses including Precarious Masculinity (with PParty)

REGRESSION
  /MISSING LISTWISE
  /STATISTICS COEFF OUTS R ANOVA CHANGE ZPP
  /CRITERIA=PIN(.05) POUT(.10)
  /NOORIGIN
  /DEPENDENT Concern_Tot
  /METHOD=ENTER Party0
  /METHOD=ENTER GenderCC RaceCC SES0
```

```
  /METHOD=ENTER MGRS0 MRN0
  /METHOD=ENTER MRN0xRace MRN0xSES0 MRN0xGender MRN0xParty0 MRN0xMGRS0 MGRS0xGender MGRS0
xRace MGRS0xSES0 MGRS0xParty0.
```

Regression

Notes

Output Created		15-DEC-2021 13:07:58
Comments		
Input	Data	C:\Users\njs5478\Dropbox\HM and COVID\0. Revise and Resubmit\2. R and R Data\Study 1b\Study1b_Data.sav
	Active Dataset	DataSet1
	Filter	<none>
	Weight	<none>
	Split File	<none>
	N of Rows in Working Data File	241
Missing Value Handling	Definition of Missing	User-defined missing values are treated as missing.
	Cases Used	Statistics are based on cases with no missing values for any variable used.

Notes

Syntax		REGRESSION<br>/MISSING LISTWISE<br>/STATISTICS COEFF OUTS R ANOVA CHANGE ZPP<br>/CRITERIA=PIN(.05) POUT(.10)<br>/NOORIGIN<br>/DEPENDENT Concern_Tot<br>/METHOD=ENTER Party0<br>/METHOD=ENTER GenderCC RaceCC SES0<br>/METHOD=ENTER MGRS0 MRN0<br>/METHOD=ENTER MRN0xRace MRN0xSES0 MRN0xGender MRN0xParty0 MRN0xMGRS0 MGRS0xGender MGRS0xRace MGRS0xSES0 MGRS0xParty0.
Resources	Processor Time	00:00:00.03
	Elapsed Time	00:00:00.03
	Memory Required	52240 bytes
	Additional Memory Required for Residual Plots	0 bytes

Variables Entered/Removed[a]

Model	Variables Entered	Variables Removed	Method
1	Party0[b]	.	Enter
2	SES0, GenderCC, RaceCC[b]	.	Enter
3	MGRS0, MRN0[b]	.	Enter
4	MRN0xMGRS0, MRN0xSES0, MRN0xParty0, MGRS0xSES0, MGRS0xGender, MGRS0xRace, MRN0xRace, MRN0xGender, ...[b]	.	Enter

a. Dependent Variable: Concern_Tot

b. All requested variables entered.

Model Summary

Model	R	R Square	Adjusted R Square	Std. Error of the Estimate	Change Statistics		
					R Square Change	F Change	df1
1	.434[a]	.188	.185	1.25740	.188	54.484	1
2	.459[b]	.211	.197	1.24791	.022	2.196	3
3	.500[c]	.250	.230	1.22187	.039	5.997	2
4	.565[d]	.319	.273	1.18739	.069	2.506	9

Model Summary

Model	Change Statistics df2	Sig. F Change
1	235	.000
2	232	.089
3	230	.003
4	221	.009

a. Predictors: (Constant), Party0

b. Predictors: (Constant), Party0, SES0, GenderCC, RaceCC

c. Predictors: (Constant), Party0, SES0, GenderCC, RaceCC, MGRS0, MRN0

d. Predictors: (Constant), Party0, SES0, GenderCC, RaceCC, MGRS0, MRN0, MRN0xMGRS0, MRN0xSES0, MRN0xParty0, MGRS0xSES0, MGRS0xGender, MGRS0xRace, MRN0xRace, MRN0xGender, MGRS0xParty0

ANOVA[a]

Model		Sum of Squares	df	Mean Square	F	Sig.
1	Regression	86.143	1	86.143	54.484	.000[b]
	Residual	371.551	235	1.581		
	Total	457.693	236			
2	Regression	96.404	4	24.101	15.476	.000[c]
	Residual	361.289	232	1.557		
	Total	457.693	236			
3	Regression	114.310	6	19.052	12.761	.000[d]
	Residual	343.383	230	1.493		
	Total	457.693	236			
4	Regression	146.108	15	9.741	6.909	.000[e]
	Residual	311.585	221	1.410		
	Total	457.693	236			

a. Dependent Variable: Concern_Tot

b. Predictors: (Constant), Party0

c. Predictors: (Constant), Party0, SES0, GenderCC, RaceCC

d. Predictors: (Constant), Party0, SES0, GenderCC, RaceCC, MGRS0, MRN0

e. Predictors: (Constant), Party0, SES0, GenderCC, RaceCC, MGRS0, MRN0, MRN0xMGRS0, MRN0xSES0, MRN0xParty0, MGRS0xSES0, MGRS0xGender, MGRS0xRace, MRN0xRace, MRN0xGender, MGRS0xParty0

Coefficients[a]

Model		Unstandardized Coefficients B	Std. Error	Standardized Coefficients Beta	t	Sig.
1	(Constant)	4.334	.082		53.059	.000
	Party0	-.434	.059	-.434	-7.381	.000
2	(Constant)	4.375	.094		46.394	.000
	Party0	-.383	.063	-.384	-6.120	.000
	GenderCC	-.204	.084	-.147	-2.440	.015
	RaceCC	-.084	.097	-.053	-.867	.387
	SES0	-.002	.101	-.001	-.023	.982
3	(Constant)	4.382	.092		47.384	.000
	Party0	-.283	.068	-.283	-4.160	.000
	GenderCC	-.043	.097	-.031	-.437	.662
	RaceCC	-.104	.096	-.065	-1.080	.281
	SES0	.045	.099	.026	.449	.654
	MGRS0	.214	.140	.100	1.534	.126
	MRN0	-.409	.119	-.255	-3.438	.001
4	(Constant)	4.213	.120		35.118	.000
	Party0	-.239	.069	-.239	-3.486	.001
	GenderCC	-.140	.099	-.101	-1.419	.157
	RaceCC	-.105	.096	-.066	-1.087	.278
	SES0	.091	.099	.053	.920	.358
	MGRS0	.105	.172	.049	.609	.543
	MRN0	-.223	.130	-.139	-1.714	.088
	MRN0xRace	-.255	.109	-.159	-2.328	.021
	MRN0xSES0	.197	.105	.111	1.875	.062
	MRN0xGender	.181	.128	.102	1.416	.158
	MRN0xParty0	.011	.074	.010	.151	.880
	MRN0xMGRS0	.517	.188	.238	2.748	.006
	MGRS0xGender	-.269	.165	-.117	-1.631	.104
	MGRS0xRace	.118	.160	.054	.736	.462
	MGRS0xSES0	.193	.164	.073	1.177	.241
	MGRS0xParty0	-.193	.125	-.131	-1.538	.126

Coefficients[a]

Model		Correlations Zero-order	Partial	Part
1	(Constant)			
	Party0	-.434	-.434	-.434
2	(Constant)			
	Party0	-.434	-.373	-.357
	GenderCC	-.240	-.158	-.142
	RaceCC	-.168	-.057	-.051
	SES0	-.027	-.001	-.001
3	(Constant)			
	Party0	-.434	-.265	-.238
	GenderCC	-.240	-.029	-.025
	RaceCC	-.168	-.071	-.062
	SES0	-.027	.030	.026
	MGRS0	.104	.101	.088
	MRN0	-.388	-.221	-.196
4	(Constant)			
	Party0	-.434	-.228	-.193
	GenderCC	-.240	-.095	-.079
	RaceCC	-.168	-.073	-.060
	SES0	-.027	.062	.051
	MGRS0	.104	.041	.034
	MRN0	-.388	-.115	-.095
	MRN0xRace	-.326	-.155	-.129
	MRN0xSES0	.197	.125	.104
	MRN0xGender	-.075	.095	.079
	MRN0xParty0	-.051	.010	.008
	MRN0xMGRS0	.132	.182	.153
	MGRS0xGender	-.113	-.109	-.091
	MGRS0xRace	.015	.049	.041
	MGRS0xSES0	.089	.079	.065
	MGRS0xParty0	-.039	-.103	-.085

a. Dependent Variable: Concern_Tot

Excluded Variables[a]

Model		Beta In	t	Sig.	Partial Correlation	Collinearity Statistics Tolerance
1	GenderCC	-.145[b]	-2.422	.016	-.156	.943
	RaceCC	-.048[b]	-.778	.438	-.051	.918
	SES0	-.009[b]	-.152	.879	-.010	.998
	MGRS0	.075[b]	1.268	.206	.083	.995
	MRN0	-.237[b]	-3.668	.000	-.233	.783
	MRN0xRace	-.204[b]	-3.333	.001	-.213	.887
	MRN0xSES0	.151[b]	2.576	.011	.166	.988
	MRN0xGender	-.025[b]	-.429	.669	-.028	.987
	MRN0xParty0	.015[b]	.247	.805	.016	.978
	MRN0xMGRS0	.118[b]	2.015	.045	.131	.999
	MGRS0xGender	-.075[b]	-1.277	.203	-.083	.992
	MGRS0xRace	.038[b]	.652	.515	.043	.997
	MGRS0xSES0	.077[b]	1.315	.190	.086	.999
	MGRS0xParty0	.014[b]	.237	.813	.016	.985
2	MGRS0	.025[c]	.406	.685	.027	.871
	MRN0	-.217[c]	-3.096	.002	-.200	.668
	MRN0xRace	-.206[c]	-3.360	.001	-.216	.866
	MRN0xSES0	.158[c]	2.721	.007	.176	.984
	MRN0xGender	-.038[c]	-.633	.527	-.042	.968
	MRN0xParty0	.030[c]	.501	.617	.033	.949
	MRN0xMGRS0	.137[c]	2.333	.020	.152	.972
	MGRS0xGender	-.077[c]	-1.311	.191	-.086	.984
	MGRS0xRace	.020[c]	.340	.734	.022	.975
	MGRS0xSES0	.097[c]	1.641	.102	.107	.969
	MGRS0xParty0	.025[c]	.421	.674	.028	.977
3	MRN0xRace	-.156[d]	-2.399	.017	-.157	.753
	MRN0xSES0	.137[d]	2.355	.019	.154	.946
	MRN0xGender	-.013[d]	-.222	.825	-.015	.938
	MRN0xParty0	.000[d]	.002	.999	.000	.928
	MRN0xMGRS0	.120[d]	2.070	.040	.136	.964
	MGRS0xGender	-.048[d]	-.810	.419	-.053	.945

Excluded Variables[a]

Model		Beta In	t	Sig.	Partial Correlation	Collinearity Statistics Tolerance
	MGRS0xRace	-.009[d]	-.137	.891	-.009	.712
	MGRS0xSES0	.091[d]	1.515	.131	.100	.892
	MGRS0xParty0	.005[d]	.081	.935	.005	.960

a. Dependent Variable: Concern_Tot

b. Predictors in the Model: (Constant), Party0

c. Predictors in the Model: (Constant), Party0, SES0, GenderCC, RaceCC

d. Predictors in the Model: (Constant), Party0, SES0, GenderCC, RaceCC, MGRS0, MRN0

```
REGRESSION
  /MISSING LISTWISE
  /STATISTICS COEFF OUTS R ANOVA CHANGE ZPP
  /CRITERIA=PIN(.05) POUT(.10)
  /NOORIGIN
  /DEPENDENT Finance_Tot
  /METHOD=ENTER Party0
  /METHOD=ENTER GenderCC RaceCC SES0
  /METHOD=ENTER MGRS0 MRN0
  /METHOD=ENTER MRN0xRace MRN0xSES0 MRN0xGender MRN0xParty0 MRN0xMGRS0 MGRS0xGender MGRS0
xRace MGRS0xSES0 MGRS0xParty0.
```

Regression

Notes

Output Created		15-DEC-2021 13:07:58
Comments		
Input	Data	C:\Users\njs5478\Dropbox\HM and COVID\0. Revise and Resubmit\2. R and R Data\Study 1b\Study1b_Data.sav
	Active Dataset	DataSet1
	Filter	<none>
	Weight	<none>
	Split File	<none>
	N of Rows in Working Data File	241
Missing Value Handling	Definition of Missing	User-defined missing values are treated as missing.
	Cases Used	Statistics are based on cases with no missing values for any variable used.
Syntax		REGRESSION<br>/MISSING LISTWISE<br>/STATISTICS COEFF OUTS R ANOVA CHANGE ZPP<br>/CRITERIA=PIN(.05) POUT(.10)<br>/NOORIGIN<br>/DEPENDENT Finance_Tot<br>/METHOD=ENTER Party0<br>/METHOD=ENTER GenderCC RaceCC SES0<br>/METHOD=ENTER MGRS0 MRN0<br>/METHOD=ENTER MRN0xRace MRN0xSES0 MRN0xGender MRN0xParty0 MRN0xMGRS0 MGRS0xGender MGRS0xRace MGRS0xSES0 MGRS0xParty0.

Notes

Resources	Processor Time	00:00:00.02
	Elapsed Time	00:00:00.03
	Memory Required	52240 bytes
	Additional Memory Required for Residual Plots	0 bytes

Variables Entered/Removed[a]

Model	Variables Entered	Variables Removed	Method
1	Party0[b]	.	Enter
2	SES0, GenderCC, RaceCC[b]	.	Enter
3	MGRS0, MRN0[b]	.	Enter
4	MRN0xMGRS0, MRN0xSES0, MRN0xParty0, MGRS0xSES0, MGRS0xGender, MGRS0xRace, MRN0xRace, MRN0xGender, ...[b]	.	Enter

a. Dependent Variable: Finance_Tot

b. All requested variables entered.

Model Summary

Model	R	R Square	Adjusted R Square	Std. Error of the Estimate	Change Statistics				
					R Square Change	F Change	df1	df2	Sig. F Change
1	.126[a]	.016	.012	1.52829	.016	3.814	1	235	.052
2	.218[b]	.048	.031	1.51317	.032	2.574	3	232	.055
3	.237[c]	.056	.031	1.51307	.008	1.015	2	230	.364
4	.339[d]	.115	.055	1.49473	.059	1.631	9	221	.108

a. Predictors: (Constant), Party0

b. Predictors: (Constant), Party0, SES0, GenderCC, RaceCC

c. Predictors: (Constant), Party0, SES0, GenderCC, RaceCC, MGRS0, MRN0

d. Predictors: (Constant), Party0, SES0, GenderCC, RaceCC, MGRS0, MRN0, MRN0xMGRS0, MRN0xSES0, MRN0xParty0, MGRS0xSES0, MGRS0xGender, MGRS0xRace, MRN0xRace, MRN0xGender, MGRS0xParty0

ANOVA[a]

Model		Sum of Squares	df	Mean Square	F	Sig.
1	Regression	8.909	1	8.909	3.814	.052[b]
	Residual	548.883	235	2.336		
	Total	557.792	236			
2	Regression	26.588	4	6.647	2.903	.023[c]
	Residual	531.204	232	2.290		
	Total	557.792	236			
3	Regression	31.236	6	5.206	2.274	.038[d]
	Residual	526.556	230	2.289		
	Total	557.792	236			
4	Regression	64.033	15	4.269	1.911	.023[e]
	Residual	493.759	221	2.234		
	Total	557.792	236			

a. Dependent Variable: Finance_Tot

b. Predictors: (Constant), Party0

c. Predictors: (Constant), Party0, SES0, GenderCC, RaceCC

d. Predictors: (Constant), Party0, SES0, GenderCC, RaceCC, MGRS0, MRN0

e. Predictors: (Constant), Party0, SES0, GenderCC, RaceCC, MGRS0, MRN0, MRN0xMGRS0, MRN0xSES0, MRN0xParty0, MGRS0xSES0, MGRS0xGender, MGRS0xRace, MRN0xRace, MRN0xGender, MGRS0xParty0

Coefficients[a]

Model		Unstandardized Coefficients B	Std. Error	Standardized Coefficients Beta	t	Sig.
1	(Constant)	3.898	.099		39.267	.000
	Party0	-.139	.071	-.126	-1.953	.052
2	(Constant)	3.913	.114		34.227	.000
	Party0	-.095	.076	-.086	-1.255	.211
	GenderCC	-.182	.101	-.119	-1.793	.074
	RaceCC	-.039	.118	-.022	-.328	.743
	SES0	-.245	.122	-.129	-2.014	.045
3	(Constant)	3.923	.115		34.254	.000
	Party0	-.064	.084	-.058	-.756	.451
	GenderCC	-.185	.120	-.120	-1.535	.126
	RaceCC	-.059	.119	-.033	-.492	.623
	SES0	-.241	.123	-.127	-1.958	.051
	MGRS0	-.152	.173	-.064	-.881	.379
	MRN0	-.112	.147	-.063	-.763	.446
4	(Constant)	3.855	.151		25.529	.000
	Party0	-.049	.086	-.044	-.563	.574
	GenderCC	-.213	.124	-.139	-1.720	.087
	RaceCC	-.058	.121	-.033	-.480	.631
	SES0	-.255	.125	-.135	-2.043	.042
	MGRS0	-.218	.217	-.092	-1.004	.316
	MRN0	-.039	.164	-.022	-.239	.811
	MRN0xRace	-.100	.138	-.056	-.724	.470
	MRN0xSES0	.397	.132	.201	2.996	.003
	MRN0xGender	.043	.161	.022	.270	.788
	MRN0xParty0	.120	.093	.094	1.281	.202
	MRN0xMGRS0	.171	.237	.071	.723	.470
	MGRS0xGender	.304	.208	.120	1.463	.145
	MGRS0xRace	.173	.201	.073	.862	.390
	MGRS0xSES0	-.143	.207	-.049	-.691	.490
	MGRS0xParty0	-.250	.158	-.154	-1.584	.115

Coefficients[a]

Model		Correlations Zero-order	Partial	Part
1	(Constant)			
	Party0	-.126	-.126	-.126
2	(Constant)			
	Party0	-.126	-.082	-.080
	GenderCC	-.147	-.117	-.115
	RaceCC	-.055	-.022	-.021
	SES0	-.140	-.131	-.129
3	(Constant)			
	Party0	-.126	-.050	-.048
	GenderCC	-.147	-.101	-.098
	RaceCC	-.055	-.032	-.032
	SES0	-.140	-.128	-.125
	MGRS0	-.014	-.058	-.056
	MRN0	-.165	-.050	-.049
4	(Constant)			
	Party0	-.126	-.038	-.036
	GenderCC	-.147	-.115	-.109
	RaceCC	-.055	-.032	-.030
	SES0	-.140	-.136	-.129
	MGRS0	-.014	-.067	-.064
	MRN0	-.165	-.016	-.015
	MRN0xRace	-.099	-.049	-.046
	MRN0xSES0	.193	.198	.190
	MRN0xGender	.042	.018	.017
	MRN0xParty0	.044	.086	.081
	MRN0xMGRS0	.012	.049	.046
	MGRS0xGender	.044	.098	.093
	MGRS0xRace	-.017	.058	.055
	MGRS0xSES0	.014	-.046	-.044
	MGRS0xParty0	-.061	-.106	-.100

a. Dependent Variable: Finance_Tot

Excluded Variables[a]

Model		Beta In	t	Sig.	Partial Correlation	Collinearity Statistics Tolerance
1	GenderCC	-.124[b]	-1.869	.063	-.121	.943
	RaceCC	-.021[b]	-.308	.758	-.020	.918
	SES0	-.135[b]	-2.100	.037	-.136	.998
	MGRS0	-.023[b]	-.352	.725	-.023	.995
	MRN0	-.136[b]	-1.866	.063	-.121	.783
	MRN0xRace	-.064[b]	-.929	.354	-.061	.887
	MRN0xSES0	.182[b]	2.832	.005	.182	.988
	MRN0xGender	.058[b]	.882	.378	.058	.987
	MRN0xParty0	.064[b]	.977	.330	.064	.978
	MRN0xMGRS0	.007[b]	.113	.910	.007	.999
	MGRS0xGender	.056[b]	.858	.392	.056	.992
	MGRS0xRace	-.010[b]	-.157	.875	-.010	.997
	MGRS0xSES0	.011[b]	.171	.864	.011	.999
	MGRS0xParty0	-.046[b]	-.709	.479	-.046	.985
2	MGRS0	-.083[c]	-1.204	.230	-.079	.871
	MRN0	-.088[c]	-1.120	.264	-.074	.668
	MRN0xRace	-.062[c]	-.901	.368	-.059	.866
	MRN0xSES0	.188[c]	2.961	.003	.191	.984
	MRN0xGender	.057[c]	.869	.386	.057	.968
	MRN0xParty0	.060[c]	.907	.365	.060	.949
	MRN0xMGRS0	.004[c]	.063	.950	.004	.972
	MGRS0xGender	.066[c]	1.018	.310	.067	.984
	MGRS0xRace	-.026[c]	-.397	.692	-.026	.975
	MGRS0xSES0	.008[c]	.118	.906	.008	.969
	MGRS0xParty0	-.043[c]	-.669	.504	-.044	.977
3	MRN0xRace	-.040[d]	-.546	.586	-.036	.753
	MRN0xSES0	.178[d]	2.736	.007	.178	.946
	MRN0xGender	.074[d]	1.126	.261	.074	.938
	MRN0xParty0	.053[d]	.795	.427	.052	.928
	MRN0xMGRS0	.000[d]	-.003	.998	.000	.964
	MGRS0xGender	.064[d]	.975	.330	.064	.945

Excluded Variables[a]

Model		Beta In	t	Sig.	Partial Correlation	Collinearity Statistics Tolerance
	MGRS0xRace	.015[d]	.193	.847	.013	.712
	MGRS0xSES0	-.019[d]	-.283	.777	-.019	.892
	MGRS0xParty0	-.055[d]	-.845	.399	-.056	.960

a. Dependent Variable: Finance_Tot

b. Predictors in the Model: (Constant), Party0

c. Predictors in the Model: (Constant), Party0, SES0, GenderCC, RaceCC

d. Predictors in the Model: (Constant), Party0, SES0, GenderCC, RaceCC, MGRS0, MRN0

```
REGRESSION
  /MISSING LISTWISE
  /STATISTICS COEFF OUTS R ANOVA CHANGE ZPP
  /CRITERIA=PIN(.05) POUT(.10)
  /NOORIGIN
  /DEPENDENT Resource_Tot
  /METHOD=ENTER Party0
  /METHOD=ENTER GenderCC RaceCC SES0
  /METHOD=ENTER MGRS0 MRN0
  /METHOD=ENTER MRN0xRace MRN0xSES0 MRN0xGender MRN0xParty0 MRN0xMGRS0 MGRS0xGender MGRS0
xRace MGRS0xSES0 MGRS0xParty0.
```

Regression

Notes

Output Created		15-DEC-2021 13:07:58
Comments		
Input	Data	C: \Users\njs5478\Dropbox\HM and COVID\0. Revise and Resubmit\2. R and R Data\Study 1b\Study1b_Data.sav
	Active Dataset	DataSet1
	Filter	<none>
	Weight	<none>
	Split File	<none>
	N of Rows in Working Data File	241
Missing Value Handling	Definition of Missing	User-defined missing values are treated as missing.
	Cases Used	Statistics are based on cases with no missing values for any variable used.
Syntax		REGRESSION<br>/MISSING LISTWISE<br>/STATISTICS COEFF OUTS R ANOVA CHANGE ZPP<br>/CRITERIA=PIN(.05) POUT(.10)<br>/NOORIGIN<br>/DEPENDENT Resource_Tot<br>/METHOD=ENTER Party0<br>/METHOD=ENTER GenderCC RaceCC SES0<br>/METHOD=ENTER MGRS0 MRN0<br>/METHOD=ENTER MRN0xRace MRN0xSES0 MRN0xGender MRN0xParty0 MRN0xMGRS0 MGRS0xGender MGRS0xRace MGRS0xSES0 MGRS0xParty0.

Notes

Resources	Processor Time	00:00:00.03
	Elapsed Time	00:00:00.03
	Memory Required	52240 bytes
	Additional Memory Required for Residual Plots	0 bytes

Variables Entered/Removed[a]

Model	Variables Entered	Variables Removed	Method
1	Party0[b]	.	Enter
2	SES0, GenderCC, RaceCC[b]	.	Enter
3	MGRS0, MRN0[b]	.	Enter
4	MRN0xMGRS0, MRN0xSES0, MRN0xParty0, MGRS0xSES0, MGRS0xGender, MGRS0xRace, MRN0xRace, MRN0xGender, ...[b]	.	Enter

a. Dependent Variable: Resource_Tot

b. All requested variables entered.

Model Summary

Model	R	R Square	Adjusted R Square	Std. Error of the Estimate	Change Statistics		
					R Square Change	F Change	df1
1	.077[a]	.006	.002	1.39071	.006	1.411	1
2	.139[b]	.019	.002	1.39021	.013	1.057	3
3	.149[c]	.022	-.003	1.39414	.003	.346	2
4	.278[d]	.077	.015	1.38152	.055	1.469	9

Model Summary

Model	Change Statistics	
	df2	Sig. F Change
1	235	.236
2	232	.368
3	230	.708
4	221	.161

a. Predictors: (Constant), Party0

b. Predictors: (Constant), Party0, SES0, GenderCC, RaceCC

c. Predictors: (Constant), Party0, SES0, GenderCC, RaceCC, MGRS0, MRN0

d. Predictors: (Constant), Party0, SES0, GenderCC, RaceCC, MGRS0, MRN0, MRN0xMGRS0, MRN0xSES0, MRN0xParty0, MGRS0xSES0, MGRS0xGender, MGRS0xRace, MRN0xRace, MRN0xGender, MGRS0xParty0

ANOVA[a]

Model		Sum of Squares	df	Mean Square	F	Sig.
1	Regression	2.728	1	2.728	1.411	.236[b]
	Residual	454.507	235	1.934		
	Total	457.236	236			
2	Regression	8.856	4	2.214	1.146	.336[c]
	Residual	448.380	232	1.933		
	Total	457.236	236			
3	Regression	10.201	6	1.700	.875	.514[d]
	Residual	447.034	230	1.944		
	Total	457.236	236			
4	Regression	35.435	15	2.362	1.238	.245[e]
	Residual	421.800	221	1.909		
	Total	457.236	236			

a. Dependent Variable: Resource_Tot

b. Predictors: (Constant), Party0

c. Predictors: (Constant), Party0, SES0, GenderCC, RaceCC

d. Predictors: (Constant), Party0, SES0, GenderCC, RaceCC, MGRS0, MRN0

e. Predictors: (Constant), Party0, SES0, GenderCC, RaceCC, MGRS0, MRN0, MRN0xMGRS0, MRN0xSES0, MRN0xParty0, MGRS0xSES0, MGRS0xGender, MGRS0xRace, MRN0xRace, MRN0xGender, MGRS0xParty0

Coefficients[a]

Model		Unstandardized Coefficients B	Std. Error	Standardized Coefficients Beta	t	Sig.
1	(Constant)	3.619	.090		40.056	.000
	Party0	-.077	.065	-.077	-1.188	.236
2	(Constant)	3.665	.105		34.888	.000
	Party0	-.036	.070	-.036	-.522	.602
	GenderCC	-.125	.093	-.090	-1.341	.181
	RaceCC	-.097	.108	-.061	-.893	.373
	SES0	-.081	.112	-.047	-.727	.468
3	(Constant)	3.668	.106		34.760	.000
	Party0	-.008	.078	-.008	-.108	.914
	GenderCC	-.087	.111	-.063	-.786	.433
	RaceCC	-.104	.110	-.065	-.951	.343
	SES0	-.070	.113	-.041	-.614	.540
	MGRS0	.029	.159	.014	.185	.854
	MRN0	-.112	.136	-.070	-.827	.409
4	(Constant)	3.544	.140		25.391	.000
	Party0	.022	.080	.022	.273	.785
	GenderCC	-.117	.115	-.084	-1.021	.308
	RaceCC	-.107	.112	-.067	-.955	.341
	SES0	-.059	.116	-.034	-.509	.611
	MGRS0	.126	.200	.058	.626	.532
	MRN0	.025	.152	.016	.168	.867
	MRN0xRace	-.293	.127	-.183	-2.298	.022
	MRN0xSES0	.182	.122	.102	1.490	.138
	MRN0xGender	.201	.149	.114	1.351	.178
	MRN0xParty0	.075	.086	.065	.869	.386
	MRN0xMGRS0	-.004	.219	-.002	-.019	.985
	MGRS0xGender	-.012	.192	-.005	-.065	.948
	MGRS0xRace	-.124	.186	-.057	-.667	.505
	MGRS0xSES0	.214	.191	.081	1.121	.264
	MGRS0xParty0	-.077	.146	-.052	-.526	.599

Coefficients[a]

Model		Correlations Zero-order	Partial	Part
1	(Constant)			
	Party0	-.077	-.077	-.077
2	(Constant)			
	Party0	-.077	-.034	-.034
	GenderCC	-.103	-.088	-.087
	RaceCC	-.076	-.059	-.058
	SES0	-.056	-.048	-.047
3	(Constant)			
	Party0	-.077	-.007	-.007
	GenderCC	-.103	-.052	-.051
	RaceCC	-.076	-.063	-.062
	SES0	-.056	-.040	-.040
	MGRS0	.037	.012	.012
	MRN0	-.107	-.054	-.054
4	(Constant)			
	Party0	-.077	.018	.018
	GenderCC	-.103	-.069	-.066
	RaceCC	-.076	-.064	-.062
	SES0	-.056	-.034	-.033
	MGRS0	.037	.042	.040
	MRN0	-.107	.011	.011
	MRN0xRace	-.152	-.153	-.148
	MRN0xSES0	.111	.100	.096
	MRN0xGender	.086	.090	.087
	MRN0xParty0	.030	.058	.056
	MRN0xMGRS0	-.018	-.001	-.001
	MGRS0xGender	-.001	-.004	-.004
	MGRS0xRace	-.062	-.045	-.043
	MGRS0xSES0	.057	.075	.072
	MGRS0xParty0	-.075	-.035	-.034

a. Dependent Variable: Resource_Tot

Excluded Variables[a]

Model		Beta In	t	Sig.	Partial Correlation	Collinearity Statistics Tolerance
1	GenderCC	-.090[b]	-1.348	.179	-.088	.943
	RaceCC	-.059[b]	-.864	.389	-.056	.918
	SES0	-.053[b]	-.808	.420	-.053	.998
	MGRS0	.032[b]	.483	.630	.032	.995
	MRN0	-.090[b]	-1.228	.221	-.080	.783
	MRN0xRace	-.142[b]	-2.071	.039	-.134	.887
	MRN0xSES0	.104[b]	1.592	.113	.103	.988
	MRN0xGender	.096[b]	1.476	.141	.096	.987
	MRN0xParty0	.043[b]	.649	.517	.042	.978
	MRN0xMGRS0	-.021[b]	-.318	.751	-.021	.999
	MGRS0xGender	.006[b]	.089	.929	.006	.992
	MGRS0xRace	-.058[b]	-.895	.372	-.058	.997
	MGRS0xSES0	.055[b]	.850	.396	.055	.999
	MGRS0xParty0	-.066[b]	-1.012	.313	-.066	.985
2	MGRS0	-.007[c]	-.095	.924	-.006	.871
	MRN0	-.065[c]	-.813	.417	-.053	.668
	MRN0xRace	-.149[c]	-2.146	.033	-.140	.866
	MRN0xSES0	.107[c]	1.646	.101	.108	.984
	MRN0xGender	.089[c]	1.356	.176	.089	.968
	MRN0xParty0	.050[c]	.741	.459	.049	.949
	MRN0xMGRS0	-.017[c]	-.257	.797	-.017	.972
	MGRS0xGender	.010[c]	.153	.879	.010	.984
	MGRS0xRace	-.070[c]	-1.064	.288	-.070	.975
	MGRS0xSES0	.063[c]	.960	.338	.063	.969
	MGRS0xParty0	-.060[c]	-.912	.363	-.060	.977
3	MRN0xRace	-.147[d]	-1.972	.050	-.129	.753
	MRN0xSES0	.103[d]	1.535	.126	.101	.946
	MRN0xGender	.101[d]	1.502	.134	.099	.938
	MRN0xParty0	.042[d]	.622	.535	.041	.928
	MRN0xMGRS0	-.022[d]	-.333	.740	-.022	.964
	MGRS0xGender	.017[d]	.260	.795	.017	.945

Excluded Variables[a]

Model		Beta In	t	Sig.	Partial Correlation	Collinearity Statistics Tolerance
	MGRS0xRace	-.097[d]	-1.256	.210	-.083	.712
	MGRS0xSES0	.061[d]	.888	.375	.059	.892
	MGRS0xParty0	-.068[d]	-1.016	.311	-.067	.960

a. Dependent Variable: Resource_Tot

b. Predictors in the Model: (Constant), Party0

c. Predictors in the Model: (Constant), Party0, SES0, GenderCC, RaceCC

d. Predictors in the Model: (Constant), Party0, SES0, GenderCC, RaceCC, MGRS0, MRN0

```
REGRESSION
  /MISSING LISTWISE
  /STATISTICS COEFF OUTS R ANOVA CHANGE ZPP
  /CRITERIA=PIN(.05) POUT(.10)
  /NOORIGIN
  /DEPENDENT Psychology_Tot
  /METHOD=ENTER Party0
  /METHOD=ENTER GenderCC RaceCC SES0
  /METHOD=ENTER MGRS0 MRN0
  /METHOD=ENTER MRN0xRace MRN0xSES0 MRN0xGender MRN0xParty0 MRN0xMGRS0 MGRS0xGender MGRS0
xRace MGRS0xSES0 MGRS0xParty0.
```

Regression

Notes

Output Created		15-DEC-2021 13:07:58
Comments		
Input	Data	C:\Users\njs5478\Dropbox\HM and COVID\0. Revise and Resubmit\2. R and R Data\Study 1b\Study1b_Data.sav
	Active Dataset	DataSet1
	Filter	<none>
	Weight	<none>
	Split File	<none>
	N of Rows in Working Data File	241
Missing Value Handling	Definition of Missing	User-defined missing values are treated as missing.
	Cases Used	Statistics are based on cases with no missing values for any variable used.
Syntax		REGRESSION<br>/MISSING LISTWISE<br>/STATISTICS COEFF OUTS R ANOVA CHANGE ZPP<br>/CRITERIA=PIN(.05) POUT(.10)<br>/NOORIGIN<br>/DEPENDENT Psychology_Tot<br>/METHOD=ENTER Party0<br>/METHOD=ENTER GenderCC RaceCC SES0<br>/METHOD=ENTER MGRS0 MRN0<br>/METHOD=ENTER MRN0xRace MRN0xSES0 MRN0xGender MRN0xParty0 MRN0xMGRS0 MGRS0xGender MGRS0xRace MGRS0xSES0 MGRS0xParty0.

Notes

Resources	Processor Time	00:00:00.02
	Elapsed Time	00:00:00.02
	Memory Required	52240 bytes
	Additional Memory Required for Residual Plots	0 bytes

Variables Entered/Removed[a]

Model	Variables Entered	Variables Removed	Method
1	Party0[b]	.	Enter
2	SES0, GenderCC, RaceCC[b]	.	Enter
3	MGRS0, MRN0[b]	.	Enter
4	MRN0xMGRS0, MRN0xSES0, MRN0xParty0, MGRS0xSES0, MGRS0xGender, MGRS0xRace, MRN0xRace, MRN0xGender, ...[b]	.	Enter

a. Dependent Variable: Psychology_Tot

b. All requested variables entered.

Model Summary

Model	R	R Square	Adjusted R Square	Std. Error of the Estimate	Change Statistics		
					R Square Change	F Change	df1
1	.260[a]	.068	.064	1.44368	.068	17.107	1
2	.292[b]	.086	.070	1.43917	.018	1.492	3
3	.316[c]	.100	.076	1.43412	.014	1.818	2
4	.330[d]	.109	.049	1.45531	.009	.261	9

Model Summary

Model	Change Statistics	
	df2	Sig. F Change
1	235	.000
2	232	.217
3	230	.165
4	221	.984

a. Predictors: (Constant), Party0

b. Predictors: (Constant), Party0, SES0, GenderCC, RaceCC

c. Predictors: (Constant), Party0, SES0, GenderCC, RaceCC, MGRS0, MRN0

d. Predictors: (Constant), Party0, SES0, GenderCC, RaceCC, MGRS0, MRN0, MRN0xMGRS0, MRN0xSES0, MRN0xParty0, MGRS0xSES0, MGRS0xGender, MGRS0xRace, MRN0xRace, MRN0xGender, MGRS0xParty0

ANOVA[a]

Model		Sum of Squares	df	Mean Square	F	Sig.
1	Regression	35.654	1	35.654	17.107	.000[b]
	Residual	489.790	235	2.084		
	Total	525.444	236			
2	Regression	44.926	4	11.231	5.423	.000[c]
	Residual	480.518	232	2.071		
	Total	525.444	236			
3	Regression	52.405	6	8.734	4.247	.000[d]
	Residual	473.039	230	2.057		
	Total	525.444	236			
4	Regression	57.383	15	3.826	1.806	.035[e]
	Residual	468.061	221	2.118		
	Total	525.444	236			

a. Dependent Variable: Psychology_Tot

b. Predictors: (Constant), Party0

c. Predictors: (Constant), Party0, SES0, GenderCC, RaceCC

d. Predictors: (Constant), Party0, SES0, GenderCC, RaceCC, MGRS0, MRN0

e. Predictors: (Constant), Party0, SES0, GenderCC, RaceCC, MGRS0, MRN0, MRN0xMGRS0, MRN0xSES0, MRN0xParty0, MGRS0xSES0, MGRS0xGender, MGRS0xRace, MRN0xRace, MRN0xGender, MGRS0xParty0

Coefficients[a]

Model		Unstandardized Coefficients B	Std. Error	Standardized Coefficients Beta	t	Sig.
1	(Constant)	4.664	.094		49.736	.000
	Party0	-.279	.067	-.260	-4.136	.000
2	(Constant)	4.687	.109		43.099	.000
	Party0	-.239	.072	-.223	-3.303	.001
	GenderCC	-.197	.096	-.132	-2.038	.043
	RaceCC	-.046	.112	-.027	-.406	.685
	SES0	.065	.116	.035	.562	.575
3	(Constant)	4.694	.109		43.242	.000
	Party0	-.173	.080	-.161	-2.159	.032
	GenderCC	-.105	.114	-.071	-.921	.358
	RaceCC	-.062	.113	-.036	-.552	.581
	SES0	.093	.117	.051	.798	.426
	MGRS0	.079	.164	.034	.484	.629
	MRN0	-.265	.139	-.154	-1.901	.059
4	(Constant)	4.620	.147		31.427	.000
	Party0	-.164	.084	-.153	-1.948	.053
	GenderCC	-.134	.121	-.090	-1.110	.268
	RaceCC	-.074	.118	-.043	-.630	.529
	SES0	.110	.122	.060	.903	.367
	MGRS0	.048	.211	.021	.229	.819
	MRN0	-.193	.160	-.112	-1.209	.228
	MRN0xRace	-.148	.134	-.086	-1.105	.270
	MRN0xSES0	-.026	.129	-.014	-.201	.841
	MRN0xGender	.112	.156	.059	.713	.477
	MRN0xParty0	.051	.091	.042	.564	.573
	MRN0xMGRS0	.109	.231	.047	.472	.638
	MGRS0xGender	-.059	.202	-.024	-.291	.771
	MGRS0xRace	.027	.196	.012	.138	.890
	MGRS0xSES0	.107	.201	.038	.533	.595
	MGRS0xParty0	-.049	.153	-.031	-.322	.748

Coefficients[a]

Model		Correlations Zero-order	Partial	Part
1	(Constant)			
	Party0	-.260	-.260	-.260
2	(Constant)			
	Party0	-.260	-.212	-.207
	GenderCC	-.184	-.133	-.128
	RaceCC	-.094	-.027	-.025
	SES0	.018	.037	.035
3	(Constant)			
	Party0	-.260	-.141	-.135
	GenderCC	-.184	-.061	-.058
	RaceCC	-.094	-.036	-.035
	SES0	.018	.053	.050
	MGRS0	.052	.032	.030
	MRN0	-.251	-.124	-.119
4	(Constant)			
	Party0	-.260	-.130	-.124
	GenderCC	-.184	-.074	-.070
	RaceCC	-.094	-.042	-.040
	SES0	.018	.061	.057
	MGRS0	.052	.015	.015
	MRN0	-.251	-.081	-.077
	MRN0xRace	-.182	-.074	-.070
	MRN0xSES0	.035	-.014	-.013
	MRN0xGender	-.003	.048	.045
	MRN0xParty0	-.007	.038	.036
	MRN0xMGRS0	.022	.032	.030
	MGRS0xGender	-.027	-.020	-.018
	MGRS0xRace	-.005	.009	.009
	MGRS0xSES0	.025	.036	.034
	MGRS0xParty0	-.032	-.022	-.020

a. Dependent Variable: Psychology_Tot

Excluded Variables[a]

Model		Beta In	t	Sig.	Partial Correlation	Collinearity Statistics Tolerance
1	GenderCC	-.129[b]	-2.008	.046	-.130	.943
	RaceCC	-.021[b]	-.322	.748	-.021	.918
	SES0	.029[b]	.458	.648	.030	.998
	MGRS0	.034[b]	.535	.593	.035	.995
	MRN0	-.165[b]	-2.342	.020	-.151	.783
	MRN0xRace	-.107[b]	-1.606	.110	-.104	.887
	MRN0xSES0	.006[b]	.092	.927	.006	.988
	MRN0xGender	.028[b]	.436	.663	.028	.987
	MRN0xParty0	.033[b]	.513	.608	.034	.978
	MRN0xMGRS0	.013[b]	.212	.832	.014	.999
	MGRS0xGender	-.004[b]	-.061	.951	-.004	.992
	MGRS0xRace	.009[b]	.140	.889	.009	.997
	MGRS0xSES0	.018[b]	.292	.770	.019	.999
	MGRS0xParty0	.000[b]	-.003	.998	.000	.985
2	MGRS0	-.010[c]	-.156	.876	-.010	.871
	MRN0	-.141[c]	-1.848	.066	-.121	.668
	MRN0xRace	-.104[c]	-1.543	.124	-.101	.866
	MRN0xSES0	.013[c]	.198	.843	.013	.984
	MRN0xGender	.019[c]	.294	.769	.019	.968
	MRN0xParty0	.050[c]	.777	.438	.051	.949
	MRN0xMGRS0	.032[c]	.505	.614	.033	.972
	MGRS0xGender	-.009[c]	-.136	.892	-.009	.984
	MGRS0xRace	-.009[c]	-.144	.886	-.009	.975
	MGRS0xSES0	.038[c]	.594	.553	.039	.969
	MGRS0xParty0	.009[c]	.146	.884	.010	.977
3	MRN0xRace	-.067[d]	-.933	.352	-.062	.753
	MRN0xSES0	-.007[d]	-.103	.918	-.007	.946
	MRN0xGender	.038[d]	.584	.560	.039	.938
	MRN0xParty0	.033[d]	.500	.617	.033	.928
	MRN0xMGRS0	.021[d]	.336	.737	.022	.964
	MGRS0xGender	.007[d]	.111	.912	.007	.945

Excluded Variables[a]

Model		Beta In	t	Sig.	Partial Correlation	Collinearity Statistics Tolerance
	MGRS0xRace	-.018[d]	-.237	.813	-.016	.712
	MGRS0xSES0	.026[d]	.389	.698	.026	.892
	MGRS0xParty0	-.005[d]	-.074	.941	-.005	.960

a. Dependent Variable: Psychology_Tot

b. Predictors in the Model: (Constant), Party0

c. Predictors in the Model: (Constant), Party0, SES0, GenderCC, RaceCC

d. Predictors in the Model: (Constant), Party0, SES0, GenderCC, RaceCC, MGRS0, MRN0

```
REGRESSION
  /MISSING LISTWISE
  /STATISTICS COEFF OUTS R ANOVA CHANGE ZPP
  /CRITERIA=PIN(.05) POUT(.10)
  /NOORIGIN
  /DEPENDENT TrumpApproval
  /METHOD=ENTER Party0
  /METHOD=ENTER GenderCC RaceCC SES0
  /METHOD=ENTER MGRS0 MRN0
  /METHOD=ENTER MRN0xRace MRN0xSES0 MRN0xGender MRN0xParty0 MRN0xMGRS0 MGRS0xGender MGRS0
xRace MGRS0xSES0 MGRS0xParty0.
```

Regression

Notes

Output Created		15-DEC-2021 13:07:58
Comments		
Input	Data	C: \Users\njs5478\Dropbox\HM and COVID\0. Revise and Resubmit\2. R and R Data\Study 1b\Study1b_Data.sav
	Active Dataset	DataSet1
	Filter	<none>
	Weight	<none>
	Split File	<none>
	N of Rows in Working Data File	241
Missing Value Handling	Definition of Missing	User-defined missing values are treated as missing.
	Cases Used	Statistics are based on cases with no missing values for any variable used.
Syntax		REGRESSION<br>/MISSING LISTWISE<br>/STATISTICS COEFF OUTS R ANOVA CHANGE ZPP<br>/CRITERIA=PIN(.05) POUT(.10)<br>/NOORIGIN<br>/DEPENDENT TrumpApproval<br>/METHOD=ENTER Party0<br>/METHOD=ENTER GenderCC RaceCC SES0<br>/METHOD=ENTER MGRS0 MRN0<br>/METHOD=ENTER MRN0xRace MRN0xSES0 MRN0xGender MRN0xParty0 MRN0xMGRS0 MGRS0xGender MGRS0xRace MGRS0xSES0 MGRS0xParty0.

Notes

Resources	Processor Time	00:00:00.02
	Elapsed Time	00:00:00.02
	Memory Required	52240 bytes
	Additional Memory Required for Residual Plots	0 bytes

Variables Entered/Removed[a]

Model	Variables Entered	Variables Removed	Method
1	Party0[b]	.	Enter
2	SES0, GenderCC, RaceCC[b]	.	Enter
3	MGRS0, MRN0[b]	.	Enter
4	MRN0xMGRS0, MRN0xSES0, MRN0xParty0, MGRS0xSES0, MGRS0xGender, MGRS0xRace, MRN0xRace, MRN0xGender, ...[b]	.	Enter

a. Dependent Variable: Do you approve or disapprove of the way Donald Trump is handling his job as President?

b. All requested variables entered.

Model Summary

Model	R	R Square	Adjusted R Square	Std. Error of the Estimate	Change Statistics		
					R Square Change	F Change	df1
1	.780[a]	.609	.607	1.313	.609	364.344	1
2	.793[b]	.628	.622	1.288	.019	4.033	3
3	.809[c]	.655	.646	1.247	.026	8.688	2
4	.816[d]	.666	.643	1.252	.011	.821	9

Model Summary

Model	Change Statistics	
	df2	Sig. F Change
1	234	.000
2	231	.008
3	229	.000
4	220	.597

a. Predictors: (Constant), Party0

b. Predictors: (Constant), Party0, SES0, GenderCC, RaceCC

c. Predictors: (Constant), Party0, SES0, GenderCC, RaceCC, MGRS0, MRN0

d. Predictors: (Constant), Party0, SES0, GenderCC, RaceCC, MGRS0, MRN0, MRN0xMGRS0, MRN0xSES0, MRN0xParty0, MGRS0xSES0, MGRS0xGender, MGRS0xRace, MRN0xRace, MRN0xGender, MGRS0xParty0

ANOVA[a]

Model		Sum of Squares	df	Mean Square	F	Sig.
1	Regression	628.200	1	628.200	364.344	.000[b]
	Residual	403.461	234	1.724		
	Total	1031.661	235			
2	Regression	648.280	4	162.070	97.653	.000[c]
	Residual	383.381	231	1.660		
	Total	1031.661	235			
3	Regression	675.318	6	112.553	72.331	.000[d]
	Residual	356.343	229	1.556		
	Total	1031.661	235			
4	Regression	686.898	15	45.793	29.222	.000[e]
	Residual	344.763	220	1.567		
	Total	1031.661	235			

a. Dependent Variable: Do you approve or disapprove of the way Donald Trump is handling his job as President?

b. Predictors: (Constant), Party0

c. Predictors: (Constant), Party0, SES0, GenderCC, RaceCC

d. Predictors: (Constant), Party0, SES0, GenderCC, RaceCC, MGRS0, MRN0

e. Predictors: (Constant), Party0, SES0, GenderCC, RaceCC, MGRS0, MRN0, MRN0xMGRS0, MRN0xSES0, MRN0xParty0, MGRS0xSES0, MGRS0xGender, MGRS0xRace, MRN0xRace, MRN0xGender, MGRS0xParty0

Coefficients[a]

Model		Unstandardized Coefficients B	Std. Error	Standardized Coefficients Beta	t	Sig.
1	(Constant)	3.142	.085		36.761	.000
	Party0	1.171	.061	.780	19.088	.000
2	(Constant)	3.003	.097		30.822	.000
	Party0	1.119	.065	.745	17.286	.000
	GenderCC	.049	.087	.024	.570	.569
	RaceCC	.278	.101	.116	2.764	.006
	SES0	-.223	.104	-.086	-2.145	.033
3	(Constant)	2.984	.094		31.580	.000
	Party0	1.004	.070	.669	14.443	.000
	GenderCC	-.052	.100	-.025	-.522	.602
	RaceCC	.323	.098	.134	3.290	.001
	SES0	-.261	.102	-.101	-2.568	.011
	MGRS0	.103	.143	.032	.719	.473
	MRN0	.442	.122	.183	3.635	.000
4	(Constant)	3.024	.127		23.872	.000
	Party0	.964	.072	.642	13.310	.000
	GenderCC	-.009	.104	-.004	-.088	.930
	RaceCC	.325	.102	.135	3.193	.002
	SES0	-.277	.105	-.107	-2.646	.009
	MGRS0	.190	.182	.059	1.049	.295
	MRN0	.361	.138	.150	2.622	.009
	MRN0xRace	.152	.115	.063	1.312	.191
	MRN0xSES0	-.018	.111	-.007	-.159	.874
	MRN0xGender	-.041	.135	-.015	-.305	.761
	MRN0xParty0	.056	.078	.032	.716	.475
	MRN0xMGRS0	-.352	.198	-.108	-1.772	.078
	MGRS0xGender	.162	.174	.047	.928	.354
	MGRS0xRace	-.079	.169	-.024	-.470	.639
	MGRS0xSES0	-.038	.173	-.010	-.220	.826
	MGRS0xParty0	.148	.132	.067	1.122	.263

Coefficients[a]

Model		Correlations		
		Zero-order	Partial	Part
1	(Constant)			
	Party0	.780	.780	.780
2	(Constant)			
	Party0	.780	.751	.693
	GenderCC	.203	.037	.023
	RaceCC	.326	.179	.111
	SES0	-.049	-.140	-.086
3	(Constant)			
	Party0	.780	.690	.561
	GenderCC	.203	-.034	-.020
	RaceCC	.326	.212	.128
	SES0	-.049	-.167	-.100
	MGRS0	.008	.047	.028
	MRN0	.482	.234	.141
4	(Constant)			
	Party0	.780	.668	.519
	GenderCC	.203	-.006	-.003
	RaceCC	.326	.210	.124
	SES0	-.049	-.176	-.103
	MGRS0	.008	.071	.041
	MRN0	.482	.174	.102
	MRN0xRace	.361	.088	.051
	MRN0xSES0	-.133	-.011	-.006
	MRN0xGender	.131	-.021	-.012
	MRN0xParty0	.158	.048	.028
	MRN0xMGRS0	-.076	-.119	-.069
	MGRS0xGender	.089	.062	.036
	MGRS0xRace	.076	-.032	-.018
	MGRS0xSES0	-.048	-.015	-.009
	MGRS0xParty0	.091	.075	.044

a. Dependent Variable: Do you approve or disapprove of the way Donald Trump is handling his job as President?

Excluded Variables[a]

Model		Beta In	t	Sig.	Partial Correlation	Collinearity Statistics Tolerance
1	GenderCC	.016[b]	.381	.704	.025	.942
	RaceCC	.113[b]	2.681	.008	.173	.919
	SES0	-.083[b]	-2.032	.043	-.132	.998
	MGRS0	.063[b]	1.554	.122	.101	.995
	MRN0	.152[b]	3.355	.001	.215	.783
	MRN0xRace	.112[b]	2.606	.010	.168	.888
	MRN0xSES0	-.047[b]	-1.149	.252	-.075	.988
	MRN0xGender	.040[b]	.980	.328	.064	.986
	MRN0xParty0	.042[b]	1.020	.309	.067	.977
	MRN0xMGRS0	-.049[b]	-1.199	.232	-.078	.999
	MGRS0xGender	.020[b]	.494	.622	.032	.992
	MGRS0xRace	.035[b]	.846	.398	.055	.997
	MGRS0xSES0	-.028[b]	-.679	.498	-.044	.999
	MGRS0xParty0	-.005[b]	-.113	.910	-.007	.985
2	MGRS0	.085[c]	1.988	.048	.130	.872
	MRN0	.195[c]	4.110	.000	.262	.666
	MRN0xRace	.129[c]	3.043	.003	.197	.866
	MRN0xSES0	-.046[c]	-1.133	.258	-.075	.984
	MRN0xGender	.059[c]	1.451	.148	.095	.968
	MRN0xParty0	.021[c]	.510	.611	.034	.949
	MRN0xMGRS0	-.065[c]	-1.602	.111	-.105	.972
	MGRS0xGender	.025[c]	.615	.539	.041	.984
	MGRS0xRace	.035[c]	.873	.384	.057	.976
	MGRS0xSES0	-.049[c]	-1.207	.229	-.079	.969
	MGRS0xParty0	-.016[c]	-.390	.697	-.026	.977
3	MRN0xRace	.078[d]	1.745	.082	.115	.753
	MRN0xSES0	-.015[d]	-.381	.704	-.025	.947
	MRN0xGender	.030[d]	.757	.450	.050	.936
	MRN0xParty0	.044[d]	1.088	.278	.072	.928
	MRN0xMGRS0	-.052[d]	-1.327	.186	-.088	.964
	MGRS0xGender	.017[d]	.413	.680	.027	.945

Excluded Variables[a]

Model		Beta In	t	Sig.	Partial Correlation	Collinearity Statistics Tolerance
	MGRS0xRace	.007[d]	.146	.884	.010	.713
	MGRS0xSES0	-.015[d]	-.375	.708	-.025	.893
	MGRS0xParty0	.006[d]	.146	.884	.010	.960

a. Dependent Variable: Do you approve or disapprove of the way Donald Trump is handling his job as President?

b. Predictors in the Model: (Constant), Party0

c. Predictors in the Model: (Constant), Party0, SES0, GenderCC, RaceCC

d. Predictors in the Model: (Constant), Party0, SES0, GenderCC, RaceCC, MGRS0, MRN0

```
REGRESSION
  /MISSING LISTWISE
  /STATISTICS COEFF OUTS R ANOVA CHANGE ZPP
  /CRITERIA=PIN(.05) POUT(.10)
  /NOORIGIN
  /DEPENDENT TrumpX
  /METHOD=ENTER Party0
  /METHOD=ENTER GenderCC RaceCC SES0
  /METHOD=ENTER MGRS0 MRN0
  /METHOD=ENTER MRN0xRace MRN0xSES0 MRN0xGender MRN0xParty0 MRN0xMGRS0 MGRS0xGender MGRS0
xRace MGRS0xSES0 MGRS0xParty0.
```

Regression

Notes

Output Created		15-DEC-2021 13:07:58
Comments		
Input	Data	C: \Users\njs5478\Dropbox\HM and COVID\0. Revise and Resubmit\2. R and R Data\Study 1b\Study1b_Data.sav
	Active Dataset	DataSet1
	Filter	<none>
	Weight	<none>
	Split File	<none>
	N of Rows in Working Data File	241
Missing Value Handling	Definition of Missing	User-defined missing values are treated as missing.
	Cases Used	Statistics are based on cases with no missing values for any variable used.
Syntax		REGRESSION<br>/MISSING LISTWISE<br>/STATISTICS COEFF OUTS R ANOVA CHANGE ZPP<br>/CRITERIA=PIN(.05) POUT(.10)<br>/NOORIGIN<br>/DEPENDENT TrumpX<br>/METHOD=ENTER Party0<br>/METHOD=ENTER GenderCC RaceCC SES0<br>/METHOD=ENTER MGRS0 MRN0<br>/METHOD=ENTER MRN0xRace MRN0xSES0 MRN0xGender MRN0xParty0 MRN0xMGRS0 MGRS0xGender MGRS0xRace MGRS0xSES0 MGRS0xParty0.
Resources	Processor Time	00:00:00.03
	Elapsed Time	00:00:00.03

Notes

	Memory Required	52240 bytes
	Additional Memory Required for Residual Plots	0 bytes

Variables Entered/Removed[a]

Model	Variables Entered	Variables Removed	Method
1	Party0[b]	.	Enter
2	SES0, GenderCC, RaceCC[b]	.	Enter
3	MGRS0, MRN0[b]	.	Enter
4	MRN0xMGRS0, MRN0xSES0, MRN0xParty0, MGRS0xSES0, MGRS0xGender, MGRS0xRace, MRN0xRace, MRN0xGender, ...[b]	.	Enter

a. Dependent Variable: TrumpX

b. All requested variables entered.

Model Summary

Model	R	R Square	Adjusted R Square	Std. Error of the Estimate	Change Statistics		
					R Square Change	F Change	df1
1	.778[a]	.605	.604	1.29362	.605	355.751	1
2	.785[b]	.616	.610	1.28386	.011	2.180	3
3	.801[c]	.642	.632	1.24569	.026	8.125	2
4	.811[d]	.658	.634	1.24263	.016	1.125	9

Model Summary

Model	Change Statistics	
	df2	Sig. F Change
1	232	.000
2	229	.091
3	227	.000
4	218	.346

a. Predictors: (Constant), Party0

b. Predictors: (Constant), Party0, SES0, GenderCC, RaceCC

c. Predictors: (Constant), Party0, SES0, GenderCC, RaceCC, MGRS0, MRN0

d. Predictors: (Constant), Party0, SES0, GenderCC, RaceCC, MGRS0, MRN0, MRN0xMGRS0, MRN0xSES0, MRN0xParty0, MGRS0xSES0, MGRS0xGender, MGRS0xRace, MRN0xRace, MRN0xGender, MGRS0xParty0

ANOVA[a]

Model		Sum of Squares	df	Mean Square	F	Sig.
1	Regression	595.332	1	595.332	355.751	.000[b]
	Residual	388.240	232	1.673		
	Total	983.573	233			
2	Regression	606.110	4	151.527	91.929	.000[c]
	Residual	377.463	229	1.648		
	Total	983.573	233			
3	Regression	631.325	6	105.221	67.808	.000[d]
	Residual	352.248	227	1.552		
	Total	983.573	233			
4	Regression	646.955	15	43.130	27.932	.000[e]
	Residual	336.618	218	1.544		
	Total	983.573	233			

a. Dependent Variable: TrumpX

b. Predictors: (Constant), Party0

c. Predictors: (Constant), Party0, SES0, GenderCC, RaceCC

d. Predictors: (Constant), Party0, SES0, GenderCC, RaceCC, MGRS0, MRN0

e. Predictors: (Constant), Party0, SES0, GenderCC, RaceCC, MGRS0, MRN0, MRN0xMGRS0, MRN0xSES0, MRN0xParty0, MGRS0xSES0, MGRS0xGender, MGRS0xRace, MRN0xRace, MRN0xGender, MGRS0xParty0

Coefficients[a]

Model		Unstandardized Coefficients B	Std. Error	Standardized Coefficients Beta	t	Sig.
1	(Constant)	3.058	.085		36.157	.000
	Party0	1.143	.061	.778	18.861	.000
2	(Constant)	2.961	.097		30.435	.000
	Party0	1.103	.065	.751	17.053	.000
	GenderCC	.055	.087	.027	.630	.529
	RaceCC	.193	.100	.082	1.925	.056
	SES0	-.171	.104	-.068	-1.652	.100
3	(Constant)	2.942	.095		31.120	.000
	Party0	.999	.069	.680	14.372	.000
	GenderCC	-.026	.101	-.013	-.260	.795
	RaceCC	.238	.098	.101	2.429	.016
	SES0	-.205	.102	-.081	-2.013	.045
	MGRS0	.151	.143	.048	1.052	.294
	MRN0	.404	.122	.171	3.309	.001
4	(Constant)	2.914	.127		22.954	.000
	Party0	.957	.072	.651	13.305	.000
	GenderCC	.023	.104	.011	.221	.825
	RaceCC	.264	.101	.112	2.614	.010
	SES0	-.224	.104	-.089	-2.153	.032
	MGRS0	.317	.180	.100	1.754	.081
	MRN0	.324	.137	.137	2.362	.019
	MRN0xRace	.127	.115	.054	1.108	.269
	MRN0xSES0	.073	.110	.028	.665	.507
	MRN0xGender	.107	.136	.041	.787	.432
	MRN0xParty0	.018	.078	.011	.235	.815
	MRN0xMGRS0	-.376	.197	-.118	-1.906	.058
	MGRS0xGender	.092	.174	.027	.529	.597
	MGRS0xRace	-.232	.167	-.073	-1.389	.166
	MGRS0xSES0	-.057	.172	-.015	-.328	.743
	MGRS0xParty0	.257	.131	.119	1.960	.051

Coefficients[a]

Model		Correlations Zero-order	Partial	Part
1	(Constant)			
	Party0	.778	.778	.778
2	(Constant)			
	Party0	.778	.748	.698
	GenderCC	.212	.042	.026
	RaceCC	.294	.126	.079
	SES0	-.031	-.109	-.068
3	(Constant)			
	Party0	.778	.690	.571
	GenderCC	.212	-.017	-.010
	RaceCC	.294	.159	.096
	SES0	-.031	-.132	-.080
	MGRS0	.022	.070	.042
	MRN0	.481	.215	.131
4	(Constant)			
	Party0	.778	.669	.527
	GenderCC	.212	.015	.009
	RaceCC	.294	.174	.104
	SES0	-.031	-.144	-.085
	MGRS0	.022	.118	.069
	MRN0	.481	.158	.094
	MRN0xRace	.351	.075	.044
	MRN0xSES0	-.103	.045	.026
	MRN0xGender	.175	.053	.031
	MRN0xParty0	.152	.016	.009
	MRN0xMGRS0	-.072	-.128	-.076
	MGRS0xGender	.091	.036	.021
	MGRS0xRace	.061	-.094	-.055
	MGRS0xSES0	-.061	-.022	-.013
	MGRS0xParty0	.103	.132	.078

a. Dependent Variable: TrumpX

Excluded Variables[a]

Model		Beta In	t	Sig.	Partial Correlation	Collinearity Statistics Tolerance
1	GenderCC	.021[b]	.499	.618	.033	.939
	RaceCC	.080[b]	1.866	.063	.122	.920
	SES0	-.065[b]	-1.575	.117	-.103	.998
	MGRS0	.075[b]	1.826	.069	.119	.995
	MRN0	.153[b]	3.366	.001	.216	.785
	MRN0xRace	.103[b]	2.372	.019	.154	.889
	MRN0xSES0	-.018[b]	-.442	.659	-.029	.988
	MRN0xGender	.080[b]	1.933	.055	.126	.985
	MRN0xParty0	.036[b]	.873	.383	.057	.978
	MRN0xMGRS0	-.047[b]	-1.132	.259	-.074	.999
	MGRS0xGender	.026[b]	.621	.535	.041	.993
	MGRS0xRace	.018[b]	.439	.661	.029	.997
	MGRS0xSES0	-.040[b]	-.965	.336	-.063	.999
	MGRS0xParty0	.006[b]	.156	.876	.010	.985
2	MGRS0	.098[c]	2.253	.025	.148	.870
	MRN0	.190[c]	3.890	.000	.249	.665
	MRN0xRace	.115[c]	2.647	.009	.173	.867
	MRN0xSES0	-.018[c]	-.433	.666	-.029	.985
	MRN0xGender	.095[c]	2.296	.023	.150	.966
	MRN0xParty0	.020[c]	.478	.633	.032	.950
	MRN0xMGRS0	-.060[c]	-1.454	.147	-.096	.972
	MGRS0xGender	.030[c]	.724	.470	.048	.985
	MGRS0xRace	.020[c]	.482	.631	.032	.975
	MGRS0xSES0	-.057[c]	-1.383	.168	-.091	.968
	MGRS0xParty0	-.002[c]	-.052	.959	-.003	.977
3	MRN0xRace	.064[d]	1.410	.160	.093	.755
	MRN0xSES0	.014[d]	.337	.736	.022	.948
	MRN0xGender	.067[d]	1.623	.106	.107	.932
	MRN0xParty0	.042[d]	1.013	.312	.067	.928
	MRN0xMGRS0	-.049[d]	-1.205	.229	-.080	.964
	MGRS0xGender	.025[d]	.615	.539	.041	.947

Excluded Variables[a]

Model		Beta In	t	Sig.	Partial Correlation	Collinearity Statistics Tolerance
	MGRS0xRace	-.024[d]	-.511	.610	-.034	.713
	MGRS0xSES0	-.022[d]	-.518	.605	-.034	.893
	MGRS0xParty0	.019[d]	.476	.635	.032	.960

a. Dependent Variable: TrumpX

b. Predictors in the Model: (Constant), Party0

c. Predictors in the Model: (Constant), Party0, SES0, GenderCC, RaceCC

d. Predictors in the Model: (Constant), Party0, SES0, GenderCC, RaceCC, MGRS0, MRN0

```
REGRESSION
  /MISSING LISTWISE
  /STATISTICS COEFF OUTS R ANOVA CHANGE ZPP
  /CRITERIA=PIN(.05) POUT(.10)
  /NOORIGIN
  /DEPENDENT BidenX
  /METHOD=ENTER Party0
  /METHOD=ENTER GenderCC RaceCC SES0
  /METHOD=ENTER MGRS0 MRN0
  /METHOD=ENTER MRN0xRace MRN0xSES0 MRN0xGender MRN0xParty0 MRN0xMGRS0 MGRS0xGender MGRS0
xRace MGRS0xSES0 MGRS0xParty0.
```

Regression

Notes

Output Created		15-DEC-2021 13:07:58
Comments		
Input	Data	C: \Users\njs5478\Dropbox\HM and COVID\0. Revise and Resubmit\2. R and R Data\Study 1b\Study1b_Data.sav
	Active Dataset	DataSet1
	Filter	<none>
	Weight	<none>
	Split File	<none>
	N of Rows in Working Data File	241
Missing Value Handling	Definition of Missing	User-defined missing values are treated as missing.
	Cases Used	Statistics are based on cases with no missing values for any variable used.
Syntax		REGRESSION<br>/MISSING LISTWISE<br>/STATISTICS COEFF OUTS R ANOVA CHANGE ZPP<br>/CRITERIA=PIN(.05) POUT(.10)<br>/NOORIGIN<br>/DEPENDENT BidenX<br>/METHOD=ENTER Party0<br>/METHOD=ENTER GenderCC RaceCC SES0<br>/METHOD=ENTER MGRS0 MRN0<br>/METHOD=ENTER MRN0xRace MRN0xSES0 MRN0xGender MRN0xParty0 MRN0xMGRS0 MGRS0xGender MGRS0xRace MGRS0xSES0 MGRS0xParty0.
Resources	Processor Time	00:00:00.02
	Elapsed Time	00:00:00.02

Notes

	Memory Required	52240 bytes
	Additional Memory Required for Residual Plots	0 bytes

Variables Entered/Removed[a]

Model	Variables Entered	Variables Removed	Method
1	Party0[b]	.	Enter
2	SES0, GenderCC, RaceCC[b]	.	Enter
3	MGRS0, MRN0[b]	.	Enter
4	MRN0xMGRS0, MRN0xSES0, MRN0xParty0, MGRS0xSES0, MGRS0xGender, MGRS0xRace, MRN0xRace, MRN0xGender, ...[b]	.	Enter

a. Dependent Variable: BidenX

b. All requested variables entered.

Model Summary

Model	R	R Square	Adjusted R Square	Std. Error of the Estimate	Change Statistics R Square Change	F Change	df1
1	.703[a]	.495	.493	1.27907	.495	222.410	1
2	.707[b]	.500	.491	1.28083	.005	.792	3
3	.715[c]	.512	.499	1.27147	.012	2.655	2
4	.729[d]	.531	.498	1.27223	.019	.971	9

Model Summary

Model	Change Statistics df2	Sig. F Change
1	227	.000
2	224	.500
3	222	.073
4	213	.465

a. Predictors: (Constant), Party0

b. Predictors: (Constant), Party0, SES0, GenderCC, RaceCC

c. Predictors: (Constant), Party0, SES0, GenderCC, RaceCC, MGRS0, MRN0

d. Predictors: (Constant), Party0, SES0, GenderCC, RaceCC, MGRS0, MRN0, MRN0xMGRS0, MRN0xSES0, MRN0xParty0, MGRS0xSES0, MGRS0xGender, MGRS0xRace, MRN0xRace, MRN0xGender, MGRS0xParty0

ANOVA[a]

Model		Sum of Squares	df	Mean Square	F	Sig.
1	Regression	363.866	1	363.866	222.410	.000[b]
	Residual	371.374	227	1.636		
	Total	735.240	228			
2	Regression	367.763	4	91.941	56.043	.000[c]
	Residual	367.477	224	1.641		
	Total	735.240	228			
3	Regression	376.346	6	62.724	38.799	.000[d]
	Residual	358.894	222	1.617		
	Total	735.240	228			
4	Regression	390.486	15	26.032	16.084	.000[e]
	Residual	344.755	213	1.619		
	Total	735.240	228			

a. Dependent Variable: BidenX

b. Predictors: (Constant), Party0

c. Predictors: (Constant), Party0, SES0, GenderCC, RaceCC

d. Predictors: (Constant), Party0, SES0, GenderCC, RaceCC, MGRS0, MRN0

e. Predictors: (Constant), Party0, SES0, GenderCC, RaceCC, MGRS0, MRN0, MRN0xMGRS0, MRN0xSES0, MRN0xParty0, MGRS0xSES0, MGRS0xGender, MGRS0xRace, MRN0xRace, MRN0xGender, MGRS0xParty0

Coefficients[a]

Model		Unstandardized Coefficients B	Std. Error	Standardized Coefficients Beta	t	Sig.
1	(Constant)	4.479	.085		52.980	.000
	Party0	-.900	.060	-.703	-14.913	.000
2	(Constant)	4.476	.098		45.600	.000
	Party0	-.896	.065	-.701	-13.758	.000
	GenderCC	-.053	.088	-.029	-.602	.548
	RaceCC	.014	.101	.007	.139	.889
	SES0	.148	.105	.067	1.419	.157
3	(Constant)	4.485	.098		45.912	.000
	Party0	-.829	.071	-.648	-11.607	.000
	GenderCC	.019	.104	.011	.186	.852
	RaceCC	-.009	.101	-.004	-.089	.929
	SES0	.173	.105	.078	1.653	.100
	MGRS0	-.015	.147	-.005	-.101	.920
	MRN0	-.267	.125	-.130	-2.131	.034
4	(Constant)	4.544	.131		34.590	.000
	Party0	-.797	.074	-.623	-10.712	.000
	GenderCC	-.022	.108	-.012	-.202	.840
	RaceCC	-.053	.105	-.026	-.504	.615
	SES0	.179	.107	.081	1.663	.098
	MGRS0	-.177	.189	-.064	-.939	.349
	MRN0	-.194	.142	-.094	-1.367	.173
	MRN0xRace	-.096	.119	-.047	-.806	.421
	MRN0xSES0	-.020	.113	-.009	-.179	.858
	MRN0xGender	-.226	.142	-.100	-1.595	.112
	MRN0xParty0	.101	.080	.069	1.257	.210
	MRN0xMGRS0	.188	.202	.068	.930	.353
	MGRS0xGender	.104	.180	.036	.579	.563
	MGRS0xRace	.249	.174	.090	1.429	.154
	MGRS0xSES0	-.061	.179	-.018	-.341	.733
	MGRS0xParty0	-.333	.135	-.178	-2.465	.014

Coefficients[a]

Model		Correlations Zero-order	Partial	Part
1	(Constant)			
	Party0	-.703	-.703	-.703
2	(Constant)			
	Party0	-.703	-.677	-.650
	GenderCC	-.208	-.040	-.028
	RaceCC	-.190	.009	.007
	SES0	.034	.094	.067
3	(Constant)			
	Party0	-.703	-.615	-.544
	GenderCC	-.208	.013	.009
	RaceCC	-.190	-.006	-.004
	SES0	.034	.110	.078
	MGRS0	.011	-.007	-.005
	MRN0	-.420	-.142	-.100
4	(Constant)			
	Party0	-.703	-.592	-.503
	GenderCC	-.208	-.014	-.009
	RaceCC	-.190	-.034	-.024
	SES0	.034	.113	.078
	MGRS0	.011	-.064	-.044
	MRN0	-.420	-.093	-.064
	MRN0xRace	-.304	-.055	-.038
	MRN0xSES0	.091	-.012	-.008
	MRN0xGender	-.162	-.109	-.075
	MRN0xParty0	-.093	.086	.059
	MRN0xMGRS0	.012	.064	.044
	MGRS0xGender	-.083	.040	.027
	MGRS0xRace	-.032	.097	.067
	MGRS0xSES0	.021	-.023	-.016
	MGRS0xParty0	-.147	-.167	-.116

a. Dependent Variable: BidenX

Excluded Variables[a]

Model		Beta In	t	Sig.	Partial Correlation	Collinearity Statistics Tolerance
1	GenderCC	-.028[b]	-.568	.571	-.038	.933
	RaceCC	.010[b]	.207	.836	.014	.920
	SES0	.067[b]	1.416	.158	.094	.998
	MGRS0	-.033[b]	-.699	.485	-.046	.996
	MRN0	-.117[b]	-2.213	.028	-.146	.782
	MRN0xRace	-.074[b]	-1.485	.139	-.098	.886
	MRN0xSES0	.013[b]	.274	.784	.018	.988
	MRN0xGender	-.070[b]	-1.483	.139	-.098	.983
	MRN0xParty0	.015[b]	.310	.757	.021	.977
	MRN0xMGRS0	-.008[b]	-.169	.866	-.011	.999
	MGRS0xGender	-.027[b]	-.565	.573	-.038	.994
	MGRS0xRace	.008[b]	.178	.859	.012	.997
	MGRS0xSES0	.003[b]	.056	.956	.004	.999
	MGRS0xParty0	-.057[b]	-1.189	.236	-.079	.983
2	MGRS0	-.044[c]	-.870	.385	-.058	.870
	MRN0	-.132[c]	-2.307	.022	-.153	.668
	MRN0xRace	-.073[c]	-1.435	.153	-.096	.862
	MRN0xSES0	.016[c]	.329	.743	.022	.985
	MRN0xGender	-.075[c]	-1.555	.121	-.104	.965
	MRN0xParty0	.025[c]	.521	.603	.035	.949
	MRN0xMGRS0	.003[c]	.057	.955	.004	.974
	MGRS0xGender	-.034[c]	-.714	.476	-.048	.983
	MGRS0xRace	.004[c]	.083	.934	.006	.971
	MGRS0xSES0	.013[c]	.270	.787	.018	.972
	MGRS0xParty0	-.054[c]	-1.127	.261	-.075	.976
3	MRN0xRace	-.036[d]	-.664	.507	-.045	.751
	MRN0xSES0	-.004[d]	-.092	.926	-.006	.949
	MRN0xGender	-.056[d]	-1.145	.254	-.077	.928
	MRN0xParty0	.009[d]	.192	.848	.013	.926
	MRN0xMGRS0	-.006[d]	-.133	.895	-.009	.966
	MGRS0xGender	-.027[d]	-.548	.584	-.037	.942

Excluded Variables[a]

Model		Beta In	t	Sig.	Partial Correlation	Collinearity Statistics Tolerance
	MGRS0xRace	.024[d]	.432	.666	.029	.702
	MGRS0xSES0	-.009[d]	-.173	.863	-.012	.887
	MGRS0xParty0	-.069[d]	-1.448	.149	-.097	.958

a. Dependent Variable: BidenX

b. Predictors in the Model: (Constant), Party0

c. Predictors in the Model: (Constant), Party0, SES0, GenderCC, RaceCC

d. Predictors in the Model: (Constant), Party0, SES0, GenderCC, RaceCC, MGRS0, MRN0

```
REGRESSION
  /MISSING LISTWISE
  /STATISTICS COEFF OUTS R ANOVA CHANGE ZPP
  /CRITERIA=PIN(.05) POUT(.10)
  /NOORIGIN
  /DEPENDENT PelosiX
  /METHOD=ENTER Party0
  /METHOD=ENTER GenderCC RaceCC SES0
  /METHOD=ENTER MGRS0 MRN0
  /METHOD=ENTER MRN0xRace MRN0xSES0 MRN0xGender MRN0xParty0 MRN0xMGRS0 MGRS0xGender MGRS0
xRace MGRS0xSES0 MGRS0xParty0.
```

Regression

Notes

Output Created		15-DEC-2021 13:07:58
Comments		
Input	Data	C:<br>\Users\njs5478\Dropbox\HM and COVID\0. Revise and Resubmit\2. R and R Data\Study 1b\Study1b_Data.sav
	Active Dataset	DataSet1
	Filter	<none>
	Weight	<none>
	Split File	<none>
	N of Rows in Working Data File	241
Missing Value Handling	Definition of Missing	User-defined missing values are treated as missing.
	Cases Used	Statistics are based on cases with no missing values for any variable used.
Syntax		REGRESSION<br>/MISSING LISTWISE<br>/STATISTICS COEFF OUTS R ANOVA CHANGE ZPP<br>/CRITERIA=PIN(.05) POUT(.10)<br>/NOORIGIN<br>/DEPENDENT PelosiX<br>/METHOD=ENTER Party0<br>/METHOD=ENTER GenderCC RaceCC SES0<br>/METHOD=ENTER MGRS0 MRN0<br>/METHOD=ENTER MRN0xRace MRN0xSES0 MRN0xGender MRN0xParty0 MRN0xMGRS0 MGRS0xGender MGRS0xRace MGRS0xSES0 MGRS0xParty0.
Resources	Processor Time	00:00:00.02
	Elapsed Time	00:00:00.02

Notes

	Memory Required	52240 bytes
	Additional Memory Required for Residual Plots	0 bytes

Variables Entered/Removed[a]

Model	Variables Entered	Variables Removed	Method
1	Party0[b]	.	Enter
2	SES0, GenderCC, RaceCC[b]	.	Enter
3	MGRS0, MRN0[b]	.	Enter
4	MRN0xMGRS0, MRN0xSES0, MRN0xParty0, MGRS0xGender, MGRS0xSES0, MRN0xRace, MGRS0xRace, MRN0xGender, MGRS0xParty[b]...	.	Enter

a. Dependent Variable: PelosiX

b. All requested variables entered.

Model Summary

Model	R	R Square	Adjusted R Square	Std. Error of the Estimate	Change Statistics				
					R Square Change	F Change	df1	df2	Sig. F Change
1	.620[a]	.384	.381	1.28690	.384	122.748	1	197	.000
2	.635[b]	.403	.391	1.27630	.019	2.095	3	194	.102
3	.653[c]	.426	.408	1.25819	.023	3.812	2	192	.024
4	.669[d]	.448	.403	1.26371	.022	.814	9	183	.604

a. Predictors: (Constant), Party0

b. Predictors: (Constant), Party0, SES0, GenderCC, RaceCC

c. Predictors: (Constant), Party0, SES0, GenderCC, RaceCC, MGRS0, MRN0

d. Predictors: (Constant), Party0, SES0, GenderCC, RaceCC, MGRS0, MRN0, MRN0xMGRS0, MRN0xSES0, MRN0xParty0, MGRS0xGender, MGRS0xSES0, MRN0xRace, MGRS0xRace, MRN0xGender, MGRS0xParty0

ANOVA[a]

Model		Sum of Squares	df	Mean Square	F	Sig.
1	Regression	203.285	1	203.285	122.748	.000[b]
	Residual	326.253	197	1.656		
	Total	529.538	198			
2	Regression	213.524	4	53.381	32.771	.000[c]
	Residual	316.013	194	1.629		
	Total	529.538	198			
3	Regression	225.595	6	37.599	23.751	.000[d]
	Residual	303.943	192	1.583		
	Total	529.538	198			
4	Regression	237.295	15	15.820	9.906	.000[e]
	Residual	292.243	183	1.597		
	Total	529.538	198			

a. Dependent Variable: PelosiX

b. Predictors: (Constant), Party0

c. Predictors: (Constant), Party0, SES0, GenderCC, RaceCC

d. Predictors: (Constant), Party0, SES0, GenderCC, RaceCC, MGRS0, MRN0

e. Predictors: (Constant), Party0, SES0, GenderCC, RaceCC, MGRS0, MRN0, MRN0xMGRS0, MRN0xSES0, MRN0xParty0, MGRS0xGender, MGRS0xSES0, MRN0xRace, MGRS0xRace, MRN0xGender, MGRS0xParty0

Coefficients[a]

Model		Unstandardized Coefficients B	Std. Error	Standardized Coefficients Beta	t	Sig.
1	(Constant)	3.575	.091		39.155	.000
	Party0	-.726	.066	-.620	-11.079	.000
2	(Constant)	3.527	.106		33.184	.000
	Party0	-.704	.069	-.600	-10.146	.000
	GenderCC	-.198	.093	-.121	-2.117	.036
	RaceCC	.100	.109	.052	.914	.362
	SES0	-.103	.112	-.051	-.914	.362
3	(Constant)	3.548	.105		33.770	.000
	Party0	-.625	.076	-.533	-8.220	.000
	GenderCC	-.148	.109	-.091	-1.353	.178
	RaceCC	.062	.109	.033	.573	.567
	SES0	-.087	.111	-.043	-.786	.433
	MGRS0	-.154	.158	-.060	-.973	.332
	MRN0	-.287	.137	-.151	-2.100	.037
4	(Constant)	3.628	.138		26.197	.000
	Party0	-.619	.079	-.528	-7.783	.000
	GenderCC	-.165	.115	-.101	-1.439	.152
	RaceCC	.045	.112	.023	.400	.690
	SES0	-.056	.114	-.028	-.489	.625
	MGRS0	-.055	.211	-.022	-.262	.794
	MRN0	-.251	.160	-.132	-1.568	.119
	MRN0xRace	-.017	.134	-.009	-.126	.900
	MRN0xSES0	.048	.131	.022	.366	.715
	MRN0xGender	-.130	.152	-.062	-.854	.394
	MRN0xParty0	.030	.087	.022	.343	.732
	MRN0xMGRS0	.126	.227	.049	.555	.580
	MGRS0xGender	.241	.187	.089	1.289	.199
	MGRS0xRace	-.014	.198	-.006	-.073	.942
	MGRS0xSES0	.253	.194	.080	1.302	.194
	MGRS0xParty0	-.030	.149	-.017	-.199	.843

Coefficients[a]

Model		Correlations		
		Zero-order	Partial	Part
1	(Constant)			
	Party0	-.620	-.620	-.620
2	(Constant)			
	Party0	-.620	-.589	-.563
	GenderCC	-.267	-.150	-.117
	RaceCC	-.111	.065	.051
	SES0	-.094	-.066	-.051
3	(Constant)			
	Party0	-.620	-.510	-.449
	GenderCC	-.267	-.097	-.074
	RaceCC	-.111	.041	.031
	SES0	-.094	-.057	-.043
	MGRS0	-.036	-.070	-.053
	MRN0	-.456	-.150	-.115
4	(Constant)			
	Party0	-.620	-.499	-.427
	GenderCC	-.267	-.106	-.079
	RaceCC	-.111	.030	.022
	SES0	-.094	-.036	-.027
	MGRS0	-.036	-.019	-.014
	MRN0	-.456	-.115	-.086
	MRN0xRace	-.319	-.009	-.007
	MRN0xSES0	.107	.027	.020
	MRN0xGender	-.186	-.063	-.047
	MRN0xParty0	-.094	.025	.019
	MRN0xMGRS0	.108	.041	.030
	MGRS0xGender	.004	.095	.071
	MGRS0xRace	-.094	-.005	-.004
	MGRS0xSES0	.153	.096	.072
	MGRS0xParty0	-.010	-.015	-.011

a. Dependent Variable: PelosiX

Excluded Variables[a]

Model		Beta In	t	Sig.	Partial Correlation	Collinearity Statistics Tolerance
1	GenderCC	-.123[b]	-2.154	.032	-.152	.940
	RaceCC	.052[b]	.901	.369	.064	.933
	SES0	-.054[b]	-.972	.332	-.069	.996
	MGRS0	-.057[b]	-1.023	.308	-.073	.999
	MRN0	-.208[b]	-3.355	.001	-.233	.774
	MRN0xRace	-.109[b]	-1.832	.068	-.130	.869
	MRN0xSES0	.044[b]	.784	.434	.056	.989
	MRN0xGender	-.089[b]	-1.572	.118	-.112	.974
	MRN0xParty0	.014[b]	.241	.809	.017	.970
	MRN0xMGRS0	.102[b]	1.829	.069	.130	1.000
	MGRS0xGender	.065[b]	1.163	.246	.083	.990
	MGRS0xRace	-.043[b]	-.771	.442	-.055	.993
	MGRS0xSES0	.128[b]	2.315	.022	.163	.998
	MGRS0xParty0	.077[b]	1.365	.174	.097	.981
2	MGRS0	-.104[c]	-1.777	.077	-.127	.886
	MRN0	-.174[c]	-2.584	.010	-.183	.656
	MRN0xRace	-.089[c]	-1.478	.141	-.106	.847
	MRN0xSES0	.052[c]	.928	.355	.067	.979
	MRN0xGender	-.085[c]	-1.501	.135	-.107	.963
	MRN0xParty0	.017[c]	.294	.769	.021	.934
	MRN0xMGRS0	.108[c]	1.945	.053	.139	.978
	MGRS0xGender	.067[c]	1.207	.229	.087	.981
	MGRS0xRace	-.064[c]	-1.136	.257	-.081	.968
	MGRS0xSES0	.135[c]	2.421	.016	.172	.965
	MGRS0xParty0	.077[c]	1.364	.174	.098	.972
3	MRN0xRace	-.028[d]	-.415	.679	-.030	.681
	MRN0xSES0	.033[d]	.599	.550	.043	.963
	MRN0xGender	-.059[d]	-1.042	.298	-.075	.932
	MRN0xParty0	.005[d]	.090	.929	.006	.914
	MRN0xMGRS0	.100[d]	1.812	.072	.130	.967
	MGRS0xGender	.074[d]	1.324	.187	.095	.953

Excluded Variables[a]

Model		Beta In	t	Sig.	Partial Correlation	Collinearity Statistics Tolerance
	MGRS0xRace	-.026[d]	-.377	.707	-.027	.629
	MGRS0xSES0	.107[d]	1.856	.065	.133	.893
	MGRS0xParty0	.066[d]	1.189	.236	.086	.961

a. Dependent Variable: PelosiX

b. Predictors in the Model: (Constant), Party0

c. Predictors in the Model: (Constant), Party0, SES0, GenderCC, RaceCC

d. Predictors in the Model: (Constant), Party0, SES0, GenderCC, RaceCC, MGRS0, MRN0

```
REGRESSION
  /MISSING LISTWISE
  /STATISTICS COEFF OUTS R ANOVA CHANGE ZPP
  /CRITERIA=PIN(.05) POUT(.10)
  /NOORIGIN
  /DEPENDENT McConnellX
  /METHOD=ENTER Party0
  /METHOD=ENTER GenderCC RaceCC SES0
  /METHOD=ENTER MGRS0 MRN0
  /METHOD=ENTER MRN0xRace MRN0xSES0 MRN0xGender MRN0xParty0 MRN0xMGRS0 MGRS0xGender MGRS0
xRace MGRS0xSES0 MGRS0xParty0.
```

Regression

Notes

Output Created		15-DEC-2021 13:07:58
Comments		
Input	Data	C: \Users\njs5478\Dropbox\HM and COVID\0. Revise and Resubmit\2. R and R Data\Study 1b\Study1b_Data.sav
	Active Dataset	DataSet1
	Filter	<none>
	Weight	<none>
	Split File	<none>
	N of Rows in Working Data File	241
Missing Value Handling	Definition of Missing	User-defined missing values are treated as missing.
	Cases Used	Statistics are based on cases with no missing values for any variable used.
Syntax		REGRESSION<br>/MISSING LISTWISE<br>/STATISTICS COEFF OUTS R ANOVA CHANGE ZPP<br>/CRITERIA=PIN(.05) POUT(.10)<br>/NOORIGIN<br>/DEPENDENT McConnellX<br>/METHOD=ENTER Party0<br>/METHOD=ENTER GenderCC RaceCC SES0<br>/METHOD=ENTER MGRS0 MRN0<br>/METHOD=ENTER MRN0xRace MRN0xSES0 MRN0xGender MRN0xParty0 MRN0xMGRS0 MGRS0xGender MGRS0xRace MGRS0xSES0 MGRS0xParty0.

Notes

Resources	Processor Time	00:00:00.03
	Elapsed Time	00:00:00.03
	Memory Required	52240 bytes
	Additional Memory Required for Residual Plots	0 bytes

Variables Entered/Removed[a]

Model	Variables Entered	Variables Removed	Method
1	Party0[b]	.	Enter
2	SES0, RaceCC, GenderCC[b]	.	Enter
3	MGRS0, MRN0[b]	.	Enter
4	MRN0xMGRS0, MRN0xSES0, MRN0xParty0, MGRS0xGender, MGRS0xSES0, MGRS0xRace, MRN0xRace, MRN0xGender, ...[b]	.	Enter

a. Dependent Variable: McConnellX

b. All requested variables entered.

Model Summary

Model	R	R Square	Adjusted R Square	Std. Error of the Estimate	Change Statistics		
					R Square Change	F Change	df1
1	.563[a]	.317	.314	1.27464	.317	88.752	1
2	.586[b]	.343	.329	1.26024	.026	2.464	3
3	.626[c]	.392	.373	1.21852	.049	7.546	2
4	.659[d]	.435	.387	1.20472	.042	1.476	9

Model Summary

Model	Change Statistics	
	df2	Sig. F Change
1	191	.000
2	188	.064
3	186	.001
4	177	.160

a. Predictors: (Constant), Party0

b. Predictors: (Constant), Party0, SES0, RaceCC, GenderCC

c. Predictors: (Constant), Party0, SES0, RaceCC, GenderCC, MGRS0, MRN0

d. Predictors: (Constant), Party0, SES0, RaceCC, GenderCC, MGRS0, MRN0, MRN0xMGRS0, MRN0xSES0, MRN0xParty0, MGRS0xGender, MGRS0xSES0, MGRS0xRace, MRN0xRace, MRN0xGender, MGRS0xParty0

ANOVA[a]

Model		Sum of Squares	df	Mean Square	F	Sig.
1	Regression	144.197	1	144.197	88.752	.000[b]
	Residual	310.321	191	1.625		
	Total	454.518	192			
2	Regression	155.938	4	38.984	24.546	.000[c]
	Residual	298.580	188	1.588		
	Total	454.518	192			
3	Regression	178.346	6	29.724	20.019	.000[d]
	Residual	276.172	186	1.485		
	Total	454.518	192			
4	Regression	197.629	15	13.175	9.078	.000[e]
	Residual	256.889	177	1.451		
	Total	454.518	192			

a. Dependent Variable: McConnellX

b. Predictors: (Constant), Party0

c. Predictors: (Constant), Party0, SES0, RaceCC, GenderCC

d. Predictors: (Constant), Party0, SES0, RaceCC, GenderCC, MGRS0, MRN0

e. Predictors: (Constant), Party0, SES0, RaceCC, GenderCC, MGRS0, MRN0, MRN0xMGRS0, MRN0xSES0, MRN0xParty0, MGRS0xGender, MGRS0xSES0, MGRS0xRace, MRN0xRace, MRN0xGender, MGRS0xParty0

Coefficients[a]

Model		Unstandardized Coefficients B	Std. Error	Standardized Coefficients Beta	t	Sig.
1	(Constant)	3.212	.092		35.005	.000
	Party0	.634	.067	.563	9.421	.000
2	(Constant)	3.185	.107		29.804	.000
	Party0	.668	.071	.593	9.445	.000
	GenderCC	-.246	.094	-.160	-2.626	.009
	RaceCC	.073	.109	.041	.672	.502
	SES0	-.026	.114	-.014	-.231	.817
3	(Constant)	3.173	.104		30.653	.000
	Party0	.576	.076	.512	7.604	.000
	GenderCC	-.263	.109	-.171	-2.424	.016
	RaceCC	.122	.106	.068	1.151	.251
	SES0	-.052	.111	-.027	-.473	.637
	MGRS0	.309	.154	.131	2.006	.046
	MRN0	.315	.130	.180	2.413	.017
4	(Constant)	3.172	.136		23.265	.000
	Party0	.526	.078	.467	6.735	.000
	GenderCC	-.233	.112	-.151	-2.081	.039
	RaceCC	.162	.109	.091	1.490	.138
	SES0	-.057	.113	-.030	-.509	.611
	MGRS0	.446	.195	.189	2.290	.023
	MRN0	.136	.148	.078	.919	.360
	MRN0xRace	.338	.126	.193	2.691	.008
	MRN0xSES0	-.102	.120	-.051	-.852	.395
	MRN0xGender	-.015	.142	-.008	-.108	.914
	MRN0xParty0	.023	.082	.019	.283	.777
	MRN0xMGRS0	-.003	.213	-.001	-.015	.988
	MGRS0xGender	.182	.181	.072	1.004	.317
	MGRS0xRace	-.227	.182	-.096	-1.248	.214
	MGRS0xSES0	-.030	.184	-.010	-.164	.870
	MGRS0xParty0	.124	.144	.079	.862	.390

Coefficients[a]

Model		Correlations Zero-order	Partial	Part
1	(Constant)			
	Party0	.563	.563	.563
2	(Constant)			
	Party0	.563	.567	.558
	GenderCC	-.011	-.188	-.155
	RaceCC	.175	.049	.040
	SES0	.009	-.017	-.014
3	(Constant)			
	Party0	.563	.487	.435
	GenderCC	-.011	-.175	-.139
	RaceCC	.175	.084	.066
	SES0	.009	-.035	-.027
	MGRS0	.186	.145	.115
	MRN0	.366	.174	.138
4	(Constant)			
	Party0	.563	.452	.381
	GenderCC	-.011	-.155	-.118
	RaceCC	.175	.111	.084
	SES0	.009	-.038	-.029
	MGRS0	.186	.170	.129
	MRN0	.366	.069	.052
	MRN0xRace	.369	.198	.152
	MRN0xSES0	-.163	-.064	-.048
	MRN0xGender	.158	-.008	-.006
	MRN0xParty0	.096	.021	.016
	MRN0xMGRS0	.030	-.001	-.001
	MGRS0xGender	.146	.075	.057
	MGRS0xRace	.117	-.093	-.071
	MGRS0xSES0	-.109	-.012	-.009
	MGRS0xParty0	.125	.065	.049

a. Dependent Variable: McConnellX

Excluded Variables[a]

Model		Beta In	t	Sig.	Partial Correlation	Collinearity Statistics Tolerance
1	GenderCC	-.160[b]	-2.635	.009	-.188	.939
	RaceCC	.038[b]	.609	.543	.044	.938
	SES0	-.020[b]	-.336	.737	-.024	.997
	MGRS0	.213[b]	3.674	.000	.258	.998
	MRN0	.131[b]	1.955	.052	.140	.781
	MRN0xRace	.198[b]	3.179	.002	.225	.881
	MRN0xSES0	-.110[b]	-1.839	.067	-.132	.991
	MRN0xGender	.074[b]	1.225	.222	.089	.977
	MRN0xParty0	.036[b]	.590	.556	.043	.988
	MRN0xMGRS0	.030[b]	.499	.619	.036	1.000
	MGRS0xGender	.084[b]	1.396	.164	.101	.987
	MGRS0xRace	.069[b]	1.159	.248	.084	.993
	MGRS0xSES0	-.064[b]	-1.075	.284	-.078	.994
	MGRS0xParty0	.043[b]	.718	.473	.052	.979
2	MGRS0	.187[c]	3.006	.003	.215	.868
	MRN0	.233[c]	3.300	.001	.235	.669
	MRN0xRace	.230[c]	3.691	.000	.261	.846
	MRN0xSES0	-.105[c]	-1.766	.079	-.128	.982
	MRN0xGender	.063[c]	1.035	.302	.076	.954
	MRN0xParty0	.045[c]	.752	.453	.055	.957
	MRN0xMGRS0	.042[c]	.700	.485	.051	.970
	MGRS0xGender	.080[c]	1.339	.182	.097	.980
	MGRS0xRace	.046[c]	.768	.444	.056	.970
	MGRS0xSES0	-.057[c]	-.940	.349	-.069	.958
	MGRS0xParty0	.051[c]	.841	.401	.061	.961
3	MRN0xRace	.174[d]	2.568	.011	.186	.695
	MRN0xSES0	-.070[d]	-1.198	.232	-.088	.955
	MRN0xGender	.031[d]	.529	.597	.039	.934
	MRN0xParty0	.063[d]	1.061	.290	.078	.926
	MRN0xMGRS0	.057[d]	.977	.330	.072	.960
	MGRS0xGender	.087[d]	1.502	.135	.110	.957

Excluded Variables[a]

Model		Beta In	t	Sig.	Partial Correlation	Collinearity Statistics Tolerance
	MGRS0xRace	-.058[d]	-.832	.406	-.061	.673
	MGRS0xSES0	.002[d]	.028	.977	.002	.886
	MGRS0xParty0	.068[d]	1.162	.247	.085	.950

a. Dependent Variable: McConnellX

b. Predictors in the Model: (Constant), Party0

c. Predictors in the Model: (Constant), Party0, SES0, RaceCC, GenderCC

d. Predictors in the Model: (Constant), Party0, SES0, RaceCC, GenderCC, MGRS0, MRN0

```
REGRESSION
  /MISSING LISTWISE
  /STATISTICS COEFF OUTS R ANOVA CHANGE ZPP
  /CRITERIA=PIN(.05) POUT(.10)
  /NOORIGIN
  /DEPENDENT FauciX
  /METHOD=ENTER Party0
  /METHOD=ENTER GenderCC RaceCC SES0
  /METHOD=ENTER MGRS0 MRN0
  /METHOD=ENTER MRN0xRace MRN0xSES0 MRN0xGender MRN0xParty0 MRN0xMGRS0 MGRS0xGender MGRS0
xRace MGRS0xSES0 MGRS0xParty0.
```

Regression

Notes

Output Created		15-DEC-2021 13:07:58
Comments		
Input	Data	C: \Users\njs5478\Dropbox\HM and COVID\0. Revise and Resubmit\2. R and R Data\Study 1b\Study1b_Data.sav
	Active Dataset	DataSet1
	Filter	<none>
	Weight	<none>
	Split File	<none>
	N of Rows in Working Data File	241
Missing Value Handling	Definition of Missing	User-defined missing values are treated as missing.
	Cases Used	Statistics are based on cases with no missing values for any variable used.
Syntax		REGRESSION<br>/MISSING LISTWISE<br>/STATISTICS COEFF OUTS R ANOVA CHANGE ZPP<br>/CRITERIA=PIN(.05) POUT(.10)<br>/NOORIGIN<br>/DEPENDENT FauciX<br>/METHOD=ENTER Party0<br>/METHOD=ENTER GenderCC RaceCC SES0<br>/METHOD=ENTER MGRS0 MRN0<br>/METHOD=ENTER MRN0xRace MRN0xSES0 MRN0xGender MRN0xParty0 MRN0xMGRS0 MGRS0xGender MGRS0xRace MGRS0xSES0 MGRS0xParty0.
Resources	Processor Time	00:00:00.06
	Elapsed Time	00:00:00.03

Notes

	Memory Required	52240 bytes
	Additional Memory Required for Residual Plots	0 bytes

Variables Entered/Removed[a]

Model	Variables Entered	Variables Removed	Method
1	Party0[b]	.	Enter
2	SES0, GenderCC, RaceCC[b]	.	Enter
3	MGRS0, MRN0[b]	.	Enter
4	MGRS0xGender, MRN0xSES0, MRN0xParty0, MGRS0xSES0, MGRS0xParty0, MRN0xRace, MRN0xGender, MGRS0xRace, MRN0xMGRS...[b]	.	Enter

a. Dependent Variable: FauciX

b. All requested variables entered.

Model Summary

Model	R	R Square	Adjusted R Square	Std. Error of the Estimate	Change Statistics				
					R Square Change	F Change	df1	df2	Sig. F Change
1	.337[a]	.113	.109	1.55499	.113	26.748	1	209	.000
2	.361[b]	.131	.114	1.55100	.017	1.359	3	206	.257
3	.397[c]	.158	.133	1.53418	.027	3.272	2	204	.040
4	.460[d]	.212	.151	1.51769	.054	1.495	9	195	.152

a. Predictors: (Constant), Party0

b. Predictors: (Constant), Party0, SES0, GenderCC, RaceCC

c. Predictors: (Constant), Party0, SES0, GenderCC, RaceCC, MGRS0, MRN0

d. Predictors: (Constant), Party0, SES0, GenderCC, RaceCC, MGRS0, MRN0, MGRS0xGender, MRN0xSES0, MRN0xParty0, MGRS0xSES0, MGRS0xParty0, MRN0xRace, MRN0xGender, MGRS0xRace, MRN0xMGRS0

ANOVA[a]

Model		Sum of Squares	df	Mean Square	F	Sig.
1	Regression	64.677	1	64.677	26.748	.000[b]
	Residual	505.361	209	2.418		
	Total	570.038	210			
2	Regression	74.482	4	18.621	7.740	.000[c]
	Residual	495.556	206	2.406		
	Total	570.038	210			
3	Regression	89.883	6	14.981	6.365	.000[d]
	Residual	480.154	204	2.354		
	Total	570.038	210			
4	Regression	120.876	15	8.058	3.498	.000[e]
	Residual	449.162	195	2.303		
	Total	570.038	210			

a. Dependent Variable: FauciX

b. Predictors: (Constant), Party0

c. Predictors: (Constant), Party0, SES0, GenderCC, RaceCC

d. Predictors: (Constant), Party0, SES0, GenderCC, RaceCC, MGRS0, MRN0

e. Predictors: (Constant), Party0, SES0, GenderCC, RaceCC, MGRS0, MRN0, MGRS0xGender, MRN0xSES0, MRN0xParty0, MGRS0xSES0, MGRS0xParty0, MRN0xRace, MRN0xGender, MGRS0xRace, MRN0xMGRS0

Coefficients[a]

Model		Unstandardized Coefficients B	Unstandardized Coefficients Std. Error	Standardized Coefficients Beta	t	Sig.
1	(Constant)	5.128	.107		47.898	.000
	Party0	-.399	.077	-.337	-5.172	.000
2	(Constant)	5.045	.121		41.641	.000
	Party0	-.442	.083	-.374	-5.311	.000
	GenderCC	.022	.111	.013	.198	.844
	RaceCC	.174	.126	.094	1.383	.168
	SES0	.185	.134	.090	1.382	.168
3	(Constant)	5.055	.120		42.011	.000
	Party0	-.346	.091	-.292	-3.825	.000
	GenderCC	.188	.133	.114	1.412	.160
	RaceCC	.154	.125	.083	1.226	.221
	SES0	.226	.134	.110	1.692	.092
	MGRS0	.160	.187	.062	.854	.394
	MRN0	-.416	.163	-.218	-2.558	.011
4	(Constant)	4.933	.161		30.645	.000
	Party0	-.316	.095	-.267	-3.340	.001
	GenderCC	.133	.135	.081	.983	.327
	RaceCC	.160	.128	.086	1.251	.213
	SES0	.266	.135	.130	1.967	.051
	MGRS0	.058	.231	.023	.253	.801
	MRN0	-.338	.176	-.177	-1.917	.057
	MRN0xRace	-.046	.148	-.024	-.310	.757
	MRN0xSES0	.006	.146	.003	.044	.965
	MRN0xGender	-.117	.175	-.054	-.670	.504
	MRN0xParty0	.019	.100	.014	.187	.852
	MRN0xMGRS0	.368	.260	.138	1.416	.158
	MGRS0xGender	-.550	.221	-.203	-2.493	.014
	MGRS0xRace	-.069	.213	-.027	-.323	.747
	MGRS0xSES0	-.134	.220	-.042	-.608	.544
	MGRS0xParty0	-.133	.170	-.076	-.782	.435

Coefficients[a]

Model		Correlations Zero-order	Partial	Part
1	(Constant)			
	Party0	-.337	-.337	-.337
2	(Constant)			
	Party0	-.337	-.347	-.345
	GenderCC	-.073	.014	.013
	RaceCC	-.008	.096	.090
	SES0	.073	.096	.090
3	(Constant)			
	Party0	-.337	-.259	-.246
	GenderCC	-.073	.098	.091
	RaceCC	-.008	.086	.079
	SES0	.073	.118	.109
	MGRS0	.009	.060	.055
	MRN0	-.273	-.176	-.164
4	(Constant)			
	Party0	-.337	-.233	-.212
	GenderCC	-.073	.070	.062
	RaceCC	-.008	.089	.079
	SES0	.073	.139	.125
	MGRS0	.009	.018	.016
	MRN0	-.273	-.136	-.122
	MRN0xRace	-.196	-.022	-.020
	MRN0xSES0	.058	.003	.003
	MRN0xGender	-.189	-.048	-.043
	MRN0xParty0	-.022	.013	.012
	MRN0xMGRS0	.054	.101	.090
	MGRS0xGender	-.215	-.176	-.158
	MGRS0xRace	-.034	-.023	-.021
	MGRS0xSES0	.005	-.044	-.039
	MGRS0xParty0	-.056	-.056	-.050

a. Dependent Variable: FauciX

Excluded Variables[a]

Model		Beta In	t	Sig.	Partial Correlation	Collinearity Statistics Tolerance
1	GenderCC	.018[b]	.265	.791	.018	.929
	RaceCC	.098[b]	1.443	.151	.100	.916
	SES0	.095[b]	1.461	.146	.101	.996
	MGRS0	-.022[b]	-.340	.734	-.024	.991
	MRN0	-.150[b]	-2.059	.041	-.141	.789
	MRN0xRace	-.095[b]	-1.380	.169	-.095	.892
	MRN0xSES0	.035[b]	.540	.590	.037	.995
	MRN0xGender	-.154[b]	-2.385	.018	-.163	.989
	MRN0xParty0	.046[b]	.694	.488	.048	.962
	MRN0xMGRS0	.043[b]	.660	.510	.046	.999
	MGRS0xGender	-.194[b]	-3.028	.003	-.205	.996
	MGRS0xRace	-.008[b]	-.127	.899	-.009	.994
	MGRS0xSES0	-.009[b]	-.144	.885	-.010	.998
	MGRS0xParty0	.001[b]	.010	.992	.001	.972
2	MGRS0	-.003[c]	-.046	.963	-.003	.881
	MRN0	-.193[c]	-2.413	.017	-.166	.647
	MRN0xRace	-.087[c]	-1.262	.208	-.088	.878
	MRN0xSES0	.038[c]	.587	.558	.041	.992
	MRN0xGender	-.147[c]	-2.253	.025	-.155	.973
	MRN0xParty0	.048[c]	.719	.473	.050	.944
	MRN0xMGRS0	.053[c]	.802	.423	.056	.976
	MGRS0xGender	-.204[c]	-3.195	.002	-.218	.991
	MGRS0xRace	-.014[c]	-.218	.827	-.015	.973
	MGRS0xSES0	-.008[c]	-.120	.904	-.008	.972
	MGRS0xParty0	-.002[c]	-.033	.974	-.002	.962
3	MRN0xRace	-.037[d]	-.515	.607	-.036	.784
	MRN0xSES0	.029[d]	.445	.657	.031	.970
	MRN0xGender	-.131[d]	-2.007	.046	-.139	.959
	MRN0xParty0	.024[d]	.362	.718	.025	.924
	MRN0xMGRS0	.037[d]	.556	.579	.039	.954
	MGRS0xGender	-.193[d]	-3.026	.003	-.208	.973

Excluded Variables[a]

Model		Beta In	t	Sig.	Partial Correlation	Collinearity Statistics Tolerance
	MGRS0xRace	-.034[d]	-.455	.649	-.032	.727
	MGRS0xSES0	-.024[d]	-.355	.723	-.025	.912
	MGRS0xParty0	-.026[d]	-.388	.698	-.027	.944

a. Dependent Variable: FauciX

b. Predictors in the Model: (Constant), Party0

c. Predictors in the Model: (Constant), Party0, SES0, GenderCC, RaceCC

d. Predictors in the Model: (Constant), Party0, SES0, GenderCC, RaceCC, MGRS0, MRN0

```
REGRESSION
  /MISSING LISTWISE
  /STATISTICS COEFF OUTS R ANOVA CHANGE ZPP
  /CRITERIA=PIN(.05) POUT(.10)
  /NOORIGIN
  /DEPENDENT RepCongressX
  /METHOD=ENTER Party0
  /METHOD=ENTER GenderCC RaceCC SES0
  /METHOD=ENTER MGRS0 MRN0
  /METHOD=ENTER MRN0xRace MRN0xSES0 MRN0xGender MRN0xParty0 MRN0xMGRS0 MGRS0xGender MGRS0
xRace MGRS0xSES0 MGRS0xParty0.
```

Regression

Notes

Output Created		15-DEC-2021 13:07:58
Comments		
Input	Data	C: \Users\njs5478\Dropbox\HM and COVID\0. Revise and Resubmit\2. R and R Data\Study 1b\Study1b_Data.sav
	Active Dataset	DataSet1
	Filter	<none>
	Weight	<none>
	Split File	<none>
	N of Rows in Working Data File	241
Missing Value Handling	Definition of Missing	User-defined missing values are treated as missing.
	Cases Used	Statistics are based on cases with no missing values for any variable used.
Syntax		REGRESSION<br>/MISSING LISTWISE<br>/STATISTICS COEFF OUTS R ANOVA CHANGE ZPP<br>/CRITERIA=PIN(.05) POUT(.10)<br>/NOORIGIN<br>/DEPENDENT RepCongressX<br>/METHOD=ENTER Party0<br>/METHOD=ENTER GenderCC RaceCC SES0<br>/METHOD=ENTER MGRS0 MRN0<br>/METHOD=ENTER MRN0xRace MRN0xSES0 MRN0xGender MRN0xParty0 MRN0xMGRS0 MGRS0xGender MGRS0xRace MGRS0xSES0 MGRS0xParty0.

Notes

Resources	Processor Time	00:00:00.02
	Elapsed Time	00:00:00.02
	Memory Required	52240 bytes
	Additional Memory Required for Residual Plots	0 bytes

Variables Entered/Removed[a]

Model	Variables Entered	Variables Removed	Method
1	Party0[b]	.	Enter
2	SES0, GenderCC, RaceCC[b]	.	Enter
3	MGRS0, MRN0[b]	.	Enter
4	MRN0xMGRS0, MRN0xSES0, MRN0xParty0, MGRS0xSES0, MGRS0xGender, MRN0xRace, MGRS0xRace, MRN0xGender, MGRS0xParty...[b]	.	Enter

a. Dependent Variable: RepCongressX

b. All requested variables entered.

Model Summary

Model	R	R Square	Adjusted R Square	Std. Error of the Estimate	Change Statistics		
					R Square Change	F Change	df1
1	.701[a]	.491	.489	1.24536	.491	204.745	1
2	.718[b]	.516	.506	1.22405	.024	3.483	3
3	.728[c]	.530	.516	1.21172	.014	3.136	2
4	.750[d]	.563	.529	1.19497	.033	1.649	9

Model Summary

Model	Change Statistics	
	df2	Sig. F Change
1	212	.000
2	209	.017
3	207	.046
4	198	.104

a. Predictors: (Constant), Party0

b. Predictors: (Constant), Party0, SES0, GenderCC, RaceCC

c. Predictors: (Constant), Party0, SES0, GenderCC, RaceCC, MGRS0, MRN0

d. Predictors: (Constant), Party0, SES0, GenderCC, RaceCC, MGRS0, MRN0, MRN0xMGRS0, MRN0xSES0, MRN0xParty0, MGRS0xSES0, MGRS0xGender, MRN0xRace, MGRS0xRace, MRN0xGender, MGRS0xParty0

ANOVA[a]

Model		Sum of Squares	df	Mean Square	F	Sig.
1	Regression	317.545	1	317.545	204.745	.000[b]
	Residual	328.797	212	1.551		
	Total	646.341	213			
2	Regression	333.199	4	83.300	55.597	.000[c]
	Residual	313.142	209	1.498		
	Total	646.341	213			
3	Regression	342.408	6	57.068	38.867	.000[d]
	Residual	303.933	207	1.468		
	Total	646.341	213			
4	Regression	363.604	15	24.240	16.975	.000[e]
	Residual	282.737	198	1.428		
	Total	646.341	213			

a. Dependent Variable: RepCongressX

b. Predictors: (Constant), Party0

c. Predictors: (Constant), Party0, SES0, GenderCC, RaceCC

d. Predictors: (Constant), Party0, SES0, GenderCC, RaceCC, MGRS0, MRN0

e. Predictors: (Constant), Party0, SES0, GenderCC, RaceCC, MGRS0, MRN0, MRN0xMGRS0, MRN0xSES0, MRN0xParty0, MGRS0xSES0, MGRS0xGender, MRN0xRace, MGRS0xRace, MRN0xGender, MGRS0xParty0

Coefficients[a]

Model		Unstandardized Coefficients B	Unstandardized Coefficients Std. Error	Standardized Coefficients Beta	t	Sig.
1	(Constant)	3.385	.085		39.767	.000
	Party0	.884	.062	.701	14.309	.000
2	(Constant)	3.228	.097		33.352	.000
	Party0	.824	.065	.654	12.632	.000
	GenderCC	.028	.087	.016	.320	.749
	RaceCC	.321	.099	.161	3.223	.001
	SES0	-.022	.101	-.011	-.220	.826
3	(Constant)	3.218	.096		33.496	.000
	Party0	.758	.071	.601	10.683	.000
	GenderCC	-.036	.104	-.021	-.344	.731
	RaceCC	.338	.099	.170	3.423	.001
	SES0	-.041	.100	-.020	-.412	.680
	MGRS0	.063	.144	.024	.438	.662
	MRN0	.266	.124	.134	2.148	.033
4	(Constant)	3.165	.130		24.379	.000
	Party0	.697	.074	.553	9.465	.000
	GenderCC	-.012	.106	-.007	-.115	.908
	RaceCC	.363	.101	.183	3.609	.000
	SES0	-.014	.102	-.007	-.143	.887
	MGRS0	.238	.179	.090	1.329	.185
	MRN0	.171	.137	.086	1.251	.213
	MRN0xRace	.213	.113	.107	1.883	.061
	MRN0xSES0	-.105	.108	-.049	-.978	.329
	MRN0xGender	.072	.140	.032	.512	.609
	MRN0xParty0	.026	.080	.018	.328	.744
	MRN0xMGRS0	-.123	.193	-.046	-.635	.526
	MGRS0xGender	-.016	.174	-.006	-.091	.928
	MGRS0xRace	-.332	.164	-.126	-2.031	.044
	MGRS0xSES0	-.061	.167	-.019	-.364	.716
	MGRS0xParty0	.299	.131	.166	2.280	.024

Coefficients[a]

Model		Correlations Zero-order	Partial	Part
1	(Constant)			
	Party0	.701	.701	.701
2	(Constant)			
	Party0	.701	.658	.608
	GenderCC	.192	.022	.015
	RaceCC	.337	.218	.155
	SES0	.036	-.015	-.011
3	(Constant)			
	Party0	.701	.596	.509
	GenderCC	.192	-.024	-.016
	RaceCC	.337	.231	.163
	SES0	.036	-.029	-.020
	MGRS0	-.002	.030	.021
	MRN0	.416	.148	.102
4	(Constant)			
	Party0	.701	.558	.445
	GenderCC	.192	-.008	-.005
	RaceCC	.337	.248	.170
	SES0	.036	-.010	-.007
	MGRS0	-.002	.094	.062
	MRN0	.416	.089	.059
	MRN0xRace	.319	.133	.089
	MRN0xSES0	-.171	-.069	-.046
	MRN0xGender	.140	.036	.024
	MRN0xParty0	.182	.023	.015
	MRN0xMGRS0	.022	-.045	-.030
	MGRS0xGender	.113	-.006	-.004
	MGRS0xRace	.029	-.143	-.095
	MGRS0xSES0	-.054	-.026	-.017
	MGRS0xParty0	.201	.160	.107

a. Dependent Variable: RepCongressX

Excluded Variables[a]

Model		Beta In	t	Sig.	Partial Correlation	Collinearity Statistics Tolerance
1	GenderCC	.011[b]	.219	.827	.015	.933
	RaceCC	.160[b]	3.224	.001	.217	.928
	SES0	-.005[b]	-.110	.912	-.008	.996
	MGRS0	.042[b]	.859	.391	.059	.996
	MRN0	.116[b]	2.122	.035	.145	.785
	MRN0xRace	.109[b]	2.135	.034	.145	.901
	MRN0xSES0	-.086[b]	-1.741	.083	-.119	.985
	MRN0xGender	.028[b]	.572	.568	.039	.974
	MRN0xParty0	.066[b]	1.336	.183	.092	.972
	MRN0xMGRS0	.035[b]	.708	.479	.049	1.000
	MGRS0xGender	.044[b]	.903	.368	.062	.990
	MGRS0xRace	-.022[b]	-.440	.660	-.030	.995
	MGRS0xSES0	-.023[b]	-.478	.633	-.033	.998
	MGRS0xParty0	.089[b]	1.803	.073	.123	.974
2	MGRS0	.066[c]	1.277	.203	.088	.869
	MRN0	.144[c]	2.471	.014	.169	.669
	MRN0xRace	.131[c]	2.599	.010	.177	.881
	MRN0xSES0	-.082[c]	-1.694	.092	-.117	.981
	MRN0xGender	.051[c]	1.038	.300	.072	.954
	MRN0xParty0	.056[c]	1.124	.262	.078	.939
	MRN0xMGRS0	.032[c]	.658	.511	.046	.971
	MGRS0xGender	.044[c]	.906	.366	.063	.981
	MGRS0xRace	-.027[c]	-.559	.577	-.039	.966
	MGRS0xSES0	-.036[c]	-.742	.459	-.051	.966
	MGRS0xParty0	.077[c]	1.584	.115	.109	.963
3	MRN0xRace	.102[d]	1.905	.058	.132	.787
	MRN0xSES0	-.060[d]	-1.225	.222	-.085	.940
	MRN0xGender	.029[d]	.588	.557	.041	.919
	MRN0xParty0	.076[d]	1.529	.128	.106	.911
	MRN0xMGRS0	.039[d]	.813	.417	.057	.965
	MGRS0xGender	.037[d]	.759	.449	.053	.944

Excluded Variables[a]

Model		Beta In	t	Sig.	Partial Correlation	Collinearity Statistics Tolerance
	MGRS0xRace	-.069[d]	-1.219	.224	-.085	.708
	MGRS0xSES0	-.011[d]	-.216	.829	-.015	.887
	MGRS0xParty0	.095[d]	1.953	.052	.135	.946

a. Dependent Variable: RepCongressX

b. Predictors in the Model: (Constant), Party0

c. Predictors in the Model: (Constant), Party0, SES0, GenderCC, RaceCC

d. Predictors in the Model: (Constant), Party0, SES0, GenderCC, RaceCC, MGRS0, MRN0

```
REGRESSION
  /MISSING LISTWISE
  /STATISTICS COEFF OUTS R ANOVA CHANGE ZPP
  /CRITERIA=PIN(.05) POUT(.10)
  /NOORIGIN
  /DEPENDENT DemCongressX
  /METHOD=ENTER Party0
  /METHOD=ENTER GenderCC RaceCC SES0
  /METHOD=ENTER MGRS0 MRN0
  /METHOD=ENTER MRN0xRace MRN0xSES0 MRN0xGender MRN0xParty0 MRN0xMGRS0 MGRS0xGender MGRS0
xRace MGRS0xSES0 MGRS0xParty0.
```

Regression

Notes

Output Created		15-DEC-2021 13:07:58
Comments		
Input	Data	C:\Users\njs5478\Dropbox\HM and COVID\0. Revise and Resubmit\2. R and R Data\Study 1b\Study1b_Data.sav
	Active Dataset	DataSet1
	Filter	<none>
	Weight	<none>
	Split File	<none>
	N of Rows in Working Data File	241
Missing Value Handling	Definition of Missing	User-defined missing values are treated as missing.
	Cases Used	Statistics are based on cases with no missing values for any variable used.
Syntax		REGRESSION<br>/MISSING LISTWISE<br>/STATISTICS COEFF OUTS R ANOVA CHANGE ZPP<br>/CRITERIA=PIN(.05) POUT(.10)<br>/NOORIGIN<br>/DEPENDENT DemCongressX<br>/METHOD=ENTER Party0<br>/METHOD=ENTER GenderCC RaceCC SES0<br>/METHOD=ENTER MGRS0 MRN0<br>/METHOD=ENTER MRN0xRace MRN0xSES0 MRN0xGender MRN0xParty0 MRN0xMGRS0 MGRS0xGender MGRS0xRace MGRS0xSES0 MGRS0xParty0.

Notes

Resources	Processor Time	00:00:00.02
	Elapsed Time	00:00:00.01
	Memory Required	52240 bytes
	Additional Memory Required for Residual Plots	0 bytes

Variables Entered/Removed[a]

Model	Variables Entered	Variables Removed	Method
1	Party0[b]	.	Enter
2	SES0, GenderCC, RaceCC[b]	.	Enter
3	MGRS0, MRN0[b]	.	Enter
4	MRN0xMGRS0, MRN0xSES0, MRN0xParty0, MGRS0xSES0, MGRS0xGender, MRN0xRace, MGRS0xRace, MRN0xGender, MGRS0xParty...[b]	.	Enter

a. Dependent Variable: DemCongressX

b. All requested variables entered.

Model Summary

Model	R	R Square	Adjusted R Square	Std. Error of the Estimate	Change Statistics		
					R Square Change	F Change	df1
1	.611[a]	.373	.370	1.26386	.373	125.085	1
2	.614[b]	.377	.365	1.26933	.004	.398	3
3	.620[c]	.384	.366	1.26833	.007	1.163	2
4	.644[d]	.415	.371	1.26364	.031	1.169	9

Model Summary

Model	Change Statistics	
	df2	Sig. F Change
1	210	.000
2	207	.755
3	205	.315
4	196	.317

a. Predictors: (Constant), Party0

b. Predictors: (Constant), Party0, SES0, GenderCC, RaceCC

c. Predictors: (Constant), Party0, SES0, GenderCC, RaceCC, MGRS0, MRN0

d. Predictors: (Constant), Party0, SES0, GenderCC, RaceCC, MGRS0, MRN0, MRN0xMGRS0, MRN0xSES0, MRN0xParty0, MGRS0xSES0, MGRS0xGender, MRN0xRace, MGRS0xRace, MRN0xGender, MGRS0xParty0

ANOVA[a]

Model		Sum of Squares	df	Mean Square	F	Sig.
1	Regression	199.804	1	199.804	125.085	.000[b]
	Residual	335.442	210	1.597		
	Total	535.245	211			
2	Regression	201.727	4	50.432	31.301	.000[c]
	Residual	333.518	207	1.611		
	Total	535.245	211			
3	Regression	205.468	6	34.245	21.288	.000[d]
	Residual	329.777	205	1.609		
	Total	535.245	211			
4	Regression	222.273	15	14.818	9.280	.000[e]
	Residual	312.972	196	1.597		
	Total	535.245	211			

a. Dependent Variable: DemCongressX

b. Predictors: (Constant), Party0

c. Predictors: (Constant), Party0, SES0, GenderCC, RaceCC

d. Predictors: (Constant), Party0, SES0, GenderCC, RaceCC, MGRS0, MRN0

e. Predictors: (Constant), Party0, SES0, GenderCC, RaceCC, MGRS0, MRN0, MRN0xMGRS0, MRN0xSES0, MRN0xParty0, MGRS0xSES0, MGRS0xGender, MRN0xRace, MGRS0xRace, MRN0xGender, MGRS0xParty0

Coefficients[a]

Model		Unstandardized Coefficients B	Std. Error	Standardized Coefficients Beta	t	Sig.
1	(Constant)	4.244	.087		48.891	.000
	Party0	-.703	.063	-.611	-11.184	.000
2	(Constant)	4.214	.100		41.939	.000
	Party0	-.700	.068	-.608	-10.270	.000
	GenderCC	-.078	.091	-.049	-.866	.387
	RaceCC	.064	.103	.035	.616	.539
	SES0	.015	.104	.008	.147	.884
3	(Constant)	4.218	.101		41.898	.000
	Party0	-.655	.074	-.569	-8.808	.000
	GenderCC	-.013	.110	-.008	-.122	.903
	RaceCC	.057	.104	.031	.545	.587
	SES0	.031	.105	.016	.298	.766
	MGRS0	.027	.151	.011	.176	.861
	MRN0	-.194	.131	-.106	-1.478	.141
4	(Constant)	4.411	.138		32.004	.000
	Party0	-.636	.078	-.552	-8.162	.000
	GenderCC	-.041	.113	-.026	-.366	.715
	RaceCC	.004	.107	.002	.042	.966
	SES0	.037	.108	.020	.345	.730
	MGRS0	.053	.190	.022	.279	.781
	MRN0	-.122	.145	-.067	-.843	.400
	MRN0xRace	-.026	.120	-.014	-.214	.831
	MRN0xSES0	.036	.114	.018	.316	.752
	MRN0xGender	-.393	.150	-.192	-2.614	.010
	MRN0xParty0	.051	.086	.039	.597	.551
	MRN0xMGRS0	-.026	.205	-.011	-.126	.900
	MGRS0xGender	.166	.184	.065	.900	.369
	MGRS0xRace	.087	.173	.036	.500	.617
	MGRS0xSES0	.065	.177	.023	.370	.712
	MGRS0xParty0	-.012	.139	-.007	-.085	.932

Coefficients[a]

Model		Correlations		
		Zero-order	Partial	Part
1	(Constant)			
	Party0	-.611	-.611	-.611
2	(Constant)			
	Party0	-.611	-.581	-.563
	GenderCC	-.209	-.060	-.048
	RaceCC	-.132	.043	.034
	SES0	-.026	.010	.008
3	(Constant)			
	Party0	-.611	-.524	-.483
	GenderCC	-.209	-.009	-.007
	RaceCC	-.132	.038	.030
	SES0	-.026	.021	.016
	MGRS0	.035	.012	.010
	MRN0	-.365	-.103	-.081
4	(Constant)			
	Party0	-.611	-.504	-.446
	GenderCC	-.209	-.026	-.020
	RaceCC	-.132	.003	.002
	SES0	-.026	.025	.019
	MGRS0	.035	.020	.015
	MRN0	-.365	-.060	-.046
	MRN0xRace	-.240	-.015	-.012
	MRN0xSES0	.122	.023	.017
	MRN0xGender	-.263	-.184	-.143
	MRN0xParty0	-.102	.043	.033
	MRN0xMGRS0	.076	-.009	-.007
	MGRS0xGender	-.082	.064	.049
	MGRS0xRace	-.005	.036	.027
	MGRS0xSES0	.085	.026	.020
	MGRS0xParty0	-.024	-.006	-.005

a. Dependent Variable: DemCongressX

Excluded Variables[a]

Model		Beta In	t	Sig.	Partial Correlation	Collinearity Statistics Tolerance
1	GenderCC	-.050[b]	-.891	.374	-.061	.929
	RaceCC	.037[b]	.655	.513	.045	.926
	SES0	.008[b]	.148	.882	.010	.997
	MGRS0	-.006[b]	-.104	.917	-.007	.995
	MRN0	-.108[b]	-1.763	.079	-.121	.790
	MRN0xRace	-.057[b]	-.995	.321	-.069	.905
	MRN0xSES0	.049[b]	.881	.379	.061	.985
	MRN0xGender	-.176[b]	-3.262	.001	-.220	.978
	MRN0xParty0	.008[b]	.145	.885	.010	.967
	MRN0xMGRS0	.069[b]	1.268	.206	.087	1.000
	MGRS0xGender	-.023[b]	-.412	.681	-.028	.991
	MGRS0xRace	.037[b]	.678	.499	.047	.995
	MGRS0xSES0	.059[b]	1.087	.278	.075	.998
	MGRS0xParty0	.081[b]	1.468	.143	.101	.972
2	MGRS0	-.022[c]	-.375	.708	-.026	.870
	MRN0	-.102[c]	-1.518	.130	-.105	.668
	MRN0xRace	-.050[c]	-.850	.397	-.059	.886
	MRN0xSES0	.052[c]	.943	.347	.066	.982
	MRN0xGender	-.177[c]	-3.236	.001	-.220	.961
	MRN0xParty0	.012[c]	.208	.836	.014	.939
	MRN0xMGRS0	.078[c]	1.401	.163	.097	.973
	MGRS0xGender	-.023[c]	-.420	.675	-.029	.982
	MGRS0xRace	.029[c]	.512	.609	.036	.968
	MGRS0xSES0	.066[c]	1.187	.237	.082	.967
	MGRS0xParty0	.082[c]	1.475	.142	.102	.963
3	MRN0xRace	-.024[d]	-.392	.695	-.027	.798
	MRN0xSES0	.038[d]	.678	.498	.047	.942
	MRN0xGender	-.169[d]	-3.043	.003	-.208	.932
	MRN0xParty0	-.001[d]	-.022	.982	-.002	.915
	MRN0xMGRS0	.073[d]	1.310	.192	.091	.968
	MGRS0xGender	-.014[d]	-.248	.804	-.017	.944

Excluded Variables[a]

Model		Beta In	t	Sig.	Partial Correlation	Collinearity Statistics Tolerance
	MGRS0xRace	.044[d]	.672	.502	.047	.708
	MGRS0xSES0	.058[d]	.993	.322	.069	.887
	MGRS0xParty0	.073[d]	1.303	.194	.091	.948

a. Dependent Variable: DemCongressX

b. Predictors in the Model: (Constant), Party0

c. Predictors in the Model: (Constant), Party0, SES0, GenderCC, RaceCC

d. Predictors in the Model: (Constant), Party0, SES0, GenderCC, RaceCC, MGRS0, MRN0

```
REGRESSION
  /MISSING LISTWISE
  /STATISTICS COEFF OUTS R ANOVA CHANGE ZPP
  /CRITERIA=PIN(.05) POUT(.10)
  /NOORIGIN
  /DEPENDENT StateX
  /METHOD=ENTER Party0
  /METHOD=ENTER GenderCC RaceCC SES0
  /METHOD=ENTER MGRS0 MRN0
  /METHOD=ENTER MRN0xRace MRN0xSES0 MRN0xGender MRN0xParty0 MRN0xMGRS0 MGRS0xGender MGRS0
xRace MGRS0xSES0 MGRS0xParty0.
```

Regression

Notes

Output Created		15-DEC-2021 13:07:58
Comments		
Input	Data	C:\Users\njs5478\Dropbox\HM and COVID\0. Revise and Resubmit\2. R and R Data\Study 1b\Study1b_Data.sav
	Active Dataset	DataSet1
	Filter	<none>
	Weight	<none>
	Split File	<none>
	N of Rows in Working Data File	241
Missing Value Handling	Definition of Missing	User-defined missing values are treated as missing.
	Cases Used	Statistics are based on cases with no missing values for any variable used.
Syntax		REGRESSION<br>/MISSING LISTWISE<br>/STATISTICS COEFF OUTS R ANOVA CHANGE ZPP<br>/CRITERIA=PIN(.05) POUT(.10)<br>/NOORIGIN<br>/DEPENDENT StateX<br>/METHOD=ENTER Party0<br>/METHOD=ENTER GenderCC RaceCC SES0<br>/METHOD=ENTER MGRS0 MRN0<br>/METHOD=ENTER MRN0xRace MRN0xSES0 MRN0xGender MRN0xParty0 MRN0xMGRS0 MGRS0xGender MGRS0xRace MGRS0xSES0 MGRS0xParty0.
Resources	Processor Time	00:00:00.03
	Elapsed Time	00:00:00.03

Notes

	Memory Required	52240 bytes
	Additional Memory Required for Residual Plots	0 bytes

Variables Entered/Removed[a]

Model	Variables Entered	Variables Removed	Method
1	Party0[b]	.	Enter
2	SES0, GenderCC, RaceCC[b]	.	Enter
3	MGRS0, MRN0[b]	.	Enter
4	MRN0xMGRS0, MRN0xSES0, MRN0xParty0, MGRS0xGender, MGRS0xSES0, MRN0xRace, MGRS0xRace, MRN0xGender, MGRS0xParty...[b]	.	Enter

a. Dependent Variable: StateX

b. All requested variables entered.

Model Summary

Model	R	R Square	Adjusted R Square	Std. Error of the Estimate	Change Statistics		
					R Square Change	F Change	df1
1	.335[a]	.112	.108	1.51428	.112	27.808	1
2	.337[b]	.114	.097	1.52359	.001	.107	3
3	.337[c]	.114	.089	1.53055	.000	.015	2
4	.394[d]	.155	.094	1.52665	.041	1.122	9

Model Summary

Model	Change Statistics	
	df2	Sig. F Change
1	220	.000
2	217	.956
3	215	.985
4	206	.348

a. Predictors: (Constant), Party0

b. Predictors: (Constant), Party0, SES0, GenderCC, RaceCC

c. Predictors: (Constant), Party0, SES0, GenderCC, RaceCC, MGRS0, MRN0

d. Predictors: (Constant), Party0, SES0, GenderCC, RaceCC, MGRS0, MRN0, MRN0xMGRS0, MRN0xSES0, MRN0xParty0, MGRS0xGender, MGRS0xSES0, MRN0xRace, MGRS0xRace, MRN0xGender, MGRS0xParty0

ANOVA[a]

Model		Sum of Squares	df	Mean Square	F	Sig.
1	Regression	63.766	1	63.766	27.808	.000[b]
	Residual	504.473	220	2.293		
	Total	568.239	221			
2	Regression	64.511	4	16.128	6.948	.000[c]
	Residual	503.728	217	2.321		
	Total	568.239	221			
3	Regression	64.581	6	10.764	4.595	.000[d]
	Residual	503.657	215	2.343		
	Total	568.239	221			
4	Regression	88.123	15	5.875	2.521	.002[e]
	Residual	480.116	206	2.331		
	Total	568.239	221			

a. Dependent Variable: StateX

b. Predictors: (Constant), Party0

c. Predictors: (Constant), Party0, SES0, GenderCC, RaceCC

d. Predictors: (Constant), Party0, SES0, GenderCC, RaceCC, MGRS0, MRN0

e. Predictors: (Constant), Party0, SES0, GenderCC, RaceCC, MGRS0, MRN0, MRN0xMGRS0, MRN0xSES0, MRN0xParty0, MGRS0xGender, MGRS0xSES0, MRN0xRace, MGRS0xRace, MRN0xGender, MGRS0xParty0

Coefficients[a]

Model		Unstandardized Coefficients B	Std. Error	Standardized Coefficients Beta	t	Sig.
1	(Constant)	4.068	.102		40.019	.000
	Party0	-.386	.073	-.335	-5.273	.000
2	(Constant)	4.102	.120		34.318	.000
	Party0	-.371	.079	-.323	-4.721	.000
	GenderCC	-.017	.105	-.011	-.164	.870
	RaceCC	-.067	.123	-.036	-.547	.585
	SES0	-.002	.125	-.001	-.014	.989
3	(Constant)	4.102	.120		34.103	.000
	Party0	-.365	.088	-.317	-4.156	.000
	GenderCC	-.006	.127	-.004	-.051	.960
	RaceCC	-.069	.124	-.037	-.552	.582
	SES0	.001	.126	.001	.010	.992
	MGRS0	.015	.181	.006	.084	.933
	MRN0	-.026	.154	-.014	-.172	.864
4	(Constant)	4.114	.161		25.622	.000
	Party0	-.378	.092	-.328	-4.117	.000
	GenderCC	-.078	.132	-.049	-.594	.553
	RaceCC	-.095	.128	-.051	-.747	.456
	SES0	.025	.130	.013	.194	.846
	MGRS0	-.099	.235	-.040	-.421	.674
	MRN0	.052	.175	.028	.295	.768
	MRN0xRace	.105	.145	.057	.723	.471
	MRN0xSES0	.076	.136	.038	.556	.579
	MRN0xGender	-.297	.171	-.147	-1.731	.085
	MRN0xParty0	.109	.098	.083	1.115	.266
	MRN0xMGRS0	.174	.248	.070	.703	.483
	MGRS0xGender	-.103	.218	-.039	-.471	.638
	MGRS0xRace	.105	.215	.043	.490	.624
	MGRS0xSES0	-.184	.215	-.062	-.854	.394
	MGRS0xParty0	-.033	.165	-.020	-.197	.844

Coefficients[a]

Model		Correlations Zero-order	Partial	Part
1	(Constant)			
	Party0	-.335	-.335	-.335
2	(Constant)			
	Party0	-.335	-.305	-.302
	GenderCC	-.088	-.011	-.010
	RaceCC	-.122	-.037	-.035
	SES0	-.024	-.001	-.001
3	(Constant)			
	Party0	-.335	-.273	-.267
	GenderCC	-.088	-.003	-.003
	RaceCC	-.122	-.038	-.035
	SES0	-.024	.001	.001
	MGRS0	.026	.006	.005
	MRN0	-.165	-.012	-.011
4	(Constant)			
	Party0	-.335	-.276	-.264
	GenderCC	-.088	-.041	-.038
	RaceCC	-.122	-.052	-.048
	SES0	-.024	.014	.012
	MGRS0	.026	-.029	-.027
	MRN0	-.165	.021	.019
	MRN0xRace	-.076	.050	.046
	MRN0xSES0	.091	.039	.036
	MRN0xGender	-.194	-.120	-.111
	MRN0xParty0	.013	.077	.071
	MRN0xMGRS0	.089	.049	.045
	MGRS0xGender	-.121	-.033	-.030
	MGRS0xRace	.021	.034	.031
	MGRS0xSES0	-.004	-.059	-.055
	MGRS0xParty0	.024	-.014	-.013

a. Dependent Variable: StateX

Excluded Variables[a]

Model		Beta In	t	Sig.	Partial Correlation	Collinearity Statistics Tolerance
1	GenderCC	-.009[b]	-.144	.886	-.010	.944
	RaceCC	-.036[b]	-.544	.587	-.037	.929
	SES0	-.002[b]	-.034	.973	-.002	.996
	MGRS0	.008[b]	.120	.905	.008	.997
	MRN0	-.010[b]	-.143	.886	-.010	.781
	MRN0xRace	.050[b]	.730	.466	.049	.873
	MRN0xSES0	.054[b]	.839	.402	.057	.987
	MRN0xGender	-.144[b]	-2.264	.025	-.151	.975
	MRN0xParty0	.074[b]	1.153	.250	.078	.969
	MRN0xMGRS0	.069[b]	1.080	.281	.073	.996
	MGRS0xGender	-.098[b]	-1.538	.126	-.103	.995
	MGRS0xRace	.042[b]	.664	.507	.045	.996
	MGRS0xSES0	-.015[b]	-.231	.817	-.016	.999
	MGRS0xParty0	.067[b]	1.049	.295	.071	.984
2	MGRS0	.002[c]	.024	.981	.002	.870
	MRN0	-.012[c]	-.152	.879	-.010	.668
	MRN0xRace	.047[c]	.680	.497	.046	.856
	MRN0xSES0	.053[c]	.824	.411	.056	.984
	MRN0xGender	-.152[c]	-2.345	.020	-.158	.958
	MRN0xParty0	.082[c]	1.238	.217	.084	.933
	MRN0xMGRS0	.071[c]	1.099	.273	.075	.971
	MGRS0xGender	-.098[c]	-1.532	.127	-.104	.988
	MGRS0xRace	.043[c]	.656	.512	.045	.965
	MGRS0xSES0	-.012[c]	-.181	.857	-.012	.965
	MGRS0xParty0	.070[c]	1.089	.277	.074	.977
3	MRN0xRace	.059[d]	.792	.429	.054	.739
	MRN0xSES0	.054[d]	.815	.416	.056	.945
	MRN0xGender	-.156[d]	-2.356	.019	-.159	.919
	MRN0xParty0	.082[d]	1.221	.223	.083	.915
	MRN0xMGRS0	.071[d]	1.081	.281	.074	.958
	MGRS0xGender	-.100[d]	-1.524	.129	-.104	.955

Excluded Variables[a]

Model		Beta In	t	Sig.	Partial Correlation	Collinearity Statistics Tolerance
	MGRS0xRace	.058[d]	.744	.458	.051	.675
	MGRS0xSES0	-.014[d]	-.197	.844	-.013	.881
	MGRS0xParty0	.070[d]	1.073	.284	.073	.954

a. Dependent Variable: StateX

b. Predictors in the Model: (Constant), Party0

c. Predictors in the Model: (Constant), Party0, SES0, GenderCC, RaceCC

d. Predictors in the Model: (Constant), Party0, SES0, GenderCC, RaceCC, MGRS0, MRN0

```
REGRESSION
  /MISSING LISTWISE
  /STATISTICS COEFF OUTS R ANOVA CHANGE ZPP
  /CRITERIA=PIN(.05) POUT(.10)
  /NOORIGIN
  /DEPENDENT Risk_Rules
  /METHOD=ENTER Party0
  /METHOD=ENTER GenderCC RaceCC SES0
  /METHOD=ENTER MGRS0 MRN0
  /METHOD=ENTER MRN0xRace MRN0xSES0 MRN0xGender MRN0xParty0 MRN0xMGRS0 MGRS0xGender MGRS0
xRace MGRS0xSES0 MGRS0xParty0.
```

Regression

Notes

Output Created		15-DEC-2021 13:07:58
Comments		
Input	Data	C:\Users\njs5478\Dropbox\HM and COVID\0. Revise and Resubmit\2. R and R Data\Study 1b\Study1b_Data.sav
	Active Dataset	DataSet1
	Filter	<none>
	Weight	<none>
	Split File	<none>
	N of Rows in Working Data File	241
Missing Value Handling	Definition of Missing	User-defined missing values are treated as missing.
	Cases Used	Statistics are based on cases with no missing values for any variable used.
Syntax		REGRESSION<br>/MISSING LISTWISE<br>/STATISTICS COEFF OUTS R ANOVA CHANGE ZPP<br>/CRITERIA=PIN(.05) POUT(.10)<br>/NOORIGIN<br>/DEPENDENT Risk_Rules<br>/METHOD=ENTER Party0<br>/METHOD=ENTER GenderCC RaceCC SES0<br>/METHOD=ENTER MGRS0 MRN0<br>/METHOD=ENTER MRN0xRace MRN0xSES0 MRN0xGender MRN0xParty0 MRN0xMGRS0 MGRS0xGender MGRS0xRace MGRS0xSES0 MGRS0xParty0.

Notes

Resources	Processor Time	00:00:00.02
	Elapsed Time	00:00:00.03
	Memory Required	52240 bytes
	Additional Memory Required for Residual Plots	0 bytes

Variables Entered/Removed[a]

Model	Variables Entered	Variables Removed	Method
1	Party0[b]	.	Enter
2	SES0, GenderCC, RaceCC[b]	.	Enter
3	MGRS0, MRN0[b]	.	Enter
4	MRN0xMGRS0, MRN0xSES0, MRN0xParty0, MGRS0xSES0, MGRS0xGender, MGRS0xRace, MRN0xRace, MRN0xGender, ...[b]	.	Enter

a. Dependent Variable: Risk_Rules

b. All requested variables entered.

Model Summary

Model	R	R Square	Adjusted R Square	Std. Error of the Estimate	Change Statistics		
					R Square Change	F Change	df1
1	.423[a]	.179	.176	.90957	.179	51.349	1
2	.443[b]	.196	.182	.90615	.017	1.593	3
3	.542[c]	.294	.276	.85262	.098	16.023	2
4	.569[d]	.324	.278	.85148	.029	1.068	9

Model Summary

Model	Change Statistics	
	df2	Sig. F Change
1	235	.000
2	232	.192
3	230	.000
4	221	.387

a. Predictors: (Constant), Party0

b. Predictors: (Constant), Party0, SES0, GenderCC, RaceCC

c. Predictors: (Constant), Party0, SES0, GenderCC, RaceCC, MGRS0, MRN0

d. Predictors: (Constant), Party0, SES0, GenderCC, RaceCC, MGRS0, MRN0, MRN0xMGRS0, MRN0xSES0, MRN0xParty0, MGRS0xSES0, MGRS0xGender, MGRS0xRace, MRN0xRace, MRN0xGender, MGRS0xParty0

ANOVA[a]

Model		Sum of Squares	df	Mean Square	F	Sig.
1	Regression	42.483	1	42.483	51.349	.000[b]
	Residual	194.421	235	.827		
	Total	236.903	236			
2	Regression	46.408	4	11.602	14.130	.000[c]
	Residual	190.496	232	.821		
	Total	236.903	236			
3	Regression	69.704	6	11.617	15.981	.000[d]
	Residual	167.200	230	.727		
	Total	236.903	236			
4	Regression	76.674	15	5.112	7.050	.000[e]
	Residual	160.230	221	.725		
	Total	236.903	236			

a. Dependent Variable: Risk_Rules

b. Predictors: (Constant), Party0

c. Predictors: (Constant), Party0, SES0, GenderCC, RaceCC

d. Predictors: (Constant), Party0, SES0, GenderCC, RaceCC, MGRS0, MRN0

e. Predictors: (Constant), Party0, SES0, GenderCC, RaceCC, MGRS0, MRN0, MRN0xMGRS0, MRN0xSES0, MRN0xParty0, MGRS0xSES0, MGRS0xGender, MGRS0xRace, MRN0xRace, MRN0xGender, MGRS0xParty0

Coefficients[a]

Model		Unstandardized Coefficients B	Std. Error	Standardized Coefficients Beta	t	Sig.
1	(Constant)	3.302	.059		55.886	.000
	Party0	.305	.042	.423	7.166	.000
2	(Constant)	3.272	.068		47.784	.000
	Party0	.273	.046	.380	6.007	.000
	GenderCC	.122	.061	.122	2.015	.045
	RaceCC	.061	.071	.053	.864	.389
	SES0	-.030	.073	-.024	-.411	.681
3	(Constant)	3.257	.065		50.468	.000
	Party0	.158	.048	.219	3.321	.001
	GenderCC	-.018	.068	-.018	-.259	.796
	RaceCC	.095	.067	.083	1.419	.157
	SES0	-.075	.069	-.061	-1.084	.280
	MGRS0	-.059	.098	-.038	-.601	.548
	MRN0	.457	.083	.396	5.509	.000
4	(Constant)	3.313	.086		38.516	.000
	Party0	.137	.049	.191	2.789	.006
	GenderCC	.031	.071	.031	.442	.659
	RaceCC	.106	.069	.092	1.529	.128
	SES0	-.088	.071	-.071	-1.237	.217
	MGRS0	.043	.123	.028	.348	.728
	MRN0	.354	.093	.306	3.787	.000
	MRN0xRace	.147	.079	.127	1.870	.063
	MRN0xSES0	-.053	.075	-.042	-.707	.480
	MRN0xGender	-.048	.092	-.038	-.528	.598
	MRN0xParty0	.005	.053	.006	.089	.929
	MRN0xMGRS0	-.188	.135	-.121	-1.394	.165
	MGRS0xGender	.181	.118	.109	1.525	.129
	MGRS0xRace	-.117	.115	-.075	-1.020	.309
	MGRS0xSES0	-.052	.118	-.027	-.438	.662
	MGRS0xParty0	.059	.090	.056	.657	.512

Coefficients[a]

Model		Correlations Zero-order	Partial	Part
1	(Constant)			
	Party0	.423	.423	.423
2	(Constant)			
	Party0	.423	.367	.354
	GenderCC	.214	.131	.119
	RaceCC	.165	.057	.051
	SES0	.000	-.027	-.024
3	(Constant)			
	Party0	.423	.214	.184
	GenderCC	.214	-.017	-.014
	RaceCC	.165	.093	.079
	SES0	.000	-.071	-.060
	MGRS0	-.004	-.040	-.033
	MRN0	.483	.341	.305
4	(Constant)			
	Party0	.423	.184	.154
	GenderCC	.214	.030	.024
	RaceCC	.165	.102	.085
	SES0	.000	-.083	-.068
	MGRS0	-.004	.023	.019
	MRN0	.483	.247	.209
	MRN0xRace	.340	.125	.103
	MRN0xSES0	-.159	-.048	-.039
	MRN0xGender	.129	-.036	-.029
	MRN0xParty0	.050	.006	.005
	MRN0xMGRS0	-.080	-.093	-.077
	MGRS0xGender	.137	.102	.084
	MGRS0xRace	-.012	-.068	-.056
	MGRS0xSES0	-.068	-.029	-.024
	MGRS0xParty0	.009	.044	.036

a. Dependent Variable: Risk_Rules

Excluded Variables[a]

Model		Beta In	t	Sig.	Partial Correlation	Collinearity Statistics Tolerance
1	GenderCC	.119[b]	1.975	.049	.128	.943
	RaceCC	.048[b]	.782	.435	.051	.918
	SES0	-.018[b]	-.298	.766	-.019	.998
	MGRS0	.025[b]	.423	.673	.028	.995
	MRN0	.365[b]	5.836	.000	.356	.783
	MRN0xRace	.223[b]	3.640	.000	.232	.887
	MRN0xSES0	-.113[b]	-1.914	.057	-.124	.988
	MRN0xGender	.081[b]	1.363	.174	.089	.987
	MRN0xParty0	-.013[b]	-.224	.823	-.015	.978
	MRN0xMGRS0	-.066[b]	-1.114	.267	-.073	.999
	MGRS0xGender	.101[b]	1.703	.090	.111	.992
	MGRS0xRace	-.035[b]	-.585	.559	-.038	.997
	MGRS0xSES0	-.057[b]	-.957	.340	-.062	.999
	MGRS0xParty0	-.043[b]	-.727	.468	-.047	.985
2	MGRS0	.077[c]	1.228	.221	.081	.871
	MRN0	.381[c]	5.637	.000	.348	.668
	MRN0xRace	.227[c]	3.684	.000	.236	.866
	MRN0xSES0	-.119[c]	-2.018	.045	-.132	.984
	MRN0xGender	.094[c]	1.582	.115	.104	.968
	MRN0xParty0	-.030[c]	-.500	.618	-.033	.949
	MRN0xMGRS0	-.084[c]	-1.410	.160	-.092	.972
	MGRS0xGender	.104[c]	1.765	.079	.115	.984
	MGRS0xRace	-.020[c]	-.329	.742	-.022	.975
	MGRS0xSES0	-.077[c]	-1.281	.201	-.084	.969
	MGRS0xParty0	-.054[c]	-.906	.366	-.059	.977
3	MRN0xRace	.121[d]	1.909	.057	.125	.753
	MRN0xSES0	-.067[d]	-1.175	.241	-.077	.946
	MRN0xGender	.045[d]	.782	.435	.052	.938
	MRN0xParty0	.017[d]	.301	.764	.020	.928
	MRN0xMGRS0	-.057[d]	-1.003	.317	-.066	.964
	MGRS0xGender	.074[d]	1.304	.193	.086	.945

Excluded Variables[a]

Model		Beta In	t	Sig.	Partial Correlation	Collinearity Statistics Tolerance
	MGRS0xRace	-.048[d]	-.723	.470	-.048	.712
	MGRS0xSES0	-.030[d]	-.508	.612	-.034	.892
	MGRS0xParty0	-.015[d]	-.266	.790	-.018	.960

a. Dependent Variable: Risk_Rules

b. Predictors in the Model: (Constant), Party0

c. Predictors in the Model: (Constant), Party0, SES0, GenderCC, RaceCC

d. Predictors in the Model: (Constant), Party0, SES0, GenderCC, RaceCC, MGRS0, MRN0

```
REGRESSION
  /MISSING LISTWISE
  /STATISTICS COEFF OUTS R ANOVA CHANGE ZPP
  /CRITERIA=PIN(.05) POUT(.10)
  /NOORIGIN
  /DEPENDENT Risk_Help
  /METHOD=ENTER Party0
  /METHOD=ENTER GenderCC RaceCC SES0
  /METHOD=ENTER MGRS0 MRN0
  /METHOD=ENTER MRN0xRace MRN0xSES0 MRN0xGender MRN0xParty0 MRN0xMGRS0 MGRS0xGender MGRS0
xRace MGRS0xSES0 MGRS0xParty0.
```

Regression

Notes

Output Created		15-DEC-2021 13:07:58
Comments		
Input	Data	C:\Users\njs5478\Dropbox\HM and COVID\0. Revise and Resubmit\2. R and R Data\Study 1b\Study1b_Data.sav
	Active Dataset	DataSet1
	Filter	<none>
	Weight	<none>
	Split File	<none>
	N of Rows in Working Data File	241
Missing Value Handling	Definition of Missing	User-defined missing values are treated as missing.
	Cases Used	Statistics are based on cases with no missing values for any variable used.
Syntax		REGRESSION<br>/MISSING LISTWISE<br>/STATISTICS COEFF OUTS R ANOVA CHANGE ZPP<br>/CRITERIA=PIN(.05) POUT(.10)<br>/NOORIGIN<br>/DEPENDENT Risk_Help<br>/METHOD=ENTER Party0<br>/METHOD=ENTER GenderCC RaceCC SES0<br>/METHOD=ENTER MGRS0 MRN0<br>/METHOD=ENTER MRN0xRace MRN0xSES0 MRN0xGender MRN0xParty0 MRN0xMGRS0 MGRS0xGender MGRS0xRace MGRS0xSES0 MGRS0xParty0.

Notes

Resources	Processor Time	00:00:00.03
	Elapsed Time	00:00:00.03
	Memory Required	52240 bytes
	Additional Memory Required for Residual Plots	0 bytes

Variables Entered/Removed[a]

Model	Variables Entered	Variables Removed	Method
1	Party0[b]	.	Enter
2	SES0, GenderCC, RaceCC[b]	.	Enter
3	MGRS0, MRN0[b]	.	Enter
4	MRN0xMGRS0, MRN0xSES0, MRN0xParty0, MGRS0xSES0, MGRS0xGender, MGRS0xRace, MRN0xRace, MRN0xGender, ...[b]	.	Enter

a. Dependent Variable: Risk_Help

b. All requested variables entered.

Model Summary

Model	R	R Square	Adjusted R Square	Std. Error of the Estimate	Change Statistics		
					R Square Change	F Change	df1
1	.168[a]	.028	.024	1.49077	.028	6.817	1
2	.280[b]	.079	.063	1.46100	.050	4.225	3
3	.288[c]	.083	.059	1.46396	.004	.531	2
4	.334[d]	.112	.051	1.46967	.029	.802	9

Model Summary

Model	Change Statistics	
	df2	Sig. F Change
1	235	.010
2	232	.006
3	230	.589
4	221	.615

a. Predictors: (Constant), Party0

b. Predictors: (Constant), Party0, SES0, GenderCC, RaceCC

c. Predictors: (Constant), Party0, SES0, GenderCC, RaceCC, MGRS0, MRN0

d. Predictors: (Constant), Party0, SES0, GenderCC, RaceCC, MGRS0, MRN0, MRN0xMGRS0, MRN0xSES0, MRN0xParty0, MGRS0xSES0, MGRS0xGender, MGRS0xRace, MRN0xRace, MRN0xGender, MGRS0xParty0

ANOVA[a]

Model		Sum of Squares	df	Mean Square	F	Sig.
1	Regression	15.149	1	15.149	6.817	.010[b]
	Residual	522.264	235	2.222		
	Total	537.413	236			
2	Regression	42.207	4	10.552	4.943	.001[c]
	Residual	495.206	232	2.135		
	Total	537.413	236			
3	Regression	44.482	6	7.414	3.459	.003[d]
	Residual	492.931	230	2.143		
	Total	537.413	236			
4	Regression	60.068	15	4.005	1.854	.029[e]
	Residual	477.345	221	2.160		
	Total	537.413	236			

a. Dependent Variable: Risk_Help

b. Predictors: (Constant), Party0

c. Predictors: (Constant), Party0, SES0, GenderCC, RaceCC

d. Predictors: (Constant), Party0, SES0, GenderCC, RaceCC, MGRS0, MRN0

e. Predictors: (Constant), Party0, SES0, GenderCC, RaceCC, MGRS0, MRN0, MRN0xMGRS0, MRN0xSES0, MRN0xParty0, MGRS0xSES0, MGRS0xGender, MGRS0xRace, MRN0xRace, MRN0xGender, MGRS0xParty0

Coefficients[a]

Model		Unstandardized Coefficients B	Std. Error	Standardized Coefficients Beta	t	Sig.
1	(Constant)	3.798	.097		39.222	.000
	Party0	-.182	.070	-.168	-2.611	.010
2	(Constant)	3.934	.110		35.634	.000
	Party0	-.104	.073	-.096	-1.421	.157
	GenderCC	-.122	.098	-.081	-1.246	.214
	RaceCC	-.282	.114	-.163	-2.476	.014
	SES0	-.255	.118	-.137	-2.165	.031
3	(Constant)	3.936	.111		35.525	.000
	Party0	-.123	.082	-.114	-1.511	.132
	GenderCC	-.183	.117	-.122	-1.572	.117
	RaceCC	-.286	.115	-.165	-2.489	.014
	SES0	-.270	.119	-.145	-2.263	.025
	MGRS0	-.165	.167	-.071	-.985	.325
	MRN0	.087	.142	.050	.611	.542
4	(Constant)	3.893	.148		26.219	.000
	Party0	-.090	.085	-.083	-1.064	.289
	GenderCC	-.225	.122	-.149	-1.845	.066
	RaceCC	-.296	.119	-.171	-2.482	.014
	SES0	-.293	.123	-.157	-2.386	.018
	MGRS0	-.370	.213	-.158	-1.735	.084
	MRN0	.196	.161	.113	1.217	.225
	MRN0xRace	-.207	.136	-.119	-1.526	.129
	MRN0xSES0	.129	.130	.067	.993	.322
	MRN0xGender	.050	.158	.026	.319	.750
	MRN0xParty0	-.025	.092	-.020	-.275	.784
	MRN0xMGRS0	.187	.233	.079	.801	.424
	MGRS0xGender	-.123	.204	-.049	-.601	.548
	MGRS0xRace	.246	.198	.105	1.241	.216
	MGRS0xSES0	-.179	.203	-.062	-.878	.381
	MGRS0xParty0	-.129	.155	-.081	-.830	.408

Coefficients[a]

Model		Correlations Zero-order	Partial	Part
1	(Constant)			
	Party0	-.168	-.168	-.168
2	(Constant)			
	Party0	-.168	-.093	-.090
	GenderCC	-.117	-.082	-.079
	RaceCC	-.198	-.160	-.156
	SES0	-.151	-.141	-.136
3	(Constant)			
	Party0	-.168	-.099	-.095
	GenderCC	-.117	-.103	-.099
	RaceCC	-.198	-.162	-.157
	SES0	-.151	-.148	-.143
	MGRS0	.011	-.065	-.062
	MRN0	-.087	.040	.039
4	(Constant)			
	Party0	-.168	-.071	-.067
	GenderCC	-.117	-.123	-.117
	RaceCC	-.198	-.165	-.157
	SES0	-.151	-.158	-.151
	MGRS0	.011	-.116	-.110
	MRN0	-.087	.082	.077
	MRN0xRace	-.122	-.102	-.097
	MRN0xSES0	.087	.067	.063
	MRN0xGender	-.011	.021	.020
	MRN0xParty0	-.076	-.018	-.017
	MRN0xMGRS0	.008	.054	.051
	MGRS0xGender	-.062	-.040	-.038
	MGRS0xRace	.019	.083	.079
	MGRS0xSES0	-.042	-.059	-.056
	MGRS0xParty0	-.056	-.056	-.053

a. Dependent Variable: Risk_Help

Excluded Variables[a]

Model		Beta In	t	Sig.	Partial Correlation	Collinearity Statistics Tolerance
1	GenderCC	-.082[b]	-1.238	.217	-.081	.943
	RaceCC	-.163[b]	-2.461	.015	-.159	.918
	SES0	-.144[b]	-2.254	.025	-.146	.998
	MGRS0	-.001[b]	-.014	.989	-.001	.995
	MRN0	-.012[b]	-.162	.872	-.011	.783
	MRN0xRace	-.074[b]	-1.080	.281	-.070	.887
	MRN0xSES0	.069[b]	1.065	.288	.069	.988
	MRN0xGender	.008[b]	.125	.901	.008	.987
	MRN0xParty0	-.052[b]	-.802	.423	-.052	.978
	MRN0xMGRS0	.002[b]	.028	.977	.002	.999
	MGRS0xGender	-.047[b]	-.732	.465	-.048	.992
	MGRS0xRace	.028[b]	.431	.667	.028	.997
	MGRS0xSES0	-.046[b]	-.716	.475	-.047	.999
	MGRS0xParty0	-.036[b]	-.556	.579	-.036	.985
2	MGRS0	-.056[c]	-.830	.407	-.055	.871
	MRN0	.023[c]	.301	.764	.020	.668
	MRN0xRace	-.096[c]	-1.415	.158	-.093	.866
	MRN0xSES0	.069[c]	1.084	.279	.071	.984
	MRN0xGender	-.009[c]	-.144	.886	-.009	.968
	MRN0xParty0	-.052[c]	-.804	.422	-.053	.949
	MRN0xMGRS0	-.004[c]	-.070	.944	-.005	.972
	MGRS0xGender	-.033[c]	-.516	.607	-.034	.984
	MGRS0xRace	.024[c]	.369	.713	.024	.975
	MGRS0xSES0	-.047[c]	-.739	.461	-.049	.969
	MGRS0xParty0	-.026[c]	-.414	.680	-.027	.977
3	MRN0xRace	-.120[d]	-1.650	.100	-.108	.753
	MRN0xSES0	.071[d]	1.087	.278	.072	.946
	MRN0xGender	-.009[d]	-.138	.890	-.009	.938
	MRN0xParty0	-.048[d]	-.724	.470	-.048	.928
	MRN0xMGRS0	-.001[d]	-.012	.991	-.001	.964
	MGRS0xGender	-.048[d]	-.732	.465	-.048	.945

Excluded Variables[a]

Model		Beta In	t	Sig.	Partial Correlation	Collinearity Statistics Tolerance
	MGRS0xRace	.074[d]	.982	.327	.065	.712
	MGRS0xSES0	-.063[d]	-.936	.350	-.062	.892
	MGRS0xParty0	-.026[d]	-.407	.685	-.027	.960

a. Dependent Variable: Risk_Help

b. Predictors in the Model: (Constant), Party0

c. Predictors in the Model: (Constant), Party0, SES0, GenderCC, RaceCC

d. Predictors in the Model: (Constant), Party0, SES0, GenderCC, RaceCC, MGRS0, MRN0

```
 *FOR MEN ONLY

USE ALL.
COMPUTE filter_$=(GenderCC=1).
VARIABLE LABELS filter_$ 'GenderCC=1 (FILTER)'.
VALUE LABELS filter_$ 0 'Not Selected' 1 'Selected'.
FORMATS filter_$ (f1.0).
FILTER BY filter_$.
EXECUTE.

REGRESSION
  /MISSING LISTWISE
  /STATISTICS COEFF OUTS R ANOVA CHANGE ZPP
  /CRITERIA=PIN(.05) POUT(.10)
  /NOORIGIN
  /DEPENDENT Concern_Tot
  /METHOD=ENTER Party0
  /METHOD=ENTER RaceCC SES0
  /METHOD=ENTER MGRS0 MRN0
  /METHOD=ENTER MRN0xRace MRN0xSES0 MRN0xParty0 MRN0xMGRS0 MGRS0xRace MGRS0xSES0 MGRS0xPa
rty0.
```

Regression

Notes

Output Created		15-DEC-2021 13:07:58
Comments		
Input	Data	C: \Users\njs5478\Dropbox\HM and COVID\0. Revise and Resubmit\2. R and R Data\Study 1b\Study1b_Data.sav
	Active Dataset	DataSet1
	Filter	GenderCC=1 (FILTER)
	Weight	<none>
	Split File	<none>
	N of Rows in Working Data File	119
Missing Value Handling	Definition of Missing	User-defined missing values are treated as missing.
	Cases Used	Statistics are based on cases with no missing values for any variable used.
Syntax		REGRESSION<br>/MISSING LISTWISE<br>/STATISTICS COEFF OUTS R ANOVA CHANGE ZPP<br>/CRITERIA=PIN(.05) POUT(.10)<br>/NOORIGIN<br>/DEPENDENT Concern_Tot<br>/METHOD=ENTER Party0<br>/METHOD=ENTER RaceCC SES0<br>/METHOD=ENTER MGRS0 MRN0<br>/METHOD=ENTER MRN0xRace MRN0xSES0 MRN0xParty0 MRN0xMGRS0 MGRS0xRace MGRS0xSES0 MGRS0xParty0.
Resources	Processor Time	00:00:00.02
	Elapsed Time	00:00:00.02

Notes

	Memory Required	48560 bytes
	Additional Memory Required for Residual Plots	0 bytes

Variables Entered/Removed[a]

Model	Variables Entered	Variables Removed	Method
1	Party0[b]	.	Enter
2	SES0, RaceCC[b]	.	Enter
3	MGRS0, MRN0[b]	.	Enter
4	MRN0xSES0, MGRS0xRace , MRN0xParty0 , MGRS0xParty 0, MGRS0xSES 0, MRN0xRace, MRN0xMGRS...[b]	.	Enter

a. Dependent Variable: Concern_Tot

b. All requested variables entered.

Model Summary

Model	R	R Square	Adjusted R Square	Std. Error of the Estimate	Change Statistics		
					R Square Change	F Change	df1
1	.536[a]	.287	.281	1.20167	.287	46.704	1
2	.557[b]	.310	.292	1.19264	.023	1.881	2
3	.573[c]	.328	.298	1.18745	.018	1.500	2
4	.622[d]	.387	.317	1.17098	.059	1.453	7

Model Summary

Model	Change Statistics df2	Sig. F Change
1	116	.000
2	114	.157
3	112	.228
4	105	.192

a. Predictors: (Constant), Party0

b. Predictors: (Constant), Party0, SES0, RaceCC

c. Predictors: (Constant), Party0, SES0, RaceCC, MGRS0, MRN0

d. Predictors: (Constant), Party0, SES0, RaceCC, MGRS0, MRN0, MRN0xSES0, MGRS0xRace, MRN0xParty0, MGRS0xParty0, MGRS0xSES0, MRN0xRace, MRN0xMGRS0

ANOVA[a]

Model		Sum of Squares	df	Mean Square	F	Sig.
1	Regression	67.440	1	67.440	46.704	.000[b]
	Residual	167.504	116	1.444		
	Total	234.944	117			
2	Regression	72.792	3	24.264	17.059	.000[c]
	Residual	162.152	114	1.422		
	Total	234.944	117			
3	Regression	77.021	5	15.404	10.925	.000[d]
	Residual	157.923	112	1.410		
	Total	234.944	117			
4	Regression	90.968	12	7.581	5.528	.000[e]
	Residual	143.976	105	1.371		
	Total	234.944	117			

a. Dependent Variable: Concern_Tot

b. Predictors: (Constant), Party0

c. Predictors: (Constant), Party0, SES0, RaceCC

d. Predictors: (Constant), Party0, SES0, RaceCC, MGRS0, MRN0

e. Predictors: (Constant), Party0, SES0, RaceCC, MGRS0, MRN0, MRN0xSES0, MGRS0xRace, MRN0xParty0, MGRS0xParty0, MGRS0xSES0, MRN0xRace, MRN0xMGRS0

Coefficients[a]

Model		Unstandardized Coefficients B	Unstandardized Coefficients Std. Error	Standardized Coefficients Beta	t	Sig.
1	(Constant)	4.183	.114		36.751	.000
	Party0	-.557	.081	-.536	-6.834	.000
2	(Constant)	4.303	.130		33.199	.000
	Party0	-.512	.084	-.493	-6.064	.000
	RaceCC	-.250	.135	-.151	-1.846	.067
	SES0	-.096	.127	-.059	-.753	.453
3	(Constant)	4.401	.155		28.387	.000
	Party0	-.422	.099	-.406	-4.251	.000
	RaceCC	-.300	.138	-.181	-2.177	.032
	SES0	-.066	.127	-.041	-.522	.603
	MGRS0	.034	.216	.013	.157	.876
	MRN0	-.267	.164	-.164	-1.635	.105
4	(Constant)	4.101	.217		18.926	.000
	Party0	-.392	.111	-.378	-3.542	.001
	RaceCC	-.199	.186	-.120	-1.071	.287
	SES0	.036	.188	.022	.191	.849
	MGRS0	-.180	.347	-.072	-.519	.605
	MRN0	.078	.239	.048	.325	.746
	MRN0xRace	-.259	.203	-.170	-1.275	.205
	MRN0xSES0	.119	.158	.080	.757	.451
	MRN0xParty0	.064	.102	.063	.627	.532
	MRN0xMGRS0	.395	.311	.179	1.271	.207
	MGRS0xRace	.019	.317	.008	.059	.953
	MGRS0xSES0	.171	.317	.065	.539	.591
	MGRS0xParty0	-.043	.207	-.026	-.208	.835

Coefficients[a]

Model		Correlations Zero-order	Partial	Part
1	(Constant)			
	Party0	-.536	-.536	-.536
2	(Constant)			
	Party0	-.536	-.494	-.472
	RaceCC	-.287	-.170	-.144
	SES0	-.045	-.070	-.059
3	(Constant)			
	Party0	-.536	-.373	-.329
	RaceCC	-.287	-.202	-.169
	SES0	-.045	-.049	-.040
	MGRS0	-.095	.015	.012
	MRN0	-.353	-.153	-.127
4	(Constant)			
	Party0	-.536	-.327	-.271
	RaceCC	-.287	-.104	-.082
	SES0	-.045	.019	.015
	MGRS0	-.095	-.051	-.040
	MRN0	-.353	.032	.025
	MRN0xRace	-.453	-.123	-.097
	MRN0xSES0	.259	.074	.058
	MRN0xParty0	-.145	.061	.048
	MRN0xMGRS0	.364	.123	.097
	MGRS0xRace	-.070	.006	.004
	MGRS0xSES0	.205	.053	.041
	MGRS0xParty0	.197	-.020	-.016

a. Dependent Variable: Concern_Tot

Excluded Variables[a]

Model		Beta In	t	Sig.	Partial Correlation	Collinearity Statistics Tolerance
1	RaceCC	-.145[b]	-1.791	.076	-.165	.918
	SES0	-.046[b]	-.589	.557	-.055	1.000
	MGRS0	-.036[b]	-.450	.653	-.042	.988
	MRN0	-.121[b]	-1.359	.177	-.126	.765
	MRN0xRace	-.251[b]	-2.883	.005	-.260	.762
	MRN0xSES0	.154[b]	1.945	.054	.178	.957
	MRN0xParty0	.093[b]	1.083	.281	.101	.829
	MRN0xMGRS0	.205[b]	2.520	.013	.229	.885
	MGRS0xRace	-.056[b]	-.710	.479	-.066	.999
	MGRS0xSES0	.125[b]	1.579	.117	.146	.976
	MGRS0xParty0	.085[b]	1.065	.289	.099	.953
2	MGRS0	-.045[c]	-.567	.572	-.053	.981
	MRN0	-.158[c]	-1.732	.086	-.161	.716
	MRN0xRace	-.223[c]	-2.470	.015	-.226	.712
	MRN0xSES0	.181[c]	2.244	.027	.207	.900
	MRN0xParty0	.111[c]	1.256	.212	.117	.770
	MRN0xMGRS0	.208[c]	2.429	.017	.223	.791
	MGRS0xRace	-.107[c]	-1.295	.198	-.121	.888
	MGRS0xSES0	.171[c]	1.813	.073	.168	.670
	MGRS0xParty0	.081[c]	1.007	.316	.094	.930
3	MRN0xRace	-.194[d]	-1.837	.069	-.172	.529
	MRN0xSES0	.167[d]	1.979	.050	.185	.823
	MRN0xParty0	.092[d]	1.024	.308	.097	.737
	MRN0xMGRS0	.258[d]	2.389	.019	.221	.493
	MGRS0xRace	-.125[d]	-1.400	.164	-.132	.741
	MGRS0xSES0	.138[d]	1.399	.165	.132	.611
	MGRS0xParty0	.047[d]	.525	.600	.050	.740

a. Dependent Variable: Concern_Tot

b. Predictors in the Model: (Constant), Party0

c. Predictors in the Model: (Constant), Party0, SES0, RaceCC

d. Predictors in the Model: (Constant), Party0, SES0, RaceCC, MGRS0, MRN0

```
REGRESSION
  /MISSING LISTWISE
  /STATISTICS COEFF OUTS R ANOVA CHANGE ZPP
  /CRITERIA=PIN(.05) POUT(.10)
  /NOORIGIN
  /DEPENDENT Finance_Tot
  /METHOD=ENTER Party0
  /METHOD=ENTER RaceCC SES0
  /METHOD=ENTER MGRS0 MRN0
  /METHOD=ENTER MRN0xRace MRN0xSES0 MRN0xParty0 MRN0xMGRS0 MGRS0xRace MGRS0xSES0 MGRS0xPa
rty0.
```

Regression

Notes

Output Created		15-DEC-2021 13:07:58
Comments		
Input	Data	C:\Users\njs5478\Dropbox\HM and COVID\0. Revise and Resubmit\2. R and R Data\Study 1b\Study1b_Data.sav
	Active Dataset	DataSet1
	Filter	GenderCC=1 (FILTER)
	Weight	<none>
	Split File	<none>
	N of Rows in Working Data File	119
Missing Value Handling	Definition of Missing	User-defined missing values are treated as missing.
	Cases Used	Statistics are based on cases with no missing values for any variable used.

Notes

Syntax		REGRESSION<br>/MISSING LISTWISE<br>/STATISTICS COEFF OUTS R ANOVA CHANGE ZPP<br>/CRITERIA=PIN(.05) POUT(.10)<br>/NOORIGIN<br>/DEPENDENT Finance_Tot<br>/METHOD=ENTER Party0<br>/METHOD=ENTER RaceCC SES0<br>/METHOD=ENTER MGRS0 MRN0<br>/METHOD=ENTER MRN0xRace MRN0xSES0 MRN0xParty0 MRN0xMGRS0 MGRS0xRace MGRS0xSES0 MGRS0xParty0.
Resources	Processor Time	00:00:00.03
	Elapsed Time	00:00:00.05
	Memory Required	48560 bytes
	Additional Memory Required for Residual Plots	0 bytes

Variables Entered/Removed[a]

Model	Variables Entered	Variables Removed	Method
1	Party0[b]	.	Enter
2	SES0, RaceCC[b]	.	Enter
3	MGRS0, MRN0[b]	.	Enter
4	MRN0xSES0, MGRS0xRace, MRN0xParty0, MGRS0xParty0, MGRS0xSES0, MRN0xRace, MRN0xMGRS...[b]	.	Enter

a. Dependent Variable: Finance_Tot

b. All requested variables entered.

Model Summary

Model	R	R Square	Adjusted R Square	Std. Error of the Estimate	Change Statistics		
					R Square Change	F Change	df1
1	.059[a]	.003	-.005	1.49325	.003	.402	1
2	.153[b]	.023	-.002	1.49113	.020	1.165	2
3	.158[c]	.025	-.018	1.50312	.002	.094	2
4	.304[d]	.092	-.012	1.49806	.067	1.108	7

Model Summary

Model	Change Statistics	
	df2	Sig. F Change
1	116	.527
2	114	.316
3	112	.910
4	105	.363

a. Predictors: (Constant), Party0

b. Predictors: (Constant), Party0, SES0, RaceCC

c. Predictors: (Constant), Party0, SES0, RaceCC, MGRS0, MRN0

d. Predictors: (Constant), Party0, SES0, RaceCC, MGRS0, MRN0, MRN0xSES0, MGRS0xRace, MRN0xParty0, MGRS0xParty0, MGRS0xSES0, MRN0xRace, MRN0xMGRS0

ANOVA[a]

Model		Sum of Squares	df	Mean Square	F	Sig.
1	Regression	.896	1	.896	.402	.527[b]
	Residual	258.656	116	2.230		
	Total	259.552	117			
2	Regression	6.075	3	2.025	.911	.438[c]
	Residual	253.477	114	2.223		
	Total	259.552	117			
3	Regression	6.502	5	1.300	.576	.719[d]
	Residual	253.050	112	2.259		
	Total	259.552	117			
4	Regression	23.912	12	1.993	.888	.562[e]
	Residual	235.640	105	2.244		
	Total	259.552	117			

a. Dependent Variable: Finance_Tot

b. Predictors: (Constant), Party0

c. Predictors: (Constant), Party0, SES0, RaceCC

d. Predictors: (Constant), Party0, SES0, RaceCC, MGRS0, MRN0

e. Predictors: (Constant), Party0, SES0, RaceCC, MGRS0, MRN0, MRN0xSES0, MGRS0xRace, MRN0xParty0, MGRS0xParty0, MGRS0xSES0, MRN0xRace, MRN0xMGRS0

Coefficients[a]

Model		Unstandardized Coefficients B	Unstandardized Coefficients Std. Error	Standardized Coefficients Beta	t	Sig.
1	(Constant)	3.693	.141		26.112	.000
	Party0	-.064	.101	-.059	-.634	.527
2	(Constant)	3.749	.162		23.138	.000
	Party0	-.046	.106	-.042	-.434	.665
	RaceCC	-.104	.169	-.060	-.616	.539
	SES0	-.229	.159	-.134	-1.444	.151
3	(Constant)	3.773	.196		19.225	.000
	Party0	-.018	.126	-.017	-.146	.884
	RaceCC	-.120	.174	-.069	-.688	.493
	SES0	-.220	.161	-.129	-1.365	.175
	MGRS0	-.012	.273	-.004	-.043	.966
	MRN0	-.078	.207	-.046	-.376	.708
4	(Constant)	3.634	.277		13.108	.000
	Party0	-.062	.142	-.057	-.436	.664
	RaceCC	.058	.238	.033	.244	.808
	SES0	-.334	.240	-.196	-1.392	.167
	MGRS0	.058	.443	.022	.132	.895
	MRN0	.120	.306	.070	.391	.697
	MRN0xRace	-.348	.260	-.217	-1.337	.184
	MRN0xSES0	.326	.202	.207	1.614	.110
	MRN0xParty0	.151	.130	.142	1.163	.248
	MRN0xMGRS0	-.131	.398	-.057	-.329	.743
	MGRS0xRace	.431	.405	.171	1.063	.290
	MGRS0xSES0	.044	.406	.016	.108	.915
	MGRS0xParty0	-.167	.265	-.094	-.628	.531

Coefficients[a]

Model		Correlations Zero-order	Partial	Part
1	(Constant)			
	Party0	-.059	-.059	-.059
2	(Constant)			
	Party0	-.059	-.041	-.040
	RaceCC	-.061	-.058	-.057
	SES0	-.129	-.134	-.134
3	(Constant)			
	Party0	-.059	-.014	-.014
	RaceCC	-.061	-.065	-.064
	SES0	-.129	-.128	-.127
	MGRS0	-.027	-.004	-.004
	MRN0	-.069	-.035	-.035
4	(Constant)			
	Party0	-.059	-.043	-.041
	RaceCC	-.061	.024	.023
	SES0	-.129	-.135	-.129
	MGRS0	-.027	.013	.012
	MRN0	-.069	.038	.036
	MRN0xRace	-.116	-.129	-.124
	MRN0xSES0	.173	.156	.150
	MRN0xParty0	.079	.113	.108
	MRN0xMGRS0	.072	-.032	-.031
	MGRS0xRace	.033	.103	.099
	MGRS0xSES0	.168	.010	.010
	MGRS0xParty0	.056	-.061	-.058

a. Dependent Variable: Finance_Tot

Excluded Variables[a]

Model		Beta In	t	Sig.	Partial Correlation	Collinearity Statistics Tolerance
1	RaceCC	-.048[b]	-.490	.625	-.046	.918
	SES0	-.129[b]	-1.400	.164	-.129	1.000
	MGRS0	-.021[b]	-.221	.825	-.021	.988
	MRN0	-.053[b]	-.494	.622	-.046	.765
	MRN0xRace	-.114[b]	-1.075	.285	-.100	.762
	MRN0xSES0	.168[b]	1.785	.077	.164	.957
	MRN0xParty0	.124[b]	1.223	.224	.113	.829
	MRN0xMGRS0	.059[b]	.593	.554	.055	.885
	MGRS0xRace	.035[b]	.374	.709	.035	.999
	MGRS0xSES0	.163[b]	1.753	.082	.161	.976
	MGRS0xParty0	.046[b]	.480	.632	.045	.953
2	MGRS0	-.021[c]	-.219	.827	-.021	.981
	MRN0	-.048[c]	-.434	.665	-.041	.716
	MRN0xRace	-.101[c]	-.924	.358	-.087	.712
	MRN0xSES0	.214[c]	2.229	.028	.205	.900
	MRN0xParty0	.108[c]	1.025	.308	.096	.770
	MRN0xMGRS0	.014[c]	.135	.893	.013	.791
	MGRS0xRace	.037[c]	.377	.707	.035	.888
	MGRS0xSES0	.144[c]	1.278	.204	.119	.670
	MGRS0xParty0	.026[c]	.270	.787	.025	.930
3	MRN0xRace	-.106[d]	-.826	.410	-.078	.529
	MRN0xSES0	.223[d]	2.203	.030	.205	.823
	MRN0xParty0	.105[d]	.962	.338	.091	.737
	MRN0xMGRS0	.008[d]	.057	.955	.005	.493
	MGRS0xRace	.049[d]	.452	.652	.043	.741
	MGRS0xSES0	.142[d]	1.194	.235	.113	.611
	MGRS0xParty0	.020[d]	.182	.856	.017	.740

a. Dependent Variable: Finance_Tot

b. Predictors in the Model: (Constant), Party0

c. Predictors in the Model: (Constant), Party0, SES0, RaceCC

d. Predictors in the Model: (Constant), Party0, SES0, RaceCC, MGRS0, MRN0

```
REGRESSION
  /MISSING LISTWISE
  /STATISTICS COEFF OUTS R ANOVA CHANGE ZPP
  /CRITERIA=PIN(.05) POUT(.10)
  /NOORIGIN
  /DEPENDENT Resource_Tot
  /METHOD=ENTER Party0
  /METHOD=ENTER RaceCC SES0
  /METHOD=ENTER MGRS0 MRN0
  /METHOD=ENTER MRN0xRace MRN0xSES0 MRN0xParty0 MRN0xMGRS0 MGRS0xRace MGRS0xSES0 MGRS0xPa
rty0.
```

Regression

Notes

Output Created		15-DEC-2021 13:07:58
Comments		
Input	Data	C: \Users\njs5478\Dropbox\HM and COVID\0. Revise and Resubmit\2. R and R Data\Study 1b\Study1b_Data.sav
	Active Dataset	DataSet1
	Filter	GenderCC=1 (FILTER)
	Weight	<none>
	Split File	<none>
	N of Rows in Working Data File	119
Missing Value Handling	Definition of Missing	User-defined missing values are treated as missing.
	Cases Used	Statistics are based on cases with no missing values for any variable used.

Notes

Syntax		REGRESSION<br>/MISSING LISTWISE<br>/STATISTICS COEFF OUTS R ANOVA CHANGE ZPP<br>/CRITERIA=PIN(.05) POUT(.10)<br>/NOORIGIN<br>/DEPENDENT Resource_Tot<br>/METHOD=ENTER Party0<br>/METHOD=ENTER RaceCC SES0<br>/METHOD=ENTER MGRS0 MRN0<br>/METHOD=ENTER MRN0xRace MRN0xSES0 MRN0xParty0 MRN0xMGRS0 MGRS0xRace MGRS0xSES0 MGRS0xParty0.
Resources	Processor Time	00:00:00.05
	Elapsed Time	00:00:00.03
	Memory Required	48560 bytes
	Additional Memory Required for Residual Plots	0 bytes

Variables Entered/Removed[a]

Model	Variables Entered	Variables Removed	Method
1	Party0[b]	.	Enter
2	SES0, RaceCC[b]	.	Enter
3	MGRS0, MRN0[b]	.	Enter
4	MRN0xSES0, MGRS0xRace, MRN0xParty0, MGRS0xParty0, MGRS0xSES0, MRN0xRace, MRN0xMGRS...[b]	.	Enter

a. Dependent Variable: Resource_Tot

b. All requested variables entered.

Model Summary

Model	R	R Square	Adjusted R Square	Std. Error of the Estimate	Change Statistics		
					R Square Change	F Change	df1
1	.144[a]	.021	.012	1.40715	.021	2.448	1
2	.176[b]	.031	.005	1.41199	.010	.603	2
3	.204[c]	.042	-.001	1.41651	.011	.637	2
4	.318[d]	.101	-.001	1.41688	.059	.992	7

Model Summary

Model	Change Statistics	
	df2	Sig. F Change
1	116	.120
2	114	.549
3	112	.531
4	105	.442

a. Predictors: (Constant), Party0

b. Predictors: (Constant), Party0, SES0, RaceCC

c. Predictors: (Constant), Party0, SES0, RaceCC, MGRS0, MRN0

d. Predictors: (Constant), Party0, SES0, RaceCC, MGRS0, MRN0, MRN0xSES0, MGRS0xRace, MRN0xParty0, MGRS0xParty0, MGRS0xSES0, MRN0xRace, MRN0xMGRS0

ANOVA[a]

Model		Sum of Squares	df	Mean Square	F	Sig.
1	Regression	4.848	1	4.848	2.448	.120[b]
	Residual	229.687	116	1.980		
	Total	234.535	117			
2	Regression	7.250	3	2.417	1.212	.309[c]
	Residual	227.285	114	1.994		
	Total	234.535	117			
3	Regression	9.807	5	1.961	.978	.435[d]
	Residual	224.727	112	2.006		
	Total	234.535	117			
4	Regression	23.741	12	1.978	.985	.468[e]
	Residual	210.794	105	2.008		
	Total	234.535	117			

a. Dependent Variable: Resource_Tot

b. Predictors: (Constant), Party0

c. Predictors: (Constant), Party0, SES0, RaceCC

d. Predictors: (Constant), Party0, SES0, RaceCC, MGRS0, MRN0

e. Predictors: (Constant), Party0, SES0, RaceCC, MGRS0, MRN0, MRN0xSES0, MGRS0xRace, MRN0xParty0, MGRS0xParty0, MGRS0xSES0, MRN0xRace, MRN0xMGRS0

Coefficients[a]

Model		Unstandardized Coefficients B	Unstandardized Coefficients Std. Error	Standardized Coefficients Beta	t	Sig.
1	(Constant)	3.524	.133		26.437	.000
	Party0	-.149	.095	-.144	-1.565	.120
2	(Constant)	3.572	.153		23.279	.000
	Party0	-.133	.100	-.128	-1.328	.187
	RaceCC	-.093	.160	-.056	-.583	.561
	SES0	-.147	.150	-.090	-.977	.330
3	(Constant)	3.480	.185		18.816	.000
	Party0	-.203	.118	-.196	-1.720	.088
	RaceCC	-.055	.164	-.033	-.334	.739
	SES0	-.170	.152	-.104	-1.115	.267
	MGRS0	-.079	.258	-.031	-.306	.760
	MRN0	.218	.195	.134	1.116	.267
4	(Constant)	3.281	.262		12.516	.000
	Party0	-.257	.134	-.248	-1.918	.058
	RaceCC	.039	.225	.024	.176	.861
	SES0	-.071	.227	-.044	-.314	.754
	MGRS0	-.032	.419	-.013	-.076	.939
	MRN0	.483	.290	.297	1.668	.098
	MRN0xRace	-.398	.246	-.262	-1.621	.108
	MRN0xSES0	.023	.191	.015	.118	.906
	MRN0xParty0	.209	.123	.207	1.697	.093
	MRN0xMGRS0	-.037	.376	-.017	-.098	.922
	MGRS0xRace	.013	.383	.005	.034	.973
	MGRS0xSES0	.214	.384	.082	.558	.578
	MGRS0xParty0	-.051	.251	-.031	-.205	.838

Coefficients[a]

Model		Correlations Zero-order	Partial	Part
1	(Constant)			
	Party0	-.144	-.144	-.144
2	(Constant)			
	Party0	-.144	-.123	-.122
	RaceCC	-.085	-.055	-.054
	SES0	-.085	-.091	-.090
3	(Constant)			
	Party0	-.144	-.160	-.159
	RaceCC	-.085	-.032	-.031
	SES0	-.085	-.105	-.103
	MGRS0	.001	-.029	-.028
	MRN0	.014	.105	.103
4	(Constant)			
	Party0	-.144	-.184	-.177
	RaceCC	-.085	.017	.016
	SES0	-.085	-.031	-.029
	MGRS0	.001	-.007	-.007
	MRN0	.014	.161	.154
	MRN0xRace	-.156	-.156	-.150
	MRN0xSES0	.071	.012	.011
	MRN0xParty0	.061	.163	.157
	MRN0xMGRS0	.093	-.010	-.009
	MGRS0xRace	-.092	.003	.003
	MGRS0xSES0	.097	.054	.052
	MGRS0xParty0	.019	-.020	-.019

a. Dependent Variable: Resource_Tot

Excluded Variables[a]

Model		Beta In	t	Sig.	Partial Correlation	Collinearity Statistics Tolerance
1	RaceCC	-.048[b]	-.500	.618	-.047	.918
	SES0	-.086[b]	-.933	.353	-.087	1.000
	MGRS0	.017[b]	.181	.857	.017	.988
	MRN0	.110[b]	1.044	.298	.097	.765
	MRN0xRace	-.112[b]	-1.066	.289	-.099	.762
	MRN0xSES0	.042[b]	.451	.653	.042	.957
	MRN0xParty0	.146[b]	1.449	.150	.134	.829
	MRN0xMGRS0	.050[b]	.512	.609	.048	.885
	MGRS0xRace	-.088[b]	-.960	.339	-.089	.999
	MGRS0xSES0	.076[b]	.821	.414	.076	.976
	MGRS0xParty0	-.012[b]	-.131	.896	-.012	.953
2	MGRS0	.016[c]	.171	.865	.016	.981
	MRN0	.119[c]	1.091	.278	.102	.716
	MRN0xRace	-.101[c]	-.926	.356	-.087	.712
	MRN0xSES0	.069[c]	.713	.478	.067	.900
	MRN0xParty0	.142[c]	1.358	.177	.127	.770
	MRN0xMGRS0	.022[c]	.208	.836	.020	.791
	MGRS0xRace	-.106[c]	-1.086	.280	-.102	.888
	MGRS0xSES0	.052[c]	.462	.645	.043	.670
	MGRS0xParty0	-.026[c]	-.275	.784	-.026	.930
3	MRN0xRace	-.221[d]	-1.753	.082	-.164	.529
	MRN0xSES0	.095[d]	.930	.355	.088	.823
	MRN0xParty0	.163[d]	1.525	.130	.143	.737
	MRN0xMGRS0	.111[d]	.840	.403	.079	.493
	MGRS0xRace	-.119[d]	-1.110	.270	-.105	.741
	MGRS0xSES0	.092[d]	.774	.440	.073	.611
	MGRS0xParty0	.017[d]	.156	.876	.015	.740

a. Dependent Variable: Resource_Tot

b. Predictors in the Model: (Constant), Party0

c. Predictors in the Model: (Constant), Party0, SES0, RaceCC

d. Predictors in the Model: (Constant), Party0, SES0, RaceCC, MGRS0, MRN0

```
 REGRESSION
  /MISSING LISTWISE
  /STATISTICS COEFF OUTS R ANOVA CHANGE ZPP
  /CRITERIA=PIN(.05) POUT(.10)
  /NOORIGIN
  /DEPENDENT Psychology_Tot
  /METHOD=ENTER Party0
  /METHOD=ENTER RaceCC SES0
  /METHOD=ENTER MGRS0 MRN0
  /METHOD=ENTER MRN0xRace MRN0xSES0 MRN0xParty0 MRN0xMGRS0 MGRS0xRace MGRS0xSES0 MGRS0xPa
rty0.
```

Regression

Notes

Output Created		15-DEC-2021 13:07:58
Comments		
Input	Data	C:\Users\njs5478\Dropbox\HM and COVID\0. Revise and Resubmit\2. R and R Data\Study 1b\Study1b_Data.sav
	Active Dataset	DataSet1
	Filter	GenderCC=1 (FILTER)
	Weight	<none>
	Split File	<none>
	N of Rows in Working Data File	119
Missing Value Handling	Definition of Missing	User-defined missing values are treated as missing.
	Cases Used	Statistics are based on cases with no missing values for any variable used.

Notes

Syntax		REGRESSION<br>/MISSING LISTWISE<br>/STATISTICS COEFF OUTS R ANOVA CHANGE ZPP<br>/CRITERIA=PIN(.05) POUT(.10)<br>/NOORIGIN<br>/DEPENDENT Psychology_Tot<br>/METHOD=ENTER Party0<br>/METHOD=ENTER RaceCC SES0<br>/METHOD=ENTER MGRS0 MRN0<br>/METHOD=ENTER MRN0xRace MRN0xSES0 MRN0xParty0 MRN0xMGRS0 MGRS0xRace MGRS0xSES0 MGRS0xParty0.
Resources	Processor Time	00:00:00.03
	Elapsed Time	00:00:00.03
	Memory Required	48560 bytes
	Additional Memory Required for Residual Plots	0 bytes

Variables Entered/Removed[a]

Model	Variables Entered	Variables Removed	Method
1	Party0[b]	.	Enter
2	SES0, RaceCC[b]	.	Enter
3	MGRS0, MRN0[b]	.	Enter
4	MRN0xSES0, MGRS0xRace, MRN0xParty0, MGRS0xParty0, MGRS0xSES0, MRN0xRace, MRN0xMGRS...[b]	.	Enter

a. Dependent Variable: Psychology_Tot

b. All requested variables entered.

Model Summary

Model	R	R Square	Adjusted R Square	Std. Error of the Estimate	Change Statistics		
					R Square Change	F Change	df1
1	.303[a]	.092	.084	1.44249	.092	11.763	1
2	.313[b]	.098	.074	1.45038	.006	.371	2
3	.317[c]	.101	.061	1.46099	.003	.175	2
4	.382[d]	.146	.048	1.47063	.045	.791	7

Model Summary

Model	Change Statistics	
	df2	Sig. F Change
1	116	.001
2	114	.691
3	112	.840
4	105	.596

a. Predictors: (Constant), Party0

b. Predictors: (Constant), Party0, SES0, RaceCC

c. Predictors: (Constant), Party0, SES0, RaceCC, MGRS0, MRN0

d. Predictors: (Constant), Party0, SES0, RaceCC, MGRS0, MRN0, MRN0xSES0, MGRS0xRace, MRN0xParty0, MGRS0xParty0, MGRS0xSES0, MRN0xRace, MRN0xMGRS0

ANOVA[a]

Model		Sum of Squares	df	Mean Square	F	Sig.
1	Regression	24.475	1	24.475	11.763	.001[b]
	Residual	241.370	116	2.081		
	Total	265.846	117			
2	Regression	26.035	3	8.678	4.125	.008[c]
	Residual	239.811	114	2.104		
	Total	265.846	117			
3	Regression	26.782	5	5.356	2.509	.034[d]
	Residual	239.064	112	2.135		
	Total	265.846	117			
4	Regression	38.756	12	3.230	1.493	.138[e]
	Residual	227.090	105	2.163		
	Total	265.846	117			

a. Dependent Variable: Psychology_Tot

b. Predictors: (Constant), Party0

c. Predictors: (Constant), Party0, SES0, RaceCC

d. Predictors: (Constant), Party0, SES0, RaceCC, MGRS0, MRN0

e. Predictors: (Constant), Party0, SES0, RaceCC, MGRS0, MRN0, MRN0xSES0, MGRS0xRace, MRN0xParty0, MGRS0xParty0, MGRS0xSES0, MRN0xRace, MRN0xMGRS0

Coefficients[a]

Model		Unstandardized Coefficients B	Unstandardized Coefficients Std. Error	Standardized Coefficients Beta	t	Sig.
1	(Constant)	4.500	.137		32.936	.000
	Party0	-.335	.098	-.303	-3.430	.001
2	(Constant)	4.565	.158		28.967	.000
	Party0	-.310	.103	-.281	-3.021	.003
	RaceCC	-.140	.164	-.080	-.853	.396
	SES0	.007	.154	.004	.043	.966
3	(Constant)	4.610	.191		24.167	.000
	Party0	-.272	.122	-.246	-2.228	.028
	RaceCC	-.161	.169	-.091	-.952	.343
	SES0	.019	.157	.011	.121	.904
	MGRS0	.027	.266	.010	.100	.921
	MRN0	-.115	.201	-.067	-.572	.568
4	(Constant)	4.409	.272		16.202	.000
	Party0	-.263	.139	-.238	-1.892	.061
	RaceCC	-.240	.233	-.136	-1.027	.307
	SES0	.343	.236	.198	1.454	.149
	MGRS0	-.237	.435	-.089	-.545	.587
	MRN0	.096	.301	.056	.320	.750
	MRN0xRace	.075	.255	.046	.294	.769
	MRN0xSES0	-.243	.198	-.153	-1.227	.222
	MRN0xParty0	.027	.128	.025	.212	.832
	MRN0xMGRS0	.392	.390	.167	1.003	.318
	MGRS0xRace	-.271	.398	-.106	-.680	.498
	MGRS0xSES0	.422	.398	.151	1.059	.292
	MGRS0xParty0	.203	.261	.113	.780	.437

Coefficients[a]

Model		Correlations Zero-order	Partial	Part
1	(Constant)			
	Party0	-.303	-.303	-.303
2	(Constant)			
	Party0	-.303	-.272	-.269
	RaceCC	-.160	-.080	-.076
	SES0	.011	.004	.004
3	(Constant)			
	Party0	-.303	-.206	-.200
	RaceCC	-.160	-.090	-.085
	SES0	.011	.011	.011
	MGRS0	-.041	.009	.009
	MRN0	-.176	-.054	-.051
4	(Constant)			
	Party0	-.303	-.182	-.171
	RaceCC	-.160	-.100	-.093
	SES0	.011	.140	.131
	MGRS0	-.041	-.053	-.049
	MRN0	-.176	.031	.029
	MRN0xRace	-.192	.029	.026
	MRN0xSES0	.048	-.119	-.111
	MRN0xParty0	-.063	.021	.019
	MRN0xMGRS0	.231	.097	.090
	MGRS0xRace	-.043	-.066	-.061
	MGRS0xSES0	.087	.103	.095
	MGRS0xParty0	.152	.076	.070

a. Dependent Variable: Psychology_Tot

Excluded Variables[a]

Model		Beta In	t	Sig.	Partial Correlation	Collinearity Statistics Tolerance
1	RaceCC	-.080[b]	-.864	.390	-.080	.918
	SES0	.010[b]	.118	.907	.011	1.000
	MGRS0	-.008[b]	-.086	.931	-.008	.988
	MRN0	-.038[b]	-.375	.709	-.035	.765
	MRN0xRace	-.058[b]	-.567	.571	-.053	.762
	MRN0xSES0	-.016[b]	-.172	.863	-.016	.957
	MRN0xParty0	.076[b]	.781	.436	.073	.829
	MRN0xMGRS0	.144[b]	1.544	.125	.142	.885
	MGRS0xRace	-.035[b]	-.398	.691	-.037	.999
	MGRS0xSES0	.040[b]	.448	.655	.042	.976
	MGRS0xParty0	.090[b]	.995	.322	.092	.953
2	MGRS0	-.014[c]	-.151	.880	-.014	.981
	MRN0	-.062[c]	-.586	.559	-.055	.716
	MRN0xRace	-.038[c]	-.362	.718	-.034	.712
	MRN0xSES0	-.017[c]	-.177	.860	-.017	.900
	MRN0xParty0	.097[c]	.956	.341	.090	.770
	MRN0xMGRS0	.163[c]	1.647	.102	.153	.791
	MGRS0xRace	-.068[c]	-.717	.475	-.067	.888
	MGRS0xSES0	.081[c]	.745	.458	.070	.670
	MGRS0xParty0	.095[c]	1.028	.306	.096	.930
3	MRN0xRace	-.009[d]	-.072	.942	-.007	.529
	MRN0xSES0	-.029[d]	-.296	.768	-.028	.823
	MRN0xParty0	.093[d]	.887	.377	.084	.737
	MRN0xMGRS0	.228[d]	1.808	.073	.169	.493
	MGRS0xRace	-.083[d]	-.792	.430	-.075	.741
	MGRS0xSES0	.070[d]	.611	.542	.058	.611
	MGRS0xParty0	.096[d]	.920	.360	.087	.740

a. Dependent Variable: Psychology_Tot

b. Predictors in the Model: (Constant), Party0

c. Predictors in the Model: (Constant), Party0, SES0, RaceCC

d. Predictors in the Model: (Constant), Party0, SES0, RaceCC, MGRS0, MRN0

```
REGRESSION
  /MISSING LISTWISE
  /STATISTICS COEFF OUTS R ANOVA CHANGE ZPP
  /CRITERIA=PIN(.05) POUT(.10)
  /NOORIGIN
  /DEPENDENT TrumpApproval
  /METHOD=ENTER Party0
  /METHOD=ENTER RaceCC SES0
  /METHOD=ENTER MGRS0 MRN0
  /METHOD=ENTER MRN0xRace MRN0xSES0 MRN0xParty0 MRN0xMGRS0 MGRS0xRace MGRS0xSES0 MGRS0xPa
rty0.
```

Regression

Notes

Output Created		15-DEC-2021 13:07:58
Comments		
Input	Data	C:\Users\njs5478\Dropbox\HM and COVID\0. Revise and Resubmit\2. R and R Data\Study 1b\Study1b_Data.sav
	Active Dataset	DataSet1
	Filter	GenderCC=1 (FILTER)
	Weight	<none>
	Split File	<none>
	N of Rows in Working Data File	119
Missing Value Handling	Definition of Missing	User-defined missing values are treated as missing.
	Cases Used	Statistics are based on cases with no missing values for any variable used.

Notes

Syntax		REGRESSION<br>/MISSING LISTWISE<br>/STATISTICS COEFF OUTS R ANOVA CHANGE ZPP<br>/CRITERIA=PIN(.05) POUT(.10)<br>/NOORIGIN<br>/DEPENDENT TrumpApproval<br>/METHOD=ENTER Party0<br>/METHOD=ENTER RaceCC SES0<br>/METHOD=ENTER MGRS0 MRN0<br>/METHOD=ENTER MRN0xRace MRN0xSES0 MRN0xParty0 MRN0xMGRS0 MGRS0xRace MGRS0xSES0 MGRS0xParty0.
Resources	Processor Time	00:00:00.02
	Elapsed Time	00:00:00.02
	Memory Required	48560 bytes
	Additional Memory Required for Residual Plots	0 bytes

Variables Entered/Removed[a]

Model	Variables Entered	Variables Removed	Method
1	Party0[b]	.	Enter
2	SES0, RaceCC[b]	.	Enter
3	MGRS0, MRN0[b]	.	Enter
4	MRN0xSES0, MGRS0xRace, MRN0xParty0, MGRS0xParty0, MGRS0xSES0, MRN0xRace, MRN0xMGRS...[b]	.	Enter

a. Dependent Variable: Do you approve or disapprove of the way Donald Trump is handling his job as President?

b. All requested variables entered.

Model Summary

Model	R	R Square	Adjusted R Square	Std. Error of the Estimate	Change Statistics		
					R Square Change	F Change	df1
1	.743[a]	.551	.547	1.484	.551	142.562	1
2	.763[b]	.583	.572	1.443	.031	4.273	2
3	.790[c]	.624	.607	1.382	.041	6.143	2
4	.796[d]	.634	.592	1.409	.010	.404	7

Model Summary

Model	Change Statistics	
	df2	Sig. F Change
1	116	.000
2	114	.016
3	112	.003
4	105	.898

a. Predictors: (Constant), Party0

b. Predictors: (Constant), Party0, SES0, RaceCC

c. Predictors: (Constant), Party0, SES0, RaceCC, MGRS0, MRN0

d. Predictors: (Constant), Party0, SES0, RaceCC, MGRS0, MRN0, MRN0xSES0, MGRS0xRace, MRN0xParty0, MGRS0xParty0, MGRS0xSES0, MRN0xRace, MRN0xMGRS0

ANOVA[a]

Model		Sum of Squares	df	Mean Square	F	Sig.
1	Regression	313.774	1	313.774	142.562	.000[b]
	Residual	255.311	116	2.201		
	Total	569.085	117			
2	Regression	331.578	3	110.526	53.051	.000[c]
	Residual	237.507	114	2.083		
	Total	569.085	117			
3	Regression	355.056	5	71.011	37.160	.000[d]
	Residual	214.029	112	1.911		
	Total	569.085	117			
4	Regression	360.673	12	30.056	15.143	.000[e]
	Residual	208.412	105	1.985		
	Total	569.085	117			

a. Dependent Variable: Do you approve or disapprove of the way Donald Trump is handling his job as President?

b. Predictors: (Constant), Party0

c. Predictors: (Constant), Party0, SES0, RaceCC

d. Predictors: (Constant), Party0, SES0, RaceCC, MGRS0, MRN0

e. Predictors: (Constant), Party0, SES0, RaceCC, MGRS0, MRN0, MRN0xSES0, MGRS0xRace, MRN0xParty0, MGRS0xParty0, MGRS0xSES0, MRN0xRace, MRN0xMGRS0

Coefficients[a]

Model		Unstandardized Coefficients		Standardized Coefficients	t	Sig.
		B	Std. Error	Beta		
1	(Constant)	3.164	.141		22.517	.000
	Party0	1.201	.101	.743	11.940	.000
2	(Constant)	2.956	.157		18.846	.000
	Party0	1.119	.102	.692	10.952	.000
	RaceCC	.454	.164	.176	2.772	.007
	SES0	-.105	.154	-.042	-.685	.495
3	(Constant)	2.788	.181		15.444	.000
	Party0	.918	.115	.568	7.950	.000
	RaceCC	.569	.160	.220	3.549	.001
	SES0	-.170	.148	-.067	-1.145	.255
	MGRS0	.119	.251	.031	.475	.636
	MRN0	.565	.190	.223	2.965	.004
4	(Constant)	2.857	.261		10.961	.000
	Party0	.831	.133	.514	6.236	.000
	RaceCC	.533	.223	.207	2.386	.019
	SES0	-.191	.226	-.075	-.844	.400
	MGRS0	.259	.417	.066	.621	.536
	MRN0	.431	.288	.170	1.497	.137
	MRN0xRace	.114	.244	.048	.466	.642
	MRN0xSES0	.014	.190	.006	.073	.942
	MRN0xParty0	.121	.122	.077	.992	.323
	MRN0xMGRS0	-.168	.374	-.049	-.450	.654
	MGRS0xRace	.179	.381	.048	.469	.640
	MGRS0xSES0	-.007	.382	-.002	-.018	.986
	MGRS0xParty0	-.080	.250	-.031	-.321	.749

Coefficients[a]

Model		Correlations Zero-order	Partial	Part
1	(Constant)			
	Party0	.743	.743	.743
2	(Constant)			
	Party0	.743	.716	.663
	RaceCC	.378	.251	.168
	SES0	-.058	-.064	-.041
3	(Constant)			
	Party0	.743	.601	.461
	RaceCC	.378	.318	.206
	SES0	-.058	-.108	-.066
	MGRS0	.176	.045	.028
	MRN0	.493	.270	.172
4	(Constant)			
	Party0	.743	.520	.368
	RaceCC	.378	.227	.141
	SES0	-.058	-.082	-.050
	MGRS0	.176	.060	.037
	MRN0	.493	.145	.088
	MRN0xRace	.529	.045	.028
	MRN0xSES0	-.213	.007	.004
	MRN0xParty0	.354	.096	.059
	MRN0xMGRS0	-.300	-.044	-.027
	MGRS0xRace	.035	.046	.028
	MGRS0xSES0	-.111	-.002	-.001
	MGRS0xParty0	-.192	-.031	-.019

a. Dependent Variable: Do you approve or disapprove of the way Donald Trump is handling his job as President?

Excluded Variables[a]

Model		Beta In	t	Sig.	Partial Correlation	Collinearity Statistics Tolerance
1	RaceCC	.180[b]	2.849	.005	.257	.918
	SES0	-.056[b]	-.903	.368	-.084	1.000
	MGRS0	.095[b]	1.522	.131	.141	.988
	MRN0	.173[b]	2.494	.014	.227	.765
	MRN0xRace	.219[b]	3.194	.002	.285	.762
	MRN0xSES0	-.061[b]	-.952	.343	-.088	.957
	MRN0xParty0	.056[b]	.825	.411	.077	.829
	MRN0xMGRS0	-.055[b]	-.824	.412	-.077	.885
	MGRS0xRace	.016[b]	.252	.802	.023	.999
	MGRS0xSES0	.004[b]	.071	.943	.007	.976
	MGRS0xParty0	-.033[b]	-.518	.605	-.048	.953
2	MGRS0	.109[c]	1.808	.073	.168	.981
	MRN0	.238[c]	3.485	.001	.312	.716
	MRN0xRace	.183[c]	2.621	.010	.239	.712
	MRN0xSES0	-.056[c]	-.873	.385	-.082	.900
	MRN0xParty0	.018[c]	.266	.790	.025	.770
	MRN0xMGRS0	-.078[c]	-1.153	.251	-.108	.791
	MGRS0xRace	.084[c]	1.316	.191	.123	.888
	MGRS0xSES0	-.070[c]	-.941	.349	-.088	.670
	MGRS0xParty0	-.045[c]	-.711	.479	-.067	.930
3	MRN0xRace	.097[d]	1.222	.224	.115	.529
	MRN0xSES0	-.004[d]	-.064	.949	-.006	.823
	MRN0xParty0	.062[d]	.918	.361	.087	.737
	MRN0xMGRS0	-.058[d]	-.696	.488	-.066	.493
	MGRS0xRace	.074[d]	1.100	.274	.104	.741
	MGRS0xSES0	.003[d]	.043	.966	.004	.611
	MGRS0xParty0	.005[d]	.075	.941	.007	.740

a. Dependent Variable: Do you approve or disapprove of the way Donald Trump is handling his job as President?

b. Predictors in the Model: (Constant), Party0

c. Predictors in the Model: (Constant), Party0, SES0, RaceCC

d. Predictors in the Model: (Constant), Party0, SES0, RaceCC, MGRS0, MRN0

```
REGRESSION
  /MISSING LISTWISE
  /STATISTICS COEFF OUTS R ANOVA CHANGE ZPP
  /CRITERIA=PIN(.05) POUT(.10)
  /NOORIGIN
  /DEPENDENT TrumpX
  /METHOD=ENTER Party0
  /METHOD=ENTER RaceCC SES0
  /METHOD=ENTER MGRS0 MRN0
  /METHOD=ENTER MRN0xRace MRN0xSES0 MRN0xParty0 MRN0xMGRS0 MGRS0xRace MGRS0xSES0 MGRS0xPa
rty0.
```

Regression

Notes

Output Created		15-DEC-2021 13:07:58
Comments		
Input	Data	C:\Users\njs5478\Dropbox\HM and COVID\0. Revise and Resubmit\2. R and R Data\Study 1b\Study1b_Data.sav
	Active Dataset	DataSet1
	Filter	GenderCC=1 (FILTER)
	Weight	<none>
	Split File	<none>
	N of Rows in Working Data File	119
Missing Value Handling	Definition of Missing	User-defined missing values are treated as missing.
	Cases Used	Statistics are based on cases with no missing values for any variable used.

Notes

Syntax		REGRESSION<br>/MISSING LISTWISE<br>/STATISTICS COEFF OUTS R ANOVA CHANGE ZPP<br>/CRITERIA=PIN(.05) POUT(.10)<br>/NOORIGIN<br>/DEPENDENT TrumpX<br>/METHOD=ENTER Party0<br>/METHOD=ENTER RaceCC SES0<br>/METHOD=ENTER MGRS0 MRN0<br>/METHOD=ENTER MRN0xRace MRN0xSES0 MRN0xParty0 MRN0xMGRS0 MGRS0xRace MGRS0xSES0 MGRS0xParty0.
Resources	Processor Time	00:00:00.08
	Elapsed Time	00:00:00.03
	Memory Required	48560 bytes
	Additional Memory Required for Residual Plots	0 bytes

Variables Entered/Removed[a]

Model	Variables Entered	Variables Removed	Method
1	Party0[b]	.	Enter
2	SES0, RaceCC[b]	.	Enter
3	MGRS0, MRN0[b]	.	Enter
4	MRN0xSES0, MGRS0xRace, MRN0xParty0, MGRS0xParty0, MGRS0xSES0, MRN0xRace, MRN0xMGRS...[b]	.	Enter

a. Dependent Variable: TrumpX

b. All requested variables entered.

Model Summary

Model	R	R Square	Adjusted R Square	Std. Error of the Estimate	Change Statistics		
					R Square Change	F Change	df1
1	.746[a]	.557	.553	1.42962	.557	145.972	1
2	.760[b]	.577	.566	1.40960	.020	2.659	2
3	.794[c]	.631	.614	1.32842	.054	8.179	2
4	.799[d]	.639	.598	1.35714	.008	.330	7

Model Summary

Model	Change Statistics	
	df2	Sig. F Change
1	116	.000
2	114	.074
3	112	.000
4	105	.939

a. Predictors: (Constant), Party0

b. Predictors: (Constant), Party0, SES0, RaceCC

c. Predictors: (Constant), Party0, SES0, RaceCC, MGRS0, MRN0

d. Predictors: (Constant), Party0, SES0, RaceCC, MGRS0, MRN0, MRN0xSES0, MGRS0xRace, MRN0xParty0, MGRS0xParty0, MGRS0xSES0, MRN0xRace, MRN0xMGRS0

ANOVA[a]

Model		Sum of Squares	df	Mean Square	F	Sig.
1	Regression	298.341	1	298.341	145.972	.000[b]
	Residual	237.083	116	2.044		
	Total	535.424	117			
2	Regression	308.909	3	102.970	51.822	.000[c]
	Residual	226.515	114	1.987		
	Total	535.424	117			
3	Regression	337.777	5	67.555	38.282	.000[d]
	Residual	197.647	112	1.765		
	Total	535.424	117			
4	Regression	342.032	12	28.503	15.475	.000[e]
	Residual	193.392	105	1.842		
	Total	535.424	117			

a. Dependent Variable: TrumpX

b. Predictors: (Constant), Party0

c. Predictors: (Constant), Party0, SES0, RaceCC

d. Predictors: (Constant), Party0, SES0, RaceCC, MGRS0, MRN0

e. Predictors: (Constant), Party0, SES0, RaceCC, MGRS0, MRN0, MRN0xSES0, MGRS0xRace, MRN0xParty0, MGRS0xParty0, MGRS0xSES0, MRN0xRace, MRN0xMGRS0

Coefficients[a]

Model		Unstandardized Coefficients B	Unstandardized Coefficients Std. Error	Standardized Coefficients Beta	t	Sig.
1	(Constant)	3.089	.135		22.813	.000
	Party0	1.171	.097	.746	12.082	.000
2	(Constant)	2.920	.153		19.061	.000
	Party0	1.105	.100	.705	11.076	.000
	RaceCC	.365	.160	.146	2.284	.024
	SES0	-.018	.150	-.007	-.121	.904
3	(Constant)	2.737	.173		15.779	.000
	Party0	.883	.111	.563	7.960	.000
	RaceCC	.493	.154	.197	3.197	.002
	SES0	-.090	.143	-.037	-.628	.531
	MGRS0	.143	.242	.038	.593	.554
	MRN0	.621	.183	.253	3.397	.001
4	(Constant)	2.834	.251		11.284	.000
	Party0	.827	.128	.528	6.441	.000
	RaceCC	.412	.215	.165	1.913	.058
	SES0	-.109	.218	-.045	-.503	.616
	MGRS0	.378	.402	.100	.941	.349
	MRN0	.519	.277	.212	1.872	.064
	MRN0xRace	.111	.235	.048	.470	.640
	MRN0xSES0	.077	.183	.034	.419	.676
	MRN0xParty0	.083	.118	.055	.708	.480
	MRN0xMGRS0	-.314	.360	-.094	-.872	.385
	MGRS0xRace	-.056	.367	-.015	-.152	.880
	MGRS0xSES0	.061	.368	.016	.167	.868
	MGRS0xParty0	.121	.240	.047	.502	.617

Coefficients[a]

Model		Correlations		
		Zero-order	Partial	Part
1	(Constant)			
	Party0	.746	.746	.746
2	(Constant)			
	Party0	.746	.720	.675
	RaceCC	.349	.209	.139
	SES0	-.021	-.011	-.007
3	(Constant)			
	Party0	.746	.601	.457
	RaceCC	.349	.289	.184
	SES0	-.021	-.059	-.036
	MGRS0	.197	.056	.034
	MRN0	.528	.306	.195
4	(Constant)			
	Party0	.746	.532	.378
	RaceCC	.349	.184	.112
	SES0	-.021	-.049	-.030
	MGRS0	.197	.091	.055
	MRN0	.528	.180	.110
	MRN0xRace	.519	.046	.028
	MRN0xSES0	-.183	.041	.025
	MRN0xParty0	.338	.069	.042
	MRN0xMGRS0	-.307	-.085	-.051
	MGRS0xRace	.033	-.015	-.009
	MGRS0xSES0	-.131	.016	.010
	MGRS0xParty0	-.177	.049	.029

a. Dependent Variable: TrumpX

Excluded Variables[a]

Model		Beta In	t	Sig.	Partial Correlation	Collinearity Statistics Tolerance
1	RaceCC	.146[b]	2.313	.022	.211	.918
	SES0	-.020[b]	-.315	.754	-.029	1.000
	MGRS0	.116[b]	1.883	.062	.173	.988
	MRN0	.218[b]	3.204	.002	.286	.765
	MRN0xRace	.203[b]	2.958	.004	.266	.762
	MRN0xSES0	-.029[b]	-.458	.648	-.043	.957
	MRN0xParty0	.035[b]	.515	.607	.048	.829
	MRN0xMGRS0	-.061[b]	-.930	.354	-.086	.885
	MGRS0xRace	.014[b]	.223	.824	.021	.999
	MGRS0xSES0	-.015[b]	-.241	.810	-.022	.976
	MGRS0xParty0	-.017[b]	-.260	.795	-.024	.953
2	MGRS0	.127[c]	2.100	.038	.194	.981
	MRN0	.271[c]	4.012	.000	.353	.716
	MRN0xRace	.174[c]	2.462	.015	.226	.712
	MRN0xSES0	-.031[c]	-.477	.634	-.045	.900
	MRN0xParty0	.010[c]	.143	.887	.013	.770
	MRN0xMGRS0	-.072[c]	-1.056	.293	-.099	.791
	MGRS0xRace	.067[c]	1.036	.302	.097	.888
	MGRS0xSES0	-.063[c]	-.850	.397	-.080	.670
	MGRS0xParty0	-.021[c]	-.337	.737	-.032	.930
3	MRN0xRace	.064[d]	.811	.419	.077	.529
	MRN0xSES0	.032[d]	.502	.617	.048	.823
	MRN0xParty0	.060[d]	.890	.375	.084	.737
	MRN0xMGRS0	-.041[d]	-.498	.619	-.047	.493
	MGRS0xRace	.048[d]	.721	.472	.068	.741
	MGRS0xSES0	.022[d]	.293	.770	.028	.611
	MGRS0xParty0	.042[d]	.625	.534	.059	.740

a. Dependent Variable: TrumpX

b. Predictors in the Model: (Constant), Party0

c. Predictors in the Model: (Constant), Party0, SES0, RaceCC

d. Predictors in the Model: (Constant), Party0, SES0, RaceCC, MGRS0, MRN0

```
REGRESSION
  /MISSING LISTWISE
  /STATISTICS COEFF OUTS R ANOVA CHANGE ZPP
  /CRITERIA=PIN(.05) POUT(.10)
  /NOORIGIN
  /DEPENDENT BidenX
  /METHOD=ENTER Party0
  /METHOD=ENTER RaceCC SES0
  /METHOD=ENTER MGRS0 MRN0
  /METHOD=ENTER MRN0xRace MRN0xSES0 MRN0xParty0 MRN0xMGRS0 MGRS0xRace MGRS0xSES0 MGRS0xPa
rty0.
```

Regression

Notes

Output Created		15-DEC-2021 13:07:58
Comments		
Input	Data	C:\Users\njs5478\Dropbox\HM and COVID\0. Revise and Resubmit\2. R and R Data\Study 1b\Study1b_Data.sav
	Active Dataset	DataSet1
	Filter	GenderCC=1 (FILTER)
	Weight	<none>
	Split File	<none>
	N of Rows in Working Data File	119
Missing Value Handling	Definition of Missing	User-defined missing values are treated as missing.
	Cases Used	Statistics are based on cases with no missing values for any variable used.

Notes

Syntax		REGRESSION<br>/MISSING LISTWISE<br>/STATISTICS COEFF OUTS R ANOVA CHANGE ZPP<br>/CRITERIA=PIN(.05) POUT(.10)<br>/NOORIGIN<br>/DEPENDENT BidenX<br>/METHOD=ENTER Party0<br>/METHOD=ENTER RaceCC SES0<br>/METHOD=ENTER MGRS0 MRN0<br>/METHOD=ENTER MRN0xRace MRN0xSES0 MRN0xParty0 MRN0xMGRS0 MGRS0xRace MGRS0xSES0 MGRS0xParty0.
Resources	Processor Time	00:00:00.03
	Elapsed Time	00:00:00.03
	Memory Required	48560 bytes
	Additional Memory Required for Residual Plots	0 bytes

Variables Entered/Removed[a]

Model	Variables Entered	Variables Removed	Method
1	Party0[b]	.	Enter
2	SES0, RaceCC[b]	.	Enter
3	MGRS0, MRN0[b]	.	Enter
4	MRN0xSES0, MGRS0xRace, MRN0xParty0, MGRS0xParty0, MGRS0xSES0, MRN0xRace, MRN0xMGRS...[b]	.	Enter

a. Dependent Variable: BidenX

b. All requested variables entered.

Model Summary

Model	R	R Square	Adjusted R Square	Std. Error of the Estimate	Change Statistics		
					R Square Change	F Change	df1
1	.675[a]	.456	.451	1.39512	.456	95.516	1
2	.679[b]	.461	.447	1.40083	.005	.537	2
3	.699[c]	.489	.466	1.37628	.028	3.016	2
4	.708[d]	.501	.443	1.40586	.012	.346	7

Model Summary

Model	Change Statistics	
	df2	Sig. F Change
1	114	.000
2	112	.586
3	110	.053
4	103	.931

a. Predictors: (Constant), Party0

b. Predictors: (Constant), Party0, SES0, RaceCC

c. Predictors: (Constant), Party0, SES0, RaceCC, MGRS0, MRN0

d. Predictors: (Constant), Party0, SES0, RaceCC, MGRS0, MRN0, MRN0xSES0, MGRS0xRace, MRN0xParty0, MGRS0xParty0, MGRS0xSES0, MRN0xRace, MRN0xMGRS0

ANOVA[a]

Model		Sum of Squares	df	Mean Square	F	Sig.
1	Regression	185.908	1	185.908	95.516	.000[b]
	Residual	221.885	114	1.946		
	Total	407.793	115			
2	Regression	188.014	3	62.671	31.938	.000[c]
	Residual	219.779	112	1.962		
	Total	407.793	115			
3	Regression	199.438	5	39.888	21.058	.000[d]
	Residual	208.355	110	1.894		
	Total	407.793	115			
4	Regression	204.219	12	17.018	8.611	.000[e]
	Residual	203.574	103	1.976		
	Total	407.793	115			

a. Dependent Variable: BidenX

b. Predictors: (Constant), Party0

c. Predictors: (Constant), Party0, SES0, RaceCC

d. Predictors: (Constant), Party0, SES0, RaceCC, MGRS0, MRN0

e. Predictors: (Constant), Party0, SES0, RaceCC, MGRS0, MRN0, MRN0xSES0, MGRS0xRace, MRN0xParty0, MGRS0xParty0, MGRS0xSES0, MRN0xRace, MRN0xMGRS0

Coefficients[a]

Model		Unstandardized Coefficients B	Unstandardized Coefficients Std. Error	Standardized Coefficients Beta	t	Sig.
1	(Constant)	4.441	.133		33.343	.000
	Party0	-.924	.095	-.675	-9.773	.000
2	(Constant)	4.517	.154		29.289	.000
	Party0	-.895	.099	-.654	-9.017	.000
	RaceCC	-.160	.161	-.072	-.994	.323
	SES0	.035	.151	.016	.230	.819
3	(Constant)	4.651	.180		25.821	.000
	Party0	-.751	.115	-.549	-6.533	.000
	RaceCC	-.242	.162	-.109	-1.494	.138
	SES0	.082	.150	.038	.547	.586
	MGRS0	-.035	.254	-.011	-.139	.889
	MRN0	-.412	.190	-.192	-2.169	.032
4	(Constant)	4.580	.261		17.525	.000
	Party0	-.818	.133	-.597	-6.145	.000
	RaceCC	-.227	.225	-.102	-1.011	.314
	SES0	.178	.226	.082	.785	.434
	MGRS0	-.004	.429	-.001	-.010	.992
	MRN0	-.391	.291	-.182	-1.342	.183
	MRN0xRace	-.062	.253	-.031	-.247	.805
	MRN0xSES0	-.055	.192	-.027	-.284	.777
	MRN0xParty0	.150	.123	.113	1.223	.224
	MRN0xMGRS0	.110	.375	.037	.292	.771
	MGRS0xRace	.154	.401	.048	.384	.701
	MGRS0xSES0	.175	.396	.049	.442	.659
	MGRS0xParty0	-.238	.253	-.107	-.939	.350

Coefficients[a]

Model		Correlations Zero-order	Partial	Part
1	(Constant)			
	Party0	-.675	-.675	-.675
2	(Constant)			
	Party0	-.675	-.649	-.626
	RaceCC	-.264	-.093	-.069
	SES0	.023	.022	.016
3	(Constant)			
	Party0	-.675	-.529	-.445
	RaceCC	-.264	-.141	-.102
	SES0	.023	.052	.037
	MGRS0	-.147	-.013	-.010
	MRN0	-.455	-.203	-.148
4	(Constant)			
	Party0	-.675	-.518	-.428
	RaceCC	-.264	-.099	-.070
	SES0	.023	.077	.055
	MGRS0	-.147	-.001	-.001
	MRN0	-.455	-.131	-.093
	MRN0xRace	-.408	-.024	-.017
	MRN0xSES0	.184	-.028	-.020
	MRN0xParty0	-.211	.120	.085
	MRN0xMGRS0	.271	.029	.020
	MGRS0xRace	-.015	.038	.027
	MGRS0xSES0	.164	.044	.031
	MGRS0xParty0	.148	-.092	-.065

a. Dependent Variable: BidenX

Excluded Variables[a]

Model		Beta In	t	Sig.	Partial Correlation	Collinearity Statistics Tolerance
1	RaceCC	-.073[b]	-1.014	.313	-.095	.915
	SES0	.020[b]	.293	.770	.028	1.000
	MGRS0	-.071[b]	-1.028	.306	-.096	.987
	MRN0	-.166[b]	-2.132	.035	-.197	.764
	MRN0xRace	-.101[b]	-1.278	.204	-.119	.759
	MRN0xSES0	.043[b]	.610	.543	.057	.956
	MRN0xParty0	.083[b]	1.093	.277	.102	.828
	MRN0xMGRS0	.046[b]	.627	.532	.059	.883
	MGRS0xRace	.003[b]	.047	.963	.004	.999
	MGRS0xSES0	.058[b]	.828	.410	.078	.975
	MGRS0xParty0	.002[b]	.033	.973	.003	.953
2	MGRS0	-.079[c]	-1.133	.260	-.107	.977
	MRN0	-.197[c]	-2.463	.015	-.228	.718
	MRN0xRace	-.088[c]	-1.075	.285	-.102	.714
	MRN0xSES0	.044[c]	.607	.545	.058	.905
	MRN0xParty0	.106[c]	1.348	.180	.127	.769
	MRN0xMGRS0	.056[c]	.717	.475	.068	.798
	MGRS0xRace	-.021[c]	-.289	.773	-.027	.902
	MGRS0xSES0	.108[c]	1.295	.198	.122	.693
	MGRS0xParty0	.006[c]	.087	.931	.008	.931
3	MRN0xRace	.009[d]	.092	.927	.009	.526
	MRN0xSES0	.004[d]	.057	.955	.005	.830
	MRN0xParty0	.077[d]	.974	.332	.093	.736
	MRN0xMGRS0	.019[d]	.200	.841	.019	.501
	MGRS0xRace	-.010[d]	-.128	.898	-.012	.745
	MGRS0xSES0	.054[d]	.622	.535	.059	.625
	MGRS0xParty0	-.049[d]	-.620	.537	-.059	.740

a. Dependent Variable: BidenX

b. Predictors in the Model: (Constant), Party0

c. Predictors in the Model: (Constant), Party0, SES0, RaceCC

d. Predictors in the Model: (Constant), Party0, SES0, RaceCC, MGRS0, MRN0

```
REGRESSION
  /MISSING LISTWISE
  /STATISTICS COEFF OUTS R ANOVA CHANGE ZPP
  /CRITERIA=PIN(.05) POUT(.10)
  /NOORIGIN
  /DEPENDENT Pelosi
  /METHOD=ENTER Party0
  /METHOD=ENTER RaceCC SES0
  /METHOD=ENTER MGRS0 MRN0
  /METHOD=ENTER MRN0xRace MRN0xSES0 MRN0xParty0 MRN0xMGRS0 MGRS0xRace MGRS0xSES0 MGRS0xPa
rty0.
```

Regression

Notes

Output Created		15-DEC-2021 13:07:58
Comments		
Input	Data	C:\Users\njs5478\Dropbox\HM and COVID\0. Revise and Resubmit\2. R and R Data\Study 1b\Study1b_Data.sav
	Active Dataset	DataSet1
	Filter	GenderCC=1 (FILTER)
	Weight	<none>
	Split File	<none>
	N of Rows in Working Data File	119
Missing Value Handling	Definition of Missing	User-defined missing values are treated as missing.
	Cases Used	Statistics are based on cases with no missing values for any variable used.

Notes

Syntax		REGRESSION<br>/MISSING LISTWISE<br>/STATISTICS COEFF OUTS R ANOVA CHANGE ZPP<br>/CRITERIA=PIN(.05) POUT(.10)<br>/NOORIGIN<br>/DEPENDENT Pelosi<br>/METHOD=ENTER Party0<br>/METHOD=ENTER RaceCC SES0<br>/METHOD=ENTER MGRS0 MRN0<br>/METHOD=ENTER MRN0xRace MRN0xSES0 MRN0xParty0 MRN0xMGRS0 MGRS0xRace MGRS0xSES0 MGRS0xParty0.
Resources	Processor Time	00:00:00.03
	Elapsed Time	00:00:00.03
	Memory Required	48560 bytes
	Additional Memory Required for Residual Plots	0 bytes

Variables Entered/Removed[a]

Model	Variables Entered	Variables Removed	Method
1	Party0[b]	.	Enter
2	SES0, RaceCC[b]	.	Enter
3	MGRS0, MRN0[b]	.	Enter
4	MRN0xSES0, MGRS0xRace , MRN0xParty0 , MGRS0xParty 0, MGRS0xSES 0, MRN0xRace, MRN0xMGRS...[b]	.	Enter

a. Dependent Variable: Using the scale provided, please indicate your personal opinion regarding how each of the following is handling the response to COVID-19 (Coronavirus) in the United States: - Nancy Pelosi

b. All requested variables entered.

Model Summary

Model	R	R Square	Adjusted R Square	Std. Error of the Estimate	Change Statistics		
					R Square Change	F Change	df1
1	.465[a]	.217	.210	1.991	.217	32.087	1
2	.493[b]	.243	.223	1.974	.026	1.959	2
3	.517[c]	.267	.235	1.959	.025	1.885	2
4	.542[d]	.293	.213	1.987	.026	.550	7

Model Summary

Model	Change Statistics df2	Sig. F Change
1	116	.000
2	114	.146
3	112	.157
4	105	.794

a. Predictors: (Constant), Party0

b. Predictors: (Constant), Party0, SES0, RaceCC

c. Predictors: (Constant), Party0, SES0, RaceCC, MGRS0, MRN0

d. Predictors: (Constant), Party0, SES0, RaceCC, MGRS0, MRN0, MRN0xSES0, MGRS0xRace, MRN0xParty0, MGRS0xParty0, MGRS0xSES0, MRN0xRace, MRN0xMGRS0

ANOVA[a]

Model		Sum of Squares	df	Mean Square	F	Sig.
1	Regression	127.150	1	127.150	32.087	.000[b]
	Residual	459.672	116	3.963		
	Total	586.822	117			
2	Regression	142.424	3	47.475	12.179	.000[c]
	Residual	444.398	114	3.898		
	Total	586.822	117			
3	Regression	156.897	5	31.379	8.175	.000[d]
	Residual	429.925	112	3.839		
	Total	586.822	117			
4	Regression	172.111	12	14.343	3.631	.000[e]
	Residual	414.711	105	3.950		
	Total	586.822	117			

a. Dependent Variable: Using the scale provided, please indicate your personal opinion regarding how each of the following is handling the response to COVID-19 (Coronavirus) in the United States: - Nancy Pelosi

b. Predictors: (Constant), Party0

c. Predictors: (Constant), Party0, SES0, RaceCC

d. Predictors: (Constant), Party0, SES0, RaceCC, MGRS0, MRN0

e. Predictors: (Constant), Party0, SES0, RaceCC, MGRS0, MRN0, MRN0xSES0, MGRS0xRace, MRN0xParty0, MGRS0xParty0, MGRS0xSES0, MRN0xRace, MRN0xMGRS0

Coefficients[a]

Model		Unstandardized Coefficients B	Unstandardized Coefficients Std. Error	Standardized Coefficients Beta	t	Sig.
1	(Constant)	4.023	.189		21.334	.000
	Party0	-.764	.135	-.465	-5.665	.000
2	(Constant)	4.094	.215		19.082	.000
	Party0	-.742	.140	-.452	-5.312	.000
	RaceCC	-.126	.224	-.048	-.561	.576
	SES0	-.407	.210	-.159	-1.940	.055
3	(Constant)	4.201	.256		16.420	.000
	Party0	-.591	.164	-.360	-3.614	.000
	RaceCC	-.214	.227	-.082	-.940	.349
	SES0	-.359	.210	-.140	-1.706	.091
	MGRS0	-.163	.356	-.041	-.457	.649
	MRN0	-.413	.270	-.161	-1.529	.129
4	(Constant)	4.202	.368		11.428	.000
	Party0	-.535	.188	-.326	-2.846	.005
	RaceCC	-.056	.315	-.021	-.178	.859
	SES0	-.742	.319	-.289	-2.330	.022
	MGRS0	-.234	.588	-.059	-.397	.692
	MRN0	-.492	.406	-.192	-1.212	.228
	MRN0xRace	-.124	.345	-.051	-.360	.720
	MRN0xSES0	.295	.268	.124	1.101	.274
	MRN0xParty0	-.098	.173	-.061	-.565	.573
	MRN0xMGRS0	.209	.528	.060	.395	.693
	MGRS0xRace	.275	.537	.073	.512	.610
	MGRS0xSES0	-.818	.538	-.198	-1.521	.131
	MGRS0xParty0	-.361	.352	-.136	-1.025	.308

Coefficients[a]

Model		Correlations: Zero-order	Partial	Part
1	(Constant)			
	Party0	-.465	-.465	-.465
2	(Constant)			
	Party0	-.465	-.445	-.433
	RaceCC	-.164	-.052	-.046
	SES0	-.154	-.179	-.158
3	(Constant)			
	Party0	-.465	-.323	-.292
	RaceCC	-.164	-.088	-.076
	SES0	-.154	-.159	-.138
	MGRS0	-.150	-.043	-.037
	MRN0	-.366	-.143	-.124
4	(Constant)			
	Party0	-.465	-.268	-.233
	RaceCC	-.164	-.017	-.015
	SES0	-.154	-.222	-.191
	MGRS0	-.150	-.039	-.033
	MRN0	-.366	-.117	-.099
	MRN0xRace	-.331	-.035	-.030
	MRN0xSES0	.115	.107	.090
	MRN0xParty0	-.205	-.055	-.046
	MRN0xMGRS0	.209	.039	.032
	MGRS0xRace	-.051	.050	.042
	MGRS0xSES0	.126	-.147	-.125
	MGRS0xParty0	.096	-.100	-.084

a. Dependent Variable: Using the scale provided, please indicate your personal opinion regarding how each of the following is handling the response to COVID-19 (Coronavirus) in the United States: - Nancy Pelosi

Excluded Variables[a]

Model		Beta In	t	Sig.	Partial Correlation	Collinearity Statistics Tolerance
1	RaceCC	-.034[b]	-.390	.698	-.036	.918
	SES0	-.155[b]	-1.904	.059	-.175	1.000
	MGRS0	-.099[b]	-1.200	.232	-.111	.988
	MRN0	-.184[b]	-1.979	.050	-.181	.765
	MRN0xRace	-.136[b]	-1.451	.149	-.134	.762
	MRN0xSES0	.019[b]	.227	.821	.021	.957
	MRN0xParty0	-.014[b]	-.158	.875	-.015	.829
	MRN0xMGRS0	.058[b]	.661	.510	.062	.885
	MGRS0xRace	-.038[b]	-.466	.642	-.043	.999
	MGRS0xSES0	.055[b]	.661	.510	.062	.976
	MGRS0xParty0	-.004[b]	-.052	.959	-.005	.953
2	MGRS0	-.098[c]	-1.190	.236	-.111	.981
	MRN0	-.180[c]	-1.894	.061	-.175	.716
	MRN0xRace	-.128[c]	-1.327	.187	-.124	.712
	MRN0xSES0	.062[c]	.723	.471	.068	.900
	MRN0xParty0	-.050[c]	-.534	.594	-.050	.770
	MRN0xMGRS0	.004[c]	.042	.967	.004	.791
	MGRS0xRace	-.038[c]	-.435	.665	-.041	.888
	MGRS0xSES0	-.036[c]	-.358	.721	-.034	.670
	MGRS0xParty0	-.030[c]	-.349	.728	-.033	.930
3	MRN0xRace	-.062[d]	-.559	.577	-.053	.529
	MRN0xSES0	.021[d]	.239	.812	.023	.823
	MRN0xParty0	-.087[d]	-.922	.359	-.087	.737
	MRN0xMGRS0	-.029[d]	-.253	.800	-.024	.493
	MGRS0xRace	-.017[d]	-.175	.861	-.017	.741
	MGRS0xSES0	-.101[d]	-.980	.329	-.093	.611
	MGRS0xParty0	-.076[d]	-.809	.420	-.077	.740

a. Dependent Variable: Using the scale provided, please indicate your personal opinion regarding how each of the following is handling the response to COVID-19 (Coronavirus) in the United States: - Nancy Pelosi

b. Predictors in the Model: (Constant), Party0

c. Predictors in the Model: (Constant), Party0, SES0, RaceCC

d. Predictors in the Model: (Constant), Party0, SES0, RaceCC, MGRS0, MRN0

```
REGRESSION
  /MISSING LISTWISE
  /STATISTICS COEFF OUTS R ANOVA CHANGE ZPP
  /CRITERIA=PIN(.05) POUT(.10)
  /NOORIGIN
  /DEPENDENT McConnellX
  /METHOD=ENTER Party0
  /METHOD=ENTER RaceCC SES0
  /METHOD=ENTER MGRS0 MRN0
  /METHOD=ENTER MRN0xRace MRN0xSES0 MRN0xParty0 MRN0xMGRS0 MGRS0xRace MGRS0xSES0 MGRS0xPa
rty0.
```

Regression

Notes

Output Created		15-DEC-2021 13:07:58
Comments		
Input	Data	C:\Users\njs5478\Dropbox\HM and COVID\0. Revise and Resubmit\2. R and R Data\Study 1b\Study1b_Data.sav
	Active Dataset	DataSet1
	Filter	GenderCC=1 (FILTER)
	Weight	<none>
	Split File	<none>
	N of Rows in Working Data File	119
Missing Value Handling	Definition of Missing	User-defined missing values are treated as missing.
	Cases Used	Statistics are based on cases with no missing values for any variable used.

Notes

Syntax		REGRESSION<br>/MISSING LISTWISE<br>/STATISTICS COEFF OUTS R ANOVA CHANGE ZPP<br>/CRITERIA=PIN(.05) POUT(.10)<br>/NOORIGIN<br>/DEPENDENT McConnellX<br>/METHOD=ENTER Party0<br>/METHOD=ENTER RaceCC SES0<br>/METHOD=ENTER MGRS0 MRN0<br>/METHOD=ENTER MRN0xRace MRN0xSES0 MRN0xParty0 MRN0xMGRS0 MGRS0xRace MGRS0xSES0 MGRS0xParty0.
Resources	Processor Time	00:00:00.02
	Elapsed Time	00:00:00.02
	Memory Required	48560 bytes
	Additional Memory Required for Residual Plots	0 bytes

Variables Entered/Removed[a]

Model	Variables Entered	Variables Removed	Method
1	Party0[b]	.	Enter
2	SES0, RaceCC[b]	.	Enter
3	MGRS0, MRN0[b]	.	Enter
4	MRN0xSES0, MRN0xParty0, MGRS0xRace, MGRS0xParty0, MGRS0xSES0, MRN0xMGRS0, ...[b]	.	Enter

a. Dependent Variable: McConnellX

b. All requested variables entered.

Model Summary

Model	R	R Square	Adjusted R Square	Std. Error of the Estimate	Change Statistics		
					R Square Change	F Change	df1
1	.635[a]	.404	.397	1.29316	.404	66.304	1
2	.657[b]	.432	.414	1.27502	.028	2.404	2
3	.706[c]	.498	.471	1.21142	.066	6.173	2
4	.718[d]	.516	.449	1.23652	.018	.460	7

Model Summary

Model	Change Statistics	
	df2	Sig. F Change
1	98	.000
2	96	.096
3	94	.003
4	87	.861

a. Predictors: (Constant), Party0

b. Predictors: (Constant), Party0, SES0, RaceCC

c. Predictors: (Constant), Party0, SES0, RaceCC, MGRS0, MRN0

d. Predictors: (Constant), Party0, SES0, RaceCC, MGRS0, MRN0, MRN0xSES0, MRN0xParty0, MGRS0xRace, MGRS0xParty0, MGRS0xSES0, MRN0xMGRS0, MRN0xRace

ANOVA[a]

Model		Sum of Squares	df	Mean Square	F	Sig.
1	Regression	110.878	1	110.878	66.304	.000[b]
	Residual	163.882	98	1.672		
	Total	274.760	99			
2	Regression	118.695	3	39.565	24.337	.000[c]
	Residual	156.065	96	1.626		
	Total	274.760	99			
3	Regression	136.812	5	27.362	18.645	.000[d]
	Residual	137.948	94	1.468		
	Total	274.760	99			
4	Regression	141.739	12	11.812	7.725	.000[e]
	Residual	133.021	87	1.529		
	Total	274.760	99			

a. Dependent Variable: McConnellX

b. Predictors: (Constant), Party0

c. Predictors: (Constant), Party0, SES0, RaceCC

d. Predictors: (Constant), Party0, SES0, RaceCC, MGRS0, MRN0

e. Predictors: (Constant), Party0, SES0, RaceCC, MGRS0, MRN0, MRN0xSES0, MRN0xParty0, MGRS0xRace, MGRS0xParty0, MGRS0xSES0, MRN0xMGRS0, MRN0xRace

Coefficients[a]

Model		Unstandardized Coefficients B	Unstandardized Coefficients Std. Error	Standardized Coefficients Beta	t	Sig.
1	(Constant)	2.941	.133		22.184	.000
	Party0	.795	.098	.635	8.143	.000
2	(Constant)	2.750	.157		17.474	.000
	Party0	.750	.099	.599	7.610	.000
	RaceCC	.346	.160	.170	2.157	.034
	SES0	.081	.148	.042	.550	.584
3	(Constant)	2.804	.175		16.054	.000
	Party0	.614	.112	.490	5.467	.000
	RaceCC	.410	.155	.201	2.645	.010
	SES0	.035	.142	.018	.248	.804
	MGRS0	.571	.238	.195	2.402	.018
	MRN0	.258	.185	.134	1.395	.166
4	(Constant)	2.854	.255		11.174	.000
	Party0	.572	.131	.457	4.364	.000
	RaceCC	.264	.222	.130	1.192	.237
	SES0	.048	.213	.025	.224	.823
	MGRS0	.630	.406	.215	1.552	.124
	MRN0	.055	.305	.029	.181	.856
	MRN0xRace	.354	.271	.192	1.303	.196
	MRN0xSES0	-.045	.200	-.024	-.225	.823
	MRN0xParty0	.036	.120	.029	.298	.767
	MRN0xMGRS0	.278	.370	.105	.751	.455
	MGRS0xRace	-.145	.368	-.052	-.395	.694
	MGRS0xSES0	-.092	.384	-.030	-.240	.811
	MGRS0xParty0	-.133	.256	-.067	-.521	.604

Coefficients[a]

Model		Correlations Zero-order	Partial	Part
1	(Constant)			
	Party0	.635	.635	.635
2	(Constant)			
	Party0	.635	.613	.585
	RaceCC	.293	.215	.166
	SES0	.040	.056	.042
3	(Constant)			
	Party0	.635	.491	.400
	RaceCC	.293	.263	.193
	SES0	.040	.026	.018
	MGRS0	.329	.240	.176
	MRN0	.469	.142	.102
4	(Constant)			
	Party0	.635	.424	.326
	RaceCC	.293	.127	.089
	SES0	.040	.024	.017
	MGRS0	.329	.164	.116
	MRN0	.469	.019	.014
	MRN0xRace	.502	.138	.097
	MRN0xSES0	-.215	-.024	-.017
	MRN0xParty0	.251	.032	.022
	MRN0xMGRS0	-.143	.080	.056
	MGRS0xRace	.102	-.042	-.029
	MGRS0xSES0	-.195	-.026	-.018
	MGRS0xParty0	-.115	-.056	-.039

a. Dependent Variable: McConnellX

Excluded Variables[a]

Model		Beta In	t	Sig.	Partial Correlation	Collinearity Statistics Tolerance
1	RaceCC	.167[b]	2.130	.036	.211	.956
	SES0	.031[b]	.390	.697	.040	1.000
	MGRS0	.230[b]	3.033	.003	.294	.973
	MRN0	.186[b]	2.065	.042	.205	.723
	MRN0xRace	.236[b]	2.664	.009	.261	.730
	MRN0xSES0	-.084[b]	-1.053	.295	-.106	.955
	MRN0xParty0	.010[b]	.118	.906	.012	.855
	MRN0xMGRS0	.055[b]	.672	.503	.068	.908
	MGRS0xRace	.060[b]	.762	.448	.077	.995
	MGRS0xSES0	-.077[b]	-.964	.338	-.097	.964
	MGRS0xParty0	.016[b]	.197	.845	.020	.958
2	MGRS0	.240[c]	3.209	.002	.313	.967
	MRN0	.226[c]	2.502	.014	.249	.688
	MRN0xRace	.199[c]	2.170	.033	.217	.677
	MRN0xSES0	-.109[c]	-1.372	.173	-.139	.921
	MRN0xParty0	-.012[c]	-.141	.888	-.014	.781
	MRN0xMGRS0	.072[c]	.850	.398	.087	.822
	MGRS0xRace	.122[c]	1.497	.138	.152	.886
	MGRS0xSES0	-.108[c]	-1.171	.245	-.119	.692
	MGRS0xParty0	.023[c]	.284	.777	.029	.924
3	MRN0xRace	.143[d]	1.282	.203	.132	.426
	MRN0xSES0	-.050[d]	-.632	.529	-.065	.867
	MRN0xParty0	.046[d]	.538	.592	.056	.742
	MRN0xMGRS0	.049[d]	.489	.626	.051	.536
	MGRS0xRace	.000[d]	-.002	.998	.000	.639
	MGRS0xSES0	-.026[d]	-.281	.780	-.029	.637
	MGRS0xParty0	-.020[d]	-.223	.824	-.023	.677

a. Dependent Variable: McConnellX

b. Predictors in the Model: (Constant), Party0

c. Predictors in the Model: (Constant), Party0, SES0, RaceCC

d. Predictors in the Model: (Constant), Party0, SES0, RaceCC, MGRS0, MRN0

```
REGRESSION
  /MISSING LISTWISE
  /STATISTICS COEFF OUTS R ANOVA CHANGE ZPP
  /CRITERIA=PIN(.05) POUT(.10)
  /NOORIGIN
  /DEPENDENT FauciX
  /METHOD=ENTER Party0
  /METHOD=ENTER RaceCC SES0
  /METHOD=ENTER MGRS0 MRN0
  /METHOD=ENTER MRN0xRace MRN0xSES0 MRN0xParty0 MRN0xMGRS0 MGRS0xRace MGRS0xSES0 MGRS0xPa
rty0.
```

Regression

Notes

Output Created		15-DEC-2021 13:07:58
Comments		
Input	Data	C:\Users\njs5478\Dropbox\HM and COVID\0. Revise and Resubmit\2. R and R Data\Study 1b\Study1b_Data.sav
	Active Dataset	DataSet1
	Filter	GenderCC=1 (FILTER)
	Weight	<none>
	Split File	<none>
	N of Rows in Working Data File	119
Missing Value Handling	Definition of Missing	User-defined missing values are treated as missing.
	Cases Used	Statistics are based on cases with no missing values for any variable used.

Notes

Syntax		REGRESSION<br>/MISSING LISTWISE<br>/STATISTICS COEFF OUTS R ANOVA CHANGE ZPP<br>/CRITERIA=PIN(.05) POUT(.10)<br>/NOORIGIN<br>/DEPENDENT FauciX<br>/METHOD=ENTER Party0<br>/METHOD=ENTER RaceCC SES0<br>/METHOD=ENTER MGRS0 MRN0<br>/METHOD=ENTER MRN0xRace MRN0xSES0 MRN0xParty0 MRN0xMGRS0 MGRS0xRace MGRS0xSES0 MGRS0xParty0.
Resources	Processor Time	00:00:00.02
	Elapsed Time	00:00:00.03
	Memory Required	48560 bytes
	Additional Memory Required for Residual Plots	0 bytes

Variables Entered/Removed[a]

Model	Variables Entered	Variables Removed	Method
1	Party0[b]	.	Enter
2	SES0, RaceCC[b]	.	Enter
3	MGRS0, MRN0[b]	.	Enter
4	MRN0xSES0, MGRS0xRace, MRN0xParty0, MGRS0xParty0, MGRS0xSES0, MRN0xMGRS0, ...[b]	.	Enter

a. Dependent Variable: FauciX

b. All requested variables entered.

Model Summary

Model	R	R Square	Adjusted R Square	Std. Error of the Estimate	Change Statistics		
					R Square Change	F Change	df1
1	.385[a]	.148	.140	1.64089	.148	18.305	1
2	.390[b]	.152	.127	1.65340	.003	.208	2
3	.472[c]	.223	.185	1.59795	.071	4.636	2
4	.504[d]	.254	.159	1.62285	.031	.561	7

Model Summary

Model	Change Statistics	
	df2	Sig. F Change
1	105	.000
2	103	.812
3	101	.012
4	94	.786

a. Predictors: (Constant), Party0

b. Predictors: (Constant), Party0, SES0, RaceCC

c. Predictors: (Constant), Party0, SES0, RaceCC, MGRS0, MRN0

d. Predictors: (Constant), Party0, SES0, RaceCC, MGRS0, MRN0, MRN0xSES0, MGRS0xRace, MRN0xParty0, MGRS0xParty0, MGRS0xSES0, MRN0xMGRS0, MRN0xRace

ANOVA[a]

Model		Sum of Squares	df	Mean Square	F	Sig.
1	Regression	49.286	1	49.286	18.305	.000[b]
	Residual	282.714	105	2.693		
	Total	332.000	106			
2	Regression	50.426	3	16.809	6.149	.001[c]
	Residual	281.574	103	2.734		
	Total	332.000	106			
3	Regression	74.101	5	14.820	5.804	.000[d]
	Residual	257.899	101	2.553		
	Total	332.000	106			
4	Regression	84.439	12	7.037	2.672	.004[e]
	Residual	247.561	94	2.634		
	Total	332.000	106			

a. Dependent Variable: FauciX

b. Predictors: (Constant), Party0

c. Predictors: (Constant), Party0, SES0, RaceCC

d. Predictors: (Constant), Party0, SES0, RaceCC, MGRS0, MRN0

e. Predictors: (Constant), Party0, SES0, RaceCC, MGRS0, MRN0, MRN0xSES0, MGRS0xRace, MRN0xParty0, MGRS0xParty0, MGRS0xSES0, MRN0xMGRS0, MRN0xRace

Coefficients[a]

Model		Unstandardized Coefficients B	Std. Error	Standardized Coefficients Beta	t	Sig.
1	(Constant)	5.200	.165		31.444	.000
	Party0	-.513	.120	-.385	-4.278	.000
2	(Constant)	5.145	.189		27.231	.000
	Party0	-.534	.126	-.401	-4.235	.000
	RaceCC	.112	.195	.055	.575	.567
	SES0	.065	.189	.031	.342	.733
3	(Constant)	5.267	.220		23.952	.000
	Party0	-.368	.142	-.276	-2.589	.011
	RaceCC	.004	.193	.002	.023	.982
	SES0	.104	.184	.050	.565	.573
	MGRS0	-.385	.297	-.124	-1.295	.198
	MRN0	-.484	.240	-.224	-2.016	.046
4	(Constant)	5.369	.318		16.883	.000
	Party0	-.365	.174	-.274	-2.103	.038
	RaceCC	.012	.280	.006	.043	.965
	SES0	-.168	.275	-.081	-.611	.543
	MGRS0	-.060	.510	-.019	-.117	.907
	MRN0	-.713	.379	-.329	-1.884	.063
	MRN0xRace	-.076	.325	-.039	-.235	.815
	MRN0xSES0	.219	.260	.108	.843	.401
	MRN0xParty0	.056	.152	.044	.369	.713
	MRN0xMGRS0	-.102	.452	-.037	-.225	.822
	MGRS0xRace	-.145	.445	-.048	-.326	.745
	MGRS0xSES0	-.667	.458	-.203	-1.455	.149
	MGRS0xParty0	-.263	.304	-.124	-.866	.389

Coefficients[a]

Model		Correlations		
		Zero-order	Partial	Part
1	(Constant)			
	Party0	-.385	-.385	-.385
2	(Constant)			
	Party0	-.385	-.385	-.384
	RaceCC	-.062	.057	.052
	SES0	.025	.034	.031
3	(Constant)			
	Party0	-.385	-.249	-.227
	RaceCC	-.062	.002	.002
	SES0	.025	.056	.050
	MGRS0	-.223	-.128	-.114
	MRN0	-.393	-.197	-.177
4	(Constant)			
	Party0	-.385	-.212	-.187
	RaceCC	-.062	.004	.004
	SES0	.025	-.063	-.054
	MGRS0	-.223	-.012	-.010
	MRN0	-.393	-.191	-.168
	MRN0xRace	-.308	-.024	-.021
	MRN0xSES0	.117	.087	.075
	MRN0xParty0	-.145	.038	.033
	MRN0xMGRS0	.067	-.023	-.020
	MGRS0xRace	-.147	-.034	-.029
	MGRS0xSES0	.032	-.148	-.130
	MGRS0xParty0	-.008	-.089	-.077

a. Dependent Variable: FauciX

Excluded Variables[a]

Model		Beta In	t	Sig.	Partial Correlation	Collinearity Statistics Tolerance
1	RaceCC	.052[b]	.550	.583	.054	.919
	SES0	.027[b]	.295	.769	.029	1.000
	MGRS0	-.204[b]	-2.304	.023	-.220	.997
	MRN0	-.274[b]	-2.778	.006	-.263	.786
	MRN0xRace	-.153[b]	-1.480	.142	-.144	.748
	MRN0xSES0	.058[b]	.632	.529	.062	.975
	MRN0xParty0	.073[b]	.689	.492	.067	.735
	MRN0xMGRS0	-.060[b]	-.627	.532	-.061	.902
	MGRS0xRace	-.134[b]	-1.492	.139	-.145	.999
	MGRS0xSES0	-.026[b]	-.289	.773	-.028	.977
	MGRS0xParty0	-.053[b]	-.581	.563	-.057	.987
2	MGRS0	-.201[c]	-2.249	.027	-.217	.992
	MRN0	-.281[c]	-2.747	.007	-.262	.742
	MRN0xRace	-.203[c]	-1.824	.071	-.178	.651
	MRN0xSES0	.051[c]	.529	.598	.052	.908
	MRN0xParty0	.074[c]	.680	.498	.067	.693
	MRN0xMGRS0	-.054[c]	-.533	.595	-.053	.814
	MGRS0xRace	-.135[c]	-1.429	.156	-.140	.918
	MGRS0xSES0	-.028[c]	-.260	.796	-.026	.710
	MGRS0xParty0	-.051[c]	-.546	.586	-.054	.962
3	MRN0xRace	-.100[d]	-.770	.443	-.077	.462
	MRN0xSES0	-.005[d]	-.057	.954	-.006	.844
	MRN0xParty0	.006[d]	.056	.955	.006	.661
	MRN0xMGRS0	-.049[d]	-.401	.689	-.040	.510
	MGRS0xRace	-.096[d]	-.942	.348	-.094	.744
	MGRS0xSES0	-.125[d]	-1.154	.251	-.115	.656
	MGRS0xParty0	-.098[d]	-.946	.346	-.094	.721

a. Dependent Variable: FauciX

b. Predictors in the Model: (Constant), Party0

c. Predictors in the Model: (Constant), Party0, SES0, RaceCC

d. Predictors in the Model: (Constant), Party0, SES0, RaceCC, MGRS0, MRN0

```
REGRESSION
  /MISSING LISTWISE
  /STATISTICS COEFF OUTS R ANOVA CHANGE ZPP
  /CRITERIA=PIN(.05) POUT(.10)
  /NOORIGIN
  /DEPENDENT RepCongressX
  /METHOD=ENTER Party0
  /METHOD=ENTER RaceCC SES0
  /METHOD=ENTER MGRS0 MRN0
  /METHOD=ENTER MRN0xRace MRN0xSES0 MRN0xParty0 MRN0xMGRS0 MGRS0xRace MGRS0xSES0 MGRS0xPa
rty0.
```

Regression

Notes

Output Created		15-DEC-2021 13:07:58
Comments		
Input	Data	C:\Users\njs5478\Dropbox\HM and COVID\0. Revise and Resubmit\2. R and R Data\Study 1b\Study1b_Data.sav
	Active Dataset	DataSet1
	Filter	GenderCC=1 (FILTER)
	Weight	<none>
	Split File	<none>
	N of Rows in Working Data File	119
Missing Value Handling	Definition of Missing	User-defined missing values are treated as missing.
	Cases Used	Statistics are based on cases with no missing values for any variable used.

Notes

Syntax		REGRESSION<br>/MISSING LISTWISE<br>/STATISTICS COEFF OUTS R ANOVA CHANGE ZPP<br>/CRITERIA=PIN(.05) POUT(.10)<br>/NOORIGIN<br>/DEPENDENT RepCongressX<br>/METHOD=ENTER Party0<br>/METHOD=ENTER RaceCC SES0<br>/METHOD=ENTER MGRS0 MRN0<br>/METHOD=ENTER MRN0xRace MRN0xSES0 MRN0xParty0 MRN0xMGRS0 MGRS0xRace MGRS0xSES0 MGRS0xParty0.
Resources	Processor Time	00:00:00.08
	Elapsed Time	00:00:00.03
	Memory Required	48560 bytes
	Additional Memory Required for Residual Plots	0 bytes

Variables Entered/Removed[a]

Model	Variables Entered	Variables Removed	Method
1	Party0[b]	.	Enter
2	SES0, RaceCC[b]	.	Enter
3	MGRS0, MRN0[b]	.	Enter
4	MRN0xSES0, MGRS0xRace, MRN0xParty0, MGRS0xParty0, MGRS0xSES0, MRN0xRace, MRN0xMGRS...[b]	.	Enter

a. Dependent Variable: RepCongressX

b. All requested variables entered.

Model Summary

Model	R	R Square	Adjusted R Square	Std. Error of the Estimate	Change Statistics		
					R Square Change	F Change	df1
1	.694[a]	.481	.476	1.39017	.481	99.255	1
2	.738[b]	.545	.532	1.31398	.064	7.385	2
3	.750[c]	.563	.541	1.30096	.017	2.055	2
4	.764[d]	.584	.532	1.31485	.021	.691	7

Model Summary

Model	Change Statistics	
	df2	Sig. F Change
1	107	.000
2	105	.001
3	103	.133
4	96	.680

a. Predictors: (Constant), Party0

b. Predictors: (Constant), Party0, SES0, RaceCC

c. Predictors: (Constant), Party0, SES0, RaceCC, MGRS0, MRN0

d. Predictors: (Constant), Party0, SES0, RaceCC, MGRS0, MRN0, MRN0xSES0, MGRS0xRace, MRN0xParty0, MGRS0xParty0, MGRS0xSES0, MRN0xRace, MRN0xMGRS0

ANOVA[a]

Model		Sum of Squares	df	Mean Square	F	Sig.
1	Regression	191.819	1	191.819	99.255	.000[b]
	Residual	206.786	107	1.933		
	Total	398.606	108			
2	Regression	217.319	3	72.440	41.957	.000[c]
	Residual	181.286	105	1.727		
	Total	398.606	108			
3	Regression	224.277	5	44.855	26.502	.000[d]
	Residual	174.328	103	1.693		
	Total	398.606	108			
4	Regression	232.637	12	19.386	11.213	.000[e]
	Residual	165.969	96	1.729		
	Total	398.606	108			

a. Dependent Variable: RepCongressX

b. Predictors: (Constant), Party0

c. Predictors: (Constant), Party0, SES0, RaceCC

d. Predictors: (Constant), Party0, SES0, RaceCC, MGRS0, MRN0

e. Predictors: (Constant), Party0, SES0, RaceCC, MGRS0, MRN0, MRN0xSES0, MGRS0xRace, MRN0xParty0, MGRS0xParty0, MGRS0xSES0, MRN0xRace, MRN0xMGRS0

Coefficients[a]

Model		Unstandardized Coefficients B	Unstandardized Coefficients Std. Error	Standardized Coefficients Beta	t	Sig.
1	(Constant)	3.367	.137		24.493	.000
	Party0	.990	.099	.694	9.963	.000
2	(Constant)	3.092	.148		20.856	.000
	Party0	.886	.098	.621	9.060	.000
	RaceCC	.592	.154	.264	3.842	.000
	SES0	.056	.142	.026	.393	.695
3	(Constant)	3.043	.176		17.250	.000
	Party0	.783	.115	.549	6.805	.000
	RaceCC	.645	.155	.288	4.155	.000
	SES0	.029	.141	.013	.203	.839
	MGRS0	.190	.245	.057	.773	.441
	MRN0	.255	.191	.116	1.337	.184
4	(Constant)	3.011	.251		11.989	.000
	Party0	.836	.138	.586	6.055	.000
	RaceCC	.492	.214	.219	2.303	.023
	SES0	.147	.217	.069	.679	.499
	MGRS0	.064	.404	.019	.158	.875
	MRN0	.280	.282	.127	.993	.323
	MRN0xRace	.262	.234	.127	1.120	.265
	MRN0xSES0	-.096	.184	-.049	-.518	.605
	MRN0xParty0	-.107	.128	-.076	-.833	.407
	MRN0xMGRS0	.201	.356	.068	.566	.573
	MGRS0xRace	-.438	.362	-.136	-1.208	.230
	MGRS0xSES0	.056	.364	.016	.155	.878
	MGRS0xParty0	.331	.243	.146	1.364	.176

Coefficients[a]

Model		Correlations Zero-order	Partial	Part
1	(Constant)			
	Party0	.694	.694	.694
2	(Constant)			
	Party0	.694	.662	.596
	RaceCC	.432	.351	.253
	SES0	.024	.038	.026
3	(Constant)			
	Party0	.694	.557	.443
	RaceCC	.432	.379	.271
	SES0	.024	.020	.013
	MGRS0	.169	.076	.050
	MRN0	.414	.131	.087
4	(Constant)			
	Party0	.694	.526	.399
	RaceCC	.432	.229	.152
	SES0	.024	.069	.045
	MGRS0	.169	.016	.010
	MRN0	.414	.101	.065
	MRN0xRace	.456	.114	.074
	MRN0xSES0	-.212	-.053	-.034
	MRN0xParty0	.301	-.085	-.055
	MRN0xMGRS0	-.195	.058	.037
	MGRS0xRace	-.034	-.122	-.080
	MGRS0xSES0	-.138	.016	.010
	MGRS0xParty0	-.064	.138	.090

a. Dependent Variable: RepCongressX

Excluded Variables[a]

Model		Beta In	t	Sig.	Partial Correlation	Collinearity Statistics Tolerance
1	RaceCC	.262[b]	3.838	.000	.349	.925
	SES0	.005[b]	.066	.948	.006	.999
	MGRS0	.081[b]	1.150	.253	.111	.983
	MRN0	.085[b]	1.049	.297	.101	.744
	MRN0xRace	.155[b]	1.977	.051	.189	.764
	MRN0xSES0	-.046[b]	-.638	.525	-.062	.941
	MRN0xParty0	.002[b]	.022	.983	.002	.814
	MRN0xMGRS0	.052[b]	.703	.483	.068	.880
	MGRS0xRace	-.085[b]	-1.215	.227	-.117	.995
	MGRS0xSES0	-.018[b]	-.249	.804	-.024	.970
	MGRS0xParty0	.057[b]	.810	.420	.078	.970
2	MGRS0	.101[c]	1.518	.132	.147	.976
	MRN0	.145[c]	1.878	.063	.181	.708
	MRN0xRace	.084[c]	1.073	.286	.105	.709
	MRN0xSES0	-.063[c]	-.898	.371	-.088	.879
	MRN0xParty0	-.036[c]	-.474	.636	-.046	.737
	MRN0xMGRS0	.070[c]	.946	.346	.092	.796
	MGRS0xRace	-.011[c]	-.151	.880	-.015	.891
	MGRS0xSES0	-.069[c]	-.859	.392	-.084	.674
	MGRS0xParty0	.050[c]	.741	.461	.072	.944
3	MRN0xRace	.035[d]	.404	.687	.040	.557
	MRN0xSES0	-.024[d]	-.329	.743	-.033	.800
	MRN0xParty0	.005[d]	.061	.951	.006	.685
	MRN0xMGRS0	.113[d]	1.242	.217	.122	.507
	MGRS0xRace	-.046[d]	-.605	.547	-.060	.737
	MGRS0xSES0	-.020[d]	-.241	.810	-.024	.607
	MGRS0xParty0	.091[d]	1.179	.241	.116	.714

a. Dependent Variable: RepCongressX

b. Predictors in the Model: (Constant), Party0

c. Predictors in the Model: (Constant), Party0, SES0, RaceCC

d. Predictors in the Model: (Constant), Party0, SES0, RaceCC, MGRS0, MRN0

```
REGRESSION
  /MISSING LISTWISE
  /STATISTICS COEFF OUTS R ANOVA CHANGE ZPP
  /CRITERIA=PIN(.05) POUT(.10)
  /NOORIGIN
  /DEPENDENT DemCongressX
  /METHOD=ENTER Party0
  /METHOD=ENTER RaceCC SES0
  /METHOD=ENTER MGRS0 MRN0
  /METHOD=ENTER MRN0xRace MRN0xSES0 MRN0xParty0 MRN0xMGRS0 MGRS0xRace MGRS0xSES0 MGRS0xPa
rty0.
```

Regression

Notes

Output Created		15-DEC-2021 13:07:58
Comments		
Input	Data	C: \Users\njs5478\Dropbox\HM and COVID\0. Revise and Resubmit\2. R and R Data\Study 1b\Study1b_Data.sav
	Active Dataset	DataSet1
	Filter	GenderCC=1 (FILTER)
	Weight	<none>
	Split File	<none>
	N of Rows in Working Data File	119
Missing Value Handling	Definition of Missing	User-defined missing values are treated as missing.
	Cases Used	Statistics are based on cases with no missing values for any variable used.

Notes

Syntax		REGRESSION<br>/MISSING LISTWISE<br>/STATISTICS COEFF OUTS R ANOVA CHANGE ZPP<br>/CRITERIA=PIN(.05) POUT(.10)<br>/NOORIGIN<br>/DEPENDENT DemCongressX<br>/METHOD=ENTER Party0<br>/METHOD=ENTER RaceCC SES0<br>/METHOD=ENTER MGRS0 MRN0<br>/METHOD=ENTER MRN0xRace MRN0xSES0 MRN0xParty0 MRN0xMGRS0 MGRS0xRace MGRS0xSES0 MGRS0xParty0.
Resources	Processor Time	00:00:00.02
	Elapsed Time	00:00:00.02
	Memory Required	48560 bytes
	Additional Memory Required for Residual Plots	0 bytes

Variables Entered/Removed[a]

Model	Variables Entered	Variables Removed	Method
1	Party0[b]	.	Enter
2	SES0, RaceCC[b]	.	Enter
3	MGRS0, MRN0[b]	.	Enter
4	MRN0xSES0, MGRS0xRace , MRN0xParty0 , MGRS0xParty 0, MGRS0xSES 0, MRN0xRace, MRN0xMGRS...[b]	.	Enter

a. Dependent Variable: DemCongressX

b. All requested variables entered.

Model Summary

Model	R	R Square	Adjusted R Square	Std. Error of the Estimate	Change Statistics		
					R Square Change	F Change	df1
1	.644[a]	.415	.410	1.28888	.415	74.533	1
2	.647[b]	.419	.402	1.29742	.004	.311	2
3	.679[c]	.461	.435	1.26134	.043	3.988	2
4	.685[d]	.469	.401	1.29796	.008	.197	7

Model Summary

Model	Change Statistics	
	df2	Sig. F Change
1	105	.000
2	103	.733
3	101	.022
4	94	.985

a. Predictors: (Constant), Party0

b. Predictors: (Constant), Party0, SES0, RaceCC

c. Predictors: (Constant), Party0, SES0, RaceCC, MGRS0, MRN0

d. Predictors: (Constant), Party0, SES0, RaceCC, MGRS0, MRN0, MRN0xSES0, MGRS0xRace, MRN0xParty0, MGRS0xParty0, MGRS0xSES0, MRN0xRace, MRN0xMGRS0

ANOVA[a]

Model		Sum of Squares	df	Mean Square	F	Sig.
1	Regression	123.815	1	123.815	74.533	.000[b]
	Residual	174.428	105	1.661		
	Total	298.243	106			
2	Regression	124.862	3	41.621	24.726	.000[c]
	Residual	173.381	103	1.683		
	Total	298.243	106			
3	Regression	137.554	5	27.511	17.292	.000[d]
	Residual	160.689	101	1.591		
	Total	298.243	106			
4	Regression	139.881	12	11.657	6.919	.000[e]
	Residual	158.362	94	1.685		
	Total	298.243	106			

a. Dependent Variable: DemCongressX

b. Predictors: (Constant), Party0

c. Predictors: (Constant), Party0, SES0, RaceCC

d. Predictors: (Constant), Party0, SES0, RaceCC, MGRS0, MRN0

e. Predictors: (Constant), Party0, SES0, RaceCC, MGRS0, MRN0, MRN0xSES0, MGRS0xRace, MRN0xParty0, MGRS0xParty0, MGRS0xSES0, MRN0xRace, MRN0xMGRS0

Coefficients[a]

Model		Unstandardized Coefficients B	Std. Error	Standardized Coefficients Beta	t	Sig.
1	(Constant)	4.206	.129		32.591	.000
	Party0	-.803	.093	-.644	-8.633	.000
2	(Constant)	4.225	.147		28.753	.000
	Party0	-.794	.098	-.637	-8.120	.000
	RaceCC	-.041	.153	-.021	-.267	.790
	SES0	-.107	.140	-.057	-.762	.448
3	(Constant)	4.448	.173		25.732	.000
	Party0	-.628	.112	-.504	-5.624	.000
	RaceCC	-.113	.151	-.058	-.748	.456
	SES0	-.068	.137	-.036	-.492	.624
	MGRS0	.196	.238	.068	.822	.413
	MRN0	-.524	.188	-.267	-2.785	.006
4	(Constant)	4.469	.249		17.961	.000
	Party0	-.658	.138	-.528	-4.765	.000
	RaceCC	-.210	.212	-.108	-.991	.324
	SES0	-.083	.215	-.045	-.386	.700
	MGRS0	.321	.401	.111	.802	.425
	MRN0	-.576	.280	-.294	-2.055	.043
	MRN0xRace	.127	.234	.070	.542	.589
	MRN0xSES0	.054	.182	.032	.298	.767
	MRN0xParty0	.053	.131	.043	.400	.690
	MRN0xMGRS0	-.081	.353	-.031	-.228	.820
	MGRS0xRace	-.241	.358	-.087	-.673	.503
	MGRS0xSES0	-.116	.361	-.039	-.321	.749
	MGRS0xParty0	.131	.241	.066	.541	.589

Coefficients[a]

Model		Correlations		
		Zero-order	Partial	Part
1	(Constant)			
	Party0	-.644	-.644	-.644
2	(Constant)			
	Party0	-.644	-.625	-.610
	RaceCC	-.198	-.026	-.020
	SES0	-.070	-.075	-.057
3	(Constant)			
	Party0	-.644	-.488	-.411
	RaceCC	-.198	-.074	-.055
	SES0	-.070	-.049	-.036
	MGRS0	-.115	.081	.060
	MRN0	-.487	-.267	-.203
4	(Constant)			
	Party0	-.644	-.441	-.358
	RaceCC	-.198	-.102	-.074
	SES0	-.070	-.040	-.029
	MGRS0	-.115	.082	.060
	MRN0	-.487	-.207	-.154
	MRN0xRace	-.371	.056	.041
	MRN0xSES0	.190	.031	.022
	MRN0xParty0	-.207	.041	.030
	MRN0xMGRS0	.327	-.024	-.017
	MGRS0xRace	-.042	-.069	-.051
	MGRS0xSES0	.166	-.033	-.024
	MGRS0xParty0	.238	.056	.041

a. Dependent Variable: DemCongressX

Excluded Variables[a]

Model		Beta In	t	Sig.	Partial Correlation	Collinearity Statistics Tolerance
1	RaceCC	-.016[b]	-.204	.839	-.020	.919
	SES0	-.056[b]	-.746	.458	-.073	1.000
	MGRS0	-.035[b]	-.466	.642	-.046	.985
	MRN0	-.225[b]	-2.703	.008	-.256	.758
	MRN0xRace	-.086[b]	-1.018	.311	-.099	.777
	MRN0xSES0	.039[b]	.502	.617	.049	.943
	MRN0xParty0	.115[b]	1.368	.174	.133	.788
	MRN0xMGRS0	.123[b]	1.559	.122	.151	.885
	MGRS0xRace	.000[b]	.005	.996	.001	.996
	MGRS0xSES0	.057[b]	.753	.453	.074	.971
	MGRS0xParty0	.136[b]	1.823	.071	.176	.973
2	MGRS0	-.035[c]	-.457	.649	-.045	.978
	MRN0	-.232[c]	-2.707	.008	-.259	.725
	MRN0xRace	-.087[c]	-.982	.328	-.097	.714
	MRN0xSES0	.058[c]	.720	.473	.071	.882
	MRN0xParty0	.110[c]	1.238	.219	.122	.717
	MRN0xMGRS0	.115[c]	1.374	.172	.135	.803
	MGRS0xRace	.001[c]	.016	.987	.002	.893
	MGRS0xSES0	.042[c]	.460	.646	.046	.674
	MGRS0xParty0	.131[c]	1.720	.089	.168	.948
3	MRN0xRace	.026[d]	.272	.787	.027	.573
	MRN0xSES0	.019[d]	.229	.819	.023	.803
	MRN0xParty0	.071[d]	.801	.425	.080	.674
	MRN0xMGRS0	.039[d]	.377	.707	.038	.511
	MGRS0xRace	-.020[d]	-.238	.812	-.024	.738
	MGRS0xSES0	-.027[d]	-.291	.772	-.029	.603
	MGRS0xParty0	.061[d]	.704	.483	.070	.717

a. Dependent Variable: DemCongressX

b. Predictors in the Model: (Constant), Party0

c. Predictors in the Model: (Constant), Party0, SES0, RaceCC

d. Predictors in the Model: (Constant), Party0, SES0, RaceCC, MGRS0, MRN0

```
 REGRESSION
  /MISSING LISTWISE
  /STATISTICS COEFF OUTS R ANOVA CHANGE ZPP
  /CRITERIA=PIN(.05) POUT(.10)
  /NOORIGIN
  /DEPENDENT StateX
  /METHOD=ENTER Party0
  /METHOD=ENTER RaceCC SES0
  /METHOD=ENTER MGRS0 MRN0
  /METHOD=ENTER MRN0xRace MRN0xSES0 MRN0xParty0 MRN0xMGRS0 MGRS0xRace MGRS0xSES0 MGRS0xPa
rty0.
```

Regression

Notes

Output Created		15-DEC-2021 13:07:58
Comments		
Input	Data	C:\Users\njs5478\Dropbox\HM and COVID\0. Revise and Resubmit\2. R and R Data\Study 1b\Study1b_Data.sav
	Active Dataset	DataSet1
	Filter	GenderCC=1 (FILTER)
	Weight	<none>
	Split File	<none>
	N of Rows in Working Data File	119
Missing Value Handling	Definition of Missing	User-defined missing values are treated as missing.
	Cases Used	Statistics are based on cases with no missing values for any variable used.

Notes

Syntax		REGRESSION<br>/MISSING LISTWISE<br>/STATISTICS COEFF OUTS R ANOVA CHANGE ZPP<br>/CRITERIA=PIN(.05) POUT(.10)<br>/NOORIGIN<br>/DEPENDENT StateX<br>/METHOD=ENTER Party0<br>/METHOD=ENTER RaceCC SES0<br>/METHOD=ENTER MGRS0 MRN0<br>/METHOD=ENTER MRN0xRace MRN0xSES0 MRN0xParty0 MRN0xMGRS0 MGRS0xRace MGRS0xSES0 MGRS0xParty0.
Resources	Processor Time	00:00:00.03
	Elapsed Time	00:00:00.03
	Memory Required	48560 bytes
	Additional Memory Required for Residual Plots	0 bytes

Variables Entered/Removed[a]

Model	Variables Entered	Variables Removed	Method
1	Party0[b]	.	Enter
2	SES0, RaceCC[b]	.	Enter
3	MGRS0, MRN0[b]	.	Enter
4	MRN0xSES0, MGRS0xRace, MRN0xParty0, MGRS0xParty0, MGRS0xSES0, MRN0xRace, MRN0xMGRS...[b]	.	Enter

a. Dependent Variable: StateX

b. All requested variables entered.

Model Summary

Model	R	R Square	Adjusted R Square	Std. Error of the Estimate	Change Statistics		
					R Square Change	F Change	df1
1	.330[a]	.109	.101	1.62596	.109	13.690	1
2	.333[b]	.111	.087	1.63873	.002	.130	2
3	.362[c]	.131	.091	1.63487	.020	1.260	2
4	.403[d]	.163	.063	1.65972	.031	.541	7

Model Summary

Model	Change Statistics	
	df2	Sig. F Change
1	112	.000
2	110	.878
3	108	.288
4	101	.801

a. Predictors: (Constant), Party0

b. Predictors: (Constant), Party0, SES0, RaceCC

c. Predictors: (Constant), Party0, SES0, RaceCC, MGRS0, MRN0

d. Predictors: (Constant), Party0, SES0, RaceCC, MGRS0, MRN0, MRN0xSES0, MGRS0xRace, MRN0xParty0, MGRS0xParty0, MGRS0xSES0, MRN0xRace, MRN0xMGRS0

ANOVA[a]

Model		Sum of Squares	df	Mean Square	F	Sig.
1	Regression	36.192	1	36.192	13.690	.000[b]
	Residual	296.098	112	2.644		
	Total	332.289	113			
2	Regression	36.891	3	12.297	4.579	.005[c]
	Residual	295.399	110	2.685		
	Total	332.289	113			
3	Regression	43.626	5	8.725	3.264	.009[d]
	Residual	288.664	108	2.673		
	Total	332.289	113			
4	Regression	54.067	12	4.506	1.636	.093[e]
	Residual	278.222	101	2.755		
	Total	332.289	113			

a. Dependent Variable: StateX

b. Predictors: (Constant), Party0

c. Predictors: (Constant), Party0, SES0, RaceCC

d. Predictors: (Constant), Party0, SES0, RaceCC, MGRS0, MRN0

e. Predictors: (Constant), Party0, SES0, RaceCC, MGRS0, MRN0, MRN0xSES0, MGRS0xRace, MRN0xParty0, MGRS0xParty0, MGRS0xSES0, MRN0xRace, MRN0xMGRS0

Coefficients[a]

Model		Unstandardized Coefficients B	Std. Error	Standardized Coefficients Beta	t	Sig.
1	(Constant)	4.066	.157		25.860	.000
	Party0	-.419	.113	-.330	-3.700	.000
2	(Constant)	4.110	.181		22.670	.000
	Party0	-.402	.119	-.317	-3.381	.001
	RaceCC	-.095	.189	-.048	-.506	.614
	SES0	-.019	.175	-.010	-.109	.913
3	(Constant)	4.177	.216		19.349	.000
	Party0	-.292	.143	-.230	-2.038	.044
	RaceCC	-.158	.193	-.079	-.821	.413
	SES0	.009	.176	.005	.050	.960
	MGRS0	-.129	.302	-.043	-.427	.670
	MRN0	-.279	.232	-.143	-1.201	.232
4	(Constant)	4.098	.311		13.184	.000
	Party0	-.441	.167	-.347	-2.640	.010
	RaceCC	-.188	.265	-.094	-.711	.479
	SES0	.145	.269	.075	.539	.591
	MGRS0	-.055	.503	-.018	-.109	.913
	MRN0	-.230	.352	-.119	-.654	.514
	MRN0xRace	.002	.291	.001	.005	.996
	MRN0xSES0	-.090	.225	-.050	-.399	.691
	MRN0xParty0	.250	.148	.205	1.694	.093
	MRN0xMGRS0	-.129	.442	-.049	-.293	.770
	MGRS0xRace	.124	.453	.043	.275	.784
	MGRS0xSES0	.219	.452	.070	.484	.630
	MGRS0xParty0	.010	.301	.005	.034	.973

Coefficients[a]

Model		Correlations Zero-order	Partial	Part
1	(Constant)			
	Party0	-.330	-.330	-.330
2	(Constant)			
	Party0	-.330	-.307	-.304
	RaceCC	-.135	-.048	-.046
	SES0	-.012	-.010	-.010
3	(Constant)			
	Party0	-.330	-.192	-.183
	RaceCC	-.135	-.079	-.074
	SES0	-.012	.005	.004
	MGRS0	-.124	-.041	-.038
	MRN0	-.277	-.115	-.108
4	(Constant)			
	Party0	-.330	-.254	-.240
	RaceCC	-.135	-.071	-.065
	SES0	-.012	.054	.049
	MGRS0	-.124	-.011	-.010
	MRN0	-.277	-.065	-.060
	MRN0xRace	-.183	.001	.000
	MRN0xSES0	.103	-.040	-.036
	MRN0xParty0	.019	.166	.154
	MRN0xMGRS0	.137	-.029	-.027
	MGRS0xRace	.022	.027	.025
	MGRS0xSES0	.113	.048	.044
	MGRS0xParty0	.137	.003	.003

a. Dependent Variable: StateX

Excluded Variables[a]

Model		Beta In	t	Sig.	Partial Correlation	Collinearity Statistics Tolerance
1	RaceCC	-.047[b]	-.501	.618	-.047	.923
	SES0	-.006[b]	-.065	.948	-.006	1.000
	MGRS0	-.089[b]	-.993	.323	-.094	.988
	MRN0	-.145[b]	-1.397	.165	-.131	.734
	MRN0xRace	-.022[b]	-.207	.836	-.020	.744
	MRN0xSES0	.035[b]	.378	.706	.036	.955
	MRN0xParty0	.194[b]	1.994	.049	.186	.820
	MRN0xMGRS0	.020[b]	.209	.835	.020	.868
	MGRS0xRace	.040[b]	.452	.652	.043	.997
	MGRS0xSES0	.058[b]	.637	.526	.060	.970
	MGRS0xParty0	.073[b]	.803	.424	.076	.959
2	MGRS0	-.094[c]	-1.036	.303	-.099	.979
	MRN0	-.165[c]	-1.535	.128	-.145	.689
	MRN0xRace	-.009[c]	-.082	.935	-.008	.698
	MRN0xSES0	.040[c]	.420	.675	.040	.898
	MRN0xParty0	.216[c]	2.122	.036	.199	.758
	MRN0xMGRS0	.018[c]	.179	.858	.017	.781
	MGRS0xRace	.030[c]	.319	.750	.031	.892
	MGRS0xSES0	.087[c]	.787	.433	.075	.671
	MGRS0xParty0	.076[c]	.813	.418	.078	.932
3	MRN0xRace	.080[d]	.647	.519	.062	.531
	MRN0xSES0	.002[d]	.016	.988	.002	.822
	MRN0xParty0	.189[d]	1.803	.074	.172	.717
	MRN0xMGRS0	.005[d]	.038	.970	.004	.492
	MGRS0xRace	.065[d]	.614	.540	.059	.728
	MGRS0xSES0	.037[d]	.325	.746	.031	.610
	MGRS0xParty0	.064[d]	.601	.549	.058	.702

a. Dependent Variable: StateX

b. Predictors in the Model: (Constant), Party0

c. Predictors in the Model: (Constant), Party0, SES0, RaceCC

d. Predictors in the Model: (Constant), Party0, SES0, RaceCC, MGRS0, MRN0

```
REGRESSION
  /MISSING LISTWISE
  /STATISTICS COEFF OUTS R ANOVA CHANGE ZPP
  /CRITERIA=PIN(.05) POUT(.10)
  /NOORIGIN
  /DEPENDENT Risk_Rules
  /METHOD=ENTER Party0
  /METHOD=ENTER RaceCC SES0
  /METHOD=ENTER MGRS0 MRN0
  /METHOD=ENTER MRN0xRace MRN0xSES0 MRN0xParty0 MRN0xMGRS0 MGRS0xRace MGRS0xSES0 MGRS0xPa
rty0.
```

Regression

Notes

Output Created		15-DEC-2021 13:07:59
Comments		
Input	Data	C:\Users\njs5478\Dropbox\HM and COVID\0. Revise and Resubmit\2. R and R Data\Study 1b\Study1b_Data.sav
	Active Dataset	DataSet1
	Filter	GenderCC=1 (FILTER)
	Weight	<none>
	Split File	<none>
	N of Rows in Working Data File	119
Missing Value Handling	Definition of Missing	User-defined missing values are treated as missing.
	Cases Used	Statistics are based on cases with no missing values for any variable used.

Notes

Syntax		REGRESSION<br>/MISSING LISTWISE<br>/STATISTICS COEFF OUTS R ANOVA CHANGE ZPP<br>/CRITERIA=PIN(.05) POUT(.10)<br>/NOORIGIN<br>/DEPENDENT Risk_Rules<br>/METHOD=ENTER Party0<br>/METHOD=ENTER RaceCC SES0<br>/METHOD=ENTER MGRS0 MRN0<br>/METHOD=ENTER MRN0xRace MRN0xSES0 MRN0xParty0 MRN0xMGRS0 MGRS0xRace MGRS0xSES0 MGRS0xParty0.
Resources	Processor Time	00:00:00.03
	Elapsed Time	00:00:00.02
	Memory Required	48560 bytes
	Additional Memory Required for Residual Plots	0 bytes

Variables Entered/Removed[a]

Model	Variables Entered	Variables Removed	Method
1	Party0[b]	.	Enter
2	SES0, RaceCC[b]	.	Enter
3	MGRS0, MRN0[b]	.	Enter
4	MRN0xSES0, MGRS0xRace, MRN0xParty0, MGRS0xParty0, MGRS0xSES0, MRN0xRace, MRN0xMGRS...[b]	.	Enter

a. Dependent Variable: Risk_Rules

b. All requested variables entered.

Model Summary

Model	R	R Square	Adjusted R Square	Std. Error of the Estimate	Change Statistics		
					R Square Change	F Change	df1
1	.518[a]	.268	.262	.94666	.268	42.470	1
2	.530[b]	.280	.262	.94676	.012	.987	2
3	.601[c]	.361	.332	.90033	.080	7.031	2
4	.621[d]	.385	.315	.91175	.025	.602	7

Model Summary

Model	Change Statistics	
	df2	Sig. F Change
1	116	.000
2	114	.376
3	112	.001
4	105	.753

a. Predictors: (Constant), Party0

b. Predictors: (Constant), Party0, SES0, RaceCC

c. Predictors: (Constant), Party0, SES0, RaceCC, MGRS0, MRN0

d. Predictors: (Constant), Party0, SES0, RaceCC, MGRS0, MRN0, MRN0xSES0, MGRS0xRace, MRN0xParty0, MGRS0xParty0, MGRS0xSES0, MRN0xRace, MRN0xMGRS0

ANOVA[a]

Model		Sum of Squares	df	Mean Square	F	Sig.
1	Regression	38.060	1	38.060	42.470	.000[b]
	Residual	103.955	116	.896		
	Total	142.015	117			
2	Regression	39.830	3	13.277	14.812	.000[c]
	Residual	102.185	114	.896		
	Total	142.015	117			
3	Regression	51.229	5	10.246	12.640	.000[d]
	Residual	90.786	112	.811		
	Total	142.015	117			
4	Regression	54.730	12	4.561	5.486	.000[e]
	Residual	87.285	105	.831		
	Total	142.015	117			

a. Dependent Variable: Risk_Rules

b. Predictors: (Constant), Party0

c. Predictors: (Constant), Party0, SES0, RaceCC

d. Predictors: (Constant), Party0, SES0, RaceCC, MGRS0, MRN0

e. Predictors: (Constant), Party0, SES0, RaceCC, MGRS0, MRN0, MRN0xSES0, MGRS0xRace, MRN0xParty0, MGRS0xParty0, MGRS0xSES0, MRN0xRace, MRN0xMGRS0

Coefficients[a]

Model		Unstandardized Coefficients B	Std. Error	Standardized Coefficients Beta	t	Sig.
1	(Constant)	3.378	.090		37.668	.000
	Party0	.418	.064	.518	6.517	.000
2	(Constant)	3.307	.103		32.143	.000
	Party0	.391	.067	.484	5.836	.000
	RaceCC	.151	.107	.117	1.404	.163
	SES0	.018	.101	.014	.176	.860
3	(Constant)	3.197	.118		27.192	.000
	Party0	.253	.075	.313	3.361	.001
	RaceCC	.230	.104	.179	2.207	.029
	SES0	-.027	.097	-.021	-.277	.782
	MGRS0	.103	.164	.053	.630	.530
	MRN0	.385	.124	.305	3.104	.002
4	(Constant)	3.297	.169		19.545	.000
	Party0	.238	.086	.295	2.761	.007
	RaceCC	.174	.145	.135	1.204	.231
	SES0	-.147	.146	-.116	-1.003	.318
	MGRS0	.172	.270	.088	.638	.525
	MRN0	.209	.186	.165	1.122	.265
	MRN0xRace	.214	.158	.180	1.351	.180
	MRN0xSES0	.080	.123	.069	.651	.517
	MRN0xParty0	-.022	.079	-.028	-.275	.784
	MRN0xMGRS0	-.042	.242	-.025	-.175	.862
	MGRS0xRace	.022	.247	.012	.090	.929
	MGRS0xSES0	-.276	.247	-.135	-1.116	.267
	MGRS0xParty0	-.010	.162	-.008	-.063	.950

Coefficients[a]

Model		Correlations		
		Zero-order	Partial	Part
1	(Constant)			
	Party0	.518	.518	.518
2	(Constant)			
	Party0	.518	.480	.464
	RaceCC	.255	.130	.112
	SES0	.003	.017	.014
3	(Constant)			
	Party0	.518	.303	.254
	RaceCC	.255	.204	.167
	SES0	.003	-.026	-.021
	MGRS0	.207	.059	.048
	MRN0	.468	.281	.235
4	(Constant)			
	Party0	.518	.260	.211
	RaceCC	.255	.117	.092
	SES0	.003	-.097	-.077
	MGRS0	.207	.062	.049
	MRN0	.468	.109	.086
	MRN0xRace	.477	.131	.103
	MRN0xSES0	-.185	.063	.050
	MRN0xParty0	.177	-.027	-.021
	MRN0xMGRS0	-.257	-.017	-.013
	MGRS0xRace	.085	.009	.007
	MGRS0xSES0	-.193	-.108	-.085
	MGRS0xParty0	-.158	-.006	-.005

a. Dependent Variable: Risk_Rules

Excluded Variables[a]

Model		Beta In	t	Sig.	Partial Correlation	Collinearity Statistics Tolerance
1	RaceCC	.116[b]	1.400	.164	.129	.918
	SES0	.004[b]	.054	.957	.005	1.000
	MGRS0	.152[b]	1.918	.058	.176	.988
	MRN0	.283[b]	3.243	.002	.289	.765
	MRN0xRace	.295[b]	3.380	.001	.301	.762
	MRN0xSES0	-.081[b]	-.995	.322	-.092	.957
	MRN0xParty0	-.045[b]	-.514	.609	-.048	.829
	MRN0xMGRS0	-.092[b]	-1.091	.278	-.101	.885
	MGRS0xRace	.071[b]	.892	.374	.083	.999
	MGRS0xSES0	-.115[b]	-1.435	.154	-.133	.976
	MGRS0xParty0	-.048[b]	-.590	.556	-.055	.953
2	MGRS0	.160[c]	2.027	.045	.187	.981
	MRN0	.330[c]	3.707	.000	.329	.716
	MRN0xRace	.280[c]	3.087	.003	.279	.712
	MRN0xSES0	-.091[c]	-1.088	.279	-.102	.900
	MRN0xParty0	-.065[c]	-.719	.473	-.068	.770
	MRN0xMGRS0	-.098[c]	-1.102	.273	-.103	.791
	MGRS0xRace	.118[c]	1.409	.162	.131	.888
	MGRS0xSES0	-.184[c]	-1.922	.057	-.178	.670
	MGRS0xParty0	-.050[c]	-.601	.549	-.056	.930
3	MRN0xRace	.172[d]	1.670	.098	.157	.529
	MRN0xSES0	-.019[d]	-.222	.825	-.021	.823
	MRN0xParty0	-.007[d]	-.084	.933	-.008	.737
	MRN0xMGRS0	-.073[d]	-.674	.502	-.064	.493
	MGRS0xRace	.100[d]	1.136	.258	.107	.741
	MGRS0xSES0	-.091[d]	-.938	.350	-.089	.611
	MGRS0xParty0	.018[d]	.207	.837	.020	.740

a. Dependent Variable: Risk_Rules

b. Predictors in the Model: (Constant), Party0

c. Predictors in the Model: (Constant), Party0, SES0, RaceCC

d. Predictors in the Model: (Constant), Party0, SES0, RaceCC, MGRS0, MRN0

```
REGRESSION
  /MISSING LISTWISE
  /STATISTICS COEFF OUTS R ANOVA CHANGE ZPP
  /CRITERIA=PIN(.05) POUT(.10)
  /NOORIGIN
  /DEPENDENT Risk_Help
  /METHOD=ENTER Party0
  /METHOD=ENTER RaceCC SES0
  /METHOD=ENTER MGRS0 MRN0
  /METHOD=ENTER MRN0xRace MRN0xSES0 MRN0xParty0 MRN0xMGRS0 MGRS0xRace MGRS0xSES0 MGRS0xPa
rty0.
```

Regression

Notes

Output Created		15-DEC-2021 13:07:59
Comments		
Input	Data	C:\Users\njs5478\Dropbox\HM and COVID\0. Revise and Resubmit\2. R and R Data\Study 1b\Study1b_Data.sav
	Active Dataset	DataSet1
	Filter	GenderCC=1 (FILTER)
	Weight	<none>
	Split File	<none>
	N of Rows in Working Data File	119
Missing Value Handling	Definition of Missing	User-defined missing values are treated as missing.
	Cases Used	Statistics are based on cases with no missing values for any variable used.

Notes

Syntax		REGRESSION<br>/MISSING LISTWISE<br>/STATISTICS COEFF OUTS R ANOVA CHANGE ZPP<br>/CRITERIA=PIN(.05) POUT(.10)<br>/NOORIGIN<br>/DEPENDENT Risk_Help<br>/METHOD=ENTER Party0<br>/METHOD=ENTER RaceCC SES0<br>/METHOD=ENTER MGRS0 MRN0<br>/METHOD=ENTER MRN0xRace MRN0xSES0 MRN0xParty0 MRN0xMGRS0 MGRS0xRace MGRS0xSES0 MGRS0xParty0.
Resources	Processor Time	00:00:00.02
	Elapsed Time	00:00:00.01
	Memory Required	48560 bytes
	Additional Memory Required for Residual Plots	0 bytes

Variables Entered/Removed[a]

Model	Variables Entered	Variables Removed	Method
1	Party0[b]	.	Enter
2	SES0, RaceCC[b]	.	Enter
3	MGRS0, MRN0[b]	.	Enter
4	MRN0xSES0, MGRS0xRace, MRN0xParty0, MGRS0xParty0, MGRS0xSES0, MRN0xRace, MRN0xMGRS0[b]	.	Enter

a. Dependent Variable: Risk_Help

b. All requested variables entered.

Model Summary

Model	R	R Square	Adjusted R Square	Std. Error of the Estimate	Change Statistics		
					R Square Change	F Change	df1
1	.186[a]	.035	.026	1.47961	.035	4.175	1
2	.334[b]	.112	.088	1.43195	.077	4.925	2
3	.353[c]	.124	.085	1.43427	.013	.816	2
4	.414[d]	.172	.077	1.44060	.047	.860	7

Model Summary

Model	Change Statistics	
	df2	Sig. F Change
1	116	.043
2	114	.009
3	112	.445
4	105	.541

a. Predictors: (Constant), Party0

b. Predictors: (Constant), Party0, SES0, RaceCC

c. Predictors: (Constant), Party0, SES0, RaceCC, MGRS0, MRN0

d. Predictors: (Constant), Party0, SES0, RaceCC, MGRS0, MRN0, MRN0xSES0, MGRS0xRace, MRN0xParty0, MGRS0xParty0, MGRS0xSES0, MRN0xRace, MRN0xMGRS0

ANOVA[a]

Model		Sum of Squares	df	Mean Square	F	Sig.
1	Regression	9.139	1	9.139	4.175	.043[b]
	Residual	253.953	116	2.189		
	Total	263.092	117			
2	Regression	29.336	3	9.779	4.769	.004[c]
	Residual	233.756	114	2.050		
	Total	263.092	117			
3	Regression	32.694	5	6.539	3.179	.010[d]
	Residual	230.398	112	2.057		
	Total	263.092	117			
4	Regression	45.181	12	3.765	1.814	.055[e]
	Residual	217.911	105	2.075		
	Total	263.092	117			

a. Dependent Variable: Risk_Help

b. Predictors: (Constant), Party0

c. Predictors: (Constant), Party0, SES0, RaceCC

d. Predictors: (Constant), Party0, SES0, RaceCC, MGRS0, MRN0

e. Predictors: (Constant), Party0, SES0, RaceCC, MGRS0, MRN0, MRN0xSES0, MGRS0xRace, MRN0xParty0, MGRS0xParty0, MGRS0xSES0, MRN0xRace, MRN0xMGRS0

Coefficients[a]

Model		Unstandardized Coefficients B	Unstandardized Coefficients Std. Error	Standardized Coefficients Beta	t	Sig.
1	(Constant)	3.689	.140		26.321	.000
	Party0	-.205	.100	-.186	-2.043	.043
2	(Constant)	3.900	.156		25.061	.000
	Party0	-.128	.101	-.116	-1.258	.211
	RaceCC	-.433	.162	-.247	-2.666	.009
	SES0	-.287	.152	-.167	-1.882	.062
3	(Constant)	3.781	.187		20.189	.000
	Party0	-.159	.120	-.144	-1.324	.188
	RaceCC	-.421	.166	-.240	-2.533	.013
	SES0	-.297	.154	-.173	-1.927	.056
	MGRS0	-.328	.261	-.123	-1.259	.211
	MRN0	.142	.198	.083	.719	.474
4	(Constant)	3.797	.267		14.245	.000
	Party0	-.127	.136	-.116	-.932	.354
	RaceCC	-.263	.229	-.150	-1.150	.253
	SES0	-.555	.231	-.323	-2.406	.018
	MGRS0	-.346	.426	-.130	-.812	.419
	MRN0	.081	.294	.047	.275	.784
	MRN0xRace	-.118	.250	-.073	-.473	.637
	MRN0xSES0	.279	.194	.176	1.437	.154
	MRN0xParty0	-.059	.125	-.055	-.475	.636
	MRN0xMGRS0	.133	.383	.057	.348	.728
	MGRS0xRace	.464	.390	.183	1.192	.236
	MGRS0xSES0	-.352	.390	-.127	-.903	.369
	MGRS0xParty0	-.439	.255	-.246	-1.721	.088

Coefficients[a]

Model		Correlations		
		Zero-order	Partial	Part
1	(Constant)			
	Party0	-.186	-.186	-.186
2	(Constant)			
	Party0	-.186	-.117	-.111
	RaceCC	-.266	-.242	-.235
	SES0	-.146	-.174	-.166
3	(Constant)			
	Party0	-.186	-.124	-.117
	RaceCC	-.266	-.233	-.224
	SES0	-.146	-.179	-.170
	MGRS0	-.101	-.118	-.111
	MRN0	-.050	.068	.064
4	(Constant)			
	Party0	-.186	-.091	-.083
	RaceCC	-.266	-.111	-.102
	SES0	-.146	-.229	-.214
	MGRS0	-.101	-.079	-.072
	MRN0	-.050	.027	.024
	MRN0xRace	-.137	-.046	-.042
	MRN0xSES0	.086	.139	.128
	MRN0xParty0	-.148	-.046	-.042
	MRN0xMGRS0	.011	.034	.031
	MGRS0xRace	.041	.116	.106
	MGRS0xSES0	.090	-.088	-.080
	MGRS0xParty0	-.091	-.166	-.153

a. Dependent Variable: Risk_Help

Excluded Variables[a]

Model		Beta In	t	Sig.	Partial Correlation	Collinearity Statistics Tolerance
1	RaceCC	-.231[b]	-2.484	.014	-.226	.918
	SES0	-.146[b]	-1.614	.109	-.149	1.000
	MGRS0	-.082[b]	-.889	.376	-.083	.988
	MRN0	.053[b]	.505	.614	.047	.765
	MRN0xRace	-.061[b]	-.581	.562	-.054	.762
	MRN0xSES0	.049[b]	.526	.600	.049	.957
	MRN0xParty0	-.086[b]	-.855	.394	-.080	.829
	MRN0xMGRS0	-.059[b]	-.605	.546	-.056	.885
	MGRS0xRace	.045[b]	.496	.621	.046	.999
	MGRS0xSES0	.062[b]	.675	.501	.063	.976
	MGRS0xParty0	-.138[b]	-1.480	.142	-.137	.953
2	MGRS0	-.094[c]	-1.058	.292	-.099	.981
	MRN0	.023[c]	.217	.829	.020	.716
	MRN0xRace	.012[c]	.110	.913	.010	.712
	MRN0xSES0	.099[c]	1.062	.290	.099	.900
	MRN0xParty0	-.094[c]	-.931	.354	-.087	.770
	MRN0xMGRS0	-.128[c]	-1.297	.197	-.121	.791
	MGRS0xRace	-.011[c]	-.116	.908	-.011	.888
	MGRS0xSES0	.017[c]	.154	.878	.015	.670
	MGRS0xParty0	-.163[c]	-1.799	.075	-.167	.930
3	MRN0xRace	-.025[d]	-.208	.836	-.020	.529
	MRN0xSES0	.088[d]	.902	.369	.085	.823
	MRN0xParty0	-.112[d]	-1.084	.281	-.102	.737
	MRN0xMGRS0	-.086[d]	-.678	.499	-.064	.493
	MGRS0xRace	.043[d]	.415	.679	.039	.741
	MGRS0xSES0	.012[d]	.105	.917	.010	.611
	MGRS0xParty0	-.147[d]	-1.433	.155	-.135	.740

a. Dependent Variable: Risk_Help

b. Predictors in the Model: (Constant), Party0

c. Predictors in the Model: (Constant), Party0, SES0, RaceCC

d. Predictors in the Model: (Constant), Party0, SES0, RaceCC, MGRS0, MRN0

```
 FILTER OFF.
USE ALL.
EXECUTE.

**PIdeology

REGRESSION
  /MISSING LISTWISE
  /STATISTICS COEFF OUTS R ANOVA CHANGE ZPP
  /CRITERIA=PIN(.05) POUT(.10)
  /NOORIGIN
  /DEPENDENT Concern_Tot
  /METHOD=ENTER Ideology0
  /METHOD=ENTER GenderCC RaceCC SES0
  /METHOD=ENTER MGRS0 MRN0
  /METHOD=ENTER MRN0xRace MRN0xSES0 MRN0xGender MRN0xIdeology0 MRN0xMGRS0 MGRS0xGender MG
RS0xRace MGRS0xSES0 MGRS0xIdeology0.
```

Regression

Notes

Output Created		15-DEC-2021 13:07:59
Comments		
Input	Data	C: \Users\njs5478\Dropbox\HM and COVID\0. Revise and Resubmit\2. R and R Data\Study 1b\Study1b_Data.sav
	Active Dataset	DataSet1
	Filter	<none>
	Weight	<none>
	Split File	<none>
	N of Rows in Working Data File	241
Missing Value Handling	Definition of Missing	User-defined missing values are treated as missing.
	Cases Used	Statistics are based on cases with no missing values for any variable used.

Notes

Syntax		REGRESSION<br>/MISSING LISTWISE<br>/STATISTICS COEFF OUTS R ANOVA CHANGE ZPP<br>/CRITERIA=PIN(.05) POUT(.10)<br>/NOORIGIN<br>/DEPENDENT Concern_Tot<br>/METHOD=ENTER Ideology0<br>/METHOD=ENTER GenderCC RaceCC SES0<br>/METHOD=ENTER MGRS0 MRN0<br>/METHOD=ENTER MRN0xRace MRN0xSES0 MRN0xGender MRN0xIdeology0 MRN0xMGRS0 MGRS0xGender MGRS0xRace MGRS0xSES0 MGRS0xIdeology0.
Resources	Processor Time	00:00:00.05
	Elapsed Time	00:00:00.05
	Memory Required	52240 bytes
	Additional Memory Required for Residual Plots	0 bytes

Variables Entered/Removed[a]

Model	Variables Entered	Variables Removed	Method
1	Ideology0[b]	.	Enter
2	SES0, GenderCC, RaceCC[b]	.	Enter
3	MGRS0, MRN0[b]	.	Enter
4	MRN0xMGRS0, MRN0xSES0, MRN0xIdeology0, MGRS0xSES0, MGRS0xGender, MGRS0xRace, MRN0xRace, MRN0xGender, MGRS0xIdeol...[b]	.	Enter

a. Dependent Variable: Concern_Tot

b. All requested variables entered.

Model Summary

Model	R	R Square	Adjusted R Square	Std. Error of the Estimate	Change Statistics		
					R Square Change	F Change	df1
1	.499[a]	.249	.246	1.20930	.249	77.973	1
2	.512[b]	.262	.249	1.20645	.013	1.371	3
3	.530[c]	.281	.263	1.19583	.019	3.069	2
4	.576[d]	.332	.287	1.17604	.051	1.867	9

Model Summary

Model	Change Statistics df2	Sig. F Change
1	235	.000
2	232	.253
3	230	.048
4	221	.058

a. Predictors: (Constant), Ideology0

b. Predictors: (Constant), Ideology0, SES0, GenderCC, RaceCC

c. Predictors: (Constant), Ideology0, SES0, GenderCC, RaceCC, MGRS0, MRN0

d. Predictors: (Constant), Ideology0, SES0, GenderCC, RaceCC, MGRS0, MRN0, MRN0xMGRS0, MRN0xSES0, MRN0xIdeology0, MGRS0xSES0, MGRS0xGender, MGRS0xRace, MRN0xRace, MRN0xGender, MGRS0xIdeology0

ANOVA[a]

Model		Sum of Squares	df	Mean Square	F	Sig.
1	Regression	114.028	1	114.028	77.973	.000[b]
	Residual	343.666	235	1.462		
	Total	457.693	236			
2	Regression	120.012	4	30.003	20.613	.000[c]
	Residual	337.681	232	1.456		
	Total	457.693	236			
3	Regression	128.791	6	21.465	15.010	.000[d]
	Residual	328.902	230	1.430		
	Total	457.693	236			
4	Regression	152.034	15	10.136	7.328	.000[e]
	Residual	305.660	221	1.383		
	Total	457.693	236			

a. Dependent Variable: Concern_Tot

b. Predictors: (Constant), Ideology0

c. Predictors: (Constant), Ideology0, SES0, GenderCC, RaceCC

d. Predictors: (Constant), Ideology0, SES0, GenderCC, RaceCC, MGRS0, MRN0

e. Predictors: (Constant), Ideology0, SES0, GenderCC, RaceCC, MGRS0, MRN0, MRN0xMGRS0, MRN0xSES0, MRN0xIdeology0, MGRS0xSES0, MGRS0xGender, MGRS0xRace, MRN0xRace, MRN0xGender, MGRS0xIdeology0

Coefficients[a]

Model		Unstandardized Coefficients B	Std. Error	Standardized Coefficients Beta	t	Sig.
1	(Constant)	4.333	.079		55.157	.000
	Ideology0	-.437	.049	-.499	-8.830	.000
2	(Constant)	4.342	.092		47.442	.000
	Ideology0	-.406	.054	-.464	-7.502	.000
	GenderCC	-.165	.081	-.119	-2.025	.044
	RaceCC	-.019	.095	-.012	-.203	.839
	SES0	.010	.097	.006	.103	.918
3	(Constant)	4.352	.091		47.807	.000
	Ideology0	-.333	.063	-.380	-5.310	.000
	GenderCC	-.047	.095	-.034	-.495	.621
	RaceCC	-.042	.096	-.026	-.434	.665
	SES0	.043	.097	.025	.441	.659
	MGRS0	.196	.137	.091	1.431	.154
	MRN0	-.291	.122	-.181	-2.381	.018
4	(Constant)	4.201	.120		35.022	.000
	Ideology0	-.290	.064	-.332	-4.506	.000
	GenderCC	-.135	.098	-.097	-1.375	.170
	RaceCC	-.054	.099	-.034	-.544	.587
	SES0	.082	.098	.048	.834	.405
	MGRS0	.139	.176	.065	.791	.430
	MRN0	-.137	.134	-.085	-1.021	.309
	MRN0xRace	-.218	.114	-.136	-1.918	.056
	MRN0xSES0	.192	.105	.108	1.836	.068
	MRN0xGender	.170	.127	.096	1.344	.180
	MRN0xIdeology0	.007	.058	.008	.121	.904
	MRN0xMGRS0	.350	.199	.161	1.762	.079
	MGRS0xGender	-.294	.162	-.128	-1.818	.070
	MGRS0xRace	.034	.154	.016	.222	.824
	MGRS0xSES0	.164	.163	.062	1.004	.317
	MGRS0xIdeology0	-.029	.106	-.023	-.271	.786

Coefficients[a]

Model		Correlations Zero-order	Partial	Part
1	(Constant)			
	Ideology0	-.499	-.499	-.499
2	(Constant)			
	Ideology0	-.499	-.442	-.423
	GenderCC	-.240	-.132	-.114
	RaceCC	-.168	-.013	-.011
	SES0	-.027	.007	.006
3	(Constant)			
	Ideology0	-.499	-.330	-.297
	GenderCC	-.240	-.033	-.028
	RaceCC	-.168	-.029	-.024
	SES0	-.027	.029	.025
	MGRS0	.104	.094	.080
	MRN0	-.388	-.155	-.133
4	(Constant)			
	Ideology0	-.499	-.290	-.248
	GenderCC	-.240	-.092	-.076
	RaceCC	-.168	-.037	-.030
	SES0	-.027	.056	.046
	MGRS0	.104	.053	.043
	MRN0	-.388	-.068	-.056
	MRN0xRace	-.326	-.128	-.105
	MRN0xSES0	.197	.123	.101
	MRN0xGender	-.075	.090	.074
	MRN0xIdeology0	.011	.008	.007
	MRN0xMGRS0	.132	.118	.097
	MGRS0xGender	-.113	-.121	-.100
	MGRS0xRace	.015	.015	.012
	MGRS0xSES0	.089	.067	.055
	MGRS0xIdeology0	.032	-.018	-.015

a. Dependent Variable: Concern_Tot

Excluded Variables[a]

Model		Beta In	t	Sig.	Partial Correlation	Collinearity Statistics Tolerance
1	GenderCC	-.118[b]	-2.023	.044	-.131	.931
	RaceCC	-.006[b]	-.092	.927	-.006	.893
	SES0	.001[b]	.017	.987	.001	.997
	MGRS0	.075[b]	1.335	.183	.087	.997
	MRN0	-.168[b]	-2.527	.012	-.163	.709
	MRN0xRace	-.152[b]	-2.491	.013	-.161	.842
	MRN0xSES0	.134[b]	2.376	.018	.153	.983
	MRN0xGender	-.029[b]	-.505	.614	-.033	.991
	MRN0xIdeology0	.027[b]	.479	.632	.031	.999
	MRN0xMGRS0	.098[b]	1.745	.082	.113	.995
	MGRS0xGender	-.077[b]	-1.370	.172	-.089	.995
	MGRS0xRace	.046[b]	.815	.416	.053	.996
	MGRS0xSES0	.053[b]	.941	.347	.061	.995
	MGRS0xIdeology0	.069[b]	1.215	.226	.079	.995
2	MGRS0	.041[c]	.679	.498	.045	.868
	MRN0	-.145[c]	-2.018	.045	-.132	.605
	MRN0xRace	-.154[c]	-2.493	.013	-.162	.812
	MRN0xSES0	.142[c]	2.522	.012	.164	.979
	MRN0xGender	-.035[c]	-.608	.544	-.040	.973
	MRN0xIdeology0	.039[c]	.671	.503	.044	.961
	MRN0xMGRS0	.116[c]	2.037	.043	.133	.966
	MGRS0xGender	-.080[c]	-1.415	.158	-.093	.987
	MGRS0xRace	.030[c]	.527	.599	.035	.973
	MGRS0xSES0	.070[c]	1.220	.224	.080	.959
	MGRS0xIdeology0	.076[c]	1.341	.181	.088	.981
3	MRN0xRace	-.127[d]	-1.959	.051	-.128	.734
	MRN0xSES0	.133[d]	2.341	.020	.153	.946
	MRN0xGender	-.020[d]	-.352	.725	-.023	.942
	MRN0xIdeology0	.019[d]	.331	.741	.022	.935
	MRN0xMGRS0	.107[d]	1.894	.059	.124	.961
	MGRS0xGender	-.057[d]	-.993	.322	-.066	.945

Excluded Variables[a]

Model		Beta In	t	Sig.	Partial Correlation	Collinearity Statistics Tolerance
	MGRS0xRace	-.001[d]	-.011	.991	-.001	.711
	MGRS0xSES0	.077[d]	1.295	.197	.085	.890
	MGRS0xIdeology0	.071[d]	1.234	.218	.081	.950

a. Dependent Variable: Concern_Tot

b. Predictors in the Model: (Constant), Ideology0

c. Predictors in the Model: (Constant), Ideology0, SES0, GenderCC, RaceCC

d. Predictors in the Model: (Constant), Ideology0, SES0, GenderCC, RaceCC, MGRS0, MRN0

```
 REGRESSION
  /MISSING LISTWISE
  /STATISTICS COEFF OUTS R ANOVA CHANGE ZPP
  /CRITERIA=PIN(.05) POUT(.10)
  /NOORIGIN
  /DEPENDENT Finance_Tot
  /METHOD=ENTER Ideology0
  /METHOD=ENTER GenderCC RaceCC SES0
  /METHOD=ENTER MGRS0 MRN0
  /METHOD=ENTER MRN0xRace MRN0xSES0 MRN0xGender MRN0xIdeology0 MRN0xMGRS0 MGRS0xGender MG
RS0xRace MGRS0xSES0 MGRS0xIdeology0.
```

Regression

Notes

Output Created		15-DEC-2021 13:07:59
Comments		
Input	Data	C: \Users\njs5478\Dropbox\HM and COVID\0. Revise and Resubmit\2. R and R Data\Study 1b\Study1b_Data.sav
	Active Dataset	DataSet1
	Filter	<none>
	Weight	<none>
	Split File	<none>
	N of Rows in Working Data File	241
Missing Value Handling	Definition of Missing	User-defined missing values are treated as missing.
	Cases Used	Statistics are based on cases with no missing values for any variable used.
Syntax		REGRESSION /MISSING LISTWISE /STATISTICS COEFF OUTS R ANOVA CHANGE ZPP /CRITERIA=PIN(.05) POUT(.10) /NOORIGIN /DEPENDENT Finance_Tot /METHOD=ENTER Ideology0 /METHOD=ENTER GenderCC RaceCC SES0 /METHOD=ENTER MGRS0 MRN0 /METHOD=ENTER MRN0xRace MRN0xSES0 MRN0xGender MRN0xIdeology0 MRN0xMGRS0 MGRS0xGender MGRS0xRace MGRS0xSES0 MGRS0xIdeology0.

Notes

Resources	Processor Time	00:00:00.03
	Elapsed Time	00:00:00.02
	Memory Required	52240 bytes
	Additional Memory Required for Residual Plots	0 bytes

Variables Entered/Removed[a]

Model	Variables Entered	Variables Removed	Method
1	Ideology0[b]	.	Enter
2	SES0, GenderCC, RaceCC[b]	.	Enter
3	MGRS0, MRN0[b]	.	Enter
4	MRN0xMGRS0, MRN0xSES0, MRN0xIdeology0, MGRS0xSES0, MGRS0xGender, MGRS0xRace, MRN0xRace, MRN0xGender, MGRS0xIdeol...[b]	.	Enter

a. Dependent Variable: Finance_Tot

b. All requested variables entered.

Model Summary

Model	R	R Square	Adjusted R Square	Std. Error of the Estimate	Change Statistics R Square Change	F Change	df1
1	.141[a]	.020	.016	1.52520	.020	4.782	1
2	.222[b]	.049	.033	1.51175	.029	2.400	3
3	.238[c]	.056	.032	1.51267	.007	.860	2
4	.324[d]	.105	.044	1.50305	.048	1.328	9

Model Summary

Model	Change Statistics df2	Sig. F Change
1	235	.030
2	232	.069
3	230	.425
4	221	.223

a. Predictors: (Constant), Ideology0

b. Predictors: (Constant), Ideology0, SES0, GenderCC, RaceCC

c. Predictors: (Constant), Ideology0, SES0, GenderCC, RaceCC, MGRS0, MRN0

d. Predictors: (Constant), Ideology0, SES0, GenderCC, RaceCC, MGRS0, MRN0, MRN0xMGRS0, MRN0xSES0, MRN0xIdeology0, MGRS0xSES0, MGRS0xGender, MGRS0xRace, MRN0xRace, MRN0xGender, MGRS0xIdeology0

ANOVA[a]

Model		Sum of Squares	df	Mean Square	F	Sig.
1	Regression	11.125	1	11.125	4.782	.030[b]
	Residual	546.667	235	2.326		
	Total	557.792	236			
2	Regression	27.579	4	6.895	3.017	.019[c]
	Residual	530.213	232	2.285		
	Total	557.792	236			
3	Regression	31.513	6	5.252	2.295	.036[d]
	Residual	526.279	230	2.288		
	Total	557.792	236			
4	Regression	58.518	15	3.901	1.727	.047[e]
	Residual	499.274	221	2.259		
	Total	557.792	236			

a. Dependent Variable: Finance_Tot

b. Predictors: (Constant), Ideology0

c. Predictors: (Constant), Ideology0, SES0, GenderCC, RaceCC

d. Predictors: (Constant), Ideology0, SES0, GenderCC, RaceCC, MGRS0, MRN0

e. Predictors: (Constant), Ideology0, SES0, GenderCC, RaceCC, MGRS0, MRN0, MRN0xMGRS0, MRN0xSES0, MRN0xIdeology0, MGRS0xSES0, MGRS0xGender, MGRS0xRace, MRN0xRace, MRN0xGender, MGRS0xIdeology0

Coefficients[a]

Model		Unstandardized Coefficients B	Std. Error	Standardized Coefficients Beta	t	Sig.
1	(Constant)	3.898	.099		39.344	.000
	Ideology0	-.136	.062	-.141	-2.187	.030
2	(Constant)	3.907	.115		34.065	.000
	Ideology0	-.096	.068	-.100	-1.419	.157
	GenderCC	-.174	.102	-.113	-1.706	.089
	RaceCC	-.025	.120	-.014	-.211	.833
	SES0	-.243	.122	-.128	-1.992	.048
3	(Constant)	3.918	.115		34.031	.000
	Ideology0	-.066	.079	-.068	-.832	.406
	GenderCC	-.186	.120	-.121	-1.542	.124
	RaceCC	-.049	.121	-.028	-.406	.685
	SES0	-.241	.123	-.127	-1.958	.051
	MGRS0	-.155	.173	-.065	-.893	.373
	MRN0	-.095	.154	-.053	-.613	.540
4	(Constant)	3.857	.153		25.157	.000
	Ideology0	-.043	.082	-.044	-.518	.605
	GenderCC	-.209	.126	-.136	-1.664	.097
	RaceCC	-.046	.127	-.026	-.365	.715
	SES0	-.268	.126	-.141	-2.135	.034
	MGRS0	-.192	.225	-.081	-.856	.393
	MRN0	-.041	.171	-.023	-.238	.812
	MRN0xRace	-.089	.145	-.050	-.611	.542
	MRN0xSES0	.388	.134	.197	2.897	.004
	MRN0xGender	.085	.162	.044	.528	.598
	MRN0xIdeology0	.045	.075	.046	.601	.548
	MRN0xMGRS0	.103	.254	.043	.406	.685
	MGRS0xGender	.215	.207	.085	1.041	.299
	MGRS0xRace	.095	.197	.040	.483	.629
	MGRS0xSES0	-.136	.208	-.047	-.655	.513
	MGRS0xIdeology0	-.116	.135	-.085	-.859	.391

Coefficients[a]

Model		Correlations Zero-order	Partial	Part
1	(Constant)			
	Ideology0	-.141	-.141	-.141
2	(Constant)			
	Ideology0	-.141	-.093	-.091
	GenderCC	-.147	-.111	-.109
	RaceCC	-.055	-.014	-.014
	SES0	-.140	-.130	-.128
3	(Constant)			
	Ideology0	-.141	-.055	-.053
	GenderCC	-.147	-.101	-.099
	RaceCC	-.055	-.027	-.026
	SES0	-.140	-.128	-.125
	MGRS0	-.014	-.059	-.057
	MRN0	-.165	-.040	-.039
4	(Constant)			
	Ideology0	-.141	-.035	-.033
	GenderCC	-.147	-.111	-.106
	RaceCC	-.055	-.025	-.023
	SES0	-.140	-.142	-.136
	MGRS0	-.014	-.057	-.054
	MRN0	-.165	-.016	-.015
	MRN0xRace	-.099	-.041	-.039
	MRN0xSES0	.193	.191	.184
	MRN0xGender	.042	.036	.034
	MRN0xIdeology0	.046	.040	.038
	MRN0xMGRS0	.012	.027	.026
	MGRS0xGender	.044	.070	.066
	MGRS0xRace	-.017	.033	.031
	MGRS0xSES0	.014	-.044	-.042
	MGRS0xIdeology0	-.030	-.058	-.055

a. Dependent Variable: Finance_Tot

Excluded Variables[a]

Model		Beta In	t	Sig.	Partial Correlation	Collinearity Statistics Tolerance
1	GenderCC	-.118[b]	-1.773	.078	-.115	.931
	RaceCC	-.010[b]	-.150	.881	-.010	.893
	SES0	-.133[b]	-2.064	.040	-.134	.997
	MGRS0	-.022[b]	-.345	.731	-.023	.997
	MRN0	-.125[b]	-1.642	.102	-.107	.709
	MRN0xRace	-.051[b]	-.724	.470	-.047	.842
	MRN0xSES0	.178[b]	2.773	.006	.178	.983
	MRN0xGender	.056[b]	.861	.390	.056	.991
	MRN0xIdeology0	.050[b]	.780	.436	.051	.999
	MRN0xMGRS0	.002[b]	.029	.977	.002	.995
	MGRS0xGender	.055[b]	.843	.400	.055	.995
	MGRS0xRace	-.008[b]	-.127	.899	-.008	.996
	MGRS0xSES0	.004[b]	.068	.946	.004	.995
	MGRS0xIdeology0	-.020[b]	-.306	.760	-.020	.995
2	MGRS0	-.080[c]	-1.161	.247	-.076	.868
	MRN0	-.079[c]	-.961	.338	-.063	.605
	MRN0xRace	-.054[c]	-.756	.451	-.050	.812
	MRN0xSES0	.186[c]	2.921	.004	.189	.979
	MRN0xGender	.056[c]	.867	.387	.057	.973
	MRN0xIdeology0	.046[c]	.703	.483	.046	.961
	MRN0xMGRS0	.000[c]	-.004	.997	.000	.966
	MGRS0xGender	.065[c]	1.002	.318	.066	.987
	MGRS0xRace	-.024[c]	-.369	.712	-.024	.973
	MGRS0xSES0	.002[c]	.032	.975	.002	.959
	MGRS0xIdeology0	-.023[c]	-.360	.720	-.024	.981
3	MRN0xRace	-.037[d]	-.489	.625	-.032	.734
	MRN0xSES0	.177[d]	2.729	.007	.177	.946
	MRN0xGender	.072[d]	1.098	.274	.072	.942
	MRN0xIdeology0	.037[d]	.563	.574	.037	.935
	MRN0xMGRS0	-.002[d]	-.036	.972	-.002	.961
	MGRS0xGender	.063[d]	.949	.344	.063	.945

Excluded Variables[a]

Model		Beta In	t	Sig.	Partial Correlation	Collinearity Statistics Tolerance
	MGRS0xRace	.016[d]	.205	.838	.014	.711
	MGRS0xSES0	-.022[d]	-.323	.747	-.021	.890
	MGRS0xIdeology0	-.040[d]	-.600	.549	-.040	.950

a. Dependent Variable: Finance_Tot

b. Predictors in the Model: (Constant), Ideology0

c. Predictors in the Model: (Constant), Ideology0, SES0, GenderCC, RaceCC

d. Predictors in the Model: (Constant), Ideology0, SES0, GenderCC, RaceCC, MGRS0, MRN0

```
REGRESSION
  /MISSING LISTWISE
  /STATISTICS COEFF OUTS R ANOVA CHANGE ZPP
  /CRITERIA=PIN(.05) POUT(.10)
  /NOORIGIN
  /DEPENDENT Resource_Tot
  /METHOD=ENTER Ideology0
  /METHOD=ENTER GenderCC RaceCC SES0
  /METHOD=ENTER MGRS0 MRN0
  /METHOD=ENTER MRN0xRace MRN0xSES0 MRN0xGender MRN0xIdeology0 MRN0xMGRS0 MGRS0xGender MG
RS0xRace MGRS0xSES0 MGRS0xIdeology0.
```

Regression

Notes

Output Created		15-DEC-2021 13:07:59
Comments		
Input	Data	C: \Users\njs5478\Dropbox\HM and COVID\0. Revise and Resubmit\2. R and R Data\Study 1b\Study1b_Data.sav
	Active Dataset	DataSet1
	Filter	<none>
	Weight	<none>
	Split File	<none>
	N of Rows in Working Data File	241
Missing Value Handling	Definition of Missing	User-defined missing values are treated as missing.
	Cases Used	Statistics are based on cases with no missing values for any variable used.
Syntax		REGRESSION /MISSING LISTWISE /STATISTICS COEFF OUTS R ANOVA CHANGE ZPP /CRITERIA=PIN(.05) POUT(.10) /NOORIGIN /DEPENDENT Resource_Tot /METHOD=ENTER Ideology0 /METHOD=ENTER GenderCC RaceCC SES0 /METHOD=ENTER MGRS0 MRN0 /METHOD=ENTER MRN0xRace MRN0xSES0 MRN0xGender MRN0xIdeology0 MRN0xMGRS0 MGRS0xGender MGRS0xRace MGRS0xSES0 MGRS0xIdeology0.

Notes

Resources	Processor Time	00:00:00.03
	Elapsed Time	00:00:00.02
	Memory Required	52240 bytes
	Additional Memory Required for Residual Plots	0 bytes

Variables Entered/Removed[a]

Model	Variables Entered	Variables Removed	Method
1	Ideology0[b]	.	Enter
2	SES0, GenderCC, RaceCC[b]	.	Enter
3	MGRS0, MRN0[b]	.	Enter
4	MRN0xMGRS0, MRN0xSES0, MRN0xIdeology0, MGRS0xSES0, MGRS0xGender, MGRS0xRace, MRN0xRace, MRN0xGender, MGRS0xIdeol...[b]	.	Enter

a. Dependent Variable: Resource_Tot

b. All requested variables entered.

Model Summary

Model	R	R Square	Adjusted R Square	Std. Error of the Estimate	Change Statistics: R Square Change	F Change	df1	df2	Sig. F Change
1	.111[a]	.012	.008	1.38622	.012	2.944	1	235	.088
2	.150[b]	.023	.006	1.38797	.010	.802	3	232	.494
3	.154[c]	.024	-.002	1.39313	.001	.143	2	230	.867
4	.281[d]	.079	.016	1.38063	.055	1.465	9	221	.162

a. Predictors: (Constant), Ideology0

b. Predictors: (Constant), Ideology0, SES0, GenderCC, RaceCC

c. Predictors: (Constant), Ideology0, SES0, GenderCC, RaceCC, MGRS0, MRN0

d. Predictors: (Constant), Ideology0, SES0, GenderCC, RaceCC, MGRS0, MRN0, MRN0xMGRS0, MRN0xSES0, MRN0xIdeology0, MGRS0xSES0, MGRS0xGender, MGRS0xRace, MRN0xRace, MRN0xGender, MGRS0xIdeology0

ANOVA[a]

Model		Sum of Squares	df	Mean Square	F	Sig.
1	Regression	5.658	1	5.658	2.944	.088[b]
	Residual	451.578	235	1.922		
	Total	457.236	236			
2	Regression	10.294	4	2.574	1.336	.257[c]
	Residual	446.942	232	1.926		
	Total	457.236	236			
3	Regression	10.848	6	1.808	.932	.473[d]
	Residual	446.388	230	1.941		
	Total	457.236	236			
4	Regression	35.979	15	2.399	1.258	.231[e]
	Residual	421.257	221	1.906		
	Total	457.236	236			

a. Dependent Variable: Resource_Tot

b. Predictors: (Constant), Ideology0

c. Predictors: (Constant), Ideology0, SES0, GenderCC, RaceCC

d. Predictors: (Constant), Ideology0, SES0, GenderCC, RaceCC, MGRS0, MRN0

e. Predictors: (Constant), Ideology0, SES0, GenderCC, RaceCC, MGRS0, MRN0, MRN0xMGRS0, MRN0xSES0, MRN0xIdeology0, MGRS0xSES0, MGRS0xGender, MGRS0xRace, MRN0xRace, MRN0xGender, MGRS0xIdeology0

Coefficients[a]

Model		Unstandardized Coefficients B	Unstandardized Coefficients Std. Error	Standardized Coefficients Beta	t	Sig.
1	(Constant)	3.618	.090		40.182	.000
	Ideology0	-.097	.057	-.111	-1.716	.088
2	(Constant)	3.655	.105		34.711	.000
	Ideology0	-.063	.062	-.072	-1.010	.314
	GenderCC	-.112	.094	-.080	-1.192	.234
	RaceCC	-.077	.110	-.048	-.698	.486
	SES0	-.079	.112	-.046	-.703	.483
3	(Constant)	3.658	.106		34.500	.000
	Ideology0	-.043	.073	-.049	-.587	.558
	GenderCC	-.088	.111	-.063	-.792	.429
	RaceCC	-.085	.112	-.053	-.758	.449
	SES0	-.071	.113	-.042	-.630	.530
	MGRS0	.021	.159	.010	.131	.896
	MRN0	-.076	.142	-.047	-.532	.595
4	(Constant)	3.547	.141		25.190	.000
	Ideology0	.006	.076	.007	.077	.939
	GenderCC	-.117	.115	-.084	-1.010	.314
	RaceCC	-.114	.116	-.071	-.982	.327
	SES0	-.061	.115	-.035	-.527	.599
	MGRS0	.154	.206	.071	.744	.458
	MRN0	.056	.157	.035	.356	.722
	MRN0xRace	-.315	.133	-.196	-2.364	.019
	MRN0xSES0	.169	.123	.095	1.372	.171
	MRN0xGender	.195	.149	.110	1.310	.192
	MRN0xIdeology0	.075	.069	.085	1.089	.277
	MRN0xMGRS0	-.095	.234	-.044	-.409	.683
	MGRS0xGender	-.013	.190	-.006	-.066	.947
	MGRS0xRace	-.158	.181	-.073	-.875	.382
	MGRS0xSES0	.224	.191	.084	1.170	.243
	MGRS0xIdeology0	.001	.124	.001	.012	.991

Coefficients[a]

Model		Correlations Zero-order	Partial	Part
1	(Constant)			
	Ideology0	-.111	-.111	-.111
2	(Constant)			
	Ideology0	-.111	-.066	-.066
	GenderCC	-.103	-.078	-.077
	RaceCC	-.076	-.046	-.045
	SES0	-.056	-.046	-.046
3	(Constant)			
	Ideology0	-.111	-.039	-.038
	GenderCC	-.103	-.052	-.052
	RaceCC	-.076	-.050	-.049
	SES0	-.056	-.041	-.041
	MGRS0	.037	.009	.009
	MRN0	-.107	-.035	-.035
4	(Constant)			
	Ideology0	-.111	.005	.005
	GenderCC	-.103	-.068	-.065
	RaceCC	-.076	-.066	-.063
	SES0	-.056	-.035	-.034
	MGRS0	.037	.050	.048
	MRN0	-.107	.024	.023
	MRN0xRace	-.152	-.157	-.153
	MRN0xSES0	.111	.092	.089
	MRN0xGender	.086	.088	.085
	MRN0xIdeology0	.052	.073	.070
	MRN0xMGRS0	-.018	-.027	-.026
	MGRS0xGender	-.001	-.004	-.004
	MGRS0xRace	-.062	-.059	-.057
	MGRS0xSES0	.057	.078	.076
	MGRS0xIdeology0	-.041	.001	.001

a. Dependent Variable: Resource_Tot

Excluded Variables[a]

Model		Beta In	t	Sig.	Partial Correlation	Collinearity Statistics Tolerance
1	GenderCC	-.080[b]	-1.189	.236	-.077	.931
	RaceCC	-.044[b]	-.646	.519	-.042	.893
	SES0	-.050[b]	-.765	.445	-.050	.997
	MGRS0	.030[b]	.467	.641	.030	.997
	MRN0	-.066[b]	-.853	.395	-.056	.709
	MRN0xRace	-.128[b]	-1.820	.070	-.118	.842
	MRN0xSES0	.098[b]	1.508	.133	.098	.983
	MRN0xGender	.097[b]	1.501	.135	.098	.991
	MRN0xIdeology0	.056[b]	.858	.392	.056	.999
	MRN0xMGRS0	-.026[b]	-.397	.692	-.026	.995
	MGRS0xGender	.007[b]	.108	.914	.007	.995
	MGRS0xRace	-.056[b]	-.855	.393	-.056	.996
	MGRS0xSES0	.050[b]	.764	.446	.050	.995
	MGRS0xIdeology0	-.034[b]	-.515	.607	-.034	.995
2	MGRS0	-.003[c]	-.047	.962	-.003	.868
	MRN0	-.043[c]	-.519	.604	-.034	.605
	MRN0xRace	-.140[c]	-1.951	.052	-.127	.812
	MRN0xSES0	.103[c]	1.576	.116	.103	.979
	MRN0xGender	.093[c]	1.412	.159	.092	.973
	MRN0xIdeology0	.064[c]	.962	.337	.063	.961
	MRN0xMGRS0	-.022[c]	-.328	.743	-.022	.966
	MGRS0xGender	.011[c]	.170	.865	.011	.987
	MGRS0xRace	-.067[c]	-1.018	.310	-.067	.973
	MGRS0xSES0	.058[c]	.880	.380	.058	.959
	MGRS0xIdeology0	-.030[c]	-.460	.646	-.030	.981
3	MRN0xRace	-.142[d]	-1.872	.062	-.123	.734
	MRN0xSES0	.101[d]	1.518	.130	.100	.946
	MRN0xGender	.101[d]	1.513	.132	.100	.942
	MRN0xIdeology0	.060[d]	.883	.378	.058	.935
	MRN0xMGRS0	-.024[d]	-.363	.717	-.024	.961
	MGRS0xGender	.017[d]	.247	.805	.016	.945

Excluded Variables[a]

Model		Beta In	t	Sig.	Partial Correlation	Collinearity Statistics Tolerance
	MGRS0xRace	-.093[d]	-1.209	.228	-.080	.711
	MGRS0xSES0	.059[d]	.860	.391	.057	.890
	MGRS0xIdeology0	-.035[d]	-.527	.599	-.035	.950

a. Dependent Variable: Resource_Tot

b. Predictors in the Model: (Constant), Ideology0

c. Predictors in the Model: (Constant), Ideology0, SES0, GenderCC, RaceCC

d. Predictors in the Model: (Constant), Ideology0, SES0, GenderCC, RaceCC, MGRS0, MRN0

```
REGRESSION
  /MISSING LISTWISE
  /STATISTICS COEFF OUTS R ANOVA CHANGE ZPP
  /CRITERIA=PIN(.05) POUT(.10)
  /NOORIGIN
  /DEPENDENT Psychology_Tot
  /METHOD=ENTER Ideology0
  /METHOD=ENTER GenderCC RaceCC SES0
  /METHOD=ENTER MGRS0 MRN0
  /METHOD=ENTER MRN0xRace MRN0xSES0 MRN0xGender MRN0xIdeology0 MRN0xMGRS0 MGRS0xGender MG
RS0xRace MGRS0xSES0 MGRS0xIdeology0.
```

Regression

Notes

Output Created		15-DEC-2021 13:07:59
Comments		
Input	Data	C: \Users\njs5478\Dropbox\HM and COVID\0. Revise and Resubmit\2. R and R Data\Study 1b\Study1b_Data.sav
	Active Dataset	DataSet1
	Filter	<none>
	Weight	<none>
	Split File	<none>
	N of Rows in Working Data File	241
Missing Value Handling	Definition of Missing	User-defined missing values are treated as missing.
	Cases Used	Statistics are based on cases with no missing values for any variable used.
Syntax		REGRESSION<br>/MISSING LISTWISE<br>/STATISTICS COEFF OUTS R ANOVA CHANGE ZPP<br>/CRITERIA=PIN(.05) POUT(.10)<br>/NOORIGIN<br>/DEPENDENT Psychology_Tot<br>/METHOD=ENTER Ideology0<br>/METHOD=ENTER GenderCC RaceCC SES0<br>/METHOD=ENTER MGRS0 MRN0<br>/METHOD=ENTER MRN0xRace MRN0xSES0 MRN0xGender MRN0xIdeology0 MRN0xMGRS0 MGRS0xGender MGRS0xRace MGRS0xSES0 MGRS0xIdeology0.

Notes

Resources	Processor Time	00:00:00.02
	Elapsed Time	00:00:00.02
	Memory Required	52240 bytes
	Additional Memory Required for Residual Plots	0 bytes

Variables Entered/Removed[a]

Model	Variables Entered	Variables Removed	Method
1	Ideology0[b]	.	Enter
2	SES0, GenderCC, RaceCC[b]	.	Enter
3	MGRS0, MRN0[b]	.	Enter
4	MRN0xMGRS0, MRN0xSES0, MRN0xIdeology0, MGRS0xSES0, MGRS0xGender, MGRS0xRace, MRN0xRace, MRN0xGender, MGRS0xIdeol...[b]	.	Enter

a. Dependent Variable: Psychology_Tot

b. All requested variables entered.

Model Summary

Model	R	R Square	Adjusted R Square	Std. Error of the Estimate	Change Statistics		
					R Square Change	F Change	df1
1	.271[a]	.073	.069	1.43942	.073	18.602	1
2	.298[b]	.089	.073	1.43661	.015	1.307	3
3	.315[c]	.099	.075	1.43470	.010	1.309	2
4	.332[d]	.110	.050	1.45443	.011	.311	9

Model Summary

Model	Change Statistics	
	df2	Sig. F Change
1	235	.000
2	232	.273
3	230	.272
4	221	.971

a. Predictors: (Constant), Ideology0

b. Predictors: (Constant), Ideology0, SES0, GenderCC, RaceCC

c. Predictors: (Constant), Ideology0, SES0, GenderCC, RaceCC, MGRS0, MRN0

d. Predictors: (Constant), Ideology0, SES0, GenderCC, RaceCC, MGRS0, MRN0, MRN0xMGRS0, MRN0xSES0, MRN0xIdeology0, MGRS0xSES0, MGRS0xGender, MGRS0xRace, MRN0xRace, MRN0xGender, MGRS0xIdeology0

ANOVA[a]

Model		Sum of Squares	df	Mean Square	F	Sig.
1	Regression	38.541	1	38.541	18.602	.000[b]
	Residual	486.903	235	2.072		
	Total	525.444	236			
2	Regression	46.631	4	11.658	5.649	.000[c]
	Residual	478.813	232	2.064		
	Total	525.444	236			
3	Regression	52.020	6	8.670	4.212	.000[d]
	Residual	473.424	230	2.058		
	Total	525.444	236			
4	Regression	57.950	15	3.863	1.826	.032[e]
	Residual	467.494	221	2.115		
	Total	525.444	236			

a. Dependent Variable: Psychology_Tot

b. Predictors: (Constant), Ideology0

c. Predictors: (Constant), Ideology0, SES0, GenderCC, RaceCC

d. Predictors: (Constant), Ideology0, SES0, GenderCC, RaceCC, MGRS0, MRN0

e. Predictors: (Constant), Ideology0, SES0, GenderCC, RaceCC, MGRS0, MRN0, MRN0xMGRS0, MRN0xSES0, MRN0xIdeology0, MGRS0xSES0, MGRS0xGender, MGRS0xRace, MRN0xRace, MRN0xGender, MGRS0xIdeology0

Coefficients[a]

Model		Unstandardized Coefficients B	Std. Error	Standardized Coefficients Beta	t	Sig.
1	(Constant)	4.664	.094		49.879	.000
	Ideology0	-.254	.059	-.271	-4.313	.000
2	(Constant)	4.676	.109		42.903	.000
	Ideology0	-.221	.064	-.236	-3.431	.001
	GenderCC	-.185	.097	-.124	-1.905	.058
	RaceCC	-.023	.114	-.014	-.206	.837
	SES0	.071	.116	.038	.612	.541
3	(Constant)	4.687	.109		42.916	.000
	Ideology0	-.159	.075	-.169	-2.114	.036
	GenderCC	-.107	.114	-.072	-.939	.349
	RaceCC	-.048	.115	-.028	-.415	.679
	SES0	.094	.117	.051	.808	.420
	MGRS0	.079	.164	.034	.479	.632
	MRN0	-.237	.146	-.138	-1.617	.107
4	(Constant)	4.624	.148		31.164	.000
	Ideology0	-.139	.080	-.148	-1.742	.083
	GenderCC	-.141	.122	-.095	-1.157	.249
	RaceCC	-.086	.122	-.050	-.701	.484
	SES0	.112	.121	.061	.922	.357
	MGRS0	.067	.217	.029	.310	.757
	MRN0	-.150	.166	-.087	-.905	.367
	MRN0xRace	-.175	.140	-.102	-1.243	.215
	MRN0xSES0	-.040	.130	-.021	-.307	.759
	MRN0xGender	.067	.157	.035	.427	.670
	MRN0xIdeology0	.081	.072	.086	1.122	.263
	MRN0xMGRS0	.033	.246	.014	.134	.894
	MGRS0xGender	-.038	.200	-.015	-.188	.851
	MGRS0xRace	.010	.190	.004	.050	.960
	MGRS0xSES0	.093	.202	.033	.463	.644
	MGRS0xIdeology0	-.014	.131	-.011	-.110	.913

Coefficients[a]

Model		Correlations Zero-order	Partial	Part
1	(Constant)			
	Ideology0	-.271	-.271	-.271
2	(Constant)			
	Ideology0	-.271	-.220	-.215
	GenderCC	-.184	-.124	-.119
	RaceCC	-.094	-.014	-.013
	SES0	.018	.040	.038
3	(Constant)			
	Ideology0	-.271	-.138	-.132
	GenderCC	-.184	-.062	-.059
	RaceCC	-.094	-.027	-.026
	SES0	.018	.053	.051
	MGRS0	.052	.032	.030
	MRN0	-.251	-.106	-.101
4	(Constant)			
	Ideology0	-.271	-.116	-.111
	GenderCC	-.184	-.078	-.073
	RaceCC	-.094	-.047	-.044
	SES0	.018	.062	.059
	MGRS0	.052	.021	.020
	MRN0	-.251	-.061	-.057
	MRN0xRace	-.182	-.083	-.079
	MRN0xSES0	.035	-.021	-.019
	MRN0xGender	-.003	.029	.027
	MRN0xIdeology0	.051	.075	.071
	MRN0xMGRS0	.022	.009	.008
	MGRS0xGender	-.027	-.013	-.012
	MGRS0xRace	-.005	.003	.003
	MGRS0xSES0	.025	.031	.029
	MGRS0xIdeology0	-.008	-.007	-.007

a. Dependent Variable: Psychology_Tot

Excluded Variables[a]

Model		Beta In	t	Sig.	Partial Correlation	Collinearity Statistics Tolerance
1	GenderCC	-.122[b]	-1.879	.061	-.122	.931
	RaceCC	-.006[b]	-.093	.926	-.006	.893
	SES0	.033[b]	.529	.598	.035	.997
	MGRS0	.036[b]	.572	.568	.037	.997
	MRN0	-.148[b]	-1.991	.048	-.129	.709
	MRN0xRace	-.089[b]	-1.298	.195	-.085	.842
	MRN0xSES0	-.001[b]	-.009	.993	-.001	.983
	MRN0xGender	.023[b]	.360	.719	.024	.991
	MRN0xIdeology0	.059[b]	.946	.345	.062	.999
	MRN0xMGRS0	.004[b]	.058	.954	.004	.995
	MGRS0xGender	-.007[b]	-.117	.907	-.008	.995
	MGRS0xRace	.012[b]	.186	.853	.012	.996
	MGRS0xSES0	.006[b]	.096	.923	.006	.995
	MGRS0xIdeology0	.012[b]	.197	.844	.013	.995
2	MGRS0	-.004[c]	-.056	.955	-.004	.868
	MRN0	-.124[c]	-1.548	.123	-.101	.605
	MRN0xRace	-.088[c]	-1.269	.206	-.083	.812
	MRN0xSES0	.007[c]	.112	.911	.007	.979
	MRN0xGender	.016[c]	.252	.802	.017	.973
	MRN0xIdeology0	.077[c]	1.203	.230	.079	.961
	MRN0xMGRS0	.023[c]	.360	.719	.024	.966
	MGRS0xGender	-.012[c]	-.195	.846	-.013	.987
	MGRS0xRace	-.006[c]	-.095	.924	-.006	.973
	MGRS0xSES0	.026[c]	.405	.686	.027	.959
	MGRS0xIdeology0	.022[c]	.343	.732	.023	.981
3	MRN0xRace	-.061[d]	-.829	.408	-.055	.734
	MRN0xSES0	-.007[d]	-.115	.909	-.008	.946
	MRN0xGender	.032[d]	.498	.619	.033	.942
	MRN0xIdeology0	.062[d]	.956	.340	.063	.935
	MRN0xMGRS0	.016[d]	.255	.799	.017	.961
	MGRS0xGender	.003[d]	.039	.969	.003	.945

Excluded Variables[a]

Model		Beta In	t	Sig.	Partial Correlation	Collinearity Statistics Tolerance
	MGRS0xRace	-.017[d]	-.229	.819	-.015	.711
	MGRS0xSES0	.019[d]	.291	.771	.019	.890
	MGRS0xIdeology0	.011[d]	.177	.860	.012	.950

a. Dependent Variable: Psychology_Tot

b. Predictors in the Model: (Constant), Ideology0

c. Predictors in the Model: (Constant), Ideology0, SES0, GenderCC, RaceCC

d. Predictors in the Model: (Constant), Ideology0, SES0, GenderCC, RaceCC, MGRS0, MRN0

```
REGRESSION
  /MISSING LISTWISE
  /STATISTICS COEFF OUTS R ANOVA CHANGE ZPP
  /CRITERIA=PIN(.05) POUT(.10)
  /NOORIGIN
  /DEPENDENT TrumpApproval
  /METHOD=ENTER Ideology0
  /METHOD=ENTER GenderCC RaceCC SES0
  /METHOD=ENTER MGRS0 MRN0
  /METHOD=ENTER MRN0xRace MRN0xSES0 MRN0xGender MRN0xIdeology0 MRN0xMGRS0 MGRS0xGender MG
RS0xRace MGRS0xSES0 MGRS0xIdeology0.
```

Regression

Notes

Output Created		15-DEC-2021 13:07:59
Comments		
Input	Data	C:\Users\njs5478\Dropbox\HM and COVID\0. Revise and Resubmit\2. R and R Data\Study 1b\Study1b_Data.sav
	Active Dataset	DataSet1
	Filter	<none>
	Weight	<none>
	Split File	<none>
	N of Rows in Working Data File	241
Missing Value Handling	Definition of Missing	User-defined missing values are treated as missing.
	Cases Used	Statistics are based on cases with no missing values for any variable used.
Syntax		REGRESSION<br>/MISSING LISTWISE<br>/STATISTICS COEFF OUTS R ANOVA CHANGE ZPP<br>/CRITERIA=PIN(.05) POUT(.10)<br>/NOORIGIN<br>/DEPENDENT TrumpApproval<br>/METHOD=ENTER Ideology0<br>/METHOD=ENTER GenderCC RaceCC SES0<br>/METHOD=ENTER MGRS0 MRN0<br>/METHOD=ENTER MRN0xRace MRN0xSES0 MRN0xGender MRN0xIdeology0 MRN0xMGRS0 MGRS0xGender MGRS0xRace MGRS0xSES0 MGRS0xIdeology0.

Notes

Resources	Processor Time	00:00:00.03
	Elapsed Time	00:00:00.05
	Memory Required	52240 bytes
	Additional Memory Required for Residual Plots	0 bytes

Variables Entered/Removed[a]

Model	Variables Entered	Variables Removed	Method
1	Ideology0[b]	.	Enter
2	SES0, GenderCC, RaceCC[b]	.	Enter
3	MGRS0, MRN0[b]	.	Enter
4	MRN0xMGRS0, MRN0xSES0, MRN0xIdeology0, MGRS0xSES0, MGRS0xGender, MGRS0xRace, MRN0xRace, MRN0xGender, MGRS0xIdeol...[b]	.	Enter

a. Dependent Variable: Do you approve or disapprove of the way Donald Trump is handling his job as President?

b. All requested variables entered.

Model Summary

Model	R	R Square	Adjusted R Square	Std. Error of the Estimate	Change Statistics				
					R Square Change	F Change	df1	df2	Sig. F Change
1	.805[a]	.647	.646	1.247	.647	429.448	1	234	.000
2	.813[b]	.662	.656	1.229	.014	3.243	3	231	.023
3	.819[c]	.670	.662	1.219	.009	3.062	2	229	.049
4	.827[d]	.684	.663	1.216	.014	1.092	9	220	.370

a. Predictors: (Constant), Ideology0

b. Predictors: (Constant), Ideology0, SES0, GenderCC, RaceCC

c. Predictors: (Constant), Ideology0, SES0, GenderCC, RaceCC, MGRS0, MRN0

d. Predictors: (Constant), Ideology0, SES0, GenderCC, RaceCC, MGRS0, MRN0, MRN0xMGRS0, MRN0xSES0, MRN0xIdeology0, MGRS0xSES0, MGRS0xGender, MGRS0xRace, MRN0xRace, MRN0xGender, MGRS0xIdeology0

ANOVA[a]

Model		Sum of Squares	df	Mean Square	F	Sig.
1	Regression	667.791	1	667.791	429.448	.000[b]
	Residual	363.870	234	1.555		
	Total	1031.661	235			
2	Regression	682.496	4	170.624	112.881	.000[c]
	Residual	349.165	231	1.512		
	Total	1031.661	235			
3	Regression	691.591	6	115.265	77.618	.000[d]
	Residual	340.070	229	1.485		
	Total	1031.661	235			
4	Regression	706.133	15	47.076	31.815	.000[e]
	Residual	325.528	220	1.480		
	Total	1031.661	235			

a. Dependent Variable: Do you approve or disapprove of the way Donald Trump is handling his job as President?

b. Predictors: (Constant), Ideology0

c. Predictors: (Constant), Ideology0, SES0, GenderCC, RaceCC

d. Predictors: (Constant), Ideology0, SES0, GenderCC, RaceCC, MGRS0, MRN0

e. Predictors: (Constant), Ideology0, SES0, GenderCC, RaceCC, MGRS0, MRN0, MRN0xMGRS0, MRN0xSES0, MRN0xIdeology0, MGRS0xSES0, MGRS0xGender, MGRS0xRace, MRN0xRace, MRN0xGender, MGRS0xIdeology0

Coefficients[a]

Model		Unstandardized Coefficients B	Unstandardized Coefficients Std. Error	Standardized Coefficients Beta	t	Sig.
1	(Constant)	3.149	.081		38.787	.000
	Ideology0	1.059	.051	.805	20.723	.000
2	(Constant)	3.058	.093		32.749	.000
	Ideology0	1.036	.055	.787	18.728	.000
	GenderCC	-.011	.083	-.005	-.128	.898
	RaceCC	.178	.097	.074	1.828	.069
	SES0	-.252	.099	-.098	-2.541	.012
3	(Constant)	3.039	.093		32.736	.000
	Ideology0	.968	.064	.735	15.150	.000
	GenderCC	-.044	.097	-.021	-.457	.648
	RaceCC	.218	.098	.091	2.226	.027
	SES0	-.268	.099	-.104	-2.699	.007
	MGRS0	.117	.139	.036	.839	.402
	MRN0	.239	.124	.099	1.923	.056
4	(Constant)	3.077	.124		24.748	.000
	Ideology0	.938	.067	.713	14.084	.000
	GenderCC	-.003	.102	-.002	-.033	.974
	RaceCC	.194	.102	.081	1.893	.060
	SES0	-.268	.102	-.104	-2.637	.009
	MGRS0	.279	.182	.086	1.535	.126
	MRN0	.233	.139	.097	1.678	.095
	MRN0xRace	.012	.117	.005	.104	.917
	MRN0xSES0	-.026	.108	-.010	-.244	.807
	MRN0xGender	.023	.131	.009	.173	.863
	MRN0xIdeology0	.077	.060	.058	1.273	.204
	MRN0xMGRS0	-.436	.206	-.134	-2.118	.035
	MGRS0xGender	.223	.167	.065	1.332	.184
	MGRS0xRace	-.083	.159	-.026	-.521	.603
	MGRS0xSES0	.102	.169	.025	.602	.548
	MGRS0xIdeology0	.213	.109	.114	1.950	.052

Coefficients[a]

Model		Correlations Zero-order	Partial	Part
1	(Constant)			
	Ideology0	.805	.805	.805
2	(Constant)			
	Ideology0	.805	.776	.717
	GenderCC	.203	-.008	-.005
	RaceCC	.326	.119	.070
	SES0	-.049	-.165	-.097
3	(Constant)			
	Ideology0	.805	.708	.575
	GenderCC	.203	-.030	-.017
	RaceCC	.326	.146	.084
	SES0	-.049	-.176	-.102
	MGRS0	.008	.055	.032
	MRN0	.482	.126	.073
4	(Constant)			
	Ideology0	.805	.689	.533
	GenderCC	.203	-.002	-.001
	RaceCC	.326	.127	.072
	SES0	-.049	-.175	-.100
	MGRS0	.008	.103	.058
	MRN0	.482	.112	.064
	MRN0xRace	.361	.007	.004
	MRN0xSES0	-.133	-.016	-.009
	MRN0xGender	.131	.012	.007
	MRN0xIdeology0	.095	.086	.048
	MRN0xMGRS0	-.076	-.141	-.080
	MGRS0xGender	.089	.089	.050
	MGRS0xRace	.076	-.035	-.020
	MGRS0xSES0	-.048	.041	.023
	MGRS0xIdeology0	.086	.130	.074

a. Dependent Variable: Do you approve or disapprove of the way Donald Trump is handling his job as President?

Excluded Variables[a]

Model		Beta In	t	Sig.	Partial Correlation	Collinearity Statistics Tolerance
1	GenderCC	-.013[b]	-.317	.752	-.021	.929
	RaceCC	.073[b]	1.778	.077	.116	.894
	SES0	-.097[b]	-2.509	.013	-.162	.997
	MGRS0	.058[b]	1.487	.138	.097	.996
	MRN0	.069[b]	1.505	.134	.098	.710
	MRN0xRace	.049[b]	1.170	.243	.076	.843
	MRN0xSES0	-.030[b]	-.757	.450	-.050	.983
	MRN0xGender	.053[b]	1.355	.177	.088	.990
	MRN0xIdeology0	.068[b]	1.767	.079	.115	.999
	MRN0xMGRS0	-.020[b]	-.505	.614	-.033	.995
	MGRS0xGender	.031[b]	.783	.434	.051	.995
	MGRS0xRace	.028[b]	.721	.472	.047	.996
	MGRS0xSES0	.008[b]	.217	.829	.014	.995
	MGRS0xIdeology0	.028[b]	.728	.468	.048	.995
2	MGRS0	.063[c]	1.549	.123	.102	.870
	MRN0	.114[c]	2.330	.021	.152	.605
	MRN0xRace	.064[c]	1.509	.133	.099	.812
	MRN0xSES0	-.028[c]	-.729	.467	-.048	.979
	MRN0xGender	.066[c]	1.699	.091	.111	.972
	MRN0xIdeology0	.050[c]	1.277	.203	.084	.961
	MRN0xMGRS0	-.033[c]	-.859	.391	-.057	.965
	MGRS0xGender	.037[c]	.951	.343	.063	.986
	MGRS0xRace	.026[c]	.680	.497	.045	.975
	MGRS0xSES0	-.009[c]	-.226	.822	-.015	.960
	MGRS0xIdeology0	.017[c]	.432	.666	.028	.981
3	MRN0xRace	.038[d]	.848	.397	.056	.735
	MRN0xSES0	-.011[d]	-.288	.774	-.019	.946
	MRN0xGender	.050[d]	1.278	.203	.084	.940
	MRN0xIdeology0	.066[d]	1.690	.092	.111	.935
	MRN0xMGRS0	-.029[d]	-.751	.453	-.050	.961
	MGRS0xGender	.036[d]	.909	.364	.060	.944

Excluded Variables[a]

Model		Beta In	t	Sig.	Partial Correlation	Collinearity Statistics Tolerance
	MGRS0xRace	.002[d]	.052	.959	.003	.712
	MGRS0xSES0	.013[d]	.319	.750	.021	.890
	MGRS0xIdeology0	.034[d]	.875	.382	.058	.950

a. Dependent Variable: Do you approve or disapprove of the way Donald Trump is handling his job as President?

b. Predictors in the Model: (Constant), Ideology0

c. Predictors in the Model: (Constant), Ideology0, SES0, GenderCC, RaceCC

d. Predictors in the Model: (Constant), Ideology0, SES0, GenderCC, RaceCC, MGRS0, MRN0

```
REGRESSION
  /MISSING LISTWISE
  /STATISTICS COEFF OUTS R ANOVA CHANGE ZPP
  /CRITERIA=PIN(.05) POUT(.10)
  /NOORIGIN
  /DEPENDENT TrumpX
  /METHOD=ENTER Ideology0
  /METHOD=ENTER GenderCC RaceCC SES0
  /METHOD=ENTER MGRS0 MRN0
  /METHOD=ENTER MRN0xRace MRN0xSES0 MRN0xGender MRN0xIdeology0 MRN0xMGRS0 MGRS0xGender MG
RS0xRace MGRS0xSES0 MGRS0xIdeology0.
```

Regression

Notes

Output Created		15-DEC-2021 13:07:59
Comments		
Input	Data	C: \Users\njs5478\Dropbox\HM and COVID\0. Revise and Resubmit\2. R and R Data\Study 1b\Study1b_Data.sav
	Active Dataset	DataSet1
	Filter	<none>
	Weight	<none>
	Split File	<none>
	N of Rows in Working Data File	241
Missing Value Handling	Definition of Missing	User-defined missing values are treated as missing.
	Cases Used	Statistics are based on cases with no missing values for any variable used.
Syntax		REGRESSION /MISSING LISTWISE /STATISTICS COEFF OUTS R ANOVA CHANGE ZPP /CRITERIA=PIN(.05) POUT(.10) /NOORIGIN /DEPENDENT TrumpX /METHOD=ENTER Ideology0 /METHOD=ENTER GenderCC RaceCC SES0 /METHOD=ENTER MGRS0 MRN0 /METHOD=ENTER MRN0xRace MRN0xSES0 MRN0xGender MRN0xIdeology0 MRN0xMGRS0 MGRS0xGender MGRS0xRace MGRS0xSES0 MGRS0xIdeology0.
Resources	Processor Time	00:00:00.02
	Elapsed Time	00:00:00.03

Notes

	Memory Required	52240 bytes
	Additional Memory Required for Residual Plots	0 bytes

Variables Entered/Removed[a]

Model	Variables Entered	Variables Removed	Method
1	Ideology0[b]	.	Enter
2	SES0, GenderCC, RaceCC[b]	.	Enter
3	MGRS0, MRN0[b]	.	Enter
4	MRN0xMGRS0, MRN0xSES0, MRN0xIdeology0, MGRS0xSES0, MGRS0xGender, MGRS0xRace, MRN0xRace, MRN0xGender, MGRS0xIdeol...[b]	.	Enter

a. Dependent Variable: TrumpX

b. All requested variables entered.

Model Summary

Model	R	R Square	Adjusted R Square	Std. Error of the Estimate	Change Statistics		
					R Square Change	F Change	df1
1	.780[a]	.609	.607	1.28725	.609	361.585	1
2	.786[b]	.617	.611	1.28193	.008	1.643	3
3	.793[c]	.628	.619	1.26875	.011	3.391	2
4	.807[d]	.651	.627	1.25503	.022	1.555	9

Model Summary

Model	Change Statistics	
	df2	Sig. F Change
1	232	.000
2	229	.180
3	227	.035
4	218	.131

a. Predictors: (Constant), Ideology0

b. Predictors: (Constant), Ideology0, SES0, GenderCC, RaceCC

c. Predictors: (Constant), Ideology0, SES0, GenderCC, RaceCC, MGRS0, MRN0

d. Predictors: (Constant), Ideology0, SES0, GenderCC, RaceCC, MGRS0, MRN0, MRN0xMGRS0, MRN0xSES0, MRN0xIdeology0, MGRS0xSES0, MGRS0xGender, MGRS0xRace, MRN0xRace, MRN0xGender, MGRS0xIdeology0

ANOVA[a]

Model		Sum of Squares	df	Mean Square	F	Sig.
1	Regression	599.148	1	599.148	361.585	.000[b]
	Residual	384.425	232	1.657		
	Total	983.573	233			
2	Regression	607.246	4	151.812	92.380	.000[c]
	Residual	376.326	229	1.643		
	Total	983.573	233			
3	Regression	618.163	6	103.027	64.003	.000[d]
	Residual	365.410	227	1.610		
	Total	983.573	233			
4	Regression	640.200	15	42.680	27.097	.000[e]
	Residual	343.373	218	1.575		
	Total	983.573	233			

a. Dependent Variable: TrumpX

b. Predictors: (Constant), Ideology0

c. Predictors: (Constant), Ideology0, SES0, GenderCC, RaceCC

d. Predictors: (Constant), Ideology0, SES0, GenderCC, RaceCC, MGRS0, MRN0

e. Predictors: (Constant), Ideology0, SES0, GenderCC, RaceCC, MGRS0, MRN0, MRN0xMGRS0, MRN0xSES0, MRN0xIdeology0, MGRS0xSES0, MGRS0xGender, MGRS0xRace, MRN0xRace, MRN0xGender, MGRS0xIdeology0

Coefficients[a]

Model		Unstandardized Coefficients B	Unstandardized Coefficients Std. Error	Standardized Coefficients Beta	t	Sig.
1	(Constant)	3.067	.084		36.447	.000
	Ideology0	1.008	.053	.780	19.015	.000
2	(Constant)	3.009	.098		30.833	.000
	Ideology0	.993	.058	.769	17.099	.000
	GenderCC	.003	.087	.001	.035	.972
	RaceCC	.114	.102	.048	1.118	.265
	SES0	-.200	.104	-.079	-1.935	.054
3	(Constant)	2.989	.097		30.843	.000
	Ideology0	.921	.067	.713	13.818	.000
	GenderCC	-.026	.102	-.013	-.257	.798
	RaceCC	.157	.102	.067	1.541	.125
	SES0	-.216	.104	-.086	-2.088	.038
	MGRS0	.148	.146	.047	1.014	.312
	MRN0	.250	.130	.106	1.925	.056
4	(Constant)	2.967	.129		22.913	.000
	Ideology0	.892	.069	.690	12.922	.000
	GenderCC	.025	.106	.012	.234	.815
	RaceCC	.152	.106	.065	1.440	.151
	SES0	-.215	.105	-.085	-2.047	.042
	MGRS0	.386	.188	.122	2.057	.041
	MRN0	.236	.144	.100	1.645	.102
	MRN0xRace	.003	.121	.001	.021	.983
	MRN0xSES0	.065	.112	.025	.580	.562
	MRN0xGender	.142	.138	.054	1.027	.306
	MRN0xIdeology0	.065	.063	.050	1.028	.305
	MRN0xMGRS0	-.457	.212	-.143	-2.149	.033
	MGRS0xGender	.201	.174	.060	1.157	.249
	MGRS0xRace	-.205	.164	-.065	-1.249	.213
	MGRS0xSES0	.073	.174	.019	.417	.677
	MGRS0xIdeology0	.279	.113	.153	2.465	.014

Coefficients[a]

Model		Correlations Zero-order	Partial	Part
1	(Constant)			
	Ideology0	.780	.780	.780
2	(Constant)			
	Ideology0	.780	.749	.699
	GenderCC	.212	.002	.001
	RaceCC	.294	.074	.046
	SES0	-.031	-.127	-.079
3	(Constant)			
	Ideology0	.780	.676	.559
	GenderCC	.212	-.017	-.010
	RaceCC	.294	.102	.062
	SES0	-.031	-.137	-.084
	MGRS0	.022	.067	.041
	MRN0	.481	.127	.078
4	(Constant)			
	Ideology0	.780	.659	.517
	GenderCC	.212	.016	.009
	RaceCC	.294	.097	.058
	SES0	-.031	-.137	-.082
	MGRS0	.022	.138	.082
	MRN0	.481	.111	.066
	MRN0xRace	.351	.001	.001
	MRN0xSES0	-.103	.039	.023
	MRN0xGender	.175	.069	.041
	MRN0xIdeology0	.095	.069	.041
	MRN0xMGRS0	-.072	-.144	-.086
	MGRS0xGender	.091	.078	.046
	MGRS0xRace	.061	-.084	-.050
	MGRS0xSES0	-.061	.028	.017
	MGRS0xIdeology0	.093	.165	.099

a. Dependent Variable: TrumpX

Excluded Variables[a]

Model		Beta In	t	Sig.	Partial Correlation	Collinearity Statistics Tolerance
1	GenderCC	-.004[b]	-.094	.925	-.006	.923
	RaceCC	.047[b]	1.084	.280	.071	.896
	SES0	-.079[b]	-1.921	.056	-.125	.996
	MGRS0	.067[b]	1.644	.102	.108	.997
	MRN0	.088[b]	1.816	.071	.119	.712
	MRN0xRace	.051[b]	1.141	.255	.075	.845
	MRN0xSES0	-.004[b]	-.100	.920	-.007	.984
	MRN0xGender	.092[b]	2.238	.026	.146	.988
	MRN0xIdeology0	.072[b]	1.764	.079	.115	.999
	MRN0xMGRS0	-.019[b]	-.473	.637	-.031	.996
	MGRS0xGender	.039[b]	.945	.345	.062	.996
	MGRS0xRace	.012[b]	.293	.770	.019	.996
	MGRS0xSES0	-.004[b]	-.109	.913	-.007	.995
	MGRS0xIdeology0	.034[b]	.834	.405	.055	.994
2	MGRS0	.076[c]	1.744	.083	.115	.866
	MRN0	.125[c]	2.398	.017	.157	.606
	MRN0xRace	.061[c]	1.339	.182	.088	.814
	MRN0xSES0	-.003[c]	-.084	.933	-.006	.980
	MRN0xGender	.102[c]	2.476	.014	.162	.969
	MRN0xIdeology0	.059[c]	1.417	.158	.093	.961
	MRN0xMGRS0	-.031[c]	-.750	.454	-.050	.966
	MGRS0xGender	.045[c]	1.083	.280	.072	.987
	MGRS0xRace	.012[c]	.278	.781	.018	.973
	MGRS0xSES0	-.019[c]	-.445	.657	-.029	.959
	MGRS0xIdeology0	.025[c]	.617	.538	.041	.981
3	MRN0xRace	.031[d]	.651	.516	.043	.737
	MRN0xSES0	.017[d]	.398	.691	.026	.947
	MRN0xGender	.085[d]	2.045	.042	.135	.936
	MRN0xIdeology0	.077[d]	1.850	.066	.122	.934
	MRN0xMGRS0	-.027[d]	-.644	.520	-.043	.962
	MGRS0xGender	.045[d]	1.074	.284	.071	.947

Excluded Variables[a]

Model		Beta In	t	Sig.	Partial Correlation	Collinearity Statistics Tolerance
	MGRS0xRace	-.026[d]	-.536	.592	-.036	.713
	MGRS0xSES0	.006[d]	.141	.888	.009	.890
	MGRS0xIdeology0	.045[d]	1.096	.274	.073	.949

a. Dependent Variable: TrumpX

b. Predictors in the Model: (Constant), Ideology0

c. Predictors in the Model: (Constant), Ideology0, SES0, GenderCC, RaceCC

d. Predictors in the Model: (Constant), Ideology0, SES0, GenderCC, RaceCC, MGRS0, MRN0

```
REGRESSION
  /MISSING LISTWISE
  /STATISTICS COEFF OUTS R ANOVA CHANGE ZPP
  /CRITERIA=PIN(.05) POUT(.10)
  /NOORIGIN
  /DEPENDENT BidenX
  /METHOD=ENTER Ideology0
  /METHOD=ENTER GenderCC RaceCC SES0
  /METHOD=ENTER MGRS0 MRN0
  /METHOD=ENTER MRN0xRace MRN0xSES0 MRN0xGender MRN0xIdeology0 MRN0xMGRS0 MGRS0xGender MG
RS0xRace MGRS0xSES0 MGRS0xIdeology0.
```

Regression

Notes

Output Created		15-DEC-2021 13:07:59
Comments		
Input	Data	C: \Users\njs5478\Dropbox\HM and COVID\0. Revise and Resubmit\2. R and R Data\Study 1b\Study1b_Data.sav
	Active Dataset	DataSet1
	Filter	<none>
	Weight	<none>
	Split File	<none>
	N of Rows in Working Data File	241
Missing Value Handling	Definition of Missing	User-defined missing values are treated as missing.
	Cases Used	Statistics are based on cases with no missing values for any variable used.
Syntax		REGRESSION<br>/MISSING LISTWISE<br>/STATISTICS COEFF OUTS R ANOVA CHANGE ZPP<br>/CRITERIA=PIN(.05) POUT(.10)<br>/NOORIGIN<br>/DEPENDENT BidenX<br>/METHOD=ENTER Ideology0<br>/METHOD=ENTER GenderCC RaceCC SES0<br>/METHOD=ENTER MGRS0 MRN0<br>/METHOD=ENTER MRN0xRace MRN0xSES0 MRN0xGender MRN0xIdeology0 MRN0xMGRS0 MGRS0xGender MGRS0xRace MGRS0xSES0 MGRS0xIdeology0.
Resources	Processor Time	00:00:00.02
	Elapsed Time	00:00:00.02

Notes

	Memory Required	52240 bytes
	Additional Memory Required for Residual Plots	0 bytes

Variables Entered/Removed[a]

Model	Variables Entered	Variables Removed	Method
1	Ideology0[b]	.	Enter
2	SES0, GenderCC, RaceCC[b]	.	Enter
3	MGRS0, MRN0[b]	.	Enter
4	MRN0xMGRS0, MRN0xSES0, MRN0xIdeology0, MGRS0xSES0, MGRS0xGender, MGRS0xRace, MRN0xRace, MRN0xGender, MGRS0xIdeol...[b]	.	Enter

a. Dependent Variable: BidenX

b. All requested variables entered.

Model Summary

Model	R	R Square	Adjusted R Square	Std. Error of the Estimate	Change Statistics		
					R Square Change	F Change	df1
1	.700[a]	.491	.488	1.28443	.491	218.664	1
2	.706[b]	.498	.489	1.28361	.007	1.097	3
3	.708[c]	.501	.488	1.28556	.003	.660	2
4	.723[d]	.523	.490	1.28270	.022	1.110	9

Model Summary

Model	Change Statistics	
	df2	Sig. F Change
1	227	.000
2	224	.351
3	222	.518
4	213	.357

a. Predictors: (Constant), Ideology0

b. Predictors: (Constant), Ideology0, SES0, GenderCC, RaceCC

c. Predictors: (Constant), Ideology0, SES0, GenderCC, RaceCC, MGRS0, MRN0

d. Predictors: (Constant), Ideology0, SES0, GenderCC, RaceCC, MGRS0, MRN0, MRN0xMGRS0, MRN0xSES0, MRN0xIdeology0, MGRS0xSES0, MGRS0xGender, MGRS0xRace, MRN0xRace, MRN0xGender, MGRS0xIdeology0

ANOVA[a]

Model		Sum of Squares	df	Mean Square	F	Sig.
1	Regression	360.744	1	360.744	218.664	.000[b]
	Residual	374.496	227	1.650		
	Total	735.240	228			
2	Regression	366.167	4	91.542	55.559	.000[c]
	Residual	369.073	224	1.648		
	Total	735.240	228			
3	Regression	368.348	6	61.391	37.147	.000[d]
	Residual	366.892	222	1.653		
	Total	735.240	228			
4	Regression	384.789	15	25.653	15.591	.000[e]
	Residual	350.452	213	1.645		
	Total	735.240	228			

a. Dependent Variable: BidenX

b. Predictors: (Constant), Ideology0

c. Predictors: (Constant), Ideology0, SES0, GenderCC, RaceCC

d. Predictors: (Constant), Ideology0, SES0, GenderCC, RaceCC, MGRS0, MRN0

e. Predictors: (Constant), Ideology0, SES0, GenderCC, RaceCC, MGRS0, MRN0, MRN0xMGRS0, MRN0xSES0, MRN0xIdeology0, MGRS0xSES0, MGRS0xGender, MGRS0xRace, MRN0xRace, MRN0xGender, MGRS0xIdeology0

Coefficients[a]

Model		Unstandardized Coefficients B	Std. Error	Standardized Coefficients Beta	t	Sig.
1	(Constant)	4.483	.085		52.811	.000
	Ideology0	-.783	.053	-.700	-14.787	.000
2	(Constant)	4.446	.099		45.027	.000
	Ideology0	-.798	.058	-.714	-13.693	.000
	GenderCC	-.026	.088	-.014	-.293	.770
	RaceCC	.082	.103	.040	.800	.424
	SES0	.165	.105	.075	1.573	.117
3	(Constant)	4.455	.099		44.875	.000
	Ideology0	-.761	.068	-.681	-11.267	.000
	GenderCC	.001	.105	.001	.013	.989
	RaceCC	.063	.104	.031	.606	.545
	SES0	.176	.106	.079	1.656	.099
	MGRS0	-.029	.149	-.011	-.194	.846
	MRN0	-.132	.132	-.065	-1.000	.319
4	(Constant)	4.521	.134		33.856	.000
	Ideology0	-.741	.071	-.663	-10.498	.000
	GenderCC	-.032	.110	-.018	-.289	.773
	RaceCC	.048	.110	.024	.440	.660
	SES0	.157	.108	.071	1.449	.149
	MGRS0	-.223	.196	-.081	-1.136	.257
	MRN0	-.110	.148	-.054	-.743	.458
	MRN0xRace	.005	.126	.002	.038	.969
	MRN0xSES0	-.023	.115	-.010	-.203	.839
	MRN0xGender	-.242	.143	-.107	-1.698	.091
	MRN0xIdeology0	.009	.064	.008	.146	.884
	MRN0xMGRS0	.192	.218	.070	.883	.378
	MGRS0xGender	-.017	.180	-.006	-.097	.923
	MGRS0xRace	.179	.171	.065	1.046	.297
	MGRS0xSES0	-.159	.180	-.047	-.883	.378
	MGRS0xIdeology0	-.270	.116	-.171	-2.333	.021

Coefficients[a]

Model		Correlations Zero-order	Partial	Part
1	(Constant)			
	Ideology0	-.700	-.700	-.700
2	(Constant)			
	Ideology0	-.700	-.675	-.648
	GenderCC	-.208	-.020	-.014
	RaceCC	-.190	.053	.038
	SES0	.034	.105	.074
3	(Constant)			
	Ideology0	-.700	-.603	-.534
	GenderCC	-.208	.001	.001
	RaceCC	-.190	.041	.029
	SES0	.034	.110	.079
	MGRS0	.011	-.013	-.009
	MRN0	-.420	-.067	-.047
4	(Constant)			
	Ideology0	-.700	-.584	-.497
	GenderCC	-.208	-.020	-.014
	RaceCC	-.190	.030	.021
	SES0	.034	.099	.069
	MGRS0	.011	-.078	-.054
	MRN0	-.420	-.051	-.035
	MRN0xRace	-.304	.003	.002
	MRN0xSES0	.091	-.014	-.010
	MRN0xGender	-.162	-.116	-.080
	MRN0xIdeology0	-.057	.010	.007
	MRN0xMGRS0	.012	.060	.042
	MGRS0xGender	-.083	-.007	-.005
	MGRS0xRace	-.032	.072	.050
	MGRS0xSES0	.021	-.060	-.042
	MGRS0xIdeology0	-.130	-.158	-.110

a. Dependent Variable: BidenX

Excluded Variables[a]

Model		Beta In	t	Sig.	Partial Correlation	Collinearity Statistics Tolerance
1	GenderCC	-.014[b]	-.289	.773	-.019	.922
	RaceCC	.043[b]	.865	.388	.057	.893
	SES0	.076[b]	1.600	.111	.106	.997
	MGRS0	-.028[b]	-.584	.560	-.039	.997
	MRN0	-.063[b]	-1.115	.266	-.074	.712
	MRN0xRace	-.031[b]	-.606	.545	-.040	.843
	MRN0xSES0	.001[b]	.020	.984	.001	.984
	MRN0xGender	-.084[b]	-1.774	.077	-.117	.987
	MRN0xIdeology0	-.035[b]	-.738	.461	-.049	.999
	MRN0xMGRS0	-.033[b]	-.698	.486	-.046	.996
	MGRS0xGender	-.035[b]	-.734	.464	-.049	.995
	MGRS0xRace	.012[b]	.248	.804	.017	.996
	MGRS0xSES0	-.028[b]	-.589	.556	-.039	.995
	MGRS0xIdeology0	-.077[b]	-1.626	.105	-.108	.994
2	MGRS0	-.029[c]	-.566	.572	-.038	.867
	MRN0	-.069[c]	-1.135	.258	-.076	.608
	MRN0xRace	-.022[c]	-.427	.670	-.029	.810
	MRN0xSES0	.003[c]	.063	.950	.004	.980
	MRN0xGender	-.084[c]	-1.756	.080	-.117	.969
	MRN0xIdeology0	-.031[c]	-.650	.517	-.043	.961
	MRN0xMGRS0	-.025[c]	-.512	.609	-.034	.968
	MGRS0xGender	-.044[c]	-.915	.361	-.061	.985
	MGRS0xRace	.008[c]	.172	.863	.012	.970
	MGRS0xSES0	-.022[c]	-.450	.653	-.030	.962
	MGRS0xIdeology0	-.075[c]	-1.576	.117	-.105	.981
3	MRN0xRace	-.005[d]	-.086	.932	-.006	.733
	MRN0xSES0	-.007[d]	-.143	.887	-.010	.948
	MRN0xGender	-.076[d]	-1.557	.121	-.104	.932
	MRN0xIdeology0	-.042[d]	-.847	.398	-.057	.934
	MRN0xMGRS0	-.028[d]	-.575	.566	-.039	.964
	MGRS0xGender	-.042[d]	-.863	.389	-.058	.941

Excluded Variables[a]

Model		Beta In	t	Sig.	Partial Correlation	Collinearity Statistics Tolerance
	MGRS0xRace	.025[d]	.446	.656	.030	.701
	MGRS0xSES0	-.035[d]	-.698	.486	-.047	.885
	MGRS0xIdeology0	-.087[d]	-1.790	.075	-.120	.949

a. Dependent Variable: BidenX

b. Predictors in the Model: (Constant), Ideology0

c. Predictors in the Model: (Constant), Ideology0, SES0, GenderCC, RaceCC

d. Predictors in the Model: (Constant), Ideology0, SES0, GenderCC, RaceCC, MGRS0, MRN0

```
 REGRESSION
  /MISSING LISTWISE
  /STATISTICS COEFF OUTS R ANOVA CHANGE ZPP
  /CRITERIA=PIN(.05) POUT(.10)
  /NOORIGIN
  /DEPENDENT PelosiX
  /METHOD=ENTER Ideology0
  /METHOD=ENTER GenderCC RaceCC SES0
  /METHOD=ENTER MGRS0 MRN0
  /METHOD=ENTER MRN0xRace MRN0xSES0 MRN0xGender MRN0xIdeology0 MRN0xMGRS0 MGRS0xGender MG
RS0xRace MGRS0xSES0 MGRS0xIdeology0.
```

Regression

Notes

Output Created		15-DEC-2021 13:07:59
Comments		
Input	Data	C: \Users\njs5478\Dropbox\HM and COVID\0. Revise and Resubmit\2. R and R Data\Study 1b\Study1b_Data.sav
	Active Dataset	DataSet1
	Filter	<none>
	Weight	<none>
	Split File	<none>
	N of Rows in Working Data File	241
Missing Value Handling	Definition of Missing	User-defined missing values are treated as missing.
	Cases Used	Statistics are based on cases with no missing values for any variable used.
Syntax		REGRESSION<br>/MISSING LISTWISE<br>/STATISTICS COEFF OUTS R ANOVA CHANGE ZPP<br>/CRITERIA=PIN(.05) POUT(.10)<br>/NOORIGIN<br>/DEPENDENT PelosiX<br>/METHOD=ENTER Ideology0<br>/METHOD=ENTER GenderCC RaceCC SES0<br>/METHOD=ENTER MGRS0 MRN0<br>/METHOD=ENTER MRN0xRace MRN0xSES0 MRN0xGender MRN0xIdeology0 MRN0xMGRS0 MGRS0xGender MGRS0xRace MGRS0xSES0 MGRS0xIdeology0.
Resources	Processor Time	00:00:00.02
	Elapsed Time	00:00:00.02

Notes

	Memory Required	52240 bytes
	Additional Memory Required for Residual Plots	0 bytes

Variables Entered/Removed[a]

Model	Variables Entered	Variables Removed	Method
1	Ideology0[b]	.	Enter
2	SES0, GenderCC, RaceCC[b]	.	Enter
3	MGRS0, MRN0[b]	.	Enter
4	MRN0xMGRS0, MRN0xSES0, MRN0xIdeology0, MGRS0xGender, MGRS0xSES0, MGRS0xRace, MRN0xGender, MRN0xRace, MGRS0xIdeology0[b]	.	Enter

a. Dependent Variable: PelosiX

b. All requested variables entered.

Model Summary

Model	R	R Square	Adjusted R Square	Std. Error of the Estimate	Change Statistics		
					R Square Change	F Change	df1
1	.638[a]	.407	.404	1.26248	.407	135.237	1
2	.652[b]	.426	.414	1.25213	.019	2.091	3
3	.662[c]	.438	.420	1.24516	.012	2.088	2
4	.677[d]	.458	.414	1.25177	.021	.775	9

Model Summary

Model	Change Statistics	
	df2	Sig. F Change
1	197	.000
2	194	.103
3	192	.127
4	183	.639

a. Predictors: (Constant), Ideology0

b. Predictors: (Constant), Ideology0, SES0, GenderCC, RaceCC

c. Predictors: (Constant), Ideology0, SES0, GenderCC, RaceCC, MGRS0, MRN0

d. Predictors: (Constant), Ideology0, SES0, GenderCC, RaceCC, MGRS0, MRN0, MRN0xMGRS0, MRN0xSES0, MRN0xIdeology0, MGRS0xGender, MGRS0xSES0, MGRS0xRace, MRN0xGender, MRN0xRace, MGRS0xIdeology0

ANOVA[a]

Model		Sum of Squares	df	Mean Square	F	Sig.
1	Regression	215.548	1	215.548	135.237	.000[b]
	Residual	313.989	197	1.594		
	Total	529.538	198			
2	Regression	225.381	4	56.345	35.939	.000[c]
	Residual	304.157	194	1.568		
	Total	529.538	198			
3	Regression	231.855	6	38.643	24.924	.000[d]
	Residual	297.682	192	1.550		
	Total	529.538	198			
4	Regression	242.791	15	16.186	10.330	.000[e]
	Residual	286.747	183	1.567		
	Total	529.538	198			

a. Dependent Variable: PelosiX

b. Predictors: (Constant), Ideology0

c. Predictors: (Constant), Ideology0, SES0, GenderCC, RaceCC

d. Predictors: (Constant), Ideology0, SES0, GenderCC, RaceCC, MGRS0, MRN0

e. Predictors: (Constant), Ideology0, SES0, GenderCC, RaceCC, MGRS0, MRN0, MRN0xMGRS0, MRN0xSES0, MRN0xIdeology0, MGRS0xGender, MGRS0xSES0, MGRS0xRace, MRN0xGender, MRN0xRace, MGRS0xIdeology0

Coefficients[a]

Model		Unstandardized Coefficients B	Unstandardized Coefficients Std. Error	Standardized Coefficients Beta	t	Sig.
1	(Constant)	3.574	.090		39.906	.000
	Ideology0	-.657	.056	-.638	-11.629	.000
2	(Constant)	3.496	.105		33.436	.000
	Ideology0	-.651	.061	-.632	-10.702	.000
	GenderCC	-.170	.092	-.104	-1.846	.066
	RaceCC	.158	.109	.083	1.459	.146
	SES0	-.082	.110	-.041	-.747	.456
3	(Constant)	3.515	.104		33.664	.000
	Ideology0	-.599	.070	-.582	-8.546	.000
	GenderCC	-.162	.108	-.099	-1.498	.136
	RaceCC	.123	.109	.064	1.121	.263
	SES0	-.077	.110	-.038	-.704	.482
	MGRS0	-.176	.156	-.069	-1.129	.260
	MRN0	-.172	.141	-.090	-1.217	.225
4	(Constant)	3.627	.138		26.189	.000
	Ideology0	-.602	.074	-.585	-8.144	.000
	GenderCC	-.151	.114	-.092	-1.319	.189
	RaceCC	.120	.114	.063	1.051	.295
	SES0	-.061	.113	-.030	-.541	.589
	MGRS0	-.013	.212	-.005	-.059	.953
	MRN0	-.219	.162	-.115	-1.354	.177
	MRN0xRace	.123	.139	.065	.885	.377
	MRN0xSES0	.102	.131	.046	.781	.436
	MRN0xGender	-.163	.150	-.077	-1.089	.278
	MRN0xIdeology0	-.017	.067	-.016	-.251	.802
	MRN0xMGRS0	.011	.232	.004	.049	.961
	MGRS0xGender	.244	.182	.091	1.339	.182
	MGRS0xRace	-.097	.187	-.038	-.519	.604
	MGRS0xSES0	.169	.193	.054	.876	.382
	MGRS0xIdeology0	.044	.121	.030	.363	.717

Coefficients[a]

Model		Correlations Zero-order	Partial	Part
1	(Constant)			
	Ideology0	-.638	-.638	-.638
2	(Constant)			
	Ideology0	-.638	-.609	-.582
	GenderCC	-.267	-.131	-.100
	RaceCC	-.111	.104	.079
	SES0	-.094	-.054	-.041
3	(Constant)			
	Ideology0	-.638	-.525	-.462
	GenderCC	-.267	-.107	-.081
	RaceCC	-.111	.081	.061
	SES0	-.094	-.051	-.038
	MGRS0	-.036	-.081	-.061
	MRN0	-.456	-.087	-.066
4	(Constant)			
	Ideology0	-.638	-.516	-.443
	GenderCC	-.267	-.097	-.072
	RaceCC	-.111	.077	.057
	SES0	-.094	-.040	-.029
	MGRS0	-.036	-.004	-.003
	MRN0	-.456	-.100	-.074
	MRN0xRace	-.319	.065	.048
	MRN0xSES0	.107	.058	.042
	MRN0xGender	-.186	-.080	-.059
	MRN0xIdeology0	-.042	-.019	-.014
	MRN0xMGRS0	.108	.004	.003
	MGRS0xGender	.004	.098	.073
	MGRS0xRace	-.094	-.038	-.028
	MGRS0xSES0	.153	.065	.048
	MGRS0xIdeology0	.019	.027	.020

a. Dependent Variable: PelosiX

Excluded Variables[a]

Model		Beta In	t	Sig.	Partial Correlation	Collinearity Statistics Tolerance
1	GenderCC	-.107[b]	-1.892	.060	-.134	.931
	RaceCC	.085[b]	1.482	.140	.105	.913
	SES0	-.044[b]	-.791	.430	-.056	.994
	MGRS0	-.057[b]	-1.038	.301	-.074	.999
	MRN0	-.160[b]	-2.505	.013	-.176	.715
	MRN0xRace	-.039[b]	-.636	.525	-.045	.796
	MRN0xSES0	.054[b]	.975	.331	.069	.993
	MRN0xGender	-.092[b]	-1.662	.098	-.118	.977
	MRN0xIdeology0	.004[b]	.081	.936	.006	.995
	MRN0xMGRS0	.075[b]	1.371	.172	.097	.997
	MGRS0xGender	.060[b]	1.095	.275	.078	.992
	MGRS0xRace	-.045[b]	-.817	.415	-.058	.994
	MGRS0xSES0	.104[b]	1.909	.058	.135	.994
	MGRS0xIdeology0	.076[b]	1.388	.167	.099	.992
2	MGRS0	-.094[c]	-1.639	.103	-.117	.884
	MRN0	-.118[c]	-1.702	.090	-.122	.606
	MRN0xRace	-.015[c]	-.237	.813	-.017	.772
	MRN0xSES0	.059[c]	1.070	.286	.077	.983
	MRN0xGender	-.085[c]	-1.547	.124	-.111	.965
	MRN0xIdeology0	-.001[c]	-.023	.982	-.002	.948
	MRN0xMGRS0	.081[c]	1.479	.141	.106	.974
	MGRS0xGender	.061[c]	1.121	.264	.080	.984
	MGRS0xRace	-.064[c]	-1.159	.248	-.083	.969
	MGRS0xSES0	.109[c]	1.968	.050	.140	.957
	MGRS0xIdeology0	.073[c]	1.324	.187	.095	.979
3	MRN0xRace	.030[d]	.442	.659	.032	.655
	MRN0xSES0	.045[d]	.812	.418	.059	.966
	MRN0xGender	-.068[d]	-1.223	.223	-.088	.936
	MRN0xIdeology0	-.011[d]	-.198	.843	-.014	.928
	MRN0xMGRS0	.079[d]	1.447	.150	.104	.967
	MGRS0xGender	.063[d]	1.130	.260	.082	.954

Excluded Variables[a]

Model		Beta In	t	Sig.	Partial Correlation	Collinearity Statistics Tolerance
	MGRS0xRace	-.028[d]	-.403	.687	-.029	.630
	MGRS0xSES0	.087[d]	1.524	.129	.110	.890
	MGRS0xIdeology0	.059[d]	1.078	.283	.078	.962

a. Dependent Variable: PelosiX

b. Predictors in the Model: (Constant), Ideology0

c. Predictors in the Model: (Constant), Ideology0, SES0, GenderCC, RaceCC

d. Predictors in the Model: (Constant), Ideology0, SES0, GenderCC, RaceCC, MGRS0, MRN0

```
 REGRESSION
  /MISSING LISTWISE
  /STATISTICS COEFF OUTS R ANOVA CHANGE ZPP
  /CRITERIA=PIN(.05) POUT(.10)
  /NOORIGIN
  /DEPENDENT McConnellX
  /METHOD=ENTER Ideology0
  /METHOD=ENTER GenderCC RaceCC SES0
  /METHOD=ENTER MGRS0 MRN0
  /METHOD=ENTER MRN0xRace MRN0xSES0 MRN0xGender MRN0xIdeology0 MRN0xMGRS0 MGRS0xGender MG
RS0xRace MGRS0xSES0 MGRS0xIdeology0.
```

Regression

Notes

Output Created		15-DEC-2021 13:07:59
Comments		
Input	Data	C: \Users\njs5478\Dropbox\HM and COVID\0. Revise and Resubmit\2. R and R Data\Study 1b\Study1b_Data.sav
	Active Dataset	DataSet1
	Filter	<none>
	Weight	<none>
	Split File	<none>
	N of Rows in Working Data File	241
Missing Value Handling	Definition of Missing	User-defined missing values are treated as missing.
	Cases Used	Statistics are based on cases with no missing values for any variable used.
Syntax		REGRESSION /MISSING LISTWISE /STATISTICS COEFF OUTS R ANOVA CHANGE ZPP /CRITERIA=PIN(.05) POUT(.10) /NOORIGIN /DEPENDENT McConnellX /METHOD=ENTER Ideology0 /METHOD=ENTER GenderCC RaceCC SES0 /METHOD=ENTER MGRS0 MRN0 /METHOD=ENTER MRN0xRace MRN0xSES0 MRN0xGender MRN0xIdeology0 MRN0xMGRS0 MGRS0xGender MGRS0xRace MGRS0xSES0 MGRS0xIdeology0.

Notes

Resources	Processor Time	00:00:00.02
	Elapsed Time	00:00:00.02
	Memory Required	52240 bytes
	Additional Memory Required for Residual Plots	0 bytes

Variables Entered/Removed[a]

Model	Variables Entered	Variables Removed	Method
1	Ideology0[b]	.	Enter
2	SES0, GenderCC, RaceCC[b]	.	Enter
3	MGRS0, MRN0[b]	.	Enter
4	MRN0xMGRS0, MRN0xSES0, MRN0xIdeology0, MGRS0xGender, MGRS0xSES0, MGRS0xRace, MRN0xGender, MRN0xRace, MGRS0xIdeology0[b]	.	Enter

a. Dependent Variable: McConnellX

b. All requested variables entered.

Model Summary

Model	R	R Square	Adjusted R Square	Std. Error of the Estimate	Change Statistics		
					R Square Change	F Change	df1
1	.535[a]	.286	.283	1.30321	.286	76.622	1
2	.560[b]	.314	.299	1.28787	.028	2.525	3
3	.595[c]	.354	.333	1.25682	.040	5.703	2
4	.634[d]	.402	.352	1.23894	.049	1.601	9

Model Summary

Model	Change Statistics	
	df2	Sig. F Change
1	191	.000
2	188	.059
3	186	.004
4	177	.118

a. Predictors: (Constant), Ideology0

b. Predictors: (Constant), Ideology0, SES0, GenderCC, RaceCC

c. Predictors: (Constant), Ideology0, SES0, GenderCC, RaceCC, MGRS0, MRN0

d. Predictors: (Constant), Ideology0, SES0, GenderCC, RaceCC, MGRS0, MRN0, MRN0xMGRS0, MRN0xSES0, MRN0xIdeology0, MGRS0xGender, MGRS0xSES0, MGRS0xRace, MRN0xGender, MRN0xRace, MGRS0xIdeology0

ANOVA[a]

Model		Sum of Squares	df	Mean Square	F	Sig.
1	Regression	130.131	1	130.131	76.622	.000[b]
	Residual	324.387	191	1.698		
	Total	454.518	192			
2	Regression	142.697	4	35.674	21.508	.000[c]
	Residual	311.821	188	1.659		
	Total	454.518	192			
3	Regression	160.713	6	26.786	16.957	.000[d]
	Residual	293.805	186	1.580		
	Total	454.518	192			
4	Regression	182.829	15	12.189	7.941	.000[e]
	Residual	271.689	177	1.535		
	Total	454.518	192			

a. Dependent Variable: McConnellX

b. Predictors: (Constant), Ideology0

c. Predictors: (Constant), Ideology0, SES0, GenderCC, RaceCC

d. Predictors: (Constant), Ideology0, SES0, GenderCC, RaceCC, MGRS0, MRN0

e. Predictors: (Constant), Ideology0, SES0, GenderCC, RaceCC, MGRS0, MRN0, MRN0xMGRS0, MRN0xSES0, MRN0xIdeology0, MGRS0xGender, MGRS0xSES0, MGRS0xRace, MRN0xGender, MRN0xRace, MGRS0xIdeology0

Coefficients[a]

Model		Unstandardized Coefficients B	Unstandardized Coefficients Std. Error	Standardized Coefficients Beta	t	Sig.
1	(Constant)	3.216	.094		34.271	.000
	Ideology0	.524	.060	.535	8.753	.000
2	(Constant)	3.212	.110		29.240	.000
	Ideology0	.566	.064	.578	8.800	.000
	GenderCC	-.255	.096	-.166	-2.648	.009
	RaceCC	.031	.113	.017	.271	.787
	SES0	-.073	.117	-.038	-.621	.535
3	(Constant)	3.191	.107		29.711	.000
	Ideology0	.477	.073	.487	6.572	.000
	GenderCC	-.258	.112	-.168	-2.301	.023
	RaceCC	.091	.112	.051	.810	.419
	SES0	-.087	.114	-.045	-.760	.448
	MGRS0	.296	.159	.126	1.858	.065
	MRN0	.282	.140	.161	2.009	.046
4	(Constant)	3.187	.141		22.531	.000
	Ideology0	.419	.076	.428	5.536	.000
	GenderCC	-.218	.115	-.142	-1.890	.060
	RaceCC	.118	.117	.066	1.010	.314
	SES0	-.078	.116	-.040	-.669	.504
	MGRS0	.462	.204	.196	2.266	.025
	MRN0	.156	.157	.089	.992	.323
	MRN0xRace	.261	.137	.149	1.900	.059
	MRN0xSES0	-.126	.124	-.063	-1.017	.310
	MRN0xGender	.045	.145	.023	.310	.757
	MRN0xIdeology0	.036	.068	.038	.536	.593
	MRN0xMGRS0	-.098	.227	-.042	-.434	.665
	MGRS0xGender	.229	.183	.091	1.249	.213
	MGRS0xRace	-.204	.179	-.086	-1.138	.256
	MGRS0xSES0	.014	.190	.005	.072	.943
	MGRS0xIdeology0	.197	.120	.151	1.647	.101

Coefficients[a]

Model		Correlations Zero-order	Partial	Part
1	(Constant)			
	Ideology0	.535	.535	.535
2	(Constant)			
	Ideology0	.535	.540	.532
	GenderCC	-.011	-.190	-.160
	RaceCC	.175	.020	.016
	SES0	.009	-.045	-.038
3	(Constant)			
	Ideology0	.535	.434	.387
	GenderCC	-.011	-.166	-.136
	RaceCC	.175	.059	.048
	SES0	.009	-.056	-.045
	MGRS0	.186	.135	.110
	MRN0	.366	.146	.118
4	(Constant)			
	Ideology0	.535	.384	.322
	GenderCC	-.011	-.141	-.110
	RaceCC	.175	.076	.059
	SES0	.009	-.050	-.039
	MGRS0	.186	.168	.132
	MRN0	.366	.074	.058
	MRN0xRace	.369	.141	.110
	MRN0xSES0	-.163	-.076	-.059
	MRN0xGender	.158	.023	.018
	MRN0xIdeology0	.053	.040	.031
	MRN0xMGRS0	.030	-.033	-.025
	MGRS0xGender	.146	.093	.073
	MGRS0xRace	.117	-.085	-.066
	MGRS0xSES0	-.109	.005	.004
	MGRS0xIdeology0	.123	.123	.096

a. Dependent Variable: McConnellX

Excluded Variables[a]

Model		Beta In	t	Sig.	Partial Correlation	Collinearity Statistics Tolerance
1	GenderCC	-.167[b]	-2.677	.008	-.191	.928
	RaceCC	.018[b]	.275	.784	.020	.912
	SES0	-.043[b]	-.692	.490	-.050	.991
	MGRS0	.205[b]	3.441	.001	.242	.999
	MRN0	.112[b]	1.551	.123	.112	.713
	MRN0xRace	.170[b]	2.553	.011	.182	.815
	MRN0xSES0	-.113[b]	-1.852	.066	-.133	.991
	MRN0xGender	.099[b]	1.611	.109	.116	.987
	MRN0xIdeology0	.063[b]	1.030	.304	.075	1.000
	MRN0xMGRS0	.051[b]	.836	.404	.061	.998
	MGRS0xGender	.099[b]	1.621	.107	.117	.992
	MGRS0xRace	.070[b]	1.138	.257	.082	.992
	MGRS0xSES0	-.049[b]	-.804	.423	-.058	.987
	MGRS0xIdeology0	.072[b]	1.176	.241	.085	.991
2	MGRS0	.172[c]	2.693	.008	.193	.863
	MRN0	.212[c]	2.802	.006	.201	.613
	MRN0xRace	.197[c]	2.926	.004	.209	.771
	MRN0xSES0	-.108[c]	-1.774	.078	-.129	.982
	MRN0xGender	.086[c]	1.411	.160	.103	.966
	MRN0xIdeology0	.071[c]	1.154	.250	.084	.956
	MRN0xMGRS0	.063[c]	1.032	.303	.075	.967
	MGRS0xGender	.098[c]	1.618	.107	.118	.986
	MGRS0xRace	.046[c]	.744	.458	.054	.968
	MGRS0xSES0	-.042[c]	-.674	.501	-.049	.950
	MGRS0xIdeology0	.078[c]	1.266	.207	.092	.969
3	MRN0xRace	.147[d]	2.056	.041	.149	.667
	MRN0xSES0	-.077[d]	-1.286	.200	-.094	.956
	MRN0xGender	.059[d]	.969	.334	.071	.943
	MRN0xIdeology0	.096[d]	1.568	.119	.114	.925
	MRN0xMGRS0	.073[d]	1.216	.225	.089	.961
	MGRS0xGender	.106[d]	1.765	.079	.129	.960

Excluded Variables[a]

Model		Beta In	t	Sig.	Partial Correlation	Collinearity Statistics Tolerance
	MGRS0xRace	-.048[d]	-.674	.501	-.049	.674
	MGRS0xSES0	.009[d]	.144	.885	.011	.883
	MGRS0xIdeology0	.109[d]	1.807	.072	.132	.949

a. Dependent Variable: McConnellX

b. Predictors in the Model: (Constant), Ideology0

c. Predictors in the Model: (Constant), Ideology0, SES0, GenderCC, RaceCC

d. Predictors in the Model: (Constant), Ideology0, SES0, GenderCC, RaceCC, MGRS0, MRN0

```
REGRESSION
  /MISSING LISTWISE
  /STATISTICS COEFF OUTS R ANOVA CHANGE ZPP
  /CRITERIA=PIN(.05) POUT(.10)
  /NOORIGIN
  /DEPENDENT FauciX
  /METHOD=ENTER Ideology0
  /METHOD=ENTER GenderCC RaceCC SES0
  /METHOD=ENTER MGRS0 MRN0
  /METHOD=ENTER MRN0xRace MRN0xSES0 MRN0xGender MRN0xIdeology0 MRN0xMGRS0 MGRS0xGender MG
RS0xRace MGRS0xSES0 MGRS0xIdeology0.
```

Regression

Notes

Output Created		15-DEC-2021 13:07:59
Comments		
Input	Data	C: \Users\njs5478\Dropbox\HM and COVID\0. Revise and Resubmit\2. R and R Data\Study 1b\Study1b_Data.sav
	Active Dataset	DataSet1
	Filter	<none>
	Weight	<none>
	Split File	<none>
	N of Rows in Working Data File	241
Missing Value Handling	Definition of Missing	User-defined missing values are treated as missing.
	Cases Used	Statistics are based on cases with no missing values for any variable used.
Syntax		REGRESSION /MISSING LISTWISE /STATISTICS COEFF OUTS R ANOVA CHANGE ZPP /CRITERIA=PIN(.05) POUT(.10) /NOORIGIN /DEPENDENT FauciX /METHOD=ENTER Ideology0 /METHOD=ENTER GenderCC RaceCC SES0 /METHOD=ENTER MGRS0 MRN0 /METHOD=ENTER MRN0xRace MRN0xSES0 MRN0xGender MRN0xIdeology0 MRN0xMGRS0 MGRS0xGender MGRS0xRace MGRS0xSES0 MGRS0xIdeology0.
Resources	Processor Time	00:00:00.02
	Elapsed Time	00:00:00.03

Notes

	Memory Required	52240 bytes
	Additional Memory Required for Residual Plots	0 bytes

Variables Entered/Removed[a]

Model	Variables Entered	Variables Removed	Method
1	Ideology0[b]	.	Enter
2	SES0, GenderCC, RaceCC[b]	.	Enter
3	MGRS0, MRN0[b]	.	Enter
4	MRN0xSES0, MRN0xGender, MGRS0xIdeology0, MGRS0xSES0, MRN0xIdeology0, MGRS0xGender, MGRS0xRace, MRN0xRace, MRN0xMGRS...[b]	.	Enter

a. Dependent Variable: FauciX

b. All requested variables entered.

Model Summary

Model	R	R Square	Adjusted R Square	Std. Error of the Estimate	Change Statistics		
					R Square Change	F Change	df1
1	.310[a]	.096	.092	1.57019	.096	22.205	1
2	.340[b]	.116	.099	1.56426	.020	1.529	3
3	.376[c]	.142	.116	1.54869	.026	3.082	2
4	.445[d]	.198	.137	1.53075	.057	1.534	9

Model Summary

Model	Change Statistics	
	df2	Sig. F Change
1	209	.000
2	206	.208
3	204	.048
4	195	.138

a. Predictors: (Constant), Ideology0

b. Predictors: (Constant), Ideology0, SES0, GenderCC, RaceCC

c. Predictors: (Constant), Ideology0, SES0, GenderCC, RaceCC, MGRS0, MRN0

d. Predictors: (Constant), Ideology0, SES0, GenderCC, RaceCC, MGRS0, MRN0, MRN0xSES0, MRN0xGender, MGRS0xIdeology0, MGRS0xSES0, MRN0xIdeology0, MGRS0xGender, MGRS0xRace, MRN0xRace, MRN0xMGRS0

ANOVA[a]

Model		Sum of Squares	df	Mean Square	F	Sig.
1	Regression	54.746	1	54.746	22.205	.000[b]
	Residual	515.292	209	2.466		
	Total	570.038	210			
2	Regression	65.974	4	16.493	6.740	.000[c]
	Residual	504.064	206	2.447		
	Total	570.038	210			
3	Regression	80.758	6	13.460	5.612	.000[d]
	Residual	489.280	204	2.398		
	Total	570.038	210			
4	Regression	113.115	15	7.541	3.218	.000[e]
	Residual	456.923	195	2.343		
	Total	570.038	210			

a. Dependent Variable: FauciX

b. Predictors: (Constant), Ideology0

c. Predictors: (Constant), Ideology0, SES0, GenderCC, RaceCC

d. Predictors: (Constant), Ideology0, SES0, GenderCC, RaceCC, MGRS0, MRN0

e. Predictors: (Constant), Ideology0, SES0, GenderCC, RaceCC, MGRS0, MRN0, MRN0xSES0, MRN0xGender, MGRS0xIdeology0, MGRS0xSES0, MRN0xIdeology0, MGRS0xGender, MGRS0xRace, MRN0xRace, MRN0xMGRS0

Coefficients[a]

Model		Unstandardized Coefficients B	Std. Error	Standardized Coefficients Beta	t	Sig.
1	(Constant)	5.116	.108		47.331	.000
	Ideology0	-.325	.069	-.310	-4.712	.000
2	(Constant)	5.025	.123		40.881	.000
	Ideology0	-.377	.077	-.359	-4.925	.000
	GenderCC	.041	.114	.025	.363	.717
	RaceCC	.187	.128	.101	1.459	.146
	SES0	.198	.135	.097	1.468	.144
3	(Constant)	5.043	.122		41.185	.000
	Ideology0	-.279	.086	-.266	-3.249	.001
	GenderCC	.201	.134	.122	1.495	.136
	RaceCC	.157	.129	.085	1.219	.224
	SES0	.236	.135	.115	1.751	.081
	MGRS0	.182	.189	.071	.962	.337
	MRN0	-.418	.169	-.220	-2.480	.014
4	(Constant)	4.937	.164		30.050	.000
	Ideology0	-.275	.089	-.262	-3.078	.002
	GenderCC	.154	.137	.094	1.121	.264
	RaceCC	.167	.133	.090	1.258	.210
	SES0	.271	.136	.132	1.989	.048
	MGRS0	.128	.238	.050	.539	.591
	MRN0	-.324	.183	-.170	-1.775	.078
	MRN0xRace	-.038	.157	-.020	-.245	.807
	MRN0xSES0	.016	.149	.007	.107	.915
	MRN0xGender	-.137	.176	-.063	-.776	.439
	MRN0xIdeology0	.012	.083	.011	.144	.885
	MRN0xMGRS0	.218	.273	.082	.799	.425
	MGRS0xGender	-.574	.219	-.212	-2.624	.009
	MGRS0xRace	-.148	.209	-.058	-.708	.480
	MGRS0xSES0	-.147	.222	-.047	-.663	.508
	MGRS0xIdeology0	-.004	.144	-.003	-.030	.976

Coefficients[a]

Model		Correlations Zero-order	Partial	Part
1	(Constant)			
	Ideology0	-.310	-.310	-.310
2	(Constant)			
	Ideology0	-.310	-.325	-.323
	GenderCC	-.073	.025	.024
	RaceCC	-.008	.101	.096
	SES0	.073	.102	.096
3	(Constant)			
	Ideology0	-.310	-.222	-.211
	GenderCC	-.073	.104	.097
	RaceCC	-.008	.085	.079
	SES0	.073	.122	.114
	MGRS0	.009	.067	.062
	MRN0	-.273	-.171	-.161
4	(Constant)			
	Ideology0	-.310	-.215	-.197
	GenderCC	-.073	.080	.072
	RaceCC	-.008	.090	.081
	SES0	.073	.141	.128
	MGRS0	.009	.039	.035
	MRN0	-.273	-.126	-.114
	MRN0xRace	-.196	-.018	-.016
	MRN0xSES0	.058	.008	.007
	MRN0xGender	-.189	-.055	-.050
	MRN0xIdeology0	.026	.010	.009
	MRN0xMGRS0	.054	.057	.051
	MGRS0xGender	-.215	-.185	-.168
	MGRS0xRace	-.034	-.051	-.045
	MGRS0xSES0	.005	-.047	-.043
	MGRS0xIdeology0	.013	-.002	-.002

a. Dependent Variable: FauciX

Excluded Variables[a]

Model		Beta In	t	Sig.	Partial Correlation	Collinearity Statistics Tolerance
1	GenderCC	.026[b]	.379	.705	.026	.902
	RaceCC	.104[b]	1.493	.137	.103	.894
	SES0	.101[b]	1.540	.125	.106	.992
	MGRS0	-.017[b]	-.252	.801	-.017	.993
	MRN0	-.153[b]	-2.000	.047	-.137	.729
	MRN0xRace	-.089[b]	-1.252	.212	-.086	.851
	MRN0xSES0	.042[b]	.632	.528	.044	.997
	MRN0xGender	-.167[b]	-2.563	.011	-.175	.995
	MRN0xIdeology0	.051[b]	.771	.442	.053	.994
	MRN0xMGRS0	.033[b]	.502	.616	.035	.995
	MGRS0xGender	-.206[b]	-3.205	.002	-.217	.999
	MGRS0xRace	-.007[b]	-.109	.913	-.008	.992
	MGRS0xSES0	-.017[b]	-.259	.796	-.018	.995
	MGRS0xIdeology0	.049[b]	.739	.461	.051	.987
2	MGRS0	.008[c]	.117	.907	.008	.879
	MRN0	-.190[c]	-2.289	.023	-.158	.608
	MRN0xRace	-.075[c]	-1.051	.295	-.073	.832
	MRN0xSES0	.045[c]	.679	.498	.047	.994
	MRN0xGender	-.159[c]	-2.436	.016	-.168	.981
	MRN0xIdeology0	.046[c]	.695	.488	.048	.972
	MRN0xMGRS0	.041[c]	.610	.543	.043	.970
	MGRS0xGender	-.219[c]	-3.417	.001	-.232	.992
	MGRS0xRace	-.011[c]	-.170	.865	-.012	.970
	MGRS0xSES0	-.018[c]	-.273	.785	-.019	.965
	MGRS0xIdeology0	.048[c]	.715	.475	.050	.971
3	MRN0xRace	-.032[d]	-.428	.669	-.030	.764
	MRN0xSES0	.036[d]	.546	.586	.038	.971
	MRN0xGender	-.143[d]	-2.178	.031	-.151	.963
	MRN0xIdeology0	.021[d]	.316	.752	.022	.943
	MRN0xMGRS0	.027[d]	.407	.684	.029	.951
	MGRS0xGender	-.205[d]	-3.186	.002	-.218	.970

Excluded Variables[a]

Model		Beta In	t	Sig.	Partial Correlation	Collinearity Statistics Tolerance
	MGRS0xRace	-.038[d]	-.502	.616	-.035	.726
	MGRS0xSES0	-.029[d]	-.426	.671	-.030	.910
	MGRS0xIdeology0	.029[d]	.432	.666	.030	.950

a. Dependent Variable: FauciX

b. Predictors in the Model: (Constant), Ideology0

c. Predictors in the Model: (Constant), Ideology0, SES0, GenderCC, RaceCC

d. Predictors in the Model: (Constant), Ideology0, SES0, GenderCC, RaceCC, MGRS0, MRN0

```
REGRESSION
  /MISSING LISTWISE
  /STATISTICS COEFF OUTS R ANOVA CHANGE ZPP
  /CRITERIA=PIN(.05) POUT(.10)
  /NOORIGIN
  /DEPENDENT RepCongressX
  /METHOD=ENTER Ideology0
  /METHOD=ENTER GenderCC RaceCC SES0
  /METHOD=ENTER MGRS0 MRN0
  /METHOD=ENTER MRN0xRace MRN0xSES0 MRN0xGender MRN0xIdeology0 MRN0xMGRS0 MGRS0xGender MG
RS0xRace MGRS0xSES0 MGRS0xIdeology0.
```

Regression

Notes

Output Created		15-DEC-2021 13:07:59
Comments		
Input	Data	C: \Users\njs5478\Dropbox\HM and COVID\0. Revise and Resubmit\2. R and R Data\Study 1b\Study1b_Data.sav
	Active Dataset	DataSet1
	Filter	<none>
	Weight	<none>
	Split File	<none>
	N of Rows in Working Data File	241
Missing Value Handling	Definition of Missing	User-defined missing values are treated as missing.
	Cases Used	Statistics are based on cases with no missing values for any variable used.
Syntax		REGRESSION /MISSING LISTWISE /STATISTICS COEFF OUTS R ANOVA CHANGE ZPP /CRITERIA=PIN(.05) POUT(.10) /NOORIGIN /DEPENDENT RepCongressX /METHOD=ENTER Ideology0 /METHOD=ENTER GenderCC RaceCC SES0 /METHOD=ENTER MGRS0 MRN0 /METHOD=ENTER MRN0xRace MRN0xSES0 MRN0xGender MRN0xIdeology0 MRN0xMGRS0 MGRS0xGender MGRS0xRace MGRS0xSES0 MGRS0xIdeology0.

Notes

Resources	Processor Time	00:00:00.06
	Elapsed Time	00:00:00.03
	Memory Required	52240 bytes
	Additional Memory Required for Residual Plots	0 bytes

Variables Entered/Removed[a]

Model	Variables Entered	Variables Removed	Method
1	Ideology0[b]	.	Enter
2	SES0, GenderCC, RaceCC[b]	.	Enter
3	MGRS0, MRN0[b]	.	Enter
4	MRN0xMGRS0, MRN0xSES0, MRN0xIdeology0, MGRS0xSES0, MGRS0xGender, MGRS0xRace, MRN0xRace, MRN0xGender, MGRS0xIdeol...[b]	.	Enter

a. Dependent Variable: RepCongressX

b. All requested variables entered.

Model Summary

Model	R	R Square	Adjusted R Square	Std. Error of the Estimate	Change Statistics		
					R Square Change	F Change	df1
1	.725[a]	.526	.523	1.20275	.526	234.801	1
2	.733[b]	.538	.529	1.19540	.012	1.871	3
3	.735[c]	.541	.528	1.19726	.003	.675	2
4	.757[d]	.573	.541	1.18052	.032	1.657	9

Model Summary

Model	Change Statistics	
	df2	Sig. F Change
1	212	.000
2	209	.136
3	207	.510
4	198	.102

a. Predictors: (Constant), Ideology0

b. Predictors: (Constant), Ideology0, SES0, GenderCC, RaceCC

c. Predictors: (Constant), Ideology0, SES0, GenderCC, RaceCC, MGRS0, MRN0

d. Predictors: (Constant), Ideology0, SES0, GenderCC, RaceCC, MGRS0, MRN0, MRN0xMGRS0, MRN0xSES0, MRN0xIdeology0, MGRS0xSES0, MGRS0xGender, MGRS0xRace, MRN0xRace, MRN0xGender, MGRS0xIdeology0

ANOVA[a]

Model		Sum of Squares	df	Mean Square	F	Sig.
1	Regression	339.662	1	339.662	234.801	.000[b]
	Residual	306.679	212	1.447		
	Total	646.341	213			
2	Regression	347.683	4	86.921	60.827	.000[c]
	Residual	298.658	209	1.429		
	Total	646.341	213			
3	Regression	349.619	6	58.270	40.650	.000[d]
	Residual	296.722	207	1.433		
	Total	646.341	213			
4	Regression	370.401	15	24.693	17.719	.000[e]
	Residual	275.940	198	1.394		
	Total	646.341	213			

a. Dependent Variable: RepCongressX

b. Predictors: (Constant), Ideology0

c. Predictors: (Constant), Ideology0, SES0, GenderCC, RaceCC

d. Predictors: (Constant), Ideology0, SES0, GenderCC, RaceCC, MGRS0, MRN0

e. Predictors: (Constant), Ideology0, SES0, GenderCC, RaceCC, MGRS0, MRN0, MRN0xMGRS0, MRN0xSES0, MRN0xIdeology0, MGRS0xSES0, MGRS0xGender, MGRS0xRace, MRN0xRace, MRN0xGender, MGRS0xIdeology0

Coefficients[a]

Model		Unstandardized Coefficients B	Std. Error	Standardized Coefficients Beta	t	Sig.
1	(Constant)	3.386	.082		41.186	.000
	Ideology0	.788	.051	.725	15.323	.000
2	(Constant)	3.274	.095		34.459	.000
	Ideology0	.750	.056	.690	13.321	.000
	GenderCC	-.002	.085	-.001	-.019	.985
	RaceCC	.229	.099	.115	2.316	.022
	SES0	-.052	.098	-.025	-.531	.596
3	(Constant)	3.264	.096		34.168	.000
	Ideology0	.722	.065	.664	11.042	.000
	GenderCC	-.007	.102	-.004	-.070	.945
	RaceCC	.245	.100	.123	2.447	.015
	SES0	-.055	.099	-.026	-.556	.579
	MGRS0	.082	.143	.031	.578	.564
	MRN0	.095	.129	.048	.738	.461
4	(Constant)	3.210	.129		24.835	.000
	Ideology0	.677	.068	.622	9.920	.000
	GenderCC	.025	.106	.015	.239	.811
	RaceCC	.261	.103	.131	2.524	.012
	SES0	-.017	.100	-.008	-.168	.867
	MGRS0	.304	.182	.116	1.670	.096
	MRN0	.033	.141	.017	.234	.816
	MRN0xRace	.120	.117	.060	1.024	.307
	MRN0xSES0	-.109	.107	-.051	-1.019	.310
	MRN0xGender	.131	.137	.059	.951	.343
	MRN0xIdeology0	.027	.062	.025	.437	.662
	MRN0xMGRS0	-.118	.205	-.044	-.576	.566
	MGRS0xGender	.063	.168	.023	.377	.707
	MGRS0xRace	-.303	.158	-.115	-1.916	.057
	MGRS0xSES0	.018	.165	.006	.109	.914
	MGRS0xIdeology0	.258	.109	.171	2.361	.019

Coefficients[a]

Model		Correlations Zero-order	Partial	Part
1	(Constant)			
	Ideology0	.725	.725	.725
2	(Constant)			
	Ideology0	.725	.678	.626
	GenderCC	.192	-.001	-.001
	RaceCC	.337	.158	.109
	SES0	.036	-.037	-.025
3	(Constant)			
	Ideology0	.725	.609	.520
	GenderCC	.192	-.005	-.003
	RaceCC	.337	.168	.115
	SES0	.036	-.039	-.026
	MGRS0	-.002	.040	.027
	MRN0	.416	.051	.035
4	(Constant)			
	Ideology0	.725	.576	.461
	GenderCC	.192	.017	.011
	RaceCC	.337	.177	.117
	SES0	.036	-.012	-.008
	MGRS0	-.002	.118	.078
	MRN0	.416	.017	.011
	MRN0xRace	.319	.073	.048
	MRN0xSES0	-.171	-.072	-.047
	MRN0xGender	.140	.067	.044
	MRN0xIdeology0	.111	.031	.020
	MRN0xMGRS0	.022	-.041	-.027
	MGRS0xGender	.113	.027	.017
	MGRS0xRace	.029	-.135	-.089
	MGRS0xSES0	-.054	.008	.005
	MGRS0xIdeology0	.185	.165	.110

a. Dependent Variable: RepCongressX

Excluded Variables[a]

Model		Beta In	t	Sig.	Partial Correlation	Collinearity Statistics Tolerance
1	GenderCC	-.007[b]	-.142	.887	-.010	.925
	RaceCC	.115[b]	2.318	.021	.158	.895
	SES0	-.023[b]	-.479	.633	-.033	.993
	MGRS0	.034[b]	.709	.479	.049	.998
	MRN0	.032[b]	.568	.571	.039	.705
	MRN0xRace	.048[b]	.930	.354	.064	.852
	MRN0xSES0	-.071[b]	-1.498	.136	-.103	.981
	MRN0xGender	.045[b]	.952	.342	.065	.983
	MRN0xIdeology0	.077[b]	1.622	.106	.111	.998
	MRN0xMGRS0	.061[b]	1.288	.199	.088	.997
	MGRS0xGender	.054[b]	1.135	.258	.078	.993
	MGRS0xRace	-.034[b]	-.713	.476	-.049	.993
	MGRS0xSES0	.005[b]	.103	.918	.007	.993
	MGRS0xIdeology0	.100[b]	2.116	.036	.144	.986
2	MGRS0	.045[c]	.899	.370	.062	.865
	MRN0	.061[c]	1.010	.314	.070	.601
	MRN0xRace	.070[c]	1.357	.176	.094	.822
	MRN0xSES0	-.069[c]	-1.464	.145	-.101	.977
	MRN0xGender	.062[c]	1.294	.197	.089	.962
	MRN0xIdeology0	.064[c]	1.329	.185	.092	.960
	MRN0xMGRS0	.058[c]	1.219	.224	.084	.966
	MGRS0xGender	.056[c]	1.174	.242	.081	.984
	MGRS0xRace	-.040[c]	-.829	.408	-.057	.963
	MGRS0xSES0	-.006[c]	-.123	.902	-.009	.957
	MGRS0xIdeology0	.090[c]	1.904	.058	.131	.969
3	MRN0xRace	.061[d]	1.126	.261	.078	.764
	MRN0xSES0	-.061[d]	-1.263	.208	-.088	.940
	MRN0xGender	.054[d]	1.095	.275	.076	.927
	MRN0xIdeology0	.075[d]	1.534	.127	.106	.923
	MRN0xMGRS0	.060[d]	1.243	.215	.086	.963
	MGRS0xGender	.058[d]	1.188	.236	.083	.944

Excluded Variables[a]

Model		Beta In	t	Sig.	Partial Correlation	Collinearity Statistics Tolerance
	MGRS0xRace	-.079[d]	-1.420	.157	-.098	.705
	MGRS0xSES0	.009[d]	.172	.864	.012	.884
	MGRS0xIdeology0	.104[d]	2.161	.032	.149	.936

a. Dependent Variable: RepCongressX

b. Predictors in the Model: (Constant), Ideology0

c. Predictors in the Model: (Constant), Ideology0, SES0, GenderCC, RaceCC

d. Predictors in the Model: (Constant), Ideology0, SES0, GenderCC, RaceCC, MGRS0, MRN0

```
 REGRESSION
  /MISSING LISTWISE
  /STATISTICS COEFF OUTS R ANOVA CHANGE ZPP
  /CRITERIA=PIN(.05) POUT(.10)
  /NOORIGIN
  /DEPENDENT DemCongressX
  /METHOD=ENTER Ideology0
  /METHOD=ENTER GenderCC RaceCC SES0
  /METHOD=ENTER MGRS0 MRN0
  /METHOD=ENTER MRN0xRace MRN0xSES0 MRN0xGender MRN0xIdeology0 MRN0xMGRS0 MGRS0xGender MG
RS0xRace MGRS0xSES0 MGRS0xIdeology0.
```

Regression

Notes

Output Created		15-DEC-2021 13:07:59
Comments		
Input	Data	C:\Users\njs5478\Dropbox\HM and COVID\0. Revise and Resubmit\2. R and R Data\Study 1b\Study1b_Data.sav
	Active Dataset	DataSet1
	Filter	<none>
	Weight	<none>
	Split File	<none>
	N of Rows in Working Data File	241
Missing Value Handling	Definition of Missing	User-defined missing values are treated as missing.
	Cases Used	Statistics are based on cases with no missing values for any variable used.
Syntax		REGRESSION<br>/MISSING LISTWISE<br>/STATISTICS COEFF OUTS R ANOVA CHANGE ZPP<br>/CRITERIA=PIN(.05) POUT(.10)<br>/NOORIGIN<br>/DEPENDENT DemCongressX<br>/METHOD=ENTER Ideology0<br>/METHOD=ENTER GenderCC RaceCC SES0<br>/METHOD=ENTER MGRS0 MRN0<br>/METHOD=ENTER MRN0xRace MRN0xSES0 MRN0xGender MRN0xIdeology0 MRN0xMGRS0 MGRS0xGender MGRS0xRace MGRS0xSES0 MGRS0xIdeology0.

Notes

Resources	Processor Time	00:00:00.03
	Elapsed Time	00:00:00.03
	Memory Required	52240 bytes
	Additional Memory Required for Residual Plots	0 bytes

Variables Entered/Removed[a]

Model	Variables Entered	Variables Removed	Method
1	Ideology0[b]	.	Enter
2	SES0, GenderCC, RaceCC[b]	.	Enter
3	MGRS0, MRN0[b]	.	Enter
4	MRN0xMGRS0, MRN0xSES0, MRN0xIdeology0, MGRS0xSES0, MGRS0xGender, MGRS0xRace, MRN0xRace, MRN0xGender, MGRS0xIdeol...[b]	.	Enter

a. Dependent Variable: DemCongressX

b. All requested variables entered.

Model Summary

Model	R	R Square	Adjusted R Square	Std. Error of the Estimate	Change Statistics		
					R Square Change	F Change	df1
1	.606[a]	.367	.364	1.27042	.367	121.635	1
2	.611[b]	.373	.361	1.27280	.007	.738	3
3	.612[c]	.374	.356	1.27801	.001	.157	2
4	.645[d]	.415	.371	1.26344	.041	1.529	9

Model Summary

Model	Change Statistics	
	df2	Sig. F Change
1	210	.000
2	207	.530
3	205	.855
4	196	.140

a. Predictors: (Constant), Ideology0

b. Predictors: (Constant), Ideology0, SES0, GenderCC, RaceCC

c. Predictors: (Constant), Ideology0, SES0, GenderCC, RaceCC, MGRS0, MRN0

d. Predictors: (Constant), Ideology0, SES0, GenderCC, RaceCC, MGRS0, MRN0, MRN0xMGRS0, MRN0xSES0, MRN0xIdeology0, MGRS0xSES0, MGRS0xGender, MGRS0xRace, MRN0xRace, MRN0xGender, MGRS0xIdeology0

ANOVA[a]

Model		Sum of Squares	df	Mean Square	F	Sig.
1	Regression	196.314	1	196.314	121.635	.000[b]
	Residual	338.932	210	1.614		
	Total	535.245	211			
2	Regression	199.903	4	49.976	30.849	.000[c]
	Residual	335.343	207	1.620		
	Total	535.245	211			
3	Regression	200.415	6	33.402	20.451	.000[d]
	Residual	334.831	205	1.633		
	Total	535.245	211			
4	Regression	222.376	15	14.825	9.287	.000[e]
	Residual	312.869	196	1.596		
	Total	535.245	211			

a. Dependent Variable: DemCongressX

b. Predictors: (Constant), Ideology0

c. Predictors: (Constant), Ideology0, SES0, GenderCC, RaceCC

d. Predictors: (Constant), Ideology0, SES0, GenderCC, RaceCC, MGRS0, MRN0

e. Predictors: (Constant), Ideology0, SES0, GenderCC, RaceCC, MGRS0, MRN0, MRN0xMGRS0, MRN0xSES0, MRN0xIdeology0, MGRS0xSES0, MGRS0xGender, MGRS0xRace, MRN0xRace, MRN0xGender, MGRS0xIdeology0

Coefficients[a]

Model		Unstandardized Coefficients		Standardized Coefficients	t	Sig.
		B	Std. Error	Beta		
1	(Constant)	4.243	.087		48.624	.000
	Ideology0	-.604	.055	-.606	-11.029	.000
2	(Constant)	4.181	.101		41.280	.000
	Ideology0	-.618	.061	-.620	-10.186	.000
	GenderCC	-.061	.091	-.039	-.670	.503
	RaceCC	.131	.106	.072	1.238	.217
	SES0	.035	.105	.019	.336	.737
3	(Constant)	4.184	.102		40.979	.000
	Ideology0	-.599	.070	-.601	-8.563	.000
	GenderCC	-.036	.111	-.023	-.326	.745
	RaceCC	.125	.107	.069	1.164	.246
	SES0	.041	.106	.022	.386	.700
	MGRS0	.016	.152	.007	.108	.914
	MRN0	-.077	.139	-.042	-.552	.581
4	(Constant)	4.428	.139		31.876	.000
	Ideology0	-.604	.073	-.605	-8.259	.000
	GenderCC	-.033	.114	-.021	-.287	.774
	RaceCC	.091	.111	.050	.818	.414
	SES0	.036	.108	.019	.339	.735
	MGRS0	.091	.195	.038	.463	.644
	MRN0	-.043	.151	-.023	-.282	.778
	MRN0xRace	.075	.126	.041	.598	.550
	MRN0xSES0	.041	.114	.021	.359	.720
	MRN0xGender	-.443	.150	-.217	-2.953	.004
	MRN0xIdeology0	-.018	.067	-.018	-.270	.788
	MRN0xMGRS0	-.148	.220	-.061	-.676	.500
	MGRS0xGender	.150	.181	.059	.828	.409
	MGRS0xRace	.031	.169	.013	.183	.855
	MGRS0xSES0	.006	.177	.002	.031	.975
	MGRS0xIdeology0	.074	.117	.054	.636	.526

Coefficients[a]

Model		Correlations		
		Zero-order	Partial	Part
1	(Constant)			
	Ideology0	-.606	-.606	-.606
2	(Constant)			
	Ideology0	-.606	-.578	-.560
	GenderCC	-.209	-.047	-.037
	RaceCC	-.132	.086	.068
	SES0	-.026	.023	.018
3	(Constant)			
	Ideology0	-.606	-.513	-.473
	GenderCC	-.209	-.023	-.018
	RaceCC	-.132	.081	.064
	SES0	-.026	.027	.021
	MGRS0	.035	.008	.006
	MRN0	-.365	-.039	-.030
4	(Constant)			
	Ideology0	-.606	-.508	-.451
	GenderCC	-.209	-.020	-.016
	RaceCC	-.132	.058	.045
	SES0	-.026	.024	.018
	MGRS0	.035	.033	.025
	MRN0	-.365	-.020	-.015
	MRN0xRace	-.240	.043	.033
	MRN0xSES0	.122	.026	.020
	MRN0xGender	-.263	-.206	-.161
	MRN0xIdeology0	-.074	-.019	-.015
	MRN0xMGRS0	.076	-.048	-.037
	MGRS0xGender	-.082	.059	.045
	MGRS0xRace	-.005	.013	.010
	MGRS0xSES0	.085	.002	.002
	MGRS0xIdeology0	.004	.045	.035

a. Dependent Variable: DemCongressX

Excluded Variables[a]

Model		Beta In	t	Sig.	Partial Correlation	Collinearity Statistics Tolerance
1	GenderCC	-.042[b]	-.742	.459	-.051	.921
	RaceCC	.075[b]	1.293	.197	.089	.892
	SES0	.020[b]	.354	.724	.024	.994
	MGRS0	.002[b]	.042	.966	.003	.997
	MRN0	-.058[b]	-.890	.374	-.061	.714
	MRN0xRace	-.016[b]	-.269	.788	-.019	.861
	MRN0xSES0	.039[b]	.712	.478	.049	.981
	MRN0xGender	-.197[b]	-3.675	.000	-.246	.987
	MRN0xIdeology0	-.035[b]	-.632	.528	-.044	.996
	MRN0xMGRS0	.049[b]	.898	.370	.062	.998
	MGRS0xGender	-.034[b]	-.612	.541	-.042	.994
	MGRS0xRace	.045[b]	.820	.413	.057	.993
	MGRS0xSES0	.037[b]	.675	.500	.047	.994
	MGRS0xIdeology0	.083[b]	1.504	.134	.103	.983
2	MGRS0	-.006[c]	-.094	.925	-.007	.867
	MRN0	-.039[c]	-.551	.582	-.038	.605
	MRN0xRace	.000[c]	-.002	.998	.000	.832
	MRN0xSES0	.043[c]	.775	.439	.054	.978
	MRN0xGender	-.194[c]	-3.567	.000	-.241	.971
	MRN0xIdeology0	-.038[c]	-.677	.499	-.047	.963
	MRN0xMGRS0	.057[c]	1.021	.308	.071	.969
	MGRS0xGender	-.036[c]	-.647	.518	-.045	.985
	MGRS0xRace	.037[c]	.664	.508	.046	.965
	MGRS0xSES0	.041[c]	.724	.470	.050	.957
	MGRS0xIdeology0	.082[c]	1.469	.143	.102	.969
3	MRN0xRace	.009[d]	.144	.885	.010	.776
	MRN0xSES0	.039[d]	.693	.489	.048	.942
	MRN0xGender	-.195[d]	-3.520	.001	-.239	.941
	MRN0xIdeology0	-.045[d]	-.788	.431	-.055	.931
	MRN0xMGRS0	.056[d]	.995	.321	.069	.966
	MGRS0xGender	-.033[d]	-.579	.563	-.041	.944

Excluded Variables[a]

Model		Beta In	t	Sig.	Partial Correlation	Collinearity Statistics Tolerance
	MGRS0xRace	.050[d]	.764	.446	.053	.706
	MGRS0xSES0	.040[d]	.686	.494	.048	.884
	MGRS0xIdeology0	.080[d]	1.414	.159	.099	.938

a. Dependent Variable: DemCongressX

b. Predictors in the Model: (Constant), Ideology0

c. Predictors in the Model: (Constant), Ideology0, SES0, GenderCC, RaceCC

d. Predictors in the Model: (Constant), Ideology0, SES0, GenderCC, RaceCC, MGRS0, MRN0

```
REGRESSION
  /MISSING LISTWISE
  /STATISTICS COEFF OUTS R ANOVA CHANGE ZPP
  /CRITERIA=PIN(.05) POUT(.10)
  /NOORIGIN
  /DEPENDENT StateX
  /METHOD=ENTER Ideology0
  /METHOD=ENTER GenderCC RaceCC SES0
  /METHOD=ENTER MGRS0 MRN0
  /METHOD=ENTER MRN0xRace MRN0xSES0 MRN0xGender MRN0xIdeology0 MRN0xMGRS0 MGRS0xGender MG
RS0xRace MGRS0xSES0 MGRS0xIdeology0.
```

Regression

Notes

Output Created		15-DEC-2021 13:07:59
Comments		
Input	Data	C:\Users\njs5478\Dropbox\HM and COVID\0. Revise and Resubmit\2. R and R Data\Study 1b\Study1b_Data.sav
	Active Dataset	DataSet1
	Filter	<none>
	Weight	<none>
	Split File	<none>
	N of Rows in Working Data File	241
Missing Value Handling	Definition of Missing	User-defined missing values are treated as missing.
	Cases Used	Statistics are based on cases with no missing values for any variable used.
Syntax		REGRESSION /MISSING LISTWISE /STATISTICS COEFF OUTS R ANOVA CHANGE ZPP /CRITERIA=PIN(.05) POUT(.10) /NOORIGIN /DEPENDENT StateX /METHOD=ENTER Ideology0 /METHOD=ENTER GenderCC RaceCC SES0 /METHOD=ENTER MGRS0 MRN0 /METHOD=ENTER MRN0xRace MRN0xSES0 MRN0xGender MRN0xIdeology0 MRN0xMGRS0 MGRS0xGender MGRS0xRace MGRS0xSES0 MGRS0xIdeology0.
Resources	Processor Time	00:00:00.02
	Elapsed Time	00:00:00.05

Notes

	Memory Required	52240 bytes
	Additional Memory Required for Residual Plots	0 bytes

Variables Entered/Removed[a]

Model	Variables Entered	Variables Removed	Method
1	Ideology0[b]	.	Enter
2	SES0, GenderCC, RaceCC[b]	.	Enter
3	MGRS0, MRN0[b]	.	Enter
4	MGRS0xGender, MGRS0xIdeology0, MRN0xSES0, MRN0xIdeology0, MGRS0xSES0, MGRS0xRace, MRN0xGender, MRN0xRace, MRN0xMGRS0[b]	.	Enter

a. Dependent Variable: StateX

b. All requested variables entered.

Model Summary

Model	R	R Square	Adjusted R Square	Std. Error of the Estimate	Change Statistics		
					R Square Change	F Change	df1
1	.302[a]	.091	.087	1.53232	.091	22.008	1
2	.304[b]	.092	.075	1.54188	.001	.094	3
3	.304[c]	.092	.067	1.54892	.000	.016	2
4	.374[d]	.140	.077	1.54033	.048	1.267	9

Model Summary

Model	Change Statistics	
	df2	Sig. F Change
1	220	.000
2	217	.963
3	215	.984
4	206	.257

a. Predictors: (Constant), Ideology0

b. Predictors: (Constant), Ideology0, SES0, GenderCC, RaceCC

c. Predictors: (Constant), Ideology0, SES0, GenderCC, RaceCC, MGRS0, MRN0

d. Predictors: (Constant), Ideology0, SES0, GenderCC, RaceCC, MGRS0, MRN0, MGRS0xGender, MGRS0xIdeology0, MRN0xSES0, MRN0xIdeology0, MGRS0xSES0, MGRS0xRace, MRN0xGender, MRN0xRace, MRN0xMGRS0

ANOVA[a]

Model		Sum of Squares	df	Mean Square	F	Sig.
1	Regression	51.675	1	51.675	22.008	.000[b]
	Residual	516.564	220	2.348		
	Total	568.239	221			
2	Regression	52.346	4	13.086	5.505	.000[c]
	Residual	515.893	217	2.377		
	Total	568.239	221			
3	Regression	52.423	6	8.737	3.642	.002[d]
	Residual	515.816	215	2.399		
	Total	568.239	221			
4	Regression	79.481	15	5.299	2.233	.006[e]
	Residual	488.757	206	2.373		
	Total	568.239	221			

a. Dependent Variable: StateX

b. Predictors: (Constant), Ideology0

c. Predictors: (Constant), Ideology0, SES0, GenderCC, RaceCC

d. Predictors: (Constant), Ideology0, SES0, GenderCC, RaceCC, MGRS0, MRN0

e. Predictors: (Constant), Ideology0, SES0, GenderCC, RaceCC, MGRS0, MRN0, MGRS0xGender, MGRS0xIdeology0, MRN0xSES0, MRN0xIdeology0, MGRS0xSES0, MGRS0xRace, MRN0xGender, MRN0xRace, MRN0xMGRS0

Coefficients[a]

Model		Unstandardized Coefficients B	Std. Error	Standardized Coefficients Beta	t	Sig.
1	(Constant)	4.064	.103		39.518	.000
	Ideology0	-.306	.065	-.302	-4.691	.000
2	(Constant)	4.097	.121		33.739	.000
	Ideology0	-.292	.072	-.288	-4.080	.000
	GenderCC	-.020	.107	-.012	-.182	.856
	RaceCC	-.064	.126	-.035	-.509	.611
	SES0	.003	.126	.002	.023	.982
3	(Constant)	4.097	.122		33.473	.000
	Ideology0	-.287	.084	-.283	-3.435	.001
	GenderCC	-.007	.128	-.004	-.056	.955
	RaceCC	-.065	.128	-.035	-.506	.613
	SES0	.006	.128	.003	.046	.963
	MGRS0	.028	.183	.011	.153	.878
	MRN0	-.023	.163	-.012	-.140	.888
4	(Constant)	4.143	.163		25.340	.000
	Ideology0	-.311	.088	-.306	-3.549	.000
	GenderCC	-.062	.134	-.039	-.464	.643
	RaceCC	-.097	.133	-.052	-.726	.469
	SES0	.026	.131	.013	.198	.843
	MGRS0	-.013	.242	-.005	-.055	.956
	MRN0	.069	.182	.038	.382	.703
	MRN0xRace	.112	.153	.061	.729	.467
	MRN0xSES0	.062	.139	.031	.445	.657
	MRN0xGender	-.332	.173	-.165	-1.927	.055
	MRN0xIdeology0	.081	.078	.081	1.035	.302
	MRN0xMGRS0	.006	.264	.003	.024	.981
	MGRS0xGender	-.095	.217	-.036	-.440	.661
	MGRS0xRace	.023	.209	.009	.109	.913
	MGRS0xSES0	-.208	.217	-.070	-.958	.339
	MGRS0xIdeology0	.080	.141	.056	.565	.573

Coefficients[a]

Model		Correlations Zero-order	Partial	Part
1	(Constant)			
	Ideology0	-.302	-.302	-.302
2	(Constant)			
	Ideology0	-.302	-.267	-.264
	GenderCC	-.088	-.012	-.012
	RaceCC	-.122	-.035	-.033
	SES0	-.024	.002	.002
3	(Constant)			
	Ideology0	-.302	-.228	-.223
	GenderCC	-.088	-.004	-.004
	RaceCC	-.122	-.034	-.033
	SES0	-.024	.003	.003
	MGRS0	.026	.010	.010
	MRN0	-.165	-.010	-.009
4	(Constant)			
	Ideology0	-.302	-.240	-.229
	GenderCC	-.088	-.032	-.030
	RaceCC	-.122	-.050	-.047
	SES0	-.024	.014	.013
	MGRS0	.026	-.004	-.004
	MRN0	-.165	.027	.025
	MRN0xRace	-.076	.051	.047
	MRN0xSES0	.091	.031	.029
	MRN0xGender	-.194	-.133	-.124
	MRN0xIdeology0	.057	.072	.067
	MRN0xMGRS0	.089	.002	.002
	MGRS0xGender	-.121	-.031	-.028
	MGRS0xRace	.021	.008	.007
	MGRS0xSES0	-.004	-.067	-.062
	MGRS0xIdeology0	.079	.039	.037

a. Dependent Variable: StateX

Excluded Variables[a]

Model		Beta In	t	Sig.	Partial Correlation	Collinearity Statistics Tolerance
1	GenderCC	-.010[b]	-.153	.879	-.010	.932
	RaceCC	-.034[b]	-.501	.617	-.034	.907
	SES0	.001[b]	.008	.994	.001	.993
	MGRS0	.013[b]	.205	.838	.014	.998
	MRN0	-.005[b]	-.064	.949	-.004	.714
	MRN0xRace	.063[b]	.885	.377	.060	.821
	MRN0xSES0	.051[b]	.781	.436	.053	.981
	MRN0xGender	-.153[b]	-2.379	.018	-.159	.978
	MRN0xIdeology0	.075[b]	1.165	.245	.079	.997
	MRN0xMGRS0	.060[b]	.924	.357	.062	.990
	MGRS0xGender	-.105[b]	-1.639	.103	-.110	.997
	MGRS0xRace	.040[b]	.620	.536	.042	.996
	MGRS0xSES0	-.027[b]	-.422	.673	-.029	.994
	MGRS0xIdeology0	.099[b]	1.545	.124	.104	.996
2	MGRS0	.008[c]	.111	.912	.008	.867
	MRN0	-.008[c]	-.092	.926	-.006	.612
	MRN0xRace	.059[c]	.819	.414	.056	.797
	MRN0xSES0	.051[c]	.775	.439	.053	.978
	MRN0xGender	-.161[c]	-2.461	.015	-.165	.960
	MRN0xIdeology0	.085[c]	1.283	.201	.087	.949
	MRN0xMGRS0	.063[c]	.959	.339	.065	.963
	MGRS0xGender	-.106[c]	-1.635	.104	-.111	.990
	MGRS0xRace	.040[c]	.606	.545	.041	.965
	MGRS0xSES0	-.024[c]	-.360	.719	-.024	.955
	MGRS0xIdeology0	.103[c]	1.589	.113	.108	.983
3	MRN0xRace	.069[d]	.895	.372	.061	.717
	MRN0xSES0	.052[d]	.781	.435	.053	.944
	MRN0xGender	-.166[d]	-2.486	.014	-.168	.923
	MRN0xIdeology0	.086[d]	1.279	.202	.087	.925
	MRN0xMGRS0	.063[d]	.943	.347	.064	.955
	MGRS0xGender	-.107[d]	-1.622	.106	-.110	.955

Excluded Variables[a]

Model		Beta In	t	Sig.	Partial Correlation	Collinearity Statistics Tolerance
	MGRS0xRace	.050[d]	.631	.528	.043	.676
	MGRS0xSES0	-.024[d]	-.350	.726	-.024	.878
	MGRS0xIdeology0	.107[d]	1.605	.110	.109	.951

a. Dependent Variable: StateX

b. Predictors in the Model: (Constant), Ideology0

c. Predictors in the Model: (Constant), Ideology0, SES0, GenderCC, RaceCC

d. Predictors in the Model: (Constant), Ideology0, SES0, GenderCC, RaceCC, MGRS0, MRN0

```
REGRESSION
  /MISSING LISTWISE
  /STATISTICS COEFF OUTS R ANOVA CHANGE ZPP
  /CRITERIA=PIN(.05) POUT(.10)
  /NOORIGIN
  /DEPENDENT Risk_Rules
  /METHOD=ENTER Ideology0
  /METHOD=ENTER GenderCC RaceCC SES0
  /METHOD=ENTER MGRS0 MRN0
  /METHOD=ENTER MRN0xRace MRN0xSES0 MRN0xGender MRN0xIdeology0 MRN0xMGRS0 MGRS0xGender MG
RS0xRace MGRS0xSES0 MGRS0xIdeology0.
```

Regression

Notes

Output Created		15-DEC-2021 13:07:59
Comments		
Input	Data	C: \Users\njs5478\Dropbox\HM and COVID\0. Revise and Resubmit\2. R and R Data\Study 1b\Study1b_Data.sav
	Active Dataset	DataSet1
	Filter	<none>
	Weight	<none>
	Split File	<none>
	N of Rows in Working Data File	241
Missing Value Handling	Definition of Missing	User-defined missing values are treated as missing.
	Cases Used	Statistics are based on cases with no missing values for any variable used.
Syntax		REGRESSION /MISSING LISTWISE /STATISTICS COEFF OUTS R ANOVA CHANGE ZPP /CRITERIA=PIN(.05) POUT(.10) /NOORIGIN /DEPENDENT Risk_Rules /METHOD=ENTER Ideology0 /METHOD=ENTER GenderCC RaceCC SES0 /METHOD=ENTER MGRS0 MRN0 /METHOD=ENTER MRN0xRace MRN0xSES0 MRN0xGender MRN0xIdeology0 MRN0xMGRS0 MGRS0xGender MGRS0xRace MGRS0xSES0 MGRS0xIdeology0.

Notes

Resources	Processor Time	00:00:00.03
	Elapsed Time	00:00:00.03
	Memory Required	52240 bytes
	Additional Memory Required for Residual Plots	0 bytes

Variables Entered/Removed[a]

Model	Variables Entered	Variables Removed	Method
1	Ideology0[b]	.	Enter
2	SES0, GenderCC, RaceCC[b]	.	Enter
3	MGRS0, MRN0[b]	.	Enter
4	MRN0xMGRS0, MRN0xSES0, MRN0xIdeology0, MGRS0xSES0, MGRS0xGender, MGRS0xRace, MRN0xRace, MRN0xGender, MGRS0xIdeol...[b]	.	Enter

a. Dependent Variable: Risk_Rules

b. All requested variables entered.

Model Summary

Model	R	R Square	Adjusted R Square	Std. Error of the Estimate	Change Statistics		
					R Square Change	F Change	df1
1	.474[a]	.225	.221	.88403	.225	68.134	1
2	.484[b]	.235	.221	.88407	.010	.992	3
3	.552[c]	.305	.287	.84614	.070	11.635	2
4	.574[d]	.329	.284	.84803	.024	.886	9

Model Summary

Model	Change Statistics	
	df2	Sig. F Change
1	235	.000
2	232	.397
3	230	.000
4	221	.539

a. Predictors: (Constant), Ideology0

b. Predictors: (Constant), Ideology0, SES0, GenderCC, RaceCC

c. Predictors: (Constant), Ideology0, SES0, GenderCC, RaceCC, MGRS0, MRN0

d. Predictors: (Constant), Ideology0, SES0, GenderCC, RaceCC, MGRS0, MRN0, MRN0xMGRS0, MRN0xSES0, MRN0xIdeology0, MGRS0xSES0, MGRS0xGender, MGRS0xRace, MRN0xRace, MRN0xGender, MGRS0xIdeology0

ANOVA[a]

Model		Sum of Squares	df	Mean Square	F	Sig.
1	Regression	53.248	1	53.248	68.134	.000[b]
	Residual	183.655	235	.782		
	Total	236.903	236			
2	Regression	55.575	4	13.894	17.776	.000[c]
	Residual	181.328	232	.782		
	Total	236.903	236			
3	Regression	72.235	6	12.039	16.816	.000[d]
	Residual	164.669	230	.716		
	Total	236.903	236			
4	Regression	77.968	15	5.198	7.228	.000[e]
	Residual	158.935	221	.719		
	Total	236.903	236			

a. Dependent Variable: Risk_Rules

b. Predictors: (Constant), Ideology0

c. Predictors: (Constant), Ideology0, SES0, GenderCC, RaceCC

d. Predictors: (Constant), Ideology0, SES0, GenderCC, RaceCC, MGRS0, MRN0

e. Predictors: (Constant), Ideology0, SES0, GenderCC, RaceCC, MGRS0, MRN0, MRN0xMGRS0, MRN0xSES0, MRN0xIdeology0, MGRS0xSES0, MGRS0xGender, MGRS0xRace, MRN0xRace, MRN0xGender, MGRS0xIdeology0

Coefficients[a]

Model		Unstandardized Coefficients B	Unstandardized Coefficients Std. Error	Standardized Coefficients Beta	t	Sig.
1	(Constant)	3.303	.057		57.511	.000
	Ideology0	.298	.036	.474	8.254	.000
2	(Constant)	3.292	.067		49.088	.000
	Ideology0	.280	.040	.444	7.045	.000
	GenderCC	.098	.060	.098	1.649	.100
	RaceCC	.021	.070	.018	.293	.769
	SES0	-.038	.071	-.031	-.536	.593
3	(Constant)	3.270	.064		50.772	.000
	Ideology0	.170	.044	.270	3.839	.000
	GenderCC	-.015	.067	-.015	-.227	.821
	RaceCC	.069	.068	.060	1.010	.313
	SES0	-.075	.069	-.061	-1.089	.277
	MGRS0	-.052	.097	-.034	-.537	.592
	MRN0	.406	.086	.352	4.703	.000
4	(Constant)	3.314	.087		38.316	.000
	Ideology0	.153	.046	.244	3.303	.001
	GenderCC	.027	.071	.027	.376	.707
	RaceCC	.081	.071	.071	1.139	.256
	SES0	-.084	.071	-.068	-1.192	.235
	MGRS0	.022	.127	.014	.175	.861
	MRN0	.319	.097	.277	3.308	.001
	MRN0xRace	.125	.082	.108	1.526	.128
	MRN0xSES0	-.054	.076	-.042	-.710	.478
	MRN0xGender	-.041	.091	-.032	-.450	.653
	MRN0xIdeology0	.008	.042	.013	.190	.849
	MRN0xMGRS0	-.110	.143	-.070	-.766	.445
	MGRS0xGender	.186	.117	.112	1.590	.113
	MGRS0xRace	-.079	.111	-.051	-.715	.476
	MGRS0xSES0	-.035	.118	-.018	-.298	.766
	MGRS0xIdeology0	-.011	.076	-.012	-.145	.885

Coefficients[a]

Model		Correlations Zero-order	Partial	Part
1	(Constant)			
	Ideology0	.474	.474	.474
2	(Constant)			
	Ideology0	.474	.420	.405
	GenderCC	.214	.108	.095
	RaceCC	.165	.019	.017
	SES0	.000	-.035	-.031
3	(Constant)			
	Ideology0	.474	.245	.211
	GenderCC	.214	-.015	-.012
	RaceCC	.165	.066	.056
	SES0	.000	-.072	-.060
	MGRS0	-.004	-.035	-.029
	MRN0	.483	.296	.259
4	(Constant)			
	Ideology0	.474	.217	.182
	GenderCC	.214	.025	.021
	RaceCC	.165	.076	.063
	SES0	.000	-.080	-.066
	MGRS0	-.004	.012	.010
	MRN0	.483	.217	.182
	MRN0xRace	.340	.102	.084
	MRN0xSES0	-.159	-.048	-.039
	MRN0xGender	.129	-.030	-.025
	MRN0xIdeology0	.000	.013	.010
	MRN0xMGRS0	-.080	-.051	-.042
	MGRS0xGender	.137	.106	.088
	MGRS0xRace	-.012	-.048	-.039
	MGRS0xSES0	-.068	-.020	-.016
	MGRS0xIdeology0	-.046	-.010	-.008

a. Dependent Variable: Risk_Rules

Excluded Variables[a]

Model		Beta In	t	Sig.	Partial Correlation	Collinearity Statistics Tolerance
1	GenderCC	.096[b]	1.621	.106	.105	.931
	RaceCC	.012[b]	.196	.845	.013	.893
	SES0	-.027[b]	-.463	.644	-.030	.997
	MGRS0	.023[b]	.406	.685	.027	.997
	MRN0	.321[b]	4.929	.000	.307	.709
	MRN0xRace	.180[b]	2.914	.004	.187	.842
	MRN0xSES0	-.099[b]	-1.712	.088	-.111	.983
	MRN0xGender	.085[b]	1.480	.140	.096	.991
	MRN0xIdeology0	-.015[b]	-.266	.791	-.017	.999
	MRN0xMGRS0	-.048[b]	-.829	.408	-.054	.995
	MGRS0xGender	.104[b]	1.809	.072	.117	.995
	MGRS0xRace	-.041[b]	-.720	.472	-.047	.996
	MGRS0xSES0	-.034[b]	-.592	.554	-.039	.995
	MGRS0xIdeology0	-.081[b]	-1.412	.159	-.092	.995
2	MGRS0	.063[c]	1.026	.306	.067	.868
	MRN0	.339[c]	4.801	.000	.301	.605
	MRN0xRace	.185[c]	2.942	.004	.190	.812
	MRN0xSES0	-.105[c]	-1.820	.070	-.119	.979
	MRN0xGender	.093[c]	1.607	.109	.105	.973
	MRN0xIdeology0	-.029[c]	-.491	.624	-.032	.961
	MRN0xMGRS0	-.065[c]	-1.108	.269	-.073	.966
	MGRS0xGender	.108[c]	1.882	.061	.123	.987
	MGRS0xRace	-.028[c]	-.486	.627	-.032	.973
	MGRS0xSES0	-.052[c]	-.880	.380	-.058	.959
	MGRS0xIdeology0	-.090[c]	-1.559	.120	-.102	.981
3	MRN0xRace	.104[d]	1.626	.105	.107	.734
	MRN0xSES0	-.065[d]	-1.145	.253	-.075	.946
	MRN0xGender	.051[d]	.898	.370	.059	.942
	MRN0xIdeology0	.016[d]	.287	.775	.019	.935
	MRN0xMGRS0	-.048[d]	-.857	.393	-.057	.961
	MGRS0xGender	.081[d]	1.438	.152	.095	.945

Excluded Variables[a]

Model		Beta In	t	Sig.	Partial Correlation	Collinearity Statistics Tolerance
	MGRS0xRace	-.052[d]	-.797	.426	-.053	.711
	MGRS0xSES0	-.019[d]	-.331	.741	-.022	.890
	MGRS0xIdeology0	-.057[d]	-1.012	.313	-.067	.950

a. Dependent Variable: Risk_Rules

b. Predictors in the Model: (Constant), Ideology0

c. Predictors in the Model: (Constant), Ideology0, SES0, GenderCC, RaceCC

d. Predictors in the Model: (Constant), Ideology0, SES0, GenderCC, RaceCC, MGRS0, MRN0

```
REGRESSION
  /MISSING LISTWISE
  /STATISTICS COEFF OUTS R ANOVA CHANGE ZPP
  /CRITERIA=PIN(.05) POUT(.10)
  /NOORIGIN
  /DEPENDENT Risk_Help
  /METHOD=ENTER Ideology0
  /METHOD=ENTER GenderCC RaceCC SES0
  /METHOD=ENTER MGRS0 MRN0
  /METHOD=ENTER MRN0xRace MRN0xSES0 MRN0xGender MRN0xIdeology0 MRN0xMGRS0 MGRS0xGender MG
RS0xRace MGRS0xSES0 MGRS0xIdeology0.
```

Regression

Notes

Output Created		15-DEC-2021 13:07:59
Comments		
Input	Data	C:\Users\njs5478\Dropbox\HM and COVID\0. Revise and Resubmit\2. R and R Data\Study 1b\Study1b_Data.sav
	Active Dataset	DataSet1
	Filter	<none>
	Weight	<none>
	Split File	<none>
	N of Rows in Working Data File	241
Missing Value Handling	Definition of Missing	User-defined missing values are treated as missing.
	Cases Used	Statistics are based on cases with no missing values for any variable used.
Syntax		REGRESSION<br>/MISSING LISTWISE<br>/STATISTICS COEFF OUTS R ANOVA CHANGE ZPP<br>/CRITERIA=PIN(.05) POUT(.10)<br>/NOORIGIN<br>/DEPENDENT Risk_Help<br>/METHOD=ENTER Ideology0<br>/METHOD=ENTER GenderCC RaceCC SES0<br>/METHOD=ENTER MGRS0 MRN0<br>/METHOD=ENTER MRN0xRace MRN0xSES0 MRN0xGender MRN0xIdeology0 MRN0xMGRS0 MGRS0xGender MGRS0xRace MGRS0xSES0 MGRS0xIdeology0.

Notes

Resources	Processor Time	00:00:00.02
	Elapsed Time	00:00:00.02
	Memory Required	52240 bytes
	Additional Memory Required for Residual Plots	0 bytes

Variables Entered/Removed[a]

Model	Variables Entered	Variables Removed	Method
1	Ideology0[b]	.	Enter
2	SES0, GenderCC, RaceCC[b]	.	Enter
3	MGRS0, MRN0[b]	.	Enter
4	MRN0xMGRS0, MRN0xSES0, MRN0xIdeology0, MGRS0xSES0, MGRS0xGender, MGRS0xRace, MRN0xRace, MRN0xGender, MGRS0xIdeol...[b]	.	Enter

a. Dependent Variable: Risk_Help

b. All requested variables entered.

Model Summary

Model	R	R Square	Adjusted R Square	Std. Error of the Estimate	Change Statistics		
					R Square Change	F Change	df1
1	.159[a]	.025	.021	1.49292	.025	6.120	1
2	.274[b]	.075	.059	1.46360	.050	4.170	3
3	.281[c]	.079	.055	1.46685	.004	.486	2
4	.327[d]	.107	.046	1.47391	.028	.756	9

Model Summary

Model	Change Statistics	
	df2	Sig. F Change
1	235	.014
2	232	.007
3	230	.616
4	221	.657

a. Predictors: (Constant), Ideology0

b. Predictors: (Constant), Ideology0, SES0, GenderCC, RaceCC

c. Predictors: (Constant), Ideology0, SES0, GenderCC, RaceCC, MGRS0, MRN0

d. Predictors: (Constant), Ideology0, SES0, GenderCC, RaceCC, MGRS0, MRN0, MRN0xMGRS0, MRN0xSES0, MRN0xIdeology0, MGRS0xSES0, MGRS0xGender, MGRS0xRace, MRN0xRace, MRN0xGender, MGRS0xIdeology0

ANOVA[a]

Model		Sum of Squares	df	Mean Square	F	Sig.
1	Regression	13.641	1	13.641	6.120	.014[b]
	Residual	523.772	235	2.229		
	Total	537.413	236			
2	Regression	40.440	4	10.110	4.720	.001[c]
	Residual	496.973	232	2.142		
	Total	537.413	236			
3	Regression	42.532	6	7.089	3.295	.004[d]
	Residual	494.881	230	2.152		
	Total	537.413	236			
4	Regression	57.312	15	3.821	1.759	.042[e]
	Residual	480.101	221	2.172		
	Total	537.413	236			

a. Dependent Variable: Risk_Help

b. Predictors: (Constant), Ideology0

c. Predictors: (Constant), Ideology0, SES0, GenderCC, RaceCC

d. Predictors: (Constant), Ideology0, SES0, GenderCC, RaceCC, MGRS0, MRN0

e. Predictors: (Constant), Ideology0, SES0, GenderCC, RaceCC, MGRS0, MRN0, MRN0xMGRS0, MRN0xSES0, MRN0xIdeology0, MGRS0xSES0, MGRS0xGender, MGRS0xRace, MRN0xRace, MRN0xGender, MGRS0xIdeology0

Coefficients[a]

Model		Unstandardized Coefficients B	Std. Error	Standardized Coefficients Beta	t	Sig.
1	(Constant)	3.798	.097		39.164	.000
	Ideology0	-.151	.061	-.159	-2.474	.014
2	(Constant)	3.936	.111		35.452	.000
	Ideology0	-.072	.066	-.076	-1.090	.277
	GenderCC	-.127	.099	-.084	-1.283	.201
	RaceCC	-.287	.116	-.166	-2.478	.014
	SES0	-.254	.118	-.136	-2.152	.032
3	(Constant)	3.937	.112		35.265	.000
	Ideology0	-.090	.077	-.095	-1.169	.243
	GenderCC	-.184	.117	-.122	-1.578	.116
	RaceCC	-.289	.118	-.167	-2.452	.015
	SES0	-.268	.119	-.144	-2.242	.026
	MGRS0	-.160	.168	-.068	-.951	.342
	MRN0	.084	.150	.048	.559	.577
4	(Constant)	3.915	.150		26.042	.000
	Ideology0	-.071	.081	-.075	-.883	.378
	GenderCC	-.210	.123	-.140	-1.706	.089
	RaceCC	-.294	.124	-.170	-2.367	.019
	SES0	-.299	.123	-.160	-2.429	.016
	MGRS0	-.286	.220	-.123	-1.298	.196
	MRN0	.177	.168	.102	1.054	.293
	MRN0xRace	-.193	.142	-.111	-1.354	.177
	MRN0xSES0	.139	.131	.072	1.058	.291
	MRN0xGender	.071	.159	.037	.448	.654
	MRN0xIdeology0	-.047	.073	-.050	-.646	.519
	MRN0xMGRS0	.012	.249	.005	.049	.961
	MGRS0xGender	-.146	.203	-.059	-.718	.473
	MGRS0xRace	.142	.193	.060	.734	.464
	MGRS0xSES0	-.175	.204	-.061	-.858	.392
	MGRS0xIdeology0	.051	.132	.038	.382	.703

Coefficients[a]

Model		Correlations		
		Zero-order	Partial	Part
1	(Constant)			
	Ideology0	-.159	-.159	-.159
2	(Constant)			
	Ideology0	-.159	-.071	-.069
	GenderCC	-.117	-.084	-.081
	RaceCC	-.198	-.161	-.156
	SES0	-.151	-.140	-.136
3	(Constant)			
	Ideology0	-.159	-.077	-.074
	GenderCC	-.117	-.103	-.100
	RaceCC	-.198	-.160	-.155
	SES0	-.151	-.146	-.142
	MGRS0	.011	-.063	-.060
	MRN0	-.087	.037	.035
4	(Constant)			
	Ideology0	-.159	-.059	-.056
	GenderCC	-.117	-.114	-.108
	RaceCC	-.198	-.157	-.150
	SES0	-.151	-.161	-.154
	MGRS0	.011	-.087	-.083
	MRN0	-.087	.071	.067
	MRN0xRace	-.122	-.091	-.086
	MRN0xSES0	.087	.071	.067
	MRN0xGender	-.011	.030	.029
	MRN0xIdeology0	-.072	-.043	-.041
	MRN0xMGRS0	.008	.003	.003
	MGRS0xGender	-.062	-.048	-.046
	MGRS0xRace	.019	.049	.047
	MGRS0xSES0	-.042	-.058	-.055
	MGRS0xIdeology0	.014	.026	.024

a. Dependent Variable: Risk_Help

Excluded Variables[a]

Model		Beta In	t	Sig.	Partial Correlation	Collinearity Statistics Tolerance
1	GenderCC	-.081[b]	-1.218	.225	-.079	.931
	RaceCC	-.163[b]	-2.424	.016	-.157	.893
	SES0	-.142[b]	-2.222	.027	-.144	.997
	MGRS0	.001[b]	.022	.982	.001	.997
	MRN0	-.002[b]	-.029	.977	-.002	.709
	MRN0xRace	-.069[b]	-.988	.324	-.064	.842
	MRN0xSES0	.067[b]	1.033	.303	.067	.983
	MRN0xGender	.004[b]	.055	.956	.004	.991
	MRN0xIdeology0	-.067[b]	-1.040	.300	-.068	.999
	MRN0xMGRS0	-.003[b]	-.053	.958	-.003	.995
	MGRS0xGender	-.051[b]	-.782	.435	-.051	.995
	MGRS0xRace	.029[b]	.445	.657	.029	.996
	MGRS0xSES0	-.053[b]	-.824	.411	-.054	.995
	MGRS0xIdeology0	.026[b]	.406	.685	.027	.995
2	MGRS0	-.055[c]	-.814	.417	-.053	.868
	MRN0	.021[c]	.260	.795	.017	.605
	MRN0xRace	-.104[c]	-1.494	.137	-.098	.812
	MRN0xSES0	.070[c]	1.098	.273	.072	.979
	MRN0xGender	-.014[c]	-.213	.832	-.014	.973
	MRN0xIdeology0	-.060[c]	-.934	.351	-.061	.961
	MRN0xMGRS0	-.006[c]	-.095	.924	-.006	.966
	MGRS0xGender	-.036[c]	-.561	.576	-.037	.987
	MGRS0xRace	.023[c]	.357	.722	.023	.973
	MGRS0xSES0	-.050[c]	-.780	.436	-.051	.959
	MGRS0xIdeology0	.033[c]	.513	.608	.034	.981
3	MRN0xRace	-.122[d]	-1.661	.098	-.109	.734
	MRN0xSES0	.071[d]	1.088	.278	.072	.946
	MRN0xGender	-.014[d]	-.208	.835	-.014	.942
	MRN0xIdeology0	-.058[d]	-.889	.375	-.059	.935
	MRN0xMGRS0	-.003[d]	-.052	.958	-.003	.961
	MGRS0xGender	-.050[d]	-.775	.439	-.051	.945

Excluded Variables[a]

Model		Beta In	t	Sig.	Partial Correlation	Collinearity Statistics Tolerance
	MGRS0xRace	.072[d]	.956	.340	.063	.711
	MGRS0xSES0	-.066[d]	-.988	.324	-.065	.890
	MGRS0xIdeology0	.030[d]	.456	.649	.030	.950

a. Dependent Variable: Risk_Help

b. Predictors in the Model: (Constant), Ideology0

c. Predictors in the Model: (Constant), Ideology0, SES0, GenderCC, RaceCC

d. Predictors in the Model: (Constant), Ideology0, SES0, GenderCC, RaceCC, MGRS0, MRN0

```
 *FOR MEN ONLY

USE ALL.
COMPUTE filter_$=(GenderCC=1).
VARIABLE LABELS filter_$ 'GenderCC=1 (FILTER)'.
VALUE LABELS filter_$ 0 'Not Selected' 1 'Selected'.
FORMATS filter_$ (f1.0).
FILTER BY filter_$.
EXECUTE.

REGRESSION
  /MISSING LISTWISE
  /STATISTICS COEFF OUTS R ANOVA CHANGE ZPP
  /CRITERIA=PIN(.05) POUT(.10)
  /NOORIGIN
  /DEPENDENT Concern_Tot
  /METHOD=ENTER Ideology0
  /METHOD=ENTER RaceCC SES0
  /METHOD=ENTER MGRS0 MRN0
  /METHOD=ENTER MRN0xRace MRN0xSES0 MRN0xIdeology0 MRN0xMGRS0 MGRS0xRace MGRS0xSES0 MGRS0
xIdeology0.
```

Regression

Notes

Output Created		15-DEC-2021 13:07:59
Comments		
Input	Data	C: \Users\njs5478\Dropbox\HM and COVID\0. Revise and Resubmit\2. R and R Data\Study 1b\Study1b_Data.sav
	Active Dataset	DataSet1
	Filter	GenderCC=1 (FILTER)
	Weight	<none>
	Split File	<none>
	N of Rows in Working Data File	119
Missing Value Handling	Definition of Missing	User-defined missing values are treated as missing.
	Cases Used	Statistics are based on cases with no missing values for any variable used.
Syntax		REGRESSION /MISSING LISTWISE /STATISTICS COEFF OUTS R ANOVA CHANGE ZPP /CRITERIA=PIN(.05) POUT(.10) /NOORIGIN /DEPENDENT Concern_Tot /METHOD=ENTER Ideology0 /METHOD=ENTER RaceCC SES0 /METHOD=ENTER MGRS0 MRN0 /METHOD=ENTER MRN0xRace MRN0xSES0 MRN0xIdeology0 MRN0xMGRS0 MGRS0xRace MGRS0xSES0 MGRS0xIdeology0.
Resources	Processor Time	00:00:00.02
	Elapsed Time	00:00:00.02

Notes

	Memory Required	48560 bytes
	Additional Memory Required for Residual Plots	0 bytes

Variables Entered/Removed[a]

Model	Variables Entered	Variables Removed	Method
1	Ideology0[b]	.	Enter
2	SES0, RaceCC[b]	.	Enter
3	MGRS0, MRN0[b]	.	Enter
4	MRN0xSES0, MRN0xIdeology0, MGRS0xRace, MGRS0xIdeology0, MGRS0xSES0, MRN0xMGRS0, MRN0xRace[b]	.	Enter

a. Dependent Variable: Concern_Tot

b. All requested variables entered.

Model Summary

Model	R	R Square	Adjusted R Square	Std. Error of the Estimate	Change Statistics		
					R Square Change	F Change	df1
1	.585[a]	.342	.336	1.15442	.342	60.295	1
2	.591[b]	.349	.332	1.15827	.007	.615	2
3	.597[c]	.356	.328	1.16201	.007	.634	2
4	.637[d]	.406	.338	1.15254	.050	1.264	7

Model Summary

Model	Change Statistics	
	df2	Sig. F Change
1	116	.000
2	114	.542
3	112	.532
4	105	.275

a. Predictors: (Constant), Ideology0

b. Predictors: (Constant), Ideology0, SES0, RaceCC

c. Predictors: (Constant), Ideology0, SES0, RaceCC, MGRS0, MRN0

d. Predictors: (Constant), Ideology0, SES0, RaceCC, MGRS0, MRN0, MRN0xSES0, MRN0xIdeology0, MGRS0xRace, MGRS0xIdeology0, MGRS0xSES0, MRN0xMGRS0, MRN0xRace

ANOVA[a]

Model		Sum of Squares	df	Mean Square	F	Sig.
1	Regression	80.353	1	80.353	60.295	.000[b]
	Residual	154.591	116	1.333		
	Total	234.944	117			
2	Regression	82.004	3	27.335	20.375	.000[c]
	Residual	152.941	114	1.342		
	Total	234.944	117			
3	Regression	83.716	5	16.743	12.400	.000[d]
	Residual	151.229	112	1.350		
	Total	234.944	117			
4	Regression	95.468	12	7.956	5.989	.000[e]
	Residual	139.476	105	1.328		
	Total	234.944	117			

a. Dependent Variable: Concern_Tot

b. Predictors: (Constant), Ideology0

c. Predictors: (Constant), Ideology0, SES0, RaceCC

d. Predictors: (Constant), Ideology0, SES0, RaceCC, MGRS0, MRN0

e. Predictors: (Constant), Ideology0, SES0, RaceCC, MGRS0, MRN0, MRN0xSES0, MRN0xIdeology0, MGRS0xRace, MGRS0xIdeology0, MGRS0xSES0, MRN0xMGRS0, MRN0xRace

Coefficients[a]

Model		Unstandardized Coefficients B	Unstandardized Coefficients Std. Error	Standardized Coefficients Beta	t	Sig.
1	(Constant)	4.213	.110		38.384	.000
	Ideology0	-.518	.067	-.585	-7.765	.000
2	(Constant)	4.280	.126		34.051	.000
	Ideology0	-.489	.072	-.551	-6.772	.000
	RaceCC	-.149	.135	-.090	-1.105	.272
	SES0	-.028	.124	-.017	-.226	.822
3	(Constant)	4.341	.154		28.239	.000
	Ideology0	-.433	.089	-.488	-4.882	.000
	RaceCC	-.194	.141	-.117	-1.371	.173
	SES0	-.017	.125	-.010	-.135	.892
	MGRS0	-.001	.212	.000	-.005	.996
	MRN0	-.169	.166	-.104	-1.021	.309
4	(Constant)	4.071	.212		19.176	.000
	Ideology0	-.403	.097	-.455	-4.151	.000
	RaceCC	-.134	.181	-.081	-.739	.462
	SES0	.072	.183	.044	.391	.697
	MGRS0	-.206	.339	-.082	-.608	.545
	MRN0	.099	.236	.061	.418	.677
	MRN0xRace	-.221	.205	-.145	-1.080	.282
	MRN0xSES0	.106	.157	.071	.675	.501
	MRN0xIdeology0	.071	.079	.089	.906	.367
	MRN0xMGRS0	.403	.284	.183	1.422	.158
	MGRS0xRace	.049	.278	.020	.176	.861
	MGRS0xSES0	.161	.301	.061	.534	.594
	MGRS0xIdeology0	-.130	.168	-.081	-.773	.441

Coefficients[a]

Model		Correlations Zero-order	Partial	Part
1	(Constant)			
	Ideology0	-.585	-.585	-.585
2	(Constant)			
	Ideology0	-.585	-.536	-.512
	RaceCC	-.287	-.103	-.083
	SES0	-.045	-.021	-.017
3	(Constant)			
	Ideology0	-.585	-.419	-.370
	RaceCC	-.287	-.128	-.104
	SES0	-.045	-.013	-.010
	MGRS0	-.095	.000	.000
	MRN0	-.353	-.096	-.077
4	(Constant)			
	Ideology0	-.585	-.375	-.312
	RaceCC	-.287	-.072	-.056
	SES0	-.045	.038	.029
	MGRS0	-.095	-.059	-.046
	MRN0	-.353	.041	.031
	MRN0xRace	-.453	-.105	-.081
	MRN0xSES0	.259	.066	.051
	MRN0xIdeology0	-.098	.088	.068
	MRN0xMGRS0	.364	.137	.107
	MGRS0xRace	-.070	.017	.013
	MGRS0xSES0	.205	.052	.040
	MGRS0xIdeology0	.251	-.075	-.058

a. Dependent Variable: Concern_Tot

Excluded Variables[a]

Model		Beta In	t	Sig.	Partial Correlation	Collinearity Statistics Tolerance
1	RaceCC	-.088[b]	-1.090	.278	-.101	.870
	SES0	-.008[b]	-.100	.921	-.009	.996
	MGRS0	-.029[b]	-.386	.700	-.036	.987
	MRN0	-.069[b]	-.783	.435	-.073	.733
	MRN0xRace	-.175[b]	-1.922	.057	-.176	.670
	MRN0xSES0	.140[b]	1.833	.069	.168	.954
	MRN0xIdeology0	.085[b]	1.081	.282	.100	.910
	MRN0xMGRS0	.157[b]	1.931	.056	.177	.843
	MGRS0xRace	-.052[b]	-.688	.493	-.064	.999
	MGRS0xSES0	.091[b]	1.189	.237	.110	.960
	MGRS0xIdeology0	.038[b]	.467	.641	.044	.861
2	MGRS0	-.036[c]	-.475	.636	-.045	.980
	MRN0	-.104[c]	-1.131	.260	-.106	.668
	MRN0xRace	-.162[c]	-1.736	.085	-.161	.647
	MRN0xSES0	.158[c]	1.996	.048	.185	.888
	MRN0xIdeology0	.111[c]	1.336	.184	.125	.828
	MRN0xMGRS0	.172[c]	2.021	.046	.187	.765
	MGRS0xRace	-.088[c]	-1.098	.275	-.103	.881
	MGRS0xSES0	.145[c]	1.566	.120	.146	.661
	MGRS0xIdeology0	.046[c]	.549	.584	.052	.817
3	MRN0xRace	-.146[d]	-1.383	.169	-.130	.510
	MRN0xSES0	.152[d]	1.835	.069	.172	.818
	MRN0xIdeology0	.101[d]	1.193	.236	.112	.792
	MRN0xMGRS0	.235[d]	2.208	.029	.205	.489
	MGRS0xRace	-.103[d]	-1.165	.247	-.110	.732
	MGRS0xSES0	.127[d]	1.311	.193	.123	.610
	MGRS0xIdeology0	.018[d]	.200	.842	.019	.718

a. Dependent Variable: Concern_Tot

b. Predictors in the Model: (Constant), Ideology0

c. Predictors in the Model: (Constant), Ideology0, SES0, RaceCC

d. Predictors in the Model: (Constant), Ideology0, SES0, RaceCC, MGRS0, MRN0

```
REGRESSION
  /MISSING LISTWISE
  /STATISTICS COEFF OUTS R ANOVA CHANGE ZPP
  /CRITERIA=PIN(.05) POUT(.10)
  /NOORIGIN
  /DEPENDENT Finance_Tot
  /METHOD=ENTER Ideology0
  /METHOD=ENTER RaceCC SES0
  /METHOD=ENTER MGRS0 MRN0
  /METHOD=ENTER MRN0xRace MRN0xSES0 MRN0xIdeology0 MRN0xMGRS0 MGRS0xRace MGRS0xSES0 MGRS0
xIdeology0.
```

Regression

Notes

Output Created		15-DEC-2021 13:07:59
Comments		
Input	Data	C:\Users\njs5478\Dropbox\HM and COVID\0. Revise and Resubmit\2. R and R Data\Study 1b\Study1b_Data.sav
	Active Dataset	DataSet1
	Filter	GenderCC=1 (FILTER)
	Weight	<none>
	Split File	<none>
	N of Rows in Working Data File	119
Missing Value Handling	Definition of Missing	User-defined missing values are treated as missing.
	Cases Used	Statistics are based on cases with no missing values for any variable used.

Notes

Syntax		REGRESSION<br>/MISSING LISTWISE<br>/STATISTICS COEFF OUTS R ANOVA CHANGE ZPP<br>/CRITERIA=PIN(.05) POUT(.10)<br>/NOORIGIN<br>/DEPENDENT Finance_Tot<br>/METHOD=ENTER Ideology0<br>/METHOD=ENTER RaceCC SES0<br>/METHOD=ENTER MGRS0 MRN0<br>/METHOD=ENTER MRN0xRace MRN0xSES0 MRN0xIdeology0 MRN0xMGRS0 MGRS0xRace MGRS0xSES0 MGRS0xIdeology0.
Resources	Processor Time	00:00:00.00
	Elapsed Time	00:00:00.02
	Memory Required	48560 bytes
	Additional Memory Required for Residual Plots	0 bytes

Variables Entered/Removed[a]

Model	Variables Entered	Variables Removed	Method
1	Ideology0[b]	.	Enter
2	SES0, RaceCC[b]	.	Enter
3	MGRS0, MRN0[b]	.	Enter
4	MRN0xSES0, MRN0xIdeology0, MGRS0xRace, MGRS0xIdeology0, MGRS0xSES0, MRN0xMGRS0, MRN0xRace[b]	.	Enter

a. Dependent Variable: Finance_Tot

b. All requested variables entered.

Model Summary

Model	R	R Square	Adjusted R Square	Std. Error of the Estimate	Change Statistics		
					R Square Change	F Change	df1
1	.129[a]	.017	.008	1.48329	.017	1.969	1
2	.179[b]	.032	.007	1.48440	.016	.913	2
3	.180[c]	.032	-.011	1.49743	.000	.012	2
4	.321[d]	.103	.001	1.48893	.071	1.183	7

Model Summary

Model	Change Statistics	
	df2	Sig. F Change
1	116	.163
2	114	.404
3	112	.988
4	105	.319

a. Predictors: (Constant), Ideology0

b. Predictors: (Constant), Ideology0, SES0, RaceCC

c. Predictors: (Constant), Ideology0, SES0, RaceCC, MGRS0, MRN0

d. Predictors: (Constant), Ideology0, SES0, RaceCC, MGRS0, MRN0, MRN0xSES0, MRN0xIdeology0, MGRS0xRace, MGRS0xIdeology0, MGRS0xSES0, MRN0xMGRS0, MRN0xRace

ANOVA[a]

Model		Sum of Squares	df	Mean Square	F	Sig.
1	Regression	4.333	1	4.333	1.969	.163[b]
	Residual	255.219	116	2.200		
	Total	259.552	117			
2	Regression	8.358	3	2.786	1.264	.290[c]
	Residual	251.194	114	2.203		
	Total	259.552	117			
3	Regression	8.413	5	1.683	.750	.587[d]
	Residual	251.139	112	2.242		
	Total	259.552	117			
4	Regression	26.775	12	2.231	1.006	.448[e]
	Residual	232.777	105	2.217		
	Total	259.552	117			

a. Dependent Variable: Finance_Tot

b. Predictors: (Constant), Ideology0

c. Predictors: (Constant), Ideology0, SES0, RaceCC

d. Predictors: (Constant), Ideology0, SES0, RaceCC, MGRS0, MRN0

e. Predictors: (Constant), Ideology0, SES0, RaceCC, MGRS0, MRN0, MRN0xSES0, MRN0xIdeology0, MGRS0xRace, MGRS0xIdeology0, MGRS0xSES0, MRN0xMGRS0, MRN0xRace

Coefficients[a]

Model		Unstandardized Coefficients B	Std. Error	Standardized Coefficients Beta	t	Sig.
1	(Constant)	3.722	.141		26.389	.000
	Ideology0	-.120	.086	-.129	-1.403	.163
2	(Constant)	3.750	.161		23.276	.000
	Ideology0	-.102	.092	-.110	-1.107	.271
	RaceCC	-.055	.173	-.031	-.316	.752
	SES0	-.213	.159	-.125	-1.342	.182
3	(Constant)	3.734	.198		18.849	.000
	Ideology0	-.107	.114	-.115	-.935	.352
	RaceCC	-.052	.182	-.030	-.286	.775
	SES0	-.214	.161	-.125	-1.331	.186
	MGRS0	-.042	.273	-.016	-.154	.878
	MRN0	.020	.214	.012	.093	.926
4	(Constant)	3.610	.274		13.164	.000
	Ideology0	-.139	.126	-.149	-1.107	.271
	RaceCC	.087	.234	.050	.370	.712
	SES0	-.311	.237	-.182	-1.314	.192
	MGRS0	.031	.438	.012	.071	.944
	MRN0	.189	.305	.110	.619	.537
	MRN0xRace	-.362	.265	-.226	-1.370	.174
	MRN0xSES0	.281	.202	.178	1.387	.168
	MRN0xIdeology0	.150	.102	.179	1.480	.142
	MRN0xMGRS0	-.193	.366	-.083	-.526	.600
	MGRS0xRace	.414	.359	.164	1.154	.251
	MGRS0xSES0	.096	.389	.035	.246	.806
	MGRS0xIdeology0	-.175	.217	-.104	-.808	.421

Coefficients[a]

Model		Correlations Zero-order	Partial	Part
1	(Constant)			
	Ideology0	-.129	-.129	-.129
2	(Constant)			
	Ideology0	-.129	-.103	-.102
	RaceCC	-.061	-.030	-.029
	SES0	-.129	-.125	-.124
3	(Constant)			
	Ideology0	-.129	-.088	-.087
	RaceCC	-.061	-.027	-.027
	SES0	-.129	-.125	-.124
	MGRS0	-.027	-.015	-.014
	MRN0	-.069	.009	.009
4	(Constant)			
	Ideology0	-.129	-.107	-.102
	RaceCC	-.061	.036	.034
	SES0	-.129	-.127	-.121
	MGRS0	-.027	.007	.007
	MRN0	-.069	.060	.057
	MRN0xRace	-.116	-.132	-.127
	MRN0xSES0	.173	.134	.128
	MRN0xIdeology0	.091	.143	.137
	MRN0xMGRS0	.072	-.051	-.049
	MGRS0xRace	.033	.112	.107
	MGRS0xSES0	.168	.024	.023
	MGRS0xIdeology0	.071	-.079	-.075

a. Dependent Variable: Finance_Tot

Excluded Variables[a]

Model		Beta In	t	Sig.	Partial Correlation	Collinearity Statistics Tolerance
1	RaceCC	-.016[b]	-.163	.871	-.015	.870
	SES0	-.121[b]	-1.319	.190	-.122	.996
	MGRS0	-.013[b]	-.135	.893	-.013	.987
	MRN0	-.003[b]	-.025	.980	-.002	.733
	MRN0xRace	-.062[b]	-.548	.585	-.051	.670
	MRN0xSES0	.152[b]	1.622	.108	.150	.954
	MRN0xIdeology0	.143[b]	1.486	.140	.137	.910
	MRN0xMGRS0	.024[b]	.243	.809	.023	.843
	MGRS0xRace	.037[b]	.402	.688	.038	.999
	MGRS0xSES0	.148[b]	1.588	.115	.147	.960
	MGRS0xIdeology0	.026[b]	.261	.795	.024	.861
2	MGRS0	-.012[c]	-.128	.899	-.012	.980
	MRN0	.004[c]	.031	.975	.003	.668
	MRN0xRace	-.061[c]	-.533	.595	-.050	.647
	MRN0xSES0	.199[c]	2.064	.041	.191	.888
	MRN0xIdeology0	.132[c]	1.303	.195	.122	.828
	MRN0xMGRS0	-.016[c]	-.156	.877	-.015	.765
	MGRS0xRace	.048[c]	.490	.625	.046	.881
	MGRS0xSES0	.125[c]	1.106	.271	.103	.661
	MGRS0xIdeology0	.001[c]	.009	.993	.001	.817
3	MRN0xRace	-.083[d]	-.635	.527	-.060	.510
	MRN0xSES0	.213[d]	2.108	.037	.196	.818
	MRN0xIdeology0	.135[d]	1.301	.196	.123	.792
	MRN0xMGRS0	-.010[d]	-.077	.938	-.007	.489
	MGRS0xRace	.066[d]	.602	.548	.057	.732
	MGRS0xSES0	.135[d]	1.135	.259	.107	.610
	MGRS0xIdeology0	.005[d]	.041	.967	.004	.718

a. Dependent Variable: Finance_Tot

b. Predictors in the Model: (Constant), Ideology0

c. Predictors in the Model: (Constant), Ideology0, SES0, RaceCC

d. Predictors in the Model: (Constant), Ideology0, SES0, RaceCC, MGRS0, MRN0

```
REGRESSION
  /MISSING LISTWISE
  /STATISTICS COEFF OUTS R ANOVA CHANGE ZPP
  /CRITERIA=PIN(.05) POUT(.10)
  /NOORIGIN
  /DEPENDENT Resource_Tot
  /METHOD=ENTER Ideology0
  /METHOD=ENTER RaceCC SES0
  /METHOD=ENTER MGRS0 MRN0
  /METHOD=ENTER MRN0xRace MRN0xSES0 MRN0xIdeology0 MRN0xMGRS0 MGRS0xRace MGRS0xSES0 MGRS0
xIdeology0.
```

Regression

Notes

Output Created		15-DEC-2021 13:07:59
Comments		
Input	Data	C:\Users\njs5478\Dropbox\HM and COVID\0. Revise and Resubmit\2. R and R Data\Study 1b\Study1b_Data.sav
	Active Dataset	DataSet1
	Filter	GenderCC=1 (FILTER)
	Weight	<none>
	Split File	<none>
	N of Rows in Working Data File	119
Missing Value Handling	Definition of Missing	User-defined missing values are treated as missing.
	Cases Used	Statistics are based on cases with no missing values for any variable used.

Notes

Syntax		REGRESSION<br>/MISSING LISTWISE<br>/STATISTICS COEFF OUTS R ANOVA CHANGE ZPP<br>/CRITERIA=PIN(.05) POUT(.10)<br>/NOORIGIN<br>/DEPENDENT Resource_Tot<br>/METHOD=ENTER Ideology0<br>/METHOD=ENTER RaceCC SES0<br>/METHOD=ENTER MGRS0 MRN0<br>/METHOD=ENTER MRN0xRace MRN0xSES0 MRN0xIdeology0 MRN0xMGRS0 MGRS0xRace MGRS0xSES0 MGRS0xIdeology0.
Resources	Processor Time	00:00:00.02
	Elapsed Time	00:00:00.02
	Memory Required	48560 bytes
	Additional Memory Required for Residual Plots	0 bytes

Variables Entered/Removed[a]

Model	Variables Entered	Variables Removed	Method
1	Ideology0[b]	.	Enter
2	SES0, RaceCC[b]	.	Enter
3	MGRS0, MRN0[b]	.	Enter
4	MRN0xSES0, MRN0xIdeology0, MGRS0xRace, MGRS0xIdeology0, MGRS0xSES0, MRN0xMGRS0, MRN0xRace[b]	.	Enter

a. Dependent Variable: Resource_Tot

b. All requested variables entered.

Model Summary

					Change Statistics		
Model	R	R Square	Adjusted R Square	Std. Error of the Estimate	R Square Change	F Change	df1
1	.171[a]	.029	.021	1.40106	.029	3.480	1
2	.189[b]	.036	.011	1.40837	.007	.400	2
3	.229[c]	.052	.010	1.40863	.017	.979	2
4	.343[d]	.117	.016	1.40415	.065	1.102	7

Model Summary

	Change Statistics	
Model	df2	Sig. F Change
1	116	.065
2	114	.671
3	112	.379
4	105	.367

a. Predictors: (Constant), Ideology0

b. Predictors: (Constant), Ideology0, SES0, RaceCC

c. Predictors: (Constant), Ideology0, SES0, RaceCC, MGRS0, MRN0

d. Predictors: (Constant), Ideology0, SES0, RaceCC, MGRS0, MRN0, MRN0xSES0, MRN0xIdeology0, MGRS0xRace, MGRS0xIdeology0, MGRS0xSES0, MRN0xMGRS0, MRN0xRace

ANOVA[a]

Model		Sum of Squares	df	Mean Square	F	Sig.
1	Regression	6.831	1	6.831	3.480	.065[b]
	Residual	227.704	116	1.963		
	Total	234.535	117			
2	Regression	8.417	3	2.806	1.414	.242[c]
	Residual	226.118	114	1.983		
	Total	234.535	117			
3	Regression	12.301	5	2.460	1.240	.295[d]
	Residual	222.234	112	1.984		
	Total	234.535	117			
4	Regression	27.513	12	2.293	1.163	.319[e]
	Residual	207.022	105	1.972		
	Total	234.535	117			

a. Dependent Variable: Resource_Tot

b. Predictors: (Constant), Ideology0

c. Predictors: (Constant), Ideology0, SES0, RaceCC

d. Predictors: (Constant), Ideology0, SES0, RaceCC, MGRS0, MRN0

e. Predictors: (Constant), Ideology0, SES0, RaceCC, MGRS0, MRN0, MRN0xSES0, MRN0xIdeology0, MGRS0xRace, MGRS0xIdeology0, MGRS0xSES0, MRN0xMGRS0, MRN0xRace

Coefficients[a]

Model		Unstandardized Coefficients B	Unstandardized Coefficients Std. Error	Standardized Coefficients Beta	t	Sig.
1	(Constant)	3.537	.133		26.549	.000
	Ideology0	-.151	.081	-.171	-1.865	.065
2	(Constant)	3.566	.153		23.333	.000
	Ideology0	-.135	.088	-.152	-1.536	.127
	RaceCC	-.062	.164	-.037	-.375	.708
	SES0	-.128	.151	-.079	-.849	.397
3	(Constant)	3.446	.186		18.491	.000
	Ideology0	-.222	.107	-.250	-2.061	.042
	RaceCC	.005	.171	.003	.031	.975
	SES0	-.145	.151	-.089	-.960	.339
	MGRS0	-.100	.257	-.040	-.389	.698
	MRN0	.278	.201	.171	1.384	.169
4	(Constant)	3.267	.259		12.632	.000
	Ideology0	-.232	.118	-.262	-1.962	.052
	RaceCC	.050	.220	.030	.228	.820
	SES0	-.024	.223	-.015	-.106	.916
	MGRS0	-.019	.413	-.008	-.046	.963
	MRN0	.525	.288	.323	1.826	.071
	MRN0xRace	-.429	.250	-.282	-1.719	.089
	MRN0xSES0	-.034	.191	-.023	-.177	.860
	MRN0xIdeology0	.193	.096	.242	2.014	.047
	MRN0xMGRS0	-.112	.346	-.051	-.323	.747
	MGRS0xRace	-.005	.339	-.002	-.015	.988
	MGRS0xSES0	.235	.367	.090	.640	.524
	MGRS0xIdeology0	.010	.205	.006	.048	.962

Coefficients[a]

Model		Correlations Zero-order	Partial	Part
1	(Constant)			
	Ideology0	-.171	-.171	-.171
2	(Constant)			
	Ideology0	-.171	-.142	-.141
	RaceCC	-.085	-.035	-.035
	SES0	-.085	-.079	-.078
3	(Constant)			
	Ideology0	-.171	-.191	-.190
	RaceCC	-.085	.003	.003
	SES0	-.085	-.090	-.088
	MGRS0	.001	-.037	-.036
	MRN0	.014	.130	.127
4	(Constant)			
	Ideology0	-.171	-.188	-.180
	RaceCC	-.085	.022	.021
	SES0	-.085	-.010	-.010
	MGRS0	.001	-.005	-.004
	MRN0	.014	.175	.167
	MRN0xRace	-.156	-.165	-.158
	MRN0xSES0	.071	-.017	-.016
	MRN0xIdeology0	.093	.193	.185
	MRN0xMGRS0	.093	-.032	-.030
	MGRS0xRace	-.092	-.001	-.001
	MGRS0xSES0	.097	.062	.059
	MGRS0xIdeology0	.085	.005	.004

a. Dependent Variable: Resource_Tot

Excluded Variables[a]

Model		Beta In	t	Sig.	Partial Correlation	Collinearity Statistics Tolerance
1	RaceCC	-.028[b]	-.280	.780	-.026	.870
	SES0	-.075[b]	-.815	.417	-.076	.996
	MGRS0	.020[b]	.217	.828	.020	.987
	MRN0	.140[b]	1.310	.193	.121	.733
	MRN0xRace	-.086[b]	-.768	.444	-.071	.670
	MRN0xSES0	.036[b]	.379	.706	.035	.954
	MRN0xIdeology0	.158[b]	1.658	.100	.153	.910
	MRN0xMGRS0	.030[b]	.304	.762	.028	.843
	MGRS0xRace	-.087[b]	-.948	.345	-.088	.999
	MGRS0xSES0	.065[b]	.698	.487	.065	.960
	MGRS0xIdeology0	.024[b]	.246	.806	.023	.861
2	MGRS0	.019[c]	.207	.836	.019	.980
	MRN0	.151[c]	1.349	.180	.126	.668
	MRN0xRace	-.084[c]	-.729	.468	-.068	.647
	MRN0xSES0	.062[c]	.629	.531	.059	.888
	MRN0xIdeology0	.162[c]	1.610	.110	.150	.828
	MRN0xMGRS0	.007[c]	.069	.945	.006	.765
	MGRS0xRace	-.101[c]	-1.028	.306	-.096	.881
	MGRS0xSES0	.043[c]	.378	.706	.036	.661
	MGRS0xIdeology0	.011[c]	.106	.916	.010	.817
3	MRN0xRace	-.199[d]	-1.554	.123	-.146	.510
	MRN0xSES0	.086[d]	.848	.398	.080	.818
	MRN0xIdeology0	.183[d]	1.789	.076	.167	.792
	MRN0xMGRS0	.097[d]	.736	.463	.070	.489
	MGRS0xRace	-.107[d]	-.993	.323	-.094	.732
	MGRS0xSES0	.085[d]	.723	.471	.068	.610
	MGRS0xIdeology0	.068[d]	.627	.532	.059	.718

a. Dependent Variable: Resource_Tot

b. Predictors in the Model: (Constant), Ideology0

c. Predictors in the Model: (Constant), Ideology0, SES0, RaceCC

d. Predictors in the Model: (Constant), Ideology0, SES0, RaceCC, MGRS0, MRN0

```
REGRESSION
  /MISSING LISTWISE
  /STATISTICS COEFF OUTS R ANOVA CHANGE ZPP
  /CRITERIA=PIN(.05) POUT(.10)
  /NOORIGIN
  /DEPENDENT Psychology_Tot
  /METHOD=ENTER Ideology0
  /METHOD=ENTER RaceCC SES0
  /METHOD=ENTER MGRS0 MRN0
  /METHOD=ENTER MRN0xRace MRN0xSES0 MRN0xIdeology0 MRN0xMGRS0 MGRS0xRace MGRS0xSES0 MGRS0
xIdeology0.
```

Regression

Notes

Output Created		15-DEC-2021 13:07:59
Comments		
Input	Data	C: \Users\njs5478\Dropbox\HM and COVID\0. Revise and Resubmit\2. R and R Data\Study 1b\Study1b_Data.sav
	Active Dataset	DataSet1
	Filter	GenderCC=1 (FILTER)
	Weight	<none>
	Split File	<none>
	N of Rows in Working Data File	119
Missing Value Handling	Definition of Missing	User-defined missing values are treated as missing.
	Cases Used	Statistics are based on cases with no missing values for any variable used.

Notes

Syntax		REGRESSION<br>/MISSING LISTWISE<br>/STATISTICS COEFF OUTS R ANOVA CHANGE ZPP<br>/CRITERIA=PIN(.05) POUT(.10)<br>/NOORIGIN<br>/DEPENDENT Psychology_Tot<br>/METHOD=ENTER Ideology0<br>/METHOD=ENTER RaceCC SES0<br>/METHOD=ENTER MGRS0 MRN0<br>/METHOD=ENTER MRN0xRace MRN0xSES0 MRN0xIdeology0 MRN0xMGRS0 MGRS0xRace MGRS0xSES0 MGRS0xIdeology0.
Resources	Processor Time	00:00:00.02
	Elapsed Time	00:00:00.02
	Memory Required	48560 bytes
	Additional Memory Required for Residual Plots	0 bytes

Variables Entered/Removed[a]

Model	Variables Entered	Variables Removed	Method
1	Ideology0[b]	.	Enter
2	SES0, RaceCC[b]	.	Enter
3	MGRS0, MRN0[b]	.	Enter
4	MRN0xSES0, MRN0xIdeology0, MGRS0xRace, MGRS0xIdeology0, MGRS0xSES0, MRN0xMGRS0, MRN0xRace[b]	.	Enter

a. Dependent Variable: Psychology_Tot

b. All requested variables entered.

Model Summary

Model	R	R Square	Adjusted R Square	Std. Error of the Estimate	Change Statistics		
					R Square Change	F Change	df1
1	.302[a]	.091	.084	1.44295	.091	11.682	1
2	.308[b]	.095	.071	1.45263	.004	.230	2
3	.311[c]	.097	.057	1.46400	.002	.118	2
4	.396[d]	.157	.061	1.46091	.060	1.068	7

Model Summary

Model	Change Statistics	
	df2	Sig. F Change
1	116	.001
2	114	.795
3	112	.889
4	105	.389

a. Predictors: (Constant), Ideology0

b. Predictors: (Constant), Ideology0, SES0, RaceCC

c. Predictors: (Constant), Ideology0, SES0, RaceCC, MGRS0, MRN0

d. Predictors: (Constant), Ideology0, SES0, RaceCC, MGRS0, MRN0, MRN0xSES0, MRN0xIdeology0, MGRS0xRace, MGRS0xIdeology0, MGRS0xSES0, MRN0xMGRS0, MRN0xRace

ANOVA[a]

Model		Sum of Squares	df	Mean Square	F	Sig.
1	Regression	24.322	1	24.322	11.682	.001[b]
	Residual	241.523	116	2.082		
	Total	265.846	117			
2	Regression	25.292	3	8.431	3.995	.010[c]
	Residual	240.554	114	2.110		
	Total	265.846	117			
3	Regression	25.795	5	5.159	2.407	.041[d]
	Residual	240.050	112	2.143		
	Total	265.846	117			
4	Regression	41.749	12	3.479	1.630	.094[e]
	Residual	224.097	105	2.134		
	Total	265.846	117			

a. Dependent Variable: Psychology_Tot

b. Predictors: (Constant), Ideology0

c. Predictors: (Constant), Ideology0, SES0, RaceCC

d. Predictors: (Constant), Ideology0, SES0, RaceCC, MGRS0, MRN0

e. Predictors: (Constant), Ideology0, SES0, RaceCC, MGRS0, MRN0, MRN0xSES0, MRN0xIdeology0, MGRS0xRace, MGRS0xIdeology0, MGRS0xSES0, MRN0xMGRS0, MRN0xRace

Coefficients[a]

Model		Unstandardized Coefficients B	Std. Error	Standardized Coefficients Beta	t	Sig.
1	(Constant)	4.507	.137		32.851	.000
	Ideology0	-.285	.083	-.302	-3.418	.001
2	(Constant)	4.551	.158		28.865	.000
	Ideology0	-.268	.091	-.284	-2.957	.004
	RaceCC	-.099	.170	-.056	-.584	.560
	SES0	.043	.155	.025	.275	.784
3	(Constant)	4.589	.194		23.696	.000
	Ideology0	-.237	.112	-.251	-2.118	.036
	RaceCC	-.123	.178	-.070	-.693	.490
	SES0	.049	.157	.028	.311	.757
	MGRS0	.018	.267	.007	.067	.947
	MRN0	-.097	.209	-.056	-.464	.643
4	(Constant)	4.377	.269		16.266	.000
	Ideology0	-.238	.123	-.252	-1.930	.056
	RaceCC	-.214	.229	-.121	-.932	.353
	SES0	.393	.233	.227	1.688	.094
	MGRS0	-.153	.430	-.057	-.357	.722
	MRN0	.112	.299	.065	.375	.708
	MRN0xRace	.021	.260	.013	.079	.937
	MRN0xSES0	-.281	.198	-.176	-1.414	.160
	MRN0xIdeology0	.103	.100	.122	1.037	.302
	MRN0xMGRS0	.333	.360	.142	.926	.357
	MGRS0xRace	-.203	.352	-.079	-.575	.566
	MGRS0xSES0	.370	.381	.133	.969	.335
	MGRS0xIdeology0	.205	.213	.120	.962	.338

Coefficients[a]

Model		Correlations Zero-order	Partial	Part
1	(Constant)			
	Ideology0	-.302	-.302	-.302
2	(Constant)			
	Ideology0	-.302	-.267	-.263
	RaceCC	-.160	-.055	-.052
	SES0	.011	.026	.024
3	(Constant)			
	Ideology0	-.302	-.196	-.190
	RaceCC	-.160	-.065	-.062
	SES0	.011	.029	.028
	MGRS0	-.041	.006	.006
	MRN0	-.176	-.044	-.042
4	(Constant)			
	Ideology0	-.302	-.185	-.173
	RaceCC	-.160	-.091	-.084
	SES0	.011	.163	.151
	MGRS0	-.041	-.035	-.032
	MRN0	-.176	.037	.034
	MRN0xRace	-.192	.008	.007
	MRN0xSES0	.048	-.137	-.127
	MRN0xIdeology0	.029	.101	.093
	MRN0xMGRS0	.231	.090	.083
	MGRS0xRace	-.043	-.056	-.052
	MGRS0xSES0	.087	.094	.087
	MGRS0xIdeology0	.230	.093	.086

a. Dependent Variable: Psychology_Tot

Excluded Variables[a]

Model		Beta In	t	Sig.	Partial Correlation	Collinearity Statistics Tolerance
1	RaceCC	-.059[b]	-.622	.535	-.058	.870
	SES0	.031[b]	.345	.731	.032	.996
	MGRS0	-.007[b]	-.083	.934	-.008	.987
	MRN0	-.027[b]	-.263	.793	-.025	.733
	MRN0xRace	-.028[b]	-.254	.800	-.024	.670
	MRN0xSES0	-.017[b]	-.192	.848	-.018	.954
	MRN0xIdeology0	.132[b]	1.424	.157	.132	.910
	MRN0xMGRS0	.132[b]	1.370	.173	.127	.843
	MGRS0xRace	-.034[b]	-.384	.702	-.036	.999
	MGRS0xSES0	.027[b]	.297	.767	.028	.960
	MGRS0xIdeology0	.136[b]	1.432	.155	.132	.861
2	MGRS0	-.013[c]	-.140	.889	-.013	.980
	MRN0	-.053[c]	-.482	.631	-.045	.668
	MRN0xRace	-.015[c]	-.136	.892	-.013	.647
	MRN0xSES0	-.024[c]	-.251	.802	-.024	.888
	MRN0xIdeology0	.164[c]	1.692	.093	.157	.828
	MRN0xMGRS0	.158[c]	1.559	.122	.145	.765
	MGRS0xRace	-.062[c]	-.650	.517	-.061	.881
	MGRS0xSES0	.075[c]	.682	.497	.064	.661
	MGRS0xIdeology0	.156[c]	1.589	.115	.148	.817
3	MRN0xRace	.012[d]	.093	.926	.009	.510
	MRN0xSES0	-.034[d]	-.343	.732	-.033	.818
	MRN0xIdeology0	.166[d]	1.655	.101	.155	.792
	MRN0xMGRS0	.223[d]	1.756	.082	.164	.489
	MGRS0xRace	-.076[d]	-.724	.471	-.069	.732
	MGRS0xSES0	.067[d]	.579	.563	.055	.610
	MGRS0xIdeology0	.158[d]	1.504	.135	.141	.718

a. Dependent Variable: Psychology_Tot

b. Predictors in the Model: (Constant), Ideology0

c. Predictors in the Model: (Constant), Ideology0, SES0, RaceCC

d. Predictors in the Model: (Constant), Ideology0, SES0, RaceCC, MGRS0, MRN0

```
REGRESSION
  /MISSING LISTWISE
  /STATISTICS COEFF OUTS R ANOVA CHANGE ZPP
  /CRITERIA=PIN(.05) POUT(.10)
  /NOORIGIN
  /DEPENDENT TrumpApproval
  /METHOD=ENTER Ideology0
  /METHOD=ENTER RaceCC SES0
  /METHOD=ENTER MGRS0 MRN0
  /METHOD=ENTER MRN0xRace MRN0xSES0 MRN0xIdeology0 MRN0xMGRS0 MGRS0xRace MGRS0xSES0 MGRS0
xIdeology0.
```

Regression

Notes

Output Created		15-DEC-2021 13:07:59
Comments		
Input	Data	C:\Users\njs5478\Dropbox\HM and COVID\0. Revise and Resubmit\2. R and R Data\Study 1b\Study1b_Data.sav
	Active Dataset	DataSet1
	Filter	GenderCC=1 (FILTER)
	Weight	<none>
	Split File	<none>
	N of Rows in Working Data File	119
Missing Value Handling	Definition of Missing	User-defined missing values are treated as missing.
	Cases Used	Statistics are based on cases with no missing values for any variable used.

Notes

Syntax		REGRESSION<br>/MISSING LISTWISE<br>/STATISTICS COEFF OUTS R ANOVA CHANGE ZPP<br>/CRITERIA=PIN(.05) POUT(.10)<br>/NOORIGIN<br>/DEPENDENT TrumpApproval<br>/METHOD=ENTER Ideology0<br>/METHOD=ENTER RaceCC SES0<br>/METHOD=ENTER MGRS0 MRN0<br>/METHOD=ENTER MRN0xRace MRN0xSES0 MRN0xIdeology0 MRN0xMGRS0 MGRS0xRace MGRS0xSES0 MGRS0xIdeology0.
Resources	Processor Time	00:00:00.02
	Elapsed Time	00:00:00.02
	Memory Required	48560 bytes
	Additional Memory Required for Residual Plots	0 bytes

Variables Entered/Removed[a]

Model	Variables Entered	Variables Removed	Method
1	Ideology0[b]	.	Enter
2	SES0, RaceCC[b]	.	Enter
3	MGRS0, MRN0[b]	.	Enter
4	MRN0xSES0, MRN0xIdeology0, MGRS0xRace, MGRS0xIdeology0, MGRS0xSES0, MRN0xMGRS0, MRN0xRace[b]	.	Enter

a. Dependent Variable: Do you approve or disapprove of the way Donald Trump is handling his job as President?

b. All requested variables entered.

Model Summary

Model	R	R Square	Adjusted R Square	Std. Error of the Estimate	Change Statistics		
					R Square Change	F Change	df1
1	.767[a]	.589	.585	1.420	.589	166.200	1
2	.781[b]	.610	.600	1.395	.021	3.069	2
3	.798[c]	.637	.620	1.359	.027	4.103	2
4	.807[d]	.651	.611	1.376	.014	.615	7

Model Summary

Model	Change Statistics	
	df2	Sig. F Change
1	116	.000
2	114	.050
3	112	.019
4	105	.743

a. Predictors: (Constant), Ideology0

b. Predictors: (Constant), Ideology0, SES0, RaceCC

c. Predictors: (Constant), Ideology0, SES0, RaceCC, MGRS0, MRN0

d. Predictors: (Constant), Ideology0, SES0, RaceCC, MGRS0, MRN0, MRN0xSES0, MRN0xIdeology0, MGRS0xRace, MGRS0xIdeology0, MGRS0xSES0, MRN0xMGRS0, MRN0xRace

ANOVA[a]

Model		Sum of Squares	df	Mean Square	F	Sig.
1	Regression	335.159	1	335.159	166.200	.000[b]
	Residual	233.925	116	2.017		
	Total	569.085	117			
2	Regression	347.112	3	115.704	59.423	.000[c]
	Residual	221.973	114	1.947		
	Total	569.085	117			
3	Regression	362.266	5	72.453	39.236	.000[d]
	Residual	206.819	112	1.847		
	Total	569.085	117			
4	Regression	370.409	12	30.867	16.313	.000[e]
	Residual	198.676	105	1.892		
	Total	569.085	117			

a. Dependent Variable: Do you approve or disapprove of the way Donald Trump is handling his job as President?

b. Predictors: (Constant), Ideology0

c. Predictors: (Constant), Ideology0, SES0, RaceCC

d. Predictors: (Constant), Ideology0, SES0, RaceCC, MGRS0, MRN0

e. Predictors: (Constant), Ideology0, SES0, RaceCC, MGRS0, MRN0, MRN0xSES0, MRN0xIdeology0, MGRS0xRace, MGRS0xIdeology0, MGRS0xSES0, MRN0xMGRS0, MRN0xRace

Coefficients[a]

Model		Unstandardized Coefficients B	Std. Error	Standardized Coefficients Beta	t	Sig.
1	(Constant)	3.124	.135		23.133	.000
	Ideology0	1.059	.082	.767	12.892	.000
2	(Constant)	3.007	.151		19.854	.000
	Ideology0	1.015	.087	.736	11.676	.000
	RaceCC	.271	.163	.105	1.663	.099
	SES0	-.244	.149	-.096	-1.633	.105
3	(Constant)	2.887	.180		16.058	.000
	Ideology0	.863	.104	.626	8.325	.000
	RaceCC	.395	.165	.153	2.387	.019
	SES0	-.274	.146	-.108	-1.879	.063
	MGRS0	.169	.248	.043	.684	.495
	MRN0	.435	.194	.172	2.242	.027
4	(Constant)	2.915	.253		11.504	.000
	Ideology0	.831	.116	.602	7.166	.000
	RaceCC	.365	.216	.142	1.691	.094
	SES0	-.221	.219	-.087	-1.008	.316
	MGRS0	.346	.405	.089	.855	.394
	MRN0	.461	.282	.182	1.635	.105
	MRN0xRace	-.039	.244	-.017	-.161	.873
	MRN0xSES0	-.041	.187	-.018	-.219	.827
	MRN0xIdeology0	.097	.094	.079	1.038	.302
	MRN0xMGRS0	-.345	.339	-.100	-1.018	.311
	MGRS0xRace	.037	.332	.010	.113	.910
	MGRS0xSES0	.099	.359	.024	.276	.783
	MGRS0xIdeology0	.250	.200	.100	1.245	.216

Coefficients[a]

Model		Correlations Zero-order	Partial	Part
1	(Constant)			
	Ideology0	.767	.767	.767
2	(Constant)			
	Ideology0	.767	.738	.683
	RaceCC	.378	.154	.097
	SES0	-.058	-.151	-.096
3	(Constant)			
	Ideology0	.767	.618	.474
	RaceCC	.378	.220	.136
	SES0	-.058	-.175	-.107
	MGRS0	.176	.065	.039
	MRN0	.493	.207	.128
4	(Constant)			
	Ideology0	.767	.573	.413
	RaceCC	.378	.163	.098
	SES0	-.058	-.098	-.058
	MGRS0	.176	.083	.049
	MRN0	.493	.158	.094
	MRN0xRace	.529	-.016	-.009
	MRN0xSES0	-.213	-.021	-.013
	MRN0xIdeology0	.313	.101	.060
	MRN0xMGRS0	-.300	-.099	-.059
	MGRS0xRace	.035	.011	.007
	MGRS0xSES0	-.111	.027	.016
	MGRS0xIdeology0	-.226	.121	.072

a. Dependent Variable: Do you approve or disapprove of the way Donald Trump is handling his job as President?

Excluded Variables[a]

Model		Beta In	t	Sig.	Partial Correlation	Collinearity Statistics Tolerance
1	RaceCC	.117[b]	1.850	.067	.170	.870
	SES0	-.108[b]	-1.823	.071	-.168	.996
	MGRS0	.091[b]	1.526	.130	.141	.987
	MRN0	.131[b]	1.913	.058	.176	.733
	MRN0xRace	.132[b]	1.841	.068	.169	.670
	MRN0xSES0	-.050[b]	-.824	.412	-.077	.954
	MRN0xIdeology0	.091[b]	1.469	.144	.136	.910
	MRN0xMGRS0	.004[b]	.066	.948	.006	.843
	MGRS0xRace	.012[b]	.197	.844	.018	.999
	MGRS0xSES0	.045[b]	.733	.465	.068	.960
	MGRS0xIdeology0	.070[b]	1.089	.278	.101	.861
2	MGRS0	.103[c]	1.753	.082	.163	.980
	MRN0	.194[c]	2.788	.006	.254	.668
	MRN0xRace	.111[c]	1.531	.129	.143	.647
	MRN0xSES0	-.030[c]	-.488	.626	-.046	.888
	MRN0xIdeology0	.050[c]	.778	.438	.073	.828
	MRN0xMGRS0	-.037[c]	-.558	.578	-.052	.765
	MGRS0xRace	.064[c]	1.021	.310	.096	.881
	MGRS0xSES0	-.042[c]	-.582	.562	-.055	.661
	MGRS0xIdeology0	.039[c]	.595	.553	.056	.817
3	MRN0xRace	.040[d]	.496	.621	.047	.510
	MRN0xSES0	.012[d]	.185	.854	.018	.818
	MRN0xIdeology0	.085[d]	1.336	.184	.126	.792
	MRN0xMGRS0	-.033[d]	-.405	.686	-.038	.489
	MGRS0xRace	.050[d]	.755	.452	.071	.732
	MGRS0xSES0	.015[d]	.205	.838	.019	.610
	MGRS0xIdeology0	.102[d]	1.520	.131	.143	.718

a. Dependent Variable: Do you approve or disapprove of the way Donald Trump is handling his job as President?

b. Predictors in the Model: (Constant), Ideology0

c. Predictors in the Model: (Constant), Ideology0, SES0, RaceCC

d. Predictors in the Model: (Constant), Ideology0, SES0, RaceCC, MGRS0, MRN0

```
REGRESSION
  /MISSING LISTWISE
  /STATISTICS COEFF OUTS R ANOVA CHANGE ZPP
  /CRITERIA=PIN(.05) POUT(.10)
  /NOORIGIN
  /DEPENDENT TrumpX
  /METHOD=ENTER Ideology0
  /METHOD=ENTER RaceCC SES0
  /METHOD=ENTER MGRS0 MRN0
  /METHOD=ENTER MRN0xRace MRN0xSES0 MRN0xIdeology0 MRN0xMGRS0 MGRS0xRace MGRS0xSES0 MGRS0
xIdeology0.
```

Regression

Notes

Output Created		15-DEC-2021 13:07:59
Comments		
Input	Data	C:\Users\njs5478\Dropbox\HM and COVID\0. Revise and Resubmit\2. R and R Data\Study 1b\Study1b_Data.sav
	Active Dataset	DataSet1
	Filter	GenderCC=1 (FILTER)
	Weight	<none>
	Split File	<none>
	N of Rows in Working Data File	119
Missing Value Handling	Definition of Missing	User-defined missing values are treated as missing.
	Cases Used	Statistics are based on cases with no missing values for any variable used.

Notes

Syntax		REGRESSION<br>/MISSING LISTWISE<br>/STATISTICS COEFF OUTS R ANOVA CHANGE ZPP<br>/CRITERIA=PIN(.05) POUT(.10)<br>/NOORIGIN<br>/DEPENDENT TrumpX<br>/METHOD=ENTER Ideology0<br>/METHOD=ENTER RaceCC SES0<br>/METHOD=ENTER MGRS0 MRN0<br>/METHOD=ENTER MRN0xRace MRN0xSES0 MRN0xIdeology0 MRN0xMGRS0 MGRS0xRace MGRS0xSES0 MGRS0xIdeology0.
Resources	Processor Time	00:00:00.00
	Elapsed Time	00:00:00.02
	Memory Required	48560 bytes
	Additional Memory Required for Residual Plots	0 bytes

Variables Entered/Removed[a]

Model	Variables Entered	Variables Removed	Method
1	Ideology0[b]	.	Enter
2	SES0, RaceCC[b]	.	Enter
3	MGRS0, MRN0[b]	.	Enter
4	MRN0xSES0, MRN0xIdeology0, MGRS0xRace, MGRS0xIdeology0, MGRS0xSES0, MRN0xMGRS0, MRN0xRace[b]	.	Enter

a. Dependent Variable: TrumpX

b. All requested variables entered.

Model Summary

Model	R	R Square	Adjusted R Square	Std. Error of the Estimate	Change Statistics		
					R Square Change	F Change	df1
1	.746[a]	.556	.553	1.43077	.556	145.551	1
2	.753[b]	.567	.556	1.42532	.011	1.445	2
3	.782[c]	.612	.594	1.36272	.044	6.357	2
4	.799[d]	.638	.597	1.35851	.027	1.099	7

Model Summary

Model	Change Statistics	
	df2	Sig. F Change
1	116	.000
2	114	.240
3	112	.002
4	105	.369

a. Predictors: (Constant), Ideology0

b. Predictors: (Constant), Ideology0, SES0, RaceCC

c. Predictors: (Constant), Ideology0, SES0, RaceCC, MGRS0, MRN0

d. Predictors: (Constant), Ideology0, SES0, RaceCC, MGRS0, MRN0, MRN0xSES0, MRN0xIdeology0, MGRS0xRace, MGRS0xIdeology0, MGRS0xSES0, MRN0xMGRS0, MRN0xRace

ANOVA[a]

Model		Sum of Squares	df	Mean Square	F	Sig.
1	Regression	297.959	1	297.959	145.551	.000[b]
	Residual	237.465	116	2.047		
	Total	535.424	117			
2	Regression	303.829	3	101.276	49.852	.000[c]
	Residual	231.594	114	2.032		
	Total	535.424	117			
3	Regression	327.439	5	65.488	35.265	.000[d]
	Residual	207.985	112	1.857		
	Total	535.424	117			
4	Regression	341.640	12	28.470	15.426	.000[e]
	Residual	193.784	105	1.846		
	Total	535.424	117			

a. Dependent Variable: TrumpX

b. Predictors: (Constant), Ideology0

c. Predictors: (Constant), Ideology0, SES0, RaceCC

d. Predictors: (Constant), Ideology0, SES0, RaceCC, MGRS0, MRN0

e. Predictors: (Constant), Ideology0, SES0, RaceCC, MGRS0, MRN0, MRN0xSES0, MRN0xIdeology0, MGRS0xRace, MGRS0xIdeology0, MGRS0xSES0, MRN0xMGRS0, MRN0xRace

Coefficients[a]

Model		Unstandardized Coefficients B	Std. Error	Standardized Coefficients Beta	t	Sig.
1	(Constant)	3.064	.136		22.520	.000
	Ideology0	.998	.083	.746	12.064	.000
2	(Constant)	2.972	.155		19.210	.000
	Ideology0	.963	.089	.719	10.839	.000
	RaceCC	.212	.166	.085	1.275	.205
	SES0	-.148	.152	-.060	-.971	.334
3	(Constant)	2.806	.180		15.567	.000
	Ideology0	.769	.104	.574	7.392	.000
	RaceCC	.369	.166	.148	2.228	.028
	SES0	-.186	.146	-.076	-1.276	.205
	MGRS0	.171	.248	.045	.689	.492
	MRN0	.563	.195	.229	2.891	.005
4	(Constant)	2.864	.250		11.447	.000
	Ideology0	.762	.115	.569	6.650	.000
	RaceCC	.303	.213	.121	1.420	.158
	SES0	-.144	.216	-.059	-.665	.507
	MGRS0	.480	.400	.127	1.201	.232
	MRN0	.598	.278	.243	2.148	.034
	MRN0xRace	-.046	.241	-.020	-.189	.851
	MRN0xSES0	.020	.185	.009	.109	.913
	MRN0xIdeology0	.091	.093	.076	.981	.329
	MRN0xMGRS0	-.476	.334	-.143	-1.424	.157
	MGRS0xRace	-.112	.328	-.031	-.342	.733
	MGRS0xSES0	.096	.355	.024	.270	.788
	MGRS0xIdeology0	.410	.198	.169	2.073	.041

Coefficients[a]

Model		Correlations Zero-order	Partial	Part
1	(Constant)			
	Ideology0	.746	.746	.746
2	(Constant)			
	Ideology0	.746	.712	.668
	RaceCC	.349	.119	.079
	SES0	-.021	-.091	-.060
3	(Constant)			
	Ideology0	.746	.573	.435
	RaceCC	.349	.206	.131
	SES0	-.021	-.120	-.075
	MGRS0	.197	.065	.041
	MRN0	.528	.263	.170
4	(Constant)			
	Ideology0	.746	.544	.390
	RaceCC	.349	.137	.083
	SES0	-.021	-.065	-.039
	MGRS0	.197	.116	.071
	MRN0	.528	.205	.126
	MRN0xRace	.519	-.018	-.011
	MRN0xSES0	-.183	.011	.006
	MRN0xIdeology0	.301	.095	.058
	MRN0xMGRS0	-.307	-.138	-.084
	MGRS0xRace	.033	-.033	-.020
	MGRS0xSES0	-.131	.026	.016
	MGRS0xIdeology0	-.202	.198	.122

a. Dependent Variable: TrumpX

Excluded Variables[a]

Model		Beta In	t	Sig.	Partial Correlation	Collinearity Statistics Tolerance
1	RaceCC	.092[b]	1.396	.165	.129	.870
	SES0	-.069[b]	-1.122	.264	-.104	.996
	MGRS0	.115[b]	1.866	.065	.171	.987
	MRN0	.195[b]	2.784	.006	.251	.733
	MRN0xRace	.135[b]	1.808	.073	.166	.670
	MRN0xSES0	-.024[b]	-.383	.702	-.036	.954
	MRN0xIdeology0	.085[b]	1.308	.193	.121	.910
	MRN0xMGRS0	-.014[b]	-.211	.833	-.020	.843
	MGRS0xRace	.011[b]	.171	.864	.016	.999
	MGRS0xSES0	.020[b]	.309	.758	.029	.960
	MGRS0xIdeology0	.089[b]	1.333	.185	.123	.861
2	MGRS0	.124[c]	2.023	.045	.187	.980
	MRN0	.252[c]	3.507	.001	.313	.668
	MRN0xRace	.119[c]	1.566	.120	.146	.647
	MRN0xSES0	-.012[c]	-.182	.856	-.017	.888
	MRN0xIdeology0	.057[c]	.841	.402	.079	.828
	MRN0xMGRS0	-.043[c]	-.614	.540	-.058	.765
	MGRS0xRace	.050[c]	.763	.447	.072	.881
	MGRS0xSES0	-.044[c]	-.578	.564	-.054	.661
	MGRS0xIdeology0	.069[c]	1.017	.311	.095	.817
3	MRN0xRace	.018[d]	.219	.827	.021	.510
	MRN0xSES0	.043[d]	.655	.514	.062	.818
	MRN0xIdeology0	.101[d]	1.528	.129	.144	.792
	MRN0xMGRS0	-.025[d]	-.293	.770	-.028	.489
	MGRS0xRace	.032[d]	.459	.647	.044	.732
	MGRS0xSES0	.030[d]	.394	.694	.037	.610
	MGRS0xIdeology0	.155[d]	2.278	.025	.211	.718

a. Dependent Variable: TrumpX

b. Predictors in the Model: (Constant), Ideology0

c. Predictors in the Model: (Constant), Ideology0, SES0, RaceCC

d. Predictors in the Model: (Constant), Ideology0, SES0, RaceCC, MGRS0, MRN0

```
REGRESSION
  /MISSING LISTWISE
  /STATISTICS COEFF OUTS R ANOVA CHANGE ZPP
  /CRITERIA=PIN(.05) POUT(.10)
  /NOORIGIN
  /DEPENDENT BidenX
  /METHOD=ENTER Ideology0
  /METHOD=ENTER RaceCC SES0
  /METHOD=ENTER MGRS0 MRN0
  /METHOD=ENTER MRN0xRace MRN0xSES0 MRN0xIdeology0 MRN0xMGRS0 MGRS0xRace MGRS0xSES0 MGRS0
xIdeology0.
```

Regression

Notes

Output Created		15-DEC-2021 13:07:59
Comments		
Input	Data	C:\Users\njs5478\Dropbox\HM and COVID\0. Revise and Resubmit\2. R and R Data\Study 1b\Study1b_Data.sav
	Active Dataset	DataSet1
	Filter	GenderCC=1 (FILTER)
	Weight	<none>
	Split File	<none>
	N of Rows in Working Data File	119
Missing Value Handling	Definition of Missing	User-defined missing values are treated as missing.
	Cases Used	Statistics are based on cases with no missing values for any variable used.

Notes

Syntax		REGRESSION<br>/MISSING LISTWISE<br>/STATISTICS COEFF OUTS R ANOVA CHANGE ZPP<br>/CRITERIA=PIN(.05) POUT(.10)<br>/NOORIGIN<br>/DEPENDENT BidenX<br>/METHOD=ENTER Ideology0<br>/METHOD=ENTER RaceCC SES0<br>/METHOD=ENTER MGRS0 MRN0<br>/METHOD=ENTER MRN0xRace MRN0xSES0 MRN0xIdeology0 MRN0xMGRS0 MGRS0xRace MGRS0xSES0 MGRS0xIdeology0.
Resources	Processor Time	00:00:00.02
	Elapsed Time	00:00:00.02
	Memory Required	48560 bytes
	Additional Memory Required for Residual Plots	0 bytes

Variables Entered/Removed[a]

Model	Variables Entered	Variables Removed	Method
1	Ideology0[b]	.	Enter
2	SES0, RaceCC[b]	.	Enter
3	MGRS0, MRN0[b]	.	Enter
4	MRN0xSES0, MRN0xIdeology0, MGRS0xRace, MGRS0xIdeology0, MGRS0xSES0, MRN0xMGRS0, MRN0xRace[b]	.	Enter

a. Dependent Variable: BidenX

b. All requested variables entered.

Model Summary

Model	R	R Square	Adjusted R Square	Std. Error of the Estimate	Change Statistics		
					R Square Change	F Change	df1
1	.667[a]	.445	.440	1.40935	.445	91.306	1
2	.670[b]	.449	.435	1.41596	.005	.469	2
3	.686[c]	.471	.447	1.40065	.021	2.231	2
4	.701[d]	.492	.433	1.41804	.021	.617	7

Model Summary

Model	Change Statistics	
	df2	Sig. F Change
1	114	.000
2	112	.627
3	110	.112
4	103	.741

a. Predictors: (Constant), Ideology0

b. Predictors: (Constant), Ideology0, SES0, RaceCC

c. Predictors: (Constant), Ideology0, SES0, RaceCC, MGRS0, MRN0

d. Predictors: (Constant), Ideology0, SES0, RaceCC, MGRS0, MRN0, MRN0xSES0, MRN0xIdeology0, MGRS0xRace, MGRS0xIdeology0, MGRS0xSES0, MRN0xMGRS0, MRN0xRace

ANOVA[a]

Model		Sum of Squares	df	Mean Square	F	Sig.
1	Regression	181.359	1	181.359	91.306	.000[b]
	Residual	226.434	114	1.986		
	Total	407.793	115			
2	Regression	183.239	3	61.080	30.465	.000[c]
	Residual	224.554	112	2.005		
	Total	407.793	115			
3	Regression	191.993	5	38.399	19.573	.000[d]
	Residual	215.801	110	1.962		
	Total	407.793	115			
4	Regression	200.676	12	16.723	8.316	.000[e]
	Residual	207.117	103	2.011		
	Total	407.793	115			

a. Dependent Variable: BidenX

b. Predictors: (Constant), Ideology0

c. Predictors: (Constant), Ideology0, SES0, RaceCC

d. Predictors: (Constant), Ideology0, SES0, RaceCC, MGRS0, MRN0

e. Predictors: (Constant), Ideology0, SES0, RaceCC, MGRS0, MRN0, MRN0xSES0, MRN0xIdeology0, MGRS0xRace, MGRS0xIdeology0, MGRS0xSES0, MRN0xMGRS0, MRN0xRace

Coefficients[a]

Model		Unstandardized Coefficients B	Std. Error	Standardized Coefficients Beta	t	Sig.
1	(Constant)	4.458	.135		33.005	.000
	Ideology0	-.779	.082	-.667	-9.555	.000
2	(Constant)	4.476	.156		28.740	.000
	Ideology0	-.776	.088	-.665	-8.786	.000
	RaceCC	-.038	.168	-.017	-.227	.821
	SES0	.141	.153	.065	.917	.361
3	(Constant)	4.593	.186		24.728	.000
	Ideology0	-.654	.107	-.560	-6.117	.000
	RaceCC	-.137	.173	-.062	-.792	.430
	SES0	.166	.152	.076	1.091	.278
	MGRS0	-.063	.259	-.019	-.244	.808
	MRN0	-.361	.201	-.168	-1.799	.075
4	(Constant)	4.567	.262		17.434	.000
	Ideology0	-.716	.120	-.613	-5.987	.000
	RaceCC	-.153	.223	-.069	-.684	.495
	SES0	.229	.226	.105	1.010	.315
	MGRS0	-.117	.428	-.035	-.273	.786
	MRN0	-.438	.294	-.204	-1.488	.140
	MRN0xRace	.068	.260	.034	.261	.795
	MRN0xSES0	-.045	.195	-.023	-.232	.817
	MRN0xIdeology0	.068	.097	.064	.694	.489
	MRN0xMGRS0	.208	.350	.071	.595	.553
	MGRS0xRace	.129	.356	.040	.361	.719
	MGRS0xSES0	.189	.382	.053	.494	.622
	MGRS0xIdeology0	-.388	.207	-.183	-1.872	.064

Coefficients[a]

Model		Correlations Zero-order	Partial	Part
1	(Constant)			
	Ideology0	-.667	-.667	-.667
2	(Constant)			
	Ideology0	-.667	-.639	-.616
	RaceCC	-.264	-.021	-.016
	SES0	.023	.086	.064
3	(Constant)			
	Ideology0	-.667	-.504	-.424
	RaceCC	-.264	-.075	-.055
	SES0	.023	.103	.076
	MGRS0	-.147	-.023	-.017
	MRN0	-.455	-.169	-.125
4	(Constant)			
	Ideology0	-.667	-.508	-.420
	RaceCC	-.264	-.067	-.048
	SES0	.023	.099	.071
	MGRS0	-.147	-.027	-.019
	MRN0	-.455	-.145	-.104
	MRN0xRace	-.408	.026	.018
	MRN0xSES0	.184	-.023	-.016
	MRN0xIdeology0	-.177	.068	.049
	MRN0xMGRS0	.271	.058	.042
	MGRS0xRace	-.015	.036	.025
	MGRS0xSES0	.164	.049	.035
	MGRS0xIdeology0	.181	-.181	-.131

a. Dependent Variable: BidenX

Excluded Variables[a]

Model		Beta In	t	Sig.	Partial Correlation	Collinearity Statistics Tolerance
1	RaceCC	-.023[b]	-.312	.755	-.029	.866
	SES0	.066[b]	.945	.346	.089	.996
	MGRS0	-.072[b]	-1.024	.308	-.096	.987
	MRN0	-.150[b]	-1.856	.066	-.172	.733
	MRN0xRace	-.035[b]	-.410	.683	-.039	.667
	MRN0xSES0	.041[b]	.573	.568	.054	.953
	MRN0xIdeology0	.025[b]	.345	.731	.032	.910
	MRN0xMGRS0	.006[b]	.082	.935	.008	.841
	MGRS0xRace	.006[b]	.086	.932	.008	.999
	MGRS0xSES0	.027[b]	.382	.703	.036	.957
	MGRS0xIdeology0	-.078[b]	-1.042	.300	-.098	.861
2	MGRS0	-.078[c]	-1.096	.275	-.103	.975
	MRN0	-.178[c]	-2.107	.037	-.196	.670
	MRN0xRace	-.032[c]	-.368	.714	-.035	.648
	MRN0xSES0	.028[c]	.374	.709	.036	.893
	MRN0xIdeology0	.049[c]	.630	.530	.060	.827
	MRN0xMGRS0	.030[c]	.370	.712	.035	.771
	MGRS0xRace	-.006[c]	-.087	.931	-.008	.897
	MGRS0xSES0	.090[c]	1.065	.289	.101	.684
	MGRS0xIdeology0	-.065[c]	-.843	.401	-.080	.818
3	MRN0xRace	.056[d]	.574	.567	.055	.507
	MRN0xSES0	-.006[d]	-.082	.935	-.008	.825
	MRN0xIdeology0	.024[d]	.305	.761	.029	.791
	MRN0xMGRS0	.004[d]	.039	.969	.004	.497
	MGRS0xRace	.006[d]	.079	.937	.008	.736
	MGRS0xSES0	.044[d]	.505	.615	.048	.623
	MGRS0xIdeology0	-.130[d]	-1.607	.111	-.152	.719

a. Dependent Variable: BidenX

b. Predictors in the Model: (Constant), Ideology0

c. Predictors in the Model: (Constant), Ideology0, SES0, RaceCC

d. Predictors in the Model: (Constant), Ideology0, SES0, RaceCC, MGRS0, MRN0

```
REGRESSION
  /MISSING LISTWISE
  /STATISTICS COEFF OUTS R ANOVA CHANGE ZPP
  /CRITERIA=PIN(.05) POUT(.10)
  /NOORIGIN
  /DEPENDENT Pelosi
  /METHOD=ENTER Ideology0
  /METHOD=ENTER RaceCC SES0
  /METHOD=ENTER MGRS0 MRN0
  /METHOD=ENTER MRN0xRace MRN0xSES0 MRN0xIdeology0 MRN0xMGRS0 MGRS0xRace MGRS0xSES0 MGRS0
xIdeology0.
```

Regression

Notes

Output Created		15-DEC-2021 13:07:59
Comments		
Input	Data	C:\Users\njs5478\Dropbox\HM and COVID\0. Revise and Resubmit\2. R and R Data\Study 1b\Study1b_Data.sav
	Active Dataset	DataSet1
	Filter	GenderCC=1 (FILTER)
	Weight	<none>
	Split File	<none>
	N of Rows in Working Data File	119
Missing Value Handling	Definition of Missing	User-defined missing values are treated as missing.
	Cases Used	Statistics are based on cases with no missing values for any variable used.

Notes

Syntax		REGRESSION<br>/MISSING LISTWISE<br>/STATISTICS COEFF OUTS R ANOVA CHANGE ZPP<br>/CRITERIA=PIN(.05) POUT(.10)<br>/NOORIGIN<br>/DEPENDENT Pelosi<br>/METHOD=ENTER Ideology0<br>/METHOD=ENTER RaceCC SES0<br>/METHOD=ENTER MGRS0 MRN0<br>/METHOD=ENTER MRN0xRace MRN0xSES0 MRN0xIdeology0 MRN0xMGRS0 MGRS0xRace MGRS0xSES0 MGRS0xIdeology0.
Resources	Processor Time	00:00:00.02
	Elapsed Time	00:00:00.02
	Memory Required	48560 bytes
	Additional Memory Required for Residual Plots	0 bytes

Variables Entered/Removed[a]

Model	Variables Entered	Variables Removed	Method
1	Ideology0[b]	.	Enter
2	SES0, RaceCC[b]	.	Enter
3	MGRS0, MRN0[b]	.	Enter
4	MRN0xSES0, MRN0xIdeology0, MGRS0xRace, MGRS0xIdeology0, MGRS0xSES0, MRN0xMGRS0, MRN0xRace[b]	.	Enter

a. Dependent Variable: Using the scale provided, please indicate your personal opinion regarding how each of the following is handling the response to COVID-19 (Coronavirus) in the United States: - Nancy Pelosi

b. All requested variables entered.

Model Summary

Model	R	R Square	Adjusted R Square	Std. Error of the Estimate	Change Statistics		
					R Square Change	F Change	df1
1	.476[a]	.226	.220	1.978	.226	33.914	1
2	.491[b]	.241	.222	1.976	.015	1.147	2
3	.511[c]	.262	.229	1.967	.020	1.524	2
4	.538[d]	.289	.208	1.993	.028	.587	7

Model Summary

Model	Change Statistics df2	Sig. F Change
1	116	.000
2	114	.321
3	112	.222
4	105	.765

a. Predictors: (Constant), Ideology0

b. Predictors: (Constant), Ideology0, SES0, RaceCC

c. Predictors: (Constant), Ideology0, SES0, RaceCC, MGRS0, MRN0

d. Predictors: (Constant), Ideology0, SES0, RaceCC, MGRS0, MRN0, MRN0xSES0, MRN0xIdeology0, MGRS0xRace, MGRS0xIdeology0, MGRS0xSES0, MRN0xMGRS0, MRN0xRace

ANOVA[a]

Model		Sum of Squares	df	Mean Square	F	Sig.
1	Regression	132.753	1	132.753	33.914	.000[b]
	Residual	454.069	116	3.914		
	Total	586.822	117			
2	Regression	141.710	3	47.237	12.098	.000[c]
	Residual	445.112	114	3.904		
	Total	586.822	117			
3	Regression	153.499	5	30.700	7.935	.000[d]
	Residual	433.323	112	3.869		
	Total	586.822	117			
4	Regression	169.825	12	14.152	3.563	.000[e]
	Residual	416.997	105	3.971		
	Total	586.822	117			

a. Dependent Variable: Using the scale provided, please indicate your personal opinion regarding how each of the following is handling the response to COVID-19 (Coronavirus) in the United States: - Nancy Pelosi

b. Predictors: (Constant), Ideology0

c. Predictors: (Constant), Ideology0, SES0, RaceCC

d. Predictors: (Constant), Ideology0, SES0, RaceCC, MGRS0, MRN0

e. Predictors: (Constant), Ideology0, SES0, RaceCC, MGRS0, MRN0, MRN0xSES0, MRN0xIdeology0, MGRS0xRace, MGRS0xIdeology0, MGRS0xSES0, MRN0xMGRS0, MRN0xRace

Coefficients[a]

Model		Unstandardized Coefficients B	Unstandardized Coefficients Std. Error	Standardized Coefficients Beta	t	Sig.
1	(Constant)	4.045	.188		21.504	.000
	Ideology0	-.666	.114	-.476	-5.824	.000
2	(Constant)	4.059	.214		18.929	.000
	Ideology0	-.651	.123	-.465	-5.291	.000
	RaceCC	-.020	.231	-.007	-.085	.932
	SES0	-.319	.211	-.124	-1.512	.133
3	(Constant)	4.152	.260		15.955	.000
	Ideology0	-.522	.150	-.372	-3.475	.001
	RaceCC	-.126	.239	-.048	-.527	.599
	SES0	-.293	.211	-.114	-1.391	.167
	MGRS0	-.184	.359	-.046	-.513	.609
	MRN0	-.366	.281	-.142	-1.302	.196
4	(Constant)	4.210	.367		11.469	.000
	Ideology0	-.522	.168	-.373	-3.109	.002
	RaceCC	-.039	.313	-.015	-.124	.901
	SES0	-.672	.317	-.262	-2.120	.036
	MGRS0	-.218	.586	-.055	-.371	.711
	MRN0	-.533	.408	-.207	-1.306	.194
	MRN0xRace	.008	.354	.004	.024	.981
	MRN0xSES0	.300	.271	.127	1.110	.270
	MRN0xIdeology0	-.070	.136	-.056	-.517	.606
	MRN0xMGRS0	.108	.491	.031	.221	.826
	MGRS0xRace	.118	.480	.031	.246	.806
	MGRS0xSES0	-.718	.520	-.173	-1.381	.170
	MGRS0xIdeology0	-.348	.290	-.137	-1.198	.234

Coefficients[a]

Model		Correlations Zero-order	Partial	Part
1	(Constant)			
	Ideology0	-.476	-.476	-.476
2	(Constant)			
	Ideology0	-.476	-.444	-.432
	RaceCC	-.164	-.008	-.007
	SES0	-.154	-.140	-.123
3	(Constant)			
	Ideology0	-.476	-.312	-.282
	RaceCC	-.164	-.050	-.043
	SES0	-.154	-.130	-.113
	MGRS0	-.150	-.048	-.042
	MRN0	-.366	-.122	-.106
4	(Constant)			
	Ideology0	-.476	-.290	-.256
	RaceCC	-.164	-.012	-.010
	SES0	-.154	-.203	-.174
	MGRS0	-.150	-.036	-.031
	MRN0	-.366	-.126	-.107
	MRN0xRace	-.331	.002	.002
	MRN0xSES0	.115	.108	.091
	MRN0xIdeology0	-.156	-.050	-.043
	MRN0xMGRS0	.209	.022	.018
	MGRS0xRace	-.051	.024	.020
	MGRS0xSES0	.126	-.134	-.114
	MGRS0xIdeology0	.146	-.116	-.099

a. Dependent Variable: Using the scale provided, please indicate your personal opinion regarding how each of the following is handling the response to COVID-19 (Coronavirus) in the United States: - Nancy Pelosi

Excluded Variables[a]

Model		Beta In	t	Sig.	Partial Correlation	Collinearity Statistics Tolerance
1	RaceCC	.008[b]	.089	.930	.008	.870
	SES0	-.124[b]	-1.519	.132	-.140	.996
	MGRS0	-.097[b]	-1.186	.238	-.110	.987
	MRN0	-.164[b]	-1.738	.085	-.160	.733
	MRN0xRace	-.086[b]	-.864	.390	-.080	.670
	MRN0xSES0	.014[b]	.165	.869	.015	.954
	MRN0xIdeology0	-.015[b]	-.177	.860	-.016	.910
	MRN0xMGRS0	.025[b]	.277	.782	.026	.843
	MGRS0xRace	-.036[b]	-.440	.661	-.041	.999
	MGRS0xSES0	.032[b]	.383	.702	.036	.960
	MGRS0xIdeology0	-.037[b]	-.413	.680	-.039	.861
2	MGRS0	-.095[c]	-1.159	.249	-.108	.980
	MRN0	-.166[c]	-1.674	.097	-.156	.668
	MRN0xRace	-.092[c]	-.906	.367	-.085	.647
	MRN0xSES0	.050[c]	.576	.566	.054	.888
	MRN0xIdeology0	-.048[c]	-.529	.598	-.050	.828
	MRN0xMGRS0	-.018[c]	-.188	.851	-.018	.765
	MGRS0xRace	-.026[c]	-.303	.762	-.028	.881
	MGRS0xSES0	-.050[c]	-.499	.619	-.047	.661
	MGRS0xIdeology0	-.068[c]	-.748	.456	-.070	.817
3	MRN0xRace	-.032[d]	-.284	.777	-.027	.510
	MRN0xSES0	.014[d]	.157	.876	.015	.818
	MRN0xIdeology0	-.079[d]	-.868	.388	-.082	.792
	MRN0xMGRS0	-.041[d]	-.352	.726	-.033	.489
	MGRS0xRace	-.005[d]	-.053	.958	-.005	.732
	MGRS0xSES0	-.107[d]	-1.032	.304	-.098	.610
	MGRS0xIdeology0	-.125[d]	-1.307	.194	-.123	.718

a. Dependent Variable: Using the scale provided, please indicate your personal opinion regarding how each of the following is handling the response to COVID-19 (Coronavirus) in the United States: - Nancy Pelosi

b. Predictors in the Model: (Constant), Ideology0

c. Predictors in the Model: (Constant), Ideology0, SES0, RaceCC

d. Predictors in the Model: (Constant), Ideology0, SES0, RaceCC, MGRS0, MRN0

```
REGRESSION
  /MISSING LISTWISE
  /STATISTICS COEFF OUTS R ANOVA CHANGE ZPP
  /CRITERIA=PIN(.05) POUT(.10)
  /NOORIGIN
  /DEPENDENT McConnellX
  /METHOD=ENTER Ideology0
  /METHOD=ENTER RaceCC SES0
  /METHOD=ENTER MGRS0 MRN0
  /METHOD=ENTER MRN0xRace MRN0xSES0 MRN0xIdeology0 MRN0xMGRS0 MGRS0xRace MGRS0xSES0 MGRS0
xIdeology0.
```

Regression

Notes

Output Created		15-DEC-2021 13:07:59
Comments		
Input	Data	C:\Users\njs5478\Dropbox\HM and COVID\0. Revise and Resubmit\2. R and R Data\Study 1b\Study1b_Data.sav
	Active Dataset	DataSet1
	Filter	GenderCC=1 (FILTER)
	Weight	<none>
	Split File	<none>
	N of Rows in Working Data File	119
Missing Value Handling	Definition of Missing	User-defined missing values are treated as missing.
	Cases Used	Statistics are based on cases with no missing values for any variable used.

Notes

Syntax		REGRESSION<br>/MISSING LISTWISE<br>/STATISTICS COEFF OUTS R ANOVA CHANGE ZPP<br>/CRITERIA=PIN(.05) POUT(.10)<br>/NOORIGIN<br>/DEPENDENT McConnellX<br>/METHOD=ENTER Ideology0<br>/METHOD=ENTER RaceCC SES0<br>/METHOD=ENTER MGRS0 MRN0<br>/METHOD=ENTER MRN0xRace MRN0xSES0 MRN0xIdeology0 MRN0xMGRS0 MGRS0xRace MGRS0xSES0 MGRS0xIdeology0.
Resources	Processor Time	00:00:00.02
	Elapsed Time	00:00:00.02
	Memory Required	48560 bytes
	Additional Memory Required for Residual Plots	0 bytes

Variables Entered/Removed[a]

Model	Variables Entered	Variables Removed	Method
1	Ideology0[b]	.	Enter
2	SES0, RaceCC[b]	.	Enter
3	MGRS0, MRN0[b]	.	Enter
4	MRN0xSES0, MRN0xIdeology0, MGRS0xRace, MGRS0xIdeology0, MGRS0xSES0, MRN0xMGRS0, MRN0xRace[b]	.	Enter

a. Dependent Variable: McConnellX

b. All requested variables entered.

Model Summary

Model	R	R Square	Adjusted R Square	Std. Error of the Estimate	Change Statistics: R Square Change	F Change	df1
1	.590[a]	.348	.342	1.35165	.348	52.393	1
2	.602[b]	.363	.343	1.35058	.014	1.077	2
3	.663[c]	.439	.409	1.28044	.076	6.403	2
4	.681[d]	.464	.390	1.30153	.025	.568	7

Model Summary

Model	Change Statistics: df2	Sig. F Change
1	98	.000
2	96	.345
3	94	.002
4	87	.780

a. Predictors: (Constant), Ideology0

b. Predictors: (Constant), Ideology0, SES0, RaceCC

c. Predictors: (Constant), Ideology0, SES0, RaceCC, MGRS0, MRN0

d. Predictors: (Constant), Ideology0, SES0, RaceCC, MGRS0, MRN0, MRN0xSES0, MRN0xIdeology0, MGRS0xRace, MGRS0xIdeology0, MGRS0xSES0, MRN0xMGRS0, MRN0xRace

ANOVA[a]

Model		Sum of Squares	df	Mean Square	F	Sig.
1	Regression	95.719	1	95.719	52.393	.000[b]
	Residual	179.041	98	1.827		
	Total	274.760	99			
2	Regression	99.649	3	33.216	18.210	.000[c]
	Residual	175.111	96	1.824		
	Total	274.760	99			
3	Regression	120.645	5	24.129	14.717	.000[d]
	Residual	154.115	94	1.640		
	Total	274.760	99			
4	Regression	127.383	12	10.615	6.266	.000[e]
	Residual	147.377	87	1.694		
	Total	274.760	99			

a. Dependent Variable: McConnellX

b. Predictors: (Constant), Ideology0

c. Predictors: (Constant), Ideology0, SES0, RaceCC

d. Predictors: (Constant), Ideology0, SES0, RaceCC, MGRS0, MRN0

e. Predictors: (Constant), Ideology0, SES0, RaceCC, MGRS0, MRN0, MRN0xSES0, MRN0xIdeology0, MGRS0xRace, MGRS0xIdeology0, MGRS0xSES0, MRN0xMGRS0, MRN0xRace

Coefficients[a]

Model		Unstandardized Coefficients B	Unstandardized Coefficients Std. Error	Standardized Coefficients Beta	t	Sig.
1	(Constant)	2.944	.139		21.170	.000
	Ideology0	.639	.088	.590	7.238	.000
2	(Constant)	2.812	.166		16.900	.000
	Ideology0	.598	.093	.553	6.416	.000
	RaceCC	.253	.175	.125	1.448	.151
	SES0	-.014	.158	-.007	-.089	.929
3	(Constant)	2.834	.187		15.139	.000
	Ideology0	.449	.109	.415	4.110	.000
	RaceCC	.360	.171	.177	2.100	.038
	SES0	-.042	.150	-.022	-.282	.779
	MGRS0	.572	.252	.195	2.273	.025
	MRN0	.326	.201	.169	1.616	.109
4	(Constant)	2.804	.269		10.419	.000
	Ideology0	.419	.124	.387	3.371	.001
	RaceCC	.252	.231	.124	1.092	.278
	SES0	.113	.221	.059	.512	.610
	MGRS0	.578	.424	.197	1.365	.176
	MRN0	.330	.319	.171	1.033	.304
	MRN0xRace	.191	.305	.104	.628	.532
	MRN0xSES0	-.204	.210	-.110	-.974	.333
	MRN0xIdeology0	.052	.101	.053	.513	.609
	MRN0xMGRS0	.018	.344	.007	.051	.959
	MGRS0xRace	-.190	.357	-.068	-.533	.595
	MGRS0xSES0	.134	.376	.044	.357	.722
	MGRS0xIdeology0	.249	.210	.129	1.184	.240

Coefficients[a]

Model		Correlations Zero-order	Partial	Part
1	(Constant)			
	Ideology0	.590	.590	.590
2	(Constant)			
	Ideology0	.590	.548	.523
	RaceCC	.293	.146	.118
	SES0	.040	-.009	-.007
3	(Constant)			
	Ideology0	.590	.390	.317
	RaceCC	.293	.212	.162
	SES0	.040	-.029	-.022
	MGRS0	.329	.228	.176
	MRN0	.469	.164	.125
4	(Constant)			
	Ideology0	.590	.340	.265
	RaceCC	.293	.116	.086
	SES0	.040	.055	.040
	MGRS0	.329	.145	.107
	MRN0	.469	.110	.081
	MRN0xRace	.502	.067	.049
	MRN0xSES0	-.215	-.104	-.076
	MRN0xIdeology0	.201	.055	.040
	MRN0xMGRS0	-.143	.005	.004
	MGRS0xRace	.102	-.057	-.042
	MGRS0xSES0	-.195	.038	.028
	MGRS0xIdeology0	-.145	.126	.093

a. Dependent Variable: McConnellX

Excluded Variables[a]

Model		Beta In	t	Sig.	Partial Correlation	Collinearity Statistics Tolerance
1	RaceCC	.125[b]	1.473	.144	.148	.908
	SES0	-.020[b]	-.237	.813	-.024	.990
	MGRS0	.239[b]	3.015	.003	.293	.974
	MRN0	.209[b]	2.182	.031	.216	.701
	MRN0xRace	.207[b]	1.968	.052	.196	.582
	MRN0xSES0	-.107[b]	-1.298	.197	-.131	.964
	MRN0xIdeology0	.053[b]	.630	.530	.064	.934
	MRN0xMGRS0	.090[b]	1.024	.309	.103	.861
	MGRS0xRace	.056[b]	.678	.500	.069	.994
	MGRS0xSES0	-.055[b]	-.649	.518	-.066	.941
	MGRS0xIdeology0	.093[b]	1.054	.295	.106	.855
2	MGRS0	.251[c]	3.166	.002	.309	.967
	MRN0	.265[c]	2.706	.008	.267	.652
	MRN0xRace	.190[c]	1.771	.080	.179	.566
	MRN0xSES0	-.124[c]	-1.469	.145	-.149	.920
	MRN0xIdeology0	.019[c]	.215	.830	.022	.829
	MRN0xMGRS0	.085[c]	.924	.358	.094	.790
	MGRS0xRace	.110[c]	1.264	.209	.129	.875
	MGRS0xSES0	-.111[c]	-1.128	.262	-.115	.682
	MGRS0xIdeology0	.083[c]	.903	.369	.092	.795
3	MRN0xRace	.092[d]	.743	.460	.077	.391
	MRN0xSES0	-.063[d]	-.754	.453	-.078	.866
	MRN0xIdeology0	.085[d]	.984	.328	.101	.791
	MRN0xMGRS0	.063[d]	.590	.557	.061	.530
	MGRS0xRace	-.016[d]	-.161	.872	-.017	.629
	MGRS0xSES0	-.025[d]	-.251	.802	-.026	.634
	MGRS0xIdeology0	.123[d]	1.331	.186	.137	.698

a. Dependent Variable: McConnellX

b. Predictors in the Model: (Constant), Ideology0

c. Predictors in the Model: (Constant), Ideology0, SES0, RaceCC

d. Predictors in the Model: (Constant), Ideology0, SES0, RaceCC, MGRS0, MRN0

```
REGRESSION
  /MISSING LISTWISE
  /STATISTICS COEFF OUTS R ANOVA CHANGE ZPP
  /CRITERIA=PIN(.05) POUT(.10)
  /NOORIGIN
  /DEPENDENT FauciX
  /METHOD=ENTER Ideology0
  /METHOD=ENTER RaceCC SES0
  /METHOD=ENTER MGRS0 MRN0
  /METHOD=ENTER MRN0xRace MRN0xSES0 MRN0xIdeology0 MRN0xMGRS0 MGRS0xRace MGRS0xSES0 MGRS0
xIdeology0.
```

Regression

Notes

Output Created		15-DEC-2021 13:07:59
Comments		
Input	Data	C:\Users\njs5478\Dropbox\HM and COVID\0. Revise and Resubmit\2. R and R Data\Study 1b\Study1b_Data.sav
	Active Dataset	DataSet1
	Filter	GenderCC=1 (FILTER)
	Weight	<none>
	Split File	<none>
	N of Rows in Working Data File	119
Missing Value Handling	Definition of Missing	User-defined missing values are treated as missing.
	Cases Used	Statistics are based on cases with no missing values for any variable used.

Notes

Syntax		REGRESSION<br>/MISSING LISTWISE<br>/STATISTICS COEFF OUTS R ANOVA CHANGE ZPP<br>/CRITERIA=PIN(.05) POUT(.10)<br>/NOORIGIN<br>/DEPENDENT FauciX<br>/METHOD=ENTER Ideology0<br>/METHOD=ENTER RaceCC SES0<br>/METHOD=ENTER MGRS0 MRN0<br>/METHOD=ENTER MRN0xRace MRN0xSES0 MRN0xIdeology0 MRN0xMGRS0 MGRS0xRace MGRS0xSES0 MGRS0xIdeology0.
Resources	Processor Time	00:00:00.02
	Elapsed Time	00:00:00.02
	Memory Required	48560 bytes
	Additional Memory Required for Residual Plots	0 bytes

Variables Entered/Removed[a]

Model	Variables Entered	Variables Removed	Method
1	Ideology0[b]	.	Enter
2	SES0, RaceCC[b]	.	Enter
3	MGRS0, MRN0[b]	.	Enter
4	MRN0xSES0, MGRS0xIdeology0, MGRS0xRace, MRN0xIdeology0, MGRS0xSES0, MRN0xMGRS0, MRN0xRace[b]	.	Enter

a. Dependent Variable: FauciX

b. All requested variables entered.

Model Summary

Model	R	R Square	Adjusted R Square	Std. Error of the Estimate	Change Statistics: R Square Change	F Change	df1
1	.354[a]	.125	.117	1.66326	.125	15.010	1
2	.366[b]	.134	.109	1.67081	.009	.527	2
3	.458[c]	.210	.171	1.61183	.076	4.838	2
4	.486[d]	.236	.138	1.64288	.026	.460	7

Model Summary

Model	Change Statistics: df2	Sig. F Change
1	105	.000
2	103	.592
3	101	.010
4	94	.861

a. Predictors: (Constant), Ideology0

b. Predictors: (Constant), Ideology0, SES0, RaceCC

c. Predictors: (Constant), Ideology0, SES0, RaceCC, MGRS0, MRN0

d. Predictors: (Constant), Ideology0, SES0, RaceCC, MGRS0, MRN0, MRN0xSES0, MGRS0xIdeology0, MGRS0xRace, MRN0xIdeology0, MGRS0xSES0, MRN0xMGRS0, MRN0xRace

ANOVA[a]

Model		Sum of Squares	df	Mean Square	F	Sig.
1	Regression	41.524	1	41.524	15.010	.000[b]
	Residual	290.476	105	2.766		
	Total	332.000	106			
2	Regression	44.465	3	14.822	5.309	.002[c]
	Residual	287.535	103	2.792		
	Total	332.000	106			
3	Regression	69.602	5	13.920	5.358	.000[d]
	Residual	262.398	101	2.598		
	Total	332.000	106			
4	Regression	78.290	12	6.524	2.417	.009[e]
	Residual	253.710	94	2.699		
	Total	332.000	106			

a. Dependent Variable: FauciX

b. Predictors: (Constant), Ideology0

c. Predictors: (Constant), Ideology0, SES0, RaceCC

d. Predictors: (Constant), Ideology0, SES0, RaceCC, MGRS0, MRN0

e. Predictors: (Constant), Ideology0, SES0, RaceCC, MGRS0, MRN0, MRN0xSES0, MGRS0xIdeology0, MGRS0xRace, MRN0xIdeology0, MGRS0xSES0, MRN0xMGRS0, MRN0xRace

Coefficients[a]

Model		Unstandardized Coefficients B	Unstandardized Coefficients Std. Error	Standardized Coefficients Beta	t	Sig.
1	(Constant)	5.196	.169		30.827	.000
	Ideology0	-.411	.106	-.354	-3.874	.000
2	(Constant)	5.114	.190		26.869	.000
	Ideology0	-.453	.115	-.389	-3.927	.000
	RaceCC	.176	.204	.086	.862	.390
	SES0	.126	.193	.060	.652	.516
3	(Constant)	5.256	.224		23.458	.000
	Ideology0	-.290	.132	-.250	-2.204	.030
	RaceCC	.033	.204	.016	.161	.872
	SES0	.145	.186	.070	.778	.438
	MGRS0	-.382	.301	-.123	-1.272	.206
	MRN0	-.512	.246	-.237	-2.080	.040
4	(Constant)	5.413	.322		16.800	.000
	Ideology0	-.285	.152	-.245	-1.879	.063
	RaceCC	-.034	.281	-.017	-.121	.904
	SES0	-.111	.275	-.053	-.402	.688
	MGRS0	-.008	.510	-.002	-.015	.988
	MRN0	-.752	.378	-.347	-1.987	.050
	MRN0xRace	.010	.341	.005	.029	.977
	MRN0xSES0	.216	.263	.106	.818	.415
	MRN0xIdeology0	.016	.122	.016	.134	.894
	MRN0xMGRS0	-.247	.415	-.089	-.596	.552
	MGRS0xRace	-.350	.408	-.116	-.859	.393
	MGRS0xSES0	-.596	.445	-.181	-1.339	.184
	MGRS0xIdeology0	-.048	.248	-.023	-.195	.846

Coefficients[a]

Model		Correlations Zero-order	Partial	Part
1	(Constant)			
	Ideology0	-.354	-.354	-.354
2	(Constant)			
	Ideology0	-.354	-.361	-.360
	RaceCC	-.062	.085	.079
	SES0	.025	.064	.060
3	(Constant)			
	Ideology0	-.354	-.214	-.195
	RaceCC	-.062	.016	.014
	SES0	.025	.077	.069
	MGRS0	-.223	-.126	-.113
	MRN0	-.393	-.203	-.184
4	(Constant)			
	Ideology0	-.354	-.190	-.169
	RaceCC	-.062	-.012	-.011
	SES0	.025	-.041	-.036
	MGRS0	-.223	-.002	-.001
	MRN0	-.393	-.201	-.179
	MRN0xRace	-.308	.003	.003
	MRN0xSES0	.117	.084	.074
	MRN0xIdeology0	-.095	.014	.012
	MRN0xMGRS0	.067	-.061	-.054
	MGRS0xRace	-.147	-.088	-.077
	MGRS0xSES0	.032	-.137	-.121
	MGRS0xIdeology0	.097	-.020	-.018

a. Dependent Variable: FauciX

Excluded Variables[a]

Model		Beta In	t	Sig.	Partial Correlation	Collinearity Statistics Tolerance
1	RaceCC	.078[b]	.795	.429	.078	.866
	SES0	.051[b]	.557	.579	.055	.995
	MGRS0	-.210[b]	-2.349	.021	-.224	.999
	MRN0	-.292[b]	-2.928	.004	-.276	.783
	MRN0xRace	-.152[b]	-1.350	.180	-.131	.650
	MRN0xSES0	.077[b]	.836	.405	.082	.987
	MRN0xIdeology0	.066[b]	.658	.512	.064	.820
	MRN0xMGRS0	-.064[b]	-.659	.512	-.064	.879
	MGRS0xRace	-.136[b]	-1.494	.138	-.145	.999
	MGRS0xSES0	-.033[b]	-.354	.724	-.035	.967
	MGRS0xIdeology0	.012[b]	.131	.896	.013	.941
2	MGRS0	-.205[c]	-2.276	.025	-.220	.993
	MRN0	-.295[c]	-2.830	.006	-.270	.724
	MRN0xRace	-.198[c]	-1.684	.095	-.164	.596
	MRN0xSES0	.062[c]	.641	.523	.063	.914
	MRN0xIdeology0	.062[c]	.594	.554	.059	.767
	MRN0xMGRS0	-.052[c]	-.511	.610	-.051	.805
	MGRS0xRace	-.133[c]	-1.390	.168	-.136	.915
	MGRS0xSES0	-.028[c]	-.254	.800	-.025	.706
	MGRS0xIdeology0	.018[c]	.183	.855	.018	.897
3	MRN0xRace	-.084[d]	-.629	.531	-.063	.443
	MRN0xSES0	.004[d]	.039	.969	.004	.849
	MRN0xIdeology0	.003[d]	.026	.979	.003	.738
	MRN0xMGRS0	-.045[d]	-.362	.718	-.036	.509
	MGRS0xRace	-.097[d]	-.936	.351	-.093	.737
	MGRS0xSES0	-.125[d]	-1.144	.255	-.114	.655
	MGRS0xIdeology0	-.049[d]	-.484	.629	-.048	.777

a. Dependent Variable: FauciX

b. Predictors in the Model: (Constant), Ideology0

c. Predictors in the Model: (Constant), Ideology0, SES0, RaceCC

d. Predictors in the Model: (Constant), Ideology0, SES0, RaceCC, MGRS0, MRN0

```
REGRESSION
  /MISSING LISTWISE
  /STATISTICS COEFF OUTS R ANOVA CHANGE ZPP
  /CRITERIA=PIN(.05) POUT(.10)
  /NOORIGIN
  /DEPENDENT RepCongressX
  /METHOD=ENTER Ideology0
  /METHOD=ENTER RaceCC SES0
  /METHOD=ENTER MGRS0 MRN0
  /METHOD=ENTER MRN0xRace MRN0xSES0 MRN0xIdeology0 MRN0xMGRS0 MGRS0xRace MGRS0xSES0 MGRS0
xIdeology0.
```

Regression

Notes

Output Created		15-DEC-2021 13:07:59
Comments		
Input	Data	C:\Users\njs5478\Dropbox\HM and COVID\0. Revise and Resubmit\2. R and R Data\Study 1b\Study1b_Data.sav
	Active Dataset	DataSet1
	Filter	GenderCC=1 (FILTER)
	Weight	<none>
	Split File	<none>
	N of Rows in Working Data File	119
Missing Value Handling	Definition of Missing	User-defined missing values are treated as missing.
	Cases Used	Statistics are based on cases with no missing values for any variable used.

Notes

Syntax		REGRESSION<br>/MISSING LISTWISE<br>/STATISTICS COEFF OUTS R ANOVA CHANGE ZPP<br>/CRITERIA=PIN(.05) POUT(.10)<br>/NOORIGIN<br>/DEPENDENT RepCongressX<br>/METHOD=ENTER Ideology0<br>/METHOD=ENTER RaceCC SES0<br>/METHOD=ENTER MGRS0 MRN0<br>/METHOD=ENTER MRN0xRace MRN0xSES0 MRN0xIdeology0 MRN0xMGRS0 MGRS0xRace MGRS0xSES0 MGRS0xIdeology0.
Resources	Processor Time	00:00:00.02
	Elapsed Time	00:00:00.02
	Memory Required	48560 bytes
	Additional Memory Required for Residual Plots	0 bytes

Variables Entered/Removed[a]

Model	Variables Entered	Variables Removed	Method
1	Ideology0[b]	.	Enter
2	SES0, RaceCC[b]	.	Enter
3	MGRS0, MRN0[b]	.	Enter
4	MRN0xSES0, MGRS0xRace, MRN0xIdeology0, MGRS0xIdeology0, MGRS0xSES0, MRN0xMGRS0, MRN0xRace[b]	.	Enter

a. Dependent Variable: RepCongressX

b. All requested variables entered.

Model Summary

Model	R	R Square	Adjusted R Square	Std. Error of the Estimate	Change Statistics R Square Change	F Change	df1
1	.693[a]	.480	.475	1.39161	.480	98.829	1
2	.722[b]	.521	.507	1.34899	.040	4.434	2
3	.731[c]	.535	.512	1.34156	.014	1.583	2
4	.752[d]	.565	.511	1.34331	.030	.962	7

Model Summary

Model	Change Statistics df2	Sig. F Change
1	107	.000
2	105	.014
3	103	.210
4	96	.464

a. Predictors: (Constant), Ideology0

b. Predictors: (Constant), Ideology0, SES0, RaceCC

c. Predictors: (Constant), Ideology0, SES0, RaceCC, MGRS0, MRN0

d. Predictors: (Constant), Ideology0, SES0, RaceCC, MGRS0, MRN0, MRN0xSES0, MGRS0xRace, MRN0xIdeology0, MGRS0xIdeology0, MGRS0xSES0, MRN0xMGRS0, MRN0xRace

ANOVA[a]

Model		Sum of Squares	df	Mean Square	F	Sig.
1	Regression	191.391	1	191.391	98.829	.000[b]
	Residual	207.215	107	1.937		
	Total	398.606	108			
2	Regression	207.528	3	69.176	38.013	.000[c]
	Residual	191.077	105	1.820		
	Total	398.606	108			
3	Regression	213.228	5	42.646	23.695	.000[d]
	Residual	185.378	103	1.800		
	Total	398.606	108			
4	Regression	225.374	12	18.781	10.408	.000[e]
	Residual	173.231	96	1.804		
	Total	398.606	108			

a. Dependent Variable: RepCongressX

b. Predictors: (Constant), Ideology0

c. Predictors: (Constant), Ideology0, SES0, RaceCC

d. Predictors: (Constant), Ideology0, SES0, RaceCC, MGRS0, MRN0

e. Predictors: (Constant), Ideology0, SES0, RaceCC, MGRS0, MRN0, MRN0xSES0, MGRS0xRace, MRN0xIdeology0, MGRS0xIdeology0, MGRS0xSES0, MRN0xMGRS0, MRN0xRace

Coefficients[a]

Model		Unstandardized Coefficients B	Std. Error	Standardized Coefficients Beta	t	Sig.
1	(Constant)	3.355	.138		24.334	.000
	Ideology0	.836	.084	.693	9.941	.000
2	(Constant)	3.145	.152		20.704	.000
	Ideology0	.748	.088	.620	8.514	.000
	RaceCC	.474	.163	.211	2.908	.004
	SES0	-.044	.147	-.021	-.303	.763
3	(Constant)	3.114	.185		16.837	.000
	Ideology0	.665	.109	.551	6.116	.000
	RaceCC	.539	.168	.240	3.199	.002
	SES0	-.056	.146	-.026	-.381	.704
	MGRS0	.219	.254	.066	.863	.390
	MRN0	.206	.206	.093	1.004	.318
4	(Constant)	3.019	.256		11.792	.000
	Ideology0	.732	.123	.607	5.935	.000
	RaceCC	.458	.216	.204	2.122	.036
	SES0	.083	.221	.039	.378	.706
	MGRS0	.084	.408	.025	.207	.837
	MRN0	.349	.289	.158	1.207	.230
	MRN0xRace	.122	.245	.059	.496	.621
	MRN0xSES0	-.111	.190	-.056	-.583	.562
	MRN0xIdeology0	-.078	.098	-.072	-.797	.428
	MRN0xMGRS0	.220	.337	.075	.652	.516
	MGRS0xRace	-.312	.334	-.097	-.935	.352
	MGRS0xSES0	.016	.358	.005	.045	.964
	MGRS0xIdeology0	.418	.203	.193	2.061	.042

Coefficients[a]

Model		Correlations Zero-order	Partial	Part
1	(Constant)			
	Ideology0	.693	.693	.693
2	(Constant)			
	Ideology0	.693	.639	.575
	RaceCC	.432	.273	.196
	SES0	.024	-.030	-.020
3	(Constant)			
	Ideology0	.693	.516	.411
	RaceCC	.432	.301	.215
	SES0	.024	-.038	-.026
	MGRS0	.169	.085	.058
	MRN0	.414	.098	.067
4	(Constant)			
	Ideology0	.693	.518	.399
	RaceCC	.432	.212	.143
	SES0	.024	.039	.025
	MGRS0	.169	.021	.014
	MRN0	.414	.122	.081
	MRN0xRace	.456	.051	.033
	MRN0xSES0	-.212	-.059	-.039
	MRN0xIdeology0	.240	-.081	-.054
	MRN0xMGRS0	-.195	.066	.044
	MGRS0xRace	-.034	-.095	-.063
	MGRS0xSES0	-.138	.005	.003
	MGRS0xIdeology0	-.121	.206	.139

a. Dependent Variable: RepCongressX

Excluded Variables[a]

Model		Beta In	t	Sig.	Partial Correlation	Collinearity Statistics Tolerance
1	RaceCC	.214[b]	2.975	.004	.278	.875
	SES0	-.044[b]	-.621	.536	-.060	.991
	MGRS0	.080[b]	1.141	.257	.110	.983
	MRN0	.053[b]	.642	.522	.062	.706
	MRN0xRace	.078[b]	.910	.365	.088	.660
	MRN0xSES0	-.045[b]	-.631	.530	-.061	.940
	MRN0xIdeology0	.029[b]	.397	.692	.039	.905
	MRN0xMGRS0	.106[b]	1.391	.167	.134	.834
	MGRS0xRace	-.085[b]	-1.219	.225	-.118	.995
	MGRS0xSES0	.016[b]	.227	.821	.022	.951
	MGRS0xIdeology0	.131[b]	1.787	.077	.171	.883
2	MGRS0	.100[c]	1.469	.145	.143	.974
	MRN0	.130[c]	1.558	.122	.151	.651
	MRN0xRace	.029[c]	.344	.731	.034	.633
	MRN0xSES0	-.053[c]	-.734	.465	-.072	.866
	MRN0xIdeology0	-.025[c]	-.333	.740	-.033	.809
	MRN0xMGRS0	.097[c]	1.256	.212	.122	.767
	MGRS0xRace	-.021[c]	-.289	.773	-.028	.884
	MGRS0xSES0	-.054[c]	-.652	.516	-.064	.664
	MGRS0xIdeology0	.104[c]	1.409	.162	.137	.830
3	MRN0xRace	-.014[d]	-.146	.884	-.014	.532
	MRN0xSES0	-.019[d]	-.250	.803	-.025	.796
	MRN0xIdeology0	.010[d]	.122	.903	.012	.756
	MRN0xMGRS0	.129[d]	1.361	.177	.134	.502
	MGRS0xRace	-.060[d]	-.756	.452	-.075	.725
	MGRS0xSES0	-.012[d]	-.139	.890	-.014	.606
	MGRS0xIdeology0	.159[d]	2.015	.047	.196	.704

a. Dependent Variable: RepCongressX

b. Predictors in the Model: (Constant), Ideology0

c. Predictors in the Model: (Constant), Ideology0, SES0, RaceCC

d. Predictors in the Model: (Constant), Ideology0, SES0, RaceCC, MGRS0, MRN0

```
REGRESSION
  /MISSING LISTWISE
  /STATISTICS COEFF OUTS R ANOVA CHANGE ZPP
  /CRITERIA=PIN(.05) POUT(.10)
  /NOORIGIN
  /DEPENDENT DemCongressX
  /METHOD=ENTER Ideology0
  /METHOD=ENTER RaceCC SES0
  /METHOD=ENTER MGRS0 MRN0
  /METHOD=ENTER MRN0xRace MRN0xSES0 MRN0xIdeology0 MRN0xMGRS0 MGRS0xRace MGRS0xSES0 MGRS0
xIdeology0.
```

Regression

Notes

Output Created		15-DEC-2021 13:07:59
Comments		
Input	Data	C: \Users\njs5478\Dropbox\HM and COVID\0. Revise and Resubmit\2. R and R Data\Study 1b\Study1b_Data.sav
	Active Dataset	DataSet1
	Filter	GenderCC=1 (FILTER)
	Weight	<none>
	Split File	<none>
	N of Rows in Working Data File	119
Missing Value Handling	Definition of Missing	User-defined missing values are treated as missing.
	Cases Used	Statistics are based on cases with no missing values for any variable used.

Notes

Syntax		REGRESSION<br>/MISSING LISTWISE<br>/STATISTICS COEFF OUTS R ANOVA CHANGE ZPP<br>/CRITERIA=PIN(.05) POUT(.10)<br>/NOORIGIN<br>/DEPENDENT DemCongressX<br>/METHOD=ENTER Ideology0<br>/METHOD=ENTER RaceCC SES0<br>/METHOD=ENTER MGRS0 MRN0<br>/METHOD=ENTER MRN0xRace MRN0xSES0 MRN0xIdeology0 MRN0xMGRS0 MGRS0xRace MGRS0xSES0 MGRS0xIdeology0.
Resources	Processor Time	00:00:00.02
	Elapsed Time	00:00:00.03
	Memory Required	48560 bytes
	Additional Memory Required for Residual Plots	0 bytes

Variables Entered/Removed[a]

Model	Variables Entered	Variables Removed	Method
1	Ideology0[b]	.	Enter
2	SES0, RaceCC[b]	.	Enter
3	MGRS0, MRN0[b]	.	Enter
4	MRN0xSES0, MGRS0xRace , MRN0xIdeology0, MGRS0xIdeology0, MGRS0xSES0, MRN0xMGRS0, MRN0xRace[b]	.	Enter

a. Dependent Variable: DemCongressX

b. All requested variables entered.

Model Summary

Model	R	R Square	Adjusted R Square	Std. Error of the Estimate	Change Statistics		
					R Square Change	F Change	df1
1	.677[a]	.458	.453	1.24106	.458	88.636	1
2	.679[b]	.461	.445	1.24962	.003	.283	2
3	.696[c]	.485	.459	1.23355	.024	2.351	2
4	.710[d]	.504	.441	1.25391	.020	.535	7

Model Summary

Model	Change Statistics	
	df2	Sig. F Change
1	105	.000
2	103	.754
3	101	.101
4	94	.806

a. Predictors: (Constant), Ideology0

b. Predictors: (Constant), Ideology0, SES0, RaceCC

c. Predictors: (Constant), Ideology0, SES0, RaceCC, MGRS0, MRN0

d. Predictors: (Constant), Ideology0, SES0, RaceCC, MGRS0, MRN0, MRN0xSES0, MGRS0xRace, MRN0xIdeology0, MGRS0xIdeology0, MGRS0xSES0, MRN0xMGRS0, MRN0xRace

ANOVA[a]

Model		Sum of Squares	df	Mean Square	F	Sig.
1	Regression	136.520	1	136.520	88.636	.000[b]
	Residual	161.723	105	1.540		
	Total	298.243	106			
2	Regression	137.402	3	45.801	29.330	.000[c]
	Residual	160.841	103	1.562		
	Total	298.243	106			
3	Regression	144.556	5	28.911	19.000	.000[d]
	Residual	153.687	101	1.522		
	Total	298.243	106			
4	Regression	150.447	12	12.537	7.974	.000[e]
	Residual	147.796	94	1.572		
	Total	298.243	106			

a. Dependent Variable: DemCongressX

b. Predictors: (Constant), Ideology0

c. Predictors: (Constant), Ideology0, SES0, RaceCC

d. Predictors: (Constant), Ideology0, SES0, RaceCC, MGRS0, MRN0

e. Predictors: (Constant), Ideology0, SES0, RaceCC, MGRS0, MRN0, MRN0xSES0, MGRS0xRace, MRN0xIdeology0, MGRS0xIdeology0, MGRS0xSES0, MRN0xMGRS0, MRN0xRace

Coefficients[a]

Model		Unstandardized Coefficients B	Std. Error	Standardized Coefficients Beta	t	Sig.
1	(Constant)	4.231	.125		33.968	.000
	Ideology0	-.719	.076	-.677	-9.415	.000
2	(Constant)	4.183	.141		29.634	.000
	Ideology0	-.741	.083	-.697	-8.894	.000
	RaceCC	.112	.152	.058	.736	.463
	SES0	-.009	.136	-.005	-.066	.948
3	(Constant)	4.362	.171		25.438	.000
	Ideology0	-.616	.100	-.580	-6.138	.000
	RaceCC	.028	.156	.014	.179	.858
	SES0	.005	.135	.003	.037	.970
	MGRS0	.148	.234	.051	.633	.528
	MRN0	-.408	.191	-.208	-2.136	.035
4	(Constant)	4.446	.240		18.544	.000
	Ideology0	-.641	.117	-.603	-5.466	.000
	RaceCC	-.112	.203	-.058	-.553	.582
	SES0	-.020	.206	-.011	-.099	.922
	MGRS0	.362	.383	.125	.944	.347
	MRN0	-.519	.271	-.265	-1.917	.058
	MRN0xRace	.243	.233	.134	1.047	.298
	MRN0xSES0	.044	.178	.026	.246	.806
	MRN0xIdeology0	.027	.097	.028	.278	.781
	MRN0xMGRS0	-.155	.317	-.060	-.489	.626
	MGRS0xRace	-.271	.312	-.098	-.870	.387
	MGRS0xSES0	-.206	.335	-.069	-.616	.539
	MGRS0xIdeology0	.237	.190	.125	1.249	.215

Coefficients[a]

Model		Correlations Zero-order	Partial	Part
1	(Constant)			
	Ideology0	-.677	-.677	-.677
2	(Constant)			
	Ideology0	-.677	-.659	-.644
	RaceCC	-.198	.072	.053
	SES0	-.070	-.007	-.005
3	(Constant)			
	Ideology0	-.677	-.521	-.438
	RaceCC	-.198	.018	.013
	SES0	-.070	.004	.003
	MGRS0	-.115	.063	.045
	MRN0	-.487	-.208	-.153
4	(Constant)			
	Ideology0	-.677	-.491	-.397
	RaceCC	-.198	-.057	-.040
	SES0	-.070	-.010	-.007
	MGRS0	-.115	.097	.069
	MRN0	-.487	-.194	-.139
	MRN0xRace	-.371	.107	.076
	MRN0xSES0	.190	.025	.018
	MRN0xIdeology0	-.148	.029	.020
	MRN0xMGRS0	.327	-.050	-.035
	MGRS0xRace	-.042	-.089	-.063
	MGRS0xSES0	.166	-.063	-.045
	MGRS0xIdeology0	.364	.128	.091

a. Dependent Variable: DemCongressX

Excluded Variables[a]

Model		Beta In	t	Sig.	Partial Correlation	Collinearity Statistics Tolerance
1	RaceCC	.058[b]	.752	.454	.074	.865
	SES0	-.011[b]	-.152	.879	-.015	.992
	MGRS0	-.032[b]	-.436	.664	-.043	.985
	MRN0	-.184[b]	-2.228	.028	-.213	.727
	MRN0xRace	.017[b]	.197	.844	.019	.679
	MRN0xSES0	.029[b]	.386	.700	.038	.942
	MRN0xIdeology0	.105[b]	1.370	.174	.133	.874
	MRN0xMGRS0	.068[b]	.869	.387	.085	.841
	MGRS0xRace	.002[b]	.022	.983	.002	.996
	MGRS0xSES0	.019[b]	.250	.803	.025	.952
	MGRS0xIdeology0	.160[b]	2.141	.035	.205	.893
2	MGRS0	-.027[c]	-.365	.716	-.036	.976
	MRN0	-.180[c]	-2.080	.040	-.202	.677
	MRN0xRace	.003[c]	.033	.974	.003	.647
	MRN0xSES0	.030[c]	.384	.701	.038	.869
	MRN0xIdeology0	.102[c]	1.259	.211	.124	.788
	MRN0xMGRS0	.070[c]	.844	.400	.083	.774
	MGRS0xRace	.022[c]	.282	.779	.028	.887
	MGRS0xSES0	.006[c]	.066	.948	.006	.662
	MGRS0xIdeology0	.161[c]	2.073	.041	.201	.841
3	MRN0xRace	.091[d]	.947	.346	.094	.550
	MRN0xSES0	.004[d]	.044	.965	.004	.798
	MRN0xIdeology0	.080[d]	.969	.335	.096	.741
	MRN0xMGRS0	.011[d]	.108	.914	.011	.505
	MGRS0xRace	.005[d]	.061	.952	.006	.726
	MGRS0xSES0	-.044[d]	-.480	.632	-.048	.601
	MGRS0xIdeology0	.113[d]	1.345	.182	.133	.716

a. Dependent Variable: DemCongressX

b. Predictors in the Model: (Constant), Ideology0

c. Predictors in the Model: (Constant), Ideology0, SES0, RaceCC

d. Predictors in the Model: (Constant), Ideology0, SES0, RaceCC, MGRS0, MRN0

```
REGRESSION
  /MISSING LISTWISE
  /STATISTICS COEFF OUTS R ANOVA CHANGE ZPP
  /CRITERIA=PIN(.05) POUT(.10)
  /NOORIGIN
  /DEPENDENT StateX
  /METHOD=ENTER Ideology0
  /METHOD=ENTER RaceCC SES0
  /METHOD=ENTER MGRS0 MRN0
  /METHOD=ENTER MRN0xRace MRN0xSES0 MRN0xIdeology0 MRN0xMGRS0 MGRS0xRace MGRS0xSES0 MGRS0
xIdeology0.
```

Regression

Notes

Output Created		15-DEC-2021 13:07:59
Comments		
Input	Data	C:\Users\njs5478\Dropbox\HM and COVID\0. Revise and Resubmit\2. R and R Data\Study 1b\Study1b_Data.sav
	Active Dataset	DataSet1
	Filter	GenderCC=1 (FILTER)
	Weight	<none>
	Split File	<none>
	N of Rows in Working Data File	119
Missing Value Handling	Definition of Missing	User-defined missing values are treated as missing.
	Cases Used	Statistics are based on cases with no missing values for any variable used.

Notes

Syntax		REGRESSION<br>/MISSING LISTWISE<br>/STATISTICS COEFF OUTS R ANOVA CHANGE ZPP<br>/CRITERIA=PIN(.05) POUT(.10)<br>/NOORIGIN<br>/DEPENDENT StateX<br>/METHOD=ENTER Ideology0<br>/METHOD=ENTER RaceCC SES0<br>/METHOD=ENTER MGRS0 MRN0<br>/METHOD=ENTER MRN0xRace MRN0xSES0 MRN0xIdeology0 MRN0xMGRS0 MGRS0xRace MGRS0xSES0 MGRS0xIdeology0.
Resources	Processor Time	00:00:00.03
	Elapsed Time	00:00:00.02
	Memory Required	48560 bytes
	Additional Memory Required for Residual Plots	0 bytes

Variables Entered/Removed[a]

Model	Variables Entered	Variables Removed	Method
1	Ideology0[b]	.	Enter
2	SES0, RaceCC[b]	.	Enter
3	MGRS0, MRN0[b]	.	Enter
4	MRN0xSES0, MRN0xIdeology0, MGRS0xRace, MGRS0xIdeology0, MGRS0xSES0, MRN0xMGRS0, MRN0xRace[b]	.	Enter

a. Dependent Variable: StateX

b. All requested variables entered.

Model Summary

Model	R	R Square	Adjusted R Square	Std. Error of the Estimate	Change Statistics		
					R Square Change	F Change	df1
1	.308[a]	.095	.087	1.63863	.095	11.752	1
2	.310[b]	.096	.071	1.65267	.001	.053	2
3	.344[c]	.118	.078	1.64698	.023	1.380	2
4	.402[d]	.162	.062	1.66083	.043	.744	7

Model Summary

Model	Change Statistics	
	df2	Sig. F Change
1	112	.001
2	110	.948
3	108	.256
4	101	.635

a. Predictors: (Constant), Ideology0

b. Predictors: (Constant), Ideology0, SES0, RaceCC

c. Predictors: (Constant), Ideology0, SES0, RaceCC, MGRS0, MRN0

d. Predictors: (Constant), Ideology0, SES0, RaceCC, MGRS0, MRN0, MRN0xSES0, MRN0xIdeology0, MGRS0xRace, MGRS0xIdeology0, MGRS0xSES0, MRN0xMGRS0, MRN0xRace

ANOVA[a]

Model		Sum of Squares	df	Mean Square	F	Sig.
1	Regression	31.556	1	31.556	11.752	.001[b]
	Residual	300.734	112	2.685		
	Total	332.289	113			
2	Regression	31.846	3	10.615	3.887	.011[c]
	Residual	300.443	110	2.731		
	Total	332.289	113			
3	Regression	39.333	5	7.867	2.900	.017[d]
	Residual	292.956	108	2.713		
	Total	332.289	113			
4	Regression	53.695	12	4.475	1.622	.097[e]
	Residual	278.594	101	2.758		
	Total	332.289	113			

a. Dependent Variable: StateX

b. Predictors: (Constant), Ideology0

c. Predictors: (Constant), Ideology0, SES0, RaceCC

d. Predictors: (Constant), Ideology0, SES0, RaceCC, MGRS0, MRN0

e. Predictors: (Constant), Ideology0, SES0, RaceCC, MGRS0, MRN0, MRN0xSES0, MRN0xIdeology0, MGRS0xRace, MGRS0xIdeology0, MGRS0xSES0, MRN0xMGRS0, MRN0xRace

Coefficients[a]

Model		Unstandardized Coefficients B	Unstandardized Coefficients Std. Error	Standardized Coefficients Beta	t	Sig.
1	(Constant)	4.061	.159		25.574	.000
	Ideology0	-.332	.097	-.308	-3.428	.001
2	(Constant)	4.086	.183		22.390	.000
	Ideology0	-.322	.105	-.299	-3.065	.003
	RaceCC	-.057	.196	-.029	-.294	.770
	SES0	.019	.178	.010	.106	.915
3	(Constant)	4.172	.221		18.919	.000
	Ideology0	-.207	.131	-.192	-1.584	.116
	RaceCC	-.149	.204	-.074	-.732	.466
	SES0	.035	.178	.018	.196	.845
	MGRS0	-.119	.305	-.040	-.390	.697
	MRN0	-.312	.242	-.161	-1.291	.199
4	(Constant)	4.105	.310		13.238	.000
	Ideology0	-.303	.147	-.281	-2.061	.042
	RaceCC	-.207	.263	-.103	-.788	.433
	SES0	.205	.267	.106	.767	.445
	MGRS0	-.011	.500	-.004	-.022	.983
	MRN0	-.235	.352	-.121	-.667	.506
	MRN0xRace	-.031	.299	-.017	-.105	.917
	MRN0xSES0	-.146	.227	-.082	-.642	.523
	MRN0xIdeology0	.207	.115	.215	1.798	.075
	MRN0xMGRS0	-.215	.409	-.082	-.525	.601
	MGRS0xRace	.080	.409	.028	.195	.846
	MGRS0xSES0	.214	.436	.068	.491	.624
	MGRS0xIdeology0	.146	.246	.076	.595	.553

Coefficients[a]

Model		Correlations Zero-order	Partial	Part
1	(Constant)			
	Ideology0	-.308	-.308	-.308
2	(Constant)			
	Ideology0	-.308	-.280	-.278
	RaceCC	-.135	-.028	-.027
	SES0	-.012	.010	.010
3	(Constant)			
	Ideology0	-.308	-.151	-.143
	RaceCC	-.135	-.070	-.066
	SES0	-.012	.019	.018
	MGRS0	-.124	-.038	-.035
	MRN0	-.277	-.123	-.117
4	(Constant)			
	Ideology0	-.308	-.201	-.188
	RaceCC	-.135	-.078	-.072
	SES0	-.012	.076	.070
	MGRS0	-.124	-.002	-.002
	MRN0	-.277	-.066	-.061
	MRN0xRace	-.183	-.010	-.010
	MRN0xSES0	.103	-.064	-.058
	MRN0xIdeology0	.088	.176	.164
	MRN0xMGRS0	.137	-.052	-.048
	MGRS0xRace	.022	.019	.018
	MGRS0xSES0	.113	.049	.045
	MGRS0xIdeology0	.235	.059	.054

a. Dependent Variable: StateX

Excluded Variables[a]

Model		Beta In	t	Sig.	Partial Correlation	Collinearity Statistics Tolerance
1	RaceCC	-.030[b]	-.309	.758	-.029	.876
	SES0	.013[b]	.142	.887	.013	.993
	MGRS0	-.091[b]	-1.010	.315	-.095	.988
	MRN0	-.155[b]	-1.461	.147	-.137	.708
	MRN0xRace	-.003[b]	-.026	.979	-.003	.655
	MRN0xSES0	.037[b]	.402	.688	.038	.952
	MRN0xIdeology0	.199[b]	2.148	.034	.200	.909
	MRN0xMGRS0	.011[b]	.111	.912	.011	.827
	MGRS0xRace	.039[b]	.435	.665	.041	.997
	MGRS0xSES0	.049[b]	.529	.598	.050	.953
	MGRS0xIdeology0	.142[b]	1.475	.143	.139	.866
2	MGRS0	-.096[c]	-1.042	.300	-.099	.977
	MRN0	-.182[c]	-1.621	.108	-.153	.646
	MRN0xRace	.004[c]	.033	.973	.003	.632
	MRN0xSES0	.038[c]	.395	.694	.038	.885
	MRN0xIdeology0	.230[c]	2.344	.021	.219	.823
	MRN0xMGRS0	.017[c]	.162	.871	.016	.755
	MGRS0xRace	.033[c]	.343	.732	.033	.886
	MGRS0xSES0	.086[c]	.769	.444	.073	.663
	MGRS0xIdeology0	.157[c]	1.573	.119	.149	.815
3	MRN0xRace	.091[d]	.721	.473	.069	.511
	MRN0xSES0	.002[d]	.016	.988	.002	.817
	MRN0xIdeology0	.207[d]	2.052	.043	.195	.783
	MRN0xMGRS0	.005[d]	.038	.969	.004	.487
	MGRS0xRace	.064[d]	.594	.554	.057	.718
	MGRS0xSES0	.036[d]	.314	.754	.030	.608
	MGRS0xIdeology0	.127[d]	1.177	.242	.113	.701

a. Dependent Variable: StateX

b. Predictors in the Model: (Constant), Ideology0

c. Predictors in the Model: (Constant), Ideology0, SES0, RaceCC

d. Predictors in the Model: (Constant), Ideology0, SES0, RaceCC, MGRS0, MRN0

```
REGRESSION
  /MISSING LISTWISE
  /STATISTICS COEFF OUTS R ANOVA CHANGE ZPP
  /CRITERIA=PIN(.05) POUT(.10)
  /NOORIGIN
  /DEPENDENT Risk_Rules
  /METHOD=ENTER Ideology0
  /METHOD=ENTER RaceCC SES0
  /METHOD=ENTER MGRS0 MRN0
  /METHOD=ENTER MRN0xRace MRN0xSES0 MRN0xIdeology0 MRN0xMGRS0 MGRS0xRace MGRS0xSES0 MGRS0
xIdeology0.
```

Regression

Notes

Output Created		15-DEC-2021 13:07:59
Comments		
Input	Data	C:\Users\njs5478\Dropbox\HM and COVID\0. Revise and Resubmit\2. R and R Data\Study 1b\Study1b_Data.sav
	Active Dataset	DataSet1
	Filter	GenderCC=1 (FILTER)
	Weight	<none>
	Split File	<none>
	N of Rows in Working Data File	119
Missing Value Handling	Definition of Missing	User-defined missing values are treated as missing.
	Cases Used	Statistics are based on cases with no missing values for any variable used.

Notes

Syntax		REGRESSION<br>/MISSING LISTWISE<br>/STATISTICS COEFF OUTS R ANOVA CHANGE ZPP<br>/CRITERIA=PIN(.05) POUT(.10)<br>/NOORIGIN<br>/DEPENDENT Risk_Rules<br>/METHOD=ENTER Ideology0<br>/METHOD=ENTER RaceCC SES0<br>/METHOD=ENTER MGRS0 MRN0<br>/METHOD=ENTER MRN0xRace MRN0xSES0 MRN0xIdeology0 MRN0xMGRS0 MGRS0xRace MGRS0xSES0 MGRS0xIdeology0.
Resources	Processor Time	00:00:00.02
	Elapsed Time	00:00:00.02
	Memory Required	48560 bytes
	Additional Memory Required for Residual Plots	0 bytes

Variables Entered/Removed[a]

Model	Variables Entered	Variables Removed	Method
1	Ideology0[b]	.	Enter
2	SES0, RaceCC[b]	.	Enter
3	MGRS0, MRN0[b]	.	Enter
4	MRN0xSES0, MRN0xIdeology0, MGRS0xRace, MGRS0xIdeology0, MGRS0xSES0, MRN0xMGRS0, MRN0xRace[b]	.	Enter

a. Dependent Variable: Risk_Rules

b. All requested variables entered.

Model Summary

Model	R	R Square	Adjusted R Square	Std. Error of the Estimate	Change Statistics		
					R Square Change	F Change	df1
1	.545[a]	.297	.291	.92766	.297	49.028	1
2	.549[b]	.302	.283	.93273	.005	.372	2
3	.605[c]	.366	.338	.89631	.065	5.725	2
4	.624[d]	.390	.320	.90863	.023	.569	7

Model Summary

Model	Change Statistics	
	df2	Sig. F Change
1	116	.000
2	114	.690
3	112	.004
4	105	.779

a. Predictors: (Constant), Ideology0

b. Predictors: (Constant), Ideology0, SES0, RaceCC

c. Predictors: (Constant), Ideology0, SES0, RaceCC, MGRS0, MRN0

d. Predictors: (Constant), Ideology0, SES0, RaceCC, MGRS0, MRN0, MRN0xSES0, MRN0xIdeology0, MGRS0xRace, MGRS0xIdeology0, MGRS0xSES0, MRN0xMGRS0, MRN0xRace

ANOVA[a]

Model		Sum of Squares	df	Mean Square	F	Sig.
1	Regression	42.191	1	42.191	49.028	.000[b]
	Residual	99.824	116	.861		
	Total	142.015	117			
2	Regression	42.837	3	14.279	16.413	.000[c]
	Residual	99.177	114	.870		
	Total	142.015	117			
3	Regression	52.036	5	10.407	12.954	.000[d]
	Residual	89.979	112	.803		
	Total	142.015	117			
4	Regression	55.326	12	4.610	5.584	.000[e]
	Residual	86.689	105	.826		
	Total	142.015	117			

a. Dependent Variable: Risk_Rules

b. Predictors: (Constant), Ideology0

c. Predictors: (Constant), Ideology0, SES0, RaceCC

d. Predictors: (Constant), Ideology0, SES0, RaceCC, MGRS0, MRN0

e. Predictors: (Constant), Ideology0, SES0, RaceCC, MGRS0, MRN0, MRN0xSES0, MRN0xIdeology0, MGRS0xRace, MGRS0xIdeology0, MGRS0xSES0, MRN0xMGRS0, MRN0xRace

Coefficients[a]

Model		Unstandardized Coefficients B	Unstandardized Coefficients Std. Error	Standardized Coefficients Beta	t	Sig.
1	(Constant)	3.361	.088		38.100	.000
	Ideology0	.376	.054	.545	7.002	.000
2	(Constant)	3.324	.101		32.839	.000
	Ideology0	.361	.058	.524	6.209	.000
	RaceCC	.083	.109	.064	.760	.449
	SES0	-.032	.100	-.025	-.318	.751
3	(Constant)	3.225	.119		27.201	.000
	Ideology0	.241	.068	.349	3.522	.001
	RaceCC	.180	.109	.140	1.652	.101
	SES0	-.056	.096	-.044	-.578	.565
	MGRS0	.118	.163	.060	.722	.472
	MRN0	.346	.128	.274	2.702	.008
4	(Constant)	3.319	.167		19.829	.000
	Ideology0	.227	.077	.329	2.957	.004
	RaceCC	.127	.143	.099	.894	.374
	SES0	-.151	.145	-.119	-1.043	.299
	MGRS0	.221	.267	.113	.828	.409
	MRN0	.219	.186	.174	1.179	.241
	MRN0xRace	.190	.162	.160	1.174	.243
	MRN0xSES0	.069	.123	.059	.557	.579
	MRN0xIdeology0	-.017	.062	-.028	-.280	.780
	MRN0xMGRS0	-.122	.224	-.071	-.544	.587
	MGRS0xRace	-.042	.219	-.023	-.192	.848
	MGRS0xSES0	-.246	.237	-.121	-1.039	.301
	MGRS0xIdeology0	.122	.132	.098	.920	.360

Coefficients[a]

Model		Correlations Zero-order	Partial	Part
1	(Constant)			
	Ideology0	.545	.545	.545
2	(Constant)			
	Ideology0	.545	.503	.486
	RaceCC	.255	.071	.059
	SES0	.003	-.030	-.025
3	(Constant)			
	Ideology0	.545	.316	.265
	RaceCC	.255	.154	.124
	SES0	.003	-.055	-.043
	MGRS0	.207	.068	.054
	MRN0	.468	.247	.203
4	(Constant)			
	Ideology0	.545	.277	.225
	RaceCC	.255	.087	.068
	SES0	.003	-.101	-.080
	MGRS0	.207	.081	.063
	MRN0	.468	.114	.090
	MRN0xRace	.477	.114	.089
	MRN0xSES0	-.185	.054	.042
	MRN0xIdeology0	.159	-.027	-.021
	MRN0xMGRS0	-.257	-.053	-.041
	MGRS0xRace	.085	-.019	-.015
	MGRS0xSES0	-.193	-.101	-.079
	MGRS0xIdeology0	-.201	.089	.070

a. Dependent Variable: Risk_Rules

Excluded Variables[a]

Model		Beta In	t	Sig.	Partial Correlation	Collinearity Statistics Tolerance
1	RaceCC	.067[b]	.804	.423	.075	.870
	SES0	-.032[b]	-.408	.684	-.038	.996
	MGRS0	.148[b]	1.908	.059	.175	.987
	MRN0	.254[b]	2.879	.005	.259	.733
	MRN0xRace	.245[b]	2.643	.009	.239	.670
	MRN0xSES0	-.072[b]	-.897	.372	-.083	.954
	MRN0xIdeology0	-.005[b]	-.064	.949	-.006	.910
	MRN0xMGRS0	-.049[b]	-.576	.566	-.054	.843
	MGRS0xRace	.068[b]	.871	.386	.081	.999
	MGRS0xSES0	-.087[b]	-1.093	.277	-.101	.960
	MGRS0xIdeology0	.002[b]	.029	.977	.003	.861
2	MGRS0	.155[c]	1.982	.050	.183	.980
	MRN0	.304[c]	3.313	.001	.298	.668
	MRN0xRace	.239[c]	2.511	.013	.230	.647
	MRN0xSES0	-.072[c]	-.864	.389	-.081	.888
	MRN0xIdeology0	-.028[c]	-.319	.750	-.030	.828
	MRN0xMGRS0	-.067[c]	-.752	.453	-.071	.765
	MGRS0xRace	.103[c]	1.238	.218	.116	.881
	MGRS0xSES0	-.164[c]	-1.722	.088	-.160	.661
	MGRS0xIdeology0	-.011[c]	-.122	.903	-.011	.817
3	MRN0xRace	.143[d]	1.368	.174	.129	.510
	MRN0xSES0	-.009[d]	-.113	.910	-.011	.818
	MRN0xIdeology0	.022[d]	.254	.800	.024	.792
	MRN0xMGRS0	-.059[d]	-.545	.587	-.052	.489
	MGRS0xRace	.087[d]	.984	.327	.093	.732
	MGRS0xSES0	-.084[d]	-.872	.385	-.082	.610
	MGRS0xIdeology0	.080[d]	.895	.373	.085	.718

a. Dependent Variable: Risk_Rules

b. Predictors in the Model: (Constant), Ideology0

c. Predictors in the Model: (Constant), Ideology0, SES0, RaceCC

d. Predictors in the Model: (Constant), Ideology0, SES0, RaceCC, MGRS0, MRN0

```
REGRESSION
  /MISSING LISTWISE
  /STATISTICS COEFF OUTS R ANOVA CHANGE ZPP
  /CRITERIA=PIN(.05) POUT(.10)
  /NOORIGIN
  /DEPENDENT Risk_Help
  /METHOD=ENTER Ideology0
  /METHOD=ENTER RaceCC SES0
  /METHOD=ENTER MGRS0 MRN0
  /METHOD=ENTER MRN0xRace MRN0xSES0 MRN0xIdeology0 MRN0xMGRS0 MGRS0xRace MGRS0xSES0 MGRS0
xIdeology0.
```

Regression

Notes

Output Created		15-DEC-2021 13:07:59
Comments		
Input	Data	C: \Users\njs5478\Dropbox\HM and COVID\0. Revise and Resubmit\2. R and R Data\Study 1b\Study1b_Data.sav
	Active Dataset	DataSet1
	Filter	GenderCC=1 (FILTER)
	Weight	<none>
	Split File	<none>
	N of Rows in Working Data File	119
Missing Value Handling	Definition of Missing	User-defined missing values are treated as missing.
	Cases Used	Statistics are based on cases with no missing values for any variable used.

Notes

Syntax		REGRESSION<br>/MISSING LISTWISE<br>/STATISTICS COEFF OUTS R ANOVA CHANGE ZPP<br>/CRITERIA=PIN(.05) POUT(.10)<br>/NOORIGIN<br>/DEPENDENT Risk_Help<br>/METHOD=ENTER Ideology0<br>/METHOD=ENTER RaceCC SES0<br>/METHOD=ENTER MGRS0 MRN0<br>/METHOD=ENTER MRN0xRace MRN0xSES0 MRN0xIdeology0 MRN0xMGRS0 MGRS0xRace MGRS0xSES0 MGRS0xIdeology0.
Resources	Processor Time	00:00:00.02
	Elapsed Time	00:00:00.02
	Memory Required	48560 bytes
	Additional Memory Required for Residual Plots	0 bytes

Variables Entered/Removed[a]

Model	Variables Entered	Variables Removed	Method
1	Ideology0[b]	.	Enter
2	SES0, RaceCC[b]	.	Enter
3	MGRS0, MRN0[b]	.	Enter
4	MRN0xSES0, MRN0xIdeology0, MGRS0xRace, MGRS0xIdeology0, MGRS0xSES0, MRN0xMGRS0, MRN0xRace[b]	.	Enter

a. Dependent Variable: Risk_Help

b. All requested variables entered.

Model Summary

Model	R	R Square	Adjusted R Square	Std. Error of the Estimate	Change Statistics		
					R Square Change	F Change	df1
1	.205[a]	.042	.034	1.47415	.042	5.067	1
2	.330[b]	.109	.086	1.43382	.067	4.308	2
3	.349[c]	.122	.083	1.43604	.013	.824	2
4	.379[d]	.144	.046	1.46488	.021	.376	7

Model Summary

Model	Change Statistics	
	df2	Sig. F Change
1	116	.026
2	114	.016
3	112	.441
4	105	.914

a. Predictors: (Constant), Ideology0

b. Predictors: (Constant), Ideology0, SES0, RaceCC

c. Predictors: (Constant), Ideology0, SES0, RaceCC, MGRS0, MRN0

d. Predictors: (Constant), Ideology0, SES0, RaceCC, MGRS0, MRN0, MRN0xSES0, MRN0xIdeology0, MGRS0xRace, MGRS0xIdeology0, MGRS0xSES0, MRN0xMGRS0, MRN0xRace

ANOVA[a]

Model		Sum of Squares	df	Mean Square	F	Sig.
1	Regression	11.011	1	11.011	5.067	.026[b]
	Residual	252.081	116	2.173		
	Total	263.092	117			
2	Regression	28.726	3	9.575	4.658	.004[c]
	Residual	234.366	114	2.056		
	Total	263.092	117			
3	Regression	32.126	5	6.425	3.116	.011[d]
	Residual	230.967	112	2.062		
	Total	263.092	117			
4	Regression	37.776	12	3.148	1.467	.148[e]
	Residual	225.316	105	2.146		
	Total	263.092	117			

a. Dependent Variable: Risk_Help

b. Predictors: (Constant), Ideology0

c. Predictors: (Constant), Ideology0, SES0, RaceCC

d. Predictors: (Constant), Ideology0, SES0, RaceCC, MGRS0, MRN0

e. Predictors: (Constant), Ideology0, SES0, RaceCC, MGRS0, MRN0, MRN0xSES0, MRN0xIdeology0, MGRS0xRace, MGRS0xIdeology0, MGRS0xSES0, MRN0xMGRS0, MRN0xRace

Coefficients[a]

Model		Unstandardized Coefficients B	Unstandardized Coefficients Std. Error	Standardized Coefficients Beta	t	Sig.
1	(Constant)	3.700	.140		26.400	.000
	Ideology0	-.192	.085	-.205	-2.251	.026
2	(Constant)	3.893	.156		25.018	.000
	Ideology0	-.101	.089	-.108	-1.133	.260
	RaceCC	-.422	.167	-.240	-2.521	.013
	SES0	-.273	.153	-.159	-1.784	.077
3	(Constant)	3.771	.190		19.849	.000
	Ideology0	-.133	.110	-.142	-1.213	.228
	RaceCC	-.403	.175	-.230	-2.305	.023
	SES0	-.279	.154	-.163	-1.815	.072
	MGRS0	-.332	.262	-.125	-1.267	.208
	MRN0	.147	.205	.086	.718	.474
4	(Constant)	3.867	.270		14.330	.000
	Ideology0	-.111	.124	-.119	-.900	.370
	RaceCC	-.352	.230	-.200	-1.529	.129
	SES0	-.479	.233	-.279	-2.054	.042
	MGRS0	-.223	.431	-.084	-.517	.606
	MRN0	.076	.300	.044	.255	.799
	MRN0xRace	-.011	.260	-.007	-.041	.967
	MRN0xSES0	.246	.199	.155	1.238	.218
	MRN0xIdeology0	-.072	.100	-.085	-.721	.472
	MRN0xMGRS0	-.182	.361	-.078	-.503	.616
	MGRS0xRace	.105	.353	.042	.298	.766
	MGRS0xSES0	-.173	.382	-.062	-.451	.653
	MGRS0xIdeology0	-.039	.214	-.023	-.185	.854

Coefficients[a]

Model		Correlations Zero-order	Partial	Part
1	(Constant)			
	Ideology0	-.205	-.205	-.205
2	(Constant)			
	Ideology0	-.205	-.105	-.100
	RaceCC	-.266	-.230	-.223
	SES0	-.146	-.165	-.158
3	(Constant)			
	Ideology0	-.205	-.114	-.107
	RaceCC	-.266	-.213	-.204
	SES0	-.146	-.169	-.161
	MGRS0	-.101	-.119	-.112
	MRN0	-.050	.068	.064
4	(Constant)			
	Ideology0	-.205	-.088	-.081
	RaceCC	-.266	-.148	-.138
	SES0	-.146	-.197	-.186
	MGRS0	-.101	-.050	-.047
	MRN0	-.050	.025	.023
	MRN0xRace	-.137	-.004	-.004
	MRN0xSES0	.086	.120	.112
	MRN0xIdeology0	-.124	-.070	-.065
	MRN0xMGRS0	.011	-.049	-.045
	MGRS0xRace	.041	.029	.027
	MGRS0xSES0	.090	-.044	-.041
	MGRS0xIdeology0	.039	-.018	-.017

a. Dependent Variable: Risk_Help

Excluded Variables[a]

Model		Beta In	t	Sig.	Partial Correlation	Collinearity Statistics Tolerance
1	RaceCC	-.221[b]	-2.310	.023	-.211	.870
	SES0	-.133[b]	-1.470	.144	-.136	.996
	MGRS0	-.079[b]	-.867	.388	-.081	.987
	MRN0	.076[b]	.714	.477	.066	.733
	MRN0xRace	-.030[b]	-.267	.790	-.025	.670
	MRN0xSES0	.044[b]	.472	.638	.044	.954
	MRN0xIdeology0	-.069[b]	-.718	.474	-.067	.910
	MRN0xMGRS0	-.083[b]	-.835	.405	-.078	.843
	MGRS0xRace	.047[b]	.513	.609	.048	.999
	MGRS0xSES0	.051[b]	.547	.585	.051	.960
	MGRS0xIdeology0	-.043[b]	-.435	.665	-.040	.861
2	MGRS0	-.095[c]	-1.067	.288	-.100	.980
	MRN0	.022[c]	.207	.836	.019	.668
	MRN0xRace	.017[c]	.157	.875	.015	.647
	MRN0xSES0	.100[c]	1.063	.290	.099	.888
	MRN0xIdeology0	-.060[c]	-.618	.538	-.058	.828
	MRN0xMGRS0	-.133[c]	-1.324	.188	-.124	.765
	MGRS0xRace	-.010[c]	-.102	.919	-.010	.881
	MGRS0xSES0	.017[c]	.153	.879	.014	.661
	MGRS0xIdeology0	-.056[c]	-.574	.567	-.054	.817
3	MRN0xRace	-.015[d]	-.123	.902	-.012	.510
	MRN0xSES0	.086[d]	.881	.380	.083	.818
	MRN0xIdeology0	-.079[d]	-.795	.428	-.075	.792
	MRN0xMGRS0	-.090[d]	-.706	.482	-.067	.489
	MGRS0xRace	.047[d]	.451	.653	.043	.732
	MGRS0xSES0	.010[d]	.090	.929	.009	.610
	MGRS0xIdeology0	-.038[d]	-.364	.716	-.035	.718

a. Dependent Variable: Risk_Help

b. Predictors in the Model: (Constant), Ideology0

c. Predictors in the Model: (Constant), Ideology0, SES0, RaceCC

d. Predictors in the Model: (Constant), Ideology0, SES0, RaceCC, MGRS0, MRN0

```
 FILTER OFF.
USE ALL.
EXECUTE.

***Simple Slopes***

DESCRIPTIVES VARIABLES=MRN MGRS SES
  /STATISTICS=MEAN STDDEV MIN MAX.
```

Descriptives

Notes

Output Created		15-DEC-2021 13:07:59
Comments		
Input	Data	C: \Users\njs5478\Dropbox\HM and COVID\0. Revise and Resubmit\2. R and R Data\Study 1b\Study1b_Data.sav
	Active Dataset	DataSet1
	Filter	<none>
	Weight	<none>
	Split File	<none>
	N of Rows in Working Data File	241
Missing Value Handling	Definition of Missing	User defined missing values are treated as missing.
	Cases Used	All non-missing data are used.
Syntax		DESCRIPTIVES VARIABLES=MRN MGRS SES /STATISTICS=MEAN STDDEV MIN MAX.
Resources	Processor Time	00:00:00.00
	Elapsed Time	00:00:00.00

Descriptive Statistics

	N	Minimum	Maximum	Mean	Std. Deviation
MRN	240	1.08	5.35	3.2638	.86575
MGRS	240	1.00	4.90	2.4581	.64339
Self Reported Socioeconomic Status	241	1	5	3.41	.807
Valid N (listwise)	239				

```
 COMPUTE MRN.High = MRN0 - .86575.
COMPUTE MRN.Low = MRN0 +.86575.

COMPUTE MGRS.High = MGRS0 - .64339.
COMPUTE MGRS.Low = MGRS0 + .64339.

COMPUTE SES.High = SES0 - 0.807.
COMPUTE SES.Low = SES0 + 0.807.

IF(RaceCC=1) Race.White=0.
IF(RaceCC=-1) Race.White=1.
IF(RaceCC=1) Race.Non=1.
IF(RaceCC=-1) Race.Non=0.

**MRN*Race on Personal Threat

UNIANOVA Concern_Tot WITH MRN0 Race.White GenderCC SES0 Party0
  /PRINT=ETASQ PARAMETER
  /DESIGN=MRN0 Race.White GenderCC SES0 Party0
  MRN0*Race.White MRN0*GenderCC MRN0*SES0 MRN0*Party0.
```

Univariate Analysis of Variance

Notes

Output Created		15-DEC-2021 13:08:00
Comments		
Input	Data	C:\Users\njs5478\Dropbox\HM and COVID\0. Revise and Resubmit\2. R and R Data\Study 1b\Study1b_Data.sav
	Active Dataset	DataSet1
	Filter	<none>
	Weight	<none>
	Split File	<none>
	N of Rows in Working Data File	241
Missing Value Handling	Definition of Missing	User-defined missing values are treated as missing.
	Cases Used	Statistics are based on all cases with valid data for all variables in the model.
Syntax		UNIANOVA Concern_Tot WITH MRN0 Race.White GenderCC SES0 Party0<br>/PRINT=ETASQ PARAMETER<br>/DESIGN=MRN0 Race.White GenderCC SES0 Party0<br>MRN0*Race.White MRN0*GenderCC MRN0*SES0 MRN0*Party0.
Resources	Processor Time	00:00:00.02
	Elapsed Time	00:00:00.02

Tests of Between-Subjects Effects

Dependent Variable: Concern_Tot

Source	Type III Sum of Squares	df	Mean Square	F	Sig.	Partial Eta Squared
Corrected Model	127.444[a]	9	14.160	9.763	.000	.278
Intercept	2378.046	1	2378.046	1639.590	.000	.878
MRN0	17.562	1	17.562	12.109	.001	.050
Race.White	2.691	1	2.691	1.855	.175	.008
GenderCC	4.495	1	4.495	3.099	.080	.013
SES0	.070	1	.070	.048	.826	.000
Party0	22.340	1	22.340	15.403	.000	.063
MRN0 * Race.White	9.101	1	9.101	6.275	.013	.027
MRN0 * GenderCC	.062	1	.062	.043	.836	.000
MRN0 * SES0	7.279	1	7.279	5.019	.026	.022
MRN0 * Party0	.499	1	.499	.344	.558	.002
Error	330.689	228	1.450			
Total	4937.361	238				
Corrected Total	458.133	237				

a. R Squared = .278 (Adjusted R Squared = .250)

Parameter Estimates

Dependent Variable: Concern_Tot

Parameter	B	Std. Error	t	Sig.	95% Confidence Interval	
					Lower Bound	Upper Bound
Intercept	4.227	.104	40.492	.000	4.021	4.433
MRN0	-.452	.130	-3.480	.001	-.707	-.196
Race.White	.262	.192	1.362	.175	-.117	.641
GenderCC	-.155	.088	-1.760	.080	-.329	.019
SES0	.022	.098	.220	.826	-.172	.215
Party0	-.268	.068	-3.925	.000	-.402	-.133
MRN0 * Race.White	.540	.216	2.505	.013	.115	.964
MRN0 * GenderCC	.022	.105	.208	.836	-.184	.228
MRN0 * SES0	.232	.103	2.240	.026	.028	.436
MRN0 * Party0	.041	.070	.587	.558	-.097	.179

Parameter Estimates

Dependent Variable: Concern_Tot

Parameter	Partial Eta Squared
Intercept	.878
MRN0	.050
Race.White	.008
GenderCC	.013
SES0	.000
Party0	.063
MRN0 * Race.White	.027
MRN0 * GenderCC	.000
MRN0 * SES0	.022
MRN0 * Party0	.002

```
UNIANOVA Concern_Tot WITH MRN0 Race.Non GenderCC SES0 Party0
 /PRINT=ETASQ PARAMETER
 /DESIGN=MRN0 Race.Non GenderCC SES0 Party0
 MRN0*Race.Non MRN0*GenderCC MRN0*SES0 MRN0*Party0.
```

Univariate Analysis of Variance

Notes

Output Created		15-DEC-2021 13:08:00
Comments		
Input	Data	C: \Users\njs5478\Dropbox\HM and COVID\0. Revise and Resubmit\2. R and R Data\Study 1b\Study1b_Data.sav
	Active Dataset	DataSet1
	Filter	<none>
	Weight	<none>
	Split File	<none>
	N of Rows in Working Data File	241
Missing Value Handling	Definition of Missing	User-defined missing values are treated as missing.
	Cases Used	Statistics are based on all cases with valid data for all variables in the model.
Syntax		UNIANOVA Concern_Tot WITH MRN0 Race.Non GenderCC SES0 Party0 /PRINT=ETASQ PARAMETER /DESIGN=MRN0 Race.Non GenderCC SES0 Party0 MRN0*Race.Non MRN0*GenderCC MRN0*SES0 MRN0*Party0.
Resources	Processor Time	00:00:00.00
	Elapsed Time	00:00:00.02

Tests of Between-Subjects Effects

Dependent Variable: Concern_Tot

Source	Type III Sum of Squares	df	Mean Square	F	Sig.	Partial Eta Squared
Corrected Model	127.444[a]	9	14.160	9.763	.000	.278
Intercept	986.332	1	986.332	680.046	.000	.749
MRN0	.303	1	.303	.209	.648	.001
Race.Non	2.691	1	2.691	1.855	.175	.008
GenderCC	4.495	1	4.495	3.099	.080	.013
SES0	.070	1	.070	.048	.826	.000
Party0	22.340	1	22.340	15.403	.000	.063
MRN0 * Race.Non	9.101	1	9.101	6.275	.013	.027
MRN0 * GenderCC	.062	1	.062	.043	.836	.000
MRN0 * SES0	7.279	1	7.279	5.019	.026	.022
MRN0 * Party0	.499	1	.499	.344	.558	.002
Error	330.689	228	1.450			
Total	4937.361	238				
Corrected Total	458.133	237				

a. R Squared = .278 (Adjusted R Squared = .250)

Parameter Estimates

Dependent Variable: Concern_Tot

Parameter	B	Std. Error	t	Sig.	95% Confidence Interval	
					Lower Bound	Upper Bound
Intercept	4.489	.172	26.078	.000	4.150	4.828
MRN0	.088	.193	.457	.648	-.292	.468
Race.Non	-.262	.192	-1.362	.175	-.641	.117
GenderCC	-.155	.088	-1.760	.080	-.329	.019
SES0	.022	.098	.220	.826	-.172	.215
Party0	-.268	.068	-3.925	.000	-.402	-.133
MRN0 * Race.Non	-.540	.216	-2.505	.013	-.964	-.115
MRN0 * GenderCC	.022	.105	.208	.836	-.184	.228
MRN0 * SES0	.232	.103	2.240	.026	.028	.436
MRN0 * Party0	.041	.070	.587	.558	-.097	.179

Parameter Estimates

Dependent Variable: Concern_Tot

Parameter	Partial Eta Squared
Intercept	.749
MRN0	.001
Race.Non	.008
GenderCC	.013
SES0	.000
Party0	.063
MRN0 * Race.Non	.027
MRN0 * GenderCC	.000
MRN0 * SES0	.022
MRN0 * Party0	.002

```
UNIANOVA Concern_Tot WITH MRN.Low RaceCC GenderCC SES0 Party0
 /PRINT=ETASQ PARAMETER
 /DESIGN=MRN.Low RaceCC GenderCC SES0 Party0
 MRN.Low*RaceCC MRN.Low*GenderCC MRN.Low*SES0 MRN.Low*Party0.
```

Univariate Analysis of Variance

Notes

Output Created		15-DEC-2021 13:08:00
Comments		
Input	Data	C:\Users\njs5478\Dropbox\HM and COVID\0. Revise and Resubmit\2. R and R Data\Study 1b\Study1b_Data.sav
	Active Dataset	DataSet1
	Filter	<none>
	Weight	<none>
	Split File	<none>
	N of Rows in Working Data File	241
Missing Value Handling	Definition of Missing	User-defined missing values are treated as missing.
	Cases Used	Statistics are based on all cases with valid data for all variables in the model.
Syntax		UNIANOVA Concern_Tot WITH MRN.Low RaceCC GenderCC SES0 Party0 /PRINT=ETASQ PARAMETER /DESIGN=MRN.Low RaceCC GenderCC SES0 Party0 MRN.Low*RaceCC MRN.Low*GenderCC MRN.Low*SES0 MRN.Low*Party0.
Resources	Processor Time	00:00:00.02
	Elapsed Time	00:00:00.02

Tests of Between-Subjects Effects

Dependent Variable: Concern_Tot

Source	Type III Sum of Squares	df	Mean Square	F	Sig.
Corrected Model	127.444[a]	9	14.160	9.763	.000
Intercept	1257.429	1	1257.429	866.959	.000
MRN.Low	3.111	1	3.111	2.145	.144
RaceCC	.970	1	.970	.669	.414
GenderCC	3.059	1	3.059	2.109	.148
SES0	2.569	1	2.569	1.772	.185
Party0	14.487	1	14.487	9.988	.002
MRN.Low * RaceCC	9.101	1	9.101	6.275	.013
MRN.Low * GenderCC	.062	1	.062	.043	.836
MRN.Low * SES0	7.279	1	7.279	5.019	.026
MRN.Low * Party0	.499	1	.499	.344	.558
Error	330.689	228	1.450		
Total	4937.361	238			
Corrected Total	458.133	237			

Tests of Between-Subjects Effects

Dependent Variable: Concern_Tot

Source	Partial Eta Squared
Corrected Model	.278
Intercept	.792
MRN.Low	.009
RaceCC	.003
GenderCC	.009
SES0	.008
Party0	.042
MRN.Low * RaceCC	.027
MRN.Low * GenderCC	.000
MRN.Low * SES0	.022
MRN.Low * Party0	.002
Error	
Total	
Corrected Total	

a. R Squared = .278 (Adjusted R Squared = .250)

Parameter Estimates

Dependent Variable: Concern_Tot

Parameter	B	Std. Error	t	Sig.	95% Confidence Interval	
					Lower Bound	Upper Bound
Intercept	4.516	.153	29.444	.000	4.213	4.818
MRN.Low	-.182	.124	-1.464	.144	-.426	.063
RaceCC	.103	.125	.818	.414	-.145	.350
GenderCC	-.174	.120	-1.452	.148	-.411	.062
SES0	-.179	.135	-1.331	.185	-.444	.086
Party0	-.304	.096	-3.160	.002	-.493	-.114
MRN.Low * RaceCC	-.270	.108	-2.505	.013	-.482	-.058
MRN.Low * GenderCC	.022	.105	.208	.836	-.184	.228
MRN.Low * SES0	.232	.103	2.240	.026	.028	.436
MRN.Low * Party0	.041	.070	.587	.558	-.097	.179

Parameter Estimates

Dependent Variable: Concern_Tot

Parameter	Partial Eta Squared
Intercept	.792
MRN.Low	.009
RaceCC	.003
GenderCC	.009
SES0	.008
Party0	.042
MRN.Low * RaceCC	.027
MRN.Low * GenderCC	.000
MRN.Low * SES0	.022
MRN.Low * Party0	.002

```
UNIANOVA Concern_Tot WITH MRN.High RaceCC GenderCC SES0 Party0
 /PRINT=ETASQ PARAMETER
 /DESIGN=MRN.High RaceCC GenderCC SES0 Party0
 MRN.High*RaceCC MRN.High*GenderCC MRN.High*SES0 MRN.High*Party0.
```

Univariate Analysis of Variance

Notes

Output Created		15-DEC-2021 13:08:00
Comments		
Input	Data	C:\Users\njs5478\Dropbox\HM and COVID\0. Revise and Resubmit\2. R and R Data\Study 1b\Study1b_Data.sav
	Active Dataset	DataSet1
	Filter	<none>
	Weight	<none>
	Split File	<none>
	N of Rows in Working Data File	241
Missing Value Handling	Definition of Missing	User-defined missing values are treated as missing.
	Cases Used	Statistics are based on all cases with valid data for all variables in the model.
Syntax		UNIANOVA Concern_Tot WITH MRN.High RaceCC GenderCC SES0 Party0 /PRINT=ETASQ PARAMETER /DESIGN=MRN.High RaceCC GenderCC SES0 Party0 MRN.High*RaceCC MRN.High*GenderCC MRN.High*SES0 MRN.High*Party0.
Resources	Processor Time	00:00:00.00
	Elapsed Time	00:00:00.02

Tests of Between-Subjects Effects

Dependent Variable: Concern_Tot

Source	Type III Sum of Squares	df	Mean Square	F	Sig.
Corrected Model	127.444[a]	9	14.160	9.763	.000
Intercept	1185.548	1	1185.548	817.399	.000
MRN.High	3.111	1	3.111	2.145	.144
RaceCC	9.566	1	9.566	6.595	.011
GenderCC	1.541	1	1.541	1.063	.304
SES0	4.150	1	4.150	2.862	.092
Party0	10.514	1	10.514	7.249	.008
MRN.High * RaceCC	9.101	1	9.101	6.275	.013
MRN.High * GenderCC	.062	1	.062	.043	.836
MRN.High * SES0	7.279	1	7.279	5.019	.026
MRN.High * Party0	.499	1	.499	.344	.558
Error	330.689	228	1.450		
Total	4937.361	238			
Corrected Total	458.133	237			

Tests of Between-Subjects Effects

Dependent Variable: Concern_Tot

Source	Partial Eta Squared
Corrected Model	.278
Intercept	.782
MRN.High	.009
RaceCC	.028
GenderCC	.005
SES0	.012
Party0	.031
MRN.High * RaceCC	.027
MRN.High * GenderCC	.000
MRN.High * SES0	.022
MRN.High * Party0	.002
Error	
Total	
Corrected Total	

a. R Squared = .278 (Adjusted R Squared = .250)

Parameter Estimates

Dependent Variable: Concern_Tot

Parameter	B	Std. Error	t	Sig.	95% Confidence Interval	
					Lower Bound	Upper Bound
Intercept	4.201	.147	28.590	.000	3.911	4.490
MRN.High	-.182	.124	-1.464	.144	-.426	.063
RaceCC	-.365	.142	-2.568	.011	-.645	-.085
GenderCC	-.137	.133	-1.031	.304	-.398	.125
SES0	.222	.131	1.692	.092	-.037	.481
Party0	-.232	.086	-2.692	.008	-.402	-.062
MRN.High * RaceCC	-.270	.108	-2.505	.013	-.482	-.058
MRN.High * GenderCC	.022	.105	.208	.836	-.184	.228
MRN.High * SES0	.232	.103	2.240	.026	.028	.436
MRN.High * Party0	.041	.070	.587	.558	-.097	.179

Parameter Estimates

Dependent Variable: Concern_Tot

Parameter	Partial Eta Squared
Intercept	.782
MRN.High	.009
RaceCC	.028
GenderCC	.005
SES0	.012
Party0	.031
MRN.High * RaceCC	.027
MRN.High * GenderCC	.000
MRN.High * SES0	.022
MRN.High * Party0	.002

```
**MRN*SES on Personal Threat

UNIANOVA Concern_Tot WITH MRN0 RaceCC GenderCC SES.Low Party0
  /PRINT=ETASQ PARAMETER
  /DESIGN=MRN0 RaceCC GenderCC SES.Low Party0
  MRN0*RaceCC MRN0*GenderCC MRN0*SES.Low MRN0*Party0.
```

Univariate Analysis of Variance

Notes

Output Created		15-DEC-2021 13:08:00
Comments		
Input	Data	C:\Users\njs5478\Dropbox\HM and COVID\0. Revise and Resubmit\2. R and R Data\Study 1b\Study1b_Data.sav
	Active Dataset	DataSet1
	Filter	<none>
	Weight	<none>
	Split File	<none>
	N of Rows in Working Data File	241
Missing Value Handling	Definition of Missing	User-defined missing values are treated as missing.
	Cases Used	Statistics are based on all cases with valid data for all variables in the model.
Syntax		UNIANOVA Concern_Tot WITH MRN0 RaceCC GenderCC SES.Low Party0<br>/PRINT=ETASQ PARAMETER<br>/DESIGN=MRN0 RaceCC GenderCC SES.Low Party0<br>MRN0*RaceCC MRN0*GenderCC MRN0*SES.Low MRN0*Party0.
Resources	Processor Time	00:00:00.02
	Elapsed Time	00:00:00.02

Tests of Between-Subjects Effects

Dependent Variable: Concern_Tot

Source	Type III Sum of Squares	df	Mean Square	F	Sig.	Partial Eta Squared
Corrected Model	127.444[a]	9	14.160	9.763	.000	.278
Intercept	1589.300	1	1589.300	1095.774	.000	.828
MRN0	10.163	1	10.163	7.007	.009	.030
RaceCC	2.691	1	2.691	1.855	.175	.008
GenderCC	4.495	1	4.495	3.099	.080	.013
SES.Low	.070	1	.070	.048	.826	.000
Party0	22.340	1	22.340	15.403	.000	.063
MRN0 * RaceCC	9.101	1	9.101	6.275	.013	.027
MRN0 * GenderCC	.062	1	.062	.043	.836	.000
MRN0 * SES.Low	7.279	1	7.279	5.019	.026	.022
MRN0 * Party0	.499	1	.499	.344	.558	.002
Error	330.689	228	1.450			
Total	4937.361	238				
Corrected Total	458.133	237				

a. R Squared = .278 (Adjusted R Squared = .250)

Parameter Estimates

Dependent Variable: Concern_Tot

Parameter	B	Std. Error	t	Sig.	95% Confidence Interval	
					Lower Bound	Upper Bound
Intercept	4.341	.131	33.102	.000	4.082	4.599
MRN0	-.369	.139	-2.647	.009	-.643	-.094
RaceCC	-.131	.096	-1.362	.175	-.321	.059
GenderCC	-.155	.088	-1.760	.080	-.329	.019
SES.Low	.022	.098	.220	.826	-.172	.215
Party0	-.268	.068	-3.925	.000	-.402	-.133
MRN0 * RaceCC	-.270	.108	-2.505	.013	-.482	-.058
MRN0 * GenderCC	.022	.105	.208	.836	-.184	.228
MRN0 * SES.Low	.232	.103	2.240	.026	.028	.436
MRN0 * Party0	.041	.070	.587	.558	-.097	.179

Parameter Estimates

Dependent Variable: Concern_Tot

Parameter	Partial Eta Squared
Intercept	.828
MRN0	.030
RaceCC	.008
GenderCC	.013
SES.Low	.000
Party0	.063
MRN0 * RaceCC	.027
MRN0 * GenderCC	.000
MRN0 * SES.Low	.022
MRN0 * Party0	.002

```
UNIANOVA Concern_Tot WITH MRN0 RaceCC GenderCC SES.High Party0
 /PRINT=ETASQ PARAMETER
 /DESIGN=MRN0 RaceCC GenderCC SES.High Party0
 MRN0*RaceCC MRN0*GenderCC MRN0*SES.High MRN0*Party0.
```

Univariate Analysis of Variance

Notes

Output Created		15-DEC-2021 13:08:00
Comments		
Input	Data	C:\Users\njs5478\Dropbox\HM and COVID\0. Revise and Resubmit\2. R and R Data\Study 1b\Study1b_Data.sav
	Active Dataset	DataSet1
	Filter	<none>
	Weight	<none>
	Split File	<none>
	N of Rows in Working Data File	241
Missing Value Handling	Definition of Missing	User-defined missing values are treated as missing.
	Cases Used	Statistics are based on all cases with valid data for all variables in the model.
Syntax		UNIANOVA Concern_Tot WITH MRN0 RaceCC GenderCC SES.High Party0<br>/PRINT=ETASQ PARAMETER<br>/DESIGN=MRN0 RaceCC GenderCC SES.High Party0<br>MRN0*RaceCC MRN0*GenderCC MRN0*SES.High MRN0*Party0.
Resources	Processor Time	00:00:00.00
	Elapsed Time	00:00:00.02

Tests of Between-Subjects Effects

Dependent Variable: Concern_Tot

Source	Type III Sum of Squares	df	Mean Square	F	Sig.	Partial Eta Squared
Corrected Model	127.444[a]	9	14.160	9.763	.000	.278
Intercept	1594.812	1	1594.812	1099.574	.000	.828
MRN0	.002	1	.002	.001	.973	.000
RaceCC	2.691	1	2.691	1.855	.175	.008
GenderCC	4.495	1	4.495	3.099	.080	.013
SES.High	.070	1	.070	.048	.826	.000
Party0	22.340	1	22.340	15.403	.000	.063
MRN0 * RaceCC	9.101	1	9.101	6.275	.013	.027
MRN0 * GenderCC	.062	1	.062	.043	.836	.000
MRN0 * SES.High	7.279	1	7.279	5.019	.026	.022
MRN0 * Party0	.499	1	.499	.344	.558	.002
Error	330.689	228	1.450			
Total	4937.361	238				
Corrected Total	458.133	237				

a. R Squared = .278 (Adjusted R Squared = .250)

Parameter Estimates

Dependent Variable: Concern_Tot

Parameter	B	Std. Error	t	Sig.	95% Confidence Interval	
					Lower Bound	Upper Bound
Intercept	4.376	.132	33.160	.000	4.116	4.636
MRN0	.005	.159	.034	.973	-.308	.319
RaceCC	-.131	.096	-1.362	.175	-.321	.059
GenderCC	-.155	.088	-1.760	.080	-.329	.019
SES.High	.022	.098	.220	.826	-.172	.215
Party0	-.268	.068	-3.925	.000	-.402	-.133
MRN0 * RaceCC	-.270	.108	-2.505	.013	-.482	-.058
MRN0 * GenderCC	.022	.105	.208	.836	-.184	.228
MRN0 * SES.High	.232	.103	2.240	.026	.028	.436
MRN0 * Party0	.041	.070	.587	.558	-.097	.179

Parameter Estimates

Dependent Variable: Concern_Tot

Parameter	Partial Eta Squared
Intercept	.828
MRN0	.000
RaceCC	.008
GenderCC	.013
SES.High	.000
Party0	.063
MRN0 * RaceCC	.027
MRN0 * GenderCC	.000
MRN0 * SES.High	.022
MRN0 * Party0	.002

```
UNIANOVA Concern_Tot WITH MRN.Low RaceCC GenderCC SES0 Party0
 /PRINT=ETASQ PARAMETER
 /DESIGN=MRN.Low RaceCC GenderCC SES0 Party0
 MRN.Low*RaceCC MRN.Low*GenderCC MRN.Low*SES0 MRN.Low*Party0.
```

Univariate Analysis of Variance

Notes

Output Created		15-DEC-2021 13:08:00
Comments		
Input	Data	C:\Users\njs5478\Dropbox\HM and COVID\0. Revise and Resubmit\2. R and R Data\Study 1b\Study1b_Data.sav
	Active Dataset	DataSet1
	Filter	<none>
	Weight	<none>
	Split File	<none>
	N of Rows in Working Data File	241
Missing Value Handling	Definition of Missing	User-defined missing values are treated as missing.
	Cases Used	Statistics are based on all cases with valid data for all variables in the model.
Syntax		UNIANOVA Concern_Tot WITH MRN.Low RaceCC GenderCC SES0 Party0 /PRINT=ETASQ PARAMETER /DESIGN=MRN.Low RaceCC GenderCC SES0 Party0 MRN.Low*RaceCC MRN.Low*GenderCC MRN.Low*SES0 MRN.Low*Party0.
Resources	Processor Time	00:00:00.02
	Elapsed Time	00:00:00.02

Tests of Between-Subjects Effects

Dependent Variable: Concern_Tot

Source	Type III Sum of Squares	df	Mean Square	F	Sig.
Corrected Model	127.444[a]	9	14.160	9.763	.000
Intercept	1257.429	1	1257.429	866.959	.000
MRN.Low	3.111	1	3.111	2.145	.144
RaceCC	.970	1	.970	.669	.414
GenderCC	3.059	1	3.059	2.109	.148
SES0	2.569	1	2.569	1.772	.185
Party0	14.487	1	14.487	9.988	.002
MRN.Low * RaceCC	9.101	1	9.101	6.275	.013
MRN.Low * GenderCC	.062	1	.062	.043	.836
MRN.Low * SES0	7.279	1	7.279	5.019	.026
MRN.Low * Party0	.499	1	.499	.344	.558
Error	330.689	228	1.450		
Total	4937.361	238			
Corrected Total	458.133	237			

Tests of Between-Subjects Effects

Dependent Variable: Concern_Tot

Source	Partial Eta Squared
Corrected Model	.278
Intercept	.792
MRN.Low	.009
RaceCC	.003
GenderCC	.009
SES0	.008
Party0	.042
MRN.Low * RaceCC	.027
MRN.Low * GenderCC	.000
MRN.Low * SES0	.022
MRN.Low * Party0	.002
Error	
Total	
Corrected Total	

a. R Squared = .278 (Adjusted R Squared = .250)

Parameter Estimates

Dependent Variable: Concern_Tot

Parameter	B	Std. Error	t	Sig.	95% Confidence Interval Lower Bound	95% Confidence Interval Upper Bound
Intercept	4.516	.153	29.444	.000	4.213	4.818
MRN.Low	-.182	.124	-1.464	.144	-.426	.063
RaceCC	.103	.125	.818	.414	-.145	.350
GenderCC	-.174	.120	-1.452	.148	-.411	.062
SES0	-.179	.135	-1.331	.185	-.444	.086
Party0	-.304	.096	-3.160	.002	-.493	-.114
MRN.Low * RaceCC	-.270	.108	-2.505	.013	-.482	-.058
MRN.Low * GenderCC	.022	.105	.208	.836	-.184	.228
MRN.Low * SES0	.232	.103	2.240	.026	.028	.436
MRN.Low * Party0	.041	.070	.587	.558	-.097	.179

Parameter Estimates

Dependent Variable: Concern_Tot

Parameter	Partial Eta Squared
Intercept	.792
MRN.Low	.009
RaceCC	.003
GenderCC	.009
SES0	.008
Party0	.042
MRN.Low * RaceCC	.027
MRN.Low * GenderCC	.000
MRN.Low * SES0	.022
MRN.Low * Party0	.002

```
UNIANOVA Concern_Tot WITH MRN.High RaceCC GenderCC SES0 Party0
 /PRINT=ETASQ PARAMETER
 /DESIGN=MRN.High RaceCC GenderCC SES0 Party0
 MRN.High*RaceCC MRN.High*GenderCC MRN.High*SES0 MRN.High*Party0.
```

Univariate Analysis of Variance

Notes

Output Created		15-DEC-2021 13:08:00
Comments		
Input	Data	C:\Users\njs5478\Dropbox\HM and COVID\0. Revise and Resubmit\2. R and R Data\Study 1b\Study1b_Data.sav
	Active Dataset	DataSet1
	Filter	<none>
	Weight	<none>
	Split File	<none>
	N of Rows in Working Data File	241
Missing Value Handling	Definition of Missing	User-defined missing values are treated as missing.
	Cases Used	Statistics are based on all cases with valid data for all variables in the model.
Syntax		UNIANOVA Concern_Tot WITH MRN.High RaceCC GenderCC SES0 Party0 /PRINT=ETASQ PARAMETER /DESIGN=MRN.High RaceCC GenderCC SES0 Party0 MRN.High*RaceCC MRN.High*GenderCC MRN.High*SES0 MRN.High*Party0.
Resources	Processor Time	00:00:00.02
	Elapsed Time	00:00:00.02

Tests of Between-Subjects Effects

Dependent Variable: Concern_Tot

Source	Type III Sum of Squares	df	Mean Square	F	Sig.
Corrected Model	127.444[a]	9	14.160	9.763	.000
Intercept	1185.548	1	1185.548	817.399	.000
MRN.High	3.111	1	3.111	2.145	.144
RaceCC	9.566	1	9.566	6.595	.011
GenderCC	1.541	1	1.541	1.063	.304
SES0	4.150	1	4.150	2.862	.092
Party0	10.514	1	10.514	7.249	.008
MRN.High * RaceCC	9.101	1	9.101	6.275	.013
MRN.High * GenderCC	.062	1	.062	.043	.836
MRN.High * SES0	7.279	1	7.279	5.019	.026
MRN.High * Party0	.499	1	.499	.344	.558
Error	330.689	228	1.450		
Total	4937.361	238			
Corrected Total	458.133	237			

Tests of Between-Subjects Effects

Dependent Variable: Concern_Tot

Source	Partial Eta Squared
Corrected Model	.278
Intercept	.782
MRN.High	.009
RaceCC	.028
GenderCC	.005
SES0	.012
Party0	.031
MRN.High * RaceCC	.027
MRN.High * GenderCC	.000
MRN.High * SES0	.022
MRN.High * Party0	.002
Error	
Total	
Corrected Total	

a. R Squared = .278 (Adjusted R Squared = .250)

Parameter Estimates

Dependent Variable: Concern_Tot

Parameter	B	Std. Error	t	Sig.	95% Confidence Interval	
					Lower Bound	Upper Bound
Intercept	4.201	.147	28.590	.000	3.911	4.490
MRN.High	-.182	.124	-1.464	.144	-.426	.063
RaceCC	-.365	.142	-2.568	.011	-.645	-.085
GenderCC	-.137	.133	-1.031	.304	-.398	.125
SES0	.222	.131	1.692	.092	-.037	.481
Party0	-.232	.086	-2.692	.008	-.402	-.062
MRN.High * RaceCC	-.270	.108	-2.505	.013	-.482	-.058
MRN.High * GenderCC	.022	.105	.208	.836	-.184	.228
MRN.High * SES0	.232	.103	2.240	.026	.028	.436
MRN.High * Party0	.041	.070	.587	.558	-.097	.179

Parameter Estimates

Dependent Variable: Concern_Tot

Parameter	Partial Eta Squared
Intercept	.782
MRN.High	.009
RaceCC	.028
GenderCC	.005
SES0	.012
Party0	.031
MRN.High * RaceCC	.027
MRN.High * GenderCC	.000
MRN.High * SES0	.022
MRN.High * Party0	.002

```
**MRN*SES on Financial

UNIANOVA Finance_Tot WITH MRN0 RaceCC GenderCC SES.Low Party0
  /PRINT=ETASQ PARAMETER
  /DESIGN=MRN0 RaceCC GenderCC SES.Low Party0
  MRN0*RaceCC MRN0*GenderCC MRN0*SES.Low MRN0*Party0.
```

Univariate Analysis of Variance

Notes

Output Created		15-DEC-2021 13:08:00
Comments		
Input	Data	C:\Users\njs5478\Dropbox\HM and COVID\0. Revise and Resubmit\2. R and R Data\Study 1b\Study1b_Data.sav
	Active Dataset	DataSet1
	Filter	<none>
	Weight	<none>
	Split File	<none>
	N of Rows in Working Data File	241
Missing Value Handling	Definition of Missing	User-defined missing values are treated as missing.
	Cases Used	Statistics are based on all cases with valid data for all variables in the model.
Syntax		UNIANOVA Finance_Tot WITH MRN0 RaceCC GenderCC SES.Low Party0<br>/PRINT=ETASQ PARAMETER<br>/DESIGN=MRN0 RaceCC GenderCC SES.Low Party0<br>MRN0*RaceCC MRN0*GenderCC MRN0*SES.Low MRN0*Party0.
Resources	Processor Time	00:00:00.00
	Elapsed Time	00:00:00.02

Tests of Between-Subjects Effects

Dependent Variable: Finance_Tot

Source	Type III Sum of Squares	df	Mean Square	F	Sig.	Partial Eta Squared
Corrected Model	52.679[a]	9	5.853	2.641	.006	.094
Intercept	1338.762	1	1338.762	604.071	.000	.726
MRN0	9.139	1	9.139	4.123	.043	.018
RaceCC	.181	1	.181	.082	.775	.000
GenderCC	6.064	1	6.064	2.736	.099	.012
SES.Low	8.244	1	8.244	3.720	.055	.016
Party0	.945	1	.945	.427	.514	.002
MRN0 * RaceCC	1.433	1	1.433	.647	.422	.003
MRN0 * GenderCC	3.603	1	3.603	1.626	.204	.007
MRN0 * SES.Low	18.494	1	18.494	8.345	.004	.035
MRN0 * Party0	.917	1	.917	.414	.521	.002
Error	505.301	228	2.216			
Total	4179.000	238				
Corrected Total	557.980	237				

a. R Squared = .094 (Adjusted R Squared = .059)

Parameter Estimates

Dependent Variable: Finance_Tot

Parameter	B	Std. Error	t	Sig.	95% Confidence Interval	
					Lower Bound	Upper Bound
Intercept	3.984	.162	24.578	.000	3.665	4.303
MRN0	-.350	.172	-2.031	.043	-.689	-.010
RaceCC	-.034	.119	-.286	.775	-.268	.200
GenderCC	-.181	.109	-1.654	.099	-.396	.035
SES.Low	-.234	.122	-1.929	.055	-.474	.005
Party0	-.055	.084	-.653	.514	-.221	.111
MRN0 * RaceCC	-.107	.133	-.804	.422	-.370	.155
MRN0 * GenderCC	.165	.129	1.275	.204	-.090	.419
MRN0 * SES.Low	.370	.128	2.889	.004	.117	.622
MRN0 * Party0	.056	.087	.643	.521	-.115	.226

Parameter Estimates

Dependent Variable: Finance_Tot

Parameter	Partial Eta Squared
Intercept	.726
MRN0	.018
RaceCC	.000
GenderCC	.012
SES.Low	.016
Party0	.002
MRN0 * RaceCC	.003
MRN0 * GenderCC	.007
MRN0 * SES.Low	.035
MRN0 * Party0	.002

```
UNIANOVA Finance_Tot WITH MRN0 RaceCC GenderCC SES.High Party0
 /PRINT=ETASQ PARAMETER
 /DESIGN=MRN0 RaceCC GenderCC SES.High Party0
 MRN0*RaceCC MRN0*GenderCC MRN0*SES.High MRN0*Party0.
```

Univariate Analysis of Variance

Notes

Output Created		15-DEC-2021 13:08:00
Comments		
Input	Data	C:\Users\njs5478\Dropbox\HM and COVID\0. Revise and Resubmit\2. R and R Data\Study 1b\Study1b_Data.sav
	Active Dataset	DataSet1
	Filter	<none>
	Weight	<none>
	Split File	<none>
	N of Rows in Working Data File	241
Missing Value Handling	Definition of Missing	User-defined missing values are treated as missing.
	Cases Used	Statistics are based on all cases with valid data for all variables in the model.
Syntax		UNIANOVA Finance_Tot WITH MRN0 RaceCC GenderCC SES.High Party0<br>/PRINT=ETASQ PARAMETER<br>/DESIGN=MRN0 RaceCC GenderCC SES.High Party0<br>MRN0*RaceCC MRN0*GenderCC MRN0*SES.High MRN0*Party0.
Resources	Processor Time	00:00:00.02
	Elapsed Time	00:00:00.03

Tests of Between-Subjects Effects

Dependent Variable: Finance_Tot

Source	Type III Sum of Squares	df	Mean Square	F	Sig.	Partial Eta Squared
Corrected Model	52.679[a]	9	5.853	2.641	.006	.094
Intercept	1082.909	1	1082.909	488.626	.000	.682
MRN0	3.484	1	3.484	1.572	.211	.007
RaceCC	.181	1	.181	.082	.775	.000
GenderCC	6.064	1	6.064	2.736	.099	.012
SES.High	8.244	1	8.244	3.720	.055	.016
Party0	.945	1	.945	.427	.514	.002
MRN0 * RaceCC	1.433	1	1.433	.647	.422	.003
MRN0 * GenderCC	3.603	1	3.603	1.626	.204	.007
MRN0 * SES.High	18.494	1	18.494	8.345	.004	.035
MRN0 * Party0	.917	1	.917	.414	.521	.002
Error	505.301	228	2.216			
Total	4179.000	238				
Corrected Total	557.980	237				

a. R Squared = .094 (Adjusted R Squared = .059)

Parameter Estimates

Dependent Variable: Finance_Tot

Parameter	B	Std. Error	t	Sig.	95% Confidence Interval	
					Lower Bound	Upper Bound
Intercept	3.606	.163	22.105	.000	3.284	3.927
MRN0	.247	.197	1.254	.211	-.141	.634
RaceCC	-.034	.119	-.286	.775	-.268	.200
GenderCC	-.181	.109	-1.654	.099	-.396	.035
SES.High	-.234	.122	-1.929	.055	-.474	.005
Party0	-.055	.084	-.653	.514	-.221	.111
MRN0 * RaceCC	-.107	.133	-.804	.422	-.370	.155
MRN0 * GenderCC	.165	.129	1.275	.204	-.090	.419
MRN0 * SES.High	.370	.128	2.889	.004	.117	.622
MRN0 * Party0	.056	.087	.643	.521	-.115	.226

Parameter Estimates

Dependent Variable: Finance_Tot

Parameter	Partial Eta Squared
Intercept	.682
MRN0	.007
RaceCC	.000
GenderCC	.012
SES.High	.016
Party0	.002
MRN0 * RaceCC	.003
MRN0 * GenderCC	.007
MRN0 * SES.High	.035
MRN0 * Party0	.002

```
UNIANOVA Finance_Tot WITH MRN.Low RaceCC GenderCC SES0 Party0
 /PRINT=ETASQ PARAMETER
 /DESIGN=MRN.Low RaceCC GenderCC SES0 Party0
 MRN.Low*RaceCC MRN.Low*GenderCC MRN.Low*SES0 MRN.Low*Party0.
```

Univariate Analysis of Variance

Notes

Output Created		15-DEC-2021 13:08:00
Comments		
Input	Data	C: \Users\njs5478\Dropbox\HM and COVID\0. Revise and Resubmit\2. R and R Data\Study 1b\Study1b_Data.sav
	Active Dataset	DataSet1
	Filter	<none>
	Weight	<none>
	Split File	<none>
	N of Rows in Working Data File	241
Missing Value Handling	Definition of Missing	User-defined missing values are treated as missing.
	Cases Used	Statistics are based on all cases with valid data for all variables in the model.
Syntax		UNIANOVA Finance_Tot WITH MRN.Low RaceCC GenderCC SES0 Party0 /PRINT=ETASQ PARAMETER /DESIGN=MRN.Low RaceCC GenderCC SES0 Party0 MRN.Low*RaceCC MRN.Low*GenderCC MRN.Low*SES0 MRN.Low*Party0.
Resources	Processor Time	00:00:00.00
	Elapsed Time	00:00:00.02

Tests of Between-Subjects Effects

Dependent Variable: Finance_Tot

Source	Type III Sum of Squares	df	Mean Square	F	Sig.
Corrected Model	52.679[a]	9	5.853	2.641	.006
Intercept	909.060	1	909.060	410.182	.000
MRN.Low	.250	1	.250	.113	.737
RaceCC	.317	1	.317	.143	.705
GenderCC	10.524	1	10.524	4.748	.030
SES0	24.610	1	24.610	11.105	.001
Party0	1.679	1	1.679	.758	.385
MRN.Low * RaceCC	1.433	1	1.433	.647	.422
MRN.Low * GenderCC	3.603	1	3.603	1.626	.204
MRN.Low * SES0	18.494	1	18.494	8.345	.004
MRN.Low * Party0	.917	1	.917	.414	.521
Error	505.301	228	2.216		
Total	4179.000	238			
Corrected Total	557.980	237			

Tests of Between-Subjects Effects

Dependent Variable: Finance_Tot

Source	Partial Eta Squared
Corrected Model	.094
Intercept	.643
MRN.Low	.000
RaceCC	.001
GenderCC	.020
SES0	.046
Party0	.003
MRN.Low * RaceCC	.003
MRN.Low * GenderCC	.007
MRN.Low * SES0	.035
MRN.Low * Party0	.002
Error	
Total	
Corrected Total	

a. R Squared = .094 (Adjusted R Squared = .059)

Parameter Estimates

Dependent Variable: Finance_Tot

Parameter	B	Std. Error	t	Sig.	95% Confidence Interval Lower Bound	95% Confidence Interval Upper Bound
Intercept	3.839	.190	20.253	.000	3.466	4.213
MRN.Low	-.052	.153	-.336	.737	-.354	.251
RaceCC	.059	.155	.378	.705	-.247	.364
GenderCC	-.323	.148	-2.179	.030	-.615	-.031
SES0	-.554	.166	-3.332	.001	-.882	-.227
Party0	-.103	.119	-.870	.385	-.337	.131
MRN.Low * RaceCC	-.107	.133	-.804	.422	-.370	.155
MRN.Low * GenderCC	.165	.129	1.275	.204	-.090	.419
MRN.Low * SES0	.370	.128	2.889	.004	.117	.622
MRN.Low * Party0	.056	.087	.643	.521	-.115	.226

Parameter Estimates

Dependent Variable: Finance_Tot

Parameter	Partial Eta Squared
Intercept	.643
MRN.Low	.000
RaceCC	.001
GenderCC	.020
SES0	.046
Party0	.003
MRN.Low * RaceCC	.003
MRN.Low * GenderCC	.007
MRN.Low * SES0	.035
MRN.Low * Party0	.002

```
UNIANOVA Finance_Tot WITH MRN.High RaceCC GenderCC SES0 Party0
 /PRINT=ETASQ PARAMETER
 /DESIGN=MRN.High RaceCC GenderCC SES0 Party0
 MRN.High*RaceCC MRN.High*GenderCC MRN.High*SES0 MRN.High*Party0.
```

Univariate Analysis of Variance

Notes

Output Created		15-DEC-2021 13:08:00
Comments		
Input	Data	C:<br>\Users\njs5478\Dropbox\HM and COVID\0. Revise and Resubmit\2. R and R Data\Study 1b\Study1b_Data.sav
	Active Dataset	DataSet1
	Filter	<none>
	Weight	<none>
	Split File	<none>
	N of Rows in Working Data File	241
Missing Value Handling	Definition of Missing	User-defined missing values are treated as missing.
	Cases Used	Statistics are based on all cases with valid data for all variables in the model.
Syntax		UNIANOVA Finance_Tot WITH MRN.High RaceCC GenderCC SES0 Party0<br>/PRINT=ETASQ PARAMETER<br>/DESIGN=MRN.High RaceCC GenderCC SES0 Party0<br>MRN.High*RaceCC MRN.High*GenderCC MRN.High*SES0 MRN.High*Party0.
Resources	Processor Time	00:00:00.02
	Elapsed Time	00:00:00.09

Tests of Between-Subjects Effects

Dependent Variable: Finance_Tot

Source	Type III Sum of Squares	df	Mean Square	F	Sig.
Corrected Model	52.679[a]	9	5.853	2.641	.006
Intercept	944.822	1	944.822	426.319	.000
MRN.High	.250	1	.250	.113	.737
RaceCC	1.156	1	1.156	.521	.471
GenderCC	.119	1	.119	.054	.817
SES0	.615	1	.615	.277	.599
Party0	.009	1	.009	.004	.948
MRN.High * RaceCC	1.433	1	1.433	.647	.422
MRN.High * GenderCC	3.603	1	3.603	1.626	.204
MRN.High * SES0	18.494	1	18.494	8.345	.004
MRN.High * Party0	.917	1	.917	.414	.521
Error	505.301	228	2.216		
Total	4179.000	238			
Corrected Total	557.980	237			

Tests of Between-Subjects Effects

Dependent Variable: Finance_Tot

Source	Partial Eta Squared
Corrected Model	.094
Intercept	.652
MRN.High	.000
RaceCC	.002
GenderCC	.000
SES0	.001
Party0	.000
MRN.High * RaceCC	.003
MRN.High * GenderCC	.007
MRN.High * SES0	.035
MRN.High * Party0	.002
Error	
Total	
Corrected Total	

a. R Squared = .094 (Adjusted R Squared = .059)

Parameter Estimates

Dependent Variable: Finance_Tot

Parameter	B	Std. Error	t	Sig.	95% Confidence Interval	
					Lower Bound	Upper Bound
Intercept	3.750	.182	20.647	.000	3.392	4.108
MRN.High	-.052	.153	-.336	.737	-.354	.251
RaceCC	-.127	.176	-.722	.471	-.473	.219
GenderCC	-.038	.164	-.231	.817	-.361	.285
SES0	.086	.162	.527	.599	-.235	.406
Party0	-.007	.107	-.065	.948	-.217	.203
MRN.High * RaceCC	-.107	.133	-.804	.422	-.370	.155
MRN.High * GenderCC	.165	.129	1.275	.204	-.090	.419
MRN.High * SES0	.370	.128	2.889	.004	.117	.622
MRN.High * Party0	.056	.087	.643	.521	-.115	.226

Parameter Estimates

Dependent Variable: Finance_Tot

Parameter	Partial Eta Squared
Intercept	.652
MRN.High	.000
RaceCC	.002
GenderCC	.000
SES0	.001
Party0	.000
MRN.High * RaceCC	.003
MRN.High * GenderCC	.007
MRN.High * SES0	.035
MRN.High * Party0	.002

```
 **Simple Slopes including MGRS

**MRN*Race on Personal Threat

UNIANOVA Concern_Tot WITH MRN0 Race.White GenderCC SES0 Party0 MGRS0
  /PRINT=ETASQ PARAMETER
  /DESIGN=MRN0 Race.White GenderCC SES0 Party0 MGRS0
  MRN0*Race.White MRN0*GenderCC MRN0*SES0 MRN0*Party0 MRN0*MGRS0 MGRS0*GenderCC MGRS0*Rac
```

```
e.White MGRS0*SES0 MGRS0*Party0.
```

Univariate Analysis of Variance

Notes

Output Created		15-DEC-2021 13:08:00
Comments		
Input	Data	C:\Users\njs5478\Dropbox\HM and COVID\0. Revise and Resubmit\2. R and R Data\Study 1b\Study1b_Data.sav
	Active Dataset	DataSet1
	Filter	<none>
	Weight	<none>
	Split File	<none>
	N of Rows in Working Data File	241
Missing Value Handling	Definition of Missing	User-defined missing values are treated as missing.
	Cases Used	Statistics are based on all cases with valid data for all variables in the model.
Syntax		UNIANOVA Concern_Tot WITH MRN0 Race.White GenderCC SES0 Party0 MGRS0 /PRINT=ETASQ PARAMETER /DESIGN=MRN0 Race.White GenderCC SES0 Party0 MGRS0 MRN0*Race.White MRN0*GenderCC MRN0*SES0 MRN0*Party0 MRN0*MGRS0 MGRS0*GenderCC MGRS0*Race.White MGRS0*SES0 MGRS0*Party0.
Resources	Processor Time	00:00:00.02
	Elapsed Time	00:00:00.03

Tests of Between-Subjects Effects

Dependent Variable: Concern_Tot

Source	Type III Sum of Squares	df	Mean Square	F	Sig.
Corrected Model	146.108[a]	15	9.741	6.909	.000
Intercept	1804.046	1	1804.046	1279.566	.000
MRN0	17.916	1	17.916	12.707	.000
Race.White	1.667	1	1.667	1.183	.278
GenderCC	2.837	1	2.837	2.012	.157
SES0	1.194	1	1.194	.847	.358
Party0	17.129	1	17.129	12.149	.001
MGRS0	2.750	1	2.750	1.951	.164
MRN0 * Race.White	7.639	1	7.639	5.418	.021
MRN0 * GenderCC	2.825	1	2.825	2.004	.158
MRN0 * SES0	4.958	1	4.958	3.517	.062
MRN0 * Party0	.032	1	.032	.023	.880
MRN0 * MGRS0	10.645	1	10.645	7.550	.006
GenderCC * MGRS0	3.750	1	3.750	2.660	.104
Race.White * MGRS0	.764	1	.764	.542	.462
SES0 * MGRS0	1.952	1	1.952	1.384	.241
Party0 * MGRS0	3.334	1	3.334	2.365	.126
Error	311.585	221	1.410		
Total	4912.361	237			
Corrected Total	457.693	236			

Tests of Between-Subjects Effects

Dependent Variable: Concern_Tot

Source	Partial Eta Squared
Corrected Model	.319
Intercept	.853
MRN0	.054
Race.White	.005
GenderCC	.009
SES0	.004
Party0	.052
MGRS0	.009
MRN0 * Race.White	.024
MRN0 * GenderCC	.009
MRN0 * SES0	.016
MRN0 * Party0	.000
MRN0 * MGRS0	.033
GenderCC * MGRS0	.012
Race.White * MGRS0	.002
SES0 * MGRS0	.006
Party0 * MGRS0	.011
Error	
Total	
Corrected Total	

a. R Squared = .319 (Adjusted R Squared = .273)

Parameter Estimates

Dependent Variable: Concern_Tot

Parameter	B	Std. Error	t	Sig.	95% Confidence Interval	
					Lower Bound	Upper Bound
Intercept	4.108	.115	35.771	.000	3.881	4.334
MRN0	-.478	.134	-3.565	.000	-.742	-.214
Race.White	.210	.193	1.087	.278	-.170	.590
GenderCC	-.140	.099	-1.419	.157	-.334	.054
SES0	.091	.099	.920	.358	-.104	.287
Party0	-.239	.069	-3.486	.001	-.375	-.104
MGRS0	.223	.159	1.397	.164	-.091	.537
MRN0 * Race.White	.510	.219	2.328	.021	.078	.941
MRN0 * GenderCC	.181	.128	1.416	.158	-.071	.432
MRN0 * SES0	.197	.105	1.875	.062	-.010	.405
MRN0 * Party0	.011	.074	.151	.880	-.135	.157
MRN0 * MGRS0	.517	.188	2.748	.006	.146	.888
GenderCC * MGRS0	-.269	.165	-1.631	.104	-.594	.056
Race.White * MGRS0	-.235	.320	-.736	.462	-.865	.395
SES0 * MGRS0	.193	.164	1.177	.241	-.131	.517
Party0 * MGRS0	-.193	.125	-1.538	.126	-.439	.054

Parameter Estimates

Dependent Variable: Concern_Tot

Parameter	Partial Eta Squared
Intercept	.853
MRN0	.054
Race.White	.005
GenderCC	.009
SES0	.004
Party0	.052
MGRS0	.009
MRN0 * Race.White	.024
MRN0 * GenderCC	.009
MRN0 * SES0	.016
MRN0 * Party0	.000
MRN0 * MGRS0	.033
GenderCC * MGRS0	.012
Race.White * MGRS0	.002
SES0 * MGRS0	.006
Party0 * MGRS0	.011

```
UNIANOVA Concern_Tot WITH MRN0 Race.Non GenderCC SES0 Party0 MGRS0
  /PRINT=ETASQ PARAMETER
  /DESIGN=MRN0 Race.Non GenderCC SES0 Party0 MGRS0
  MRN0*Race.Non MRN0*GenderCC MRN0*SES0 MRN0*Party0 MRN0*MGRS0 MGRS0*GenderCC MGRS0*Race.
Non MGRS0*SES0 MGRS0*Party0.
```

Univariate Analysis of Variance

Notes

Output Created		15-DEC-2021 13:08:00
Comments		
Input	Data	C:\Users\njs5478\Dropbox\HM and COVID\0. Revise and Resubmit\2. R and R Data\Study 1b\Study1b_Data.sav
	Active Dataset	DataSet1
	Filter	<none>
	Weight	<none>
	Split File	<none>
	N of Rows in Working Data File	241
Missing Value Handling	Definition of Missing	User-defined missing values are treated as missing.
	Cases Used	Statistics are based on all cases with valid data for all variables in the model.
Syntax		UNIANOVA Concern_Tot WITH MRN0 Race.Non GenderCC SES0 Party0 MGRS0<br>/PRINT=ETASQ PARAMETER<br>/DESIGN=MRN0 Race.Non GenderCC SES0 Party0 MGRS0<br>MRN0*Race.Non MRN0*GenderCC MRN0*SES0 MRN0*Party0 MRN0*MGRS0 MGRS0*GenderCC MGRS0*Race.Non MGRS0*SES0 MGRS0*Party0.
Resources	Processor Time	00:00:00.02
	Elapsed Time	00:00:00.03

Tests of Between-Subjects Effects

Dependent Variable: Concern_Tot

Source	Type III Sum of Squares	df	Mean Square	F	Sig.	Partial Eta Squared
Corrected Model	146.108[a]	15	9.741	6.909	.000	.319
Intercept	768.858	1	768.858	545.333	.000	.712
MRN0	.035	1	.035	.025	.874	.000
Race.Non	1.667	1	1.667	1.183	.278	.005
GenderCC	2.837	1	2.837	2.012	.157	.009
SES0	1.194	1	1.194	.847	.358	.004
Party0	17.129	1	17.129	12.149	.001	.052
MGRS0	.003	1	.003	.002	.965	.000
MRN0 * Race.Non	7.639	1	7.639	5.418	.021	.024
MRN0 * GenderCC	2.825	1	2.825	2.004	.158	.009
MRN0 * SES0	4.958	1	4.958	3.517	.062	.016
MRN0 * Party0	.032	1	.032	.023	.880	.000
MRN0 * MGRS0	10.645	1	10.645	7.550	.006	.033
GenderCC * MGRS0	3.750	1	3.750	2.660	.104	.012
Race.Non * MGRS0	.764	1	.764	.542	.462	.002
SES0 * MGRS0	1.952	1	1.952	1.384	.241	.006
Party0 * MGRS0	3.334	1	3.334	2.365	.126	.011
Error	311.585	221	1.410			
Total	4912.361	237				
Corrected Total	457.693	236				

a. R Squared = .319 (Adjusted R Squared = .273)

Parameter Estimates

Dependent Variable: Concern_Tot

Parameter	B	Std. Error	t	Sig.	95% Confidence Interval	
					Lower Bound	Upper Bound
Intercept	4.317	.185	23.352	.000	3.953	4.682
MRN0	.032	.200	.158	.874	-.362	.425
Race.Non	-.210	.193	-1.087	.278	-.590	.170
GenderCC	-.140	.099	-1.419	.157	-.334	.054
SES0	.091	.099	.920	.358	-.104	.287
Party0	-.239	.069	-3.486	.001	-.375	-.104
MGRS0	-.013	.292	-.044	.965	-.587	.562
MRN0 * Race.Non	-.510	.219	-2.328	.021	-.941	-.078
MRN0 * GenderCC	.181	.128	1.416	.158	-.071	.432
MRN0 * SES0	.197	.105	1.875	.062	-.010	.405
MRN0 * Party0	.011	.074	.151	.880	-.135	.157
MRN0 * MGRS0	.517	.188	2.748	.006	.146	.888
GenderCC * MGRS0	-.269	.165	-1.631	.104	-.594	.056
Race.Non * MGRS0	.235	.320	.736	.462	-.395	.865
SES0 * MGRS0	.193	.164	1.177	.241	-.131	.517
Party0 * MGRS0	-.193	.125	-1.538	.126	-.439	.054

Parameter Estimates

Dependent Variable: Concern_Tot

Parameter	Partial Eta Squared
Intercept	.712
MRN0	.000
Race.Non	.005
GenderCC	.009
SES0	.004
Party0	.052
MGRS0	.000
MRN0 * Race.Non	.024
MRN0 * GenderCC	.009
MRN0 * SES0	.016
MRN0 * Party0	.000
MRN0 * MGRS0	.033
GenderCC * MGRS0	.012
Race.Non * MGRS0	.002
SES0 * MGRS0	.006
Party0 * MGRS0	.011

```
 UNIANOVA Concern_Tot WITH MRN.Low RaceCC GenderCC SES0 Party0 MGRS0
  /PRINT=ETASQ PARAMETER
  /DESIGN=MRN.Low RaceCC GenderCC SES0 Party0 MGRS0
  MRN.Low*RaceCC MRN.Low*GenderCC MRN.Low*SES0 MRN.Low*Party0 MRN.Low*MGRS0 MGRS0*GenderC
C MGRS0*RaceCC MGRS0*SES0 MGRS0*Party0.
```

Univariate Analysis of Variance

Notes

Output Created		15-DEC-2021 13:08:00
Comments		
Input	Data	C:\Users\njs5478\Dropbox\HM and COVID\0. Revise and Resubmit\2. R and R Data\Study 1b\Study1b_Data.sav
	Active Dataset	DataSet1
	Filter	<none>
	Weight	<none>
	Split File	<none>
	N of Rows in Working Data File	241
Missing Value Handling	Definition of Missing	User-defined missing values are treated as missing.
	Cases Used	Statistics are based on all cases with valid data for all variables in the model.
Syntax		UNIANOVA Concern_Tot WITH MRN.Low RaceCC GenderCC SES0 Party0 MGRS0 /PRINT=ETASQ PARAMETER /DESIGN=MRN.Low RaceCC GenderCC SES0 Party0 MGRS0 MRN.Low*RaceCC MRN.Low*GenderCC MRN.Low*SES0 MRN.Low*Party0 MRN.Low*MGRS0 MGRS0*GenderCC MGRS0*RaceCC MGRS0*SES0 MGRS0*Party0.
Resources	Processor Time	00:00:00.02
	Elapsed Time	00:00:00.03

Tests of Between-Subjects Effects

Dependent Variable: Concern_Tot

Source	Type III Sum of Squares	df	Mean Square	F	Sig.
Corrected Model	146.108[a]	15	9.741	6.909	.000
Intercept	963.157	1	963.157	683.144	.000
MRN.Low	4.144	1	4.144	2.939	.088
RaceCC	1.212	1	1.212	.860	.355
GenderCC	5.464	1	5.464	3.876	.050
SES0	.471	1	.471	.334	.564
Party0	9.165	1	9.165	6.501	.011
MGRS0	2.269	1	2.269	1.609	.206
MRN.Low * RaceCC	7.639	1	7.639	5.418	.021
MRN.Low * GenderCC	2.825	1	2.825	2.004	.158
MRN.Low * SES0	4.958	1	4.958	3.517	.062
MRN.Low * Party0	.032	1	.032	.023	.880
MRN.Low * MGRS0	10.645	1	10.645	7.550	.006
GenderCC * MGRS0	3.750	1	3.750	2.660	.104
RaceCC * MGRS0	.764	1	.764	.542	.462
SES0 * MGRS0	1.952	1	1.952	1.384	.241
Party0 * MGRS0	3.334	1	3.334	2.365	.126
Error	311.585	221	1.410		
Total	4912.361	237			
Corrected Total	457.693	236			

Tests of Between-Subjects Effects

Dependent Variable: Concern_Tot

Source	Partial Eta Squared
Corrected Model	.319
Intercept	.756
MRN.Low	.013
RaceCC	.004
GenderCC	.017
SES0	.002
Party0	.029
MGRS0	.007
MRN.Low * RaceCC	.024
MRN.Low * GenderCC	.009
MRN.Low * SES0	.016
MRN.Low * Party0	.000
MRN.Low * MGRS0	.033
GenderCC * MGRS0	.012
RaceCC * MGRS0	.002
SES0 * MGRS0	.006
Party0 * MGRS0	.011
Error	
Total	
Corrected Total	

a. R Squared = .319 (Adjusted R Squared = .273)

Parameter Estimates

Dependent Variable: Concern_Tot

Parameter	B	Std. Error	t	Sig.	95% Confidence Interval	
					Lower Bound	Upper Bound
Intercept	4.406	.169	26.137	.000	4.074	4.738
MRN.Low	-.223	.130	-1.714	.088	-.480	.033
RaceCC	.116	.125	.927	.355	-.130	.362
GenderCC	-.296	.150	-1.969	.050	-.593	.000
SES0	-.079	.138	-.578	.564	-.350	.192
Party0	-.249	.098	-2.550	.011	-.441	-.057
MGRS0	-.343	.270	-1.269	.206	-.875	.190
MRN.Low * RaceCC	-.255	.109	-2.328	.021	-.471	-.039
MRN.Low * GenderCC	.181	.128	1.416	.158	-.071	.432
MRN.Low * SES0	.197	.105	1.875	.062	-.010	.405
MRN.Low * Party0	.011	.074	.151	.880	-.135	.157
MRN.Low * MGRS0	.517	.188	2.748	.006	.146	.888
GenderCC * MGRS0	-.269	.165	-1.631	.104	-.594	.056
RaceCC * MGRS0	.118	.160	.736	.462	-.197	.433
SES0 * MGRS0	.193	.164	1.177	.241	-.131	.517
Party0 * MGRS0	-.193	.125	-1.538	.126	-.439	.054

Parameter Estimates

Dependent Variable: Concern_Tot

Parameter	Partial Eta Squared
Intercept	.756
MRN.Low	.013
RaceCC	.004
GenderCC	.017
SES0	.002
Party0	.029
MGRS0	.007
MRN.Low * RaceCC	.024
MRN.Low * GenderCC	.009
MRN.Low * SES0	.016
MRN.Low * Party0	.000
MRN.Low * MGRS0	.033
GenderCC * MGRS0	.012
RaceCC * MGRS0	.002
SES0 * MGRS0	.006
Party0 * MGRS0	.011

```
 UNIANOVA Concern_Tot WITH MRN.High RaceCC GenderCC SES0 Party0 MGRS0
  /PRINT=ETASQ PARAMETER
  /DESIGN=MRN.High RaceCC GenderCC SES0 Party0 MGRS0
  MRN.High*RaceCC MRN.High*GenderCC MRN.High*SES0 MRN.High*Party0 MRN.High*MGRS0 MGRS0*Ge
nderCC MGRS0*RaceCC MGRS0*SES0 MGRS0*Party0.
```

Univariate Analysis of Variance

Notes

Output Created		15-DEC-2021 13:08:00
Comments		
Input	Data	C: \Users\njs5478\Dropbox\HM and COVID\0. Revise and Resubmit\2. R and R Data\Study 1b\Study1b_Data.sav
	Active Dataset	DataSet1
	Filter	<none>
	Weight	<none>
	Split File	<none>
	N of Rows in Working Data File	241
Missing Value Handling	Definition of Missing	User-defined missing values are treated as missing.
	Cases Used	Statistics are based on all cases with valid data for all variables in the model.
Syntax		UNIANOVA Concern_Tot WITH MRN.High RaceCC GenderCC SES0 Party0 MGRS0<br>/PRINT=ETASQ PARAMETER<br>/DESIGN=MRN.High RaceCC GenderCC SES0 Party0 MGRS0<br>MRN.High*RaceCC MRN.High*GenderCC MRN.High*SES0 MRN.High*Party0 MRN.High*MGRS0 MGRS0*GenderCC MGRS0*RaceCC MGRS0*SES0 MGRS0*Party0.
Resources	Processor Time	00:00:00.02
	Elapsed Time	00:00:00.02

Tests of Between-Subjects Effects

Dependent Variable: Concern_Tot

Source	Type III Sum of Squares	df	Mean Square	F	Sig.
Corrected Model	146.108[a]	15	9.741	6.909	.000
Intercept	883.368	1	883.368	626.552	.000
MRN.High	4.144	1	4.144	2.939	.088
RaceCC	7.127	1	7.127	5.055	.026
GenderCC	.019	1	.019	.013	.909
SES0	5.566	1	5.566	3.948	.048
Party0	9.129	1	9.129	6.475	.012
MGRS0	10.931	1	10.931	7.753	.006
MRN.High * RaceCC	7.639	1	7.639	5.418	.021
MRN.High * GenderCC	2.825	1	2.825	2.004	.158
MRN.High * SES0	4.958	1	4.958	3.517	.062
MRN.High * Party0	.032	1	.032	.023	.880
MRN.High * MGRS0	10.645	1	10.645	7.550	.006
GenderCC * MGRS0	3.750	1	3.750	2.660	.104
RaceCC * MGRS0	.764	1	.764	.542	.462
SES0 * MGRS0	1.952	1	1.952	1.384	.241
Party0 * MGRS0	3.334	1	3.334	2.365	.126
Error	311.585	221	1.410		
Total	4912.361	237			
Corrected Total	457.693	236			

Tests of Between-Subjects Effects

Dependent Variable: Concern_Tot

Source	Partial Eta Squared
Corrected Model	.319
Intercept	.739
MRN.High	.013
RaceCC	.022
GenderCC	.000
SES0	.018
Party0	.028
MGRS0	.034
MRN.High * RaceCC	.024
MRN.High * GenderCC	.009
MRN.High * SES0	.016
MRN.High * Party0	.000
MRN.High * MGRS0	.033
GenderCC * MGRS0	.012
RaceCC * MGRS0	.002
SES0 * MGRS0	.006
Party0 * MGRS0	.011
Error	
Total	
Corrected Total	

a. R Squared = .319 (Adjusted R Squared = .273)

Parameter Estimates

Dependent Variable: Concern_Tot

Parameter	B	Std. Error	t	Sig.	95% Confidence Interval Lower Bound	95% Confidence Interval Upper Bound
Intercept	4.019	.161	25.031	.000	3.703	4.336
MRN.High	-.223	.130	-1.714	.088	-.480	.033
RaceCC	-.325	.145	-2.248	.026	-.611	-.040
GenderCC	.017	.146	.115	.909	-.270	.304
SES0	.262	.132	1.987	.048	.002	.522
Party0	-.230	.090	-2.545	.012	-.407	-.052
MGRS0	.552	.198	2.784	.006	.161	.944
MRN.High * RaceCC	-.255	.109	-2.328	.021	-.471	-.039
MRN.High * GenderCC	.181	.128	1.416	.158	-.071	.432
MRN.High * SES0	.197	.105	1.875	.062	-.010	.405
MRN.High * Party0	.011	.074	.151	.880	-.135	.157
MRN.High * MGRS0	.517	.188	2.748	.006	.146	.888
GenderCC * MGRS0	-.269	.165	-1.631	.104	-.594	.056
RaceCC * MGRS0	.118	.160	.736	.462	-.197	.433
SES0 * MGRS0	.193	.164	1.177	.241	-.131	.517
Party0 * MGRS0	-.193	.125	-1.538	.126	-.439	.054

Parameter Estimates

Dependent Variable: Concern_Tot

Parameter	Partial Eta Squared
Intercept	.739
MRN.High	.013
RaceCC	.022
GenderCC	.000
SES0	.018
Party0	.028
MGRS0	.034
MRN.High * RaceCC	.024
MRN.High * GenderCC	.009
MRN.High * SES0	.016
MRN.High * Party0	.000
MRN.High * MGRS0	.033
GenderCC * MGRS0	.012
RaceCC * MGRS0	.002
SES0 * MGRS0	.006
Party0 * MGRS0	.011

```
 **MRN*MGRS on Personal Threat

UNIANOVA Concern_Tot WITH MRN.Low RaceCC GenderCC SES0 Party0 MGRS0
  /PRINT=ETASQ PARAMETER
  /DESIGN=MRN.Low RaceCC GenderCC SES0 Party0 MGRS0
  MRN.Low*RaceCC MRN.Low*GenderCC MRN.Low*SES0 MRN.Low*Party0 MRN.Low*MGRS0 MGRS0*GenderC
C MGRS0*RaceCC MGRS0*SES0 MGRS0*Party0.
```

Univariate Analysis of Variance

Notes

Output Created		15-DEC-2021 13:08:00
Comments		
Input	Data	C:\Users\njs5478\Dropbox\HM and COVID\0. Revise and Resubmit\2. R and R Data\Study 1b\Study1b_Data.sav
	Active Dataset	DataSet1
	Filter	<none>
	Weight	<none>
	Split File	<none>
	N of Rows in Working Data File	241
Missing Value Handling	Definition of Missing	User-defined missing values are treated as missing.
	Cases Used	Statistics are based on all cases with valid data for all variables in the model.
Syntax		UNIANOVA Concern_Tot WITH MRN.Low RaceCC GenderCC SES0 Party0 MGRS0<br>/PRINT=ETASQ PARAMETER<br>/DESIGN=MRN.Low RaceCC GenderCC SES0 Party0 MGRS0<br>MRN.Low*RaceCC MRN.Low*GenderCC MRN.Low*SES0 MRN.Low*Party0 MRN.Low*MGRS0 MGRS0*GenderCC MGRS0*RaceCC MGRS0*SES0 MGRS0*Party0.
Resources	Processor Time	00:00:00.02
	Elapsed Time	00:00:00.02

Tests of Between-Subjects Effects

Dependent Variable: Concern_Tot

Source	Type III Sum of Squares	df	Mean Square	F	Sig.
Corrected Model	146.108[a]	15	9.741	6.909	.000
Intercept	963.157	1	963.157	683.144	.000
MRN.Low	4.144	1	4.144	2.939	.088
RaceCC	1.212	1	1.212	.860	.355
GenderCC	5.464	1	5.464	3.876	.050
SES0	.471	1	.471	.334	.564
Party0	9.165	1	9.165	6.501	.011
MGRS0	2.269	1	2.269	1.609	.206
MRN.Low * RaceCC	7.639	1	7.639	5.418	.021
MRN.Low * GenderCC	2.825	1	2.825	2.004	.158
MRN.Low * SES0	4.958	1	4.958	3.517	.062
MRN.Low * Party0	.032	1	.032	.023	.880
MRN.Low * MGRS0	10.645	1	10.645	7.550	.006
GenderCC * MGRS0	3.750	1	3.750	2.660	.104
RaceCC * MGRS0	.764	1	.764	.542	.462
SES0 * MGRS0	1.952	1	1.952	1.384	.241
Party0 * MGRS0	3.334	1	3.334	2.365	.126
Error	311.585	221	1.410		
Total	4912.361	237			
Corrected Total	457.693	236			

Tests of Between-Subjects Effects

Dependent Variable: Concern_Tot

Source	Partial Eta Squared
Corrected Model	.319
Intercept	.756
MRN.Low	.013
RaceCC	.004
GenderCC	.017
SES0	.002
Party0	.029
MGRS0	.007
MRN.Low * RaceCC	.024
MRN.Low * GenderCC	.009
MRN.Low * SES0	.016
MRN.Low * Party0	.000
MRN.Low * MGRS0	.033
GenderCC * MGRS0	.012
RaceCC * MGRS0	.002
SES0 * MGRS0	.006
Party0 * MGRS0	.011
Error	
Total	
Corrected Total	

a. R Squared = .319 (Adjusted R Squared = .273)

Parameter Estimates

Dependent Variable: Concern_Tot

Parameter	B	Std. Error	t	Sig.	95% Confidence Interval	
					Lower Bound	Upper Bound
Intercept	4.406	.169	26.137	.000	4.074	4.738
MRN.Low	-.223	.130	-1.714	.088	-.480	.033
RaceCC	.116	.125	.927	.355	-.130	.362
GenderCC	-.296	.150	-1.969	.050	-.593	.000
SES0	-.079	.138	-.578	.564	-.350	.192
Party0	-.249	.098	-2.550	.011	-.441	-.057
MGRS0	-.343	.270	-1.269	.206	-.875	.190
MRN.Low * RaceCC	-.255	.109	-2.328	.021	-.471	-.039
MRN.Low * GenderCC	.181	.128	1.416	.158	-.071	.432
MRN.Low * SES0	.197	.105	1.875	.062	-.010	.405
MRN.Low * Party0	.011	.074	.151	.880	-.135	.157
MRN.Low * MGRS0	.517	.188	2.748	.006	.146	.888
GenderCC * MGRS0	-.269	.165	-1.631	.104	-.594	.056
RaceCC * MGRS0	.118	.160	.736	.462	-.197	.433
SES0 * MGRS0	.193	.164	1.177	.241	-.131	.517
Party0 * MGRS0	-.193	.125	-1.538	.126	-.439	.054

Parameter Estimates

Dependent Variable: Concern_Tot

Parameter	Partial Eta Squared
Intercept	.756
MRN.Low	.013
RaceCC	.004
GenderCC	.017
SES0	.002
Party0	.029
MGRS0	.007
MRN.Low * RaceCC	.024
MRN.Low * GenderCC	.009
MRN.Low * SES0	.016
MRN.Low * Party0	.000
MRN.Low * MGRS0	.033
GenderCC * MGRS0	.012
RaceCC * MGRS0	.002
SES0 * MGRS0	.006
Party0 * MGRS0	.011

```
 UNIANOVA Concern_Tot WITH MRN.High RaceCC GenderCC SES0 Party0 MGRS0
  /PRINT=ETASQ PARAMETER
  /DESIGN=MRN.High RaceCC GenderCC SES0 Party0 MGRS0
  MRN.High*RaceCC MRN.High*GenderCC MRN.High*SES0 MRN.High*Party0 MRN.High*MGRS0 MGRS0*Ge
nderCC MGRS0*RaceCC MGRS0*SES0 MGRS0*Party0.
```

Univariate Analysis of Variance

Notes

Output Created		15-DEC-2021 13:08:00
Comments		
Input	Data	C:\Users\njs5478\Dropbox\HM and COVID\0. Revise and Resubmit\2. R and R Data\Study 1b\Study1b_Data.sav
	Active Dataset	DataSet1
	Filter	<none>
	Weight	<none>
	Split File	<none>
	N of Rows in Working Data File	241
Missing Value Handling	Definition of Missing	User-defined missing values are treated as missing.
	Cases Used	Statistics are based on all cases with valid data for all variables in the model.
Syntax		UNIANOVA Concern_Tot WITH MRN.High RaceCC GenderCC SES0 Party0 MGRS0<br>/PRINT=ETASQ PARAMETER<br>/DESIGN=MRN.High RaceCC GenderCC SES0 Party0 MGRS0<br>MRN.High*RaceCC MRN.High*GenderCC MRN.High*SES0 MRN.High*Party0 MRN.High*MGRS0 MGRS0*GenderCC MGRS0*RaceCC MGRS0*SES0 MGRS0*Party0.
Resources	Processor Time	00:00:00.02
	Elapsed Time	00:00:00.03

Tests of Between-Subjects Effects

Dependent Variable: Concern_Tot

Source	Type III Sum of Squares	df	Mean Square	F	Sig.
Corrected Model	146.108[a]	15	9.741	6.909	.000
Intercept	883.368	1	883.368	626.552	.000
MRN.High	4.144	1	4.144	2.939	.088
RaceCC	7.127	1	7.127	5.055	.026
GenderCC	.019	1	.019	.013	.909
SES0	5.566	1	5.566	3.948	.048
Party0	9.129	1	9.129	6.475	.012
MGRS0	10.931	1	10.931	7.753	.006
MRN.High * RaceCC	7.639	1	7.639	5.418	.021
MRN.High * GenderCC	2.825	1	2.825	2.004	.158
MRN.High * SES0	4.958	1	4.958	3.517	.062
MRN.High * Party0	.032	1	.032	.023	.880
MRN.High * MGRS0	10.645	1	10.645	7.550	.006
GenderCC * MGRS0	3.750	1	3.750	2.660	.104
RaceCC * MGRS0	.764	1	.764	.542	.462
SES0 * MGRS0	1.952	1	1.952	1.384	.241
Party0 * MGRS0	3.334	1	3.334	2.365	.126
Error	311.585	221	1.410		
Total	4912.361	237			
Corrected Total	457.693	236			

Tests of Between-Subjects Effects

Dependent Variable: Concern_Tot

Source	Partial Eta Squared
Corrected Model	.319
Intercept	.739
MRN.High	.013
RaceCC	.022
GenderCC	.000
SES0	.018
Party0	.028
MGRS0	.034
MRN.High * RaceCC	.024
MRN.High * GenderCC	.009
MRN.High * SES0	.016
MRN.High * Party0	.000
MRN.High * MGRS0	.033
GenderCC * MGRS0	.012
RaceCC * MGRS0	.002
SES0 * MGRS0	.006
Party0 * MGRS0	.011
Error	
Total	
Corrected Total	

a. R Squared = .319 (Adjusted R Squared = .273)

Parameter Estimates

Dependent Variable: Concern_Tot

Parameter	B	Std. Error	t	Sig.	95% Confidence Interval Lower Bound	95% Confidence Interval Upper Bound
Intercept	4.019	.161	25.031	.000	3.703	4.336
MRN.High	-.223	.130	-1.714	.088	-.480	.033
RaceCC	-.325	.145	-2.248	.026	-.611	-.040
GenderCC	.017	.146	.115	.909	-.270	.304
SES0	.262	.132	1.987	.048	.002	.522
Party0	-.230	.090	-2.545	.012	-.407	-.052
MGRS0	.552	.198	2.784	.006	.161	.944
MRN.High * RaceCC	-.255	.109	-2.328	.021	-.471	-.039
MRN.High * GenderCC	.181	.128	1.416	.158	-.071	.432
MRN.High * SES0	.197	.105	1.875	.062	-.010	.405
MRN.High * Party0	.011	.074	.151	.880	-.135	.157
MRN.High * MGRS0	.517	.188	2.748	.006	.146	.888
GenderCC * MGRS0	-.269	.165	-1.631	.104	-.594	.056
RaceCC * MGRS0	.118	.160	.736	.462	-.197	.433
SES0 * MGRS0	.193	.164	1.177	.241	-.131	.517
Party0 * MGRS0	-.193	.125	-1.538	.126	-.439	.054

Parameter Estimates

Dependent Variable: Concern_Tot

Parameter	Partial Eta Squared
Intercept	.739
MRN.High	.013
RaceCC	.022
GenderCC	.000
SES0	.018
Party0	.028
MGRS0	.034
MRN.High * RaceCC	.024
MRN.High * GenderCC	.009
MRN.High * SES0	.016
MRN.High * Party0	.000
MRN.High * MGRS0	.033
GenderCC * MGRS0	.012
RaceCC * MGRS0	.002
SES0 * MGRS0	.006
Party0 * MGRS0	.011

```
UNIANOVA Concern_Tot WITH MRN0 RaceCC GenderCC SES0 Party0 MGRS.Low
  /PRINT=ETASQ PARAMETER
  /DESIGN=MRN0 RaceCC GenderCC SES0 Party0 MGRS.Low
  MRN0*RaceCC MRN0*GenderCC MRN0*SES0 MRN0*Party0 MRN0*MGRS.Low MGRS.Low*GenderCC MGRS.Lo
w*RaceCC MGRS.Low*SES0 MGRS.Low*Party0.
```

Univariate Analysis of Variance

Notes

Output Created		15-DEC-2021 13:08:00
Comments		
Input	Data	C: \Users\njs5478\Dropbox\HM and COVID\0. Revise and Resubmit\2. R and R Data\Study 1b\Study1b_Data.sav
	Active Dataset	DataSet1
	Filter	<none>
	Weight	<none>
	Split File	<none>
	N of Rows in Working Data File	241
Missing Value Handling	Definition of Missing	User-defined missing values are treated as missing.
	Cases Used	Statistics are based on all cases with valid data for all variables in the model.
Syntax		UNIANOVA Concern_Tot WITH MRN0 RaceCC GenderCC SES0 Party0 MGRS.Low /PRINT=ETASQ PARAMETER /DESIGN=MRN0 RaceCC GenderCC SES0 Party0 MGRS.Low MRN0*RaceCC MRN0*GenderCC MRN0*SES0 MRN0*Party0 MRN0*MGRS.Low MGRS.Low*GenderCC MGRS.Low*RaceCC MGRS.Low*SES0 MGRS.Low*Party0.
Resources	Processor Time	00:00:00.02
	Elapsed Time	00:00:00.02

Tests of Between-Subjects Effects

Dependent Variable: Concern_Tot

Source	Type III Sum of Squares	df	Mean Square	F	Sig.
Corrected Model	146.108[a]	15	9.741	6.909	.000
Intercept	988.874	1	988.874	701.384	.000
MRN0	13.769	1	13.769	9.766	.002
RaceCC	2.095	1	2.095	1.486	.224
GenderCC	.086	1	.086	.061	.805
SES0	.082	1	.082	.058	.809
Party0	1.489	1	1.489	1.056	.305
MGRS.Low	.523	1	.523	.371	.543
MRN0 * RaceCC	7.639	1	7.639	5.418	.021
MRN0 * GenderCC	2.825	1	2.825	2.004	.158
MRN0 * SES0	4.958	1	4.958	3.517	.062
MRN0 * Party0	.032	1	.032	.023	.880
MRN0 * MGRS.Low	10.645	1	10.645	7.550	.006
GenderCC * MGRS.Low	3.750	1	3.750	2.660	.104
RaceCC * MGRS.Low	.764	1	.764	.542	.462
SES0 * MGRS.Low	1.952	1	1.952	1.384	.241
Party0 * MGRS.Low	3.334	1	3.334	2.365	.126
Error	311.585	221	1.410		
Total	4912.361	237			
Corrected Total	457.693	236			

Tests of Between-Subjects Effects

Dependent Variable: Concern_Tot

Source	Partial Eta Squared
Corrected Model	.319
Intercept	.760
MRN0	.042
RaceCC	.007
GenderCC	.000
SES0	.000
Party0	.005
MGRS.Low	.002
MRN0 * RaceCC	.024
MRN0 * GenderCC	.009
MRN0 * SES0	.016
MRN0 * Party0	.000
MRN0 * MGRS.Low	.033
GenderCC * MGRS.Low	.012
RaceCC * MGRS.Low	.002
SES0 * MGRS.Low	.006
Party0 * MGRS.Low	.011
Error	
Total	
Corrected Total	

a. R Squared = .319 (Adjusted R Squared = .273)

Parameter Estimates

Dependent Variable: Concern_Tot

Parameter	B	Std. Error	t	Sig.	95% Confidence Interval Lower Bound	95% Confidence Interval Upper Bound
Intercept	4.145	.157	26.484	.000	3.837	4.454
MRN0	-.556	.178	-3.125	.002	-.906	-.205
RaceCC	-.181	.148	-1.219	.224	-.472	.111
GenderCC	.033	.135	.248	.805	-.233	.300
SES0	-.033	.137	-.241	.809	-.303	.237
Party0	-.115	.112	-1.028	.305	-.337	.106
MGRS.Low	.105	.172	.609	.543	-.234	.444
MRN0 * RaceCC	-.255	.109	-2.328	.021	-.471	-.039
MRN0 * GenderCC	.181	.128	1.416	.158	-.071	.432
MRN0 * SES0	.197	.105	1.875	.062	-.010	.405
MRN0 * Party0	.011	.074	.151	.880	-.135	.157
MRN0 * MGRS.Low	.517	.188	2.748	.006	.146	.888
GenderCC * MGRS.Low	-.269	.165	-1.631	.104	-.594	.056
RaceCC * MGRS.Low	.118	.160	.736	.462	-.197	.433
SES0 * MGRS.Low	.193	.164	1.177	.241	-.131	.517
Party0 * MGRS.Low	-.193	.125	-1.538	.126	-.439	.054

Parameter Estimates

Dependent Variable: Concern_Tot

Parameter	Partial Eta Squared
Intercept	.760
MRN0	.042
RaceCC	.007
GenderCC	.000
SES0	.000
Party0	.005
MGRS.Low	.002
MRN0 * RaceCC	.024
MRN0 * GenderCC	.009
MRN0 * SES0	.016
MRN0 * Party0	.000
MRN0 * MGRS.Low	.033
GenderCC * MGRS.Low	.012
RaceCC * MGRS.Low	.002
SES0 * MGRS.Low	.006
Party0 * MGRS.Low	.011

```
 UNIANOVA Concern_Tot WITH MRN0 RaceCC GenderCC SES0 Party0 MGRS.High
  /PRINT=ETASQ PARAMETER
  /DESIGN=MRN0 RaceCC GenderCC SES0 Party0 MGRS.High
  MRN0*RaceCC MRN0*GenderCC MRN0*SES0 MRN0*Party0 MRN0*MGRS.High MGRS.High*GenderCC MGRS.
High*RaceCC MGRS.High*SES0 MGRS.High*Party0.
```

Univariate Analysis of Variance

Notes

Output Created		15-DEC-2021 13:08:00
Comments		
Input	Data	C:\Users\njs5478\Dropbox\HM and COVID\0. Revise and Resubmit\2. R and R Data\Study 1b\Study1b_Data.sav
	Active Dataset	DataSet1
	Filter	<none>
	Weight	<none>
	Split File	<none>
	N of Rows in Working Data File	241
Missing Value Handling	Definition of Missing	User-defined missing values are treated as missing.
	Cases Used	Statistics are based on all cases with valid data for all variables in the model.
Syntax		UNIANOVA Concern_Tot WITH MRN0 RaceCC GenderCC SES0 Party0 MGRS.High /PRINT=ETASQ PARAMETER /DESIGN=MRN0 RaceCC GenderCC SES0 Party0 MGRS.High MRN0*RaceCC MRN0*GenderCC MRN0*SES0 MRN0*Party0 MRN0*MGRS.High MGRS.High*GenderCC MGRS.High*RaceCC MGRS.High*SES0 MGRS.High*Party0.
Resources	Processor Time	00:00:00.02
	Elapsed Time	00:00:00.02

Tests of Between-Subjects Effects

Dependent Variable: Concern_Tot

Source	Type III Sum of Squares	df	Mean Square	F	Sig.
Corrected Model	146.108[a]	15	9.741	6.909	.000
Intercept	895.849	1	895.849	635.404	.000
MRN0	.534	1	.534	.379	.539
RaceCC	.067	1	.067	.048	.827
GenderCC	5.828	1	5.828	4.134	.043
SES0	2.809	1	2.809	1.992	.160
Party0	18.989	1	18.989	13.468	.000
MGRS.High	.523	1	.523	.371	.543
MRN0 * RaceCC	7.639	1	7.639	5.418	.021
MRN0 * GenderCC	2.825	1	2.825	2.004	.158
MRN0 * SES0	4.958	1	4.958	3.517	.062
MRN0 * Party0	.032	1	.032	.023	.880
MRN0 * MGRS.High	10.645	1	10.645	7.550	.006
GenderCC * MGRS.High	3.750	1	3.750	2.660	.104
RaceCC * MGRS.High	.764	1	.764	.542	.462
SES0 * MGRS.High	1.952	1	1.952	1.384	.241
Party0 * MGRS.High	3.334	1	3.334	2.365	.126
Error	311.585	221	1.410		
Total	4912.361	237			
Corrected Total	457.693	236			

Tests of Between-Subjects Effects

Dependent Variable: Concern_Tot

Source	Partial Eta Squared
Corrected Model	.319
Intercept	.742
MRN0	.002
RaceCC	.000
GenderCC	.018
SES0	.009
Party0	.057
MGRS.High	.002
MRN0 * RaceCC	.024
MRN0 * GenderCC	.009
MRN0 * SES0	.016
MRN0 * Party0	.000
MRN0 * MGRS.High	.033
GenderCC * MGRS.High	.012
RaceCC * MGRS.High	.002
SES0 * MGRS.High	.006
Party0 * MGRS.High	.011
Error	
Total	
Corrected Total	

a. R Squared = .319 (Adjusted R Squared = .273)

Parameter Estimates

Dependent Variable: Concern_Tot

Parameter	B	Std. Error	t	Sig.	95% Confidence Interval Lower Bound	95% Confidence Interval Upper Bound
Intercept	4.280	.170	25.207	.000	3.945	4.615
MRN0	.109	.178	.616	.539	-.241	.460
RaceCC	-.029	.133	-.218	.827	-.292	.234
GenderCC	-.313	.154	-2.033	.043	-.616	-.010
SES0	.216	.153	1.411	.160	-.086	.517
Party0	-.363	.099	-3.670	.000	-.558	-.168
MGRS.High	.105	.172	.609	.543	-.234	.444
MRN0 * RaceCC	-.255	.109	-2.328	.021	-.471	-.039
MRN0 * GenderCC	.181	.128	1.416	.158	-.071	.432
MRN0 * SES0	.197	.105	1.875	.062	-.010	.405
MRN0 * Party0	.011	.074	.151	.880	-.135	.157
MRN0 * MGRS.High	.517	.188	2.748	.006	.146	.888
GenderCC * MGRS.High	-.269	.165	-1.631	.104	-.594	.056
RaceCC * MGRS.High	.118	.160	.736	.462	-.197	.433
SES0 * MGRS.High	.193	.164	1.177	.241	-.131	.517
Party0 * MGRS.High	-.193	.125	-1.538	.126	-.439	.054

Parameter Estimates

Dependent Variable: Concern_Tot

Parameter	Partial Eta Squared
Intercept	.742
MRN0	.002
RaceCC	.000
GenderCC	.018
SES0	.009
Party0	.057
MGRS.High	.002
MRN0 * RaceCC	.024
MRN0 * GenderCC	.009
MRN0 * SES0	.016
MRN0 * Party0	.000
MRN0 * MGRS.High	.033
GenderCC * MGRS.High	.012
RaceCC * MGRS.High	.002
SES0 * MGRS.High	.006
Party0 * MGRS.High	.011

```
 **Simple Slopes that emerge in analyses including IDEOLOGY

IF (GenderCC=1) Male = 0.
IF (GenderCC=-1) Male= 1.
IF (GenderCC= 1) Female = 1.
IF (GenderCC=-1) Female = 0.

*HM*Gender on Democratic Congress (without Precarious Masculinity)

UNIANOVA DemCongressX WITH MRN0 RaceCC Male SES0 Ideology0
  /PRINT=ETASQ PARAMETER
  /DESIGN=MRN0 RaceCC Male SES0 Ideology0
  MRN0*RaceCC MRN0*Male MRN0*SES0 MRN0*Ideology0.
```

Univariate Analysis of Variance

Notes

Output Created		15-DEC-2021 13:08:00
Comments		
Input	Data	C: \Users\njs5478\Dropbox\HM and COVID\0. Revise and Resubmit\2. R and R Data\Study 1b\Study1b_Data.sav
	Active Dataset	DataSet1
	Filter	<none>
	Weight	<none>
	Split File	<none>
	N of Rows in Working Data File	241
Missing Value Handling	Definition of Missing	User-defined missing values are treated as missing.
	Cases Used	Statistics are based on all cases with valid data for all variables in the model.
Syntax		UNIANOVA DemCongressX WITH MRN0 RaceCC Male SES0 Ideology0 /PRINT=ETASQ PARAMETER /DESIGN=MRN0 RaceCC Male SES0 Ideology0 MRN0*RaceCC MRN0*Male MRN0*SES0 MRN0*Ideology0.
Resources	Processor Time	00:00:00.02
	Elapsed Time	00:00:00.02

Tests of Between-Subjects Effects

Dependent Variable: DemCongressX

Source	Type III Sum of Squares	df	Mean Square	F	Sig.	Partial Eta Squared
Corrected Model	220.137[a]	9	24.460	15.754	.000	.411
Intercept	1245.348	1	1245.348	802.129	.000	.798
MRN0	9.656	1	9.656	6.219	.013	.030
RaceCC	1.367	1	1.367	.881	.349	.004
Male	.664	1	.664	.428	.514	.002
SES0	.252	1	.252	.162	.688	.001
Ideology0	112.819	1	112.819	72.667	.000	.264
MRN0 * RaceCC	.704	1	.704	.453	.502	.002
MRN0 * Male	17.370	1	17.370	11.188	.001	.052
MRN0 * SES0	.119	1	.119	.076	.783	.000
MRN0 * Ideology0	.251	1	.251	.161	.688	.001
Error	315.168	203	1.553			
Total	4372.000	213				
Corrected Total	535.305	212				

a. R Squared = .411 (Adjusted R Squared = .385)

Parameter Estimates

Dependent Variable: DemCongressX

Parameter	B	Std. Error	t	Sig.	95% Confidence Interval	
					Lower Bound	Upper Bound
Intercept	4.293	.152	28.322	.000	3.994	4.592
MRN0	-.419	.168	-2.494	.013	-.751	-.088
RaceCC	.101	.107	.938	.349	-.111	.313
Male	.128	.196	.654	.514	-.258	.515
SES0	.042	.104	.403	.688	-.163	.246
Ideology0	-.596	.070	-8.524	.000	-.734	-.458
MRN0 * RaceCC	.081	.120	.673	.502	-.156	.318
MRN0 * Male	.781	.233	3.345	.001	.320	1.241
MRN0 * SES0	.030	.110	.276	.783	-.187	.248
MRN0 * Ideology0	-.025	.061	-.402	.688	-.146	.096

Parameter Estimates

Dependent Variable: DemCongressX

Parameter	Partial Eta Squared
Intercept	.798
MRN0	.030
RaceCC	.004
Male	.002
SES0	.001
Ideology0	.264
MRN0 * RaceCC	.002
MRN0 * Male	.052
MRN0 * SES0	.000
MRN0 * Ideology0	.001

```
UNIANOVA DemCongressX WITH MRN0 RaceCC Female SES0 Ideology0
 /PRINT=ETASQ PARAMETER
 /DESIGN=MRN0 RaceCC Female SES0 Ideology0
 MRN0*RaceCC MRN0*Female MRN0*SES0 MRN0*Ideology0.
```

Univariate Analysis of Variance

Notes

Output Created		15-DEC-2021 13:08:00
Comments		
Input	Data	C:\Users\njs5478\Dropbox\HM and COVID\0. Revise and Resubmit\2. R and R Data\Study 1b\Study1b_Data.sav
	Active Dataset	DataSet1
	Filter	<none>
	Weight	<none>
	Split File	<none>
	N of Rows in Working Data File	241
Missing Value Handling	Definition of Missing	User-defined missing values are treated as missing.
	Cases Used	Statistics are based on all cases with valid data for all variables in the model.
Syntax		UNIANOVA DemCongressX WITH MRN0 RaceCC Female SES0 Ideology0<br>/PRINT=ETASQ PARAMETER<br>/DESIGN=MRN0 RaceCC Female SES0 Ideology0<br>MRN0*RaceCC MRN0*Female MRN0*SES0 MRN0*Ideology0.
Resources	Processor Time	00:00:00.00
	Elapsed Time	00:00:00.02

Tests of Between-Subjects Effects

Dependent Variable: DemCongressX

Source	Type III Sum of Squares	df	Mean Square	F	Sig.	Partial Eta Squared
Corrected Model	220.137[a]	9	24.460	15.754	.000	.411
Intercept	1333.881	1	1333.881	859.153	.000	.809
MRN0	5.386	1	5.386	3.469	.064	.017
RaceCC	1.367	1	1.367	.881	.349	.004
Female	.664	1	.664	.428	.514	.002
SES0	.252	1	.252	.162	.688	.001
Ideology0	112.819	1	112.819	72.667	.000	.264
MRN0 * RaceCC	.704	1	.704	.453	.502	.002
MRN0 * Female	17.370	1	17.370	11.188	.001	.052
MRN0 * SES0	.119	1	.119	.076	.783	.000
MRN0 * Ideology0	.251	1	.251	.161	.688	.001
Error	315.168	203	1.553			
Total	4372.000	213				
Corrected Total	535.305	212				

a. R Squared = .411 (Adjusted R Squared = .385)

Parameter Estimates

Dependent Variable: DemCongressX

Parameter	B	Std. Error	t	Sig.	95% Confidence Interval	
					Lower Bound	Upper Bound
Intercept	4.421	.151	29.311	.000	4.124	4.718
MRN0	.361	.194	1.863	.064	-.021	.744
RaceCC	.101	.107	.938	.349	-.111	.313
Female	-.128	.196	-.654	.514	-.515	.258
SES0	.042	.104	.403	.688	-.163	.246
Ideology0	-.596	.070	-8.524	.000	-.734	-.458
MRN0 * RaceCC	.081	.120	.673	.502	-.156	.318
MRN0 * Female	-.781	.233	-3.345	.001	-1.241	-.320
MRN0 * SES0	.030	.110	.276	.783	-.187	.248
MRN0 * Ideology0	-.025	.061	-.402	.688	-.146	.096

Parameter Estimates

Dependent Variable: DemCongressX

Parameter	Partial Eta Squared
Intercept	.809
MRN0	.017
RaceCC	.004
Female	.002
SES0	.001
Ideology0	.264
MRN0 * RaceCC	.002
MRN0 * Female	.052
MRN0 * SES0	.000
MRN0 * Ideology0	.001

```
UNIANOVA DemCongressX WITH MRN.Low RaceCC GenderCC SES0 Ideology0
 /PRINT=ETASQ PARAMETER
 /DESIGN=MRN.Low RaceCC GenderCC SES0 Ideology0
 MRN.Low*RaceCC MRN.Low*GenderCC MRN.Low*SES0 MRN.Low*Ideology0.
```

Univariate Analysis of Variance

Notes

Output Created		15-DEC-2021 13:08:00
Comments		
Input	Data	C:<br>\Users\njs5478\Dropbox\HM and COVID\0. Revise and Resubmit\2. R and R Data\Study 1b\Study1b_Data.sav
	Active Dataset	DataSet1
	Filter	<none>
	Weight	<none>
	Split File	<none>
	N of Rows in Working Data File	241
Missing Value Handling	Definition of Missing	User-defined missing values are treated as missing.
	Cases Used	Statistics are based on all cases with valid data for all variables in the model.
Syntax		UNIANOVA DemCongressX WITH MRN.Low RaceCC GenderCC SES0 Ideology0<br>/PRINT=ETASQ PARAMETER<br>/DESIGN=MRN.Low RaceCC GenderCC SES0 Ideology0<br>MRN.Low*RaceCC MRN.Low*GenderCC MRN.Low*SES0 MRN.Low*Ideology0.
Resources	Processor Time	00:00:00.00
	Elapsed Time	00:00:00.00

Tests of Between-Subjects Effects

Dependent Variable: DemCongressX

Source	Type III Sum of Squares	df	Mean Square	F	Sig.
Corrected Model	220.137[a]	9	24.460	15.754	.000
Intercept	1014.596	1	1014.596	653.502	.000
MRN.Low	.068	1	.068	.044	.835
RaceCC	.080	1	.080	.051	.821
GenderCC	6.497	1	6.497	4.185	.042
SES0	.018	1	.018	.012	.914
Ideology0	65.733	1	65.733	42.338	.000
MRN.Low * RaceCC	.704	1	.704	.453	.502
MRN.Low * GenderCC	17.370	1	17.370	11.188	.001
MRN.Low * SES0	.119	1	.119	.076	.783
MRN.Low * Ideology0	.251	1	.251	.161	.688
Error	315.168	203	1.553		
Total	4372.000	213			
Corrected Total	535.305	212			

Tests of Between-Subjects Effects

Dependent Variable: DemCongressX

Source	Partial Eta Squared
Corrected Model	.411
Intercept	.763
MRN.Low	.000
RaceCC	.000
GenderCC	.020
SES0	.000
Ideology0	.173
MRN.Low * RaceCC	.002
MRN.Low * GenderCC	.052
MRN.Low * SES0	.000
MRN.Low * Ideology0	.001
Error	
Total	
Corrected Total	

a. R Squared = .411 (Adjusted R Squared = .385)

Parameter Estimates

Dependent Variable: DemCongressX

Parameter	B	Std. Error	t	Sig.	95% Confidence Interval	
					Lower Bound	Upper Bound
Intercept	4.382	.171	25.564	.000	4.044	4.720
MRN.Low	-.029	.139	-.209	.835	-.303	.245
RaceCC	.031	.136	.227	.821	-.237	.298
GenderCC	.274	.134	2.046	.042	.010	.538
SES0	.015	.143	.108	.914	-.267	.298
Ideology0	-.575	.088	-6.507	.000	-.749	-.401
MRN.Low * RaceCC	.081	.120	.673	.502	-.156	.318
MRN.Low * GenderCC	-.390	.117	-3.345	.001	-.620	-.160
MRN.Low * SES0	.030	.110	.276	.783	-.187	.248
MRN.Low * Ideology0	-.025	.061	-.402	.688	-.146	.096

Parameter Estimates

Dependent Variable: DemCongressX

Parameter	Partial Eta Squared
Intercept	.763
MRN.Low	.000
RaceCC	.000
GenderCC	.020
SES0	.000
Ideology0	.173
MRN.Low * RaceCC	.002
MRN.Low * GenderCC	.052
MRN.Low * SES0	.000
MRN.Low * Ideology0	.001

```
UNIANOVA DemCongressX WITH MRN.High RaceCC GenderCC SES0 Ideology0
 /PRINT=ETASQ PARAMETER
 /DESIGN=MRN.High RaceCC GenderCC SES0 Ideology0
 MRN.High*RaceCC MRN.High*GenderCC MRN.High*SES0 MRN.High*Ideology0.
```

Univariate Analysis of Variance

Notes

Output Created		15-DEC-2021 13:08:00
Comments		
Input	Data	C:\Users\njs5478\Dropbox\HM and COVID\0. Revise and Resubmit\2. R and R Data\Study 1b\Study1b_Data.sav
	Active Dataset	DataSet1
	Filter	<none>
	Weight	<none>
	Split File	<none>
	N of Rows in Working Data File	241
Missing Value Handling	Definition of Missing	User-defined missing values are treated as missing.
	Cases Used	Statistics are based on all cases with valid data for all variables in the model.
Syntax		UNIANOVA DemCongressX WITH MRN.High RaceCC GenderCC SES0 Ideology0<br>/PRINT=ETASQ PARAMETER<br>/DESIGN=MRN.High RaceCC GenderCC SES0 Ideology0<br>MRN.High*RaceCC MRN.High*GenderCC MRN.High*SES0 MRN.High*Ideology0.
Resources	Processor Time	00:00:00.02
	Elapsed Time	00:00:00.02

Tests of Between-Subjects Effects

Dependent Variable: DemCongressX

Source	Type III Sum of Squares	df	Mean Square	F	Sig.
Corrected Model	220.137[a]	9	24.460	15.754	.000
Intercept	1114.971	1	1114.971	718.153	.000
MRN.High	.068	1	.068	.044	.835
RaceCC	1.720	1	1.720	1.108	.294
GenderCC	11.567	1	11.567	7.450	.007
SES0	.376	1	.376	.242	.623
Ideology0	77.704	1	77.704	50.049	.000
MRN.High * RaceCC	.704	1	.704	.453	.502
MRN.High * GenderCC	17.370	1	17.370	11.188	.001
MRN.High * SES0	.119	1	.119	.076	.783
MRN.High * Ideology0	.251	1	.251	.161	.688
Error	315.168	203	1.553		
Total	4372.000	213			
Corrected Total	535.305	212			

Tests of Between-Subjects Effects

Dependent Variable: DemCongressX

Source	Partial Eta Squared
Corrected Model	.411
Intercept	.780
MRN.High	.000
RaceCC	.005
GenderCC	.035
SES0	.001
Ideology0	.198
MRN.High * RaceCC	.002
MRN.High * GenderCC	.052
MRN.High * SES0	.000
MRN.High * Ideology0	.001
Error	
Total	
Corrected Total	

a. R Squared = .411 (Adjusted R Squared = .385)

Parameter Estimates

Dependent Variable: DemCongressX

Parameter	B	Std. Error	t	Sig.	95% Confidence Interval Lower Bound	95% Confidence Interval Upper Bound
Intercept	4.332	.162	26.798	.000	4.013	4.651
MRN.High	-.029	.139	-.209	.835	-.303	.245
RaceCC	.171	.162	1.053	.294	-.149	.491
GenderCC	-.402	.147	-2.730	.007	-.692	-.112
SES0	.068	.138	.492	.623	-.205	.341
Ideology0	-.617	.087	-7.075	.000	-.789	-.445
MRN.High * RaceCC	.081	.120	.673	.502	-.156	.318
MRN.High * GenderCC	-.390	.117	-3.345	.001	-.620	-.160
MRN.High * SES0	.030	.110	.276	.783	-.187	.248
MRN.High * Ideology0	-.025	.061	-.402	.688	-.146	.096

Parameter Estimates

Dependent Variable: DemCongressX

Parameter	Partial Eta Squared
Intercept	.780
MRN.High	.000
RaceCC	.005
GenderCC	.035
SES0	.001
Ideology0	.198
MRN.High * RaceCC	.002
MRN.High * GenderCC	.052
MRN.High * SES0	.000
MRN.High * Ideology0	.001

```
*HM*Gender on State Leaders (without Precarious Masculinity)

UNIANOVA StateX WITH MRN0 RaceCC Male SES0 Ideology0
  /PRINT=ETASQ PARAMETER
  /DESIGN=MRN0 RaceCC Male SES0 Ideology0
  MRN0*RaceCC MRN0*Male MRN0*SES0 MRN0*Ideology0.
```

Univariate Analysis of Variance

Notes

Output Created		15-DEC-2021 13:08:00
Comments		
Input	Data	C:\Users\njs5478\Dropbox\HM and COVID\0. Revise and Resubmit\2. R and R Data\Study 1b\Study1b_Data.sav
	Active Dataset	DataSet1
	Filter	<none>
	Weight	<none>
	Split File	<none>
	N of Rows in Working Data File	241
Missing Value Handling	Definition of Missing	User-defined missing values are treated as missing.
	Cases Used	Statistics are based on all cases with valid data for all variables in the model.
Syntax		UNIANOVA StateX WITH MRN0 RaceCC Male SES0 Ideology0 /PRINT=ETASQ PARAMETER /DESIGN=MRN0 RaceCC Male SES0 Ideology0 MRN0*RaceCC MRN0*Male MRN0*SES0 MRN0*Ideology0.
Resources	Processor Time	00:00:00.02
	Elapsed Time	00:00:00.02

Tests of Between-Subjects Effects

Dependent Variable: StateX

Source	Type III Sum of Squares	df	Mean Square	F	Sig.	Partial Eta Squared
Corrected Model	75.615[a]	9	8.402	3.626	.000	.133
Intercept	1227.764	1	1227.764	529.909	.000	.713
MRN0	5.217	1	5.217	2.252	.135	.010
RaceCC	1.686	1	1.686	.728	.395	.003
Male	1.266	1	1.266	.547	.461	.003
SES0	.197	1	.197	.085	.771	.000
Ideology0	28.625	1	28.625	12.355	.001	.055
MRN0 * RaceCC	1.036	1	1.036	.447	.505	.002
MRN0 * Male	17.940	1	17.940	7.743	.006	.035
MRN0 * SES0	.288	1	.288	.124	.725	.001
MRN0 * Ideology0	4.697	1	4.697	2.027	.156	.009
Error	493.507	213	2.317			
Total	4250.000	223				
Corrected Total	569.121	222				

a. R Squared = .133 (Adjusted R Squared = .096)

Parameter Estimates

Dependent Variable: StateX

Parameter	B	Std. Error	t	Sig.	95% Confidence Interval	
					Lower Bound	Upper Bound
Intercept	4.097	.178	23.020	.000	3.746	4.448
MRN0	-.295	.196	-1.501	.135	-.682	.092
RaceCC	-.111	.130	-.853	.395	-.367	.145
Male	.171	.232	.739	.461	-.285	.628
SES0	.037	.126	.291	.771	-.212	.285
Ideology0	-.297	.085	-3.515	.001	-.464	-.131
MRN0 * RaceCC	.098	.146	.669	.505	-.190	.385
MRN0 * Male	.762	.274	2.783	.006	.222	1.301
MRN0 * SES0	.047	.133	.352	.725	-.215	.309
MRN0 * Ideology0	.102	.072	1.424	.156	-.039	.244

Parameter Estimates

Dependent Variable: StateX

Parameter	Partial Eta Squared
Intercept	.713
MRN0	.010
RaceCC	.003
Male	.003
SES0	.000
Ideology0	.055
MRN0 * RaceCC	.002
MRN0 * Male	.035
MRN0 * SES0	.001
MRN0 * Ideology0	.009

```
UNIANOVA StateX WITH MRN0 RaceCC Female SES0 Ideology0
 /PRINT=ETASQ PARAMETER
 /DESIGN=MRN0 RaceCC Female SES0 Ideology0
 MRN0*RaceCC MRN0*Female MRN0*SES0 MRN0*Ideology0.
```

Univariate Analysis of Variance

Notes

Output Created		15-DEC-2021 13:08:00
Comments		
Input	Data	C:\Users\njs5478\Dropbox\HM and COVID\0. Revise and Resubmit\2. R and R Data\Study 1b\Study1b_Data.sav
	Active Dataset	DataSet1
	Filter	<none>
	Weight	<none>
	Split File	<none>
	N of Rows in Working Data File	241
Missing Value Handling	Definition of Missing	User-defined missing values are treated as missing.
	Cases Used	Statistics are based on all cases with valid data for all variables in the model.
Syntax		UNIANOVA StateX WITH MRN0 RaceCC Female SES0 Ideology0 /PRINT=ETASQ PARAMETER /DESIGN=MRN0 RaceCC Female SES0 Ideology0 MRN0*RaceCC MRN0*Female MRN0*SES0 MRN0*Ideology0.
Resources	Processor Time	00:00:00.02
	Elapsed Time	00:00:00.02

Tests of Between-Subjects Effects

Dependent Variable: StateX

Source	Type III Sum of Squares	df	Mean Square	F	Sig.	Partial Eta Squared
Corrected Model	75.615[a]	9	8.402	3.626	.000	.133
Intercept	1304.568	1	1304.568	563.058	.000	.726
MRN0	9.225	1	9.225	3.982	.047	.018
RaceCC	1.686	1	1.686	.728	.395	.003
Female	1.266	1	1.266	.547	.461	.003
SES0	.197	1	.197	.085	.771	.000
Ideology0	28.625	1	28.625	12.355	.001	.055
MRN0 * RaceCC	1.036	1	1.036	.447	.505	.002
MRN0 * Female	17.940	1	17.940	7.743	.006	.035
MRN0 * SES0	.288	1	.288	.124	.725	.001
MRN0 * Ideology0	4.697	1	4.697	2.027	.156	.009
Error	493.507	213	2.317			
Total	4250.000	223				
Corrected Total	569.121	222				

a. R Squared = .133 (Adjusted R Squared = .096)

Parameter Estimates

Dependent Variable: StateX

Parameter	B	Std. Error	t	Sig.	95% Confidence Interval	
					Lower Bound	Upper Bound
Intercept	4.268	.180	23.729	.000	3.914	4.623
MRN0	.467	.234	1.995	.047	.006	.928
RaceCC	-.111	.130	-.853	.395	-.367	.145
Female	-.171	.232	-.739	.461	-.628	.285
SES0	.037	.126	.291	.771	-.212	.285
Ideology0	-.297	.085	-3.515	.001	-.464	-.131
MRN0 * RaceCC	.098	.146	.669	.505	-.190	.385
MRN0 * Female	-.762	.274	-2.783	.006	-1.301	-.222
MRN0 * SES0	.047	.133	.352	.725	-.215	.309
MRN0 * Ideology0	.102	.072	1.424	.156	-.039	.244

Parameter Estimates

Dependent Variable: StateX

Parameter	Partial Eta Squared
Intercept	.726
MRN0	.018
RaceCC	.003
Female	.003
SES0	.000
Ideology0	.055
MRN0 * RaceCC	.002
MRN0 * Female	.035
MRN0 * SES0	.001
MRN0 * Ideology0	.009

```
UNIANOVA StateX WITH MRN.Low RaceCC GenderCC SES0 Ideology0
 /PRINT=ETASQ PARAMETER
 /DESIGN=MRN.Low RaceCC GenderCC SES0 Ideology0
 MRN.Low*RaceCC MRN.Low*GenderCC MRN.Low*SES0 MRN.Low*Ideology0.
```

Univariate Analysis of Variance

Notes

Output Created		15-DEC-2021 13:08:00
Comments		
Input	Data	C:\Users\njs5478\Dropbox\HM and COVID\0. Revise and Resubmit\2. R and R Data\Study 1b\Study1b_Data.sav
	Active Dataset	DataSet1
	Filter	<none>
	Weight	<none>
	Split File	<none>
	N of Rows in Working Data File	241
Missing Value Handling	Definition of Missing	User-defined missing values are treated as missing.
	Cases Used	Statistics are based on all cases with valid data for all variables in the model.
Syntax		UNIANOVA StateX WITH MRN.Low RaceCC GenderCC SES0 Ideology0<br>/PRINT=ETASQ PARAMETER<br>/DESIGN=MRN.Low RaceCC GenderCC SES0 Ideology0<br>MRN.Low*RaceCC MRN.Low*GenderCC MRN.Low*SES0 MRN.Low*Ideology0.
Resources	Processor Time	00:00:00.02
	Elapsed Time	00:00:00.02

Tests of Between-Subjects Effects

Dependent Variable: StateX

Source	Type III Sum of Squares	df	Mean Square	F	Sig.
Corrected Model	75.615[a]	9	8.402	3.626	.000
Intercept	939.410	1	939.410	405.454	.000
MRN.Low	.613	1	.613	.265	.607
RaceCC	3.198	1	3.198	1.380	.241
GenderCC	5.755	1	5.755	2.484	.117
SES0	.001	1	.001	.000	.982
Ideology0	31.683	1	31.683	13.674	.000
MRN.Low * RaceCC	1.036	1	1.036	.447	.505
MRN.Low * GenderCC	17.940	1	17.940	7.743	.006
MRN.Low * SES0	.288	1	.288	.124	.725
MRN.Low * Ideology0	4.697	1	4.697	2.027	.156
Error	493.507	213	2.317		
Total	4250.000	223			
Corrected Total	569.121	222			

Tests of Between-Subjects Effects

Dependent Variable: StateX

Source	Partial Eta Squared
Corrected Model	.133
Intercept	.656
MRN.Low	.001
RaceCC	.006
GenderCC	.012
SES0	.000
Ideology0	.060
MRN.Low * RaceCC	.002
MRN.Low * GenderCC	.035
MRN.Low * SES0	.001
MRN.Low * Ideology0	.009
Error	
Total	
Corrected Total	

a. R Squared = .133 (Adjusted R Squared = .096)

Parameter Estimates

Dependent Variable: StateX

Parameter	B	Std. Error	t	Sig.	95% Confidence Interval	
					Lower Bound	Upper Bound
Intercept	4.108	.204	20.136	.000	3.706	4.510
MRN.Low	.086	.167	.514	.607	-.243	.415
RaceCC	-.195	.166	-1.175	.241	-.523	.132
GenderCC	.244	.155	1.576	.117	-.061	.549
SES0	-.004	.174	-.022	.982	-.347	.340
Ideology0	-.386	.104	-3.698	.000	-.591	-.180
MRN.Low * RaceCC	.098	.146	.669	.505	-.190	.385
MRN.Low * GenderCC	-.381	.137	-2.783	.006	-.650	-.111
MRN.Low * SES0	.047	.133	.352	.725	-.215	.309
MRN.Low * Ideology0	.102	.072	1.424	.156	-.039	.244

Parameter Estimates

Dependent Variable: StateX

Parameter	Partial Eta Squared
Intercept	.656
MRN.Low	.001
RaceCC	.006
GenderCC	.012
SES0	.000
Ideology0	.060
MRN.Low * RaceCC	.002
MRN.Low * GenderCC	.035
MRN.Low * SES0	.001
MRN.Low * Ideology0	.009

```
UNIANOVA StateX WITH MRN.High RaceCC GenderCC SES0 Ideology0
 /PRINT=ETASQ PARAMETER
 /DESIGN=MRN.High RaceCC GenderCC SES0 Ideology0
 MRN.High*RaceCC MRN.High*GenderCC MRN.High*SES0 MRN.High*Ideology0.
```

Univariate Analysis of Variance

Notes

Output Created		15-DEC-2021 13:08:00
Comments		
Input	Data	C:\Users\njs5478\Dropbox\HM and COVID\0. Revise and Resubmit\2. R and R Data\Study 1b\Study1b_Data.sav
	Active Dataset	DataSet1
	Filter	<none>
	Weight	<none>
	Split File	<none>
	N of Rows in Working Data File	241
Missing Value Handling	Definition of Missing	User-defined missing values are treated as missing.
	Cases Used	Statistics are based on all cases with valid data for all variables in the model.
Syntax		UNIANOVA StateX WITH MRN.High RaceCC GenderCC SES0 Ideology0 /PRINT=ETASQ PARAMETER /DESIGN=MRN.High RaceCC GenderCC SES0 Ideology0 MRN.High*RaceCC MRN.High*GenderCC MRN.High*SES0 MRN.High*Ideology0.
Resources	Processor Time	00:00:00.02
	Elapsed Time	00:00:00.03

Tests of Between-Subjects Effects

Dependent Variable: StateX

Source	Type III Sum of Squares	df	Mean Square	F	Sig.
Corrected Model	75.615[a]	9	8.402	3.626	.000
Intercept	1121.260	1	1121.260	483.942	.000
MRN.High	.613	1	.613	.265	.607
RaceCC	.042	1	.042	.018	.893
GenderCC	12.923	1	12.923	5.578	.019
SES0	.496	1	.496	.214	.644
Ideology0	9.061	1	9.061	3.911	.049
MRN.High * RaceCC	1.036	1	1.036	.447	.505
MRN.High * GenderCC	17.940	1	17.940	7.743	.006
MRN.High * SES0	.288	1	.288	.124	.725
MRN.High * Ideology0	4.697	1	4.697	2.027	.156
Error	493.507	213	2.317		
Total	4250.000	223			
Corrected Total	569.121	222			

Tests of Between-Subjects Effects

Dependent Variable: StateX

Source	Partial Eta Squared
Corrected Model	.133
Intercept	.694
MRN.High	.001
RaceCC	.000
GenderCC	.026
SES0	.001
Ideology0	.018
MRN.High * RaceCC	.002
MRN.High * GenderCC	.035
MRN.High * SES0	.001
MRN.High * Ideology0	.009
Error	
Total	
Corrected Total	

a. R Squared = .133 (Adjusted R Squared = .096)

Parameter Estimates

Dependent Variable: StateX

Parameter	B	Std. Error	t	Sig.	95% Confidence Interval	
					Lower Bound	Upper Bound
Intercept	4.257	.194	21.999	.000	3.876	4.638
MRN.High	.086	.167	.514	.607	-.243	.415
RaceCC	-.026	.195	-.135	.893	-.411	.358
GenderCC	-.415	.176	-2.362	.019	-.762	-.069
SES0	.077	.167	.463	.644	-.252	.407
Ideology0	-.209	.106	-1.978	.049	-.417	-.001
MRN.High * RaceCC	.098	.146	.669	.505	-.190	.385
MRN.High * GenderCC	-.381	.137	-2.783	.006	-.650	-.111
MRN.High * SES0	.047	.133	.352	.725	-.215	.309
MRN.High * Ideology0	.102	.072	1.424	.156	-.039	.244

Parameter Estimates

Dependent Variable: StateX

Parameter	Partial Eta Squared
Intercept	.694
MRN.High	.001
RaceCC	.000
GenderCC	.026
SES0	.001
Ideology0	.018
MRN.High * RaceCC	.002
MRN.High * GenderCC	.035
MRN.High * SES0	.001
MRN.High * Ideology0	.009
