# Supplementary material for: Men’s and women’s endorsement of hegemonic masculinity and responses to COVID-19
Source: J Health Psychol. 2022 Mar 11;28(3):251–66. doi: 10.1177/13591053221081905 (PMC9982413; doi:10.1177/13591053221081905)
Supplement: sj-pdf-3-hpq-10.1177_13591053221081905 – for Men’s and women’s endorsement of hegemonic masculinity and responses to COVID-19 [file sj-pdf-3-hpq-10.1177_13591053221081905.pdf]

```
 * Encoding: UTF-8.
**Study 2a Syntax**

*No participants meet exclusion criteria

***Variable Creation***

**Male Role Norms**

RECODE Tough_8 Fem_6 (1=7) (2=6) (3=5) (4=4) (5=3) (6=2) (7=1) INTO Tough_8_Recode Fem_6_
Recode.
EXECUTE.

COMPUTE MRN=(Power_1 + Power_2 + Power_3 + Power_4 + Power_5 + Power_6 + Power_7 + Power_
8 +
    Power_9  + Power_10 + Power_11 + Tough_1 + Tough_2 + Tough_3 + Tough_4 + Tough_5 + To
ugh_6 +
    Tough_7 + Tough_8_Recode + Fem_1 + Fem_2 + Fem_3 + Fem_4 + Fem_5 + Fem_6_Recode + Fem
_7)/26.
EXECUTE.

COMPUTE Power=(Power_1 + Power_2 + Power_3 + Power_4 + Power_5 + Power_6 + Power_7 + Powe
r_8 +
    Power_9  + Power_10 + Power_11)/11.
EXECUTE.

COMPUTE Tough=(Tough_1 + Tough_2 + Tough_3 + Tough_4 + Tough_5 + Tough_6 +
    Tough_7 + Tough_8_Recode)/8.
EXECUTE.

COMPUTE Fem=(Fem_1 + Fem_2 + Fem_3 + Fem_4 + Fem_5 + Fem_6_Recode + Fem_7)/7.
EXECUTE.

RELIABILITY
  /VARIABLES=Power_1 Power_2 Power_3 Power_4 Power_5 Power_6 Power_7 Power_8 Power_9 Powe
r_10
    Power_11 Tough_1 Tough_2 Tough_3 Tough_4 Tough_5 Tough_6 Tough_7 Tough_8_Recode Fem_1
 Fem_2 Fem_3
    Fem_4 Fem_5 Fem_6_Recode Fem_7
  /SCALE('ALL VARIABLES') ALL
  /MODEL=ALPHA.
```

Reliability

Notes

Output Created		15-DEC-2021 13:09:40
Comments		
Input	Data	C: \Users\njs5478\Dropbox\HM and COVID\0. Revise and Resubmit\2. R and R Data\Study 2a\Study2a_Data.sav
	Active Dataset	DataSet1
	Filter	<none>
	Weight	<none>
	Split File	<none>
	N of Rows in Working Data File	188
	Matrix Input	
Missing Value Handling	Definition of Missing	User-defined missing values are treated as missing.
	Cases Used	Statistics are based on all cases with valid data for all variables in the procedure.
Syntax		RELIABILITY /VARIABLES=Power_1 Power_2 Power_3 Power_4 Power_5 Power_6 Power_7 Power_8 Power_9 Power_10 Power_11 Tough_1 Tough_2 Tough_3 Tough_4 Tough_5 Tough_6 Tough_7 Tough_8_Recode Fem_1 Fem_2 Fem_3 Fem_4 Fem_5 Fem_6_Recode Fem_7 /SCALE('ALL VARIABLES') ALL /MODEL=ALPHA.
Resources	Processor Time	00:00:00.00
	Elapsed Time	00:00:00.00

```
[DataSet1] C:\Users\njs5478\Dropbox\HM and COVID\0. Revise and Resubmit\2. R and R Data\Study 2a\Study2a_Data.sav
```

Scale: ALL VARIABLES

Case Processing Summary

		N	%
Cases	Valid	188	100.0
	Excluded[a]	0	.0
	Total	188	100.0

a. Listwise deletion based on all variables in the procedure.

Reliability Statistics

Cronbach's Alpha	N of Items
.908	26

```
 **Risk During COVID-19*

*Note, we removed the 3 "help-based" items from Studies 2a and 2b

RECODE Risk1 Risk3 Risk4 Risk8 Risk11 (1=7) (2=6) (3=5) (4=4) (5=3) (6=2) (7=1) INTO Risk
1_R Risk3_R Risk4_R Risk8_R Risk11_R.
EXECUTE.

RELIABILITY
  /VARIABLES=Risk1_R Risk2 Risk3_R Risk4_R Risk5 Risk6 Risk7 Risk8_R Risk9 Risk10 Risk11_
R Risk12 Risk16 Risk17 Risk18
  /SCALE('ALL VARIABLES') ALL
  /MODEL=ALPHA.
```

Reliability

Notes

Output Created		15-DEC-2021 13:09:40
Comments		
Input	Data	C: \Users\njs5478\Dropbox\HM and COVID\0. Revise and Resubmit\2. R and R Data\Study 2a\Study2a_Data.sav
	Active Dataset	DataSet1
	Filter	<none>
	Weight	<none>
	Split File	<none>
	N of Rows in Working Data File	188
	Matrix Input	
Missing Value Handling	Definition of Missing	User-defined missing values are treated as missing.
	Cases Used	Statistics are based on all cases with valid data for all variables in the procedure.
Syntax		RELIABILITY /VARIABLES=Risk1_R Risk2 Risk3_R Risk4_R Risk5 Risk6 Risk7 Risk8_R Risk9 Risk10 Risk11_R Risk12 Risk16 Risk17 Risk18 /SCALE('ALL VARIABLES') ALL /MODEL=ALPHA.
Resources	Processor Time	00:00:00.00
	Elapsed Time	00:00:00.00

Scale: ALL VARIABLES

Case Processing Summary

		N	%
Cases	Valid	188	100.0
	Excluded[a]	0	.0
	Total	188	100.0

a. Listwise deletion based on all variables in the procedure.

Reliability Statistics

Cronbach's Alpha	N of Items
.864	15

```
COMPUTE Risk_Rules = (Risk1_R + Risk2 + Risk3_R + Risk4_R + Risk5 + Risk6 + Risk7 + Risk8
_R + Risk9 + Risk10 + Risk11_R + Risk12 + Risk16 + Risk17 + Risk18)/15.

**Coronavirus Concern**

RECODE Concern3 (1=7) (2=6) (3=5) (4=4) (5=3) (6=2) (7=1) INTO Concern3_R.
EXECUTE.

RELIABILITY
  /VARIABLES=Concern1 Concern2 Concern3_R Concern4 Concern5 Concern6
  /SCALE('ALL VARIABLES') ALL
  /MODEL=ALPHA
  /SUMMARY=TOTAL.
```

Reliability

Notes

Output Created		15-DEC-2021 13:09:40
Comments		
Input	Data	C: \Users\njs5478\Dropbox\HM and COVID\0. Revise and Resubmit\2. R and R Data\Study 2a\Study2a_Data.sav
	Active Dataset	DataSet1
	Filter	<none>
	Weight	<none>
	Split File	<none>
	N of Rows in Working Data File	188
	Matrix Input	
Missing Value Handling	Definition of Missing	User-defined missing values are treated as missing.
	Cases Used	Statistics are based on all cases with valid data for all variables in the procedure.
Syntax		RELIABILITY /VARIABLES=Concern1 Concern2 Concern3_R Concern4 Concern5 Concern6 /SCALE('ALL VARIABLES') ALL /MODEL=ALPHA /SUMMARY=TOTAL.
Resources	Processor Time	00:00:00.00
	Elapsed Time	00:00:00.00

Scale: ALL VARIABLES

Case Processing Summary

		N	%
Cases	Valid	188	100.0
	Excluded[a]	0	.0
	Total	188	100.0

a. Listwise deletion based on all variables in the procedure.

Reliability Statistics

Cronbach's Alpha	N of Items
.862	6

Item-Total Statistics

	Scale Mean if Item Deleted	Scale Variance if Item Deleted	Corrected Item-Total Correlation	Cronbach's Alpha if Item Deleted
Please indicate your agreement/disagreement with each statement using the scale provided: - Thinking about the coronavirus (COVID-19) makes me feel threatened.	18.7553	49.009	.695	.834
Please indicate your agreement/disagreement with each statement using the scale provided: - I am afraid of the coronavirus (COVID-19).	18.4468	44.142	.772	.816
Concern3_R	17.7553	48.485	.548	.860
Please indicate your agreement/disagreement with each statement using the scale provided: - I am worried that I or people I love will get sick from the coronavirus (COVID-19).	16.6170	51.382	.535	.859
Please indicate your agreement/disagreement with each statement using the scale provided: - I am stressed around other people because I worry I'll catch the coronavirus (COVID-19).	18.8936	46.598	.752	.822
Please indicate your agreement/disagreement with each statement using the scale provided: - I have tried hard to avoid other people because I don't want to get sick.	18.3883	46.741	.655	.839

```
COMPUTE Concern_Tot=mean(concern1, Concern2, Concern3_R, Concern4, Concern5, Concern6).

**Financial

RECODE Finance3 (1=7) (2=6) (3=5) (4=4) (5=3) (6=2) (7=1) INTO Finance3_R.
EXECUTE.

RELIABILITY
  /VARIABLES=Finance1 Finance2 Finance3_R
  /SCALE('ALL VARIABLES') ALL
  /MODEL=ALPHA
  /SUMMARY=TOTAL.
```

Reliability

Notes

Output Created		15-DEC-2021 13:09:40
Comments		
Input	Data	C:\Users\njs5478\Dropbox\HM and COVID\0. Revise and Resubmit\2. R and R Data\Study 2a\Study2a_Data.sav
	Active Dataset	DataSet1
	Filter	<none>
	Weight	<none>
	Split File	<none>
	N of Rows in Working Data File	188
	Matrix Input	
Missing Value Handling	Definition of Missing	User-defined missing values are treated as missing.
	Cases Used	Statistics are based on all cases with valid data for all variables in the procedure.
Syntax		RELIABILITY /VARIABLES=Finance1 Finance2 Finance3_R /SCALE('ALL VARIABLES') ALL /MODEL=ALPHA /SUMMARY=TOTAL.

Notes

Resources	Processor Time	00:00:00.02
	Elapsed Time	00:00:00.02

Scale: ALL VARIABLES

Case Processing Summary

		N	%
Cases	Valid	188	100.0
	Excluded[a]	0	.0
	Total	188	100.0

a. Listwise deletion based on all variables in the procedure.

Reliability Statistics

Cronbach's Alpha	N of Items
.773	3

Item-Total Statistics

	Scale Mean if Item Deleted	Scale Variance if Item Deleted	Corrected Item-Total Correlation	Cronbach's Alpha if Item Deleted
Please indicate your agreement/disagreement with each statement using the scale provided: - The Coronavirus (COVID-19) has impacted me negatively from a financial point of view.	7.4362	8.835	.742	.545
Please indicate your agreement/disagreement with each statement using the scale provided: - I have lost job-related income due to the Coronavirus (COVID-19).	8.2979	9.889	.535	.775
Finance3_R	6.8830	9.826	.559	.747

```
 COMPUTE Finance_Tot=mean(Finance1, Finance2, Finance3_R).

*Resources

*Note. We removed the resource items from Studies 2a and 2b

*Psychology

RECODE Psychology3 (1=7) (2=6) (3=5) (4=4) (5=3) (6=2) (7=1) INTO Psychology3_R.
EXECUTE.

RELIABILITY
  /VARIABLES=Psychology1 Psychology2 Psychology3_R
  /SCALE('ALL VARIABLES') ALL
  /MODEL=ALPHA
  /SUMMARY=TOTAL.
```

Reliability

Notes

Output Created		15-DEC-2021 13:09:40
Comments		
Input	Data	C:\Users\njs5478\Dropbox\HM and COVID\0. Revise and Resubmit\2. R and R Data\Study 2a\Study2a_Data.sav
	Active Dataset	DataSet1
	Filter	<none>
	Weight	<none>
	Split File	<none>
	N of Rows in Working Data File	188
	Matrix Input	
Missing Value Handling	Definition of Missing	User-defined missing values are treated as missing.
	Cases Used	Statistics are based on all cases with valid data for all variables in the procedure.

Notes

Syntax		RELIABILITY<br><br>/VARIABLES=Psychology1 Psychology2 Psychology3_R<br>/SCALE('ALL VARIABLES') ALL<br>/MODEL=ALPHA<br>/SUMMARY=TOTAL.
Resources	Processor Time	00:00:00.02
	Elapsed Time	00:00:00.02

Scale: ALL VARIABLES

Case Processing Summary

		N	%
Cases	Valid	188	100.0
	Excluded[a]	0	.0
	Total	188	100.0

a. Listwise deletion based on all variables in the procedure.

Reliability Statistics

Cronbach's Alpha	N of Items
.852	3

Item-Total Statistics

	Scale Mean if Item Deleted	Scale Variance if Item Deleted	Corrected Item-Total Correlation	Cronbach's Alpha if Item Deleted
Please indicate your agreement/disagreement with each statement using the scale provided: - I have become depressed because of the Coronavirus (COVID-19).	9.6649	8.417	.692	.825
Please indicate your agreement/disagreement with each statement using the scale provided: - The Coronavirus (COVID-19) outbreak has impacted my psychological health negatively.	8.8085	8.626	.792	.729
Psychology3_R	8.7713	9.183	.689	.823

```
 COMPUTE Psychology_Tot=mean(Psychology1, Psychology2, Psychology3_R).

*Mandates

RELIABILITY
  /VARIABLES=Mandate1 Mandate2 Mandate3 Mandate4 Mandate5 Mandate6
  /SCALE('ALL VARIABLES') ALL
  /MODEL=ALPHA
  /SUMMARY=TOTAL.
```

Reliability

Notes

Output Created		15-DEC-2021 13:09:40
Comments		
Input	Data	C:\Users\njs5478\Dropbox\HM and COVID\0. Revise and Resubmit\2. R and R Data\Study 2a\Study2a_Data.sav
	Active Dataset	DataSet1
	Filter	<none>
	Weight	<none>
	Split File	<none>
	N of Rows in Working Data File	188
	Matrix Input	
Missing Value Handling	Definition of Missing	User-defined missing values are treated as missing.
	Cases Used	Statistics are based on all cases with valid data for all variables in the procedure.
Syntax		RELIABILITY /VARIABLES=Mandate1 Mandate2 Mandate3 Mandate4 Mandate5 Mandate6 /SCALE('ALL VARIABLES') ALL /MODEL=ALPHA /SUMMARY=TOTAL.
Resources	Processor Time	00:00:00.00
	Elapsed Time	00:00:00.00

Scale: ALL VARIABLES

Case Processing Summary

		N	%
Cases	Valid	188	100.0
	Excluded[a]	0	.0
	Total	188	100.0

a. Listwise deletion based on all variables in the procedure.

Reliability Statistics

Cronbach's Alpha	N of Items
.940	6

Item-Total Statistics

	Scale Mean if Item Deleted	Scale Variance if Item Deleted	Corrected Item-Total Correlation	Cronbach's Alpha if Item Deleted
Please indicate your agreement/disagreement with each statement using the scale provided: - Employers with over 100 employees should require that all workers get the COVID-19 vaccine.	23.41	76.008	.892	.919
Please indicate your agreement/disagreement with each statement using the scale provided: - Health care workers should be required to get the COVID-19 vaccine or be terminated.	23.15	75.389	.871	.922
Please indicate your agreement/disagreement with each statement using the scale provided: - School employees should be required to get the COVID-19 vaccine or be terminated.	23.53	75.876	.866	.923
Please indicate your agreement/disagreement with each statement using the scale provided: - People should continue to be required to wear masks indoors.	23.89	85.319	.605	.953

Item-Total Statistics

	Scale Mean if Item Deleted	Scale Variance if Item Deleted	Corrected Item-Total Correlation	Cronbach's Alpha if Item Deleted
Please indicate your agreement/disagreement with each statement using the scale provided: - People should have to provide proof of vaccination to enter entertainment venues (e.g., concerts).	23.35	76.923	.840	.926
Please indicate your agreement/disagreement with each statement using the scale provided: - People should have to provide proof of vaccination to travel.	22.97	77.871	.847	.925

```
 COMPUTE Mandate_Tot=mean(Mandate1, Mandate2, Mandate3, Mandate4, Mandate5, Mandate6).

COMPUTE Vaccine_Tot=mean(Mandate1, Mandate2, Mandate3, Mandate5, Mandate6).

*Conspiracy

 RELIABILITY
  /VARIABLES=Conspiracy1 Conspiracy2 Conspiracy3 Conspiracy4 Conspiracy5 Conspiracy6 Cons
piracy7 Conspiracy8 Conspiracy9
  /SCALE('ALL VARIABLES') ALL
  /MODEL=ALPHA
  /SUMMARY=TOTAL.
```

Reliability

Notes

Output Created		15-DEC-2021 13:09:40
Comments		
Input	Data	C:\Users\njs5478\Dropbox\HM and COVID\0. Revise and Resubmit\2. R and R Data\Study 2a\Study2a_Data.sav
	Active Dataset	DataSet1
	Filter	<none>
	Weight	<none>
	Split File	<none>
	N of Rows in Working Data File	188
	Matrix Input	
Missing Value Handling	Definition of Missing	User-defined missing values are treated as missing.
	Cases Used	Statistics are based on all cases with valid data for all variables in the procedure.
Syntax		RELIABILITY /VARIABLES=Conspiracy1 Conspiracy2 Conspiracy3 Conspiracy4 Conspiracy5 Conspiracy6 Conspiracy7 Conspiracy8 Conspiracy9 /SCALE('ALL VARIABLES') ALL /MODEL=ALPHA...
Resources	Processor Time	00:00:00.00
	Elapsed Time	00:00:00.00

Scale: ALL VARIABLES

Case Processing Summary

		N	%
Cases	Valid	188	100.0
	Excluded[a]	0	.0
	Total	188	100.0

a. Listwise deletion based on all variables in the procedure.

Reliability Statistics

Cronbach's Alpha	N of Items
.881	9

Item-Total Statistics

	Scale Mean if Item Deleted	Scale Variance if Item Deleted	Corrected Item-Total Correlation	Cronbach's Alpha if Item Deleted
Please indicate, using the scale provided, how credible you believe each theory about COVID-19 to be: - COVID-19 has been released by the US government to destabilize China	12.60	26.787	.725	.862
Please indicate, using the scale provided, how credible you believe each theory about COVID-19 to be: - COVID-19 was developed to control population growth	12.36	25.270	.732	.858

Item-Total Statistics

	Scale Mean if Item Deleted	Scale Variance if Item Deleted	Corrected Item-Total Correlation	Cronbach's Alpha if Item Deleted
Please indicate, using the scale provided, how credible you believe each theory about COVID-19 to be: - COVID-19 is a way to cover up the effects of 5G towers	12.68	26.710	.704	.863
Please indicate, using the scale provided, how credible you believe each theory about COVID-19 to be: - COVID-19 was developed by pharmaceutical companies	12.29	24.965	.634	.868
Please indicate, using the scale provided, how credible you believe each theory about COVID-19 to be: - Together with the vaccine, a chip will be injected to permanently track people	12.69	27.126	.742	.863
Please indicate, using the scale provided, how credible you believe each theory about COVID-19 to be: - The Chinese government lies about the number of COVID-19 deaths in China	11.29	25.618	.381	.906

Item-Total Statistics

	Scale Mean if Item Deleted	Scale Variance if Item Deleted	Corrected Item-Total Correlation	Cronbach's Alpha if Item Deleted
Please indicate, using the scale provided, how credible you believe each theory about COVID-19 to be: - COVID-19 was spread deliberately among the Chinese population	12.23	24.726	.662	.865
Please indicate, using the scale provided, how credible you believe each theory about COVID-19 to be: - COVID-19 was developed by the Chinese government to damage the Western world and its economies, in order to become the strongest economy in the world	12.41	25.752	.728	.859
Please indicate, using the scale provided, how credible you believe each theory about COVID-19 to be: - COVID-19 was developed by climate activists to counteract climate change	12.68	27.331	.716	.865

```
COMPUTE Conspiracy_Tot=mean(Conspiracy1, Conspiracy2, Conspiracy3, Conspiracy4, Conspirac
y5, Conspiracy6, Conspiracy7, Conspiracy8, Conspiracy9).

*National Identity

 RELIABILITY
  /VARIABLES=National1 National2
  /SCALE('ALL VARIABLES') ALL
  /MODEL=ALPHA
  /SUMMARY=TOTAL.
```

Reliability

Notes

Output Created		15-DEC-2021 13:09:40
Comments		
Input	Data	C: \Users\njs5478\Dropbox\HM and COVID\0. Revise and Resubmit\2. R and R Data\Study 2a\Study2a_Data.sav
	Active Dataset	DataSet1
	Filter	<none>
	Weight	<none>
	Split File	<none>
	N of Rows in Working Data File	188
	Matrix Input	
Missing Value Handling	Definition of Missing	User-defined missing values are treated as missing.
	Cases Used	Statistics are based on all cases with valid data for all variables in the procedure.
Syntax		RELIABILITY /VARIABLES=National1 National2 /SCALE('ALL VARIABLES') ALL /MODEL=ALPHA /SUMMARY=TOTAL.
Resources	Processor Time	00:00:00.00
	Elapsed Time	00:00:00.00

Scale: ALL VARIABLES

Case Processing Summary

		N	%
Cases	Valid	188	100.0
	Excluded[a]	0	.0
	Total	188	100.0

a. Listwise deletion based on all variables in the procedure.

Reliability Statistics

Cronbach's Alpha	N of Items
.784	2

Item-Total Statistics

	Scale Mean if Item Deleted	Scale Variance if Item Deleted	Corrected Item-Total Correlation	Cronbach's Alpha if Item Deleted
I identify as American.	4.78	3.198	.651	.
Being an American is an important reflection of who I am.	6.09	2.436	.651	.

```
COMPUTE National_Tot=mean(National1, National2).

**Political Identity

CORRELATIONS
  /VARIABLES=PParty PIdeology
  /PRINT=TWOTAIL NOSIG FULL
  /MISSING=PAIRWISE.
```

Correlations

Notes

Output Created		15-DEC-2021 13:09:40
Comments		
Input	Data	C: \Users\njs5478\Dropbox\HM and COVID\0. Revise and Resubmit\2. R and R Data\Study 2a\Study2a_Data.sav
	Active Dataset	DataSet1
	Filter	<none>
	Weight	<none>
	Split File	<none>
	N of Rows in Working Data File	188
Missing Value Handling	Definition of Missing	User-defined missing values are treated as missing.
	Cases Used	Statistics for each pair of variables are based on all the cases with valid data for that pair.
Syntax		CORRELATIONS /VARIABLES=PParty PIdeology /PRINT=TWOTAIL NOSIG FULL /MISSING=PAIRWISE.
Resources	Processor Time	00:00:00.00
	Elapsed Time	00:00:00.00

Correlations

		Which of the following best describes your political party affiliation?	Which of the following best describes your political ideology?
Which of the following best describes your political party affiliation?	Pearson Correlation	1	.785**
	Sig. (2-tailed)		.000
	N	188	188
Which of the following best describes your political ideology?	Pearson Correlation	.785**	1
	Sig. (2-tailed)	.000	
	N	188	188

**. Correlation is significant at the 0.01 level (2-tailed).

```
**Descriptives and Frequencies**

FREQUENCIES VARIABLES=PParty PIdeology SES Education Gender Race Age MRN Finance_Tot Psyc
hology_Tot Mandate_Tot Conspiracy_Tot National_Tot
  /STATISTICS=STDDEV MINIMUM MAXIMUM MEAN
  /ORDER=ANALYSIS.
```

Frequencies

Notes

Output Created		15-DEC-2021 13:09:40
Comments		
Input	Data	C:\Users\njs5478\Dropbox\HM and COVID\0. Revise and Resubmit\2. R and R Data\Study 2a\Study2a_Data.sav
	Active Dataset	DataSet1
	Filter	<none>
	Weight	<none>
	Split File	<none>
	N of Rows in Working Data File	188
Missing Value Handling	Definition of Missing	User-defined missing values are treated as missing.
	Cases Used	Statistics are based on all cases with valid data.
Syntax		FREQUENCIES VARIABLES=PParty PIdeology SES Education Gender Race Age MRN Finance_Tot Psychology_Tot Mandate_Tot Conspiracy_Tot National_Tot /STATISTICS=STDDEV MINIMUM MAXIMUM MEAN...
Resources	Processor Time	00:00:00.00
	Elapsed Time	00:00:00.00

Statistics

		Which of the following best describes your political party affiliation?	Which of the following best describes your political ideology?	Self Reported Socioeconomic Status	Please indicate the highest level of education that you have received:	Gender
N	Valid	188	188	188	188	188
	Missing	0	0	0	0	0
Mean		2.76	3.55	3.35	2.77	.0000
Std. Deviation		1.220	1.514	.734	.634	1.00267
Minimum		1	1	1	2	-1.00
Maximum		5	7	5	6	1.00

Statistics

		Racial Identity - Selected Choice	Age	MRN	Finance_Tot	Psychology_Tot	Mandate_Tot
N	Valid	188	188	188	188	188	188
	Missing	0	0	0	0	0	0
Mean		1.96	18.84	3.2162	3.7695	4.5408	4.6764
Std. Deviation		1.885	2.262	.85462	1.46175	1.42638	1.75465
Minimum		1	18	1.27	1.00	1.00	1.00
Maximum		9	47	5.42	7.00	7.00	7.00

Statistics

		Conspiracy_Tot	National_Tot
N	Valid	188	188
	Missing	0	0
Mean		1.5449	5.4362
Std. Deviation		.63305	1.52209
Minimum		1.00	1.00
Maximum		5.00	7.00

Frequency Table

Which of the following best describes your political party affiliation?

		Frequency	Percent	Valid Percent	Cumulative Percent
Valid	Democrat	32	17.0	17.0	17.0
	Democrat Leaning	53	28.2	28.2	45.2
	Independent	49	26.1	26.1	71.3
	Republican Leaning	36	19.1	19.1	90.4
	Republican	18	9.6	9.6	100.0
	Total	188	100.0	100.0	

Which of the following best describes your political ideology?

		Frequency	Percent	Valid Percent	Cumulative Percent
Valid	Very Liberal	9	4.8	4.8	4.8
	Liberal	52	27.7	27.7	32.4
	Somewhat Liberal	34	18.1	18.1	50.5
	Neither Liberal Nor Conservative	40	21.3	21.3	71.8
	Somewhat Conservative	28	14.9	14.9	86.7
	Conservative	22	11.7	11.7	98.4
	Very Conservative	3	1.6	1.6	100.0
	Total	188	100.0	100.0	

Self Reported Socioeconomic Status

		Frequency	Percent	Valid Percent	Cumulative Percent
Valid	Poor	1	.5	.5	.5
	Working Class	19	10.1	10.1	10.6
	Middle Class	88	46.8	46.8	57.4
	Upper Middle Class	73	38.8	38.8	96.3
	Upper Class	7	3.7	3.7	100.0
	Total	188	100.0	100.0	

Please indicate the highest level of education that you have received:

		Frequency	Percent	Valid Percent	Cumulative Percent
Valid	Completed high school	56	29.8	29.8	29.8
	Some college	126	67.0	67.0	96.8
	Bachelor's Degree	5	2.7	2.7	99.5
	Master's Degree	1	.5	.5	100.0
	Total	188	100.0	100.0	

Gender

		Frequency	Percent	Valid Percent	Cumulative Percent
Valid	-1.00	94	50.0	50.0	50.0
	1.00	94	50.0	50.0	100.0
	Total	188	100.0	100.0	

Racial Identity - Selected Choice

		Frequency	Percent	Valid Percent	Cumulative Percent
Valid	White/Caucasian	132	70.2	70.2	70.2
	Black/African American	10	5.3	5.3	75.5
	Asian	25	13.3	13.3	88.8
	Native American	1	.5	.5	89.4
	Hispanic/Latino(a)	10	5.3	5.3	94.7
	Biracial	5	2.7	2.7	97.3
	Multiracial	2	1.1	1.1	98.4
	Racial Identity Not Listed	3	1.6	1.6	100.0
	Total	188	100.0	100.0	

Age

		Frequency	Percent	Valid Percent	Cumulative Percent
Valid	18	104	55.3	55.3	55.3
	19	50	26.6	26.6	81.9
	20	21	11.2	11.2	93.1
	21	11	5.9	5.9	98.9
	22	1	.5	.5	99.5
	47	1	.5	.5	100.0
	Total	188	100.0	100.0	

MRN

		Frequency	Percent	Valid Percent	Cumulative Percent
Valid	1.27	1	.5	.5	.5
	1.46	1	.5	.5	1.1
	1.54	2	1.1	1.1	2.1
	1.62	1	.5	.5	2.7
	1.65	1	.5	.5	3.2
	1.69	2	1.1	1.1	4.3
	1.73	2	1.1	1.1	5.3
	1.81	1	.5	.5	5.9
	1.88	2	1.1	1.1	6.9
	1.92	2	1.1	1.1	8.0
	1.96	1	.5	.5	8.5
	2.08	2	1.1	1.1	9.6
	2.15	1	.5	.5	10.1
	2.19	3	1.6	1.6	11.7
	2.23	2	1.1	1.1	12.8
	2.27	4	2.1	2.1	14.9
	2.31	3	1.6	1.6	16.5
	2.38	2	1.1	1.1	17.6
	2.42	3	1.6	1.6	19.1
	2.46	3	1.6	1.6	20.7
	2.50	1	.5	.5	21.3
	2.54	5	2.7	2.7	23.9
	2.58	3	1.6	1.6	25.5
	2.62	5	2.7	2.7	28.2

MRN

	Frequency	Percent	Valid Percent	Cumulative Percent
2.65	1	.5	.5	28.7
2.69	3	1.6	1.6	30.3
2.73	5	2.7	2.7	33.0
2.77	2	1.1	1.1	34.0
2.85	2	1.1	1.1	35.1
2.88	3	1.6	1.6	36.7
2.92	3	1.6	1.6	38.3
2.96	5	2.7	2.7	41.0
3.00	1	.5	.5	41.5
3.04	2	1.1	1.1	42.6
3.08	2	1.1	1.1	43.6
3.12	2	1.1	1.1	44.7
3.15	5	2.7	2.7	47.3
3.19	4	2.1	2.1	49.5
3.23	3	1.6	1.6	51.1
3.27	1	.5	.5	51.6
3.31	5	2.7	2.7	54.3
3.35	7	3.7	3.7	58.0
3.38	3	1.6	1.6	59.6
3.42	2	1.1	1.1	60.6
3.46	3	1.6	1.6	62.2
3.50	3	1.6	1.6	63.8
3.54	4	2.1	2.1	66.0
3.58	4	2.1	2.1	68.1
3.62	1	.5	.5	68.6
3.65	3	1.6	1.6	70.2
3.69	3	1.6	1.6	71.8
3.77	2	1.1	1.1	72.9
3.81	2	1.1	1.1	73.9
3.85	3	1.6	1.6	75.5
3.88	1	.5	.5	76.1
3.96	5	2.7	2.7	78.7
4.00	8	4.3	4.3	83.0
4.04	4	2.1	2.1	85.1
4.08	3	1.6	1.6	86.7

MRN

	Frequency	Percent	Valid Percent	Cumulative Percent
4.12	2	1.1	1.1	87.8
4.15	2	1.1	1.1	88.8
4.19	1	.5	.5	89.4
4.23	1	.5	.5	89.9
4.27	1	.5	.5	90.4
4.42	1	.5	.5	91.0
4.46	1	.5	.5	91.5
4.50	1	.5	.5	92.0
4.54	1	.5	.5	92.6
4.58	2	1.1	1.1	93.6
4.62	1	.5	.5	94.1
4.65	2	1.1	1.1	95.2
4.69	1	.5	.5	95.7
4.73	1	.5	.5	96.3
4.85	2	1.1	1.1	97.3
4.96	2	1.1	1.1	98.4
5.04	1	.5	.5	98.9
5.31	1	.5	.5	99.5
5.42	1	.5	.5	100.0
Total	188	100.0	100.0	

Finance_Tot

		Frequency	Percent	Valid Percent	Cumulative Percent
Valid	1.00	8	4.3	4.3	4.3
	1.33	7	3.7	3.7	8.0
	1.67	6	3.2	3.2	11.2
	2.00	11	5.9	5.9	17.0
	2.33	5	2.7	2.7	19.7
	2.67	11	5.9	5.9	25.5
	3.00	17	9.0	9.0	34.6
	3.33	17	9.0	9.0	43.6
	3.67	6	3.2	3.2	46.8
	4.00	28	14.9	14.9	61.7
	4.33	13	6.9	6.9	68.6
	4.67	16	8.5	8.5	77.1
	5.00	10	5.3	5.3	82.4
	5.33	13	6.9	6.9	89.4
	5.67	1	.5	.5	89.9
	6.00	9	4.8	4.8	94.7
	6.33	4	2.1	2.1	96.8
	6.67	2	1.1	1.1	97.9
	7.00	4	2.1	2.1	100.0
	Total	188	100.0	100.0	

Psychology_Tot

		Frequency	Percent	Valid Percent	Cumulative Percent
Valid	1.00	3	1.6	1.6	1.6
	1.33	1	.5	.5	2.1
	1.67	5	2.7	2.7	4.8
	2.00	7	3.7	3.7	8.5
	2.33	6	3.2	3.2	11.7
	2.67	2	1.1	1.1	12.8
	3.00	10	5.3	5.3	18.1
	3.33	8	4.3	4.3	22.3
	3.67	12	6.4	6.4	28.7
	4.00	13	6.9	6.9	35.6
	4.33	11	5.9	5.9	41.5
	4.67	18	9.6	9.6	51.1
	5.00	25	13.3	13.3	64.4
	5.33	19	10.1	10.1	74.5
	5.67	18	9.6	9.6	84.0
	6.00	10	5.3	5.3	89.4
	6.33	6	3.2	3.2	92.6
	6.67	3	1.6	1.6	94.1
	7.00	11	5.9	5.9	100.0
	Total	188	100.0	100.0	

Mandate_Tot

		Frequency	Percent	Valid Percent	Cumulative Percent
Valid	1.00	12	6.4	6.4	6.4
	1.17	4	2.1	2.1	8.5

Mandate_Tot

	Frequency	Percent	Valid Percent	Cumulative Percent
1.33	2	1.1	1.1	9.6
1.50	3	1.6	1.6	11.2
1.67	1	.5	.5	11.7
1.83	1	.5	.5	12.2
2.00	1	.5	.5	12.8
2.17	2	1.1	1.1	13.8
2.33	2	1.1	1.1	14.9
2.50	1	.5	.5	15.4
2.67	2	1.1	1.1	16.5
2.83	3	1.6	1.6	18.1
3.33	3	1.6	1.6	19.7
3.50	2	1.1	1.1	20.7
3.67	3	1.6	1.6	22.3
3.83	4	2.1	2.1	24.5
4.00	18	9.6	9.6	34.0
4.17	3	1.6	1.6	35.6
4.33	5	2.7	2.7	38.3
4.50	5	2.7	2.7	41.0
4.67	5	2.7	2.7	43.6
4.83	7	3.7	3.7	47.3
5.00	5	2.7	2.7	50.0
5.17	9	4.8	4.8	54.8
5.33	8	4.3	4.3	59.0
5.50	6	3.2	3.2	62.2
5.67	12	6.4	6.4	68.6
5.83	6	3.2	3.2	71.8
6.00	10	5.3	5.3	77.1
6.17	10	5.3	5.3	82.4
6.33	4	2.1	2.1	84.6
6.50	6	3.2	3.2	87.8
6.67	2	1.1	1.1	88.8
6.83	7	3.7	3.7	92.6
7.00	14	7.4	7.4	100.0
Total	188	100.0	100.0	

Conspiracy_Tot

		Frequency	Percent	Valid Percent	Cumulative Percent
Valid	1.00	40	21.3	21.3	21.3
	1.11	26	13.8	13.8	35.1
	1.22	13	6.9	6.9	42.0
	1.33	15	8.0	8.0	50.0
	1.44	20	10.6	10.6	60.6
	1.56	14	7.4	7.4	68.1
	1.67	9	4.8	4.8	72.9
	1.78	9	4.8	4.8	77.7
	1.89	11	5.9	5.9	83.5
	2.00	7	3.7	3.7	87.2
	2.11	1	.5	.5	87.8
	2.22	2	1.1	1.1	88.8
	2.33	4	2.1	2.1	91.0
	2.56	4	2.1	2.1	93.1
	2.89	2	1.1	1.1	94.1
	3.00	4	2.1	2.1	96.3
	3.11	2	1.1	1.1	97.3
	3.33	1	.5	.5	97.9
	3.44	1	.5	.5	98.4
	3.56	1	.5	.5	98.9
	3.78	1	.5	.5	99.5
	5.00	1	.5	.5	100.0
	Total	188	100.0	100.0	

National_Tot

		Frequency	Percent	Valid Percent	Cumulative Percent
Valid	1.00	8	4.3	4.3	4.3
	2.00	3	1.6	1.6	5.9
	2.50	3	1.6	1.6	7.4
	3.00	5	2.7	2.7	10.1
	3.50	6	3.2	3.2	13.3
	4.00	9	4.8	4.8	18.1
	4.50	11	5.9	5.9	23.9
	5.00	15	8.0	8.0	31.9
	5.50	32	17.0	17.0	48.9
	6.00	24	12.8	12.8	61.7
	6.50	40	21.3	21.3	83.0
	7.00	32	17.0	17.0	100.0
	Total	188	100.0	100.0	

```
 **Contrast Codes and Mean Centering**

IF (Gender=1) GenderCC=1.
IF (Gender=-1) GenderCC=-1.

IF (Race=1) RaceCC=1.
IF (Race>=2) RaceCC=-1.

COMPUTE Party0=PParty-2.76.
COMPUTE Ideology0=PIdeology-3.55.
COMPUTE SES0= SES - 3.35.
COMPUTE MRN0=MRN-3.22.
COMPUTE National0=National_Tot-5.44.

**Interactions**

COMPUTE MRN0xRace=MRN0 * RaceCC.
COMPUTE MRN0xSES0=MRN0 * SES0.
COMPUTE MRN0xGender=MRN0 * GenderCC.
COMPUTE MRN0xParty0=MRN0 * Party0.
COMPUTE MRN0xIdeology0=MRN0*Ideology0.
COMPUTE MRN0xNational0=MRN0*National0.

**Regression Analyses
```

```
**Including PParty

REGRESSION
  /MISSING LISTWISE
  /STATISTICS COEFF OUTS R ANOVA CHANGE ZPP
  /CRITERIA=PIN(.05) POUT(.10)
  /NOORIGIN
  /DEPENDENT Concern_Tot
  /METHOD=ENTER Party0
  /METHOD=ENTER GenderCC RaceCC SES0
  /METHOD=ENTER MRN0
  /METHOD=ENTER MRN0xRace MRN0xSES0 MRN0xGender MRN0xParty0.
```

Regression

Notes

Output Created		15-DEC-2021 13:09:40
Comments		
Input	Data	C:\Users\njs5478\Dropbox\HM and COVID\0. Revise and Resubmit\2. R and R Data\Study 2a\Study2a_Data.sav
	Active Dataset	DataSet1
	Filter	<none>
	Weight	<none>
	Split File	<none>
	N of Rows in Working Data File	188
Missing Value Handling	Definition of Missing	User-defined missing values are treated as missing.
	Cases Used	Statistics are based on cases with no missing values for any variable used.

Notes

Syntax		REGRESSION<br>/MISSING LISTWISE<br>/STATISTICS COEFF OUTS R ANOVA CHANGE ZPP<br>/CRITERIA=PIN(.05) POUT(.10)<br>/NOORIGIN<br>/DEPENDENT Concern_Tot<br>/METHOD=ENTER Party0<br>/METHOD=ENTER GenderCC RaceCC SES0<br>/METHOD=ENTER MRN0<br>/METHOD=ENTER MRN0xRace MRN0xSES0 MRN0xGender MRN0xParty0.
Resources	Processor Time	00:00:00.05
	Elapsed Time	00:00:00.02
	Memory Required	33072 bytes
	Additional Memory Required for Residual Plots	0 bytes

Variables Entered/Removed[a]

Model	Variables Entered	Variables Removed	Method
1	Party0[b]	.	Enter
2	GenderCC, SES0, RaceCC[b]	.	Enter
3	MRN0[b]	.	Enter
4	MRN0xGender, MRN0xSES0, MRN0xParty0, MRN0xRace[b]	.	Enter

a. Dependent Variable: Concern_Tot

b. All requested variables entered.

Model Summary

Model	R	R Square	Adjusted R Square	Std. Error of the Estimate	Change Statistics				
					R Square Change	F Change	df1	df2	Sig. F Change
1	.493[a]	.243	.239	1.18922	.243	59.660	1	186	.000
2	.577[b]	.333	.318	1.12526	.090	8.248	3	183	.000
3	.588[c]	.346	.328	1.11727	.013	3.627	1	182	.058
4	.600[d]	.360	.328	1.11770	.014	.965	4	178	.428

a. Predictors: (Constant), Party0

b. Predictors: (Constant), Party0, GenderCC, SES0, RaceCC

c. Predictors: (Constant), Party0, GenderCC, SES0, RaceCC, MRN0

d. Predictors: (Constant), Party0, GenderCC, SES0, RaceCC, MRN0, MRN0xGender, MRN0xSES0, MRN0xParty0, MRN0xRace

ANOVA[a]

Model		Sum of Squares	df	Mean Square	F	Sig.
1	Regression	84.373	1	84.373	59.660	.000[b]
	Residual	263.048	186	1.414		
	Total	347.421	187			
2	Regression	115.704	4	28.926	22.845	.000[c]
	Residual	231.717	183	1.266		
	Total	347.421	187			
3	Regression	120.232	5	24.046	19.263	.000[d]
	Residual	227.190	182	1.248		
	Total	347.421	187			
4	Regression	125.054	9	13.895	11.123	.000[e]
	Residual	222.368	178	1.249		
	Total	347.421	187			

a. Dependent Variable: Concern_Tot

b. Predictors: (Constant), Party0

c. Predictors: (Constant), Party0, GenderCC, SES0, RaceCC

d. Predictors: (Constant), Party0, GenderCC, SES0, RaceCC, MRN0

e. Predictors: (Constant), Party0, GenderCC, SES0, RaceCC, MRN0, MRN0xGender, MRN0xSES0, MRN0xParty0, MRN0xRace

Coefficients[a]

Model		Unstandardized Coefficients B	Unstandardized Coefficients Std. Error	Standardized Coefficients Beta	t	Sig.
1	(Constant)	3.629	.087		41.840	.000
	Party0	-.551	.071	-.493	-7.724	.000
2	(Constant)	3.704	.091		40.891	.000
	Party0	-.498	.072	-.446	-6.954	.000
	GenderCC	-.401	.083	-.295	-4.818	.000
	RaceCC	-.185	.095	-.124	-1.949	.053
	SES0	-2.312E-5	.115	.000	.000	1.000
3	(Constant)	3.701	.090		41.149	.000
	Party0	-.444	.077	-.398	-5.804	.000
	GenderCC	-.316	.094	-.233	-3.364	.001
	RaceCC	-.180	.094	-.121	-1.916	.057
	SES0	.024	.114	.013	.206	.837
	MRN0	-.226	.119	-.142	-1.904	.058
4	(Constant)	3.694	.103		35.708	.000
	Party0	-.420	.079	-.376	-5.332	.000
	GenderCC	-.314	.094	-.231	-3.335	.001
	RaceCC	-.207	.096	-.139	-2.164	.032
	SES0	.031	.117	.017	.263	.793
	MRN0	-.166	.123	-.104	-1.351	.179
	MRN0xRace	-.215	.110	-.134	-1.945	.053
	MRN0xSES0	.033	.158	.013	.210	.834
	MRN0xGender	.010	.110	.005	.087	.931
	MRN0xParty0	.052	.092	.038	.565	.573

Coefficients[a]

Model		Correlations Zero-order	Partial	Part
1	(Constant)			
	Party0	-.493	-.493	-.493
2	(Constant)			
	Party0	-.493	-.457	-.420
	GenderCC	-.295	-.335	-.291
	RaceCC	-.209	-.143	-.118
	SES0	-.116	.000	.000
3	(Constant)			
	Party0	-.493	-.395	-.348
	GenderCC	-.295	-.242	-.202
	RaceCC	-.209	-.141	-.115
	SES0	-.116	.015	.012
	MRN0	-.405	-.140	-.114
4	(Constant)			
	Party0	-.493	-.371	-.320
	GenderCC	-.295	-.243	-.200
	RaceCC	-.209	-.160	-.130
	SES0	-.116	.020	.016
	MRN0	-.405	-.101	-.081
	MRN0xRace	-.288	-.144	-.117
	MRN0xSES0	-.016	.016	.013
	MRN0xGender	.063	.007	.005
	MRN0xParty0	-.054	.042	.034

a. Dependent Variable: Concern_Tot

Excluded Variables[a]

Model		Beta In	t	Sig.	Partial Correlation	Collinearity Statistics Tolerance
1	GenderCC	-.276[b]	-4.554	.000	-.317	.998
	RaceCC	-.076[b]	-1.143	.254	-.084	.920
	SES0	-.020[b]	-.306	.760	-.022	.962
	MRN0	-.254[b]	-3.814	.000	-.270	.854
	MRN0xRace	-.163[b]	-2.491	.014	-.180	.922
	MRN0xSES0	.004[b]	.067	.947	.005	.998
	MRN0xGender	.016[b]	.242	.809	.018	.991
	MRN0xParty0	.024[b]	.368	.713	.027	.975
2	MRN0	-.142[c]	-1.904	.058	-.140	.650
	MRN0xRace	-.141[c]	-2.226	.027	-.163	.895
	MRN0xSES0	.008[c]	.132	.895	.010	.956
	MRN0xGender	.012[c]	.194	.847	.014	.989
	MRN0xParty0	.007[c]	.117	.907	.009	.958
3	MRN0xRace	-.120[d]	-1.862	.064	-.137	.853
	MRN0xSES0	.006[d]	.092	.927	.007	.955
	MRN0xGender	.016[d]	.257	.797	.019	.988
	MRN0xParty0	-.001[d]	-.009	.993	-.001	.953

a. Dependent Variable: Concern_Tot

b. Predictors in the Model: (Constant), Party0

c. Predictors in the Model: (Constant), Party0, GenderCC, SES0, RaceCC

d. Predictors in the Model: (Constant), Party0, GenderCC, SES0, RaceCC, MRN0

```
REGRESSION
 /MISSING LISTWISE
 /STATISTICS COEFF OUTS R ANOVA CHANGE ZPP
 /CRITERIA=PIN(.05) POUT(.10)
 /NOORIGIN
 /DEPENDENT Finance_Tot
 /METHOD=ENTER Party0
 /METHOD=ENTER GenderCC RaceCC SES0
 /METHOD=ENTER MRN0
 /METHOD=ENTER MRN0xRace MRN0xSES0 MRN0xGender MRN0xParty0.
```

Regression

Notes

Output Created		15-DEC-2021 13:09:40
Comments		
Input	Data	C:\Users\njs5478\Dropbox\HM and COVID\0. Revise and Resubmit\2. R and R Data\Study 2a\Study2a_Data.sav
	Active Dataset	DataSet1
	Filter	<none>
	Weight	<none>
	Split File	<none>
	N of Rows in Working Data File	188
Missing Value Handling	Definition of Missing	User-defined missing values are treated as missing.
	Cases Used	Statistics are based on cases with no missing values for any variable used.
Syntax		REGRESSION /MISSING LISTWISE /STATISTICS COEFF OUTS R ANOVA CHANGE ZPP /CRITERIA=PIN(.05) POUT(.10) /NOORIGIN /DEPENDENT Finance_Tot /METHOD=ENTER Party0 /METHOD=ENTER GenderCC RaceCC SES0 /METHOD=ENTER MRN0 /METHOD=ENTER MRN0xRace MRN0xSES0 MRN0xGender MRN0xParty0.
Resources	Processor Time	00:00:00.02
	Elapsed Time	00:00:00.02

Notes

	Memory Required	33072 bytes
	Additional Memory Required for Residual Plots	0 bytes

Variables Entered/Removed[a]

Model	Variables Entered	Variables Removed	Method
1	Party0[b]	.	Enter
2	GenderCC, SES0, RaceCC[b]	.	Enter
3	MRN0[b]	.	Enter
4	MRN0xGender, MRN0xSES0, MRN0xParty0, MRN0xRace[b]	.	Enter

a. Dependent Variable: Finance_Tot

b. All requested variables entered.

Model Summary

Model	R	R Square	Adjusted R Square	Std. Error of the Estimate	Change Statistics		
					R Square Change	F Change	df1
1	.070[a]	.005	.000	1.46207	.005	.918	1
2	.357[b]	.127	.108	1.38029	.123	8.564	3
3	.365[c]	.133	.109	1.37961	.006	1.181	1
4	.372[d]	.138	.095	1.39064	.005	.281	4

Model Summary

Model	Change Statistics	
	df2	Sig. F Change
1	186	.339
2	183	.000
3	182	.279
4	178	.890

a. Predictors: (Constant), Party0

b. Predictors: (Constant), Party0, GenderCC, SES0, RaceCC

c. Predictors: (Constant), Party0, GenderCC, SES0, RaceCC, MRN0

d. Predictors: (Constant), Party0, GenderCC, SES0, RaceCC, MRN0, MRN0xGender, MRN0xSES0, MRN0xParty0, MRN0xRace

ANOVA[a]

Model		Sum of Squares	df	Mean Square	F	Sig.
1	Regression	1.963	1	1.963	.918	.339[b]
	Residual	397.604	186	2.138		
	Total	399.567	187			
2	Regression	50.914	4	12.728	6.681	.000[c]
	Residual	348.654	183	1.905		
	Total	399.567	187			
3	Regression	53.160	5	10.632	5.586	.000[d]
	Residual	346.407	182	1.903		
	Total	399.567	187			
4	Regression	55.334	9	6.148	3.179	.001[e]
	Residual	344.233	178	1.934		
	Total	399.567	187			

a. Dependent Variable: Finance_Tot

b. Predictors: (Constant), Party0

c. Predictors: (Constant), Party0, GenderCC, SES0, RaceCC

d. Predictors: (Constant), Party0, GenderCC, SES0, RaceCC, MRN0

e. Predictors: (Constant), Party0, GenderCC, SES0, RaceCC, MRN0, MRN0xGender, MRN0xSES0, MRN0xParty0, MRN0xRace

Coefficients[a]

Model		Unstandardized Coefficients B	Std. Error	Standardized Coefficients Beta	t	Sig.
1	(Constant)	3.770	.107		35.351	.000
	Party0	-.084	.088	-.070	-.958	.339
2	(Constant)	3.809	.111		34.285	.000
	Party0	.019	.088	.016	.217	.829
	GenderCC	-.152	.102	-.104	-1.486	.139
	RaceCC	-.096	.116	-.060	-.827	.409
	SES0	-.659	.141	-.331	-4.690	.000
3	(Constant)	3.807	.111		34.281	.000
	Party0	.057	.095	.048	.603	.547
	GenderCC	-.092	.116	-.063	-.791	.430
	RaceCC	-.093	.116	-.058	-.801	.424
	SES0	-.642	.141	-.323	-4.547	.000
	MRN0	-.159	.146	-.093	-1.087	.279
4	(Constant)	3.805	.129		29.559	.000
	Party0	.041	.098	.034	.414	.680
	GenderCC	-.090	.117	-.062	-.768	.444
	RaceCC	-.109	.119	-.068	-.911	.364
	SES0	-.633	.146	-.318	-4.347	.000
	MRN0	-.140	.153	-.082	-.914	.362
	MRN0xRace	-.025	.137	-.014	-.179	.858
	MRN0xSES0	.053	.197	.020	.269	.788
	MRN0xGender	-.087	.137	-.045	-.637	.525
	MRN0xParty0	.096	.115	.064	.833	.406

Coefficients[a]

Model		Correlations Zero-order	Partial	Part
1	(Constant)			
	Party0	-.070	-.070	-.070
2	(Constant)			
	Party0	-.070	.016	.015
	GenderCC	-.114	-.109	-.103
	RaceCC	-.071	-.061	-.057
	SES0	-.339	-.328	-.324
3	(Constant)			
	Party0	-.070	.045	.042
	GenderCC	-.114	-.059	-.055
	RaceCC	-.071	-.059	-.055
	SES0	-.339	-.319	-.314
	MRN0	-.167	-.080	-.075
4	(Constant)			
	Party0	-.070	.031	.029
	GenderCC	-.114	-.057	-.053
	RaceCC	-.071	-.068	-.063
	SES0	-.339	-.310	-.302
	MRN0	-.167	-.068	-.064
	MRN0xRace	-.044	-.013	-.012
	MRN0xSES0	.090	.020	.019
	MRN0xGender	-.038	-.048	-.044
	MRN0xParty0	.050	.062	.058

a. Dependent Variable: Finance_Tot

Excluded Variables[a]

Model		Beta In	t	Sig.	Partial Correlation	Collinearity Statistics Tolerance
1	GenderCC	-.112[b]	-1.532	.127	-.112	.998
	RaceCC	-.056[b]	-.729	.467	-.054	.920
	SES0	-.339[b]	-4.802	.000	-.333	.962
	MRN0	-.164[b]	-2.091	.038	-.152	.854
	MRN0xRace	-.027[b]	-.350	.727	-.026	.922
	MRN0xSES0	.093[b]	1.273	.205	.093	.998
	MRN0xGender	-.045[b]	-.609	.544	-.045	.991
	MRN0xParty0	.063[b]	.847	.398	.062	.975
2	MRN0	-.093[c]	-1.087	.279	-.080	.650
	MRN0xRace	-.006[c]	-.080	.936	-.006	.895
	MRN0xSES0	.029[c]	.414	.680	.031	.956
	MRN0xGender	-.038[c]	-.541	.589	-.040	.989
	MRN0xParty0	.062[c]	.877	.382	.065	.958
3	MRN0xRace	.012[d]	.159	.874	.012	.853
	MRN0xSES0	.028[d]	.390	.697	.029	.955
	MRN0xGender	-.035[d]	-.506	.614	-.038	.988
	MRN0xParty0	.057[d]	.807	.421	.060	.953

a. Dependent Variable: Finance_Tot

b. Predictors in the Model: (Constant), Party0

c. Predictors in the Model: (Constant), Party0, GenderCC, SES0, RaceCC

d. Predictors in the Model: (Constant), Party0, GenderCC, SES0, RaceCC, MRN0

```
REGRESSION
 /MISSING LISTWISE
 /STATISTICS COEFF OUTS R ANOVA CHANGE ZPP
 /CRITERIA=PIN(.05) POUT(.10)
 /NOORIGIN
 /DEPENDENT Psychology_Tot
 /METHOD=ENTER Party0
 /METHOD=ENTER GenderCC RaceCC SES0
 /METHOD=ENTER MRN0
 /METHOD=ENTER MRN0xRace MRN0xSES0 MRN0xGender MRN0xParty0.
```

Regression

Notes

Output Created		15-DEC-2021 13:09:40
Comments		
Input	Data	C: \Users\njs5478\Dropbox\HM and COVID\0. Revise and Resubmit\2. R and R Data\Study 2a\Study2a_Data.sav
	Active Dataset	DataSet1
	Filter	<none>
	Weight	<none>
	Split File	<none>
	N of Rows in Working Data File	188
Missing Value Handling	Definition of Missing	User-defined missing values are treated as missing.
	Cases Used	Statistics are based on cases with no missing values for any variable used.
Syntax		REGRESSION<br>/MISSING LISTWISE<br>/STATISTICS COEFF OUTS R ANOVA CHANGE ZPP<br>/CRITERIA=PIN(.05) POUT(.10)<br>/NOORIGIN<br>/DEPENDENT Psychology_Tot<br>/METHOD=ENTER Party0<br>/METHOD=ENTER GenderCC RaceCC SES0<br>/METHOD=ENTER MRN0<br>/METHOD=ENTER MRN0xRace MRN0xSES0 MRN0xGender MRN0xParty0.
Resources	Processor Time	00:00:00.03
	Elapsed Time	00:00:00.02

Notes

	Memory Required	33072 bytes
	Additional Memory Required for Residual Plots	0 bytes

Variables Entered/Removed[a]

Model	Variables Entered	Variables Removed	Method
1	Party0[b]	.	Enter
2	GenderCC, SES0, RaceCC[b]	.	Enter
3	MRN0[b]	.	Enter
4	MRN0xGender, MRN0xSES0, MRN0xParty0, MRN0xRace[b]	.	Enter

a. Dependent Variable: Psychology_Tot

b. All requested variables entered.

Model Summary

Model	R	R Square	Adjusted R Square	Std. Error of the Estimate	Change Statistics		
					R Square Change	F Change	df1
1	.180[a]	.033	.027	1.40677	.033	6.250	1
2	.227[b]	.052	.031	1.40411	.019	1.235	3
3	.253[c]	.064	.039	1.39863	.013	2.438	1
4	.342[d]	.117	.072	1.37407	.052	2.641	4

Model Summary

Model	Change Statistics	
	df2	Sig. F Change
1	186	.013
2	183	.298
3	182	.120
4	178	.035

a. Predictors: (Constant), Party0

b. Predictors: (Constant), Party0, GenderCC, SES0, RaceCC

c. Predictors: (Constant), Party0, GenderCC, SES0, RaceCC, MRN0

d. Predictors: (Constant), Party0, GenderCC, SES0, RaceCC, MRN0, MRN0xGender, MRN0xSES0, MRN0xParty0, MRN0xRace

ANOVA[a]

Model		Sum of Squares	df	Mean Square	F	Sig.
1	Regression	12.370	1	12.370	6.250	.013[b]
	Residual	368.096	186	1.979		
	Total	380.465	187			
2	Regression	19.674	4	4.918	2.495	.045[c]
	Residual	360.791	183	1.972		
	Total	380.465	187			
3	Regression	24.442	5	4.888	2.499	.032[d]
	Residual	356.023	182	1.956		
	Total	380.465	187			
4	Regression	44.390	9	4.932	2.612	.007[e]
	Residual	336.075	178	1.888		
	Total	380.465	187			

a. Dependent Variable: Psychology_Tot

b. Predictors: (Constant), Party0

c. Predictors: (Constant), Party0, GenderCC, SES0, RaceCC

d. Predictors: (Constant), Party0, GenderCC, SES0, RaceCC, MRN0

e. Predictors: (Constant), Party0, GenderCC, SES0, RaceCC, MRN0, MRN0xGender, MRN0xSES0, MRN0xParty0, MRN0xRace

Coefficients[a]

Model		Unstandardized Coefficients B	Std. Error	Standardized Coefficients Beta	t	Sig.
1	(Constant)	4.541	.103		44.259	.000
	Party0	-.211	.084	-.180	-2.500	.013
2	(Constant)	4.507	.113		39.876	.000
	Party0	-.217	.089	-.186	-2.432	.016
	GenderCC	-.166	.104	-.117	-1.599	.111
	RaceCC	.085	.118	.055	.718	.474
	SES0	-.051	.143	-.026	-.360	.719
3	(Constant)	4.504	.113		40.003	.000
	Party0	-.162	.096	-.139	-1.691	.092
	GenderCC	-.079	.118	-.055	-.671	.503
	RaceCC	.089	.118	.058	.759	.449
	SES0	-.027	.143	-.014	-.190	.849
	MRN0	-.232	.148	-.139	-1.561	.120
4	(Constant)	4.376	.127		34.406	.000
	Party0	-.160	.097	-.137	-1.656	.099
	GenderCC	-.064	.116	-.045	-.553	.581
	RaceCC	.032	.118	.021	.273	.785
	SES0	-.020	.144	-.010	-.140	.889
	MRN0	-.137	.151	-.082	-.906	.366
	MRN0xRace	-.265	.136	-.159	-1.956	.052
	MRN0xSES0	.074	.194	.028	.381	.704
	MRN0xGender	.083	.135	.044	.614	.540
	MRN0xParty0	.312	.113	.215	2.750	.007

Coefficients[a]

Model		Correlations		
		Zero-order	Partial	Part
1	(Constant)			
	Party0	-.180	-.180	-.180
2	(Constant)			
	Party0	-.180	-.177	-.175
	GenderCC	-.133	-.117	-.115
	RaceCC	.016	.053	.052
	SES0	-.065	-.027	-.026
3	(Constant)			
	Party0	-.180	-.124	-.121
	GenderCC	-.133	-.050	-.048
	RaceCC	.016	.056	.054
	SES0	-.065	-.014	-.014
	MRN0	-.216	-.115	-.112
4	(Constant)			
	Party0	-.180	-.123	-.117
	GenderCC	-.133	-.041	-.039
	RaceCC	.016	.020	.019
	SES0	-.065	-.011	-.010
	MRN0	-.216	-.068	-.064
	MRN0xRace	-.167	-.145	-.138
	MRN0xSES0	.049	.029	.027
	MRN0xGender	.091	.046	.043
	MRN0xParty0	.165	.202	.194

a. Dependent Variable: Psychology_Tot

Excluded Variables[a]

Model		Beta In	t	Sig.	Partial Correlation	Collinearity Statistics Tolerance
1	GenderCC	-.126[b]	-1.762	.080	-.128	.998
	RaceCC	.073[b]	.968	.334	.071	.920
	SES0	-.031[b]	-.416	.678	-.031	.962
	MRN0	-.172[b]	-2.233	.027	-.162	.854
	MRN0xRace	-.126[b]	-1.685	.094	-.123	.922
	MRN0xSES0	.057[b]	.786	.433	.058	.998
	MRN0xGender	.074[b]	1.018	.310	.075	.991
	MRN0xParty0	.198[b]	2.757	.006	.199	.975
2	MRN0	-.139[c]	-1.561	.120	-.115	.650
	MRN0xRace	-.105[c]	-1.385	.168	-.102	.895
	MRN0xSES0	.054[c]	.733	.464	.054	.956
	MRN0xGender	.076[c]	1.050	.295	.078	.989
	MRN0xParty0	.184[c]	2.540	.012	.185	.958
3	MRN0xRace	-.083[d]	-1.074	.284	-.080	.853
	MRN0xSES0	.052[d]	.702	.483	.052	.955
	MRN0xGender	.080[d]	1.106	.270	.082	.988
	MRN0xParty0	.177[d]	2.447	.015	.179	.953

a. Dependent Variable: Psychology_Tot

b. Predictors in the Model: (Constant), Party0

c. Predictors in the Model: (Constant), Party0, GenderCC, SES0, RaceCC

d. Predictors in the Model: (Constant), Party0, GenderCC, SES0, RaceCC, MRN0

```
REGRESSION
 /MISSING LISTWISE
 /STATISTICS COEFF OUTS R ANOVA CHANGE ZPP
 /CRITERIA=PIN(.05) POUT(.10)
 /NOORIGIN
 /DEPENDENT Risk_Rules
 /METHOD=ENTER Party0
 /METHOD=ENTER GenderCC RaceCC SES0
 /METHOD=ENTER MRN0
 /METHOD=ENTER MRN0xRace MRN0xSES0 MRN0xGender MRN0xParty0.
```

Regression

Notes

Output Created		15-DEC-2021 13:09:40
Comments		
Input	Data	C: \Users\njs5478\Dropbox\HM and COVID\0. Revise and Resubmit\2. R and R Data\Study 2a\Study2a_Data.sav
	Active Dataset	DataSet1
	Filter	<none>
	Weight	<none>
	Split File	<none>
	N of Rows in Working Data File	188
Missing Value Handling	Definition of Missing	User-defined missing values are treated as missing.
	Cases Used	Statistics are based on cases with no missing values for any variable used.
Syntax		REGRESSION<br>/MISSING LISTWISE<br>/STATISTICS COEFF OUTS R ANOVA CHANGE ZPP<br>/CRITERIA=PIN(.05) POUT(.10)<br>/NOORIGIN<br>/DEPENDENT Risk_Rules<br>/METHOD=ENTER Party0<br>/METHOD=ENTER GenderCC RaceCC SES0<br>/METHOD=ENTER MRN0<br>/METHOD=ENTER MRN0xRace MRN0xSES0 MRN0xGender MRN0xParty0.
Resources	Processor Time	00:00:00.00
	Elapsed Time	00:00:00.02

Notes

	Memory Required	33072 bytes
	Additional Memory Required for Residual Plots	0 bytes

Variables Entered/Removed[a]

Model	Variables Entered	Variables Removed	Method
1	Party0[b]	.	Enter
2	GenderCC, SES0, RaceCC[b]	.	Enter
3	MRN0[b]	.	Enter
4	MRN0xGender, MRN0xSES0, MRN0xParty0, MRN0xRace[b]	.	Enter

a. Dependent Variable: Risk_Rules

b. All requested variables entered.

Model Summary

Model	R	R Square	Adjusted R Square	Std. Error of the Estimate	Change Statistics		
					R Square Change	F Change	df1
1	.515[a]	.265	.261	.92388	.265	66.974	1
2	.580[b]	.336	.322	.88488	.072	6.585	3
3	.636[c]	.404	.388	.84067	.068	20.753	1
4	.642[d]	.413	.383	.84407	.008	.634	4

Model Summary

Model	Change Statistics	
	df2	Sig. F Change
1	186	.000
2	183	.000
3	182	.000
4	178	.639

a. Predictors: (Constant), Party0

b. Predictors: (Constant), Party0, GenderCC, SES0, RaceCC

c. Predictors: (Constant), Party0, GenderCC, SES0, RaceCC, MRN0

d. Predictors: (Constant), Party0, GenderCC, SES0, RaceCC, MRN0, MRN0xGender, MRN0xSES0, MRN0xParty0, MRN0xRace

ANOVA[a]

Model		Sum of Squares	df	Mean Square	F	Sig.
1	Regression	57.166	1	57.166	66.974	.000[b]
	Residual	158.761	186	.854		
	Total	215.926	187			
2	Regression	72.635	4	18.159	23.191	.000[c]
	Residual	143.292	183	.783		
	Total	215.926	187			
3	Regression	87.302	5	17.460	24.706	.000[d]
	Residual	128.625	182	.707		
	Total	215.926	187			
4	Regression	89.110	9	9.901	13.897	.000[e]
	Residual	126.816	178	.712		
	Total	215.926	187			

a. Dependent Variable: Risk_Rules

b. Predictors: (Constant), Party0

c. Predictors: (Constant), Party0, GenderCC, SES0, RaceCC

d. Predictors: (Constant), Party0, GenderCC, SES0, RaceCC, MRN0

e. Predictors: (Constant), Party0, GenderCC, SES0, RaceCC, MRN0, MRN0xGender, MRN0xSES0, MRN0xParty0, MRN0xRace

Coefficients[a]

Model		Unstandardized Coefficients B	Unstandardized Coefficients Std. Error	Standardized Coefficients Beta	t	Sig.
1	(Constant)	3.925	.067		58.255	.000
	Party0	.453	.055	.515	8.184	.000
2	(Constant)	3.844	.071		53.976	.000
	Party0	.389	.056	.441	6.894	.000
	GenderCC	.230	.066	.215	3.510	.001
	RaceCC	.200	.075	.171	2.679	.008
	SES0	.126	.090	.086	1.395	.165
3	(Constant)	3.849	.068		56.878	.000
	Party0	.291	.058	.331	5.058	.000
	GenderCC	.077	.071	.072	1.086	.279
	RaceCC	.192	.071	.164	2.709	.007
	SES0	.083	.086	.057	.966	.335
	MRN0	.407	.089	.323	4.556	.000
4	(Constant)	3.837	.078		49.116	.000
	Party0	.271	.060	.308	4.557	.000
	GenderCC	.080	.071	.075	1.123	.263
	RaceCC	.189	.072	.161	2.612	.010
	SES0	.079	.088	.054	.889	.375
	MRN0	.400	.093	.318	4.306	.000
	MRN0xRace	.057	.083	.045	.687	.493
	MRN0xSES0	-.021	.119	-.011	-.177	.860
	MRN0xGender	-.043	.083	-.031	-.525	.600
	MRN0xParty0	.075	.070	.069	1.082	.281

Coefficients[a]

Model		Correlations Zero-order	Partial	Part
1	(Constant)			
	Party0	.515	.515	.515
2	(Constant)			
	Party0	.515	.454	.415
	GenderCC	.213	.251	.211
	RaceCC	.273	.194	.161
	SES0	.200	.103	.084
3	(Constant)			
	Party0	.515	.351	.289
	GenderCC	.213	.080	.062
	RaceCC	.273	.197	.155
	SES0	.200	.071	.055
	MRN0	.503	.320	.261
4	(Constant)			
	Party0	.515	.323	.262
	GenderCC	.213	.084	.064
	RaceCC	.273	.192	.150
	SES0	.200	.066	.051
	MRN0	.503	.307	.247
	MRN0xRace	.269	.051	.039
	MRN0xSES0	.007	-.013	-.010
	MRN0xGender	-.067	-.039	-.030
	MRN0xParty0	.134	.081	.062

a. Dependent Variable: Risk_Rules

Excluded Variables[a]

Model		Beta In	t	Sig.	Partial Correlation	Collinearity Statistics Tolerance
1	GenderCC	.193[b]	3.142	.002	.225	.998
	RaceCC	.139[b]	2.134	.034	.155	.920
	SES0	.103[b]	1.617	.108	.118	.962
	MRN0	.359[b]	5.712	.000	.387	.854
	MRN0xRace	.135[b]	2.087	.038	.152	.922
	MRN0xSES0	-.014[b]	-.225	.822	-.017	.998
	MRN0xGender	-.017[b]	-.276	.783	-.020	.991
	MRN0xParty0	.054[b]	.851	.396	.062	.975
2	MRN0	.323[c]	4.556	.000	.320	.650
	MRN0xRace	.126[c]	1.989	.048	.146	.895
	MRN0xSES0	.000[c]	-.007	.994	-.001	.956
	MRN0xGender	-.015[c]	-.245	.807	-.018	.989
	MRN0xParty0	.059[c]	.952	.342	.070	.958
3	MRN0xRace	.069[d]	1.116	.266	.083	.853
	MRN0xSES0	.005[d]	.092	.927	.007	.955
	MRN0xGender	-.023[d]	-.406	.685	-.030	.988
	MRN0xParty0	.077[d]	1.311	.191	.097	.953

a. Dependent Variable: Risk_Rules

b. Predictors in the Model: (Constant), Party0

c. Predictors in the Model: (Constant), Party0, GenderCC, SES0, RaceCC

d. Predictors in the Model: (Constant), Party0, GenderCC, SES0, RaceCC, MRN0

```
REGRESSION
 /MISSING LISTWISE
 /STATISTICS COEFF OUTS R ANOVA CHANGE ZPP
 /CRITERIA=PIN(.05) POUT(.10)
 /NOORIGIN
 /DEPENDENT Mandate_Tot
 /METHOD=ENTER Party0
 /METHOD=ENTER GenderCC RaceCC SES0
 /METHOD=ENTER MRN0
 /METHOD=ENTER MRN0xRace MRN0xSES0 MRN0xGender MRN0xParty0.
```

Regression

Notes

Output Created		15-DEC-2021 13:09:40
Comments		
Input	Data	C:\Users\njs5478\Dropbox\HM and COVID\0. Revise and Resubmit\2. R and R Data\Study 2a\Study2a_Data.sav
	Active Dataset	DataSet1
	Filter	<none>
	Weight	<none>
	Split File	<none>
	N of Rows in Working Data File	188
Missing Value Handling	Definition of Missing	User-defined missing values are treated as missing.
	Cases Used	Statistics are based on cases with no missing values for any variable used.
Syntax		REGRESSION /MISSING LISTWISE /STATISTICS COEFF OUTS R ANOVA CHANGE ZPP /CRITERIA=PIN(.05) POUT(.10) /NOORIGIN /DEPENDENT Mandate_Tot /METHOD=ENTER Party0 /METHOD=ENTER GenderCC RaceCC SES0 /METHOD=ENTER MRN0 /METHOD=ENTER MRN0xRace MRN0xSES0 MRN0xGender MRN0xParty0.
Resources	Processor Time	00:00:00.02
	Elapsed Time	00:00:00.03

Notes

	Memory Required	33072 bytes
	Additional Memory Required for Residual Plots	0 bytes

Variables Entered/Removed[a]

Model	Variables Entered	Variables Removed	Method
1	Party0[b]	.	Enter
2	GenderCC, SES0, RaceCC[b]	.	Enter
3	MRN0[b]	.	Enter
4	MRN0xGender, MRN0xSES0, MRN0xParty0, MRN0xRace[b]	.	Enter

a. Dependent Variable: Mandate_Tot

b. All requested variables entered.

Model Summary

Model	R	R Square	Adjusted R Square	Std. Error of the Estimate	Change Statistics		
					R Square Change	F Change	df1
1	.548[a]	.300	.296	1.47195	.300	79.727	1
2	.606[b]	.368	.354	1.41041	.068	6.528	3
3	.628[c]	.395	.378	1.38344	.027	8.205	1
4	.641[d]	.411	.381	1.38033	.016	1.205	4

Model Summary

Model	Change Statistics	
	df2	Sig. F Change
1	186	.000
2	183	.000
3	182	.005
4	178	.310

a. Predictors: (Constant), Party0

b. Predictors: (Constant), Party0, GenderCC, SES0, RaceCC

c. Predictors: (Constant), Party0, GenderCC, SES0, RaceCC, MRN0

d. Predictors: (Constant), Party0, GenderCC, SES0, RaceCC, MRN0, MRN0xGender, MRN0xSES0, MRN0xParty0, MRN0xRace

ANOVA[a]

Model		Sum of Squares	df	Mean Square	F	Sig.
1	Regression	172.739	1	172.739	79.727	.000[b]
	Residual	402.994	186	2.167		
	Total	575.732	187			
2	Regression	211.697	4	52.924	26.605	.000[c]
	Residual	364.035	183	1.989		
	Total	575.732	187			
3	Regression	227.401	5	45.480	23.763	.000[d]
	Residual	348.332	182	1.914		
	Total	575.732	187			
4	Regression	236.586	9	26.287	13.797	.000[e]
	Residual	339.146	178	1.905		
	Total	575.732	187			

a. Dependent Variable: Mandate_Tot

b. Predictors: (Constant), Party0

c. Predictors: (Constant), Party0, GenderCC, SES0, RaceCC

d. Predictors: (Constant), Party0, GenderCC, SES0, RaceCC, MRN0

e. Predictors: (Constant), Party0, GenderCC, SES0, RaceCC, MRN0, MRN0xGender, MRN0xSES0, MRN0xParty0, MRN0xRace

Coefficients[a]

Model		Unstandardized Coefficients B	Unstandardized Coefficients Std. Error	Standardized Coefficients Beta	t	Sig.
1	(Constant)	4.677	.107		43.566	.000
	Party0	-.788	.088	-.548	-8.929	.000
2	(Constant)	4.755	.114		41.885	.000
	Party0	-.770	.090	-.536	-8.576	.000
	GenderCC	-.403	.104	-.230	-3.857	.000
	RaceCC	-.194	.119	-.101	-1.631	.105
	SES0	.311	.144	.130	2.167	.032
3	(Constant)	4.750	.111		42.652	.000
	Party0	-.670	.095	-.466	-7.065	.000
	GenderCC	-.244	.116	-.140	-2.098	.037
	RaceCC	-.186	.117	-.097	-1.593	.113
	SES0	.355	.142	.149	2.507	.013
	MRN0	-.421	.147	-.205	-2.864	.005
4	(Constant)	4.818	.128		37.712	.000
	Party0	-.626	.097	-.435	-6.426	.000
	GenderCC	-.250	.116	-.143	-2.148	.033
	RaceCC	-.187	.118	-.097	-1.576	.117
	SES0	.353	.145	.148	2.440	.016
	MRN0	-.393	.152	-.192	-2.588	.010
	MRN0xRace	-.163	.136	-.079	-1.199	.232
	MRN0xSES0	-.050	.195	-.015	-.258	.797
	MRN0xGender	.005	.135	.002	.039	.969
	MRN0xParty0	-.142	.114	-.079	-1.244	.215

Coefficients[a]

Model		Correlations Zero-order	Partial	Part
1	(Constant)			
	Party0	-.548	-.548	-.548
2	(Constant)			
	Party0	-.548	-.535	-.504
	GenderCC	-.229	-.274	-.227
	RaceCC	-.209	-.120	-.096
	SES0	.003	.158	.127
3	(Constant)			
	Party0	-.548	-.464	-.407
	GenderCC	-.229	-.154	-.121
	RaceCC	-.209	-.117	-.092
	SES0	.003	.183	.145
	MRN0	-.425	-.208	-.165
4	(Constant)			
	Party0	-.548	-.434	-.370
	GenderCC	-.229	-.159	-.124
	RaceCC	-.209	-.117	-.091
	SES0	.003	.180	.140
	MRN0	-.425	-.190	-.149
	MRN0xRace	-.294	-.090	-.069
	MRN0xSES0	-.085	-.019	-.015
	MRN0xGender	.048	.003	.002
	MRN0xParty0	-.163	-.093	-.072

a. Dependent Variable: Mandate_Tot

Excluded Variables[a]

Model		Beta In	t	Sig.	Partial Correlation	Collinearity Statistics Tolerance
1	GenderCC	-.208[b]	-3.493	.001	-.249	.998
	RaceCC	-.059[b]	-.918	.360	-.067	.920
	SES0	.115[b]	1.842	.067	.134	.962
	MRN0	-.253[b]	-3.961	.000	-.280	.854
	MRN0xRace	-.152[b]	-2.415	.017	-.175	.922
	MRN0xSES0	-.062[b]	-1.011	.313	-.074	.998
	MRN0xGender	-.005[b]	-.083	.934	-.006	.991
	MRN0xParty0	-.079[b]	-1.280	.202	-.094	.975
2	MRN0	-.205[c]	-2.864	.005	-.208	.650
	MRN0xRace	-.141[c]	-2.303	.022	-.168	.895
	MRN0xSES0	-.035[c]	-.574	.567	-.043	.956
	MRN0xGender	-.012[c]	-.199	.842	-.015	.989
	MRN0xParty0	-.095[c]	-1.580	.116	-.116	.958
3	MRN0xRace	-.109[d]	-1.750	.082	-.129	.853
	MRN0xSES0	-.038[d]	-.648	.518	-.048	.955
	MRN0xGender	-.006[d]	-.110	.913	-.008	.988
	MRN0xParty0	-.106[d]	-1.811	.072	-.133	.953

a. Dependent Variable: Mandate_Tot

b. Predictors in the Model: (Constant), Party0

c. Predictors in the Model: (Constant), Party0, GenderCC, SES0, RaceCC

d. Predictors in the Model: (Constant), Party0, GenderCC, SES0, RaceCC, MRN0

```
REGRESSION
 /MISSING LISTWISE
 /STATISTICS COEFF OUTS R ANOVA CHANGE ZPP
 /CRITERIA=PIN(.05) POUT(.10)
 /NOORIGIN
 /DEPENDENT Conspiracy_Tot
 /METHOD=ENTER Party0
 /METHOD=ENTER GenderCC RaceCC SES0
 /METHOD=ENTER MRN0
 /METHOD=ENTER MRN0xRace MRN0xSES0 MRN0xGender MRN0xParty0.
```

Regression

Notes

Output Created		15-DEC-2021 13:09:40
Comments		
Input	Data	C: \Users\njs5478\Dropbox\HM and COVID\0. Revise and Resubmit\2. R and R Data\Study 2a\Study2a_Data.sav
	Active Dataset	DataSet1
	Filter	<none>
	Weight	<none>
	Split File	<none>
	N of Rows in Working Data File	188
Missing Value Handling	Definition of Missing	User-defined missing values are treated as missing.
	Cases Used	Statistics are based on cases with no missing values for any variable used.
Syntax		REGRESSION /MISSING LISTWISE /STATISTICS COEFF OUTS R ANOVA CHANGE ZPP /CRITERIA=PIN(.05) POUT(.10) /NOORIGIN /DEPENDENT Conspiracy_Tot /METHOD=ENTER Party0 /METHOD=ENTER GenderCC RaceCC SES0 /METHOD=ENTER MRN0 /METHOD=ENTER MRN0xRace MRN0xSES0 MRN0xGender MRN0xParty0.
Resources	Processor Time	00:00:00.02
	Elapsed Time	00:00:00.02

Notes

	Memory Required	33072 bytes
	Additional Memory Required for Residual Plots	0 bytes

Variables Entered/Removed[a]

Model	Variables Entered	Variables Removed	Method
1	Party0[b]	.	Enter
2	GenderCC, SES0, RaceCC[b]	.	Enter
3	MRN0[b]	.	Enter
4	MRN0xGender, MRN0xSES0, MRN0xParty0, MRN0xRace[b]	.	Enter

a. Dependent Variable: Conspiracy_Tot

b. All requested variables entered.

Model Summary

Model	R	R Square	Adjusted R Square	Std. Error of the Estimate	Change Statistics		
					R Square Change	F Change	df1
1	.361[a]	.130	.125	.59204	.130	27.809	1
2	.384[b]	.147	.129	.59092	.017	1.235	3
3	.438[c]	.192	.170	.57679	.045	10.076	1
4	.522[d]	.273	.236	.55339	.081	4.929	4

Model Summary

Model	Change Statistics	
	df2	Sig. F Change
1	186	.000
2	183	.298
3	182	.002
4	178	.001

a. Predictors: (Constant), Party0

b. Predictors: (Constant), Party0, GenderCC, SES0, RaceCC

c. Predictors: (Constant), Party0, GenderCC, SES0, RaceCC, MRN0

d. Predictors: (Constant), Party0, GenderCC, SES0, RaceCC, MRN0, MRN0xGender, MRN0xSES0, MRN0xParty0, MRN0xRace

ANOVA[a]

Model		Sum of Squares	df	Mean Square	F	Sig.
1	Regression	9.747	1	9.747	27.809	.000[b]
	Residual	65.194	186	.351		
	Total	74.942	187			
2	Regression	11.041	4	2.760	7.905	.000[c]
	Residual	63.901	183	.349		
	Total	74.942	187			
3	Regression	14.393	5	2.879	8.653	.000[d]
	Residual	60.549	182	.333		
	Total	74.942	187			
4	Regression	20.431	9	2.270	7.413	.000[e]
	Residual	54.511	178	.306		
	Total	74.942	187			

a. Dependent Variable: Conspiracy_Tot

b. Predictors: (Constant), Party0

c. Predictors: (Constant), Party0, GenderCC, SES0, RaceCC

d. Predictors: (Constant), Party0, GenderCC, SES0, RaceCC, MRN0

e. Predictors: (Constant), Party0, GenderCC, SES0, RaceCC, MRN0, MRN0xGender, MRN0xSES0, MRN0xParty0, MRN0xRace

Coefficients[a]

Model		Unstandardized Coefficients B	Unstandardized Coefficients Std. Error	Standardized Coefficients Beta	t	Sig.
1	(Constant)	1.545	.043		35.777	.000
	Party0	.187	.035	.361	5.273	.000
2	(Constant)	1.567	.048		32.939	.000
	Party0	.188	.038	.363	5.003	.000
	GenderCC	.029	.044	.046	.665	.507
	RaceCC	-.054	.050	-.079	-1.092	.276
	SES0	.081	.060	.094	1.341	.182
3	(Constant)	1.569	.046		33.792	.000
	Party0	.142	.040	.273	3.589	.000
	GenderCC	-.044	.049	-.070	-.909	.364
	RaceCC	-.058	.049	-.084	-1.195	.234
	SES0	.060	.059	.070	1.021	.309
	MRN0	.194	.061	.262	3.174	.002
4	(Constant)	1.488	.051		29.056	.000
	Party0	.128	.039	.246	3.275	.001
	GenderCC	-.033	.047	-.053	-.715	.476
	RaceCC	-.062	.047	-.089	-1.302	.195
	SES0	.033	.058	.039	.577	.565
	MRN0	.186	.061	.252	3.058	.003
	MRN0xRace	.058	.055	.078	1.060	.290
	MRN0xSES0	-.133	.078	-.113	-1.703	.090
	MRN0xGender	.104	.054	.125	1.920	.057
	MRN0xParty0	.137	.046	.212	2.990	.003

Coefficients[a]

Model		Correlations Zero-order	Partial	Part
1	(Constant)			
	Party0	.361	.361	.361
2	(Constant)			
	Party0	.361	.347	.342
	GenderCC	.077	.049	.045
	RaceCC	.026	-.080	-.075
	SES0	.160	.099	.092
3	(Constant)			
	Party0	.361	.257	.239
	GenderCC	.077	-.067	-.061
	RaceCC	.026	-.088	-.080
	SES0	.160	.075	.068
	MRN0	.342	.229	.211
4	(Constant)			
	Party0	.361	.238	.209
	GenderCC	.077	-.053	-.046
	RaceCC	.026	-.097	-.083
	SES0	.160	.043	.037
	MRN0	.342	.223	.195
	MRN0xRace	.275	.079	.068
	MRN0xSES0	-.067	-.127	-.109
	MRN0xGender	.121	.142	.123
	MRN0xParty0	.253	.219	.191

a. Dependent Variable: Conspiracy_Tot

Excluded Variables[a]

Model		Beta In	t	Sig.	Partial Correlation	Collinearity Statistics Tolerance
1	GenderCC	.063[b]	.915	.361	.067	.998
	RaceCC	-.083[b]	-1.162	.247	-.085	.920
	SES0	.093[b]	1.338	.182	.098	.962
	MRN0	.239[b]	3.323	.001	.237	.854
	MRN0xRace	.189[b]	2.696	.008	.194	.922
	MRN0xSES0	-.082[b]	-1.206	.229	-.088	.998
	MRN0xGender	.158[b]	2.319	.021	.168	.991
	MRN0xParty0	.201[b]	2.970	.003	.213	.975
2	MRN0	.262[c]	3.174	.002	.229	.650
	MRN0xRace	.174[c]	2.450	.015	.179	.895
	MRN0xSES0	-.066[c]	-.944	.346	-.070	.956
	MRN0xGender	.153[c]	2.255	.025	.165	.989
	MRN0xParty0	.217[c]	3.195	.002	.230	.958
3	MRN0xRace	.132[d]	1.843	.067	.136	.853
	MRN0xSES0	-.061[d]	-.898	.370	-.067	.955
	MRN0xGender	.146[d]	2.206	.029	.162	.988
	MRN0xParty0	.233[d]	3.518	.001	.253	.953

a. Dependent Variable: Conspiracy_Tot

b. Predictors in the Model: (Constant), Party0

c. Predictors in the Model: (Constant), Party0, GenderCC, SES0, RaceCC

d. Predictors in the Model: (Constant), Party0, GenderCC, SES0, RaceCC, MRN0

```
 *Including PIdeology

REGRESSION
  /MISSING LISTWISE
  /STATISTICS COEFF OUTS R ANOVA CHANGE ZPP
  /CRITERIA=PIN(.05) POUT(.10)
  /NOORIGIN
  /DEPENDENT Concern_Tot
  /METHOD=ENTER Ideology0
  /METHOD=ENTER GenderCC RaceCC SES0
```

```
/METHOD=ENTER MRN0
/METHOD=ENTER MRN0xRace MRN0xSES0 MRN0xGender MRN0xIdeology0.
```

Regression

Notes

Output Created		15-DEC-2021 13:09:40
Comments		
Input	Data	C: \Users\njs5478\Dropbox\HM and COVID\0. Revise and Resubmit\2. R and R Data\Study 2a\Study2a_Data.sav
	Active Dataset	DataSet1
	Filter	<none>
	Weight	<none>
	Split File	<none>
	N of Rows in Working Data File	188
Missing Value Handling	Definition of Missing	User-defined missing values are treated as missing.
	Cases Used	Statistics are based on cases with no missing values for any variable used.
Syntax		REGRESSION /MISSING LISTWISE /STATISTICS COEFF OUTS R ANOVA CHANGE ZPP /CRITERIA=PIN(.05) POUT(.10) /NOORIGIN /DEPENDENT Concern_Tot /METHOD=ENTER Ideology0 /METHOD=ENTER GenderCC RaceCC SES0 /METHOD=ENTER MRN0 /METHOD=ENTER MRN0xRace MRN0xSES0 MRN0xGender MRN0xIdeology0.

Notes

Resources	Processor Time	00:00:00.02
	Elapsed Time	00:00:00.02
	Memory Required	33072 bytes
	Additional Memory Required for Residual Plots	0 bytes

Variables Entered/Removed[a]

Model	Variables Entered	Variables Removed	Method
1	Ideology0[b]	.	Enter
2	SES0, GenderCC, RaceCC[b]	.	Enter
3	MRN0[b]	.	Enter
4	MRN0xGender, MRN0xSES0, MRN0xIdeology0, MRN0xRace[b]	.	Enter

a. Dependent Variable: Concern_Tot

b. All requested variables entered.

Model Summary

Model	R	R Square	Adjusted R Square	Std. Error of the Estimate	Change Statistics		
					R Square Change	F Change	df1
1	.504[a]	.254	.250	1.18060	.254	63.260	1
2	.575[b]	.331	.316	1.12737	.077	6.993	3
3	.583[c]	.340	.322	1.12274	.009	2.511	1
4	.602[d]	.363	.331	1.11506	.023	1.629	4

Model Summary

Model	Change Statistics df2	Sig. F Change
1	186	.000
2	183	.000
3	182	.115
4	178	.169

a. Predictors: (Constant), Ideology0

b. Predictors: (Constant), Ideology0, SES0, GenderCC, RaceCC

c. Predictors: (Constant), Ideology0, SES0, GenderCC, RaceCC, MRN0

d. Predictors: (Constant), Ideology0, SES0, GenderCC, RaceCC, MRN0, MRN0xGender, MRN0xSES0, MRN0xIdeology0, MRN0xRace

ANOVA[a]

Model		Sum of Squares	df	Mean Square	F	Sig.
1	Regression	88.173	1	88.173	63.260	.000[b]
	Residual	259.248	186	1.394		
	Total	347.421	187			
2	Regression	114.837	4	28.709	22.589	.000[c]
	Residual	232.585	183	1.271		
	Total	347.421	187			
3	Regression	118.002	5	23.600	18.722	.000[d]
	Residual	229.419	182	1.261		
	Total	347.421	187			
4	Regression	126.104	9	14.012	11.269	.000[e]
	Residual	221.317	178	1.243		
	Total	347.421	187			

a. Dependent Variable: Concern_Tot

b. Predictors: (Constant), Ideology0

c. Predictors: (Constant), Ideology0, SES0, GenderCC, RaceCC

d. Predictors: (Constant), Ideology0, SES0, GenderCC, RaceCC, MRN0

e. Predictors: (Constant), Ideology0, SES0, GenderCC, RaceCC, MRN0, MRN0xGender, MRN0xSES0, MRN0xIdeology0, MRN0xRace

Coefficients[a]

Model		Unstandardized Coefficients B	Unstandardized Coefficients Std. Error	Standardized Coefficients Beta	t	Sig.
1	(Constant)	3.630	.086		42.158	.000
	Ideology0	-.454	.057	-.504	-7.954	.000
2	(Constant)	3.715	.091		41.019	.000
	Ideology0	-.392	.057	-.435	-6.892	.000
	GenderCC	-.355	.084	-.261	-4.209	.000
	RaceCC	-.211	.094	-.142	-2.239	.026
	SES0	-.072	.113	-.039	-.631	.529
3	(Constant)	3.712	.090		41.154	.000
	Ideology0	-.351	.062	-.389	-5.620	.000
	GenderCC	-.287	.094	-.211	-3.055	.003
	RaceCC	-.207	.094	-.139	-2.204	.029
	SES0	-.046	.114	-.025	-.400	.690
	MRN0	-.193	.122	-.121	-1.585	.115
4	(Constant)	3.660	.104		35.324	.000
	Ideology0	-.332	.065	-.369	-5.094	.000
	GenderCC	-.284	.093	-.209	-3.037	.003
	RaceCC	-.265	.096	-.178	-2.751	.007
	SES0	-.054	.117	-.029	-.466	.641
	MRN0	-.128	.124	-.080	-1.028	.305
	MRN0xRace	-.218	.111	-.136	-1.966	.051
	MRN0xSES0	.041	.159	.016	.259	.796
	MRN0xGender	-.002	.109	-.001	-.014	.989
	MRN0xIdeology0	.144	.072	.134	2.009	.046

Coefficients[a]

Model		Correlations		
		Zero-order	Partial	Part
1	(Constant)			
	Ideology0	-.504	-.504	-.504
2	(Constant)			
	Ideology0	-.504	-.454	-.417
	GenderCC	-.295	-.297	-.255
	RaceCC	-.209	-.163	-.135
	SES0	-.116	-.047	-.038
3	(Constant)			
	Ideology0	-.504	-.385	-.339
	GenderCC	-.295	-.221	-.184
	RaceCC	-.209	-.161	-.133
	SES0	-.116	-.030	-.024
	MRN0	-.405	-.117	-.095
4	(Constant)			
	Ideology0	-.504	-.357	-.305
	GenderCC	-.295	-.222	-.182
	RaceCC	-.209	-.202	-.165
	SES0	-.116	-.035	-.028
	MRN0	-.405	-.077	-.062
	MRN0xRace	-.288	-.146	-.118
	MRN0xSES0	-.016	.019	.016
	MRN0xGender	.063	-.001	-.001
	MRN0xIdeology0	.024	.149	.120

a. Dependent Variable: Concern_Tot

Excluded Variables[a]

Model		Beta In	t	Sig.	Partial Correlation	Collinearity Statistics
						Tolerance
1	GenderCC	-.238[b]	-3.877	.000	-.274	.986
	RaceCC	-.098[b]	-1.514	.132	-.111	.947
	SES0	-.060[b]	-.933	.352	-.068	.987
	MRN0	-.225[b]	-3.260	.001	-.233	.800
	MRN0xRace	-.118[b]	-1.747	.082	-.127	.864
	MRN0xSES0	.045[b]	.701	.484	.051	.986
	MRN0xGender	.022[b]	.345	.730	.025	.993
	MRN0xIdeology0	.097[b]	1.525	.129	.111	.980
2	MRN0	-.121[c]	-1.585	.115	-.117	.621
	MRN0xRace	-.109[c]	-1.658	.099	-.122	.841
	MRN0xSES0	.036[c]	.580	.563	.043	.943
	MRN0xGender	.019[c]	.310	.757	.023	.991
	MRN0xIdeology0	.107[c]	1.721	.087	.127	.937
3	MRN0xRace	-.094[d]	-1.412	.160	-.104	.816
	MRN0xSES0	.031[d]	.493	.623	.037	.940
	MRN0xGender	.022[d]	.359	.720	.027	.991
	MRN0xIdeology0	.100[d]	1.607	.110	.119	.931

a. Dependent Variable: Concern_Tot

b. Predictors in the Model: (Constant), Ideology0

c. Predictors in the Model: (Constant), Ideology0, SES0, GenderCC, RaceCC

d. Predictors in the Model: (Constant), Ideology0, SES0, GenderCC, RaceCC, MRN0

```
REGRESSION
 /MISSING LISTWISE
 /STATISTICS COEFF OUTS R ANOVA CHANGE ZPP
 /CRITERIA=PIN(.05) POUT(.10)
 /NOORIGIN
 /DEPENDENT Finance_Tot
 /METHOD=ENTER Ideology0
 /METHOD=ENTER GenderCC RaceCC SES0
 /METHOD=ENTER MRN0
 /METHOD=ENTER MRN0xRace MRN0xSES0 MRN0xGender MRN0xIdeology0.
```

Regression

Notes

Output Created		15-DEC-2021 13:09:40
Comments		
Input	Data	C: \Users\njs5478\Dropbox\HM and COVID\0. Revise and Resubmit\2. R and R Data\Study 2a\Study2a_Data.sav
	Active Dataset	DataSet1
	Filter	<none>
	Weight	<none>
	Split File	<none>
	N of Rows in Working Data File	188
Missing Value Handling	Definition of Missing	User-defined missing values are treated as missing.
	Cases Used	Statistics are based on cases with no missing values for any variable used.
Syntax		REGRESSION /MISSING LISTWISE /STATISTICS COEFF OUTS R ANOVA CHANGE ZPP /CRITERIA=PIN(.05) POUT(.10) /NOORIGIN /DEPENDENT Finance_Tot /METHOD=ENTER Ideology0 /METHOD=ENTER GenderCC RaceCC SES0 /METHOD=ENTER MRN0 /METHOD=ENTER MRN0xRace MRN0xSES0 MRN0xGender MRN0xIdeology0.
Resources	Processor Time	00:00:00.02
	Elapsed Time	00:00:00.02

Notes

	Memory Required	33072 bytes
	Additional Memory Required for Residual Plots	0 bytes

Variables Entered/Removed[a]

Model	Variables Entered	Variables Removed	Method
1	Ideology0[b]	.	Enter
2	SES0, GenderCC, RaceCC[b]	.	Enter
3	MRN0[b]	.	Enter
4	MRN0xGender, MRN0xSES0, MRN0xIdeology0, MRN0xRace[b]	.	Enter

a. Dependent Variable: Finance_Tot

b. All requested variables entered.

Model Summary

Model	R	R Square	Adjusted R Square	Std. Error of the Estimate	Change Statistics		
					R Square Change	F Change	df1
1	.080[a]	.006	.001	1.46100	.006	1.193	1
2	.357[b]	.128	.108	1.38021	.121	8.471	3
3	.362[c]	.131	.108	1.38092	.004	.811	1
4	.371[d]	.138	.094	1.39136	.006	.320	4

Model Summary

Model	Change Statistics	
	df2	Sig. F Change
1	186	.276
2	183	.000
3	182	.369
4	178	.864

a. Predictors: (Constant), Ideology0

b. Predictors: (Constant), Ideology0, SES0, GenderCC, RaceCC

c. Predictors: (Constant), Ideology0, SES0, GenderCC, RaceCC, MRN0

d. Predictors: (Constant), Ideology0, SES0, GenderCC, RaceCC, MRN0, MRN0xGender, MRN0xSES0, MRN0xIdeology0, MRN0xRace

ANOVA[a]

Model		Sum of Squares	df	Mean Square	F	Sig.
1	Regression	2.546	1	2.546	1.193	.276[b]
	Residual	397.021	186	2.135		
	Total	399.567	187			
2	Regression	50.957	4	12.739	6.687	.000[c]
	Residual	348.610	183	1.905		
	Total	399.567	187			
3	Regression	52.504	5	10.501	5.507	.000[d]
	Residual	347.063	182	1.907		
	Total	399.567	187			
4	Regression	54.982	9	6.109	3.156	.001[e]
	Residual	344.585	178	1.936		
	Total	399.567	187			

a. Dependent Variable: Finance_Tot

b. Predictors: (Constant), Ideology0

c. Predictors: (Constant), Ideology0, SES0, GenderCC, RaceCC

d. Predictors: (Constant), Ideology0, SES0, GenderCC, RaceCC, MRN0

e. Predictors: (Constant), Ideology0, SES0, GenderCC, RaceCC, MRN0, MRN0xGender, MRN0xSES0, MRN0xIdeology0, MRN0xRace

Coefficients[a]

Model		Unstandardized Coefficients B	Unstandardized Coefficients Std. Error	Standardized Coefficients Beta	t	Sig.
1	(Constant)	3.770	.107		35.379	.000
	Ideology0	-.077	.071	-.080	-1.092	.276
2	(Constant)	3.803	.111		34.301	.000
	Ideology0	-.018	.070	-.019	-.264	.792
	GenderCC	-.146	.103	-.100	-1.417	.158
	RaceCC	-.082	.115	-.051	-.710	.479
	SES0	-.651	.139	-.327	-4.685	.000
3	(Constant)	3.802	.111		34.262	.000
	Ideology0	.011	.077	.011	.138	.891
	GenderCC	-.099	.116	-.068	-.856	.393
	RaceCC	-.079	.115	-.050	-.685	.494
	SES0	-.632	.140	-.318	-4.504	.000
	MRN0	-.135	.150	-.079	-.901	.369
4	(Constant)	3.797	.129		29.370	.000
	Ideology0	-.004	.081	-.004	-.049	.961
	GenderCC	-.099	.117	-.068	-.846	.399
	RaceCC	-.102	.120	-.064	-.848	.397
	SES0	-.635	.146	-.319	-4.359	.000
	MRN0	-.117	.155	-.068	-.752	.453
	MRN0xRace	-.010	.138	-.006	-.069	.945
	MRN0xSES0	.054	.199	.020	.270	.787
	MRN0xGender	-.093	.136	-.048	-.682	.496
	MRN0xIdeology0	.077	.089	.067	.866	.388

Coefficients[a]

Model		Correlations		
		Zero-order	Partial	Part
1	(Constant)			
	Ideology0	-.080	-.080	-.080
2	(Constant)			
	Ideology0	-.080	-.020	-.018
	GenderCC	-.114	-.104	-.098
	RaceCC	-.071	-.052	-.049
	SES0	-.339	-.327	-.323
3	(Constant)			
	Ideology0	-.080	.010	.010
	GenderCC	-.114	-.063	-.059
	RaceCC	-.071	-.051	-.047
	SES0	-.339	-.317	-.311
	MRN0	-.167	-.067	-.062
4	(Constant)			
	Ideology0	-.080	-.004	-.003
	GenderCC	-.114	-.063	-.059
	RaceCC	-.071	-.063	-.059
	SES0	-.339	-.311	-.303
	MRN0	-.167	-.056	-.052
	MRN0xRace	-.044	-.005	-.005
	MRN0xSES0	.090	.020	.019
	MRN0xGender	-.038	-.051	-.047
	MRN0xIdeology0	.023	.065	.060

a. Dependent Variable: Finance_Tot

Excluded Variables[a]

Model		Beta In	t	Sig.	Partial Correlation	Collinearity Statistics Tolerance
1	GenderCC	-.106[b]	-1.448	.149	-.106	.986
	RaceCC	-.056[b]	-.739	.461	-.054	.947
	SES0	-.335[b]	-4.812	.000	-.334	.987
	MRN0	-.164[b]	-2.023	.045	-.147	.800
	MRN0xRace	-.017[b]	-.217	.829	-.016	.864
	MRN0xSES0	.101[b]	1.375	.171	.101	.986
	MRN0xGender	-.044[b]	-.605	.546	-.044	.993
	MRN0xIdeology0	.035[b]	.467	.641	.034	.980
2	MRN0	-.079[c]	-.901	.369	-.067	.621
	MRN0xRace	.007[c]	.087	.931	.006	.841
	MRN0xSES0	.033[c]	.469	.640	.035	.943
	MRN0xGender	-.040[c]	-.578	.564	-.043	.991
	MRN0xIdeology0	.067[c]	.941	.348	.070	.937
3	MRN0xRace	.019[d]	.244	.808	.018	.816
	MRN0xSES0	.030[d]	.418	.676	.031	.940
	MRN0xGender	-.038[d]	-.551	.582	-.041	.991
	MRN0xIdeology0	.063[d]	.873	.384	.065	.931

a. Dependent Variable: Finance_Tot

b. Predictors in the Model: (Constant), Ideology0

c. Predictors in the Model: (Constant), Ideology0, SES0, GenderCC, RaceCC

d. Predictors in the Model: (Constant), Ideology0, SES0, GenderCC, RaceCC, MRN0

```
REGRESSION
 /MISSING LISTWISE
 /STATISTICS COEFF OUTS R ANOVA CHANGE ZPP
 /CRITERIA=PIN(.05) POUT(.10)
 /NOORIGIN
 /DEPENDENT Psychology_Tot
 /METHOD=ENTER Ideology0
 /METHOD=ENTER GenderCC RaceCC SES0
 /METHOD=ENTER MRN0
 /METHOD=ENTER MRN0xRace MRN0xSES0 MRN0xGender MRN0xIdeology0.
```

Regression

Notes

Output Created		15-DEC-2021 13:09:40
Comments		
Input	Data	C:\Users\njs5478\Dropbox\HM and COVID\0. Revise and Resubmit\2. R and R Data\Study 2a\Study2a_Data.sav
	Active Dataset	DataSet1
	Filter	<none>
	Weight	<none>
	Split File	<none>
	N of Rows in Working Data File	188
Missing Value Handling	Definition of Missing	User-defined missing values are treated as missing.
	Cases Used	Statistics are based on cases with no missing values for any variable used.
Syntax		REGRESSION /MISSING LISTWISE /STATISTICS COEFF OUTS R ANOVA CHANGE ZPP /CRITERIA=PIN(.05) POUT(.10) /NOORIGIN /DEPENDENT Psychology_Tot /METHOD=ENTER Ideology0 /METHOD=ENTER GenderCC RaceCC SES0 /METHOD=ENTER MRN0 /METHOD=ENTER MRN0xRace MRN0xSES0 MRN0xGender MRN0xIdeology0.
Resources	Processor Time	00:00:00.00
	Elapsed Time	00:00:00.02

Notes

	Memory Required	33072 bytes
	Additional Memory Required for Residual Plots	0 bytes

Variables Entered/Removed[a]

Model	Variables Entered	Variables Removed	Method
1	Ideology0[b]	.	Enter
2	SES0, GenderCC, RaceCC[b]	.	Enter
3	MRN0[b]	.	Enter
4	MRN0xGender, MRN0xSES0, MRN0xIdeology0, MRN0xRace[b]	.	Enter

a. Dependent Variable: Psychology_Tot

b. All requested variables entered.

Model Summary

Model	R	R Square	Adjusted R Square	Std. Error of the Estimate	Change Statistics		
					R Square Change	F Change	df1
1	.172[a]	.030	.024	1.40882	.030	5.692	1
2	.214[b]	.046	.025	1.40838	.016	1.039	3
3	.242[c]	.059	.033	1.40275	.013	2.472	1
4	.323[d]	.104	.059	1.38371	.046	2.261	4

Model Summary

Model	Change Statistics	
	df2	Sig. F Change
1	186	.018
2	183	.377
3	182	.118
4	178	.064

a. Predictors: (Constant), Ideology0

b. Predictors: (Constant), Ideology0, SES0, GenderCC, RaceCC

c. Predictors: (Constant), Ideology0, SES0, GenderCC, RaceCC, MRN0

d. Predictors: (Constant), Ideology0, SES0, GenderCC, RaceCC, MRN0, MRN0xGender, MRN0xSES0, MRN0xIdeology0, MRN0xRace

ANOVA[a]

Model		Sum of Squares	df	Mean Square	F	Sig.
1	Regression	11.297	1	11.297	5.692	.018[b]
	Residual	369.168	186	1.985		
	Total	380.465	187			
2	Regression	17.478	4	4.369	2.203	.070[c]
	Residual	362.987	183	1.984		
	Total	380.465	187			
3	Regression	22.342	5	4.468	2.271	.049[d]
	Residual	358.123	182	1.968		
	Total	380.465	187			
4	Regression	39.656	9	4.406	2.301	.018[e]
	Residual	340.809	178	1.915		
	Total	380.465	187			

a. Dependent Variable: Psychology_Tot

b. Predictors: (Constant), Ideology0

c. Predictors: (Constant), Ideology0, SES0, GenderCC, RaceCC

d. Predictors: (Constant), Ideology0, SES0, GenderCC, RaceCC, MRN0

e. Predictors: (Constant), Ideology0, SES0, GenderCC, RaceCC, MRN0, MRN0xGender, MRN0xSES0, MRN0xIdeology0, MRN0xRace

Coefficients[a]

Model		Unstandardized Coefficients B	Unstandardized Coefficients Std. Error	Standardized Coefficients Beta	t	Sig.
1	(Constant)	4.541	.103		44.198	.000
	Ideology0	-.162	.068	-.172	-2.386	.018
2	(Constant)	4.514	.113		39.897	.000
	Ideology0	-.155	.071	-.165	-2.184	.030
	GenderCC	-.149	.105	-.105	-1.420	.157
	RaceCC	.067	.118	.043	.573	.567
	SES0	-.085	.142	-.044	-.603	.547
3	(Constant)	4.511	.113		40.024	.000
	Ideology0	-.104	.078	-.110	-1.333	.184
	GenderCC	-.066	.117	-.046	-.562	.575
	RaceCC	.072	.117	.047	.618	.537
	SES0	-.053	.143	-.027	-.373	.710
	MRN0	-.240	.152	-.144	-1.572	.118
4	(Constant)	4.381	.129		34.075	.000
	Ideology0	-.088	.081	-.093	-1.086	.279
	GenderCC	-.060	.116	-.042	-.513	.609
	RaceCC	-.002	.119	-.001	-.017	.986
	SES0	-.069	.145	-.035	-.476	.635
	MRN0	-.161	.154	-.097	-1.046	.297
	MRN0xRace	-.239	.138	-.143	-1.740	.084
	MRN0xSES0	.079	.198	.030	.402	.688
	MRN0xGender	.098	.136	.052	.723	.471
	MRN0xIdeology0	.216	.089	.192	2.437	.016

Coefficients[a]

Model		Correlations Zero-order	Partial	Part
1	(Constant)			
	Ideology0	-.172	-.172	-.172
2	(Constant)			
	Ideology0	-.172	-.159	-.158
	GenderCC	-.133	-.104	-.103
	RaceCC	.016	.042	.041
	SES0	-.065	-.045	-.044
3	(Constant)			
	Ideology0	-.172	-.098	-.096
	GenderCC	-.133	-.042	-.040
	RaceCC	.016	.046	.044
	SES0	-.065	-.028	-.027
	MRN0	-.216	-.116	-.113
4	(Constant)			
	Ideology0	-.172	-.081	-.077
	GenderCC	-.133	-.038	-.036
	RaceCC	.016	-.001	-.001
	SES0	-.065	-.036	-.034
	MRN0	-.216	-.078	-.074
	MRN0xRace	-.167	-.129	-.123
	MRN0xSES0	.049	.030	.028
	MRN0xGender	.091	.054	.051
	MRN0xIdeology0	.153	.180	.173

a. Dependent Variable: Psychology_Tot

Excluded Variables[a]

Model		Beta In	t	Sig.	Partial Correlation	Collinearity Statistics Tolerance
1	GenderCC	-.114[b]	-1.578	.116	-.115	.986
	RaceCC	.059[b]	.792	.429	.058	.947
	SES0	-.046[b]	-.631	.529	-.046	.987
	MRN0	-.174[b]	-2.175	.031	-.158	.800
	MRN0xRace	-.119[b]	-1.539	.126	-.112	.864
	MRN0xSES0	.071[b]	.974	.331	.071	.986
	MRN0xGender	.077[b]	1.062	.290	.078	.993
	MRN0xIdeology0	.181[b]	2.512	.013	.182	.980
2	MRN0	-.144[c]	-1.572	.118	-.116	.621
	MRN0xRace	-.102[c]	-1.295	.197	-.096	.841
	MRN0xSES0	.064[c]	.862	.390	.064	.943
	MRN0xGender	.080[c]	1.103	.272	.081	.991
	MRN0xIdeology0	.175[c]	2.374	.019	.173	.937
3	MRN0xRace	-.083[d]	-1.046	.297	-.078	.816
	MRN0xSES0	.058[d]	.777	.438	.058	.940
	MRN0xGender	.083[d]	1.156	.249	.086	.991
	MRN0xIdeology0	.167[d]	2.263	.025	.166	.931

a. Dependent Variable: Psychology_Tot

b. Predictors in the Model: (Constant), Ideology0

c. Predictors in the Model: (Constant), Ideology0, SES0, GenderCC, RaceCC

d. Predictors in the Model: (Constant), Ideology0, SES0, GenderCC, RaceCC, MRN0

```
REGRESSION
 /MISSING LISTWISE
 /STATISTICS COEFF OUTS R ANOVA CHANGE ZPP
 /CRITERIA=PIN(.05) POUT(.10)
 /NOORIGIN
 /DEPENDENT Risk_Rules
 /METHOD=ENTER Ideology0
 /METHOD=ENTER GenderCC RaceCC SES0
 /METHOD=ENTER MRN0
 /METHOD=ENTER MRN0xRace MRN0xSES0 MRN0xGender MRN0xIdeology0.
```

Regression

Notes

Output Created		15-DEC-2021 13:09:40
Comments		
Input	Data	C: \Users\njs5478\Dropbox\HM and COVID\0. Revise and Resubmit\2. R and R Data\Study 2a\Study2a_Data.sav
	Active Dataset	DataSet1
	Filter	<none>
	Weight	<none>
	Split File	<none>
	N of Rows in Working Data File	188
Missing Value Handling	Definition of Missing	User-defined missing values are treated as missing.
	Cases Used	Statistics are based on cases with no missing values for any variable used.
Syntax		REGRESSION /MISSING LISTWISE /STATISTICS COEFF OUTS R ANOVA CHANGE ZPP /CRITERIA=PIN(.05) POUT(.10) /NOORIGIN /DEPENDENT Risk_Rules /METHOD=ENTER Ideology0 /METHOD=ENTER GenderCC RaceCC SES0 /METHOD=ENTER MRN0 /METHOD=ENTER MRN0xRace MRN0xSES0 MRN0xGender MRN0xIdeology0.
Resources	Processor Time	00:00:00.00
	Elapsed Time	00:00:00.03

Notes

	Memory Required	33072 bytes
	Additional Memory Required for Residual Plots	0 bytes

Variables Entered/Removed[a]

Model	Variables Entered	Variables Removed	Method
1	Ideology0[b]	.	Enter
2	SES0, GenderCC, RaceCC[b]	.	Enter
3	MRN0[b]	.	Enter
4	MRN0xGender, MRN0xSES0, MRN0xIdeology0, MRN0xRace[b]	.	Enter

a. Dependent Variable: Risk_Rules

b. All requested variables entered.

Model Summary

Model	R	R Square	Adjusted R Square	Std. Error of the Estimate	Change Statistics		
					R Square Change	F Change	df1
1	.528[a]	.279	.275	.91470	.279	72.077	1
2	.592[b]	.351	.336	.87535	.071	6.699	3
3	.638[c]	.407	.390	.83911	.056	17.147	1
4	.640[d]	.410	.380	.84625	.003	.235	4

Model Summary

Model	Change Statistics	
	df2	Sig. F Change
1	186	.000
2	183	.000
3	182	.000
4	178	.918

a. Predictors: (Constant), Ideology0

b. Predictors: (Constant), Ideology0, SES0, GenderCC, RaceCC

c. Predictors: (Constant), Ideology0, SES0, GenderCC, RaceCC, MRN0

d. Predictors: (Constant), Ideology0, SES0, GenderCC, RaceCC, MRN0, MRN0xGender, MRN0xSES0, MRN0xIdeology0, MRN0xRace

ANOVA[a]

Model		Sum of Squares	df	Mean Square	F	Sig.
1	Regression	60.305	1	60.305	72.077	.000[b]
	Residual	155.621	186	.837		
	Total	215.926	187			
2	Regression	75.705	4	18.926	24.700	.000[c]
	Residual	140.221	183	.766		
	Total	215.926	187			
3	Regression	87.779	5	17.556	24.933	.000[d]
	Residual	128.147	182	.704		
	Total	215.926	187			
4	Regression	88.453	9	9.828	13.724	.000[e]
	Residual	127.474	178	.716		
	Total	215.926	187			

a. Dependent Variable: Risk_Rules

b. Predictors: (Constant), Ideology0

c. Predictors: (Constant), Ideology0, SES0, GenderCC, RaceCC

d. Predictors: (Constant), Ideology0, SES0, GenderCC, RaceCC, MRN0

e. Predictors: (Constant), Ideology0, SES0, GenderCC, RaceCC, MRN0, MRN0xGender, MRN0xSES0, MRN0xIdeology0, MRN0xRace

Coefficients[a]

Model		Unstandardized Coefficients B	Unstandardized Coefficients Std. Error	Standardized Coefficients Beta	t	Sig.
1	(Constant)	3.924	.067		58.826	.000
	Ideology0	.375	.044	.528	8.490	.000
2	(Constant)	3.838	.070		54.574	.000
	Ideology0	.320	.044	.451	7.251	.000
	GenderCC	.190	.065	.177	2.907	.004
	RaceCC	.214	.073	.183	2.930	.004
	SES0	.179	.088	.122	2.032	.044
3	(Constant)	3.843	.067		56.995	.000
	Ideology0	.239	.047	.337	5.134	.000
	GenderCC	.059	.070	.055	.835	.405
	RaceCC	.206	.070	.176	2.943	.004
	SES0	.128	.085	.088	1.503	.134
	MRN0	.377	.091	.300	4.141	.000
4	(Constant)	3.864	.079		49.139	.000
	Ideology0	.231	.049	.326	4.675	.000
	GenderCC	.058	.071	.054	.821	.413
	RaceCC	.218	.073	.186	2.986	.003
	SES0	.126	.089	.086	1.419	.158
	MRN0	.361	.094	.287	3.826	.000
	MRN0xRace	.065	.084	.051	.769	.443
	MRN0xSES0	-.030	.121	-.015	-.245	.806
	MRN0xGender	-.031	.083	-.022	-.371	.711
	MRN0xIdeology0	-.024	.054	-.028	-.435	.664

Coefficients[a]

Model		Correlations		
		Zero-order	Partial	Part
1	(Constant)			
	Ideology0	.528	.528	.528
2	(Constant)			
	Ideology0	.528	.472	.432
	GenderCC	.213	.210	.173
	RaceCC	.273	.212	.175
	SES0	.200	.149	.121
3	(Constant)			
	Ideology0	.528	.356	.293
	GenderCC	.213	.062	.048
	RaceCC	.273	.213	.168
	SES0	.200	.111	.086
	MRN0	.503	.293	.236
4	(Constant)			
	Ideology0	.528	.331	.269
	GenderCC	.213	.061	.047
	RaceCC	.273	.218	.172
	SES0	.200	.106	.082
	MRN0	.503	.276	.220
	MRN0xRace	.269	.058	.044
	MRN0xSES0	.007	-.018	-.014
	MRN0xGender	-.067	-.028	-.021
	MRN0xIdeology0	.065	-.033	-.025

a. Dependent Variable: Risk_Rules

Excluded Variables[a]

Model		Beta In	t	Sig.	Partial Correlation	Collinearity Statistics Tolerance
1	GenderCC	.152[b]	2.456	.015	.178	.986
	RaceCC	.160[b]	2.530	.012	.183	.947
	SES0	.142[b]	2.294	.023	.166	.987
	MRN0	.334[b]	5.110	.000	.352	.800
	MRN0xRace	.085[b]	1.275	.204	.093	.864
	MRN0xSES0	-.057[b]	-.907	.366	-.067	.986
	MRN0xGender	-.024[b]	-.383	.702	-.028	.993
	MRN0xIdeology0	-.010[b]	-.151	.880	-.011	.980
2	MRN0	.300[c]	4.141	.000	.293	.621
	MRN0xRace	.085[c]	1.309	.192	.097	.841
	MRN0xSES0	-.031[c]	-.501	.617	-.037	.943
	MRN0xGender	-.020[c]	-.340	.734	-.025	.991
	MRN0xIdeology0	-.039[c]	-.632	.528	-.047	.937
3	MRN0xRace	.042[d]	.666	.506	.049	.816
	MRN0xSES0	-.017[d]	-.289	.773	-.021	.940
	MRN0xGender	-.028[d]	-.482	.631	-.036	.991
	MRN0xIdeology0	-.020[d]	-.337	.736	-.025	.931

a. Dependent Variable: Risk_Rules

b. Predictors in the Model: (Constant), Ideology0

c. Predictors in the Model: (Constant), Ideology0, SES0, GenderCC, RaceCC

d. Predictors in the Model: (Constant), Ideology0, SES0, GenderCC, RaceCC, MRN0

```
REGRESSION
 /MISSING LISTWISE
 /STATISTICS COEFF OUTS R ANOVA CHANGE ZPP
 /CRITERIA=PIN(.05) POUT(.10)
 /NOORIGIN
 /DEPENDENT Mandate_Tot
 /METHOD=ENTER Ideology0
 /METHOD=ENTER GenderCC RaceCC SES0
 /METHOD=ENTER MRN0
 /METHOD=ENTER MRN0xRace MRN0xSES0 MRN0xGender MRN0xIdeology0.
```

Regression

Notes

Output Created		15-DEC-2021 13:09:40
Comments		
Input	Data	C: \Users\njs5478\Dropbox\HM and COVID\0. Revise and Resubmit\2. R and R Data\Study 2a\Study2a_Data.sav
	Active Dataset	DataSet1
	Filter	<none>
	Weight	<none>
	Split File	<none>
	N of Rows in Working Data File	188
Missing Value Handling	Definition of Missing	User-defined missing values are treated as missing.
	Cases Used	Statistics are based on cases with no missing values for any variable used.
Syntax		REGRESSION /MISSING LISTWISE /STATISTICS COEFF OUTS R ANOVA CHANGE ZPP /CRITERIA=PIN(.05) POUT(.10) /NOORIGIN /DEPENDENT Mandate_Tot /METHOD=ENTER Ideology0 /METHOD=ENTER GenderCC RaceCC SES0 /METHOD=ENTER MRN0 /METHOD=ENTER MRN0xRace MRN0xSES0 MRN0xGender MRN0xIdeology0.
Resources	Processor Time	00:00:00.02
	Elapsed Time	00:00:00.02

Notes

	Memory Required	33072 bytes
	Additional Memory Required for Residual Plots	0 bytes

Variables Entered/Removed[a]

Model	Variables Entered	Variables Removed	Method
1	Ideology0[b]	.	Enter
2	SES0, GenderCC, RaceCC[b]	.	Enter
3	MRN0[b]	.	Enter
4	MRN0xGender, MRN0xSES0, MRN0xIdeology0, MRN0xRace[b]	.	Enter

a. Dependent Variable: Mandate_Tot

b. All requested variables entered.

Model Summary

Model	R	R Square	Adjusted R Square	Std. Error of the Estimate	Change Statistics		
					R Square Change	F Change	df1
1	.591[a]	.349	.345	1.41970	.349	99.644	1
2	.626[b]	.392	.379	1.38249	.044	4.383	3
3	.640[c]	.410	.393	1.36667	.017	5.259	1
4	.643[d]	.413	.384	1.37758	.004	.282	4

Model Summary

Model	Change Statistics	
	df2	Sig. F Change
1	186	.000
2	183	.005
3	182	.023
4	178	.889

a. Predictors: (Constant), Ideology0

b. Predictors: (Constant), Ideology0, SES0, GenderCC, RaceCC

c. Predictors: (Constant), Ideology0, SES0, GenderCC, RaceCC, MRN0

d. Predictors: (Constant), Ideology0, SES0, GenderCC, RaceCC, MRN0, MRN0xGender, MRN0xSES0, MRN0xIdeology0, MRN0xRace

ANOVA[a]

Model		Sum of Squares	df	Mean Square	F	Sig.
1	Regression	200.838	1	200.838	99.644	.000[b]
	Residual	374.894	186	2.016		
	Total	575.732	187			
2	Regression	225.970	4	56.493	29.558	.000[c]
	Residual	349.762	183	1.911		
	Total	575.732	187			
3	Regression	235.793	5	47.159	25.248	.000[d]
	Residual	339.939	182	1.868		
	Total	575.732	187			
4	Regression	237.935	9	26.437	13.931	.000[e]
	Residual	337.797	178	1.898		
	Total	575.732	187			

a. Dependent Variable: Mandate_Tot

b. Predictors: (Constant), Ideology0

c. Predictors: (Constant), Ideology0, SES0, GenderCC, RaceCC

d. Predictors: (Constant), Ideology0, SES0, GenderCC, RaceCC, MRN0

e. Predictors: (Constant), Ideology0, SES0, GenderCC, RaceCC, MRN0, MRN0xGender, MRN0xSES0, MRN0xIdeology0, MRN0xRace

Coefficients[a]

Model		Unstandardized Coefficients B	Unstandardized Coefficients Std. Error	Standardized Coefficients Beta	t	Sig.
1	(Constant)	4.679	.104		45.185	.000
	Ideology0	-.685	.069	-.591	-9.982	.000
2	(Constant)	4.767	.111		42.924	.000
	Ideology0	-.640	.070	-.552	-9.166	.000
	GenderCC	-.323	.103	-.184	-3.126	.002
	RaceCC	-.220	.115	-.115	-1.910	.058
	SES0	.206	.139	.086	1.482	.140
3	(Constant)	4.763	.110		43.373	.000
	Ideology0	-.567	.076	-.489	-7.459	.000
	GenderCC	-.204	.114	-.117	-1.785	.076
	RaceCC	-.213	.114	-.111	-1.869	.063
	SES0	.252	.139	.105	1.813	.071
	MRN0	-.340	.148	-.166	-2.293	.023
4	(Constant)	4.778	.128		37.325	.000
	Ideology0	-.543	.081	-.469	-6.742	.000
	GenderCC	-.205	.115	-.117	-1.774	.078
	RaceCC	-.229	.119	-.120	-1.931	.055
	SES0	.257	.144	.107	1.781	.077
	MRN0	-.312	.154	-.152	-2.031	.044
	MRN0xRace	-.140	.137	-.068	-1.024	.307
	MRN0xSES0	.000	.197	.000	.002	.998
	MRN0xGender	-.008	.135	-.004	-.063	.950
	MRN0xIdeology0	.003	.088	.002	.035	.972

Coefficients[a]

Model		Correlations		
		Zero-order	Partial	Part
1	(Constant)			
	Ideology0	-.591	-.591	-.591
2	(Constant)			
	Ideology0	-.591	-.561	-.528
	GenderCC	-.229	-.225	-.180
	RaceCC	-.209	-.140	-.110
	SES0	.003	.109	.085
3	(Constant)			
	Ideology0	-.591	-.484	-.425
	GenderCC	-.229	-.131	-.102
	RaceCC	-.209	-.137	-.106
	SES0	.003	.133	.103
	MRN0	-.425	-.168	-.131
4	(Constant)			
	Ideology0	-.591	-.451	-.387
	GenderCC	-.229	-.132	-.102
	RaceCC	-.209	-.143	-.111
	SES0	.003	.132	.102
	MRN0	-.425	-.150	-.117
	MRN0xRace	-.294	-.077	-.059
	MRN0xSES0	-.085	.000	.000
	MRN0xGender	.048	-.005	-.004
	MRN0xIdeology0	-.085	.003	.002

a. Dependent Variable: Mandate_Tot

Excluded Variables[a]

Model		Beta In	t	Sig.	Partial Correlation	Collinearity Statistics Tolerance
1	GenderCC	-.161[b]	-2.749	.007	-.198	.986
	RaceCC	-.077[b]	-1.264	.208	-.093	.947
	SES0	.071[b]	1.185	.237	.087	.987
	MRN0	-.202[b]	-3.119	.002	-.224	.800
	MRN0xRace	-.087[b]	-1.377	.170	-.101	.864
	MRN0xSES0	-.014[b]	-.238	.812	-.017	.986
	MRN0xGender	.000[b]	-.008	.994	-.001	.993
	MRN0xIdeology0	-.002[b]	-.028	.978	-.002	.980
2	MRN0	-.166[c]	-2.293	.023	-.168	.621
	MRN0xRace	-.090[c]	-1.431	.154	-.105	.841
	MRN0xSES0	.002[c]	.039	.969	.003	.943
	MRN0xGender	-.005[c]	-.091	.928	-.007	.991
	MRN0xIdeology0	-.006[c]	-.100	.920	-.007	.937
3	MRN0xRace	-.067[d]	-1.069	.286	-.079	.816
	MRN0xSES0	-.005[d]	-.090	.928	-.007	.940
	MRN0xGender	-.001[d]	-.022	.983	-.002	.991
	MRN0xIdeology0	-.017[d]	-.281	.779	-.021	.931

a. Dependent Variable: Mandate_Tot

b. Predictors in the Model: (Constant), Ideology0

c. Predictors in the Model: (Constant), Ideology0, SES0, GenderCC, RaceCC

d. Predictors in the Model: (Constant), Ideology0, SES0, GenderCC, RaceCC, MRN0

```
REGRESSION
 /MISSING LISTWISE
 /STATISTICS COEFF OUTS R ANOVA CHANGE ZPP
 /CRITERIA=PIN(.05) POUT(.10)
 /NOORIGIN
 /DEPENDENT Conspiracy_Tot
 /METHOD=ENTER Ideology0
 /METHOD=ENTER GenderCC RaceCC SES0
 /METHOD=ENTER MRN0
 /METHOD=ENTER MRN0xRace MRN0xSES0 MRN0xGender MRN0xIdeology0.
```

Regression

Notes

Output Created		15-DEC-2021 13:09:40
Comments		
Input	Data	C:\Users\njs5478\Dropbox\HM and COVID\0. Revise and Resubmit\2. R and R Data\Study 2a\Study2a_Data.sav
	Active Dataset	DataSet1
	Filter	<none>
	Weight	<none>
	Split File	<none>
	N of Rows in Working Data File	188
Missing Value Handling	Definition of Missing	User-defined missing values are treated as missing.
	Cases Used	Statistics are based on cases with no missing values for any variable used.
Syntax		REGRESSION<br>/MISSING LISTWISE<br>/STATISTICS COEFF OUTS R ANOVA CHANGE ZPP<br>/CRITERIA=PIN(.05) POUT(.10)<br>/NOORIGIN<br>/DEPENDENT Conspiracy_Tot<br>/METHOD=ENTER Ideology0<br>/METHOD=ENTER GenderCC RaceCC SES0<br>/METHOD=ENTER MRN0<br>/METHOD=ENTER MRN0xRace MRN0xSES0 MRN0xGender MRN0xIdeology0.
Resources	Processor Time	00:00:00.02
	Elapsed Time	00:00:00.02

Notes

	Memory Required	33072 bytes
	Additional Memory Required for Residual Plots	0 bytes

Variables Entered/Removed[a]

Model	Variables Entered	Variables Removed	Method
1	Ideology0[b]	.	Enter
2	SES0, GenderCC, RaceCC[b]	.	Enter
3	MRN0[b]	.	Enter
4	MRN0xGender, MRN0xSES0, MRN0xIdeology0, MRN0xRace[b]	.	Enter

a. Dependent Variable: Conspiracy_Tot

b. All requested variables entered.

Model Summary

Model	R	R Square	Adjusted R Square	Std. Error of the Estimate	Change Statistics		
					R Square Change	F Change	df1
1	.358[a]	.128	.124	.59259	.128	27.408	1
2	.384[b]	.148	.129	.59080	.019	1.377	3
3	.432[c]	.187	.164	.57868	.039	8.746	1
4	.485[d]	.235	.197	.56736	.049	2.834	4

Model Summary

Model	Change Statistics	
	df2	Sig. F Change
1	186	.000
2	183	.251
3	182	.004
4	178	.026

a. Predictors: (Constant), Ideology0

b. Predictors: (Constant), Ideology0, SES0, GenderCC, RaceCC

c. Predictors: (Constant), Ideology0, SES0, GenderCC, RaceCC, MRN0

d. Predictors: (Constant), Ideology0, SES0, GenderCC, RaceCC, MRN0, MRN0xGender, MRN0xSES0, MRN0xIdeology0, MRN0xRace

ANOVA[a]

Model		Sum of Squares	df	Mean Square	F	Sig.
1	Regression	9.625	1	9.625	27.408	.000[b]
	Residual	65.317	186	.351		
	Total	74.942	187			
2	Regression	11.066	4	2.767	7.926	.000[c]
	Residual	63.875	183	.349		
	Total	74.942	187			
3	Regression	13.995	5	2.799	8.359	.000[d]
	Residual	60.946	182	.335		
	Total	74.942	187			
4	Regression	17.644	9	1.960	6.090	.000[e]
	Residual	57.298	178	.322		
	Total	74.942	187			

a. Dependent Variable: Conspiracy_Tot

b. Predictors: (Constant), Ideology0

c. Predictors: (Constant), Ideology0, SES0, GenderCC, RaceCC

d. Predictors: (Constant), Ideology0, SES0, GenderCC, RaceCC, MRN0

e. Predictors: (Constant), Ideology0, SES0, GenderCC, RaceCC, MRN0, MRN0xGender, MRN0xSES0, MRN0xIdeology0, MRN0xRace

Coefficients[a]

Model		Unstandardized Coefficients		Standardized Coefficients	t	Sig.
		B	Std. Error	Beta		
1	(Constant)	1.544	.043		35.735	.000
	Ideology0	.150	.029	.358	5.235	.000
2	(Constant)	1.563	.047		32.922	.000
	Ideology0	.149	.030	.357	5.012	.000
	GenderCC	.011	.044	.018	.252	.802
	RaceCC	-.045	.049	-.065	-.916	.361
	SES0	.107	.059	.125	1.808	.072
3	(Constant)	1.565	.046		33.659	.000
	Ideology0	.110	.032	.262	3.407	.001
	GenderCC	-.054	.048	-.085	-1.107	.270
	RaceCC	-.049	.048	-.071	-1.015	.311
	SES0	.082	.059	.096	1.402	.163
	MRN0	.186	.063	.251	2.957	.004
4	(Constant)	1.503	.053		28.518	.000
	Ideology0	.104	.033	.248	3.128	.002
	GenderCC	-.048	.048	-.075	-1.000	.319
	RaceCC	-.048	.049	-.069	-.973	.332
	SES0	.050	.059	.058	.846	.398
	MRN0	.168	.063	.227	2.653	.009
	MRN0xRace	.077	.056	.104	1.365	.174
	MRN0xSES0	-.133	.081	-.113	-1.638	.103
	MRN0xGender	.114	.056	.137	2.049	.042
	MRN0xIdeology0	.049	.036	.098	1.345	.180

Coefficients[a]

Model		Correlations		
		Zero-order	Partial	Part
1	(Constant)			
	Ideology0	.358	.358	.358
2	(Constant)			
	Ideology0	.358	.347	.342
	GenderCC	.077	.019	.017
	RaceCC	.026	-.068	-.062
	SES0	.160	.132	.123
3	(Constant)			
	Ideology0	.358	.245	.228
	GenderCC	.077	-.082	-.074
	RaceCC	.026	-.075	-.068
	SES0	.160	.103	.094
	MRN0	.342	.214	.198
4	(Constant)			
	Ideology0	.358	.228	.205
	GenderCC	.077	-.075	-.066
	RaceCC	.026	-.073	-.064
	SES0	.160	.063	.055
	MRN0	.342	.195	.174
	MRN0xRace	.275	.102	.089
	MRN0xSES0	-.067	-.122	-.107
	MRN0xGender	.121	.152	.134
	MRN0xIdeology0	.148	.100	.088

a. Dependent Variable: Conspiracy_Tot

Excluded Variables[a]

Model		Beta In	t	Sig.	Partial Correlation	Collinearity Statistics Tolerance
1	GenderCC	.034[b]	.497	.620	.036	.986
	RaceCC	-.060[b]	-.854	.394	-.063	.947
	SES0	.121[b]	1.770	.078	.129	.987
	MRN0	.228[b]	3.039	.003	.218	.800
	MRN0xRace	.165[b]	2.267	.025	.164	.864
	MRN0xSES0	-.112[b]	-1.629	.105	-.119	.986
	MRN0xGender	.152[b]	2.229	.027	.162	.993
	MRN0xIdeology0	.100[b]	1.446	.150	.106	.980
2	MRN0	.251[c]	2.957	.004	.214	.621
	MRN0xRace	.151[c]	2.049	.042	.150	.841
	MRN0xSES0	-.090[c]	-1.282	.201	-.095	.943
	MRN0xGender	.147[c]	2.168	.031	.159	.991
	MRN0xIdeology0	.107[c]	1.516	.131	.112	.937
3	MRN0xRace	.118[d]	1.600	.111	.118	.816
	MRN0xSES0	-.079[d]	-1.142	.255	-.085	.940
	MRN0xGender	.141[d]	2.122	.035	.156	.991
	MRN0xIdeology0	.123[d]	1.789	.075	.132	.931

a. Dependent Variable: Conspiracy_Tot

b. Predictors in the Model: (Constant), Ideology0

c. Predictors in the Model: (Constant), Ideology0, SES0, GenderCC, RaceCC

d. Predictors in the Model: (Constant), Ideology0, SES0, GenderCC, RaceCC, MRN0

```
***Simple Slopes***

DESCRIPTIVES VARIABLES=MRN PParty
  /STATISTICS=MEAN STDDEV MIN MAX.
```

Descriptives

Notes

Output Created		15-DEC-2021 13:09:41
Comments		
Input	Data	C:\Users\njs5478\Dropbox\HM and COVID\0. Revise and Resubmit\2. R and R Data\Study 2a\Study2a_Data.sav
	Active Dataset	DataSet1
	Filter	<none>
	Weight	<none>
	Split File	<none>
	N of Rows in Working Data File	188
Missing Value Handling	Definition of Missing	User defined missing values are treated as missing.
	Cases Used	All non-missing data are used.
Syntax		DESCRIPTIVES VARIABLES=MRN PParty /STATISTICS=MEAN STDDEV MIN MAX.
Resources	Processor Time	00:00:00.02
	Elapsed Time	00:00:00.02

Descriptive Statistics

	N	Minimum	Maximum	Mean	Std. Deviation
MRN	188	1.27	5.42	3.2162	.85462
Which of the following best describes your political party affiliation?	188	1	5	2.76	1.220
Valid N (listwise)	188				

```
 COMPUTE MRN.High = MRN0 - .85462.
COMPUTE MRN.Low = MRN0 +.85462.

COMPUTE Party.Rep = Party0 - 1.220.
COMPUTE Party.Dem = Party0 + 1.220.
```

```
**MRN*Party on Psychological Impact

UNIANOVA Psychology_Tot WITH MRN0 RaceCC GenderCC SES0 Party.Rep
  /PRINT=ETASQ PARAMETER
  /DESIGN=MRN0 RaceCC GenderCC SES0 Party.Rep
  MRN0*RaceCC MRN0*GenderCC MRN0*SES0 MRN0*Party.Rep.
```

Univariate Analysis of Variance

Notes

Output Created		15-DEC-2021 13:09:41
Comments		
Input	Data	C:\Users\njs5478\Dropbox\HM and COVID\0. Revise and Resubmit\2. R and R Data\Study 2a\Study2a_Data.sav
	Active Dataset	DataSet1
	Filter	<none>
	Weight	<none>
	Split File	<none>
	N of Rows in Working Data File	188
Missing Value Handling	Definition of Missing	User-defined missing values are treated as missing.
	Cases Used	Statistics are based on all cases with valid data for all variables in the model.
Syntax		UNIANOVA Psychology_Tot WITH MRN0 RaceCC GenderCC SES0 Party.Rep /PRINT=ETASQ PARAMETER /DESIGN=MRN0 RaceCC GenderCC SES0 Party.Rep MRN0*RaceCC MRN0*GenderCC MRN0*SES0 MRN0*Party.Rep.
Resources	Processor Time	00:00:00.00
	Elapsed Time	00:00:00.00

Tests of Between-Subjects Effects

Dependent Variable: Psychology_Tot

Source	Type III Sum of Squares	df	Mean Square	F	Sig.	Partial Eta Squared
Corrected Model	44.390[a]	9	4.932	2.612	.007	.117
Intercept	1000.697	1	1000.697	530.012	.000	.749
MRN0	2.329	1	2.329	1.234	.268	.007
RaceCC	.141	1	.141	.075	.785	.000
GenderCC	.577	1	.577	.306	.581	.002
SES0	.037	1	.037	.020	.889	.000
Party.Rep	5.179	1	5.179	2.743	.099	.015
MRN0 * RaceCC	7.226	1	7.226	3.827	.052	.021
MRN0 * GenderCC	.711	1	.711	.377	.540	.002
MRN0 * SES0	.274	1	.274	.145	.704	.001
MRN0 * Party.Rep	14.275	1	14.275	7.560	.007	.041
Error	336.075	178	1.888			
Total	4256.778	188				
Corrected Total	380.465	187				

a. R Squared = .117 (Adjusted R Squared = .072)

Parameter Estimates

Dependent Variable: Psychology_Tot

Parameter	B	Std. Error	t	Sig.	95% Confidence Interval Lower Bound	95% Confidence Interval Upper Bound
Intercept	4.180	.182	23.022	.000	3.822	4.538
MRN0	.244	.219	1.111	.268	-.189	.676
RaceCC	.032	.118	.273	.785	-.200	.265
GenderCC	-.064	.116	-.553	.581	-.293	.165
SES0	-.020	.144	-.140	.889	-.304	.264
Party.Rep	-.160	.097	-1.656	.099	-.352	.031
MRN0 * RaceCC	-.265	.136	-1.956	.052	-.533	.002
MRN0 * GenderCC	.083	.135	.614	.540	-.183	.349
MRN0 * SES0	.074	.194	.381	.704	-.309	.458
MRN0 * Party.Rep	.312	.113	2.750	.007	.088	.536

Parameter Estimates

Dependent Variable: Psychology_Tot

Parameter	Partial Eta Squared
Intercept	.749
MRN0	.007
RaceCC	.000
GenderCC	.002
SES0	.000
Party.Rep	.015
MRN0 * RaceCC	.021
MRN0 * GenderCC	.002
MRN0 * SES0	.001
MRN0 * Party.Rep	.041

```
UNIANOVA Psychology_Tot WITH MRN0 RaceCC GenderCC SES0 Party.Dem
 /PRINT=ETASQ PARAMETER
 /DESIGN=MRN0 RaceCC GenderCC SES0 Party.Dem
 MRN0*RaceCC MRN0*GenderCC MRN0*SES0 MRN0*Party.Dem.
```

Univariate Analysis of Variance

Notes

Output Created		15-DEC-2021 13:09:41
Comments		
Input	Data	C:\Users\njs5478\Dropbox\HM and COVID\0. Revise and Resubmit\2. R and R Data\Study 2a\Study2a_Data.sav
	Active Dataset	DataSet1
	Filter	<none>
	Weight	<none>
	Split File	<none>
	N of Rows in Working Data File	188
Missing Value Handling	Definition of Missing	User-defined missing values are treated as missing.
	Cases Used	Statistics are based on all cases with valid data for all variables in the model.
Syntax		UNIANOVA Psychology_Tot WITH MRN0 RaceCC GenderCC SES0 Party.Dem /PRINT=ETASQ PARAMETER /DESIGN=MRN0 RaceCC GenderCC SES0 Party.Dem MRN0*RaceCC MRN0*GenderCC MRN0*SES0 MRN0*Party.Dem.
Resources	Processor Time	00:00:00.02
	Elapsed Time	00:00:00.03

Tests of Between-Subjects Effects

Dependent Variable: Psychology_Tot

Source	Type III Sum of Squares	df	Mean Square	F	Sig.	Partial Eta Squared
Corrected Model	44.390[a]	9	4.932	2.612	.007	.117
Intercept	1443.585	1	1443.585	764.585	.000	.811
MRN0	14.052	1	14.052	7.443	.007	.040
RaceCC	.141	1	.141	.075	.785	.000
GenderCC	.577	1	.577	.306	.581	.002
SES0	.037	1	.037	.020	.889	.000
Party.Dem	5.179	1	5.179	2.743	.099	.015
MRN0 * RaceCC	7.226	1	7.226	3.827	.052	.021
MRN0 * GenderCC	.711	1	.711	.377	.540	.002
MRN0 * SES0	.274	1	.274	.145	.704	.001
MRN0 * Party.Dem	14.275	1	14.275	7.560	.007	.041
Error	336.075	178	1.888			
Total	4256.778	188				
Corrected Total	380.465	187				

a. R Squared = .117 (Adjusted R Squared = .072)

Parameter Estimates

Dependent Variable: Psychology_Tot

Parameter	B	Std. Error	t	Sig.	95% Confidence Interval Lower Bound	95% Confidence Interval Upper Bound
Intercept	4.572	.165	27.651	.000	4.245	4.898
MRN0	-.518	.190	-2.728	.007	-.892	-.143
RaceCC	.032	.118	.273	.785	-.200	.265
GenderCC	-.064	.116	-.553	.581	-.293	.165
SES0	-.020	.144	-.140	.889	-.304	.264
Party.Dem	-.160	.097	-1.656	.099	-.352	.031
MRN0 * RaceCC	-.265	.136	-1.956	.052	-.533	.002
MRN0 * GenderCC	.083	.135	.614	.540	-.183	.349
MRN0 * SES0	.074	.194	.381	.704	-.309	.458
MRN0 * Party.Dem	.312	.113	2.750	.007	.088	.536

Parameter Estimates

Dependent Variable: Psychology_Tot

Parameter	Partial Eta Squared
Intercept	.811
MRN0	.040
RaceCC	.000
GenderCC	.002
SES0	.000
Party.Dem	.015
MRN0 * RaceCC	.021
MRN0 * GenderCC	.002
MRN0 * SES0	.001
MRN0 * Party.Dem	.041

```
UNIANOVA Psychology_Tot WITH MRN.Low RaceCC GenderCC SES0 Party0
 /PRINT=ETASQ PARAMETER
 /DESIGN=MRN.Low RaceCC GenderCC SES0 Party0
 MRN.Low*RaceCC MRN.Low*GenderCC MRN.Low*SES0 MRN.Low*Party0.
```

Univariate Analysis of Variance

Notes

Output Created		15-DEC-2021 13:09:41
Comments		
Input	Data	C:\Users\njs5478\Dropbox\HM and COVID\0. Revise and Resubmit\2. R and R Data\Study 2a\Study2a_Data.sav
	Active Dataset	DataSet1
	Filter	<none>
	Weight	<none>
	Split File	<none>
	N of Rows in Working Data File	188
Missing Value Handling	Definition of Missing	User-defined missing values are treated as missing.
	Cases Used	Statistics are based on all cases with valid data for all variables in the model.
Syntax		UNIANOVA Psychology_Tot WITH MRN.Low RaceCC GenderCC SES0 Party0 /PRINT=ETASQ PARAMETER /DESIGN=MRN.Low RaceCC GenderCC SES0 Party0 MRN.Low*RaceCC MRN.Low*GenderCC MRN.Low*SES0 MRN.Low*Party0.
Resources	Processor Time	00:00:00.00
	Elapsed Time	00:00:00.02

Tests of Between-Subjects Effects

Dependent Variable: Psychology_Tot

Source	Type III Sum of Squares	df	Mean Square	F	Sig.
Corrected Model	44.390[a]	9	4.932	2.612	.007
Intercept	1175.398	1	1175.398	622.542	.000
MRN.Low	1.550	1	1.550	.821	.366
RaceCC	5.452	1	5.452	2.888	.091
GenderCC	1.306	1	1.306	.692	.407
SES0	.344	1	.344	.182	.670
Party0	16.849	1	16.849	8.924	.003
MRN.Low * RaceCC	7.226	1	7.226	3.827	.052
MRN.Low * GenderCC	.711	1	.711	.377	.540
MRN.Low * SES0	.274	1	.274	.145	.704
MRN.Low * Party0	14.275	1	14.275	7.560	.007
Error	336.075	178	1.888		
Total	4256.778	188			
Corrected Total	380.465	187			

Tests of Between-Subjects Effects

Dependent Variable: Psychology_Tot

Source	Partial Eta Squared
Corrected Model	.117
Intercept	.778
MRN.Low	.005
RaceCC	.016
GenderCC	.004
SES0	.001
Party0	.048
MRN.Low * RaceCC	.021
MRN.Low * GenderCC	.002
MRN.Low * SES0	.001
MRN.Low * Party0	.041
Error	
Total	
Corrected Total	

a. R Squared = .117 (Adjusted R Squared = .072)

Parameter Estimates

Dependent Variable: Psychology_Tot

Parameter	B	Std. Error	t	Sig.	95% Confidence Interval	
					Lower Bound	Upper Bound
Intercept	4.493	.180	24.951	.000	4.138	4.848
MRN.Low	-.137	.151	-.906	.366	-.436	.161
RaceCC	.259	.152	1.699	.091	-.042	.559
GenderCC	-.135	.162	-.832	.407	-.455	.185
SES0	-.083	.196	-.427	.670	-.470	.303
Party0	-.427	.143	-2.987	.003	-.709	-.145
MRN.Low * RaceCC	-.265	.136	-1.956	.052	-.533	.002
MRN.Low * GenderCC	.083	.135	.614	.540	-.183	.349
MRN.Low * SES0	.074	.194	.381	.704	-.309	.458
MRN.Low * Party0	.312	.113	2.750	.007	.088	.536

Parameter Estimates

Dependent Variable: Psychology_Tot

Parameter	Partial Eta Squared
Intercept	.778
MRN.Low	.005
RaceCC	.016
GenderCC	.004
SES0	.001
Party0	.048
MRN.Low * RaceCC	.021
MRN.Low * GenderCC	.002
MRN.Low * SES0	.001
MRN.Low * Party0	.041

```
UNIANOVA Psychology_Tot WITH MRN.High RaceCC GenderCC SES0 Party0
 /PRINT=ETASQ PARAMETER
 /DESIGN=MRN.High RaceCC GenderCC SES0 Party0
 MRN.High*RaceCC MRN.High*GenderCC MRN.High*SES0 MRN.High*Party0.
```

Univariate Analysis of Variance

Notes

Output Created		15-DEC-2021 13:09:41
Comments		
Input	Data	C:\Users\njs5478\Dropbox\HM and COVID\0. Revise and Resubmit\2. R and R Data\Study 2a\Study2a_Data.sav
	Active Dataset	DataSet1
	Filter	<none>
	Weight	<none>
	Split File	<none>
	N of Rows in Working Data File	188
Missing Value Handling	Definition of Missing	User-defined missing values are treated as missing.
	Cases Used	Statistics are based on all cases with valid data for all variables in the model.
Syntax		UNIANOVA Psychology_Tot WITH MRN.High RaceCC GenderCC SES0 Party0 /PRINT=ETASQ PARAMETER /DESIGN=MRN.High RaceCC GenderCC SES0 Party0 MRN.High*RaceCC MRN.High*GenderCC MRN.High*SES0 MRN.High*Party0.
Resources	Processor Time	00:00:00.02
	Elapsed Time	00:00:00.02

Tests of Between-Subjects Effects

Dependent Variable: Psychology_Tot

Source	Type III Sum of Squares	df	Mean Square	F	Sig.
Corrected Model	44.390[a]	9	4.932	2.612	.007
Intercept	1026.465	1	1026.465	543.660	.000
MRN.High	1.550	1	1.550	.821	.366
RaceCC	2.276	1	2.276	1.205	.274
GenderCC	.003	1	.003	.002	.968
SES0	.060	1	.060	.032	.859
Party0	1.241	1	1.241	.657	.419
MRN.High * RaceCC	7.226	1	7.226	3.827	.052
MRN.High * GenderCC	.711	1	.711	.377	.540
MRN.High * SES0	.274	1	.274	.145	.704
MRN.High * Party0	14.275	1	14.275	7.560	.007
Error	336.075	178	1.888		
Total	4256.778	188			
Corrected Total	380.465	187			

Tests of Between-Subjects Effects

Dependent Variable: Psychology_Tot

Source	Partial Eta Squared
Corrected Model	.117
Intercept	.753
MRN.High	.005
RaceCC	.007
GenderCC	.000
SES0	.000
Party0	.004
MRN.High * RaceCC	.021
MRN.High * GenderCC	.002
MRN.High * SES0	.001
MRN.High * Party0	.041
Error	
Total	
Corrected Total	

a. R Squared = .117 (Adjusted R Squared = .072)

Parameter Estimates

Dependent Variable: Psychology_Tot

Parameter	B	Std. Error	t	Sig.	95% Confidence Interval Lower Bound	95% Confidence Interval Upper Bound
Intercept	4.259	.183	23.317	.000	3.898	4.619
MRN.High	-.137	.151	-.906	.366	-.436	.161
RaceCC	-.195	.177	-1.098	.274	-.544	.155
GenderCC	.007	.165	.041	.968	-.318	.332
SES0	.043	.242	.178	.859	-.434	.520
Party0	.106	.131	.811	.419	-.152	.365
MRN.High * RaceCC	-.265	.136	-1.956	.052	-.533	.002
MRN.High * GenderCC	.083	.135	.614	.540	-.183	.349
MRN.High * SES0	.074	.194	.381	.704	-.309	.458
MRN.High * Party0	.312	.113	2.750	.007	.088	.536

Parameter Estimates

Dependent Variable: Psychology_Tot

Parameter	Partial Eta Squared
Intercept	.753
MRN.High	.005
RaceCC	.007
GenderCC	.000
SES0	.000
Party0	.004
MRN.High * RaceCC	.021
MRN.High * GenderCC	.002
MRN.High * SES0	.001
MRN.High * Party0	.041

```
**MRN*Party on COVID-19 Conspiracy Theories

UNIANOVA Conspiracy_Tot WITH MRN0 RaceCC GenderCC SES0 Party.Rep
  /PRINT=ETASQ PARAMETER
  /DESIGN=MRN0 RaceCC GenderCC SES0 Party.Rep
  MRN0*RaceCC MRN0*GenderCC MRN0*SES0 MRN0*Party.Rep.
```

Univariate Analysis of Variance

Notes

Output Created		15-DEC-2021 13:09:41
Comments		
Input	Data	C:\Users\njs5478\Dropbox\HM and COVID\0. Revise and Resubmit\2. R and R Data\Study 2a\Study2a_Data.sav
	Active Dataset	DataSet1
	Filter	<none>
	Weight	<none>
	Split File	<none>
	N of Rows in Working Data File	188
Missing Value Handling	Definition of Missing	User-defined missing values are treated as missing.
	Cases Used	Statistics are based on all cases with valid data for all variables in the model.
Syntax		UNIANOVA Conspiracy_Tot WITH MRN0 RaceCC GenderCC SES0 Party.Rep<br>/PRINT=ETASQ PARAMETER<br>/DESIGN=MRN0 RaceCC GenderCC SES0 Party.Rep<br>MRN0*RaceCC MRN0*GenderCC MRN0*SES0 MRN0*Party.Rep.
Resources	Processor Time	00:00:00.02
	Elapsed Time	00:00:00.02

Tests of Between-Subjects Effects

Dependent Variable: Conspiracy_Tot

Source	Type III Sum of Squares	df	Mean Square	F	Sig.	Partial Eta Squared
Corrected Model	20.431[a]	9	2.270	7.413	.000	.273
Intercept	154.825	1	154.825	505.565	.000	.740
MRN0	4.893	1	4.893	15.976	.000	.082
RaceCC	.519	1	.519	1.695	.195	.009
GenderCC	.156	1	.156	.511	.476	.003
SES0	.102	1	.102	.333	.565	.002
Party.Rep	3.284	1	3.284	10.724	.001	.057
MRN0 * RaceCC	.344	1	.344	1.124	.290	.006
MRN0 * GenderCC	1.128	1	1.128	3.685	.057	.020
MRN0 * SES0	.888	1	.888	2.899	.090	.016
MRN0 * Party.Rep	2.738	1	2.738	8.939	.003	.048
Error	54.511	178	.306			
Total	523.654	188				
Corrected Total	74.942	187				

a. R Squared = .273 (Adjusted R Squared = .236)

Parameter Estimates

Dependent Variable: Conspiracy_Tot

Parameter	B	Std. Error	t	Sig.	95% Confidence Interval	
					Lower Bound	Upper Bound
Intercept	1.644	.073	22.485	.000	1.500	1.788
MRN0	.353	.088	3.997	.000	.179	.527
RaceCC	-.062	.047	-1.302	.195	-.155	.032
GenderCC	-.033	.047	-.715	.476	-.125	.059
SES0	.033	.058	.577	.565	-.081	.148
Party.Rep	.128	.039	3.275	.001	.051	.205
MRN0 * RaceCC	.058	.055	1.060	.290	-.050	.166
MRN0 * GenderCC	.104	.054	1.920	.057	-.003	.211
MRN0 * SES0	-.133	.078	-1.703	.090	-.288	.021
MRN0 * Party.Rep	.137	.046	2.990	.003	.046	.227

Parameter Estimates

Dependent Variable: Conspiracy_Tot

Parameter	Partial Eta Squared
Intercept	.740
MRN0	.082
RaceCC	.009
GenderCC	.003
SES0	.002
Party.Rep	.057
MRN0 * RaceCC	.006
MRN0 * GenderCC	.020
MRN0 * SES0	.016
MRN0 * Party.Rep	.048

```
UNIANOVA Conspiracy_Tot WITH MRN0 RaceCC GenderCC SES0 Party.Dem
 /PRINT=ETASQ PARAMETER
 /DESIGN=MRN0 RaceCC GenderCC SES0 Party.Dem
 MRN0*RaceCC MRN0*GenderCC MRN0*SES0 MRN0*Party.Dem.
```

Univariate Analysis of Variance

Notes

Output Created		15-DEC-2021 13:09:41
Comments		
Input	Data	C:\Users\njs5478\Dropbox\HM and COVID\0. Revise and Resubmit\2. R and R Data\Study 2a\Study2a_Data.sav
	Active Dataset	DataSet1
	Filter	<none>
	Weight	<none>
	Split File	<none>
	N of Rows in Working Data File	188
Missing Value Handling	Definition of Missing	User-defined missing values are treated as missing.
	Cases Used	Statistics are based on all cases with valid data for all variables in the model.
Syntax		UNIANOVA Conspiracy_Tot WITH MRN0 RaceCC GenderCC SES0 Party.Dem<br>/PRINT=ETASQ PARAMETER<br>/DESIGN=MRN0 RaceCC GenderCC SES0 Party.Dem<br>MRN0*RaceCC MRN0*GenderCC MRN0*SES0 MRN0*Party.Dem.
Resources	Processor Time	00:00:00.02
	Elapsed Time	00:00:00.02

Tests of Between-Subjects Effects

Dependent Variable: Conspiracy_Tot

Source	Type III Sum of Squares	df	Mean Square	F	Sig.	Partial Eta Squared
Corrected Model	20.431[a]	9	2.270	7.413	.000	.273
Intercept	122.612	1	122.612	400.375	.000	.692
MRN0	.020	1	.020	.066	.797	.000
RaceCC	.519	1	.519	1.695	.195	.009
GenderCC	.156	1	.156	.511	.476	.003
SES0	.102	1	.102	.333	.565	.002
Party.Dem	3.284	1	3.284	10.724	.001	.057
MRN0 * RaceCC	.344	1	.344	1.124	.290	.006
MRN0 * GenderCC	1.128	1	1.128	3.685	.057	.020
MRN0 * SES0	.888	1	.888	2.899	.090	.016
MRN0 * Party.Dem	2.738	1	2.738	8.939	.003	.048
Error	54.511	178	.306			
Total	523.654	188				
Corrected Total	74.942	187				

a. R Squared = .273 (Adjusted R Squared = .236)

Parameter Estimates

Dependent Variable: Conspiracy_Tot

Parameter	B	Std. Error	t	Sig.	95% Confidence Interval	
					Lower Bound	Upper Bound
Intercept	1.332	.067	20.009	.000	1.201	1.464
MRN0	.020	.076	.257	.797	-.131	.170
RaceCC	-.062	.047	-1.302	.195	-.155	.032
GenderCC	-.033	.047	-.715	.476	-.125	.059
SES0	.033	.058	.577	.565	-.081	.148
Party.Dem	.128	.039	3.275	.001	.051	.205
MRN0 * RaceCC	.058	.055	1.060	.290	-.050	.166
MRN0 * GenderCC	.104	.054	1.920	.057	-.003	.211
MRN0 * SES0	-.133	.078	-1.703	.090	-.288	.021
MRN0 * Party.Dem	.137	.046	2.990	.003	.046	.227

Parameter Estimates

Dependent Variable: Conspiracy_Tot

Parameter	Partial Eta Squared
Intercept	.692
MRN0	.000
RaceCC	.009
GenderCC	.003
SES0	.002
Party.Dem	.057
MRN0 * RaceCC	.006
MRN0 * GenderCC	.020
MRN0 * SES0	.016
MRN0 * Party.Dem	.048

```
UNIANOVA Conspiracy_Tot WITH MRN.Low RaceCC GenderCC SES0 Party0
 /PRINT=ETASQ PARAMETER
 /DESIGN=MRN.Low RaceCC GenderCC SES0 Party0
 MRN.Low*RaceCC MRN.Low*GenderCC MRN.Low*SES0 MRN.Low*Party0.
```

Univariate Analysis of Variance

Notes

Output Created		15-DEC-2021 13:09:41
Comments		
Input	Data	C: \Users\njs5478\Dropbox\HM and COVID\0. Revise and Resubmit\2. R and R Data\Study 2a\Study2a_Data.sav
	Active Dataset	DataSet1
	Filter	<none>
	Weight	<none>
	Split File	<none>
	N of Rows in Working Data File	188
Missing Value Handling	Definition of Missing	User-defined missing values are treated as missing.
	Cases Used	Statistics are based on all cases with valid data for all variables in the model.
Syntax		UNIANOVA Conspiracy_Tot WITH MRN.Low RaceCC GenderCC SES0 Party0<br>/PRINT=ETASQ PARAMETER<br>/DESIGN=MRN.Low RaceCC GenderCC SES0 Party0<br>MRN.Low*RaceCC MRN.Low*GenderCC MRN.Low*SES0 MRN.Low*Party0.
Resources	Processor Time	00:00:00.02
	Elapsed Time	00:00:00.03

Tests of Between-Subjects Effects

Dependent Variable: Conspiracy_Tot

Source	Type III Sum of Squares	df	Mean Square	F	Sig.
Corrected Model	20.431[a]	9	2.270	7.413	.000
Intercept	102.843	1	102.843	335.823	.000
MRN.Low	2.864	1	2.864	9.352	.003
RaceCC	1.007	1	1.007	3.288	.071
GenderCC	1.077	1	1.077	3.518	.062
SES0	1.071	1	1.071	3.496	.063
Party0	.011	1	.011	.037	.848
MRN.Low * RaceCC	.344	1	.344	1.124	.290
MRN.Low * GenderCC	1.128	1	1.128	3.685	.057
MRN.Low * SES0	.888	1	.888	2.899	.090
MRN.Low * Party0	2.738	1	2.738	8.939	.003
Error	54.511	178	.306		
Total	523.654	188			
Corrected Total	74.942	187			

Tests of Between-Subjects Effects

Dependent Variable: Conspiracy_Tot

Source	Partial Eta Squared
Corrected Model	.273
Intercept	.654
MRN.Low	.050
RaceCC	.018
GenderCC	.019
SES0	.019
Party0	.000
MRN.Low * RaceCC	.006
MRN.Low * GenderCC	.020
MRN.Low * SES0	.016
MRN.Low * Party0	.048
Error	
Total	
Corrected Total	

a. R Squared = .273 (Adjusted R Squared = .236)

Parameter Estimates

Dependent Variable: Conspiracy_Tot

Parameter	B	Std. Error	t	Sig.	95% Confidence Interval Lower Bound	95% Confidence Interval Upper Bound
Intercept	1.329	.073	18.325	.000	1.186	1.472
MRN.Low	.186	.061	3.058	.003	.066	.307
RaceCC	-.111	.061	-1.813	.071	-.232	.010
GenderCC	-.122	.065	-1.876	.062	-.251	.006
SES0	.147	.079	1.870	.063	-.008	.303
Party0	.011	.058	.192	.848	-.103	.125
MRN.Low * RaceCC	.058	.055	1.060	.290	-.050	.166
MRN.Low * GenderCC	.104	.054	1.920	.057	-.003	.211
MRN.Low * SES0	-.133	.078	-1.703	.090	-.288	.021
MRN.Low * Party0	.137	.046	2.990	.003	.046	.227

Parameter Estimates

Dependent Variable: Conspiracy_Tot

Parameter	Partial Eta Squared
Intercept	.654
MRN.Low	.050
RaceCC	.018
GenderCC	.019
SES0	.019
Party0	.000
MRN.Low * RaceCC	.006
MRN.Low * GenderCC	.020
MRN.Low * SES0	.016
MRN.Low * Party0	.048

```
UNIANOVA Conspiracy_Tot WITH MRN.High RaceCC GenderCC SES0 Party0
 /PRINT=ETASQ PARAMETER
 /DESIGN=MRN.High RaceCC GenderCC SES0 Party0
 MRN.High*RaceCC MRN.High*GenderCC MRN.High*SES0 MRN.High*Party0.
```

Univariate Analysis of Variance

Notes

Output Created		15-DEC-2021 13:09:41
Comments		
Input	Data	C:\Users\njs5478\Dropbox\HM and COVID\0. Revise and Resubmit\2. R and R Data\Study 2a\Study2a_Data.sav
	Active Dataset	DataSet1
	Filter	<none>
	Weight	<none>
	Split File	<none>
	N of Rows in Working Data File	188
Missing Value Handling	Definition of Missing	User-defined missing values are treated as missing.
	Cases Used	Statistics are based on all cases with valid data for all variables in the model.
Syntax		UNIANOVA Conspiracy_Tot WITH MRN.High RaceCC GenderCC SES0 Party0 /PRINT=ETASQ PARAMETER /DESIGN=MRN.High RaceCC GenderCC SES0 Party0 MRN.High*RaceCC MRN.High*GenderCC MRN.High*SES0 MRN.High*Party0.
Resources	Processor Time	00:00:00.02
	Elapsed Time	00:00:00.02

Tests of Between-Subjects Effects

Dependent Variable: Conspiracy_Tot

Source	Type III Sum of Squares	df	Mean Square	F	Sig.
Corrected Model	20.431[a]	9	2.270	7.413	.000
Intercept	153.620	1	153.620	501.630	.000
MRN.High	2.864	1	2.864	9.352	.003
RaceCC	.009	1	.009	.030	.864
GenderCC	.217	1	.217	.707	.402
SES0	.209	1	.209	.684	.409
Party0	6.588	1	6.588	21.512	.000
MRN.High * RaceCC	.344	1	.344	1.124	.290
MRN.High * GenderCC	1.128	1	1.128	3.685	.057
MRN.High * SES0	.888	1	.888	2.899	.090
MRN.High * Party0	2.738	1	2.738	8.939	.003
Error	54.511	178	.306		
Total	523.654	188			
Corrected Total	74.942	187			

Tests of Between-Subjects Effects

Dependent Variable: Conspiracy_Tot

Source	Partial Eta Squared
Corrected Model	.273
Intercept	.738
MRN.High	.050
RaceCC	.000
GenderCC	.004
SES0	.004
Party0	.108
MRN.High * RaceCC	.006
MRN.High * GenderCC	.020
MRN.High * SES0	.016
MRN.High * Party0	.048
Error	
Total	
Corrected Total	

a. R Squared = .273 (Adjusted R Squared = .236)

Parameter Estimates

Dependent Variable: Conspiracy_Tot

Parameter	B	Std. Error	t	Sig.	95% Confidence Interval	
					Lower Bound	Upper Bound
Intercept	1.648	.074	22.397	.000	1.502	1.793
MRN.High	.186	.061	3.058	.003	.066	.307
RaceCC	-.012	.071	-.172	.864	-.153	.129
GenderCC	.056	.066	.841	.402	-.075	.187
SES0	-.080	.097	-.827	.409	-.272	.112
Party0	.245	.053	4.638	.000	.141	.349
MRN.High * RaceCC	.058	.055	1.060	.290	-.050	.166
MRN.High * GenderCC	.104	.054	1.920	.057	-.003	.211
MRN.High * SES0	-.133	.078	-1.703	.090	-.288	.021
MRN.High * Party0	.137	.046	2.990	.003	.046	.227

Parameter Estimates

Dependent Variable: Conspiracy_Tot

Parameter	Partial Eta Squared
Intercept	.738
MRN.High	.050
RaceCC	.000
GenderCC	.004
SES0	.004
Party0	.108
MRN.High * RaceCC	.006
MRN.High * GenderCC	.020
MRN.High * SES0	.016
MRN.High * Party0	.048

```
 **Regression Analyses WITH Nationalism**

**With PParty

REGRESSION
  /MISSING LISTWISE
  /STATISTICS COEFF OUTS R ANOVA CHANGE ZPP
  /CRITERIA=PIN(.05) POUT(.10)
```

```
/NOORIGIN
/DEPENDENT Concern_Tot
/METHOD=ENTER Party0
/METHOD=ENTER GenderCC RaceCC SES0
 /METHOD=ENTER National0
/METHOD=ENTER MRN0
/METHOD=ENTER MRN0xRace MRN0xSES0 MRN0xGender MRN0xParty0 MRN0xNational0.
```

Regression

Notes

Output Created		15-DEC-2021 13:09:41
Comments		
Input	Data	C:\Users\njs5478\Dropbox\HM and COVID\0. Revise and Resubmit\2. R and R Data\Study 2a\Study2a_Data.sav
	Active Dataset	DataSet1
	Filter	<none>
	Weight	<none>
	Split File	<none>
	N of Rows in Working Data File	188
Missing Value Handling	Definition of Missing	User-defined missing values are treated as missing.
	Cases Used	Statistics are based on cases with no missing values for any variable used.

Notes

Syntax		REGRESSION<br>/MISSING LISTWISE<br>/STATISTICS COEFF OUTS R ANOVA CHANGE ZPP<br>/CRITERIA=PIN(.05) POUT(.10)<br>/NOORIGIN<br>/DEPENDENT Concern_Tot<br>/METHOD=ENTER Party0<br>/METHOD=ENTER GenderCC RaceCC SES0<br>/METHOD=ENTER National0<br>/METHOD=ENTER MRN0<br>/METHOD=ENTER MRN0xRace MRN0xSES0 MRN0xGender MRN0xParty0 MRN0xNational0.
Resources	Processor Time	00:00:00.02
	Elapsed Time	00:00:00.02
	Memory Required	35200 bytes
	Additional Memory Required for Residual Plots	0 bytes

Variables Entered/Removed[a]

Model	Variables Entered	Variables Removed	Method
1	Party0[b]	.	Enter
2	GenderCC, SES0, RaceCC[b]	.	Enter
3	National0[b]	.	Enter
4	MRN0[b]	.	Enter
5	MRN0xGender, MRN0xSES0, MRN0xNational0, MRN0xParty0, MRN0xRace[b]	.	Enter

a. Dependent Variable: Concern_Tot

b. All requested variables entered.

Model Summary

Model	R	R Square	Adjusted R Square	Std. Error of the Estimate	Change Statistics		
					R Square Change	F Change	df1
1	.493[a]	.243	.239	1.18922	.243	59.660	1
2	.577[b]	.333	.318	1.12526	.090	8.248	3
3	.590[c]	.348	.330	1.11536	.015	4.265	1
4	.598[d]	.357	.336	1.11073	.009	2.519	1
5	.612[e]	.374	.335	1.11122	.017	.968	5

Model Summary

Model	Change Statistics	
	df2	Sig. F Change
1	186	.000
2	183	.000
3	182	.040
4	181	.114
5	176	.439

a. Predictors: (Constant), Party0

b. Predictors: (Constant), Party0, GenderCC, SES0, RaceCC

c. Predictors: (Constant), Party0, GenderCC, SES0, RaceCC, National0

d. Predictors: (Constant), Party0, GenderCC, SES0, RaceCC, National0, MRN0

e. Predictors: (Constant), Party0, GenderCC, SES0, RaceCC, National0, MRN0, MRN0xGender, MRN0xSES0, MRN0xNational0, MRN0xParty0, MRN0xRace

ANOVA[a]

Model		Sum of Squares	df	Mean Square	F	Sig.
1	Regression	84.373	1	84.373	59.660	.000[b]
	Residual	263.048	186	1.414		
	Total	347.421	187			
2	Regression	115.704	4	28.926	22.845	.000[c]
	Residual	231.717	183	1.266		
	Total	347.421	187			
3	Regression	121.010	5	24.202	19.455	.000[d]
	Residual	226.411	182	1.244		
	Total	347.421	187			
4	Regression	124.118	6	20.686	16.767	.000[e]
	Residual	223.303	181	1.234		
	Total	347.421	187			
5	Regression	130.096	11	11.827	9.578	.000[f]
	Residual	217.326	176	1.235		
	Total	347.421	187			

a. Dependent Variable: Concern_Tot

b. Predictors: (Constant), Party0

c. Predictors: (Constant), Party0, GenderCC, SES0, RaceCC

d. Predictors: (Constant), Party0, GenderCC, SES0, RaceCC, National0

e. Predictors: (Constant), Party0, GenderCC, SES0, RaceCC, National0, MRN0

f. Predictors: (Constant), Party0, GenderCC, SES0, RaceCC, National0, MRN0, MRN0xGender, MRN0xSES0, MRN0xNational0, MRN0xParty0, MRN0xRace

Coefficients[a]

Model		Unstandardized Coefficients B	Unstandardized Coefficients Std. Error	Standardized Coefficients Beta	t	Sig.
1	(Constant)	3.629	.087		41.840	.000
	Party0	-.551	.071	-.493	-7.724	.000
2	(Constant)	3.704	.091		40.891	.000
	Party0	-.498	.072	-.446	-6.954	.000
	GenderCC	-.401	.083	-.295	-4.818	.000
	RaceCC	-.185	.095	-.124	-1.949	.053
	SES0	-2.312E-5	.115	.000	.000	1.000
3	(Constant)	3.675	.091		40.443	.000
	Party0	-.461	.073	-.413	-6.297	.000
	GenderCC	-.403	.083	-.297	-4.884	.000
	RaceCC	-.115	.100	-.077	-1.146	.253
	SES0	.012	.114	.007	.108	.914
	National0	-.126	.061	-.140	-2.065	.040
4	(Constant)	3.676	.090		40.626	.000
	Party0	-.421	.077	-.377	-5.446	.000
	GenderCC	-.332	.094	-.244	-3.533	.001
	RaceCC	-.120	.100	-.081	-1.206	.229
	SES0	.031	.114	.016	.268	.789
	National0	-.109	.061	-.122	-1.775	.078
	MRN0	-.190	.120	-.119	-1.587	.114
5	(Constant)	3.668	.104		35.270	.000
	Party0	-.396	.080	-.354	-4.971	.000
	GenderCC	-.331	.094	-.244	-3.517	.001
	RaceCC	-.138	.101	-.093	-1.366	.174
	SES0	.039	.117	.021	.332	.740
	National0	-.121	.062	-.135	-1.937	.054
	MRN0	-.106	.128	-.066	-.828	.409
	MRN0xRace	-.251	.115	-.157	-2.184	.030
	MRN0xSES0	.053	.158	.021	.335	.738
	MRN0xGender	-.011	.110	-.006	-.105	.917
	MRN0xParty0	.041	.097	.029	.420	.675
	MRN0xNational0	.034	.067	.036	.508	.612

Coefficients[a]

Model		Correlations Zero-order	Partial	Part
1	(Constant)			
	Party0	-.493	-.493	-.493
2	(Constant)			
	Party0	-.493	-.457	-.420
	GenderCC	-.295	-.335	-.291
	RaceCC	-.209	-.143	-.118
	SES0	-.116	.000	.000
3	(Constant)			
	Party0	-.493	-.423	-.377
	GenderCC	-.295	-.340	-.292
	RaceCC	-.209	-.085	-.069
	SES0	-.116	.008	.006
	National0	-.298	-.151	-.124
4	(Constant)			
	Party0	-.493	-.375	-.325
	GenderCC	-.295	-.254	-.211
	RaceCC	-.209	-.089	-.072
	SES0	-.116	.020	.016
	National0	-.298	-.131	-.106
	MRN0	-.405	-.117	-.095
5	(Constant)			
	Party0	-.493	-.351	-.296
	GenderCC	-.295	-.256	-.210
	RaceCC	-.209	-.102	-.081
	SES0	-.116	.025	.020
	National0	-.298	-.144	-.115
	MRN0	-.405	-.062	-.049
	MRN0xRace	-.288	-.162	-.130
	MRN0xSES0	-.016	.025	.020
	MRN0xGender	.063	-.008	-.006
	MRN0xParty0	-.054	.032	.025
	MRN0xNational0	.027	.038	.030

a. Dependent Variable: Concern_Tot

Excluded Variables[a]

Model		Beta In	t	Sig.	Partial Correlation	Collinearity Statistics Tolerance
1	GenderCC	-.276[b]	-4.554	.000	-.317	.998
	RaceCC	-.076[b]	-1.143	.254	-.084	.920
	SES0	-.020[b]	-.306	.760	-.022	.962
	National0	-.147[b]	-2.195	.029	-.159	.884
	MRN0	-.254[b]	-3.814	.000	-.270	.854
	MRN0xRace	-.163[b]	-2.491	.014	-.180	.922
	MRN0xSES0	.004[b]	.067	.947	.005	.998
	MRN0xGender	.016[b]	.242	.809	.018	.991
	MRN0xParty0	.024[b]	.368	.713	.027	.975
	MRN0xNational0	.056[b]	.878	.381	.064	.997
2	National0	-.140[c]	-2.065	.040	-.151	.776
	MRN0	-.142[c]	-1.904	.058	-.140	.650
	MRN0xRace	-.141[c]	-2.226	.027	-.163	.895
	MRN0xSES0	.008[c]	.132	.895	.010	.956
	MRN0xGender	.012[c]	.194	.847	.014	.989
	MRN0xParty0	.007[c]	.117	.907	.009	.958
	MRN0xNational0	.017[c]	.275	.783	.020	.974
3	MRN0	-.119[d]	-1.587	.114	-.117	.631
	MRN0xRace	-.146[d]	-2.331	.021	-.171	.894
	MRN0xSES0	.015[d]	.248	.805	.018	.953
	MRN0xGender	.001[d]	.015	.988	.001	.981
	MRN0xParty0	.005[d]	.074	.941	.005	.957
	MRN0xNational0	.007[d]	.115	.909	.009	.968
4	MRN0xRace	-.130[e]	-2.022	.045	-.149	.848
	MRN0xSES0	.012[e]	.199	.843	.015	.952
	MRN0xGender	.006[e]	.091	.927	.007	.979
	MRN0xParty0	-.002[e]	-.027	.979	-.002	.953
	MRN0xNational0	-.008[e]	-.129	.897	-.010	.945

a. Dependent Variable: Concern_Tot

b. Predictors in the Model: (Constant), Party0

c. Predictors in the Model: (Constant), Party0, GenderCC, SES0, RaceCC

d. Predictors in the Model: (Constant), Party0, GenderCC, SES0, RaceCC, National0

e. Predictors in the Model: (Constant), Party0, GenderCC, SES0, RaceCC, National0, MRN0

```
REGRESSION
 /MISSING LISTWISE
 /STATISTICS COEFF OUTS R ANOVA CHANGE ZPP
 /CRITERIA=PIN(.05) POUT(.10)
 /NOORIGIN
 /DEPENDENT Finance_Tot
 /METHOD=ENTER Party0
 /METHOD=ENTER GenderCC RaceCC SES0
  /METHOD=ENTER National0
 /METHOD=ENTER MRN0
 /METHOD=ENTER MRN0xRace MRN0xSES0 MRN0xGender MRN0xParty0 MRN0xNational0.
```

Regression

Notes

Output Created		15-DEC-2021 13:09:41
Comments		
Input	Data	C: \Users\njs5478\Dropbox\HM and COVID\0. Revise and Resubmit\2. R and R Data\Study 2a\Study2a_Data.sav
	Active Dataset	DataSet1
	Filter	<none>
	Weight	<none>
	Split File	<none>
	N of Rows in Working Data File	188
Missing Value Handling	Definition of Missing	User-defined missing values are treated as missing.
	Cases Used	Statistics are based on cases with no missing values for any variable used.

Notes

Syntax		REGRESSION<br>/MISSING LISTWISE<br>/STATISTICS COEFF OUTS R ANOVA CHANGE ZPP<br>/CRITERIA=PIN(.05) POUT(.10)<br>/NOORIGIN<br>/DEPENDENT Finance_Tot<br>/METHOD=ENTER Party0<br>/METHOD=ENTER GenderCC RaceCC SES0<br>/METHOD=ENTER National0<br>/METHOD=ENTER MRN0<br>/METHOD=ENTER MRN0xRace MRN0xSES0 MRN0xGender MRN0xParty0 MRN0xNational0.
Resources	Processor Time	00:00:00.02
	Elapsed Time	00:00:00.02
	Memory Required	35200 bytes
	Additional Memory Required for Residual Plots	0 bytes

Variables Entered/Removed[a]

Model	Variables Entered	Variables Removed	Method
1	Party0[b]	.	Enter
2	GenderCC, SES0, RaceCC[b]	.	Enter
3	National0[b]	.	Enter
4	MRN0[b]	.	Enter
5	MRN0xGender, MRN0xSES0, MRN0xNational0, MRN0xParty0, MRN0xRace[b]	.	Enter

a. Dependent Variable: Finance_Tot

b. All requested variables entered.

Model Summary

Model	R	R Square	Adjusted R Square	Std. Error of the Estimate	Change Statistics		
					R Square Change	F Change	df1
1	.070[a]	.005	.000	1.46207	.005	.918	1
2	.357[b]	.127	.108	1.38029	.123	8.564	3
3	.361[c]	.130	.106	1.38200	.003	.549	1
4	.367[d]	.135	.106	1.38220	.005	.947	1
5	.375[e]	.141	.087	1.39669	.006	.253	5

Model Summary

Model	Change Statistics	
	df2	Sig. F Change
1	186	.339
2	183	.000
3	182	.460
4	181	.332
5	176	.938

a. Predictors: (Constant), Party0

b. Predictors: (Constant), Party0, GenderCC, SES0, RaceCC

c. Predictors: (Constant), Party0, GenderCC, SES0, RaceCC, National0

d. Predictors: (Constant), Party0, GenderCC, SES0, RaceCC, National0, MRN0

e. Predictors: (Constant), Party0, GenderCC, SES0, RaceCC, National0, MRN0, MRN0xGender, MRN0xSES0, MRN0xNational0, MRN0xParty0, MRN0xRace

ANOVA[a]

Model		Sum of Squares	df	Mean Square	F	Sig.
1	Regression	1.963	1	1.963	.918	.339[b]
	Residual	397.604	186	2.138		
	Total	399.567	187			
2	Regression	50.914	4	12.728	6.681	.000[c]
	Residual	348.654	183	1.905		
	Total	399.567	187			
3	Regression	51.962	5	10.392	5.441	.000[d]
	Residual	347.605	182	1.910		
	Total	399.567	187			
4	Regression	53.771	6	8.962	4.691	.000[e]
	Residual	345.797	181	1.910		
	Total	399.567	187			
5	Regression	56.237	11	5.112	2.621	.004[f]
	Residual	343.331	176	1.951		
	Total	399.567	187			

a. Dependent Variable: Finance_Tot

b. Predictors: (Constant), Party0

c. Predictors: (Constant), Party0, GenderCC, SES0, RaceCC

d. Predictors: (Constant), Party0, GenderCC, SES0, RaceCC, National0

e. Predictors: (Constant), Party0, GenderCC, SES0, RaceCC, National0, MRN0

f. Predictors: (Constant), Party0, GenderCC, SES0, RaceCC, National0, MRN0, MRN0xGender, MRN0xSES0, MRN0xNational0, MRN0xParty0, MRN0xRace

Coefficients[a]

Model		Unstandardized Coefficients B	Unstandardized Coefficients Std. Error	Standardized Coefficients Beta	t	Sig.
1	(Constant)	3.770	.107		35.351	.000
	Party0	-.084	.088	-.070	-.958	.339
2	(Constant)	3.809	.111		34.285	.000
	Party0	.019	.088	.016	.217	.829
	GenderCC	-.152	.102	-.104	-1.486	.139
	RaceCC	-.096	.116	-.060	-.827	.409
	SES0	-.659	.141	-.331	-4.690	.000
3	(Constant)	3.796	.113		33.720	.000
	Party0	.036	.091	.030	.391	.696
	GenderCC	-.153	.102	-.105	-1.493	.137
	RaceCC	-.065	.124	-.041	-.524	.601
	SES0	-.653	.141	-.328	-4.639	.000
	National0	-.056	.075	-.058	-.741	.460
4	(Constant)	3.797	.113		33.723	.000
	Party0	.066	.096	.055	.690	.491
	GenderCC	-.098	.117	-.067	-.839	.403
	RaceCC	-.069	.124	-.043	-.559	.577
	SES0	-.640	.142	-.321	-4.516	.000
	National0	-.043	.077	-.045	-.565	.573
	MRN0	-.145	.149	-.085	-.973	.332
5	(Constant)	3.799	.131		29.064	.000
	Party0	.052	.100	.044	.525	.601
	GenderCC	-.098	.118	-.067	-.827	.409
	RaceCC	-.084	.127	-.053	-.664	.508
	SES0	-.627	.147	-.315	-4.266	.000
	National0	-.051	.078	-.053	-.656	.513
	MRN0	-.127	.160	-.075	-.794	.428
	MRN0xRace	-.024	.145	-.014	-.168	.867
	MRN0xSES0	.063	.198	.023	.319	.750
	MRN0xGender	-.097	.138	-.050	-.703	.483
	MRN0xParty0	.105	.121	.071	.864	.389
	MRN0xNational0	-.017	.084	-.017	-.202	.840

Coefficients[a]

Model		Correlations Zero-order	Partial	Part
1	(Constant)			
	Party0	-.070	-.070	-.070
2	(Constant)			
	Party0	-.070	.016	.015
	GenderCC	-.114	-.109	-.103
	RaceCC	-.071	-.061	-.057
	SES0	-.339	-.328	-.324
3	(Constant)			
	Party0	-.070	.029	.027
	GenderCC	-.114	-.110	-.103
	RaceCC	-.071	-.039	-.036
	SES0	-.339	-.325	-.321
	National0	-.100	-.055	-.051
4	(Constant)			
	Party0	-.070	.051	.048
	GenderCC	-.114	-.062	-.058
	RaceCC	-.071	-.041	-.039
	SES0	-.339	-.318	-.312
	National0	-.100	-.042	-.039
	MRN0	-.167	-.072	-.067
5	(Constant)			
	Party0	-.070	.040	.037
	GenderCC	-.114	-.062	-.058
	RaceCC	-.071	-.050	-.046
	SES0	-.339	-.306	-.298
	National0	-.100	-.049	-.046
	MRN0	-.167	-.060	-.056
	MRN0xRace	-.044	-.013	-.012
	MRN0xSES0	.090	.024	.022
	MRN0xGender	-.038	-.053	-.049
	MRN0xParty0	.050	.065	.060
	MRN0xNational0	.023	-.015	-.014

a. Dependent Variable: Finance_Tot

Excluded Variables[a]

Model		Beta In	t	Sig.	Partial Correlation	Collinearity Statistics Tolerance
1	GenderCC	-.112[b]	-1.532	.127	-.112	.998
	RaceCC	-.056[b]	-.729	.467	-.054	.920
	SES0	-.339[b]	-4.802	.000	-.333	.962
	National0	-.087[b]	-1.113	.267	-.082	.884
	MRN0	-.164[b]	-2.091	.038	-.152	.854
	MRN0xRace	-.027[b]	-.350	.727	-.026	.922
	MRN0xSES0	.093[b]	1.273	.205	.093	.998
	MRN0xGender	-.045[b]	-.609	.544	-.045	.991
	MRN0xParty0	.063[b]	.847	.398	.062	.975
	MRN0xNational0	.028[b]	.377	.707	.028	.997
2	National0	-.058[c]	-.741	.460	-.055	.776
	MRN0	-.093[c]	-1.087	.279	-.080	.650
	MRN0xRace	-.006[c]	-.080	.936	-.006	.895
	MRN0xSES0	.029[c]	.414	.680	.031	.956
	MRN0xGender	-.038[c]	-.541	.589	-.040	.989
	MRN0xParty0	.062[c]	.877	.382	.065	.958
	MRN0xNational0	.023[c]	.331	.741	.025	.974
3	MRN0	-.085[d]	-.973	.332	-.072	.631
	MRN0xRace	-.008[d]	-.109	.914	-.008	.894
	MRN0xSES0	.032[d]	.455	.650	.034	.953
	MRN0xGender	-.042[d]	-.608	.544	-.045	.981
	MRN0xParty0	.061[d]	.860	.391	.064	.957
	MRN0xNational0	.019[d]	.273	.786	.020	.968
4	MRN0xRace	.009[e]	.115	.909	.009	.848
	MRN0xSES0	.030[e]	.424	.672	.032	.952
	MRN0xGender	-.039[e]	-.561	.575	-.042	.979
	MRN0xParty0	.057[e]	.799	.425	.059	.953
	MRN0xNational0	.009[e]	.125	.901	.009	.945

a. Dependent Variable: Finance_Tot

b. Predictors in the Model: (Constant), Party0

c. Predictors in the Model: (Constant), Party0, GenderCC, SES0, RaceCC

d. Predictors in the Model: (Constant), Party0, GenderCC, SES0, RaceCC, National0

e. Predictors in the Model: (Constant), Party0, GenderCC, SES0, RaceCC, National0, MRN0

```
REGRESSION
 /MISSING LISTWISE
 /STATISTICS COEFF OUTS R ANOVA CHANGE ZPP
 /CRITERIA=PIN(.05) POUT(.10)
 /NOORIGIN
 /DEPENDENT Psychology_Tot
 /METHOD=ENTER Party0
 /METHOD=ENTER GenderCC RaceCC SES0
  /METHOD=ENTER National0
 /METHOD=ENTER MRN0
 /METHOD=ENTER MRN0xRace MRN0xSES0 MRN0xGender MRN0xParty0 MRN0xNational0.
```

Regression

Notes

Output Created		15-DEC-2021 13:09:41
Comments		
Input	Data	C: \Users\njs5478\Dropbox\HM and COVID\0. Revise and Resubmit\2. R and R Data\Study 2a\Study2a_Data.sav
	Active Dataset	DataSet1
	Filter	<none>
	Weight	<none>
	Split File	<none>
	N of Rows in Working Data File	188
Missing Value Handling	Definition of Missing	User-defined missing values are treated as missing.
	Cases Used	Statistics are based on cases with no missing values for any variable used.

Notes

Syntax		REGRESSION<br>/MISSING LISTWISE<br>/STATISTICS COEFF OUTS R ANOVA CHANGE ZPP<br>/CRITERIA=PIN(.05) POUT(.10)<br>/NOORIGIN<br>/DEPENDENT Psychology_Tot<br>/METHOD=ENTER Party0<br>/METHOD=ENTER GenderCC RaceCC SES0<br>/METHOD=ENTER National0<br>/METHOD=ENTER MRN0<br>/METHOD=ENTER MRN0xRace MRN0xSES0 MRN0xGender MRN0xParty0 MRN0xNational0.
Resources	Processor Time	00:00:00.02
	Elapsed Time	00:00:00.02
	Memory Required	35200 bytes
	Additional Memory Required for Residual Plots	0 bytes

Variables Entered/Removed[a]

Model	Variables Entered	Variables Removed	Method
1	Party0[b]	.	Enter
2	GenderCC, SES0, RaceCC[b]	.	Enter
3	National0[b]	.	Enter
4	MRN0[b]	.	Enter
5	MRN0xGender, MRN0xSES0, MRN0xNational0, MRN0xParty0, MRN0xRace[b]	.	Enter

a. Dependent Variable: Psychology_Tot

b. All requested variables entered.

Model Summary

Model	R	R Square	Adjusted R Square	Std. Error of the Estimate	Change Statistics		
					R Square Change	F Change	df1
1	.180[a]	.033	.027	1.40677	.033	6.250	1
2	.227[b]	.052	.031	1.40411	.019	1.235	3
3	.229[c]	.052	.026	1.40757	.001	.103	1
4	.257[d]	.066	.035	1.40112	.014	2.679	1
5	.364[e]	.132	.078	1.36970	.066	2.680	5

Model Summary

Model	Change Statistics	
	df2	Sig. F Change
1	186	.013
2	183	.298
3	182	.749
4	181	.103
5	176	.023

a. Predictors: (Constant), Party0

b. Predictors: (Constant), Party0, GenderCC, SES0, RaceCC

c. Predictors: (Constant), Party0, GenderCC, SES0, RaceCC, National0

d. Predictors: (Constant), Party0, GenderCC, SES0, RaceCC, National0, MRN0

e. Predictors: (Constant), Party0, GenderCC, SES0, RaceCC, National0, MRN0, MRN0xGender, MRN0xSES0, MRN0xNational0, MRN0xParty0, MRN0xRace

ANOVA[a]

Model		Sum of Squares	df	Mean Square	F	Sig.
1	Regression	12.370	1	12.370	6.250	.013[b]
	Residual	368.096	186	1.979		
	Total	380.465	187			
2	Regression	19.674	4	4.918	2.495	.045[c]
	Residual	360.791	183	1.972		
	Total	380.465	187			
3	Regression	19.877	5	3.975	2.006	.080[d]
	Residual	360.588	182	1.981		
	Total	380.465	187			
4	Regression	25.136	6	4.189	2.134	.052[e]
	Residual	355.330	181	1.963		
	Total	380.465	187			
5	Regression	50.275	11	4.570	2.436	.008[f]
	Residual	330.190	176	1.876		
	Total	380.465	187			

a. Dependent Variable: Psychology_Tot

b. Predictors: (Constant), Party0

c. Predictors: (Constant), Party0, GenderCC, SES0, RaceCC

d. Predictors: (Constant), Party0, GenderCC, SES0, RaceCC, National0

e. Predictors: (Constant), Party0, GenderCC, SES0, RaceCC, National0, MRN0

f. Predictors: (Constant), Party0, GenderCC, SES0, RaceCC, National0, MRN0, MRN0xGender, MRN0xSES0, MRN0xNational0, MRN0xParty0, MRN0xRace

Coefficients[a]

Model		Unstandardized Coefficients B	Unstandardized Coefficients Std. Error	Standardized Coefficients Beta	t	Sig.
1	(Constant)	4.541	.103		44.259	.000
	Party0	-.211	.084	-.180	-2.500	.013
2	(Constant)	4.507	.113		39.876	.000
	Party0	-.217	.089	-.186	-2.432	.016
	GenderCC	-.166	.104	-.117	-1.599	.111
	RaceCC	.085	.118	.055	.718	.474
	SES0	-.051	.143	-.026	-.360	.719
3	(Constant)	4.512	.115		39.352	.000
	Party0	-.225	.092	-.192	-2.431	.016
	GenderCC	-.166	.104	-.117	-1.592	.113
	RaceCC	.071	.126	.046	.565	.573
	SES0	-.054	.143	-.028	-.376	.708
	National0	.025	.077	.026	.320	.749
4	(Constant)	4.514	.114		39.549	.000
	Party0	-.172	.097	-.147	-1.765	.079
	GenderCC	-.072	.118	-.051	-.612	.541
	RaceCC	.064	.126	.041	.509	.611
	SES0	-.030	.144	-.016	-.210	.834
	National0	.046	.078	.049	.594	.553
	MRN0	-.247	.151	-.148	-1.637	.103
5	(Constant)	4.359	.128		34.007	.000
	Party0	-.177	.098	-.151	-1.799	.074
	GenderCC	-.055	.116	-.038	-.471	.638
	RaceCC	.032	.125	.021	.259	.796
	SES0	-.040	.144	-.021	-.278	.782
	National0	.044	.077	.047	.576	.565
	MRN0	-.095	.157	-.057	-.604	.546
	MRN0xRace	-.329	.142	-.197	-2.317	.022
	MRN0xSES0	.058	.194	.022	.299	.765
	MRN0xGender	.095	.135	.051	.704	.482
	MRN0xParty0	.248	.119	.170	2.078	.039
	MRN0xNational0	.140	.082	.140	1.694	.092

Coefficients[a]

Model		Correlations		
		Zero-order	Partial	Part
1	(Constant)			
	Party0	-.180	-.180	-.180
2	(Constant)			
	Party0	-.180	-.177	-.175
	GenderCC	-.133	-.117	-.115
	RaceCC	.016	.053	.052
	SES0	-.065	-.027	-.026
3	(Constant)			
	Party0	-.180	-.177	-.175
	GenderCC	-.133	-.117	-.115
	RaceCC	.016	.042	.041
	SES0	-.065	-.028	-.027
	National0	-.018	.024	.023
4	(Constant)			
	Party0	-.180	-.130	-.127
	GenderCC	-.133	-.045	-.044
	RaceCC	.016	.038	.037
	SES0	-.065	-.016	-.015
	National0	-.018	.044	.043
	MRN0	-.216	-.121	-.118
5	(Constant)			
	Party0	-.180	-.134	-.126
	GenderCC	-.133	-.035	-.033
	RaceCC	.016	.020	.018
	SES0	-.065	-.021	-.019
	National0	-.018	.043	.040
	MRN0	-.216	-.045	-.042
	MRN0xRace	-.167	-.172	-.163
	MRN0xSES0	.049	.023	.021
	MRN0xGender	.091	.053	.049
	MRN0xParty0	.165	.155	.146
	MRN0xNational0	.144	.127	.119

a. Dependent Variable: Psychology_Tot

Excluded Variables[a]

Model		Beta In	t	Sig.	Partial Correlation	Collinearity Statistics Tolerance
1	GenderCC	-.126[b]	-1.762	.080	-.128	.998
	RaceCC	.073[b]	.968	.334	.071	.920
	SES0	-.031[b]	-.416	.678	-.031	.962
	National0	.048[b]	.631	.529	.046	.884
	MRN0	-.172[b]	-2.233	.027	-.162	.854
	MRN0xRace	-.126[b]	-1.685	.094	-.123	.922
	MRN0xSES0	.057[b]	.786	.433	.058	.998
	MRN0xGender	.074[b]	1.018	.310	.075	.991
	MRN0xParty0	.198[b]	2.757	.006	.199	.975
	MRN0xNational0	.155[b]	2.170	.031	.158	.997
2	National0	.026[c]	.320	.749	.024	.776
	MRN0	-.139[c]	-1.561	.120	-.115	.650
	MRN0xRace	-.105[c]	-1.385	.168	-.102	.895
	MRN0xSES0	.054[c]	.733	.464	.054	.956
	MRN0xGender	.076[c]	1.050	.295	.078	.989
	MRN0xParty0	.184[c]	2.540	.012	.185	.958
	MRN0xNational0	.153[c]	2.112	.036	.155	.974
3	MRN0	-.148[d]	-1.637	.103	-.121	.631
	MRN0xRace	-.104[d]	-1.370	.172	-.101	.894
	MRN0xSES0	.053[d]	.715	.476	.053	.953
	MRN0xGender	.079[d]	1.080	.281	.080	.981
	MRN0xParty0	.185[d]	2.541	.012	.186	.957
	MRN0xNational0	.155[d]	2.140	.034	.157	.968
4	MRN0xRace	-.080[e]	-1.028	.305	-.076	.848
	MRN0xSES0	.049[e]	.666	.506	.050	.952
	MRN0xGender	.085[e]	1.166	.245	.087	.979
	MRN0xParty0	.178[e]	2.449	.015	.180	.953
	MRN0xNational0	.140[e]	1.914	.057	.141	.945

a. Dependent Variable: Psychology_Tot

b. Predictors in the Model: (Constant), Party0

c. Predictors in the Model: (Constant), Party0, GenderCC, SES0, RaceCC

d. Predictors in the Model: (Constant), Party0, GenderCC, SES0, RaceCC, National0

e. Predictors in the Model: (Constant), Party0, GenderCC, SES0, RaceCC, National0, MRN0

```
REGRESSION
 /MISSING LISTWISE
 /STATISTICS COEFF OUTS R ANOVA CHANGE ZPP
 /CRITERIA=PIN(.05) POUT(.10)
 /NOORIGIN
 /DEPENDENT Risk_Rules
 /METHOD=ENTER Party0
 /METHOD=ENTER GenderCC RaceCC SES0
  /METHOD=ENTER National0
 /METHOD=ENTER MRN0
 /METHOD=ENTER MRN0xRace MRN0xSES0 MRN0xGender MRN0xParty0 MRN0xNational0.
```

Regression

Notes

Output Created		15-DEC-2021 13:09:41
Comments		
Input	Data	C:\Users\njs5478\Dropbox\HM and COVID\0. Revise and Resubmit\2. R and R Data\Study 2a\Study2a_Data.sav
	Active Dataset	DataSet1
	Filter	<none>
	Weight	<none>
	Split File	<none>
	N of Rows in Working Data File	188
Missing Value Handling	Definition of Missing	User-defined missing values are treated as missing.
	Cases Used	Statistics are based on cases with no missing values for any variable used.

Notes

Syntax		REGRESSION<br>/MISSING LISTWISE<br>/STATISTICS COEFF OUTS R ANOVA CHANGE ZPP<br>/CRITERIA=PIN(.05) POUT(.10)<br>/NOORIGIN<br>/DEPENDENT Risk_Rules<br>/METHOD=ENTER Party0<br>/METHOD=ENTER GenderCC RaceCC SES0<br>/METHOD=ENTER National0<br>/METHOD=ENTER MRN0<br>/METHOD=ENTER MRN0xRace MRN0xSES0 MRN0xGender MRN0xParty0 MRN0xNational0.
Resources	Processor Time	00:00:00.02
	Elapsed Time	00:00:00.03
	Memory Required	35200 bytes
	Additional Memory Required for Residual Plots	0 bytes

Variables Entered/Removed[a]

Model	Variables Entered	Variables Removed	Method
1	Party0[b]	.	Enter
2	GenderCC, SES0, RaceCC[b]	.	Enter
3	National0[b]	.	Enter
4	MRN0[b]	.	Enter
5	MRN0xGender, MRN0xSES0, MRN0xNational0, MRN0xParty0, MRN0xRace[b]	.	Enter

a. Dependent Variable: Risk_Rules

b. All requested variables entered.

Model Summary

Model	R	R Square	Adjusted R Square	Std. Error of the Estimate	Change Statistics		
					R Square Change	F Change	df1
1	.515[a]	.265	.261	.92388	.265	66.974	1
2	.580[b]	.336	.322	.88488	.072	6.585	3
3	.597[c]	.357	.339	.87353	.020	5.788	1
4	.644[d]	.414	.395	.83584	.058	17.782	1
5	.651[e]	.424	.388	.84075	.009	.579	5

Model Summary

Model	Change Statistics	
	df2	Sig. F Change
1	186	.000
2	183	.000
3	182	.017
4	181	.000
5	176	.716

a. Predictors: (Constant), Party0

b. Predictors: (Constant), Party0, GenderCC, SES0, RaceCC

c. Predictors: (Constant), Party0, GenderCC, SES0, RaceCC, National0

d. Predictors: (Constant), Party0, GenderCC, SES0, RaceCC, National0, MRN0

e. Predictors: (Constant), Party0, GenderCC, SES0, RaceCC, National0, MRN0, MRN0xGender, MRN0xSES0, MRN0xNational0, MRN0xParty0, MRN0xRace

ANOVA[a]

Model		Sum of Squares	df	Mean Square	F	Sig.
1	Regression	57.166	1	57.166	66.974	.000[b]
	Residual	158.761	186	.854		
	Total	215.926	187			
2	Regression	72.635	4	18.159	23.191	.000[c]
	Residual	143.292	183	.783		
	Total	215.926	187			
3	Regression	77.051	5	15.410	20.196	.000[d]
	Residual	138.875	182	.763		
	Total	215.926	187			
4	Regression	89.474	6	14.912	21.345	.000[e]
	Residual	126.452	181	.699		
	Total	215.926	187			
5	Regression	91.519	11	8.320	11.770	.000[f]
	Residual	124.407	176	.707		
	Total	215.926	187			

a. Dependent Variable: Risk_Rules

b. Predictors: (Constant), Party0

c. Predictors: (Constant), Party0, GenderCC, SES0, RaceCC

d. Predictors: (Constant), Party0, GenderCC, SES0, RaceCC, National0

e. Predictors: (Constant), Party0, GenderCC, SES0, RaceCC, National0, MRN0

f. Predictors: (Constant), Party0, GenderCC, SES0, RaceCC, National0, MRN0, MRN0xGender, MRN0xSES0, MRN0xNational0, MRN0xParty0, MRN0xRace

Coefficients[a]

Model		Unstandardized Coefficients B	Unstandardized Coefficients Std. Error	Standardized Coefficients Beta	t	Sig.
1	(Constant)	3.925	.067		58.255	.000
	Party0	.453	.055	.515	8.184	.000
2	(Constant)	3.844	.071		53.976	.000
	Party0	.389	.056	.441	6.894	.000
	GenderCC	.230	.066	.215	3.510	.001
	RaceCC	.200	.075	.171	2.679	.008
	SES0	.126	.090	.086	1.395	.165
3	(Constant)	3.871	.071		54.395	.000
	Party0	.355	.057	.403	6.183	.000
	GenderCC	.232	.065	.216	3.584	.000
	RaceCC	.136	.078	.116	1.734	.085
	SES0	.114	.089	.078	1.286	.200
	National0	.115	.048	.162	2.406	.017
4	(Constant)	3.868	.068		56.798	.000
	Party0	.274	.058	.311	4.709	.000
	GenderCC	.088	.071	.082	1.249	.213
	RaceCC	.147	.075	.125	1.960	.052
	SES0	.078	.086	.053	.911	.364
	National0	.082	.046	.116	1.763	.080
	MRN0	.380	.090	.302	4.217	.000
5	(Constant)	3.848	.079		48.908	.000
	Party0	.252	.060	.286	4.181	.000
	GenderCC	.093	.071	.087	1.302	.194
	RaceCC	.147	.077	.126	1.926	.056
	SES0	.068	.088	.046	.770	.443
	National0	.085	.047	.121	1.815	.071
	MRN0	.376	.097	.299	3.893	.000
	MRN0xRace	.061	.087	.048	.699	.485
	MRN0xSES0	-.038	.119	-.019	-.316	.753
	MRN0xGender	-.027	.083	-.019	-.328	.743
	MRN0xParty0	.064	.073	.058	.869	.386
	MRN0xNational0	.020	.051	.027	.401	.689

Coefficients[a]

Model		Correlations		
		Zero-order	Partial	Part
1	(Constant)			
	Party0	.515	.515	.515
2	(Constant)			
	Party0	.515	.454	.415
	GenderCC	.213	.251	.211
	RaceCC	.273	.194	.161
	SES0	.200	.103	.084
3	(Constant)			
	Party0	.515	.417	.368
	GenderCC	.213	.257	.213
	RaceCC	.273	.127	.103
	SES0	.200	.095	.076
	National0	.347	.176	.143
4	(Constant)			
	Party0	.515	.330	.268
	GenderCC	.213	.092	.071
	RaceCC	.273	.144	.111
	SES0	.200	.068	.052
	National0	.347	.130	.100
	MRN0	.503	.299	.240
5	(Constant)			
	Party0	.515	.301	.239
	GenderCC	.213	.098	.075
	RaceCC	.273	.144	.110
	SES0	.200	.058	.044
	National0	.347	.136	.104
	MRN0	.503	.282	.223
	MRN0xRace	.269	.053	.040
	MRN0xSES0	.007	-.024	-.018
	MRN0xGender	-.067	-.025	-.019
	MRN0xParty0	.134	.065	.050
	MRN0xNational0	.013	.030	.023

a. Dependent Variable: Risk_Rules

Excluded Variables[a]

Model		Beta In	t	Sig.	Partial Correlation	Collinearity Statistics Tolerance
1	GenderCC	.193[b]	3.142	.002	.225	.998
	RaceCC	.139[b]	2.134	.034	.155	.920
	SES0	.103[b]	1.617	.108	.118	.962
	National0	.194[b]	2.961	.003	.213	.884
	MRN0	.359[b]	5.712	.000	.387	.854
	MRN0xRace	.135[b]	2.087	.038	.152	.922
	MRN0xSES0	-.014[b]	-.225	.822	-.017	.998
	MRN0xGender	-.017[b]	-.276	.783	-.020	.991
	MRN0xParty0	.054[b]	.851	.396	.062	.975
	MRN0xNational0	-.018[b]	-.282	.778	-.021	.997
2	National0	.162[c]	2.406	.017	.176	.776
	MRN0	.323[c]	4.556	.000	.320	.650
	MRN0xRace	.126[c]	1.989	.048	.146	.895
	MRN0xSES0	.000[c]	-.007	.994	-.001	.956
	MRN0xGender	-.015[c]	-.245	.807	-.018	.989
	MRN0xParty0	.059[c]	.952	.342	.070	.958
	MRN0xNational0	.015[c]	.252	.801	.019	.974
3	MRN0	.302[d]	4.217	.000	.299	.631
	MRN0xRace	.132[d]	2.113	.036	.155	.894
	MRN0xSES0	-.009[d]	-.140	.889	-.010	.953
	MRN0xGender	-.002[d]	-.037	.970	-.003	.981
	MRN0xParty0	.062[d]	1.016	.311	.075	.957
	MRN0xNational0	.027[d]	.447	.655	.033	.968
4	MRN0xRace	.078[e]	1.265	.208	.094	.848
	MRN0xSES0	-.001[e]	-.013	.990	-.001	.952
	MRN0xGender	-.014[e]	-.243	.808	-.018	.979
	MRN0xParty0	.078[e]	1.337	.183	.099	.953
	MRN0xNational0	.066[e]	1.131	.260	.084	.945

a. Dependent Variable: Risk_Rules

b. Predictors in the Model: (Constant), Party0

c. Predictors in the Model: (Constant), Party0, GenderCC, SES0, RaceCC

d. Predictors in the Model: (Constant), Party0, GenderCC, SES0, RaceCC, National0

e. Predictors in the Model: (Constant), Party0, GenderCC, SES0, RaceCC, National0, MRN0

```
REGRESSION
 /MISSING LISTWISE
 /STATISTICS COEFF OUTS R ANOVA CHANGE ZPP
 /CRITERIA=PIN(.05) POUT(.10)
 /NOORIGIN
 /DEPENDENT Mandate_Tot
 /METHOD=ENTER Party0
 /METHOD=ENTER GenderCC RaceCC SES0
  /METHOD=ENTER National0
 /METHOD=ENTER MRN0
 /METHOD=ENTER MRN0xRace MRN0xSES0 MRN0xGender MRN0xParty0 MRN0xNational0.
```

Regression

Notes

Output Created		15-DEC-2021 13:09:41
Comments		
Input	Data	C: \Users\njs5478\Dropbox\HM and COVID\0. Revise and Resubmit\2. R and R Data\Study 2a\Study2a_Data.sav
	Active Dataset	DataSet1
	Filter	<none>
	Weight	<none>
	Split File	<none>
	N of Rows in Working Data File	188
Missing Value Handling	Definition of Missing	User-defined missing values are treated as missing.
	Cases Used	Statistics are based on cases with no missing values for any variable used.

Notes

Syntax		REGRESSION<br>/MISSING LISTWISE<br>/STATISTICS COEFF OUTS R ANOVA CHANGE ZPP<br>/CRITERIA=PIN(.05) POUT(.10)<br>/NOORIGIN<br>/DEPENDENT Mandate_Tot<br>/METHOD=ENTER Party0<br>/METHOD=ENTER GenderCC RaceCC SES0<br>/METHOD=ENTER National0<br>/METHOD=ENTER MRN0<br>/METHOD=ENTER MRN0xRace MRN0xSES0 MRN0xGender MRN0xParty0 MRN0xNational0.
Resources	Processor Time	00:00:00.05
	Elapsed Time	00:00:00.03
	Memory Required	35200 bytes
	Additional Memory Required for Residual Plots	0 bytes

Variables Entered/Removed[a]

Model	Variables Entered	Variables Removed	Method
1	Party0[b]	.	Enter
2	GenderCC, SES0, RaceCC[b]	.	Enter
3	National0[b]	.	Enter
4	MRN0[b]	.	Enter
5	MRN0xGender, MRN0xSES0, MRN0xNational0, MRN0xParty0, MRN0xRace[b]	.	Enter

a. Dependent Variable: Mandate_Tot

b. All requested variables entered.

Model Summary

Model	R	R Square	Adjusted R Square	Std. Error of the Estimate	Change Statistics		
					R Square Change	F Change	df1
1	.548[a]	.300	.296	1.47195	.300	79.727	1
2	.606[b]	.368	.354	1.41041	.068	6.528	3
3	.618[c]	.382	.365	1.39801	.014	4.262	1
4	.635[d]	.404	.384	1.37717	.022	6.550	1
5	.649[e]	.421	.385	1.37586	.018	1.069	5

Model Summary

Model	Change Statistics	
	df2	Sig. F Change
1	186	.000
2	183	.000
3	182	.040
4	181	.011
5	176	.379

a. Predictors: (Constant), Party0

b. Predictors: (Constant), Party0, GenderCC, SES0, RaceCC

c. Predictors: (Constant), Party0, GenderCC, SES0, RaceCC, National0

d. Predictors: (Constant), Party0, GenderCC, SES0, RaceCC, National0, MRN0

e. Predictors: (Constant), Party0, GenderCC, SES0, RaceCC, National0, MRN0, MRN0xGender, MRN0xSES0, MRN0xNational0, MRN0xParty0, MRN0xRace

ANOVA[a]

Model		Sum of Squares	df	Mean Square	F	Sig.
1	Regression	172.739	1	172.739	79.727	.000[b]
	Residual	402.994	186	2.167		
	Total	575.732	187			
2	Regression	211.697	4	52.924	26.605	.000[c]
	Residual	364.035	183	1.989		
	Total	575.732	187			
3	Regression	220.027	5	44.005	22.516	.000[d]
	Residual	355.705	182	1.954		
	Total	575.732	187			
4	Regression	232.450	6	38.742	20.427	.000[e]
	Residual	343.282	181	1.897		
	Total	575.732	187			
5	Regression	242.568	11	22.052	11.649	.000[f]
	Residual	333.164	176	1.893		
	Total	575.732	187			

a. Dependent Variable: Mandate_Tot

b. Predictors: (Constant), Party0

c. Predictors: (Constant), Party0, GenderCC, SES0, RaceCC

d. Predictors: (Constant), Party0, GenderCC, SES0, RaceCC, National0

e. Predictors: (Constant), Party0, GenderCC, SES0, RaceCC, National0, MRN0

f. Predictors: (Constant), Party0, GenderCC, SES0, RaceCC, National0, MRN0, MRN0xGender, MRN0xSES0, MRN0xNational0, MRN0xParty0, MRN0xRace

Coefficients[a]

Model		Unstandardized Coefficients B	Unstandardized Coefficients Std. Error	Standardized Coefficients Beta	t	Sig.
1	(Constant)	4.677	.107		43.566	.000
	Party0	-.788	.088	-.548	-8.929	.000
2	(Constant)	4.755	.114		41.885	.000
	Party0	-.770	.090	-.536	-8.576	.000
	GenderCC	-.403	.104	-.230	-3.857	.000
	RaceCC	-.194	.119	-.101	-1.631	.105
	SES0	.311	.144	.130	2.167	.032
3	(Constant)	4.719	.114		41.434	.000
	Party0	-.724	.092	-.503	-7.884	.000
	GenderCC	-.405	.104	-.232	-3.915	.000
	RaceCC	-.106	.125	-.055	-.845	.399
	SES0	.327	.143	.137	2.291	.023
	National0	-.157	.076	-.137	-2.064	.040
4	(Constant)	4.722	.112		42.086	.000
	Party0	-.643	.096	-.447	-6.711	.000
	GenderCC	-.262	.116	-.150	-2.249	.026
	RaceCC	-.117	.123	-.061	-.948	.344
	SES0	.363	.141	.152	2.573	.011
	National0	-.124	.076	-.108	-1.632	.104
	MRN0	-.380	.148	-.185	-2.559	.011
5	(Constant)	4.791	.129		37.207	.000
	Party0	-.598	.099	-.416	-6.066	.000
	GenderCC	-.269	.117	-.154	-2.306	.022
	RaceCC	-.112	.125	-.058	-.891	.374
	SES0	.363	.145	.152	2.508	.013
	National0	-.134	.077	-.116	-1.734	.085
	MRN0	-.330	.158	-.161	-2.091	.038
	MRN0xRace	-.199	.143	-.097	-1.397	.164
	MRN0xSES0	-.028	.195	-.009	-.144	.886
	MRN0xGender	-.018	.136	-.008	-.135	.893
	MRN0xParty0	-.150	.120	-.084	-1.254	.212
	MRN0xNational0	.027	.083	.022	.332	.741

Coefficients[a]

Model		Correlations		
		Zero-order	Partial	Part
1	(Constant)			
	Party0	-.548	-.548	-.548
2	(Constant)			
	Party0	-.548	-.535	-.504
	GenderCC	-.229	-.274	-.227
	RaceCC	-.209	-.120	-.096
	SES0	.003	.158	.127
3	(Constant)			
	Party0	-.548	-.505	-.459
	GenderCC	-.229	-.279	-.228
	RaceCC	-.209	-.063	-.049
	SES0	.003	.167	.134
	National0	-.303	-.151	-.120
4	(Constant)			
	Party0	-.548	-.446	-.385
	GenderCC	-.229	-.165	-.129
	RaceCC	-.209	-.070	-.054
	SES0	.003	.188	.148
	National0	-.303	-.120	-.094
	MRN0	-.425	-.187	-.147
5	(Constant)			
	Party0	-.548	-.416	-.348
	GenderCC	-.229	-.171	-.132
	RaceCC	-.209	-.067	-.051
	SES0	.003	.186	.144
	National0	-.303	-.130	-.099
	MRN0	-.425	-.156	-.120
	MRN0xRace	-.294	-.105	-.080
	MRN0xSES0	-.085	-.011	-.008
	MRN0xGender	.048	-.010	-.008
	MRN0xParty0	-.163	-.094	-.072
	MRN0xNational0	-.011	.025	.019

a. Dependent Variable: Mandate_Tot

Excluded Variables[a]

Model		Beta In	t	Sig.	Partial Correlation	Collinearity Statistics Tolerance
1	GenderCC	-.208[b]	-3.493	.001	-.249	.998
	RaceCC	-.059[b]	-.918	.360	-.067	.920
	SES0	.115[b]	1.842	.067	.134	.962
	National0	-.132[b]	-2.041	.043	-.148	.884
	MRN0	-.253[b]	-3.961	.000	-.280	.854
	MRN0xRace	-.152[b]	-2.415	.017	-.175	.922
	MRN0xSES0	-.062[b]	-1.011	.313	-.074	.998
	MRN0xGender	-.005[b]	-.083	.934	-.006	.991
	MRN0xParty0	-.079[b]	-1.280	.202	-.094	.975
	MRN0xNational0	.021[b]	.344	.731	.025	.997
2	National0	-.137[c]	-2.064	.040	-.151	.776
	MRN0	-.205[c]	-2.864	.005	-.208	.650
	MRN0xRace	-.141[c]	-2.303	.022	-.168	.895
	MRN0xSES0	-.035[c]	-.574	.567	-.043	.956
	MRN0xGender	-.012[c]	-.199	.842	-.015	.989
	MRN0xParty0	-.095[c]	-1.580	.116	-.116	.958
	MRN0xNational0	-.015[c]	-.243	.808	-.018	.974
3	MRN0	-.185[d]	-2.559	.011	-.187	.631
	MRN0xRace	-.147[d]	-2.408	.017	-.176	.894
	MRN0xSES0	-.028[d]	-.466	.642	-.035	.953
	MRN0xGender	-.023[d]	-.383	.702	-.028	.981
	MRN0xParty0	-.097[d]	-1.640	.103	-.121	.957
	MRN0xNational0	-.024[d]	-.410	.682	-.030	.968
4	MRN0xRace	-.117[e]	-1.895	.060	-.140	.848
	MRN0xSES0	-.033[e]	-.555	.580	-.041	.952
	MRN0xGender	-.015[e]	-.266	.791	-.020	.979
	MRN0xParty0	-.107[e]	-1.836	.068	-.136	.953
	MRN0xNational0	-.048[e]	-.819	.414	-.061	.945

a. Dependent Variable: Mandate_Tot

b. Predictors in the Model: (Constant), Party0

c. Predictors in the Model: (Constant), Party0, GenderCC, SES0, RaceCC

d. Predictors in the Model: (Constant), Party0, GenderCC, SES0, RaceCC, National0

e. Predictors in the Model: (Constant), Party0, GenderCC, SES0, RaceCC, National0, MRN0

```
REGRESSION
 /MISSING LISTWISE
 /STATISTICS COEFF OUTS R ANOVA CHANGE ZPP
 /CRITERIA=PIN(.05) POUT(.10)
 /NOORIGIN
 /DEPENDENT Conspiracy_Tot
 /METHOD=ENTER Party0
 /METHOD=ENTER GenderCC RaceCC SES0
  /METHOD=ENTER National0
 /METHOD=ENTER MRN0
 /METHOD=ENTER MRN0xRace MRN0xSES0 MRN0xGender MRN0xParty0 MRN0xNational0.
```

Regression

Notes

Output Created		15-DEC-2021 13:09:41
Comments		
Input	Data	C: \Users\njs5478\Dropbox\HM and COVID\0. Revise and Resubmit\2. R and R Data\Study 2a\Study2a_Data.sav
	Active Dataset	DataSet1
	Filter	<none>
	Weight	<none>
	Split File	<none>
	N of Rows in Working Data File	188
Missing Value Handling	Definition of Missing	User-defined missing values are treated as missing.
	Cases Used	Statistics are based on cases with no missing values for any variable used.

Notes

Syntax		REGRESSION<br>/MISSING LISTWISE<br>/STATISTICS COEFF OUTS R ANOVA CHANGE ZPP<br>/CRITERIA=PIN(.05) POUT(.10)<br>/NOORIGIN<br>/DEPENDENT Conspiracy_Tot<br>/METHOD=ENTER Party0<br>/METHOD=ENTER GenderCC RaceCC SES0<br>/METHOD=ENTER National0<br>/METHOD=ENTER MRN0<br>/METHOD=ENTER MRN0xRace MRN0xSES0 MRN0xGender MRN0xParty0 MRN0xNational0.
Resources	Processor Time	00:00:00.02
	Elapsed Time	00:00:00.03
	Memory Required	35200 bytes
	Additional Memory Required for Residual Plots	0 bytes

Variables Entered/Removed[a]

Model	Variables Entered	Variables Removed	Method
1	Party0[b]	.	Enter
2	GenderCC, SES0, RaceCC[b]	.	Enter
3	National0[b]	.	Enter
4	MRN0[b]	.	Enter
5	MRN0xGender, MRN0xSES0, MRN0xNational0, MRN0xParty0, MRN0xRace[b]	.	Enter

a. Dependent Variable: Conspiracy_Tot

b. All requested variables entered.

Model Summary

Model	R	R Square	Adjusted R Square	Std. Error of the Estimate	Change Statistics		
					R Square Change	F Change	df1
1	.361[a]	.130	.125	.59204	.130	27.809	1
2	.384[b]	.147	.129	.59092	.017	1.235	3
3	.388[c]	.150	.127	.59149	.003	.644	1
4	.448[d]	.201	.174	.57534	.050	11.362	1
5	.543[e]	.295	.251	.54793	.094	4.712	5

Model Summary

Model	Change Statistics	
	df2	Sig. F Change
1	186	.000
2	183	.298
3	182	.423
4	181	.001
5	176	.000

a. Predictors: (Constant), Party0

b. Predictors: (Constant), Party0, GenderCC, SES0, RaceCC

c. Predictors: (Constant), Party0, GenderCC, SES0, RaceCC, National0

d. Predictors: (Constant), Party0, GenderCC, SES0, RaceCC, National0, MRN0

e. Predictors: (Constant), Party0, GenderCC, SES0, RaceCC, National0, MRN0, MRN0xGender, MRN0xSES0, MRN0xNational0, MRN0xParty0, MRN0xRace

ANOVA[a]

Model		Sum of Squares	df	Mean Square	F	Sig.
1	Regression	9.747	1	9.747	27.809	.000[b]
	Residual	65.194	186	.351		
	Total	74.942	187			
2	Regression	11.041	4	2.760	7.905	.000[c]
	Residual	63.901	183	.349		
	Total	74.942	187			
3	Regression	11.266	5	2.253	6.440	.000[d]
	Residual	63.676	182	.350		
	Total	74.942	187			
4	Regression	15.027	6	2.505	7.566	.000[e]
	Residual	59.914	181	.331		
	Total	74.942	187			
5	Regression	22.101	11	2.009	6.692	.000[f]
	Residual	52.840	176	.300		
	Total	74.942	187			

a. Dependent Variable: Conspiracy_Tot

b. Predictors: (Constant), Party0

c. Predictors: (Constant), Party0, GenderCC, SES0, RaceCC

d. Predictors: (Constant), Party0, GenderCC, SES0, RaceCC, National0

e. Predictors: (Constant), Party0, GenderCC, SES0, RaceCC, National0, MRN0

f. Predictors: (Constant), Party0, GenderCC, SES0, RaceCC, National0, MRN0, MRN0xGender, MRN0xSES0, MRN0xNational0, MRN0xParty0, MRN0xRace

Coefficients[a]

Model		Unstandardized Coefficients B	Unstandardized Coefficients Std. Error	Standardized Coefficients Beta	t	Sig.
1	(Constant)	1.545	.043		35.777	.000
	Party0	.187	.035	.361	5.273	.000
2	(Constant)	1.567	.048		32.939	.000
	Party0	.188	.038	.363	5.003	.000
	GenderCC	.029	.044	.046	.665	.507
	RaceCC	-.054	.050	-.079	-1.092	.276
	SES0	.081	.060	.094	1.341	.182
3	(Constant)	1.561	.048		32.391	.000
	Party0	.196	.039	.378	5.043	.000
	GenderCC	.029	.044	.045	.654	.514
	RaceCC	-.040	.053	-.058	-.753	.453
	SES0	.083	.060	.096	1.380	.169
	National0	-.026	.032	-.062	-.802	.423
4	(Constant)	1.559	.047		33.261	.000
	Party0	.151	.040	.292	3.782	.000
	GenderCC	-.050	.049	-.080	-1.035	.302
	RaceCC	-.034	.052	-.049	-.654	.514
	SES0	.063	.059	.073	1.070	.286
	National0	-.044	.032	-.106	-1.384	.168
	MRN0	.209	.062	.282	3.371	.001
5	(Constant)	1.471	.051		28.690	.000
	Party0	.131	.039	.253	3.340	.001
	GenderCC	-.036	.046	-.057	-.780	.436
	RaceCC	-.034	.050	-.050	-.688	.492
	SES0	.029	.058	.033	.497	.620
	National0	-.030	.031	-.072	-.977	.330
	MRN0	.227	.063	.307	3.611	.000
	MRN0xRace	.018	.057	.024	.311	.756
	MRN0xSES0	-.132	.078	-.112	-1.698	.091
	MRN0xGender	.101	.054	.121	1.866	.064
	MRN0xParty0	.106	.048	.165	2.227	.027
	MRN0xNational0	.070	.033	.158	2.112	.036

Coefficients[a]

Model		Correlations		
		Zero-order	Partial	Part
1	(Constant)			
	Party0	.361	.361	.361
2	(Constant)			
	Party0	.361	.347	.342
	GenderCC	.077	.049	.045
	RaceCC	.026	-.080	-.075
	SES0	.160	.099	.092
3	(Constant)			
	Party0	.361	.350	.345
	GenderCC	.077	.048	.045
	RaceCC	.026	-.056	-.051
	SES0	.160	.102	.094
	National0	.052	-.059	-.055
4	(Constant)			
	Party0	.361	.271	.251
	GenderCC	.077	-.077	-.069
	RaceCC	.026	-.049	-.043
	SES0	.160	.079	.071
	National0	.052	-.102	-.092
	MRN0	.342	.243	.224
5	(Constant)			
	Party0	.361	.244	.211
	GenderCC	.077	-.059	-.049
	RaceCC	.026	-.052	-.044
	SES0	.160	.037	.031
	National0	.052	-.073	-.062
	MRN0	.342	.263	.229
	MRN0xRace	.275	.023	.020
	MRN0xSES0	-.067	-.127	-.107
	MRN0xGender	.121	.139	.118
	MRN0xParty0	.253	.166	.141
	MRN0xNational0	.206	.157	.134

a. Dependent Variable: Conspiracy_Tot

Excluded Variables[a]

Model		Beta In	t	Sig.	Partial Correlation	Collinearity Statistics Tolerance
1	GenderCC	.063[b]	.915	.361	.067	.998
	RaceCC	-.083[b]	-1.162	.247	-.085	.920
	SES0	.093[b]	1.338	.182	.098	.962
	National0	-.080[b]	-1.095	.275	-.080	.884
	MRN0	.239[b]	3.323	.001	.237	.854
	MRN0xRace	.189[b]	2.696	.008	.194	.922
	MRN0xSES0	-.082[b]	-1.206	.229	-.088	.998
	MRN0xGender	.158[b]	2.319	.021	.168	.991
	MRN0xParty0	.201[b]	2.970	.003	.213	.975
	MRN0xNational0	.186[b]	2.761	.006	.199	.997
2	National0	-.062[c]	-.802	.423	-.059	.776
	MRN0	.262[c]	3.174	.002	.229	.650
	MRN0xRace	.174[c]	2.450	.015	.179	.895
	MRN0xSES0	-.066[c]	-.944	.346	-.070	.956
	MRN0xGender	.153[c]	2.255	.025	.165	.989
	MRN0xParty0	.217[c]	3.195	.002	.230	.958
	MRN0xNational0	.185[c]	2.721	.007	.198	.974
3	MRN0	.282[d]	3.371	.001	.243	.631
	MRN0xRace	.172[d]	2.417	.017	.177	.894
	MRN0xSES0	-.063[d]	-.900	.369	-.067	.953
	MRN0xGender	.149[d]	2.188	.030	.161	.981
	MRN0xParty0	.216[d]	3.174	.002	.230	.957
	MRN0xNational0	.182[d]	2.660	.009	.194	.968
4	MRN0xRace	.125[e]	1.742	.083	.129	.848
	MRN0xSES0	-.056[e]	-.818	.414	-.061	.952
	MRN0xGender	.139[e]	2.085	.038	.154	.979
	MRN0xParty0	.232[e]	3.512	.001	.253	.953
	MRN0xNational0	.222[e]	3.336	.001	.241	.945

a. Dependent Variable: Conspiracy_Tot

b. Predictors in the Model: (Constant), Party0

c. Predictors in the Model: (Constant), Party0, GenderCC, SES0, RaceCC

d. Predictors in the Model: (Constant), Party0, GenderCC, SES0, RaceCC, National0

e. Predictors in the Model: (Constant), Party0, GenderCC, SES0, RaceCC, National0, MRN0

```
 **Simple Slopes

**MRN*National ID on COVID-19 Conspiracy Theories

UNIANOVA Conspiracy_Tot WITH MRN.Low RaceCC GenderCC SES0 Party0 National0
  /PRINT=ETASQ PARAMETER
  /DESIGN=MRN.Low RaceCC GenderCC SES0 Party0 National0
  MRN.Low*RaceCC MRN.Low*GenderCC MRN.Low*SES0 MRN.Low*Party0 MRN.Low*National0.
```

Univariate Analysis of Variance

Notes

Output Created		15-DEC-2021 13:09:41
Comments		
Input	Data	C:\Users\njs5478\Dropbox\HM and COVID\0. Revise and Resubmit\2. R and R Data\Study 2a\Study2a_Data.sav
	Active Dataset	DataSet1
	Filter	<none>
	Weight	<none>
	Split File	<none>
	N of Rows in Working Data File	188
Missing Value Handling	Definition of Missing	User-defined missing values are treated as missing.
	Cases Used	Statistics are based on all cases with valid data for all variables in the model.

Notes

Syntax		UNIANOVA Conspiracy_Tot WITH MRN.Low RaceCC GenderCC SES0 Party0 National0<br>/PRINT=ETASQ PARAMETER<br>/DESIGN=MRN.Low RaceCC GenderCC SES0 Party0 National0<br>MRN.Low*RaceCC MRN.Low*GenderCC MRN.Low*SES0 MRN.Low*Party0 MRN.Low*National0.
Resources	Processor Time	00:00:00.02
	Elapsed Time	00:00:00.02

Tests of Between-Subjects Effects

Dependent Variable: Conspiracy_Tot

Source	Type III Sum of Squares	df	Mean Square	F	Sig.
Corrected Model	22.101[a]	11	2.009	6.692	.000
Intercept	86.188	1	86.188	287.074	.000
MRN.Low	3.914	1	3.914	13.038	.000
RaceCC	.164	1	.164	.547	.461
GenderCC	1.078	1	1.078	3.589	.060
SES0	.985	1	.985	3.281	.072
Party0	.144	1	.144	.479	.490
National0	1.435	1	1.435	4.779	.030
MRN.Low * RaceCC	.029	1	.029	.097	.756
MRN.Low * GenderCC	1.045	1	1.045	3.482	.064
MRN.Low * SES0	.865	1	.865	2.882	.091
MRN.Low * Party0	1.489	1	1.489	4.959	.027
MRN.Low * National0	1.339	1	1.339	4.460	.036
Error	52.840	176	.300		
Total	523.654	188			
Corrected Total	74.942	187			

Tests of Between-Subjects Effects

Dependent Variable: Conspiracy_Tot

Source	Partial Eta Squared
Corrected Model	.295
Intercept	.620
MRN.Low	.069
RaceCC	.003
GenderCC	.020
SES0	.018
Party0	.003
National0	.026
MRN.Low * RaceCC	.001
MRN.Low * GenderCC	.019
MRN.Low * SES0	.016
MRN.Low * Party0	.027
MRN.Low * National0	.025
Error	
Total	
Corrected Total	

a. R Squared = .295 (Adjusted R Squared = .251)

Parameter Estimates

Dependent Variable: Conspiracy_Tot

Parameter	B	Std. Error	t	Sig.	95% Confidence Interval Lower Bound	95% Confidence Interval Upper Bound
Intercept	1.277	.075	16.943	.000	1.128	1.426
MRN.Low	.227	.063	3.611	.000	.103	.351
RaceCC	-.049	.067	-.739	.461	-.181	.083
GenderCC	-.122	.065	-1.894	.060	-.250	.005
SES0	.141	.078	1.811	.072	-.013	.295
Party0	.040	.058	.692	.490	-.075	.156
National0	-.089	.041	-2.186	.030	-.170	-.009
MRN.Low * RaceCC	.018	.057	.311	.756	-.094	.130
MRN.Low * GenderCC	.101	.054	1.866	.064	-.006	.208
MRN.Low * SES0	-.132	.078	-1.698	.091	-.285	.021
MRN.Low * Party0	.106	.048	2.227	.027	.012	.200
MRN.Low * National0	.070	.033	2.112	.036	.005	.135

Parameter Estimates

Dependent Variable: Conspiracy_Tot

Parameter	Partial Eta Squared
Intercept	.620
MRN.Low	.069
RaceCC	.003
GenderCC	.020
SES0	.018
Party0	.003
National0	.026
MRN.Low * RaceCC	.001
MRN.Low * GenderCC	.019
MRN.Low * SES0	.016
MRN.Low * Party0	.027
MRN.Low * National0	.025

```
UNIANOVA Conspiracy_Tot WITH MRN.High RaceCC GenderCC SES0 Party0 National0
 /PRINT=ETASQ PARAMETER
```

```
/DESIGN=MRN.High RaceCC GenderCC SES0 Party0 National0
MRN.High*RaceCC MRN.High*GenderCC MRN.High*SES0 MRN.High*Party0 MRN.High*National0.
```

Univariate Analysis of Variance

Notes

Output Created		15-DEC-2021 13:09:41
Comments		
Input	Data	C: \Users\njs5478\Dropbox\HM and COVID\0. Revise and Resubmit\2. R and R Data\Study 2a\Study2a_Data.sav
	Active Dataset	DataSet1
	Filter	<none>
	Weight	<none>
	Split File	<none>
	N of Rows in Working Data File	188
Missing Value Handling	Definition of Missing	User-defined missing values are treated as missing.
	Cases Used	Statistics are based on all cases with valid data for all variables in the model.
Syntax		UNIANOVA Conspiracy_Tot WITH MRN.High RaceCC GenderCC SES0 Party0 National0 /PRINT=ETASQ PARAMETER /DESIGN=MRN.High RaceCC GenderCC SES0 Party0 National0 MRN.High*RaceCC MRN.High*GenderCC MRN.High*SES0 MRN.High*Party0 MRN.High*National0.
Resources	Processor Time	00:00:00.00
	Elapsed Time	00:00:00.02

Tests of Between-Subjects Effects

Dependent Variable: Conspiracy_Tot

Source	Type III Sum of Squares	df	Mean Square	F	Sig.
Corrected Model	22.101[a]	11	2.009	6.692	.000
Intercept	155.209	1	155.209	516.969	.000
MRN.High	3.914	1	3.914	13.038	.000
RaceCC	.021	1	.021	.071	.790
GenderCC	.171	1	.171	.569	.452
SES0	.227	1	.227	.755	.386
Party0	4.935	1	4.935	16.436	.000
National0	.146	1	.146	.486	.487
MRN.High * RaceCC	.029	1	.029	.097	.756
MRN.High * GenderCC	1.045	1	1.045	3.482	.064
MRN.High * SES0	.865	1	.865	2.882	.091
MRN.High * Party0	1.489	1	1.489	4.959	.027
MRN.High * National0	1.339	1	1.339	4.460	.036
Error	52.840	176	.300		
Total	523.654	188			
Corrected Total	74.942	187			

Tests of Between-Subjects Effects

Dependent Variable: Conspiracy_Tot

Source	Partial Eta Squared
Corrected Model	.295
Intercept	.746
MRN.High	.069
RaceCC	.000
GenderCC	.003
SES0	.004
Party0	.085
National0	.003
MRN.High * RaceCC	.001
MRN.High * GenderCC	.019
MRN.High * SES0	.016
MRN.High * Party0	.027
MRN.High * National0	.025
Error	
Total	
Corrected Total	

a. R Squared = .295 (Adjusted R Squared = .251)

Parameter Estimates

Dependent Variable: Conspiracy_Tot

Parameter	B	Std. Error	t	Sig.	95% Confidence Interval	
					Lower Bound	Upper Bound
Intercept	1.665	.073	22.737	.000	1.521	1.810
MRN.High	.227	.063	3.611	.000	.103	.351
RaceCC	-.019	.072	-.266	.790	-.162	.123
GenderCC	.050	.066	.754	.452	-.081	.181
SES0	-.084	.097	-.869	.386	-.275	.107
Party0	.222	.055	4.054	.000	.114	.330
National0	.030	.042	.697	.487	-.054	.113
MRN.High * RaceCC	.018	.057	.311	.756	-.094	.130
MRN.High * GenderCC	.101	.054	1.866	.064	-.006	.208
MRN.High * SES0	-.132	.078	-1.698	.091	-.285	.021
MRN.High * Party0	.106	.048	2.227	.027	.012	.200
MRN.High * National0	.070	.033	2.112	.036	.005	.135

Parameter Estimates

Dependent Variable: Conspiracy_Tot

Parameter	Partial Eta Squared
Intercept	.746
MRN.High	.069
RaceCC	.000
GenderCC	.003
SES0	.004
Party0	.085
National0	.003
MRN.High * RaceCC	.001
MRN.High * GenderCC	.019
MRN.High * SES0	.016
MRN.High * Party0	.027
MRN.High * National0	.025

**With PIdeology

```
REGRESSION
  /MISSING LISTWISE
  /STATISTICS COEFF OUTS R ANOVA CHANGE ZPP
  /CRITERIA=PIN(.05) POUT(.10)
  /NOORIGIN
  /DEPENDENT Concern_Tot
  /METHOD=ENTER Ideology0
  /METHOD=ENTER GenderCC RaceCC SES0
   /METHOD=ENTER National0
  /METHOD=ENTER MRN0
  /METHOD=ENTER MRN0xRace MRN0xSES0 MRN0xGender MRN0xIdeology0 MRN0xNational0.
```

Regression

Notes

Output Created		15-DEC-2021 13:09:41
Comments		
Input	Data	C:\Users\njs5478\Dropbox\HM and COVID\0. Revise and Resubmit\2. R and R Data\Study 2a\Study2a_Data.sav
	Active Dataset	DataSet1
	Filter	<none>
	Weight	<none>
	Split File	<none>
	N of Rows in Working Data File	188
Missing Value Handling	Definition of Missing	User-defined missing values are treated as missing.
	Cases Used	Statistics are based on cases with no missing values for any variable used.

Notes

Syntax		REGRESSION<br>/MISSING LISTWISE<br>/STATISTICS COEFF OUTS R ANOVA CHANGE ZPP<br>/CRITERIA=PIN(.05) POUT(.10)<br>/NOORIGIN<br>/DEPENDENT Concern_Tot<br>/METHOD=ENTER Ideology0<br>/METHOD=ENTER GenderCC RaceCC SES0<br>/METHOD=ENTER National0<br>/METHOD=ENTER MRN0<br>/METHOD=ENTER MRN0xRace MRN0xSES0 MRN0xGender MRN0xIdeology0 MRN0xNational0.
Resources	Processor Time	00:00:00.02
	Elapsed Time	00:00:00.02
	Memory Required	35200 bytes
	Additional Memory Required for Residual Plots	0 bytes

Variables Entered/Removed[a]

Model	Variables Entered	Variables Removed	Method
1	Ideology0[b]	.	Enter
2	SES0, GenderCC, RaceCC[b]	.	Enter
3	National0[b]	.	Enter
4	MRN0[b]	.	Enter
5	MRN0xGender, MRN0xNational0, MRN0xSES0, MRN0xIdeology0, MRN0xRace[b]	.	Enter

a. Dependent Variable: Concern_Tot

b. All requested variables entered.

Model Summary

Model	R	R Square	Adjusted R Square	Std. Error of the Estimate	Change Statistics		
					R Square Change	F Change	df1
1	.504[a]	.254	.250	1.18060	.254	63.260	1
2	.575[b]	.331	.316	1.12737	.077	6.993	3
3	.590[c]	.348	.330	1.11581	.017	4.811	1
4	.594[d]	.353	.332	1.11411	.006	1.555	1
5	.615[e]	.378	.339	1.10818	.025	1.389	5

Model Summary

Model	Change Statistics	
	df2	Sig. F Change
1	186	.000
2	183	.000
3	182	.030
4	181	.214
5	176	.231

a. Predictors: (Constant), Ideology0

b. Predictors: (Constant), Ideology0, SES0, GenderCC, RaceCC

c. Predictors: (Constant), Ideology0, SES0, GenderCC, RaceCC, National0

d. Predictors: (Constant), Ideology0, SES0, GenderCC, RaceCC, National0, MRN0

e. Predictors: (Constant), Ideology0, SES0, GenderCC, RaceCC, National0, MRN0, MRN0xGender, MRN0xNational0, MRN0xSES0, MRN0xIdeology0, MRN0xRace

ANOVA[a]

Model		Sum of Squares	df	Mean Square	F	Sig.
1	Regression	88.173	1	88.173	63.260	.000[b]
	Residual	259.248	186	1.394		
	Total	347.421	187			
2	Regression	114.837	4	28.709	22.589	.000[c]
	Residual	232.585	183	1.271		
	Total	347.421	187			
3	Regression	120.826	5	24.165	19.409	.000[d]
	Residual	226.595	182	1.245		
	Total	347.421	187			
4	Regression	122.756	6	20.459	16.483	.000[e]
	Residual	224.665	181	1.241		
	Total	347.421	187			
5	Regression	131.283	11	11.935	9.718	.000[f]
	Residual	216.139	176	1.228		
	Total	347.421	187			

a. Dependent Variable: Concern_Tot

b. Predictors: (Constant), Ideology0

c. Predictors: (Constant), Ideology0, SES0, GenderCC, RaceCC

d. Predictors: (Constant), Ideology0, SES0, GenderCC, RaceCC, National0

e. Predictors: (Constant), Ideology0, SES0, GenderCC, RaceCC, National0, MRN0

f. Predictors: (Constant), Ideology0, SES0, GenderCC, RaceCC, National0, MRN0, MRN0xGender, MRN0xNational0, MRN0xSES0, MRN0xIdeology0, MRN0xRace

Coefficients[a]

Model		Unstandardized Coefficients B	Unstandardized Coefficients Std. Error	Standardized Coefficients Beta	t	Sig.
1	(Constant)	3.630	.086		42.158	.000
	Ideology0	-.454	.057	-.504	-7.954	.000
2	(Constant)	3.715	.091		41.019	.000
	Ideology0	-.392	.057	-.435	-6.892	.000
	GenderCC	-.355	.084	-.261	-4.209	.000
	RaceCC	-.211	.094	-.142	-2.239	.026
	SES0	-.072	.113	-.039	-.631	.529
3	(Constant)	3.683	.091		40.561	.000
	Ideology0	-.363	.058	-.403	-6.283	.000
	GenderCC	-.360	.083	-.265	-4.315	.000
	RaceCC	-.133	.100	-.090	-1.341	.182
	SES0	-.053	.113	-.028	-.468	.641
	National0	-.133	.061	-.148	-2.193	.030
4	(Constant)	3.684	.091		40.633	.000
	Ideology0	-.333	.063	-.370	-5.328	.000
	GenderCC	-.306	.094	-.225	-3.263	.001
	RaceCC	-.138	.099	-.093	-1.385	.168
	SES0	-.034	.113	-.018	-.299	.765
	National0	-.120	.061	-.134	-1.957	.052
	MRN0	-.153	.123	-.096	-1.247	.214
5	(Constant)	3.642	.104		35.028	.000
	Ideology0	-.312	.066	-.347	-4.766	.000
	GenderCC	-.305	.093	-.224	-3.263	.001
	RaceCC	-.195	.102	-.131	-1.906	.058
	SES0	-.036	.116	-.019	-.310	.757
	National0	-.127	.062	-.141	-2.051	.042
	MRN0	-.084	.129	-.053	-.654	.514
	MRN0xRace	-.232	.116	-.145	-1.996	.047
	MRN0xSES0	.065	.159	.026	.408	.684
	MRN0xGender	-.024	.109	-.013	-.221	.825
	MRN0xIdeology0	.141	.074	.131	1.899	.059
	MRN0xNational0	-.008	.066	-.008	-.121	.904

Coefficients[a]

Model		Correlations		
		Zero-order	Partial	Part
1	(Constant)			
	Ideology0	-.504	-.504	-.504
2	(Constant)			
	Ideology0	-.504	-.454	-.417
	GenderCC	-.295	-.297	-.255
	RaceCC	-.209	-.163	-.135
	SES0	-.116	-.047	-.038
3	(Constant)			
	Ideology0	-.504	-.422	-.376
	GenderCC	-.295	-.305	-.258
	RaceCC	-.209	-.099	-.080
	SES0	-.116	-.035	-.028
	National0	-.298	-.160	-.131
4	(Constant)			
	Ideology0	-.504	-.368	-.318
	GenderCC	-.295	-.236	-.195
	RaceCC	-.209	-.102	-.083
	SES0	-.116	-.022	-.018
	National0	-.298	-.144	-.117
	MRN0	-.405	-.092	-.075
5	(Constant)			
	Ideology0	-.504	-.338	-.283
	GenderCC	-.295	-.239	-.194
	RaceCC	-.209	-.142	-.113
	SES0	-.116	-.023	-.018
	National0	-.298	-.153	-.122
	MRN0	-.405	-.049	-.039
	MRN0xRace	-.288	-.149	-.119
	MRN0xSES0	-.016	.031	.024
	MRN0xGender	.063	-.017	-.013
	MRN0xIdeology0	.024	.142	.113
	MRN0xNational0	.027	-.009	-.007

a. Dependent Variable: Concern_Tot

Excluded Variables[a]

Model		Beta In	t	Sig.	Partial Correlation	Collinearity Statistics Tolerance
1	GenderCC	-.238[b]	-3.877	.000	-.274	.986
	RaceCC	-.098[b]	-1.514	.132	-.111	.947
	SES0	-.060[b]	-.933	.352	-.068	.987
	National0	-.161[b]	-2.457	.015	-.178	.909
	MRN0	-.225[b]	-3.260	.001	-.233	.800
	MRN0xRace	-.118[b]	-1.747	.082	-.127	.864
	MRN0xSES0	.045[b]	.701	.484	.051	.986
	MRN0xGender	.022[b]	.345	.730	.025	.993
	MRN0xIdeology0	.097[b]	1.525	.129	.111	.980
	MRN0xNational0	.048[b]	.758	.449	.056	.998
2	National0	-.148[c]	-2.193	.030	-.160	.782
	MRN0	-.121[c]	-1.585	.115	-.117	.621
	MRN0xRace	-.109[c]	-1.658	.099	-.122	.841
	MRN0xSES0	.036[c]	.580	.563	.043	.943
	MRN0xGender	.019[c]	.310	.757	.023	.991
	MRN0xIdeology0	.107[c]	1.721	.087	.127	.937
	MRN0xNational0	.012[c]	.203	.839	.015	.976
3	MRN0	-.096[d]	-1.247	.214	-.092	.603
	MRN0xRace	-.117[d]	-1.803	.073	-.133	.838
	MRN0xSES0	.042[d]	.677	.499	.050	.941
	MRN0xGender	.007[d]	.110	.912	.008	.983
	MRN0xIdeology0	.097[d]	1.576	.117	.116	.932
	MRN0xNational0	.003[d]	.041	.967	.003	.970
4	MRN0xRace	-.106[e]	-1.600	.111	-.118	.810
	MRN0xSES0	.037[e]	.599	.550	.045	.937
	MRN0xGender	.010[e]	.169	.866	.013	.981
	MRN0xIdeology0	.093[e]	1.497	.136	.111	.927
	MRN0xNational0	-.010[e]	-.157	.875	-.012	.946

a. Dependent Variable: Concern_Tot

b. Predictors in the Model: (Constant), Ideology0

c. Predictors in the Model: (Constant), Ideology0, SES0, GenderCC, RaceCC

d. Predictors in the Model: (Constant), Ideology0, SES0, GenderCC, RaceCC, National0

e. Predictors in the Model: (Constant), Ideology0, SES0, GenderCC, RaceCC, National0, MRN0

```
REGRESSION
 /MISSING LISTWISE
 /STATISTICS COEFF OUTS R ANOVA CHANGE ZPP
 /CRITERIA=PIN(.05) POUT(.10)
 /NOORIGIN
 /DEPENDENT Finance_Tot
 /METHOD=ENTER Ideology0
 /METHOD=ENTER GenderCC RaceCC SES0
  /METHOD=ENTER National0
 /METHOD=ENTER MRN0
 /METHOD=ENTER MRN0xRace MRN0xSES0 MRN0xGender MRN0xIdeology0 MRN0xNational0.
```

Regression

Notes

Output Created		15-DEC-2021 13:09:41
Comments		
Input	Data	C:\Users\njs5478\Dropbox\HM and COVID\0. Revise and Resubmit\2. R and R Data\Study 2a\Study2a_Data.sav
	Active Dataset	DataSet1
	Filter	<none>
	Weight	<none>
	Split File	<none>
	N of Rows in Working Data File	188
Missing Value Handling	Definition of Missing	User-defined missing values are treated as missing.
	Cases Used	Statistics are based on cases with no missing values for any variable used.

Notes

Syntax		REGRESSION<br>/MISSING LISTWISE<br>/STATISTICS COEFF OUTS R ANOVA CHANGE ZPP<br>/CRITERIA=PIN(.05) POUT(.10)<br>/NOORIGIN<br>/DEPENDENT Finance_Tot<br>/METHOD=ENTER Ideology0<br>/METHOD=ENTER GenderCC RaceCC SES0<br>/METHOD=ENTER National0<br>/METHOD=ENTER MRN0<br>/METHOD=ENTER MRN0xRace MRN0xSES0 MRN0xGender MRN0xIdeology0 MRN0xNational0.
Resources	Processor Time	00:00:00.02
	Elapsed Time	00:00:00.03
	Memory Required	35200 bytes
	Additional Memory Required for Residual Plots	0 bytes

Variables Entered/Removed[a]

Model	Variables Entered	Variables Removed	Method
1	Ideology0[b]	.	Enter
2	SES0, GenderCC, RaceCC[b]	.	Enter
3	National0[b]	.	Enter
4	MRN0[b]	.	Enter
5	MRN0xGender, MRN0xNational0, MRN0xSES0, MRN0xIdeology0, MRN0xRace[b]	.	Enter

a. Dependent Variable: Finance_Tot

b. All requested variables entered.

Model Summary

Model	R	R Square	Adjusted R Square	Std. Error of the Estimate	Change Statistics		
					R Square Change	F Change	df1
1	.080[a]	.006	.001	1.46100	.006	1.193	1
2	.357[b]	.128	.108	1.38021	.121	8.471	3
3	.360[c]	.129	.105	1.38253	.002	.386	1
4	.364[d]	.132	.104	1.38386	.003	.650	1
5	.373[e]	.139	.085	1.39814	.006	.264	5

Model Summary

Model	Change Statistics	
	df2	Sig. F Change
1	186	.276
2	183	.000
3	182	.535
4	181	.421
5	176	.932

a. Predictors: (Constant), Ideology0

b. Predictors: (Constant), Ideology0, SES0, GenderCC, RaceCC

c. Predictors: (Constant), Ideology0, SES0, GenderCC, RaceCC, National0

d. Predictors: (Constant), Ideology0, SES0, GenderCC, RaceCC, National0, MRN0

e. Predictors: (Constant), Ideology0, SES0, GenderCC, RaceCC, National0, MRN0, MRN0xGender, MRN0xNational0, MRN0xSES0, MRN0xIdeology0, MRN0xRace

ANOVA[a]

Model		Sum of Squares	df	Mean Square	F	Sig.
1	Regression	2.546	1	2.546	1.193	.276[b]
	Residual	397.021	186	2.135		
	Total	399.567	187			
2	Regression	50.957	4	12.739	6.687	.000[c]
	Residual	348.610	183	1.905		
	Total	399.567	187			
3	Regression	51.695	5	10.339	5.409	.000[d]
	Residual	347.872	182	1.911		
	Total	399.567	187			
4	Regression	52.940	6	8.823	4.607	.000[e]
	Residual	346.627	181	1.915		
	Total	399.567	187			
5	Regression	55.525	11	5.048	2.582	.005[f]
	Residual	344.043	176	1.955		
	Total	399.567	187			

a. Dependent Variable: Finance_Tot

b. Predictors: (Constant), Ideology0

c. Predictors: (Constant), Ideology0, SES0, GenderCC, RaceCC

d. Predictors: (Constant), Ideology0, SES0, GenderCC, RaceCC, National0

e. Predictors: (Constant), Ideology0, SES0, GenderCC, RaceCC, National0, MRN0

f. Predictors: (Constant), Ideology0, SES0, GenderCC, RaceCC, National0, MRN0, MRN0xGender, MRN0xNational0, MRN0xSES0, MRN0xIdeology0, MRN0xRace

Coefficients[a]

Model		Unstandardized Coefficients B	Unstandardized Coefficients Std. Error	Standardized Coefficients Beta	t	Sig.
1	(Constant)	3.770	.107		35.379	.000
	Ideology0	-.077	.071	-.080	-1.092	.276
2	(Constant)	3.803	.111		34.301	.000
	Ideology0	-.018	.070	-.019	-.264	.792
	GenderCC	-.146	.103	-.100	-1.417	.158
	RaceCC	-.082	.115	-.051	-.710	.479
	SES0	-.651	.139	-.327	-4.685	.000
3	(Constant)	3.792	.113		33.704	.000
	Ideology0	-.008	.072	-.009	-.115	.908
	GenderCC	-.148	.103	-.102	-1.432	.154
	RaceCC	-.055	.123	-.034	-.443	.658
	SES0	-.644	.140	-.323	-4.615	.000
	National0	-.047	.075	-.049	-.622	.535
4	(Constant)	3.793	.113		33.678	.000
	Ideology0	.016	.078	.016	.204	.839
	GenderCC	-.105	.117	-.072	-.899	.370
	RaceCC	-.058	.124	-.036	-.470	.639
	SES0	-.629	.141	-.316	-4.463	.000
	National0	-.036	.076	-.038	-.477	.634
	MRN0	-.123	.152	-.072	-.806	.421
5	(Constant)	3.793	.131		28.913	.000
	Ideology0	.002	.083	.002	.028	.977
	GenderCC	-.106	.118	-.072	-.896	.372
	RaceCC	-.082	.129	-.051	-.632	.528
	SES0	-.629	.147	-.316	-4.277	.000
	National0	-.040	.078	-.041	-.510	.611
	MRN0	-.107	.162	-.062	-.657	.512
	MRN0xRace	-.009	.147	-.005	-.063	.950
	MRN0xSES0	.062	.200	.023	.307	.759
	MRN0xGender	-.100	.138	-.052	-.728	.468
	MRN0xIdeology0	.079	.094	.069	.846	.399
	MRN0xNational0	-.011	.083	-.011	-.135	.893

Coefficients[a]

Model		Correlations Zero-order	Partial	Part
1	(Constant)			
	Ideology0	-.080	-.080	-.080
2	(Constant)			
	Ideology0	-.080	-.020	-.018
	GenderCC	-.114	-.104	-.098
	RaceCC	-.071	-.052	-.049
	SES0	-.339	-.327	-.323
3	(Constant)			
	Ideology0	-.080	-.009	-.008
	GenderCC	-.114	-.106	-.099
	RaceCC	-.071	-.033	-.031
	SES0	-.339	-.324	-.319
	National0	-.100	-.046	-.043
4	(Constant)			
	Ideology0	-.080	.015	.014
	GenderCC	-.114	-.067	-.062
	RaceCC	-.071	-.035	-.033
	SES0	-.339	-.315	-.309
	National0	-.100	-.035	-.033
	MRN0	-.167	-.060	-.056
5	(Constant)			
	Ideology0	-.080	.002	.002
	GenderCC	-.114	-.067	-.063
	RaceCC	-.071	-.048	-.044
	SES0	-.339	-.307	-.299
	National0	-.100	-.038	-.036
	MRN0	-.167	-.049	-.046
	MRN0xRace	-.044	-.005	-.004
	MRN0xSES0	.090	.023	.021
	MRN0xGender	-.038	-.055	-.051
	MRN0xIdeology0	.023	.064	.059
	MRN0xNational0	.023	-.010	-.009

a. Dependent Variable: Finance_Tot

Excluded Variables[a]

Model		Beta In	t	Sig.	Partial Correlation	Collinearity Statistics Tolerance
1	GenderCC	-.106[b]	-1.448	.149	-.106	.986
	RaceCC	-.056[b]	-.739	.461	-.054	.947
	SES0	-.335[b]	-4.812	.000	-.334	.987
	National0	-.084[b]	-1.096	.274	-.080	.909
	MRN0	-.164[b]	-2.023	.045	-.147	.800
	MRN0xRace	-.017[b]	-.217	.829	-.016	.864
	MRN0xSES0	.101[b]	1.375	.171	.101	.986
	MRN0xGender	-.044[b]	-.605	.546	-.044	.993
	MRN0xIdeology0	.035[b]	.467	.641	.034	.980
	MRN0xNational0	.027[b]	.366	.715	.027	.998
2	National0	-.049[c]	-.622	.535	-.046	.782
	MRN0	-.079[c]	-.901	.369	-.067	.621
	MRN0xRace	.007[c]	.087	.931	.006	.841
	MRN0xSES0	.033[c]	.469	.640	.035	.943
	MRN0xGender	-.040[c]	-.578	.564	-.043	.991
	MRN0xIdeology0	.067[c]	.941	.348	.070	.937
	MRN0xNational0	.026[c]	.368	.714	.027	.976
3	MRN0	-.072[d]	-.806	.421	-.060	.603
	MRN0xRace	.004[d]	.053	.958	.004	.838
	MRN0xSES0	.035[d]	.494	.622	.037	.941
	MRN0xGender	-.045[d]	-.638	.524	-.047	.983
	MRN0xIdeology0	.064[d]	.895	.372	.066	.932
	MRN0xNational0	.023[d]	.321	.748	.024	.970
4	MRN0xRace	.016[e]	.203	.839	.015	.810
	MRN0xSES0	.032[e]	.443	.659	.033	.937
	MRN0xGender	-.042[e]	-.600	.549	-.045	.981
	MRN0xIdeology0	.061[e]	.842	.401	.063	.927
	MRN0xNational0	.014[e]	.196	.845	.015	.946

a. Dependent Variable: Finance_Tot

b. Predictors in the Model: (Constant), Ideology0

c. Predictors in the Model: (Constant), Ideology0, SES0, GenderCC, RaceCC

d. Predictors in the Model: (Constant), Ideology0, SES0, GenderCC, RaceCC, National0

e. Predictors in the Model: (Constant), Ideology0, SES0, GenderCC, RaceCC, National0, MRN0

```
REGRESSION
 /MISSING LISTWISE
 /STATISTICS COEFF OUTS R ANOVA CHANGE ZPP
 /CRITERIA=PIN(.05) POUT(.10)
 /NOORIGIN
 /DEPENDENT Psychology_Tot
 /METHOD=ENTER Ideology0
 /METHOD=ENTER GenderCC RaceCC SES0
  /METHOD=ENTER National0
 /METHOD=ENTER MRN0
 /METHOD=ENTER MRN0xRace MRN0xSES0 MRN0xGender MRN0xIdeology0 MRN0xNational0.
```

Regression

Notes

Output Created		15-DEC-2021 13:09:41
Comments		
Input	Data	C: \Users\njs5478\Dropbox\HM and COVID\0. Revise and Resubmit\2. R and R Data\Study 2a\Study2a_Data.sav
	Active Dataset	DataSet1
	Filter	<none>
	Weight	<none>
	Split File	<none>
	N of Rows in Working Data File	188
Missing Value Handling	Definition of Missing	User-defined missing values are treated as missing.
	Cases Used	Statistics are based on cases with no missing values for any variable used.

Notes

Syntax		REGRESSION<br>/MISSING LISTWISE<br>/STATISTICS COEFF OUTS R ANOVA CHANGE ZPP<br>/CRITERIA=PIN(.05) POUT(.10)<br>/NOORIGIN<br>/DEPENDENT Psychology_Tot<br>/METHOD=ENTER Ideology0<br>/METHOD=ENTER GenderCC RaceCC SES0<br>/METHOD=ENTER National0<br>/METHOD=ENTER MRN0<br>/METHOD=ENTER MRN0xRace MRN0xSES0 MRN0xGender MRN0xIdeology0 MRN0xNational0.
Resources	Processor Time	00:00:00.02
	Elapsed Time	00:00:00.02
	Memory Required	35200 bytes
	Additional Memory Required for Residual Plots	0 bytes

Variables Entered/Removed[a]

Model	Variables Entered	Variables Removed	Method
1	Ideology0[b]	.	Enter
2	SES0, GenderCC, RaceCC[b]	.	Enter
3	National0[b]	.	Enter
4	MRN0[b]	.	Enter
5	MRN0xGender, MRN0xNational0, MRN0xSES0, MRN0xIdeology0, MRN0xRace[b]	.	Enter

a. Dependent Variable: Psychology_Tot

b. All requested variables entered.

Model Summary

Model	R	R Square	Adjusted R Square	Std. Error of the Estimate	Change Statistics		
					R Square Change	F Change	df1
1	.172[a]	.030	.024	1.40882	.030	5.692	1
2	.214[b]	.046	.025	1.40838	.016	1.039	3
3	.215[c]	.046	.020	1.41206	.000	.048	1
4	.245[d]	.060	.029	1.40569	.014	2.653	1
5	.348[e]	.121	.066	1.37854	.061	2.440	5

Model Summary

Model	Change Statistics	
	df2	Sig. F Change
1	186	.018
2	183	.377
3	182	.827
4	181	.105
5	176	.036

a. Predictors: (Constant), Ideology0

b. Predictors: (Constant), Ideology0, SES0, GenderCC, RaceCC

c. Predictors: (Constant), Ideology0, SES0, GenderCC, RaceCC, National0

d. Predictors: (Constant), Ideology0, SES0, GenderCC, RaceCC, National0, MRN0

e. Predictors: (Constant), Ideology0, SES0, GenderCC, RaceCC, National0, MRN0, MRN0xGender, MRN0xNational0, MRN0xSES0, MRN0xIdeology0, MRN0xRace

ANOVA[a]

Model		Sum of Squares	df	Mean Square	F	Sig.
1	Regression	11.297	1	11.297	5.692	.018[b]
	Residual	369.168	186	1.985		
	Total	380.465	187			
2	Regression	17.478	4	4.369	2.203	.070[c]
	Residual	362.987	183	1.984		
	Total	380.465	187			
3	Regression	17.573	5	3.515	1.763	.123[d]
	Residual	362.892	182	1.994		
	Total	380.465	187			
4	Regression	22.815	6	3.802	1.924	.079[e]
	Residual	357.651	181	1.976		
	Total	380.465	187			
5	Regression	45.999	11	4.182	2.200	.016[f]
	Residual	334.466	176	1.900		
	Total	380.465	187			

a. Dependent Variable: Psychology_Tot

b. Predictors: (Constant), Ideology0

c. Predictors: (Constant), Ideology0, SES0, GenderCC, RaceCC

d. Predictors: (Constant), Ideology0, SES0, GenderCC, RaceCC, National0

e. Predictors: (Constant), Ideology0, SES0, GenderCC, RaceCC, National0, MRN0

f. Predictors: (Constant), Ideology0, SES0, GenderCC, RaceCC, National0, MRN0, MRN0xGender, MRN0xNational0, MRN0xSES0, MRN0xIdeology0, MRN0xRace

Coefficients[a]

Model		Unstandardized Coefficients B	Unstandardized Coefficients Std. Error	Standardized Coefficients Beta	t	Sig.
1	(Constant)	4.541	.103		44.198	.000
	Ideology0	-.162	.068	-.172	-2.386	.018
2	(Constant)	4.514	.113		39.897	.000
	Ideology0	-.155	.071	-.165	-2.184	.030
	GenderCC	-.149	.105	-.105	-1.420	.157
	RaceCC	.067	.118	.043	.573	.567
	SES0	-.085	.142	-.044	-.603	.547
3	(Constant)	4.518	.115		39.317	.000
	Ideology0	-.159	.073	-.169	-2.171	.031
	GenderCC	-.149	.106	-.105	-1.409	.160
	RaceCC	.058	.126	.037	.457	.648
	SES0	-.088	.142	-.045	-.616	.539
	National0	.017	.077	.018	.218	.827
4	(Constant)	4.520	.114		39.509	.000
	Ideology0	-.109	.079	-.116	-1.386	.167
	GenderCC	-.060	.118	-.042	-.507	.613
	RaceCC	.051	.125	.033	.404	.687
	SES0	-.057	.143	-.029	-.398	.691
	National0	.038	.077	.040	.489	.626
	MRN0	-.252	.155	-.151	-1.629	.105
5	(Constant)	4.364	.129		33.738	.000
	Ideology0	-.097	.082	-.102	-1.184	.238
	GenderCC	-.046	.116	-.033	-.399	.690
	RaceCC	-.002	.127	-.001	-.012	.990
	SES0	-.087	.145	-.045	-.600	.549
	National0	.044	.077	.047	.577	.565
	MRN0	-.119	.160	-.071	-.744	.458
	MRN0xRace	-.311	.145	-.186	-2.150	.033
	MRN0xSES0	.064	.197	.024	.323	.747
	MRN0xGender	.110	.136	.058	.809	.420
	MRN0xIdeology0	.172	.092	.153	1.866	.064
	MRN0xNational0	.143	.082	.143	1.738	.084

Coefficients[a]

Model		Correlations Zero-order	Partial	Part
1	(Constant)			
	Ideology0	-.172	-.172	-.172
2	(Constant)			
	Ideology0	-.172	-.159	-.158
	GenderCC	-.133	-.104	-.103
	RaceCC	.016	.042	.041
	SES0	-.065	-.045	-.044
3	(Constant)			
	Ideology0	-.172	-.159	-.157
	GenderCC	-.133	-.104	-.102
	RaceCC	.016	.034	.033
	SES0	-.065	-.046	-.045
	National0	-.018	.016	.016
4	(Constant)			
	Ideology0	-.172	-.102	-.100
	GenderCC	-.133	-.038	-.037
	RaceCC	.016	.030	.029
	SES0	-.065	-.030	-.029
	National0	-.018	.036	.035
	MRN0	-.216	-.120	-.117
5	(Constant)			
	Ideology0	-.172	-.089	-.084
	GenderCC	-.133	-.030	-.028
	RaceCC	.016	-.001	-.001
	SES0	-.065	-.045	-.042
	National0	-.018	.043	.041
	MRN0	-.216	-.056	-.053
	MRN0xRace	-.167	-.160	-.152
	MRN0xSES0	.049	.024	.023
	MRN0xGender	.091	.061	.057
	MRN0xIdeology0	.153	.139	.132
	MRN0xNational0	.144	.130	.123

a. Dependent Variable: Psychology_Tot

Excluded Variables[a]

Model		Beta In	t	Sig.	Partial Correlation	Collinearity Statistics Tolerance
1	GenderCC	-.114[b]	-1.578	.116	-.115	.986
	RaceCC	.059[b]	.792	.429	.058	.947
	SES0	-.046[b]	-.631	.529	-.046	.987
	National0	.037[b]	.483	.630	.036	.909
	MRN0	-.174[b]	-2.175	.031	-.158	.800
	MRN0xRace	-.119[b]	-1.539	.126	-.112	.864
	MRN0xSES0	.071[b]	.974	.331	.071	.986
	MRN0xGender	.077[b]	1.062	.290	.078	.993
	MRN0xIdeology0	.181[b]	2.512	.013	.182	.980
	MRN0xNational0	.152[b]	2.116	.036	.154	.998
2	National0	.018[c]	.218	.827	.016	.782
	MRN0	-.144[c]	-1.572	.118	-.116	.621
	MRN0xRace	-.102[c]	-1.295	.197	-.096	.841
	MRN0xSES0	.064[c]	.862	.390	.064	.943
	MRN0xGender	.080[c]	1.103	.272	.081	.991
	MRN0xIdeology0	.175[c]	2.374	.019	.173	.937
	MRN0xNational0	.149[c]	2.061	.041	.151	.976
3	MRN0	-.151[d]	-1.629	.105	-.120	.603
	MRN0xRace	-.101[d]	-1.282	.202	-.095	.838
	MRN0xSES0	.064[d]	.851	.396	.063	.941
	MRN0xGender	.082[d]	1.125	.262	.083	.983
	MRN0xIdeology0	.177[d]	2.392	.018	.175	.932
	MRN0xNational0	.151[d]	2.079	.039	.153	.970
4	MRN0xRace	-.080[e]	-1.005	.316	-.075	.810
	MRN0xSES0	.056[e]	.751	.453	.056	.937
	MRN0xGender	.088[e]	1.209	.228	.090	.981
	MRN0xIdeology0	.170[e]	2.295	.023	.169	.927
	MRN0xNational0	.136[e]	1.847	.066	.136	.946

a. Dependent Variable: Psychology_Tot

b. Predictors in the Model: (Constant), Ideology0

c. Predictors in the Model: (Constant), Ideology0, SES0, GenderCC, RaceCC

d. Predictors in the Model: (Constant), Ideology0, SES0, GenderCC, RaceCC, National0

e. Predictors in the Model: (Constant), Ideology0, SES0, GenderCC, RaceCC, National0, MRN0

```
REGRESSION
 /MISSING LISTWISE
 /STATISTICS COEFF OUTS R ANOVA CHANGE ZPP
 /CRITERIA=PIN(.05) POUT(.10)
 /NOORIGIN
 /DEPENDENT Risk_Rules
 /METHOD=ENTER Ideology0
 /METHOD=ENTER GenderCC RaceCC SES0
  /METHOD=ENTER National0
 /METHOD=ENTER MRN0
 /METHOD=ENTER MRN0xRace MRN0xSES0 MRN0xGender MRN0xIdeology0 MRN0xNational0.
```

Regression

Notes

Output Created		15-DEC-2021 13:09:41
Comments		
Input	Data	C:\Users\njs5478\Dropbox\HM and COVID\0. Revise and Resubmit\2. R and R Data\Study 2a\Study2a_Data.sav
	Active Dataset	DataSet1
	Filter	<none>
	Weight	<none>
	Split File	<none>
	N of Rows in Working Data File	188
Missing Value Handling	Definition of Missing	User-defined missing values are treated as missing.
	Cases Used	Statistics are based on cases with no missing values for any variable used.

Notes

Syntax		REGRESSION<br>/MISSING LISTWISE<br>/STATISTICS COEFF OUTS R ANOVA CHANGE ZPP<br>/CRITERIA=PIN(.05) POUT(.10)<br>/NOORIGIN<br>/DEPENDENT Risk_Rules<br>/METHOD=ENTER Ideology0<br>/METHOD=ENTER GenderCC RaceCC SES0<br>/METHOD=ENTER National0<br>/METHOD=ENTER MRN0<br>/METHOD=ENTER MRN0xRace MRN0xSES0 MRN0xGender MRN0xIdeology0 MRN0xNational0.
Resources	Processor Time	00:00:00.02
	Elapsed Time	00:00:00.02
	Memory Required	35200 bytes
	Additional Memory Required for Residual Plots	0 bytes

Variables Entered/Removed[a]

Model	Variables Entered	Variables Removed	Method
1	Ideology0[b]	.	Enter
2	SES0, GenderCC, RaceCC[b]	.	Enter
3	National0[b]	.	Enter
4	MRN0[b]	.	Enter
5	MRN0xGender, MRN0xNational0, MRN0xSES0, MRN0xIdeology0, MRN0xRace[b]	.	Enter

a. Dependent Variable: Risk_Rules

b. All requested variables entered.

Model Summary

Model	R	R Square	Adjusted R Square	Std. Error of the Estimate	Change Statistics		
					R Square Change	F Change	df1
1	.528[a]	.279	.275	.91470	.279	72.077	1
2	.592[b]	.351	.336	.87535	.071	6.699	3
3	.610[c]	.372	.355	.86323	.021	6.173	1
4	.647[d]	.418	.399	.83313	.046	14.390	1
5	.652[e]	.425	.390	.83960	.007	.444	5

Model Summary

Model	Change Statistics	
	df2	Sig. F Change
1	186	.000
2	183	.000
3	182	.014
4	181	.000
5	176	.817

a. Predictors: (Constant), Ideology0

b. Predictors: (Constant), Ideology0, SES0, GenderCC, RaceCC

c. Predictors: (Constant), Ideology0, SES0, GenderCC, RaceCC, National0

d. Predictors: (Constant), Ideology0, SES0, GenderCC, RaceCC, National0, MRN0

e. Predictors: (Constant), Ideology0, SES0, GenderCC, RaceCC, National0, MRN0, MRN0xGender, MRN0xNational0, MRN0xSES0, MRN0xIdeology0, MRN0xRace

ANOVA[a]

Model		Sum of Squares	df	Mean Square	F	Sig.
1	Regression	60.305	1	60.305	72.077	.000[b]
	Residual	155.621	186	.837		
	Total	215.926	187			
2	Regression	75.705	4	18.926	24.700	.000[c]
	Residual	140.221	183	.766		
	Total	215.926	187			
3	Regression	80.305	5	16.061	21.553	.000[d]
	Residual	135.621	182	.745		
	Total	215.926	187			
4	Regression	90.293	6	15.049	21.681	.000[e]
	Residual	125.633	181	.694		
	Total	215.926	187			
5	Regression	91.859	11	8.351	11.846	.000[f]
	Residual	124.068	176	.705		
	Total	215.926	187			

a. Dependent Variable: Risk_Rules

b. Predictors: (Constant), Ideology0

c. Predictors: (Constant), Ideology0, SES0, GenderCC, RaceCC

d. Predictors: (Constant), Ideology0, SES0, GenderCC, RaceCC, National0

e. Predictors: (Constant), Ideology0, SES0, GenderCC, RaceCC, National0, MRN0

f. Predictors: (Constant), Ideology0, SES0, GenderCC, RaceCC, National0, MRN0, MRN0xGender, MRN0xNational0, MRN0xSES0, MRN0xIdeology0, MRN0xRace

Coefficients[a]

Model		Unstandardized Coefficients B	Unstandardized Coefficients Std. Error	Standardized Coefficients Beta	t	Sig.
1	(Constant)	3.924	.067		58.826	.000
	Ideology0	.375	.044	.528	8.490	.000
2	(Constant)	3.838	.070		54.574	.000
	Ideology0	.320	.044	.451	7.251	.000
	GenderCC	.190	.065	.177	2.907	.004
	RaceCC	.214	.073	.183	2.930	.004
	SES0	.179	.088	.122	2.032	.044
3	(Constant)	3.866	.070		55.027	.000
	Ideology0	.295	.045	.416	6.596	.000
	GenderCC	.195	.065	.182	3.020	.003
	RaceCC	.146	.077	.125	1.901	.059
	SES0	.162	.087	.111	1.864	.064
	National0	.116	.047	.165	2.484	.014
4	(Constant)	3.863	.068		56.977	.000
	Ideology0	.227	.047	.319	4.847	.000
	GenderCC	.072	.070	.068	1.032	.303
	RaceCC	.156	.074	.133	2.098	.037
	SES0	.120	.085	.082	1.411	.160
	National0	.087	.046	.124	1.903	.059
	MRN0	.348	.092	.277	3.793	.000
5	(Constant)	3.869	.079		49.109	.000
	Ideology0	.217	.050	.305	4.363	.000
	GenderCC	.075	.071	.070	1.063	.289
	RaceCC	.177	.077	.151	2.278	.024
	SES0	.109	.088	.074	1.232	.220
	National0	.091	.047	.128	1.936	.055
	MRN0	.349	.097	.278	3.585	.000
	MRN0xRace	.049	.088	.039	.555	.579
	MRN0xSES0	-.049	.120	-.025	-.407	.684
	MRN0xGender	-.013	.083	-.009	-.161	.872
	MRN0xIdeology0	-.037	.056	-.043	-.655	.513
	MRN0xNational0	.053	.050	.070	1.057	.292

Coefficients[a]

Model		Correlations Zero-order	Partial	Part
1	(Constant)			
	Ideology0	.528	.528	.528
2	(Constant)			
	Ideology0	.528	.472	.432
	GenderCC	.213	.210	.173
	RaceCC	.273	.212	.175
	SES0	.200	.149	.121
3	(Constant)			
	Ideology0	.528	.439	.387
	GenderCC	.213	.218	.177
	RaceCC	.273	.140	.112
	SES0	.200	.137	.110
	National0	.347	.181	.146
4	(Constant)			
	Ideology0	.528	.339	.275
	GenderCC	.213	.076	.059
	RaceCC	.273	.154	.119
	SES0	.200	.104	.080
	National0	.347	.140	.108
	MRN0	.503	.271	.215
5	(Constant)			
	Ideology0	.528	.312	.249
	GenderCC	.213	.080	.061
	RaceCC	.273	.169	.130
	SES0	.200	.092	.070
	National0	.347	.144	.111
	MRN0	.503	.261	.205
	MRN0xRace	.269	.042	.032
	MRN0xSES0	.007	-.031	-.023
	MRN0xGender	-.067	-.012	-.009
	MRN0xIdeology0	.065	-.049	-.037
	MRN0xNational0	.013	.079	.060

a. Dependent Variable: Risk_Rules

Excluded Variables[a]

Model		Beta In	t	Sig.	Partial Correlation	Collinearity Statistics Tolerance
1	GenderCC	.152[b]	2.456	.015	.178	.986
	RaceCC	.160[b]	2.530	.012	.183	.947
	SES0	.142[b]	2.294	.023	.166	.987
	National0	.206[b]	3.239	.001	.232	.909
	MRN0	.334[b]	5.110	.000	.352	.800
	MRN0xRace	.085[b]	1.275	.204	.093	.864
	MRN0xSES0	-.057[b]	-.907	.366	-.067	.986
	MRN0xGender	-.024[b]	-.383	.702	-.028	.993
	MRN0xIdeology0	-.010[b]	-.151	.880	-.011	.980
	MRN0xNational0	-.010[b]	-.154	.878	-.011	.998
2	National0	.165[c]	2.484	.014	.181	.782
	MRN0	.300[c]	4.141	.000	.293	.621
	MRN0xRace	.085[c]	1.309	.192	.097	.841
	MRN0xSES0	-.031[c]	-.501	.617	-.037	.943
	MRN0xGender	-.020[c]	-.340	.734	-.025	.991
	MRN0xIdeology0	-.039[c]	-.632	.528	-.047	.937
	MRN0xNational0	.018[c]	.302	.763	.022	.976
3	MRN0	.277[d]	3.793	.000	.271	.603
	MRN0xRace	.094[d]	1.469	.144	.109	.838
	MRN0xSES0	-.037[d]	-.612	.541	-.045	.941
	MRN0xGender	-.007[d]	-.116	.908	-.009	.983
	MRN0xIdeology0	-.028[d]	-.456	.649	-.034	.932
	MRN0xNational0	.030[d]	.494	.622	.037	.970
4	MRN0xRace	.053[e]	.839	.403	.062	.810
	MRN0xSES0	-.023[e]	-.390	.697	-.029	.937
	MRN0xGender	-.017[e]	-.298	.766	-.022	.981
	MRN0xIdeology0	-.013[e]	-.221	.825	-.016	.927
	MRN0xNational0	.066[e]	1.128	.261	.084	.946

a. Dependent Variable: Risk_Rules

b. Predictors in the Model: (Constant), Ideology0

c. Predictors in the Model: (Constant), Ideology0, SES0, GenderCC, RaceCC

d. Predictors in the Model: (Constant), Ideology0, SES0, GenderCC, RaceCC, National0

e. Predictors in the Model: (Constant), Ideology0, SES0, GenderCC, RaceCC, National0, MRN0

```
REGRESSION
 /MISSING LISTWISE
 /STATISTICS COEFF OUTS R ANOVA CHANGE ZPP
 /CRITERIA=PIN(.05) POUT(.10)
 /NOORIGIN
 /DEPENDENT Mandate_Tot
 /METHOD=ENTER Ideology0
 /METHOD=ENTER GenderCC RaceCC SES0
  /METHOD=ENTER National0
 /METHOD=ENTER MRN0
 /METHOD=ENTER MRN0xRace MRN0xSES0 MRN0xGender MRN0xIdeology0 MRN0xNational0.
```

Regression

Notes

Output Created		15-DEC-2021 13:09:41
Comments		
Input	Data	C: \Users\njs5478\Dropbox\HM and COVID\0. Revise and Resubmit\2. R and R Data\Study 2a\Study2a_Data.sav
	Active Dataset	DataSet1
	Filter	<none>
	Weight	<none>
	Split File	<none>
	N of Rows in Working Data File	188
Missing Value Handling	Definition of Missing	User-defined missing values are treated as missing.
	Cases Used	Statistics are based on cases with no missing values for any variable used.

Notes

Syntax		REGRESSION<br>/MISSING LISTWISE<br>/STATISTICS COEFF OUTS R ANOVA CHANGE ZPP<br>/CRITERIA=PIN(.05) POUT(.10)<br>/NOORIGIN<br>/DEPENDENT Mandate_Tot<br>/METHOD=ENTER Ideology0<br>/METHOD=ENTER GenderCC RaceCC SES0<br>/METHOD=ENTER National0<br>/METHOD=ENTER MRN0<br>/METHOD=ENTER MRN0xRace MRN0xSES0 MRN0xGender MRN0xIdeology0 MRN0xNational0.
Resources	Processor Time	00:00:00.02
	Elapsed Time	00:00:00.02
	Memory Required	35200 bytes
	Additional Memory Required for Residual Plots	0 bytes

Variables Entered/Removed[a]

Model	Variables Entered	Variables Removed	Method
1	Ideology0[b]	.	Enter
2	SES0, GenderCC, RaceCC[b]	.	Enter
3	National0[b]	.	Enter
4	MRN0[b]	.	Enter
5	MRN0xGender, MRN0xNational0, MRN0xSES0, MRN0xIdeology0, MRN0xRace[b]	.	Enter

a. Dependent Variable: Mandate_Tot

b. All requested variables entered.

Model Summary

					Change Statistics		
Model	R	R Square	Adjusted R Square	Std. Error of the Estimate	R Square Change	F Change	df1
1	.591[a]	.349	.345	1.41970	.349	99.644	1
2	.626[b]	.392	.379	1.38249	.044	4.383	3
3	.638[c]	.408	.391	1.36884	.015	4.667	1
4	.648[d]	.420	.401	1.35812	.012	3.886	1
5	.652[e]	.426	.390	1.37063	.006	.342	5

Model Summary

	Change Statistics	
Model	df2	Sig. F Change
1	186	.000
2	183	.005
3	182	.032
4	181	.050
5	176	.887

a. Predictors: (Constant), Ideology0

b. Predictors: (Constant), Ideology0, SES0, GenderCC, RaceCC

c. Predictors: (Constant), Ideology0, SES0, GenderCC, RaceCC, National0

d. Predictors: (Constant), Ideology0, SES0, GenderCC, RaceCC, National0, MRN0

e. Predictors: (Constant), Ideology0, SES0, GenderCC, RaceCC, National0, MRN0, MRN0xGender, MRN0xNational0, MRN0xSES0, MRN0xIdeology0, MRN0xRace

ANOVA[a]

Model		Sum of Squares	df	Mean Square	F	Sig.
1	Regression	200.838	1	200.838	99.644	.000[b]
	Residual	374.894	186	2.016		
	Total	575.732	187			
2	Regression	225.970	4	56.493	29.558	.000[c]
	Residual	349.762	183	1.911		
	Total	575.732	187			
3	Regression	234.714	5	46.943	25.053	.000[d]
	Residual	341.018	182	1.874		
	Total	575.732	187			
4	Regression	241.881	6	40.314	21.856	.000[e]
	Residual	333.851	181	1.844		
	Total	575.732	187			
5	Regression	245.092	11	22.281	11.860	.000[f]
	Residual	330.640	176	1.879		
	Total	575.732	187			

a. Dependent Variable: Mandate_Tot

b. Predictors: (Constant), Ideology0

c. Predictors: (Constant), Ideology0, SES0, GenderCC, RaceCC

d. Predictors: (Constant), Ideology0, SES0, GenderCC, RaceCC, National0

e. Predictors: (Constant), Ideology0, SES0, GenderCC, RaceCC, National0, MRN0

f. Predictors: (Constant), Ideology0, SES0, GenderCC, RaceCC, National0, MRN0, MRN0xGender, MRN0xNational0, MRN0xSES0, MRN0xIdeology0, MRN0xRace

Coefficients[a]

Model		Unstandardized Coefficients B	Std. Error	Standardized Coefficients Beta	t	Sig.
1	(Constant)	4.679	.104		45.185	.000
	Ideology0	-.685	.069	-.591	-9.982	.000
2	(Constant)	4.767	.111		42.924	.000
	Ideology0	-.640	.070	-.552	-9.166	.000
	GenderCC	-.323	.103	-.184	-3.126	.002
	RaceCC	-.220	.115	-.115	-1.910	.058
	SES0	.206	.139	.086	1.482	.140
3	(Constant)	4.729	.111		42.450	.000
	Ideology0	-.605	.071	-.522	-8.525	.000
	GenderCC	-.329	.102	-.188	-3.219	.002
	RaceCC	-.127	.122	-.066	-1.041	.299
	SES0	.229	.138	.096	1.658	.099
	National0	-.161	.074	-.139	-2.160	.032
4	(Constant)	4.731	.111		42.802	.000
	Ideology0	-.547	.076	-.472	-7.171	.000
	GenderCC	-.226	.114	-.129	-1.973	.050
	RaceCC	-.135	.121	-.071	-1.116	.266
	SES0	.265	.138	.111	1.918	.057
	National0	-.136	.075	-.118	-1.817	.071
	MRN0	-.295	.150	-.144	-1.971	.050
5	(Constant)	4.760	.129		37.016	.000
	Ideology0	-.520	.081	-.449	-6.414	.000
	GenderCC	-.230	.116	-.132	-1.991	.048
	RaceCC	-.152	.127	-.080	-1.203	.231
	SES0	.280	.144	.117	1.942	.054
	National0	-.147	.076	-.127	-1.923	.056
	MRN0	-.269	.159	-.131	-1.693	.092
	MRN0xRace	-.146	.144	-.071	-1.015	.311
	MRN0xSES0	.029	.196	.009	.146	.884
	MRN0xGender	-.035	.135	-.015	-.260	.795
	MRN0xIdeology0	.006	.092	.004	.068	.946
	MRN0xNational0	-.029	.082	-.023	-.352	.725

Coefficients[a]

Model		Correlations		
		Zero-order	Partial	Part
1	(Constant)			
	Ideology0	-.591	-.591	-.591
2	(Constant)			
	Ideology0	-.591	-.561	-.528
	GenderCC	-.229	-.225	-.180
	RaceCC	-.209	-.140	-.110
	SES0	.003	.109	.085
3	(Constant)			
	Ideology0	-.591	-.534	-.486
	GenderCC	-.229	-.232	-.184
	RaceCC	-.209	-.077	-.059
	SES0	.003	.122	.095
	National0	-.303	-.158	-.123
4	(Constant)			
	Ideology0	-.591	-.470	-.406
	GenderCC	-.229	-.145	-.112
	RaceCC	-.209	-.083	-.063
	SES0	.003	.141	.109
	National0	-.303	-.134	-.103
	MRN0	-.425	-.145	-.112
5	(Constant)			
	Ideology0	-.591	-.435	-.366
	GenderCC	-.229	-.148	-.114
	RaceCC	-.209	-.090	-.069
	SES0	.003	.145	.111
	National0	-.303	-.143	-.110
	MRN0	-.425	-.127	-.097
	MRN0xRace	-.294	-.076	-.058
	MRN0xSES0	-.085	.011	.008
	MRN0xGender	.048	-.020	-.015
	MRN0xIdeology0	-.085	.005	.004
	MRN0xNational0	-.011	-.027	-.020

a. Dependent Variable: Mandate_Tot

Excluded Variables[a]

Model		Beta In	t	Sig.	Partial Correlation	Collinearity Statistics
						Tolerance
1	GenderCC	-.161[b]	-2.749	.007	-.198	.986
	RaceCC	-.077[b]	-1.264	.208	-.093	.947
	SES0	.071[b]	1.185	.237	.087	.987
	National0	-.138[b]	-2.246	.026	-.163	.909
	MRN0	-.202[b]	-3.119	.002	-.224	.800
	MRN0xRace	-.087[b]	-1.377	.170	-.101	.864
	MRN0xSES0	-.014[b]	-.238	.812	-.017	.986
	MRN0xGender	.000[b]	-.008	.994	-.001	.993
	MRN0xIdeology0	-.002[b]	-.028	.978	-.002	.980
	MRN0xNational0	.014[b]	.229	.819	.017	.998
2	National0	-.139[c]	-2.160	.032	-.158	.782
	MRN0	-.166[c]	-2.293	.023	-.168	.621
	MRN0xRace	-.090[c]	-1.431	.154	-.105	.841
	MRN0xSES0	.002[c]	.039	.969	.003	.943
	MRN0xGender	-.005[c]	-.091	.928	-.007	.991
	MRN0xIdeology0	-.006[c]	-.100	.920	-.007	.937
	MRN0xNational0	-.018[c]	-.303	.762	-.022	.976
3	MRN0	-.144[d]	-1.971	.050	-.145	.603
	MRN0xRace	-.097[d]	-1.569	.118	-.116	.838
	MRN0xSES0	.008[d]	.129	.897	.010	.941
	MRN0xGender	-.017[d]	-.293	.770	-.022	.983
	MRN0xIdeology0	-.016[d]	-.263	.793	-.020	.932
	MRN0xNational0	-.027[d]	-.469	.640	-.035	.970
4	MRN0xRace	-.078[e]	-1.239	.217	-.092	.810
	MRN0xSES0	.000[e]	.003	.998	.000	.937
	MRN0xGender	-.012[e]	-.203	.839	-.015	.981
	MRN0xIdeology0	-.023[e]	-.397	.692	-.030	.927
	MRN0xNational0	-.046[e]	-.795	.428	-.059	.946

a. Dependent Variable: Mandate_Tot

b. Predictors in the Model: (Constant), Ideology0

c. Predictors in the Model: (Constant), Ideology0, SES0, GenderCC, RaceCC

d. Predictors in the Model: (Constant), Ideology0, SES0, GenderCC, RaceCC, National0

e. Predictors in the Model: (Constant), Ideology0, SES0, GenderCC, RaceCC, National0, MRN0

```
REGRESSION
 /MISSING LISTWISE
 /STATISTICS COEFF OUTS R ANOVA CHANGE ZPP
 /CRITERIA=PIN(.05) POUT(.10)
 /NOORIGIN
 /DEPENDENT Conspiracy_Tot
 /METHOD=ENTER Ideology0
 /METHOD=ENTER GenderCC RaceCC SES0
  /METHOD=ENTER National0
 /METHOD=ENTER MRN0
 /METHOD=ENTER MRN0xRace MRN0xSES0 MRN0xGender MRN0xIdeology0 MRN0xNational0.
```

Regression

Notes

Output Created		15-DEC-2021 13:09:41
Comments		
Input	Data	C: \Users\njs5478\Dropbox\HM and COVID\0. Revise and Resubmit\2. R and R Data\Study 2a\Study2a_Data.sav
	Active Dataset	DataSet1
	Filter	<none>
	Weight	<none>
	Split File	<none>
	N of Rows in Working Data File	188
Missing Value Handling	Definition of Missing	User-defined missing values are treated as missing.
	Cases Used	Statistics are based on cases with no missing values for any variable used.

Notes

Syntax		REGRESSION<br>/MISSING LISTWISE<br>/STATISTICS COEFF OUTS R ANOVA CHANGE ZPP<br>/CRITERIA=PIN(.05) POUT(.10)<br>/NOORIGIN<br>/DEPENDENT Conspiracy_Tot<br>/METHOD=ENTER Ideology0<br>/METHOD=ENTER GenderCC RaceCC SES0<br>/METHOD=ENTER National0<br>/METHOD=ENTER MRN0<br>/METHOD=ENTER MRN0xRace MRN0xSES0 MRN0xGender MRN0xIdeology0 MRN0xNational0.
Resources	Processor Time	00:00:00.02
	Elapsed Time	00:00:00.03
	Memory Required	35200 bytes
	Additional Memory Required for Residual Plots	0 bytes

Variables Entered/Removed[a]

Model	Variables Entered	Variables Removed	Method
1	Ideology0[b]	.	Enter
2	SES0, GenderCC, RaceCC[b]	.	Enter
3	National0[b]	.	Enter
4	MRN0[b]	.	Enter
5	MRN0xGender, MRN0xNational0, MRN0xSES0, MRN0xIdeology0, MRN0xRace[b]	.	Enter

a. Dependent Variable: Conspiracy_Tot

b. All requested variables entered.

Model Summary

Model	R	R Square	Adjusted R Square	Std. Error of the Estimate	Change Statistics		
					R Square Change	F Change	df1
1	.358[a]	.128	.124	.59259	.128	27.408	1
2	.384[b]	.148	.129	.59080	.019	1.377	3
3	.387[c]	.150	.127	.59160	.002	.505	1
4	.440[d]	.194	.167	.57783	.044	9.782	1
5	.520[e]	.271	.225	.55724	.077	3.724	5

Model Summary

Model	Change Statistics	
	df2	Sig. F Change
1	186	.000
2	183	.251
3	182	.478
4	181	.002
5	176	.003

a. Predictors: (Constant), Ideology0

b. Predictors: (Constant), Ideology0, SES0, GenderCC, RaceCC

c. Predictors: (Constant), Ideology0, SES0, GenderCC, RaceCC, National0

d. Predictors: (Constant), Ideology0, SES0, GenderCC, RaceCC, National0, MRN0

e. Predictors: (Constant), Ideology0, SES0, GenderCC, RaceCC, National0, MRN0, MRN0xGender, MRN0xNational0, MRN0xSES0, MRN0xIdeology0, MRN0xRace

ANOVA[a]

Model		Sum of Squares	df	Mean Square	F	Sig.
1	Regression	9.625	1	9.625	27.408	.000[b]
	Residual	65.317	186	.351		
	Total	74.942	187			
2	Regression	11.066	4	2.767	7.926	.000[c]
	Residual	63.875	183	.349		
	Total	74.942	187			
3	Regression	11.243	5	2.249	6.425	.000[d]
	Residual	63.699	182	.350		
	Total	74.942	187			
4	Regression	14.509	6	2.418	7.243	.000[e]
	Residual	60.433	181	.334		
	Total	74.942	187			
5	Regression	20.290	11	1.845	5.940	.000[f]
	Residual	54.651	176	.311		
	Total	74.942	187			

a. Dependent Variable: Conspiracy_Tot

b. Predictors: (Constant), Ideology0

c. Predictors: (Constant), Ideology0, SES0, GenderCC, RaceCC

d. Predictors: (Constant), Ideology0, SES0, GenderCC, RaceCC, National0

e. Predictors: (Constant), Ideology0, SES0, GenderCC, RaceCC, National0, MRN0

f. Predictors: (Constant), Ideology0, SES0, GenderCC, RaceCC, National0, MRN0, MRN0xGender, MRN0xNational0, MRN0xSES0, MRN0xIdeology0, MRN0xRace

Coefficients[a]

Model		Unstandardized Coefficients B	Unstandardized Coefficients Std. Error	Standardized Coefficients Beta	t	Sig.
1	(Constant)	1.544	.043		35.735	.000
	Ideology0	.150	.029	.358	5.235	.000
2	(Constant)	1.563	.047		32.922	.000
	Ideology0	.149	.030	.357	5.012	.000
	GenderCC	.011	.044	.018	.252	.802
	RaceCC	-.045	.049	-.065	-.916	.361
	SES0	.107	.059	.125	1.808	.072
3	(Constant)	1.557	.048		32.342	.000
	Ideology0	.154	.031	.369	5.035	.000
	GenderCC	.010	.044	.016	.230	.818
	RaceCC	-.032	.053	-.046	-.604	.546
	SES0	.111	.060	.128	1.855	.065
	National0	-.023	.032	-.055	-.710	.478
4	(Constant)	1.556	.047		33.082	.000
	Ideology0	.115	.032	.276	3.554	.000
	GenderCC	-.060	.049	-.095	-1.230	.220
	RaceCC	-.026	.052	-.038	-.512	.610
	SES0	.086	.059	.100	1.468	.144
	National0	-.039	.032	-.095	-1.240	.216
	MRN0	.199	.064	.269	3.128	.002
5	(Constant)	1.484	.052		28.386	.000
	Ideology0	.106	.033	.254	3.227	.001
	GenderCC	-.048	.047	-.075	-1.014	.312
	RaceCC	-.018	.051	-.026	-.346	.730
	SES0	.046	.059	.053	.782	.435
	National0	-.024	.031	-.057	-.757	.450
	MRN0	.215	.065	.290	3.328	.001
	MRN0xRace	.022	.058	.030	.382	.703
	MRN0xSES0	-.133	.080	-.114	-1.671	.097
	MRN0xGender	.112	.055	.135	2.048	.042
	MRN0xIdeology0	.018	.037	.036	.481	.631
	MRN0xNational0	.093	.033	.211	2.813	.005

Coefficients[a]

Model		Correlations		
		Zero-order	Partial	Part
1	(Constant)			
	Ideology0	.358	.358	.358
2	(Constant)			
	Ideology0	.358	.347	.342
	GenderCC	.077	.019	.017
	RaceCC	.026	-.068	-.062
	SES0	.160	.132	.123
3	(Constant)			
	Ideology0	.358	.350	.344
	GenderCC	.077	.017	.016
	RaceCC	.026	-.045	-.041
	SES0	.160	.136	.127
	National0	.052	-.053	-.049
4	(Constant)			
	Ideology0	.358	.255	.237
	GenderCC	.077	-.091	-.082
	RaceCC	.026	-.038	-.034
	SES0	.160	.108	.098
	National0	.052	-.092	-.083
	MRN0	.342	.226	.209
5	(Constant)			
	Ideology0	.358	.236	.208
	GenderCC	.077	-.076	-.065
	RaceCC	.026	-.026	-.022
	SES0	.160	.059	.050
	National0	.052	-.057	-.049
	MRN0	.342	.243	.214
	MRN0xRace	.275	.029	.025
	MRN0xSES0	-.067	-.125	-.108
	MRN0xGender	.121	.153	.132
	MRN0xIdeology0	.148	.036	.031
	MRN0xNational0	.206	.207	.181

a. Dependent Variable: Conspiracy_Tot

Excluded Variables[a]

Model		Beta In	t	Sig.	Partial Correlation	Collinearity Statistics Tolerance
1	GenderCC	.034[b]	.497	.620	.036	.986
	RaceCC	-.060[b]	-.854	.394	-.063	.947
	SES0	.121[b]	1.770	.078	.129	.987
	National0	-.061[b]	-.850	.397	-.062	.909
	MRN0	.228[b]	3.039	.003	.218	.800
	MRN0xRace	.165[b]	2.267	.025	.164	.864
	MRN0xSES0	-.112[b]	-1.629	.105	-.119	.986
	MRN0xGender	.152[b]	2.229	.027	.162	.993
	MRN0xIdeology0	.100[b]	1.446	.150	.106	.980
	MRN0xNational0	.192[b]	2.853	.005	.205	.998
2	National0	-.055[c]	-.710	.478	-.053	.782
	MRN0	.251[c]	2.957	.004	.214	.621
	MRN0xRace	.151[c]	2.049	.042	.150	.841
	MRN0xSES0	-.090[c]	-1.282	.201	-.095	.943
	MRN0xGender	.147[c]	2.168	.031	.159	.991
	MRN0xIdeology0	.107[c]	1.516	.131	.112	.937
	MRN0xNational0	.188[c]	2.771	.006	.201	.976
3	MRN0	.269[d]	3.128	.002	.226	.603
	MRN0xRace	.149[d]	2.009	.046	.148	.838
	MRN0xSES0	-.088[d]	-1.252	.212	-.093	.941
	MRN0xGender	.144[d]	2.107	.037	.155	.983
	MRN0xIdeology0	.103[d]	1.464	.145	.108	.932
	MRN0xNational0	.185[d]	2.718	.007	.198	.970
4	MRN0xRace	.111[e]	1.500	.135	.111	.810
	MRN0xSES0	-.074[e]	-1.080	.282	-.080	.937
	MRN0xGender	.134[e]	2.009	.046	.148	.981
	MRN0xIdeology0	.118[e]	1.716	.088	.127	.927
	MRN0xNational0	.225[e]	3.363	.001	.243	.946

a. Dependent Variable: Conspiracy_Tot

b. Predictors in the Model: (Constant), Ideology0

c. Predictors in the Model: (Constant), Ideology0, SES0, GenderCC, RaceCC

d. Predictors in the Model: (Constant), Ideology0, SES0, GenderCC, RaceCC, National0

e. Predictors in the Model: (Constant), Ideology0, SES0, GenderCC, RaceCC, National0, MRN0

```
 **Simple Slopes with PIdeology

IF (GenderCC=1) Male = 0.
IF (GenderCC=-1) Male = 1.
IF (GenderCC=1) Female=1.
IF (GenderCC=-1) Female=0.

IF (RaceCC=1) White = 0.
IF (RaceCC=-1) White = 1.
IF (RaceCC=1) Non=1.
IF (RaceCC=-1) Non=0.

DESCRIPTIVES VARIABLES=PIdeology
  /STATISTICS=MEAN STDDEV MIN MAX.
```

Descriptives

Notes

Output Created		15-DEC-2021 13:09:41
Comments		
Input	Data	C:\Users\njs5478\Dropbox\HM and COVID\0. Revise and Resubmit\2. R and R Data\Study 2a\Study2a_Data.sav
	Active Dataset	DataSet1
	Filter	<none>
	Weight	<none>
	Split File	<none>
	N of Rows in Working Data File	188
Missing Value Handling	Definition of Missing	User defined missing values are treated as missing.
	Cases Used	All non-missing data are used.

Notes

Syntax		DESCRIPTIVES VARIABLES=PIdeology /STATISTICS=MEAN STDDEV MIN MAX.
Resources	Processor Time	00:00:00.00
	Elapsed Time	00:00:00.00

Descriptive Statistics

	N	Minimum	Maximum	Mean	Std. Deviation
Which of the following best describes your political ideology?	188	1	7	3.55	1.514
Valid N (listwise)	188				

```
 COMPUTE Ideology.Low = Ideology0 + 1.51.
COMPUTE Ideology.High = Ideology0 - 1.51.

*HM*Gender on Conspiracy

UNIANOVA Conspiracy_Tot WITH MRN0 RaceCC Male SES0 Ideology0
  /PRINT=ETASQ PARAMETER
  /DESIGN=MRN0 RaceCC Male SES0 Ideology0
  MRN0*RaceCC MRN0*Male MRN0*SES0 MRN0*Ideology0.
```

Univariate Analysis of Variance

Notes

Output Created		15-DEC-2021 13:09:41
Comments		
Input	Data	C: \Users\njs5478\Dropbox\HM and COVID\0. Revise and Resubmit\2. R and R Data\Study 2a\Study2a_Data.sav
	Active Dataset	DataSet1
	Filter	<none>
	Weight	<none>
	Split File	<none>
	N of Rows in Working Data File	188
Missing Value Handling	Definition of Missing	User-defined missing values are treated as missing.
	Cases Used	Statistics are based on all cases with valid data for all variables in the model.
Syntax		UNIANOVA Conspiracy_Tot WITH MRN0 RaceCC Male SES0 Ideology0<br>/PRINT=ETASQ PARAMETER<br>/DESIGN=MRN0 RaceCC Male SES0 Ideology0<br>MRN0*RaceCC MRN0*Male MRN0*SES0 MRN0*Ideology0.
Resources	Processor Time	00:00:00.00
	Elapsed Time	00:00:00.00

Tests of Between-Subjects Effects

Dependent Variable: Conspiracy_Tot

Source	Type III Sum of Squares	df	Mean Square	F	Sig.	Partial Eta Squared
Corrected Model	17.644[a]	9	1.960	6.090	.000	.235
Intercept	145.846	1	145.846	453.084	.000	.718
MRN0	3.821	1	3.821	11.870	.001	.063
RaceCC	.305	1	.305	.946	.332	.005
Male	.322	1	.322	1.000	.319	.006
SES0	.231	1	.231	.716	.398	.004
Ideology0	3.149	1	3.149	9.783	.002	.052
MRN0 * RaceCC	.600	1	.600	1.863	.174	.010
MRN0 * Male	1.352	1	1.352	4.199	.042	.023
MRN0 * SES0	.863	1	.863	2.682	.103	.015
MRN0 * Ideology0	.583	1	.583	1.810	.180	.010
Error	57.298	178	.322			
Total	523.654	188				
Corrected Total	74.942	187				

a. R Squared = .235 (Adjusted R Squared = .197)

Parameter Estimates

Dependent Variable: Conspiracy_Tot

Parameter	B	Std. Error	t	Sig.	95% Confidence Interval	
					Lower Bound	Upper Bound
Intercept	1.456	.068	21.286	.000	1.321	1.591
MRN0	.282	.082	3.445	.001	.120	.443
RaceCC	-.048	.049	-.973	.332	-.144	.049
Male	.095	.095	1.000	.319	-.093	.283
SES0	.050	.059	.846	.398	-.067	.167
Ideology0	.104	.033	3.128	.002	.038	.169
MRN0 * RaceCC	.077	.056	1.365	.174	-.034	.188
MRN0 * Male	-.228	.111	-2.049	.042	-.447	-.008
MRN0 * SES0	-.133	.081	-1.638	.103	-.293	.027
MRN0 * Ideology0	.049	.036	1.345	.180	-.023	.121

Parameter Estimates

Dependent Variable: Conspiracy_Tot

Parameter	Partial Eta Squared
Intercept	.718
MRN0	.063
RaceCC	.005
Male	.006
SES0	.004
Ideology0	.052
MRN0 * RaceCC	.010
MRN0 * Male	.023
MRN0 * SES0	.015
MRN0 * Ideology0	.010

```
UNIANOVA Conspiracy_Tot WITH MRN0 RaceCC Female SES0 Ideology0
 /PRINT=ETASQ PARAMETER
 /DESIGN=MRN0 RaceCC Female SES0 Ideology0
 MRN0*RaceCC MRN0*Female MRN0*SES0 MRN0*Ideology0.
```

Univariate Analysis of Variance

Notes

Output Created		15-DEC-2021 13:09:41
Comments		
Input	Data	C:\Users\njs5478\Dropbox\HM and COVID\0. Revise and Resubmit\2. R and R Data\Study 2a\Study2a_Data.sav
	Active Dataset	DataSet1
	Filter	<none>
	Weight	<none>
	Split File	<none>
	N of Rows in Working Data File	188
Missing Value Handling	Definition of Missing	User-defined missing values are treated as missing.
	Cases Used	Statistics are based on all cases with valid data for all variables in the model.
Syntax		UNIANOVA Conspiracy_Tot WITH MRN0 RaceCC Female SES0 Ideology0 /PRINT=ETASQ PARAMETER /DESIGN=MRN0 RaceCC Female SES0 Ideology0 MRN0*RaceCC MRN0*Female MRN0*SES0 MRN0*Ideology0.
Resources	Processor Time	00:00:00.02
	Elapsed Time	00:00:00.01

Tests of Between-Subjects Effects

Dependent Variable: Conspiracy_Tot

Source	Type III Sum of Squares	df	Mean Square	F	Sig.	Partial Eta Squared
Corrected Model	17.644[a]	9	1.960	6.090	.000	.235
Intercept	143.249	1	143.249	445.016	.000	.714
MRN0	.125	1	.125	.389	.534	.002
RaceCC	.305	1	.305	.946	.332	.005
Female	.322	1	.322	1.000	.319	.006
SES0	.231	1	.231	.716	.398	.004
Ideology0	3.149	1	3.149	9.783	.002	.052
MRN0 * RaceCC	.600	1	.600	1.863	.174	.010
MRN0 * Female	1.352	1	1.352	4.199	.042	.023
MRN0 * SES0	.863	1	.863	2.682	.103	.015
MRN0 * Ideology0	.583	1	.583	1.810	.180	.010
Error	57.298	178	.322			
Total	523.654	188				
Corrected Total	74.942	187				

a. R Squared = .235 (Adjusted R Squared = .197)

Parameter Estimates

Dependent Variable: Conspiracy_Tot

Parameter	B	Std. Error	t	Sig.	95% Confidence Interval	
					Lower Bound	Upper Bound
Intercept	1.551	.074	21.095	.000	1.406	1.696
MRN0	.054	.087	.623	.534	-.117	.225
RaceCC	-.048	.049	-.973	.332	-.144	.049
Female	-.095	.095	-1.000	.319	-.283	.093
SES0	.050	.059	.846	.398	-.067	.167
Ideology0	.104	.033	3.128	.002	.038	.169
MRN0 * RaceCC	.077	.056	1.365	.174	-.034	.188
MRN0 * Female	.228	.111	2.049	.042	.008	.447
MRN0 * SES0	-.133	.081	-1.638	.103	-.293	.027
MRN0 * Ideology0	.049	.036	1.345	.180	-.023	.121

Parameter Estimates

Dependent Variable: Conspiracy_Tot

Parameter	Partial Eta Squared
Intercept	.714
MRN0	.002
RaceCC	.005
Female	.006
SES0	.004
Ideology0	.052
MRN0 * RaceCC	.010
MRN0 * Female	.023
MRN0 * SES0	.015
MRN0 * Ideology0	.010

```
UNIANOVA Conspiracy_Tot WITH MRN.Low RaceCC GenderCC SES0 Ideology0
 /PRINT=ETASQ PARAMETER
 /DESIGN=MRN.Low RaceCC GenderCC SES0 Ideology0
 MRN.Low*RaceCC MRN.Low*GenderCC MRN.Low*SES0 MRN.Low*Ideology0.
```

Univariate Analysis of Variance

Notes

Output Created		15-DEC-2021 13:09:41
Comments		
Input	Data	C:\Users\njs5478\Dropbox\HM and COVID\0. Revise and Resubmit\2. R and R Data\Study 2a\Study2a_Data.sav
	Active Dataset	DataSet1
	Filter	<none>
	Weight	<none>
	Split File	<none>
	N of Rows in Working Data File	188
Missing Value Handling	Definition of Missing	User-defined missing values are treated as missing.
	Cases Used	Statistics are based on all cases with valid data for all variables in the model.
Syntax		UNIANOVA Conspiracy_Tot WITH MRN.Low RaceCC GenderCC SES0 Ideology0 /PRINT=ETASQ PARAMETER /DESIGN=MRN.Low RaceCC GenderCC SES0 Ideology0 MRN.Low*RaceCC MRN.Low*GenderCC MRN.Low*SES0 MRN.Low*Ideology0.
Resources	Processor Time	00:00:00.02
	Elapsed Time	00:00:00.02

Tests of Between-Subjects Effects

Dependent Variable: Conspiracy_Tot

Source	Type III Sum of Squares	df	Mean Square	F	Sig.
Corrected Model	17.644[a]	9	1.960	6.090	.000
Intercept	105.354	1	105.354	327.292	.000
MRN.Low	2.266	1	2.266	7.040	.009
RaceCC	1.067	1	1.067	3.316	.070
GenderCC	1.517	1	1.517	4.711	.031
SES0	1.324	1	1.324	4.113	.044
Ideology0	.580	1	.580	1.801	.181
MRN.Low * RaceCC	.600	1	.600	1.863	.174
MRN.Low * GenderCC	1.352	1	1.352	4.199	.042
MRN.Low * SES0	.863	1	.863	2.682	.103
MRN.Low * Ideology0	.583	1	.583	1.810	.180
Error	57.298	178	.322		
Total	523.654	188			
Corrected Total	74.942	187			

Tests of Between-Subjects Effects

Dependent Variable: Conspiracy_Tot

Source	Partial Eta Squared
Corrected Model	.235
Intercept	.648
MRN.Low	.038
RaceCC	.018
GenderCC	.026
SES0	.023
Ideology0	.010
MRN.Low * RaceCC	.010
MRN.Low * GenderCC	.023
MRN.Low * SES0	.015
MRN.Low * Ideology0	.010
Error	
Total	
Corrected Total	

a. R Squared = .235 (Adjusted R Squared = .197)

Parameter Estimates

Dependent Variable: Conspiracy_Tot

Parameter	B	Std. Error	t	Sig.	95% Confidence Interval	
					Lower Bound	Upper Bound
Intercept	1.360	.075	18.091	.000	1.212	1.508
MRN.Low	.168	.063	2.653	.009	.043	.293
RaceCC	-.113	.062	-1.821	.070	-.236	.009
GenderCC	-.145	.067	-2.171	.031	-.277	-.013
SES0	.164	.081	2.028	.044	.004	.323
Ideology0	.062	.046	1.342	.181	-.029	.153
MRN.Low * RaceCC	.077	.056	1.365	.174	-.034	.188
MRN.Low * GenderCC	.114	.056	2.049	.042	.004	.224
MRN.Low * SES0	-.133	.081	-1.638	.103	-.293	.027
MRN.Low * Ideology0	.049	.036	1.345	.180	-.023	.121

Parameter Estimates

Dependent Variable: Conspiracy_Tot

Parameter	Partial Eta Squared
Intercept	.648
MRN.Low	.038
RaceCC	.018
GenderCC	.026
SES0	.023
Ideology0	.010
MRN.Low * RaceCC	.010
MRN.Low * GenderCC	.023
MRN.Low * SES0	.015
MRN.Low * Ideology0	.010

```
UNIANOVA Conspiracy_Tot WITH MRN.High RaceCC GenderCC SES0 Ideology0
 /PRINT=ETASQ PARAMETER
 /DESIGN=MRN.High RaceCC GenderCC SES0 Ideology0
 MRN.High*RaceCC MRN.High*GenderCC MRN.High*SES0 MRN.High*Ideology0.
```

Univariate Analysis of Variance

Notes

Output Created		15-DEC-2021 13:09:41
Comments		
Input	Data	C:\Users\njs5478\Dropbox\HM and COVID\0. Revise and Resubmit\2. R and R Data\Study 2a\Study2a_Data.sav
	Active Dataset	DataSet1
	Filter	<none>
	Weight	<none>
	Split File	<none>
	N of Rows in Working Data File	188
Missing Value Handling	Definition of Missing	User-defined missing values are treated as missing.
	Cases Used	Statistics are based on all cases with valid data for all variables in the model.
Syntax		UNIANOVA Conspiracy_Tot WITH MRN.High RaceCC GenderCC SES0 Ideology0<br>/PRINT=ETASQ PARAMETER<br>/DESIGN=MRN.High RaceCC GenderCC SES0 Ideology0<br>MRN.High*RaceCC MRN.High*GenderCC MRN.High*SES0 MRN.High*Ideology0.
Resources	Processor Time	00:00:00.00
	Elapsed Time	00:00:00.02

Tests of Between-Subjects Effects

Dependent Variable: Conspiracy_Tot

Source	Type III Sum of Squares	df	Mean Square	F	Sig.
Corrected Model	17.644[a]	9	1.960	6.090	.000
Intercept	151.761	1	151.761	471.458	.000
MRN.High	2.266	1	2.266	7.040	.009
RaceCC	.019	1	.019	.060	.807
GenderCC	.174	1	.174	.540	.463
SES0	.127	1	.127	.393	.532
Ideology0	3.398	1	3.398	10.555	.001
MRN.High * RaceCC	.600	1	.600	1.863	.174
MRN.High * GenderCC	1.352	1	1.352	4.199	.042
MRN.High * SES0	.863	1	.863	2.682	.103
MRN.High * Ideology0	.583	1	.583	1.810	.180
Error	57.298	178	.322		
Total	523.654	188			
Corrected Total	74.942	187			

Tests of Between-Subjects Effects

Dependent Variable: Conspiracy_Tot

Source	Partial Eta Squared
Corrected Model	.235
Intercept	.726
MRN.High	.038
RaceCC	.000
GenderCC	.003
SES0	.002
Ideology0	.056
MRN.High * RaceCC	.010
MRN.High * GenderCC	.023
MRN.High * SES0	.015
MRN.High * Ideology0	.010
Error	
Total	
Corrected Total	

a. R Squared = .235 (Adjusted R Squared = .197)

Parameter Estimates

Dependent Variable: Conspiracy_Tot

Parameter	B	Std. Error	t	Sig.	95% Confidence Interval Lower Bound	95% Confidence Interval Upper Bound
Intercept	1.647	.076	21.713	.000	1.497	1.796
MRN.High	.168	.063	2.653	.009	.043	.293
RaceCC	.018	.075	.244	.807	-.129	.165
GenderCC	.050	.068	.735	.463	-.084	.183
SES0	-.063	.101	-.627	.532	-.262	.136
Ideology0	.146	.045	3.249	.001	.057	.234
MRN.High * RaceCC	.077	.056	1.365	.174	-.034	.188
MRN.High * GenderCC	.114	.056	2.049	.042	.004	.224
MRN.High * SES0	-.133	.081	-1.638	.103	-.293	.027
MRN.High * Ideology0	.049	.036	1.345	.180	-.023	.121

Parameter Estimates

Dependent Variable: Conspiracy_Tot

Parameter	Partial Eta Squared
Intercept	.726
MRN.High	.038
RaceCC	.000
GenderCC	.003
SES0	.002
Ideology0	.056
MRN.High * RaceCC	.010
MRN.High * GenderCC	.023
MRN.High * SES0	.015
MRN.High * Ideology0	.010

```
*Including National ID

*HM*Race on Psychology

UNIANOVA Psychology_Tot WITH MRN0 White GenderCC SES0 Ideology0 National0
  /PRINT=ETASQ PARAMETER
  /DESIGN=MRN0 White GenderCC SES0 Ideology0 National0
  MRN0*White MRN0*GenderCC MRN0*SES0 MRN0*Ideology0 MRN0*National0.
```

Univariate Analysis of Variance

Notes

Output Created		15-DEC-2021 13:09:41
Comments		
Input	Data	C:\Users\njs5478\Dropbox\HM and COVID\0. Revise and Resubmit\2. R and R Data\Study 2a\Study2a_Data.sav
	Active Dataset	DataSet1
	Filter	<none>
	Weight	<none>
	Split File	<none>
	N of Rows in Working Data File	188
Missing Value Handling	Definition of Missing	User-defined missing values are treated as missing.
	Cases Used	Statistics are based on all cases with valid data for all variables in the model.
Syntax		UNIANOVA Psychology_Tot WITH MRN0 White GenderCC SES0 Ideology0 National0 /PRINT=ETASQ PARAMETER /DESIGN=MRN0 White GenderCC SES0 Ideology0 National0 MRN0*White MRN0*GenderCC MRN0*SES0 MRN0*Ideology0 MRN0*National0.
Resources	Processor Time	00:00:00.02
	Elapsed Time	00:00:00.02

Tests of Between-Subjects Effects

Dependent Variable: Psychology_Tot

Source	Type III Sum of Squares	df	Mean Square	F	Sig.	Partial Eta Squared
Corrected Model	45.999[a]	11	4.182	2.200	.016	.121
Intercept	1686.928	1	1686.928	887.681	.000	.835
MRN0	10.267	1	10.267	5.403	.021	.030
White	.000	1	.000	.000	.990	.000
GenderCC	.303	1	.303	.159	.690	.001
SES0	.684	1	.684	.360	.549	.002
Ideology0	2.663	1	2.663	1.401	.238	.008
National0	.633	1	.633	.333	.565	.002
MRN0 * White	8.788	1	8.788	4.624	.033	.026
MRN0 * GenderCC	1.243	1	1.243	.654	.420	.004
MRN0 * SES0	.198	1	.198	.104	.747	.001
MRN0 * Ideology0	6.615	1	6.615	3.481	.064	.019
MRN0 * National0	5.740	1	5.740	3.020	.084	.017
Error	334.466	176	1.900			
Total	4256.778	188				
Corrected Total	380.465	187				

a. R Squared = .121 (Adjusted R Squared = .066)

Parameter Estimates

Dependent Variable: Psychology_Tot

Parameter	B	Std. Error	t	Sig.	95% Confidence Interval	
					Lower Bound	Upper Bound
Intercept	4.362	.146	29.794	.000	4.073	4.651
MRN0	-.430	.185	-2.324	.021	-.795	-.065
White	.003	.254	.012	.990	-.499	.505
GenderCC	-.046	.116	-.399	.690	-.276	.183
SES0	-.087	.145	-.600	.549	-.373	.199
Ideology0	-.097	.082	-1.184	.238	-.257	.064
National0	.044	.077	.577	.565	-.107	.196
MRN0 * White	.622	.289	2.150	.033	.051	1.193
MRN0 * GenderCC	.110	.136	.809	.420	-.158	.378
MRN0 * SES0	.064	.197	.323	.747	-.326	.453
MRN0 * Ideology0	.172	.092	1.866	.064	-.010	.355
MRN0 * National0	.143	.082	1.738	.084	-.019	.304

Parameter Estimates

Dependent Variable: Psychology_Tot

Parameter	Partial Eta Squared
Intercept	.835
MRN0	.030
White	.000
GenderCC	.001
SES0	.002
Ideology0	.008
National0	.002
MRN0 * White	.026
MRN0 * GenderCC	.004
MRN0 * SES0	.001
MRN0 * Ideology0	.019
MRN0 * National0	.017

```
UNIANOVA Psychology_Tot WITH MRN0 Non GenderCC SES0 Ideology0 National0
 /PRINT=ETASQ PARAMETER
```

```
/DESIGN=MRN0 Non GenderCC SES0 Ideology0 National0
MRN0*Non MRN0*GenderCC MRN0*SES0 MRN0*Ideology0 MRN0*National0.
```

Univariate Analysis of Variance

Notes

Output Created		15-DEC-2021 13:09:41
Comments		
Input	Data	C: \Users\njs5478\Dropbox\HM and COVID\0. Revise and Resubmit\2. R and R Data\Study 2a\Study2a_Data.sav
	Active Dataset	DataSet1
	Filter	<none>
	Weight	<none>
	Split File	<none>
	N of Rows in Working Data File	188
Missing Value Handling	Definition of Missing	User-defined missing values are treated as missing.
	Cases Used	Statistics are based on all cases with valid data for all variables in the model.
Syntax		UNIANOVA Psychology_Tot WITH MRN0 Non GenderCC SES0 Ideology0 National0 /PRINT=ETASQ PARAMETER /DESIGN=MRN0 Non GenderCC SES0 Ideology0 National0 MRN0*Non MRN0*GenderCC MRN0*SES0 MRN0*Ideology0 MRN0*National0.
Resources	Processor Time	00:00:00.02
	Elapsed Time	00:00:00.03

Tests of Between-Subjects Effects

Dependent Variable: Psychology_Tot

Source	Type III Sum of Squares	df	Mean Square	F	Sig.	Partial Eta Squared
Corrected Model	45.999[a]	11	4.182	2.200	.016	.121
Intercept	815.543	1	815.543	429.148	.000	.709
MRN0	1.193	1	1.193	.628	.429	.004
Non	.000	1	.000	.000	.990	.000
GenderCC	.303	1	.303	.159	.690	.001
SES0	.684	1	.684	.360	.549	.002
Ideology0	2.663	1	2.663	1.401	.238	.008
National0	.633	1	.633	.333	.565	.002
MRN0 * Non	8.788	1	8.788	4.624	.033	.026
MRN0 * GenderCC	1.243	1	1.243	.654	.420	.004
MRN0 * SES0	.198	1	.198	.104	.747	.001
MRN0 * Ideology0	6.615	1	6.615	3.481	.064	.019
MRN0 * National0	5.740	1	5.740	3.020	.084	.017
Error	334.466	176	1.900			
Total	4256.778	188				
Corrected Total	380.465	187				

a. R Squared = .121 (Adjusted R Squared = .066)

Parameter Estimates

Dependent Variable: Psychology_Tot

Parameter	B	Std. Error	t	Sig.	95% Confidence Interval	
					Lower Bound	Upper Bound
Intercept	4.365	.211	20.716	.000	3.949	4.781
MRN0	.192	.242	.792	.429	-.286	.670
Non	-.003	.254	-.012	.990	-.505	.499
GenderCC	-.046	.116	-.399	.690	-.276	.183
SES0	-.087	.145	-.600	.549	-.373	.199
Ideology0	-.097	.082	-1.184	.238	-.257	.064
National0	.044	.077	.577	.565	-.107	.196
MRN0 * Non	-.622	.289	-2.150	.033	-1.193	-.051
MRN0 * GenderCC	.110	.136	.809	.420	-.158	.378
MRN0 * SES0	.064	.197	.323	.747	-.326	.453
MRN0 * Ideology0	.172	.092	1.866	.064	-.010	.355
MRN0 * National0	.143	.082	1.738	.084	-.019	.304

Parameter Estimates

Dependent Variable: Psychology_Tot

Parameter	Partial Eta Squared
Intercept	.709
MRN0	.004
Non	.000
GenderCC	.001
SES0	.002
Ideology0	.008
National0	.002
MRN0 * Non	.026
MRN0 * GenderCC	.004
MRN0 * SES0	.001
MRN0 * Ideology0	.019
MRN0 * National0	.017

```
*HM*Gender on Conspiracy
```

```
UNIANOVA Conspiracy_Tot WITH MRN0 RaceCC Male SES0 Ideology0 National0
  /PRINT=ETASQ PARAMETER
  /DESIGN=MRN0 RaceCC Male SES0 Ideology0 National0
  MRN0*RaceCC MRN0*Male MRN0*SES0 MRN0*Ideology0 MRN0*National0.
```

Univariate Analysis of Variance

Notes

Output Created		15-DEC-2021 13:09:41
Comments		
Input	Data	C: \Users\njs5478\Dropbox\HM and COVID\0. Revise and Resubmit\2. R and R Data\Study 2a\Study2a_Data.sav
	Active Dataset	DataSet1
	Filter	<none>
	Weight	<none>
	Split File	<none>
	N of Rows in Working Data File	188
Missing Value Handling	Definition of Missing	User-defined missing values are treated as missing.
	Cases Used	Statistics are based on all cases with valid data for all variables in the model.
Syntax		UNIANOVA Conspiracy_Tot WITH MRN0 RaceCC Male SES0 Ideology0 National0 /PRINT=ETASQ PARAMETER /DESIGN=MRN0 RaceCC Male SES0 Ideology0 National0 MRN0*RaceCC MRN0*Male MRN0*SES0 MRN0*Ideology0 MRN0*National0.
Resources	Processor Time	00:00:00.02
	Elapsed Time	00:00:00.02

Tests of Between-Subjects Effects

Dependent Variable: Conspiracy_Tot

Source	Type III Sum of Squares	df	Mean Square	F	Sig.	Partial Eta Squared
Corrected Model	20.290[a]	11	1.845	5.940	.000	.271
Intercept	138.514	1	138.514	446.071	.000	.717
MRN0	4.970	1	4.970	16.004	.000	.083
RaceCC	.037	1	.037	.120	.730	.001
Male	.319	1	.319	1.028	.312	.006
SES0	.190	1	.190	.612	.435	.003
Ideology0	3.235	1	3.235	10.417	.001	.056
National0	.178	1	.178	.573	.450	.003
MRN0 * RaceCC	.045	1	.045	.146	.703	.001
MRN0 * Male	1.302	1	1.302	4.193	.042	.023
MRN0 * SES0	.867	1	.867	2.791	.097	.016
MRN0 * Ideology0	.072	1	.072	.231	.631	.001
MRN0 * National0	2.457	1	2.457	7.914	.005	.043
Error	54.651	176	.311			
Total	523.654	188				
Corrected Total	74.942	187				

a. R Squared = .271 (Adjusted R Squared = .225)

Parameter Estimates

Dependent Variable: Conspiracy_Tot

Parameter	B	Std. Error	t	Sig.	95% Confidence Interval	
					Lower Bound	Upper Bound
Intercept	1.436	.068	21.120	.000	1.302	1.571
MRN0	.327	.082	4.001	.000	.166	.489
RaceCC	-.018	.051	-.346	.730	-.119	.084
Male	.095	.094	1.014	.312	-.090	.281
SES0	.046	.059	.782	.435	-.070	.161
Ideology0	.106	.033	3.227	.001	.041	.171
National0	-.024	.031	-.757	.450	-.085	.038
MRN0 * RaceCC	.022	.058	.382	.703	-.093	.138
MRN0 * Male	-.225	.110	-2.048	.042	-.441	-.008
MRN0 * SES0	-.133	.080	-1.671	.097	-.291	.024
MRN0 * Ideology0	.018	.037	.481	.631	-.056	.092
MRN0 * National0	.093	.033	2.813	.005	.028	.159

Parameter Estimates

Dependent Variable: Conspiracy_Tot

Parameter	Partial Eta Squared
Intercept	.717
MRN0	.083
RaceCC	.001
Male	.006
SES0	.003
Ideology0	.056
National0	.003
MRN0 * RaceCC	.001
MRN0 * Male	.023
MRN0 * SES0	.016
MRN0 * Ideology0	.001
MRN0 * National0	.043

```
UNIANOVA Conspiracy_Tot WITH MRN0 RaceCC Female SES0 Ideology0 National0
 /PRINT=ETASQ PARAMETER
```

```
/DESIGN=MRN0 RaceCC Female SES0 Ideology0 National0
MRN0*RaceCC MRN0*Female MRN0*SES0 MRN0*Ideology0 MRN0*National0.
```

Univariate Analysis of Variance

Notes

Output Created		15-DEC-2021 13:09:41
Comments		
Input	Data	C: \Users\njs5478\Dropbox\HM and COVID\0. Revise and Resubmit\2. R and R Data\Study 2a\Study2a_Data.sav
	Active Dataset	DataSet1
	Filter	<none>
	Weight	<none>
	Split File	<none>
	N of Rows in Working Data File	188
Missing Value Handling	Definition of Missing	User-defined missing values are treated as missing.
	Cases Used	Statistics are based on all cases with valid data for all variables in the model.
Syntax		UNIANOVA Conspiracy_Tot WITH MRN0 RaceCC Female SES0 Ideology0 National0 /PRINT=ETASQ PARAMETER /DESIGN=MRN0 RaceCC Female SES0 Ideology0 National0 MRN0*RaceCC MRN0*Female MRN0*SES0 MRN0*Ideology0 MRN0*National0.
Resources	Processor Time	00:00:00.00
	Elapsed Time	00:00:00.00

Tests of Between-Subjects Effects

Dependent Variable: Conspiracy_Tot

Source	Type III Sum of Squares	df	Mean Square	F	Sig.	Partial Eta Squared
Corrected Model	20.290[a]	11	1.845	5.940	.000	.271
Intercept	138.447	1	138.447	445.858	.000	.717
MRN0	.427	1	.427	1.375	.242	.008
RaceCC	.037	1	.037	.120	.730	.001
Female	.319	1	.319	1.028	.312	.006
SES0	.190	1	.190	.612	.435	.003
Ideology0	3.235	1	3.235	10.417	.001	.056
National0	.178	1	.178	.573	.450	.003
MRN0 * RaceCC	.045	1	.045	.146	.703	.001
MRN0 * Female	1.302	1	1.302	4.193	.042	.023
MRN0 * SES0	.867	1	.867	2.791	.097	.016
MRN0 * Ideology0	.072	1	.072	.231	.631	.001
MRN0 * National0	2.457	1	2.457	7.914	.005	.043
Error	54.651	176	.311			
Total	523.654	188				
Corrected Total	74.942	187				

a. R Squared = .271 (Adjusted R Squared = .225)

Parameter Estimates

Dependent Variable: Conspiracy_Tot

Parameter	B	Std. Error	t	Sig.	95% Confidence Interval Lower Bound	95% Confidence Interval Upper Bound
Intercept	1.532	.073	21.115	.000	1.389	1.675
MRN0	.103	.088	1.173	.242	-.070	.276
RaceCC	-.018	.051	-.346	.730	-.119	.084
Female	-.095	.094	-1.014	.312	-.281	.090
SES0	.046	.059	.782	.435	-.070	.161
Ideology0	.106	.033	3.227	.001	.041	.171
National0	-.024	.031	-.757	.450	-.085	.038
MRN0 * RaceCC	.022	.058	.382	.703	-.093	.138
MRN0 * Female	.225	.110	2.048	.042	.008	.441
MRN0 * SES0	-.133	.080	-1.671	.097	-.291	.024
MRN0 * Ideology0	.018	.037	.481	.631	-.056	.092
MRN0 * National0	.093	.033	2.813	.005	.028	.159

Parameter Estimates

Dependent Variable: Conspiracy_Tot

Parameter	Partial Eta Squared
Intercept	.717
MRN0	.008
RaceCC	.001
Female	.006
SES0	.003
Ideology0	.056
National0	.003
MRN0 * RaceCC	.001
MRN0 * Female	.023
MRN0 * SES0	.016
MRN0 * Ideology0	.001
MRN0 * National0	.043

```
*HM*Ideology on Conspiracy
```

```
UNIANOVA Conspiracy_Tot WITH MRN0 RaceCC GenderCC SES0 Ideology.Low National0
  /PRINT=ETASQ PARAMETER
  /DESIGN=MRN0 RaceCC GenderCC SES0 Ideology.Low National0
  MRN0*RaceCC MRN0*GenderCC MRN0*SES0 MRN0*Ideology.Low MRN0*National0.
```

Univariate Analysis of Variance

Notes

Output Created		15-DEC-2021 13:09:41
Comments		
Input	Data	C: \Users\njs5478\Dropbox\HM and COVID\0. Revise and Resubmit\2. R and R Data\Study 2a\Study2a_Data.sav
	Active Dataset	DataSet1
	Filter	<none>
	Weight	<none>
	Split File	<none>
	N of Rows in Working Data File	188
Missing Value Handling	Definition of Missing	User-defined missing values are treated as missing.
	Cases Used	Statistics are based on all cases with valid data for all variables in the model.
Syntax		UNIANOVA Conspiracy_Tot WITH MRN0 RaceCC GenderCC SES0 Ideology.Low National0 /PRINT=ETASQ PARAMETER /DESIGN=MRN0 RaceCC GenderCC SES0 Ideology.Low National0 MRN0*RaceCC MRN0*GenderCC MRN0*SES0 MRN0*Ideology.Low MRN0*National0.
Resources	Processor Time	00:00:00.02
	Elapsed Time	00:00:00.01

Tests of Between-Subjects Effects

Dependent Variable: Conspiracy_Tot

Source	Type III Sum of Squares	df	Mean Square	F	Sig.
Corrected Model	20.290[a]	11	1.845	5.940	.000
Intercept	113.065	1	113.065	364.115	.000
MRN0	1.569	1	1.569	5.053	.026
RaceCC	.037	1	.037	.120	.730
GenderCC	.319	1	.319	1.028	.312
SES0	.190	1	.190	.612	.435
Ideology.Low	3.235	1	3.235	10.417	.001
National0	.178	1	.178	.573	.450
MRN0 * RaceCC	.045	1	.045	.146	.703
MRN0 * GenderCC	1.302	1	1.302	4.193	.042
MRN0 * SES0	.867	1	.867	2.791	.097
MRN0 * Ideology.Low	.072	1	.072	.231	.631
MRN0 * National0	2.457	1	2.457	7.914	.005
Error	54.651	176	.311		
Total	523.654	188			
Corrected Total	74.942	187			

Tests of Between-Subjects Effects

Dependent Variable: Conspiracy_Tot

Source	Partial Eta Squared
Corrected Model	.271
Intercept	.674
MRN0	.028
RaceCC	.001
GenderCC	.006
SES0	.003
Ideology.Low	.056
National0	.003
MRN0 * RaceCC	.001
MRN0 * GenderCC	.023
MRN0 * SES0	.016
MRN0 * Ideology.Low	.001
MRN0 * National0	.043
Error	
Total	
Corrected Total	

a. R Squared = .271 (Adjusted R Squared = .225)

Parameter Estimates

Dependent Variable: Conspiracy_Tot

Parameter	B	Std. Error	t	Sig.	95% Confidence Interval	
					Lower Bound	Upper Bound
Intercept	1.324	.069	19.082	.000	1.187	1.460
MRN0	.188	.084	2.248	.026	.023	.353
RaceCC	-.018	.051	-.346	.730	-.119	.084
GenderCC	-.048	.047	-1.014	.312	-.140	.045
SES0	.046	.059	.782	.435	-.070	.161
Ideology.Low	.106	.033	3.227	.001	.041	.171
National0	-.024	.031	-.757	.450	-.085	.038
MRN0 * RaceCC	.022	.058	.382	.703	-.093	.138
MRN0 * GenderCC	.112	.055	2.048	.042	.004	.221
MRN0 * SES0	-.133	.080	-1.671	.097	-.291	.024
MRN0 * Ideology.Low	.018	.037	.481	.631	-.056	.092
MRN0 * National0	.093	.033	2.813	.005	.028	.159

Parameter Estimates

Dependent Variable: Conspiracy_Tot

Parameter	Partial Eta Squared
Intercept	.674
MRN0	.028
RaceCC	.001
GenderCC	.006
SES0	.003
Ideology.Low	.056
National0	.003
MRN0 * RaceCC	.001
MRN0 * GenderCC	.023
MRN0 * SES0	.016
MRN0 * Ideology.Low	.001
MRN0 * National0	.043

```
UNIANOVA Conspiracy_Tot WITH MRN0 RaceCC GenderCC SES0 Ideology.High National0
 /PRINT=ETASQ PARAMETER
```

```
/DESIGN=MRN0 RaceCC GenderCC SES0 Ideology.High National0
MRN0*RaceCC MRN0*GenderCC MRN0*SES0 MRN0*Ideology.High MRN0*National0.
```

Univariate Analysis of Variance

Notes

Output Created		15-DEC-2021 13:09:41
Comments		
Input	Data	C:\Users\njs5478\Dropbox\HM and COVID\0. Revise and Resubmit\2. R and R Data\Study 2a\Study2a_Data.sav
	Active Dataset	DataSet1
	Filter	<none>
	Weight	<none>
	Split File	<none>
	N of Rows in Working Data File	188
Missing Value Handling	Definition of Missing	User-defined missing values are treated as missing.
	Cases Used	Statistics are based on all cases with valid data for all variables in the model.
Syntax		UNIANOVA Conspiracy_Tot WITH MRN0 RaceCC GenderCC SES0 Ideology.High National0 /PRINT=ETASQ PARAMETER /DESIGN=MRN0 RaceCC GenderCC SES0 Ideology.High National0 MRN0*RaceCC MRN0*GenderCC MRN0*SES0 MRN0*Ideology.High MRN0*National0.
Resources	Processor Time	00:00:00.00
	Elapsed Time	00:00:00.00

Tests of Between-Subjects Effects

Dependent Variable: Conspiracy_Tot

Source	Type III Sum of Squares	df	Mean Square	F	Sig.
Corrected Model	20.290[a]	11	1.845	5.940	.000
Intercept	149.750	1	149.750	482.258	.000
MRN0	2.358	1	2.358	7.594	.006
RaceCC	.037	1	.037	.120	.730
GenderCC	.319	1	.319	1.028	.312
SES0	.190	1	.190	.612	.435
Ideology.High	3.235	1	3.235	10.417	.001
National0	.178	1	.178	.573	.450
MRN0 * RaceCC	.045	1	.045	.146	.703
MRN0 * GenderCC	1.302	1	1.302	4.193	.042
MRN0 * SES0	.867	1	.867	2.791	.097
MRN0 * Ideology.High	.072	1	.072	.231	.631
MRN0 * National0	2.457	1	2.457	7.914	.005
Error	54.651	176	.311		
Total	523.654	188			
Corrected Total	74.942	187			

Tests of Between-Subjects Effects

Dependent Variable: Conspiracy_Tot

Source	Partial Eta Squared
Corrected Model	.271
Intercept	.733
MRN0	.041
RaceCC	.001
GenderCC	.006
SES0	.003
Ideology.High	.056
National0	.003
MRN0 * RaceCC	.001
MRN0 * GenderCC	.023
MRN0 * SES0	.016
MRN0 * Ideology.High	.001
MRN0 * National0	.043
Error	
Total	
Corrected Total	

a. R Squared = .271 (Adjusted R Squared = .225)

Parameter Estimates

Dependent Variable: Conspiracy_Tot

Parameter	B	Std. Error	t	Sig.	95% Confidence Interval	
					Lower Bound	Upper Bound
Intercept	1.645	.075	21.960	.000	1.497	1.793
MRN0	.242	.088	2.756	.006	.069	.416
RaceCC	-.018	.051	-.346	.730	-.119	.084
GenderCC	-.048	.047	-1.014	.312	-.140	.045
SES0	.046	.059	.782	.435	-.070	.161
Ideology.High	.106	.033	3.227	.001	.041	.171
National0	-.024	.031	-.757	.450	-.085	.038
MRN0 * RaceCC	.022	.058	.382	.703	-.093	.138
MRN0 * GenderCC	.112	.055	2.048	.042	.004	.221
MRN0 * SES0	-.133	.080	-1.671	.097	-.291	.024
MRN0 * Ideology.High	.018	.037	.481	.631	-.056	.092
MRN0 * National0	.093	.033	2.813	.005	.028	.159

Parameter Estimates

Dependent Variable: Conspiracy_Tot

Parameter	Partial Eta Squared
Intercept	.733
MRN0	.041
RaceCC	.001
GenderCC	.006
SES0	.003
Ideology.High	.056
National0	.003
MRN0 * RaceCC	.001
MRN0 * GenderCC	.023
MRN0 * SES0	.016
MRN0 * Ideology.High	.001
MRN0 * National0	.043
