# Supplementary material for: Men’s and women’s endorsement of hegemonic masculinity and responses to COVID-19
Source: J Health Psychol. 2022 Mar 11;28(3):251–66. doi: 10.1177/13591053221081905 (PMC9982413; doi:10.1177/13591053221081905)
Supplement: sj-pdf-4-hpq-10.1177_13591053221081905 – for Men’s and women’s endorsement of hegemonic masculinity and responses to COVID-19 [file sj-pdf-4-hpq-10.1177_13591053221081905.pdf]

```
 * Encoding: UTF-8.

**Study 2b Syntax**

*No participants meet exclusion criteria

***Variable Creation***

**Male Role Norms**

RECODE Tough_8 Fem_6 (1=7) (2=6) (3=5) (4=4) (5=3) (6=2) (7=1) INTO Tough_8_Recode Fem_6_
Recode.
EXECUTE.

COMPUTE MRN=(Power_1 + Power_2 + Power_3 + Power_4 + Power_5 + Power_6 + Power_7 + Power_
8 +
    Power_9  + Power_10 + Power_11 + Tough_1 + Tough_2 + Tough_3 + Tough_4 + Tough_5 + To
ugh_6 +
    Tough_7 + Tough_8_Recode + Fem_1 + Fem_2 + Fem_3 + Fem_4 + Fem_5 + Fem_6_Recode + Fem
_7)/26.
EXECUTE.

COMPUTE Power=(Power_1 + Power_2 + Power_3 + Power_4 + Power_5 + Power_6 + Power_7 + Powe
r_8 +
    Power_9  + Power_10 + Power_11)/11.
EXECUTE.

COMPUTE Tough=(Tough_1 + Tough_2 + Tough_3 + Tough_4 + Tough_5 + Tough_6 +
    Tough_7 + Tough_8_Recode)/8.
EXECUTE.

COMPUTE Fem=(Fem_1 + Fem_2 + Fem_3 + Fem_4 + Fem_5 + Fem_6_Recode + Fem_7)/7.
EXECUTE.

RELIABILITY
  /VARIABLES=Power_1 Power_2 Power_3 Power_4 Power_5 Power_6 Power_7 Power_8 Power_9 Powe
r_10
    Power_11 Tough_1 Tough_2 Tough_3 Tough_4 Tough_5 Tough_6 Tough_7 Tough_8_Recode Fem_1
 Fem_2 Fem_3
    Fem_4 Fem_5 Fem_6_Recode Fem_7
  /SCALE('ALL VARIABLES') ALL
  /MODEL=ALPHA.
```

Reliability

Notes

Output Created		15-DEC-2021 13:11:09
Comments		
Input	Data	C:\Users\njs5478\Dropbox\HM and COVID\0. Revise and Resubmit\2. R and R Data\Study 2b\Study2b_Data.sav
	Active Dataset	DataSet1
	Filter	<none>
	Weight	<none>
	Split File	<none>
	N of Rows in Working Data File	198
	Matrix Input	
Missing Value Handling	Definition of Missing	User-defined missing values are treated as missing.
	Cases Used	Statistics are based on all cases with valid data for all variables in the procedure.
Syntax		RELIABILITY /VARIABLES=Power_1 Power_2 Power_3 Power_4 Power_5 Power_6 Power_7 Power_8 Power_9 Power_10 Power_11 Tough_1 Tough_2 Tough_3 Tough_4 Tough_5 Tough_6 Tough_7 Tough_8_Recode Fem_1 Fem_2 Fem_3 Fem_4 Fem_5 Fem_6_Recode Fem_7 /SCALE('ALL VARIABLES') ALL /MODEL=ALPHA.
Resources	Processor Time	00:00:00.00
	Elapsed Time	00:00:00.00

```
[DataSet1] C:\Users\njs5478\Dropbox\HM and COVID\0. Revise and Resubmit\2. R and R Data\S
tudy 2b\Study2b_Data.sav
```

Scale: ALL VARIABLES

Case Processing Summary

		N	%
Cases	Valid	198	100.0
	Excluded[a]	0	.0
	Total	198	100.0

a. Listwise deletion based on all variables in the procedure.

Reliability Statistics

Cronbach's Alpha	N of Items
.936	26

```
 **Risk During COVID-19*

*Note, we removed the 3 "help-based" items from Studies 2a and 2b

RECODE Risk1 Risk3 Risk4 Risk8 Risk11 (1=7) (2=6) (3=5) (4=4) (5=3) (6=2) (7=1) INTO Risk
1_R Risk3_R Risk4_R Risk8_R Risk11_R.
EXECUTE.

RELIABILITY
  /VARIABLES=Risk1_R Risk2 Risk3_R Risk4_R Risk5 Risk6 Risk7 Risk8_R Risk9 Risk10 Risk11_
R Risk12 Risk16 Risk17 Risk18
  /SCALE('ALL VARIABLES') ALL
  /MODEL=ALPHA.
```

Reliability

Notes

Output Created		15-DEC-2021 13:11:09
Comments		
Input	Data	C:\Users\njs5478\Dropbox\HM and COVID\0. Revise and Resubmit\2. R and R Data\Study 2b\Study2b_Data.sav
	Active Dataset	DataSet1
	Filter	<none>
	Weight	<none>
	Split File	<none>
	N of Rows in Working Data File	198
	Matrix Input	
Missing Value Handling	Definition of Missing	User-defined missing values are treated as missing.
	Cases Used	Statistics are based on all cases with valid data for all variables in the procedure.
Syntax		RELIABILITY /VARIABLES=Risk1_R Risk2 Risk3_R Risk4_R Risk5 Risk6 Risk7 Risk8_R Risk9 Risk10 Risk11_R Risk12 Risk16 Risk17 Risk18 /SCALE('ALL VARIABLES') ALL /MODEL=ALPHA.
Resources	Processor Time	00:00:00.00
	Elapsed Time	00:00:00.00

Scale: ALL VARIABLES

Case Processing Summary

		N	%
Cases	Valid	198	100.0
	Excluded[a]	0	.0
	Total	198	100.0

a. Listwise deletion based on all variables in the procedure.

Reliability Statistics

Cronbach's Alpha	N of Items
.843	15

```
COMPUTE Risk_Rules = (Risk1_R + Risk2 + Risk3_R + Risk4_R + Risk5 + Risk6 + Risk7 + Risk8
_R + Risk9 + Risk10 + Risk11_R + Risk12 + Risk16 + Risk17 + Risk18)/15.

**Coronavirus Concern**

RECODE Concern3 (1=7) (2=6) (3=5) (4=4) (5=3) (6=2) (7=1) INTO Concern3_R.
EXECUTE.

RELIABILITY
  /VARIABLES=Concern1 Concern2 Concern3_R Concern4 Concern5 Concern6
  /SCALE('ALL VARIABLES') ALL
  /MODEL=ALPHA
  /SUMMARY=TOTAL.
```

Reliability

Notes

Output Created		15-DEC-2021 13:11:10
Comments		
Input	Data	C: \Users\njs5478\Dropbox\HM and COVID\0. Revise and Resubmit\2. R and R Data\Study 2b\Study2b_Data.sav
	Active Dataset	DataSet1
	Filter	<none>
	Weight	<none>
	Split File	<none>
	N of Rows in Working Data File	198
	Matrix Input	
Missing Value Handling	Definition of Missing	User-defined missing values are treated as missing.
	Cases Used	Statistics are based on all cases with valid data for all variables in the procedure.
Syntax		RELIABILITY /VARIABLES=Concern1 Concern2 Concern3_R Concern4 Concern5 Concern6 /SCALE('ALL VARIABLES') ALL /MODEL=ALPHA /SUMMARY=TOTAL.
Resources	Processor Time	00:00:00.00
	Elapsed Time	00:00:00.00

Scale: ALL VARIABLES

Case Processing Summary

		N	%
Cases	Valid	198	100.0
	Excluded[a]	0	.0
	Total	198	100.0

a. Listwise deletion based on all variables in the procedure.

Reliability Statistics

Cronbach's Alpha	N of Items
.924	6

Item-Total Statistics

	Scale Mean if Item Deleted	Scale Variance if Item Deleted	Corrected Item-Total Correlation	Cronbach's Alpha if Item Deleted
Please indicate your agreement/disagreement with each statement using the scale provided: - Thinking about the coronavirus (COVID-19) makes me feel threatened.	21.9646	68.105	.767	.912
Please indicate your agreement/disagreement with each statement using the scale provided: - I am afraid of the coronavirus (COVID-19).	21.5606	64.095	.870	.897
Concern3_R	21.1111	68.130	.708	.920
Please indicate your agreement/disagreement with each statement using the scale provided: - I am worried that I or people I love will get sick from the coronavirus (COVID-19).	20.6667	69.421	.767	.912
Please indicate your agreement/disagreement with each statement using the scale provided: - I am stressed around other people because I worry I'll catch the coronavirus (COVID-19).	21.9899	65.736	.809	.906
Please indicate your agreement/disagreement with each statement using the scale provided: - I have tried hard to avoid other people because I don't want to get sick.	21.2172	66.699	.766	.912

```
COMPUTE Concern_Tot=mean(concern1, Concern2, Concern3_R, Concern4, Concern5, Concern6).

**Financial

RECODE Finance3 (1=7) (2=6) (3=5) (4=4) (5=3) (6=2) (7=1) INTO Finance3_R.
EXECUTE.

RELIABILITY
  /VARIABLES=Finance1 Finance2 Finance3_R
  /SCALE('ALL VARIABLES') ALL
  /MODEL=ALPHA
  /SUMMARY=TOTAL.
```

Reliability

Notes

Output Created		15-DEC-2021 13:11:10
Comments		
Input	Data	C:\Users\njs5478\Dropbox\HM and COVID\0. Revise and Resubmit\2. R and R Data\Study 2b\Study2b_Data.sav
	Active Dataset	DataSet1
	Filter	<none>
	Weight	<none>
	Split File	<none>
	N of Rows in Working Data File	198
	Matrix Input	
Missing Value Handling	Definition of Missing	User-defined missing values are treated as missing.
	Cases Used	Statistics are based on all cases with valid data for all variables in the procedure.
Syntax		RELIABILITY /VARIABLES=Finance1 Finance2 Finance3_R /SCALE('ALL VARIABLES') ALL /MODEL=ALPHA /SUMMARY=TOTAL.

Notes

Resources	Processor Time	00:00:00.00
	Elapsed Time	00:00:00.00

Scale: ALL VARIABLES

Case Processing Summary

		N	%
Cases	Valid	198	100.0
	Excluded[a]	0	.0
	Total	198	100.0

a. Listwise deletion based on all variables in the procedure.

Reliability Statistics

Cronbach's Alpha	N of Items
.908	3

Item-Total Statistics

	Scale Mean if Item Deleted	Scale Variance if Item Deleted	Corrected Item-Total Correlation	Cronbach's Alpha if Item Deleted
Please indicate your agreement/disagreement with each statement using the scale provided: - The Coronavirus (COVID-19) has impacted me negatively from a financial point of view.	7.5101	16.292	.863	.830
Please indicate your agreement/disagreement with each statement using the scale provided: - I have lost job-related income due to the Coronavirus (COVID-19).	8.0859	16.891	.765	.911
Finance3_R	7.1818	16.292	.823	.863

```
COMPUTE Finance_Tot=mean(Finance1, Finance2, Finance3_R).

*Resources

*Note. We removed the resource items from Studies 2a and 2b

*Psychology

RECODE Psychology3 (1=7) (2=6) (3=5) (4=4) (5=3) (6=2) (7=1) INTO Psychology3_R.
EXECUTE.

RELIABILITY
  /VARIABLES=Psychology1 Psychology2 Psychology3_R
  /SCALE('ALL VARIABLES') ALL
  /MODEL=ALPHA
  /SUMMARY=TOTAL.
```

Reliability

Notes

Output Created		15-DEC-2021 13:11:10
Comments		
Input	Data	C: \Users\njs5478\Dropbox\HM and COVID\0. Revise and Resubmit\2. R and R Data\Study 2b\Study2b_Data.sav
	Active Dataset	DataSet1
	Filter	<none>
	Weight	<none>
	Split File	<none>
	N of Rows in Working Data File	198
	Matrix Input	
Missing Value Handling	Definition of Missing	User-defined missing values are treated as missing.
	Cases Used	Statistics are based on all cases with valid data for all variables in the procedure.

Notes

Syntax		RELIABILITY<br><br>/VARIABLES=Psychology1 Psychology2 Psychology3_R<br>/SCALE('ALL VARIABLES') ALL<br>/MODEL=ALPHA<br>/SUMMARY=TOTAL.
Resources	Processor Time	00:00:00.00
	Elapsed Time	00:00:00.00

Scale: ALL VARIABLES

Case Processing Summary

		N	%
Cases	Valid	198	100.0
	Excluded[a]	0	.0
	Total	198	100.0

a. Listwise deletion based on all variables in the procedure.

Reliability Statistics

Cronbach's Alpha	N of Items
.863	3

Item-Total Statistics

	Scale Mean if Item Deleted	Scale Variance if Item Deleted	Corrected Item-Total Correlation	Cronbach's Alpha if Item Deleted
Please indicate your agreement/disagreement with each statement using the scale provided: - I have become depressed because of the Coronavirus (COVID-19).	8.8737	12.101	.735	.814
Please indicate your agreement/disagreement with each statement using the scale provided: - The Coronavirus (COVID-19) outbreak has impacted my psychological health negatively.	8.0707	11.904	.796	.756
Psychology3_R	7.7525	13.081	.693	.850

```
COMPUTE Psychology_Tot=mean(Psychology1, Psychology2, Psychology3_R).

*Mandates

RELIABILITY
  /VARIABLES=Mandate1 Mandate2 Mandate3 Mandate4 Mandate5 Mandate6
  /SCALE('ALL VARIABLES') ALL
  /MODEL=ALPHA
  /SUMMARY=TOTAL.
```

Reliability

Notes

Output Created		15-DEC-2021 13:11:10
Comments		
Input	Data	C:\Users\njs5478\Dropbox\HM and COVID\0. Revise and Resubmit\2. R and R Data\Study 2b\Study2b_Data.sav
	Active Dataset	DataSet1
	Filter	<none>
	Weight	<none>
	Split File	<none>
	N of Rows in Working Data File	198
	Matrix Input	
Missing Value Handling	Definition of Missing	User-defined missing values are treated as missing.
	Cases Used	Statistics are based on all cases with valid data for all variables in the procedure.
Syntax		RELIABILITY /VARIABLES=Mandate1 Mandate2 Mandate3 Mandate4 Mandate5 Mandate6 /SCALE('ALL VARIABLES') ALL /MODEL=ALPHA /SUMMARY=TOTAL.
Resources	Processor Time	00:00:00.00
	Elapsed Time	00:00:00.00

Scale: ALL VARIABLES

Case Processing Summary

		N	%
Cases	Valid	198	100.0
	Excluded[a]	0	.0
	Total	198	100.0

a. Listwise deletion based on all variables in the procedure.

Reliability Statistics

Cronbach's Alpha	N of Items
.964	6

Item-Total Statistics

	Scale Mean if Item Deleted	Scale Variance if Item Deleted	Corrected Item-Total Correlation	Cronbach's Alpha if Item Deleted
Please indicate your agreement/disagreement with each statement using the scale provided: - Employers with over 100 employees should require that all workers get the COVID-19 vaccine.	22.65	121.700	.940	.951
Please indicate your agreement/disagreement with each statement using the scale provided: - Health care workers should be required to get the COVID-19 vaccine or be terminated.	22.40	121.490	.930	.952
Please indicate your agreement/disagreement with each statement using the scale provided: - School employees should be required to get the COVID-19 vaccine or be terminated.	22.52	120.687	.948	.950
Please indicate your agreement/disagreement with each statement using the scale provided: - People should continue to be required to wear masks indoors.	22.61	138.838	.652	.979

Item-Total Statistics

	Scale Mean if Item Deleted	Scale Variance if Item Deleted	Corrected Item-Total Correlation	Cronbach's Alpha if Item Deleted
Please indicate your agreement/disagreement with each statement using the scale provided: - People should have to provide proof of vaccination to enter entertainment venues (e.g., concerts).	22.60	123.561	.915	.954
Please indicate your agreement/disagreement with each statement using the scale provided: - People should have to provide proof of vaccination to travel.	22.34	121.252	.921	.953

```
 COMPUTE Mandate_Tot=mean(Mandate1, Mandate2, Mandate3, Mandate4, Mandate5, Mandate6).

*Conspiracy

 RELIABILITY
  /VARIABLES=Conspiracy1 Conspiracy2 Conspiracy3 Conspiracy4 Conspiracy5 Conspiracy6 Cons
piracy7 Conspiracy8 Conspiracy9
  /SCALE('ALL VARIABLES') ALL
  /MODEL=ALPHA
  /SUMMARY=TOTAL.
```

Reliability

Notes

Output Created		15-DEC-2021 13:11:10
Comments		
Input	Data	C: \Users\njs5478\Dropbox\HM and COVID\0. Revise and Resubmit\2. R and R Data\Study 2b\Study2b_Data.sav
	Active Dataset	DataSet1
	Filter	<none>
	Weight	<none>
	Split File	<none>
	N of Rows in Working Data File	198
	Matrix Input	
Missing Value Handling	Definition of Missing	User-defined missing values are treated as missing.
	Cases Used	Statistics are based on all cases with valid data for all variables in the procedure.
Syntax		RELIABILITY /VARIABLES=Conspiracy1 Conspiracy2 Conspiracy3 Conspiracy4 Conspiracy5 Conspiracy6 Conspiracy7 Conspiracy8 Conspiracy9 /SCALE('ALL VARIABLES') ALL /MODEL=ALPHA...
Resources	Processor Time	00:00:00.02
	Elapsed Time	00:00:00.02

Scale: ALL VARIABLES

Case Processing Summary

		N	%
Cases	Valid	198	100.0
	Excluded[a]	0	.0
	Total	198	100.0

a. Listwise deletion based on all variables in the procedure.

Reliability Statistics

Cronbach's Alpha	N of Items
.834	9

Item-Total Statistics

	Scale Mean if Item Deleted	Scale Variance if Item Deleted	Corrected Item-Total Correlation	Cronbach's Alpha if Item Deleted
Please indicate, using the scale provided, how credible you believe each theory about COVID-19 to be: - COVID-19 has been released by the US government to destabilize China	14.07	29.437	.519	.822
Please indicate, using the scale provided, how credible you believe each theory about COVID-19 to be: - COVID-19 was developed to control population growth	13.75	24.941	.751	.792

Item-Total Statistics

	Scale Mean if Item Deleted	Scale Variance if Item Deleted	Corrected Item-Total Correlation	Cronbach's Alpha if Item Deleted
Please indicate, using the scale provided, how credible you believe each theory about COVID-19 to be: - COVID-19 is a way to cover up the effects of 5G towers	14.20	30.281	.512	.825
Please indicate, using the scale provided, how credible you believe each theory about COVID-19 to be: - COVID-19 was developed by pharmaceutical companies	13.74	27.075	.532	.819
Please indicate, using the scale provided, how credible you believe each theory about COVID-19 to be: - Together with the vaccine, a chip will be injected to permanently track people	14.10	28.802	.544	.819
Please indicate, using the scale provided, how credible you believe each theory about COVID-19 to be: - The Chinese government lies about the number of COVID-19 deaths in China	12.19	27.476	.312	.858

Item-Total Statistics

	Scale Mean if Item Deleted	Scale Variance if Item Deleted	Corrected Item-Total Correlation	Cronbach's Alpha if Item Deleted
Please indicate, using the scale provided, how credible you believe each theory about COVID-19 to be: - COVID-19 was spread deliberately among the Chinese population	13.41	24.619	.687	.799
Please indicate, using the scale provided, how credible you believe each theory about COVID-19 to be: - COVID-19 was developed by the Chinese government to damage the Western world and its economies, in order to become the strongest economy in the world	13.39	23.559	.748	.790
Please indicate, using the scale provided, how credible you believe each theory about COVID-19 to be: - COVID-19 was developed by climate activists to counteract climate change	14.14	29.868	.523	.823

```
COMPUTE Conspiracy_Tot=mean(Conspiracy1, Conspiracy2, Conspiracy3, Conspiracy4, Conspirac
y5, Conspiracy6, Conspiracy7, Conspiracy8, Conspiracy9).

*National Identity

 RELIABILITY
  /VARIABLES=National1 National2
  /SCALE('ALL VARIABLES') ALL
  /MODEL=ALPHA
  /SUMMARY=TOTAL.
```

Reliability

Notes

Output Created		15-DEC-2021 13:11:10
Comments		
Input	Data	C:\Users\njs5478\Dropbox\HM and COVID\0. Revise and Resubmit\2. R and R Data\Study 2b\Study2b_Data.sav
	Active Dataset	DataSet1
	Filter	<none>
	Weight	<none>
	Split File	<none>
	N of Rows in Working Data File	198
	Matrix Input	
Missing Value Handling	Definition of Missing	User-defined missing values are treated as missing.
	Cases Used	Statistics are based on all cases with valid data for all variables in the procedure.
Syntax		RELIABILITY /VARIABLES=National1 National2 /SCALE('ALL VARIABLES') ALL /MODEL=ALPHA /SUMMARY=TOTAL.
Resources	Processor Time	00:00:00.02
	Elapsed Time	00:00:00.02

Scale: ALL VARIABLES

Case Processing Summary

		N	%
Cases	Valid	198	100.0
	Excluded[a]	0	.0
	Total	198	100.0

a. Listwise deletion based on all variables in the procedure.

Reliability Statistics

Cronbach's Alpha	N of Items
.615	2

Item-Total Statistics

	Scale Mean if Item Deleted	Scale Variance if Item Deleted	Corrected Item-Total Correlation	Cronbach's Alpha if Item Deleted
I identify as American.	5.04	3.527	.539	.
Being an American is an important reflection of who I am.	6.43	.977	.539	.

```
COMPUTE National_Tot=mean(National1, National2).
```

```
**Political Identity
```

```
CORRELATIONS
  /VARIABLES=PParty PIdeology
  /PRINT=TWOTAIL NOSIG FULL
  /MISSING=PAIRWISE.
```

Correlations

Notes

Output Created		15-DEC-2021 13:11:10
Comments		
Input	Data	C:\Users\njs5478\Dropbox\HM and COVID\0. Revise and Resubmit\2. R and R Data\Study 2b\Study2b_Data.sav
	Active Dataset	DataSet1
	Filter	<none>
	Weight	<none>
	Split File	<none>
	N of Rows in Working Data File	198
Missing Value Handling	Definition of Missing	User-defined missing values are treated as missing.
	Cases Used	Statistics for each pair of variables are based on all the cases with valid data for that pair.
Syntax		CORRELATIONS /VARIABLES=PParty PIdeology /PRINT=TWOTAIL NOSIG FULL /MISSING=PAIRWISE.
Resources	Processor Time	00:00:00.00
	Elapsed Time	00:00:00.00

Correlations

		Which of the following best describes your political party affiliation?	Which of the following best describes your political ideology?
Which of the following best describes your political party affiliation?	Pearson Correlation	1	.896**
	Sig. (2-tailed)		.000
	N	198	198
Which of the following best describes your political ideology?	Pearson Correlation	.896**	1
	Sig. (2-tailed)	.000	
	N	198	198

**. Correlation is significant at the 0.01 level (2-tailed).

```
 **Descriptives and Frequencies**

FREQUENCIES VARIABLES=PParty PIdeology SES Education Gender Race Age MRN Finance_Tot Psyc
hology_Tot Mandate_Tot Conspiracy_Tot National_Tot
  /STATISTICS=STDDEV MINIMUM MAXIMUM MEAN
  /ORDER=ANALYSIS.
```

Frequencies

Notes

Output Created		15-DEC-2021 13:11:10
Comments		
Input	Data	C:\Users\njs5478\Dropbox\HM and COVID\0. Revise and Resubmit\2. R and R Data\Study 2b\Study2b_Data.sav
	Active Dataset	DataSet1
	Filter	<none>
	Weight	<none>
	Split File	<none>
	N of Rows in Working Data File	198
Missing Value Handling	Definition of Missing	User-defined missing values are treated as missing.
	Cases Used	Statistics are based on all cases with valid data.
Syntax		FREQUENCIES VARIABLES=PParty PIdeology SES Education Gender Race Age MRN Finance_Tot Psychology_Tot Mandate_Tot Conspiracy_Tot National_Tot /STATISTICS=STDDEV MINIMUM MAXIMUM MEAN...
Resources	Processor Time	00:00:00.03
	Elapsed Time	00:00:00.03

Statistics

		Which of the following best describes your political party affiliation?	Which of the following best describes your political ideology?	Self Reported Socioeconomic Status	Please indicate the highest level of education that you have received:	Gender
N	Valid	198	198	198	198	196
	Missing	0	0	0	0	2
Mean		2.86	3.82	2.73	4.39	1.5051
Std. Deviation		1.560	2.031	.853	1.406	.50125
Minimum		1	1	1	1	1.00
Maximum		5	7	5	7	2.00

Statistics

		Racial Identity - Selected Choice	Age	MRN	Finance_Tot	Psychology_Tot	Mandate_Tot
N	Valid	198	198	198	198	198	198
	Missing	0	0	0	0	0	0
Mean		1.68	36.74	3.2960	3.7963	4.1162	4.5042
Std. Deviation		1.673	12.653	1.03162	1.98543	1.70083	2.22434
Minimum		1	18	1.23	1.00	1.00	1.00
Maximum		8	68	5.81	7.00	7.00	7.00

Statistics

		Conspiracy_Tot	National_Tot
N	Valid	198	198
	Missing	0	0
Mean		1.7082	5.7323
Std. Deviation		.64693	1.27513
Minimum		1.00	1.50
Maximum		3.67	7.00

Frequency Table

Which of the following best describes your political party affiliation?

		Frequency	Percent	Valid Percent	Cumulative Percent
Valid	Democrat	62	31.3	31.3	31.3
	Democrat Leaning	33	16.7	16.7	48.0
	Independent	9	4.5	4.5	52.5
	Republican Leaning	58	29.3	29.3	81.8
	Republican	36	18.2	18.2	100.0
	Total	198	100.0	100.0	

Which of the following best describes your political ideology?

		Frequency	Percent	Valid Percent	Cumulative Percent
Valid	Very Liberal	35	17.7	17.7	17.7
	Liberal	38	19.2	19.2	36.9
	Somewhat Liberal	18	9.1	9.1	46.0
	Neither Liberal Nor Conservative	12	6.1	6.1	52.0
	Somewhat Conservative	42	21.2	21.2	73.2
	Conservative	37	18.7	18.7	91.9
	Very Conservative	16	8.1	8.1	100.0
	Total	198	100.0	100.0	

Self Reported Socioeconomic Status

		Frequency	Percent	Valid Percent	Cumulative Percent
Valid	Poor	14	7.1	7.1	7.1
	Working Class	62	31.3	31.3	38.4
	Middle Class	88	44.4	44.4	82.8
	Upper Middle Class	32	16.2	16.2	99.0
	Upper Class	2	1.0	1.0	100.0
	Total	198	100.0	100.0	

Please indicate the highest level of education that you have received:

		Frequency	Percent	Valid Percent	Cumulative Percent
Valid	Some high school	2	1.0	1.0	1.0
	Completed high school	26	13.1	13.1	14.1
	Some college	29	14.6	14.6	28.8
	Associate's Degree	20	10.1	10.1	38.9
	Bachelor's Degree	85	42.9	42.9	81.8
	Master's Degree	28	14.1	14.1	96.0
	PhD or Professional Degree	8	4.0	4.0	100.0
	Total	198	100.0	100.0	

Gender

		Frequency	Percent	Valid Percent	Cumulative Percent
Valid	Male	97	49.0	49.5	49.5
	Female	99	50.0	50.5	100.0
	Total	196	99.0	100.0	
Missing	System	2	1.0		
Total		198	100.0		

Racial Identity - Selected Choice

		Frequency	Percent	Valid Percent	Cumulative Percent
Valid	White/Caucasian	159	80.3	80.3	80.3
	Black/African American	8	4.0	4.0	84.3
	Asian	14	7.1	7.1	91.4
	Hispanic/Latino(a)	9	4.5	4.5	96.0
	Biracial	3	1.5	1.5	97.5
	Multiracial	5	2.5	2.5	100.0
	Total	198	100.0	100.0	

Age

		Frequency	Percent	Valid Percent	Cumulative Percent
Valid	18	1	.5	.5	.5
	19	3	1.5	1.5	2.0
	20	2	1.0	1.0	3.0
	21	1	.5	.5	3.5
	22	9	4.5	4.5	8.1
	23	7	3.5	3.5	11.6
	24	8	4.0	4.0	15.7
	25	5	2.5	2.5	18.2
	26	11	5.6	5.6	23.7
	27	9	4.5	4.5	28.3
	28	9	4.5	4.5	32.8
	29	7	3.5	3.5	36.4
	30	4	2.0	2.0	38.4
	31	8	4.0	4.0	42.4
	32	9	4.5	4.5	47.0
	33	7	3.5	3.5	50.5
	34	9	4.5	4.5	55.1
	35	7	3.5	3.5	58.6
	36	5	2.5	2.5	61.1
	37	4	2.0	2.0	63.1
	38	2	1.0	1.0	64.1
	39	4	2.0	2.0	66.2
	40	2	1.0	1.0	67.2
	41	4	2.0	2.0	69.2
	42	6	3.0	3.0	72.2
	43	2	1.0	1.0	73.2
	44	2	1.0	1.0	74.2
	45	3	1.5	1.5	75.8
	46	3	1.5	1.5	77.3
	47	4	2.0	2.0	79.3
	48	3	1.5	1.5	80.8
	49	2	1.0	1.0	81.8
	50	1	.5	.5	82.3
	51	3	1.5	1.5	83.8
	52	3	1.5	1.5	85.4

Age

	Frequency	Percent	Valid Percent	Cumulative Percent
53	1	.5	.5	85.9
55	3	1.5	1.5	87.4
56	5	2.5	2.5	89.9
57	1	.5	.5	90.4
58	2	1.0	1.0	91.4
59	1	.5	.5	91.9
60	2	1.0	1.0	92.9
61	2	1.0	1.0	93.9
62	1	.5	.5	94.4
63	4	2.0	2.0	96.5
64	1	.5	.5	97.0
65	1	.5	.5	97.5
66	3	1.5	1.5	99.0
67	1	.5	.5	99.5
68	1	.5	.5	100.0
Total	198	100.0	100.0	

MRN

		Frequency	Percent	Valid Percent	Cumulative Percent
Valid	1.23	1	.5	.5	.5
	1.35	1	.5	.5	1.0
	1.38	1	.5	.5	1.5
	1.42	2	1.0	1.0	2.5
	1.50	1	.5	.5	3.0
	1.58	1	.5	.5	3.5
	1.62	1	.5	.5	4.0
	1.65	2	1.0	1.0	5.1
	1.69	2	1.0	1.0	6.1
	1.73	1	.5	.5	6.6
	1.77	2	1.0	1.0	7.6
	1.81	1	.5	.5	8.1
	1.85	1	.5	.5	8.6
	1.88	3	1.5	1.5	10.1
	1.92	1	.5	.5	10.6
	1.96	1	.5	.5	11.1
	2.00	1	.5	.5	11.6
	2.08	2	1.0	1.0	12.6
	2.12	1	.5	.5	13.1
	2.15	4	2.0	2.0	15.2
	2.19	2	1.0	1.0	16.2
	2.23	3	1.5	1.5	17.7
	2.27	3	1.5	1.5	19.2
	2.31	3	1.5	1.5	20.7
	2.35	1	.5	.5	21.2
	2.38	1	.5	.5	21.7
	2.42	6	3.0	3.0	24.7
	2.46	5	2.5	2.5	27.3
	2.50	2	1.0	1.0	28.3
	2.54	1	.5	.5	28.8
	2.58	2	1.0	1.0	29.8
	2.62	2	1.0	1.0	30.8
	2.65	2	1.0	1.0	31.8
	2.69	2	1.0	1.0	32.8
	2.73	4	2.0	2.0	34.8

MRN

	Frequency	Percent	Valid Percent	Cumulative Percent
2.77	3	1.5	1.5	36.4
2.81	1	.5	.5	36.9
2.85	2	1.0	1.0	37.9
2.88	3	1.5	1.5	39.4
2.96	3	1.5	1.5	40.9
3.00	2	1.0	1.0	41.9
3.08	2	1.0	1.0	42.9
3.12	1	.5	.5	43.4
3.15	3	1.5	1.5	44.9
3.19	2	1.0	1.0	46.0
3.23	2	1.0	1.0	47.0
3.27	4	2.0	2.0	49.0
3.35	5	2.5	2.5	51.5
3.38	5	2.5	2.5	54.0
3.42	2	1.0	1.0	55.1
3.46	3	1.5	1.5	56.6
3.50	2	1.0	1.0	57.6
3.54	3	1.5	1.5	59.1
3.62	1	.5	.5	59.6
3.65	1	.5	.5	60.1
3.69	1	.5	.5	60.6
3.73	5	2.5	2.5	63.1
3.77	5	2.5	2.5	65.7
3.81	1	.5	.5	66.2
3.85	3	1.5	1.5	67.7
3.88	5	2.5	2.5	70.2
3.92	3	1.5	1.5	71.7
4.00	5	2.5	2.5	74.2
4.04	5	2.5	2.5	76.8
4.08	2	1.0	1.0	77.8
4.12	3	1.5	1.5	79.3
4.15	2	1.0	1.0	80.3
4.19	4	2.0	2.0	82.3
4.23	2	1.0	1.0	83.3
4.31	1	.5	.5	83.8

MRN

	Frequency	Percent	Valid Percent	Cumulative Percent
4.38	1	.5	.5	84.3
4.42	2	1.0	1.0	85.4
4.46	1	.5	.5	85.9
4.54	4	2.0	2.0	87.9
4.65	2	1.0	1.0	88.9
4.73	5	2.5	2.5	91.4
4.77	4	2.0	2.0	93.4
4.85	1	.5	.5	93.9
4.88	1	.5	.5	94.4
4.96	2	1.0	1.0	95.5
5.00	2	1.0	1.0	96.5
5.04	1	.5	.5	97.0
5.19	1	.5	.5	97.5
5.46	1	.5	.5	98.0
5.58	1	.5	.5	98.5
5.69	1	.5	.5	99.0
5.81	2	1.0	1.0	100.0
Total	198	100.0	100.0	

Finance_Tot

		Frequency	Percent	Valid Percent	Cumulative Percent
Valid	1.00	22	11.1	11.1	11.1
	1.33	9	4.5	4.5	15.7
	1.67	15	7.6	7.6	23.2
	2.00	18	9.1	9.1	32.3
	2.33	4	2.0	2.0	34.3
	2.67	5	2.5	2.5	36.9
	3.00	10	5.1	5.1	41.9
	3.33	7	3.5	3.5	45.5
	3.67	9	4.5	4.5	50.0
	4.00	6	3.0	3.0	53.0
	4.33	15	7.6	7.6	60.6
	4.67	12	6.1	6.1	66.7
	5.00	10	5.1	5.1	71.7
	5.33	10	5.1	5.1	76.8
	5.67	6	3.0	3.0	79.8
	6.00	8	4.0	4.0	83.8
	6.33	6	3.0	3.0	86.9
	6.67	8	4.0	4.0	90.9
	7.00	18	9.1	9.1	100.0
	Total	198	100.0	100.0	

Psychology_Tot

		Frequency	Percent	Valid Percent	Cumulative Percent
Valid	1.00	8	4.0	4.0	4.0
	1.33	9	4.5	4.5	8.6
	1.67	7	3.5	3.5	12.1
	2.00	12	6.1	6.1	18.2
	2.33	6	3.0	3.0	21.2
	2.67	10	5.1	5.1	26.3
	3.00	8	4.0	4.0	30.3
	3.33	14	7.1	7.1	37.4
	3.67	8	4.0	4.0	41.4
	4.00	12	6.1	6.1	47.5
	4.33	13	6.6	6.6	54.0
	4.67	15	7.6	7.6	61.6
	5.00	14	7.1	7.1	68.7
	5.33	13	6.6	6.6	75.3
	5.67	17	8.6	8.6	83.8
	6.00	9	4.5	4.5	88.4
	6.33	6	3.0	3.0	91.4
	6.67	7	3.5	3.5	94.9
	7.00	10	5.1	5.1	100.0
	Total	198	100.0	100.0	

Mandate_Tot

		Frequency	Percent	Valid Percent	Cumulative Percent
Valid	1.00	24	12.1	12.1	12.1
	1.17	7	3.5	3.5	15.7
	1.33	3	1.5	1.5	17.2
	1.50	4	2.0	2.0	19.2
	1.67	3	1.5	1.5	20.7
	1.83	1	.5	.5	21.2
	2.00	6	3.0	3.0	24.2
	2.17	2	1.0	1.0	25.3
	2.33	3	1.5	1.5	26.8
	2.50	3	1.5	1.5	28.3
	2.67	3	1.5	1.5	29.8
	2.83	3	1.5	1.5	31.3
	3.00	2	1.0	1.0	32.3
	3.17	1	.5	.5	32.8
	3.33	1	.5	.5	33.3
	3.50	3	1.5	1.5	34.8
	3.67	2	1.0	1.0	35.9
	3.83	3	1.5	1.5	37.4
	4.00	3	1.5	1.5	38.9
	4.17	2	1.0	1.0	39.9
	4.33	2	1.0	1.0	40.9
	4.50	1	.5	.5	41.4
	4.67	4	2.0	2.0	43.4
	4.83	4	2.0	2.0	45.5
	5.00	3	1.5	1.5	47.0
	5.17	4	2.0	2.0	49.0
	5.33	6	3.0	3.0	52.0
	5.50	5	2.5	2.5	54.5
	5.67	5	2.5	2.5	57.1
	5.83	7	3.5	3.5	60.6
	6.00	8	4.0	4.0	64.6
	6.17	8	4.0	4.0	68.7
	6.33	7	3.5	3.5	72.2
	6.50	8	4.0	4.0	76.3
	6.67	13	6.6	6.6	82.8

Mandate_Tot

		Frequency	Percent	Valid Percent	Cumulative Percent
	6.83	3	1.5	1.5	84.3
	7.00	31	15.7	15.7	100.0
	Total	198	100.0	100.0	

Conspiracy_Tot

		Frequency	Percent	Valid Percent	Cumulative Percent
Valid	1.00	20	10.1	10.1	10.1
	1.11	18	9.1	9.1	19.2
	1.22	26	13.1	13.1	32.3
	1.33	23	11.6	11.6	43.9
	1.44	12	6.1	6.1	50.0
	1.56	12	6.1	6.1	56.1
	1.67	17	8.6	8.6	64.6
	1.78	3	1.5	1.5	66.2
	1.89	9	4.5	4.5	70.7
	2.00	10	5.1	5.1	75.8
	2.11	5	2.5	2.5	78.3
	2.22	3	1.5	1.5	79.8
	2.33	5	2.5	2.5	82.3
	2.44	6	3.0	3.0	85.4
	2.56	4	2.0	2.0	87.4
	2.67	5	2.5	2.5	89.9
	2.78	4	2.0	2.0	91.9
	2.89	1	.5	.5	92.4
	3.00	5	2.5	2.5	94.9
	3.11	4	2.0	2.0	97.0
	3.22	2	1.0	1.0	98.0
	3.44	2	1.0	1.0	99.0
	3.56	1	.5	.5	99.5
	3.67	1	.5	.5	100.0
	Total	198	100.0	100.0	

National_Tot

		Frequency	Percent	Valid Percent	Cumulative Percent
Valid	1.50	2	1.0	1.0	1.0
	2.00	2	1.0	1.0	2.0
	2.50	1	.5	.5	2.5
	3.00	4	2.0	2.0	4.5
	3.50	8	4.0	4.0	8.6
	4.00	13	6.6	6.6	15.2
	4.50	11	5.6	5.6	20.7
	5.00	18	9.1	9.1	29.8
	5.50	23	11.6	11.6	41.4
	6.00	28	14.1	14.1	55.6
	6.50	33	16.7	16.7	72.2
	7.00	55	27.8	27.8	100.0
	Total	198	100.0	100.0	

```
 **Contrast Codes and Mean Centering**

IF (Gender=1) GenderCC=1.
IF (Gender=2) GenderCC=-1.

IF (Race=1) RaceCC=1.
IF (Race>=2) RaceCC=-1.

COMPUTE Party0=PParty-2.86.
COMPUTE Ideology0=PIdeology-3.82.
COMPUTE SES0=SES-2.73.
COMPUTE MRN0=MRN-3.30.
COMPUTE National0=National_Tot-5.73.

**Interactions**

COMPUTE MRN0xRace=MRN0 * RaceCC.
COMPUTE MRN0xSES0=MRN0 * SES0.
COMPUTE MRN0xGender=MRN0 * GenderCC.
COMPUTE MRN0xParty0=MRN0 * Party0.
COMPUTE MRN0xIdeology0=MRN0*Ideology0.
COMPUTE MRN0xNational0=MRN0*National0.

**Regression Analyses**
```

```
**Including PParty

REGRESSION
  /MISSING LISTWISE
  /STATISTICS COEFF OUTS R ANOVA CHANGE ZPP
  /CRITERIA=PIN(.05) POUT(.10)
  /NOORIGIN
  /DEPENDENT Concern_Tot
  /METHOD=ENTER Party0
  /METHOD=ENTER GenderCC RaceCC SES0
  /METHOD=ENTER MRN0
  /METHOD=ENTER MRN0xRace MRN0xSES0 MRN0xGender MRN0xParty0.
```

Regression

Notes

Output Created		15-DEC-2021 13:11:10
Comments		
Input	Data	C:\Users\njs5478\Dropbox\HM and COVID\0. Revise and Resubmit\2. R and R Data\Study 2b\Study2b_Data.sav
	Active Dataset	DataSet1
	Filter	<none>
	Weight	<none>
	Split File	<none>
	N of Rows in Working Data File	198
Missing Value Handling	Definition of Missing	User-defined missing values are treated as missing.
	Cases Used	Statistics are based on cases with no missing values for any variable used.

Notes

Syntax		REGRESSION<br>/MISSING LISTWISE<br>/STATISTICS COEFF OUTS R ANOVA CHANGE ZPP<br>/CRITERIA=PIN(.05) POUT(.10)<br>/NOORIGIN<br>/DEPENDENT Concern_Tot<br>/METHOD=ENTER Party0<br>/METHOD=ENTER GenderCC RaceCC SES0<br>/METHOD=ENTER MRN0<br>/METHOD=ENTER MRN0xRace MRN0xSES0 MRN0xGender MRN0xParty0.
Resources	Processor Time	00:00:00.03
	Elapsed Time	00:00:00.03
	Memory Required	32640 bytes
	Additional Memory Required for Residual Plots	0 bytes

Variables Entered/Removed[a]

Model	Variables Entered	Variables Removed	Method
1	Party0[b]	.	Enter
2	GenderCC, SES0, RaceCC[b]	.	Enter
3	MRN0[b]	.	Enter
4	MRN0xSES0, MRN0xGender, MRN0xParty0, MRN0xRace[b]	.	Enter

a. Dependent Variable: Concern_Tot

b. All requested variables entered.

Model Summary

Model	R	R Square	Adjusted R Square	Std. Error of the Estimate	Change Statistics		
					R Square Change	F Change	df1
1	.485[a]	.235	.231	1.42985	.235	59.558	1
2	.528[b]	.279	.264	1.39911	.044	3.873	3
3	.530[c]	.281	.262	1.40071	.002	.566	1
4	.546[d]	.298	.264	1.39862	.017	1.142	4

Model Summary

Model	Change Statistics	
	df2	Sig. F Change
1	194	.000
2	191	.010
3	190	.453
4	186	.338

a. Predictors: (Constant), Party0

b. Predictors: (Constant), Party0, GenderCC, SES0, RaceCC

c. Predictors: (Constant), Party0, GenderCC, SES0, RaceCC, MRN0

d. Predictors: (Constant), Party0, GenderCC, SES0, RaceCC, MRN0, MRN0xSES0, MRN0xGender, MRN0xParty0, MRN0xRace

ANOVA[a]

Model		Sum of Squares	df	Mean Square	F	Sig.
1	Regression	121.766	1	121.766	59.558	.000[b]
	Residual	396.630	194	2.044		
	Total	518.396	195			
2	Regression	144.510	4	36.128	18.456	.000[c]
	Residual	373.885	191	1.958		
	Total	518.396	195			
3	Regression	145.620	5	29.124	14.844	.000[d]
	Residual	372.776	190	1.962		
	Total	518.396	195			
4	Regression	154.553	9	17.173	8.779	.000[e]
	Residual	363.843	186	1.956		
	Total	518.396	195			

a. Dependent Variable: Concern_Tot

b. Predictors: (Constant), Party0

c. Predictors: (Constant), Party0, GenderCC, SES0, RaceCC

d. Predictors: (Constant), Party0, GenderCC, SES0, RaceCC, MRN0

e. Predictors: (Constant), Party0, GenderCC, SES0, RaceCC, MRN0, MRN0xSES0, MRN0xGender, MRN0xParty0, MRN0xRace

Coefficients[a]

Model		Unstandardized Coefficients B	Unstandardized Coefficients Std. Error	Standardized Coefficients Beta	t	Sig.
1	(Constant)	4.296	.102		42.065	.000
	Party0	-.504	.065	-.485	-7.717	.000
2	(Constant)	4.263	.126		33.761	.000
	Party0	-.504	.065	-.484	-7.756	.000
	GenderCC	-.324	.102	-.199	-3.190	.002
	RaceCC	.050	.129	.025	.391	.696
	SES0	-.063	.118	-.033	-.533	.595
3	(Constant)	4.272	.127		33.624	.000
	Party0	-.479	.073	-.460	-6.553	.000
	GenderCC	-.298	.107	-.183	-2.769	.006
	RaceCC	.033	.131	.016	.254	.800
	SES0	-.053	.118	-.028	-.450	.653
	MRN0	-.087	.116	-.055	-.752	.453
4	(Constant)	4.202	.136		30.836	.000
	Party0	-.478	.075	-.460	-6.403	.000
	GenderCC	-.279	.109	-.171	-2.564	.011
	RaceCC	.045	.134	.022	.337	.736
	SES0	-.034	.119	-.018	-.287	.774
	MRN0	.013	.133	.008	.097	.923
	MRN0xRace	-.132	.122	-.084	-1.080	.282
	MRN0xSES0	.091	.113	.050	.807	.421
	MRN0xGender	-.114	.107	-.068	-1.063	.289
	MRN0xParty0	.118	.068	.114	1.744	.083

Coefficients[a]

Model		Correlations Zero-order	Partial	Part
1	(Constant)			
	Party0	-.485	-.485	-.485
2	(Constant)			
	Party0	-.485	-.489	-.477
	GenderCC	-.215	-.225	-.196
	RaceCC	-.030	.028	.024
	SES0	-.062	-.039	-.033
3	(Constant)			
	Party0	-.485	-.429	-.403
	GenderCC	-.215	-.197	-.170
	RaceCC	-.030	.018	.016
	SES0	-.062	-.033	-.028
	MRN0	-.312	-.054	-.046
4	(Constant)			
	Party0	-.485	-.425	-.393
	GenderCC	-.215	-.185	-.158
	RaceCC	-.030	.025	.021
	SES0	-.062	-.021	-.018
	MRN0	-.312	.007	.006
	MRN0xRace	-.277	-.079	-.066
	MRN0xSES0	.046	.059	.050
	MRN0xGender	.021	-.078	-.065
	MRN0xParty0	.146	.127	.107

a. Dependent Variable: Concern_Tot

Excluded Variables[a]

Model		Beta In	t	Sig.	Partial Correlation	Collinearity Statistics Tolerance
1	GenderCC	-.206[b]	-3.360	.001	-.235	1.000
	RaceCC	.055[b]	.856	.393	.062	.971
	SES0	-.050[b]	-.789	.431	-.057	.999
	MRN0	-.134[b]	-1.955	.052	-.139	.827
	MRN0xRace	-.128[b]	-1.932	.055	-.138	.886
	MRN0xSES0	.020[b]	.318	.751	.023	.997
	MRN0xGender	-.041[b]	-.647	.518	-.047	.984
	MRN0xParty0	.120[b]	1.927	.055	.137	.997
2	MRN0	-.055[c]	-.752	.453	-.054	.695
	MRN0xRace	-.071[c]	-1.041	.299	-.075	.807
	MRN0xSES0	.031[c]	.507	.613	.037	.989
	MRN0xGender	-.043[c]	-.686	.494	-.050	.966
	MRN0xParty0	.095[c]	1.530	.128	.110	.970
3	MRN0xRace	-.060[d]	-.787	.432	-.057	.648
	MRN0xSES0	.035[d]	.563	.574	.041	.983
	MRN0xGender	-.040[d]	-.637	.525	-.046	.962
	MRN0xParty0	.090[d]	1.419	.157	.103	.942

a. Dependent Variable: Concern_Tot

b. Predictors in the Model: (Constant), Party0

c. Predictors in the Model: (Constant), Party0, GenderCC, SES0, RaceCC

d. Predictors in the Model: (Constant), Party0, GenderCC, SES0, RaceCC, MRN0

```
REGRESSION
 /MISSING LISTWISE
 /STATISTICS COEFF OUTS R ANOVA CHANGE ZPP
 /CRITERIA=PIN(.05) POUT(.10)
 /NOORIGIN
 /DEPENDENT Finance_Tot
 /METHOD=ENTER Party0
 /METHOD=ENTER GenderCC RaceCC SES0
 /METHOD=ENTER MRN0
 /METHOD=ENTER MRN0xRace MRN0xSES0 MRN0xGender MRN0xParty0.
```

Regression

Notes

Output Created		15-DEC-2021 13:11:10
Comments		
Input	Data	C:\Users\njs5478\Dropbox\HM and COVID\0. Revise and Resubmit\2. R and R Data\Study 2b\Study2b_Data.sav
	Active Dataset	DataSet1
	Filter	<none>
	Weight	<none>
	Split File	<none>
	N of Rows in Working Data File	198
Missing Value Handling	Definition of Missing	User-defined missing values are treated as missing.
	Cases Used	Statistics are based on cases with no missing values for any variable used.
Syntax		REGRESSION<br>/MISSING LISTWISE<br>/STATISTICS COEFF OUTS R ANOVA CHANGE ZPP<br>/CRITERIA=PIN(.05) POUT(.10)<br>/NOORIGIN<br>/DEPENDENT Finance_Tot<br>/METHOD=ENTER Party0<br>/METHOD=ENTER GenderCC RaceCC SES0<br>/METHOD=ENTER MRN0<br>/METHOD=ENTER MRN0xRace MRN0xSES0 MRN0xGender MRN0xParty0.
Resources	Processor Time	00:00:00.03
	Elapsed Time	00:00:00.13

Notes

	Memory Required	32640 bytes
	Additional Memory Required for Residual Plots	0 bytes

Variables Entered/Removed[a]

Model	Variables Entered	Variables Removed	Method
1	Party0[b]	.	Enter
2	GenderCC, SES0, RaceCC[b]	.	Enter
3	MRN0[b]	.	Enter
4	MRN0xSES0, MRN0xGender, MRN0xParty0, MRN0xRace[b]	.	Enter

a. Dependent Variable: Finance_Tot

b. All requested variables entered.

Model Summary

Model	R	R Square	Adjusted R Square	Std. Error of the Estimate	Change Statistics		
					R Square Change	F Change	df1
1	.029[a]	.001	-.004	1.99120	.001	.164	1
2	.341[b]	.116	.098	1.88745	.115	8.305	3
3	.354[c]	.125	.102	1.88246	.009	2.014	1
4	.355[d]	.126	.084	1.90213	.000	.023	4

Model Summary

Model	Change Statistics	
	df2	Sig. F Change
1	194	.686
2	191	.000
3	190	.158
4	186	.999

a. Predictors: (Constant), Party0

b. Predictors: (Constant), Party0, GenderCC, SES0, RaceCC

c. Predictors: (Constant), Party0, GenderCC, SES0, RaceCC, MRN0

d. Predictors: (Constant), Party0, GenderCC, SES0, RaceCC, MRN0, MRN0xSES0, MRN0xGender, MRN0xParty0, MRN0xRace

ANOVA[a]

Model		Sum of Squares	df	Mean Square	F	Sig.
1	Regression	.650	1	.650	.164	.686[b]
	Residual	769.187	194	3.965		
	Total	769.837	195			
2	Regression	89.404	4	22.351	6.274	.000[c]
	Residual	680.432	191	3.562		
	Total	769.837	195			
3	Regression	96.541	5	19.308	5.449	.000[d]
	Residual	673.296	190	3.544		
	Total	769.837	195			
4	Regression	96.869	9	10.763	2.975	.002[e]
	Residual	672.967	186	3.618		
	Total	769.837	195			

a. Dependent Variable: Finance_Tot

b. Predictors: (Constant), Party0

c. Predictors: (Constant), Party0, GenderCC, SES0, RaceCC

d. Predictors: (Constant), Party0, GenderCC, SES0, RaceCC, MRN0

e. Predictors: (Constant), Party0, GenderCC, SES0, RaceCC, MRN0, MRN0xSES0, MRN0xGender, MRN0xParty0, MRN0xRace

Coefficients[a]

Model		Unstandardized Coefficients B	Unstandardized Coefficients Std. Error	Standardized Coefficients Beta	t	Sig.
1	(Constant)	3.796	.142		26.690	.000
	Party0	-.037	.091	-.029	-.405	.686
2	(Constant)	3.728	.170		21.888	.000
	Party0	-.033	.088	-.026	-.375	.708
	GenderCC	-.376	.137	-.190	-2.748	.007
	RaceCC	.106	.173	.043	.613	.541
	SES0	-.601	.159	-.259	-3.788	.000
3	(Constant)	3.703	.171		21.687	.000
	Party0	-.096	.098	-.076	-.981	.328
	GenderCC	-.443	.144	-.224	-3.067	.002
	RaceCC	.150	.176	.060	.852	.395
	SES0	-.625	.159	-.269	-3.926	.000
	MRN0	.222	.156	.115	1.419	.158
4	(Constant)	3.714	.185		20.037	.000
	Party0	-.099	.102	-.078	-.978	.329
	GenderCC	-.443	.148	-.223	-2.998	.003
	RaceCC	.157	.182	.063	.865	.388
	SES0	-.623	.162	-.268	-3.854	.000
	MRN0	.227	.181	.118	1.250	.213
	MRN0xRace	-.010	.167	-.005	-.060	.952
	MRN0xSES0	.010	.154	.005	.066	.948
	MRN0xGender	-.039	.146	-.019	-.268	.789
	MRN0xParty0	-.006	.092	-.004	-.060	.952

Coefficients[a]

Model		Correlations Zero-order	Partial	Part
1	(Constant)			
	Party0	-.029	-.029	-.029
2	(Constant)			
	Party0	-.029	-.027	-.025
	GenderCC	-.219	-.195	-.187
	RaceCC	.060	.044	.042
	SES0	-.275	-.264	-.258
3	(Constant)			
	Party0	-.029	-.071	-.067
	GenderCC	-.219	-.217	-.208
	RaceCC	.060	.062	.058
	SES0	-.275	-.274	-.266
	MRN0	-.030	.102	.096
4	(Constant)			
	Party0	-.029	-.072	-.067
	GenderCC	-.219	-.215	-.206
	RaceCC	.060	.063	.059
	SES0	-.275	-.272	-.264
	MRN0	-.030	.091	.086
	MRN0xRace	-.050	-.004	-.004
	MRN0xSES0	-.001	.005	.004
	MRN0xGender	-.020	-.020	-.018
	MRN0xParty0	.019	-.004	-.004

a. Dependent Variable: Finance_Tot

Excluded Variables[a]

Model		Beta In	t	Sig.	Partial Correlation	Collinearity Statistics Tolerance
1	GenderCC	-.219[b]	-3.112	.002	-.219	1.000
	RaceCC	.067[b]	.924	.356	.066	.971
	SES0	-.274[b]	-3.965	.000	-.274	.999
	MRN0	-.021[b]	-.269	.788	-.019	.827
	MRN0xRace	-.045[b]	-.595	.553	-.043	.886
	MRN0xSES0	-.003[b]	-.040	.968	-.003	.997
	MRN0xGender	-.024[b]	-.335	.738	-.024	.984
	MRN0xParty0	.017[b]	.236	.814	.017	.997
2	MRN0	.115[c]	1.419	.158	.102	.695
	MRN0xRace	.046[c]	.600	.549	.044	.807
	MRN0xSES0	.012[c]	.175	.861	.013	.989
	MRN0xGender	-.013[c]	-.187	.852	-.014	.966
	MRN0xParty0	-.025[c]	-.362	.718	-.026	.970
3	MRN0xRace	-.003[d]	-.030	.976	-.002	.648
	MRN0xSES0	.005[d]	.072	.943	.005	.983
	MRN0xGender	-.019[d]	-.281	.779	-.020	.962
	MRN0xParty0	-.009[d]	-.125	.901	-.009	.942

a. Dependent Variable: Finance_Tot

b. Predictors in the Model: (Constant), Party0

c. Predictors in the Model: (Constant), Party0, GenderCC, SES0, RaceCC

d. Predictors in the Model: (Constant), Party0, GenderCC, SES0, RaceCC, MRN0

```
REGRESSION
 /MISSING LISTWISE
 /STATISTICS COEFF OUTS R ANOVA CHANGE ZPP
 /CRITERIA=PIN(.05) POUT(.10)
 /NOORIGIN
 /DEPENDENT Psychology_Tot
 /METHOD=ENTER Party0
 /METHOD=ENTER GenderCC RaceCC SES0
 /METHOD=ENTER MRN0
 /METHOD=ENTER MRN0xRace MRN0xSES0 MRN0xGender MRN0xParty0.
```

Regression

Notes

Output Created		15-DEC-2021 13:11:10
Comments		
Input	Data	C: \Users\njs5478\Dropbox\HM and COVID\0. Revise and Resubmit\2. R and R Data\Study 2b\Study2b_Data.sav
	Active Dataset	DataSet1
	Filter	<none>
	Weight	<none>
	Split File	<none>
	N of Rows in Working Data File	198
Missing Value Handling	Definition of Missing	User-defined missing values are treated as missing.
	Cases Used	Statistics are based on cases with no missing values for any variable used.
Syntax		REGRESSION /MISSING LISTWISE /STATISTICS COEFF OUTS R ANOVA CHANGE ZPP /CRITERIA=PIN(.05) POUT(.10) /NOORIGIN /DEPENDENT Psychology_Tot /METHOD=ENTER Party0 /METHOD=ENTER GenderCC RaceCC SES0 /METHOD=ENTER MRN0 /METHOD=ENTER MRN0xRace MRN0xSES0 MRN0xGender MRN0xParty0.
Resources	Processor Time	00:00:00.02
	Elapsed Time	00:00:00.05

Notes

	Memory Required	32640 bytes
	Additional Memory Required for Residual Plots	0 bytes

Variables Entered/Removed[a]

Model	Variables Entered	Variables Removed	Method
1	Party0[b]	.	Enter
2	GenderCC, SES0, RaceCC[b]	.	Enter
3	MRN0[b]	.	Enter
4	MRN0xSES0, MRN0xGender, MRN0xParty0, MRN0xRace[b]	.	Enter

a. Dependent Variable: Psychology_Tot

b. All requested variables entered.

Model Summary

Model	R	R Square	Adjusted R Square	Std. Error of the Estimate	Change Statistics		
					R Square Change	F Change	df1
1	.354[a]	.125	.121	1.60103	.125	27.738	1
2	.467[b]	.218	.202	1.52513	.093	7.597	3
3	.469[c]	.220	.199	1.52780	.001	.332	1
4	.501[d]	.251	.215	1.51311	.031	1.927	4

Model Summary

Model	Change Statistics	
	df2	Sig. F Change
1	194	.000
2	191	.000
3	190	.565
4	186	.108

a. Predictors: (Constant), Party0

b. Predictors: (Constant), Party0, GenderCC, SES0, RaceCC

c. Predictors: (Constant), Party0, GenderCC, SES0, RaceCC, MRN0

d. Predictors: (Constant), Party0, GenderCC, SES0, RaceCC, MRN0, MRN0xSES0, MRN0xGender, MRN0xParty0, MRN0xRace

ANOVA[a]

Model		Sum of Squares	df	Mean Square	F	Sig.
1	Regression	71.100	1	71.100	27.738	.000[b]
	Residual	497.282	194	2.563		
	Total	568.383	195			
2	Regression	124.114	4	31.029	13.340	.000[c]
	Residual	444.268	191	2.326		
	Total	568.383	195			
3	Regression	124.889	5	24.978	10.701	.000[d]
	Residual	443.494	190	2.334		
	Total	568.383	195			
4	Regression	142.537	9	15.837	6.917	.000[e]
	Residual	425.845	186	2.289		
	Total	568.383	195			

a. Dependent Variable: Psychology_Tot

b. Predictors: (Constant), Party0

c. Predictors: (Constant), Party0, GenderCC, SES0, RaceCC

d. Predictors: (Constant), Party0, GenderCC, SES0, RaceCC, MRN0

e. Predictors: (Constant), Party0, GenderCC, SES0, RaceCC, MRN0, MRN0xSES0, MRN0xGender, MRN0xParty0, MRN0xRace

Coefficients[a]

Model		Unstandardized Coefficients B	Unstandardized Coefficients Std. Error	Standardized Coefficients Beta	t	Sig.
1	(Constant)	4.113	.114		35.968	.000
	Party0	-.385	.073	-.354	-5.267	.000
2	(Constant)	3.974	.138		28.875	.000
	Party0	-.397	.071	-.364	-5.599	.000
	GenderCC	-.400	.111	-.235	-3.618	.000
	RaceCC	.225	.140	.105	1.603	.111
	SES0	-.247	.128	-.124	-1.926	.056
3	(Constant)	3.966	.139		28.616	.000
	Party0	-.418	.080	-.383	-5.237	.000
	GenderCC	-.422	.117	-.248	-3.603	.000
	RaceCC	.239	.143	.112	1.676	.095
	SES0	-.255	.129	-.128	-1.972	.050
	MRN0	.073	.127	.044	.576	.565
4	(Constant)	3.844	.147		26.076	.000
	Party0	-.416	.081	-.381	-5.144	.000
	GenderCC	-.395	.118	-.232	-3.358	.001
	RaceCC	.242	.145	.113	1.671	.096
	SES0	-.232	.129	-.116	-1.803	.073
	MRN0	.207	.144	.126	1.437	.152
	MRN0xRace	-.154	.133	-.093	-1.165	.245
	MRN0xSES0	.055	.123	.029	.449	.654
	MRN0xGender	-.088	.116	-.050	-.755	.451
	MRN0xParty0	.192	.073	.177	2.627	.009

Coefficients[a]

Model		Correlations		
		Zero-order	Partial	Part
1	(Constant)			
	Party0	-.354	-.354	-.354
2	(Constant)			
	Party0	-.354	-.375	-.358
	GenderCC	-.268	-.253	-.231
	RaceCC	.075	.115	.103
	SES0	-.151	-.138	-.123
3	(Constant)			
	Party0	-.354	-.355	-.336
	GenderCC	-.268	-.253	-.231
	RaceCC	.075	.121	.107
	SES0	-.151	-.142	-.126
	MRN0	-.225	.042	.037
4	(Constant)			
	Party0	-.354	-.353	-.326
	GenderCC	-.268	-.239	-.213
	RaceCC	.075	.122	.106
	SES0	-.151	-.131	-.114
	MRN0	-.225	.105	.091
	MRN0xRace	-.223	-.085	-.074
	MRN0xSES0	.017	.033	.028
	MRN0xGender	.042	-.055	-.048
	MRN0xParty0	.209	.189	.167

a. Dependent Variable: Psychology_Tot

Excluded Variables[a]

Model		Beta In	t	Sig.	Partial Correlation	Collinearity Statistics Tolerance
1	GenderCC	-.261[b]	-4.044	.000	-.279	1.000
	RaceCC	.139[b]	2.059	.041	.147	.971
	SES0	-.142[b]	-2.136	.034	-.152	.999
	MRN0	-.095[b]	-1.285	.200	-.092	.827
	MRN0xRace	-.116[b]	-1.638	.103	-.117	.886
	MRN0xSES0	-.002[b]	-.030	.976	-.002	.997
	MRN0xGender	-.002[b]	-.035	.972	-.003	.984
	MRN0xParty0	.190[b]	2.876	.004	.203	.997
2	MRN0	.044[c]	.576	.565	.042	.695
	MRN0xRace	-.036[c]	-.500	.618	-.036	.807
	MRN0xSES0	.009[c]	.135	.892	.010	.989
	MRN0xGender	-.007[c]	-.113	.910	-.008	.966
	MRN0xParty0	.147[c]	2.291	.023	.164	.970
3	MRN0xRace	-.067[d]	-.843	.400	-.061	.648
	MRN0xSES0	.006[d]	.093	.926	.007	.983
	MRN0xGender	-.010[d]	-.151	.880	-.011	.962
	MRN0xParty0	.158[d]	2.425	.016	.174	.942

a. Dependent Variable: Psychology_Tot

b. Predictors in the Model: (Constant), Party0

c. Predictors in the Model: (Constant), Party0, GenderCC, SES0, RaceCC

d. Predictors in the Model: (Constant), Party0, GenderCC, SES0, RaceCC, MRN0

```
REGRESSION
 /MISSING LISTWISE
 /STATISTICS COEFF OUTS R ANOVA CHANGE ZPP
 /CRITERIA=PIN(.05) POUT(.10)
 /NOORIGIN
 /DEPENDENT Risk_Rules
 /METHOD=ENTER Party0
 /METHOD=ENTER GenderCC RaceCC SES0
 /METHOD=ENTER MRN0
 /METHOD=ENTER MRN0xRace MRN0xSES0 MRN0xGender MRN0xParty0.
```

Regression

Notes

Output Created		15-DEC-2021 13:11:10
Comments		
Input	Data	C:\Users\njs5478\Dropbox\HM and COVID\0. Revise and Resubmit\2. R and R Data\Study 2b\Study2b_Data.sav
	Active Dataset	DataSet1
	Filter	<none>
	Weight	<none>
	Split File	<none>
	N of Rows in Working Data File	198
Missing Value Handling	Definition of Missing	User-defined missing values are treated as missing.
	Cases Used	Statistics are based on cases with no missing values for any variable used.
Syntax		REGRESSION /MISSING LISTWISE /STATISTICS COEFF OUTS R ANOVA CHANGE ZPP /CRITERIA=PIN(.05) POUT(.10) /NOORIGIN /DEPENDENT Risk_Rules /METHOD=ENTER Party0 /METHOD=ENTER GenderCC RaceCC SES0 /METHOD=ENTER MRN0 /METHOD=ENTER MRN0xRace MRN0xSES0 MRN0xGender MRN0xParty0.
Resources	Processor Time	00:00:00.03
	Elapsed Time	00:00:00.05

Notes

	Memory Required	32640 bytes
	Additional Memory Required for Residual Plots	0 bytes

Variables Entered/Removed[a]

Model	Variables Entered	Variables Removed	Method
1	Party0[b]	.	Enter
2	GenderCC, SES0, RaceCC[b]	.	Enter
3	MRN0[b]	.	Enter
4	MRN0xSES0, MRN0xGender, MRN0xParty0, MRN0xRace[b]	.	Enter

a. Dependent Variable: Risk_Rules

b. All requested variables entered.

Model Summary

Model	R	R Square	Adjusted R Square	Std. Error of the Estimate	Change Statistics		
					R Square Change	F Change	df1
1	.532[a]	.284	.280	.93951	.284	76.770	1
2	.548[b]	.300	.285	.93606	.016	1.478	3
3	.573[c]	.328	.310	.91932	.028	8.016	1
4	.582[d]	.338	.306	.92214	.010	.710	4

Model Summary

Model	Change Statistics	
	df2	Sig. F Change
1	194	.000
2	191	.222
3	190	.005
4	186	.586

a. Predictors: (Constant), Party0

b. Predictors: (Constant), Party0, GenderCC, SES0, RaceCC

c. Predictors: (Constant), Party0, GenderCC, SES0, RaceCC, MRN0

d. Predictors: (Constant), Party0, GenderCC, SES0, RaceCC, MRN0, MRN0xSES0, MRN0xGender, MRN0xParty0, MRN0xRace

ANOVA[a]

Model		Sum of Squares	df	Mean Square	F	Sig.
1	Regression	67.764	1	67.764	76.770	.000[b]
	Residual	171.241	194	.883		
	Total	239.005	195			
2	Regression	71.650	4	17.912	20.443	.000[c]
	Residual	167.355	191	.876		
	Total	239.005	195			
3	Regression	78.425	5	15.685	18.559	.000[d]
	Residual	160.580	190	.845		
	Total	239.005	195			
4	Regression	80.840	9	8.982	10.563	.000[e]
	Residual	158.165	186	.850		
	Total	239.005	195			

a. Dependent Variable: Risk_Rules

b. Predictors: (Constant), Party0

c. Predictors: (Constant), Party0, GenderCC, SES0, RaceCC

d. Predictors: (Constant), Party0, GenderCC, SES0, RaceCC, MRN0

e. Predictors: (Constant), Party0, GenderCC, SES0, RaceCC, MRN0, MRN0xSES0, MRN0xGender, MRN0xParty0, MRN0xRace

Coefficients[a]

Model		Unstandardized Coefficients B	Std. Error	Standardized Coefficients Beta	t	Sig.
1	(Constant)	3.305	.067		49.254	.000
	Party0	.376	.043	.532	8.762	.000
2	(Constant)	3.325	.084		39.359	.000
	Party0	.378	.043	.534	8.686	.000
	GenderCC	.135	.068	.123	1.994	.048
	RaceCC	-.030	.086	-.022	-.347	.729
	SES0	-.025	.079	-.020	-.324	.746
3	(Constant)	3.301	.083		39.580	.000
	Party0	.316	.048	.447	6.582	.000
	GenderCC	.070	.071	.064	.999	.319
	RaceCC	.012	.086	.009	.144	.886
	SES0	-.049	.078	-.038	-.626	.532
	MRN0	.216	.076	.202	2.831	.005
4	(Constant)	3.295	.090		36.672	.000
	Party0	.310	.049	.439	6.298	.000
	GenderCC	.062	.072	.056	.871	.385
	RaceCC	-.009	.088	-.006	-.100	.920
	SES0	-.054	.078	-.042	-.690	.491
	MRN0	.180	.088	.168	2.044	.042
	MRN0xRace	.094	.081	.088	1.166	.245
	MRN0xSES0	-.053	.075	-.043	-.709	.479
	MRN0xGender	.046	.071	.041	.654	.514
	MRN0xParty0	.026	.045	.037	.588	.557

Coefficients[a]

Model		Correlations Zero-order	Partial	Part
1	(Constant)			
	Party0	.532	.532	.532
2	(Constant)			
	Party0	.532	.532	.526
	GenderCC	.134	.143	.121
	RaceCC	.051	-.025	-.021
	SES0	.005	-.023	-.020
3	(Constant)			
	Party0	.532	.431	.391
	GenderCC	.134	.072	.059
	RaceCC	.051	.010	.009
	SES0	.005	-.045	-.037
	MRN0	.403	.201	.168
4	(Constant)			
	Party0	.532	.419	.376
	GenderCC	.134	.064	.052
	RaceCC	.051	-.007	-.006
	SES0	.005	-.051	-.041
	MRN0	.403	.148	.122
	MRN0xRace	.331	.085	.070
	MRN0xSES0	-.055	-.052	-.042
	MRN0xGender	-.023	.048	.039
	MRN0xParty0	-.020	.043	.035

a. Dependent Variable: Risk_Rules

Excluded Variables[a]

Model		Beta In	t	Sig.	Partial Correlation	Collinearity Statistics Tolerance
1	GenderCC	.124[b]	2.059	.041	.147	1.000
	RaceCC	-.041[b]	-.660	.510	-.047	.971
	SES0	-.010[b]	-.156	.876	-.011	.999
	MRN0	.220[b]	3.374	.001	.236	.827
	MRN0xRace	.171[b]	2.690	.008	.190	.886
	MRN0xSES0	-.026[b]	-.434	.665	-.031	.997
	MRN0xGender	.045[b]	.727	.468	.052	.984
	MRN0xParty0	.009[b]	.141	.888	.010	.997
2	MRN0	.202[c]	2.831	.005	.201	.695
	MRN0xRace	.150[c]	2.253	.025	.161	.807
	MRN0xSES0	-.032[c]	-.529	.597	-.038	.989
	MRN0xGender	.050[c]	.805	.422	.058	.966
	MRN0xParty0	.025[c]	.406	.685	.029	.970
3	MRN0xRace	.083[d]	1.131	.260	.082	.648
	MRN0xSES0	-.045[d]	-.749	.455	-.054	.983
	MRN0xGender	.039[d]	.635	.526	.046	.962
	MRN0xParty0	.056[d]	.907	.365	.066	.942

a. Dependent Variable: Risk_Rules

b. Predictors in the Model: (Constant), Party0

c. Predictors in the Model: (Constant), Party0, GenderCC, SES0, RaceCC

d. Predictors in the Model: (Constant), Party0, GenderCC, SES0, RaceCC, MRN0

```
REGRESSION
 /MISSING LISTWISE
 /STATISTICS COEFF OUTS R ANOVA CHANGE ZPP
 /CRITERIA=PIN(.05) POUT(.10)
 /NOORIGIN
 /DEPENDENT Mandate_Tot
 /METHOD=ENTER Party0
 /METHOD=ENTER GenderCC RaceCC SES0
 /METHOD=ENTER MRN0
 /METHOD=ENTER MRN0xRace MRN0xSES0 MRN0xGender MRN0xParty0.
```

Regression

Notes

Output Created		15-DEC-2021 13:11:10
Comments		
Input	Data	C:\Users\njs5478\Dropbox\HM and COVID\0. Revise and Resubmit\2. R and R Data\Study 2b\Study2b_Data.sav
	Active Dataset	DataSet1
	Filter	<none>
	Weight	<none>
	Split File	<none>
	N of Rows in Working Data File	198
Missing Value Handling	Definition of Missing	User-defined missing values are treated as missing.
	Cases Used	Statistics are based on cases with no missing values for any variable used.
Syntax		REGRESSION /MISSING LISTWISE /STATISTICS COEFF OUTS R ANOVA CHANGE ZPP /CRITERIA=PIN(.05) POUT(.10) /NOORIGIN /DEPENDENT Mandate_Tot /METHOD=ENTER Party0 /METHOD=ENTER GenderCC RaceCC SES0 /METHOD=ENTER MRN0 /METHOD=ENTER MRN0xRace MRN0xSES0 MRN0xGender MRN0xParty0.
Resources	Processor Time	00:00:00.13
	Elapsed Time	00:00:00.23

Notes

	Memory Required	32640 bytes
	Additional Memory Required for Residual Plots	0 bytes

Variables Entered/Removed[a]

Model	Variables Entered	Variables Removed	Method
1	Party0[b]	.	Enter
2	GenderCC, SES0, RaceCC[b]	.	Enter
3	MRN0[b]	.	Enter
4	MRN0xSES0, MRN0xGender, MRN0xParty0, MRN0xRace[b]	.	Enter

a. Dependent Variable: Mandate_Tot

b. All requested variables entered.

Model Summary

Model	R	R Square	Adjusted R Square	Std. Error of the Estimate	Change Statistics		
					R Square Change	F Change	df1
1	.725[a]	.526	.523	1.54278	.526	215.194	1
2	.727[b]	.528	.518	1.55144	.002	.281	3
3	.732[c]	.535	.523	1.54322	.007	3.040	1
4	.737[d]	.543	.521	1.54716	.007	.758	4

Model Summary

Model	Change Statistics	
	df2	Sig. F Change
1	194	.000
2	191	.839
3	190	.083
4	186	.554

a. Predictors: (Constant), Party0

b. Predictors: (Constant), Party0, GenderCC, SES0, RaceCC

c. Predictors: (Constant), Party0, GenderCC, SES0, RaceCC, MRN0

d. Predictors: (Constant), Party0, GenderCC, SES0, RaceCC, MRN0, MRN0xSES0, MRN0xGender, MRN0xParty0, MRN0xRace

ANOVA[a]

Model		Sum of Squares	df	Mean Square	F	Sig.
1	Regression	512.201	1	512.201	215.194	.000[b]
	Residual	461.754	194	2.380		
	Total	973.955	195			
2	Regression	514.227	4	128.557	53.411	.000[c]
	Residual	459.728	191	2.407		
	Total	973.955	195			
3	Regression	521.468	5	104.294	43.793	.000[d]
	Residual	452.487	190	2.382		
	Total	973.955	195			
4	Regression	528.727	9	58.747	24.542	.000[e]
	Residual	445.228	186	2.394		
	Total	973.955	195			

a. Dependent Variable: Mandate_Tot

b. Predictors: (Constant), Party0

c. Predictors: (Constant), Party0, GenderCC, SES0, RaceCC

d. Predictors: (Constant), Party0, GenderCC, SES0, RaceCC, MRN0

e. Predictors: (Constant), Party0, GenderCC, SES0, RaceCC, MRN0, MRN0xSES0, MRN0xGender, MRN0xParty0, MRN0xRace

Coefficients[a]

Model		Unstandardized Coefficients B	Unstandardized Coefficients Std. Error	Standardized Coefficients Beta	t	Sig.
1	(Constant)	4.517	.110		40.989	.000
	Party0	-1.035	.071	-.725	-14.670	.000
2	(Constant)	4.492	.140		32.085	.000
	Party0	-1.040	.072	-.729	-14.433	.000
	GenderCC	.021	.113	.010	.190	.849
	RaceCC	.042	.143	.015	.293	.770
	SES0	.108	.130	.042	.832	.407
3	(Constant)	4.517	.140		32.266	.000
	Party0	-.976	.081	-.684	-12.121	.000
	GenderCC	.089	.118	.040	.748	.455
	RaceCC	-.002	.144	-.001	-.013	.990
	SES0	.132	.131	.051	1.015	.311
	MRN0	-.223	.128	-.103	-1.744	.083
4	(Constant)	4.433	.151		29.407	.000
	Party0	-.965	.083	-.677	-11.684	.000
	GenderCC	.102	.120	.046	.848	.397
	RaceCC	-.014	.148	-.005	-.094	.925
	SES0	.143	.131	.055	1.086	.279
	MRN0	-.155	.147	-.072	-1.048	.296
	MRN0xRace	-.090	.135	-.042	-.665	.507
	MRN0xSES0	.116	.125	.047	.927	.355
	MRN0xGender	-.013	.119	-.006	-.108	.914
	MRN0xParty0	.111	.075	.078	1.477	.141

Coefficients[a]

Model		Correlations		
		Zero-order	Partial	Part
1	(Constant)			
	Party0	-.725	-.725	-.725
2	(Constant)			
	Party0	-.725	-.722	-.718
	GenderCC	-.003	.014	.009
	RaceCC	-.110	.021	.015
	SES0	.023	.060	.041
3	(Constant)			
	Party0	-.725	-.660	-.599
	GenderCC	-.003	.054	.037
	RaceCC	-.110	-.001	-.001
	SES0	.023	.073	.050
	MRN0	-.369	-.125	-.086
4	(Constant)			
	Party0	-.725	-.651	-.579
	GenderCC	-.003	.062	.042
	RaceCC	-.110	-.007	-.005
	SES0	.023	.079	.054
	MRN0	-.369	-.077	-.052
	MRN0xRace	-.293	-.049	-.033
	MRN0xSES0	.072	.068	.046
	MRN0xGender	.102	-.008	-.005
	MRN0xParty0	.117	.108	.073

a. Dependent Variable: Mandate_Tot

Excluded Variables[a]

Model		Beta In	t	Sig.	Partial Correlation	Collinearity Statistics Tolerance
1	GenderCC	.011[b]	.221	.825	.016	1.000
	RaceCC	.014[b]	.283	.777	.020	.971
	SES0	.043[b]	.861	.390	.062	.999
	MRN0	-.081[b]	-1.495	.137	-.107	.827
	MRN0xRace	-.054[b]	-1.023	.308	-.073	.886
	MRN0xSES0	.032[b]	.651	.516	.047	.997
	MRN0xGender	.011[b]	.215	.830	.015	.984
	MRN0xParty0	.077[b]	1.568	.118	.112	.997
2	MRN0	-.103[c]	-1.744	.083	-.125	.695
	MRN0xRace	-.068[c]	-1.224	.222	-.088	.807
	MRN0xSES0	.030[c]	.601	.548	.044	.989
	MRN0xGender	.006[c]	.126	.900	.009	.966
	MRN0xParty0	.081[c]	1.614	.108	.116	.970
3	MRN0xRace	-.031[d]	-.507	.613	-.037	.648
	MRN0xSES0	.037[d]	.735	.464	.053	.983
	MRN0xGender	.012[d]	.241	.810	.018	.962
	MRN0xParty0	.068[d]	1.342	.181	.097	.942

a. Dependent Variable: Mandate_Tot

b. Predictors in the Model: (Constant), Party0

c. Predictors in the Model: (Constant), Party0, GenderCC, SES0, RaceCC

d. Predictors in the Model: (Constant), Party0, GenderCC, SES0, RaceCC, MRN0

```
REGRESSION
 /MISSING LISTWISE
 /STATISTICS COEFF OUTS R ANOVA CHANGE ZPP
 /CRITERIA=PIN(.05) POUT(.10)
 /NOORIGIN
 /DEPENDENT Conspiracy_Tot
 /METHOD=ENTER Party0
 /METHOD=ENTER GenderCC RaceCC SES0
 /METHOD=ENTER MRN0
 /METHOD=ENTER MRN0xRace MRN0xSES0 MRN0xGender MRN0xParty0.
```

Regression

Notes

Output Created		15-DEC-2021 13:11:10
Comments		
Input	Data	C:\Users\njs5478\Dropbox\HM and COVID\0. Revise and Resubmit\2. R and R Data\Study 2b\Study2b_Data.sav
	Active Dataset	DataSet1
	Filter	<none>
	Weight	<none>
	Split File	<none>
	N of Rows in Working Data File	198
Missing Value Handling	Definition of Missing	User-defined missing values are treated as missing.
	Cases Used	Statistics are based on cases with no missing values for any variable used.
Syntax		REGRESSION /MISSING LISTWISE /STATISTICS COEFF OUTS R ANOVA CHANGE ZPP /CRITERIA=PIN(.05) POUT(.10) /NOORIGIN /DEPENDENT Conspiracy_Tot /METHOD=ENTER Party0 /METHOD=ENTER GenderCC RaceCC SES0 /METHOD=ENTER MRN0 /METHOD=ENTER MRN0xRace MRN0xSES0 MRN0xGender MRN0xParty0.
Resources	Processor Time	00:00:00.03
	Elapsed Time	00:00:00.05

Notes

	Memory Required	32640 bytes
	Additional Memory Required for Residual Plots	0 bytes

Variables Entered/Removed[a]

Model	Variables Entered	Variables Removed	Method
1	Party0[b]	.	Enter
2	GenderCC, SES0, RaceCC[b]	.	Enter
3	MRN0[b]	.	Enter
4	MRN0xSES0, MRN0xGender, MRN0xParty0, MRN0xRace[b]	.	Enter

a. Dependent Variable: Conspiracy_Tot

b. All requested variables entered.

Model Summary

Model	R	R Square	Adjusted R Square	Std. Error of the Estimate	Change Statistics R Square Change	F Change	df1
1	.465[a]	.216	.212	.57279	.216	53.373	1
2	.466[b]	.218	.201	.57661	.002	.146	3
3	.583[c]	.340	.322	.53112	.122	35.126	1
4	.586[d]	.343	.312	.53527	.004	.265	4

Model Summary

Model	Change Statistics df2	Sig. F Change
1	194	.000
2	191	.932
3	190	.000
4	186	.900

a. Predictors: (Constant), Party0

b. Predictors: (Constant), Party0, GenderCC, SES0, RaceCC

c. Predictors: (Constant), Party0, GenderCC, SES0, RaceCC, MRN0

d. Predictors: (Constant), Party0, GenderCC, SES0, RaceCC, MRN0, MRN0xSES0, MRN0xGender, MRN0xParty0, MRN0xRace

ANOVA[a]

Model		Sum of Squares	df	Mean Square	F	Sig.
1	Regression	17.511	1	17.511	53.373	.000[b]
	Residual	63.650	194	.328		
	Total	81.162	195			
2	Regression	17.657	4	4.414	13.277	.000[c]
	Residual	63.504	191	.332		
	Total	81.162	195			
3	Regression	27.566	5	5.513	19.545	.000[d]
	Residual	53.596	190	.282		
	Total	81.162	195			
4	Regression	27.870	9	3.097	10.808	.000[e]
	Residual	53.292	186	.287		
	Total	81.162	195			

a. Dependent Variable: Conspiracy_Tot

b. Predictors: (Constant), Party0

c. Predictors: (Constant), Party0, GenderCC, SES0, RaceCC

d. Predictors: (Constant), Party0, GenderCC, SES0, RaceCC, MRN0

e. Predictors: (Constant), Party0, GenderCC, SES0, RaceCC, MRN0, MRN0xSES0, MRN0xGender, MRN0xParty0, MRN0xRace

Coefficients[a]

Model		Unstandardized Coefficients B	Unstandardized Coefficients Std. Error	Standardized Coefficients Beta	t	Sig.
1	(Constant)	1.703	.041		41.629	.000
	Party0	.191	.026	.465	7.306	.000
2	(Constant)	1.707	.052		32.802	.000
	Party0	.192	.027	.466	7.163	.000
	GenderCC	.021	.042	.033	.506	.614
	RaceCC	-.006	.053	-.007	-.107	.915
	SES0	-.021	.048	-.027	-.426	.671
3	(Constant)	1.678	.048		34.825	.000
	Party0	.117	.028	.284	4.221	.000
	GenderCC	-.057	.041	-.089	-1.408	.161
	RaceCC	.045	.050	.056	.915	.361
	SES0	-.049	.045	-.065	-1.085	.279
	MRN0	.261	.044	.419	5.927	.000
4	(Constant)	1.678	.052		32.166	.000
	Party0	.115	.029	.278	4.010	.000
	GenderCC	-.054	.042	-.083	-1.287	.200
	RaceCC	.053	.051	.066	1.042	.299
	SES0	-.047	.045	-.062	-1.025	.307
	MRN0	.275	.051	.441	5.382	.000
	MRN0xRace	-.019	.047	-.030	-.399	.691
	MRN0xSES0	-.038	.043	-.053	-.885	.378
	MRN0xGender	-.012	.041	-.018	-.294	.769
	MRN0xParty0	.003	.026	.007	.108	.914

Coefficients[a]

Model		Correlations Zero-order	Partial	Part
1	(Constant)			
	Party0	.465	.465	.465
2	(Constant)			
	Party0	.465	.460	.458
	GenderCC	.040	.037	.032
	RaceCC	.067	-.008	-.007
	SES0	-.012	-.031	-.027
3	(Constant)			
	Party0	.465	.293	.249
	GenderCC	.040	-.102	-.083
	RaceCC	.067	.066	.054
	SES0	-.012	-.078	-.064
	MRN0	.493	.395	.349
4	(Constant)			
	Party0	.465	.282	.238
	GenderCC	.040	-.094	-.076
	RaceCC	.067	.076	.062
	SES0	-.012	-.075	-.061
	MRN0	.493	.367	.320
	MRN0xRace	.284	-.029	-.024
	MRN0xSES0	-.053	-.065	-.053
	MRN0xGender	-.052	-.022	-.017
	MRN0xParty0	-.079	.008	.006

a. Dependent Variable: Conspiracy_Tot

Excluded Variables[a]

Model		Beta In	t	Sig.	Partial Correlation	Collinearity Statistics Tolerance
1	GenderCC	.032[b]	.495	.621	.036	1.000
	RaceCC	-.013[b]	-.194	.846	-.014	.971
	SES0	-.025[b]	-.387	.699	-.028	.999
	MRN0	.363[b]	5.580	.000	.373	.827
	MRN0xRace	.143[b]	2.136	.034	.152	.886
	MRN0xSES0	-.028[b]	-.436	.663	-.031	.997
	MRN0xGender	.006[b]	.099	.921	.007	.984
	MRN0xParty0	-.054[b]	-.851	.396	-.061	.997
2	MRN0	.419[c]	5.927	.000	.395	.695
	MRN0xRace	.150[c]	2.122	.035	.152	.807
	MRN0xSES0	-.029[c]	-.449	.654	-.033	.989
	MRN0xGender	.009[c]	.142	.887	.010	.966
	MRN0xParty0	-.053[c]	-.807	.420	-.058	.970
3	MRN0xRace	-.029[d]	-.390	.697	-.028	.648
	MRN0xSES0	-.055[d]	-.925	.356	-.067	.983
	MRN0xGender	-.014[d]	-.234	.815	-.017	.962
	MRN0xParty0	.008[d]	.131	.896	.010	.942

a. Dependent Variable: Conspiracy_Tot

b. Predictors in the Model: (Constant), Party0

c. Predictors in the Model: (Constant), Party0, GenderCC, SES0, RaceCC

d. Predictors in the Model: (Constant), Party0, GenderCC, SES0, RaceCC, MRN0

```
 **Including PIdeology

REGRESSION
  /MISSING LISTWISE
  /STATISTICS COEFF OUTS R ANOVA CHANGE ZPP
  /CRITERIA=PIN(.05) POUT(.10)
  /NOORIGIN
  /DEPENDENT Concern_Tot
  /METHOD=ENTER Ideology0
  /METHOD=ENTER GenderCC RaceCC SES0
```

```
/METHOD=ENTER MRN0
/METHOD=ENTER MRN0xRace MRN0xSES0 MRN0xGender MRN0xIdeology0.
```

Regression

Notes

Output Created		15-DEC-2021 13:11:11
Comments		
Input	Data	C: \Users\njs5478\Dropbox\HM and COVID\0. Revise and Resubmit\2. R and R Data\Study 2b\Study2b_Data.sav
	Active Dataset	DataSet1
	Filter	<none>
	Weight	<none>
	Split File	<none>
	N of Rows in Working Data File	198
Missing Value Handling	Definition of Missing	User-defined missing values are treated as missing.
	Cases Used	Statistics are based on cases with no missing values for any variable used.
Syntax		REGRESSION /MISSING LISTWISE /STATISTICS COEFF OUTS R ANOVA CHANGE ZPP /CRITERIA=PIN(.05) POUT(.10) /NOORIGIN /DEPENDENT Concern_Tot /METHOD=ENTER Ideology0 /METHOD=ENTER GenderCC RaceCC SES0 /METHOD=ENTER MRN0 /METHOD=ENTER MRN0xRace MRN0xSES0 MRN0xGender MRN0xIdeology0.

Notes

Resources	Processor Time	00:00:00.06
	Elapsed Time	00:00:00.05
	Memory Required	32640 bytes
	Additional Memory Required for Residual Plots	0 bytes

Variables Entered/Removed[a]

Model	Variables Entered	Variables Removed	Method
1	Ideology0[b]	.	Enter
2	SES0, GenderCC, RaceCC[b]	.	Enter
3	MRN0[b]	.	Enter
4	MRN0xGender, MRN0xSES0, MRN0xIdeology0, MRN0xRace[b]	.	Enter

a. Dependent Variable: Concern_Tot

b. All requested variables entered.

Model Summary

					Change Statistics		
Model	R	R Square	Adjusted R Square	Std. Error of the Estimate	R Square Change	F Change	df1
1	.496[a]	.246	.242	1.41975	.246	63.180	1
2	.530[b]	.281	.266	1.39724	.035	3.100	3
3	.531[c]	.281	.263	1.40016	.001	.204	1
4	.549[d]	.301	.267	1.39588	.019	1.292	4

Model Summary

Model	Change Statistics df2	Sig. F Change
1	194	.000
2	191	.028
3	190	.652
4	186	.275

a. Predictors: (Constant), Ideology0

b. Predictors: (Constant), Ideology0, SES0, GenderCC, RaceCC

c. Predictors: (Constant), Ideology0, SES0, GenderCC, RaceCC, MRN0

d. Predictors: (Constant), Ideology0, SES0, GenderCC, RaceCC, MRN0, MRN0xGender, MRN0xSES0, MRN0xIdeology0, MRN0xRace

ANOVA[a]

Model		Sum of Squares	df	Mean Square	F	Sig.
1	Regression	127.351	1	127.351	63.180	.000[b]
	Residual	391.044	194	2.016		
	Total	518.396	195			
2	Regression	145.510	4	36.377	18.633	.000[c]
	Residual	372.886	191	1.952		
	Total	518.396	195			
3	Regression	145.910	5	29.182	14.885	.000[d]
	Residual	372.486	190	1.960		
	Total	518.396	195			
4	Regression	155.980	9	17.331	8.895	.000[e]
	Residual	362.416	186	1.948		
	Total	518.396	195			

a. Dependent Variable: Concern_Tot

b. Predictors: (Constant), Ideology0

c. Predictors: (Constant), Ideology0, SES0, GenderCC, RaceCC

d. Predictors: (Constant), Ideology0, SES0, GenderCC, RaceCC, MRN0

e. Predictors: (Constant), Ideology0, SES0, GenderCC, RaceCC, MRN0, MRN0xGender, MRN0xSES0, MRN0xIdeology0, MRN0xRace

Coefficients[a]

Model		Unstandardized Coefficients B	Std. Error	Standardized Coefficients Beta	t	Sig.
1	(Constant)	4.299	.101		42.392	.000
	Ideology0	-.398	.050	-.496	-7.949	.000
2	(Constant)	4.283	.126		34.034	.000
	Ideology0	-.389	.050	-.485	-7.799	.000
	GenderCC	-.296	.102	-.182	-2.915	.004
	RaceCC	.022	.128	.011	.169	.866
	SES0	-.045	.118	-.024	-.382	.703
3	(Constant)	4.276	.127		33.693	.000
	Ideology0	-.406	.062	-.506	-6.567	.000
	GenderCC	-.312	.108	-.192	-2.898	.004
	RaceCC	.033	.131	.016	.255	.799
	SES0	-.050	.118	-.026	-.424	.672
	MRN0	.058	.128	.037	.452	.652
4	(Constant)	4.161	.143		29.195	.000
	Ideology0	-.402	.064	-.501	-6.334	.000
	GenderCC	-.284	.109	-.175	-2.614	.010
	RaceCC	.061	.134	.030	.457	.648
	SES0	-.014	.119	-.007	-.120	.905
	MRN0	.163	.143	.104	1.144	.254
	MRN0xRace	-.165	.123	-.104	-1.346	.180
	MRN0xSES0	.044	.115	.024	.379	.705
	MRN0xGender	-.113	.107	-.068	-1.064	.289
	MRN0xIdeology0	.100	.052	.124	1.914	.057

Coefficients[a]

Model		Correlations Zero-order	Partial	Part
1	(Constant)			
	Ideology0	-.496	-.496	-.496
2	(Constant)			
	Ideology0	-.496	-.491	-.479
	GenderCC	-.215	-.206	-.179
	RaceCC	-.030	.012	.010
	SES0	-.062	-.028	-.023
3	(Constant)			
	Ideology0	-.496	-.430	-.404
	GenderCC	-.215	-.206	-.178
	RaceCC	-.030	.019	.016
	SES0	-.062	-.031	-.026
	MRN0	-.312	.033	.028
4	(Constant)			
	Ideology0	-.496	-.421	-.388
	GenderCC	-.215	-.188	-.160
	RaceCC	-.030	.033	.028
	SES0	-.062	-.009	-.007
	MRN0	-.312	.084	.070
	MRN0xRace	-.277	-.098	-.083
	MRN0xSES0	.046	.028	.023
	MRN0xGender	.021	-.078	-.065
	MRN0xIdeology0	.154	.139	.117

a. Dependent Variable: Concern_Tot

Excluded Variables[a]

Model		Beta In	t	Sig.	Partial Correlation	Collinearity Statistics Tolerance
1	GenderCC	-.186[b]	-3.036	.003	-.213	.996
	RaceCC	.039[b]	.616	.539	.044	.981
	SES0	-.039[b]	-.619	.537	-.044	.998
	MRN0	-.054[b]	-.715	.475	-.051	.692
	MRN0xRace	-.101[b]	-1.494	.137	-.107	.851
	MRN0xSES0	-.005[b]	-.087	.931	-.006	.989
	MRN0xGender	-.035[b]	-.565	.573	-.041	.987
	MRN0xIdeology0	.115[b]	1.843	.067	.132	.994
2	MRN0	.037[c]	.452	.652	.033	.575
	MRN0xRace	-.049[c]	-.707	.480	-.051	.781
	MRN0xSES0	.006[c]	.103	.918	.007	.979
	MRN0xGender	-.035[c]	-.564	.573	-.041	.970
	MRN0xIdeology0	.096[c]	1.544	.124	.111	.970
3	MRN0xRace	-.074[d]	-.975	.331	-.071	.652
	MRN0xSES0	.003[d]	.045	.965	.003	.963
	MRN0xGender	-.038[d]	-.603	.547	-.044	.963
	MRN0xIdeology0	.099[d]	1.587	.114	.115	.963

a. Dependent Variable: Concern_Tot

b. Predictors in the Model: (Constant), Ideology0

c. Predictors in the Model: (Constant), Ideology0, SES0, GenderCC, RaceCC

d. Predictors in the Model: (Constant), Ideology0, SES0, GenderCC, RaceCC, MRN0

```
REGRESSION
 /MISSING LISTWISE
 /STATISTICS COEFF OUTS R ANOVA CHANGE ZPP
 /CRITERIA=PIN(.05) POUT(.10)
 /NOORIGIN
 /DEPENDENT Finance_Tot
 /METHOD=ENTER Ideology0
 /METHOD=ENTER GenderCC RaceCC SES0
 /METHOD=ENTER MRN0
 /METHOD=ENTER MRN0xRace MRN0xSES0 MRN0xGender MRN0xIdeology0.
```

Regression

Notes

Output Created		15-DEC-2021 13:11:11
Comments		
Input	Data	C: \Users\njs5478\Dropbox\HM and COVID\0. Revise and Resubmit\2. R and R Data\Study 2b\Study2b_Data.sav
	Active Dataset	DataSet1
	Filter	<none>
	Weight	<none>
	Split File	<none>
	N of Rows in Working Data File	198
Missing Value Handling	Definition of Missing	User-defined missing values are treated as missing.
	Cases Used	Statistics are based on cases with no missing values for any variable used.
Syntax		REGRESSION /MISSING LISTWISE /STATISTICS COEFF OUTS R ANOVA CHANGE ZPP /CRITERIA=PIN(.05) POUT(.10) /NOORIGIN /DEPENDENT Finance_Tot /METHOD=ENTER Ideology0 /METHOD=ENTER GenderCC RaceCC SES0 /METHOD=ENTER MRN0 /METHOD=ENTER MRN0xRace MRN0xSES0 MRN0xGender MRN0xIdeology0.
Resources	Processor Time	00:00:00.02
	Elapsed Time	00:00:00.03

Notes

	Memory Required	32640 bytes
	Additional Memory Required for Residual Plots	0 bytes

Variables Entered/Removed[a]

Model	Variables Entered	Variables Removed	Method
1	Ideology0[b]	.	Enter
2	SES0, GenderCC, RaceCC[b]	.	Enter
3	MRN0[b]	.	Enter
4	MRN0xGender, MRN0xSES0, MRN0xIdeology0, MRN0xRace[b]	.	Enter

a. Dependent Variable: Finance_Tot

b. All requested variables entered.

Model Summary

Model	R	R Square	Adjusted R Square	Std. Error of the Estimate	Change Statistics		
					R Square Change	F Change	df1
1	.038[a]	.001	-.004	1.99058	.001	.286	1
2	.340[b]	.116	.097	1.88772	.114	8.239	3
3	.357[c]	.127	.104	1.88062	.011	2.444	1
4	.358[d]	.128	.086	1.89948	.001	.062	4

Model Summary

Model	Change Statistics	
	df2	Sig. F Change
1	194	.593
2	191	.000
3	190	.120
4	186	.993

a. Predictors: (Constant), Ideology0

b. Predictors: (Constant), Ideology0, SES0, GenderCC, RaceCC

c. Predictors: (Constant), Ideology0, SES0, GenderCC, RaceCC, MRN0

d. Predictors: (Constant), Ideology0, SES0, GenderCC, RaceCC, MRN0, MRN0xGender, MRN0xSES0, MRN0xIdeology0, MRN0xRace

ANOVA[a]

Model		Sum of Squares	df	Mean Square	F	Sig.
1	Regression	1.133	1	1.133	.286	.593[b]
	Residual	768.704	194	3.962		
	Total	769.837	195			
2	Regression	89.211	4	22.303	6.259	.000[c]
	Residual	680.626	191	3.563		
	Total	769.837	195			
3	Regression	97.855	5	19.571	5.534	.000[d]
	Residual	671.982	190	3.537		
	Total	769.837	195			
4	Regression	98.746	9	10.972	3.041	.002[e]
	Residual	671.091	186	3.608		
	Total	769.837	195			

a. Dependent Variable: Finance_Tot

b. Predictors: (Constant), Ideology0

c. Predictors: (Constant), Ideology0, SES0, GenderCC, RaceCC

d. Predictors: (Constant), Ideology0, SES0, GenderCC, RaceCC, MRN0

e. Predictors: (Constant), Ideology0, SES0, GenderCC, RaceCC, MRN0, MRN0xGender, MRN0xSES0, MRN0xIdeology0, MRN0xRace

Coefficients[a]

Model		Unstandardized Coefficients B	Unstandardized Coefficients Std. Error	Standardized Coefficients Beta	t	Sig.
1	(Constant)	3.797	.142		26.701	.000
	Ideology0	-.038	.070	-.038	-.535	.593
2	(Constant)	3.731	.170		21.942	.000
	Ideology0	-.020	.067	-.020	-.293	.770
	GenderCC	-.375	.137	-.189	-2.735	.007
	RaceCC	.102	.173	.041	.593	.554
	SES0	-.601	.159	-.259	-3.781	.000
3	(Constant)	3.700	.170		21.706	.000
	Ideology0	-.096	.083	-.098	-1.156	.249
	GenderCC	-.449	.145	-.227	-3.106	.002
	RaceCC	.157	.175	.063	.893	.373
	SES0	-.625	.159	-.269	-3.932	.000
	MRN0	.268	.171	.140	1.563	.120
4	(Constant)	3.739	.194		19.275	.000
	Ideology0	-.101	.086	-.103	-1.166	.245
	GenderCC	-.452	.148	-.228	-3.053	.003
	RaceCC	.168	.182	.068	.922	.358
	SES0	-.629	.162	-.271	-3.879	.000
	MRN0	.269	.194	.140	1.387	.167
	MRN0xRace	-.003	.167	-.002	-.019	.985
	MRN0xSES0	-.015	.156	-.007	-.097	.923
	MRN0xGender	-.034	.145	-.017	-.235	.814
	MRN0xIdeology0	-.027	.071	-.028	-.386	.700

Coefficients[a]

Model		Correlations Zero-order	Partial	Part
1	(Constant)			
	Ideology0	-.038	-.038	-.038
2	(Constant)			
	Ideology0	-.038	-.021	-.020
	GenderCC	-.219	-.194	-.186
	RaceCC	.060	.043	.040
	SES0	-.275	-.264	-.257
3	(Constant)			
	Ideology0	-.038	-.084	-.078
	GenderCC	-.219	-.220	-.210
	RaceCC	.060	.065	.061
	SES0	-.275	-.274	-.266
	MRN0	-.030	.113	.106
4	(Constant)			
	Ideology0	-.038	-.085	-.080
	GenderCC	-.219	-.218	-.209
	RaceCC	.060	.067	.063
	SES0	-.275	-.274	-.266
	MRN0	-.030	.101	.095
	MRN0xRace	-.050	-.001	-.001
	MRN0xSES0	-.001	-.007	-.007
	MRN0xGender	-.020	-.017	-.016
	MRN0xIdeology0	.014	-.028	-.026

a. Dependent Variable: Finance_Tot

Excluded Variables[a]

Model		Beta In	t	Sig.	Partial Correlation	Collinearity Statistics Tolerance
1	GenderCC	-.218[b]	-3.093	.002	-.217	.996
	RaceCC	.067[b]	.924	.357	.066	.981
	SES0	-.274[b]	-3.953	.000	-.274	.998
	MRN0	-.012[b]	-.140	.889	-.010	.692
	MRN0xRace	-.041[b]	-.532	.595	-.038	.851
	MRN0xSES0	-.005[b]	-.074	.941	-.005	.989
	MRN0xGender	-.025[b]	-.343	.732	-.025	.987
	MRN0xIdeology0	.011[b]	.151	.880	.011	.994
2	MRN0	.140[c]	1.563	.120	.113	.575
	MRN0xRace	.046[c]	.594	.553	.043	.781
	MRN0xSES0	.011[c]	.166	.868	.012	.979
	MRN0xGender	-.012[c]	-.170	.865	-.012	.970
	MRN0xIdeology0	-.039[c]	-.563	.574	-.041	.970
3	MRN0xRace	-.004[d]	-.042	.966	-.003	.652
	MRN0xSES0	-.002[d]	-.036	.971	-.003	.963
	MRN0xGender	-.021[d]	-.303	.762	-.022	.963
	MRN0xIdeology0	-.030[d]	-.429	.668	-.031	.963

a. Dependent Variable: Finance_Tot

b. Predictors in the Model: (Constant), Ideology0

c. Predictors in the Model: (Constant), Ideology0, SES0, GenderCC, RaceCC

d. Predictors in the Model: (Constant), Ideology0, SES0, GenderCC, RaceCC, MRN0

```
REGRESSION
 /MISSING LISTWISE
 /STATISTICS COEFF OUTS R ANOVA CHANGE ZPP
 /CRITERIA=PIN(.05) POUT(.10)
 /NOORIGIN
 /DEPENDENT Psychology_Tot
 /METHOD=ENTER Ideology0
 /METHOD=ENTER GenderCC RaceCC SES0
 /METHOD=ENTER MRN0
 /METHOD=ENTER MRN0xRace MRN0xSES0 MRN0xGender MRN0xIdeology0.
```

Regression

Notes

Output Created		15-DEC-2021 13:11:11
Comments		
Input	Data	C:\Users\njs5478\Dropbox\HM and COVID\0. Revise and Resubmit\2. R and R Data\Study 2b\Study2b_Data.sav
	Active Dataset	DataSet1
	Filter	<none>
	Weight	<none>
	Split File	<none>
	N of Rows in Working Data File	198
Missing Value Handling	Definition of Missing	User-defined missing values are treated as missing.
	Cases Used	Statistics are based on cases with no missing values for any variable used.
Syntax		REGRESSION<br>/MISSING LISTWISE<br>/STATISTICS COEFF OUTS R ANOVA CHANGE ZPP<br>/CRITERIA=PIN(.05) POUT(.10)<br>/NOORIGIN<br>/DEPENDENT Psychology_Tot<br>/METHOD=ENTER Ideology0<br>/METHOD=ENTER GenderCC RaceCC SES0<br>/METHOD=ENTER MRN0<br>/METHOD=ENTER MRN0xRace MRN0xSES0 MRN0xGender MRN0xIdeology0.
Resources	Processor Time	00:00:00.05
	Elapsed Time	00:00:00.03

Notes

	Memory Required	32640 bytes
	Additional Memory Required for Residual Plots	0 bytes

Variables Entered/Removed[a]

Model	Variables Entered	Variables Removed	Method
1	Ideology0[b]	.	Enter
2	SES0, GenderCC, RaceCC[b]	.	Enter
3	MRN0[b]	.	Enter
4	MRN0xGender, MRN0xSES0, MRN0xIdeology0, MRN0xRace[b]	.	Enter

a. Dependent Variable: Psychology_Tot

b. All requested variables entered.

Model Summary

Model	R	R Square	Adjusted R Square	Std. Error of the Estimate	Change Statistics		
					R Square Change	F Change	df1
1	.362[a]	.131	.127	1.59561	.131	29.247	1
2	.462[b]	.213	.197	1.53008	.082	6.658	3
3	.471[c]	.222	.202	1.52557	.009	2.131	1
4	.495[d]	.245	.208	1.51931	.023	1.392	4

Model Summary

Model	Change Statistics	
	df2	Sig. F Change
1	194	.000
2	191	.000
3	190	.146
4	186	.238

a. Predictors: (Constant), Ideology0

b. Predictors: (Constant), Ideology0, SES0, GenderCC, RaceCC

c. Predictors: (Constant), Ideology0, SES0, GenderCC, RaceCC, MRN0

d. Predictors: (Constant), Ideology0, SES0, GenderCC, RaceCC, MRN0, MRN0xGender, MRN0xSES0, MRN0xIdeology0, MRN0xRace

ANOVA[a]

Model		Sum of Squares	df	Mean Square	F	Sig.
1	Regression	74.462	1	74.462	29.247	.000[b]
	Residual	493.921	194	2.546		
	Total	568.383	195			
2	Regression	121.225	4	30.306	12.945	.000[c]
	Residual	447.158	191	2.341		
	Total	568.383	195			
3	Regression	126.184	5	25.237	10.844	.000[d]
	Residual	442.198	190	2.327		
	Total	568.383	195			
4	Regression	139.038	9	15.449	6.693	.000[e]
	Residual	429.344	186	2.308		
	Total	568.383	195			

a. Dependent Variable: Psychology_Tot

b. Predictors: (Constant), Ideology0

c. Predictors: (Constant), Ideology0, SES0, GenderCC, RaceCC

d. Predictors: (Constant), Ideology0, SES0, GenderCC, RaceCC, MRN0

e. Predictors: (Constant), Ideology0, SES0, GenderCC, RaceCC, MRN0, MRN0xGender, MRN0xSES0, MRN0xIdeology0, MRN0xRace

Coefficients[a]

Model		Unstandardized Coefficients		Standardized Coefficients	t	Sig.
		B	Std. Error	Beta		
1	(Constant)	4.116	.114		36.110	.000
	Ideology0	-.304	.056	-.362	-5.408	.000
2	(Constant)	3.992	.138		28.964	.000
	Ideology0	-.299	.055	-.356	-5.469	.000
	GenderCC	-.380	.111	-.223	-3.414	.001
	RaceCC	.199	.140	.093	1.425	.156
	SES0	-.234	.129	-.117	-1.815	.071
3	(Constant)	3.969	.138		28.698	.000
	Ideology0	-.357	.067	-.424	-5.298	.000
	GenderCC	-.436	.117	-.256	-3.713	.000
	RaceCC	.240	.142	.113	1.690	.093
	SES0	-.252	.129	-.126	-1.956	.052
	MRN0	.203	.139	.123	1.460	.146
4	(Constant)	3.822	.155		24.636	.000
	Ideology0	-.352	.069	-.419	-5.097	.000
	GenderCC	-.403	.118	-.237	-3.405	.001
	RaceCC	.266	.146	.124	1.825	.070
	SES0	-.214	.130	-.107	-1.650	.101
	MRN0	.320	.155	.194	2.058	.041
	MRN0xRace	-.176	.133	-.106	-1.317	.190
	MRN0xSES0	.005	.125	.002	.038	.970
	MRN0xGender	-.073	.116	-.042	-.631	.529
	MRN0xIdeology0	.120	.057	.142	2.104	.037

Coefficients[a]

Model		Correlations Zero-order	Partial	Part
1	(Constant)			
	Ideology0	-.362	-.362	-.362
2	(Constant)			
	Ideology0	-.362	-.368	-.351
	GenderCC	-.268	-.240	-.219
	RaceCC	.075	.103	.091
	SES0	-.151	-.130	-.116
3	(Constant)			
	Ideology0	-.362	-.359	-.339
	GenderCC	-.268	-.260	-.238
	RaceCC	.075	.122	.108
	SES0	-.151	-.140	-.125
	MRN0	-.225	.105	.093
4	(Constant)			
	Ideology0	-.362	-.350	-.325
	GenderCC	-.268	-.242	-.217
	RaceCC	.075	.133	.116
	SES0	-.151	-.120	-.105
	MRN0	-.225	.149	.131
	MRN0xRace	-.223	-.096	-.084
	MRN0xSES0	.017	.003	.002
	MRN0xGender	.042	-.046	-.040
	MRN0xIdeology0	.181	.152	.134

a. Dependent Variable: Psychology_Tot

Excluded Variables[a]

Model		Beta In	t	Sig.	Partial Correlation	Collinearity Statistics Tolerance
1	GenderCC	-.247[b]	-3.815	.000	-.265	.996
	RaceCC	.127[b]	1.886	.061	.135	.981
	SES0	-.134[b]	-2.021	.045	-.144	.998
	MRN0	-.035[b]	-.438	.662	-.032	.692
	MRN0xRace	-.097[b]	-1.344	.181	-.096	.851
	MRN0xSES0	-.021[b]	-.308	.759	-.022	.989
	MRN0xGender	.002[b]	.022	.982	.002	.987
	MRN0xIdeology0	.153[b]	2.306	.022	.164	.994
2	MRN0	.123[c]	1.460	.146	.105	.575
	MRN0xRace	-.022[c]	-.309	.758	-.022	.781
	MRN0xSES0	-.009[c]	-.141	.888	-.010	.979
	MRN0xGender	-.001[c]	-.008	.993	-.001	.970
	MRN0xIdeology0	.115[c]	1.769	.079	.127	.970
3	MRN0xRace	-.078[d]	-.989	.324	-.072	.652
	MRN0xSES0	-.022[d]	-.333	.739	-.024	.963
	MRN0xGender	-.009[d]	-.131	.896	-.010	.963
	MRN0xIdeology0	.124[d]	1.913	.057	.138	.963

a. Dependent Variable: Psychology_Tot

b. Predictors in the Model: (Constant), Ideology0

c. Predictors in the Model: (Constant), Ideology0, SES0, GenderCC, RaceCC

d. Predictors in the Model: (Constant), Ideology0, SES0, GenderCC, RaceCC, MRN0

```
REGRESSION
 /MISSING LISTWISE
 /STATISTICS COEFF OUTS R ANOVA CHANGE ZPP
 /CRITERIA=PIN(.05) POUT(.10)
 /NOORIGIN
 /DEPENDENT Risk_Rules
 /METHOD=ENTER Ideology0
 /METHOD=ENTER GenderCC RaceCC SES0
 /METHOD=ENTER MRN0
 /METHOD=ENTER MRN0xRace MRN0xSES0 MRN0xGender MRN0xIdeology0.
```

Regression

Notes

Output Created		15-DEC-2021 13:11:11
Comments		
Input	Data	C:\Users\njs5478\Dropbox\HM and COVID\0. Revise and Resubmit\2. R and R Data\Study 2b\Study2b_Data.sav
	Active Dataset	DataSet1
	Filter	<none>
	Weight	<none>
	Split File	<none>
	N of Rows in Working Data File	198
Missing Value Handling	Definition of Missing	User-defined missing values are treated as missing.
	Cases Used	Statistics are based on cases with no missing values for any variable used.
Syntax		REGRESSION<br>/MISSING LISTWISE<br>/STATISTICS COEFF OUTS R ANOVA CHANGE ZPP<br>/CRITERIA=PIN(.05) POUT(.10)<br>/NOORIGIN<br>/DEPENDENT Risk_Rules<br>/METHOD=ENTER Ideology0<br>/METHOD=ENTER GenderCC RaceCC SES0<br>/METHOD=ENTER MRN0<br>/METHOD=ENTER MRN0xRace MRN0xSES0 MRN0xGender MRN0xIdeology0.
Resources	Processor Time	00:00:00.05
	Elapsed Time	00:00:00.03

Notes

	Memory Required	32640 bytes
	Additional Memory Required for Residual Plots	0 bytes

Variables Entered/Removed[a]

Model	Variables Entered	Variables Removed	Method
1	Ideology0[b]	.	Enter
2	SES0, GenderCC, RaceCC[b]	.	Enter
3	MRN0[b]	.	Enter
4	MRN0xGender, MRN0xSES0, MRN0xIdeology0, MRN0xRace[b]	.	Enter

a. Dependent Variable: Risk_Rules

b. All requested variables entered.

Model Summary

Model	R	R Square	Adjusted R Square	Std. Error of the Estimate	Change Statistics		
					R Square Change	F Change	df1
1	.549[a]	.302	.298	.92747	.302	83.850	1
2	.560[b]	.313	.299	.92714	.011	1.046	3
3	.567[c]	.321	.303	.92397	.008	2.311	1
4	.575[d]	.330	.298	.92768	.009	.621	4

Model Summary

Model	Change Statistics	
	df2	Sig. F Change
1	194	.000
2	191	.373
3	190	.130
4	186	.648

a. Predictors: (Constant), Ideology0

b. Predictors: (Constant), Ideology0, SES0, GenderCC, RaceCC

c. Predictors: (Constant), Ideology0, SES0, GenderCC, RaceCC, MRN0

d. Predictors: (Constant), Ideology0, SES0, GenderCC, RaceCC, MRN0, MRN0xGender, MRN0xSES0, MRN0xIdeology0, MRN0xRace

ANOVA[a]

Model		Sum of Squares	df	Mean Square	F	Sig.
1	Regression	72.127	1	72.127	83.850	.000[b]
	Residual	166.877	194	.860		
	Total	239.005	195			
2	Regression	74.825	4	18.706	21.762	.000[c]
	Residual	164.180	191	.860		
	Total	239.005	195			
3	Regression	76.798	5	15.360	17.991	.000[d]
	Residual	162.207	190	.854		
	Total	239.005	195			
4	Regression	78.934	9	8.770	10.191	.000[e]
	Residual	160.071	186	.861		
	Total	239.005	195			

a. Dependent Variable: Risk_Rules

b. Predictors: (Constant), Ideology0

c. Predictors: (Constant), Ideology0, SES0, GenderCC, RaceCC

d. Predictors: (Constant), Ideology0, SES0, GenderCC, RaceCC, MRN0

e. Predictors: (Constant), Ideology0, SES0, GenderCC, RaceCC, MRN0, MRN0xGender, MRN0xSES0, MRN0xIdeology0, MRN0xRace

Coefficients[a]

Model		Unstandardized Coefficients B	Unstandardized Coefficients Std. Error	Standardized Coefficients Beta	t	Sig.
1	(Constant)	3.303	.066		49.859	.000
	Ideology0	.299	.033	.549	9.157	.000
2	(Constant)	3.311	.084		39.647	.000
	Ideology0	.297	.033	.546	8.978	.000
	GenderCC	.114	.067	.103	1.688	.093
	RaceCC	-.011	.085	-.008	-.125	.901
	SES0	-.039	.078	-.030	-.504	.615
3	(Constant)	3.296	.084		39.355	.000
	Ideology0	.261	.041	.479	6.402	.000
	GenderCC	.079	.071	.071	1.105	.271
	RaceCC	.015	.086	.011	.178	.859
	SES0	-.051	.078	-.039	-.654	.514
	MRN0	.128	.084	.120	1.520	.130
4	(Constant)	3.318	.095		35.029	.000
	Ideology0	.258	.042	.473	6.104	.000
	GenderCC	.064	.072	.058	.885	.377
	RaceCC	-.008	.089	-.006	-.093	.926
	SES0	-.062	.079	-.048	-.784	.434
	MRN0	.068	.095	.064	.721	.472
	MRN0xRace	.118	.081	.110	1.444	.150
	MRN0xSES0	-.026	.076	-.021	-.346	.730
	MRN0xGender	.056	.071	.049	.786	.433
	MRN0xIdeology0	-.011	.035	-.020	-.310	.757

Coefficients[a]

Model		Correlations		
		Zero-order	Partial	Part
1	(Constant)			
	Ideology0	.549	.549	.549
2	(Constant)			
	Ideology0	.549	.545	.538
	GenderCC	.134	.121	.101
	RaceCC	.051	-.009	-.007
	SES0	.005	-.036	-.030
3	(Constant)			
	Ideology0	.549	.421	.383
	GenderCC	.134	.080	.066
	RaceCC	.051	.013	.011
	SES0	.005	-.047	-.039
	MRN0	.403	.110	.091
4	(Constant)			
	Ideology0	.549	.409	.366
	GenderCC	.134	.065	.053
	RaceCC	.051	-.007	-.006
	SES0	.005	-.057	-.047
	MRN0	.403	.053	.043
	MRN0xRace	.331	.105	.087
	MRN0xSES0	-.055	-.025	-.021
	MRN0xGender	-.023	.058	.047
	MRN0xIdeology0	-.057	-.023	-.019

a. Dependent Variable: Risk_Rules

Excluded Variables[a]

Model		Beta In	t	Sig.	Partial Correlation	Collinearity Statistics Tolerance
1	GenderCC	.102[b]	1.700	.091	.121	.996
	RaceCC	-.024[b]	-.400	.690	-.029	.981
	SES0	-.022[b]	-.363	.717	-.026	.998
	MRN0	.142[b]	1.979	.049	.141	.692
	MRN0xRace	.140[b]	2.173	.031	.155	.851
	MRN0xSES0	.002[b]	.033	.974	.002	.989
	MRN0xGender	.039[b]	.646	.519	.046	.987
	MRN0xIdeology0	-.013[b]	-.215	.830	-.015	.994
2	MRN0	.120[c]	1.520	.130	.110	.575
	MRN0xRace	.124[c]	1.833	.068	.132	.781
	MRN0xSES0	-.003[c]	-.056	.955	-.004	.979
	MRN0xGender	.042[c]	.696	.487	.050	.970
	MRN0xIdeology0	-.006[c]	-.094	.925	-.007	.970
3	MRN0xRace	.098[d]	1.328	.186	.096	.652
	MRN0xSES0	-.016[d]	-.255	.799	-.019	.963
	MRN0xGender	.035[d]	.573	.568	.042	.963
	MRN0xIdeology0	.002[d]	.040	.968	.003	.963

a. Dependent Variable: Risk_Rules

b. Predictors in the Model: (Constant), Ideology0

c. Predictors in the Model: (Constant), Ideology0, SES0, GenderCC, RaceCC

d. Predictors in the Model: (Constant), Ideology0, SES0, GenderCC, RaceCC, MRN0

```
REGRESSION
 /MISSING LISTWISE
 /STATISTICS COEFF OUTS R ANOVA CHANGE ZPP
 /CRITERIA=PIN(.05) POUT(.10)
 /NOORIGIN
 /DEPENDENT Mandate_Tot
 /METHOD=ENTER Ideology0
 /METHOD=ENTER GenderCC RaceCC SES0
 /METHOD=ENTER MRN0
 /METHOD=ENTER MRN0xRace MRN0xSES0 MRN0xGender MRN0xIdeology0.
```

Regression

Notes

Output Created		15-DEC-2021 13:11:11
Comments		
Input	Data	C:\Users\njs5478\Dropbox\HM and COVID\0. Revise and Resubmit\2. R and R Data\Study 2b\Study2b_Data.sav
	Active Dataset	DataSet1
	Filter	<none>
	Weight	<none>
	Split File	<none>
	N of Rows in Working Data File	198
Missing Value Handling	Definition of Missing	User-defined missing values are treated as missing.
	Cases Used	Statistics are based on cases with no missing values for any variable used.
Syntax		REGRESSION /MISSING LISTWISE /STATISTICS COEFF OUTS R ANOVA CHANGE ZPP /CRITERIA=PIN(.05) POUT(.10) /NOORIGIN /DEPENDENT Mandate_Tot /METHOD=ENTER Ideology0 /METHOD=ENTER GenderCC RaceCC SES0 /METHOD=ENTER MRN0 /METHOD=ENTER MRN0xRace MRN0xSES0 MRN0xGender MRN0xIdeology0.
Resources	Processor Time	00:00:00.03
	Elapsed Time	00:00:00.11

Notes

	Memory Required	32640 bytes
	Additional Memory Required for Residual Plots	0 bytes

Variables Entered/Removed[a]

Model	Variables Entered	Variables Removed	Method
1	Ideology0[b]	.	Enter
2	SES0, GenderCC, RaceCC[b]	.	Enter
3	MRN0[b]	.	Enter
4	MRN0xGender, MRN0xSES0, MRN0xIdeology0, MRN0xRace[b]	.	Enter

a. Dependent Variable: Mandate_Tot

b. All requested variables entered.

Model Summary

Model	R	R Square	Adjusted R Square	Std. Error of the Estimate	Change Statistics		
					R Square Change	F Change	df1
1	.726[a]	.527	.525	1.54056	.527	216.378	1
2	.729[b]	.532	.522	1.54481	.005	.644	3
3	.730[c]	.533	.520	1.54796	.001	.224	1
4	.738[d]	.545	.523	1.54426	.012	1.228	4

Model Summary

Model	Change Statistics	
	df2	Sig. F Change
1	194	.000
2	191	.588
3	190	.637
4	186	.300

a. Predictors: (Constant), Ideology0

b. Predictors: (Constant), Ideology0, SES0, GenderCC, RaceCC

c. Predictors: (Constant), Ideology0, SES0, GenderCC, RaceCC, MRN0

d. Predictors: (Constant), Ideology0, SES0, GenderCC, RaceCC, MRN0, MRN0xGender, MRN0xSES0, MRN0xIdeology0, MRN0xRace

ANOVA[a]

Model		Sum of Squares	df	Mean Square	F	Sig.
1	Regression	513.533	1	513.533	216.378	.000[b]
	Residual	460.423	194	2.373		
	Total	973.955	195			
2	Regression	518.143	4	129.536	54.280	.000[c]
	Residual	455.813	191	2.386		
	Total	973.955	195			
3	Regression	518.678	5	103.736	43.292	.000[d]
	Residual	455.277	190	2.396		
	Total	973.955	195			
4	Regression	530.396	9	58.933	24.713	.000[e]
	Residual	443.559	186	2.385		
	Total	973.955	195			

a. Dependent Variable: Mandate_Tot

b. Predictors: (Constant), Ideology0

c. Predictors: (Constant), Ideology0, SES0, GenderCC, RaceCC

d. Predictors: (Constant), Ideology0, SES0, GenderCC, RaceCC, MRN0

e. Predictors: (Constant), Ideology0, SES0, GenderCC, RaceCC, MRN0, MRN0xGender, MRN0xSES0, MRN0xIdeology0, MRN0xRace

Coefficients[a]

Model		Unstandardized Coefficients B	Unstandardized Coefficients Std. Error	Standardized Coefficients Beta	t	Sig.
1	(Constant)	4.523	.110		41.099	.000
	Ideology0	-.799	.054	-.726	-14.710	.000
2	(Constant)	4.534	.139		32.587	.000
	Ideology0	-.803	.055	-.730	-14.552	.000
	GenderCC	.078	.112	.035	.698	.486
	RaceCC	-.017	.141	-.006	-.122	.903
	SES0	.145	.130	.056	1.117	.266
3	(Constant)	4.527	.140		32.258	.000
	Ideology0	-.822	.068	-.747	-12.035	.000
	GenderCC	.060	.119	.027	.504	.615
	RaceCC	-.004	.144	-.001	-.026	.979
	SES0	.139	.131	.053	1.062	.290
	MRN0	.067	.141	.031	.473	.637
4	(Constant)	4.373	.158		27.734	.000
	Ideology0	-.813	.070	-.739	-11.566	.000
	GenderCC	.090	.120	.040	.749	.455
	RaceCC	.007	.148	.003	.051	.960
	SES0	.172	.132	.066	1.307	.193
	MRN0	.164	.158	.076	1.039	.300
	MRN0xRace	-.152	.136	-.070	-1.122	.263
	MRN0xSES0	.018	.127	.007	.140	.889
	MRN0xGender	-.019	.118	-.008	-.158	.875
	MRN0xIdeology0	.117	.058	.106	2.024	.044

Coefficients[a]

Model		Correlations Zero-order	Partial	Part
1	(Constant)			
	Ideology0	-.726	-.726	-.726
2	(Constant)			
	Ideology0	-.726	-.725	-.720
	GenderCC	-.003	.050	.035
	RaceCC	-.110	-.009	-.006
	SES0	.023	.081	.055
3	(Constant)			
	Ideology0	-.726	-.658	-.597
	GenderCC	-.003	.037	.025
	RaceCC	-.110	-.002	-.001
	SES0	.023	.077	.053
	MRN0	-.369	.034	.023
4	(Constant)			
	Ideology0	-.726	-.647	-.572
	GenderCC	-.003	.055	.037
	RaceCC	-.110	.004	.003
	SES0	.023	.095	.065
	MRN0	-.369	.076	.051
	MRN0xRace	-.293	-.082	-.055
	MRN0xSES0	.072	.010	.007
	MRN0xGender	.102	-.012	-.008
	MRN0xIdeology0	.139	.147	.100

a. Dependent Variable: Mandate_Tot

Excluded Variables[a]

Model		Beta In	t	Sig.	Partial Correlation	Collinearity Statistics Tolerance
1	GenderCC	.041[b]	.824	.411	.059	.996
	RaceCC	-.011[b]	-.217	.829	-.016	.981
	SES0	.058[b]	1.183	.238	.085	.998
	MRN0	.050[b]	.845	.399	.061	.692
	MRN0xRace	-.014[b]	-.265	.791	-.019	.851
	MRN0xSES0	-.004[b]	-.084	.933	-.006	.989
	MRN0xGender	.021[b]	.413	.680	.030	.987
	MRN0xIdeology0	.082[b]	1.654	.100	.118	.994
2	MRN0	.031[c]	.473	.637	.034	.575
	MRN0xRace	-.033[c]	-.594	.554	-.043	.781
	MRN0xSES0	-.008[c]	-.153	.879	-.011	.979
	MRN0xGender	.018[c]	.352	.726	.025	.970
	MRN0xIdeology0	.093[c]	1.873	.063	.135	.970
3	MRN0xRace	-.053[d]	-.859	.391	-.062	.652
	MRN0xSES0	-.011[d]	-.216	.829	-.016	.963
	MRN0xGender	.016[d]	.312	.755	.023	.963
	MRN0xIdeology0	.096[d]	1.919	.056	.138	.963

a. Dependent Variable: Mandate_Tot

b. Predictors in the Model: (Constant), Ideology0

c. Predictors in the Model: (Constant), Ideology0, SES0, GenderCC, RaceCC

d. Predictors in the Model: (Constant), Ideology0, SES0, GenderCC, RaceCC, MRN0

```
REGRESSION
 /MISSING LISTWISE
 /STATISTICS COEFF OUTS R ANOVA CHANGE ZPP
 /CRITERIA=PIN(.05) POUT(.10)
 /NOORIGIN
 /DEPENDENT Conspiracy_Tot
 /METHOD=ENTER Ideology0
 /METHOD=ENTER GenderCC RaceCC SES0
 /METHOD=ENTER MRN0
 /METHOD=ENTER MRN0xRace MRN0xSES0 MRN0xGender MRN0xIdeology0.
```

Regression

Notes

Output Created		15-DEC-2021 13:11:11
Comments		
Input	Data	C: \Users\njs5478\Dropbox\HM and COVID\0. Revise and Resubmit\2. R and R Data\Study 2b\Study2b_Data.sav
	Active Dataset	DataSet1
	Filter	<none>
	Weight	<none>
	Split File	<none>
	N of Rows in Working Data File	198
Missing Value Handling	Definition of Missing	User-defined missing values are treated as missing.
	Cases Used	Statistics are based on cases with no missing values for any variable used.
Syntax		REGRESSION /MISSING LISTWISE /STATISTICS COEFF OUTS R ANOVA CHANGE ZPP /CRITERIA=PIN(.05) POUT(.10) /NOORIGIN /DEPENDENT Conspiracy_Tot /METHOD=ENTER Ideology0 /METHOD=ENTER GenderCC RaceCC SES0 /METHOD=ENTER MRN0 /METHOD=ENTER MRN0xRace MRN0xSES0 MRN0xGender MRN0xIdeology0.
Resources	Processor Time	00:00:00.03
	Elapsed Time	00:00:00.03

Notes

	Memory Required	32640 bytes
	Additional Memory Required for Residual Plots	0 bytes

Variables Entered/Removed[a]

Model	Variables Entered	Variables Removed	Method
1	Ideology0[b]	.	Enter
2	SES0, GenderCC, RaceCC[b]	.	Enter
3	MRN0[b]	.	Enter
4	MRN0xGender, MRN0xSES0, MRN0xIdeology0, MRN0xRace[b]	.	Enter

a. Dependent Variable: Conspiracy_Tot

b. All requested variables entered.

Model Summary

Model	R	R Square	Adjusted R Square	Std. Error of the Estimate	Change Statistics		
					R Square Change	F Change	df1
1	.513[a]	.264	.260	.55505	.264	69.443	1
2	.515[b]	.265	.250	.55881	.002	.133	3
3	.583[c]	.340	.323	.53085	.075	21.649	1
4	.586[d]	.344	.312	.53511	.003	.246	4

Model Summary

Model	Change Statistics	
	df2	Sig. F Change
1	194	.000
2	191	.940
3	190	.000
4	186	.912

a. Predictors: (Constant), Ideology0

b. Predictors: (Constant), Ideology0, SES0, GenderCC, RaceCC

c. Predictors: (Constant), Ideology0, SES0, GenderCC, RaceCC, MRN0

d. Predictors: (Constant), Ideology0, SES0, GenderCC, RaceCC, MRN0, MRN0xGender, MRN0xSES0, MRN0xIdeology0, MRN0xRace

ANOVA[a]

Model		Sum of Squares	df	Mean Square	F	Sig.
1	Regression	21.394	1	21.394	69.443	.000[b]
	Residual	59.768	194	.308		
	Total	81.162	195			
2	Regression	21.519	4	5.380	17.228	.000[c]
	Residual	59.643	191	.312		
	Total	81.162	195			
3	Regression	27.620	5	5.524	19.602	.000[d]
	Residual	53.542	190	.282		
	Total	81.162	195			
4	Regression	27.901	9	3.100	10.827	.000[e]
	Residual	53.260	186	.286		
	Total	81.162	195			

a. Dependent Variable: Conspiracy_Tot

b. Predictors: (Constant), Ideology0

c. Predictors: (Constant), Ideology0, SES0, GenderCC, RaceCC

d. Predictors: (Constant), Ideology0, SES0, GenderCC, RaceCC, MRN0

e. Predictors: (Constant), Ideology0, SES0, GenderCC, RaceCC, MRN0, MRN0xGender, MRN0xSES0, MRN0xIdeology0, MRN0xRace

Coefficients[a]

Model		Unstandardized Coefficients B	Unstandardized Coefficients Std. Error	Standardized Coefficients Beta	t	Sig.
1	(Constant)	1.702	.040		42.926	.000
	Ideology0	.163	.020	.513	8.333	.000
2	(Constant)	1.702	.050		33.822	.000
	Ideology0	.163	.020	.515	8.185	.000
	GenderCC	.008	.041	.013	.203	.840
	RaceCC	.000	.051	-.001	-.009	.993
	SES0	-.029	.047	-.038	-.613	.541
3	(Constant)	1.677	.048		34.847	.000
	Ideology0	.099	.023	.313	4.245	.000
	GenderCC	-.054	.041	-.084	-1.317	.190
	RaceCC	.045	.050	.056	.911	.363
	SES0	-.049	.045	-.066	-1.102	.272
	MRN0	.225	.048	.362	4.653	.000
4	(Constant)	1.663	.055		30.436	.000
	Ideology0	.097	.024	.306	3.986	.000
	GenderCC	-.050	.042	-.077	-1.195	.233
	RaceCC	.051	.051	.064	1.003	.317
	SES0	-.044	.046	-.059	-.972	.332
	MRN0	.242	.055	.388	4.419	.000
	MRN0xRace	-.018	.047	-.029	-.384	.702
	MRN0xSES0	-.020	.044	-.028	-.464	.643
	MRN0xGender	-.017	.041	-.026	-.412	.681
	MRN0xIdeology0	.014	.020	.044	.700	.485

Coefficients[a]

Model		Correlations		
		Zero-order	Partial	Part
1	(Constant)			
	Ideology0	.513	.513	.513
2	(Constant)			
	Ideology0	.513	.510	.508
	GenderCC	.040	.015	.013
	RaceCC	.067	-.001	-.001
	SES0	-.012	-.044	-.038
3	(Constant)			
	Ideology0	.513	.294	.250
	GenderCC	.040	-.095	-.078
	RaceCC	.067	.066	.054
	SES0	-.012	-.080	-.065
	MRN0	.493	.320	.274
4	(Constant)			
	Ideology0	.513	.281	.237
	GenderCC	.040	-.087	-.071
	RaceCC	.067	.073	.060
	SES0	-.012	-.071	-.058
	MRN0	.493	.308	.262
	MRN0xRace	.284	-.028	-.023
	MRN0xSES0	-.053	-.034	-.028
	MRN0xGender	-.052	-.030	-.024
	MRN0xIdeology0	-.023	.051	.042

a. Dependent Variable: Conspiracy_Tot

Excluded Variables[a]

Model		Beta In	t	Sig.	Partial Correlation	Collinearity Statistics Tolerance
1	GenderCC	.010[b]	.156	.876	.011	.996
	RaceCC	-.003[b]	-.051	.959	-.004	.981
	SES0	-.037[b]	-.601	.549	-.043	.998
	MRN0	.301[b]	4.237	.000	.292	.692
	MRN0xRace	.100[b]	1.505	.134	.108	.851
	MRN0xSES0	.001[b]	.010	.992	.001	.989
	MRN0xGender	.006[b]	.089	.929	.006	.987
	MRN0xIdeology0	.019[b]	.304	.762	.022	.994
2	MRN0	.362[c]	4.653	.000	.320	.575
	MRN0xRace	.110[c]	1.569	.118	.113	.781
	MRN0xSES0	.001[c]	.014	.989	.001	.979
	MRN0xGender	.008[c]	.132	.895	.010	.970
	MRN0xIdeology0	.016[c]	.260	.795	.019	.970
3	MRN0xRace	-.020[d]	-.268	.789	-.020	.652
	MRN0xSES0	-.036[d]	-.594	.554	-.043	.963
	MRN0xGender	-.015[d]	-.253	.800	-.018	.963
	MRN0xIdeology0	.041[d]	.687	.493	.050	.963

a. Dependent Variable: Conspiracy_Tot

b. Predictors in the Model: (Constant), Ideology0

c. Predictors in the Model: (Constant), Ideology0, SES0, GenderCC, RaceCC

d. Predictors in the Model: (Constant), Ideology0, SES0, GenderCC, RaceCC, MRN0

```
 **Regression Analyses WITH Nationalism**

**Including PParty

REGRESSION
  /MISSING LISTWISE
  /STATISTICS COEFF OUTS R ANOVA CHANGE ZPP
  /CRITERIA=PIN(.05) POUT(.10)
  /NOORIGIN
  /DEPENDENT Concern_Tot
```

```
/METHOD=ENTER Party0
/METHOD=ENTER GenderCC RaceCC SES0
 /METHOD=ENTER National0
/METHOD=ENTER MRN0
/METHOD=ENTER MRN0xRace MRN0xSES0 MRN0xGender MRN0xParty0 MRN0xNational0.
```

Regression

Notes

Output Created		15-DEC-2021 13:11:11
Comments		
Input	Data	C: \Users\njs5478\Dropbox\HM and COVID\0. Revise and Resubmit\2. R and R Data\Study 2b\Study2b_Data.sav
	Active Dataset	DataSet1
	Filter	<none>
	Weight	<none>
	Split File	<none>
	N of Rows in Working Data File	198
Missing Value Handling	Definition of Missing	User-defined missing values are treated as missing.
	Cases Used	Statistics are based on cases with no missing values for any variable used.

Notes

Syntax		REGRESSION<br>/MISSING LISTWISE<br>/STATISTICS COEFF OUTS R ANOVA CHANGE ZPP<br>/CRITERIA=PIN(.05) POUT(.10)<br>/NOORIGIN<br>/DEPENDENT Concern_Tot<br>/METHOD=ENTER Party0<br>/METHOD=ENTER GenderCC RaceCC SES0<br>/METHOD=ENTER National0<br>/METHOD=ENTER MRN0<br>/METHOD=ENTER MRN0xRace MRN0xSES0 MRN0xGender MRN0xParty0 MRN0xNational0.
Resources	Processor Time	00:00:00.03
	Elapsed Time	00:00:00.03
	Memory Required	34768 bytes
	Additional Memory Required for Residual Plots	0 bytes

Variables Entered/Removed[a]

Model	Variables Entered	Variables Removed	Method
1	Party0[b]	.	Enter
2	GenderCC, SES0, RaceCC[b]	.	Enter
3	National0[b]	.	Enter
4	MRN0[b]	.	Enter
5	MRN0xSES0, MRN0xGender, MRN0xParty0, MRN0xNational0, ...[b]	.	Enter

a. Dependent Variable: Concern_Tot

b. All requested variables entered.

Model Summary

Model	R	R Square	Adjusted R Square	Std. Error of the Estimate	Change Statistics		
					R Square Change	F Change	df1
1	.485[a]	.235	.231	1.42985	.235	59.558	1
2	.528[b]	.279	.264	1.39911	.044	3.873	3
3	.530[c]	.281	.262	1.40090	.002	.512	1
4	.535[d]	.286	.263	1.39971	.005	1.325	1
5	.553[e]	.306	.265	1.39825	.020	1.079	5

Model Summary

Model	Change Statistics	
	df2	Sig. F Change
1	194	.000
2	191	.010
3	190	.475
4	189	.251
5	184	.374

a. Predictors: (Constant), Party0

b. Predictors: (Constant), Party0, GenderCC, SES0, RaceCC

c. Predictors: (Constant), Party0, GenderCC, SES0, RaceCC, National0

d. Predictors: (Constant), Party0, GenderCC, SES0, RaceCC, National0, MRN0

e. Predictors: (Constant), Party0, GenderCC, SES0, RaceCC, National0, MRN0, MRN0xSES0, MRN0xGender, MRN0xParty0, MRN0xNational0, MRN0xRace

ANOVA[a]

Model		Sum of Squares	df	Mean Square	F	Sig.
1	Regression	121.766	1	121.766	59.558	.000[b]
	Residual	396.630	194	2.044		
	Total	518.396	195			
2	Regression	144.510	4	36.128	18.456	.000[c]
	Residual	373.885	191	1.958		
	Total	518.396	195			
3	Regression	145.515	5	29.103	14.829	.000[d]
	Residual	372.881	190	1.963		
	Total	518.396	195			
4	Regression	148.111	6	24.685	12.600	.000[e]
	Residual	370.284	189	1.959		
	Total	518.396	195			
5	Regression	158.658	11	14.423	7.377	.000[f]
	Residual	359.738	184	1.955		
	Total	518.396	195			

a. Dependent Variable: Concern_Tot

b. Predictors: (Constant), Party0

c. Predictors: (Constant), Party0, GenderCC, SES0, RaceCC

d. Predictors: (Constant), Party0, GenderCC, SES0, RaceCC, National0

e. Predictors: (Constant), Party0, GenderCC, SES0, RaceCC, National0, MRN0

f. Predictors: (Constant), Party0, GenderCC, SES0, RaceCC, National0, MRN0, MRN0xSES0, MRN0xGender, MRN0xParty0, MRN0xNational0, MRN0xRace

Coefficients[a]

Model		Unstandardized Coefficients B	Unstandardized Coefficients Std. Error	Standardized Coefficients Beta	t	Sig.
1	(Constant)	4.296	.102		42.065	.000
	Party0	-.504	.065	-.485	-7.717	.000
2	(Constant)	4.263	.126		33.761	.000
	Party0	-.504	.065	-.484	-7.756	.000
	GenderCC	-.324	.102	-.199	-3.190	.002
	RaceCC	.050	.129	.025	.391	.696
	SES0	-.063	.118	-.033	-.533	.595
3	(Constant)	4.265	.126		33.725	.000
	Party0	-.526	.072	-.505	-7.312	.000
	GenderCC	-.327	.102	-.201	-3.216	.002
	RaceCC	.045	.129	.022	.351	.726
	SES0	-.074	.119	-.039	-.620	.536
	National0	.063	.088	.050	.715	.475
4	(Constant)	4.283	.127		33.636	.000
	Party0	-.500	.075	-.480	-6.633	.000
	GenderCC	-.286	.108	-.176	-2.648	.009
	RaceCC	.013	.132	.006	.097	.923
	SES0	-.066	.119	-.035	-.554	.580
	National0	.109	.097	.086	1.128	.261
	MRN0	-.147	.127	-.093	-1.151	.251
5	(Constant)	4.220	.143		29.419	.000
	Party0	-.507	.077	-.487	-6.561	.000
	GenderCC	-.263	.109	-.161	-2.401	.017
	RaceCC	.019	.135	.009	.137	.891
	SES0	-.049	.119	-.026	-.412	.681
	National0	.138	.103	.108	1.334	.184
	MRN0	-.061	.143	-.039	-.427	.670
	MRN0xRace	-.129	.126	-.082	-1.028	.305
	MRN0xSES0	.110	.117	.060	.937	.350
	MRN0xGender	-.133	.108	-.080	-1.226	.222
	MRN0xParty0	.132	.072	.127	1.834	.068
	MRN0xNational0	-.011	.090	-.009	-.118	.906

Coefficients[a]

Model		Correlations Zero-order	Partial	Part
1	(Constant)			
	Party0	-.485	-.485	-.485
2	(Constant)			
	Party0	-.485	-.489	-.477
	GenderCC	-.215	-.225	-.196
	RaceCC	-.030	.028	.024
	SES0	-.062	-.039	-.033
3	(Constant)			
	Party0	-.485	-.469	-.450
	GenderCC	-.215	-.227	-.198
	RaceCC	-.030	.025	.022
	SES0	-.062	-.045	-.038
	National0	-.185	.052	.044
4	(Constant)			
	Party0	-.485	-.435	-.408
	GenderCC	-.215	-.189	-.163
	RaceCC	-.030	.007	.006
	SES0	-.062	-.040	-.034
	National0	-.185	.082	.069
	MRN0	-.312	-.083	-.071
5	(Constant)			
	Party0	-.485	-.435	-.403
	GenderCC	-.215	-.174	-.147
	RaceCC	-.030	.010	.008
	SES0	-.062	-.030	-.025
	National0	-.185	.098	.082
	MRN0	-.312	-.031	-.026
	MRN0xRace	-.277	-.076	-.063
	MRN0xSES0	.046	.069	.058
	MRN0xGender	.021	-.090	-.075
	MRN0xParty0	.146	.134	.113
	MRN0xNational0	.055	-.009	-.007

a. Dependent Variable: Concern_Tot

Excluded Variables[a]

Model		Beta In	t	Sig.	Partial Correlation	Collinearity Statistics Tolerance
1	GenderCC	-.206[b]	-3.360	.001	-.235	1.000
	RaceCC	.055[b]	.856	.393	.062	.971
	SES0	-.050[b]	-.789	.431	-.057	.999
	National0	.034[b]	.480	.632	.035	.808
	MRN0	-.134[b]	-1.955	.052	-.139	.827
	MRN0xRace	-.128[b]	-1.932	.055	-.138	.886
	MRN0xSES0	.020[b]	.318	.751	.023	.997
	MRN0xGender	-.041[b]	-.647	.518	-.047	.984
	MRN0xParty0	.120[b]	1.927	.055	.137	.997
	MRN0xNational0	-.015[b]	-.242	.809	-.017	.979
2	National0	.050[c]	.715	.475	.052	.790
	MRN0	-.055[c]	-.752	.453	-.054	.695
	MRN0xRace	-.071[c]	-1.041	.299	-.075	.807
	MRN0xSES0	.031[c]	.507	.613	.037	.989
	MRN0xGender	-.043[c]	-.686	.494	-.050	.966
	MRN0xParty0	.095[c]	1.530	.128	.110	.970
	MRN0xNational0	-.002[c]	-.033	.974	-.002	.970
3	MRN0	-.093[d]	-1.151	.251	-.083	.577
	MRN0xRace	-.081[d]	-1.171	.243	-.085	.786
	MRN0xSES0	.033[d]	.536	.593	.039	.987
	MRN0xGender	-.049[d]	-.783	.435	-.057	.950
	MRN0xParty0	.103[d]	1.646	.101	.119	.951
	MRN0xNational0	.014[d]	.212	.832	.015	.866
4	MRN0xRace	-.058[e]	-.757	.450	-.055	.647
	MRN0xSES0	.041[e]	.653	.514	.048	.977
	MRN0xGender	-.048[e]	-.767	.444	-.056	.950
	MRN0xParty0	.096[e]	1.514	.132	.110	.937
	MRN0xNational0	.019[e]	.281	.779	.020	.863

a. Dependent Variable: Concern_Tot

b. Predictors in the Model: (Constant), Party0

c. Predictors in the Model: (Constant), Party0, GenderCC, SES0, RaceCC

d. Predictors in the Model: (Constant), Party0, GenderCC, SES0, RaceCC, National0

e. Predictors in the Model: (Constant), Party0, GenderCC, SES0, RaceCC, National0, MRN0

```
REGRESSION
 /MISSING LISTWISE
 /STATISTICS COEFF OUTS R ANOVA CHANGE ZPP
 /CRITERIA=PIN(.05) POUT(.10)
 /NOORIGIN
 /DEPENDENT Finance_Tot
 /METHOD=ENTER Party0
 /METHOD=ENTER GenderCC RaceCC SES0
  /METHOD=ENTER National0
 /METHOD=ENTER MRN0
 /METHOD=ENTER MRN0xRace MRN0xSES0 MRN0xGender MRN0xParty0 MRN0xNational0.
```

Regression

Notes

Output Created		15-DEC-2021 13:11:11
Comments		
Input	Data	C:\Users\njs5478\Dropbox\HM and COVID\0. Revise and Resubmit\2. R and R Data\Study 2b\Study2b_Data.sav
	Active Dataset	DataSet1
	Filter	<none>
	Weight	<none>
	Split File	<none>
	N of Rows in Working Data File	198
Missing Value Handling	Definition of Missing	User-defined missing values are treated as missing.
	Cases Used	Statistics are based on cases with no missing values for any variable used.

Notes

Syntax		REGRESSION /MISSING LISTWISE /STATISTICS COEFF OUTS R ANOVA CHANGE ZPP /CRITERIA=PIN(.05) POUT(.10) /NOORIGIN /DEPENDENT Finance_Tot /METHOD=ENTER Party0 /METHOD=ENTER GenderCC RaceCC SES0 /METHOD=ENTER National0 /METHOD=ENTER MRN0 /METHOD=ENTER MRN0xRace MRN0xSES0 MRN0xGender MRN0xParty0 MRN0xNational0.
Resources	Processor Time	00:00:00.03
	Elapsed Time	00:00:00.03
	Memory Required	34768 bytes
	Additional Memory Required for Residual Plots	0 bytes

Variables Entered/Removed[a]

Model	Variables Entered	Variables Removed	Method
1	Party0[b]	.	Enter
2	GenderCC, SES0, RaceCC[b]	.	Enter
3	National0[b]	.	Enter
4	MRN0[b]	.	Enter
5	MRN0xSES0, MRN0xGender, MRN0xParty0, MRN0xNational0, ...[b]	.	Enter

a. Dependent Variable: Finance_Tot

b. All requested variables entered.

Model Summary

Model	R	R Square	Adjusted R Square	Std. Error of the Estimate	Change Statistics		
					R Square Change	F Change	df1
1	.029[a]	.001	-.004	1.99120	.001	.164	1
2	.341[b]	.116	.098	1.88745	.115	8.305	3
3	.341[c]	.116	.093	1.89235	.000	.012	1
4	.358[d]	.128	.100	1.88454	.012	2.579	1
5	.361[e]	.131	.079	1.90720	.003	.107	5

Model Summary

Model	Change Statistics	
	df2	Sig. F Change
1	194	.686
2	191	.000
3	190	.914
4	189	.110
5	184	.991

a. Predictors: (Constant), Party0

b. Predictors: (Constant), Party0, GenderCC, SES0, RaceCC

c. Predictors: (Constant), Party0, GenderCC, SES0, RaceCC, National0

d. Predictors: (Constant), Party0, GenderCC, SES0, RaceCC, National0, MRN0

e. Predictors: (Constant), Party0, GenderCC, SES0, RaceCC, National0, MRN0, MRN0xSES0, MRN0xGender, MRN0xParty0, MRN0xNational0, MRN0xRace

ANOVA[a]

Model		Sum of Squares	df	Mean Square	F	Sig.
1	Regression	.650	1	.650	.164	.686[b]
	Residual	769.187	194	3.965		
	Total	769.837	195			
2	Regression	89.404	4	22.351	6.274	.000[c]
	Residual	680.432	191	3.562		
	Total	769.837	195			
3	Regression	89.447	5	17.889	4.996	.000[d]
	Residual	680.390	190	3.581		
	Total	769.837	195			
4	Regression	98.607	6	16.435	4.628	.000[e]
	Residual	671.229	189	3.551		
	Total	769.837	195			
5	Regression	100.550	11	9.141	2.513	.006[f]
	Residual	669.287	184	3.637		
	Total	769.837	195			

a. Dependent Variable: Finance_Tot

b. Predictors: (Constant), Party0

c. Predictors: (Constant), Party0, GenderCC, SES0, RaceCC

d. Predictors: (Constant), Party0, GenderCC, SES0, RaceCC, National0

e. Predictors: (Constant), Party0, GenderCC, SES0, RaceCC, National0, MRN0

f. Predictors: (Constant), Party0, GenderCC, SES0, RaceCC, National0, MRN0, MRN0xSES0, MRN0xGender, MRN0xParty0, MRN0xNational0, MRN0xRace

Coefficients[a]

Model		Unstandardized Coefficients B	Unstandardized Coefficients Std. Error	Standardized Coefficients Beta	t	Sig.
1	(Constant)	3.796	.142		26.690	.000
	Party0	-.037	.091	-.029	-.405	.686
2	(Constant)	3.728	.170		21.888	.000
	Party0	-.033	.088	-.026	-.375	.708
	GenderCC	-.376	.137	-.190	-2.748	.007
	RaceCC	.106	.173	.043	.613	.541
	SES0	-.601	.159	-.259	-3.788	.000
3	(Constant)	3.728	.171		21.819	.000
	Party0	-.028	.097	-.022	-.292	.771
	GenderCC	-.376	.137	-.190	-2.733	.007
	RaceCC	.107	.174	.043	.616	.538
	SES0	-.599	.160	-.258	-3.733	.000
	National0	-.013	.119	-.008	-.109	.914
4	(Constant)	3.693	.171		21.542	.000
	Party0	-.077	.101	-.061	-.762	.447
	GenderCC	-.454	.145	-.229	-3.124	.002
	RaceCC	.168	.177	.068	.947	.345
	SES0	-.614	.160	-.264	-3.833	.000
	National0	-.099	.130	-.064	-.763	.447
	MRN0	.276	.172	.144	1.606	.110
5	(Constant)	3.746	.196		19.149	.000
	Party0	-.081	.105	-.064	-.770	.442
	GenderCC	-.447	.149	-.226	-2.998	.003
	RaceCC	.173	.184	.070	.939	.349
	SES0	-.616	.163	-.265	-3.786	.000
	National0	-.129	.141	-.083	-.914	.362
	MRN0	.275	.195	.143	1.413	.159
	MRN0xRace	.016	.171	.008	.094	.925
	MRN0xSES0	.024	.159	.011	.154	.878
	MRN0xGender	-.017	.148	-.009	-.117	.907
	MRN0xParty0	.008	.098	.006	.081	.936
	MRN0xNational0	-.084	.122	-.056	-.686	.494

Coefficients[a]

Model		Correlations		
		Zero-order	Partial	Part
1	(Constant)			
	Party0	-.029	-.029	-.029
2	(Constant)			
	Party0	-.029	-.027	-.025
	GenderCC	-.219	-.195	-.187
	RaceCC	.060	.044	.042
	SES0	-.275	-.264	-.258
3	(Constant)			
	Party0	-.029	-.021	-.020
	GenderCC	-.219	-.194	-.186
	RaceCC	.060	.045	.042
	SES0	-.275	-.261	-.255
	National0	-.057	-.008	-.007
4	(Constant)			
	Party0	-.029	-.055	-.052
	GenderCC	-.219	-.222	-.212
	RaceCC	.060	.069	.064
	SES0	-.275	-.269	-.260
	National0	-.057	-.055	-.052
	MRN0	-.030	.116	.109
5	(Constant)			
	Party0	-.029	-.057	-.053
	GenderCC	-.219	-.216	-.206
	RaceCC	.060	.069	.065
	SES0	-.275	-.269	-.260
	National0	-.057	-.067	-.063
	MRN0	-.030	.104	.097
	MRN0xRace	-.050	.007	.006
	MRN0xSES0	-.001	.011	.011
	MRN0xGender	-.020	-.009	-.008
	MRN0xParty0	.019	.006	.006
	MRN0xNational0	-.033	-.050	-.047

a. Dependent Variable: Finance_Tot

Excluded Variables[a]

Model		Beta In	t	Sig.	Partial Correlation	Collinearity Statistics Tolerance
1	GenderCC	-.219[b]	-3.112	.002	-.219	1.000
	RaceCC	.067[b]	.924	.356	.066	.971
	SES0	-.274[b]	-3.965	.000	-.274	.999
	National0	-.055[b]	-.690	.491	-.050	.808
	MRN0	-.021[b]	-.269	.788	-.019	.827
	MRN0xRace	-.045[b]	-.595	.553	-.043	.886
	MRN0xSES0	-.003[b]	-.040	.968	-.003	.997
	MRN0xGender	-.024[b]	-.335	.738	-.024	.984
	MRN0xParty0	.017[b]	.236	.814	.017	.997
	MRN0xNational0	-.038[b]	-.528	.598	-.038	.979
2	National0	-.008[c]	-.109	.914	-.008	.790
	MRN0	.115[c]	1.419	.158	.102	.695
	MRN0xRace	.046[c]	.600	.549	.044	.807
	MRN0xSES0	.012[c]	.175	.861	.013	.989
	MRN0xGender	-.013[c]	-.187	.852	-.014	.966
	MRN0xParty0	-.025[c]	-.362	.718	-.026	.970
	MRN0xNational0	-.037[c]	-.537	.592	-.039	.970
3	MRN0	.144[d]	1.606	.110	.116	.577
	MRN0xRace	.048[d]	.625	.533	.045	.786
	MRN0xSES0	.012[d]	.170	.865	.012	.987
	MRN0xGender	-.012[d]	-.174	.862	-.013	.950
	MRN0xParty0	-.027[d]	-.380	.704	-.028	.951
	MRN0xNational0	-.044[d]	-.604	.546	-.044	.866
4	MRN0xRace	-.004[e]	-.051	.959	-.004	.647
	MRN0xSES0	.001[e]	.013	.990	.001	.977
	MRN0xGender	-.014[e]	-.198	.844	-.014	.950
	MRN0xParty0	-.013[e]	-.184	.854	-.013	.937
	MRN0xNational0	-.051[e]	-.703	.483	-.051	.863

a. Dependent Variable: Finance_Tot

b. Predictors in the Model: (Constant), Party0

c. Predictors in the Model: (Constant), Party0, GenderCC, SES0, RaceCC

d. Predictors in the Model: (Constant), Party0, GenderCC, SES0, RaceCC, National0

e. Predictors in the Model: (Constant), Party0, GenderCC, SES0, RaceCC, National0, MRN0

```
REGRESSION
 /MISSING LISTWISE
 /STATISTICS COEFF OUTS R ANOVA CHANGE ZPP
 /CRITERIA=PIN(.05) POUT(.10)
 /NOORIGIN
 /DEPENDENT Psychology_Tot
 /METHOD=ENTER Party0
 /METHOD=ENTER GenderCC RaceCC SES0
  /METHOD=ENTER National0
 /METHOD=ENTER MRN0
 /METHOD=ENTER MRN0xRace MRN0xSES0 MRN0xGender MRN0xParty0 MRN0xNational0.
```

Regression

Notes

Output Created		15-DEC-2021 13:11:11
Comments		
Input	Data	C: \Users\njs5478\Dropbox\HM and COVID\0. Revise and Resubmit\2. R and R Data\Study 2b\Study2b_Data.sav
	Active Dataset	DataSet1
	Filter	<none>
	Weight	<none>
	Split File	<none>
	N of Rows in Working Data File	198
Missing Value Handling	Definition of Missing	User-defined missing values are treated as missing.
	Cases Used	Statistics are based on cases with no missing values for any variable used.

Notes

Syntax		REGRESSION<br>/MISSING LISTWISE<br>/STATISTICS COEFF OUTS R ANOVA CHANGE ZPP<br>/CRITERIA=PIN(.05) POUT(.10)<br>/NOORIGIN<br>/DEPENDENT Psychology_Tot<br>/METHOD=ENTER Party0<br>/METHOD=ENTER GenderCC RaceCC SES0<br>/METHOD=ENTER National0<br>/METHOD=ENTER MRN0<br>/METHOD=ENTER MRN0xRace MRN0xSES0 MRN0xGender MRN0xParty0 MRN0xNational0.
Resources	Processor Time	00:00:00.02
	Elapsed Time	00:00:00.02
	Memory Required	34768 bytes
	Additional Memory Required for Residual Plots	0 bytes

Variables Entered/Removed[a]

Model	Variables Entered	Variables Removed	Method
1	Party0[b]	.	Enter
2	GenderCC, SES0, RaceCC[b]	.	Enter
3	National0[b]	.	Enter
4	MRN0[b]	.	Enter
5	MRN0xSES0, MRN0xGender, MRN0xParty0, MRN0xNational0, ...[b]	.	Enter

a. Dependent Variable: Psychology_Tot

b. All requested variables entered.

Model Summary

Model	R	R Square	Adjusted R Square	Std. Error of the Estimate	Change Statistics		
					R Square Change	F Change	df1
1	.354[a]	.125	.121	1.60103	.125	27.738	1
2	.467[b]	.218	.202	1.52513	.093	7.597	3
3	.467[c]	.218	.198	1.52913	.000	.001	1
4	.469[d]	.220	.195	1.53162	.002	.384	1
5	.501[e]	.251	.206	1.52110	.031	1.525	5

Model Summary

Model	Change Statistics	
	df2	Sig. F Change
1	194	.000
2	191	.000
3	190	.981
4	189	.536
5	184	.184

a. Predictors: (Constant), Party0

b. Predictors: (Constant), Party0, GenderCC, SES0, RaceCC

c. Predictors: (Constant), Party0, GenderCC, SES0, RaceCC, National0

d. Predictors: (Constant), Party0, GenderCC, SES0, RaceCC, National0, MRN0

e. Predictors: (Constant), Party0, GenderCC, SES0, RaceCC, National0, MRN0, MRN0xSES0, MRN0xGender, MRN0xParty0, MRN0xNational0, MRN0xRace

ANOVA[a]

Model		Sum of Squares	df	Mean Square	F	Sig.
1	Regression	71.100	1	71.100	27.738	.000[b]
	Residual	497.282	194	2.563		
	Total	568.383	195			
2	Regression	124.114	4	31.029	13.340	.000[c]
	Residual	444.268	191	2.326		
	Total	568.383	195			
3	Regression	124.116	5	24.823	10.616	.000[d]
	Residual	444.267	190	2.338		
	Total	568.383	195			
4	Regression	125.017	6	20.836	8.882	.000[e]
	Residual	443.365	189	2.346		
	Total	568.383	195			
5	Regression	142.656	11	12.969	5.605	.000[f]
	Residual	425.727	184	2.314		
	Total	568.383	195			

a. Dependent Variable: Psychology_Tot

b. Predictors: (Constant), Party0

c. Predictors: (Constant), Party0, GenderCC, SES0, RaceCC

d. Predictors: (Constant), Party0, GenderCC, SES0, RaceCC, National0

e. Predictors: (Constant), Party0, GenderCC, SES0, RaceCC, National0, MRN0

f. Predictors: (Constant), Party0, GenderCC, SES0, RaceCC, National0, MRN0, MRN0xSES0, MRN0xGender, MRN0xParty0, MRN0xNational0, MRN0xRace

Coefficients[a]

Model		Unstandardized Coefficients B	Unstandardized Coefficients Std. Error	Standardized Coefficients Beta	t	Sig.
1	(Constant)	4.113	.114		35.968	.000
	Party0	-.385	.073	-.354	-5.267	.000
2	(Constant)	3.974	.138		28.875	.000
	Party0	-.397	.071	-.364	-5.599	.000
	GenderCC	-.400	.111	-.235	-3.618	.000
	RaceCC	.225	.140	.105	1.603	.111
	SES0	-.247	.128	-.124	-1.926	.056
3	(Constant)	3.974	.138		28.788	.000
	Party0	-.397	.079	-.365	-5.062	.000
	GenderCC	-.401	.111	-.235	-3.606	.000
	RaceCC	.224	.141	.105	1.595	.112
	SES0	-.247	.130	-.124	-1.908	.058
	National0	.002	.096	.002	.024	.981
4	(Constant)	3.963	.139		28.443	.000
	Party0	-.413	.082	-.379	-5.006	.000
	GenderCC	-.425	.118	-.250	-3.600	.000
	RaceCC	.243	.144	.114	1.688	.093
	SES0	-.252	.130	-.126	-1.937	.054
	National0	-.025	.106	-.019	-.234	.815
	MRN0	.086	.139	.052	.620	.536
5	(Constant)	3.855	.156		24.709	.000
	Party0	-.418	.084	-.383	-4.965	.000
	GenderCC	-.392	.119	-.230	-3.298	.001
	RaceCC	.240	.147	.112	1.633	.104
	SES0	-.233	.130	-.117	-1.798	.074
	National0	-.001	.112	-.001	-.009	.993
	MRN0	.203	.155	.123	1.308	.192
	MRN0xRace	-.148	.137	-.089	-1.083	.280
	MRN0xSES0	.062	.127	.033	.488	.626
	MRN0xGender	-.087	.118	-.050	-.734	.464
	MRN0xParty0	.198	.078	.182	2.542	.012
	MRN0xNational0	-.021	.098	-.017	-.217	.828

Coefficients[a]

Model		Correlations Zero-order	Partial	Part
1	(Constant)			
	Party0	-.354	-.354	-.354
2	(Constant)			
	Party0	-.354	-.375	-.358
	GenderCC	-.268	-.253	-.231
	RaceCC	.075	.115	.103
	SES0	-.151	-.138	-.123
3	(Constant)			
	Party0	-.354	-.345	-.325
	GenderCC	-.268	-.253	-.231
	RaceCC	.075	.115	.102
	SES0	-.151	-.137	-.122
	National0	-.175	.002	.002
4	(Constant)			
	Party0	-.354	-.342	-.322
	GenderCC	-.268	-.253	-.231
	RaceCC	.075	.122	.108
	SES0	-.151	-.140	-.124
	National0	-.175	-.017	-.015
	MRN0	-.225	.045	.040
5	(Constant)			
	Party0	-.354	-.344	-.317
	GenderCC	-.268	-.236	-.210
	RaceCC	.075	.120	.104
	SES0	-.151	-.131	-.115
	National0	-.175	-.001	-.001
	MRN0	-.225	.096	.083
	MRN0xRace	-.223	-.080	-.069
	MRN0xSES0	.017	.036	.031
	MRN0xGender	.042	-.054	-.047
	MRN0xParty0	.209	.184	.162
	MRN0xNational0	.065	-.016	-.014

a. Dependent Variable: Psychology_Tot

Excluded Variables[a]

Model		Beta In	t	Sig.	Partial Correlation	Collinearity Statistics Tolerance
1	GenderCC	-.261[b]	-4.044	.000	-.279	1.000
	RaceCC	.139[b]	2.059	.041	.147	.971
	SES0	-.142[b]	-2.136	.034	-.152	.999
	National0	-.024[b]	-.324	.746	-.023	.808
	MRN0	-.095[b]	-1.285	.200	-.092	.827
	MRN0xRace	-.116[b]	-1.638	.103	-.117	.886
	MRN0xSES0	-.002[b]	-.030	.976	-.002	.997
	MRN0xGender	-.002[b]	-.035	.972	-.003	.984
	MRN0xParty0	.190[b]	2.876	.004	.203	.997
	MRN0xNational0	.014[b]	.199	.842	.014	.979
2	National0	.002[c]	.024	.981	.002	.790
	MRN0	.044[c]	.576	.565	.042	.695
	MRN0xRace	-.036[c]	-.500	.618	-.036	.807
	MRN0xSES0	.009[c]	.135	.892	.010	.989
	MRN0xGender	-.007[c]	-.113	.910	-.008	.966
	MRN0xParty0	.147[c]	2.291	.023	.164	.970
	MRN0xNational0	.024[c]	.371	.711	.027	.970
3	MRN0	.052[d]	.620	.536	.045	.577
	MRN0xRace	-.037[d]	-.509	.611	-.037	.786
	MRN0xSES0	.009[d]	.136	.892	.010	.987
	MRN0xGender	-.008[d]	-.117	.907	-.009	.950
	MRN0xParty0	.150[d]	2.312	.022	.166	.951
	MRN0xNational0	.028[d]	.400	.690	.029	.866
4	MRN0xRace	-.068[e]	-.847	.398	-.062	.647
	MRN0xSES0	.005[e]	.075	.940	.005	.977
	MRN0xGender	-.008[e]	-.126	.900	-.009	.950
	MRN0xParty0	.158[e]	2.408	.017	.173	.937
	MRN0xNational0	.025[e]	.364	.717	.027	.863

a. Dependent Variable: Psychology_Tot

b. Predictors in the Model: (Constant), Party0

c. Predictors in the Model: (Constant), Party0, GenderCC, SES0, RaceCC

d. Predictors in the Model: (Constant), Party0, GenderCC, SES0, RaceCC, National0

e. Predictors in the Model: (Constant), Party0, GenderCC, SES0, RaceCC, National0, MRN0

```
REGRESSION
 /MISSING LISTWISE
 /STATISTICS COEFF OUTS R ANOVA CHANGE ZPP
 /CRITERIA=PIN(.05) POUT(.10)
 /NOORIGIN
 /DEPENDENT Risk_Rules
 /METHOD=ENTER Party0
 /METHOD=ENTER GenderCC RaceCC SES0
  /METHOD=ENTER National0
 /METHOD=ENTER MRN0
 /METHOD=ENTER MRN0xRace MRN0xSES0 MRN0xGender MRN0xParty0 MRN0xNational0.
```

Regression

Notes

Output Created		15-DEC-2021 13:11:11
Comments		
Input	Data	C: \Users\njs5478\Dropbox\HM and COVID\0. Revise and Resubmit\2. R and R Data\Study 2b\Study2b_Data.sav
	Active Dataset	DataSet1
	Filter	<none>
	Weight	<none>
	Split File	<none>
	N of Rows in Working Data File	198
Missing Value Handling	Definition of Missing	User-defined missing values are treated as missing.
	Cases Used	Statistics are based on cases with no missing values for any variable used.

Notes

Syntax		REGRESSION<br>/MISSING LISTWISE<br>/STATISTICS COEFF OUTS R ANOVA CHANGE ZPP<br>/CRITERIA=PIN(.05) POUT(.10)<br>/NOORIGIN<br>/DEPENDENT Risk_Rules<br>/METHOD=ENTER Party0<br>/METHOD=ENTER GenderCC RaceCC SES0<br>/METHOD=ENTER National0<br>/METHOD=ENTER MRN0<br>/METHOD=ENTER MRN0xRace MRN0xSES0 MRN0xGender MRN0xParty0 MRN0xNational0.
Resources	Processor Time	00:00:00.02
	Elapsed Time	00:00:00.02
	Memory Required	34768 bytes
	Additional Memory Required for Residual Plots	0 bytes

Variables Entered/Removed[a]

Model	Variables Entered	Variables Removed	Method
1	Party0[b]	.	Enter
2	GenderCC, SES0, RaceCC[b]	.	Enter
3	National0[b]	.	Enter
4	MRN0[b]	.	Enter
5	MRN0xSES0, MRN0xGender, MRN0xParty0, MRN0xNational0, ...[b]	.	Enter

a. Dependent Variable: Risk_Rules

b. All requested variables entered.

Model Summary

					Change Statistics		
Model	R	R Square	Adjusted R Square	Std. Error of the Estimate	R Square Change	F Change	df1
1	.532[a]	.284	.280	.93951	.284	76.770	1
2	.548[b]	.300	.285	.93606	.016	1.478	3
3	.559[c]	.312	.294	.93004	.013	3.480	1
4	.575[d]	.330	.309	.92025	.018	5.065	1
5	.585[e]	.342	.302	.92468	.011	.638	5

Model Summary

	Change Statistics	
Model	df2	Sig. F Change
1	194	.000
2	191	.222
3	190	.064
4	189	.026
5	184	.671

a. Predictors: (Constant), Party0

b. Predictors: (Constant), Party0, GenderCC, SES0, RaceCC

c. Predictors: (Constant), Party0, GenderCC, SES0, RaceCC, National0

d. Predictors: (Constant), Party0, GenderCC, SES0, RaceCC, National0, MRN0

e. Predictors: (Constant), Party0, GenderCC, SES0, RaceCC, National0, MRN0, MRN0xSES0, MRN0xGender, MRN0xParty0, MRN0xNational0, MRN0xRace

ANOVA[a]

Model		Sum of Squares	df	Mean Square	F	Sig.
1	Regression	67.764	1	67.764	76.770	.000[b]
	Residual	171.241	194	.883		
	Total	239.005	195			
2	Regression	71.650	4	17.912	20.443	.000[c]
	Residual	167.355	191	.876		
	Total	239.005	195			
3	Regression	74.660	5	14.932	17.263	.000[d]
	Residual	164.345	190	.865		
	Total	239.005	195			
4	Regression	78.950	6	13.158	15.538	.000[e]
	Residual	160.055	189	.847		
	Total	239.005	195			
5	Regression	81.678	11	7.425	8.684	.000[f]
	Residual	157.327	184	.855		
	Total	239.005	195			

a. Dependent Variable: Risk_Rules

b. Predictors: (Constant), Party0

c. Predictors: (Constant), Party0, GenderCC, SES0, RaceCC

d. Predictors: (Constant), Party0, GenderCC, SES0, RaceCC, National0

e. Predictors: (Constant), Party0, GenderCC, SES0, RaceCC, National0, MRN0

f. Predictors: (Constant), Party0, GenderCC, SES0, RaceCC, National0, MRN0, MRN0xSES0, MRN0xGender, MRN0xParty0, MRN0xNational0, MRN0xRace

Coefficients[a]

Model		Unstandardized Coefficients B	Unstandardized Coefficients Std. Error	Standardized Coefficients Beta	t	Sig.
1	(Constant)	3.305	.067		49.254	.000
	Party0	.376	.043	.532	8.762	.000
2	(Constant)	3.325	.084		39.359	.000
	Party0	.378	.043	.534	8.686	.000
	GenderCC	.135	.068	.123	1.994	.048
	RaceCC	-.030	.086	-.022	-.347	.729
	SES0	-.025	.079	-.020	-.324	.746
3	(Constant)	3.329	.084		39.651	.000
	Party0	.340	.048	.481	7.113	.000
	GenderCC	.129	.068	.117	1.915	.057
	RaceCC	-.039	.086	-.028	-.451	.653
	SES0	-.044	.079	-.034	-.564	.574
	National0	.109	.059	.126	1.866	.064
4	(Constant)	3.306	.084		39.484	.000
	Party0	.306	.050	.433	6.179	.000
	GenderCC	.076	.071	.069	1.070	.286
	RaceCC	.003	.087	.002	.035	.972
	SES0	-.054	.078	-.042	-.697	.487
	National0	.050	.064	.058	.787	.432
	MRN0	.189	.084	.176	2.251	.026
5	(Constant)	3.318	.095		34.982	.000
	Party0	.300	.051	.424	5.860	.000
	GenderCC	.071	.072	.064	.975	.331
	RaceCC	-.019	.089	-.014	-.214	.831
	SES0	-.060	.079	-.047	-.767	.444
	National0	.034	.068	.040	.502	.616
	MRN0	.153	.094	.143	1.625	.106
	MRN0xRace	.106	.083	.099	1.277	.203
	MRN0xSES0	-.036	.077	-.030	-.472	.637
	MRN0xGender	.043	.072	.038	.601	.549
	MRN0xParty0	.040	.047	.056	.835	.405
	MRN0xNational0	-.039	.059	-.047	-.652	.515

Coefficients[a]

Model		Correlations		
		Zero-order	Partial	Part
1	(Constant)			
	Party0	.532	.532	.532
2	(Constant)			
	Party0	.532	.532	.526
	GenderCC	.134	.143	.121
	RaceCC	.051	-.025	-.021
	SES0	.005	-.023	-.020
3	(Constant)			
	Party0	.532	.459	.428
	GenderCC	.134	.138	.115
	RaceCC	.051	-.033	-.027
	SES0	.005	-.041	-.034
	National0	.335	.134	.112
4	(Constant)			
	Party0	.532	.410	.368
	GenderCC	.134	.078	.064
	RaceCC	.051	.003	.002
	SES0	.005	-.051	-.041
	National0	.335	.057	.047
	MRN0	.403	.162	.134
5	(Constant)			
	Party0	.532	.397	.350
	GenderCC	.134	.072	.058
	RaceCC	.051	-.016	-.013
	SES0	.005	-.056	-.046
	National0	.335	.037	.030
	MRN0	.403	.119	.097
	MRN0xRace	.331	.094	.076
	MRN0xSES0	-.055	-.035	-.028
	MRN0xGender	-.023	.044	.036
	MRN0xParty0	-.020	.061	.050
	MRN0xNational0	-.105	-.048	-.039

a. Dependent Variable: Risk_Rules

Excluded Variables[a]

Model		Beta In	t	Sig.	Partial Correlation	Collinearity Statistics
						Tolerance
1	GenderCC	.124[b]	2.059	.041	.147	1.000
	RaceCC	-.041[b]	-.660	.510	-.047	.971
	SES0	-.010[b]	-.156	.876	-.011	.999
	National0	.126[b]	1.880	.062	.134	.808
	MRN0	.220[b]	3.374	.001	.236	.827
	MRN0xRace	.171[b]	2.690	.008	.190	.886
	MRN0xSES0	-.026[b]	-.434	.665	-.031	.997
	MRN0xGender	.045[b]	.727	.468	.052	.984
	MRN0xParty0	.009[b]	.141	.888	.010	.997
	MRN0xNational0	-.029[b]	-.464	.643	-.033	.979
2	National0	.126[c]	1.866	.064	.134	.790
	MRN0	.202[c]	2.831	.005	.201	.695
	MRN0xRace	.150[c]	2.253	.025	.161	.807
	MRN0xSES0	-.032[c]	-.529	.597	-.038	.989
	MRN0xGender	.050[c]	.805	.422	.058	.966
	MRN0xParty0	.025[c]	.406	.685	.029	.970
	MRN0xNational0	-.039[c]	-.632	.528	-.046	.970
3	MRN0	.176[d]	2.251	.026	.162	.577
	MRN0xRace	.134[d]	1.984	.049	.143	.786
	MRN0xSES0	-.028[d]	-.458	.647	-.033	.987
	MRN0xGender	.036[d]	.576	.565	.042	.950
	MRN0xParty0	.042[d]	.676	.500	.049	.951
	MRN0xNational0	-.002[d]	-.028	.978	-.002	.866
4	MRN0xRace	.085[e]	1.152	.251	.084	.647
	MRN0xSES0	-.042[e]	-.689	.492	-.050	.977
	MRN0xGender	.034[e]	.550	.583	.040	.950
	MRN0xParty0	.060[e]	.971	.333	.071	.937
	MRN0xNational0	-.010[e]	-.161	.872	-.012	.863

a. Dependent Variable: Risk_Rules

b. Predictors in the Model: (Constant), Party0

c. Predictors in the Model: (Constant), Party0, GenderCC, SES0, RaceCC

d. Predictors in the Model: (Constant), Party0, GenderCC, SES0, RaceCC, National0

e. Predictors in the Model: (Constant), Party0, GenderCC, SES0, RaceCC, National0, MRN0

```
REGRESSION
 /MISSING LISTWISE
 /STATISTICS COEFF OUTS R ANOVA CHANGE ZPP
 /CRITERIA=PIN(.05) POUT(.10)
 /NOORIGIN
 /DEPENDENT Mandate_Tot
 /METHOD=ENTER Party0
 /METHOD=ENTER GenderCC RaceCC SES0
  /METHOD=ENTER National0
 /METHOD=ENTER MRN0
 /METHOD=ENTER MRN0xRace MRN0xSES0 MRN0xGender MRN0xParty0 MRN0xNational0.
```

Regression

Notes

Output Created		15-DEC-2021 13:11:11
Comments		
Input	Data	C:\Users\njs5478\Dropbox\HM and COVID\0. Revise and Resubmit\2. R and R Data\Study 2b\Study2b_Data.sav
	Active Dataset	DataSet1
	Filter	<none>
	Weight	<none>
	Split File	<none>
	N of Rows in Working Data File	198
Missing Value Handling	Definition of Missing	User-defined missing values are treated as missing.
	Cases Used	Statistics are based on cases with no missing values for any variable used.

Notes

Syntax		REGRESSION<br>/MISSING LISTWISE<br>/STATISTICS COEFF OUTS R ANOVA CHANGE ZPP<br>/CRITERIA=PIN(.05) POUT(.10)<br>/NOORIGIN<br>/DEPENDENT Mandate_Tot<br>/METHOD=ENTER Party0<br>/METHOD=ENTER GenderCC RaceCC SES0<br>/METHOD=ENTER National0<br>/METHOD=ENTER MRN0<br>/METHOD=ENTER MRN0xRace MRN0xSES0 MRN0xGender MRN0xParty0 MRN0xNational0.
Resources	Processor Time	00:00:00.02
	Elapsed Time	00:00:00.02
	Memory Required	34768 bytes
	Additional Memory Required for Residual Plots	0 bytes

Variables Entered/Removed[a]

Model	Variables Entered	Variables Removed	Method
1	Party0[b]	.	Enter
2	GenderCC, SES0, RaceCC[b]	.	Enter
3	National0[b]	.	Enter
4	MRN0[b]	.	Enter
5	MRN0xSES0, MRN0xGender, MRN0xParty0, MRN0xNational0, ...[b]	.	Enter

a. Dependent Variable: Mandate_Tot

b. All requested variables entered.

Model Summary

Model	R	R Square	Adjusted R Square	Std. Error of the Estimate	Change Statistics		
					R Square Change	F Change	df1
1	.725[a]	.526	.523	1.54278	.526	215.194	1
2	.727[b]	.528	.518	1.55144	.002	.281	3
3	.732[c]	.537	.524	1.54134	.009	3.510	1
4	.734[d]	.539	.525	1.54077	.003	1.140	1
5	.739[e]	.547	.520	1.54898	.007	.601	5

Model Summary

Model	Change Statistics	
	df2	Sig. F Change
1	194	.000
2	191	.839
3	190	.063
4	189	.287
5	184	.699

a. Predictors: (Constant), Party0

b. Predictors: (Constant), Party0, GenderCC, SES0, RaceCC

c. Predictors: (Constant), Party0, GenderCC, SES0, RaceCC, National0

d. Predictors: (Constant), Party0, GenderCC, SES0, RaceCC, National0, MRN0

e. Predictors: (Constant), Party0, GenderCC, SES0, RaceCC, National0, MRN0, MRN0xSES0, MRN0xGender, MRN0xParty0, MRN0xNational0, MRN0xRace

ANOVA[a]

Model		Sum of Squares	df	Mean Square	F	Sig.
1	Regression	512.201	1	512.201	215.194	.000[b]
	Residual	461.754	194	2.380		
	Total	973.955	195			
2	Regression	514.227	4	128.557	53.411	.000[c]
	Residual	459.728	191	2.407		
	Total	973.955	195			
3	Regression	522.566	5	104.513	43.992	.000[d]
	Residual	451.389	190	2.376		
	Total	973.955	195			
4	Regression	525.272	6	87.545	36.877	.000[e]
	Residual	448.683	189	2.374		
	Total	973.955	195			
5	Regression	532.479	11	48.407	20.175	.000[f]
	Residual	441.476	184	2.399		
	Total	973.955	195			

a. Dependent Variable: Mandate_Tot

b. Predictors: (Constant), Party0

c. Predictors: (Constant), Party0, GenderCC, SES0, RaceCC

d. Predictors: (Constant), Party0, GenderCC, SES0, RaceCC, National0

e. Predictors: (Constant), Party0, GenderCC, SES0, RaceCC, National0, MRN0

f. Predictors: (Constant), Party0, GenderCC, SES0, RaceCC, National0, MRN0, MRN0xSES0, MRN0xGender, MRN0xParty0, MRN0xNational0, MRN0xRace

Coefficients[a]

Model		Unstandardized Coefficients B	Unstandardized Coefficients Std. Error	Standardized Coefficients Beta	t	Sig.
1	(Constant)	4.517	.110		40.989	.000
	Party0	-1.035	.071	-.725	-14.670	.000
2	(Constant)	4.492	.140		32.085	.000
	Party0	-1.040	.072	-.729	-14.433	.000
	GenderCC	.021	.113	.010	.190	.849
	RaceCC	.042	.143	.015	.293	.770
	SES0	.108	.130	.042	.832	.407
3	(Constant)	4.485	.139		32.230	.000
	Party0	-.977	.079	-.685	-12.342	.000
	GenderCC	.031	.112	.014	.281	.779
	RaceCC	.056	.142	.020	.397	.692
	SES0	.140	.131	.054	1.071	.285
	National0	-.182	.097	-.104	-1.874	.063
4	(Constant)	4.503	.140		32.126	.000
	Party0	-.950	.083	-.666	-11.455	.000
	GenderCC	.074	.119	.033	.622	.534
	RaceCC	.023	.145	.008	.160	.873
	SES0	.148	.131	.057	1.131	.260
	National0	-.135	.107	-.077	-1.266	.207
	MRN0	-.150	.140	-.069	-1.068	.287
5	(Constant)	4.393	.159		27.648	.000
	Party0	-.940	.086	-.659	-10.979	.000
	GenderCC	.085	.121	.038	.698	.486
	RaceCC	.010	.150	.004	.067	.947
	SES0	.157	.132	.060	1.191	.235
	National0	-.097	.115	-.055	-.844	.400
	MRN0	-.091	.158	-.042	-.576	.565
	MRN0xRace	-.109	.139	-.050	-.782	.435
	MRN0xSES0	.086	.129	.035	.663	.508
	MRN0xGender	-.002	.120	-.001	-.015	.988
	MRN0xParty0	.086	.079	.061	1.089	.278
	MRN0xNational0	.061	.099	.036	.611	.542

Coefficients[a]

Model		Correlations Zero-order	Partial	Part
1	(Constant)			
	Party0	-.725	-.725	-.725
2	(Constant)			
	Party0	-.725	-.722	-.718
	GenderCC	-.003	.014	.009
	RaceCC	-.110	.021	.015
	SES0	.023	.060	.041
3	(Constant)			
	Party0	-.725	-.667	-.610
	GenderCC	-.003	.020	.014
	RaceCC	-.110	.029	.020
	SES0	.023	.077	.053
	National0	-.394	-.135	-.093
4	(Constant)			
	Party0	-.725	-.640	-.566
	GenderCC	-.003	.045	.031
	RaceCC	-.110	.012	.008
	SES0	.023	.082	.056
	National0	-.394	-.092	-.062
	MRN0	-.369	-.077	-.053
5	(Constant)			
	Party0	-.725	-.629	-.545
	GenderCC	-.003	.051	.035
	RaceCC	-.110	.005	.003
	SES0	.023	.087	.059
	National0	-.394	-.062	-.042
	MRN0	-.369	-.042	-.029
	MRN0xRace	-.293	-.058	-.039
	MRN0xSES0	.072	.049	.033
	MRN0xGender	.102	-.001	-.001
	MRN0xParty0	.117	.080	.054
	MRN0xNational0	.176	.045	.030

a. Dependent Variable: Mandate_Tot

Excluded Variables[a]

Model		Beta In	t	Sig.	Partial Correlation	Collinearity Statistics Tolerance
1	GenderCC	.011[b]	.221	.825	.016	1.000
	RaceCC	.014[b]	.283	.777	.020	.971
	SES0	.043[b]	.861	.390	.062	.999
	National0	-.094[b]	-1.722	.087	-.123	.808
	MRN0	-.081[b]	-1.495	.137	-.107	.827
	MRN0xRace	-.054[b]	-1.023	.308	-.073	.886
	MRN0xSES0	.032[b]	.651	.516	.047	.997
	MRN0xGender	.011[b]	.215	.830	.015	.984
	MRN0xParty0	.077[b]	1.568	.118	.112	.997
	MRN0xNational0	.073[b]	1.459	.146	.104	.979
2	National0	-.104[c]	-1.874	.063	-.135	.790
	MRN0	-.103[c]	-1.744	.083	-.125	.695
	MRN0xRace	-.068[c]	-1.224	.222	-.088	.807
	MRN0xSES0	.030[c]	.601	.548	.044	.989
	MRN0xGender	.006[c]	.126	.900	.009	.966
	MRN0xParty0	.081[c]	1.614	.108	.116	.970
	MRN0xNational0	.074[c]	1.478	.141	.107	.970
3	MRN0	-.069[d]	-1.068	.287	-.077	.577
	MRN0xRace	-.052[d]	-.941	.348	-.068	.786
	MRN0xSES0	.026[d]	.531	.596	.039	.987
	MRN0xGender	.019[d]	.368	.713	.027	.950
	MRN0xParty0	.069[d]	1.372	.172	.099	.951
	MRN0xNational0	.049[d]	.920	.359	.067	.866
4	MRN0xRace	-.033[e]	-.542	.588	-.039	.647
	MRN0xSES0	.032[e]	.639	.523	.047	.977
	MRN0xGender	.019[e]	.383	.702	.028	.950
	MRN0xParty0	.064[e]	1.249	.213	.091	.937
	MRN0xNational0	.052[e]	.986	.326	.072	.863

a. Dependent Variable: Mandate_Tot

b. Predictors in the Model: (Constant), Party0

c. Predictors in the Model: (Constant), Party0, GenderCC, SES0, RaceCC

d. Predictors in the Model: (Constant), Party0, GenderCC, SES0, RaceCC, National0

e. Predictors in the Model: (Constant), Party0, GenderCC, SES0, RaceCC, National0, MRN0

```
REGRESSION
 /MISSING LISTWISE
 /STATISTICS COEFF OUTS R ANOVA CHANGE ZPP
 /CRITERIA=PIN(.05) POUT(.10)
 /NOORIGIN
 /DEPENDENT Conspiracy_Tot
 /METHOD=ENTER Party0
 /METHOD=ENTER GenderCC RaceCC SES0
  /METHOD=ENTER National0
 /METHOD=ENTER MRN0
 /METHOD=ENTER MRN0xRace MRN0xSES0 MRN0xGender MRN0xParty0 MRN0xNational0.
```

Regression

Notes

Output Created		15-DEC-2021 13:11:11
Comments		
Input	Data	C: \Users\njs5478\Dropbox\HM and COVID\0. Revise and Resubmit\2. R and R Data\Study 2b\Study2b_Data.sav
	Active Dataset	DataSet1
	Filter	<none>
	Weight	<none>
	Split File	<none>
	N of Rows in Working Data File	198
Missing Value Handling	Definition of Missing	User-defined missing values are treated as missing.
	Cases Used	Statistics are based on cases with no missing values for any variable used.

Notes

Syntax		REGRESSION<br>/MISSING LISTWISE<br>/STATISTICS COEFF OUTS R ANOVA CHANGE ZPP<br>/CRITERIA=PIN(.05) POUT(.10)<br>/NOORIGIN<br>/DEPENDENT Conspiracy_Tot<br>/METHOD=ENTER Party0<br>/METHOD=ENTER GenderCC RaceCC SES0<br>/METHOD=ENTER National0<br>/METHOD=ENTER MRN0<br>/METHOD=ENTER MRN0xRace MRN0xSES0 MRN0xGender MRN0xParty0 MRN0xNational0.
Resources	Processor Time	00:00:00.02
	Elapsed Time	00:00:00.02
	Memory Required	34768 bytes
	Additional Memory Required for Residual Plots	0 bytes

Variables Entered/Removed[a]

Model	Variables Entered	Variables Removed	Method
1	Party0[b]	.	Enter
2	GenderCC, SES0, RaceCC[b]	.	Enter
3	National0[b]	.	Enter
4	MRN0[b]	.	Enter
5	MRN0xSES0, MRN0xGender, MRN0xParty0, MRN0xNational0, ...[b]	.	Enter

a. Dependent Variable: Conspiracy_Tot

b. All requested variables entered.

Model Summary

Model	R	R Square	Adjusted R Square	Std. Error of the Estimate	Change Statistics		
					R Square Change	F Change	df1
1	.465[a]	.216	.212	.57279	.216	53.373	1
2	.466[b]	.218	.201	.57661	.002	.146	3
3	.508[c]	.258	.238	.56314	.040	10.251	1
4	.586[d]	.343	.323	.53100	.086	24.693	1
5	.596[e]	.355	.317	.53325	.012	.682	5

Model Summary

Model	Change Statistics	
	df2	Sig. F Change
1	194	.000
2	191	.932
3	190	.002
4	189	.000
5	184	.638

a. Predictors: (Constant), Party0

b. Predictors: (Constant), Party0, GenderCC, SES0, RaceCC

c. Predictors: (Constant), Party0, GenderCC, SES0, RaceCC, National0

d. Predictors: (Constant), Party0, GenderCC, SES0, RaceCC, National0, MRN0

e. Predictors: (Constant), Party0, GenderCC, SES0, RaceCC, National0, MRN0, MRN0xSES0, MRN0xGender, MRN0xParty0, MRN0xNational0, MRN0xRace

ANOVA[a]

Model		Sum of Squares	df	Mean Square	F	Sig.
1	Regression	17.511	1	17.511	53.373	.000[b]
	Residual	63.650	194	.328		
	Total	81.162	195			
2	Regression	17.657	4	4.414	13.277	.000[c]
	Residual	63.504	191	.332		
	Total	81.162	195			
3	Regression	20.908	5	4.182	13.186	.000[d]
	Residual	60.253	190	.317		
	Total	81.162	195			
4	Regression	27.871	6	4.645	16.474	.000[e]
	Residual	53.291	189	.282		
	Total	81.162	195			
5	Regression	28.841	11	2.622	9.220	.000[f]
	Residual	52.321	184	.284		
	Total	81.162	195			

a. Dependent Variable: Conspiracy_Tot

b. Predictors: (Constant), Party0

c. Predictors: (Constant), Party0, GenderCC, SES0, RaceCC

d. Predictors: (Constant), Party0, GenderCC, SES0, RaceCC, National0

e. Predictors: (Constant), Party0, GenderCC, SES0, RaceCC, National0, MRN0

f. Predictors: (Constant), Party0, GenderCC, SES0, RaceCC, National0, MRN0, MRN0xSES0, MRN0xGender, MRN0xParty0, MRN0xNational0, MRN0xRace

Coefficients[a]

Model		Unstandardized Coefficients B	Std. Error	Standardized Coefficients Beta	t	Sig.
1	(Constant)	1.703	.041		41.629	.000
	Party0	.191	.026	.465	7.306	.000
2	(Constant)	1.707	.052		32.802	.000
	Party0	.192	.027	.466	7.163	.000
	GenderCC	.021	.042	.033	.506	.614
	RaceCC	-.006	.053	-.007	-.107	.915
	SES0	-.021	.048	-.027	-.426	.671
3	(Constant)	1.711	.051		33.664	.000
	Party0	.152	.029	.370	5.269	.000
	GenderCC	.015	.041	.023	.364	.716
	RaceCC	-.015	.052	-.018	-.284	.777
	SES0	-.040	.048	-.054	-.845	.399
	National0	.114	.035	.225	3.202	.002
4	(Constant)	1.682	.048		34.809	.000
	Party0	.110	.029	.266	3.836	.000
	GenderCC	-.053	.041	-.083	-1.301	.195
	RaceCC	.038	.050	.047	.765	.445
	SES0	-.053	.045	-.070	-1.178	.240
	National0	.038	.037	.076	1.040	.300
	MRN0	.240	.048	.386	4.969	.000
5	(Constant)	1.655	.055		30.253	.000
	Party0	.108	.029	.262	3.666	.000
	GenderCC	-.053	.042	-.083	-1.283	.201
	RaceCC	.048	.051	.060	.935	.351
	SES0	-.048	.045	-.064	-1.066	.288
	National0	.057	.039	.113	1.446	.150
	MRN0	.257	.054	.412	4.716	.000
	MRN0xRace	-.035	.048	-.056	-.733	.465
	MRN0xSES0	-.050	.045	-.070	-1.122	.263
	MRN0xGender	-.022	.041	-.034	-.540	.590
	MRN0xParty0	-.008	.027	-.018	-.275	.783
	MRN0xNational0	.053	.034	.109	1.546	.124

Coefficients[a]

Model		Correlations		
		Zero-order	Partial	Part
1	(Constant)			
	Party0	.465	.465	.465
2	(Constant)			
	Party0	.465	.460	.458
	GenderCC	.040	.037	.032
	RaceCC	.067	-.008	-.007
	SES0	-.012	-.031	-.027
3	(Constant)			
	Party0	.465	.357	.329
	GenderCC	.040	.026	.023
	RaceCC	.067	-.021	-.018
	SES0	-.012	-.061	-.053
	National0	.379	.226	.200
4	(Constant)			
	Party0	.465	.269	.226
	GenderCC	.040	-.094	-.077
	RaceCC	.067	.056	.045
	SES0	-.012	-.085	-.069
	National0	.379	.075	.061
	MRN0	.493	.340	.293
5	(Constant)			
	Party0	.465	.261	.217
	GenderCC	.040	-.094	-.076
	RaceCC	.067	.069	.055
	SES0	-.012	-.078	-.063
	National0	.379	.106	.086
	MRN0	.493	.328	.279
	MRN0xRace	.284	-.054	-.043
	MRN0xSES0	-.053	-.082	-.066
	MRN0xGender	-.052	-.040	-.032
	MRN0xParty0	-.079	-.020	-.016
	MRN0xNational0	-.047	.113	.091

a. Dependent Variable: Conspiracy_Tot

Excluded Variables[a]

Model		Beta In	t	Sig.	Partial Correlation	Collinearity Statistics Tolerance
1	GenderCC	.032[b]	.495	.621	.036	1.000
	RaceCC	-.013[b]	-.194	.846	-.014	.971
	SES0	-.025[b]	-.387	.699	-.028	.999
	National0	.218[b]	3.145	.002	.221	.808
	MRN0	.363[b]	5.580	.000	.373	.827
	MRN0xRace	.143[b]	2.136	.034	.152	.886
	MRN0xSES0	-.028[b]	-.436	.663	-.031	.997
	MRN0xGender	.006[b]	.099	.921	.007	.984
	MRN0xParty0	-.054[b]	-.851	.396	-.061	.997
	MRN0xNational0	.020[b]	.318	.751	.023	.979
2	National0	.225[c]	3.202	.002	.226	.790
	MRN0	.419[c]	5.927	.000	.395	.695
	MRN0xRace	.150[c]	2.122	.035	.152	.807
	MRN0xSES0	-.029[c]	-.449	.654	-.033	.989
	MRN0xGender	.009[c]	.142	.887	.010	.966
	MRN0xParty0	-.053[c]	-.807	.420	-.058	.970
	MRN0xNational0	.017[c]	.260	.795	.019	.970
3	MRN0	.386[d]	4.969	.000	.340	.577
	MRN0xRace	.117[d]	1.666	.097	.120	.786
	MRN0xSES0	-.021[d]	-.332	.741	-.024	.987
	MRN0xGender	-.017[d]	-.264	.792	-.019	.950
	MRN0xParty0	-.025[d]	-.382	.703	-.028	.951
	MRN0xNational0	.093[d]	1.394	.165	.101	.866
4	MRN0xRace	-.027[e]	-.362	.718	-.026	.647
	MRN0xSES0	-.051[e]	-.847	.398	-.062	.977
	MRN0xGender	-.021[e]	-.351	.726	-.026	.950
	MRN0xParty0	.013[e]	.212	.832	.015	.937
	MRN0xNational0	.075[e]	1.185	.237	.086	.863

a. Dependent Variable: Conspiracy_Tot

b. Predictors in the Model: (Constant), Party0

c. Predictors in the Model: (Constant), Party0, GenderCC, SES0, RaceCC

d. Predictors in the Model: (Constant), Party0, GenderCC, SES0, RaceCC, National0

e. Predictors in the Model: (Constant), Party0, GenderCC, SES0, RaceCC, National0, MRN0

```
 **Including PIdeology

REGRESSION
  /MISSING LISTWISE
  /STATISTICS COEFF OUTS R ANOVA CHANGE ZPP
  /CRITERIA=PIN(.05) POUT(.10)
  /NOORIGIN
  /DEPENDENT Concern_Tot
  /METHOD=ENTER Ideology0
  /METHOD=ENTER GenderCC RaceCC SES0
   /METHOD=ENTER National0
  /METHOD=ENTER MRN0
  /METHOD=ENTER MRN0xRace MRN0xSES0 MRN0xGender MRN0xIdeology0 MRN0xNational0.
```

Regression

Notes

Output Created		15-DEC-2021 13:11:11
Comments		
Input	Data	C:<br>\Users\njs5478\Dropbox\HM and COVID\0. Revise and Resubmit\2. R and R Data\Study 2b\Study2b_Data.sav
	Active Dataset	DataSet1
	Filter	<none>
	Weight	<none>
	Split File	<none>
	N of Rows in Working Data File	198
Missing Value Handling	Definition of Missing	User-defined missing values are treated as missing.
	Cases Used	Statistics are based on cases with no missing values for any variable used.

Notes

Syntax		REGRESSION<br>/MISSING LISTWISE<br>/STATISTICS COEFF OUTS R ANOVA CHANGE ZPP<br>/CRITERIA=PIN(.05) POUT(.10)<br>/NOORIGIN<br>/DEPENDENT Concern_Tot<br>/METHOD=ENTER Ideology0<br>/METHOD=ENTER GenderCC RaceCC SES0<br>/METHOD=ENTER National0<br>/METHOD=ENTER MRN0<br>/METHOD=ENTER MRN0xRace MRN0xSES0 MRN0xGender MRN0xIdeology0 MRN0xNational0.
Resources	Processor Time	00:00:00.03
	Elapsed Time	00:00:00.02
	Memory Required	34768 bytes
	Additional Memory Required for Residual Plots	0 bytes

Variables Entered/Removed[a]

Model	Variables Entered	Variables Removed	Method
1	Ideology0[b]	.	Enter
2	SES0, GenderCC, RaceCC[b]	.	Enter
3	National0[b]	.	Enter
4	MRN0[b]	.	Enter
5	MRN0xSES0, MRN0xGender, MRN0xIdeology0, MRN0xNational0, MRN0xRace[b]	.	Enter

a. Dependent Variable: Concern_Tot

b. All requested variables entered.

Model Summary

					Change Statistics		
Model	R	R Square	Adjusted R Square	Std. Error of the Estimate	R Square Change	F Change	df1
1	.496[a]	.246	.242	1.41975	.246	63.180	1
2	.530[b]	.281	.266	1.39724	.035	3.100	3
3	.542[c]	.293	.275	1.38850	.013	3.413	1
4	.542[d]	.293	.271	1.39211	.000	.014	1
5	.565[e]	.319	.278	1.38531	.025	1.372	5

Model Summary

	Change Statistics	
Model	df2	Sig. F Change
1	194	.000
2	191	.028
3	190	.066
4	189	.905
5	184	.237

a. Predictors: (Constant), Ideology0

b. Predictors: (Constant), Ideology0, SES0, GenderCC, RaceCC

c. Predictors: (Constant), Ideology0, SES0, GenderCC, RaceCC, National0

d. Predictors: (Constant), Ideology0, SES0, GenderCC, RaceCC, National0, MRN0

e. Predictors: (Constant), Ideology0, SES0, GenderCC, RaceCC, National0, MRN0, MRN0xSES0, MRN0xGender, MRN0xIdeology0, MRN0xNational0, MRN0xRace

ANOVA[a]

Model		Sum of Squares	df	Mean Square	F	Sig.
1	Regression	127.351	1	127.351	63.180	.000[b]
	Residual	391.044	194	2.016		
	Total	518.396	195			
2	Regression	145.510	4	36.377	18.633	.000[c]
	Residual	372.886	191	1.952		
	Total	518.396	195			
3	Regression	152.089	5	30.418	15.778	.000[d]
	Residual	366.306	190	1.928		
	Total	518.396	195			
4	Regression	152.117	6	25.353	13.082	.000[e]
	Residual	366.279	189	1.938		
	Total	518.396	195			
5	Regression	165.283	11	15.026	7.830	.000[f]
	Residual	353.112	184	1.919		
	Total	518.396	195			

a. Dependent Variable: Concern_Tot

b. Predictors: (Constant), Ideology0

c. Predictors: (Constant), Ideology0, SES0, GenderCC, RaceCC

d. Predictors: (Constant), Ideology0, SES0, GenderCC, RaceCC, National0

e. Predictors: (Constant), Ideology0, SES0, GenderCC, RaceCC, National0, MRN0

f. Predictors: (Constant), Ideology0, SES0, GenderCC, RaceCC, National0, MRN0, MRN0xSES0, MRN0xGender, MRN0xIdeology0, MRN0xNational0, MRN0xRace

Coefficients[a]

Model		Unstandardized Coefficients B	Std. Error	Standardized Coefficients Beta	t	Sig.
1	(Constant)	4.299	.101		42.392	.000
	Ideology0	-.398	.050	-.496	-7.949	.000
2	(Constant)	4.283	.126		34.034	.000
	Ideology0	-.389	.050	-.485	-7.799	.000
	GenderCC	-.296	.102	-.182	-2.915	.004
	RaceCC	.022	.128	.011	.169	.866
	SES0	-.045	.118	-.024	-.382	.703
3	(Constant)	4.290	.125		34.288	.000
	Ideology0	-.448	.059	-.558	-7.605	.000
	GenderCC	-.301	.101	-.185	-2.976	.003
	RaceCC	.009	.127	.004	.070	.944
	SES0	-.072	.118	-.038	-.610	.543
	National0	.174	.094	.136	1.847	.066
4	(Constant)	4.292	.126		33.931	.000
	Ideology0	-.445	.065	-.554	-6.823	.000
	GenderCC	-.296	.107	-.182	-2.757	.006
	RaceCC	.005	.131	.003	.041	.967
	SES0	-.071	.118	-.037	-.600	.549
	National0	.177	.099	.139	1.790	.075
	MRN0	-.016	.133	-.010	-.119	.905
5	(Constant)	4.170	.146		28.621	.000
	Ideology0	-.452	.067	-.564	-6.746	.000
	GenderCC	-.263	.109	-.162	-2.418	.017
	RaceCC	.028	.134	.014	.211	.833
	SES0	-.034	.119	-.018	-.287	.774
	National0	.220	.106	.173	2.084	.039
	MRN0	.077	.147	.049	.527	.599
	MRN0xRace	-.167	.124	-.106	-1.345	.180
	MRN0xSES0	.062	.118	.034	.520	.603
	MRN0xGender	-.149	.107	-.089	-1.383	.168
	MRN0xIdeology0	.119	.057	.147	2.094	.038
	MRN0xNational0	-.004	.091	-.003	-.044	.965

Coefficients[a]

Model		Correlations Zero-order	Partial	Part
1	(Constant)			
	Ideology0	-.496	-.496	-.496
2	(Constant)			
	Ideology0	-.496	-.491	-.479
	GenderCC	-.215	-.206	-.179
	RaceCC	-.030	.012	.010
	SES0	-.062	-.028	-.023
3	(Constant)			
	Ideology0	-.496	-.483	-.464
	GenderCC	-.215	-.211	-.181
	RaceCC	-.030	.005	.004
	SES0	-.062	-.044	-.037
	National0	-.185	.133	.113
4	(Constant)			
	Ideology0	-.496	-.445	-.417
	GenderCC	-.215	-.197	-.169
	RaceCC	-.030	.003	.003
	SES0	-.062	-.044	-.037
	National0	-.185	.129	.109
	MRN0	-.312	-.009	-.007
5	(Constant)			
	Ideology0	-.496	-.445	-.410
	GenderCC	-.215	-.176	-.147
	RaceCC	-.030	.016	.013
	SES0	-.062	-.021	-.017
	National0	-.185	.152	.127
	MRN0	-.312	.039	.032
	MRN0xRace	-.277	-.099	-.082
	MRN0xSES0	.046	.038	.032
	MRN0xGender	.021	-.101	-.084
	MRN0xIdeology0	.154	.153	.127
	MRN0xNational0	.055	-.003	-.003

a. Dependent Variable: Concern_Tot

Excluded Variables[a]

Model		Beta In	t	Sig.	Partial Correlation	Collinearity Statistics
						Tolerance
1	GenderCC	-.186[b]	-3.036	.003	-.213	.996
	RaceCC	.039[b]	.616	.539	.044	.981
	SES0	-.039[b]	-.619	.537	-.044	.998
	National0	.125[b]	1.685	.094	.120	.698
	MRN0	-.054[b]	-.715	.475	-.051	.692
	MRN0xRace	-.101[b]	-1.494	.137	-.107	.851
	MRN0xSES0	-.005[b]	-.087	.931	-.006	.989
	MRN0xGender	-.035[b]	-.565	.573	-.041	.987
	MRN0xIdeology0	.115[b]	1.843	.067	.132	.994
	MRN0xNational0	-.025[b]	-.401	.689	-.029	.974
2	National0	.136[c]	1.847	.066	.133	.684
	MRN0	.037[c]	.452	.652	.033	.575
	MRN0xRace	-.049[c]	-.707	.480	-.051	.781
	MRN0xSES0	.006[c]	.103	.918	.007	.979
	MRN0xGender	-.035[c]	-.564	.573	-.041	.970
	MRN0xIdeology0	.096[c]	1.544	.124	.111	.970
	MRN0xNational0	-.011[c]	-.180	.857	-.013	.964
3	MRN0	-.010[d]	-.119	.905	-.009	.520
	MRN0xRace	-.063[d]	-.901	.369	-.065	.773
	MRN0xSES0	.006[d]	.105	.916	.008	.979
	MRN0xGender	-.053[d]	-.848	.398	-.062	.950
	MRN0xIdeology0	.114[d]	1.840	.067	.133	.951
	MRN0xNational0	.028[d]	.433	.666	.031	.865
4	MRN0xRace	-.070[e]	-.928	.355	-.068	.651
	MRN0xSES0	.008[e]	.122	.903	.009	.961
	MRN0xGender	-.053[e]	-.841	.401	-.061	.948
	MRN0xIdeology0	.114[e]	1.831	.069	.132	.949
	MRN0xNational0	.029[e]	.438	.662	.032	.863

a. Dependent Variable: Concern_Tot

b. Predictors in the Model: (Constant), Ideology0

c. Predictors in the Model: (Constant), Ideology0, SES0, GenderCC, RaceCC

d. Predictors in the Model: (Constant), Ideology0, SES0, GenderCC, RaceCC, National0

e. Predictors in the Model: (Constant), Ideology0, SES0, GenderCC, RaceCC, National0, MRN0

```
REGRESSION
 /MISSING LISTWISE
 /STATISTICS COEFF OUTS R ANOVA CHANGE ZPP
 /CRITERIA=PIN(.05) POUT(.10)
 /NOORIGIN
 /DEPENDENT Finance_Tot
 /METHOD=ENTER Ideology0
 /METHOD=ENTER GenderCC RaceCC SES0
  /METHOD=ENTER National0
 /METHOD=ENTER MRN0
 /METHOD=ENTER MRN0xRace MRN0xSES0 MRN0xGender MRN0xIdeology0 MRN0xNational0.
```

Regression

Notes

Output Created		15-DEC-2021 13:11:11
Comments		
Input	Data	C: \Users\njs5478\Dropbox\HM and COVID\0. Revise and Resubmit\2. R and R Data\Study 2b\Study2b_Data.sav
	Active Dataset	DataSet1
	Filter	<none>
	Weight	<none>
	Split File	<none>
	N of Rows in Working Data File	198
Missing Value Handling	Definition of Missing	User-defined missing values are treated as missing.
	Cases Used	Statistics are based on cases with no missing values for any variable used.

Notes

Syntax		REGRESSION<br>/MISSING LISTWISE<br>/STATISTICS COEFF OUTS R ANOVA CHANGE ZPP<br>/CRITERIA=PIN(.05) POUT(.10)<br>/NOORIGIN<br>/DEPENDENT Finance_Tot<br>/METHOD=ENTER Ideology0<br>/METHOD=ENTER GenderCC RaceCC SES0<br>/METHOD=ENTER National0<br>/METHOD=ENTER MRN0<br>/METHOD=ENTER MRN0xRace MRN0xSES0 MRN0xGender MRN0xIdeology0 MRN0xNational0.
Resources	Processor Time	00:00:00.05
	Elapsed Time	00:00:00.03
	Memory Required	34768 bytes
	Additional Memory Required for Residual Plots	0 bytes

Variables Entered/Removed[a]

Model	Variables Entered	Variables Removed	Method
1	Ideology0[b]	.	Enter
2	SES0, GenderCC, RaceCC[b]	.	Enter
3	National0[b]	.	Enter
4	MRN0[b]	.	Enter
5	MRN0xSES0, MRN0xGender, MRN0xIdeology0, MRN0xNational0, MRN0xRace[b]	.	Enter

a. Dependent Variable: Finance_Tot

b. All requested variables entered.

Model Summary

					Change Statistics		
Model	R	R Square	Adjusted R Square	Std. Error of the Estimate	R Square Change	F Change	df1
1	.038[a]	.001	-.004	1.99058	.001	.286	1
2	.340[b]	.116	.097	1.88772	.114	8.239	3
3	.341[c]	.116	.093	1.89261	.000	.014	1
4	.359[d]	.129	.101	1.88361	.013	2.821	1
5	.363[e]	.132	.080	1.90616	.003	.111	5

Model Summary

	Change Statistics	
Model	df2	Sig. F Change
1	194	.593
2	191	.000
3	190	.906
4	189	.095
5	184	.990

a. Predictors: (Constant), Ideology0

b. Predictors: (Constant), Ideology0, SES0, GenderCC, RaceCC

c. Predictors: (Constant), Ideology0, SES0, GenderCC, RaceCC, National0

d. Predictors: (Constant), Ideology0, SES0, GenderCC, RaceCC, National0, MRN0

e. Predictors: (Constant), Ideology0, SES0, GenderCC, RaceCC, National0, MRN0, MRN0xSES0, MRN0xGender, MRN0xIdeology0, MRN0xNational0, MRN0xRace

ANOVA[a]

Model		Sum of Squares	df	Mean Square	F	Sig.
1	Regression	1.133	1	1.133	.286	.593[b]
	Residual	768.704	194	3.962		
	Total	769.837	195			
2	Regression	89.211	4	22.303	6.259	.000[c]
	Residual	680.626	191	3.563		
	Total	769.837	195			
3	Regression	89.261	5	17.852	4.984	.000[d]
	Residual	680.576	190	3.582		
	Total	769.837	195			
4	Regression	99.268	6	16.545	4.663	.000[e]
	Residual	670.568	189	3.548		
	Total	769.837	195			
5	Regression	101.280	11	9.207	2.534	.005[f]
	Residual	668.556	184	3.633		
	Total	769.837	195			

a. Dependent Variable: Finance_Tot

b. Predictors: (Constant), Ideology0

c. Predictors: (Constant), Ideology0, SES0, GenderCC, RaceCC

d. Predictors: (Constant), Ideology0, SES0, GenderCC, RaceCC, National0

e. Predictors: (Constant), Ideology0, SES0, GenderCC, RaceCC, National0, MRN0

f. Predictors: (Constant), Ideology0, SES0, GenderCC, RaceCC, National0, MRN0, MRN0xSES0, MRN0xGender, MRN0xIdeology0, MRN0xNational0, MRN0xRace

Coefficients[a]

Model		Unstandardized Coefficients B	Std. Error	Standardized Coefficients Beta	t	Sig.
1	(Constant)	3.797	.142		26.701	.000
	Ideology0	-.038	.070	-.038	-.535	.593
2	(Constant)	3.731	.170		21.942	.000
	Ideology0	-.020	.067	-.020	-.293	.770
	GenderCC	-.375	.137	-.189	-2.735	.007
	RaceCC	.102	.173	.041	.593	.554
	SES0	-.601	.159	-.259	-3.781	.000
3	(Constant)	3.730	.171		21.872	.000
	Ideology0	-.015	.080	-.015	-.182	.856
	GenderCC	-.375	.138	-.189	-2.725	.007
	RaceCC	.103	.173	.042	.597	.551
	SES0	-.598	.160	-.258	-3.728	.000
	National0	-.015	.128	-.010	-.119	.906
4	(Constant)	3.693	.171		21.579	.000
	Ideology0	-.077	.088	-.079	-.876	.382
	GenderCC	-.457	.145	-.231	-3.142	.002
	RaceCC	.170	.177	.068	.960	.338
	SES0	-.615	.160	-.265	-3.845	.000
	National0	-.085	.134	-.054	-.631	.529
	MRN0	.303	.181	.158	1.679	.095
5	(Constant)	3.760	.200		18.754	.000
	Ideology0	-.079	.092	-.080	-.852	.395
	GenderCC	-.454	.150	-.229	-3.037	.003
	RaceCC	.179	.184	.072	.973	.332
	SES0	-.621	.163	-.267	-3.804	.000
	National0	-.114	.145	-.073	-.784	.434
	MRN0	.299	.202	.156	1.480	.140
	MRN0xRace	.015	.171	.008	.090	.928
	MRN0xSES0	-.001	.163	.000	-.004	.997
	MRN0xGender	-.014	.148	-.007	-.093	.926
	MRN0xIdeology0	-.020	.078	-.020	-.253	.801
	MRN0xNational0	-.064	.125	-.043	-.512	.609

Coefficients[a]

Model		Correlations Zero-order	Partial	Part
1	(Constant)			
	Ideology0	-.038	-.038	-.038
2	(Constant)			
	Ideology0	-.038	-.021	-.020
	GenderCC	-.219	-.194	-.186
	RaceCC	.060	.043	.040
	SES0	-.275	-.264	-.257
3	(Constant)			
	Ideology0	-.038	-.013	-.012
	GenderCC	-.219	-.194	-.186
	RaceCC	.060	.043	.041
	SES0	-.275	-.261	-.254
	National0	-.057	-.009	-.008
4	(Constant)			
	Ideology0	-.038	-.064	-.059
	GenderCC	-.219	-.223	-.213
	RaceCC	.060	.070	.065
	SES0	-.275	-.269	-.261
	National0	-.057	-.046	-.043
	MRN0	-.030	.121	.114
5	(Constant)			
	Ideology0	-.038	-.063	-.059
	GenderCC	-.219	-.218	-.209
	RaceCC	.060	.072	.067
	SES0	-.275	-.270	-.261
	National0	-.057	-.058	-.054
	MRN0	-.030	.108	.102
	MRN0xRace	-.050	.007	.006
	MRN0xSES0	-.001	.000	.000
	MRN0xGender	-.020	-.007	-.006
	MRN0xIdeology0	.014	-.019	-.017
	MRN0xNational0	-.033	-.038	-.035

a. Dependent Variable: Finance_Tot

Excluded Variables[a]

Model		Beta In	t	Sig.	Partial Correlation	Collinearity Statistics
						Tolerance
1	GenderCC	-.218[b]	-3.093	.002	-.217	.996
	RaceCC	.067[b]	.924	.357	.066	.981
	SES0	-.274[b]	-3.953	.000	-.274	.998
	National0	-.052[b]	-.603	.547	-.043	.698
	MRN0	-.012[b]	-.140	.889	-.010	.692
	MRN0xRace	-.041[b]	-.532	.595	-.038	.851
	MRN0xSES0	-.005[b]	-.074	.941	-.005	.989
	MRN0xGender	-.025[b]	-.343	.732	-.025	.987
	MRN0xIdeology0	.011[b]	.151	.880	.011	.994
	MRN0xNational0	-.041[b]	-.557	.578	-.040	.974
2	National0	-.010[c]	-.119	.906	-.009	.684
	MRN0	.140[c]	1.563	.120	.113	.575
	MRN0xRace	.046[c]	.594	.553	.043	.781
	MRN0xSES0	.011[c]	.166	.868	.012	.979
	MRN0xGender	-.012[c]	-.170	.865	-.012	.970
	MRN0xIdeology0	-.039[c]	-.563	.574	-.041	.970
	MRN0xNational0	-.037[c]	-.531	.596	-.039	.964
3	MRN0	.158[d]	1.679	.095	.121	.520
	MRN0xRace	.047[d]	.607	.545	.044	.773
	MRN0xSES0	.011[d]	.166	.869	.012	.979
	MRN0xGender	-.011[d]	-.154	.878	-.011	.950
	MRN0xIdeology0	-.041[d]	-.584	.560	-.042	.951
	MRN0xNational0	-.044[d]	-.600	.549	-.044	.865
4	MRN0xRace	-.005[e]	-.061	.952	-.004	.651
	MRN0xSES0	-.004[e]	-.063	.950	-.005	.961
	MRN0xGender	-.016[e]	-.224	.823	-.016	.948
	MRN0xIdeology0	-.036[e]	-.509	.612	-.037	.949
	MRN0xNational0	-.050[e]	-.684	.495	-.050	.863

a. Dependent Variable: Finance_Tot

b. Predictors in the Model: (Constant), Ideology0

c. Predictors in the Model: (Constant), Ideology0, SES0, GenderCC, RaceCC

d. Predictors in the Model: (Constant), Ideology0, SES0, GenderCC, RaceCC, National0

e. Predictors in the Model: (Constant), Ideology0, SES0, GenderCC, RaceCC, National0, MRN0

```
REGRESSION
 /MISSING LISTWISE
 /STATISTICS COEFF OUTS R ANOVA CHANGE ZPP
 /CRITERIA=PIN(.05) POUT(.10)
 /NOORIGIN
 /DEPENDENT Psychology_Tot
 /METHOD=ENTER Ideology0
 /METHOD=ENTER GenderCC RaceCC SES0
  /METHOD=ENTER National0
 /METHOD=ENTER MRN0
 /METHOD=ENTER MRN0xRace MRN0xSES0 MRN0xGender MRN0xIdeology0 MRN0xNational0.
```

Regression

Notes

Output Created		15-DEC-2021 13:11:11
Comments		
Input	Data	C: \Users\njs5478\Dropbox\HM and COVID\0. Revise and Resubmit\2. R and R Data\Study 2b\Study2b_Data.sav
	Active Dataset	DataSet1
	Filter	<none>
	Weight	<none>
	Split File	<none>
	N of Rows in Working Data File	198
Missing Value Handling	Definition of Missing	User-defined missing values are treated as missing.
	Cases Used	Statistics are based on cases with no missing values for any variable used.

Notes

Syntax		REGRESSION<br>/MISSING LISTWISE<br>/STATISTICS COEFF OUTS R ANOVA CHANGE ZPP<br>/CRITERIA=PIN(.05) POUT(.10)<br>/NOORIGIN<br>/DEPENDENT Psychology_Tot<br>/METHOD=ENTER Ideology0<br>/METHOD=ENTER GenderCC RaceCC SES0<br>/METHOD=ENTER National0<br>/METHOD=ENTER MRN0<br>/METHOD=ENTER MRN0xRace MRN0xSES0 MRN0xGender MRN0xIdeology0 MRN0xNational0.
Resources	Processor Time	00:00:00.02
	Elapsed Time	00:00:00.03
	Memory Required	34768 bytes
	Additional Memory Required for Residual Plots	0 bytes

Variables Entered/Removed[a]

Model	Variables Entered	Variables Removed	Method
1	Ideology0[b]	.	Enter
2	SES0, GenderCC, RaceCC[b]	.	Enter
3	National0[b]	.	Enter
4	MRN0[b]	.	Enter
5	MRN0xSES0, MRN0xGender, MRN0xIdeology0, MRN0xNational0, MRN0xRace[b]	.	Enter

a. Dependent Variable: Psychology_Tot

b. All requested variables entered.

Model Summary

Model	R	R Square	Adjusted R Square	Std. Error of the Estimate	Change Statistics		
					R Square Change	F Change	df1
1	.362[a]	.131	.127	1.59561	.131	29.247	1
2	.462[b]	.213	.197	1.53008	.082	6.658	3
3	.464[c]	.215	.195	1.53210	.002	.496	1
4	.471[d]	.222	.198	1.52931	.007	1.696	1
5	.496[e]	.246	.201	1.52610	.024	1.159	5

Model Summary

Model	Change Statistics	
	df2	Sig. F Change
1	194	.000
2	191	.000
3	190	.482
4	189	.194
5	184	.331

a. Predictors: (Constant), Ideology0

b. Predictors: (Constant), Ideology0, SES0, GenderCC, RaceCC

c. Predictors: (Constant), Ideology0, SES0, GenderCC, RaceCC, National0

d. Predictors: (Constant), Ideology0, SES0, GenderCC, RaceCC, National0, MRN0

e. Predictors: (Constant), Ideology0, SES0, GenderCC, RaceCC, National0, MRN0, MRN0xSES0, MRN0xGender, MRN0xIdeology0, MRN0xNational0, MRN0xRace

ANOVA[a]

Model		Sum of Squares	df	Mean Square	F	Sig.
1	Regression	74.462	1	74.462	29.247	.000[b]
	Residual	493.921	194	2.546		
	Total	568.383	195			
2	Regression	121.225	4	30.306	12.945	.000[c]
	Residual	447.158	191	2.341		
	Total	568.383	195			
3	Regression	122.388	5	24.478	10.428	.000[d]
	Residual	445.995	190	2.347		
	Total	568.383	195			
4	Regression	126.354	6	21.059	9.004	.000[e]
	Residual	442.029	189	2.339		
	Total	568.383	195			
5	Regression	139.848	11	12.713	5.459	.000[f]
	Residual	428.534	184	2.329		
	Total	568.383	195			

a. Dependent Variable: Psychology_Tot

b. Predictors: (Constant), Ideology0

c. Predictors: (Constant), Ideology0, SES0, GenderCC, RaceCC

d. Predictors: (Constant), Ideology0, SES0, GenderCC, RaceCC, National0

e. Predictors: (Constant), Ideology0, SES0, GenderCC, RaceCC, National0, MRN0

f. Predictors: (Constant), Ideology0, SES0, GenderCC, RaceCC, National0, MRN0, MRN0xSES0, MRN0xGender, MRN0xIdeology0, MRN0xNational0, MRN0xRace

Coefficients[a]

Model		Unstandardized Coefficients B	Unstandardized Coefficients Std. Error	Standardized Coefficients Beta	t	Sig.
1	(Constant)	4.116	.114		36.110	.000
	Ideology0	-.304	.056	-.362	-5.408	.000
2	(Constant)	3.992	.138		28.964	.000
	Ideology0	-.299	.055	-.356	-5.469	.000
	GenderCC	-.380	.111	-.223	-3.414	.001
	RaceCC	.199	.140	.093	1.425	.156
	SES0	-.234	.129	-.117	-1.815	.071
3	(Constant)	3.994	.138		28.934	.000
	Ideology0	-.324	.065	-.385	-4.978	.000
	GenderCC	-.382	.111	-.224	-3.425	.001
	RaceCC	.194	.140	.091	1.383	.168
	SES0	-.245	.130	-.123	-1.886	.061
	National0	.073	.104	.055	.704	.482
4	(Constant)	3.971	.139		28.580	.000
	Ideology0	-.363	.072	-.432	-5.070	.000
	GenderCC	-.433	.118	-.254	-3.669	.000
	RaceCC	.236	.144	.111	1.641	.102
	SES0	-.256	.130	-.128	-1.968	.051
	National0	.029	.109	.022	.269	.788
	MRN0	.191	.147	.116	1.302	.194
5	(Constant)	3.823	.161		23.817	.000
	Ideology0	-.367	.074	-.437	-4.972	.000
	GenderCC	-.397	.120	-.233	-3.317	.001
	RaceCC	.256	.147	.120	1.740	.084
	SES0	-.220	.131	-.110	-1.682	.094
	National0	.066	.116	.050	.570	.569
	MRN0	.295	.162	.179	1.820	.070
	MRN0xRace	-.177	.137	-.107	-1.297	.196
	MRN0xSES0	.009	.130	.005	.066	.947
	MRN0xGender	-.084	.118	-.048	-.710	.479
	MRN0xIdeology0	.124	.062	.147	1.990	.048
	MRN0xNational0	.003	.100	.002	.030	.976

Coefficients[a]

Model		Correlations Zero-order	Partial	Part
1	(Constant)			
	Ideology0	-.362	-.362	-.362
2	(Constant)			
	Ideology0	-.362	-.368	-.351
	GenderCC	-.268	-.240	-.219
	RaceCC	.075	.103	.091
	SES0	-.151	-.130	-.116
3	(Constant)			
	Ideology0	-.362	-.340	-.320
	GenderCC	-.268	-.241	-.220
	RaceCC	.075	.100	.089
	SES0	-.151	-.136	-.121
	National0	-.175	.051	.045
4	(Constant)			
	Ideology0	-.362	-.346	-.325
	GenderCC	-.268	-.258	-.235
	RaceCC	.075	.119	.105
	SES0	-.151	-.142	-.126
	National0	-.175	.020	.017
	MRN0	-.225	.094	.084
5	(Constant)			
	Ideology0	-.362	-.344	-.318
	GenderCC	-.268	-.238	-.212
	RaceCC	.075	.127	.111
	SES0	-.151	-.123	-.108
	National0	-.175	.042	.037
	MRN0	-.225	.133	.116
	MRN0xRace	-.223	-.095	-.083
	MRN0xSES0	.017	.005	.004
	MRN0xGender	.042	-.052	-.045
	MRN0xIdeology0	.181	.145	.127
	MRN0xNational0	.065	.002	.002

a. Dependent Variable: Psychology_Tot

Excluded Variables[a]

Model		Beta In	t	Sig.	Partial Correlation	Collinearity Statistics Tolerance
1	GenderCC	-.247[b]	-3.815	.000	-.265	.996
	RaceCC	.127[b]	1.886	.061	.135	.981
	SES0	-.134[b]	-2.021	.045	-.144	.998
	National0	.035[b]	.436	.663	.031	.698
	MRN0	-.035[b]	-.438	.662	-.032	.692
	MRN0xRace	-.097[b]	-1.344	.181	-.096	.851
	MRN0xSES0	-.021[b]	-.308	.759	-.022	.989
	MRN0xGender	.002[b]	.022	.982	.002	.987
	MRN0xIdeology0	.153[b]	2.306	.022	.164	.994
	MRN0xNational0	.006[b]	.094	.925	.007	.974
2	National0	.055[c]	.704	.482	.051	.684
	MRN0	.123[c]	1.460	.146	.105	.575
	MRN0xRace	-.022[c]	-.309	.758	-.022	.781
	MRN0xSES0	-.009[c]	-.141	.888	-.010	.979
	MRN0xGender	-.001[c]	-.008	.993	-.001	.970
	MRN0xIdeology0	.115[c]	1.769	.079	.127	.970
	MRN0xNational0	.019[c]	.288	.774	.021	.964
3	MRN0	.116[d]	1.302	.194	.094	.520
	MRN0xRace	-.028[d]	-.380	.704	-.028	.773
	MRN0xSES0	-.009[d]	-.140	.889	-.010	.979
	MRN0xGender	-.007[d]	-.112	.911	-.008	.950
	MRN0xIdeology0	.124[d]	1.888	.061	.136	.951
	MRN0xNational0	.038[d]	.542	.589	.039	.865
4	MRN0xRace	-.078[e]	-.979	.329	-.071	.651
	MRN0xSES0	-.021[e]	-.321	.749	-.023	.961
	MRN0xGender	-.011[e]	-.166	.868	-.012	.948
	MRN0xIdeology0	.128[e]	1.957	.052	.141	.949
	MRN0xNational0	.033[e]	.480	.631	.035	.863

a. Dependent Variable: Psychology_Tot

b. Predictors in the Model: (Constant), Ideology0

c. Predictors in the Model: (Constant), Ideology0, SES0, GenderCC, RaceCC

d. Predictors in the Model: (Constant), Ideology0, SES0, GenderCC, RaceCC, National0

e. Predictors in the Model: (Constant), Ideology0, SES0, GenderCC, RaceCC, National0, MRN0

```
REGRESSION
 /MISSING LISTWISE
 /STATISTICS COEFF OUTS R ANOVA CHANGE ZPP
 /CRITERIA=PIN(.05) POUT(.10)
 /NOORIGIN
 /DEPENDENT Risk_Rules
 /METHOD=ENTER Ideology0
 /METHOD=ENTER GenderCC RaceCC SES0
  /METHOD=ENTER National0
 /METHOD=ENTER MRN0
 /METHOD=ENTER MRN0xRace MRN0xSES0 MRN0xGender MRN0xIdeology0 MRN0xNational0.
```

Regression

Notes

Output Created		15-DEC-2021 13:11:11
Comments		
Input	Data	C: \Users\njs5478\Dropbox\HM and COVID\0. Revise and Resubmit\2. R and R Data\Study 2b\Study2b_Data.sav
	Active Dataset	DataSet1
	Filter	<none>
	Weight	<none>
	Split File	<none>
	N of Rows in Working Data File	198
Missing Value Handling	Definition of Missing	User-defined missing values are treated as missing.
	Cases Used	Statistics are based on cases with no missing values for any variable used.

Notes

Syntax		REGRESSION<br>/MISSING LISTWISE<br>/STATISTICS COEFF OUTS R ANOVA CHANGE ZPP<br>/CRITERIA=PIN(.05) POUT(.10)<br>/NOORIGIN<br>/DEPENDENT Risk_Rules<br>/METHOD=ENTER Ideology0<br>/METHOD=ENTER GenderCC RaceCC SES0<br>/METHOD=ENTER National0<br>/METHOD=ENTER MRN0<br>/METHOD=ENTER MRN0xRace MRN0xSES0 MRN0xGender MRN0xIdeology0 MRN0xNational0.
Resources	Processor Time	00:00:00.05
	Elapsed Time	00:00:00.05
	Memory Required	34768 bytes
	Additional Memory Required for Residual Plots	0 bytes

Variables Entered/Removed[a]

Model	Variables Entered	Variables Removed	Method
1	Ideology0[b]	.	Enter
2	SES0, GenderCC, RaceCC[b]	.	Enter
3	National0[b]	.	Enter
4	MRN0[b]	.	Enter
5	MRN0xSES0, MRN0xGender, MRN0xIdeology0, MRN0xNational0, MRN0xRace[b]	.	Enter

a. Dependent Variable: Risk_Rules

b. All requested variables entered.

Model Summary

					Change Statistics		
Model	R	R Square	Adjusted R Square	Std. Error of the Estimate	R Square Change	F Change	df1
1	.549[a]	.302	.298	.92747	.302	83.850	1
2	.560[b]	.313	.299	.92714	.011	1.046	3
3	.561[c]	.315	.297	.92839	.002	.486	1
4	.567[d]	.322	.300	.92627	.007	1.870	1
5	.576[e]	.332	.292	.93169	.010	.561	5

Model Summary

	Change Statistics	
Model	df2	Sig. F Change
1	194	.000
2	191	.373
3	190	.487
4	189	.173
5	184	.730

a. Predictors: (Constant), Ideology0

b. Predictors: (Constant), Ideology0, SES0, GenderCC, RaceCC

c. Predictors: (Constant), Ideology0, SES0, GenderCC, RaceCC, National0

d. Predictors: (Constant), Ideology0, SES0, GenderCC, RaceCC, National0, MRN0

e. Predictors: (Constant), Ideology0, SES0, GenderCC, RaceCC, National0, MRN0, MRN0xSES0, MRN0xGender, MRN0xIdeology0, MRN0xNational0, MRN0xRace

ANOVA[a]

Model		Sum of Squares	df	Mean Square	F	Sig.
1	Regression	72.127	1	72.127	83.850	.000[b]
	Residual	166.877	194	.860		
	Total	239.005	195			
2	Regression	74.825	4	18.706	21.762	.000[c]
	Residual	164.180	191	.860		
	Total	239.005	195			
3	Regression	75.244	5	15.049	17.460	.000[d]
	Residual	163.761	190	.862		
	Total	239.005	195			
4	Regression	76.848	6	12.808	14.928	.000[e]
	Residual	162.156	189	.858		
	Total	239.005	195			
5	Regression	79.283	11	7.208	8.303	.000[f]
	Residual	159.722	184	.868		
	Total	239.005	195			

a. Dependent Variable: Risk_Rules

b. Predictors: (Constant), Ideology0

c. Predictors: (Constant), Ideology0, SES0, GenderCC, RaceCC

d. Predictors: (Constant), Ideology0, SES0, GenderCC, RaceCC, National0

e. Predictors: (Constant), Ideology0, SES0, GenderCC, RaceCC, National0, MRN0

f. Predictors: (Constant), Ideology0, SES0, GenderCC, RaceCC, National0, MRN0, MRN0xSES0, MRN0xGender, MRN0xIdeology0, MRN0xNational0, MRN0xRace

Coefficients[a]

Model		Unstandardized Coefficients B	Unstandardized Coefficients Std. Error	Standardized Coefficients Beta	t	Sig.
1	(Constant)	3.303	.066		49.859	.000
	Ideology0	.299	.033	.549	9.157	.000
2	(Constant)	3.311	.084		39.647	.000
	Ideology0	.297	.033	.546	8.978	.000
	GenderCC	.114	.067	.103	1.688	.093
	RaceCC	-.011	.085	-.008	-.125	.901
	SES0	-.039	.078	-.030	-.504	.615
3	(Constant)	3.312	.084		39.597	.000
	Ideology0	.283	.039	.519	7.173	.000
	GenderCC	.113	.068	.102	1.669	.097
	RaceCC	-.014	.085	-.010	-.162	.871
	SES0	-.046	.079	-.036	-.586	.559
	National0	.044	.063	.051	.697	.487
4	(Constant)	3.298	.084		39.183	.000
	Ideology0	.257	.043	.472	5.936	.000
	GenderCC	.080	.072	.072	1.119	.265
	RaceCC	.013	.087	.009	.147	.883
	SES0	-.053	.079	-.041	-.673	.502
	National0	.016	.066	.018	.243	.808
	MRN0	.121	.089	.114	1.368	.173
5	(Constant)	3.333	.098		34.012	.000
	Ideology0	.257	.045	.471	5.689	.000
	GenderCC	.068	.073	.062	.937	.350
	RaceCC	-.011	.090	-.008	-.123	.903
	SES0	-.063	.080	-.049	-.788	.431
	National0	-.004	.071	-.005	-.062	.950
	MRN0	.062	.099	.058	.626	.532
	MRN0xRace	.128	.084	.119	1.531	.128
	MRN0xSES0	-.013	.080	-.011	-.164	.870
	MRN0xGender	.058	.072	.051	.799	.425
	MRN0xIdeology0	-.001	.038	-.002	-.031	.975
	MRN0xNational0	-.038	.061	-.046	-.620	.536

Coefficients[a]

Model		Correlations Zero-order	Partial	Part
1	(Constant)			
	Ideology0	.549	.549	.549
2	(Constant)			
	Ideology0	.549	.545	.538
	GenderCC	.134	.121	.101
	RaceCC	.051	-.009	-.007
	SES0	.005	-.036	-.030
3	(Constant)			
	Ideology0	.549	.462	.431
	GenderCC	.134	.120	.100
	RaceCC	.051	-.012	-.010
	SES0	.005	-.042	-.035
	National0	.335	.051	.042
4	(Constant)			
	Ideology0	.549	.396	.356
	GenderCC	.134	.081	.067
	RaceCC	.051	.011	.009
	SES0	.005	-.049	-.040
	National0	.335	.018	.015
	MRN0	.403	.099	.082
5	(Constant)			
	Ideology0	.549	.387	.343
	GenderCC	.134	.069	.056
	RaceCC	.051	-.009	-.007
	SES0	.005	-.058	-.048
	National0	.335	-.005	-.004
	MRN0	.403	.046	.038
	MRN0xRace	.331	.112	.092
	MRN0xSES0	-.055	-.012	-.010
	MRN0xGender	-.023	.059	.048
	MRN0xIdeology0	-.057	-.002	-.002
	MRN0xNational0	-.105	-.046	-.037

a. Dependent Variable: Risk_Rules

Excluded Variables[a]

Model		Beta In	t	Sig.	Partial Correlation	Collinearity Statistics Tolerance
1	GenderCC	.102[b]	1.700	.091	.121	.996
	RaceCC	-.024[b]	-.400	.690	-.029	.981
	SES0	-.022[b]	-.363	.717	-.026	.998
	National0	.048[b]	.663	.508	.048	.698
	MRN0	.142[b]	1.979	.049	.141	.692
	MRN0xRace	.140[b]	2.173	.031	.155	.851
	MRN0xSES0	.002[b]	.033	.974	.002	.989
	MRN0xGender	.039[b]	.646	.519	.046	.987
	MRN0xIdeology0	-.013[b]	-.215	.830	-.015	.994
	MRN0xNational0	-.017[b]	-.279	.780	-.020	.974
2	National0	.051[c]	.697	.487	.051	.684
	MRN0	.120[c]	1.520	.130	.110	.575
	MRN0xRace	.124[c]	1.833	.068	.132	.781
	MRN0xSES0	-.003[c]	-.056	.955	-.004	.979
	MRN0xGender	.042[c]	.696	.487	.050	.970
	MRN0xIdeology0	-.006[c]	-.094	.925	-.007	.970
	MRN0xNational0	-.027[c]	-.445	.657	-.032	.964
3	MRN0	.114[d]	1.368	.173	.099	.520
	MRN0xRace	.120[d]	1.769	.079	.128	.773
	MRN0xSES0	-.003[d]	-.056	.956	-.004	.979
	MRN0xGender	.037[d]	.600	.549	.044	.950
	MRN0xIdeology0	.000[d]	.005	.996	.000	.951
	MRN0xNational0	-.015[d]	-.233	.816	-.017	.865
4	MRN0xRace	.099[e]	1.332	.184	.097	.651
	MRN0xSES0	-.015[e]	-.245	.807	-.018	.961
	MRN0xGender	.034[e]	.545	.587	.040	.948
	MRN0xIdeology0	.004[e]	.070	.945	.005	.949
	MRN0xNational0	-.019[e]	-.300	.765	-.022	.863

a. Dependent Variable: Risk_Rules

b. Predictors in the Model: (Constant), Ideology0

c. Predictors in the Model: (Constant), Ideology0, SES0, GenderCC, RaceCC

d. Predictors in the Model: (Constant), Ideology0, SES0, GenderCC, RaceCC, National0

e. Predictors in the Model: (Constant), Ideology0, SES0, GenderCC, RaceCC, National0, MRN0

```
REGRESSION
 /MISSING LISTWISE
 /STATISTICS COEFF OUTS R ANOVA CHANGE ZPP
 /CRITERIA=PIN(.05) POUT(.10)
 /NOORIGIN
 /DEPENDENT Mandate_Tot
 /METHOD=ENTER Ideology0
 /METHOD=ENTER GenderCC RaceCC SES0
  /METHOD=ENTER National0
 /METHOD=ENTER MRN0
 /METHOD=ENTER MRN0xRace MRN0xSES0 MRN0xGender MRN0xIdeology0 MRN0xNational0.
```

Regression

Notes

Output Created		15-DEC-2021 13:11:11
Comments		
Input	Data	C: \Users\njs5478\Dropbox\HM and COVID\0. Revise and Resubmit\2. R and R Data\Study 2b\Study2b_Data.sav
	Active Dataset	DataSet1
	Filter	<none>
	Weight	<none>
	Split File	<none>
	N of Rows in Working Data File	198
Missing Value Handling	Definition of Missing	User-defined missing values are treated as missing.
	Cases Used	Statistics are based on cases with no missing values for any variable used.

Notes

Syntax		REGRESSION<br>/MISSING LISTWISE<br>/STATISTICS COEFF OUTS R ANOVA CHANGE ZPP<br>/CRITERIA=PIN(.05) POUT(.10)<br>/NOORIGIN<br>/DEPENDENT Mandate_Tot<br>/METHOD=ENTER Ideology0<br>/METHOD=ENTER GenderCC RaceCC SES0<br>/METHOD=ENTER National0<br>/METHOD=ENTER MRN0<br>/METHOD=ENTER MRN0xRace MRN0xSES0 MRN0xGender MRN0xIdeology0 MRN0xNational0.
Resources	Processor Time	00:00:00.03
	Elapsed Time	00:00:00.06
	Memory Required	34768 bytes
	Additional Memory Required for Residual Plots	0 bytes

Variables Entered/Removed[a]

Model	Variables Entered	Variables Removed	Method
1	Ideology0[b]	.	Enter
2	SES0, GenderCC, RaceCC[b]	.	Enter
3	National0[b]	.	Enter
4	MRN0[b]	.	Enter
5	MRN0xSES0, MRN0xGender, MRN0xIdeology0, MRN0xNational0, MRN0xRace[b]	.	Enter

a. Dependent Variable: Mandate_Tot

b. All requested variables entered.

Model Summary

Model	R	R Square	Adjusted R Square	Std. Error of the Estimate	Change Statistics		
					R Square Change	F Change	df1
1	.726[a]	.527	.525	1.54056	.527	216.378	1
2	.729[b]	.532	.522	1.54481	.005	.644	3
3	.729[c]	.532	.520	1.54887	.000	.001	1
4	.730[d]	.533	.518	1.55192	.001	.253	1
5	.739[e]	.546	.519	1.54973	.014	1.107	5

Model Summary

Model	Change Statistics	
	df2	Sig. F Change
1	194	.000
2	191	.588
3	190	.981
4	189	.615
5	184	.358

a. Predictors: (Constant), Ideology0

b. Predictors: (Constant), Ideology0, SES0, GenderCC, RaceCC

c. Predictors: (Constant), Ideology0, SES0, GenderCC, RaceCC, National0

d. Predictors: (Constant), Ideology0, SES0, GenderCC, RaceCC, National0, MRN0

e. Predictors: (Constant), Ideology0, SES0, GenderCC, RaceCC, National0, MRN0, MRN0xSES0, MRN0xGender, MRN0xIdeology0, MRN0xNational0, MRN0xRace

ANOVA[a]

Model		Sum of Squares	df	Mean Square	F	Sig.
1	Regression	513.533	1	513.533	216.378	.000[b]
	Residual	460.423	194	2.373		
	Total	973.955	195			
2	Regression	518.143	4	129.536	54.280	.000[c]
	Residual	455.813	191	2.386		
	Total	973.955	195			
3	Regression	518.144	5	103.629	43.197	.000[d]
	Residual	455.811	190	2.399		
	Total	973.955	195			
4	Regression	518.754	6	86.459	35.898	.000[e]
	Residual	455.201	189	2.408		
	Total	973.955	195			
5	Regression	532.047	11	48.368	20.139	.000[f]
	Residual	441.908	184	2.402		
	Total	973.955	195			

a. Dependent Variable: Mandate_Tot

b. Predictors: (Constant), Ideology0

c. Predictors: (Constant), Ideology0, SES0, GenderCC, RaceCC

d. Predictors: (Constant), Ideology0, SES0, GenderCC, RaceCC, National0

e. Predictors: (Constant), Ideology0, SES0, GenderCC, RaceCC, National0, MRN0

f. Predictors: (Constant), Ideology0, SES0, GenderCC, RaceCC, National0, MRN0, MRN0xSES0, MRN0xGender, MRN0xIdeology0, MRN0xNational0, MRN0xRace

Coefficients[a]

Model		Unstandardized Coefficients B	Unstandardized Coefficients Std. Error	Standardized Coefficients Beta	t	Sig.
1	(Constant)	4.523	.110		41.099	.000
	Ideology0	-.799	.054	-.726	-14.710	.000
2	(Constant)	4.534	.139		32.587	.000
	Ideology0	-.803	.055	-.730	-14.552	.000
	GenderCC	.078	.112	.035	.698	.486
	RaceCC	-.017	.141	-.006	-.122	.903
	SES0	.145	.130	.056	1.117	.266
3	(Constant)	4.534	.140		32.486	.000
	Ideology0	-.802	.066	-.729	-12.207	.000
	GenderCC	.079	.113	.035	.697	.487
	RaceCC	-.017	.142	-.006	-.120	.904
	SES0	.146	.131	.056	1.108	.269
	National0	-.002	.105	-.001	-.023	.981
4	(Constant)	4.525	.141		32.090	.000
	Ideology0	-.818	.073	-.743	-11.253	.000
	GenderCC	.058	.120	.026	.487	.627
	RaceCC	-.001	.146	.000	-.004	.996
	SES0	.141	.132	.054	1.072	.285
	National0	-.020	.110	-.011	-.177	.859
	MRN0	.075	.149	.035	.503	.615
5	(Constant)	4.342	.163		26.634	.000
	Ideology0	-.817	.075	-.742	-10.885	.000
	GenderCC	.083	.122	.037	.680	.497
	RaceCC	.010	.149	.003	.064	.949
	SES0	.171	.133	.066	1.293	.198
	National0	.037	.118	.021	.317	.752
	MRN0	.168	.164	.078	1.020	.309
	MRN0xRace	-.175	.139	-.081	-1.260	.209
	MRN0xSES0	-.010	.132	-.004	-.073	.942
	MRN0xGender	-.027	.120	-.012	-.229	.819
	MRN0xIdeology0	.098	.063	.089	1.546	.124
	MRN0xNational0	.084	.102	.050	.826	.410

Coefficients[a]

Model		Correlations		
		Zero-order	Partial	Part
1	(Constant)			
	Ideology0	-.726	-.726	-.726
2	(Constant)			
	Ideology0	-.726	-.725	-.720
	GenderCC	-.003	.050	.035
	RaceCC	-.110	-.009	-.006
	SES0	.023	.081	.055
3	(Constant)			
	Ideology0	-.726	-.663	-.606
	GenderCC	-.003	.050	.035
	RaceCC	-.110	-.009	-.006
	SES0	.023	.080	.055
	National0	-.394	-.002	-.001
4	(Constant)			
	Ideology0	-.726	-.633	-.560
	GenderCC	-.003	.035	.024
	RaceCC	-.110	.000	.000
	SES0	.023	.078	.053
	National0	-.394	-.013	-.009
	MRN0	-.369	.037	.025
5	(Constant)			
	Ideology0	-.726	-.626	-.541
	GenderCC	-.003	.050	.034
	RaceCC	-.110	.005	.003
	SES0	.023	.095	.064
	National0	-.394	.023	.016
	MRN0	-.369	.075	.051
	MRN0xRace	-.293	-.092	-.063
	MRN0xSES0	.072	-.005	-.004
	MRN0xGender	.102	-.017	-.011
	MRN0xIdeology0	.139	.113	.077
	MRN0xNational0	.176	.061	.041

a. Dependent Variable: Mandate_Tot

Excluded Variables[a]

Model		Beta In	t	Sig.	Partial Correlation	Collinearity Statistics Tolerance
1	GenderCC	.041[b]	.824	.411	.059	.996
	RaceCC	-.011[b]	-.217	.829	-.016	.981
	SES0	.058[b]	1.183	.238	.085	.998
	National0	.008[b]	.130	.896	.009	.698
	MRN0	.050[b]	.845	.399	.061	.692
	MRN0xRace	-.014[b]	-.265	.791	-.019	.851
	MRN0xSES0	-.004[b]	-.084	.933	-.006	.989
	MRN0xGender	.021[b]	.413	.680	.030	.987
	MRN0xIdeology0	.082[b]	1.654	.100	.118	.994
	MRN0xNational0	.061[b]	1.217	.225	.087	.974
2	National0	-.001[c]	-.023	.981	-.002	.684
	MRN0	.031[c]	.473	.637	.034	.575
	MRN0xRace	-.033[c]	-.594	.554	-.043	.781
	MRN0xSES0	-.008[c]	-.153	.879	-.011	.979
	MRN0xGender	.018[c]	.352	.726	.025	.970
	MRN0xIdeology0	.093[c]	1.873	.063	.135	.970
	MRN0xNational0	.061[c]	1.211	.227	.088	.964
3	MRN0	.035[d]	.503	.615	.037	.520
	MRN0xRace	-.034[d]	-.593	.554	-.043	.773
	MRN0xSES0	-.008[d]	-.153	.879	-.011	.979
	MRN0xGender	.018[d]	.358	.721	.026	.950
	MRN0xIdeology0	.095[d]	1.884	.061	.136	.951
	MRN0xNational0	.068[d]	1.268	.206	.092	.865
4	MRN0xRace	-.053[e]	-.863	.389	-.063	.651
	MRN0xSES0	-.011[e]	-.223	.824	-.016	.961
	MRN0xGender	.017[e]	.337	.737	.025	.948
	MRN0xIdeology0	.097[e]	1.907	.058	.138	.949
	MRN0xNational0	.066[e]	1.243	.216	.090	.863

a. Dependent Variable: Mandate_Tot

b. Predictors in the Model: (Constant), Ideology0

c. Predictors in the Model: (Constant), Ideology0, SES0, GenderCC, RaceCC

d. Predictors in the Model: (Constant), Ideology0, SES0, GenderCC, RaceCC, National0

e. Predictors in the Model: (Constant), Ideology0, SES0, GenderCC, RaceCC, National0, MRN0

```
REGRESSION
 /MISSING LISTWISE
 /STATISTICS COEFF OUTS R ANOVA CHANGE ZPP
 /CRITERIA=PIN(.05) POUT(.10)
 /NOORIGIN
 /DEPENDENT Conspiracy_Tot
 /METHOD=ENTER Ideology0
 /METHOD=ENTER GenderCC RaceCC SES0
  /METHOD=ENTER National0
 /METHOD=ENTER MRN0
 /METHOD=ENTER MRN0xRace MRN0xSES0 MRN0xGender MRN0xIdeology0 MRN0xNational0.
```

Regression

Notes

Output Created		15-DEC-2021 13:11:11
Comments		
Input	Data	C:\Users\njs5478\Dropbox\HM and COVID\0. Revise and Resubmit\2. R and R Data\Study 2b\Study2b_Data.sav
	Active Dataset	DataSet1
	Filter	<none>
	Weight	<none>
	Split File	<none>
	N of Rows in Working Data File	198
Missing Value Handling	Definition of Missing	User-defined missing values are treated as missing.
	Cases Used	Statistics are based on cases with no missing values for any variable used.

Notes

Syntax		REGRESSION<br>/MISSING LISTWISE<br>/STATISTICS COEFF OUTS R ANOVA CHANGE ZPP<br>/CRITERIA=PIN(.05) POUT(.10)<br>/NOORIGIN<br>/DEPENDENT Conspiracy_Tot<br>/METHOD=ENTER Ideology0<br>/METHOD=ENTER GenderCC RaceCC SES0<br>/METHOD=ENTER National0<br>/METHOD=ENTER MRN0<br>/METHOD=ENTER MRN0xRace MRN0xSES0 MRN0xGender MRN0xIdeology0 MRN0xNational0.
Resources	Processor Time	00:00:00.05
	Elapsed Time	00:00:00.03
	Memory Required	34768 bytes
	Additional Memory Required for Residual Plots	0 bytes

Variables Entered/Removed[a]

Model	Variables Entered	Variables Removed	Method
1	Ideology0[b]	.	Enter
2	SES0, GenderCC, RaceCC[b]	.	Enter
3	National0[b]	.	Enter
4	MRN0[b]	.	Enter
5	MRN0xSES0, MRN0xGender, MRN0xIdeology0, MRN0xNational0, MRN0xRace[b]	.	Enter

a. Dependent Variable: Conspiracy_Tot

b. All requested variables entered.

Model Summary

					Change Statistics		
Model	R	R Square	Adjusted R Square	Std. Error of the Estimate	R Square Change	F Change	df1
1	.513[a]	.264	.260	.55505	.264	69.443	1
2	.515[b]	.265	.250	.55881	.002	.133	3
3	.529[c]	.280	.261	.55459	.015	3.916	1
4	.585[d]	.342	.321	.53163	.062	17.764	1
5	.592[e]	.351	.312	.53523	.009	.494	5

Model Summary

	Change Statistics	
Model	df2	Sig. F Change
1	194	.000
2	191	.940
3	190	.049
4	189	.000
5	184	.781

a. Predictors: (Constant), Ideology0

b. Predictors: (Constant), Ideology0, SES0, GenderCC, RaceCC

c. Predictors: (Constant), Ideology0, SES0, GenderCC, RaceCC, National0

d. Predictors: (Constant), Ideology0, SES0, GenderCC, RaceCC, National0, MRN0

e. Predictors: (Constant), Ideology0, SES0, GenderCC, RaceCC, National0, MRN0, MRN0xSES0, MRN0xGender, MRN0xIdeology0, MRN0xNational0, MRN0xRace

ANOVA[a]

Model		Sum of Squares	df	Mean Square	F	Sig.
1	Regression	21.394	1	21.394	69.443	.000[b]
	Residual	59.768	194	.308		
	Total	81.162	195			
2	Regression	21.519	4	5.380	17.228	.000[c]
	Residual	59.643	191	.312		
	Total	81.162	195			
3	Regression	22.724	5	4.545	14.776	.000[d]
	Residual	58.438	190	.308		
	Total	81.162	195			
4	Regression	27.744	6	4.624	16.361	.000[e]
	Residual	53.417	189	.283		
	Total	81.162	195			
5	Regression	28.452	11	2.587	9.029	.000[f]
	Residual	52.710	184	.286		
	Total	81.162	195			

a. Dependent Variable: Conspiracy_Tot

b. Predictors: (Constant), Ideology0

c. Predictors: (Constant), Ideology0, SES0, GenderCC, RaceCC

d. Predictors: (Constant), Ideology0, SES0, GenderCC, RaceCC, National0

e. Predictors: (Constant), Ideology0, SES0, GenderCC, RaceCC, National0, MRN0

f. Predictors: (Constant), Ideology0, SES0, GenderCC, RaceCC, National0, MRN0, MRN0xSES0, MRN0xGender, MRN0xIdeology0, MRN0xNational0, MRN0xRace

Coefficients[a]

Model		Unstandardized Coefficients		Standardized Coefficients	t	Sig.
		B	Std. Error	Beta		
1	(Constant)	1.702	.040		42.926	.000
	Ideology0	.163	.020	.513	8.333	.000
2	(Constant)	1.702	.050		33.822	.000
	Ideology0	.163	.020	.515	8.185	.000
	GenderCC	.008	.041	.013	.203	.840
	RaceCC	.000	.051	-.001	-.009	.993
	SES0	-.029	.047	-.038	-.613	.541
3	(Constant)	1.705	.050		34.123	.000
	Ideology0	.138	.024	.435	5.876	.000
	GenderCC	.006	.040	.010	.158	.875
	RaceCC	-.006	.051	-.007	-.117	.907
	SES0	-.040	.047	-.053	-.857	.393
	National0	.074	.038	.147	1.979	.049
4	(Constant)	1.679	.048		34.761	.000
	Ideology0	.094	.025	.296	3.772	.000
	GenderCC	-.052	.041	-.080	-1.256	.211
	RaceCC	.041	.050	.051	.824	.411
	SES0	-.052	.045	-.069	-1.160	.248
	National0	.025	.038	.050	.664	.507
	MRN0	.215	.051	.345	4.215	.000
5	(Constant)	1.649	.056		29.287	.000
	Ideology0	.089	.026	.281	3.443	.001
	GenderCC	-.051	.042	-.079	-1.210	.228
	RaceCC	.048	.052	.060	.938	.349
	SES0	-.047	.046	-.063	-1.029	.305
	National0	.045	.041	.089	1.095	.275
	MRN0	.233	.057	.374	4.105	.000
	MRN0xRace	-.029	.048	-.047	-.611	.542
	MRN0xSES0	-.032	.046	-.044	-.690	.491
	MRN0xGender	-.025	.041	-.038	-.611	.542
	MRN0xIdeology0	.007	.022	.022	.322	.747
	MRN0xNational0	.040	.035	.083	1.142	.255

Coefficients[a]

Model		Correlations Zero-order	Partial	Part
1	(Constant)			
	Ideology0	.513	.513	.513
2	(Constant)			
	Ideology0	.513	.510	.508
	GenderCC	.040	.015	.013
	RaceCC	.067	-.001	-.001
	SES0	-.012	-.044	-.038
3	(Constant)			
	Ideology0	.513	.392	.362
	GenderCC	.040	.011	.010
	RaceCC	.067	-.008	-.007
	SES0	-.012	-.062	-.053
	National0	.379	.142	.122
4	(Constant)			
	Ideology0	.513	.265	.223
	GenderCC	.040	-.091	-.074
	RaceCC	.067	.060	.049
	SES0	-.012	-.084	-.068
	National0	.379	.048	.039
	MRN0	.493	.293	.249
5	(Constant)			
	Ideology0	.513	.246	.205
	GenderCC	.040	-.089	-.072
	RaceCC	.067	.069	.056
	SES0	-.012	-.076	-.061
	National0	.379	.080	.065
	MRN0	.493	.290	.244
	MRN0xRace	.284	-.045	-.036
	MRN0xSES0	-.053	-.051	-.041
	MRN0xGender	-.052	-.045	-.036
	MRN0xIdeology0	-.023	.024	.019
	MRN0xNational0	-.047	.084	.068

a. Dependent Variable: Conspiracy_Tot

Excluded Variables[a]

Model		Beta In	t	Sig.	Partial Correlation	Collinearity Statistics Tolerance
1	GenderCC	.010[b]	.156	.876	.011	.996
	RaceCC	-.003[b]	-.051	.959	-.004	.981
	SES0	-.037[b]	-.601	.549	-.043	.998
	National0	.139[b]	1.897	.059	.135	.698
	MRN0	.301[b]	4.237	.000	.292	.692
	MRN0xRace	.100[b]	1.505	.134	.108	.851
	MRN0xSES0	.001[b]	.010	.992	.001	.989
	MRN0xGender	.006[b]	.089	.929	.006	.987
	MRN0xIdeology0	.019[b]	.304	.762	.022	.994
	MRN0xNational0	.036[b]	.582	.561	.042	.974
2	National0	.147[c]	1.979	.049	.142	.684
	MRN0	.362[c]	4.653	.000	.320	.575
	MRN0xRace	.110[c]	1.569	.118	.113	.781
	MRN0xSES0	.001[c]	.014	.989	.001	.979
	MRN0xGender	.008[c]	.132	.895	.010	.970
	MRN0xIdeology0	.016[c]	.260	.795	.019	.970
	MRN0xNational0	.034[c]	.538	.592	.039	.964
3	MRN0	.345[d]	4.215	.000	.293	.520
	MRN0xRace	.097[d]	1.388	.167	.100	.773
	MRN0xSES0	.001[d]	.015	.988	.001	.979
	MRN0xGender	-.010[d]	-.158	.875	-.011	.950
	MRN0xIdeology0	.035[d]	.548	.584	.040	.951
	MRN0xNational0	.082[d]	1.245	.215	.090	.865
4	MRN0xRace	-.018[e]	-.249	.804	-.018	.651
	MRN0xSES0	-.034[e]	-.564	.573	-.041	.961
	MRN0xGender	-.021[e]	-.340	.734	-.025	.948
	MRN0xIdeology0	.047[e]	.772	.441	.056	.949
	MRN0xNational0	.070[e]	1.095	.275	.080	.863

a. Dependent Variable: Conspiracy_Tot

b. Predictors in the Model: (Constant), Ideology0

c. Predictors in the Model: (Constant), Ideology0, SES0, GenderCC, RaceCC

d. Predictors in the Model: (Constant), Ideology0, SES0, GenderCC, RaceCC, National0

e. Predictors in the Model: (Constant), Ideology0, SES0, GenderCC, RaceCC, National0, MRN0
